# Proteomic and transcriptomic profiles of human urothelial cancer cells with histone deacetylase 5 overexpression

**DOI:** 10.1038/s41597-022-01319-0

**Published:** 2022-05-27

**Authors:** Ananda Ayyappan Jaguva Vasudevan, Michèle J. Hoffmann, Gereon Poschmann, Patrick Petzsch, Constanze Wiek, Kai Stühler, Karl Köhrer, Wolfgang A. Schulz, Günter Niegisch

**Affiliations:** 1 Department of Urology, University Hospital and Medical Faculty, Heinrich Heine University Düsseldorf, 40225 Düsseldorf, Germany; 2 Institute for Molecular Medicine, Proteome Research, University Hospital and Medical Faculty, Heinrich Heine University Düsseldorf, 40225 Düsseldorf, Germany; 3 Genomics & Transcriptomics Laboratory, Biological and Medical Research Centre (BMFZ), Heinrich Heine University Düsseldorf, 40225 Düsseldorf, Germany; 4 Department of Otorhinolaryngology, Head and Neck Surgery, University Hospital and Medical Faculty, Heinrich Heine University Düsseldorf, Düsseldorf, 40225 Germany; 5 Molecular Proteomics Laboratory, Biological and Medical Research Centre (BMFZ), Heinrich Heine University Düsseldorf, 40225 Düsseldorf, Germany; 6 Present Address: Structural Cell Biology Group, Genome Integrity and Structural Biology Laboratory, National Institute of Environmental Health Sciences (NIEHS), NIH, Research Triangle Park, NC 27709 USA

**Keywords:** Bladder cancer, Transcriptomics, Proteomics

## Abstract

Urothelial carcinoma (UC) of the urinary bladder is a prevalent cancer worldwide. Because histone deacetylases (HDACs) are important factors in cancer, targeting these epigenetic regulators is considered an attractive strategy to develop novel anticancer drugs. Whereas HDAC1 and HDAC2 promote UC, HDAC5 is often downregulated and only weakly expressed in UC cell lines, suggesting a tumor-suppressive function. We studied the effect of stable lentiviral-mediated HDAC5 overexpression in four UC cell lines with different phenotypes (RT112, VM-Cub-1, SW1710, and UM-UC-3, each with vector controls). In particular, comprehensive proteomics and RNA-seq transcriptomics analyses were performed on the four cell line pairs, which are described here. For comparison, the immortalized benign urothelial cell line HBLAK was included. These datasets will be a useful resource for researchers studying UC, and especially the influence of HDAC5 on epithelial-mesenchymal transition (EMT). Moreover, these data will inform studies on HDAC5 as a less studied member of the HDAC family in other cell types and diseases, especially fibrosis.

## Abstract (summary)

**Table:** 

Measurement(s)	RNA-seq assay • Proteome
Technology Type(s)	Whole Transcriptome Sequencing • Mass spectrometry intensity based label-free quantification method
Factor Type(s)	Effect of HDAC5 overexpression in a range of urothelial cancer cells
Sample Characteristic - Organism	Homo sapiens

## Background & Summary

In humans, the classical histone deacetylase (HDAC) family comprises 11 members. They all remove acetyl groups from histones or non-histone proteins, but differ in their substrates in detail, their cellular localization, their cell-type distribution and accordingly, their overall physiological functions and involvement in disease^1–7^. HDACs 1–3 are broadly expressed and support proliferation and survival of many cell types including cancer cells^6,8^. In contrast, HDAC5 expression is more variable between tissues^9,10^. Its function and its mechanisms of action are less well studied, but the enzyme is implicated in the differentiation and function of neurons, among others^7,11–13^. In cancers, this enzyme can act in a tumor-promoting or tumor-inhibitory fashion depending on the tissue^7,12,13^. It may also have a role in fibrotic diseases^14^.

HDAC inhibitors (HDACi) are used and further developed as drugs to treat cancer, especially hematological malignancies, and other diseases. Some of these inhibitors target a broad spectrum of HDACs (pan-HDACi), whereas others are more specific for particular isoenzymes. Since the relevance of each isoenzyme varies between tissues, diseases, and cancer types, HDACi would ideally be tailored to inhibit a specific subset of HDACs in each medical application.

Urothelial carcinoma (UC) is the most common histological subtype of urinary bladder cancer^15,16^. As few treatment options are available for advanced stage UC, our group and others have explored the usefulness of HDACi to treat this disease. In brief, previous work has confirmed the importance of HDAC1 and HDAC2 for UC cell proliferation and survival^17^. Notably, specific inhibitors of this enzyme pair are considerably more efficacious than pan-HDACi, suggesting that inhibition of other enzymes like HDAC5 may actually be counterproductive^11^. In fact, we found very low expression of HDAC5 in UC cell lines, hinting at a tumor-suppressive function^10,18^. Therefore, we studied the effect of HDAC5 overexpression in a range of UC cell lines with different phenotypes that cover the range of the disease. Endogenous protein levels of HDAC5 in these cell lines were all low. The cellular effects of HDAC5 overexpression reported previously^10^ included indeed decreased cell proliferation but also promotion of epithelial-mesenchymal transition (EMT).

**Fig. 1:**
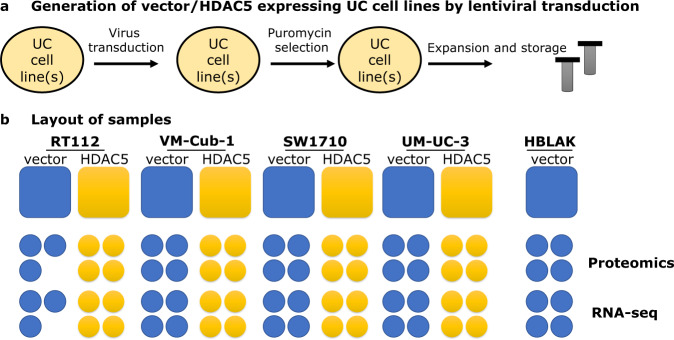
Schematic overview of the study. (**a**) An overview of stable cell line generation by lentiviral particles harboring either vector only or transgene *HDAC5*. See methods for more information. (**b**) To prepare samples for high throughput proteomics and RNA-seq analysis, each cell lines as designated in the figure were cultured before going for respective downstream procedures. “vector” indicates the absence of HDAC5 expression in cells but carrying empty vector and processed the same way as HDAC5 expressing cell lines. Transduced vector-only HBLAK cell lines, but not HDAC5 expressing HBLAKs were a part of this study.

To gain deeper insights into the cellular and molecular mechanisms underlying the effects of HDAC5, we performed comprehensive whole-cell proteome and transcriptome analyses of the UC cell lines RT112, VM-Cub-1, SW1710, and UM-UC-3 engineered to stably overexpress HDAC5 or transduced with vector only. For comparison, we analyzed a benign urothelial control cell line, HBLAK as a “vector-only” form, as this cell line does not tolerate HDAC5 overexpression (Fig. 1, Table 1, and Table 2). All analyses were performed using quadruplicates (except triplicates for RT112) from the same cell lines at one time point. These datasets will be a useful resource for researchers studying UC. In particular, since we observed an influence of HDAC5 on EMT, the datasets shed light on this process in UC. Moreover, as HDAC5 is a less studied member of the HDAC family, our data could inform studies on this enzyme in other cell types and diseases, especially fibrosis.

**Table 1:** Summary of samples and their proteomics datasets stored in PRIDE online repository.

Cell line	Replicates	Transgene expression/Variables	Method	Data collection	Data
RT112	3X	vector-only	Protein extraction	Mass spectrometry	PXD014448
RT112	4X	HDAC5	Protein extraction	Mass spectrometry	PXD014448
VM-Cub-1	4X	vector-only	Protein extraction	Mass spectrometry	PXD014448
VM-Cub-1	4X	HDAC5	Protein extraction	Mass spectrometry	PXD014448
SW1710	4X	vector-only	Protein extraction	Mass spectrometry	PXD014448
SW1710	4X	HDAC5	Protein extraction	Mass spectrometry	PXD014448
UM-UC-3	4X	vector-only	Protein extraction	Mass spectrometry	PXD014448
UM-UC-3	4X	HDAC5	Protein extraction	Mass spectrometry	PXD014448
HBLAK	4X	vector-only	Protein extraction	Mass spectrometry	PXD014448

**Table 2:** Summary of samples and their RNA-seq datasets stored in ArrayExpress online repository.

Cell line	Replicates	Transgene expression/Variables	Method	Data collection	Data
RT112	3X	vector-only	RNA extraction	RNA-seq	E-MTAB-8418
RT112	4X	HDAC5	RNA extraction	RNA-seq	E-MTAB-8418
VM-Cub-1	4X	vector-only	RNA extraction	RNA-seq	E-MTAB-8418
VM-Cub-1	4X	HDAC5	RNA extraction	RNA-seq	E-MTAB-8418
SW1710	4X	vector-only	RNA extraction	RNA-seq	E-MTAB-8418
SW1710	4X	HDAC5	RNA extraction	RNA-seq	E-MTAB-8418
UM-UC-3	4X	vector-only	RNA extraction	RNA-seq	E-MTAB-8418
UM-UC-3	4X	HDAC5	RNA extraction	RNA-seq	E-MTAB-8418
HBLAK	4X	vector-only	RNA extraction	RNA-seq	E-MTAB-8418

## Methods

### Short tandem repeat (STR) profiling

All urothelial cancer cell lines (UCCs) and the HBLAK cell line were authenticated by STR profiling.

### Cell culture

The urothelial cancer cell lines (UCCs) VM-Cub1, RT112, SW1710, and UM-UC-3 were provided by Dr. M. A. Knowles (Leeds, UK), Dr. J. Fogh (New York, USA) and Dr. B. Grossmann (Houston, USA) or by the DSMZ (Braunschweig, Germany). They were cultured in DMEM GlutaMAX-I (Gibco, Life Technologies, Darmstadt, Germany) supplemented with 10% fetal calf serum (Biochrom, Berlin, Germany) at 37 °C in a humidified atmosphere of 5% CO_2_. As a benign urothelial control, we used the HBLAK cell line (Hoffmann *et al*.^19^, spontaneously immortalized from primary human bladder epithelial cells; kindly donated by CELLnTEC, Bern, Switzerland), which were cultured in CnT-Prime Epithelial Culture Medium (CELLnTEC, Bern, Switzerland). All cell lines were authenticated by DNA fingerprint analysis. Normal urothelial cells (UP) were cultured as described^19^ with informed consent of the donors and approval by the Ethics Committee of the Medical Faculty of the Heinrich-Heine-University, study number 1788.

### Generation of stably expressing HDAC5 and vector control UC cell lines

The plasmid pcDNA3.1 + HDAC5-FLAG was a gift from Eric Verdin (Addgene plasmid #13822)^2^. The HDAC5 open reading frame with a C-terminal FLAG tag was subcloned into the lentiviral transfer vector puc2CL12IPwo using primers forward 5′-CATCTCGAGGCCACCATGCCCAGTTCCATGGG and reverse 5′-ATCGCTAGCTTACTTGTCATCGTCGTCCTTGTAGTCTCCTCCCAGGGCAGGCTCCTGC. The construct was verified by Sanger sequencing. Lentivirus production and cell transduction were performed as previously described^20,21^. Briefly, HEK-293T cells were transfected with helper plasmid expression construct pCD/NL-BH, envelope vector (pczVSV-G) and either the vector plasmids puc2CL12IPwo or puc2CL12IPwo-HDAC5-FLAG. Replication-deficient lentiviral particles were harvested 48 h after transfection and used to transduce RT112, VM-Cub-1, SW1710, and UM-UC-3 cells using 8 µg/ml polybrene (Sigma-Aldrich). Twenty-four hours after transduction, the supernatant containing viral particles was removed and the transduced cells were selected and maintained with 1 µg/ml puromycin (Invitrogen, Carlsbad, CA, USA). Stable expression of HDAC5 was confirmed by immunoblot analysis.

### Immunoblot analysis

Immunoblot analysis of whole cell extracts was performed as described in detail elsewhere^22^.

### Proteome analysis by label-free quantification based mass spectrometry

To study the effect of HDAC5 overexpression on selected cell lines, quadruplicates from individual culture dishes were prepared from RT112, VM-Cub1, SW1710, and UM-UC3 cells expressing HDAC5 as well as corresponding and HBLAK vector-only cells. Cells were harvested and protein lysates prepared in an aqueous urea-containing buffer (2 M thiourea, 7 M urea, 4% (w/v) 3-[(3-cholamidopropyl)dimethylammonio]-1-propanesulfonate, 30 mM Tris-HCl, pH 8.0) and prepared for mass spectrometric analysis, as described elsewhere^23^. Briefly, proteins were stacked in an acrylamide gel (about 4 mm running distance), subjected to silver staining, de-stained, reduced and alkylated and digested with trypsin. Resulting peptides were extracted from the gel and 500 ng peptides prepared in 0.1% trifluoroacetic acid for liquid chromatography and mass spectrometric analysis.

Here, first peptides were separated by liquid chromatography an Ultimate 3000 Rapid Separation liquid chromatography system was used for peptide separation over a two-hour gradient before analyzing peptides with a QExactive plus mass spectrometer in data dependent top ten mode as essentially as described^23^.

For spectra identification and precursor ion intensity-based quantification, the MaxQuant environment (version 1.6.0.16, MPI for Biochemistry, Planegg, Germany) was used with standard parameters. Spectra were matched against sequence data from the *Homo sapiens* reference proteome (UP000005640, 71567 entries, downloaded on August 28, 2017, from the UniProt Knowledgebase). Further search parameters and parameters for peptide and protein acceptance and quantification were essentially as described previously^23^. In brief, standard search parameters were applied with a few exceptions, including enabled label-free quantification as well as “match between runs”. Cysteine-carbamidomethylation was considered as fixed whereas protein N-terminal and lysine acetylation and methionine oxidation were considered as variable modifications.

### Sample preparation and RNA isolation for RNA-Seq

Cells were harvested with Trizol (manufacturer) and lysates were stored at −80 °C. Total RNA was then isolated by the Qiagen RNeasy Mini Kit (Qiagen, Hilden, Germany) with DNase treatment. RNA quality was checked by spectrophotometry.

### High throughput mRNA sequencing

Library preparation for RNA-Seq was performed according to the manufacturer’s protocol using the ‘TruSeq Stranded mRNA Library Prep Kit’ from Illumina®. Briefly, 250 ng total RNA were used for mRNA capturing, fragmentation, the synthesis of cDNA, adapter ligation and library amplification. Bead-purified libraries were normalized and finally sequenced on the HiSeq. 3000/4000 system (Illumina Inc. San Diego, USA) with a read setup of 1 × 150 bp. The bcl2fastq tool was used to convert the bcl files to fastq files as well for adapter trimming and demultiplexing.

## Data Records

The mass spectrometry proteomics data have been deposited to the ProteomeXchange Consortium (http://proteomecentral.proteomexchange.org) via the PRIDE partner repository^24^ with the dataset identifier PXD014448^25^. Mass spectrometric.raw files as well as the MaxQuant search result files (txt folder) have been uploaded.

The RNA-seq transcriptomics data (raw FASTQ files) have been deposited to the ArrayExpress repository^26^ (http://www.ebi.ac.uk/arrayexpress) under the accession number E-MTAB-8418^27^.

A summary of samples, data collection, experimentation, and accession numbers can be found in Table 1 and Table 2.

## Technical Validation

### Proteomic data

Proteomic analysis of quantitative data was carried out with Perseus (version 1.6.0.7, MPI for Biochemistry, Planegg, Germany) and within the R environment (R foundation for statistical computing). Here, only proteins showing at least two different peptides were considered and proteins showing at least three valid quantitative values in at least one group in the respective comparison. For the identification of HDAC5 affected proteins and differences between the cell lines, a two-way ANOVA followed by a Benjamini-Hochberg correction and by Tukey’s honest significance tests was carried out on log2 label-free quantification intensities after missing values were filled in with random values from a normal distribution (width: 0.3 standard deviations, downshift: 1.8 standard deviations). Additionally, Student’s t-test was calculated for pairwise comparisons of HDAC5-transduced and vector-only cells and cutoffs were determined by the significance analysis of microarrays method (S0 = 0.8, 5% false discovery rate).

**Fig. 2:**
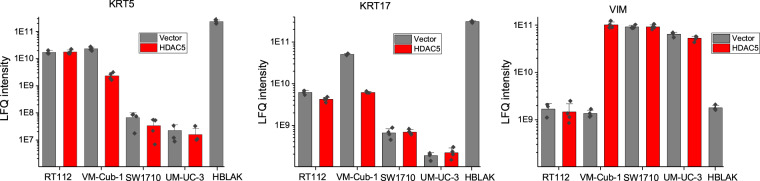
Analysis of Keratin 5, Keratin 17, and Vimentin protein abundance measured by quantitative mass spectrometry. Bar plot indicates the label-free quantification (LFQ) intensities of each protein in different cell lines. Data are represented as the mean with standard deviation. Individual data points are plotted as diamonds.

To further validate the mass spectrometry-based observations, we performed immunoblot analysis. Protein expression levels of proteins of interest (HDAC5 and other EMT markers), normalized to tubulin levels were confirmed by immunodetection as previously described in Jaguva Vasudevan *et al*.^10^. The protein abundance of KRT5, KRT17, and VIM measured by quantitative mass spectrometry strongly supports the cellular phenotypes and detection of KRT5 and VIM by immunoblotting (Fig. 2 and in our article^10^).

### Transcriptomic data

Total RNA of samples used for transcriptome analyses were quantified (Qubit RNA HS Assay, Thermo Fisher Scientific) and the quality and integrity were measured by capillary electrophoresis using the Fragment Analyzer and the Total RNA Standard Sensitivity Assay (Agilent Technologies, Inc. Santa Clara, USA). All samples in this study showed high quality RNA Quality Numbers (RQN; mean = 9.8, sample QC report is summarized in Supplementary files 1 and 2). Similarly, the quality of RNA-seq libraries was determined by capillary electrophoresis (Library QC report is summarized in Supplementary files 1 and 3).

Data analyses on fastq files were conducted with CLC Genomics Workbench (version 10.1.1, QIAGEN, Venlo. NL, USA). The reads of all probes were adapter-trimmed (Illumina TruSeq) and quality-trimmed (using the default parameters: bases below Q13 were trimmed from the end of the reads, ambiguous nucleotides maximal 2). Mapping was done against the *Homo sapiens* (hg38) (May 25, 2017) genome sequence. After grouping of samples (four biological replicates each, except for three for RT112) according to their respective experimental condition, multi-group comparisons were made and statistically determined using the Empirical Analysis of DGE (version 1.1, cutoff = 5). The resulting P values were corrected for multiple testing by FDR and Bonferroni-correction. A P value of ≤ 0.05 was considered significant (an overview of the sequencing data report is provided in Supplementary files 1).

**Fig. 3:**
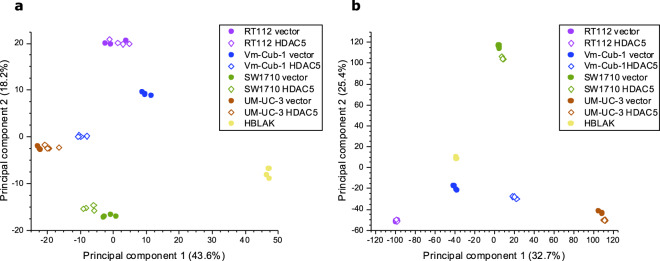
Principal component analysis (PCA) of (**a**) proteomic and (**b**) RNA-seq datasets. Each color represents a cell line. HDAC5 overexpressing cell lines are indicated by a diamond symbol, vector controls by a circle. Except for VM-Cub-1, all cell lines were tightly clustered, irrespective of HDAC5 expression. Vector- and HDAC5-expressing groups of VM-Cub-1 cell lines displayed the greatest variation in gene expression among the investigated UC lines.

To further ensure the quality of data and, especially to determine the association between samples, we performed principal component analysis (PCA) of the proteome and RNA-seq datasets (Fig. 3). As shown in the gene expression PCA plot, we observed a strong clustering of the replicates and separation among different cell lines, regardless of vector-only or HDAC5 transgene expression, except for VM-Cub-1. Rather in keeping with observed pronounced morphological changes^10^, the greatest variation in gene expression was observed between the VM-Cub-1 variants transduced with vector-only and HDAC5, respectively (Fig. 3). Furthermore, an excel file containing summary and significant differential expressed genes of the six comparisons are provided in Supplementary file 4.

Of note, compared to parental UC cell lines that displayed low or undetectable levels of *HDAC5* mRNA, in transduced cell lines RT112, VM-Cub1, SW1710, and UM-UC3 cells, we detected the *HDAC5* mRNA expression ranging from 76, 186, 195, and 205-fold higher, respectively. Indeed, this is an average value derived from polyclonal cell populations, in fact, the expression levels in individual cells might vary, and could be even more elevated. A direct comparison of this to physiological upregulated *HDAC5* levels observed in *HDAC5* expressing tumors appears a lot higher. Here, mRNA levels of 2 to 8-fold higher as in corresponding benign tissues were observed (see references in Table 3). However, HDAC5 expression is generally low in both urothelial cancer tissue (according to proteinatlas.org, based on TCGA dataset, *HDAC5* transcript levels rank lower, the 14^th^ out of 17 tested cancer types) as well as in urothelial cancer cells (mRNA level 2-fold downregulated as compared to normal uroepithelial cells^18^) and, most importantly, HDAC5 protein expression is diminished in most urothelial cancer cell lines^10^. In particular, the last point suggests that in addition to the RNA expression levels, posttranscriptional regulation and RNA turnover mechanisms are likely to contribute and responsible for an overall low expression level of HDAC5 in UC. Therefore, we would like to emphasize that the expression of relatively higher *HDAC5* transcripts in the tested cell lines may not be reflected in excessive HDAC5 expression.

**Table 3:** Summary of HDAC5 expression status in different cancers.

Tumor types	Expression status	Detection methods	References
Lung cancer	Upregulation	Western Blot, qRT-PCR	Zhong L *et al*.^28^
	4-fold higher		
	4-fold higher	qRT-PCR	Liu C *et al*.^29^
Colorectal cancer	Upregulation	qRT-PCR	Stypula-Cyrus Y *et al*.^30^
	2.5-fold higher		
Glioma	Upregulation	Western Blot, qRT-PCR	Liu Q *et al*.^31^
	2-fold higher		
Osteosarcoma	Upregulation	Western Blot, qRT-PCR	Chen J *et al*.^32^
	8-fold higher		
Wilms’ tumor	Upregulation	Western Blot, qRT-PCR	Cao X *et al*.^33^
	4-fold higher		

## Usage Notes

Analyses of parts of these datasets have been published before^10^, where we reported the effect of HDAC5 expression on cellular phenotypes such as proliferation, clonogenic potency, and migration. Intriguingly, in VM-Cub-1, HDAC5 expression dramatically triggered an epithelial-mesenchymal transition (EMT)^10^. Our proteome and transcriptome data backed and detailed the molecular changes. Specifically, they hinted at the involvement of TGFβ. However, we have not systematically analyzed additional differences among these bladder cancer cell lines. For instance, the HBLAK cells seem to rely more on the pentose phosphate pathway, whereas the other cell lines used more oxidative phosphorylation. Thus, our omics datasets from five urothelial bladder cancer cell lines could be utilized, among other prospects, (a) to identify and validate novel target genes or proteins associated with UC; (b) to uncover new metabolic pathway(s) and signaling network(s) in the direction of identifying potential target for cancer therapy, (c) to study epigenetic regulation, protein modification, and cellular consequences as a result of HDAC5 expression.

## Supplementary information

Supplementary File 1. RNA-seq data overview

Supplementary File 2. QC report of RNA samples

Supplementary File 3. QC report of library

Supplementary File 4. QC report of RNA samples

## Data Availability

The following software and versions were used for quality control and data processing: 1. Proteomic analysis: Perseus (version 1.6.0.7, MPI for Biochemistry) and within the R environment (R foundation for statistical computing). 2. Spectra identification and quantification: MaxQuant environment (version 1.6.0.16, MPI for Biochemistry). 3. Reference proteome: *Homo sapiens* reference proteome (UP000005640, 71567 entries, downloaded on August 28, 2017, from the UniProt database). 4. Transcriptomic data analysis: CLC Genomics Workbench (version 10.1.1, QIAGEN). Empirical Analysis of DGE (version 1.1, cutoff = 5) was used for multi-group comparisons and statistics. 5. Adapter trimming and demultiplexing: bcl2fastq tool. 6. RNA-seq mapping was done against the *Homo sapiens* (hg38) (May 25, 2017) genome sequence.
