# Supplementary material for: Proteomic and transcriptomic profiles of human urothelial cancer cells with histone deacetylase 5 overexpression
Source: Sci Data. 2022 May 27;9:240. doi: 10.1038/s41597-022-01319-0 (PMC9142574; doi:10.1038/s41597-022-01319-0)
Supplement: Supplementary file 1 — RNA-seq data overview [file 41597_2022_1319_MOESM1_ESM.pdf]

GGTACTCT Genomics &
CCAUGAGAC Transcriptomics
Laboratory

Sequencing Data Report

28.08.2017

1. Basic Information

BMFZ GTL contact information for NGS

Hotline: 0211 - 81 15811/14367
eMail: BMFZ-NGS@hhu.de

**ProjectID**	116-NGS-270617-0
**Customer**	PD Dr. Niegisch / Prof. Schulz
**Company/Institute**	Forschungslabor Urologie
**Library Preparation Kit**	TruSeq Stranded mRNA Library Prep Kit
**Sequencing Chemistry**	PE-410-1001 HiSeq® 3000/4000 PE Cluster Kit<br>FC-410-1002 HiSeq® 3000/4000 SBS Kit (150 cycles)
**Run Parameters**	SR 1x150bp
**Instrument Type**	HiSeq3000/4000
**Type of Analysis**	Fastq only

**Sample receipt:**	**27.06.2017**	**36 samples**
**Original Sample QC:**	**29.06.2017 – 30.06.2017**	**QC passed**<br>(as agreed upon with collaboration partner/customer)
**Library Preparation:**	**24.07.2017 – 26.07.2017**	**QC passed**
**Sequencing run:**	**15.08.2017 – 18.08.2017**	**QC passed**
**Data transfer:**	**tba**	

Comments:

- **average reads per sample = 30.61 million**
- **the sample with the lowest read count of 22.32 million reads is RT112_vector2 (see page 13)**

2. Original Sample QC

Remarks:

- The 260/280 and 260/230 ratios were determined by UV-Spectroscopy (NanoDrop 1000).
- Samples were diluted to ~5 ng/µL to fit into further downstream application. Concentration check with fluorometric assay. See below.
- The concentration of RNA was determined by Qubit RNA HS Assay, fluorescence-based quantification.
- Size distribution and quality of the RNA was checked by Fragment Analyzer using RNA Standard Sensitivity Assay. RNA Quality Number (RQN) indicates the Quality of RNA.
- The color code of the figures is due to the measurement setup and has therefore no further relevance (see below).

#	Sample	Concentration ND [ng/µL]	260/280	260/230	Concentration (dilution) Qubit [ng/µL]	RQN
1	RT112+vector 1	48.83	1.94	1.81	5.83	10.0
2	RT112+vector 2	52.44	1.82	2.29	6.44	10.0
3	RT112+vector 3	49.87	1.88	1.82	5.97	10.0
4	RT112+vector 4	50.28	1.89	1.85	5.83	10.0
5	RT112+HD5 1	47.76	1.96	2.26	5.64	10.0
6	RT112+HD5 2	48.54	1.90	2.26	5.92	10.0
7	RT112+HD5 3	49.31	1.87	2.25	5.64	10.0
8	RT112+HD5 4	48.39	1.99	1.62	5.98	10.0
9	VM-Cub-1+ vector 1	48.71	1.93	2.14	5.35	10.0
10	VM-Cub-1+ vector 2	46.12	1.92	2.13	5.06	9.8
11	VM-Cub-1+ vector 3	46.14	2.00	0.95	5.30	10.0
12	VM-Cub-1+ vector 4	47.29	1.91	2.29	5.67	10.0
13	VM-Cub-1+HD5 1	47.15	1.95	1.82	5.40	10.0
14	VM-Cub-1+HD5 2	45.76	1.92	2.15	5.16	9.7
15	VM-Cub-1+HD5 3	45.43	1.96	1.38	5.27	10.0
16	VM-Cub-1+HD5 4	46.84	1.93	2.08	5.49	10.0
17	SW1710+vector 1	48.06	1.92	2.24	5.51	9.2
18	SW1710+vector 2	46.28	1.92	2.05	5.44	9.1
19	SW1710+vector 3	46.64	1.89	2.20	5.41	9.1
20	SW1710+vector 4	44.24	1.95	1.58	5.34	9.3
21	SW1710+HD5 1	46.81	1.92	1.82	5.54	10.0
22	SW1710+HD5 2	48.57	1.92	2.26	5.69	9.6
23	SW1710+HD5 3	49.16	1.94	1.73	5.83	10.0
24	SW1710+HD5 4	51.73	1.88	2.03	6.02	10.0
25	UM-UC-3+vector 1	51.28	1.86	2.23	5.47	9.3
26	UM-UC-3+vector 2	49.47	1.85	2.06	5.80	10.0
27	UM-UC-3+vector 3	47.86	1.86	2.28	5.43	10.0
28	UM-UC-3+vector 4	46.35	1.88	2.21	5.90	10.0
29	UM-UC-3+HD5 1	51.48	1.84	2.33	5.89	10.0
30	UM-UC-3+HD5 2	50.46	1.82	2.25	5.68	10.0
31	UM-UC-3+HD5 3	52.15	1.89	1.86	6.05	10.0
32	UM-UC-3+HD5 4	53.28	1.87	1.97	6.83	10.0
33	HBLAK+vector 1	49.24	1.90	2.09	5.71	9.9
34	HBLAK+vector 2	49.32	1.91	2.16	6.28	10.0
35	HBLAK+vector 3	45.98	1.89	0.90	5.83	10.0
36	HBLAK+vector 4	47.28	1.97	2.13	5.48	9.5

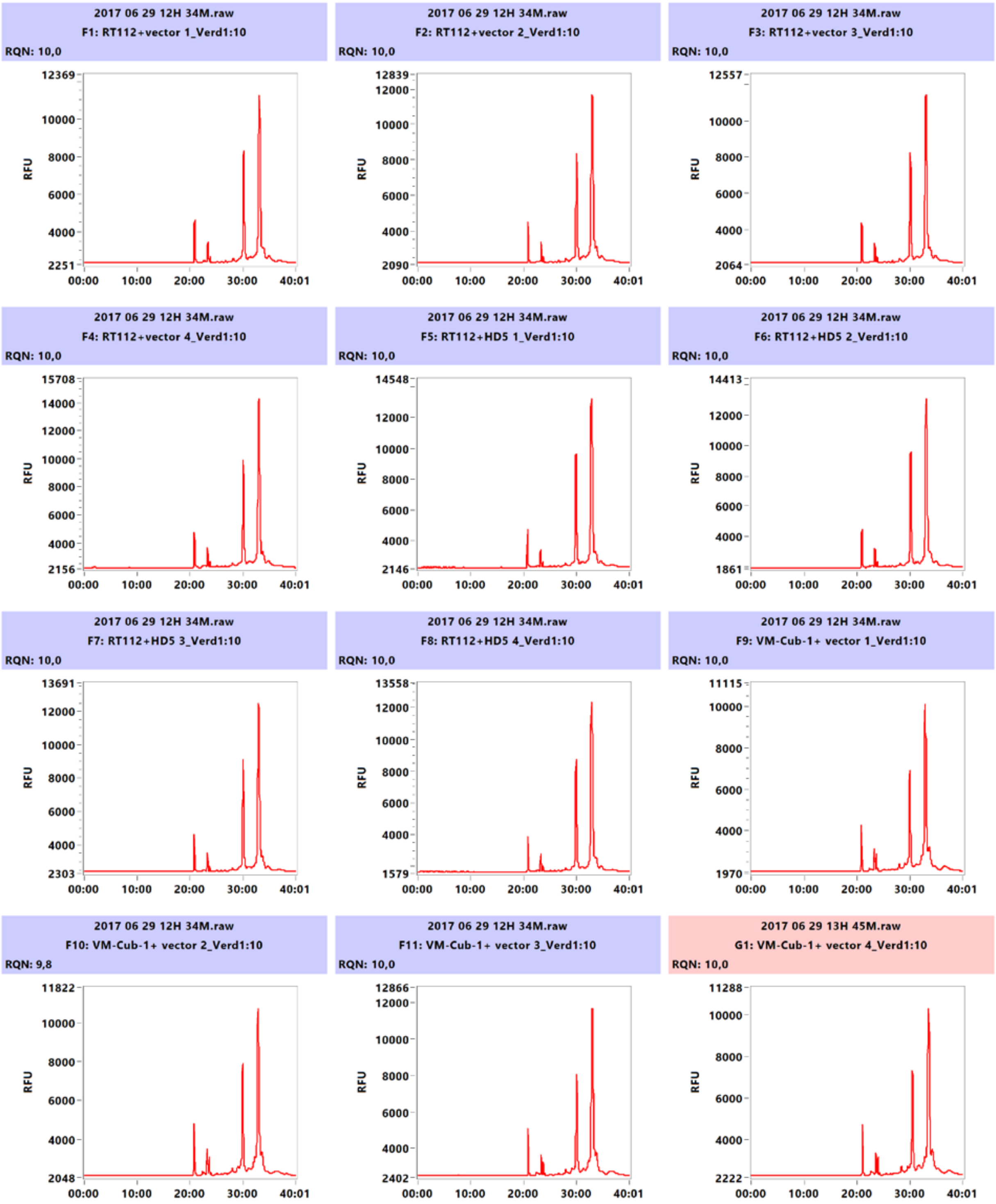
2017 06 29 12H 34M.raw
F1: RT112+vector 1_Verd1:10
RQN: 10,0
12369
10000
8000
6000
4000
2251
RFU
00:00
10:00
20:00
30:00
40:01
2017 06 29 12H 34M.raw
F2: RT112+vector 2_Verd1:10
RQN: 10,0
12839
12000
10000
8000
6000
4000
2090
RFU
00:00
10:00
20:00
30:00
40:01
2017 06 29 12H 34M.raw
F3: RT112+vector 3_Verd1:10
RQN: 10,0
12557
10000
8000
6000
4000
2064
RFU
00:00
10:00
20:00
30:00
40:01
2017 06 29 12H 34M.raw
F4: RT112+vector 4_Verd1:10
RQN: 10,0
15708
14000
12000
10000
8000
6000
4000
2156
RFU
00:00
10:00
20:00
30:00
40:01
2017 06 29 12H 34M.raw
F5: RT112+HD5 1_Verd1:10
RQN: 10,0
14548
12000
10000
8000
6000
4000
2146
RFU
00:00
10:00
20:00
30:00
40:01
2017 06 29 12H 34M.raw
F6: RT112+HD5 2_Verd1:10
RQN: 10,0
14413
12000
10000
8000
6000
4000
1861
RFU
00:00
10:00
20:00
30:00
40:01
2017 06 29 12H 34M.raw
F7: RT112+HD5 3_Verd1:10
RQN: 10,0
13691
12000
10000
8000
6000
4000
2303
RFU
00:00
10:00
20:00
30:00
40:01
2017 06 29 12H 34M.raw
F8: RT112+HD5 4_Verd1:10
RQN: 10,0
13558
12000
10000
8000
6000
4000
1579
RFU
00:00
10:00
20:00
30:00
40:01
2017 06 29 12H 34M.raw
F9: VM-Cub-1+ vector 1_Verd1:10
RQN: 10,0
11115
10000
8000
6000
4000
1970
RFU
00:00
10:00
20:00
30:00
40:01
2017 06 29 12H 34M.raw
F10: VM-Cub-1+ vector 2_Verd1:10
RQN: 9,8
11822
10000
8000
6000
4000
2048
RFU
00:00
10:00
20:00
30:00
40:01
2017 06 29 12H 34M.raw
F11: VM-Cub-1+ vector 3_Verd1:10
RQN: 10,0
12866
12000
10000
8000
6000
4000
2402
RFU
00:00
10:00
20:00
30:00
40:01
2017 06 29 13H 45M.raw
G1: VM-Cub-1+ vector 4_Verd1:10
RQN: 10,0
11288
10000
8000
6000
4000
2222
RFU
00:00
10:00
20:00
30:00
40:01

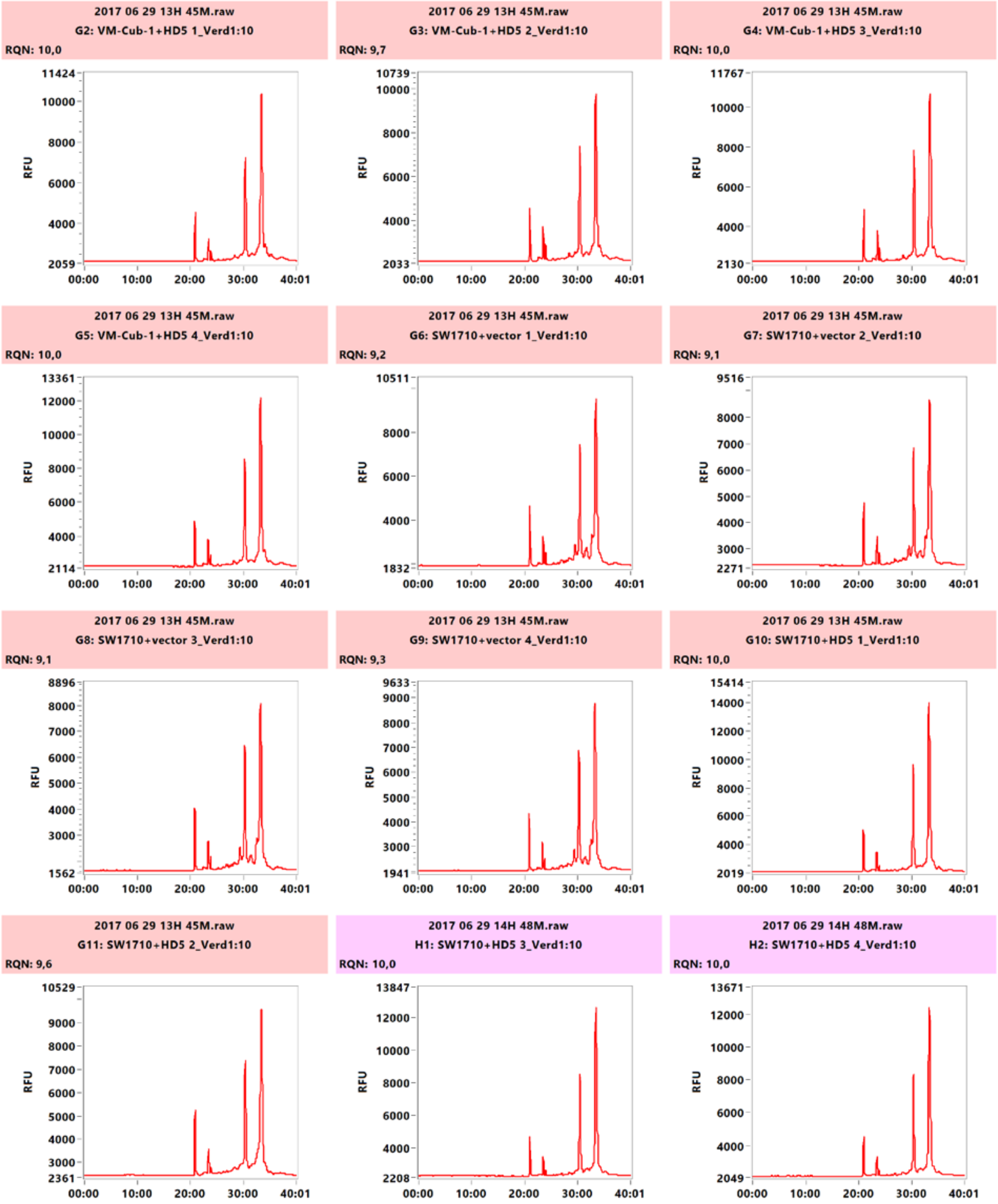
2017 06 29 13H 45M.raw
G2: VM-Cub-1+HD5 1_Verd1:10
RQN: 10,0
11424
10000
8000
6000
4000
2059
RFU
00:00 10:00 20:00 30:00 40:01
2017 06 29 13H 45M.raw
G3: VM-Cub-1+HD5 2_Verd1:10
RQN: 9,7
10739
10000
8000
6000
4000
2033
RFU
00:00 10:00 20:00 30:00 40:01
2017 06 29 13H 45M.raw
G4: VM-Cub-1+HD5 3_Verd1:10
RQN: 10,0
11767
10000
8000
6000
4000
2130
RFU
00:00 10:00 20:00 30:00 40:01
2017 06 29 13H 45M.raw
G5: VM-Cub-1+HD5 4_Verd1:10
RQN: 10,0
13361
12000
10000
8000
6000
4000
2114
RFU
00:00 10:00 20:00 30:00 40:01
2017 06 29 13H 45M.raw
G6: SW1710+vector 1_Verd1:10
RQN: 9,2
10511
8000
6000
4000
1832
RFU
00:00 10:00 20:00 30:00 40:01
2017 06 29 13H 45M.raw
G7: SW1710+vector 2_Verd1:10
RQN: 9,1
9516
8000
7000
6000
5000
4000
3000
2271
RFU
00:00 10:00 20:00 30:00 40:01
2017 06 29 13H 45M.raw
G8: SW1710+vector 3_Verd1:10
RQN: 9,1
8896
8000
7000
6000
5000
4000
3000
1562
RFU
00:00 10:00 20:00 30:00 40:01
2017 06 29 13H 45M.raw
G9: SW1710+vector 4_Verd1:10
RQN: 9,3
9633
9000
8000
7000
6000
5000
4000
3000
1941
RFU
00:00 10:00 20:00 30:00 40:01
2017 06 29 13H 45M.raw
G10: SW1710+HD5 1_Verd1:10
RQN: 10,0
15414
14000
12000
10000
8000
6000
4000
2019
RFU
00:00 10:00 20:00 30:00 40:01
2017 06 29 13H 45M.raw
G11: SW1710+HD5 2_Verd1:10
RQN: 9,6
10529
9000
8000
7000
6000
5000
4000
3000
2361
RFU
00:00 10:00 20:00 30:00 40:01
2017 06 29 14H 48M.raw
H1: SW1710+HD5 3_Verd1:10
RQN: 10,0
13847
12000
10000
8000
6000
4000
2208
RFU
00:00 10:00 20:00 30:00 40:01
2017 06 29 14H 48M.raw
H2: SW1710+HD5 4_Verd1:10
RQN: 10,0
13671
12000
10000
8000
6000
4000
2049
RFU
00:00 10:00 20:00 30:00 40:01

2017 06 30 08H 21M.raw
G1: UM-UC-3+vector 1_Verd1:10_w1
RQN: 9,3

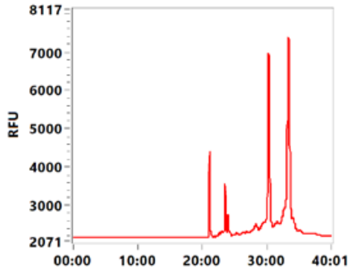

2017 06 29 14H 48M.raw
H4: UM-UC-3+vector 2_Verd1:10
RQN: 10,0

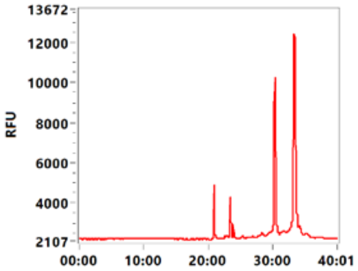

2017 06 29 14H 48M.raw
H5: UM-UC-3+vector 3_Verd1:10
RQN: 10,0

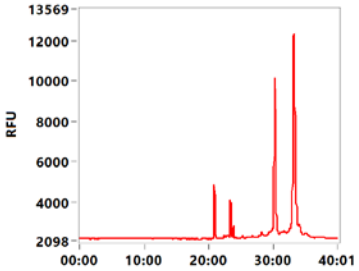

2017 06 29 14H 48M.raw
H6: UM-UC-3+vector 4_Verd1:10
RQN: 10,0

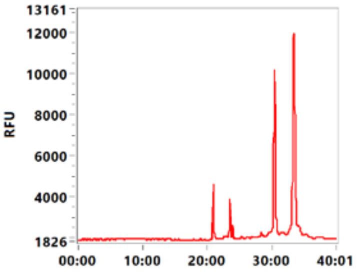

2017 06 29 14H 48M.raw
H7: UM-UC-3+HD5 1_Verd1:10
RQN: 10,0

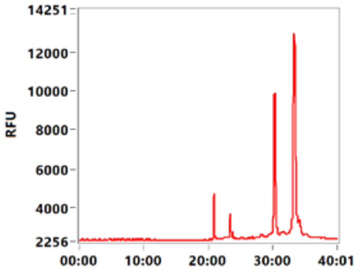

2017 06 29 14H 48M.raw
H8: UM-UC-3+HD5 2_Verd1:10
RQN: 10,0

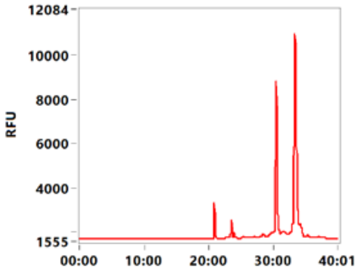

2017 06 29 14H 48M.raw
H9: UM-UC-3+HD5 3_Verd1:10
RQN: 10,0

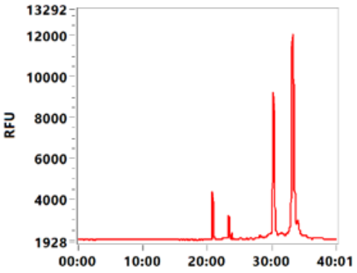

2017 06 29 14H 48M.raw
H10: UM-UC-3+HD5 4_Verd1:10
RQN: 10,0

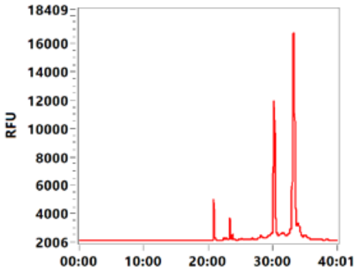

2017 06 29 14H 48M.raw
H11: HBLAK+vector 1_Verd1:10
RQN: 9,9

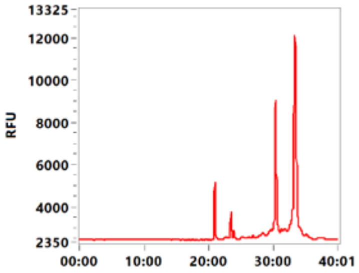

2017 06 29 15H 52M.raw
F1: HBLAK+vector 2_Verd1:10
RQN: 10,0

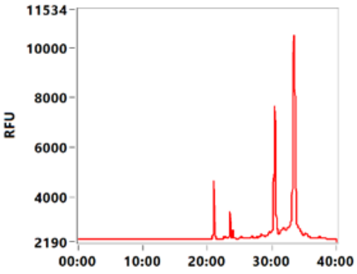

2017 06 29 15H 52M.raw
F2: HBLAK+vector 3_Verd1:11
RQN: 10,0

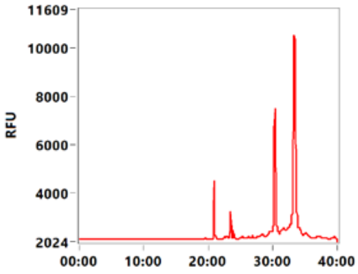

2017 06 29 15H 52M.raw
F3: HBLAK+vector 4_Verd1:12
RQN: 9,5

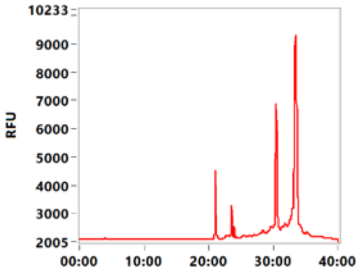

2017 06 29 13H 45M.raw
G12: Ladder

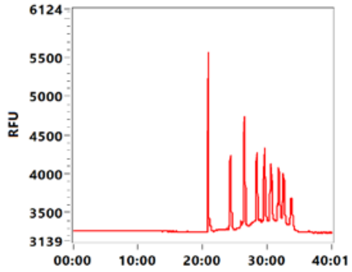

3. Library preparation

1. Normalization: Dilution to 250 ng in 50 µL
2. Library Preparation: 50 µL of total RNA sample input. The Library preparation was performed according to the TruSeq® Stranded mRNA Sample Preparation Guide (Part # 15031047 Rev. E).

4. Library QC

1. The library was first checked by NanoDrop.
2. 1:10 dilution in Resuspension Buffer (90 µL RSB + 10 µL library) to bring samples into Fragment Analyzer concentration range (<5 ng/µL).
3. Post library preparation QC was carried out using the Fragment Analyzer High Sensitivity NGS Fragment Analysis Kit (1 bp – 6,000 bp), DNF-474.
   The color code of the figures is due to the measurement setup and has therefore no further relevance (see below).
4. Library normalization was performed using Qubit DNA HS Assay for concentration determination and Fragment Analyzer DNF-474 for average size information (dilution to 2 nM). Finally each time 12 of the 36 samples were pooled together into 3 pools in order to sequence them on 3 HiSeq3000 lanes.

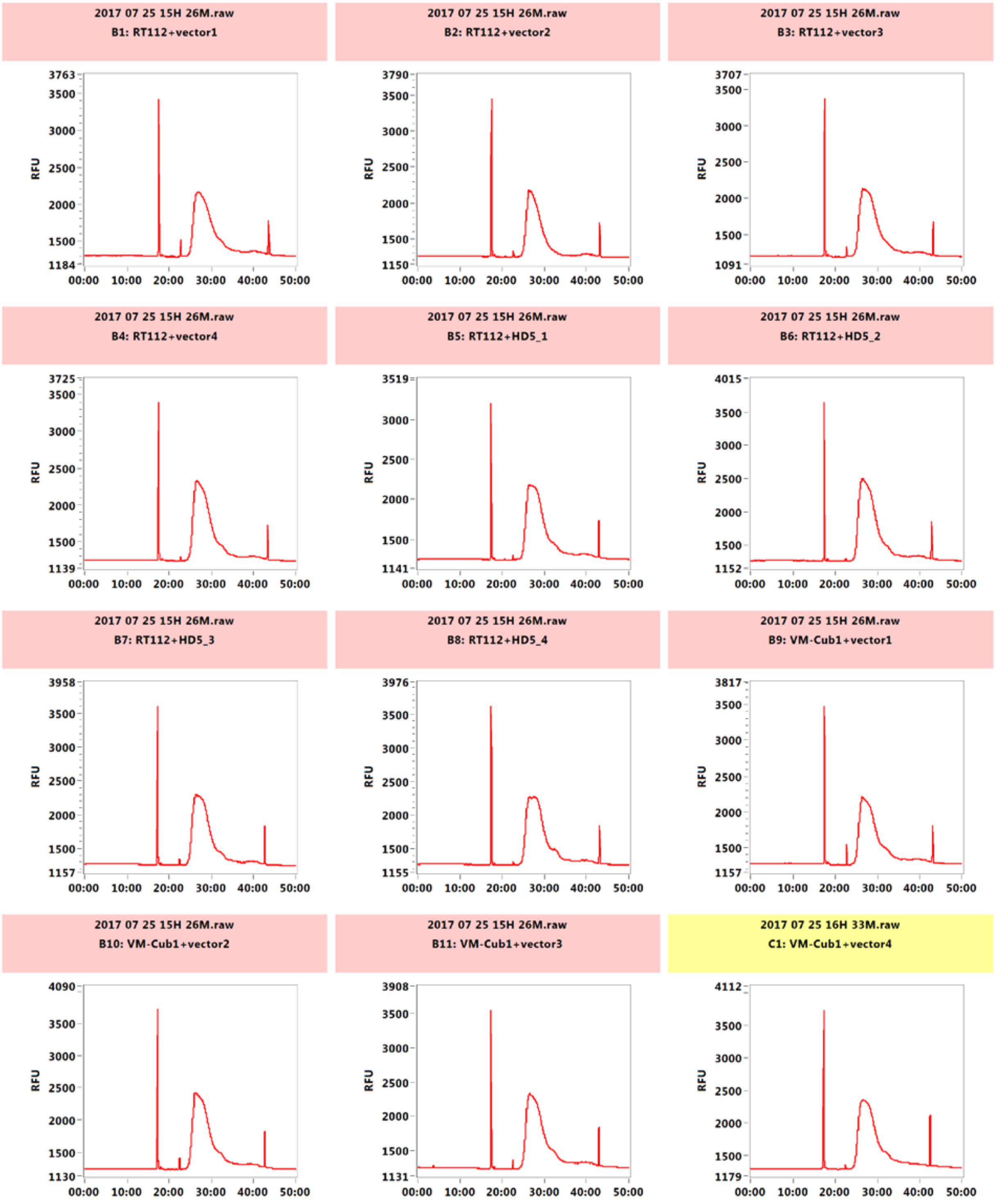
2017 07 25 15H 26M.raw
B1: RT112+vector1
3763 3500 3000 2500 2000 1500 1184
RFU
00:00 10:00 20:00 30:00 40:00 50:00
2017 07 25 15H 26M.raw
B2: RT112+vector2
3790 3500 3000 2500 2000 1500 1150
RFU
00:00 10:00 20:00 30:00 40:00 50:00
2017 07 25 15H 26M.raw
B3: RT112+vector3
3707 3500 3000 2500 2000 1500 1091
RFU
00:00 10:00 20:00 30:00 40:00 50:00
2017 07 25 15H 26M.raw
B4: RT112+vector4
3725 3500 3000 2500 2000 1500 1139
RFU
00:00 10:00 20:00 30:00 40:00 50:00
2017 07 25 15H 26M.raw
B5: RT112+HD5_1
3519 3000 2500 2000 1500 1141
RFU
00:00 10:00 20:00 30:00 40:00 50:00
2017 07 25 15H 26M.raw
B6: RT112+HD5_2
4015 3500 3000 2500 2000 1500 1152
RFU
00:00 10:00 20:00 30:00 40:00 50:00
2017 07 25 15H 26M.raw
B7: RT112+HD5_3
3958 3500 3000 2500 2000 1500 1157
RFU
00:00 10:00 20:00 30:00 40:00 50:00
2017 07 25 15H 26M.raw
B8: RT112+HD5_4
3976 3500 3000 2500 2000 1500 1155
RFU
00:00 10:00 20:00 30:00 40:00 50:00
2017 07 25 15H 26M.raw
B9: VM-Cub1+vector1
3817 3500 3000 2500 2000 1500 1157
RFU
00:00 10:00 20:00 30:00 40:00 50:00
2017 07 25 15H 26M.raw
B10: VM-Cub1+vector2
4090 3500 3000 2500 2000 1500 1130
RFU
00:00 10:00 20:00 30:00 40:00 50:00
2017 07 25 15H 26M.raw
B11: VM-Cub1+vector3
3908 3500 3000 2500 2000 1500 1131
RFU
00:00 10:00 20:00 30:00 40:00 50:00
2017 07 25 16H 33M.raw
C1: VM-Cub1+vector4
4112 3500 3000 2500 2000 1500 1179
RFU
00:00 10:00 20:00 30:00 40:00 50:00

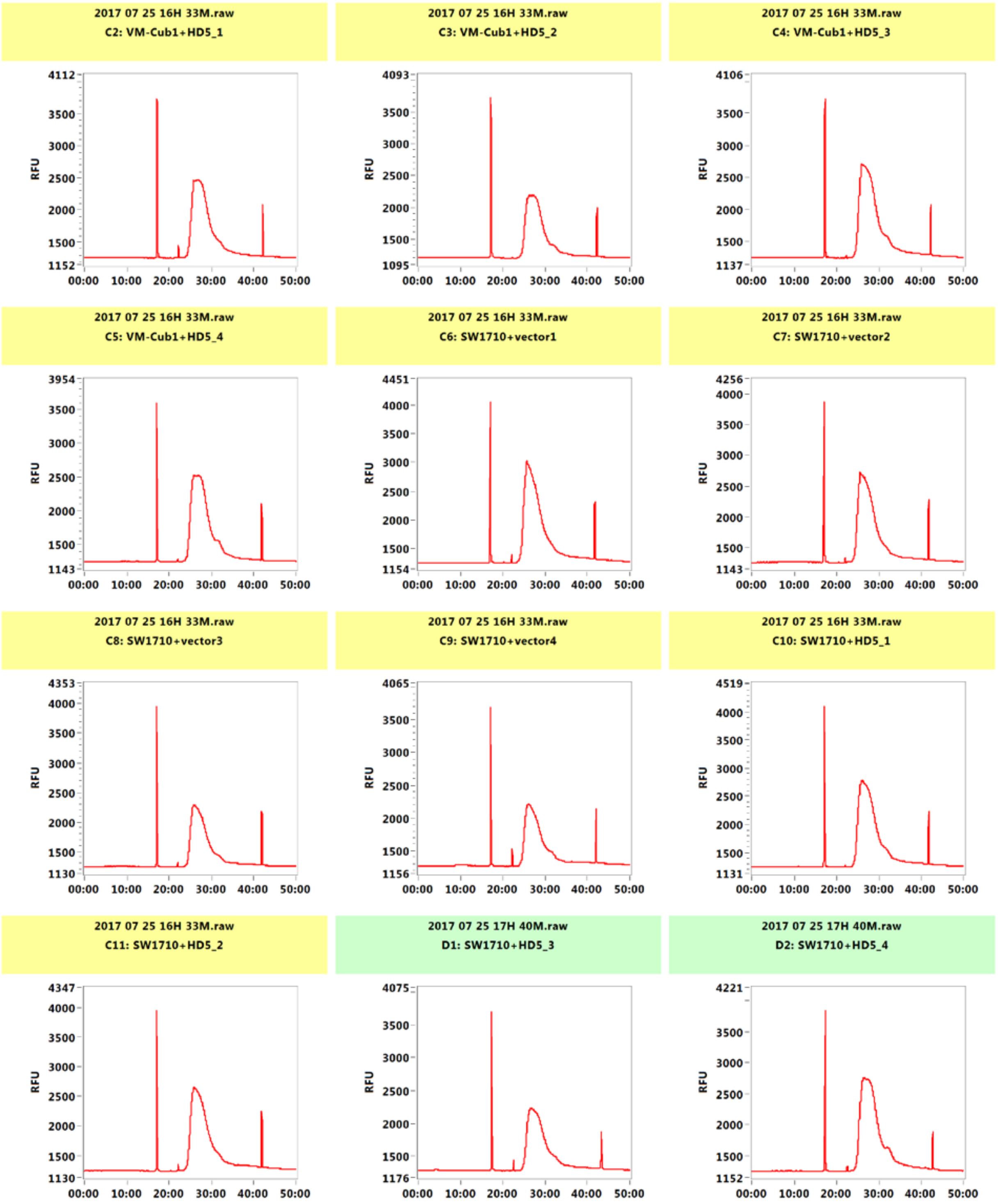
2017 07 25 16H 33M.raw
C2: VM-Cub1+HD5_1
2017 07 25 16H 33M.raw
C3: VM-Cub1+HD5_2
2017 07 25 16H 33M.raw
C4: VM-Cub1+HD5_3
2017 07 25 16H 33M.raw
C5: VM-Cub1+HD5_4
2017 07 25 16H 33M.raw
C6: SW1710+vector1
2017 07 25 16H 33M.raw
C7: SW1710+vector2
2017 07 25 16H 33M.raw
C8: SW1710+vector3
2017 07 25 16H 33M.raw
C9: SW1710+vector4
2017 07 25 16H 33M.raw
C10: SW1710+HD5_1
2017 07 25 16H 33M.raw
C11: SW1710+HD5_2
2017 07 25 17H 40M.raw
D1: SW1710+HD5_3
2017 07 25 17H 40M.raw
D2: SW1710+HD5_4
RFU
00:00 10:00 20:00 30:00 40:00 50:00

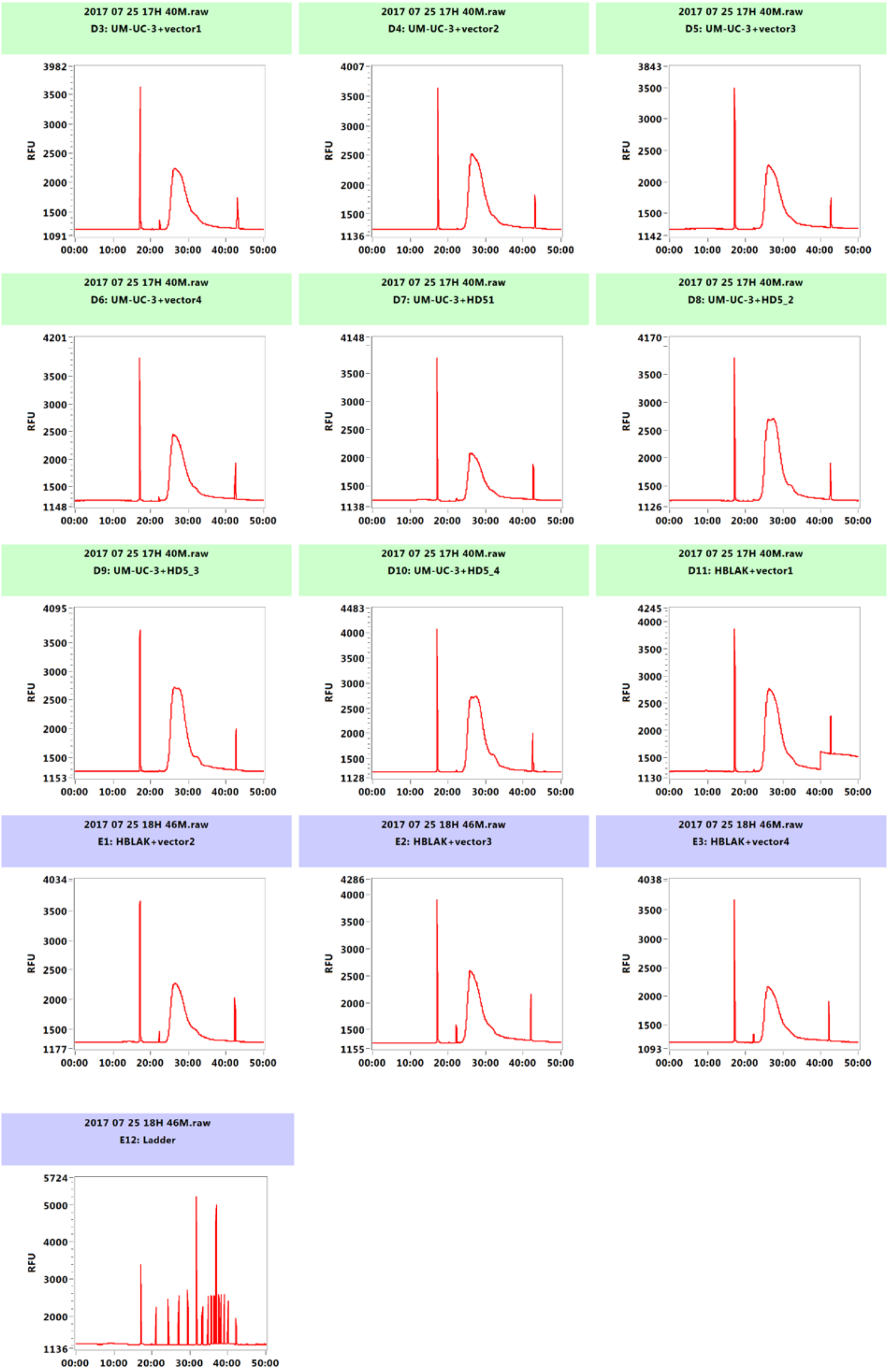
2017 07 25 17H 40M.raw
D3: UM-UC-3+vector1
2017 07 25 17H 40M.raw
D4: UM-UC-3+vector2
2017 07 25 17H 40M.raw
D5: UM-UC-3+vector3
2017 07 25 17H 40M.raw
D6: UM-UC-3+vector4
2017 07 25 17H 40M.raw
D7: UM-UC-3+HD51
2017 07 25 17H 40M.raw
D8: UM-UC-3+HD5_2
2017 07 25 17H 40M.raw
D9: UM-UC-3+HD5_3
2017 07 25 17H 40M.raw
D10: UM-UC-3+HD5_4
2017 07 25 17H 40M.raw
D11: HBLAK+vector1
2017 07 25 18H 46M.raw
E1: HBLAK+vector2
2017 07 25 18H 46M.raw
E2: HBLAK+vector3
2017 07 25 18H 46M.raw
E3: HBLAK+vector4
2017 07 25 18H 46M.raw
E12: Ladder
RFU
00:00 10:00 20:00 30:00 40:00 50:00

5. Sequencing QC

Sequencing run ID: 170816_J00150_0031_AHLJNMBBXX

Remarks:

- PhiX SpikeIn ~ 1%; represented by the aligned (%) value
- Error Rate has to be < 3%
- Phas/Prephas has to be < 0.3%
- Q30% has to be > 75%

**Run Summary**					
**Lane**	**Level**	**Aligned (%)**	**Error Rate (%)**	**Phas/Prephas (%)**	**% >= Q30**
3	Foreward read	1.70 +/- 0.22	0.31 +/- 0.15	0.103 / 0.064	95.01
6	Foreward read	1.97 +/- 0.04	0.27 +/- 0.14	0.104 / 0.067	94.60
7	Foreward read	1.86 +/- 0.06	0.25 +/- 0.05	0.104 / 0.069	94.66
3	Reverse read	0.00 +/- 0.00	0.00 +/- 0.00	0.000 / 0.000	95.59
6	Reverse read	0.00 +/- 0.00	0.00 +/- 0.00	0.000 / 0.000	95.13
7	Reverse read	0.00 +/- 0.00	0.00 +/- 0.00	0.000 / 0.000	95.01

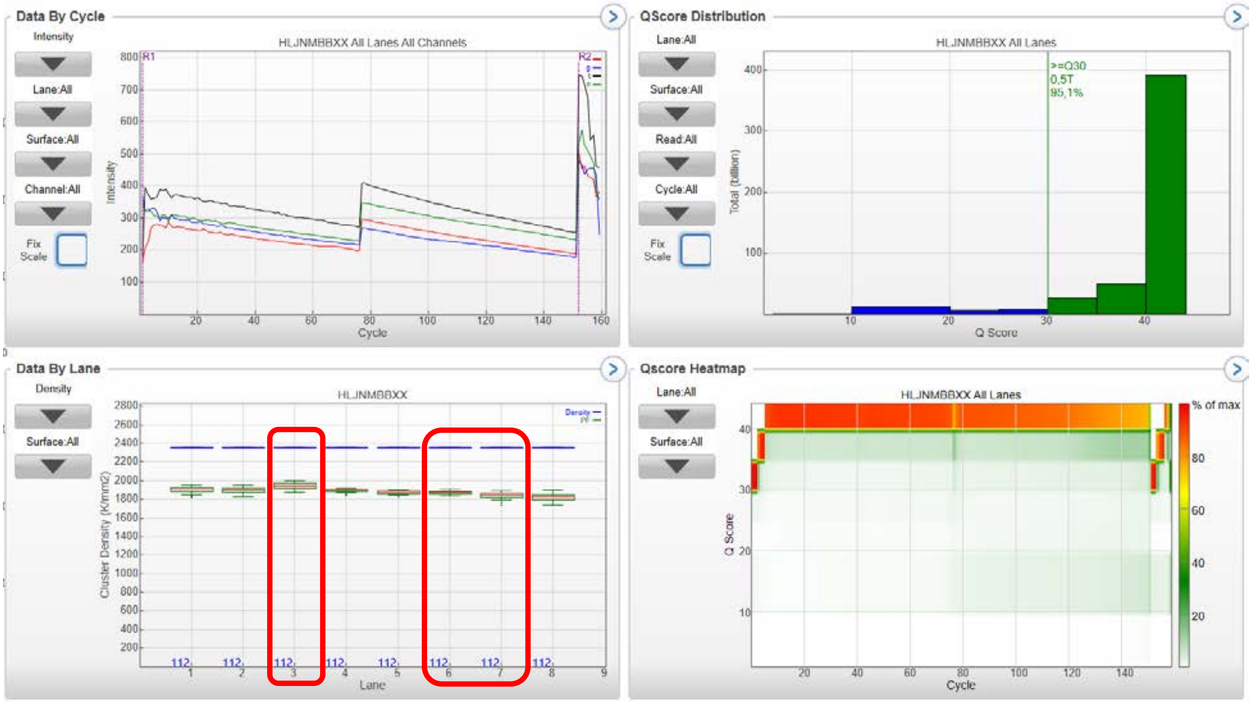

Lane 3 **Lane 6 - 7**

- All samples of this project were loaded/analyzed on lane 3, 6 and 7.

6. Reads mapped to index/ Index distribution

Lane	Sample	Barcode sequence	PF Clusters	% of the lane	% Perfect barcode	Yield (Mbases)	% >= Q30 bases	Mean Quality Score
3	HBLAK_vector1	CGTACG	29,499,846	7.39	100	4,454	95.43	39.33
3	HBLAK_vector2	GAGTGG	33,005,335	8.26	100	4,984	93.81	38.90
3	HBLAK_vector3	ACTGAT	40,816,980	10.22	100	6,163	93.45	38.81
3	HBLAK_vector4	ATTCCT	33,598,294	8.41	100	5,073	94.18	39.00
3	UM-UC-3_HD5_1	TAGCTT	31,454,551	7.87	100	4,750	95.27	39.29
3	UM-UC-3_HD5_2	GGCTAC	32,180,589	8.06	100	4,859	95.72	39.41
3	UM-UC-3_HD5_3	GTGGCC	35,056,571	8.78	100	5,294	95.98	39.48
3	UM-UC-3_HD5_4	GTTTCG	29,873,573	7.48	100	4,511	95.52	39.35
3	UM-UC-3_vector1	ATCACG	29,167,280	7.30	100	4,404	93.70	38.87
3	UM-UC-3_vector2	TTAGGC	26,066,076	6.53	100	3,936	96.02	39.49
3	UM-UC-3_vector3	ACTTGA	29,681,809	7.43	100	4,482	95.87	39.45
3	UM-UC-3_vector4	GATCAG	28,537,691	7.14	100	4,309	95.27	39.29
3	Undetermined	unknown	20,500,043	5.13	100	3,096	93.31	38.70
6	RT112_HD5_1	TAGCTT	33,360,147	8.68	100	5,037	94.97	39.20
6	RT112_HD5_2	GGCTAC	29,034,082	7.56	100	4,384	95.53	39.34
6	RT112_HD5_3	GTGGCC	32,093,808	8.36	100	4,846	95.37	39.30
6	RT112_HD5_4	GTTTCG	29,250,662	7.62	100	4,417	94.88	39.17
6	RT112_vector1	ATCACG	29,268,392	7.62	100	4,420	93.29	38.75
6	RT112_vector2	TTAGGC	22,321,159	5.81	100	3,370	94.91	39.19
6	RT112_vector3	ACTTGA	28,864,464	7.51	100	4,359	94.60	39.10
6	RT112_vector4	GATCAG	24,911,410	6.49	100	3,762	94.96	39.20
6	VM-Cub1_vector1	CGTACG	33,252,592	8.66	100	5,021	93.22	38.73
6	VM-Cub1_vector2	GAGTGG	35,575,795	9.26	100	5,372	94.07	38.95
6	VM-Cub1_vector3	ACTGAT	33,871,167	8.82	100	5,115	94.21	39.00
6	VM-Cub1_vector4	ATTCCT	31,998,304	8.33	100	4,832	94.73	39.13
6	Undetermined	unknown	20,314,102	5.29	100	3,067	93.46	38.76
6	SW1710_HD5_1	CGTACG	30,038,907	7.93	100	4,536	95.26	39.25
6	SW1710_HD5_2	GAGTGG	31,722,731	8.37	100	4,790	94.83	39.14
6	SW1710_HD5_3	ACTGAT	36,772,364	9.70	100	5,553	93.99	38.92
7	SW1710_HD5_4	ATTCCT	31,245,081	8.24	100	4,718	94.75	39.12
7	SW1710_vector1	TAGCTT	31,187,986	8.23	100	4,709	94.70	39.11
7	SW1710_vector2	GGCTAC	28,951,573	7.64	100	4,372	94.79	39.13
7	SW1710_vector3	GTGGCC	31,695,904	8.36	100	4,786	94.69	39.10
7	SW1710_vector4	GTTTCG	27,248,075	7.19	100	4,114	93.11	38.68
7	VM-Cub1_HD5_1	ATCACG	26,957,513	7.11	100	4,071	93.69	38.84
7	VM-Cub1_HD5_2	TTAGGC	28,914,931	7.63	100	4,366	95.45	39.31
7	VM-Cub1_HD5_3	ACTTGA	28,831,726	7.61	100	4,354	95.27	39.26
7	VM-Cub1_HD5_4	GATCAG	25,731,145	6.79	100	3,885	94.96	39.18
7	Undetermined	unknown	19,668,803	5.19	100	2,970	93.20	38.68

- Undetermined reads are all reads of a lane that do not match 100 % to the given barcode sequences.

7. Related files

QC step	File names
**Sequencing Data Report:**<br>Summary	116-NGS-270617-0_Sequencing Data Report.pdf
**Sample QC:**<br>Capillary gel electrophoresis of original sample /totalRNA<br>(Fragment Analyzer using Total RNA Analysis Standard Sensitivity Assay)	116-NGS-270617-3 FA-ProbenQC.pdf
**Library QC:**<br>Capillary gel electrophoresis of final library<br>(Fragment Analyzer DNF-474-33 – NGS-HS)	116-NGS-270617-4_finaleLibrary.pdf
**Sequencing Data:**<br>HiSeq3000 Data files	Fastq files for each sample and undetermined reads. Files can be found in the following location:<br>\116-NGS-270617-0\
