# Supplementary material for: Proteomic and transcriptomic profiles of human urothelial cancer cells with histone deacetylase 5 overexpression
Source: Sci Data. 2022 May 27;9:240. doi: 10.1038/s41597-022-01319-0 (PMC9142574; doi:10.1038/s41597-022-01319-0)
Supplement: Supplementary file 2 — QC report of RNA samples [file 41597_2022_1319_MOESM2_ESM.pdf]

Fragment Analyzer Project Summary:

Data Files: 2017 06 29 13H 45M.raw, 2017 06 29 15H 52M.raw, 2017 06 30 08H 21M.raw, 2017 06 29 12H 34M.raw, 2017 06 29 14H 48M.raw,

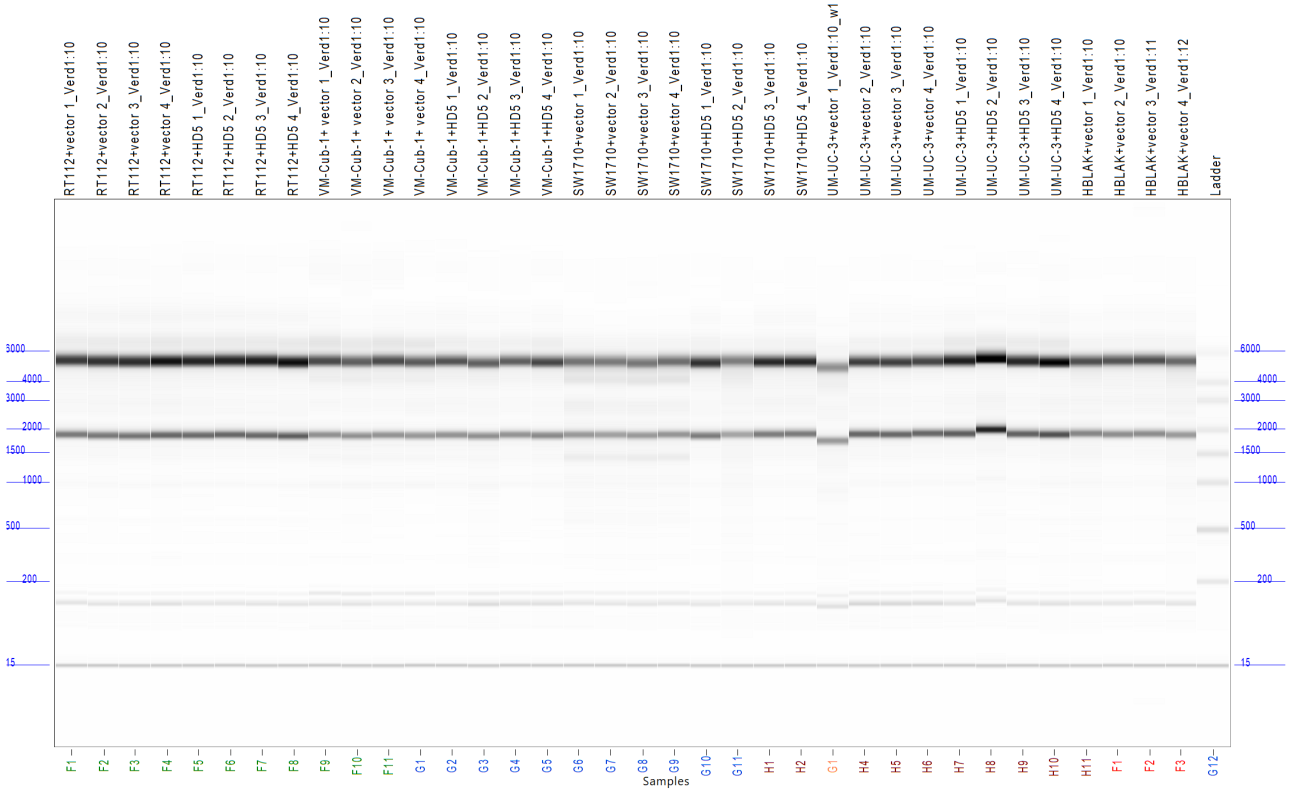

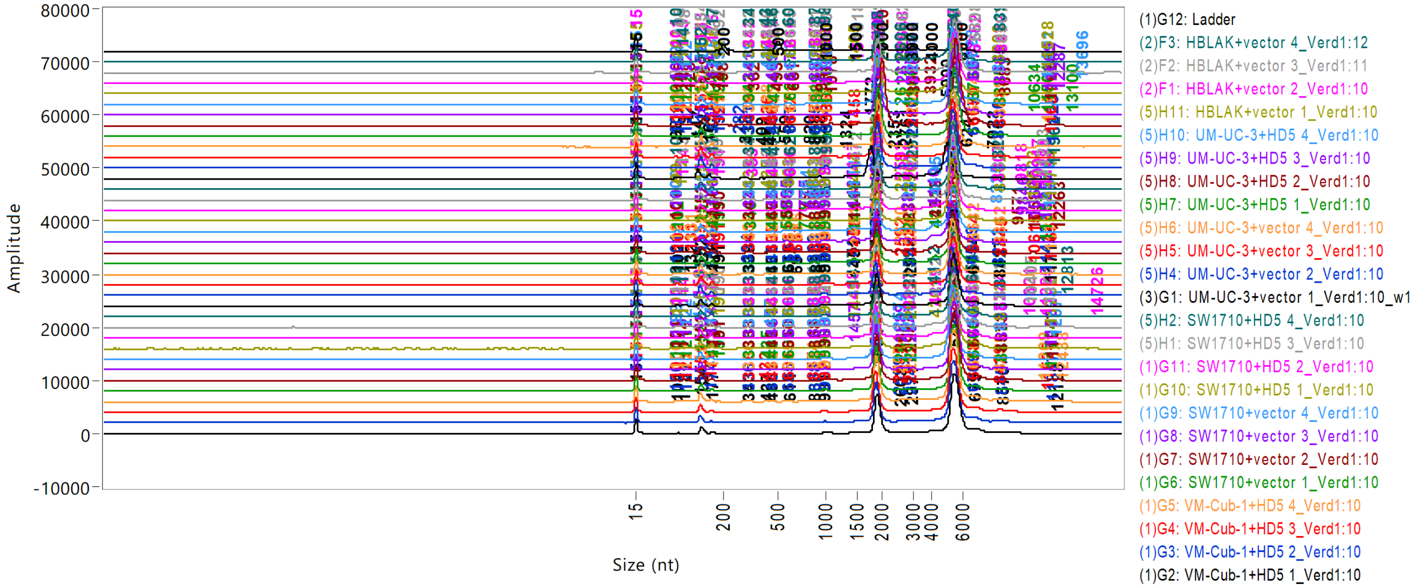

 Copyright 2015 Advanced Analytical Technologies, Inc.

Filename and Data Path: Z:\GTL_Shared\05_AuftraegeProjekte\03_NGS_MA\02_NGS_MA_Daten\2017_NGS_MA\116-NGS-270617-0\116-NGS-270617-2\2017 06 29 13H 45M.raw
Created: Thursday, June 29, 2017 2:08:28 PM
# of Capillaries: 12
Array Serial #: 102615-03SFS
Effect Length: 33 cm
Array Usage Count: 340
FA Version #: 1.1.0.11
Device Serial #: 3228

METHOD INFORMATION
Method Name: DNF-472T33 - HS Total RNA 15nt.mthds
Gel Prime: No
Full Conditioning: Yes
Gel Prime to Buffer: Yes
Gel Selection: Gel 2
Perform Prerun: 8,0 kV, 30 sec.
Rinse: No
Marker 1: No
Rinse: Tray: 3, Row: A, # Dips: 2
Sample Injection: 7,0 kV, 150 sec.
Separation: 8,0 kV, 40,0 min.
Tray Name: 116-NGS-270617-2

Analysis Mode: RNA (Eukaryotic)

NOTE
This analysis was carried out by Sandra Plante.

Filename and Data Path: Z:\GTL_Shared\05_AuftraegeProjekte\03_NGS_MA\02_NGS_MA_Daten\2017_NGS_MA\116-NGS-270617-0\116-NGS-270617-2 15-52-30\2017 06 29 15H 52M.raw
Created: Thursday, June 29, 2017 4:15:52 PM
# of Capillaries: 12
Array Serial #: 102615-03SFS
Effect Length: 33 cm
Array Usage Count: 342
FA Version #: 1.1.0.11
Device Serial #: 3228

METHOD INFORMATION
Method Name: DNF-472T33 - HS Total RNA 15nt.mthds
Gel Prime: No
Full Conditioning: Yes
Gel Prime to Buffer: Yes
Gel Selection: Gel 2
Perform Prerun: 8,0 kV, 30 sec.
Rinse: No
Marker 1: No
Rinse: Tray: 3, Row: A, # Dips: 2
Sample Injection: 7,0 kV, 150 sec.
Separation: 8,0 kV, 40,0 min.
Tray Name: Tray-2

Analysis Mode: RNA (Eukaryotic)

NOTE
This analysis was carried out by Sandra Plante.

 Copyright 2015 Advanced Analytical Technologies, Inc.

Filename and Data Path: Z:\GTL_Shared\05_AuftraegeProjekte\03_NGS_MA\02_NGS_MA_Daten\2017_NGS_MA\116-NGS-270617-0\116-NGS-270617-2_w1 08-21-57\2017 06 30 08H 21M.raw
Created: Friday, June 30, 2017 8:45:19 AM
# of Capillaries: 12
Array Serial #: 102615-03SFS
Effect Length: 33 cm
Array Usage Count: 343
FA Version #: 1.1.0.11
Device Serial #: 3228

METHOD INFORMATION
Method Name: DNF-472T33 - HS Total RNA 15nt.mthds
Gel Prime: No
Full Conditioning: Yes
Gel Prime to Buffer: Yes
Gel Selection: Gel 2
Perform Prerun: 8,0 kV, 30 sec.
Rinse: No
Marker 1: No
Rinse: Tray: 3, Row: A, # Dips: 2
Sample Injection: 7,0 kV, 150 sec.
Separation: 8,0 kV, 40,0 min.
Tray Name: Tray2

Analysis Mode: RNA (Eukaryotic)

NOTE
This analysis was carried out by Sandra Plante.

 Copyright 2015 Advanced Analytical Technologies, Inc.

Filename and Data Path: Z:\GTL_Shared\05_AuftraegeProjekte\03_NGS_MA\02_NGS_MA_Daten\2017_NGS_MA\116-NGS-270617-0\116-NGS-270617-2 12-34-50\2017 06 29 12H 34M.raw
Created: Thursday, June 29, 2017 12:58:12 PM
# of Capillaries: 12
Array Serial #: 102615-03SFS
Effect Length: 33 cm
Array Usage Count: 339
FA Version #: 1.1.0.11
Device Serial #: 3228

METHOD INFORMATION
Method Name: DNF-472T33 - HS Total RNA 15nt.mthds
Gel Prime: No
Full Conditioning: Yes
Gel Prime to Buffer: Yes
Gel Selection: Gel 2
Perform Prerun: 8,0 kV, 30 sec.
Rinse: No
Marker 1: No
Rinse: Tray: 3, Row: A, # Dips: 2
Sample Injection: 7,0 kV, 150 sec.
Separation: 8,0 kV, 40,0 min.
Tray Name: Tray-1

Analysis Mode: RNA (Eukaryotic)

NOTE
This analysis was carried out by Sandra Plante.

 Copyright 2015 Advanced Analytical Technologies, Inc.

Filename and Data Path: Z:\GTL_Shared\05_AuftraegeProjekte\03_NGS_MA\02_NGS_MA_Daten\2017_NGS_MA\116-NGS-270617-0\116-NGS-310617-2 14-48-58\2017 06 29 14H 48M.raw
Created: Thursday, June 29, 2017 3:12:16 PM
# of Capillaries: 12
Array Serial #: 102615-03SFS
Effect Length: 33 cm
Array Usage Count: 341
FA Version #: 1.1.0.11
Device Serial #: 3228

METHOD INFORMATION
Method Name: DNF-472T33 - HS Total RNA 15nt.mthds
Gel Prime: No
Full Conditioning: Yes
Gel Prime to Buffer: Yes
Gel Selection: Gel 2
Perform Prerun: 8,0 kV, 30 sec.
Rinse: No
Marker 1: No
Rinse: Tray: 3, Row: A, # Dips: 2
Sample Injection: 7,0 kV, 150 sec.
Separation: 8,0 kV, 40,0 min.
Tray Name: Tray2

Analysis Mode: RNA (Eukaryotic)

NOTE
This analysis was carried out by Sandra Plante.

 Copyright 2015 Advanced Analytical Technologies, Inc.

2017 06 29 12H 34M.raw
F1: RT112+vector 1_Verd1:10
RQN: 10,0

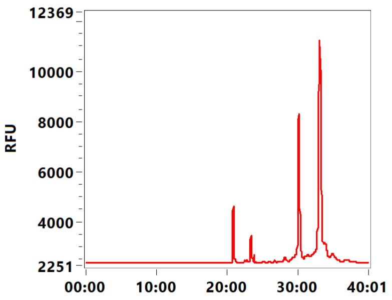

2017 06 29 12H 34M.raw
F2: RT112+vector 2_Verd1:10
RQN: 10,0

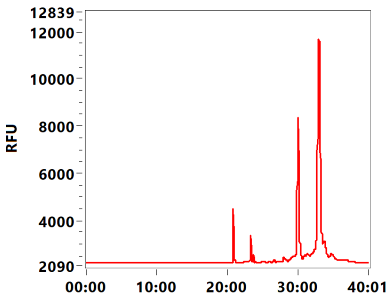

2017 06 29 12H 34M.raw
F3: RT112+vector 3_Verd1:10
RQN: 10,0

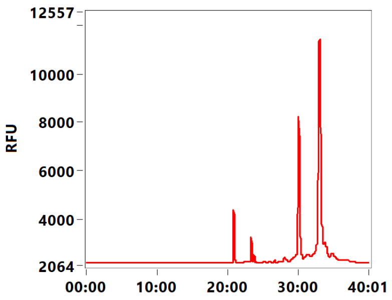

2017 06 29 12H 34M.raw
F4: RT112+vector 4_Verd1:10
RQN: 10,0

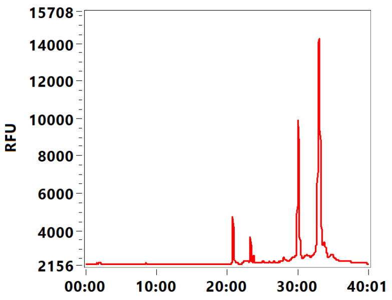

2017 06 29 12H 34M.raw
F5: RT112+HD5 1_Verd1:10
RQN: 10,0

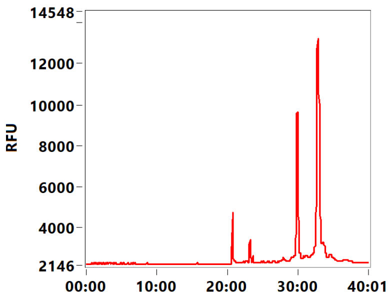

2017 06 29 12H 34M.raw
F6: RT112+HD5 2_Verd1:10
RQN: 10,0

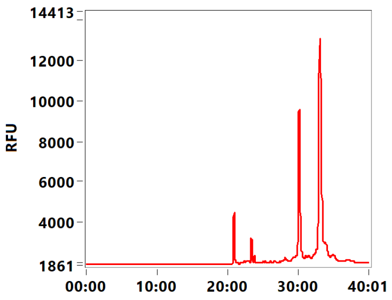

2017 06 29 12H 34M.raw
F7: RT112+HD5 3_Verd1:10
RQN: 10,0

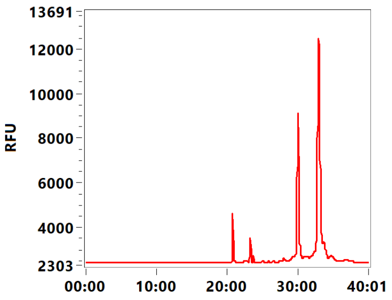

2017 06 29 12H 34M.raw
F8: RT112+HD5 4_Verd1:10
RQN: 10,0

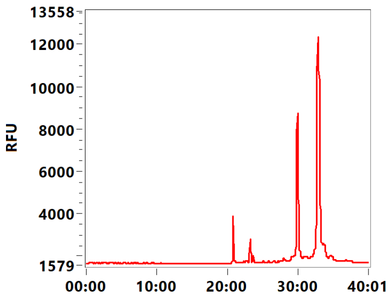

2017 06 29 12H 34M.raw
F9: VM-Cub-1+ vector 1_Verd1:10
RQN: 10,0

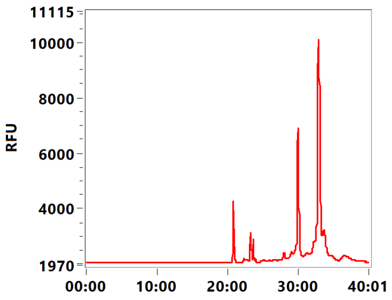

2017 06 29 12H 34M.raw
F10: VM-Cub-1+ vector 2_Verd1:10
RQN: 9,8

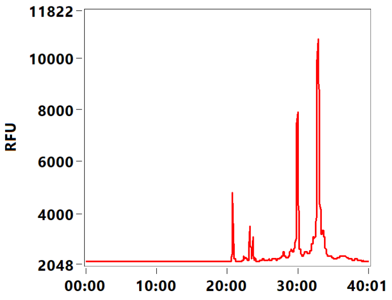

2017 06 29 12H 34M.raw
F11: VM-Cub-1+ vector 3_Verd1:10
RQN: 10,0

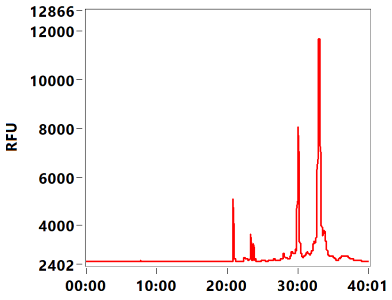

2017 06 29 13H 45M.raw
G1: VM-Cub-1+ vector 4_Verd1:10
RQN: 10,0

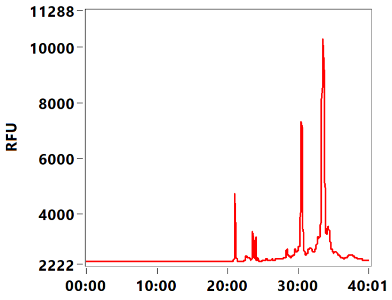

Copyright 2015 Advanced Analytical Technologies, Inc.

2017 06 29 13H 45M.raw
G2: VM-Cub-1+HD5 1_Verd1:10
RQN: 10,0

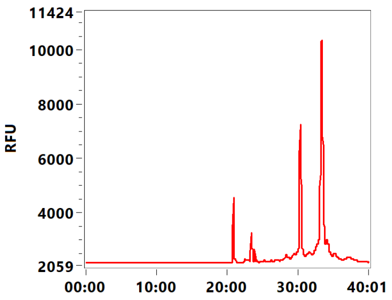

2017 06 29 13H 45M.raw
G3: VM-Cub-1+HD5 2_Verd1:10
RQN: 9,7

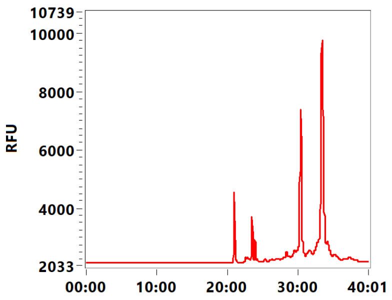

2017 06 29 13H 45M.raw
G4: VM-Cub-1+HD5 3_Verd1:10
RQN: 10,0

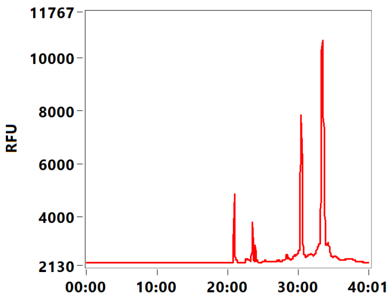

2017 06 29 13H 45M.raw
G5: VM-Cub-1+HD5 4_Verd1:10
RQN: 10,0

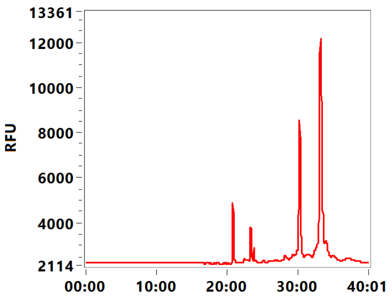

2017 06 29 13H 45M.raw
G6: SW1710+vector 1_Verd1:10
RQN: 9,2

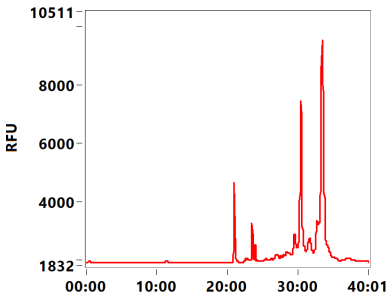

2017 06 29 13H 45M.raw
G7: SW1710+vector 2_Verd1:10
RQN: 9,1

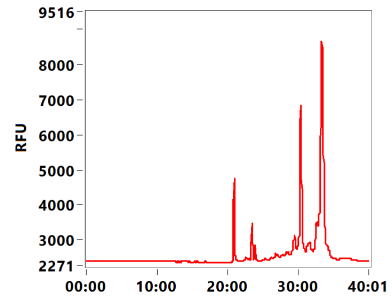

2017 06 29 13H 45M.raw
G8: SW1710+vector 3_Verd1:10
RQN: 9,1

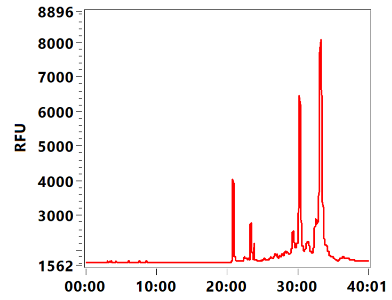

2017 06 29 13H 45M.raw
G9: SW1710+vector 4_Verd1:10
RQN: 9,3

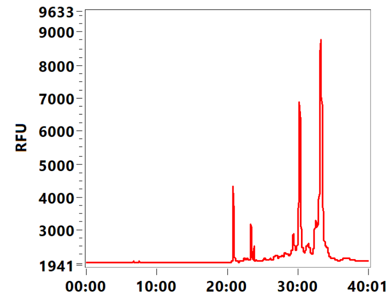

2017 06 29 13H 45M.raw
G10: SW1710+HD5 1_Verd1:10
RQN: 10,0

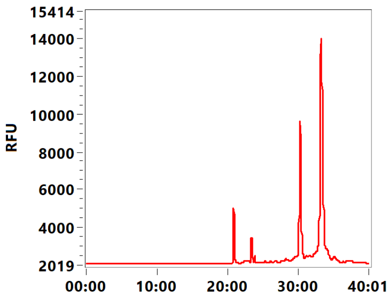

2017 06 29 13H 45M.raw
G11: SW1710+HD5 2_Verd1:10
RQN: 9,6

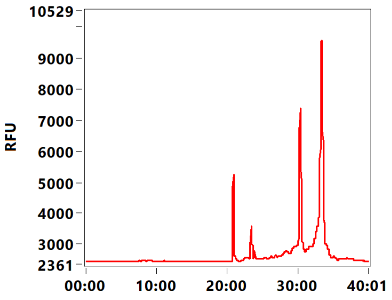

2017 06 29 14H 48M.raw
H1: SW1710+HD5 3_Verd1:10
RQN: 10,0

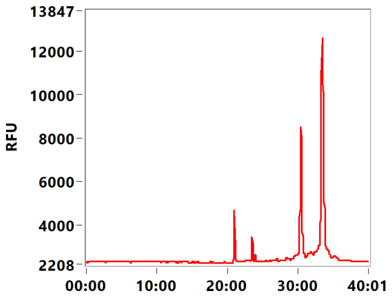

2017 06 29 14H 48M.raw
H2: SW1710+HD5 4_Verd1:10
RQN: 10,0

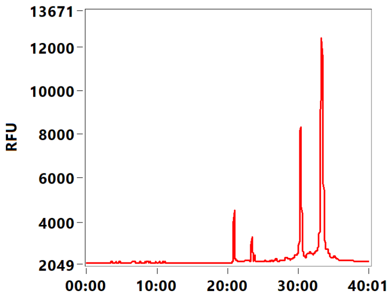

Copyright 2015 Advanced Analytical Technologies, Inc.

2017 06 30 08H 21M.raw
G1: UM-UC-3+vector 1_Verd1:10_w1
RQN: 9,3

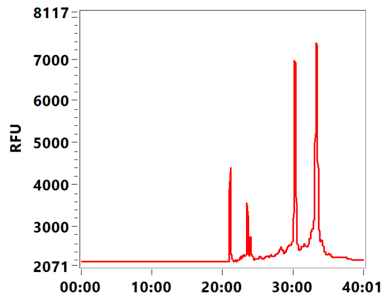

2017 06 29 14H 48M.raw
H4: UM-UC-3+vector 2_Verd1:10
RQN: 10,0

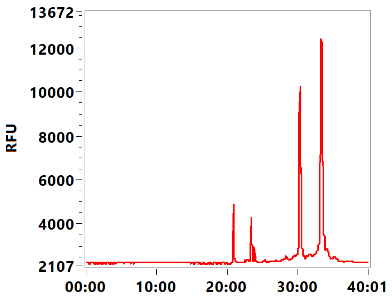

2017 06 29 14H 48M.raw
H5: UM-UC-3+vector 3_Verd1:10
RQN: 10,0

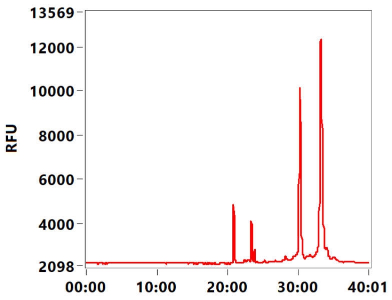

2017 06 29 14H 48M.raw
H6: UM-UC-3+vector 4_Verd1:10
RQN: 10,0

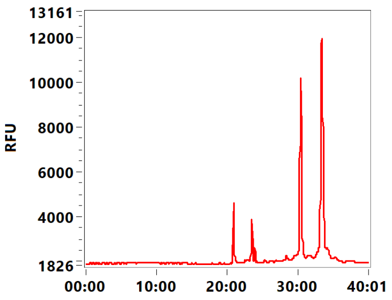

2017 06 29 14H 48M.raw
H7: UM-UC-3+HD5 1_Verd1:10
RQN: 10,0

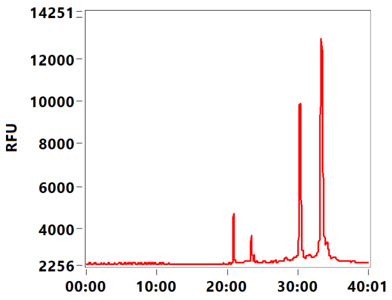

2017 06 29 14H 48M.raw
H8: UM-UC-3+HD5 2_Verd1:10
RQN: 10,0

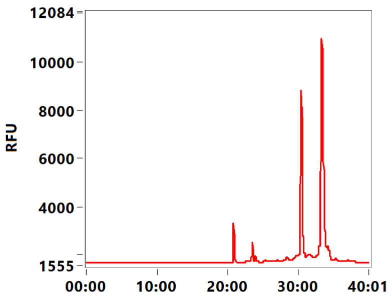

2017 06 29 14H 48M.raw
H9: UM-UC-3+HD5 3_Verd1:10
RQN: 10,0

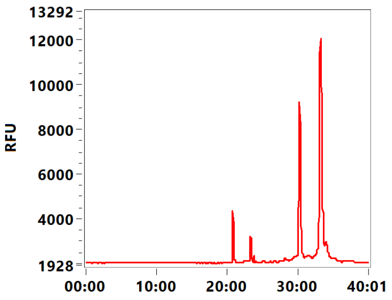

2017 06 29 14H 48M.raw
H10: UM-UC-3+HD5 4_Verd1:10
RQN: 10,0

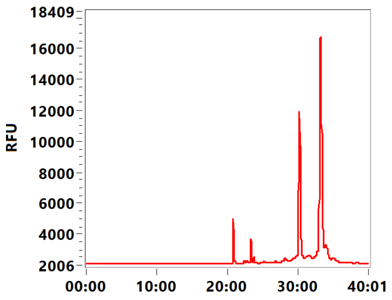

2017 06 29 14H 48M.raw
H11: HBLAK+vector 1_Verd1:10
RQN: 9,9

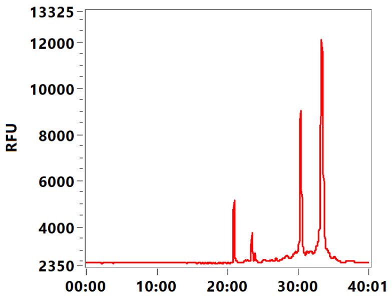

2017 06 29 15H 52M.raw
F1: HBLAK+vector 2_Verd1:10
RQN: 10,0

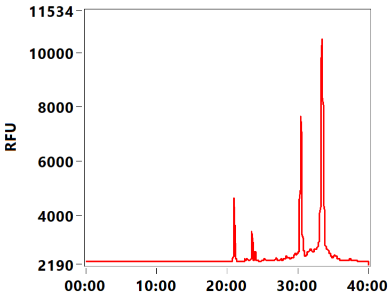

2017 06 29 15H 52M.raw
F2: HBLAK+vector 3_Verd1:11
RQN: 10,0

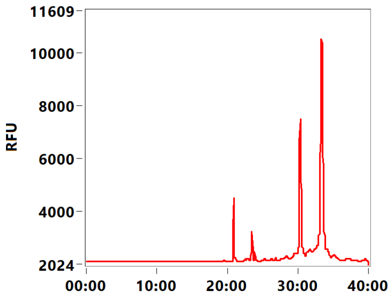

2017 06 29 15H 52M.raw
F3: HBLAK+vector 4_Verd1:12
RQN: 9,5

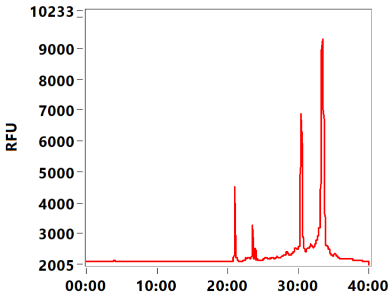

Copyright 2015 Advanced Analytical Technologies, Inc.

2017 06 29 13H 45M.raw

G12: Ladder

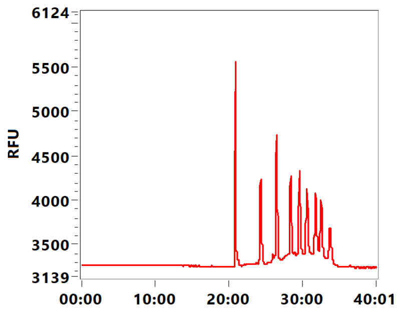

 Copyright 2015 Advanced Analytical Technologies, Inc.

Data File: 2017 06 29 12H 34M.raw
Sample: RT112+vector 1_Verd1:10
Well Location: F1

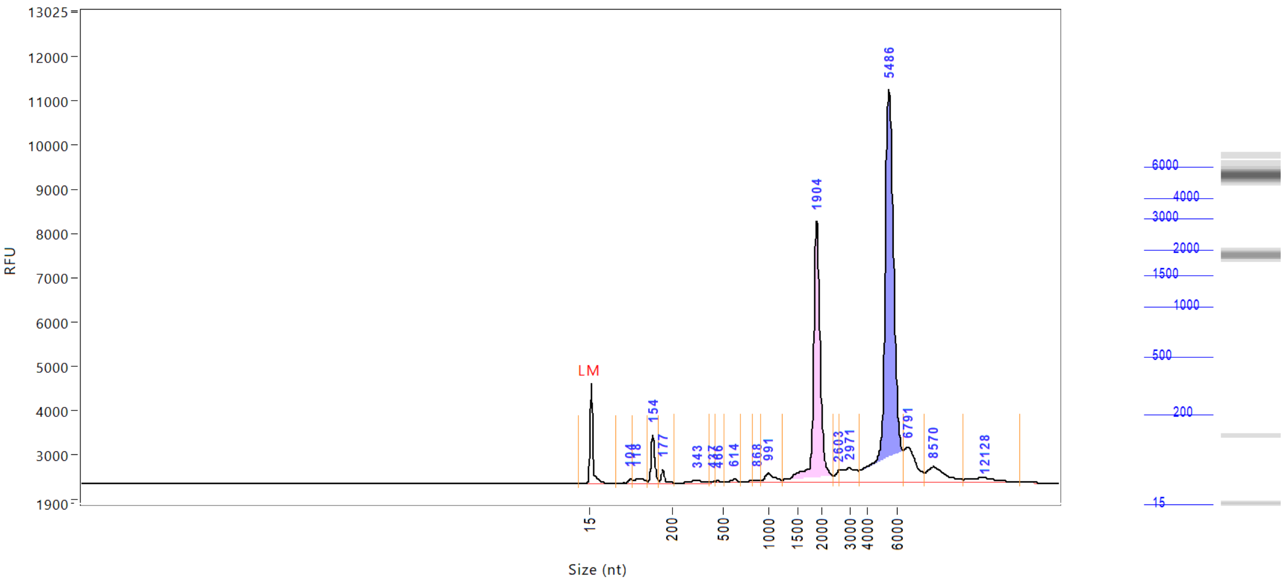

Peak	Size (nt)	Conc. (ng/uL)
1	15 (LM)	0.0329
2	104	0.0177
3	118	0.0398
4	154	0.1906
5	177	0.0439
6	343	0.0207
7	437	0.0054
8	466	0.0073
9	614	0.0203
10	868	0.0145
11	991	0.0722
12	1904	1.5019
13	2603	0.0326
14	2971	0.1692
15	5486	2.8560
16	6791	0.2979
17	8570	0.1834
18	12128	0.0499

TIC:	5.5233	ng/uL
TIM:	11.371	nmole/L
Total Conc.:	5.5112	ng/uL

28S/18S: 1.7

Sample Peak Width (sec): 6   Sample Min Peak Height: 20   Sample Baseline V to V?: Y   Sample Baseline V to V pts: 3
Sample Filter: Binomial   # of Pts for Filter: 9   Sample Start Region (min): 0   Sample End Region (min): 40
Manual Baseline Start (min): 18   Manual Baseline End (min): 38
Marker Peak Width (sec): 6   Marker Min Peak Height: 100   Marker Baseline V to V?: Y   Marker Baseline V to V pts: 3
Lower Marker Selection: First Peak > 100 RFU   Upper Marker Selection: Last Peak > 100 RFU
Ladder Size (bp): 15, 200, 500, 1000, 1500, 2000, 3000, 4000, 6000
Quantification Using: Ladder   Final Concentration (ng/uL): 0.2000   Dilution Factor: 10.0
Min. RFU for Data Processing: 2

 Copyright 2015 Advanced Analytical Technologies, Inc.

Data File: 2017 06 29 12H 34M.raw
Sample: RT112+vector 1_Verd1:10
Well Location: F1

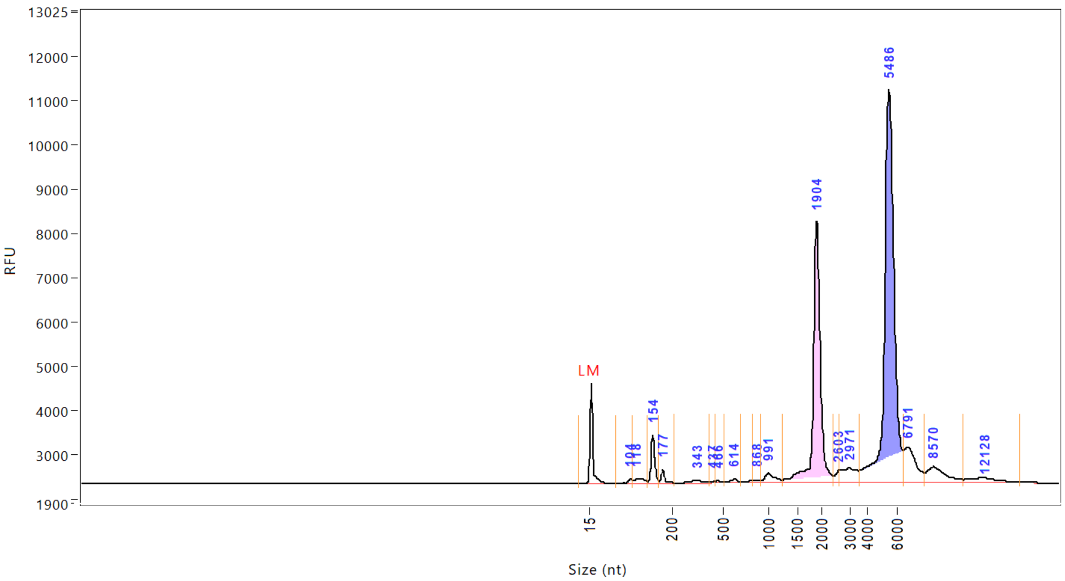

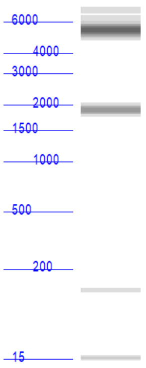

Peak	Size (nt)	Conc. (ng/uL)
	RQN	10.0

Sample Peak Width (sec): 6 Sample Min Peak Height: 20 Sample Baseline V to V?: Y Sample Baseline V to V pts: 3
Sample Filter: Binomial # of Pts for Filter: 9 Sample Start Region (min): 0 Sample End Region (min): 40
Manual Baseline Start (min): 18 Manual Baseline End (min): 38
Marker Peak Width (sec): 6 Marker Min Peak Height: 100 Marker Baseline V to V?: Y Marker Baseline V to V pts: 3
Lower Marker Selection: First Peak > 100 RFU Upper Marker Selection: Last Peak > 100 RFU
Ladder Size (bp): 15, 200, 500, 1000, 1500, 2000, 3000, 4000, 6000
Quantification Using: Ladder Final Concentration (ng/uL): 0.2000 Dilution Factor: 10.0
Min. RFU for Data Processing: 2

 Copyright 2015 Advanced Analytical Technologies, Inc.

Data File: 2017 06 29 12H 34M.raw
Sample: RT112+vector 2_Verd1:10
Well Location: F2

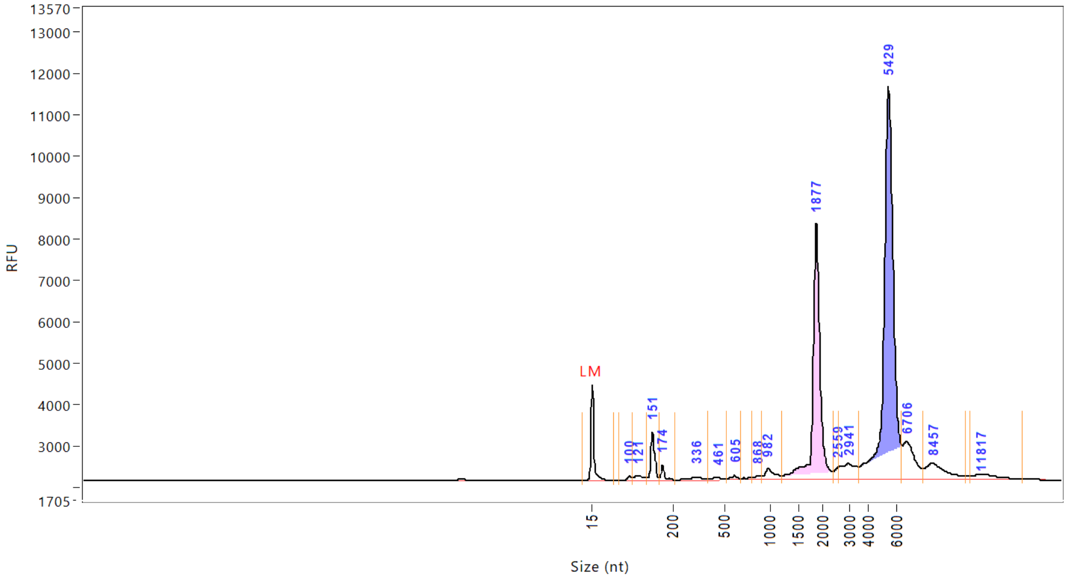

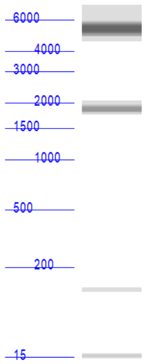

Peak	Size (nt)	Conc. (ng/uL)
1	15 (LM)	0.0329
2	100	0.0173
3	121	0.0431
4	151	0.2050
5	174	0.0507
6	336	0.0287
7	461	0.0219
8	605	0.0269
9	868	0.0217
10	982	0.0929
11	1877	1.5974
12	2559	0.0389
13	2941	0.1947
14	5429	3.0687
15	6706	0.3420
16	8457	0.2187
17	11817	0.0540

TIC:	6.0226	ng/uL
TIM:	12.618	nmole/L
Total Conc.:	6.0196	ng/uL

28S/18S: 1.7

```
Sample Peak Width (sec): 6    Sample Min Peak Height: 20    Sample Baseline V to V?: Y    Sample Baseline V to V pts: 3
Sample Filter: Binomial       # of Pts for Filter: 9        Sample Start Region (min): 0  Sample End Region (min): 40
Manual Baseline Start (min): 18     Manual Baseline End (min): 38
Marker Peak Width (sec): 6    Marker Min Peak Height: 100   Marker Baseline V to V?: Y    Marker Baseline V to V pts: 3
Lower Marker Selection: First Peak > 100 RFU                Upper Marker Selection: Last Peak > 100 RFU
Ladder Size (bp): 15, 200, 500, 1000, 1500, 2000, 3000, 4000, 6000
Quantification Using: Ladder          Final Concentration (ng/uL): 0.2000          Dilution Factor: 10.0
Min. RFU for Data Processing: 2
```

 Copyright 2015 Advanced Analytical Technologies, Inc.

Data File: 2017 06 29 12H 34M.raw
Sample: RT112+vector 2_Verd1:10
Well Location: F2

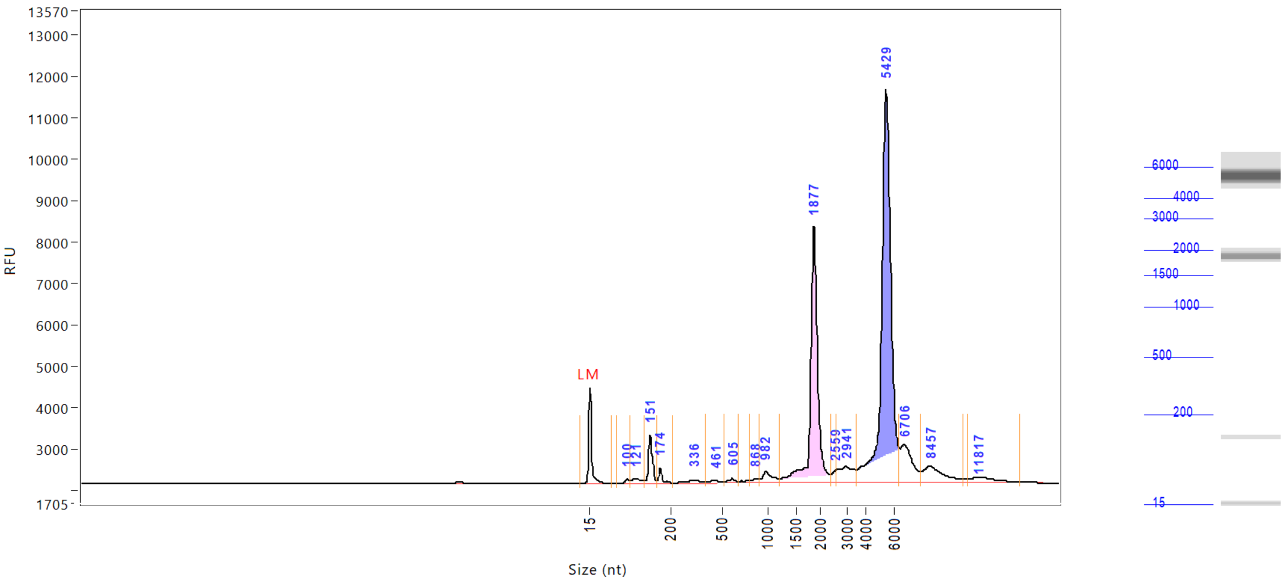

Peak	Size (nt)	Conc. (ng/uL)
	RQN	10.0

Sample Peak Width (sec): 6   Sample Min Peak Height: 20   Sample Baseline V to V?: Y   Sample Baseline V to V pts: 3
Sample Filter: Binomial   # of Pts for Filter: 9   Sample Start Region (min): 0   Sample End Region (min): 40
Manual Baseline Start (min): 18   Manual Baseline End (min): 38
Marker Peak Width (sec): 6   Marker Min Peak Height: 100   Marker Baseline V to V?: Y   Marker Baseline V to V pts: 3
Lower Marker Selection: First Peak > 100 RFU   Upper Marker Selection: Last Peak > 100 RFU
Ladder Size (bp): 15, 200, 500, 1000, 1500, 2000, 3000, 4000, 6000
Quantification Using: Ladder   Final Concentration (ng/uL): 0.2000   Dilution Factor: 10.0
Min. RFU for Data Processing: 2

 Copyright 2015 Advanced Analytical Technologies, Inc.

Data File: 2017 06 29 12H 34M.raw
Sample: RT112+vector 3_Verd1:10
Well Location: F3

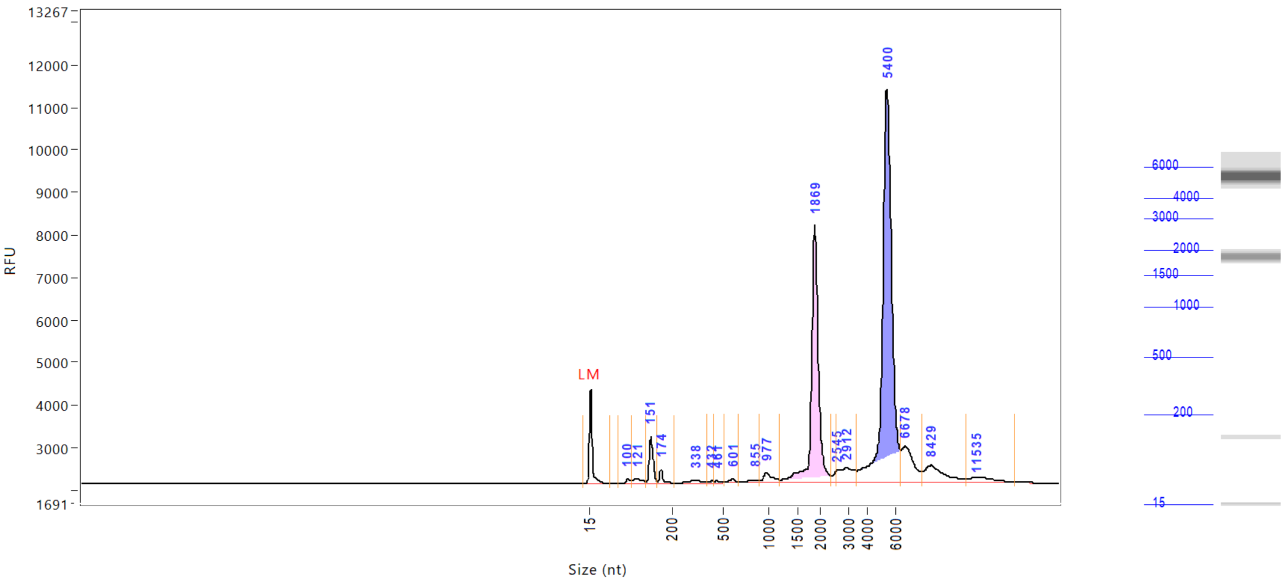

Peak	Size (nt)	Conc. (ng/uL)
1	15 (LM)	0.0329
2	100	0.0176
3	121	0.0434
4	151	0.2016
5	174	0.0476
6	338	0.0229
7	432	0.0052
8	461	0.0104
9	601	0.0200
10	855	0.0277
11	977	0.0783
12	1869	1.5967
13	2545	0.0354
14	2912	0.1858
15	5400	3.0821
16	6678	0.3308
17	8429	0.2341
18	11535	0.0625

TIC:	6.0021	ng/uL
TIM:	12.379	nmole/L
Total Conc.:	5.9778	ng/uL

28S/18S: 1.7

```
Sample Peak Width (sec): 6    Sample Min Peak Height: 20    Sample Baseline V to V?: Y    Sample Baseline V to V pts: 3
Sample Filter: Binomial       # of Pts for Filter: 9        Sample Start Region (min): 0  Sample End Region (min): 40
Manual Baseline Start (min): 18     Manual Baseline End (min): 38
Marker Peak Width (sec): 6    Marker Min Peak Height: 100   Marker Baseline V to V?: Y    Marker Baseline V to V pts: 3
Lower Marker Selection: First Peak > 100 RFU                Upper Marker Selection: Last Peak > 100 RFU
Ladder Size (bp): 15, 200, 500, 1000, 1500, 2000, 3000, 4000, 6000
Quantification Using: Ladder          Final Concentration (ng/uL): 0.2000          Dilution Factor: 10.0
Min. RFU for Data Processing: 2
```

 Copyright 2015 Advanced Analytical Technologies, Inc.

Data File: 2017 06 29 12H 34M.raw
Sample: RT112+vector 3_Verd1:10
Well Location: F3

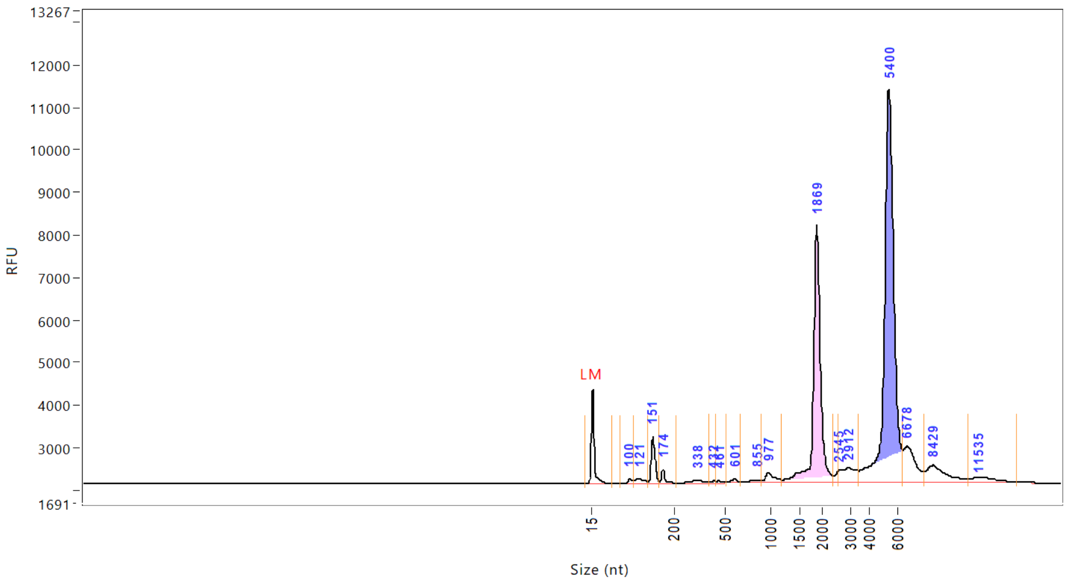

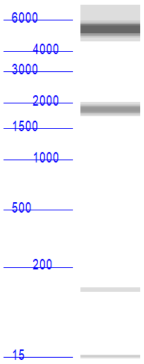

Peak	Size (nt)	Conc. (ng/uL)
	RQN	10.0

```
Sample Peak Width (sec): 6    Sample Min Peak Height: 20    Sample Baseline V to V?: Y    Sample Baseline V to V pts: 3
Sample Filter: Binomial       # of Pts for Filter: 9        Sample Start Region (min): 0  Sample End Region (min): 40
Manual Baseline Start (min): 18     Manual Baseline End (min): 38
Marker Peak Width (sec): 6    Marker Min Peak Height: 100   Marker Baseline V to V?: Y    Marker Baseline V to V pts: 3
Lower Marker Selection: First Peak > 100 RFU                Upper Marker Selection: Last Peak > 100 RFU
Ladder Size (bp): 15, 200, 500, 1000, 1500, 2000, 3000, 4000, 6000
Quantification Using: Ladder          Final Concentration (ng/uL): 0.2000          Dilution Factor: 10.0
Min. RFU for Data Processing: 2
```

 Copyright 2015 Advanced Analytical Technologies, Inc.

Data File: 2017 06 29 12H 34M.raw
Sample: RT112+vector 4_Verd1:10
Well Location: F4

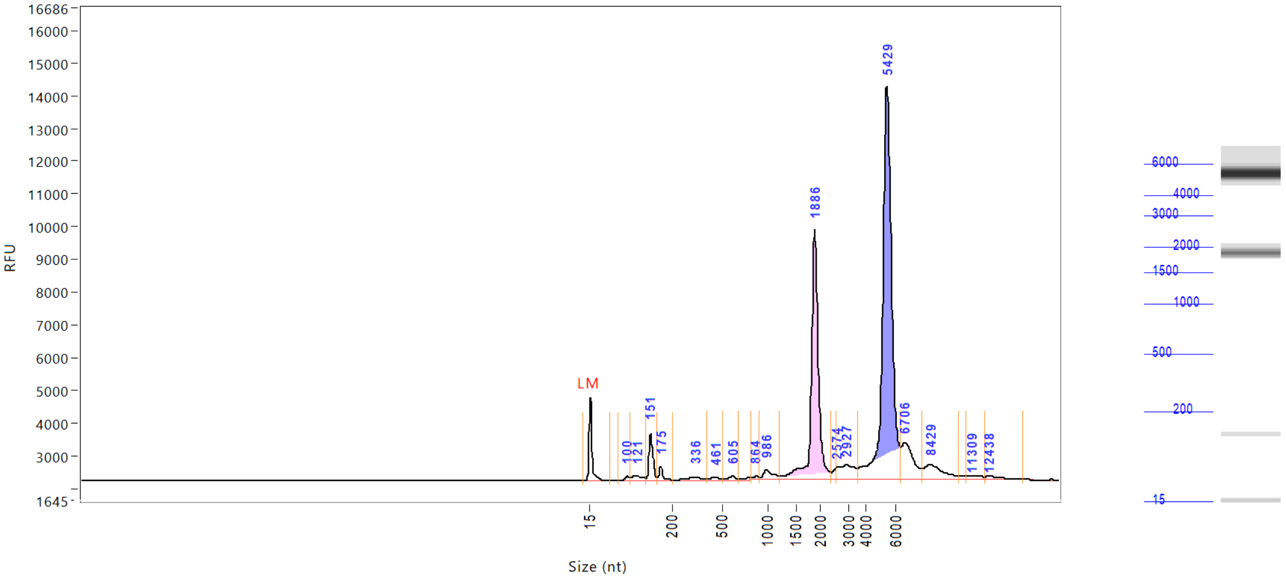

Peak	Size (nt)	Conc. (ng/uL)
1	15 (LM)	0.0329
2	100	0.0217
3	121	0.0518
4	151	0.2354
5	175	0.0588
6	336	0.0354
7	461	0.0226
8	605	0.0277
9	864	0.0211
10	986	0.0998
11	1886	1.7891
12	2574	0.0427
13	2927	0.2157
14	5429	3.4187
15	6706	0.3907
16	8429	0.2313
17	11309	0.0358
18	12438	0.0213

TIC:	6.7198	ng/uL
TIM:	14.397	nmole/L
Total Conc.:	6.7217	ng/uL

Sample Peak Width (sec): 6   Sample Min Peak Height: 20   Sample Baseline V to V?: Y   Sample Baseline V to V pts: 3
Sample Filter: Binomial   # of Pts for Filter: 9   Sample Start Region (min): 0   Sample End Region (min): 40
Manual Baseline Start (min): 18   Manual Baseline End (min): 38
Marker Peak Width (sec): 6   Marker Min Peak Height: 100   Marker Baseline V to V?: Y   Marker Baseline V to V pts: 3
Lower Marker Selection: First Peak > 100 RFU   Upper Marker Selection: Last Peak > 100 RFU
Ladder Size (bp): 15, 200, 500, 1000, 1500, 2000, 3000, 4000, 6000
Quantification Using: Ladder   Final Concentration (ng/uL): 0.2000   Dilution Factor: 10.0
Min. RFU for Data Processing: 2

 Copyright 2015 Advanced Analytical Technologies, Inc.

Data File: 2017 06 29 12H 34M.raw
Sample: RT112+vector 4_Verd1:10
Well Location: F4

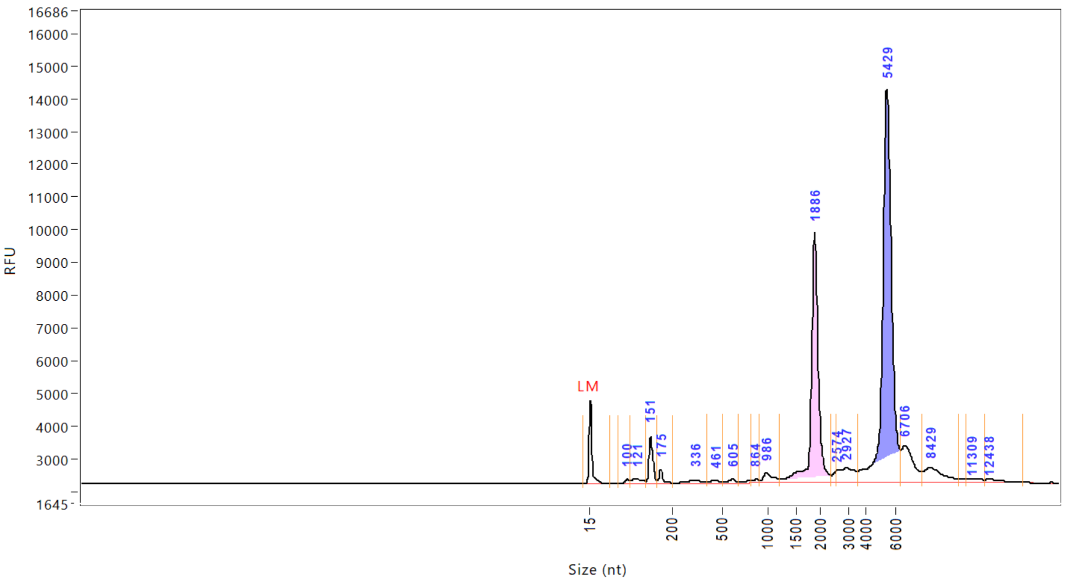

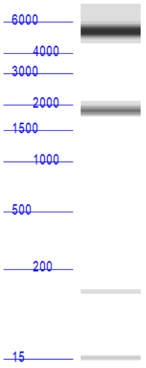

Peak	Size (nt)	Conc. (ng/uL)

28S/18S:	1.7
RQN	10.0

```
Sample Peak Width (sec): 6    Sample Min Peak Height: 20    Sample Baseline V to V?: Y    Sample Baseline V to V pts: 3
Sample Filter: Binomial       # of Pts for Filter: 9        Sample Start Region (min): 0  Sample End Region (min): 40
Manual Baseline Start (min): 18     Manual Baseline End (min): 38
Marker Peak Width (sec): 6    Marker Min Peak Height: 100   Marker Baseline V to V?: Y    Marker Baseline V to V pts: 3
Lower Marker Selection: First Peak > 100 RFU                Upper Marker Selection: Last Peak > 100 RFU
Ladder Size (bp): 15, 200, 500, 1000, 1500, 2000, 3000, 4000, 6000
Quantification Using: Ladder          Final Concentration (ng/uL): 0.2000          Dilution Factor: 10.0
Min. RFU for Data Processing: 2
```

 Copyright 2015 Advanced Analytical Technologies, Inc.

Data File: 2017 06 29 12H 34M.raw
Sample: RT112+HD5 1_Verd1:10
Well Location: F5

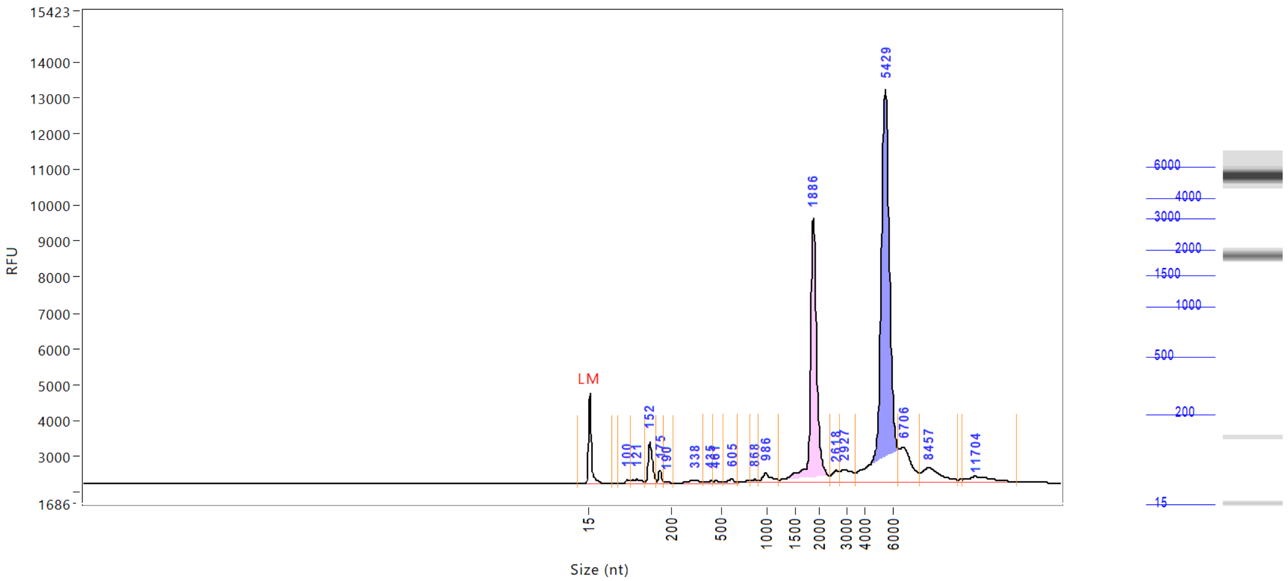

Peak	Size (nt)	Conc. (ng/uL)
1	15 (LM)	0.0329
2	100	0.0171
3	121	0.0406
4	152	0.1974
5	175	0.0417
6	190	0.0044
7	338	0.0238
8	435	0.0091
9	461	0.0121
10	605	0.0216
11	868	0.0195
12	986	0.0814
13	1886	1.7046
14	2618	0.0718
15	2927	0.1317
16	5429	3.2050
17	6706	0.3372
18	8457	0.2003
19	11704	0.0762

TIC:	6.1954	ng/uL
TIM:	12.367	nmole/L
Total Conc.:	6.1882	ng/uL

Sample Peak Width (sec): 6   Sample Min Peak Height: 20   Sample Baseline V to V?: Y   Sample Baseline V to V pts: 3
Sample Filter: Binomial   # of Pts for Filter: 9   Sample Start Region (min): 0   Sample End Region (min): 40
Manual Baseline Start (min): 18   Manual Baseline End (min): 38
Marker Peak Width (sec): 6   Marker Min Peak Height: 100   Marker Baseline V to V?: Y   Marker Baseline V to V pts: 3
Lower Marker Selection: First Peak > 100 RFU   Upper Marker Selection: Last Peak > 100 RFU
Ladder Size (bp): 15, 200, 500, 1000, 1500, 2000, 3000, 4000, 6000
Quantification Using: Ladder   Final Concentration (ng/uL): 0.2000   Dilution Factor: 10.0
Min. RFU for Data Processing: 2

 Copyright 2015 Advanced Analytical Technologies, Inc.

Data File: 2017 06 29 12H 34M.raw
Sample: RT112+HD5 1_Verd1:10
Well Location: F5

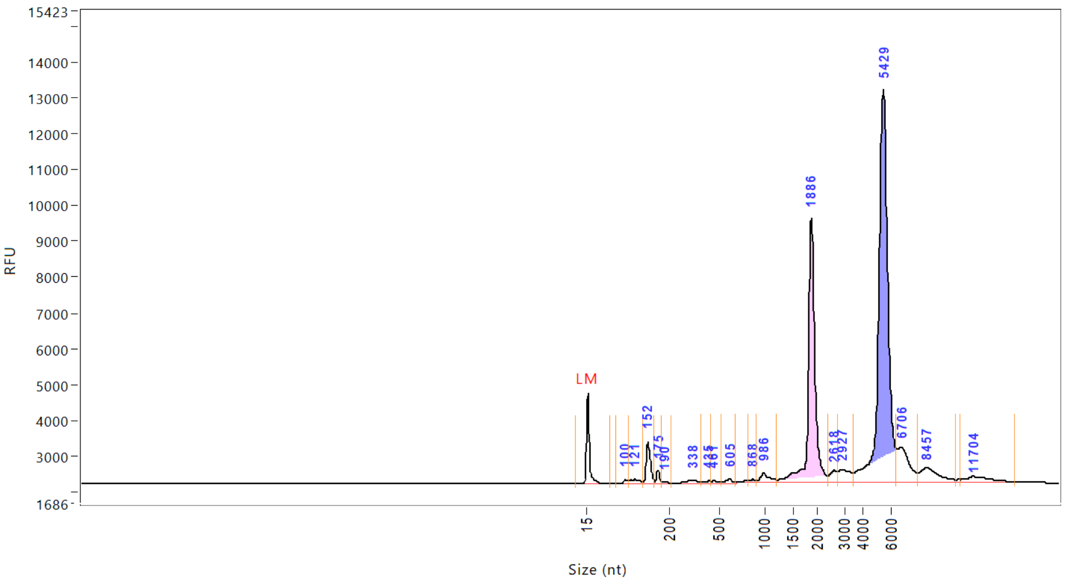

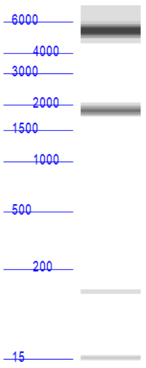

Peak	Size (nt)	Conc. (ng/uL)

28S/18S:	1.7
RQN	10.0

Sample Peak Width (sec): 6 Sample Min Peak Height: 20 Sample Baseline V to V?: Y Sample Baseline V to V pts: 3
Sample Filter: Binomial # of Pts for Filter: 9 Sample Start Region (min): 0 Sample End Region (min): 40
Manual Baseline Start (min): 18 Manual Baseline End (min): 38
Marker Peak Width (sec): 6 Marker Min Peak Height: 100 Marker Baseline V to V?: Y Marker Baseline V to V pts: 3
Lower Marker Selection: First Peak > 100 RFU Upper Marker Selection: Last Peak > 100 RFU
Ladder Size (bp): 15, 200, 500, 1000, 1500, 2000, 3000, 4000, 6000
Quantification Using: Ladder Final Concentration (ng/uL): 0.2000 Dilution Factor: 10.0
Min. RFU for Data Processing: 2

 Copyright 2015 Advanced Analytical Technologies, Inc.

Data File: 2017 06 29 12H 34M.raw
Sample: RT112+HD5 2_Verd1:10
Well Location: F6

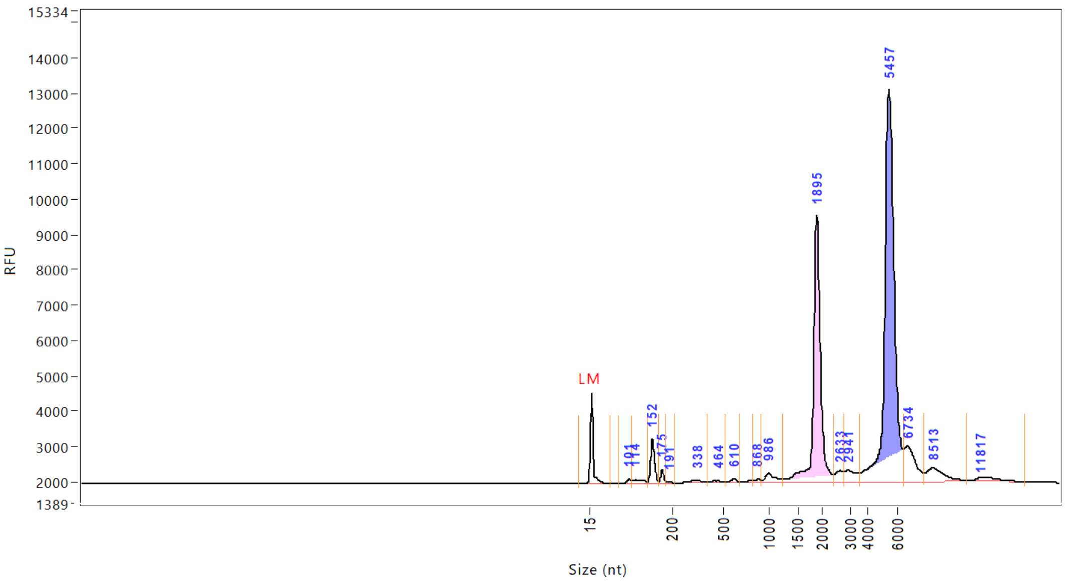

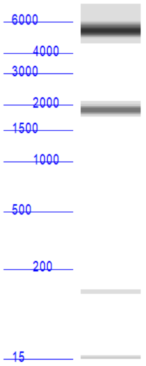

Peak	Size (nt)	Conc. (ng/uL)
1	15 (LM)	0.0329
2	101	0.0160
3	114	0.0384
4	152	0.2054
5	175	0.0427
6	191	0.0034
7	338	0.0206
8	464	0.0168
9	610	0.0208
10	868	0.0167
11	986	0.0791
12	1895	1.7342
13	2633	0.0757
14	2941	0.1142
15	5457	3.2419
16	6734	0.3366
17	8513	0.1836
18	11817	0.0557

TIC:	6.2018	ng/uL
TIM:	12.422	nmole/L
Total Conc.:	6.1892	ng/uL

28S/18S: 1.7

```
Sample Peak Width (sec): 6    Sample Min Peak Height: 20    Sample Baseline V to V?: Y    Sample Baseline V to V pts: 3
Sample Filter: Binomial       # of Pts for Filter: 9        Sample Start Region (min): 0  Sample End Region (min): 40
Manual Baseline Start (min): 18     Manual Baseline End (min): 38
Marker Peak Width (sec): 6    Marker Min Peak Height: 100   Marker Baseline V to V?: Y    Marker Baseline V to V pts: 3
Lower Marker Selection: First Peak > 100 RFU                Upper Marker Selection: Last Peak > 100 RFU
Ladder Size (bp): 15, 200, 500, 1000, 1500, 2000, 3000, 4000, 6000
Quantification Using: Ladder          Final Concentration (ng/uL): 0.2000          Dilution Factor: 10.0
Min. RFU for Data Processing: 2
```

 Copyright 2015 Advanced Analytical Technologies, Inc.

Data File: 2017 06 29 12H 34M.raw
Sample: RT112+HD5 2_Verd1:10
Well Location: F6

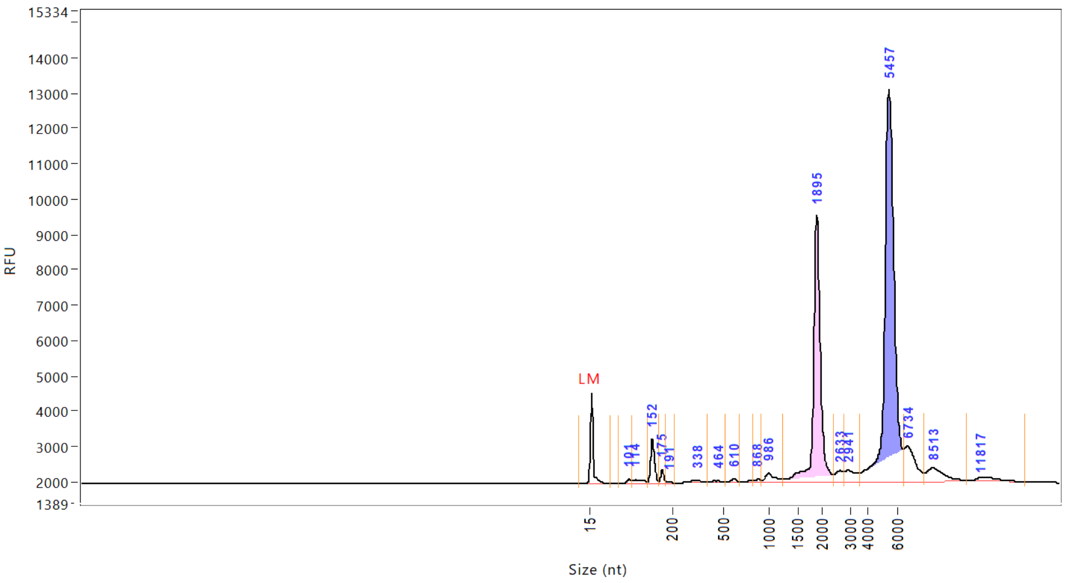

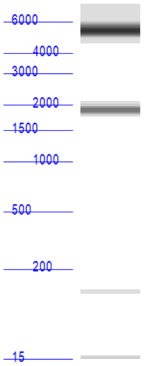

Peak	Size (nt)	Conc. (ng/uL)
	RQN	10.0

```
Sample Peak Width (sec): 6    Sample Min Peak Height: 20    Sample Baseline V to V?: Y    Sample Baseline V to V pts: 3
Sample Filter: Binomial       # of Pts for Filter: 9        Sample Start Region (min): 0  Sample End Region (min): 40
Manual Baseline Start (min): 18     Manual Baseline End (min): 38
Marker Peak Width (sec): 6    Marker Min Peak Height: 100   Marker Baseline V to V?: Y    Marker Baseline V to V pts: 3
Lower Marker Selection: First Peak > 100 RFU                Upper Marker Selection: Last Peak > 100 RFU
Ladder Size (bp): 15, 200, 500, 1000, 1500, 2000, 3000, 4000, 6000
Quantification Using: Ladder          Final Concentration (ng/uL): 0.2000          Dilution Factor: 10.0
Min. RFU for Data Processing: 2
```

 Copyright 2015 Advanced Analytical Technologies, Inc.

Data File: 2017 06 29 12H 34M.raw
Sample: RT112+HD5 3_Verd1:10
Well Location: F7

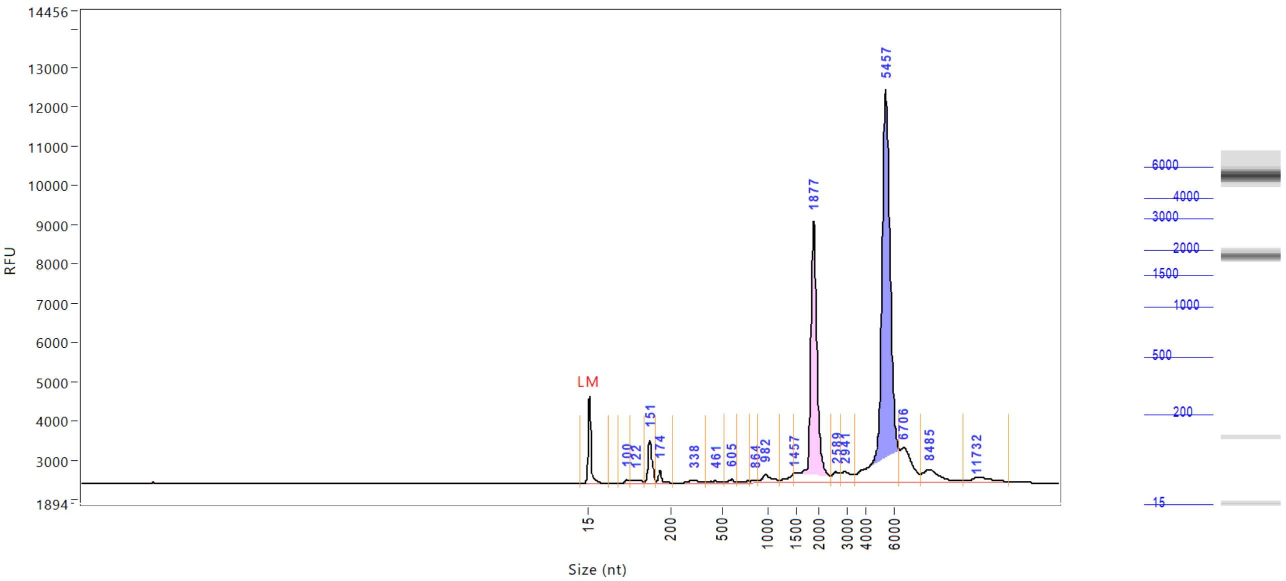

Peak	Size (nt)	Conc. (ng/uL)
1	15 (LM)	0.0329
2	100	0.0167
3	122	0.0390
4	151	0.2089
5	174	0.0468
6	338	0.0234
7	461	0.0174
8	605	0.0200
9	864	0.0149
10	982	0.0772
11	1457	0.0553
12	1877	1.6750
13	2589	0.0691
14	2941	0.1025
15	5457	3.2923
16	6706	0.3379
17	8485	0.1649
18	11732	0.0588

TIC:	6.2200	ng/uL
TIM:	12.590	nmole/L
Total Conc.:	6.2053	ng/uL

Sample Peak Width (sec): 6   Sample Min Peak Height: 20   Sample Baseline V to V?: Y   Sample Baseline V to V pts: 3
Sample Filter: Binomial   # of Pts for Filter: 9   Sample Start Region (min): 0   Sample End Region (min): 40
Manual Baseline Start (min): 18   Manual Baseline End (min): 38
Marker Peak Width (sec): 6   Marker Min Peak Height: 100   Marker Baseline V to V?: Y   Marker Baseline V to V pts: 3
Lower Marker Selection: First Peak > 100 RFU   Upper Marker Selection: Last Peak > 100 RFU
Ladder Size (bp): 15, 200, 500, 1000, 1500, 2000, 3000, 4000, 6000
Quantification Using: Ladder   Final Concentration (ng/uL): 0.2000   Dilution Factor: 10.0
Min. RFU for Data Processing: 2

Copyright 2015 Advanced Analytical Technologies, Inc.

Data File: 2017 06 29 12H 34M.raw
Sample: RT112+HD5 3_Verd1:10
Well Location: F7

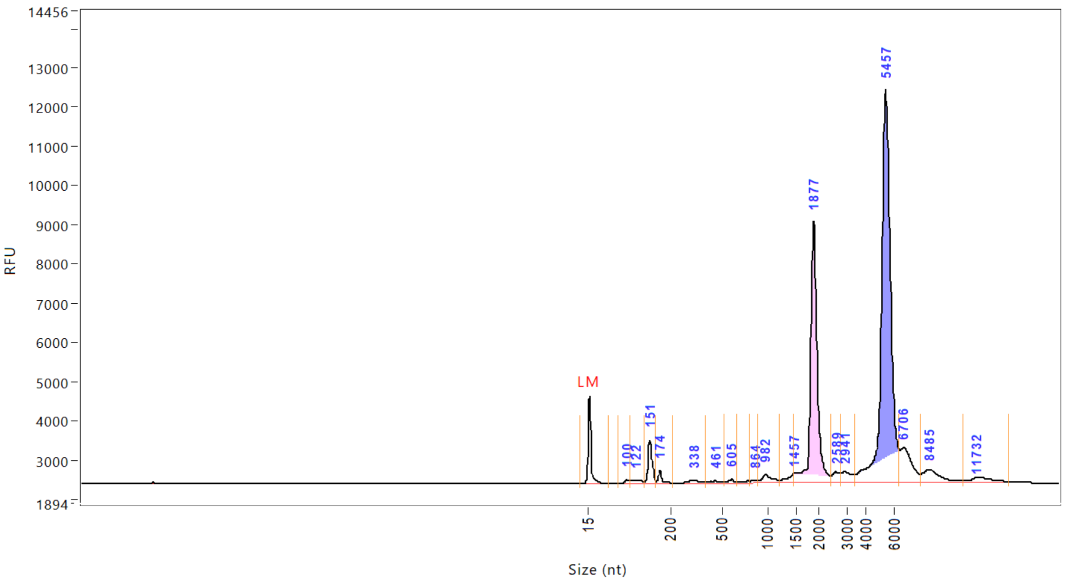

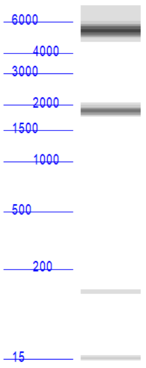

Peak	Size (nt)	Conc. (ng/uL)

28S/18S:	1.8
RQN	10.0

Sample Peak Width (sec): 6   Sample Min Peak Height: 20   Sample Baseline V to V?: Y   Sample Baseline V to V pts: 3
Sample Filter: Binomial   # of Pts for Filter: 9   Sample Start Region (min): 0   Sample End Region (min): 40
Manual Baseline Start (min): 18   Manual Baseline End (min): 38
Marker Peak Width (sec): 6   Marker Min Peak Height: 100   Marker Baseline V to V?: Y   Marker Baseline V to V pts: 3
Lower Marker Selection: First Peak > 100 RFU   Upper Marker Selection: Last Peak > 100 RFU
Ladder Size (bp): 15, 200, 500, 1000, 1500, 2000, 3000, 4000, 6000
Quantification Using: Ladder   Final Concentration (ng/uL): 0.2000   Dilution Factor: 10.0
Min. RFU for Data Processing: 2

 Copyright 2015 Advanced Analytical Technologies, Inc.

Data File: 2017 06 29 12H 34M.raw
Sample: RT112+HD5 4_Verd1:10
Well Location: F8

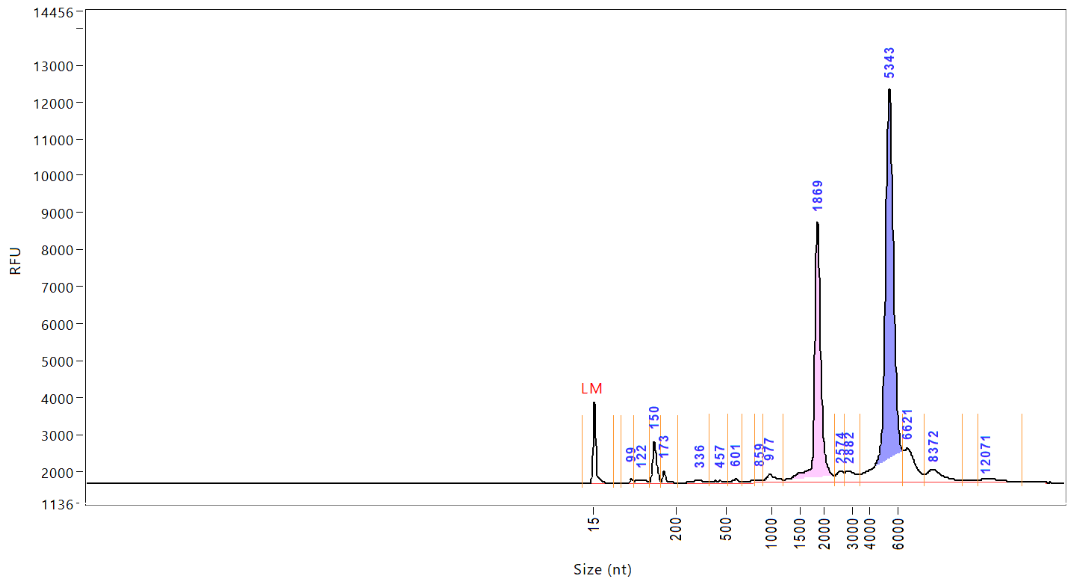

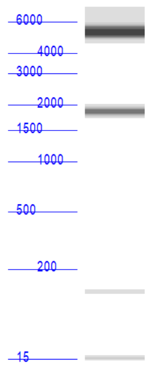

Peak	Size (nt)	Conc. (ng/uL)
1	15 (LM)	0.0329
2	99	0.0168
3	122	0.0423
4	150	0.2126
5	173	0.0466
6	336	0.0257
7	457	0.0214
8	601	0.0240
9	859	0.0181
10	977	0.0858
11	1869	1.8442
12	2574	0.0783
13	2882	0.1233
14	5343	3.4716
15	6621	0.3612
16	8372	0.1726
17	12071	0.0397

TIC:	6.5842	ng/uL
TIM:	13.269	nmole/L
Total Conc.:	6.5890	ng/uL

28S/18S:	1.7
RQN	10.0

Sample Peak Width (sec): 6   Sample Min Peak Height: 20   Sample Baseline V to V?: Y   Sample Baseline V to V pts: 3
Sample Filter: Binomial   # of Pts for Filter: 9   Sample Start Region (min): 0   Sample End Region (min): 40
Manual Baseline Start (min): 18   Manual Baseline End (min): 38
Marker Peak Width (sec): 6   Marker Min Peak Height: 100   Marker Baseline V to V?: Y   Marker Baseline V to V pts: 3
Lower Marker Selection: First Peak > 100 RFU   Upper Marker Selection: Last Peak > 100 RFU
Ladder Size (bp): 15, 200, 500, 1000, 1500, 2000, 3000, 4000, 6000
Quantification Using: Ladder   Final Concentration (ng/uL): 0.2000   Dilution Factor: 10.0
Min. RFU for Data Processing: 2

 Copyright 2015 Advanced Analytical Technologies, Inc.

Data File: 2017 06 29 12H 34M.raw
Sample: VM-Cub-1+ vector 1_Verd1:10
Well Location: F9

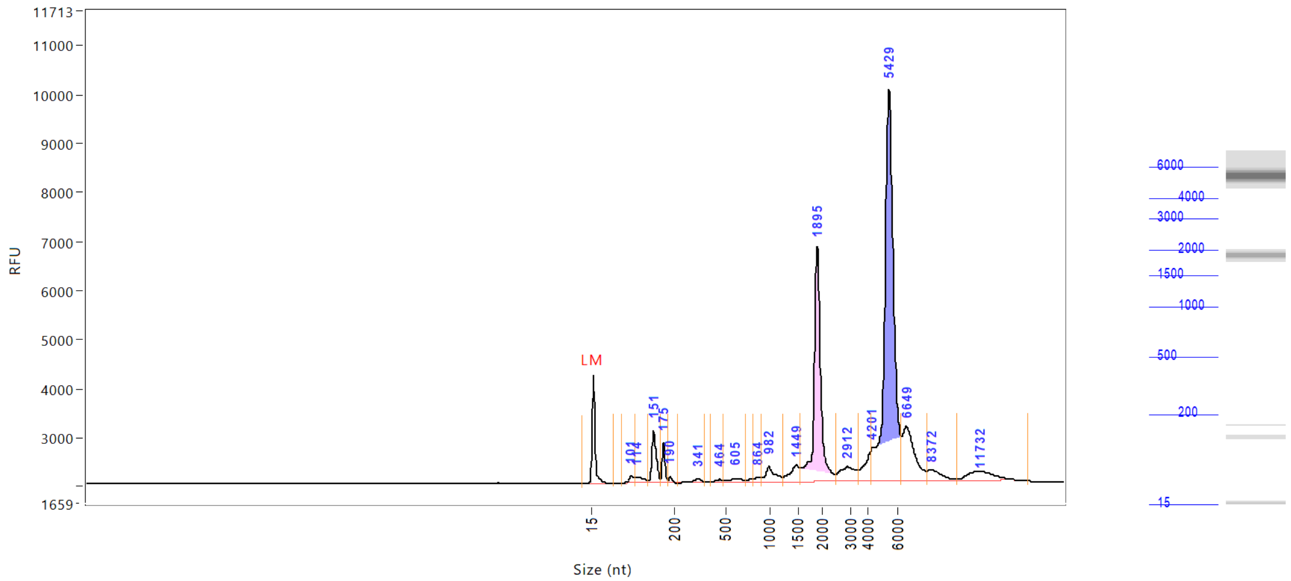

Peak	Size (nt)	Conc. (ng/uL)
1	15 (LM)	0.0329
2	101	0.0291
3	114	0.0464
4	151	0.2155
5	175	0.1063
6	190	0.0152
7	341	0.0198
8	464	0.0118
9	605	0.0359
10	864	0.0217
11	982	0.1156
12	1449	0.1277
13	1895	1.2534
14	2912	0.1670
15	4201	0.1636
16	5429	2.5962
17	6649	0.4585
18	8372	0.0921
19	11732	0.1327

TIC:	5.6087	ng/uL
TIM:	14.045	nmole/L
Total Conc.:	5.5842	ng/uL

Sample Peak Width (sec): 6   Sample Min Peak Height: 20   Sample Baseline V to V?: Y   Sample Baseline V to V pts: 3
Sample Filter: Binomial   # of Pts for Filter: 9   Sample Start Region (min): 0   Sample End Region (min): 40
Manual Baseline Start (min): 18   Manual Baseline End (min): 38
Marker Peak Width (sec): 6   Marker Min Peak Height: 100   Marker Baseline V to V?: Y   Marker Baseline V to V pts: 3
Lower Marker Selection: First Peak > 100 RFU   Upper Marker Selection: Last Peak > 100 RFU
Ladder Size (bp): 15, 200, 500, 1000, 1500, 2000, 3000, 4000, 6000
Quantification Using: Ladder   Final Concentration (ng/uL): 0.2000   Dilution Factor: 10.0
Min. RFU for Data Processing: 2

 Copyright 2015 Advanced Analytical Technologies, Inc.

Data File: 2017 06 29 12H 34M.raw
Sample: VM-Cub-1+ vector 1_Verd1:10
Well Location: F9

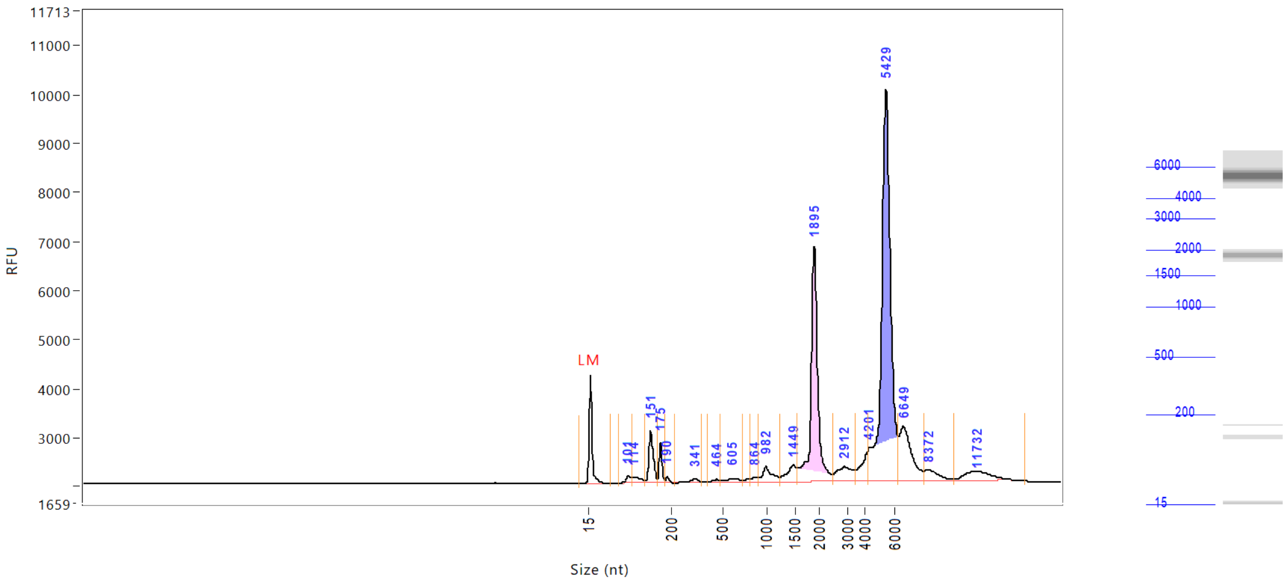

Peak	Size (nt)	Conc. (ng/uL)

28S/18S:	1.8
RQN	10.0

Sample Peak Width (sec): 6   Sample Min Peak Height: 20   Sample Baseline V to V?: Y   Sample Baseline V to V pts: 3
Sample Filter: Binomial   # of Pts for Filter: 9   Sample Start Region (min): 0   Sample End Region (min): 40
Manual Baseline Start (min): 18   Manual Baseline End (min): 38
Marker Peak Width (sec): 6   Marker Min Peak Height: 100   Marker Baseline V to V?: Y   Marker Baseline V to V pts: 3
Lower Marker Selection: First Peak > 100 RFU   Upper Marker Selection: Last Peak > 100 RFU
Ladder Size (bp): 15, 200, 500, 1000, 1500, 2000, 3000, 4000, 6000
Quantification Using: Ladder   Final Concentration (ng/uL): 0.2000   Dilution Factor: 10.0
Min. RFU for Data Processing: 2

 Copyright 2015 Advanced Analytical Technologies, Inc.

Data File: 2017 06 29 12H 34M.raw
Sample: VM-Cub-1+ vector 2_Verd1:10
Well Location: F10

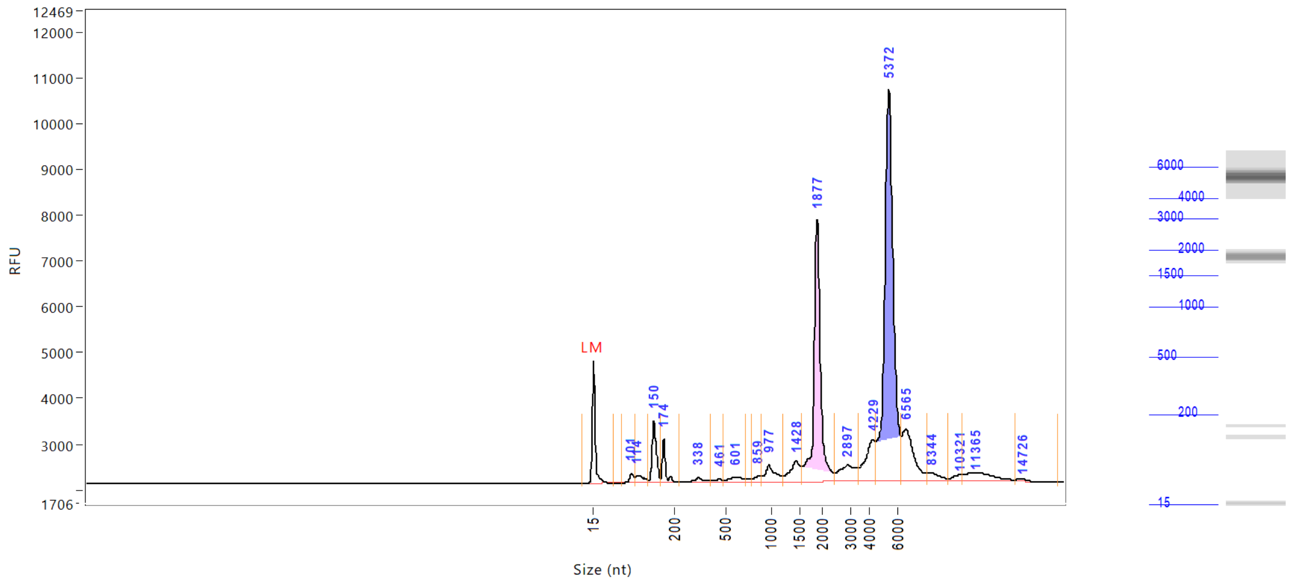

Peak	Size (nt)	Conc. (ng/uL)
1	15 (LM)	0.0329
2	101	0.0314
3	114	0.0515
4	150	0.2240
5	174	0.1178
6	338	0.0269
7	461	0.0153
8	601	0.0453
9	859	0.0284
10	977	0.1167
11	1428	0.1440
12	1877	1.2367
13	2897	0.1738
14	4229	0.2703
15	5372	2.2516
16	6565	0.3830
17	8344	0.0506
18	10321	0.0264
19	11365	0.1034
20	14726	0.0109

TIC:	5.3080	ng/uL
TIM:	14.481	nmole/L
Total Conc.:	5.2838	ng/uL

Sample Peak Width (sec): 6   Sample Min Peak Height: 20   Sample Baseline V to V?: Y   Sample Baseline V to V pts: 3
Sample Filter: Binomial   # of Pts for Filter: 9   Sample Start Region (min): 0   Sample End Region (min): 40
Manual Baseline Start (min): 18   Manual Baseline End (min): 38
Marker Peak Width (sec): 6   Marker Min Peak Height: 100   Marker Baseline V to V?: Y   Marker Baseline V to V pts: 3
Lower Marker Selection: First Peak > 100 RFU   Upper Marker Selection: Last Peak > 100 RFU
Ladder Size (bp): 15, 200, 500, 1000, 1500, 2000, 3000, 4000, 6000
Quantification Using: Ladder   Final Concentration (ng/uL): 0.2000   Dilution Factor: 10.0
Min. RFU for Data Processing: 2

 Copyright 2015 Advanced Analytical Technologies, Inc.

Data File: 2017 06 29 12H 34M.raw
Sample: VM-Cub-1+ vector 2_Verd1:10
Well Location: F10

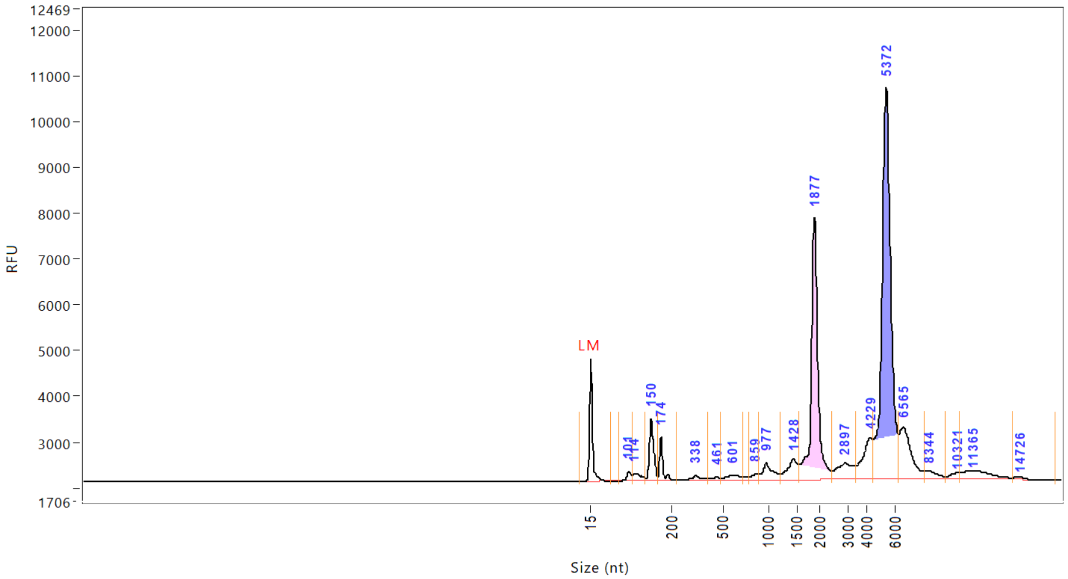

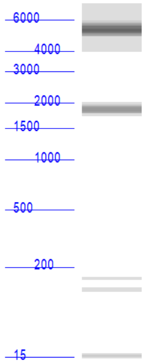

Peak	Size (nt)	Conc. (ng/uL)

28S/18S:	1.7
RQN	9.8

```
Sample Peak Width (sec): 6    Sample Min Peak Height: 20    Sample Baseline V to V?: Y    Sample Baseline V to V pts: 3
Sample Filter: Binomial       # of Pts for Filter: 9        Sample Start Region (min): 0  Sample End Region (min): 40
Manual Baseline Start (min): 18     Manual Baseline End (min): 38
Marker Peak Width (sec): 6    Marker Min Peak Height: 100   Marker Baseline V to V?: Y    Marker Baseline V to V pts: 3
Lower Marker Selection: First Peak > 100 RFU                Upper Marker Selection: Last Peak > 100 RFU
Ladder Size (bp): 15, 200, 500, 1000, 1500, 2000, 3000, 4000, 6000
Quantification Using: Ladder          Final Concentration (ng/uL): 0.2000          Dilution Factor: 10.0
Min. RFU for Data Processing: 2
```

 Copyright 2015 Advanced Analytical Technologies, Inc.

Data File: 2017 06 29 12H 34M.raw
Sample: VM-Cub-1+ vector 3_Verd1:10
Well Location: F11

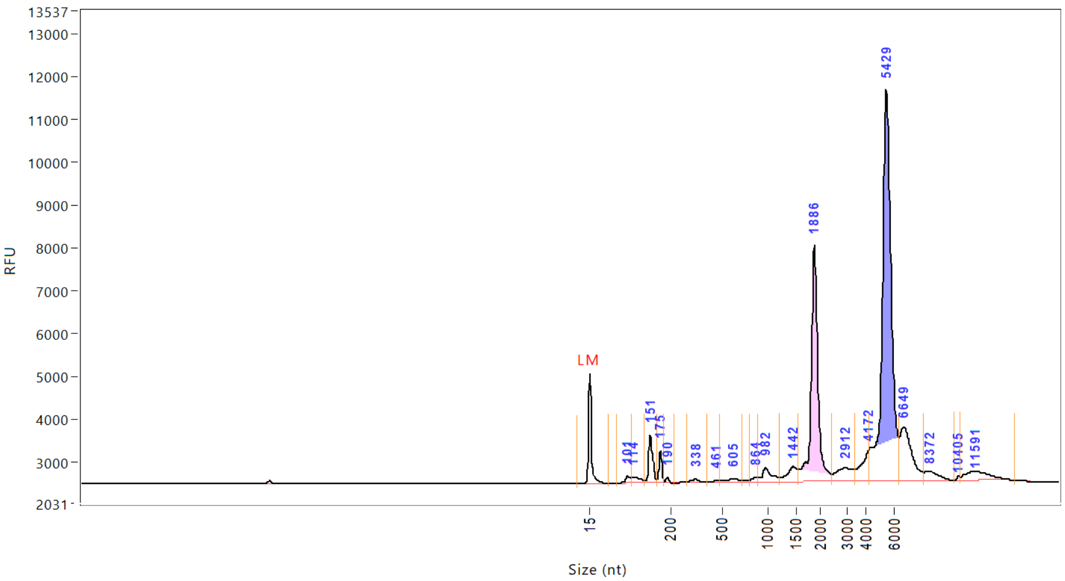

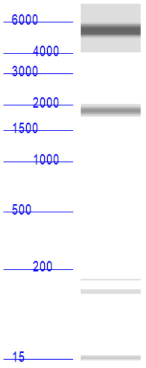

Peak	Size (nt)	Conc. (ng/uL)
1	15 (LM)	0.0329
2	101	0.0287
3	114	0.0439
4	151	0.1860
5	175	0.0833
6	190	0.0122
7	338	0.0150
8	461	0.0076
9	605	0.0275
10	864	0.0199
11	982	0.1019
12	1442	0.1125
13	1886	1.2171
14	2912	0.1611
15	4172	0.1613
16	5429	2.5471
17	6649	0.4359
18	8372	0.0727
19	10405	0.0070
20	11591	0.1153

TIC:	5.3559	ng/uL
TIM:	12.595	nmole/L
Total Conc.:	5.3294	ng/uL

```
Sample Peak Width (sec): 6    Sample Min Peak Height: 20    Sample Baseline V to V?: Y    Sample Baseline V to V pts: 3
Sample Filter: Binomial       # of Pts for Filter: 9        Sample Start Region (min): 0  Sample End Region (min): 40
Manual Baseline Start (min): 18     Manual Baseline End (min): 38
Marker Peak Width (sec): 6    Marker Min Peak Height: 100   Marker Baseline V to V?: Y    Marker Baseline V to V pts: 3
Lower Marker Selection: First Peak > 100 RFU                Upper Marker Selection: Last Peak > 100 RFU
Ladder Size (bp): 15, 200, 500, 1000, 1500, 2000, 3000, 4000, 6000
Quantification Using: Ladder          Final Concentration (ng/uL): 0.2000          Dilution Factor: 10.0
Min. RFU for Data Processing: 2
```

 Copyright 2015 Advanced Analytical Technologies, Inc.

Data File: 2017 06 29 12H 34M.raw
Sample: VM-Cub-1+ vector 3_Verd1:10
Well Location: F11

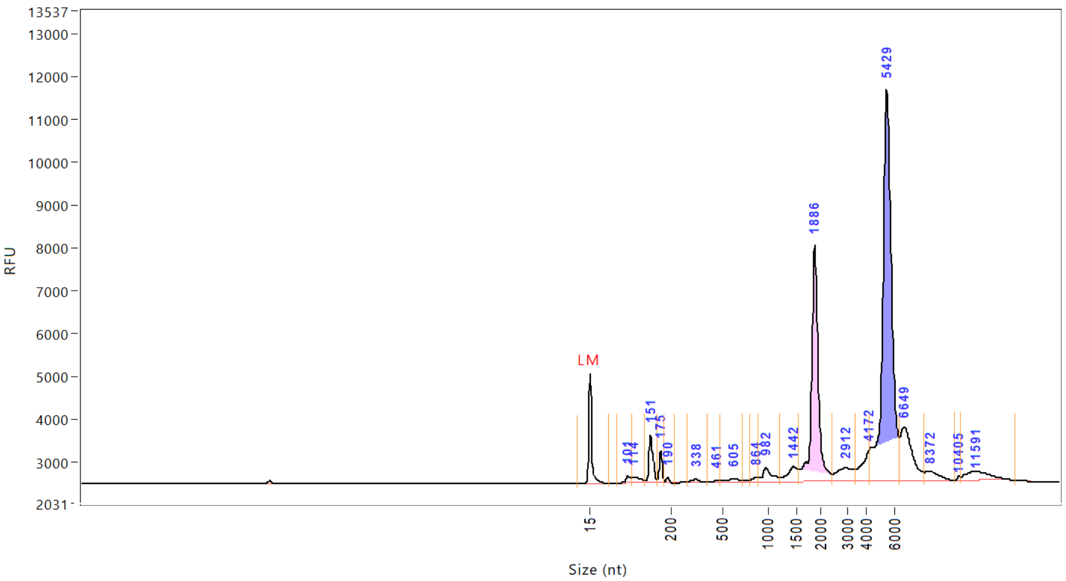

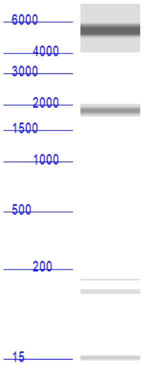

Peak	Size (nt)	Conc. (ng/uL)

28S/18S:	1.8
RQN	10.0

Sample Peak Width (sec): 6   Sample Min Peak Height: 20   Sample Baseline V to V?: Y   Sample Baseline V to V pts: 3
Sample Filter: Binomial   # of Pts for Filter: 9   Sample Start Region (min): 0   Sample End Region (min): 40
Manual Baseline Start (min): 18   Manual Baseline End (min): 38
Marker Peak Width (sec): 6   Marker Min Peak Height: 100   Marker Baseline V to V?: Y   Marker Baseline V to V pts: 3
Lower Marker Selection: First Peak > 100 RFU   Upper Marker Selection: Last Peak > 100 RFU
Ladder Size (bp): 15, 200, 500, 1000, 1500, 2000, 3000, 4000, 6000
Quantification Using: Ladder   Final Concentration (ng/uL): 0.2000   Dilution Factor: 10.0
Min. RFU for Data Processing: 2

Copyright 2015 Advanced Analytical Technologies, Inc.

Data File: 2017 06 29 13H 45M.raw
Sample: VM-Cub-1+ vector 4_Verd1:10
Well Location: G1

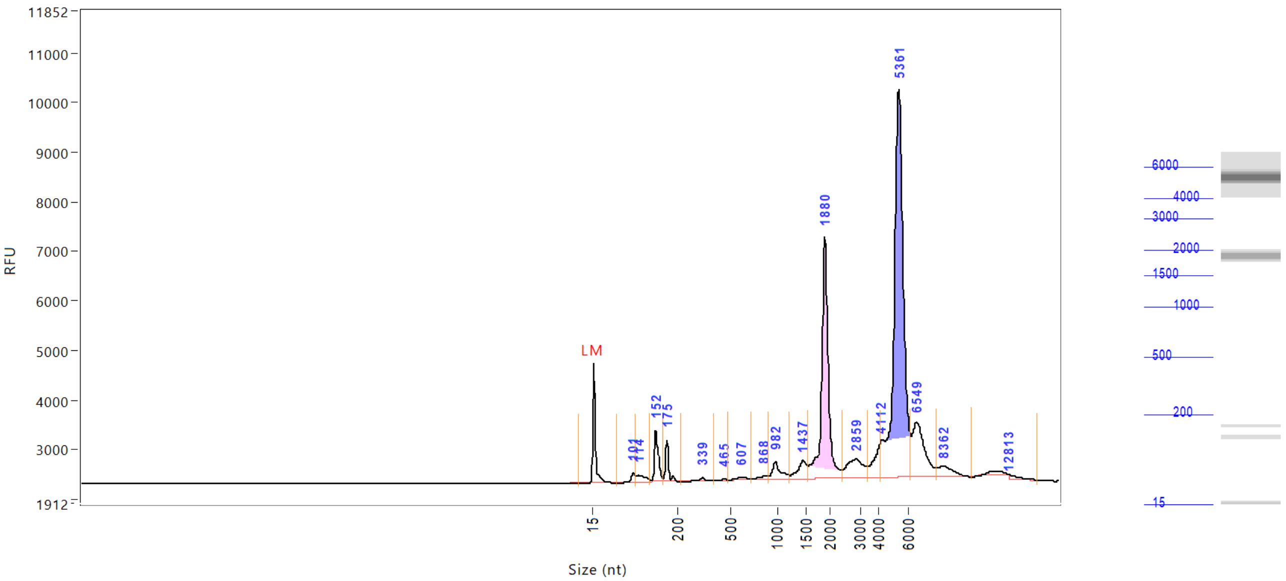

Peak	Size (nt)	Conc. (ng/uL)
1	15 (LM)	0.0310
2	101	0.0283
3	114	0.0429
4	152	0.1762
5	175	0.1042
6	339	0.0073
7	465	0.0016
8	607	0.0117
9	868	0.0178
10	982	0.1021
11	1437	0.1098
12	1880	1.1244
13	2859	0.1971
14	4112	0.1405
15	5361	2.2441
16	6549	0.4002
17	8362	0.0794
18	12813	0.0440

TIC:	4.8315	ng/uL
TIM:	11.996	nmole/L
Total Conc.:	4.8031	ng/uL

28S/18S: 1.8

Sample Peak Width (sec): 6 Sample Min Peak Height: 20 Sample Baseline V to V?: Y Sample Baseline V to V pts: 3
Sample Filter: Binomial # of Pts for Filter: 9 Sample Start Region (min): 0 Sample End Region (min): 40
Manual Baseline Start (min): 18 Manual Baseline End (min): 38
Marker Peak Width (sec): 6 Marker Min Peak Height: 100 Marker Baseline V to V?: Y Marker Baseline V to V pts: 3
Lower Marker Selection: First Peak > 100 RFU Upper Marker Selection: Last Peak > 100 RFU
Ladder Size (bp): 15, 200, 500, 1000, 1500, 2000, 3000, 4000, 6000
Quantification Using: Ladder Final Concentration (ng/uL): 0.2000 Dilution Factor: 10.0
Min. RFU for Data Processing: 2

 Copyright 2015 Advanced Analytical Technologies, Inc.

Data File: 2017 06 29 13H 45M.raw
Sample: VM-Cub-1+ vector 4_Verd1:10
Well Location: G1

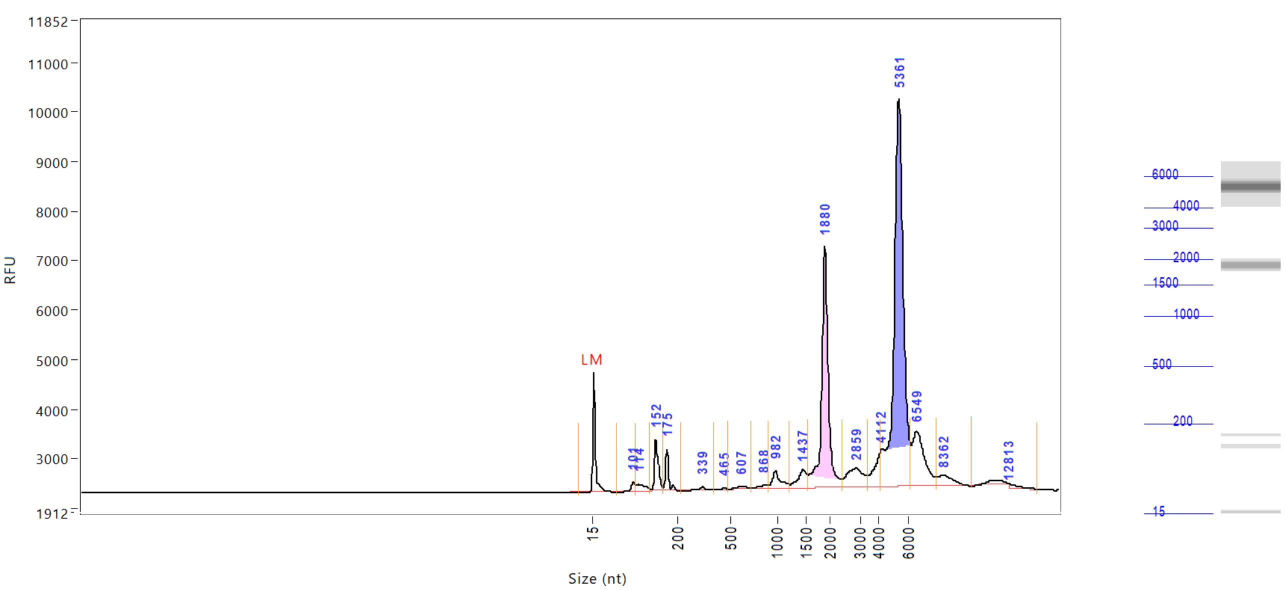

Peak	Size (nt)	Conc. (ng/uL)
	RQN	10.0

```
Sample Peak Width (sec): 6    Sample Min Peak Height: 20    Sample Baseline V to V?: Y    Sample Baseline V to V pts: 3
Sample Filter: Binomial       # of Pts for Filter: 9        Sample Start Region (min): 0  Sample End Region (min): 40
Manual Baseline Start (min): 18     Manual Baseline End (min): 38
Marker Peak Width (sec): 6    Marker Min Peak Height: 100   Marker Baseline V to V?: Y    Marker Baseline V to V pts: 3
Lower Marker Selection: First Peak > 100 RFU                Upper Marker Selection: Last Peak > 100 RFU
Ladder Size (bp): 15, 200, 500, 1000, 1500, 2000, 3000, 4000, 6000
Quantification Using: Ladder          Final Concentration (ng/uL): 0.2000          Dilution Factor: 10.0
Min. RFU for Data Processing: 2
```

 Copyright 2015 Advanced Analytical Technologies, Inc.

Data File: 2017 06 29 13H 45M.raw
Sample: VM-Cub-1+HD5 1_Verd1:10
Well Location: G2

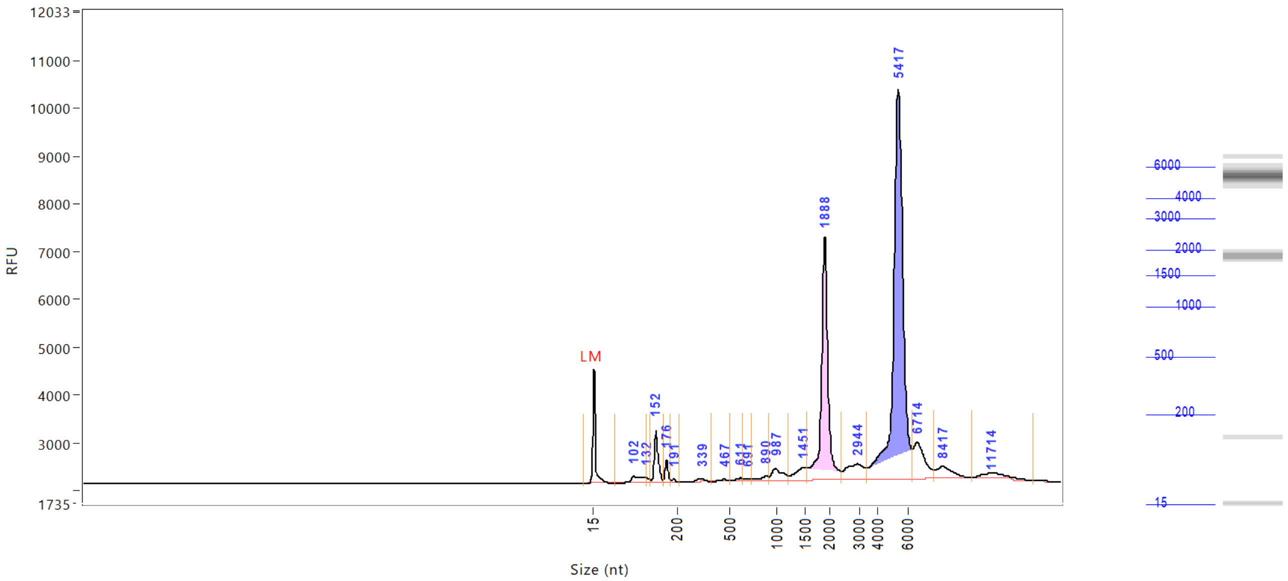

Peak	Size (nt)	Conc. (ng/uL)
1	15 (LM)	0.0310
2	102	0.0555
3	132	0.0097
4	152	0.1862
5	176	0.0528
6	191	0.0068
7	339	0.0139
8	467	0.0052
9	611	0.0112
10	691	0.0090
11	890	0.0234
12	987	0.0916
13	1451	0.1019
14	1888	1.1577
15	2944	0.1864
16	5417	2.3820
17	6714	0.2689
18	8417	0.1057
19	11714	0.0505

TIC:	4.7185	ng/uL
TIM:	11.293	nmole/L
Total Conc.:	4.6992	ng/uL

```
Sample Peak Width (sec): 6    Sample Min Peak Height: 20    Sample Baseline V to V?: Y    Sample Baseline V to V pts: 3
Sample Filter: Binomial       # of Pts for Filter: 9        Sample Start Region (min): 0  Sample End Region (min): 40
Manual Baseline Start (min): 18     Manual Baseline End (min): 38
Marker Peak Width (sec): 6    Marker Min Peak Height: 100   Marker Baseline V to V?: Y    Marker Baseline V to V pts: 3
Lower Marker Selection: First Peak > 100 RFU                Upper Marker Selection: Last Peak > 100 RFU
Ladder Size (bp): 15, 200, 500, 1000, 1500, 2000, 3000, 4000, 6000
Quantification Using: Ladder          Final Concentration (ng/uL): 0.2000          Dilution Factor: 10.0
Min. RFU for Data Processing: 2
```

 Copyright 2015 Advanced Analytical Technologies, Inc.

Data File: 2017 06 29 13H 45M.raw
Sample: VM-Cub-1+HD5 1_Verd1:10
Well Location: G2

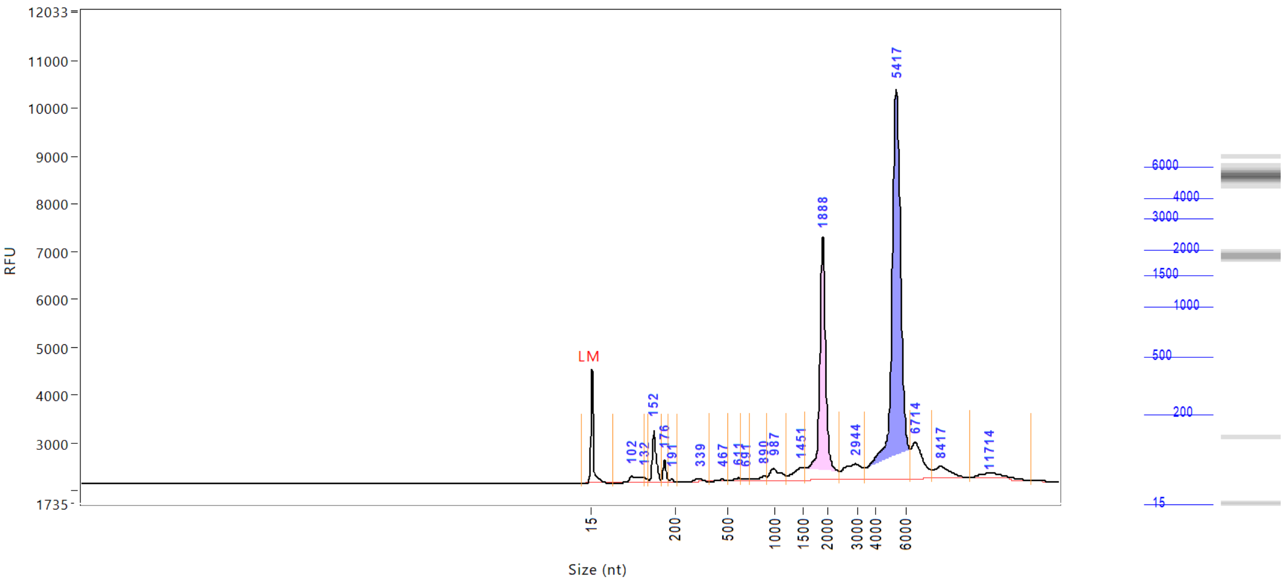

Peak	Size (nt)	Conc. (ng/uL)
	28S/18S:	2.0
	RQN	10.0

Sample Peak Width (sec): 6 Sample Min Peak Height: 20 Sample Baseline V to V?: Y Sample Baseline V to V pts: 3
Sample Filter: Binomial # of Pts for Filter: 9 Sample Start Region (min): 0 Sample End Region (min): 40
Manual Baseline Start (min): 18 Manual Baseline End (min): 38
Marker Peak Width (sec): 6 Marker Min Peak Height: 100 Marker Baseline V to V?: Y Marker Baseline V to V pts: 3
Lower Marker Selection: First Peak > 100 RFU Upper Marker Selection: Last Peak > 100 RFU
Ladder Size (bp): 15, 200, 500, 1000, 1500, 2000, 3000, 4000, 6000
Quantification Using: Ladder Final Concentration (ng/uL): 0.2000 Dilution Factor: 10.0
Min. RFU for Data Processing: 2

 Copyright 2015 Advanced Analytical Technologies, Inc.

Data File: 2017 06 29 13H 45M.raw
Sample: VM-Cub-1+HD5 2_Verd1:10
Well Location: G3

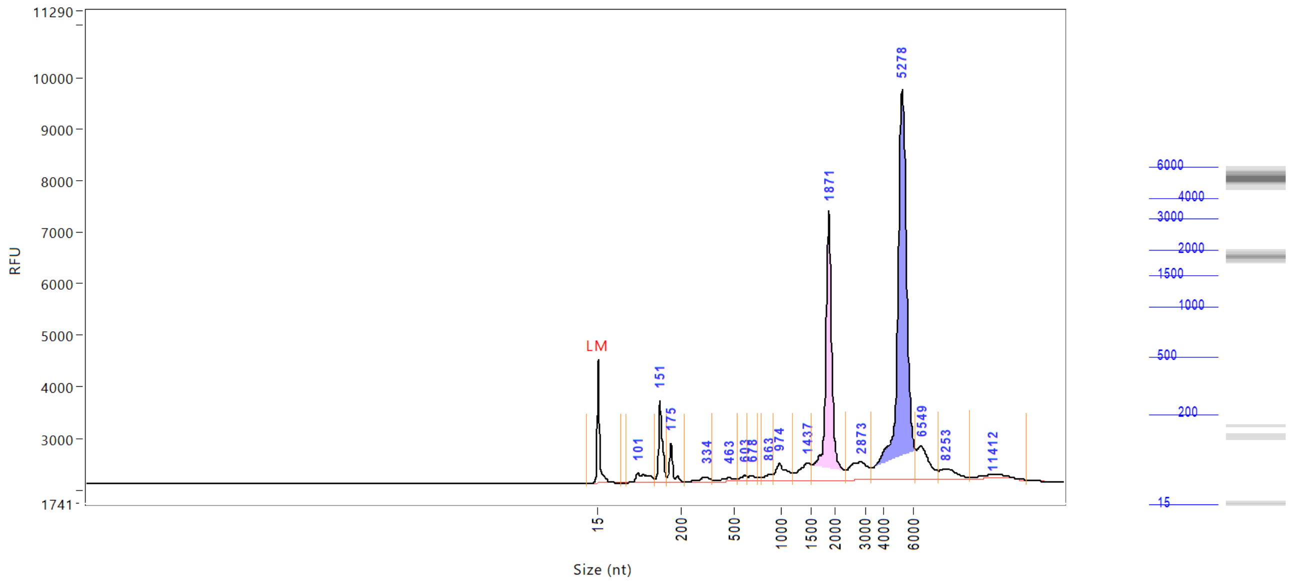

Peak	Size (nt)	Conc. (ng/uL)
1	15 (LM)	0.0310
2	101	0.0887
3	151	0.2709
4	175	0.1046
5	334	0.0317
6	463	0.0323
7	603	0.0266
8	678	0.0233
9	863	0.0328
10	974	0.1215
11	1437	0.1341
12	1871	1.1908
13	2873	0.1964
14	5278	2.2222
15	6549	0.2290
16	8253	0.0809
17	11412	0.0424

TIC:	4.8281	ng/uL
TIM:	15.393	nmole/L
Total Conc.:	4.8164	ng/uL

28S/18S:	1.8
RQN	9.7

```
Sample Peak Width (sec): 6    Sample Min Peak Height: 20    Sample Baseline V to V?: Y    Sample Baseline V to V pts: 3
Sample Filter: Binomial       # of Pts for Filter: 9        Sample Start Region (min): 0  Sample End Region (min): 40
Manual Baseline Start (min): 18     Manual Baseline End (min): 38
Marker Peak Width (sec): 6    Marker Min Peak Height: 100   Marker Baseline V to V?: Y    Marker Baseline V to V pts: 3
Lower Marker Selection: First Peak > 100 RFU                Upper Marker Selection: Last Peak > 100 RFU
Ladder Size (bp): 15, 200, 500, 1000, 1500, 2000, 3000, 4000, 6000
Quantification Using: Ladder          Final Concentration (ng/uL): 0.2000          Dilution Factor: 10.0
Min. RFU for Data Processing: 2
```

 Copyright 2015 Advanced Analytical Technologies, Inc.

Data File: 2017 06 29 13H 45M.raw
Sample: VM-Cub-1+HD5 3_Verd1:10
Well Location: G4

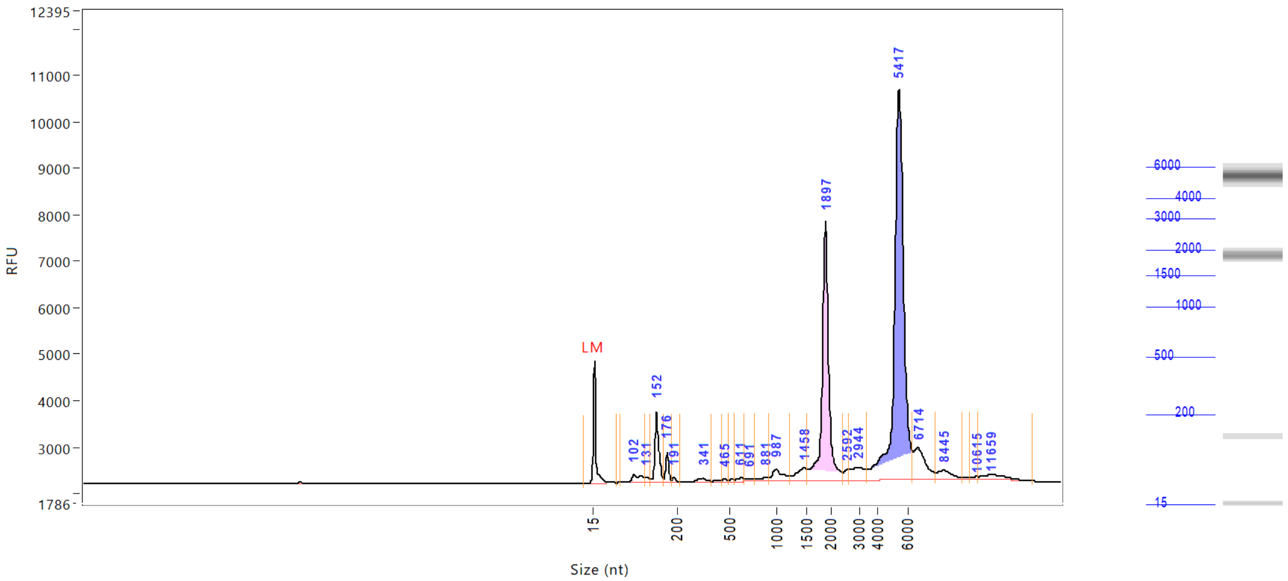

Peak	Size (nt)	Conc. (ng/uL)
1	15 (LM)	0.0310
2	102	0.0576
3	131	0.0174
4	152	0.2375
5	176	0.0699
6	191	0.0109
7	341	0.0206
8	465	0.0069
9	611	0.0140
10	691	0.0110
11	881	0.0215
12	987	0.0790
13	1458	0.0872
14	1897	1.1043
15	2592	0.0263
16	2944	0.1187
17	5417	2.1136
18	6714	0.2078
19	8445	0.0625
20	10615	0.0062
21	11659	0.0365

TIC:	4.3094	ng/uL
TIM:	12.643	nmole/L

Sample Peak Width (sec): 6 Sample Min Peak Height: 20 Sample Baseline V to V?: Y Sample Baseline V to V pts: 3
Sample Filter: Binomial # of Pts for Filter: 9 Sample Start Region (min): 0 Sample End Region (min): 40
Manual Baseline Start (min): 18 Manual Baseline End (min): 38
Marker Peak Width (sec): 6 Marker Min Peak Height: 100 Marker Baseline V to V?: Y Marker Baseline V to V pts: 3
Lower Marker Selection: First Peak > 100 RFU Upper Marker Selection: Last Peak > 100 RFU
Ladder Size (bp): 15, 200, 500, 1000, 1500, 2000, 3000, 4000, 6000
Quantification Using: Ladder Final Concentration (ng/uL): 0.2000 Dilution Factor: 10.0
Min. RFU for Data Processing: 2

 Copyright 2015 Advanced Analytical Technologies, Inc.

Data File: 2017 06 29 13H 45M.raw
Sample: VM-Cub-1+HD5 3_Verd1:10
Well Location: G4

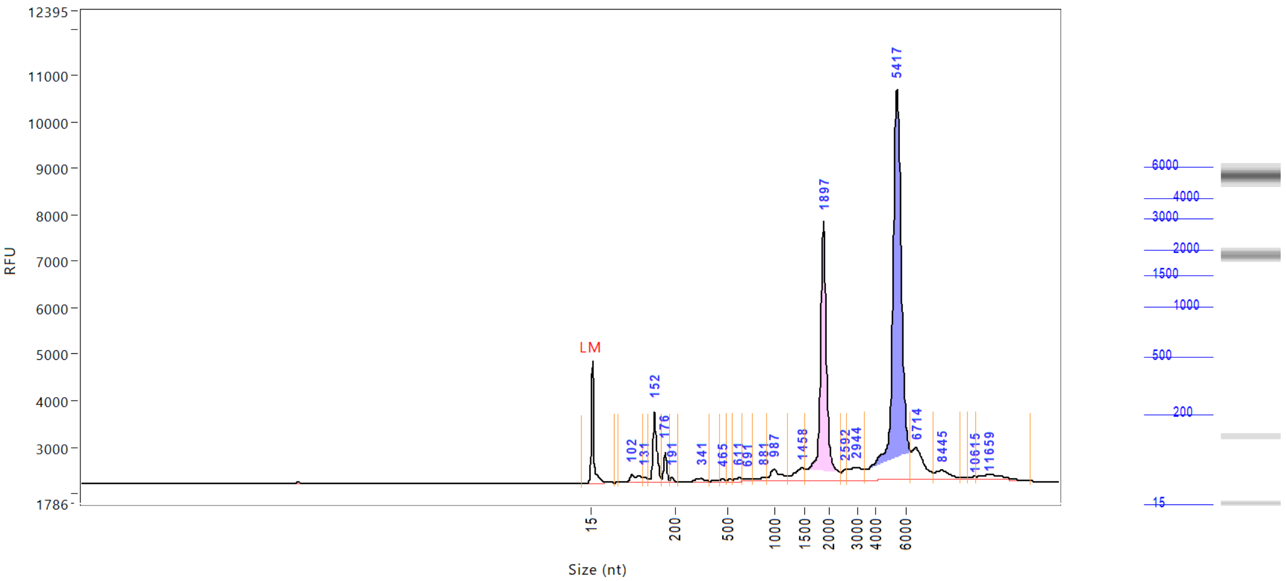

Peak	Size (nt)	Conc. (ng/uL)

Total Conc.: 4.3007 ng/uL

28S/18S: 1.8
RQN 10.0

Sample Peak Width (sec): 6   Sample Min Peak Height: 20   Sample Baseline V to V?: Y   Sample Baseline V to V pts: 3
Sample Filter: Binomial   # of Pts for Filter: 9   Sample Start Region (min): 0   Sample End Region (min): 40
Manual Baseline Start (min): 18   Manual Baseline End (min): 38
Marker Peak Width (sec): 6   Marker Min Peak Height: 100   Marker Baseline V to V?: Y   Marker Baseline V to V pts: 3
Lower Marker Selection: First Peak > 100 RFU   Upper Marker Selection: Last Peak > 100 RFU
Ladder Size (bp): 15, 200, 500, 1000, 1500, 2000, 3000, 4000, 6000
Quantification Using: Ladder   Final Concentration (ng/uL): 0.2000   Dilution Factor: 10.0
Min. RFU for Data Processing: 2

 Copyright 2015 Advanced Analytical Technologies, Inc.

Data File: 2017 06 29 13H 45M.raw
Sample: VM-Cub-1+HD5 4_Verd1:10
Well Location: G5

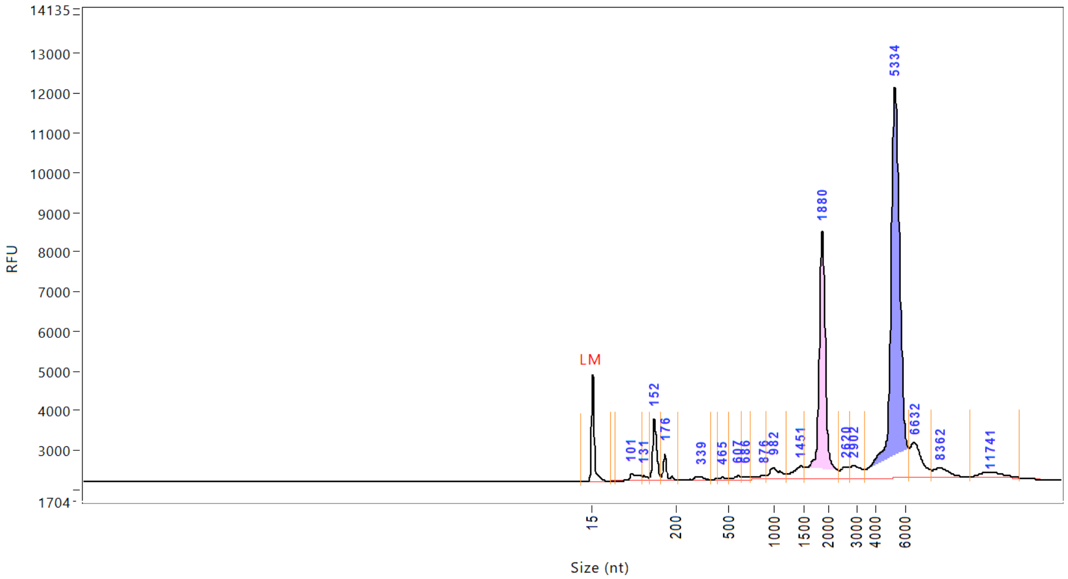

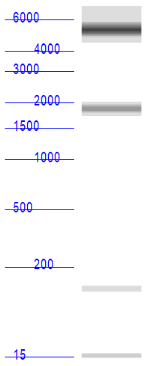

Peak	Size (nt)	Conc. (ng/uL)
1	15 (LM)	0.0310
2	101	0.0561
3	131	0.0179
4	152	0.2424
5	176	0.0817
6	339	0.0236
7	465	0.0104
8	607	0.0183
9	686	0.0132
10	876	0.0267
11	982	0.0959
12	1451	0.1057
13	1880	1.2563
14	2620	0.0671
15	2902	0.0987
16	5334	2.5105
17	6632	0.2736
18	8362	0.0849
19	11741	0.0505

TIC:	5.0336	ng/uL
TIM:	13.549	nmole/L
Total Conc.:	5.0126	ng/uL

Sample Peak Width (sec): 6   Sample Min Peak Height: 20   Sample Baseline V to V?: Y   Sample Baseline V to V pts: 3
Sample Filter: Binomial   # of Pts for Filter: 9   Sample Start Region (min): 0   Sample End Region (min): 40
Manual Baseline Start (min): 18   Manual Baseline End (min): 38
Marker Peak Width (sec): 6   Marker Min Peak Height: 100   Marker Baseline V to V?: Y   Marker Baseline V to V pts: 3
Lower Marker Selection: First Peak > 100 RFU   Upper Marker Selection: Last Peak > 100 RFU
Ladder Size (bp): 15, 200, 500, 1000, 1500, 2000, 3000, 4000, 6000
Quantification Using: Ladder   Final Concentration (ng/uL): 0.2000   Dilution Factor: 10.0
Min. RFU for Data Processing: 2

 Copyright 2015 Advanced Analytical Technologies, Inc.

Data File: 2017 06 29 13H 45M.raw
Sample: VM-Cub-1+HD5 4_Verd1:10
Well Location: G5

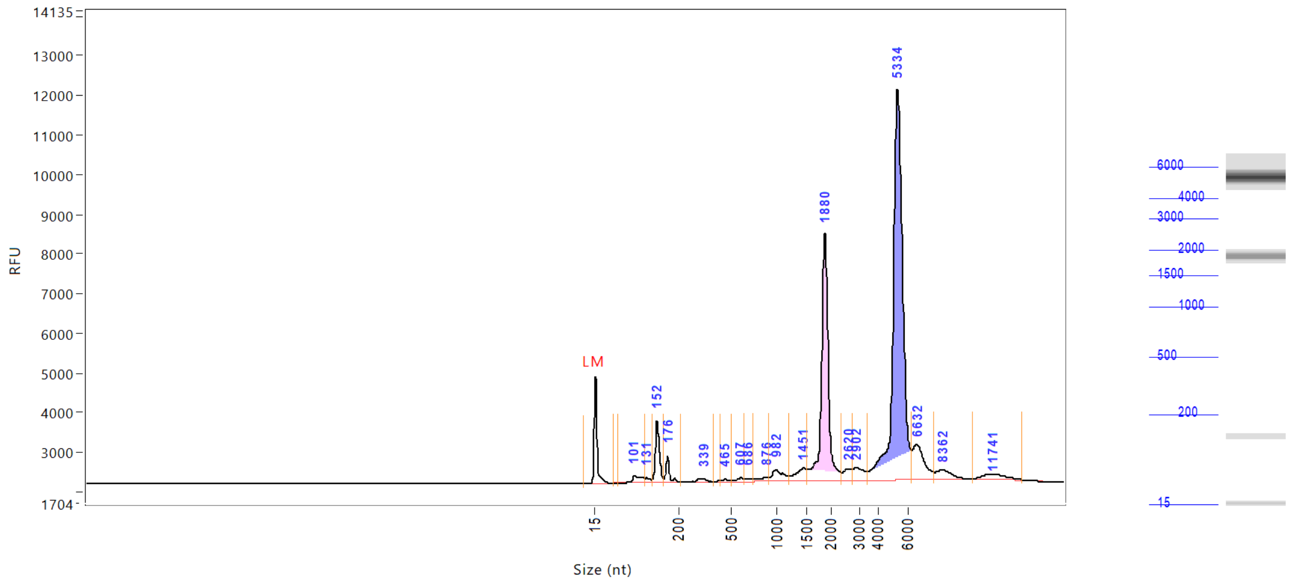

Peak	Size (nt)	Conc. (ng/uL)
	28S/18S:	1.9
	RQN	10.0

Sample Peak Width (sec): 6   Sample Min Peak Height: 20   Sample Baseline V to V?: Y   Sample Baseline V to V pts: 3
Sample Filter: Binomial   # of Pts for Filter: 9   Sample Start Region (min): 0   Sample End Region (min): 40
Manual Baseline Start (min): 18   Manual Baseline End (min): 38
Marker Peak Width (sec): 6   Marker Min Peak Height: 100   Marker Baseline V to V?: Y   Marker Baseline V to V pts: 3
Lower Marker Selection: First Peak > 100 RFU   Upper Marker Selection: Last Peak > 100 RFU
Ladder Size (bp): 15, 200, 500, 1000, 1500, 2000, 3000, 4000, 6000
Quantification Using: Ladder   Final Concentration (ng/uL): 0.2000   Dilution Factor: 10.0
Min. RFU for Data Processing: 2

 Copyright 2015 Advanced Analytical Technologies, Inc.

Data File: 2017 06 29 13H 45M.raw
Sample: SW1710+vector 1_Verd1:10
Well Location: G6

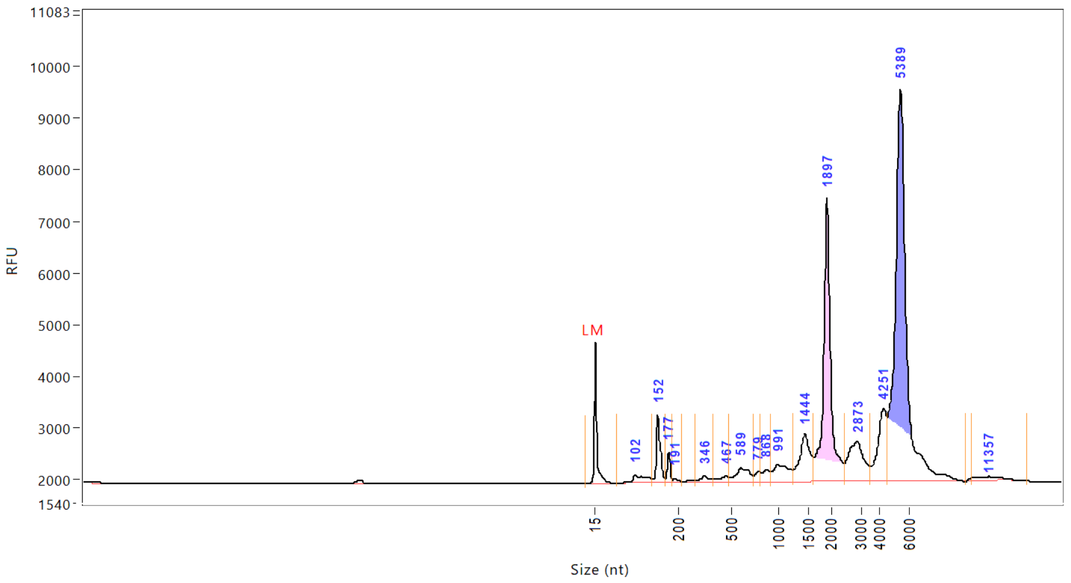

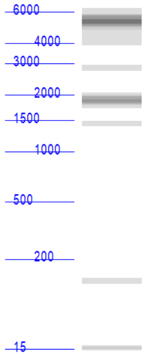

Peak	Size (nt)	Conc. (ng/uL)
1	15 (LM)	0.0310
2	102	0.0644
3	152	0.2012
4	177	0.0611
5	191	0.0116
6	346	0.0329
7	467	0.0331
8	589	0.1101
9	779	0.0359
10	868	0.0531
11	991	0.1504
12	1444	0.2889
13	1897	1.1573
14	2873	0.3249
15	4251	0.3334
16	5389	2.1712
17	11357	0.0317

TIC:	5.0612	ng/uL
TIM:	13.626	nmole/L
Total Conc.:	5.0379	ng/uL

28S/18S: 1.6

```
Sample Peak Width (sec): 6    Sample Min Peak Height: 20    Sample Baseline V to V?: Y    Sample Baseline V to V pts: 3
Sample Filter: Binomial       # of Pts for Filter: 9        Sample Start Region (min): 0  Sample End Region (min): 40
Manual Baseline Start (min): 18     Manual Baseline End (min): 38
Marker Peak Width (sec): 6    Marker Min Peak Height: 100   Marker Baseline V to V?: Y    Marker Baseline V to V pts: 3
Lower Marker Selection: First Peak > 100 RFU                Upper Marker Selection: Last Peak > 100 RFU
Ladder Size (bp): 15, 200, 500, 1000, 1500, 2000, 3000, 4000, 6000
Quantification Using: Ladder          Final Concentration (ng/uL): 0.2000          Dilution Factor: 10.0
Min. RFU for Data Processing: 2
```

 Copyright 2015 Advanced Analytical Technologies, Inc.

Data File: 2017 06 29 13H 45M.raw
Sample: SW1710+vector 1_Verd1:10
Well Location: G6

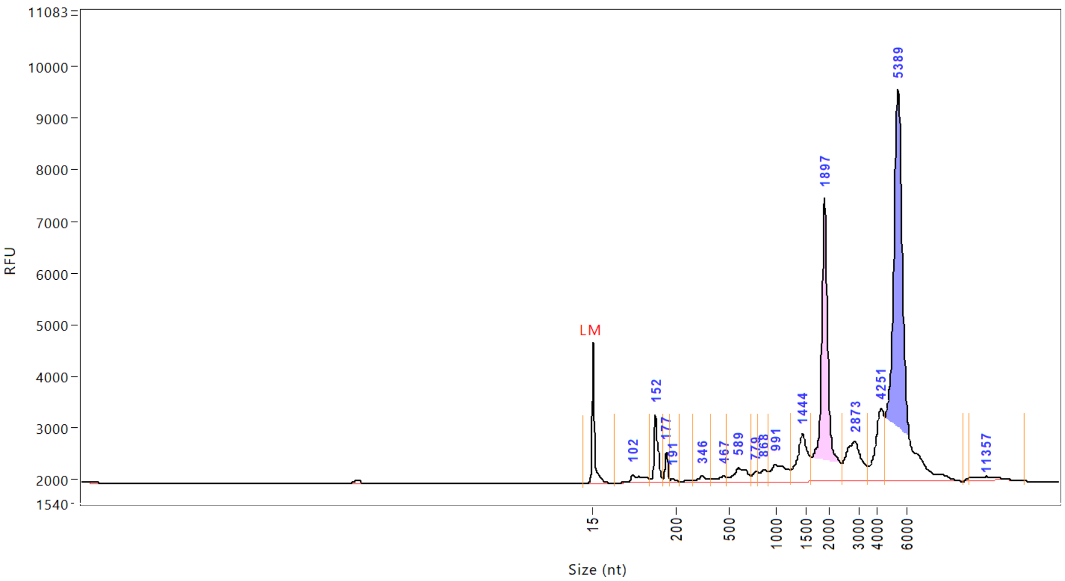

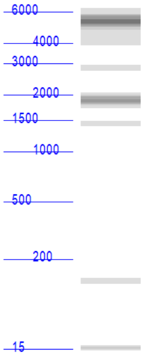

Peak	Size (nt)	Conc. (ng/uL)
	RQN	9.2

Sample Peak Width (sec): 6 Sample Min Peak Height: 20 Sample Baseline V to V?: Y Sample Baseline V to V pts: 3
Sample Filter: Binomial # of Pts for Filter: 9 Sample Start Region (min): 0 Sample End Region (min): 40
Manual Baseline Start (min): 18 Manual Baseline End (min): 38
Marker Peak Width (sec): 6 Marker Min Peak Height: 100 Marker Baseline V to V?: Y Marker Baseline V to V pts: 3
Lower Marker Selection: First Peak > 100 RFU Upper Marker Selection: Last Peak > 100 RFU
Ladder Size (bp): 15, 200, 500, 1000, 1500, 2000, 3000, 4000, 6000
Quantification Using: Ladder Final Concentration (ng/uL): 0.2000 Dilution Factor: 10.0
Min. RFU for Data Processing: 2

 Copyright 2015 Advanced Analytical Technologies, Inc.

Data File: 2017 06 29 13H 45M.raw
Sample: SW1710+vector 2_Verd1:10
Well Location: G7

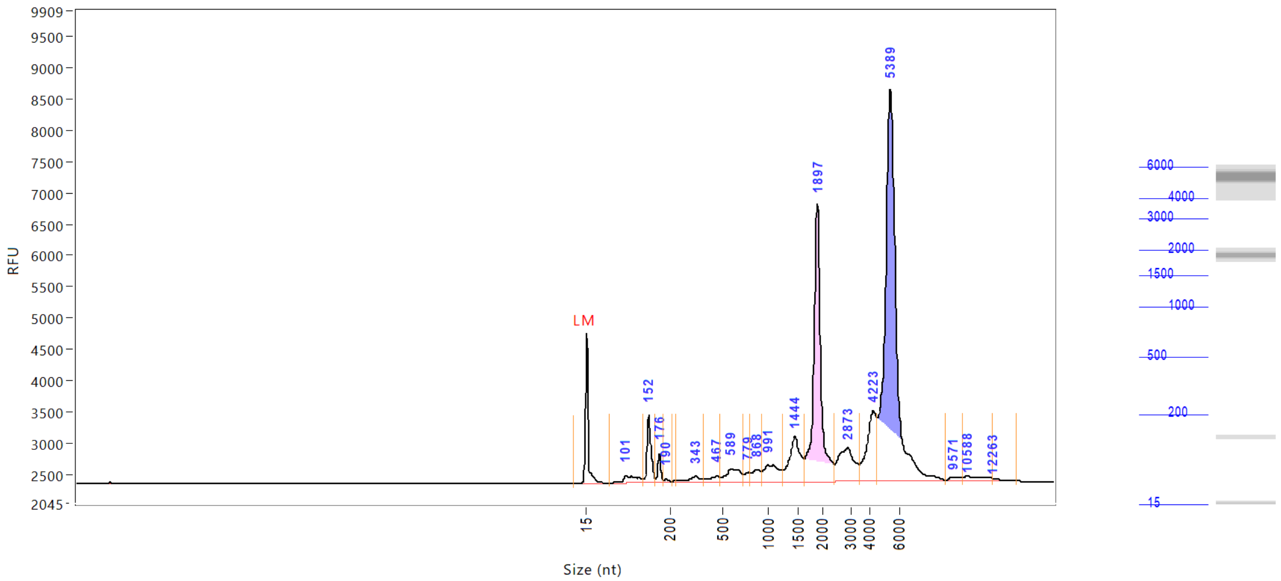

Peak	Size (nt)	Conc. (ng/uL)
1	15 (LM)	0.0310
2	101	0.0612
3	152	0.1886
4	176	0.0563
5	190	0.0106
6	343	0.0414
7	467	0.0346
8	589	0.1053
9	779	0.0306
10	868	0.0550
11	991	0.1373
12	1444	0.2648
13	1897	1.0750
14	2873	0.2664
15	4223	0.3324
16	5389	2.0161
17	9571	0.0135
18	10588	0.0308
19	12263	0.0038

TIC:	4.7236	ng/uL
TIM:	12.890	nmole/L
Total Conc.:	4.6948	ng/uL

Sample Peak Width (sec): 6 Sample Min Peak Height: 20 Sample Baseline V to V?: Y Sample Baseline V to V pts: 3
Sample Filter: Binomial # of Pts for Filter: 9 Sample Start Region (min): 0 Sample End Region (min): 40
Manual Baseline Start (min): 18 Manual Baseline End (min): 38
Marker Peak Width (sec): 6 Marker Min Peak Height: 100 Marker Baseline V to V?: Y Marker Baseline V to V pts: 3
Lower Marker Selection: First Peak > 100 RFU Upper Marker Selection: Last Peak > 100 RFU
Ladder Size (bp): 15, 200, 500, 1000, 1500, 2000, 3000, 4000, 6000
Quantification Using: Ladder Final Concentration (ng/uL): 0.2000 Dilution Factor: 10.0
Min. RFU for Data Processing: 2

 Copyright 2015 Advanced Analytical Technologies, Inc.

Data File: 2017 06 29 13H 45M.raw
Sample: SW1710+vector 2_Verd1:10
Well Location: G7

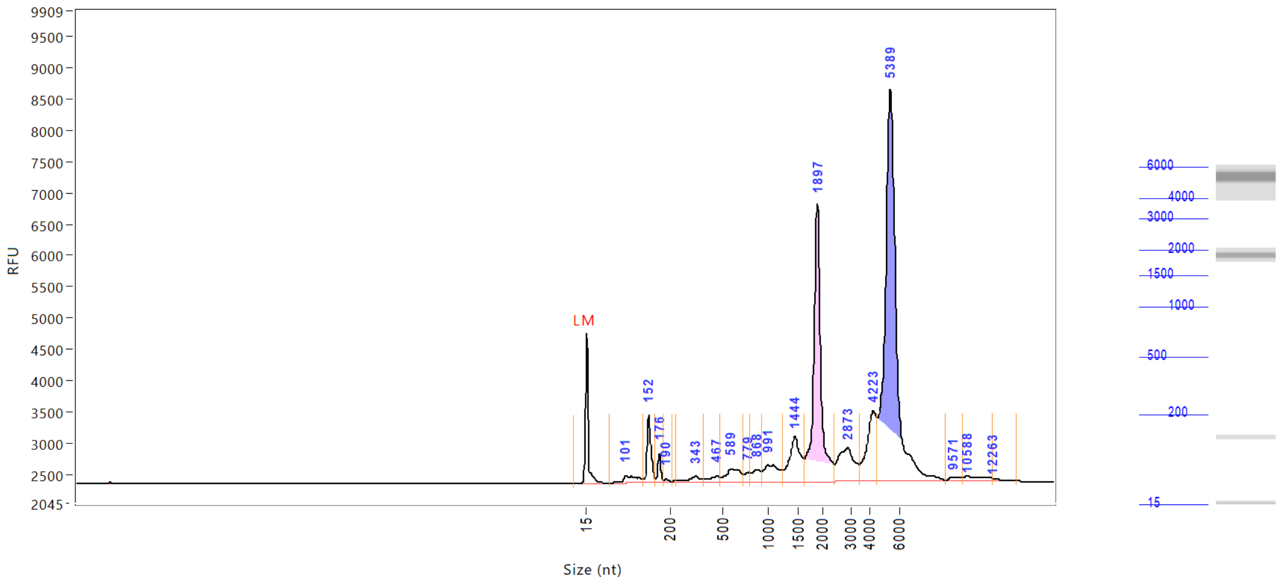

Peak	Size (nt)	Conc. (ng/uL)
	28S/18S:	1.6
	RQN	9.1

Sample Peak Width (sec): 6   Sample Min Peak Height: 20   Sample Baseline V to V?: Y   Sample Baseline V to V pts: 3
Sample Filter: Binomial   # of Pts for Filter: 9   Sample Start Region (min): 0   Sample End Region (min): 40
Manual Baseline Start (min): 18   Manual Baseline End (min): 38
Marker Peak Width (sec): 6   Marker Min Peak Height: 100   Marker Baseline V to V?: Y   Marker Baseline V to V pts: 3
Lower Marker Selection: First Peak > 100 RFU   Upper Marker Selection: Last Peak > 100 RFU
Ladder Size (bp): 15, 200, 500, 1000, 1500, 2000, 3000, 4000, 6000
Quantification Using: Ladder   Final Concentration (ng/uL): 0.2000   Dilution Factor: 10.0
Min. RFU for Data Processing: 2

 Copyright 2015 Advanced Analytical Technologies, Inc.

Data File: 2017 06 29 13H 45M.raw
Sample: SW1710+vector 3_Verd1:10
Well Location: G8

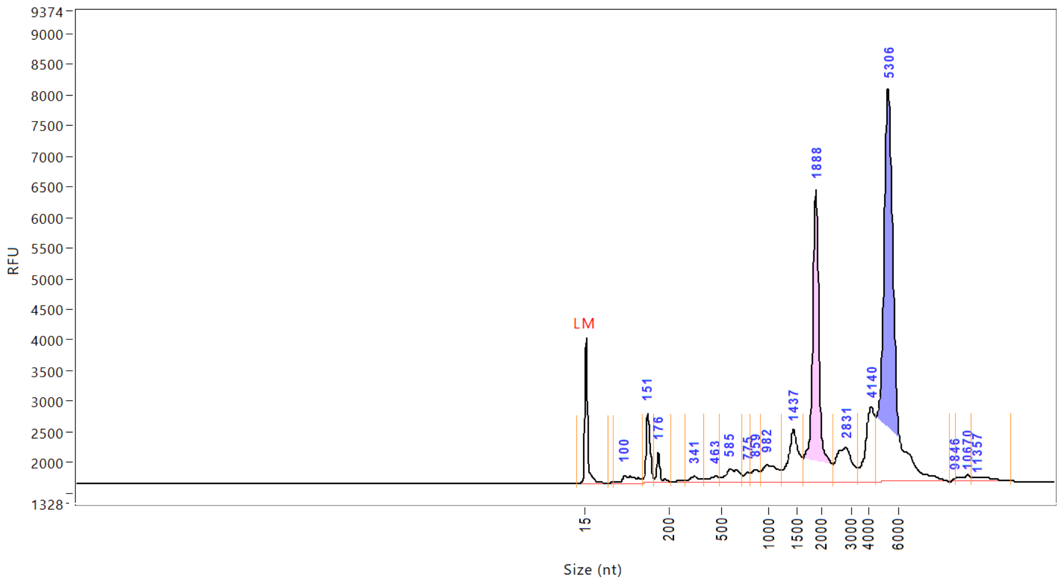

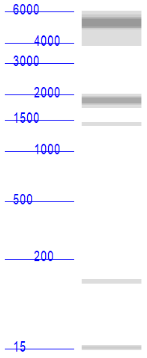

Peak	Size (nt)	Conc. (ng/uL)
1	15 (LM)	0.0310
2	100	0.0629
3	151	0.2018
4	176	0.0715
5	341	0.0368
6	463	0.0339
7	585	0.1082
8	775	0.0324
9	859	0.0550
10	982	0.1476
11	1437	0.2965
12	1888	1.1457
13	2831	0.2783
14	4140	0.3435
15	5306	2.1230
16	9846	0.0021
17	10670	0.0221
18	11357	0.0276

TIC:	4.9888	ng/uL
TIM:	13.681	nmole/L
Total Conc.:	4.9667	ng/uL

28S/18S: 1.6

```
Sample Peak Width (sec): 6    Sample Min Peak Height: 20    Sample Baseline V to V?: Y    Sample Baseline V to V pts: 3
Sample Filter: Binomial       # of Pts for Filter: 9        Sample Start Region (min): 0  Sample End Region (min): 40
Manual Baseline Start (min): 18      Manual Baseline End (min): 38
Marker Peak Width (sec): 6    Marker Min Peak Height: 100   Marker Baseline V to V?: Y    Marker Baseline V to V pts: 3
Lower Marker Selection: First Peak > 100 RFU                Upper Marker Selection: Last Peak > 100 RFU
Ladder Size (bp): 15, 200, 500, 1000, 1500, 2000, 3000, 4000, 6000
Quantification Using: Ladder          Final Concentration (ng/uL): 0.2000          Dilution Factor: 10.0
Min. RFU for Data Processing: 2
```

 Copyright 2015 Advanced Analytical Technologies, Inc.

Data File: 2017 06 29 13H 45M.raw
Sample: SW1710+vector 3_Verd1:10
Well Location: G8

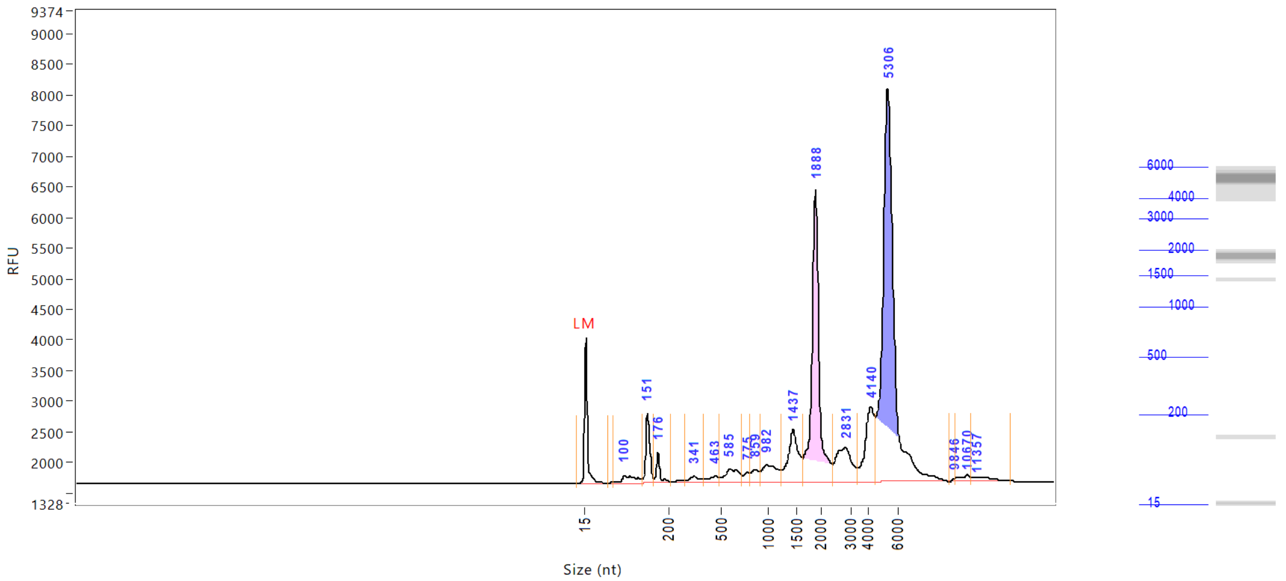

Peak	Size (nt)	Conc. (ng/uL)
	RQN	9.1

```
Sample Peak Width (sec): 6    Sample Min Peak Height: 20    Sample Baseline V to V?: Y    Sample Baseline V to V pts: 3
Sample Filter: Binomial       # of Pts for Filter: 9        Sample Start Region (min): 0  Sample End Region (min): 40
Manual Baseline Start (min): 18     Manual Baseline End (min): 38
Marker Peak Width (sec): 6    Marker Min Peak Height: 100   Marker Baseline V to V?: Y    Marker Baseline V to V pts: 3
Lower Marker Selection: First Peak > 100 RFU                Upper Marker Selection: Last Peak > 100 RFU
Ladder Size (bp): 15, 200, 500, 1000, 1500, 2000, 3000, 4000, 6000
Quantification Using: Ladder          Final Concentration (ng/uL): 0.2000          Dilution Factor: 10.0
Min. RFU for Data Processing: 2
```

 Copyright 2015 Advanced Analytical Technologies, Inc.

Data File: 2017 06 29 13H 45M.raw
Sample: SW1710+vector 4_Verd1:10
Well Location: G9

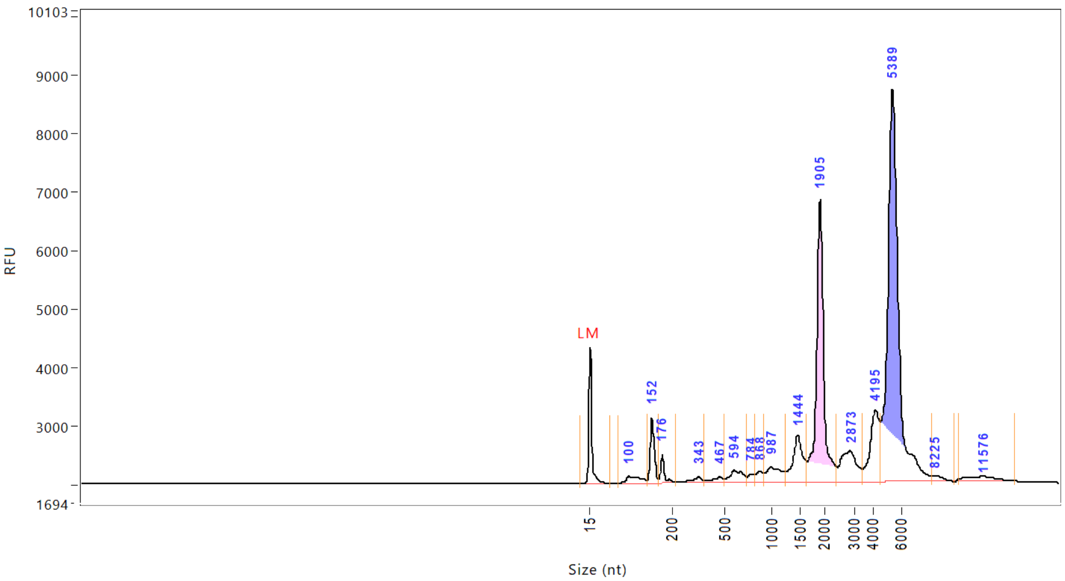

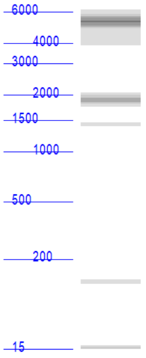

Peak	Size (nt)	Conc. (ng/uL)
1	15 (LM)	0.0310
2	100	0.0634
3	152	0.1998
4	176	0.0678
5	343	0.0348
6	467	0.0340
7	594	0.0943
8	784	0.0309
9	868	0.0451
10	987	0.1283
11	1444	0.2811
12	1905	1.1495
13	2873	0.2626
14	4195	0.3520
15	5389	2.1496
16	8225	0.0224
17	11576	0.0466

TIC:	4.9622	ng/uL
TIM:	13.268	nmole/L
Total Conc.:	4.9334	ng/uL

28S/18S:	1.6
RQN	9.3

```
Sample Peak Width (sec): 6    Sample Min Peak Height: 20    Sample Baseline V to V?: Y    Sample Baseline V to V pts: 3
Sample Filter: Binomial       # of Pts for Filter: 9        Sample Start Region (min): 0  Sample End Region (min): 40
Manual Baseline Start (min): 18     Manual Baseline End (min): 38
Marker Peak Width (sec): 6    Marker Min Peak Height: 100   Marker Baseline V to V?: Y    Marker Baseline V to V pts: 3
Lower Marker Selection: First Peak > 100 RFU                Upper Marker Selection: Last Peak > 100 RFU
Ladder Size (bp): 15, 200, 500, 1000, 1500, 2000, 3000, 4000, 6000
Quantification Using: Ladder          Final Concentration (ng/uL): 0.2000          Dilution Factor: 10.0
Min. RFU for Data Processing: 2
```

 Copyright 2015 Advanced Analytical Technologies, Inc.

Data File: 2017 06 29 13H 45M.raw
Sample: SW1710+HD5 1_Verd1:10
Well Location: G10

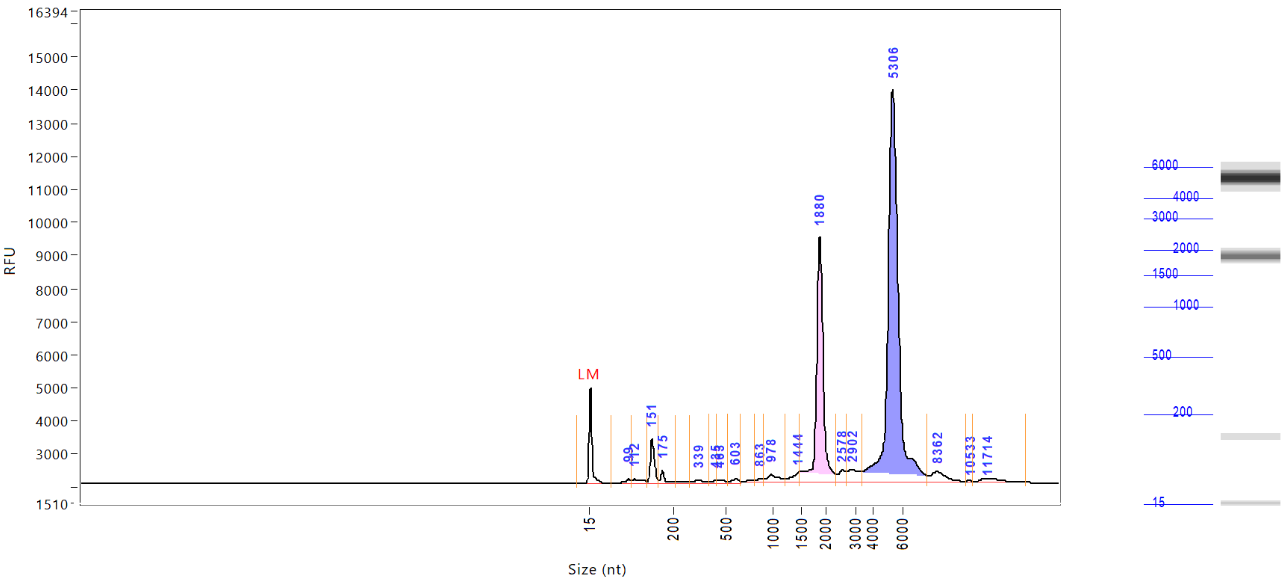

Peak	Size (nt)	Conc. (ng/uL)
1	15 (LM)	0.0310
2	99	0.0206
3	112	0.0376
4	151	0.1925
5	175	0.0441
6	339	0.0238
7	435	0.0092
8	463	0.0162
9	603	0.0251
10	863	0.0180
11	978	0.0766
12	1444	0.0559
13	1880	1.3681
14	2578	0.0669
15	2902	0.1115
16	5306	3.0386
17	8362	0.0980
18	10533	0.0034
19	11714	0.0293

TIC:	5.2355	ng/uL
TIM:	11.701	nmole/L
Total Conc.:	5.2407	ng/uL

Sample Peak Width (sec): 6   Sample Min Peak Height: 20   Sample Baseline V to V?: Y   Sample Baseline V to V pts: 3
Sample Filter: Binomial   # of Pts for Filter: 9   Sample Start Region (min): 0   Sample End Region (min): 40
Manual Baseline Start (min): 18   Manual Baseline End (min): 38
Marker Peak Width (sec): 6   Marker Min Peak Height: 100   Marker Baseline V to V?: Y   Marker Baseline V to V pts: 3
Lower Marker Selection: First Peak > 100 RFU   Upper Marker Selection: Last Peak > 100 RFU
Ladder Size (bp): 15, 200, 500, 1000, 1500, 2000, 3000, 4000, 6000
Quantification Using: Ladder   Final Concentration (ng/uL): 0.2000   Dilution Factor: 10.0
Min. RFU for Data Processing: 2

Copyright 2015 Advanced Analytical Technologies, Inc.

Data File: 2017 06 29 13H 45M.raw
Sample: SW1710+HD5 1_Verd1:10
Well Location: G10

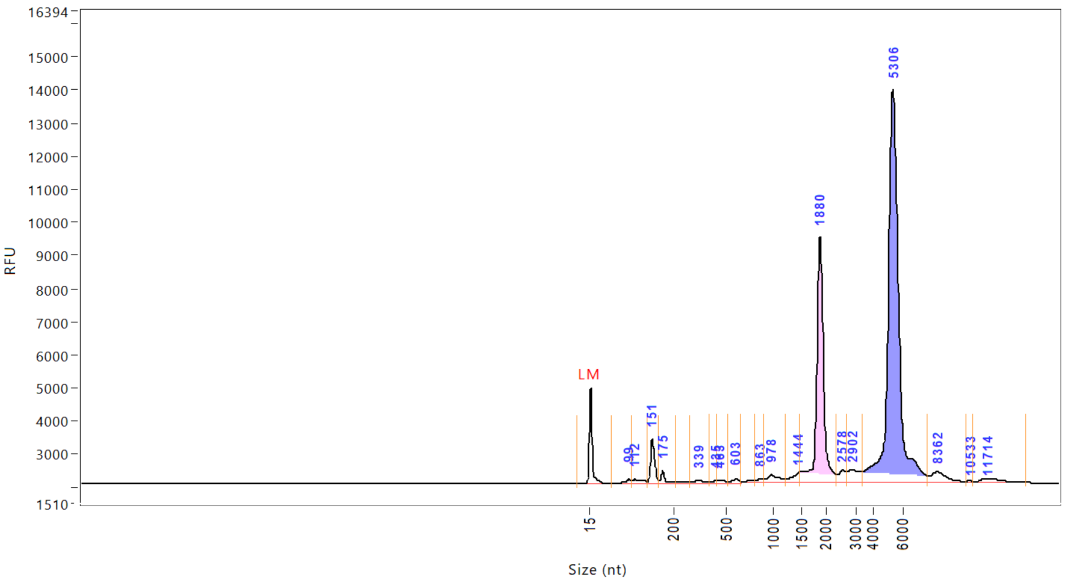

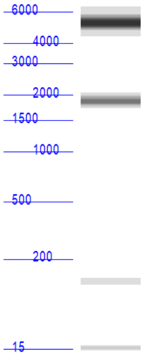

Peak	Size (nt)	Conc. (ng/uL)

28S/18S:	2.4
RQN	10.0

```
Sample Peak Width (sec): 6    Sample Min Peak Height: 20    Sample Baseline V to V?: Y    Sample Baseline V to V pts: 3
Sample Filter: Binomial       # of Pts for Filter: 9        Sample Start Region (min): 0  Sample End Region (min): 40
Manual Baseline Start (min): 18     Manual Baseline End (min): 38
Marker Peak Width (sec): 6    Marker Min Peak Height: 100   Marker Baseline V to V?: Y    Marker Baseline V to V pts: 3
Lower Marker Selection: First Peak > 100 RFU                Upper Marker Selection: Last Peak > 100 RFU
Ladder Size (bp): 15, 200, 500, 1000, 1500, 2000, 3000, 4000, 6000
Quantification Using: Ladder          Final Concentration (ng/uL): 0.2000          Dilution Factor: 10.0
Min. RFU for Data Processing: 2
```

 Copyright 2015 Advanced Analytical Technologies, Inc.

Data File: 2017 06 29 13H 45M.raw
Sample: SW1710+HD5 2_Verd1:10
Well Location: G11

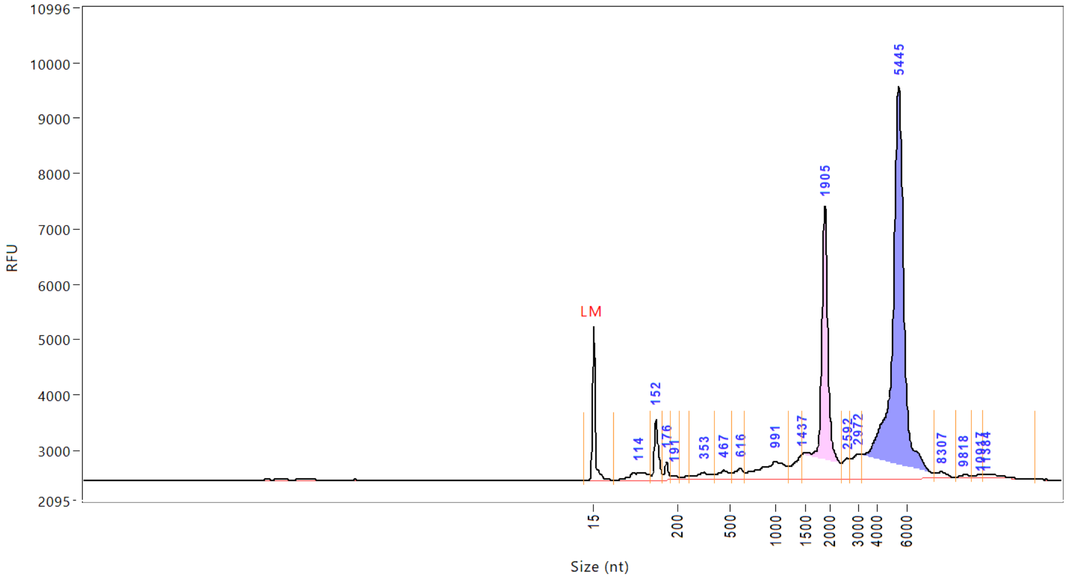

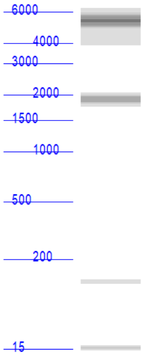

Peak	Size (nt)	Conc. (ng/uL)
1	15 (LM)	0.0310
2	114	0.0807
3	152	0.1721
4	176	0.0394
5	191	0.0161
6	353	0.0593
7	467	0.0554
8	616	0.0523
9	991	0.2392
10	1437	0.1040
11	1905	1.1201
12	2592	0.0722
13	2972	0.1088
14	5445	2.2912
15	8307	0.0275
16	9818	0.0121
17	10917	0.0116
18	11384	0.0204

TIC:	4.4823	ng/uL
TIM:	12.201	nmole/L
Total Conc.:	4.4727	ng/uL

28S/18S: 2.4

```
Sample Peak Width (sec): 6    Sample Min Peak Height: 20    Sample Baseline V to V?: Y    Sample Baseline V to V pts: 3
Sample Filter: Binomial       # of Pts for Filter: 9        Sample Start Region (min): 0  Sample End Region (min): 40
Manual Baseline Start (min): 18     Manual Baseline End (min): 38
Marker Peak Width (sec): 6    Marker Min Peak Height: 100   Marker Baseline V to V?: Y    Marker Baseline V to V pts: 3
Lower Marker Selection: First Peak > 100 RFU                Upper Marker Selection: Last Peak > 100 RFU
Ladder Size (bp): 15, 200, 500, 1000, 1500, 2000, 3000, 4000, 6000
Quantification Using: Ladder          Final Concentration (ng/uL): 0.2000          Dilution Factor: 10.0
Min. RFU for Data Processing: 2
```

 Copyright 2015 Advanced Analytical Technologies, Inc.

Data File: 2017 06 29 13H 45M.raw
Sample: SW1710+HD5 2_Verd1:10
Well Location: G11

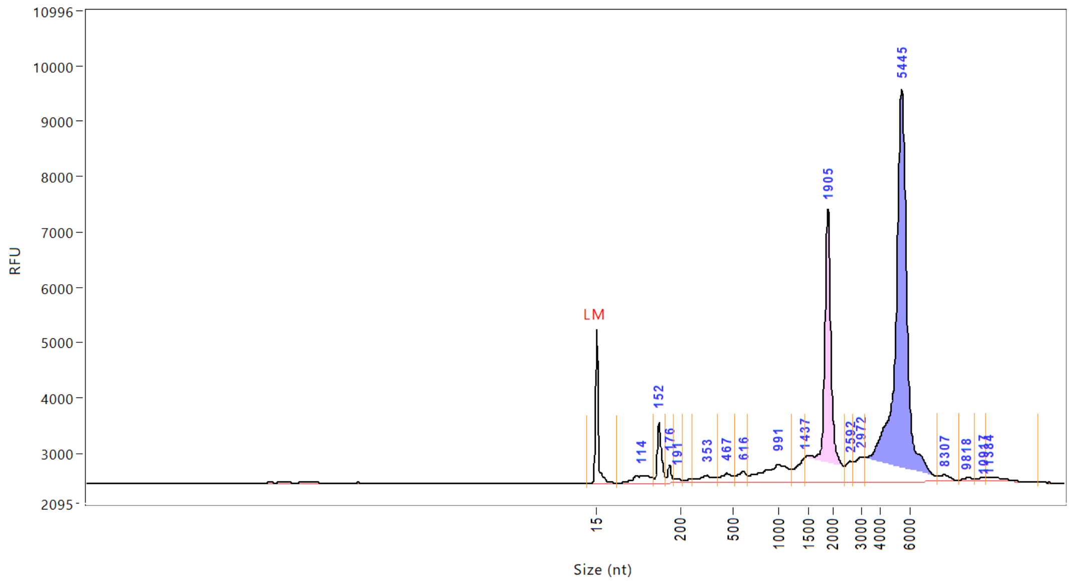

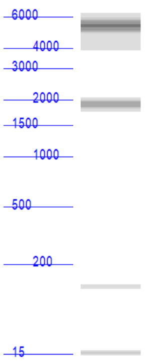

Peak	Size (nt)	Conc. (ng/uL)
	RQN	9.6

Sample Peak Width (sec): 6 Sample Min Peak Height: 20 Sample Baseline V to V?: Y Sample Baseline V to V pts: 3
Sample Filter: Binomial # of Pts for Filter: 9 Sample Start Region (min): 0 Sample End Region (min): 40
Manual Baseline Start (min): 18 Manual Baseline End (min): 38
Marker Peak Width (sec): 6 Marker Min Peak Height: 100 Marker Baseline V to V?: Y Marker Baseline V to V pts: 3
Lower Marker Selection: First Peak > 100 RFU Upper Marker Selection: Last Peak > 100 RFU
Ladder Size (bp): 15, 200, 500, 1000, 1500, 2000, 3000, 4000, 6000
Quantification Using: Ladder Final Concentration (ng/uL): 0.2000 Dilution Factor: 10.0
Min. RFU for Data Processing: 2

 Copyright 2015 Advanced Analytical Technologies, Inc.

Data File: 2017 06 29 14H 48M.raw
Sample: SW1710+HD5 3_Verd1:10
Well Location: H1

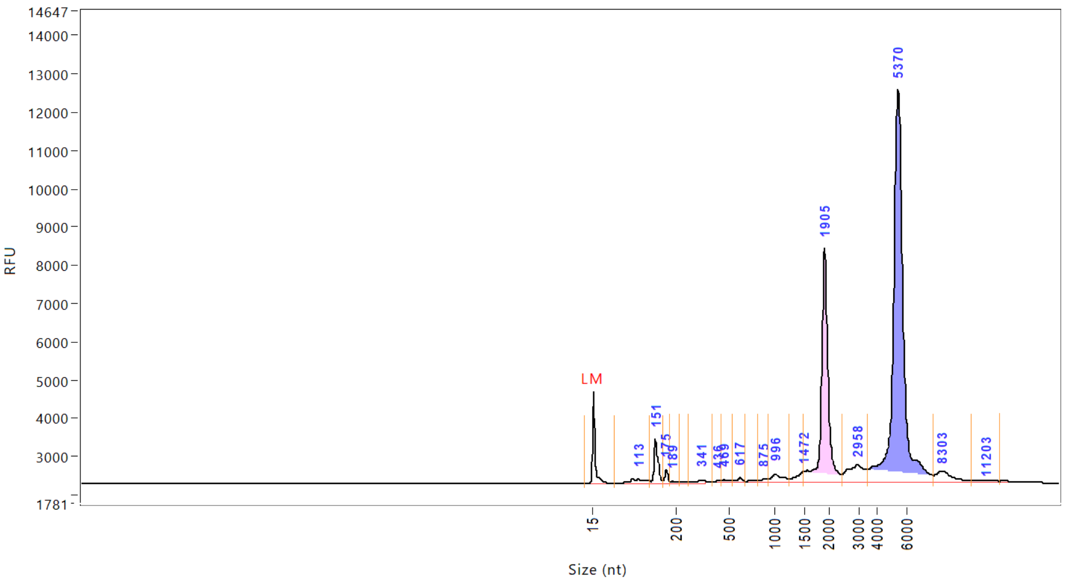

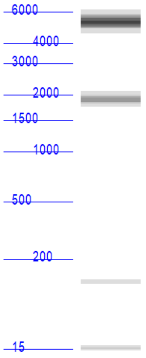

Peak	Size (nt)	Conc. (ng/uL)
1	15 (LM)	0.0135
2	113	0.0256
3	151	0.0860
4	175	0.0178
5	189	0.0031
6	341	0.0111
7	436	0.0042
8	469	0.0085
9	617	0.0117
10	875	0.0097
11	996	0.0374
12	1472	0.0288
13	1905	0.6084
14	2958	0.1080
15	5370	1.3275
16	8303	0.0494
17	11203	0.0054

TIC:	2.3425	ng/uL
TIM:	5.198	nmole/L
Total Conc.:	2.3450	ng/uL

28S/18S:	2.3
RQN	10.0

```
Sample Peak Width (sec): 6    Sample Min Peak Height: 20    Sample Baseline V to V?: Y    Sample Baseline V to V pts: 3
Sample Filter: Binomial       # of Pts for Filter: 9        Sample Start Region (min): 0  Sample End Region (min): 40
Manual Baseline Start (min): 18     Manual Baseline End (min): 38
Marker Peak Width (sec): 6    Marker Min Peak Height: 100   Marker Baseline V to V?: Y    Marker Baseline V to V pts: 3
Lower Marker Selection: First Peak > 100 RFU                Upper Marker Selection: Last Peak > 100 RFU
Ladder Size (bp): 15, 200, 500, 1000, 1500, 2000, 3000, 4000, 6000
Quantification Using: Ladder          Final Concentration (ng/uL): 0.2000          Dilution Factor: 10.0
Min. RFU for Data Processing: 2
```

 Copyright 2015 Advanced Analytical Technologies, Inc.

Data File: 2017 06 29 14H 48M.raw
Sample: SW1710+HD5 4_Verd1:10
Well Location: H2

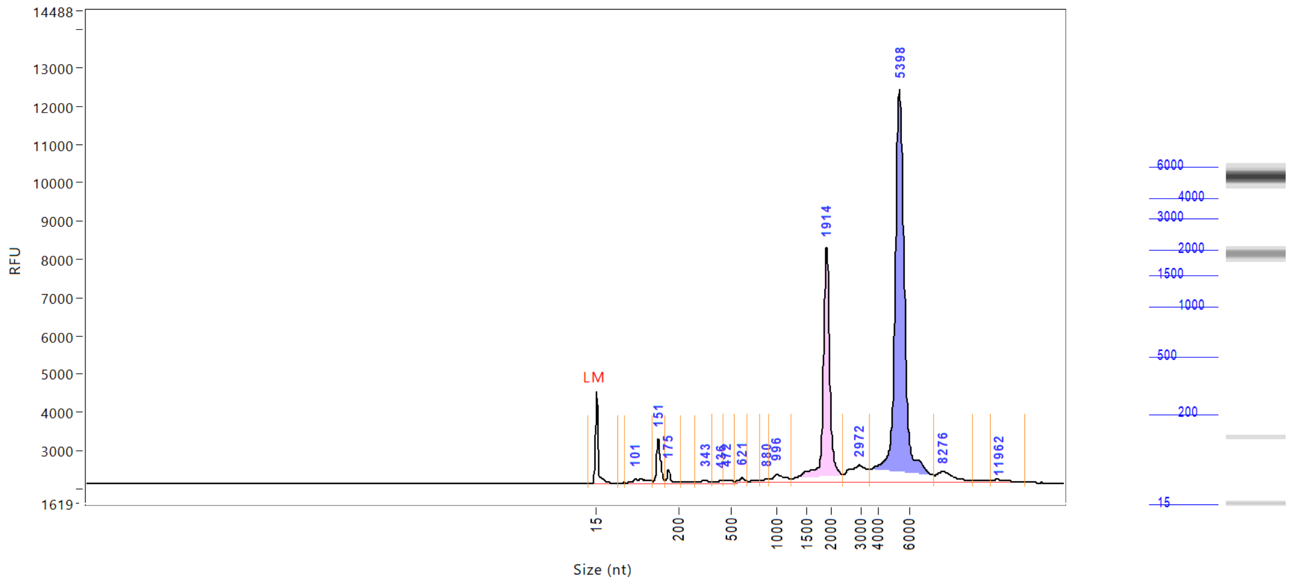

Peak	Size (nt)	Conc. (ng/uL)
1	15 (LM)	0.0135
2	101	0.0278
3	151	0.0858
4	175	0.0208
5	343	0.0098
6	436	0.0057
7	472	0.0078
8	621	0.0123
9	880	0.0088
10	996	0.0391
11	1914	0.6335
12	2972	0.1081
13	5398	1.3263
14	8276	0.0492
15	11962	0.0062

TIC:	2.3412	ng/uL
TIM:	5.320	nmole/L
Total Conc.:	2.3522	ng/uL

28S/18S:	2.1
RQN	10.0

Sample Peak Width (sec): 6   Sample Min Peak Height: 20   Sample Baseline V to V?: Y   Sample Baseline V to V pts: 3
Sample Filter: Binomial   # of Pts for Filter: 9   Sample Start Region (min): 0   Sample End Region (min): 40
Manual Baseline Start (min): 18   Manual Baseline End (min): 38
Marker Peak Width (sec): 6   Marker Min Peak Height: 100   Marker Baseline V to V?: Y   Marker Baseline V to V pts: 3
Lower Marker Selection: First Peak > 100 RFU   Upper Marker Selection: Last Peak > 100 RFU
Ladder Size (bp): 15, 200, 500, 1000, 1500, 2000, 3000, 4000, 6000
Quantification Using: Ladder   Final Concentration (ng/uL): 0.2000   Dilution Factor: 10.0
Min. RFU for Data Processing: 2

Copyright 2015 Advanced Analytical Technologies, Inc.

Data File: 2017 06 30 08H 21M.raw
Sample: UM-UC-3+vector 1_Verd1:10_w1
Well Location: G1

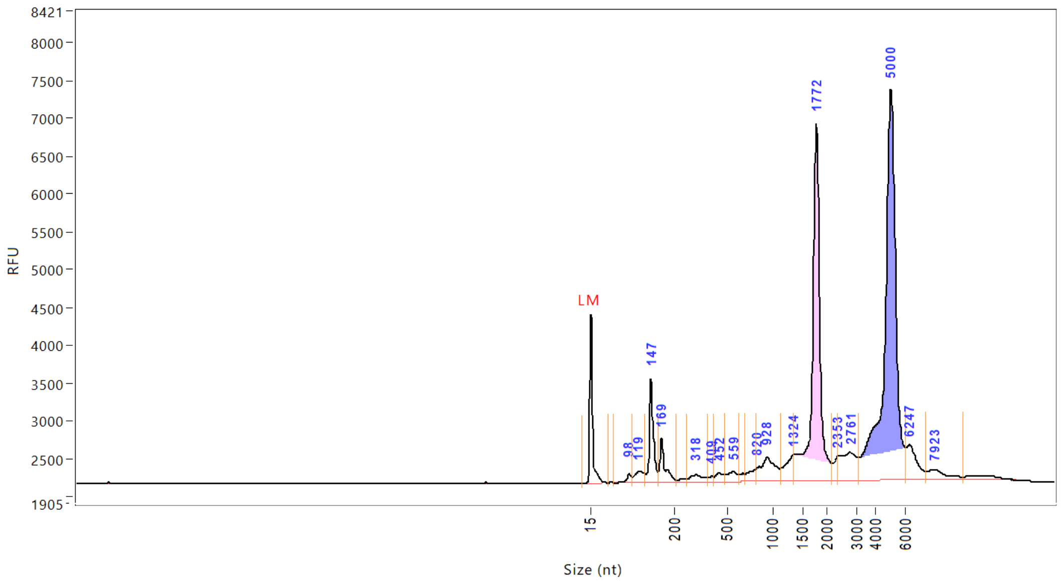

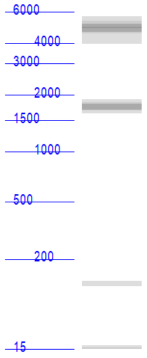

Peak	Size (nt)	Conc. (ng/uL)
1	15 (LM)	0.0219
2	98	0.0158
3	119	0.0448
4	147	0.1862
5	169	0.0896
6	318	0.0330
7	409	0.0102
8	452	0.0249
9	559	0.0330
10	820	0.0371
11	928	0.1184
12	1324	0.0747
13	1772	0.9181
14	2353	0.0337
15	2761	0.1467
16	5000	1.3428
17	6247	0.1141
18	7923	0.0437

TIC:	3.2668	ng/uL
TIM:	11.474	nmole/L
Total Conc.:	3.2819	ng/uL

28S/18S: 1.5

```
Sample Peak Width (sec): 6    Sample Min Peak Height: 20    Sample Baseline V to V?: Y    Sample Baseline V to V pts: 3
Sample Filter: Binomial       # of Pts for Filter: 9        Sample Start Region (min): 0  Sample End Region (min): 40
Manual Baseline Start (min): 18     Manual Baseline End (min): 38
Marker Peak Width (sec): 6    Marker Min Peak Height: 100   Marker Baseline V to V?: Y    Marker Baseline V to V pts: 3
Lower Marker Selection: First Peak > 100 RFU                Upper Marker Selection: Last Peak > 100 RFU
Ladder Size (bp): 15, 200, 500, 1000, 1500, 2000, 3000, 4000, 6000
Quantification Using: Ladder          Final Concentration (ng/uL): 0.2000          Dilution Factor: 10.0
Min. RFU for Data Processing: 2
```

 Copyright 2015 Advanced Analytical Technologies, Inc.

Data File: 2017 06 30 08H 21M.raw
Sample: UM-UC-3+vector 1_Verd1:10_w1
Well Location: G1

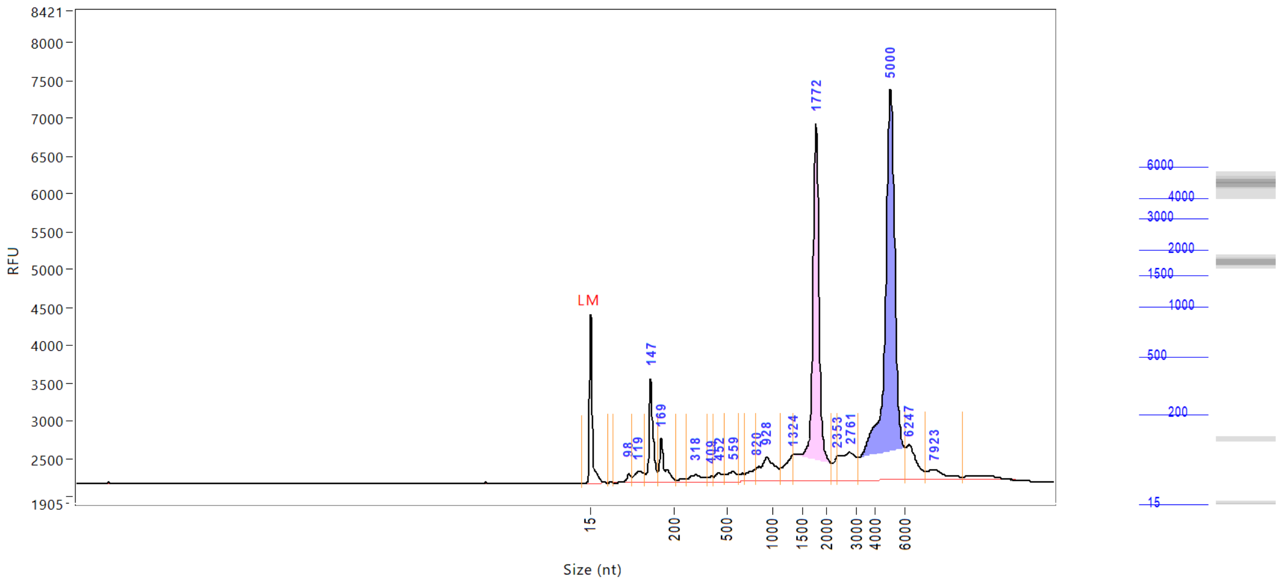

Peak	Size (nt)	Conc. (ng/uL)
	RQN	9.3

Sample Peak Width (sec): 6   Sample Min Peak Height: 20   Sample Baseline V to V?: Y   Sample Baseline V to V pts: 3
Sample Filter: Binomial   # of Pts for Filter: 9   Sample Start Region (min): 0   Sample End Region (min): 40
Manual Baseline Start (min): 18   Manual Baseline End (min): 38
Marker Peak Width (sec): 6   Marker Min Peak Height: 100   Marker Baseline V to V?: Y   Marker Baseline V to V pts: 3
Lower Marker Selection: First Peak > 100 RFU   Upper Marker Selection: Last Peak > 100 RFU
Ladder Size (bp): 15, 200, 500, 1000, 1500, 2000, 3000, 4000, 6000
Quantification Using: Ladder   Final Concentration (ng/uL): 0.2000   Dilution Factor: 10.0
Min. RFU for Data Processing: 2

 Copyright 2015 Advanced Analytical Technologies, Inc.

Data File: 2017 06 29 14H 48M.raw
Sample: UM-UC-3+vector 2_Verd1:10
Well Location: H4

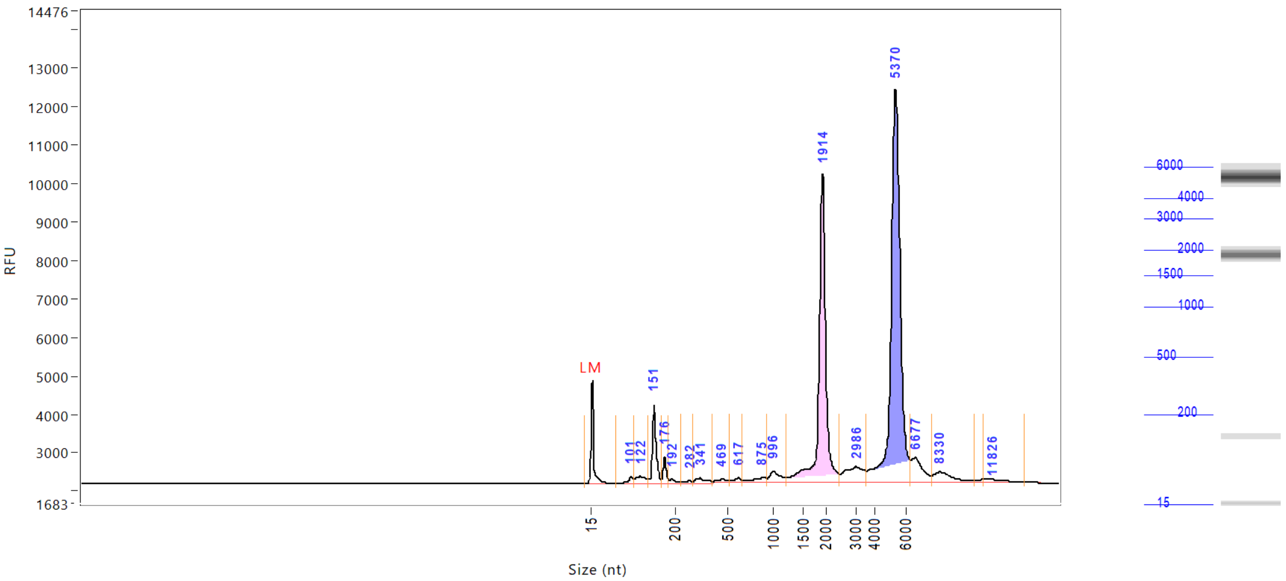

Peak	Size (nt)	Conc. (ng/uL)
1	15 (LM)	0.0135
2	101	0.0115
3	122	0.0274
4	151	0.1286
5	176	0.0311
6	192	0.0078
7	282	0.0046
8	341	0.0165
9	469	0.0135
10	617	0.0116
11	875	0.0216
12	996	0.0416
13	1914	0.7158
14	2986	0.0860
15	5370	1.0706
16	6677	0.0787
17	8330	0.0432
18	11826	0.0082

TIC:	2.3183	ng/uL
TIM:	6.862	nmole/L
Total Conc.:	2.3118	ng/uL

Sample Peak Width (sec): 6 Sample Min Peak Height: 20 Sample Baseline V to V?: Y Sample Baseline V to V pts: 3
Sample Filter: Binomial # of Pts for Filter: 9 Sample Start Region (min): 0 Sample End Region (min): 40
Manual Baseline Start (min): 18 Manual Baseline End (min): 38
Marker Peak Width (sec): 6 Marker Min Peak Height: 100 Marker Baseline V to V?: Y Marker Baseline V to V pts: 3
Lower Marker Selection: First Peak > 100 RFU Upper Marker Selection: Last Peak > 100 RFU
Ladder Size (bp): 15, 200, 500, 1000, 1500, 2000, 3000, 4000, 6000
Quantification Using: Ladder Final Concentration (ng/uL): 0.2000 Dilution Factor: 10.0
Min. RFU for Data Processing: 2

 Copyright 2015 Advanced Analytical Technologies, Inc.

Data File: 2017 06 29 14H 48M.raw
Sample: UM-UC-3+vector 2_Verd1:10
Well Location: H4

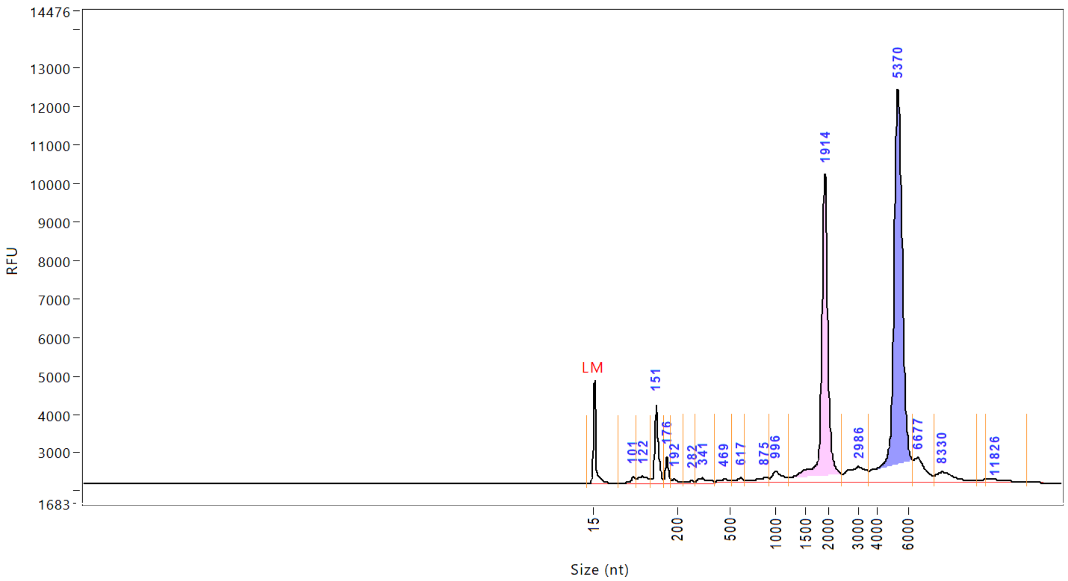

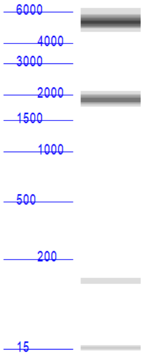

Peak	Size (nt)	Conc. (ng/uL)

28S/18S:	1.4
RQN	10.0

```
Sample Peak Width (sec): 6    Sample Min Peak Height: 20    Sample Baseline V to V?: Y    Sample Baseline V to V pts: 3
Sample Filter: Binomial       # of Pts for Filter: 9        Sample Start Region (min): 0  Sample End Region (min): 40
Manual Baseline Start (min): 18     Manual Baseline End (min): 38
Marker Peak Width (sec): 6    Marker Min Peak Height: 100   Marker Baseline V to V?: Y    Marker Baseline V to V pts: 3
Lower Marker Selection: First Peak > 100 RFU                Upper Marker Selection: Last Peak > 100 RFU
Ladder Size (bp): 15, 200, 500, 1000, 1500, 2000, 3000, 4000, 6000
Quantification Using: Ladder          Final Concentration (ng/uL): 0.2000          Dilution Factor: 10.0
Min. RFU for Data Processing: 2
```

 Copyright 2015 Advanced Analytical Technologies, Inc.

Data File: 2017 06 29 14H 48M.raw
Sample: UM-UC-3+vector 3_Verd1:10
Well Location: H5

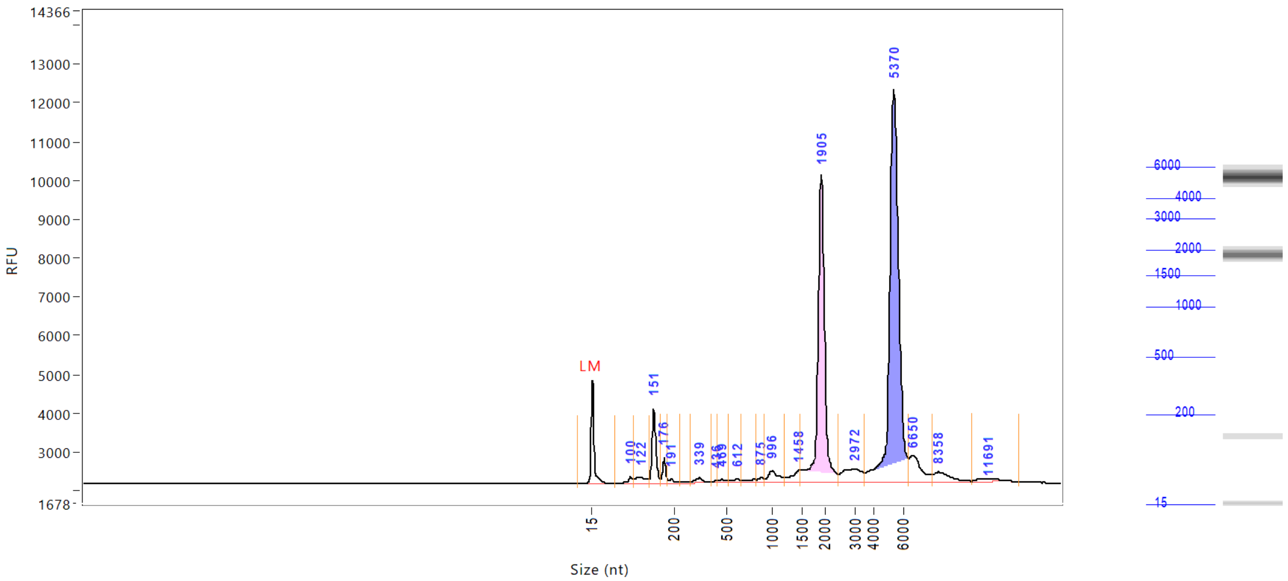

Peak	Size (nt)	Conc. (ng/uL)
1	15 (LM)	0.0135
2	100	0.0108
3	122	0.0247
4	151	0.1222
5	176	0.0307
6	191	0.0061
7	339	0.0144
8	436	0.0034
9	469	0.0077
10	612	0.0098
11	875	0.0101
12	996	0.0402
13	1458	0.0304
14	1905	0.6896
15	2972	0.0774
16	5370	1.0778
17	6650	0.0894
18	8358	0.0382
19	11691	0.0131

TIC:	2.2959	ng/uL
TIM:	6.502	nmole/L
Total Conc.:	2.2972	ng/uL

Sample Peak Width (sec): 6   Sample Min Peak Height: 20   Sample Baseline V to V?: Y   Sample Baseline V to V pts: 3
Sample Filter: Binomial   # of Pts for Filter: 9   Sample Start Region (min): 0   Sample End Region (min): 40
Manual Baseline Start (min): 18   Manual Baseline End (min): 38
Marker Peak Width (sec): 6   Marker Min Peak Height: 100   Marker Baseline V to V?: Y   Marker Baseline V to V pts: 3
Lower Marker Selection: First Peak > 100 RFU   Upper Marker Selection: Last Peak > 100 RFU
Ladder Size (bp): 15, 200, 500, 1000, 1500, 2000, 3000, 4000, 6000
Quantification Using: Ladder   Final Concentration (ng/uL): 0.2000   Dilution Factor: 10.0
Min. RFU for Data Processing: 2

 Copyright 2015 Advanced Analytical Technologies, Inc.

Data File: 2017 06 29 14H 48M.raw
Sample: UM-UC-3+vector 3_Verd1:10
Well Location: H5

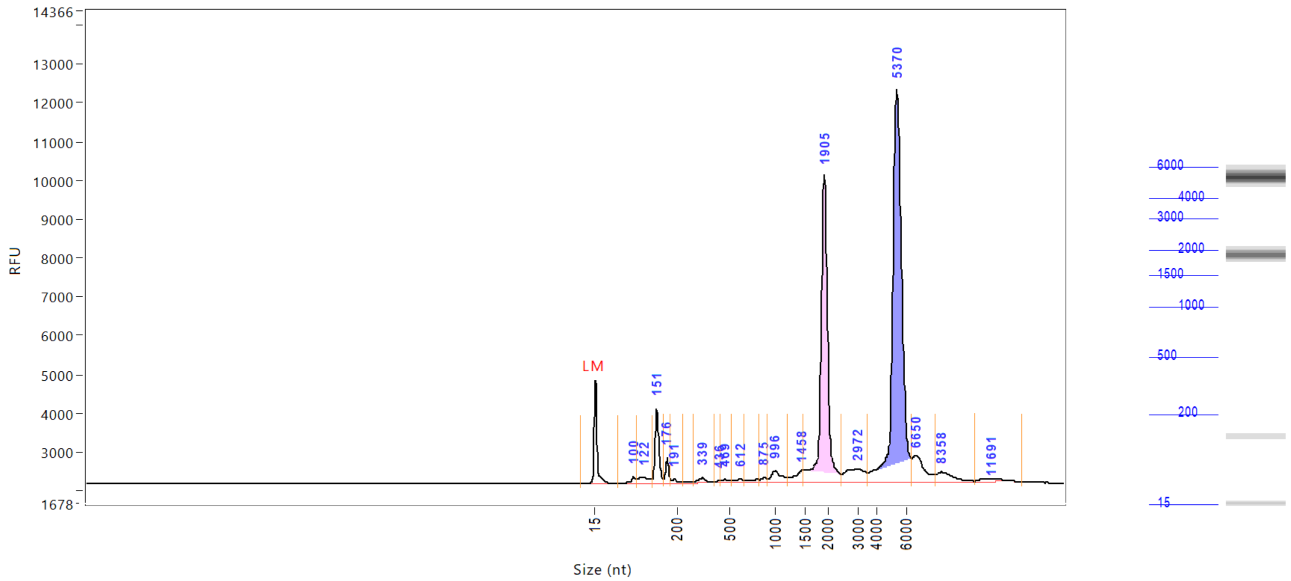

Peak	Size (nt)	Conc. (ng/uL)
	28S/18S:	1.5
	RQN	10.0

```
Sample Peak Width (sec): 6    Sample Min Peak Height: 20    Sample Baseline V to V?: Y    Sample Baseline V to V pts: 3
Sample Filter: Binomial       # of Pts for Filter: 9        Sample Start Region (min): 0  Sample End Region (min): 40
Manual Baseline Start (min): 18     Manual Baseline End (min): 38
Marker Peak Width (sec): 6    Marker Min Peak Height: 100   Marker Baseline V to V?: Y    Marker Baseline V to V pts: 3
Lower Marker Selection: First Peak > 100 RFU                Upper Marker Selection: Last Peak > 100 RFU
Ladder Size (bp): 15, 200, 500, 1000, 1500, 2000, 3000, 4000, 6000
Quantification Using: Ladder          Final Concentration (ng/uL): 0.2000          Dilution Factor: 10.0
Min. RFU for Data Processing: 2
```

 Copyright 2015 Advanced Analytical Technologies, Inc.

Data File: 2017 06 29 14H 48M.raw
Sample: UM-UC-3+vector 4_Verd1:10
Well Location: H6

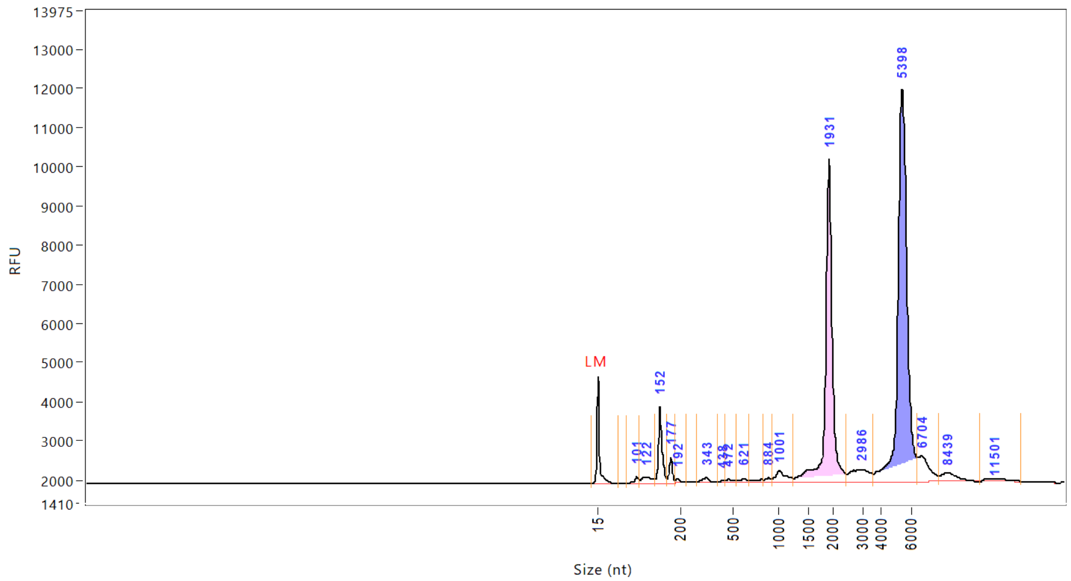

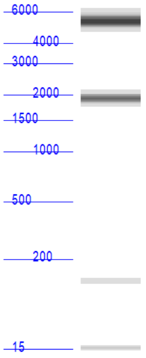

Peak	Size (nt)	Conc. (ng/uL)
1	15 (LM)	0.0135
2	101	0.0102
3	122	0.0231
4	152	0.1218
5	177	0.0301
6	192	0.0053
7	343	0.0135
8	438	0.0030
9	472	0.0072
10	621	0.0085
11	884	0.0085
12	1001	0.0392
13	1931	0.7244
14	2986	0.0689
15	5398	1.0545
16	6704	0.0814
17	8439	0.0324
18	11501	0.0090

TIC:	2.2411	ng/uL
TIM:	6.297	nmole/L
Total Conc.:	2.2424	ng/uL

28S/18S: 1.4

```
Sample Peak Width (sec): 6    Sample Min Peak Height: 20    Sample Baseline V to V?: Y    Sample Baseline V to V pts: 3
Sample Filter: Binomial       # of Pts for Filter: 9        Sample Start Region (min): 0  Sample End Region (min): 40
Manual Baseline Start (min): 18     Manual Baseline End (min): 38
Marker Peak Width (sec): 6    Marker Min Peak Height: 100   Marker Baseline V to V?: Y    Marker Baseline V to V pts: 3
Lower Marker Selection: First Peak > 100 RFU                Upper Marker Selection: Last Peak > 100 RFU
Ladder Size (bp): 15, 200, 500, 1000, 1500, 2000, 3000, 4000, 6000
Quantification Using: Ladder          Final Concentration (ng/uL): 0.2000          Dilution Factor: 10.0
Min. RFU for Data Processing: 2
```

 Copyright 2015 Advanced Analytical Technologies, Inc.

Data File: 2017 06 29 14H 48M.raw
Sample: UM-UC-3+vector 4_Verd1:10
Well Location: H6

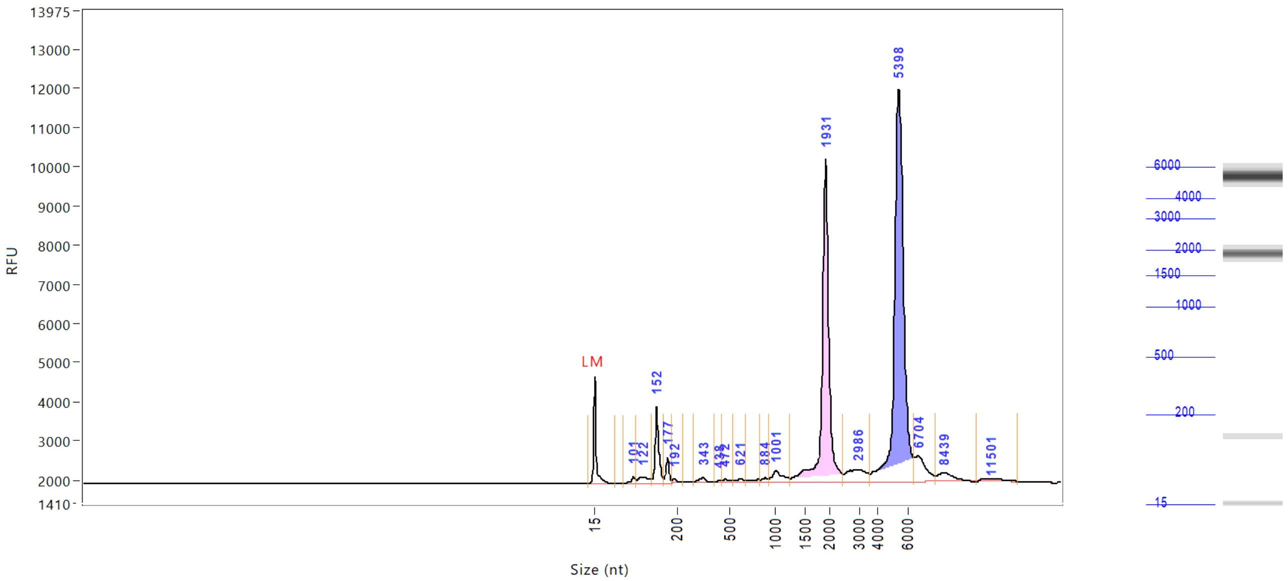

Peak	Size (nt)	Conc. (ng/uL)
	RQN	10.0

Sample Peak Width (sec): 6 Sample Min Peak Height: 20 Sample Baseline V to V?: Y Sample Baseline V to V pts: 3
Sample Filter: Binomial # of Pts for Filter: 9 Sample Start Region (min): 0 Sample End Region (min): 40
Manual Baseline Start (min): 18 Manual Baseline End (min): 38
Marker Peak Width (sec): 6 Marker Min Peak Height: 100 Marker Baseline V to V?: Y Marker Baseline V to V pts: 3
Lower Marker Selection: First Peak > 100 RFU Upper Marker Selection: Last Peak > 100 RFU
Ladder Size (bp): 15, 200, 500, 1000, 1500, 2000, 3000, 4000, 6000
Quantification Using: Ladder Final Concentration (ng/uL): 0.2000 Dilution Factor: 10.0
Min. RFU for Data Processing: 2

 Copyright 2015 Advanced Analytical Technologies, Inc.

Data File: 2017 06 29 14H 48M.raw
Sample: UM-UC-3+HD5 1_Verd1:10
Well Location: H7

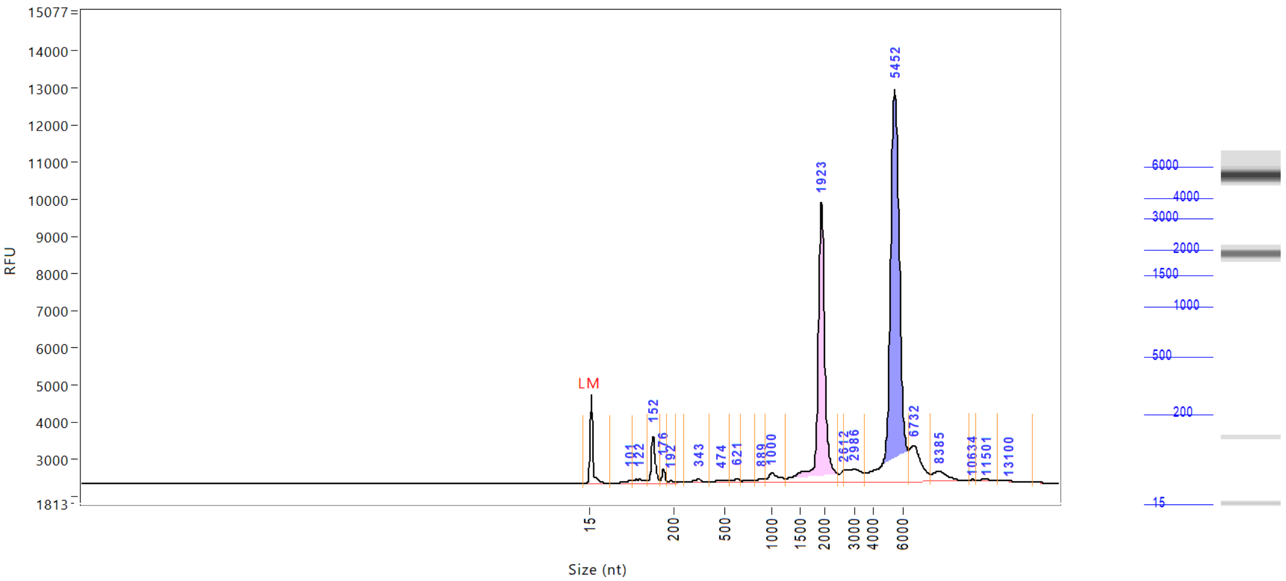

Peak	Size (nt)	Conc. (ng/uL)
1	15 (LM)	0.0135
2	101	0.0080
3	122	0.0179
4	152	0.0961
5	176	0.0198
6	192	0.0042
7	343	0.0145
8	474	0.0100
9	621	0.0100
10	889	0.0097
11	1000	0.0387
12	1923	0.7719
13	2612	0.0160
14	2986	0.0737
15	5452	1.3024
16	6732	0.1440
17	8385	0.0449
18	10634	0.0016
19	11501	0.0046
20	13100	0.0024

TIC:	2.5904	ng/uL
TIM:	5.663	nmole/L
Total Conc.:	2.5885	ng/uL

Sample Peak Width (sec): 6   Sample Min Peak Height: 20   Sample Baseline V to V?: Y   Sample Baseline V to V pts: 3
Sample Filter: Binomial   # of Pts for Filter: 9   Sample Start Region (min): 0   Sample End Region (min): 40
Manual Baseline Start (min): 18   Manual Baseline End (min): 38
Marker Peak Width (sec): 6   Marker Min Peak Height: 100   Marker Baseline V to V?: Y   Marker Baseline V to V pts: 3
Lower Marker Selection: First Peak > 100 RFU   Upper Marker Selection: Last Peak > 100 RFU
Ladder Size (bp): 15, 200, 500, 1000, 1500, 2000, 3000, 4000, 6000
Quantification Using: Ladder   Final Concentration (ng/uL): 0.2000   Dilution Factor: 10.0
Min. RFU for Data Processing: 2

 Copyright 2015 Advanced Analytical Technologies, Inc.

Data File: 2017 06 29 14H 48M.raw
Sample: UM-UC-3+HD5 1_Verd1:10
Well Location: H7

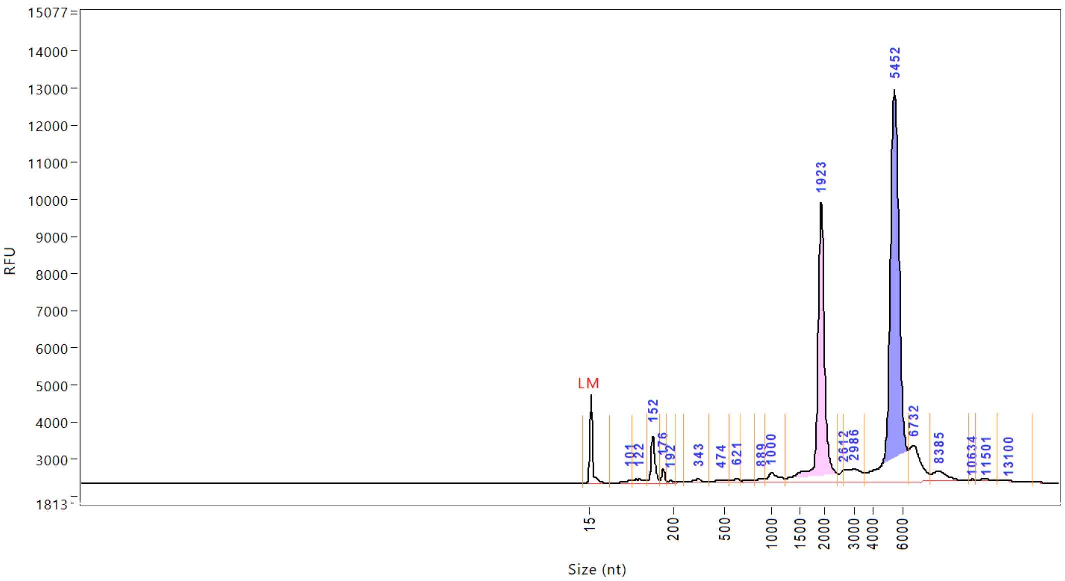

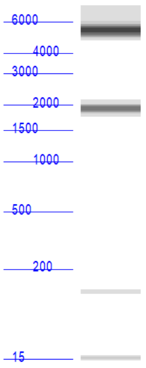

Peak	Size (nt)	Conc. (ng/uL)

28S/18S:	1.6
RQN	10.0

```
Sample Peak Width (sec): 6    Sample Min Peak Height: 20    Sample Baseline V to V?: Y    Sample Baseline V to V pts: 3
Sample Filter: Binomial       # of Pts for Filter: 9        Sample Start Region (min): 0  Sample End Region (min): 40
Manual Baseline Start (min): 18     Manual Baseline End (min): 38
Marker Peak Width (sec): 6    Marker Min Peak Height: 100   Marker Baseline V to V?: Y    Marker Baseline V to V pts: 3
Lower Marker Selection: First Peak > 100 RFU                Upper Marker Selection: Last Peak > 100 RFU
Ladder Size (bp): 15, 200, 500, 1000, 1500, 2000, 3000, 4000, 6000
Quantification Using: Ladder          Final Concentration (ng/uL): 0.2000          Dilution Factor: 10.0
Min. RFU for Data Processing: 2
```

 Copyright 2015 Advanced Analytical Technologies, Inc.

Data File: 2017 06 29 14H 48M.raw
Sample: UM-UC-3+HD5 2_Verd1:10
Well Location: H8

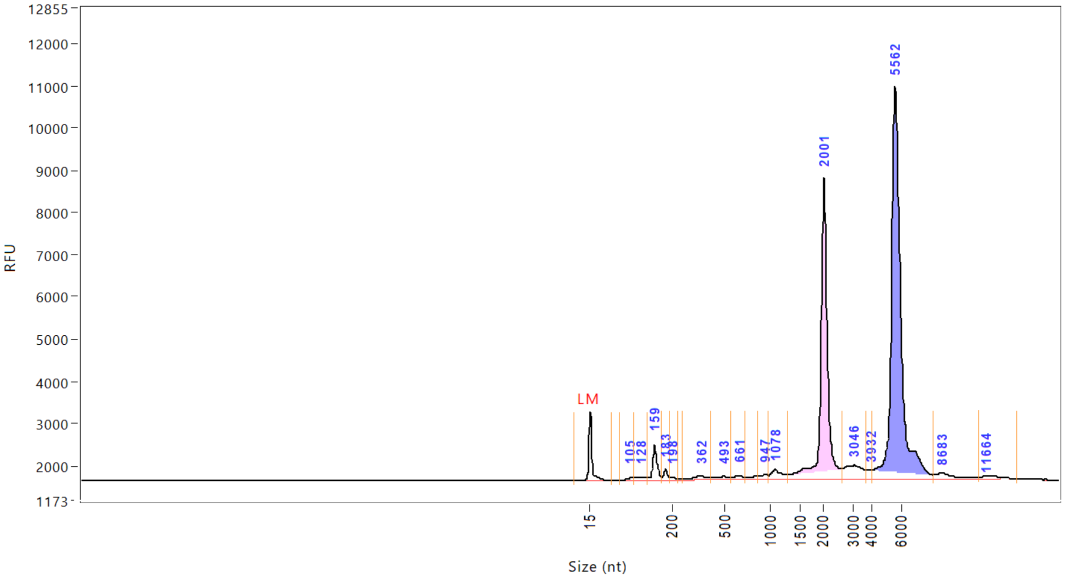

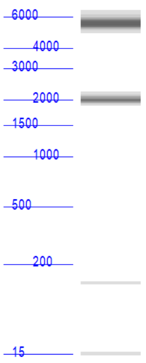

Peak	Size (nt)	Conc. (ng/uL)
1	15 (LM)	0.0135
2	105	0.0081
3	128	0.0175
4	159	0.0966
5	183	0.0217
6	198	0.0087
7	362	0.0275
8	493	0.0199
9	661	0.0171
10	947	0.0170
11	1078	0.0508
12	2001	0.9862
13	3046	0.1098
14	3932	0.0190
15	5562	1.5674
16	8683	0.0326
17	11664	0.0130

TIC:	3.0130	ng/uL
TIM:	6.272	nmole/L
Total Conc.:	3.0203	ng/uL

28S/18S:	1.7
RQN	10.0

```
Sample Peak Width (sec): 6    Sample Min Peak Height: 20    Sample Baseline V to V?: Y    Sample Baseline V to V pts: 3
Sample Filter: Binomial       # of Pts for Filter: 9        Sample Start Region (min): 0  Sample End Region (min): 40
Manual Baseline Start (min): 18     Manual Baseline End (min): 38
Marker Peak Width (sec): 6    Marker Min Peak Height: 100   Marker Baseline V to V?: Y    Marker Baseline V to V pts: 3
Lower Marker Selection: First Peak > 100 RFU                Upper Marker Selection: Last Peak > 100 RFU
Ladder Size (bp): 15, 200, 500, 1000, 1500, 2000, 3000, 4000, 6000
Quantification Using: Ladder          Final Concentration (ng/uL): 0.2000          Dilution Factor: 10.0
Min. RFU for Data Processing: 2
```

 Copyright 2015 Advanced Analytical Technologies, Inc.

Data File: 2017 06 29 14H 48M.raw
Sample: UM-UC-3+HD5 3_Verd1:10
Well Location: H9

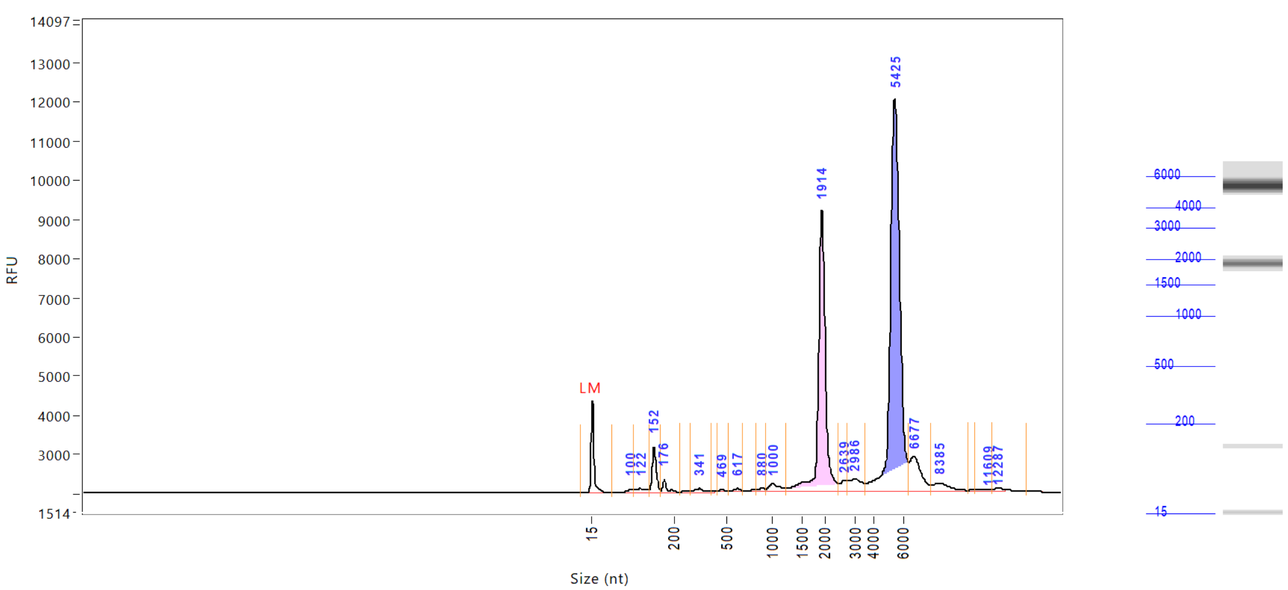

Peak	Size (nt)	Conc. (ng/uL)
1	15 (LM)	0.0135
2	100	0.0070
3	122	0.0170
4	152	0.0894
5	176	0.0211
6	341	0.0113
7	469	0.0057
8	617	0.0090
9	880	0.0077
10	1000	0.0321
11	1914	0.7410
12	2639	0.0250
13	2986	0.0557
14	5425	1.2559
15	6677	0.1326
16	8385	0.0358
17	11609	0.0034
18	12287	0.0069

TIC:	2.4567	ng/uL
TIM:	5.264	nmole/L
Total Conc.:	2.4571	ng/uL

28S/18S: 1.6

Sample Peak Width (sec): 6   Sample Min Peak Height: 20   Sample Baseline V to V?: Y   Sample Baseline V to V pts: 3
Sample Filter: Binomial   # of Pts for Filter: 9   Sample Start Region (min): 0   Sample End Region (min): 40
Manual Baseline Start (min): 18   Manual Baseline End (min): 38
Marker Peak Width (sec): 6   Marker Min Peak Height: 100   Marker Baseline V to V?: Y   Marker Baseline V to V pts: 3
Lower Marker Selection: First Peak > 100 RFU   Upper Marker Selection: Last Peak > 100 RFU
Ladder Size (bp): 15, 200, 500, 1000, 1500, 2000, 3000, 4000, 6000
Quantification Using: Ladder   Final Concentration (ng/uL): 0.2000   Dilution Factor: 10.0
Min. RFU for Data Processing: 2

 Copyright 2015 Advanced Analytical Technologies, Inc.

Data File: 2017 06 29 14H 48M.raw
Sample: UM-UC-3+HD5 3_Verd1:10
Well Location: H9

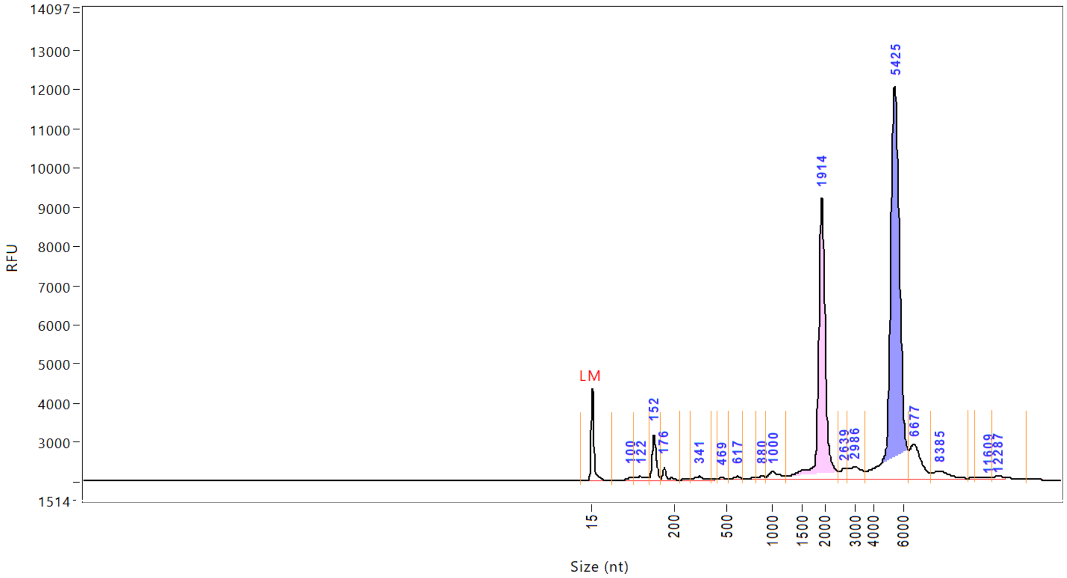

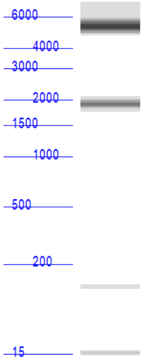

Peak	Size (nt)	Conc. (ng/uL)
	RQN	10.0

```
Sample Peak Width (sec): 6    Sample Min Peak Height: 20    Sample Baseline V to V?: Y    Sample Baseline V to V pts: 3
Sample Filter: Binomial       # of Pts for Filter: 9        Sample Start Region (min): 0  Sample End Region (min): 40
Manual Baseline Start (min): 18     Manual Baseline End (min): 38
Marker Peak Width (sec): 6    Marker Min Peak Height: 100   Marker Baseline V to V?: Y    Marker Baseline V to V pts: 3
Lower Marker Selection: First Peak > 100 RFU                Upper Marker Selection: Last Peak > 100 RFU
Ladder Size (bp): 15, 200, 500, 1000, 1500, 2000, 3000, 4000, 6000
Quantification Using: Ladder          Final Concentration (ng/uL): 0.2000          Dilution Factor: 10.0
Min. RFU for Data Processing: 2
```

 Copyright 2015 Advanced Analytical Technologies, Inc.

Data File: 2017 06 29 14H 48M.raw
Sample: UM-UC-3+HD5 4_Verd1:10
Well Location: H10

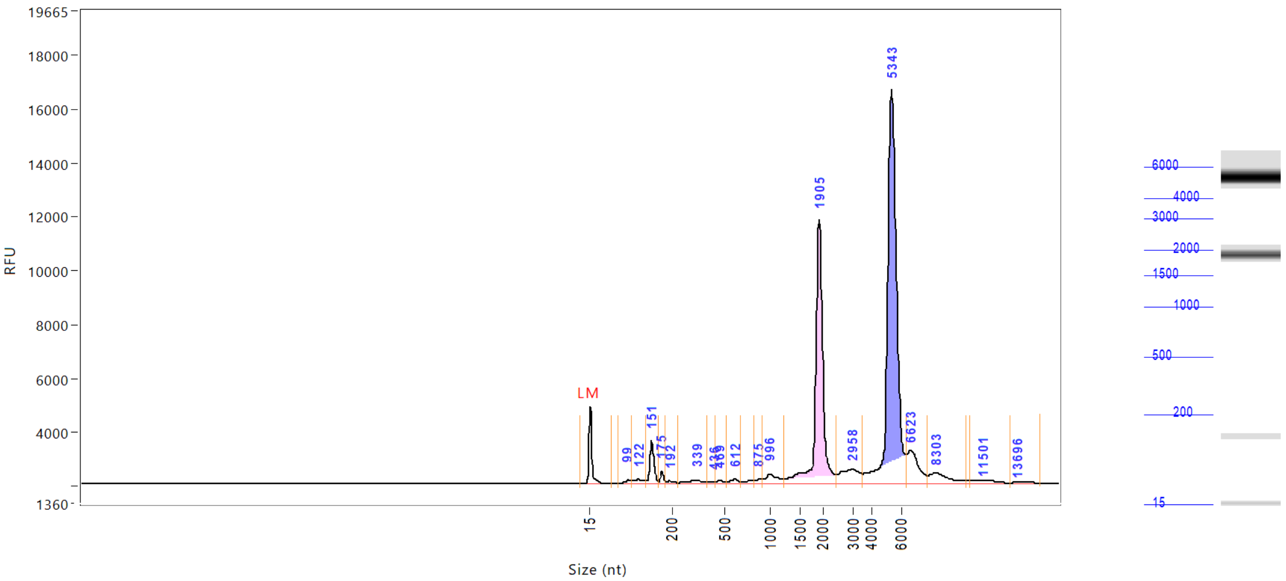

Peak	Size (nt)	Conc. (ng/uL)
1	15 (LM)	0.0135
2	99	0.0110
3	122	0.0224
4	151	0.0968
5	175	0.0205
6	192	0.0073
7	339	0.0222
8	436	0.0058
9	469	0.0104
10	612	0.0151
11	875	0.0132
12	996	0.0483
13	1905	0.8120
14	2958	0.1076
15	5343	1.3643
16	6623	0.1493
17	8303	0.0665
18	11501	0.0205
19	13696	0.0035

TIC:	2.7966	ng/uL
TIM:	6.321	nmole/L
Total Conc.:	2.8020	ng/uL

```
Sample Peak Width (sec): 6    Sample Min Peak Height: 20    Sample Baseline V to V?: Y    Sample Baseline V to V pts: 3
Sample Filter: Binomial       # of Pts for Filter: 9        Sample Start Region (min): 0  Sample End Region (min): 40
Manual Baseline Start (min): 18     Manual Baseline End (min): 38
Marker Peak Width (sec): 6    Marker Min Peak Height: 100   Marker Baseline V to V?: Y    Marker Baseline V to V pts: 3
Lower Marker Selection: First Peak > 100 RFU                Upper Marker Selection: Last Peak > 100 RFU
Ladder Size (bp): 15, 200, 500, 1000, 1500, 2000, 3000, 4000, 6000
Quantification Using: Ladder          Final Concentration (ng/uL): 0.2000          Dilution Factor: 10.0
Min. RFU for Data Processing: 2
```

 Copyright 2015 Advanced Analytical Technologies, Inc.

Data File: 2017 06 29 14H 48M.raw
Sample: UM-UC-3+HD5 4_Verd1:10
Well Location: H10

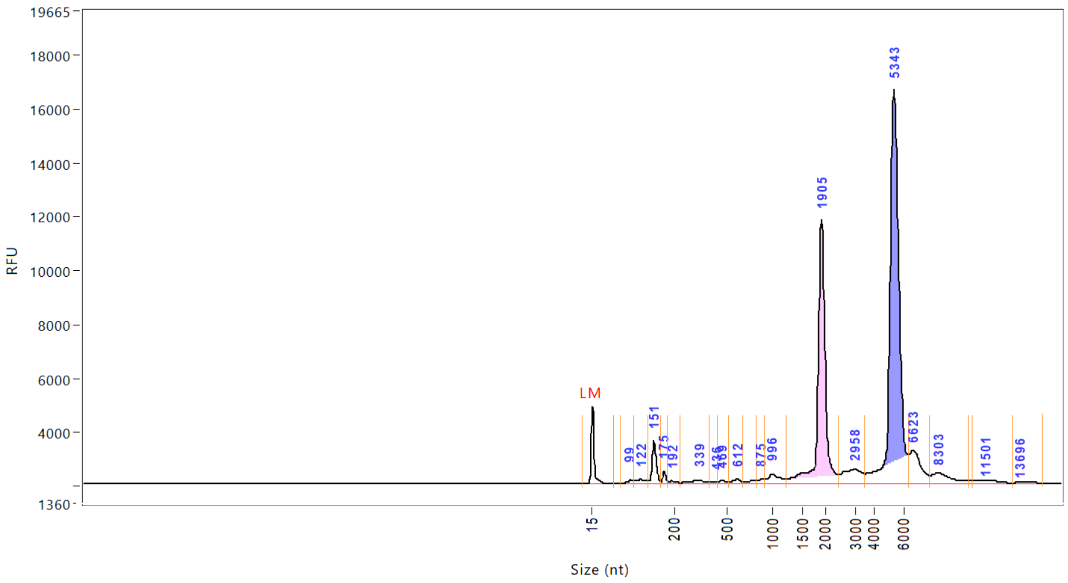

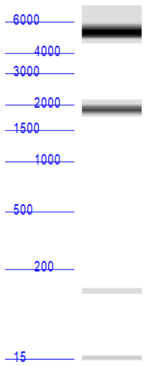

Peak	Size (nt)	Conc. (ng/uL)

28S/18S:	1.6
RQN	10.0

Sample Peak Width (sec): 6   Sample Min Peak Height: 20   Sample Baseline V to V?: Y   Sample Baseline V to V pts: 3
Sample Filter: Binomial   # of Pts for Filter: 9   Sample Start Region (min): 0   Sample End Region (min): 40
Manual Baseline Start (min): 18   Manual Baseline End (min): 38
Marker Peak Width (sec): 6   Marker Min Peak Height: 100   Marker Baseline V to V?: Y   Marker Baseline V to V pts: 3
Lower Marker Selection: First Peak > 100 RFU   Upper Marker Selection: Last Peak > 100 RFU
Ladder Size (bp): 15, 200, 500, 1000, 1500, 2000, 3000, 4000, 6000
Quantification Using: Ladder   Final Concentration (ng/uL): 0.2000   Dilution Factor: 10.0
Min. RFU for Data Processing: 2

 Copyright 2015 Advanced Analytical Technologies, Inc.

Data File: 2017 06 29 14H 48M.raw
Sample: HBLAK+vector 1_Verd1:10
Well Location: H11

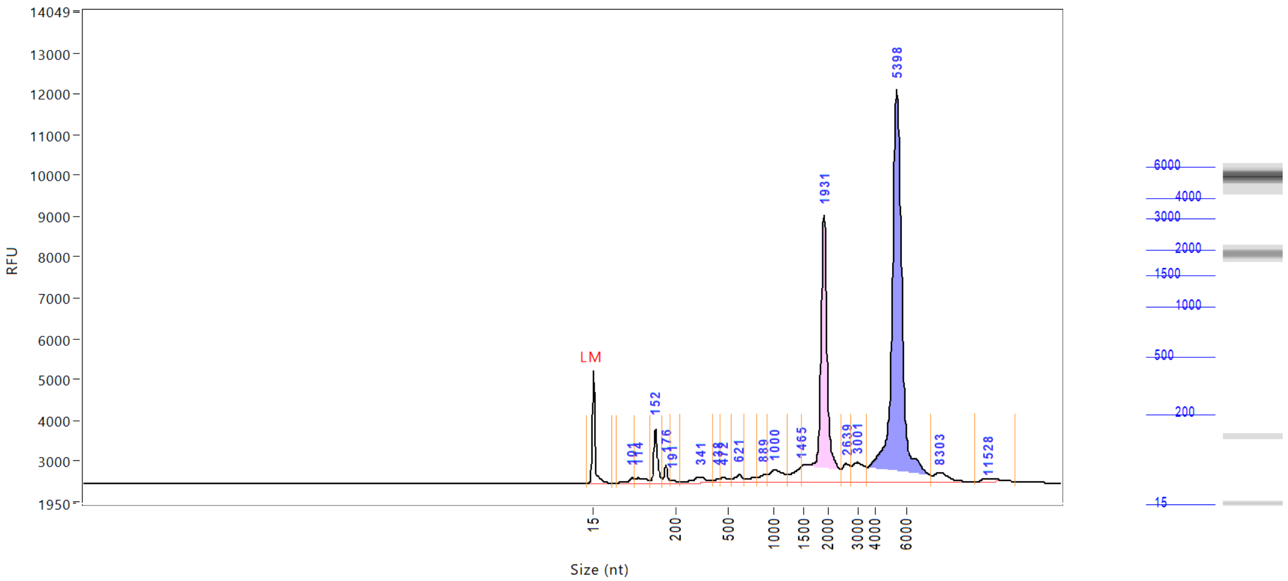

Peak	Size (nt)	Conc. (ng/uL)
1	15 (LM)	0.0135
2	101	0.0109
3	114	0.0204
4	152	0.0840
5	176	0.0213
6	191	0.0058
7	341	0.0265
8	438	0.0068
9	472	0.0130
10	621	0.0190
11	889	0.0172
12	1000	0.0542
13	1465	0.0401
14	1931	0.6064
15	2639	0.0420
16	3001	0.0703
17	5398	1.1443
18	8303	0.0301
19	11528	0.0082

TIC:	2.2204	ng/uL
TIM:	5.641	nmole/L
Total Conc.:	2.2248	ng/uL

```
Sample Peak Width (sec): 6    Sample Min Peak Height: 20    Sample Baseline V to V?: Y    Sample Baseline V to V pts: 3
Sample Filter: Binomial       # of Pts for Filter: 9        Sample Start Region (min): 0  Sample End Region (min): 40
Manual Baseline Start (min): 18     Manual Baseline End (min): 38
Marker Peak Width (sec): 6    Marker Min Peak Height: 100   Marker Baseline V to V?: Y    Marker Baseline V to V pts: 3
Lower Marker Selection: First Peak > 100 RFU                Upper Marker Selection: Last Peak > 100 RFU
Ladder Size (bp): 15, 200, 500, 1000, 1500, 2000, 3000, 4000, 6000
Quantification Using: Ladder          Final Concentration (ng/uL): 0.2000          Dilution Factor: 10.0
Min. RFU for Data Processing: 2
```

 Copyright 2015 Advanced Analytical Technologies, Inc.

Data File: 2017 06 29 14H 48M.raw
Sample: HBLAK+vector 1_Verd1:10
Well Location: H11

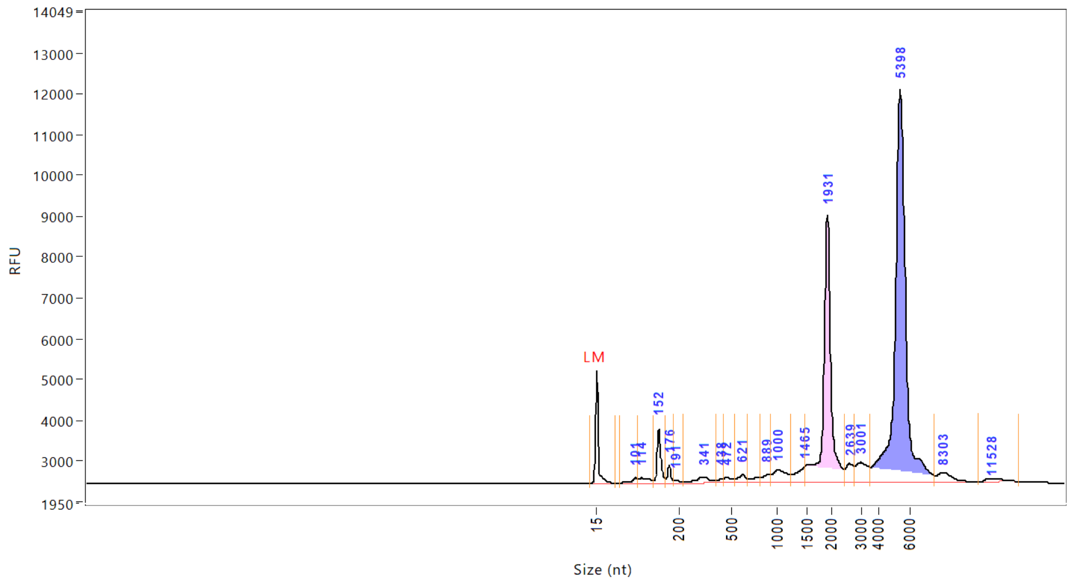

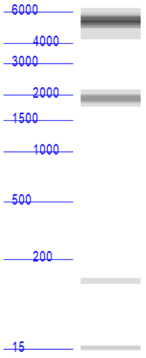

Peak	Size (nt)	Conc. (ng/uL)

28S/18S:	2.2
RQN	9.9

```
Sample Peak Width (sec): 6    Sample Min Peak Height: 20    Sample Baseline V to V?: Y    Sample Baseline V to V pts: 3
Sample Filter: Binomial       # of Pts for Filter: 9        Sample Start Region (min): 0  Sample End Region (min): 40
Manual Baseline Start (min): 18     Manual Baseline End (min): 38
Marker Peak Width (sec): 6    Marker Min Peak Height: 100   Marker Baseline V to V?: Y    Marker Baseline V to V pts: 3
Lower Marker Selection: First Peak > 100 RFU                Upper Marker Selection: Last Peak > 100 RFU
Ladder Size (bp): 15, 200, 500, 1000, 1500, 2000, 3000, 4000, 6000
Quantification Using: Ladder          Final Concentration (ng/uL): 0.2000          Dilution Factor: 10.0
Min. RFU for Data Processing: 2
```

 Copyright 2015 Advanced Analytical Technologies, Inc.

Data File: 2017 06 29 15H 52M.raw
Sample: HBLAK+vector 2_Verd1:10
Well Location: F1

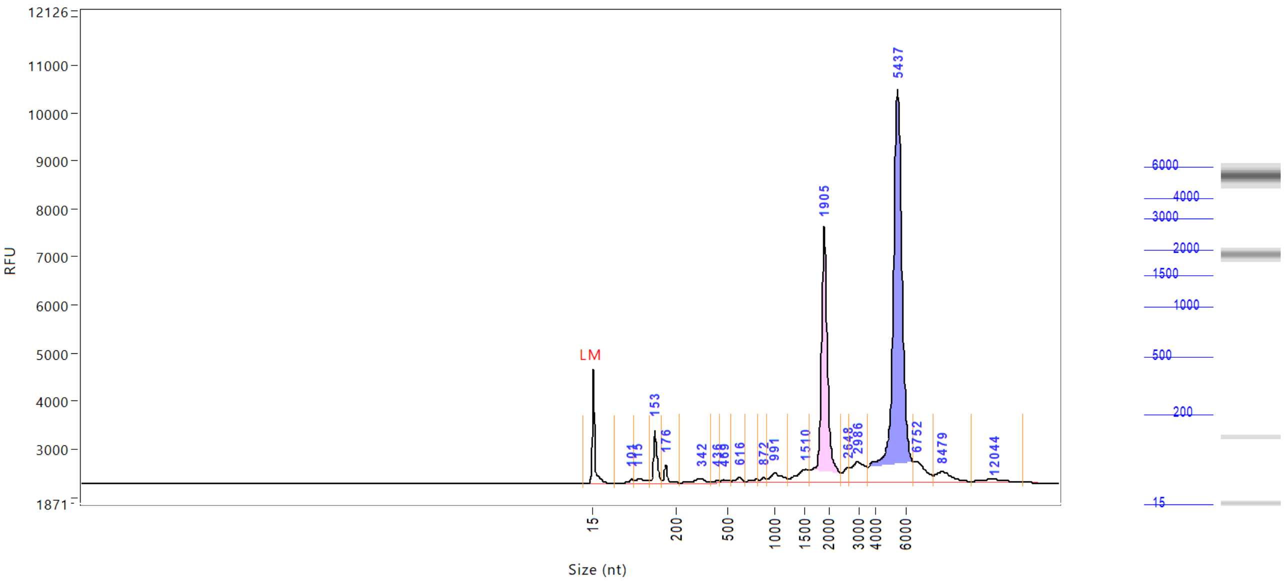

Peak	Size (nt)	Conc. (ng/uL)
1	15 (LM)	0.0345
2	101	0.0175
3	115	0.0384
4	153	0.1945
5	176	0.0543
6	342	0.0368
7	436	0.0108
8	469	0.0165
9	616	0.0277
10	872	0.0211
11	991	0.0874
12	1510	0.1251
13	1905	1.3140
14	2648	0.0693
15	2986	0.1925
16	5437	2.6564
17	6752	0.1544
18	8479	0.0899
19	12044	0.0202

TIC:	5.1268	ng/uL
TIM:	11.828	nmole/L
Total Conc.:	5.1211	ng/uL

Sample Peak Width (sec): 6   Sample Min Peak Height: 20   Sample Baseline V to V?: Y   Sample Baseline V to V pts: 3
Sample Filter: Binomial   # of Pts for Filter: 9   Sample Start Region (min): 0   Sample End Region (min): 40
Manual Baseline Start (min): 18   Manual Baseline End (min): 38
Marker Peak Width (sec): 6   Marker Min Peak Height: 100   Marker Baseline V to V?: Y   Marker Baseline V to V pts: 3
Lower Marker Selection: First Peak > 100 RFU   Upper Marker Selection: Last Peak > 100 RFU
Ladder Size (bp): 15, 200, 500, 1000, 1500, 2000, 3000, 4000, 6000
Quantification Using: Ladder   Final Concentration (ng/uL): 0.2000   Dilution Factor: 10.0
Min. RFU for Data Processing: 2

 Copyright 2015 Advanced Analytical Technologies, Inc.

Data File: 2017 06 29 15H 52M.raw
Sample: HBLAK+vector 2_Verd1:10
Well Location: F1

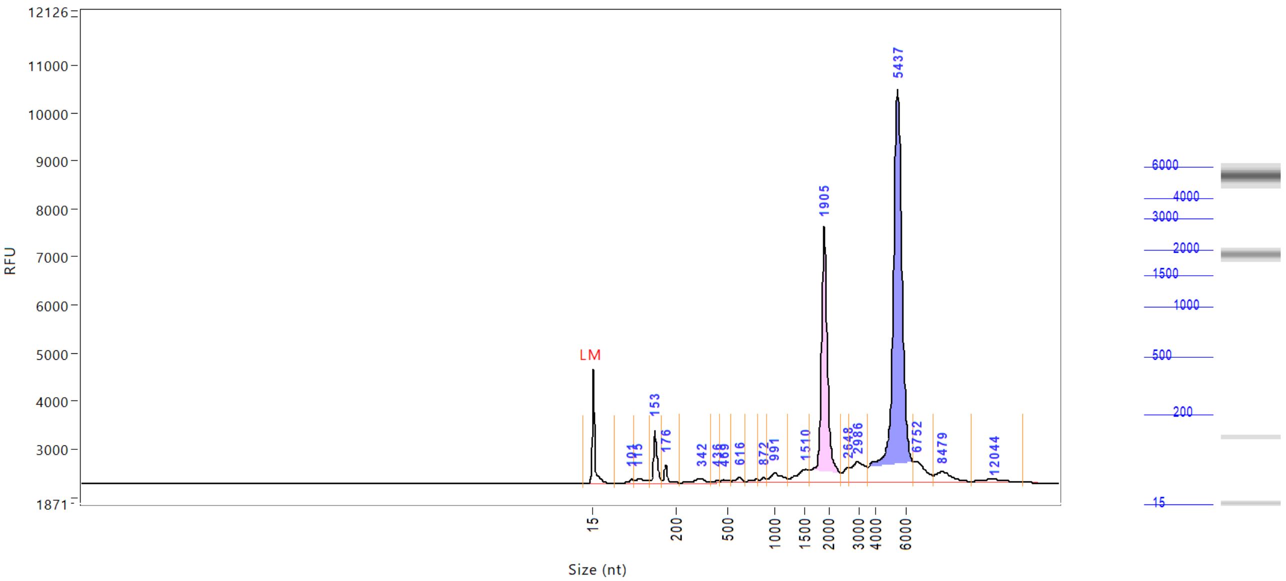

Peak	Size (nt)	Conc. (ng/uL)
	28S/18S:	2.0
	RQN	10.0

```
Sample Peak Width (sec): 6    Sample Min Peak Height: 20    Sample Baseline V to V?: Y    Sample Baseline V to V pts: 3
Sample Filter: Binomial       # of Pts for Filter: 9        Sample Start Region (min): 0  Sample End Region (min): 40
Manual Baseline Start (min): 18     Manual Baseline End (min): 38
Marker Peak Width (sec): 6    Marker Min Peak Height: 100   Marker Baseline V to V?: Y    Marker Baseline V to V pts: 3
Lower Marker Selection: First Peak > 100 RFU                Upper Marker Selection: Last Peak > 100 RFU
Ladder Size (bp): 15, 200, 500, 1000, 1500, 2000, 3000, 4000, 6000
Quantification Using: Ladder          Final Concentration (ng/uL): 0.2000          Dilution Factor: 10.0
Min. RFU for Data Processing: 2
```

 Copyright 2015 Advanced Analytical Technologies, Inc.

Data File: 2017 06 29 15H 52M.raw
Sample: HBLAK+vector 3_Verd1:11
Well Location: F2

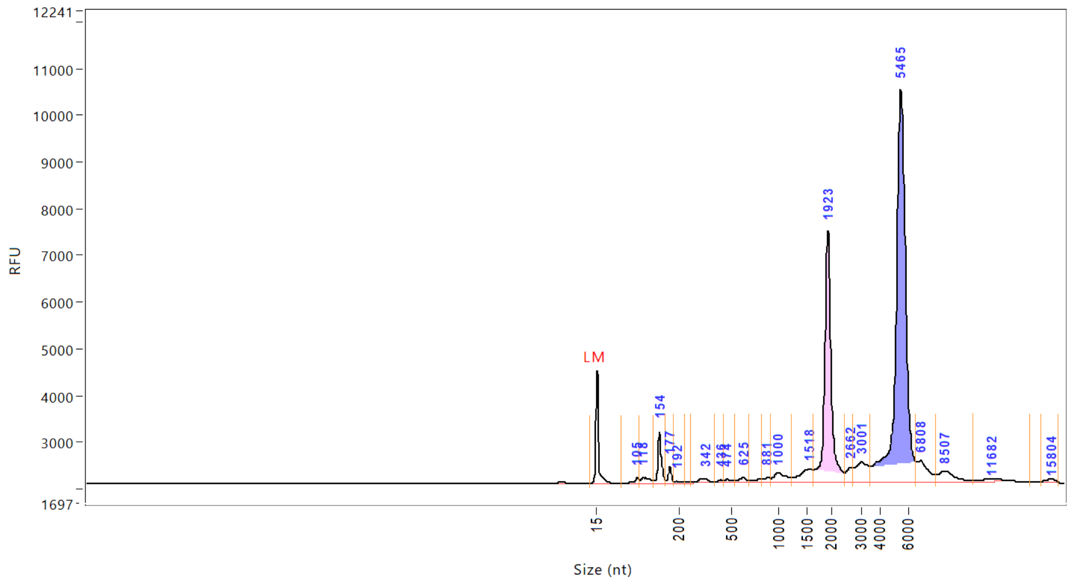

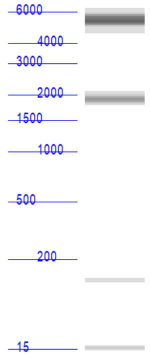

Peak	Size (nt)	Conc. (ng/uL)
1	15 (LM)	0.0345
2	105	0.0217
3	118	0.0453
4	154	0.1896
5	177	0.0446
6	192	0.0062
7	342	0.0351
8	436	0.0115
9	474	0.0174
10	625	0.0281
11	881	0.0249
12	1000	0.0882
13	1518	0.1345
14	1923	1.3102
15	2662	0.0774
16	3001	0.1875
17	5465	2.6855
18	6808	0.1549
19	8507	0.1012
20	11682	0.0287
21	15804	0.0190

TIC:	5.2116	ng/uL
TIM:	11.917	nmole/L

```
Sample Peak Width (sec): 6    Sample Min Peak Height: 20    Sample Baseline V to V?: Y    Sample Baseline V to V pts: 3
Sample Filter: Binomial       # of Pts for Filter: 9        Sample Start Region (min): 0  Sample End Region (min): 40
Manual Baseline Start (min): 18     Manual Baseline End (min): 38
Marker Peak Width (sec): 6    Marker Min Peak Height: 100   Marker Baseline V to V?: Y    Marker Baseline V to V pts: 3
Lower Marker Selection: First Peak > 100 RFU                Upper Marker Selection: Last Peak > 100 RFU
Ladder Size (bp): 15, 200, 500, 1000, 1500, 2000, 3000, 4000, 6000
Quantification Using: Ladder          Final Concentration (ng/uL): 0.2000          Dilution Factor: 10.0
Min. RFU for Data Processing: 2
```

 Copyright 2015 Advanced Analytical Technologies, Inc.

Data File: 2017 06 29 15H 52M.raw
Sample: HBLAK+vector 3_Verd1:11
Well Location: F2

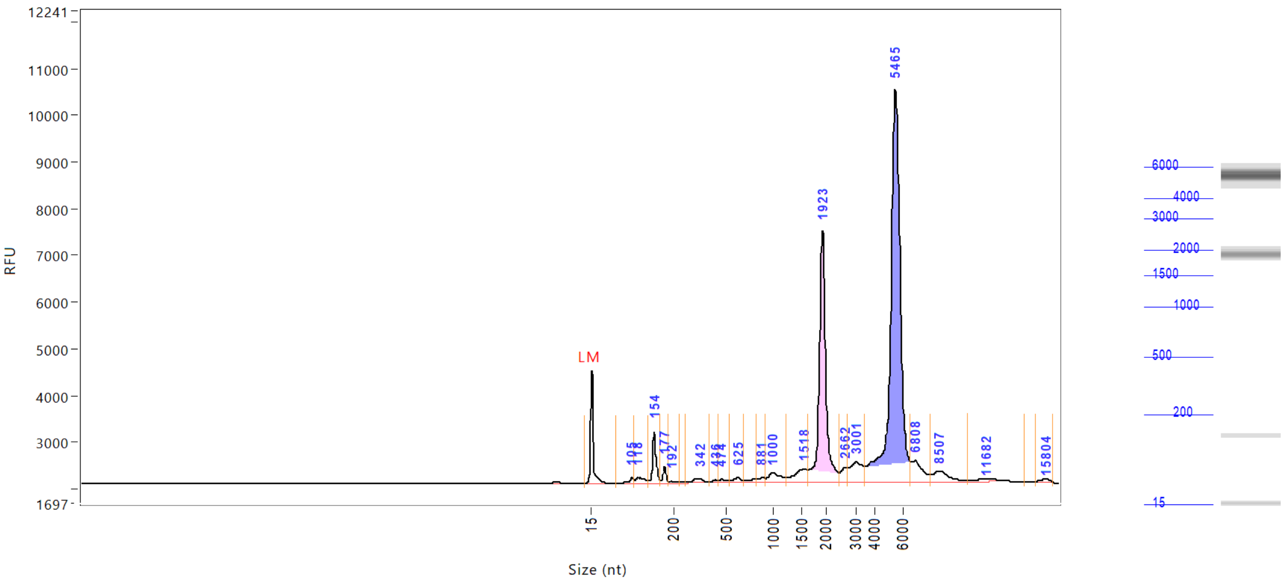

Peak	Size (nt)	Conc. (ng/uL)

Total Conc.:	5.2071	ng/uL
28S/18S:	2.0	
RQN	10.0	

Sample Peak Width (sec): 6   Sample Min Peak Height: 20   Sample Baseline V to V?: Y   Sample Baseline V to V pts: 3
Sample Filter: Binomial   # of Pts for Filter: 9   Sample Start Region (min): 0   Sample End Region (min): 40
Manual Baseline Start (min): 18   Manual Baseline End (min): 38
Marker Peak Width (sec): 6   Marker Min Peak Height: 100   Marker Baseline V to V?: Y   Marker Baseline V to V pts: 3
Lower Marker Selection: First Peak > 100 RFU   Upper Marker Selection: Last Peak > 100 RFU
Ladder Size (bp): 15, 200, 500, 1000, 1500, 2000, 3000, 4000, 6000
Quantification Using: Ladder   Final Concentration (ng/uL): 0.2000   Dilution Factor: 10.0
Min. RFU for Data Processing: 2

 Copyright 2015 Advanced Analytical Technologies, Inc.

Data File: 2017 06 29 15H 52M.raw
Sample: HBLAK+vector 4_Verd1:12
Well Location: F3

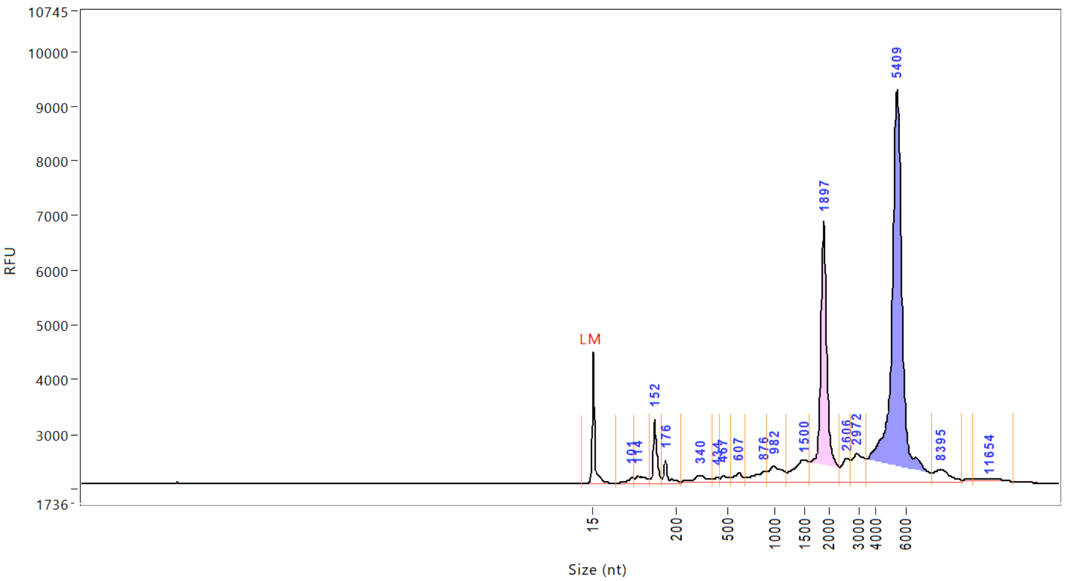

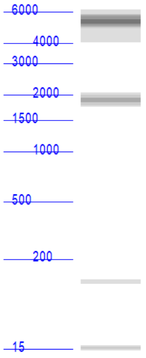

Peak	Size (nt)	Conc. (ng/uL)
1	15 (LM)	0.0345
2	101	0.0283
3	114	0.0565
4	152	0.2110
5	176	0.0727
6	340	0.0743
7	434	0.0203
8	467	0.0377
9	607	0.0576
10	876	0.0937
11	982	0.1557
12	1500	0.2276
13	1897	1.2463
14	2606	0.1182
15	2972	0.2212
16	5409	2.7122
17	8395	0.0961
18	11654	0.0271

TIC:	5.4566	ng/uL
TIM:	14.723	nmole/L
Total Conc.:	5.4340	ng/uL

28S/18S: 2.3

```
Sample Peak Width (sec): 6    Sample Min Peak Height: 20    Sample Baseline V to V?: Y    Sample Baseline V to V pts: 3
Sample Filter: Binomial       # of Pts for Filter: 9        Sample Start Region (min): 0  Sample End Region (min): 40
Manual Baseline Start (min): 18     Manual Baseline End (min): 38
Marker Peak Width (sec): 6    Marker Min Peak Height: 100   Marker Baseline V to V?: Y    Marker Baseline V to V pts: 3
Lower Marker Selection: First Peak > 100 RFU                Upper Marker Selection: Last Peak > 100 RFU
Ladder Size (bp): 15, 200, 500, 1000, 1500, 2000, 3000, 4000, 6000
Quantification Using: Ladder          Final Concentration (ng/uL): 0.2000          Dilution Factor: 10.0
Min. RFU for Data Processing: 2
```

 Copyright 2015 Advanced Analytical Technologies, Inc.

Data File: 2017 06 29 15H 52M.raw
Sample: HBLAK+vector 4_Verd1:12
Well Location: F3

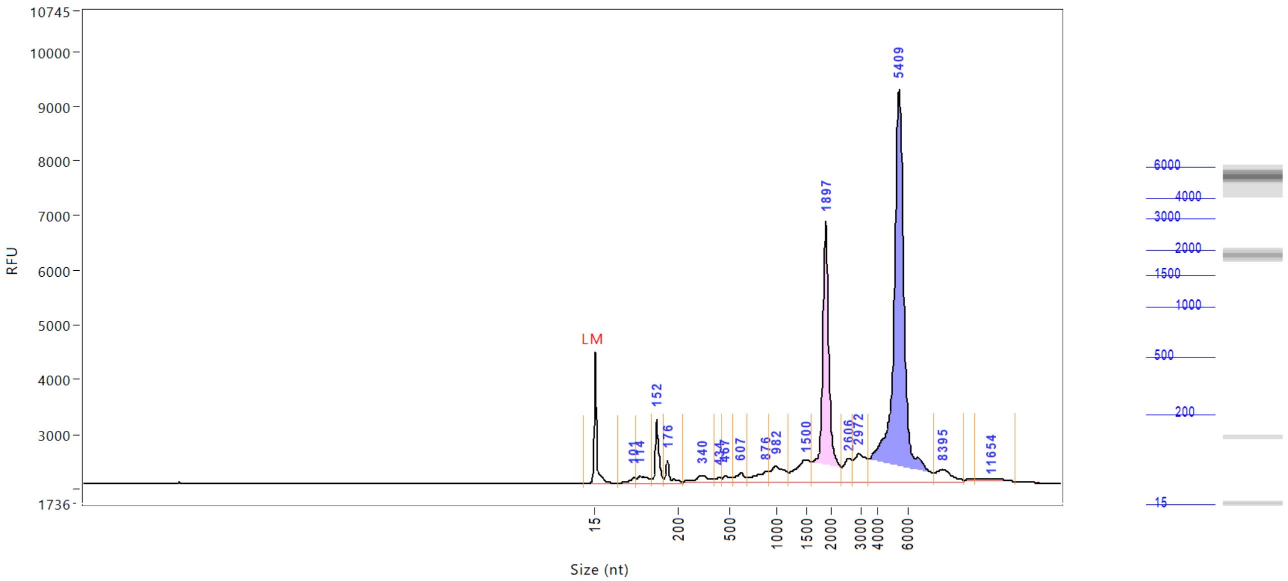

Peak	Size (nt)	Conc. (ng/uL)
	RQN	9.5

Sample Peak Width (sec): 6   Sample Min Peak Height: 20   Sample Baseline V to V?: Y   Sample Baseline V to V pts: 3
Sample Filter: Binomial   # of Pts for Filter: 9   Sample Start Region (min): 0   Sample End Region (min): 40
Manual Baseline Start (min): 18   Manual Baseline End (min): 38
Marker Peak Width (sec): 6   Marker Min Peak Height: 100   Marker Baseline V to V?: Y   Marker Baseline V to V pts: 3
Lower Marker Selection: First Peak > 100 RFU   Upper Marker Selection: Last Peak > 100 RFU
Ladder Size (bp): 15, 200, 500, 1000, 1500, 2000, 3000, 4000, 6000
Quantification Using: Ladder   Final Concentration (ng/uL): 0.2000   Dilution Factor: 10.0
Min. RFU for Data Processing: 2

 Copyright 2015 Advanced Analytical Technologies, Inc.

Data File: 2017 06 29 13H 45M.raw
Sample: Ladder
Well Location: G12

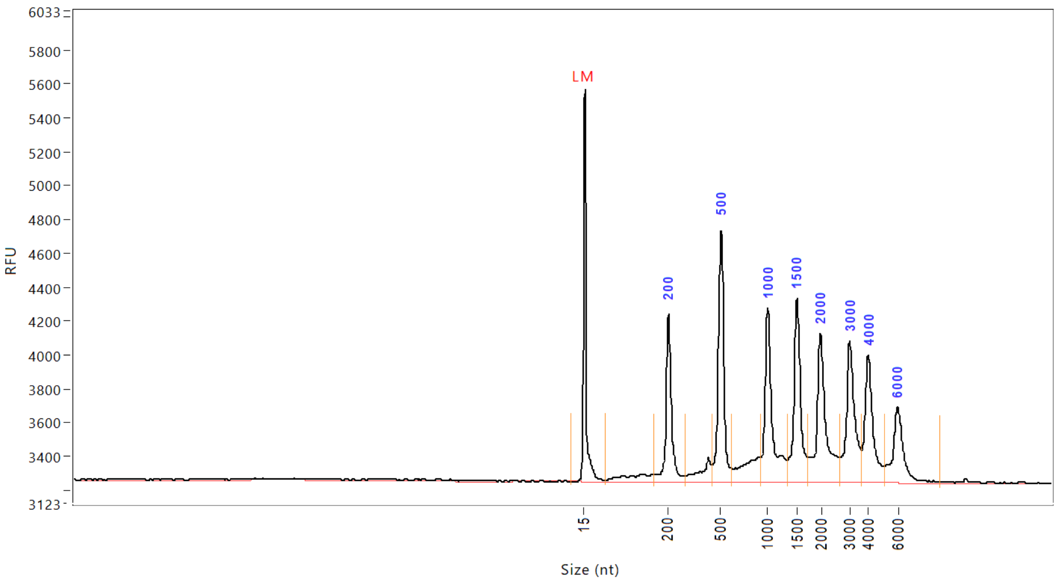

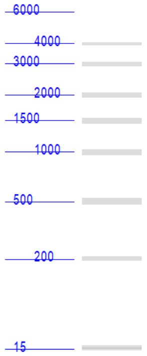

Peak	Size (nt)	Conc. (ng/uL)	
1	15 (LM)	0.0310	
2	200	0.2130	
3	500	0.3165	
4	1000	0.2715	
5	1500	0.2479	
6	2000	0.2642	
7	3000	0.2280	
8	4000	0.1833	
9	6000	0.1363	
	TIC:	1.8608	ng/uL
	TIM:	7.513	nmole/L
	Total Conc.:	2.0000	ng/uL

```
Sample Peak Width (sec): 6    Sample Min Peak Height: 100   Sample Baseline V to V?: Y    Sample Baseline V to V pts: 3
Sample Filter: Binomial       # of Pts for Filter: 9        Sample Start Region (min): 0  Sample End Region (min): 40
Manual Baseline Start (min): 18     Manual Baseline End (min): 38
Marker Peak Width (sec): 6    Marker Min Peak Height: 100   Marker Baseline V to V?: Y    Marker Baseline V to V pts: 3
Lower Marker Selection: First Peak > 100 RFU                Upper Marker Selection: Last Peak > 100 RFU
Ladder Size (bp): 15, 200, 500, 1000, 1500, 2000, 3000, 4000, 6000
Quantification Using: Ladder          Final Concentration (ng/uL): 0.2000          Dilution Factor: 10.0
Min. RFU for Data Processing: 2
```

 Copyright 2015 Advanced Analytical Technologies, Inc.
