# Supplementary material for: Proteomic and transcriptomic profiles of human urothelial cancer cells with histone deacetylase 5 overexpression
Source: Sci Data. 2022 May 27;9:240. doi: 10.1038/s41597-022-01319-0 (PMC9142574; doi:10.1038/s41597-022-01319-0)
Supplement: Supplementary file 3 — QC report of library [file 41597_2022_1319_MOESM3_ESM.pdf]

Fragment Analyzer Project Summary:

Data Files: 2017 07 25 15H 26M.raw, 2017 07 25 16H 33M.raw, 2017 07 25 17H 40M.raw, 2017 07 25 18H 46M.raw

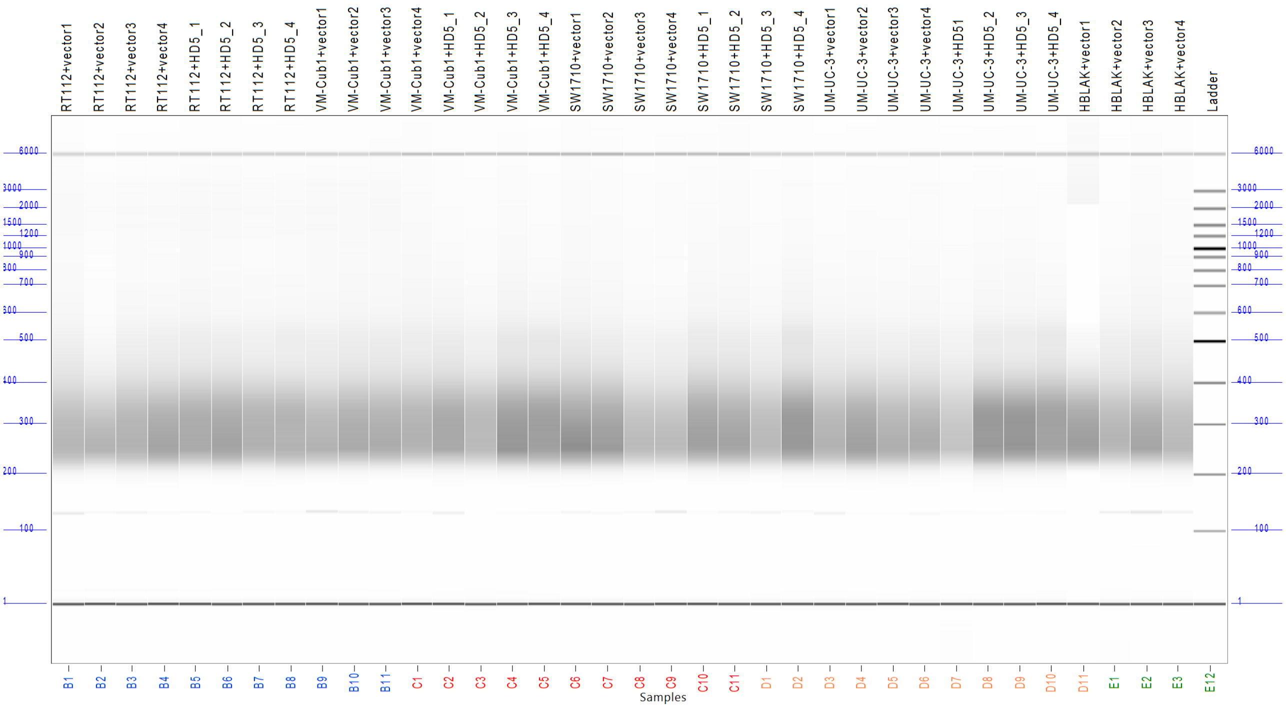

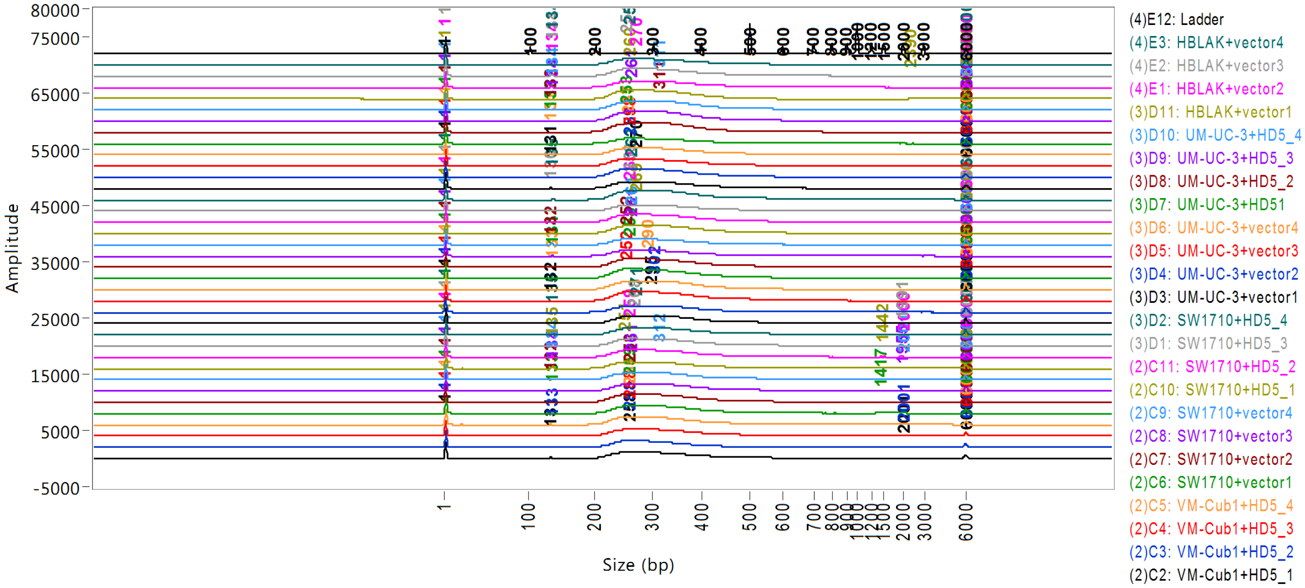

 Copyright 2015 Advanced Analytical Technologies, Inc. Date Printed: 8/21/2017 10:38 AM

Filename and Data Path: Y:\GTL_Shared\05_AuftraegeProjekte\03_NGS_MA\02_NGS_MA_Daten\2017_NGS_MA\116-NGS-270617-0\116-NGS-270617-4\15-26-48\2017 07 25 15H 26M.raw
Created: Tuesday, July 25, 2017 3:43:09 PM
# of Capillaries: 12
Array Serial #: 032417-02SFS
Effect Length: 33 cm
Array Usage Count: 2
FA Version #: 1.1.0.11
Device Serial #: 3228

METHOD INFORMATION
Method Name: DNF-474-33 - HS NGS Fragment 1-6000bp.mthds
Gel Prime: No
Full Conditioning: Yes
Gel Prime to Buffer: No
Gel Selection: Gel 2
Perform Prerun: 6.0 kV, 30 sec.
Rinse: No
Marker 1: No
Rinse: Tray: 3, Row: A, # Dips: 1
Sample Injection: 5.0 kV, 30 sec.
Separation: 6.0 kV, 50.0 min.
Tray Name: Tray-1

Analysis Mode: NGS

NOTE
This analysis was carried out by Thorsten Wachtmeister.

 Copyright 2015 Advanced Analytical Technologies, Inc.

Filename and Data Path: Y:\GTL_Shared\05_AuftraegeProjekte\03_NGS_MA\02_NGS_MA_Daten\20
Filename and Data Path: 17_NGS_MA\116-NGS-270617-0\116-NGS-270617-4\16-33-31\2017 07 2
Filename and Data Path: 5 16H 33M.raw
Created: Tuesday, July 25, 2017 4:49:50 PM
# of Capillaries: 12
Array Serial #: 032417-02SFS
Effect Length: 33 cm
Array Usage Count: 3
FA Version #: 1.1.0.11
Device Serial #: 3228

METHOD INFORMATION
Method Name: DNF-474-33 - HS NGS Fragment 1-6000bp.mthds
Gel Prime: No
Full Conditioning: Yes
Gel Prime to Buffer: No
Gel Selection: Gel 2
Perform Prerun: 6.0 kV, 30 sec.
Rinse: No
Marker 1: No
Rinse: Tray: 3, Row: A, # Dips: 1
Sample Injection: 5.0 kV, 30 sec.
Separation: 6.0 kV, 50.0 min.
Tray Name: Tray-1

Analysis Mode: NGS

NOTE
This analysis was carried out by Thorsten Wachtmeister.

 Copyright 2015 Advanced Analytical Technologies, Inc.

Filename and Data Path: Y:\GTL_Shared\05_AuftraegeProjekte\03_NGS_MA\02_NGS_MA_Daten\2017_NGS_MA\116-NGS-270617-0\116-NGS-270617-4\17-40-12\2017 07 25 17H 40M.raw
Created: Tuesday, July 25, 2017 5:56:30 PM
# of Capillaries: 12
Array Serial #: 032417-02SFS
Effect Length: 33 cm
Array Usage Count: 4
FA Version #: 1.1.0.11
Device Serial #: 3228

METHOD INFORMATION
Method Name: DNF-474-33 - HS NGS Fragment 1-6000bp.mthds
Gel Prime: No
Full Conditioning: Yes
Gel Prime to Buffer: No
Gel Selection: Gel 2
Perform Prerun: 6.0 kV, 30 sec.
Rinse: No
Marker 1: No
Rinse: Tray: 3, Row: A, # Dips: 1
Sample Injection: 5.0 kV, 30 sec.
Separation: 6.0 kV, 50.0 min.
Tray Name: Tray-1

Analysis Mode: NGS

NOTE
This analysis was carried out by Thorsten Wachtmeister.

 Copyright 2015 Advanced Analytical Technologies, Inc.

Filename and Data Path: Y:\GTL_Shared\05_AuftraegeProjekte\03_NGS_MA\02_NGS_MA_Daten\2017_NGS_MA\116-NGS-270617-0\116-NGS-270617-4\18-46-52\2017 07 25 18H 46M.raw
Created: Tuesday, July 25, 2017 7:03:11 PM
# of Capillaries: 12
Array Serial #: 032417-02SFS
Effect Length: 33 cm
Array Usage Count: 5
FA Version #: 1.1.0.11
Device Serial #: 3228

METHOD INFORMATION
Method Name: DNF-474-33 - HS NGS Fragment 1-6000bp.mthds
Gel Prime: No
Full Conditioning: Yes
Gel Prime to Buffer: No
Gel Selection: Gel 2
Perform Prerun: 6.0 kV, 30 sec.
Rinse: No
Marker 1: No
Rinse: Tray: 3, Row: A, # Dips: 1
Sample Injection: 5.0 kV, 30 sec.
Separation: 6.0 kV, 50.0 min.
Tray Name: Tray-1

Analysis Mode: NGS

NOTE
This analysis was carried out by Thorsten Wachtmeister.

 Copyright 2015 Advanced Analytical Technologies, Inc.

2017 07 25 15H 26M.raw
B1: RT112+vector1

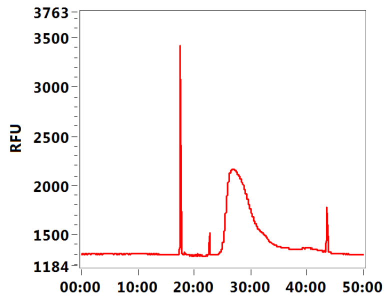

2017 07 25 15H 26M.raw
B2: RT112+vector2

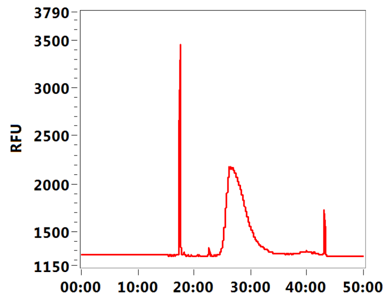

2017 07 25 15H 26M.raw
B3: RT112+vector3

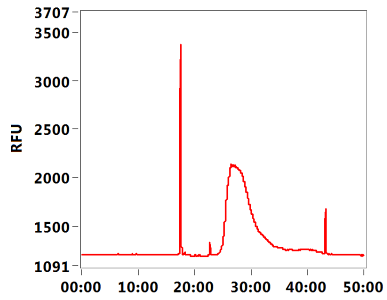

2017 07 25 15H 26M.raw
B4: RT112+vector4

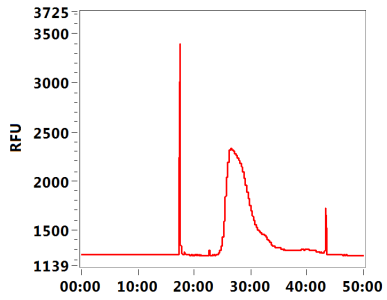

2017 07 25 15H 26M.raw
B5: RT112+HD5_1

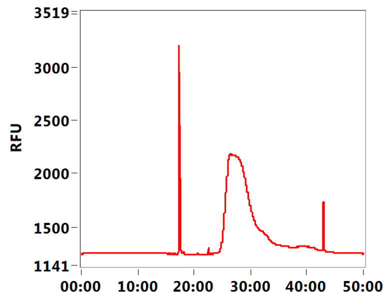

2017 07 25 15H 26M.raw
B6: RT112+HD5_2

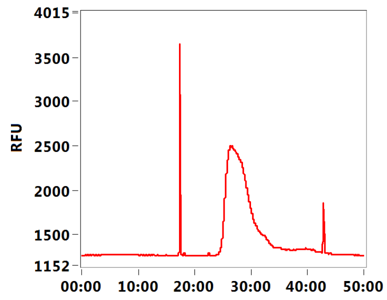

2017 07 25 15H 26M.raw
B7: RT112+HD5_3

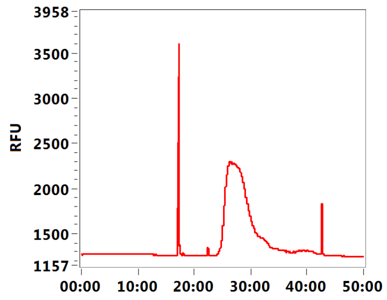

2017 07 25 15H 26M.raw
B8: RT112+HD5_4

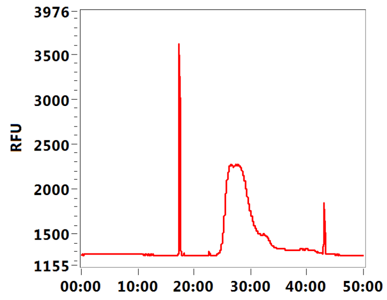

2017 07 25 15H 26M.raw
B9: VM-Cub1+vector1

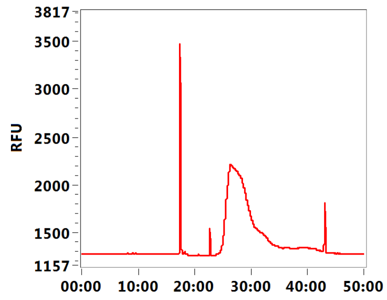

2017 07 25 15H 26M.raw
B10: VM-Cub1+vector2

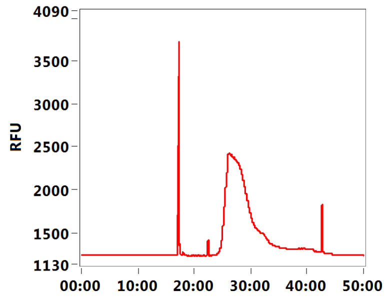

2017 07 25 15H 26M.raw
B11: VM-Cub1+vector3

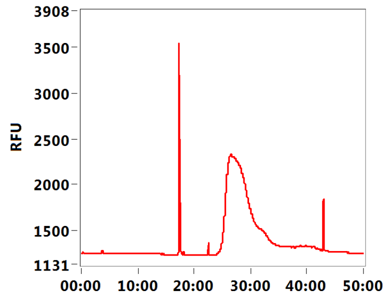

2017 07 25 16H 33M.raw
C1: VM-Cub1+vector4

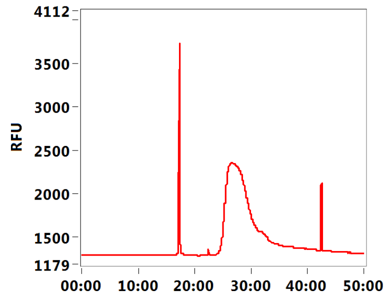

 Copyright 2015 Advanced Analytical Technologies, Inc.

2017 07 25 16H 33M.raw
C2: VM-Cub1+HD5_1

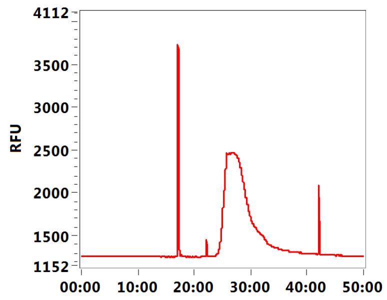

2017 07 25 16H 33M.raw
C3: VM-Cub1+HD5_2

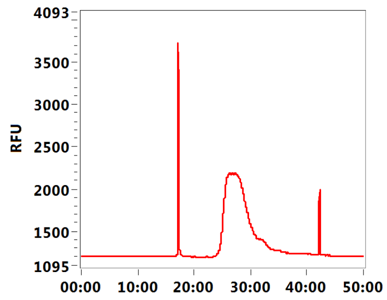

2017 07 25 16H 33M.raw
C4: VM-Cub1+HD5_3

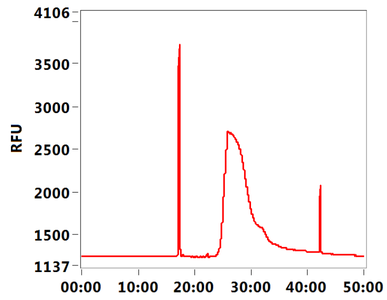

2017 07 25 16H 33M.raw
C5: VM-Cub1+HD5_4

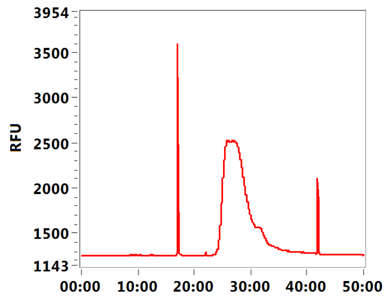

2017 07 25 16H 33M.raw
C6: SW1710+vector1

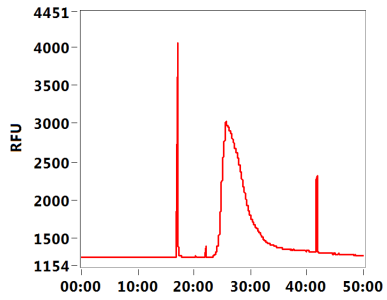

2017 07 25 16H 33M.raw
C7: SW1710+vector2

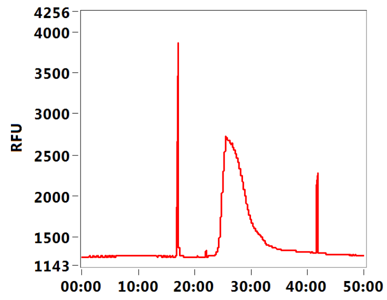

2017 07 25 16H 33M.raw
C8: SW1710+vector3

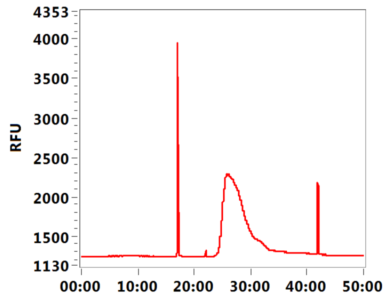

2017 07 25 16H 33M.raw
C9: SW1710+vector4

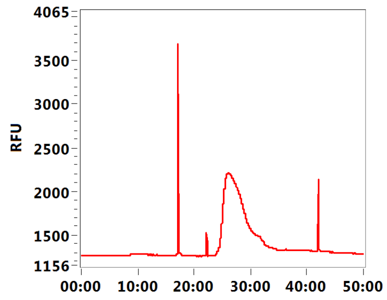

2017 07 25 16H 33M.raw
C10: SW1710+HD5_1

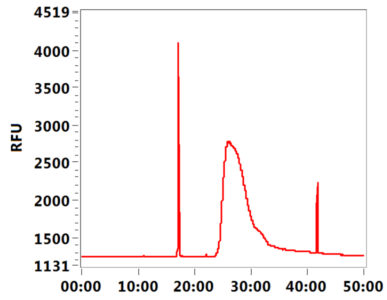

2017 07 25 16H 33M.raw
C11: SW1710+HD5_2

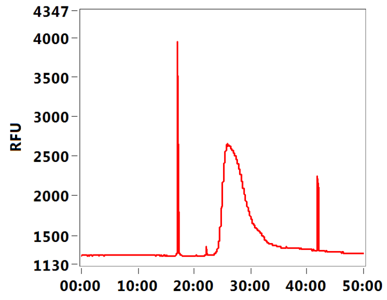

2017 07 25 17H 40M.raw
D1: SW1710+HD5_3

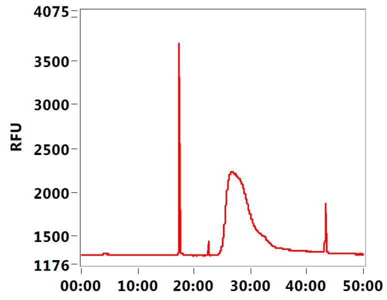

2017 07 25 17H 40M.raw
D2: SW1710+HD5_4

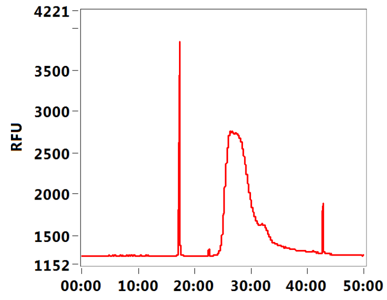

 Copyright 2015 Advanced Analytical Technologies, Inc.

2017 07 25 17H 40M.raw
D3: UM-UC-3+vector1

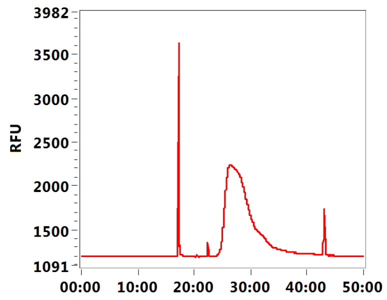

2017 07 25 17H 40M.raw
D4: UM-UC-3+vector2

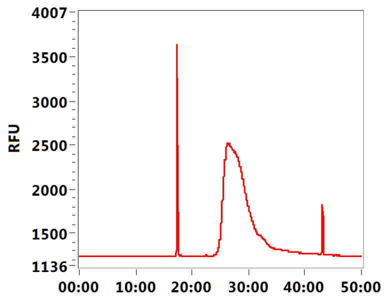

2017 07 25 17H 40M.raw
D5: UM-UC-3+vector3

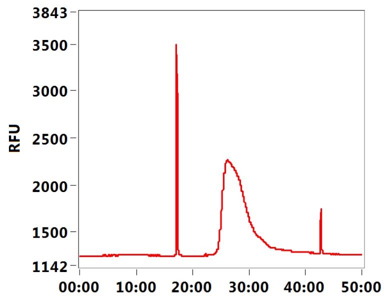

2017 07 25 17H 40M.raw
D6: UM-UC-3+vector4

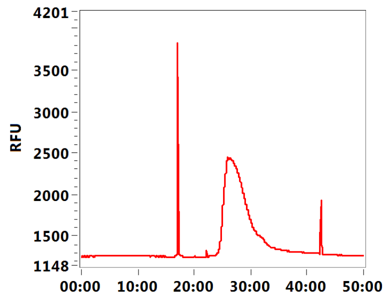

2017 07 25 17H 40M.raw
D7: UM-UC-3+HD51

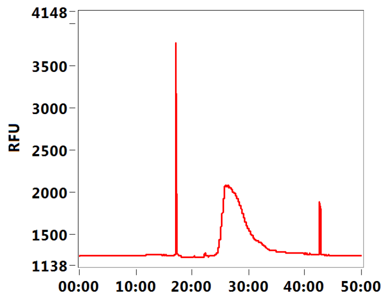

2017 07 25 17H 40M.raw
D8: UM-UC-3+HD5_2

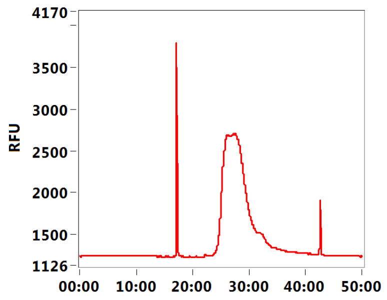

2017 07 25 17H 40M.raw
D9: UM-UC-3+HD5_3

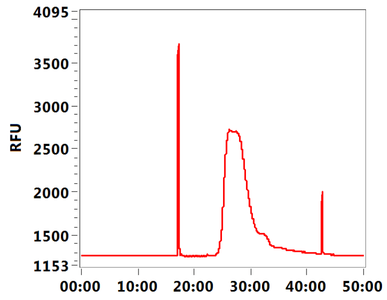

2017 07 25 17H 40M.raw
D10: UM-UC-3+HD5_4

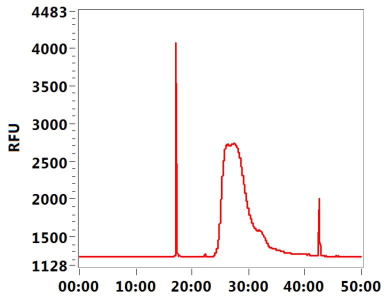

2017 07 25 17H 40M.raw
D11: HBLAK+vector1

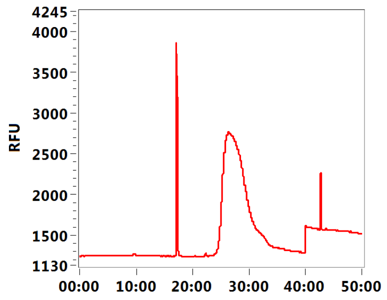

2017 07 25 18H 46M.raw
E1: HBLAK+vector2

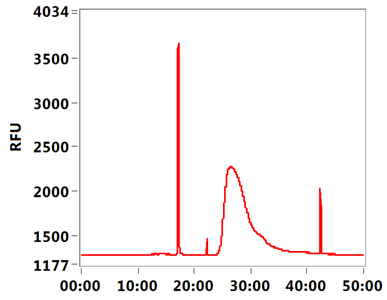

2017 07 25 18H 46M.raw
E2: HBLAK+vector3

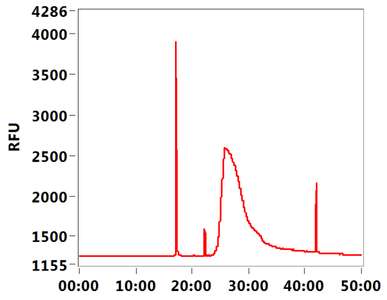

2017 07 25 18H 46M.raw
E3: HBLAK+vector4

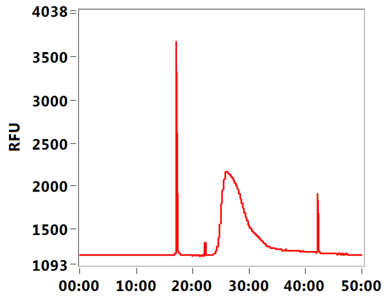

 Copyright 2015 Advanced Analytical Technologies, Inc.

2017 07 25 18H 46M.raw

E12: Ladder

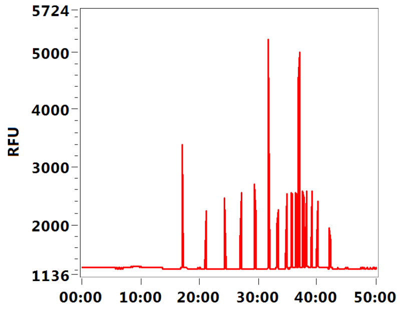

Copyright 2015 Advanced Analytical Technologies, Inc.

Data File: 2017 07 25 15H 26M.raw
Sample: RT112+vector1
Well Location: B1

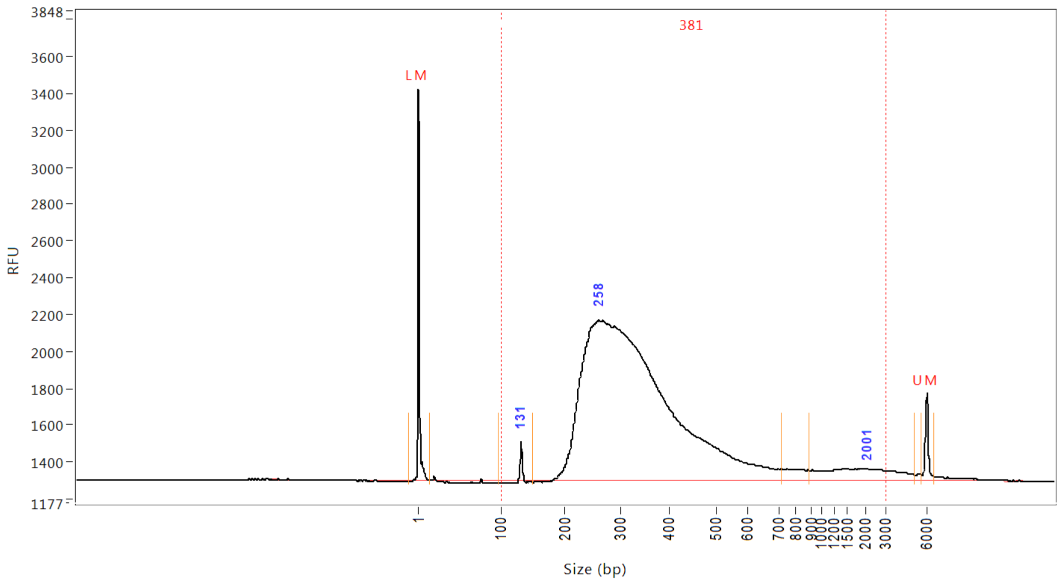

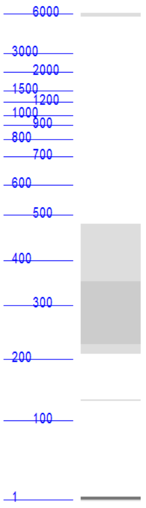

Size (bp)	Molarity (nmole/L)	Conc. (ng/uL)	From (bp)	To (bp)	Avg. Size (bp)	CV%	RFU	Corr. Peak Area
1 (LM)	16.057	0.0123	0	15	1	182.87	2118	12.181
131	0.159	0.0127	98	150	131	1.08	208	1.050
258	11.852	1.8622	150	717	331	28.89	867	153.828
2001	0.070	0.0856	891	5129	2144	52.42	66	7.073
6000 (UM)	0.001	0.0022	5637	6553	5990	2.47	477	2.220

TIC:	1.9605	ng/uL
TIM:	12.082	nmole/L
Total Conc.:	1.9907	ng/uL

Smear Analysis	100 bp to 3000 bp	1.9684 ng/ul	98.9 %Total	8.510 nmole/L	381 Avg. Size (b.p.)	74.52 %CV

```
Sample Peak Width (sec): 50   Sample Min Peak Height: 25    Sample Baseline V to V?: Y    Sample Baseline V to V pts: 3
Sample Filter: Binomial       # of Pts for Filter: 3        Sample Start Region (min): 0  Sample End Region (min): 50
Manual Baseline Start (min): 10     Manual Baseline End (min): 48
Marker Peak Width (sec): 5    Marker Min Peak Height: 200   Marker Baseline V to V?: Y    Marker Baseline V to V pts: 3
Lower Marker Selection: First Peak > 200 RFU                Upper Marker Selection: Last Peak > 200 RFU
Ladder Size (bp): 1, 100, 200, 300, 400, 500, 600, 700, 800, 900, 1000, 1200, 1500, 2000, 3000, 6000
Quantification Using: Ladder          Final Concentration (ng/uL): 0.0830          Dilution Factor: 12.0
```

 Copyright 2015 Advanced Analytical Technologies, Inc.

Data File: 2017 07 25 15H 26M.raw
Sample: RT112+vector2
Well Location: B2

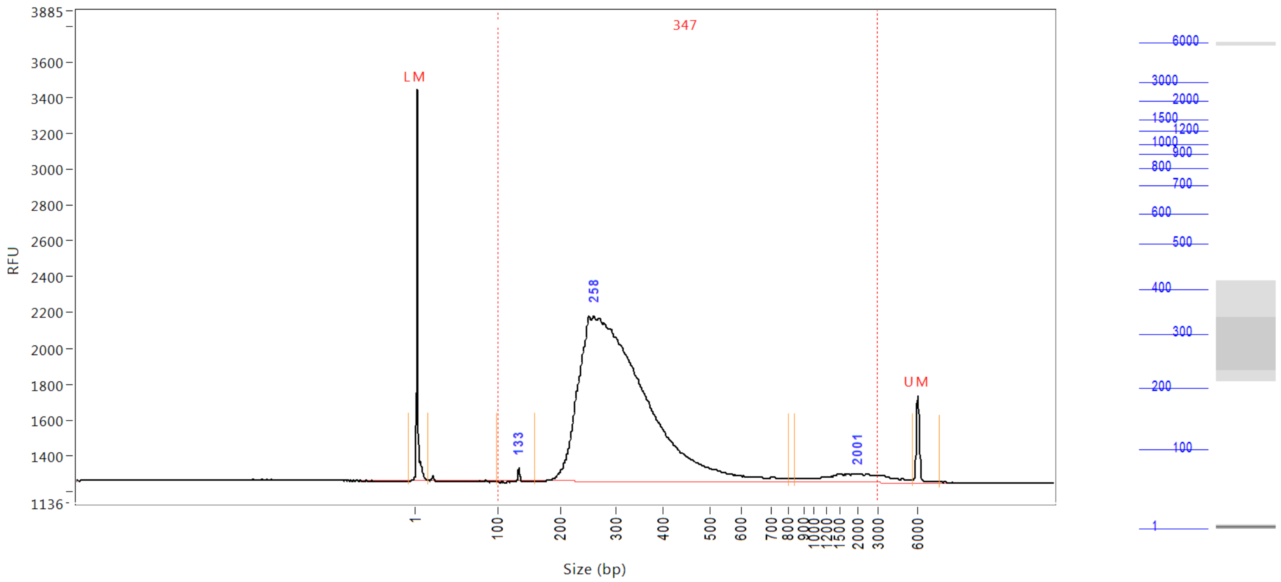

Size (bp)	Molarity (nmole/L)	Conc. (ng/uL)	From (bp)	To (bp)	Avg. Size (bp)	CV%	RFU	Corr. Peak Area
1 (LM)	16.057	0.0123	0	17	1	143.44	2182	12.660
133	0.043	0.0035	99	158	132	0.82	75	0.298
258	9.730	1.5253	158	800	312	25.51	920	130.949
2001	0.041	0.0503	837	5637	2210	51.99	47	4.322
6000 (UM)	0.001	0.0020	5637	7564	6004	3.42	480	2.045

TIC:	1.5791	ng/uL
TIM:	9.814	nmole/L
Total Conc.:	1.5823	ng/uL

Smear Analysis 100 bp to 3000 bp 1.5695 ng/ul 99.2 %Total 7.451 nmole/L 347 Avg. Size (b.p.) 71.87 %CV

```
Sample Peak Width (sec): 50   Sample Min Peak Height: 25    Sample Baseline V to V?: Y    Sample Baseline V to V pts: 3
Sample Filter: Binomial       # of Pts for Filter: 3        Sample Start Region (min): 0  Sample End Region (min): 50
Manual Baseline Start (min): 10     Manual Baseline End (min): 48
Marker Peak Width (sec): 5    Marker Min Peak Height: 200   Marker Baseline V to V?: Y    Marker Baseline V to V pts: 3
Lower Marker Selection: First Peak > 200 RFU                Upper Marker Selection: Last Peak > 200 RFU
Ladder Size (bp): 1, 100, 200, 300, 400, 500, 600, 700, 800, 900, 1000, 1200, 1500, 2000, 3000, 6000
Quantification Using: Ladder          Final Concentration (ng/uL): 0.0830          Dilution Factor: 12.0
```

 Copyright 2015 Advanced Analytical Technologies, Inc.

Data File: 2017 07 25 15H 26M.raw
Sample: RT112+vector3
Well Location: B3

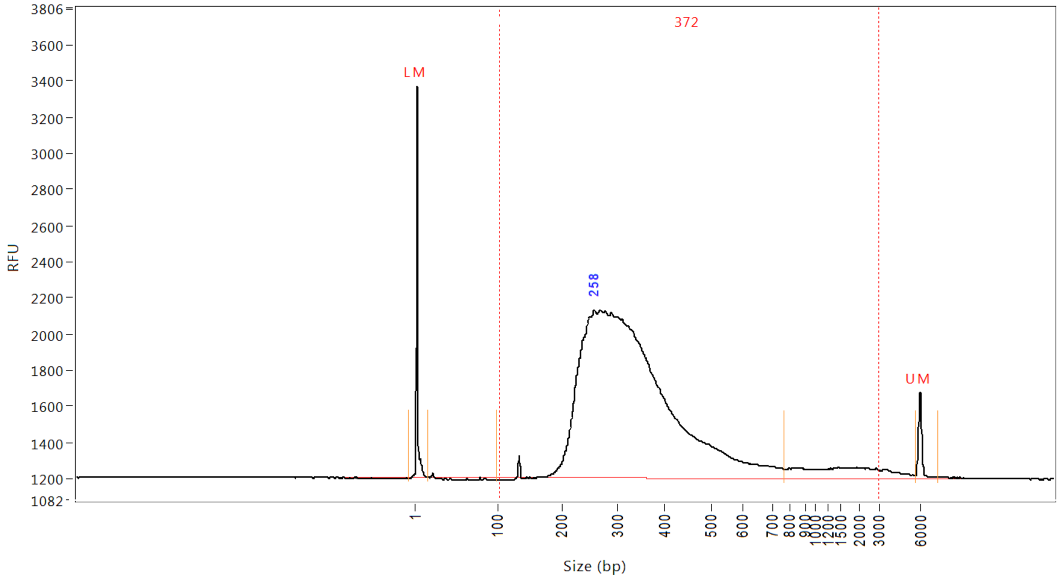

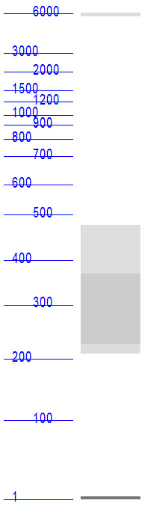

Size (bp)	Molarity (nmole/L)	Conc. (ng/uL)	From (bp)	To (bp)	Avg. Size (bp)	CV%	RFU	Corr. Peak Area
1 (LM)	16.057	0.0123	0	16	1	182.02	2163	12.511
258	12.468	1.9545	98	769	331	29.32	928	165.830
6000 (UM)	0.001	0.0022	5686	7371	6042	3.98	472	2.194

TIC:	1.9545	ng/uL
TIM:	12.468	nmole/L
Total Conc.:	2.0451	ng/uL

Smear Analysis	100 bp to 3000 bp	2.0278 ng/ul	99.2 %Total	8.971 nmole/L	372 Avg. Size (b.p.)	70.16 %CV

```
Sample Peak Width (sec): 50   Sample Min Peak Height: 25    Sample Baseline V to V?: Y    Sample Baseline V to V pts: 3
Sample Filter: Binomial       # of Pts for Filter: 3        Sample Start Region (min): 0  Sample End Region (min): 50
Manual Baseline Start (min): 10     Manual Baseline End (min): 48
Marker Peak Width (sec): 5    Marker Min Peak Height: 200   Marker Baseline V to V?: Y    Marker Baseline V to V pts: 3
Lower Marker Selection: First Peak > 200 RFU                Upper Marker Selection: Last Peak > 200 RFU
Ladder Size (bp): 1, 100, 200, 300, 400, 500, 600, 700, 800, 900, 1000, 1200, 1500, 2000, 3000, 6000
Quantification Using: Ladder          Final Concentration (ng/uL): 0.0830          Dilution Factor: 12.0
```

 Copyright 2015 Advanced Analytical Technologies, Inc.

Data File: 2017 07 25 15H 26M.raw
Sample: RT112+vector4
Well Location: B4

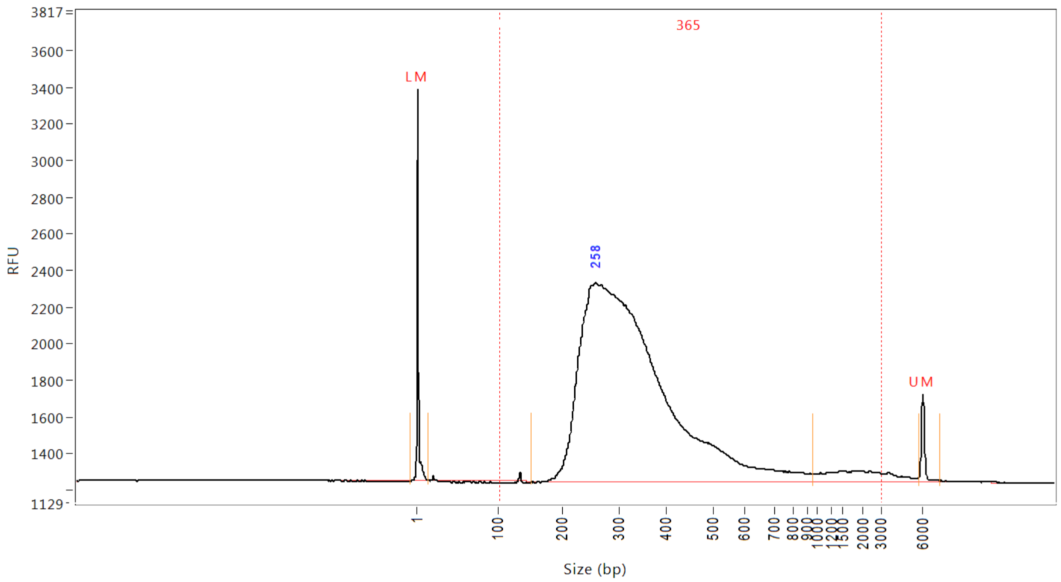

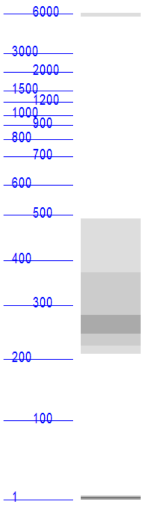

Size (bp)	Molarity (nmole/L)	Conc. (ng/uL)	From (bp)	To (bp)	Avg. Size (bp)	CV%	RFU	Corr. Peak Area
1 (LM)	16.057	0.0123	0	16	1	140.11	2135	12.542
258	13.883	2.1763	150	966	332	32.11	1083	185.098
6000 (UM)	0.001	0.0022	5661	7227	6035	3.69	478	2.196

TIC:	2.1763	ng/uL
TIM:	13.883	nmole/L
Total Conc.:	2.2483	ng/uL

Smear Analysis 100 bp to 3000 bp 2.2305 ng/ul 99.2 %Total 10.058 nmole/L 365 Avg. Size (b.p.) 68.86 %CV

```
Sample Peak Width (sec): 50   Sample Min Peak Height: 25    Sample Baseline V to V?: Y    Sample Baseline V to V pts: 3
Sample Filter: Binomial       # of Pts for Filter: 3        Sample Start Region (min): 0  Sample End Region (min): 50
Manual Baseline Start (min): 10     Manual Baseline End (min): 48
Marker Peak Width (sec): 5    Marker Min Peak Height: 200   Marker Baseline V to V?: Y    Marker Baseline V to V pts: 3
Lower Marker Selection: First Peak > 200 RFU                Upper Marker Selection: Last Peak > 200 RFU
Ladder Size (bp): 1, 100, 200, 300, 400, 500, 600, 700, 800, 900, 1000, 1200, 1500, 2000, 3000, 6000
Quantification Using: Ladder          Final Concentration (ng/uL): 0.0830          Dilution Factor: 12.0
```


Copyright 2015 Advanced Analytical Technologies, Inc.

Data File: 2017 07 25 15H 26M.raw
Sample: RT112+HD5_1
Well Location: B5

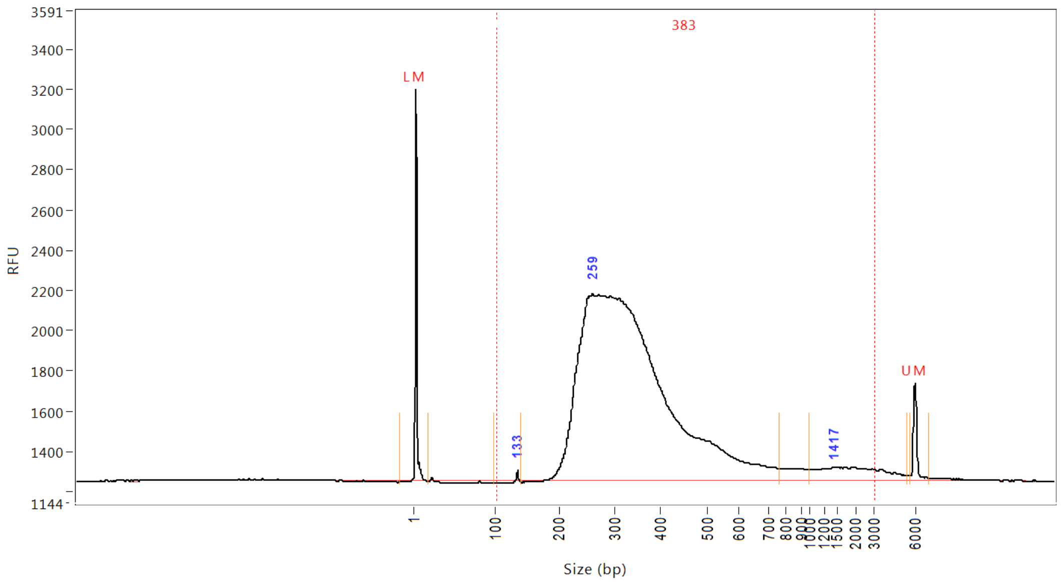

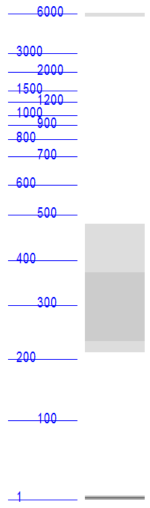

Size (bp)	Molarity (nmole/L)	Conc. (ng/uL)	From (bp)	To (bp)	Avg. Size (bp)	CV%	RFU	Corr. Peak Area
1 (LM)	16.057	0.0123	0	18	1	140.95	1941	11.057
133	0.029	0.0023	97	140	133	0.72	47	0.174
259	14.058	2.2137	140	763	336	29.00	927	165.993
1417	0.102	0.0876	983	5420	2257	50.36	65	6.568
6000 (UM)	0.001	0.0025	5661	6986	6028	3.35	486	2.293

TIC:	2.3036	ng/uL
TIM:	14.188	nmole/L
Total Conc.:	2.3358	ng/uL

Smear Analysis	100 bp to 3000 bp	2.3137 ng/ul	99.1 %Total	9.942 nmole/L	383 Avg. Size (b.p.)	71.95 %CV

```
Sample Peak Width (sec): 50   Sample Min Peak Height: 25    Sample Baseline V to V?: Y    Sample Baseline V to V pts: 3
Sample Filter: Binomial       # of Pts for Filter: 3        Sample Start Region (min): 0  Sample End Region (min): 50
Manual Baseline Start (min): 10     Manual Baseline End (min): 48
Marker Peak Width (sec): 5    Marker Min Peak Height: 200   Marker Baseline V to V?: Y    Marker Baseline V to V pts: 3
Lower Marker Selection: First Peak > 200 RFU                Upper Marker Selection: Last Peak > 200 RFU
Ladder Size (bp): 1, 100, 200, 300, 400, 500, 600, 700, 800, 900, 1000, 1200, 1500, 2000, 3000, 6000
Quantification Using: Ladder          Final Concentration (ng/uL): 0.0830          Dilution Factor: 12.0
```

 Copyright 2015 Advanced Analytical Technologies, Inc.

Data File: 2017 07 25 15H 26M.raw
Sample: RT112+HD5_2
Well Location: B6

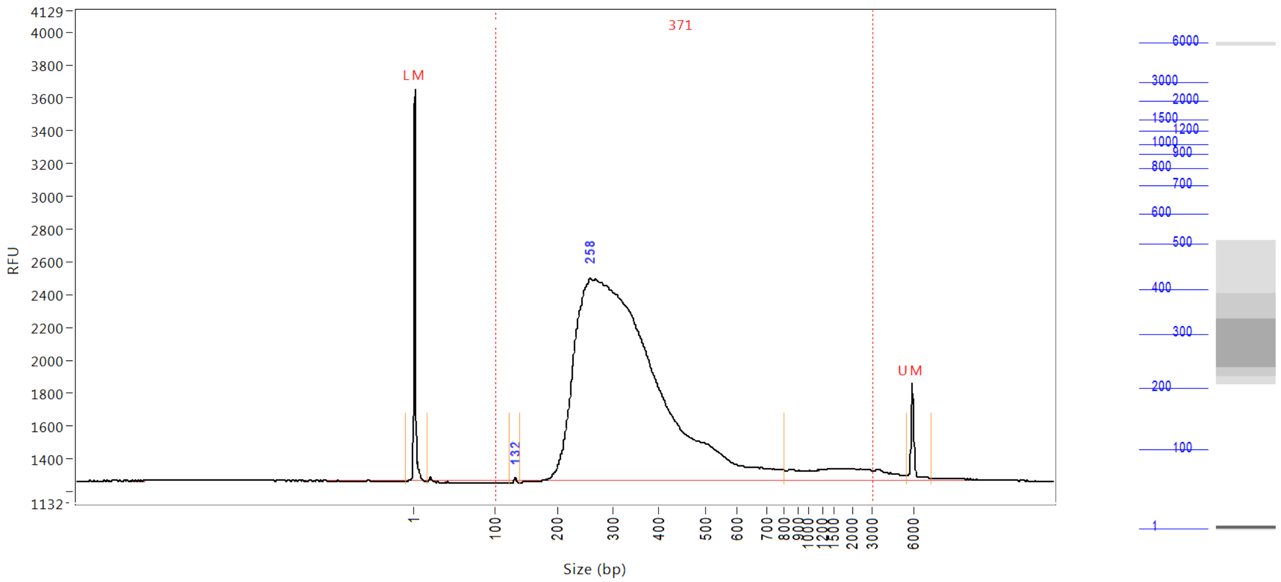

Size (bp)	Molarity (nmole/L)	Conc. (ng/uL)	From (bp)	To (bp)	Avg. Size (bp)	CV%	RFU	Corr. Peak Area
1 (LM)	16.057	0.0123	0	17	1	180.09	2381	13.758
132	0.008	0.0007	123	140	132	0.59	19	0.061
258	14.489	2.2764	140	806	331	29.26	1236	212.394
6000 (UM)	0.001	0.0026	5637	7395	6067	4.94	592	2.920

TIC:	2.2770	ng/uL
TIM:	14.497	nmole/L
Total Conc.:	2.3783	ng/uL

Smear Analysis 100 bp to 3000 bp 2.3565 ng/ul 99.1 %Total 10.441 nmole/L 371 Avg. Size (b.p.) 71.02 %CV

Sample Peak Width (sec): 50 Sample Min Peak Height: 25 Sample Baseline V to V?: Y Sample Baseline V to V pts: 3
Sample Filter: Binomial # of Pts for Filter: 3 Sample Start Region (min): 0 Sample End Region (min): 50
Manual Baseline Start (min): 10 Manual Baseline End (min): 48
Marker Peak Width (sec): 5 Marker Min Peak Height: 200 Marker Baseline V to V?: Y Marker Baseline V to V pts: 3
Lower Marker Selection: First Peak > 200 RFU Upper Marker Selection: Last Peak > 200 RFU
Ladder Size (bp): 1, 100, 200, 300, 400, 500, 600, 700, 800, 900, 1000, 1200, 1500, 2000, 3000, 6000
Quantification Using: Ladder Final Concentration (ng/uL): 0.0830 Dilution Factor: 12.0

 Copyright 2015 Advanced Analytical Technologies, Inc.

Data File: 2017 07 25 15H 26M.raw
Sample: RT112+HD5_3
Well Location: B7

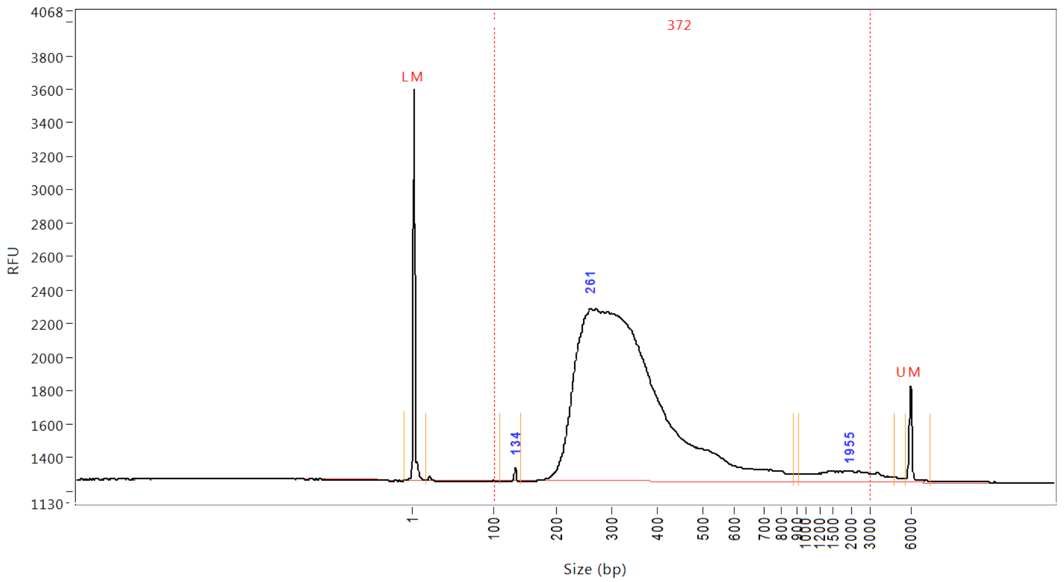

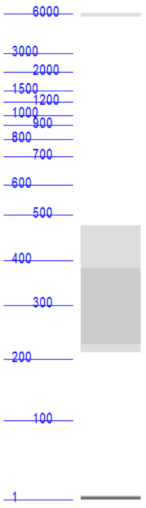

Size (bp)	Molarity (nmole/L)	Conc. (ng/uL)	From (bp)	To (bp)	Avg. Size (bp)	CV%	RFU	Corr. Peak Area
1 (LM)	16.057	0.0123	0	17	1	156.61	2329	13.344
134	0.042	0.0035	110	144	133	0.82	78	0.313
261	12.648	2.0052	144	878	336	30.35	1028	181.463
1955	0.056	0.0671	925	4815	2116	47.29	64	6.071
6000 (UM)	0.001	0.0025	5661	7515	6052	4.40	574	2.688

TIC:	2.0758	ng/uL
TIM:	12.747	nmole/L
Total Conc.:	2.0843	ng/uL

Smear Analysis 100 bp to 3000 bp 2.0662 ng/ul 99.1 %Total 9.128 nmole/L 372 Avg. Size (b.p.) 69.57 %CV

```
Sample Peak Width (sec): 50   Sample Min Peak Height: 25    Sample Baseline V to V?: Y    Sample Baseline V to V pts: 3
Sample Filter: Binomial       # of Pts for Filter: 3        Sample Start Region (min): 0  Sample End Region (min): 50
Manual Baseline Start (min): 10     Manual Baseline End (min): 48
Marker Peak Width (sec): 5    Marker Min Peak Height: 200   Marker Baseline V to V?: Y    Marker Baseline V to V pts: 3
Lower Marker Selection: First Peak > 200 RFU                Upper Marker Selection: Last Peak > 200 RFU
Ladder Size (bp): 1, 100, 200, 300, 400, 500, 600, 700, 800, 900, 1000, 1200, 1500, 2000, 3000, 6000
Quantification Using: Ladder          Final Concentration (ng/uL): 0.0830          Dilution Factor: 12.0
```

 Copyright 2015 Advanced Analytical Technologies, Inc.

Data File: 2017 07 25 15H 26M.raw
Sample: RT112+HD5_4
Well Location: B8

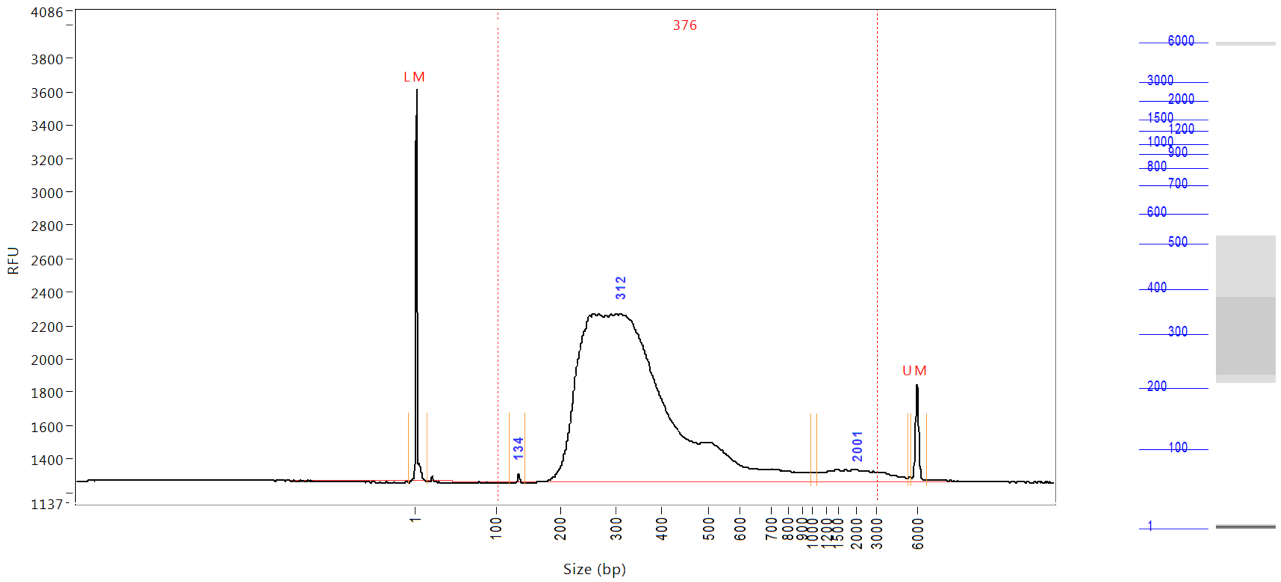

Size (bp)	Molarity (nmole/L)	Conc. (ng/uL)	From (bp)	To (bp)	Avg. Size (bp)	CV%	RFU	Corr. Peak Area
1 (LM)	16.057	0.0123	0	15	1	147.05	2344	13.392
134	0.025	0.0020	120	144	133	0.80	43	0.183
312	10.925	2.0690	144	997	340	33.15	1003	187.901
2001	0.056	0.0684	1074	5347	2346	46.68	72	6.208
6000 (UM)	0.001	0.0024	5613	6722	6002	2.47	586	2.641

TIC:	2.1393	ng/uL
TIM:	11.006	nmole/L
Total Conc.:	2.1456	ng/uL

Smear Analysis 100 bp to 3000 bp 2.1266 ng/ul 99.1 %Total 9.298 nmole/L 376 Avg. Size (b.p.) 70.92 %CV

```
Sample Peak Width (sec): 50   Sample Min Peak Height: 25    Sample Baseline V to V?: Y    Sample Baseline V to V pts: 3
Sample Filter: Binomial       # of Pts for Filter: 3        Sample Start Region (min): 0  Sample End Region (min): 50
Manual Baseline Start (min): 10     Manual Baseline End (min): 48
Marker Peak Width (sec): 5    Marker Min Peak Height: 200   Marker Baseline V to V?: Y    Marker Baseline V to V pts: 3
Lower Marker Selection: First Peak > 200 RFU                Upper Marker Selection: Last Peak > 200 RFU
Ladder Size (bp): 1, 100, 200, 300, 400, 500, 600, 700, 800, 900, 1000, 1200, 1500, 2000, 3000, 6000
Quantification Using: Ladder          Final Concentration (ng/uL): 0.0830          Dilution Factor: 12.0
```

 Copyright 2015 Advanced Analytical Technologies, Inc.

Data File: 2017 07 25 15H 26M.raw
Sample: VM-Cub1+vector1
Well Location: B9

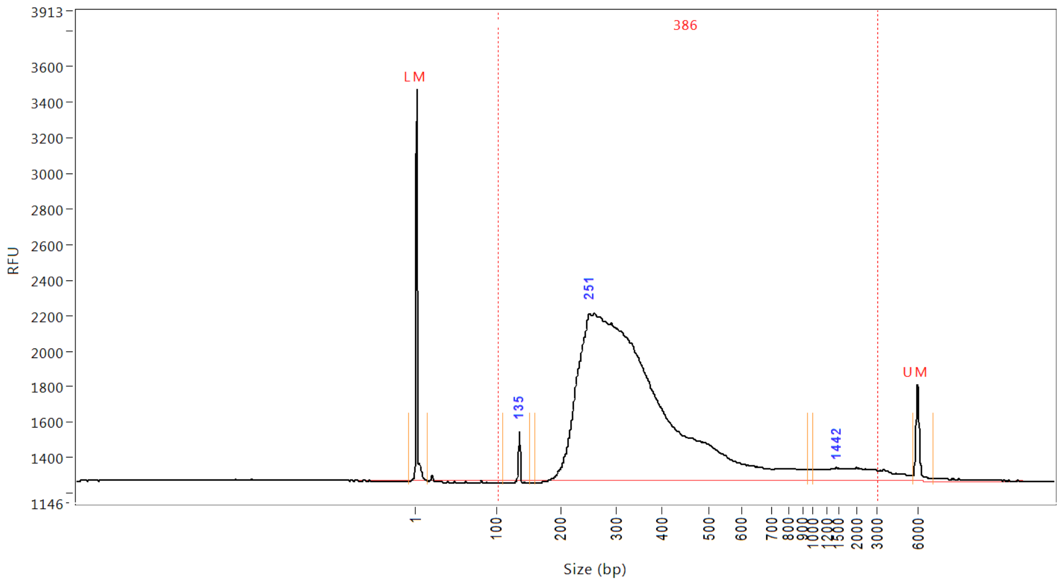

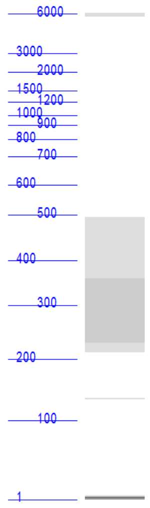

Size (bp)	Molarity (nmole/L)	Conc. (ng/uL)	From (bp)	To (bp)	Avg. Size (bp)	CV%	RFU	Corr. Peak Area
1 (LM)	16.057	0.0123	0	15	1	137.68	2195	12.655
135	0.161	0.0132	108	152	134	0.90	270	1.132
251	12.376	1.8873	161	942	340	33.79	941	161.968
1442	0.096	0.0839	1001	5637	2339	50.93	73	7.202
6000 (UM)	0.001	0.0025	5637	7082	6025	3.81	540	2.598

TIC:	1.9844	ng/uL
TIM:	12.633	nmole/L
Total Conc.:	1.9907	ng/uL

Smear Analysis 100 bp to 3000 bp 1.9686 ng/ul 98.9 %Total 8.400 nmole/L 386 Avg. Size (b.p.) 75.67 %CV

```
Sample Peak Width (sec): 50   Sample Min Peak Height: 25    Sample Baseline V to V?: Y    Sample Baseline V to V pts: 3
Sample Filter: Binomial       # of Pts for Filter: 3        Sample Start Region (min): 0  Sample End Region (min): 50
Manual Baseline Start (min): 10     Manual Baseline End (min): 48
Marker Peak Width (sec): 5    Marker Min Peak Height: 200   Marker Baseline V to V?: Y    Marker Baseline V to V pts: 3
Lower Marker Selection: First Peak > 200 RFU                Upper Marker Selection: Last Peak > 200 RFU
Ladder Size (bp): 1, 100, 200, 300, 400, 500, 600, 700, 800, 900, 1000, 1200, 1500, 2000, 3000, 6000
Quantification Using: Ladder          Final Concentration (ng/uL): 0.0830          Dilution Factor: 12.0
```

 Copyright 2015 Advanced Analytical Technologies, Inc.

Data File: 2017 07 25 15H 26M.raw
Sample: VM-Cub1+vector2
Well Location: B10

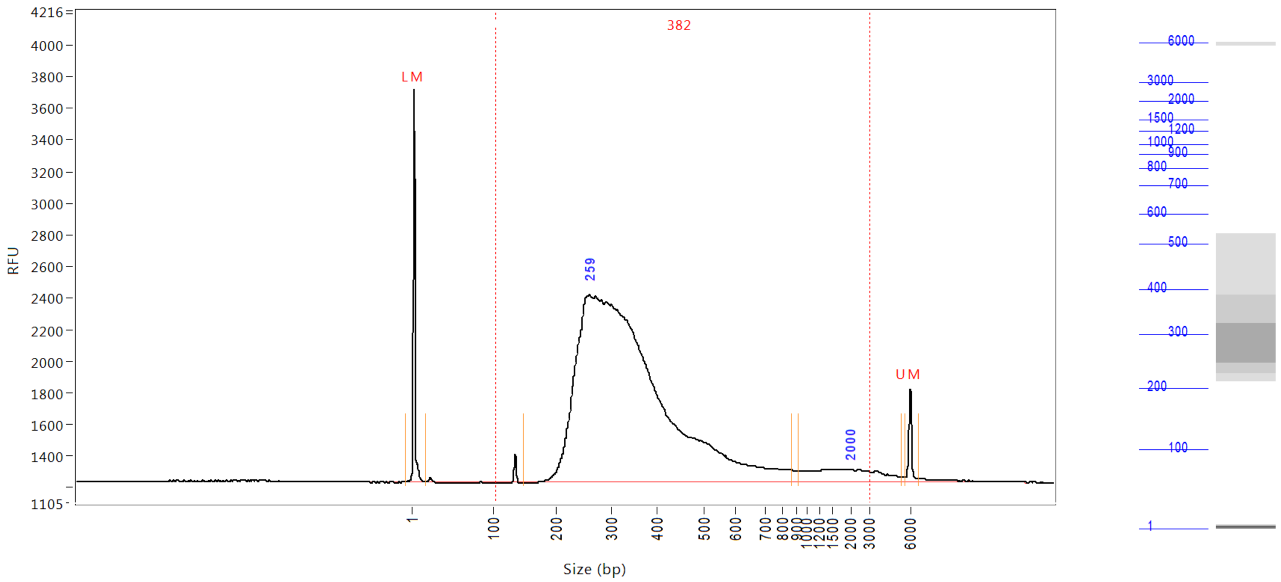

Size (bp)	Molarity (nmole/L)	Conc. (ng/uL)	From (bp)	To (bp)	Avg. Size (bp)	CV%	RFU	Corr. Peak Area
1 (LM)	16.057	0.0123	0	17	1	161.70	2476	14.265
259	13.453	2.1185	147	871	341	31.51	1181	204.939
2000	0.071	0.0862	915	5299	2154	52.10	78	8.336
6000 (UM)	0.001	0.0023	5613	6625	5990	2.52	584	2.708

TIC:	2.2046	ng/uL
TIM:	13.524	nmole/L
Total Conc.:	2.2217	ng/uL

Smear Analysis 100 bp to 3000 bp 2.2003 ng/ul 99.0 %Total 9.476 nmole/L 382 Avg. Size (b.p.) 71.32 %CV

```
Sample Peak Width (sec): 50   Sample Min Peak Height: 25    Sample Baseline V to V?: Y    Sample Baseline V to V pts: 3
Sample Filter: Binomial       # of Pts for Filter: 3        Sample Start Region (min): 0  Sample End Region (min): 50
Manual Baseline Start (min): 10     Manual Baseline End (min): 48
Marker Peak Width (sec): 5    Marker Min Peak Height: 200   Marker Baseline V to V?: Y    Marker Baseline V to V pts: 3
Lower Marker Selection: First Peak > 200 RFU                Upper Marker Selection: Last Peak > 200 RFU
Ladder Size (bp): 1, 100, 200, 300, 400, 500, 600, 700, 800, 900, 1000, 1200, 1500, 2000, 3000, 6000
Quantification Using: Ladder          Final Concentration (ng/uL): 0.0830          Dilution Factor: 12.0
```

 Copyright 2015 Advanced Analytical Technologies, Inc.

Data File: 2017 07 25 15H 26M.raw
Sample: VM-Cub1+vector3
Well Location: B11

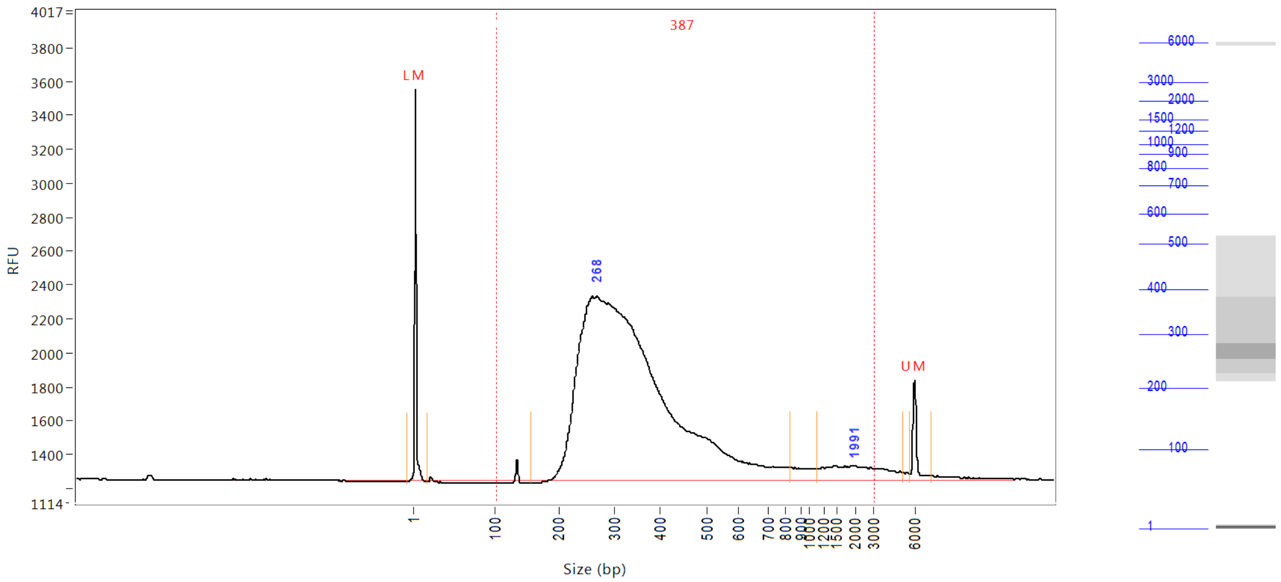

Size (bp)	Molarity (nmole/L)	Conc. (ng/uL)	From (bp)	To (bp)	Avg. Size (bp)	CV%	RFU	Corr. Peak Area
1 (LM)	16.057	0.0123	0	17	1	157.98	2300	13.037
268	13.034	2.1267	156	826	339	30.49	1079	188.017
1991	0.067	0.0816	1113	5129	2385	44.99	82	7.216
6000 (UM)	0.001	0.0027	5637	7203	6062	4.50	587	2.915

TIC:	2.2083	ng/uL
TIM:	13.102	nmole/L
Total Conc.:	2.2473	ng/uL

Smear Analysis 100 bp to 3000 bp 2.2222 ng/ul 98.9 %Total 9.439 nmole/L 387 Avg. Size (b.p.) 73.86 %CV

```
Sample Peak Width (sec): 50   Sample Min Peak Height: 25    Sample Baseline V to V?: Y    Sample Baseline V to V pts: 3
Sample Filter: Binomial       # of Pts for Filter: 3        Sample Start Region (min): 0  Sample End Region (min): 50
Manual Baseline Start (min): 10     Manual Baseline End (min): 48
Marker Peak Width (sec): 5    Marker Min Peak Height: 200   Marker Baseline V to V?: Y    Marker Baseline V to V pts: 3
Lower Marker Selection: First Peak > 200 RFU                Upper Marker Selection: Last Peak > 200 RFU
Ladder Size (bp): 1, 100, 200, 300, 400, 500, 600, 700, 800, 900, 1000, 1200, 1500, 2000, 3000, 6000
Quantification Using: Ladder          Final Concentration (ng/uL): 0.0830          Dilution Factor: 12.0
```

 Copyright 2015 Advanced Analytical Technologies, Inc.

Data File: 2017 07 25 16H 33M.raw
Sample: VM-Cub1+vector4
Well Location: C1

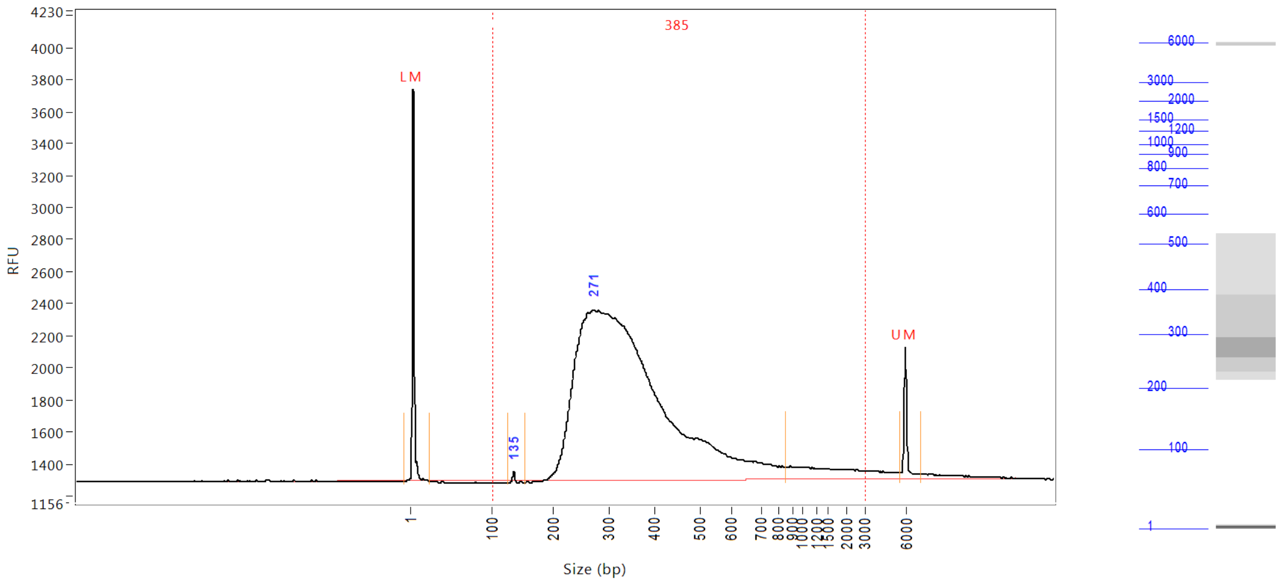

Size (bp)	Molarity (nmole/L)	Conc. (ng/uL)	From (bp)	To (bp)	Avg. Size (bp)	CV%	RFU	Corr. Peak Area
1 (LM)	16.539	0.0127	0	23	1	145.52	2443	14.256
135	0.033	0.0027	127	153	134	0.74	57	0.250
271	12.402	2.0436	153	848	347	31.56	1062	191.810
6000 (UM)	0.001	0.0030	5600	7193	6062	4.60	817	3.412

TIC:	2.0463	ng/uL
TIM:	12.435	nmole/L
Total Conc.:	2.1378	ng/uL

Smear Analysis 100 bp to 3000 bp 2.1191 ng/ul 99.1 %Total 9.053 nmole/L 385 Avg. Size (b.p.) 66.55 %CV

```
Sample Peak Width (sec): 50   Sample Min Peak Height: 25    Sample Baseline V to V?: Y    Sample Baseline V to V pts: 3
Sample Filter: Binomial       # of Pts for Filter: 3        Sample Start Region (min): 0  Sample End Region (min): 50
Manual Baseline Start (min): 10     Manual Baseline End (min): 48
Marker Peak Width (sec): 5    Marker Min Peak Height: 200   Marker Baseline V to V?: Y    Marker Baseline V to V pts: 3
Lower Marker Selection: First Peak > 200 RFU                Upper Marker Selection: Last Peak > 200 RFU
Ladder Size (bp): 1, 100, 200, 300, 400, 500, 600, 700, 800, 900, 1000, 1200, 1500, 2000, 3000, 6000
Quantification Using: Ladder          Final Concentration (ng/uL): 0.0830          Dilution Factor: 12.0
```

 Copyright 2015 Advanced Analytical Technologies, Inc.

Data File: 2017 07 25 16H 33M.raw
Sample: VM-Cub1+HD5_1
Well Location: C2

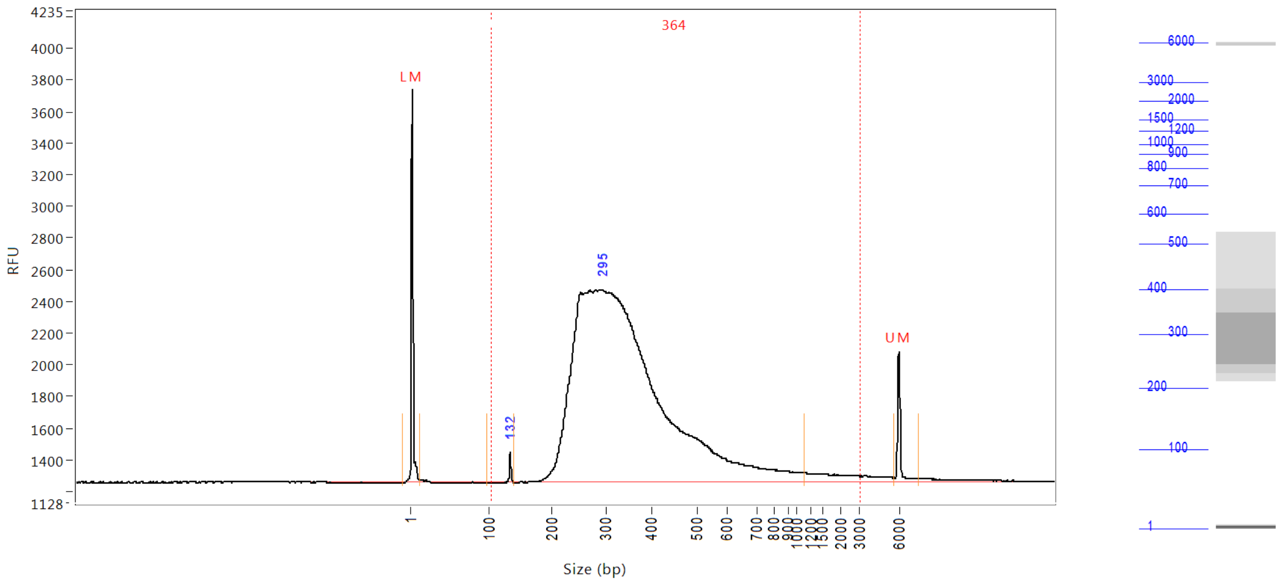

Size (bp)	Molarity (nmole/L)	Conc. (ng/uL)	From (bp)	To (bp)	Avg. Size (bp)	CV%	RFU	Corr. Peak Area
1 (LM)	16.539	0.0127	0	12	1	144.83	2477	14.292
132	0.120	0.0097	96	139	132	1.08	187	0.909
295	13.008	2.3295	139	1098	347	33.69	1207	219.192
6000 (UM)	0.001	0.0028	5625	7466	6055	4.81	820	3.165

TIC:	2.3391	ng/uL
TIM:	13.128	nmole/L
Total Conc.:	2.3831	ng/uL

Smear Analysis	100 bp to 3000 bp	2.3698 ng/ul	99.4 %Total	10.700 nmole/L	364 Avg. Size (b.p.)	56.82 %CV

```
Sample Peak Width (sec): 50   Sample Min Peak Height: 25    Sample Baseline V to V?: Y    Sample Baseline V to V pts: 3
Sample Filter: Binomial       # of Pts for Filter: 3        Sample Start Region (min): 0  Sample End Region (min): 50
Manual Baseline Start (min): 10     Manual Baseline End (min): 48
Marker Peak Width (sec): 5    Marker Min Peak Height: 200   Marker Baseline V to V?: Y    Marker Baseline V to V pts: 3
Lower Marker Selection: First Peak > 200 RFU                Upper Marker Selection: Last Peak > 200 RFU
Ladder Size (bp): 1, 100, 200, 300, 400, 500, 600, 700, 800, 900, 1000, 1200, 1500, 2000, 3000, 6000
Quantification Using: Ladder          Final Concentration (ng/uL): 0.0830          Dilution Factor: 12.0
```

 Copyright 2015 Advanced Analytical Technologies, Inc.

Data File: 2017 07 25 16H 33M.raw
Sample: VM-Cub1+HD5_2
Well Location: C3

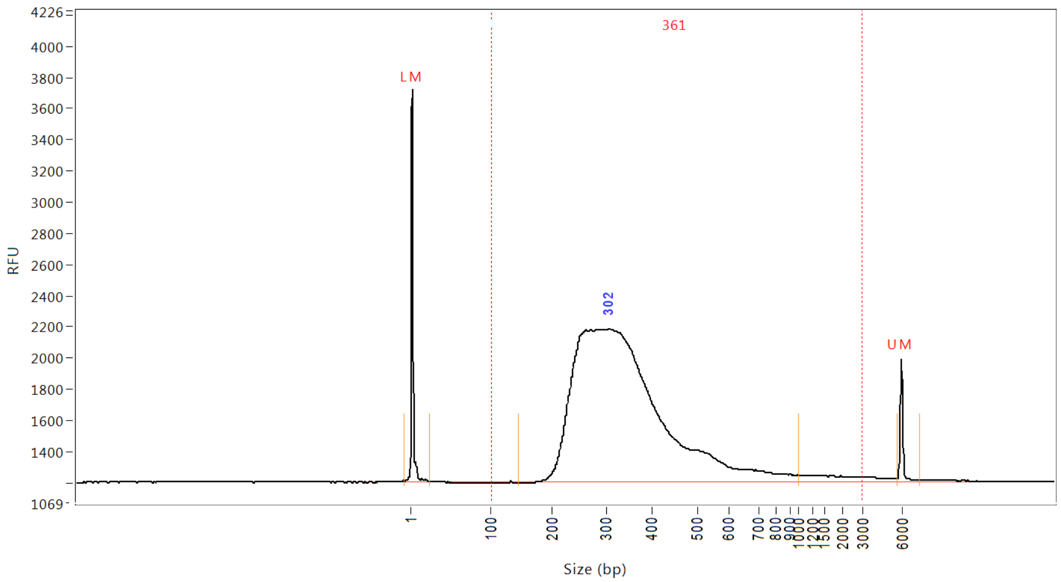

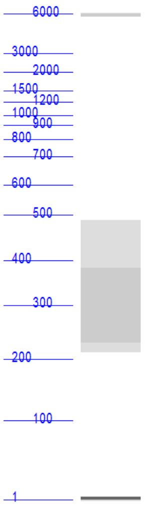

Size (bp)	Molarity (nmole/L)	Conc. (ng/uL)	From (bp)	To (bp)	Avg. Size (bp)	CV%	RFU	Corr. Peak Area
1 (LM)	16.539	0.0127	0	25	1	231.38	2518	15.192
302	9.788	1.7995	144	1001	341	31.99	980	179.991
6000 (UM)	0.001	0.0025	5700	7391	6049	4.12	791	3.008

TIC:	1.7995	ng/uL
TIM:	9.788	nmole/L
Total Conc.:	1.8372	ng/uL

Smear Analysis	100 bp to 3000 bp	1.8273 ng/ul	99.5 %Total	8.319 nmole/L	361 Avg. Size (b.p.)	57.68 %CV

```
Sample Peak Width (sec): 50   Sample Min Peak Height: 25    Sample Baseline V to V?: Y    Sample Baseline V to V pts: 3
Sample Filter: Binomial       # of Pts for Filter: 3        Sample Start Region (min): 0  Sample End Region (min): 50
Manual Baseline Start (min): 10     Manual Baseline End (min): 48
Marker Peak Width (sec): 5    Marker Min Peak Height: 200   Marker Baseline V to V?: Y    Marker Baseline V to V pts: 3
Lower Marker Selection: First Peak > 200 RFU                Upper Marker Selection: Last Peak > 200 RFU
Ladder Size (bp): 1, 100, 200, 300, 400, 500, 600, 700, 800, 900, 1000, 1200, 1500, 2000, 3000, 6000
Quantification Using: Ladder          Final Concentration (ng/uL): 0.0830          Dilution Factor: 12.0
```

 Copyright 2015 Advanced Analytical Technologies, Inc.

Data File: 2017 07 25 16H 33M.raw
Sample: VM-Cub1+HD5_3
Well Location: C4

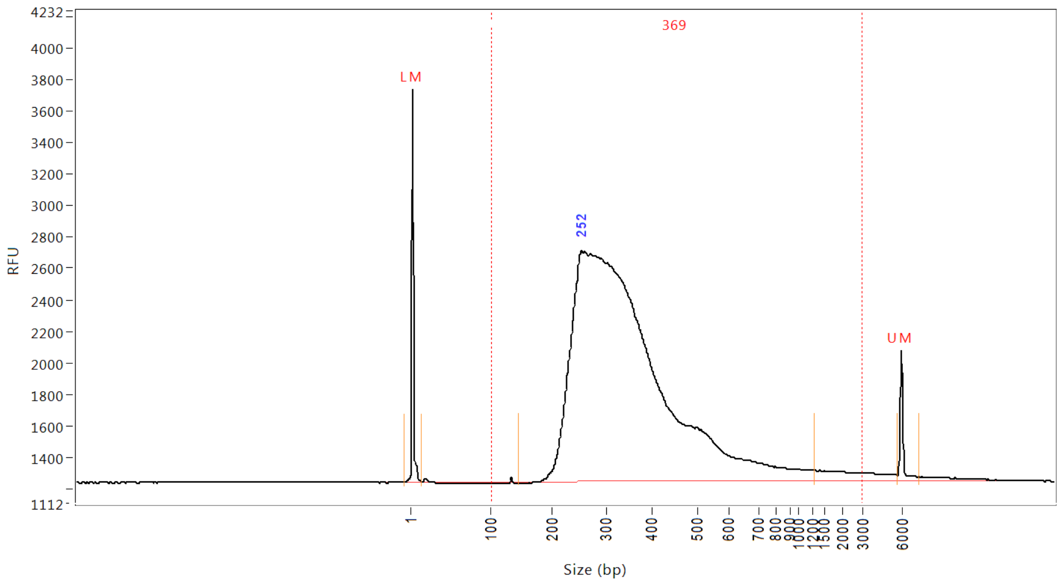

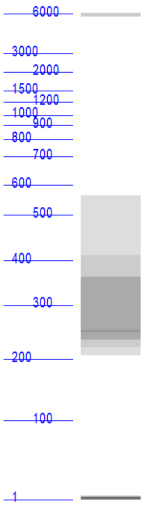

Size (bp)	Molarity (nmole/L)	Conc. (ng/uL)	From (bp)	To (bp)	Avg. Size (bp)	CV%	RFU	Corr. Peak Area
1 (LM)	16.539	0.0127	0	14	1	161.65	2483	14.090
252	18.372	2.8118	145	1227	349	36.59	1458	260.845
6000 (UM)	0.001	0.0030	5700	7267	6080	4.68	825	3.381
	TIC:	2.8118	ng/uL					
	TIM:	18.372	nmole/L					
	Total Conc.:	2.8690	ng/uL					

Smear Analysis	100 bp to 3000 bp	2.8490 ng/ul	99.3 %Total	12.713 nmole/L	369 Avg. Size (b.p.)	60.68 %CV

```
Sample Peak Width (sec): 50   Sample Min Peak Height: 25    Sample Baseline V to V?: Y    Sample Baseline V to V pts: 3
Sample Filter: Binomial       # of Pts for Filter: 3        Sample Start Region (min): 0  Sample End Region (min): 50
Manual Baseline Start (min): 10     Manual Baseline End (min): 48
Marker Peak Width (sec): 5    Marker Min Peak Height: 200   Marker Baseline V to V?: Y    Marker Baseline V to V pts: 3
Lower Marker Selection: First Peak > 200 RFU                Upper Marker Selection: Last Peak > 200 RFU
Ladder Size (bp): 1, 100, 200, 300, 400, 500, 600, 700, 800, 900, 1000, 1200, 1500, 2000, 3000, 6000
Quantification Using: Ladder          Final Concentration (ng/uL): 0.0830          Dilution Factor: 12.0
```

 Copyright 2015 Advanced Analytical Technologies, Inc.

Data File: 2017 07 25 16H 33M.raw
Sample: VM-Cub1+HD5_4
Well Location: C5

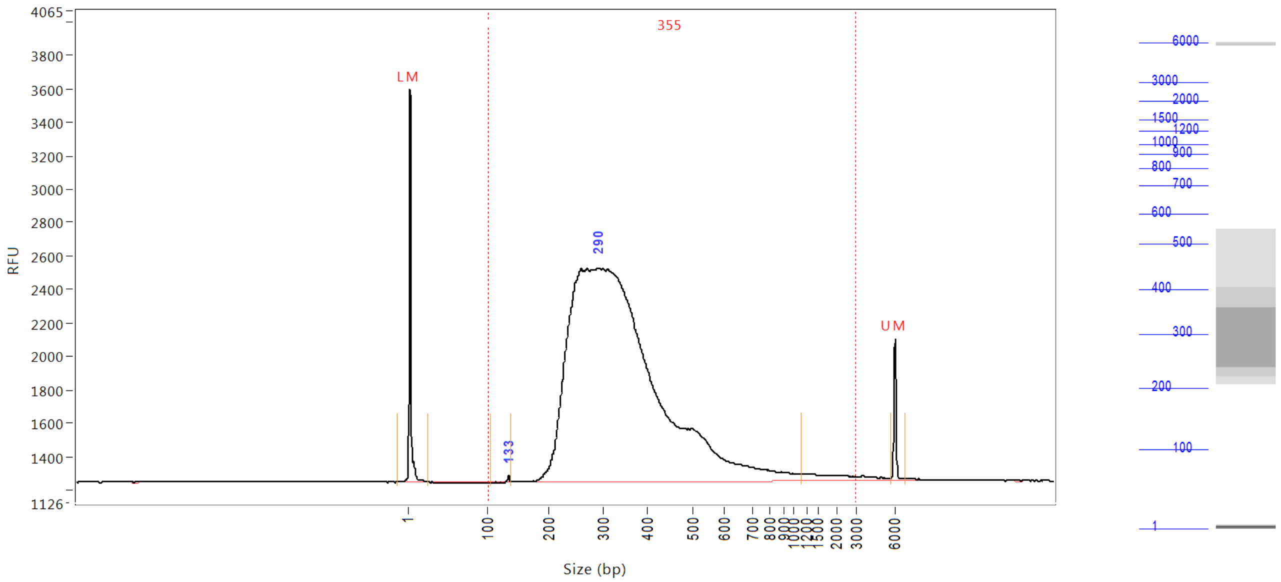

Size (bp)	Molarity (nmole/L)	Conc. (ng/uL)	From (bp)	To (bp)	Avg. Size (bp)	CV%	RFU	Corr. Peak Area
1 (LM)	16.539	0.0127	0	26	1	155.90	2339	13.674
133	0.017	0.0014	105	138	132	0.72	34	0.125
290	14.844	2.6201	138	1122	342	32.72	1269	235.884
6000 (UM)	0.001	0.0028	5700	6770	5997	2.17	847	3.060

TIC:	2.6215	ng/uL
TIM:	14.861	nmole/L
Total Conc.:	2.6558	ng/uL

Smear Analysis	100 bp to 3000 bp	2.6461 ng/ul	99.6 %Total	12.249 nmole/L	355 Avg. Size (b.p.)	52.04 %CV

Sample Peak Width (sec): 50   Sample Min Peak Height: 25   Sample Baseline V to V?: Y   Sample Baseline V to V pts: 3
Sample Filter: Binomial   # of Pts for Filter: 3   Sample Start Region (min): 0   Sample End Region (min): 50
Manual Baseline Start (min): 10   Manual Baseline End (min): 48
Marker Peak Width (sec): 5   Marker Min Peak Height: 200   Marker Baseline V to V?: Y   Marker Baseline V to V pts: 3
Lower Marker Selection: First Peak > 200 RFU   Upper Marker Selection: Last Peak > 200 RFU
Ladder Size (bp): 1, 100, 200, 300, 400, 500, 600, 700, 800, 900, 1000, 1200, 1500, 2000, 3000, 6000
Quantification Using: Ladder   Final Concentration (ng/uL): 0.0830   Dilution Factor: 12.0

 Copyright 2015 Advanced Analytical Technologies, Inc.

Data File: 2017 07 25 16H 33M.raw
Sample: SW1710+vector1
Well Location: C6

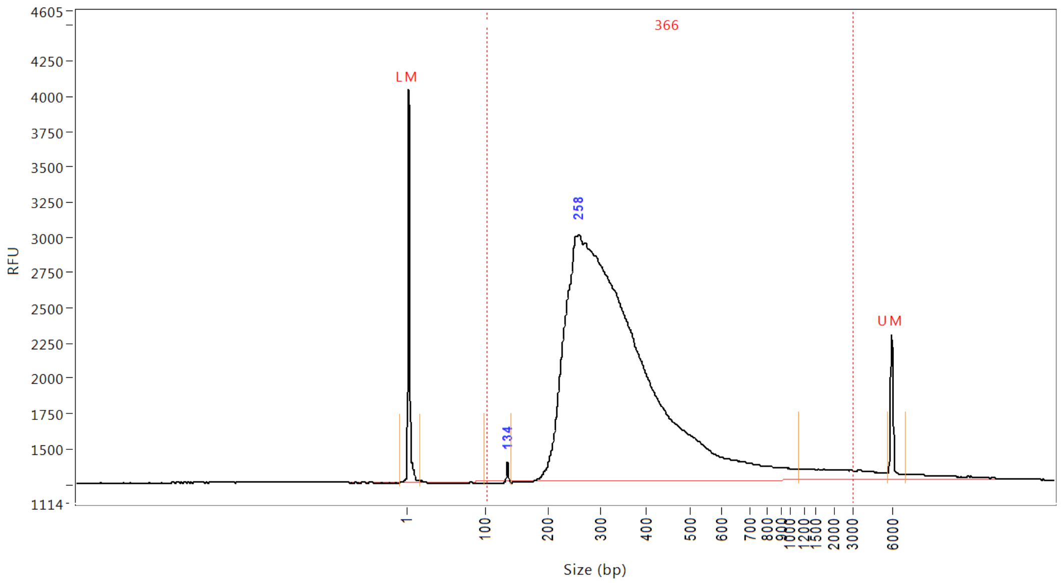

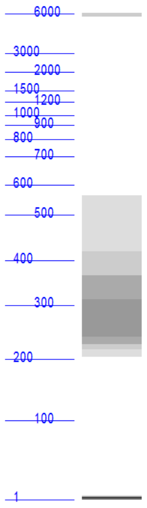

Size (bp)	Molarity (nmole/L)	Conc. (ng/uL)	From (bp)	To (bp)	Avg. Size (bp)	CV%	RFU	Corr. Peak Area
1 (LM)	16.539	0.0127	0	17	1	156.04	2778	15.861
134	0.066	0.0054	97	140	133	0.88	131	0.564
258	17.618	2.7675	140	1117	342	35.35	1747	289.004
6000 (UM)	0.001	0.0034	5625	7118	6052	4.24	1027	4.280

TIC:	2.7729	ng/uL
TIM:	17.685	nmole/L
Total Conc.:	2.8406	ng/uL

Smear Analysis	100 bp to 3000 bp	2.8194 ng/ul	99.3 %Total	12.686 nmole/L	366 Avg. Size (b.p.)	63.86 %CV

```
Sample Peak Width (sec): 50   Sample Min Peak Height: 25    Sample Baseline V to V?: Y    Sample Baseline V to V pts: 3
Sample Filter: Binomial       # of Pts for Filter: 3        Sample Start Region (min): 0  Sample End Region (min): 50
Manual Baseline Start (min): 10     Manual Baseline End (min): 48
Marker Peak Width (sec): 5    Marker Min Peak Height: 200   Marker Baseline V to V?: Y    Marker Baseline V to V pts: 3
Lower Marker Selection: First Peak > 200 RFU                Upper Marker Selection: Last Peak > 200 RFU
Ladder Size (bp): 1, 100, 200, 300, 400, 500, 600, 700, 800, 900, 1000, 1200, 1500, 2000, 3000, 6000
Quantification Using: Ladder          Final Concentration (ng/uL): 0.0830          Dilution Factor: 12.0
```

 Copyright 2015 Advanced Analytical Technologies, Inc.

Data File: 2017 07 25 16H 33M.raw
Sample: SW1710+vector2
Well Location: C7

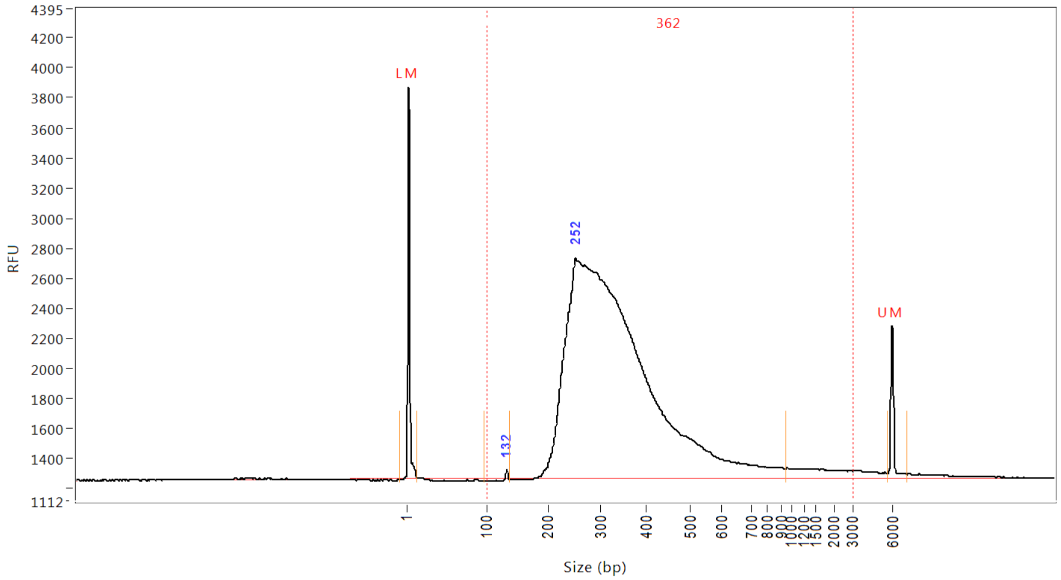

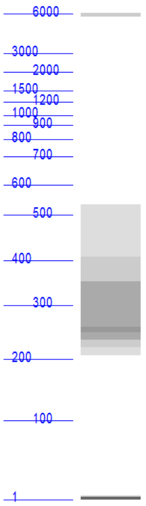

Size (bp)	Molarity (nmole/L)	Conc. (ng/uL)	From (bp)	To (bp)	Avg. Size (bp)	CV%	RFU	Corr. Peak Area
1 (LM)	16.539	0.0127	0	13	1	166.61	2609	15.246
132	0.035	0.0028	96	139	131	0.88	62	0.284
252	15.962	2.4429	139	947	337	31.76	1466	245.220
6000 (UM)	0.001	0.0034	5675	7143	6044	3.93	1015	4.048

TIC:	2.4457	ng/uL
TIM:	15.997	nmole/L
Total Conc.:	2.5112	ng/uL

Smear Analysis	100 bp to 3000 bp	2.4937 ng/ul	99.3 %Total	11.347 nmole/L	362 Avg. Size (b.p.)	61.43 %CV

```
Sample Peak Width (sec): 50   Sample Min Peak Height: 25    Sample Baseline V to V?: Y    Sample Baseline V to V pts: 3
Sample Filter: Binomial       # of Pts for Filter: 3        Sample Start Region (min): 0  Sample End Region (min): 50
Manual Baseline Start (min): 10     Manual Baseline End (min): 48
Marker Peak Width (sec): 5    Marker Min Peak Height: 200   Marker Baseline V to V?: Y    Marker Baseline V to V pts: 3
Lower Marker Selection: First Peak > 200 RFU                Upper Marker Selection: Last Peak > 200 RFU
Ladder Size (bp): 1, 100, 200, 300, 400, 500, 600, 700, 800, 900, 1000, 1200, 1500, 2000, 3000, 6000
Quantification Using: Ladder          Final Concentration (ng/uL): 0.0830          Dilution Factor: 12.0
```

 Copyright 2015 Advanced Analytical Technologies, Inc.

Data File: 2017 07 25 16H 33M.raw
Sample: SW1710+vector3
Well Location: C8

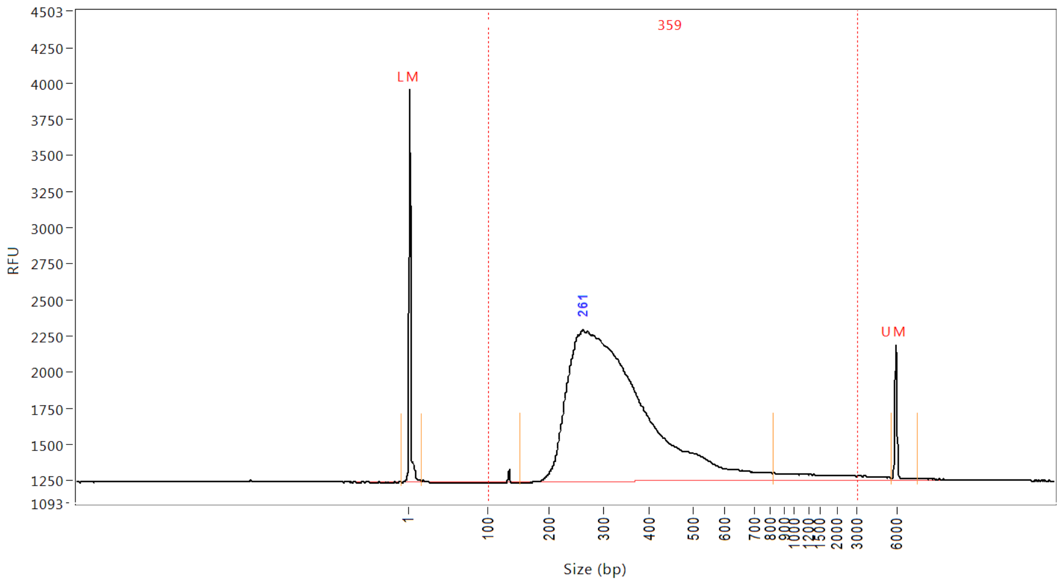

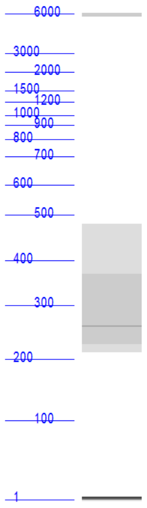

Size (bp)	Molarity (nmole/L)	Conc. (ng/uL)	From (bp)	To (bp)	Avg. Size (bp)	CV%	RFU	Corr. Peak Area
1 (LM)	16.539	0.0127	0	16	1	185.03	2712	15.509
261	10.566	1.6751	152	818	332	29.48	1041	171.052
6000 (UM)	0.001	0.0030	5675	7665	6048	4.93	940	3.624

TIC:	1.6751	ng/uL
TIM:	10.566	nmole/L
Total Conc.:	1.7306	ng/uL

Smear Analysis 100 bp to 3000 bp 1.7202 ng/ul 99.4 %Total 7.887 nmole/L 359 Avg. Size (b.p.) 60.84 %CV

```
Sample Peak Width (sec): 50   Sample Min Peak Height: 25    Sample Baseline V to V?: Y    Sample Baseline V to V pts: 3
Sample Filter: Binomial       # of Pts for Filter: 3        Sample Start Region (min): 0  Sample End Region (min): 50
Manual Baseline Start (min): 10     Manual Baseline End (min): 48
Marker Peak Width (sec): 5    Marker Min Peak Height: 200   Marker Baseline V to V?: Y    Marker Baseline V to V pts: 3
Lower Marker Selection: First Peak > 200 RFU                Upper Marker Selection: Last Peak > 200 RFU
Ladder Size (bp): 1, 100, 200, 300, 400, 500, 600, 700, 800, 900, 1000, 1200, 1500, 2000, 3000, 6000
Quantification Using: Ladder          Final Concentration (ng/uL): 0.0830          Dilution Factor: 12.0
```

 Copyright 2015 Advanced Analytical Technologies, Inc.

Data File: 2017 07 25 16H 33M.raw
Sample: SW1710+vector4
Well Location: C9

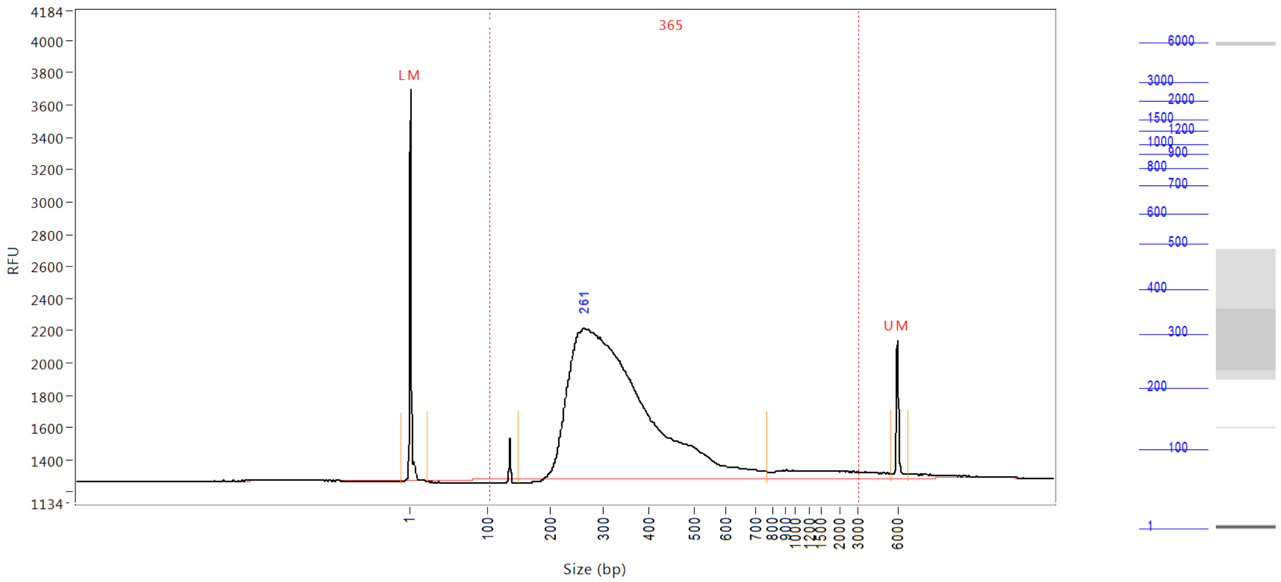

Size (bp)	Molarity (nmole/L)	Conc. (ng/uL)	From (bp)	To (bp)	Avg. Size (bp)	CV%	RFU	Corr. Peak Area
1 (LM)	16.539	0.0127	0	23	1	138.75	2418	13.710
261	10.633	1.6897	148	768	332	28.60	938	152.519
6000 (UM)	0.001	0.0031	5575	6820	6015	2.92	855	3.365
	TIC:	1.6897	ng/uL					
	TIM:	10.633	nmole/L					
	Total Conc.:	1.7743	ng/uL					

Smear Analysis 100 bp to 3000 bp 1.7596 ng/ul 99.2 %Total 7.923 nmole/L 365 Avg. Size (b.p.) 66.22 %CV

Sample Peak Width (sec): 50 Sample Min Peak Height: 25 Sample Baseline V to V?: Y Sample Baseline V to V pts: 3
Sample Filter: Binomial # of Pts for Filter: 3 Sample Start Region (min): 0 Sample End Region (min): 50
Manual Baseline Start (min): 10 Manual Baseline End (min): 48
Marker Peak Width (sec): 5 Marker Min Peak Height: 200 Marker Baseline V to V?: Y Marker Baseline V to V pts: 3
Lower Marker Selection: First Peak > 200 RFU Upper Marker Selection: Last Peak > 200 RFU
Ladder Size (bp): 1, 100, 200, 300, 400, 500, 600, 700, 800, 900, 1000, 1200, 1500, 2000, 3000, 6000
Quantification Using: Ladder Final Concentration (ng/uL): 0.0830 Dilution Factor: 12.0

 Copyright 2015 Advanced Analytical Technologies, Inc.

Data File: 2017 07 25 16H 33M.raw
Sample: SW1710+HD5_1
Well Location: C10

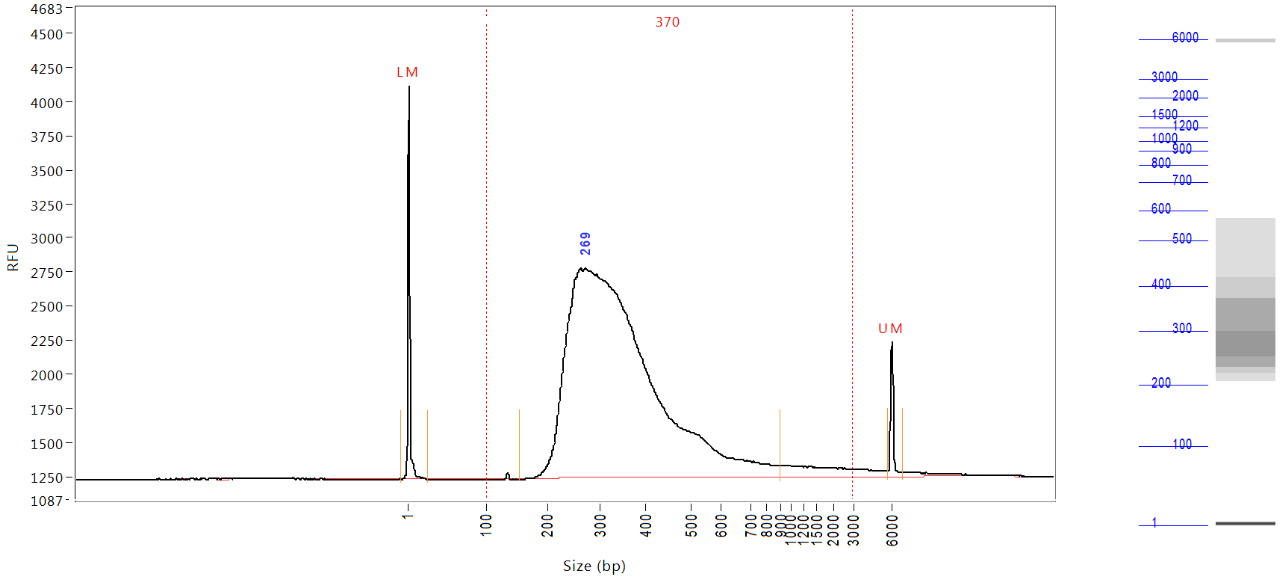

Size (bp)	Molarity (nmole/L)	Conc. (ng/uL)	From (bp)	To (bp)	Avg. Size (bp)	CV%	RFU	Corr. Peak Area
1 (LM)	16.539	0.0127	0	28	1	153.63	2864	16.676
269	15.191	2.4865	154	893	343	31.38	1526	273.005
6000 (UM)	0.001	0.0030	5700	6845	6026	2.97	986	3.913

TIC:	2.4865	ng/uL
TIM:	15.191	nmole/L
Total Conc.:	2.5633	ng/uL

Smear Analysis 100 bp to 3000 bp 2.5457 ng/ul 99.3 %Total 11.319 nmole/L 370 Avg. Size (b.p.) 61.31 %CV

Sample Peak Width (sec): 50 Sample Min Peak Height: 25 Sample Baseline V to V?: Y Sample Baseline V to V pts: 3
Sample Filter: Binomial # of Pts for Filter: 3 Sample Start Region (min): 0 Sample End Region (min): 50
Manual Baseline Start (min): 10 Manual Baseline End (min): 48
Marker Peak Width (sec): 5 Marker Min Peak Height: 200 Marker Baseline V to V?: Y Marker Baseline V to V pts: 3
Lower Marker Selection: First Peak > 200 RFU Upper Marker Selection: Last Peak > 200 RFU
Ladder Size (bp): 1, 100, 200, 300, 400, 500, 600, 700, 800, 900, 1000, 1200, 1500, 2000, 3000, 6000
Quantification Using: Ladder Final Concentration (ng/uL): 0.0830 Dilution Factor: 12.0

 Copyright 2015 Advanced Analytical Technologies, Inc.

Data File: 2017 07 25 16H 33M.raw
Sample: SW1710+HD5_2
Well Location: C11

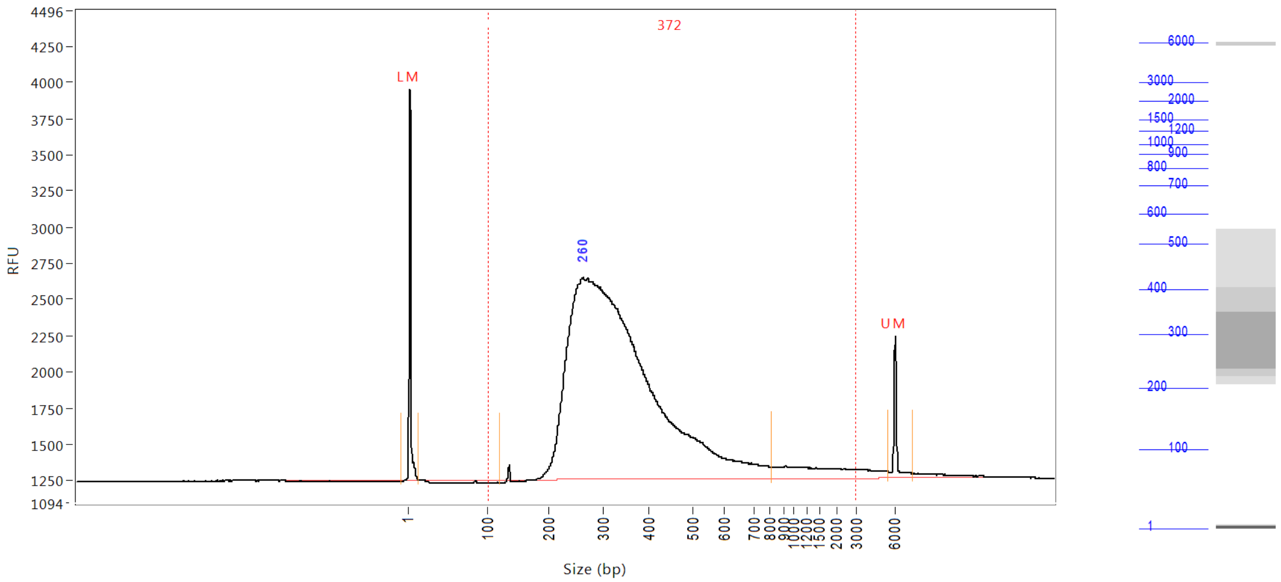

Size (bp)	Molarity (nmole/L)	Conc. (ng/uL)	From (bp)	To (bp)	Avg. Size (bp)	CV%	RFU	Corr. Peak Area
1 (LM)	16.539	0.0127	0	13	1	156.18	2699	15.199
260	15.252	2.4126	118	806	337	30.21	1397	241.429
6000 (UM)	0.001	0.0036	5525	7366	6068	5.28	984	4.265

TIC:	2.4126	ng/uL
TIM:	15.252	nmole/L
Total Conc.:	2.5133	ng/uL

Smear Analysis	100 bp to 3000 bp	2.4931 ng/ul	99.2 %Total	11.017 nmole/L	372 Avg. Size (b.p.)	65.67 %CV

```
Sample Peak Width (sec): 50   Sample Min Peak Height: 25    Sample Baseline V to V?: Y    Sample Baseline V to V pts: 3
Sample Filter: Binomial       # of Pts for Filter: 3        Sample Start Region (min): 0  Sample End Region (min): 50
Manual Baseline Start (min): 10     Manual Baseline End (min): 48
Marker Peak Width (sec): 5    Marker Min Peak Height: 200   Marker Baseline V to V?: Y    Marker Baseline V to V pts: 3
Lower Marker Selection: First Peak > 200 RFU                Upper Marker Selection: Last Peak > 200 RFU
Ladder Size (bp): 1, 100, 200, 300, 400, 500, 600, 700, 800, 900, 1000, 1200, 1500, 2000, 3000, 6000
Quantification Using: Ladder          Final Concentration (ng/uL): 0.0830          Dilution Factor: 12.0
```

 Copyright 2015 Advanced Analytical Technologies, Inc.

Data File: 2017 07 25 17H 40M.raw
Sample: SW1710+HD5_3
Well Location: D1

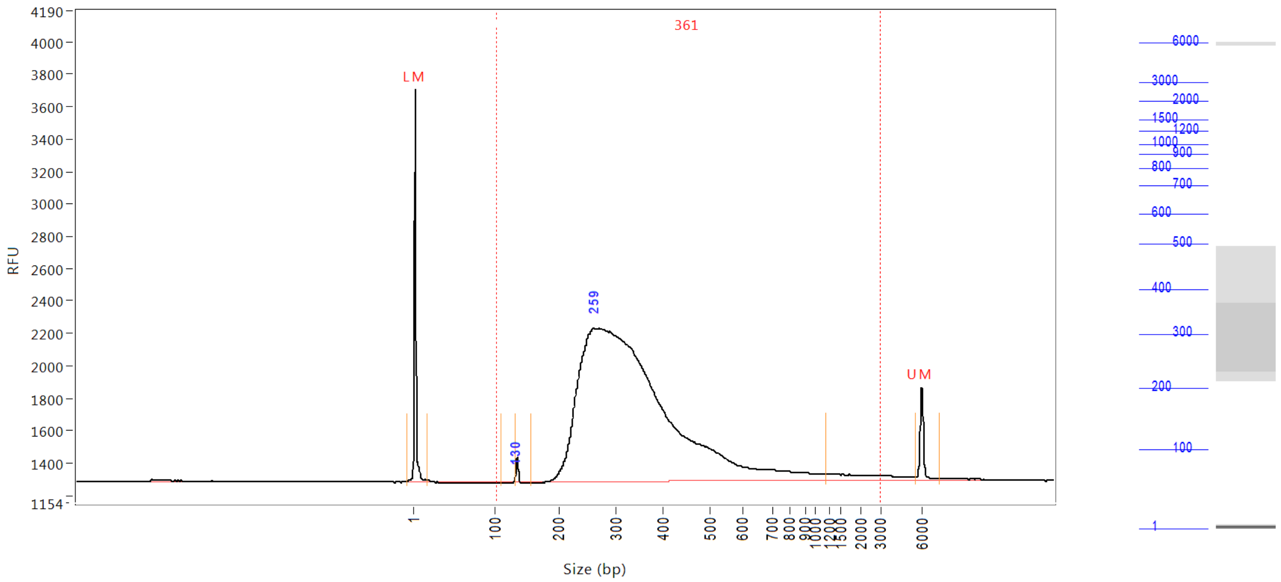

Size (bp)	Molarity (nmole/L)	Conc. (ng/uL)	From (bp)	To (bp)	Avg. Size (bp)	CV%	RFU	Corr. Peak Area
1 (LM)	18.824	0.0144	0	16	1	181.02	2417	13.955
130	0.010	0.0008	109	130	130	0.46	23	0.064
259	13.294	2.0954	156	1153	343	34.75	946	169.151
6000 (UM)	0.001	0.0029	5572	7326	6040	4.23	576	2.802

TIC:	2.0962	ng/uL
TIM:	13.304	nmole/L
Total Conc.:	2.1450	ng/uL

Smear Analysis 100 bp to 3000 bp 2.1324 ng/ul 99.4 %Total 9.722 nmole/L 361 Avg. Size (b.p.) 59.12 %CV

```
Sample Peak Width (sec): 50   Sample Min Peak Height: 25    Sample Baseline V to V?: Y    Sample Baseline V to V pts: 3
Sample Filter: Binomial       # of Pts for Filter: 3        Sample Start Region (min): 0  Sample End Region (min): 50
Manual Baseline Start (min): 10     Manual Baseline End (min): 48
Marker Peak Width (sec): 5    Marker Min Peak Height: 200   Marker Baseline V to V?: Y    Marker Baseline V to V pts: 3
Lower Marker Selection: First Peak > 200 RFU                Upper Marker Selection: Last Peak > 200 RFU
Ladder Size (bp): 1, 100, 200, 300, 400, 500, 600, 700, 800, 900, 1000, 1200, 1500, 2000, 3000, 6000
Quantification Using: Ladder          Final Concentration (ng/uL): 0.0830          Dilution Factor: 12.0
```

 Copyright 2015 Advanced Analytical Technologies, Inc.

Data File: 2017 07 25 17H 40M.raw
Sample: SW1710+HD5_4
Well Location: D2

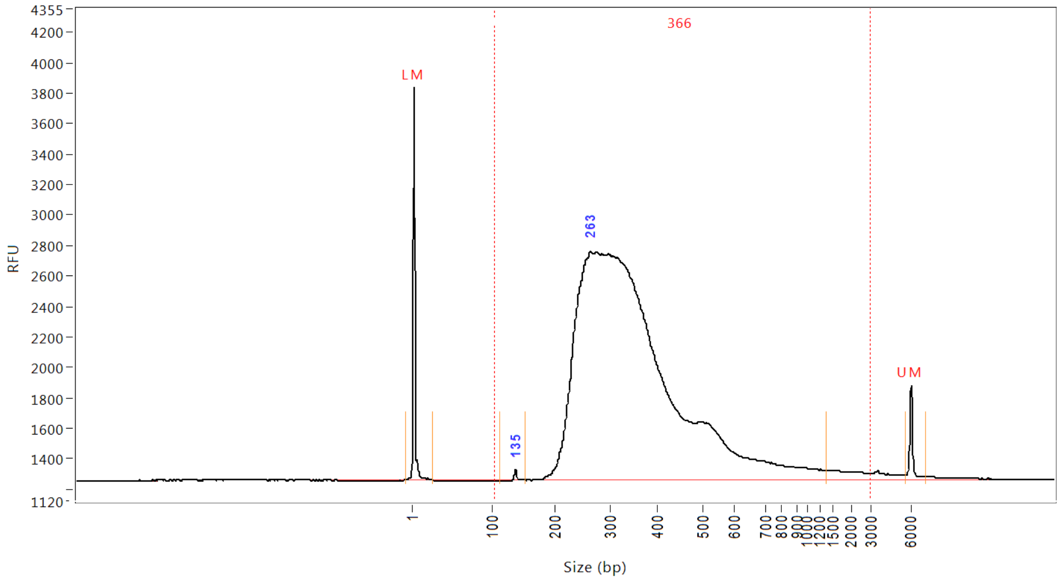

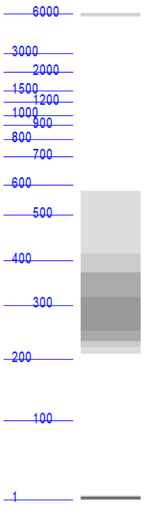

Size (bp)	Molarity (nmole/L)	Conc. (ng/uL)	From (bp)	To (bp)	Avg. Size (bp)	CV%	RFU	Corr. Peak Area
1 (LM)	18.824	0.0144	0	26	1	184.20	2579	15.062
135	0.048	0.0039	110	153	134	0.97	68	0.341
263	20.177	3.2231	153	1326	351	36.94	1498	280.817
6000 (UM)	0.001	0.0029	5619	7113	6030	3.95	623	3.009

TIC:	3.2270	ng/uL
TIM:	20.225	nmole/L
Total Conc.:	3.2766	ng/uL

Smear Analysis | 100 bp to 3000 bp | 3.2593 ng/ul | 99.5 %Total | 14.631 nmole/L | 366 Avg. Size (b.p.) | 57.45 %CV

```
Sample Peak Width (sec): 50   Sample Min Peak Height: 25    Sample Baseline V to V?: Y    Sample Baseline V to V pts: 3
Sample Filter: Binomial       # of Pts for Filter: 3        Sample Start Region (min): 0  Sample End Region (min): 50
Manual Baseline Start (min): 10     Manual Baseline End (min): 48
Marker Peak Width (sec): 5    Marker Min Peak Height: 200   Marker Baseline V to V?: Y    Marker Baseline V to V pts: 3
Lower Marker Selection: First Peak > 200 RFU                Upper Marker Selection: Last Peak > 200 RFU
Ladder Size (bp): 1, 100, 200, 300, 400, 500, 600, 700, 800, 900, 1000, 1200, 1500, 2000, 3000, 6000
Quantification Using: Ladder          Final Concentration (ng/uL): 0.0830          Dilution Factor: 12.0
```

 Copyright 2015 Advanced Analytical Technologies, Inc.

Data File: 2017 07 25 17H 40M.raw
Sample: UM-UC-3+vector1
Well Location: D3

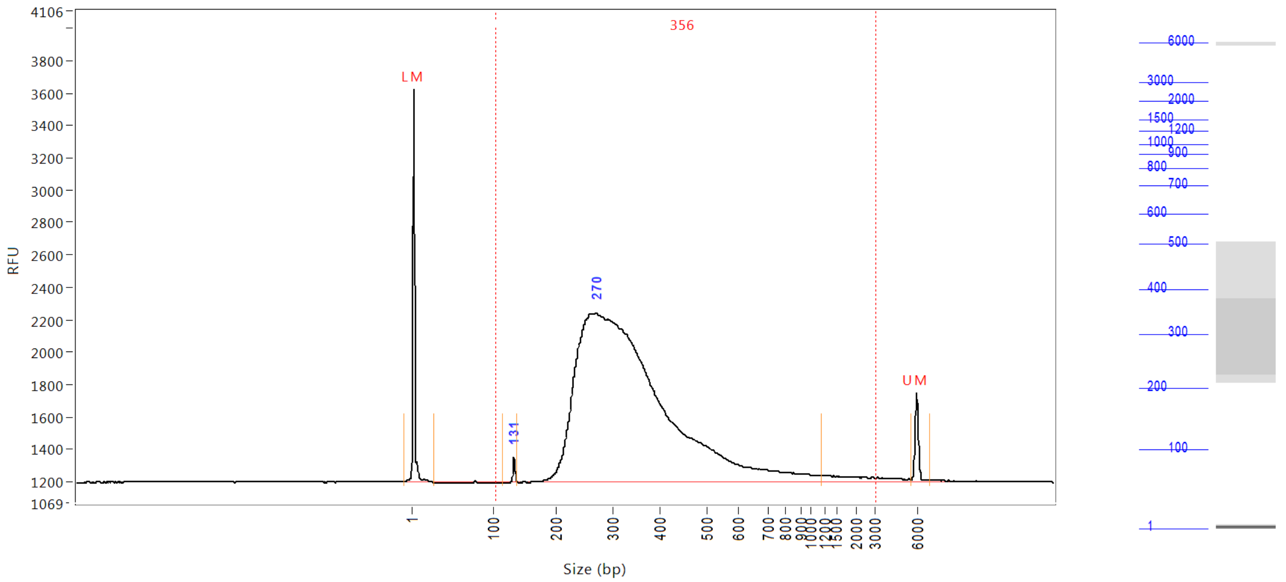

Size (bp)	Molarity (nmole/L)	Conc. (ng/uL)	From (bp)	To (bp)	Avg. Size (bp)	CV%	RFU	Corr. Peak Area
1 (LM)	18.824	0.0144	0	27	1	199.25	2421	14.248
131	0.116	0.0093	113	137	131	1.10	149	0.766
270	13.901	2.2796	137	1148	341	34.45	1039	187.881
6000 (UM)	0.001	0.0026	5643	6994	6015	2.88	546	2.529

TIC:	2.2888	ng/uL
TIM:	14.017	nmole/L
Total Conc.:	2.3228	ng/uL

Smear Analysis 100 bp to 3000 bp 2.3129 ng/ul 99.6 %Total 10.695 nmole/L 356 Avg. Size (b.p.) 55.29 %CV

```
Sample Peak Width (sec): 50   Sample Min Peak Height: 25    Sample Baseline V to V?: Y    Sample Baseline V to V pts: 3
Sample Filter: Binomial       # of Pts for Filter: 3        Sample Start Region (min): 0  Sample End Region (min): 50
Manual Baseline Start (min): 10     Manual Baseline End (min): 48
Marker Peak Width (sec): 5    Marker Min Peak Height: 200   Marker Baseline V to V?: Y    Marker Baseline V to V pts: 3
Lower Marker Selection: First Peak > 200 RFU                Upper Marker Selection: Last Peak > 200 RFU
Ladder Size (bp): 1, 100, 200, 300, 400, 500, 600, 700, 800, 900, 1000, 1200, 1500, 2000, 3000, 6000
Quantification Using: Ladder          Final Concentration (ng/uL): 0.0830          Dilution Factor: 12.0
```

 Copyright 2015 Advanced Analytical Technologies, Inc.

Data File: 2017 07 25 17H 40M.raw
Sample: UM-UC-3+vector2
Well Location: D4

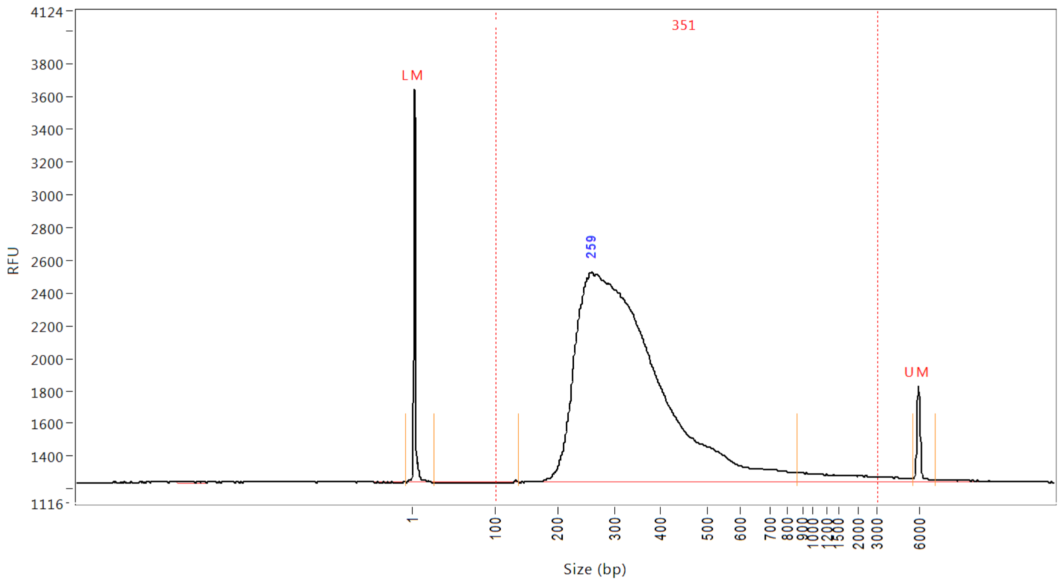

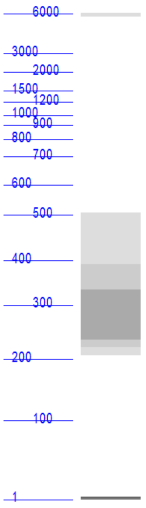

Size (bp)	Molarity (nmole/L)	Conc. (ng/uL)	From (bp)	To (bp)	Avg. Size (bp)	CV%	RFU	Corr. Peak Area
1 (LM)	18.824	0.0144	0	26	1	203.03	2399	14.053
259	17.230	2.7157	138	863	330	30.13	1281	220.772
6000 (UM)	0.001	0.0028	5619	7208	6020	3.86	583	2.779

TIC:	2.7157	ng/uL
TIM:	17.230	nmole/L
Total Conc.:	2.7794	ng/uL

Smear Analysis	100 bp to 3000 bp	2.7666 ng/ul	99.5 %Total	12.964 nmole/L	351 Avg. Size (b.p.)	56.48 %CV

```
Sample Peak Width (sec): 50   Sample Min Peak Height: 25    Sample Baseline V to V?: Y    Sample Baseline V to V pts: 3
Sample Filter: Binomial       # of Pts for Filter: 3        Sample Start Region (min): 0  Sample End Region (min): 50
Manual Baseline Start (min): 10     Manual Baseline End (min): 48
Marker Peak Width (sec): 5    Marker Min Peak Height: 200   Marker Baseline V to V?: Y    Marker Baseline V to V pts: 3
Lower Marker Selection: First Peak > 200 RFU                Upper Marker Selection: Last Peak > 200 RFU
Ladder Size (bp): 1, 100, 200, 300, 400, 500, 600, 700, 800, 900, 1000, 1200, 1500, 2000, 3000, 6000
Quantification Using: Ladder          Final Concentration (ng/uL): 0.0830          Dilution Factor: 12.0
```

 Copyright 2015 Advanced Analytical Technologies, Inc.

Data File: 2017 07 25 17H 40M.raw
Sample: UM-UC-3+vector3
Well Location: D5

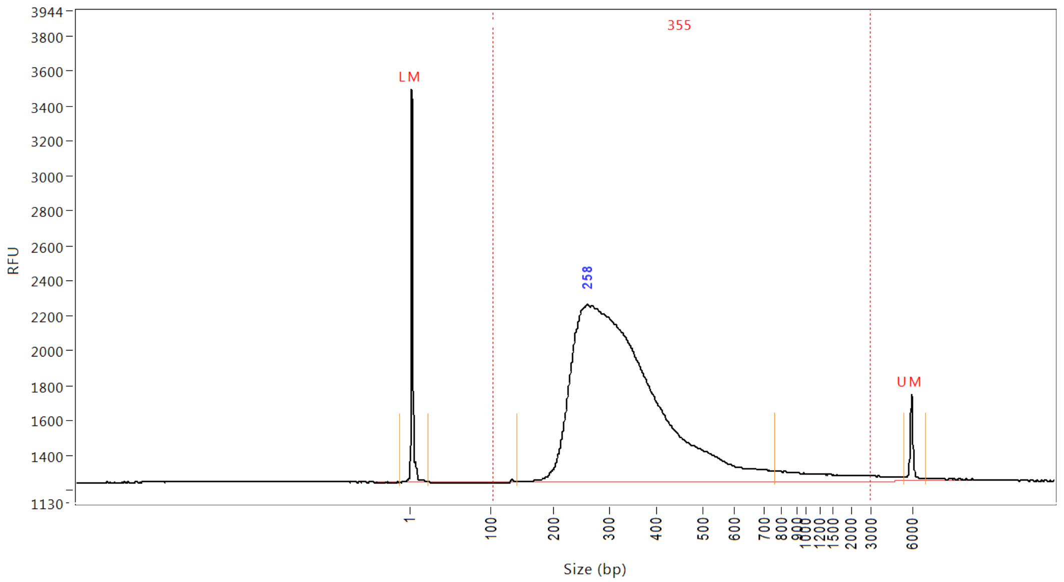

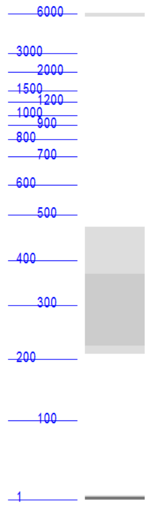

Size (bp)	Molarity (nmole/L)	Conc. (ng/uL)	From (bp)	To (bp)	Avg. Size (bp)	CV%	RFU	Corr. Peak Area
1 (LM)	18.824	0.0144	0	23	1	157.00	2241	13.008
258	14.813	2.3243	140	763	329	28.87	1012	174.897
6000 (UM)	0.001	0.0027	5500	6994	6019	3.66	493	2.453
	TIC:	2.3243	ng/uL					
	TIM:	14.813	nmole/L					
	Total Conc.:	2.4003	ng/uL					

Smear Analysis 100 bp to 3000 bp 2.3881 ng/ul 99.5 %Total 11.063 nmole/L 355 Avg. Size (b.p.) 58.88 %CV

Sample Peak Width (sec): 50 Sample Min Peak Height: 25 Sample Baseline V to V?: Y Sample Baseline V to V pts: 3
Sample Filter: Binomial # of Pts for Filter: 3 Sample Start Region (min): 0 Sample End Region (min): 50
Manual Baseline Start (min): 10 Manual Baseline End (min): 48
Marker Peak Width (sec): 5 Marker Min Peak Height: 200 Marker Baseline V to V?: Y Marker Baseline V to V pts: 3
Lower Marker Selection: First Peak > 200 RFU Upper Marker Selection: Last Peak > 200 RFU
Ladder Size (bp): 1, 100, 200, 300, 400, 500, 600, 700, 800, 900, 1000, 1200, 1500, 2000, 3000, 6000
Quantification Using: Ladder Final Concentration (ng/uL): 0.0830 Dilution Factor: 12.0

 Copyright 2015 Advanced Analytical Technologies, Inc.

Data File: 2017 07 25 17H 40M.raw
Sample: UM-UC-3+vector4
Well Location: D6

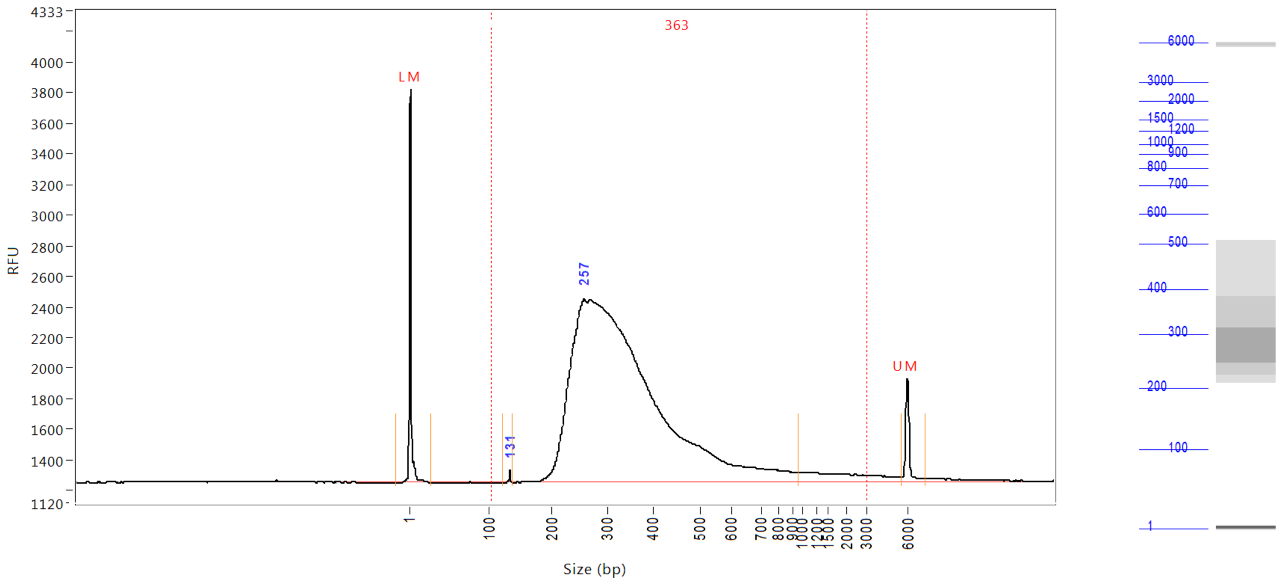

Size (bp)	Molarity (nmole/L)	Conc. (ng/uL)	From (bp)	To (bp)	Avg. Size (bp)	CV%	RFU	Corr. Peak Area
1 (LM)	18.824	0.0144	0	29	1	207.49	2561	15.055
131	0.042	0.0033	121	135	131	0.85	71	0.292
257	15.079	2.3554	135	952	339	32.70	1189	205.121
6000 (UM)	0.001	0.0032	5595	7350	6052	4.76	665	3.359

TIC:	2.3587	ng/uL
TIM:	15.121	nmole/L
Total Conc.:	2.4211	ng/uL

Smear Analysis	100 bp to 3000 bp	2.4056 ng/ul	99.4 %Total	10.891 nmole/L	363 Avg. Size (b.p.)	61.49 %CV

```
Sample Peak Width (sec): 50   Sample Min Peak Height: 25    Sample Baseline V to V?: Y    Sample Baseline V to V pts: 3
Sample Filter: Binomial       # of Pts for Filter: 3        Sample Start Region (min): 0  Sample End Region (min): 50
Manual Baseline Start (min): 10     Manual Baseline End (min): 48
Marker Peak Width (sec): 5    Marker Min Peak Height: 200   Marker Baseline V to V?: Y    Marker Baseline V to V pts: 3
Lower Marker Selection: First Peak > 200 RFU                Upper Marker Selection: Last Peak > 200 RFU
Ladder Size (bp): 1, 100, 200, 300, 400, 500, 600, 700, 800, 900, 1000, 1200, 1500, 2000, 3000, 6000
Quantification Using: Ladder          Final Concentration (ng/uL): 0.0830          Dilution Factor: 12.0
```

 Copyright 2015 Advanced Analytical Technologies, Inc.

Data File: 2017 07 25 17H 40M.raw
Sample: UM-UC-3+HD51
Well Location: D7

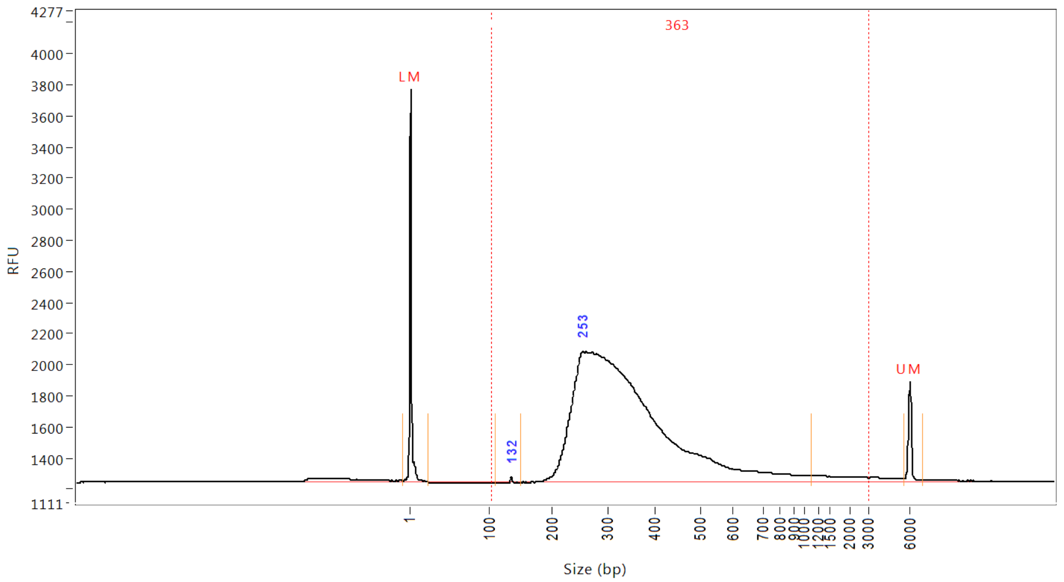

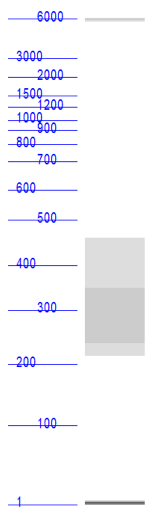

Size (bp)	Molarity (nmole/L)	Conc. (ng/uL)	From (bp)	To (bp)	Avg. Size (bp)	CV%	RFU	Corr. Peak Area
1 (LM)	18.824	0.0144	0	25	1	185.85	2523	14.965
132	0.018	0.0015	109	149	132	0.78	31	0.127
253	10.882	1.6728	149	1105	343	34.30	835	144.810
6000 (UM)	0.001	0.0029	5643	6971	6011	2.74	643	2.962

TIC:	1.6743	ng/uL
TIM:	10.900	nmole/L
Total Conc.:	1.7081	ng/uL

Smear Analysis 100 bp to 3000 bp 1.6986 ng/ul 99.4 %Total 7.690 nmole/L 363 Avg. Size (b.p.) 59.47 %CV

```
Sample Peak Width (sec): 50   Sample Min Peak Height: 25    Sample Baseline V to V?: Y    Sample Baseline V to V pts: 3
Sample Filter: Binomial       # of Pts for Filter: 3        Sample Start Region (min): 0  Sample End Region (min): 50
Manual Baseline Start (min): 10     Manual Baseline End (min): 48
Marker Peak Width (sec): 5    Marker Min Peak Height: 200   Marker Baseline V to V?: Y    Marker Baseline V to V pts: 3
Lower Marker Selection: First Peak > 200 RFU                Upper Marker Selection: Last Peak > 200 RFU
Ladder Size (bp): 1, 100, 200, 300, 400, 500, 600, 700, 800, 900, 1000, 1200, 1500, 2000, 3000, 6000
Quantification Using: Ladder          Final Concentration (ng/uL): 0.0830          Dilution Factor: 12.0
```

 Copyright 2015 Advanced Analytical Technologies, Inc.

Data File: 2017 07 25 17H 40M.raw
Sample: UM-UC-3+HD5_2
Well Location: D8

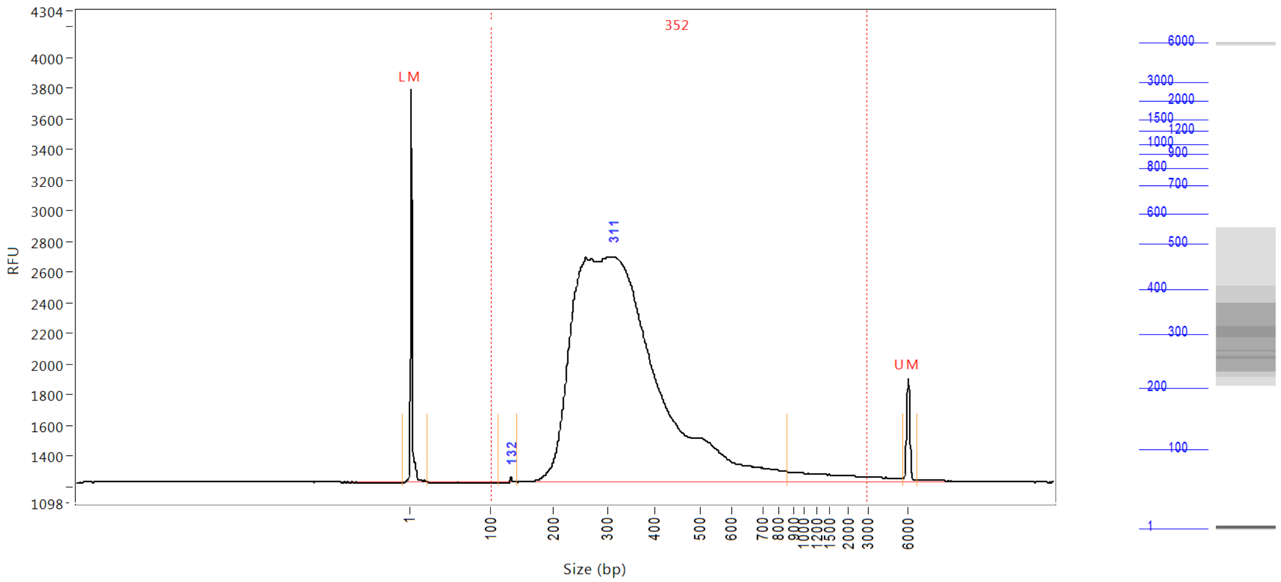

Size (bp)	Molarity (nmole/L)	Conc. (ng/uL)	From (bp)	To (bp)	Avg. Size (bp)	CV%	RFU	Corr. Peak Area
1 (LM)	18.824	0.0144	0	21	1	188.23	2553	14.700
132	0.016	0.0013	111	143	132	0.78	27	0.109
311	16.672	3.1496	143	858	333	29.92	1464	267.829
6000 (UM)	0.001	0.0030	5619	6616	5999	2.21	668	3.069
	TIC:	3.1509	ng/uL					
	TIM:	16.688	nmole/L					
	Total Conc.:	3.2176	ng/uL					

Smear Analysis 100 bp to 3000 bp 3.2051 ng/ul 99.6 %Total 14.996 nmole/L 352 Avg. Size (b.p.) 54.00 %CV

```
Sample Peak Width (sec): 50   Sample Min Peak Height: 25    Sample Baseline V to V?: Y    Sample Baseline V to V pts: 3
Sample Filter: Binomial       # of Pts for Filter: 3        Sample Start Region (min): 0  Sample End Region (min): 50
Manual Baseline Start (min): 10     Manual Baseline End (min): 48
Marker Peak Width (sec): 5    Marker Min Peak Height: 200   Marker Baseline V to V?: Y    Marker Baseline V to V pts: 3
Lower Marker Selection: First Peak > 200 RFU                Upper Marker Selection: Last Peak > 200 RFU
Ladder Size (bp): 1, 100, 200, 300, 400, 500, 600, 700, 800, 900, 1000, 1200, 1500, 2000, 3000, 6000
Quantification Using: Ladder          Final Concentration (ng/uL): 0.0830          Dilution Factor: 12.0
```

 Copyright 2015 Advanced Analytical Technologies, Inc.

Data File: 2017 07 25 17H 40M.raw
Sample: UM-UC-3+HD5_3
Well Location: D9

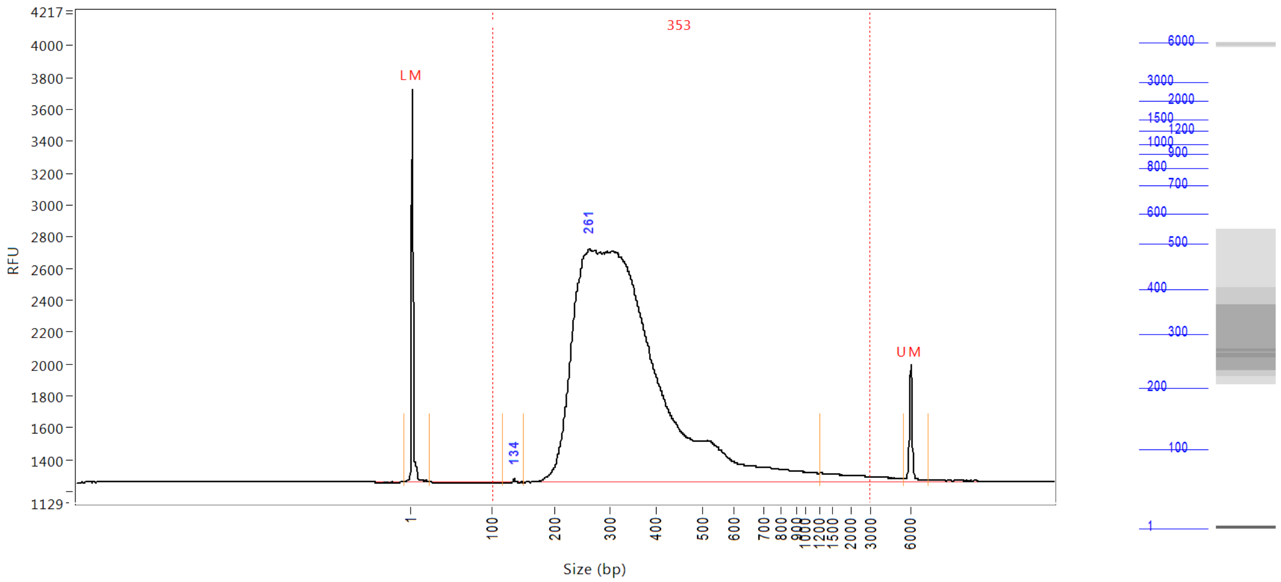

Size (bp)	Molarity (nmole/L)	Conc. (ng/uL)	From (bp)	To (bp)	Avg. Size (bp)	CV%	RFU	Corr. Peak Area
1 (LM)	18.824	0.0144	0	24	1	187.24	2458	14.179
134	0.009	0.0007	118	150	134	0.55	20	0.059
261	19.992	3.1722	150	1201	339	34.64	1460	260.181
6000 (UM)	0.001	0.0035	5476	7255	6010	3.59	734	3.481

TIC:	3.1729	ng/uL
TIM:	20.000	nmole/L
Total Conc.:	3.2149	ng/uL

Smear Analysis 100 bp to 3000 bp 3.2031 ng/ul 99.6 %Total 14.922 nmole/L 353 Avg. Size (b.p.) 54.76 %CV

```
Sample Peak Width (sec): 50   Sample Min Peak Height: 25    Sample Baseline V to V?: Y    Sample Baseline V to V pts: 3
Sample Filter: Binomial       # of Pts for Filter: 3        Sample Start Region (min): 0  Sample End Region (min): 50
Manual Baseline Start (min): 10     Manual Baseline End (min): 48
Marker Peak Width (sec): 5    Marker Min Peak Height: 200   Marker Baseline V to V?: Y    Marker Baseline V to V pts: 3
Lower Marker Selection: First Peak > 200 RFU                Upper Marker Selection: Last Peak > 200 RFU
Ladder Size (bp): 1, 100, 200, 300, 400, 500, 600, 700, 800, 900, 1000, 1200, 1500, 2000, 3000, 6000
Quantification Using: Ladder          Final Concentration (ng/uL): 0.0830          Dilution Factor: 12.0
```

 Copyright 2015 Advanced Analytical Technologies, Inc.

Data File: 2017 07 25 17H 40M.raw
Sample: UM-UC-3+HD5_4
Well Location: D10

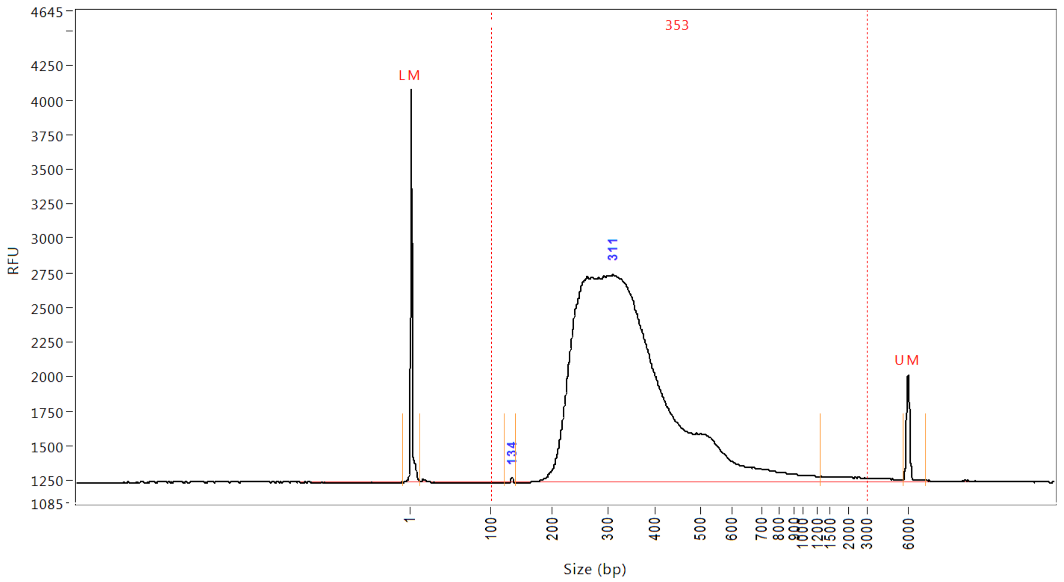

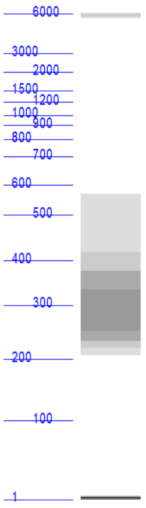

Size (bp)	Molarity (nmole/L)	Conc. (ng/uL)	From (bp)	To (bp)	Avg. Size (bp)	CV%	RFU	Corr. Peak Area
1 (LM)	18.824	0.0144	0	13	1	144.79	2839	16.585
134	0.020	0.0016	121	139	133	0.79	36	0.157
311	15.667	2.9597	139	1292	343	33.64	1497	283.948
6000 (UM)	0.001	0.0031	5619	7279	5998	3.18	771	3.526

TIC:	2.9613	ng/uL
TIM:	15.687	nmole/L
Total Conc.:	2.9914	ng/uL

Smear Analysis 100 bp to 3000 bp 2.9803 ng/ul 99.6 %Total 13.890 nmole/L 353 Avg. Size (b.p.) 49.72 %CV

```
Sample Peak Width (sec): 50   Sample Min Peak Height: 25    Sample Baseline V to V?: Y    Sample Baseline V to V pts: 3
Sample Filter: Binomial       # of Pts for Filter: 3        Sample Start Region (min): 0  Sample End Region (min): 50
Manual Baseline Start (min): 10     Manual Baseline End (min): 48
Marker Peak Width (sec): 5    Marker Min Peak Height: 200   Marker Baseline V to V?: Y    Marker Baseline V to V pts: 3
Lower Marker Selection: First Peak > 200 RFU                Upper Marker Selection: Last Peak > 200 RFU
Ladder Size (bp): 1, 100, 200, 300, 400, 500, 600, 700, 800, 900, 1000, 1200, 1500, 2000, 3000, 6000
Quantification Using: Ladder          Final Concentration (ng/uL): 0.0830          Dilution Factor: 12.0
```

 Copyright 2015 Advanced Analytical Technologies, Inc.

Data File: 2017 07 25 17H 40M.raw
Sample: HBLAK+vector1
Well Location: D11

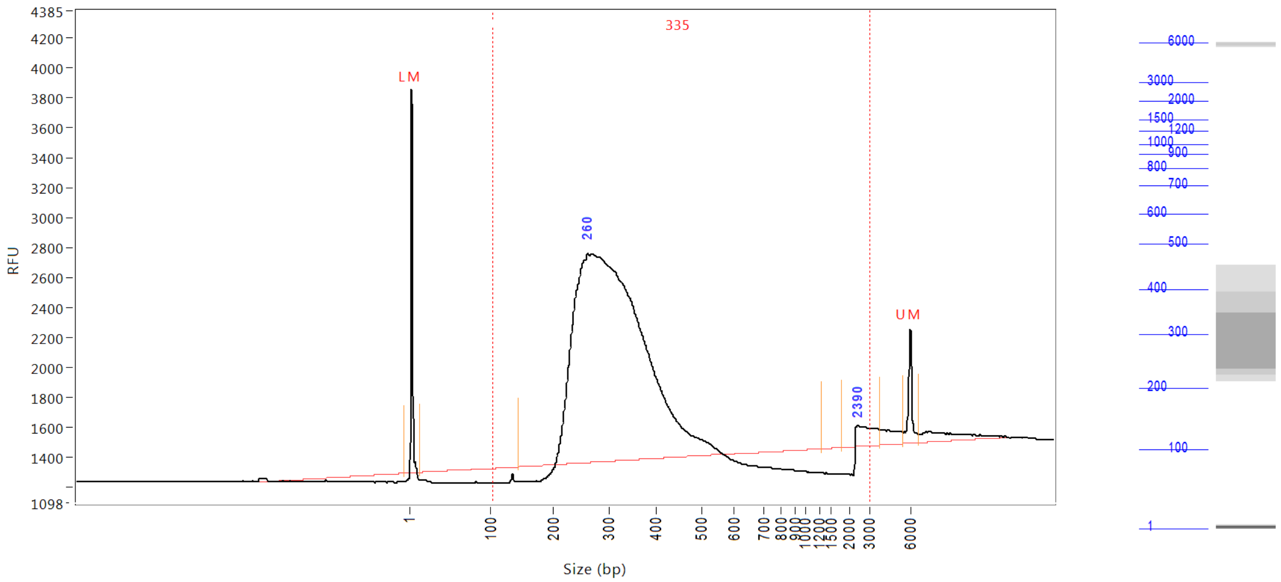

Size (bp)	Molarity (nmole/L)	Conc. (ng/uL)	From (bp)	To (bp)	Avg. Size (bp)	CV%	RFU	Corr. Peak Area
1 (LM)	18.824	0.0144	0	12	1	127.66	2561	13.483
260	17.145	2.7084	143	1259	311	20.57	1393	211.239
2390	0.032	0.0467	1764	3739	2912	14.39	135	3.646
6000 (UM)	0.001	0.0045	5500	6592	5990	3.26	768	4.235

TIC:	2.7552	ng/uL
TIM:	17.177	nmole/L
Total Conc.:	2.7883	ng/uL

Smear Analysis 100 bp to 3000 bp 2.7368 ng/ul 98.2 %Total 13.427 nmole/L 335 Avg. Size (b.p.) 73.15 %CV

```
Sample Peak Width (sec): 50   Sample Min Peak Height: 25    Sample Baseline V to V?: Y    Sample Baseline V to V pts: 3
Sample Filter: Binomial       # of Pts for Filter: 3        Sample Start Region (min): 0  Sample End Region (min): 50
Manual Baseline Start (min): 10     Manual Baseline End (min): 48
Marker Peak Width (sec): 5    Marker Min Peak Height: 200   Marker Baseline V to V?: Y    Marker Baseline V to V pts: 3
Lower Marker Selection: First Peak > 200 RFU                Upper Marker Selection: Last Peak > 200 RFU
Ladder Size (bp): 1, 100, 200, 300, 400, 500, 600, 700, 800, 900, 1000, 1200, 1500, 2000, 3000, 6000
Quantification Using: Ladder          Final Concentration (ng/uL): 0.0830          Dilution Factor: 12.0
```

 Copyright 2015 Advanced Analytical Technologies, Inc.

Data File: 2017 07 25 18H 46M.raw
Sample: HBLAK+vector2
Well Location: E1

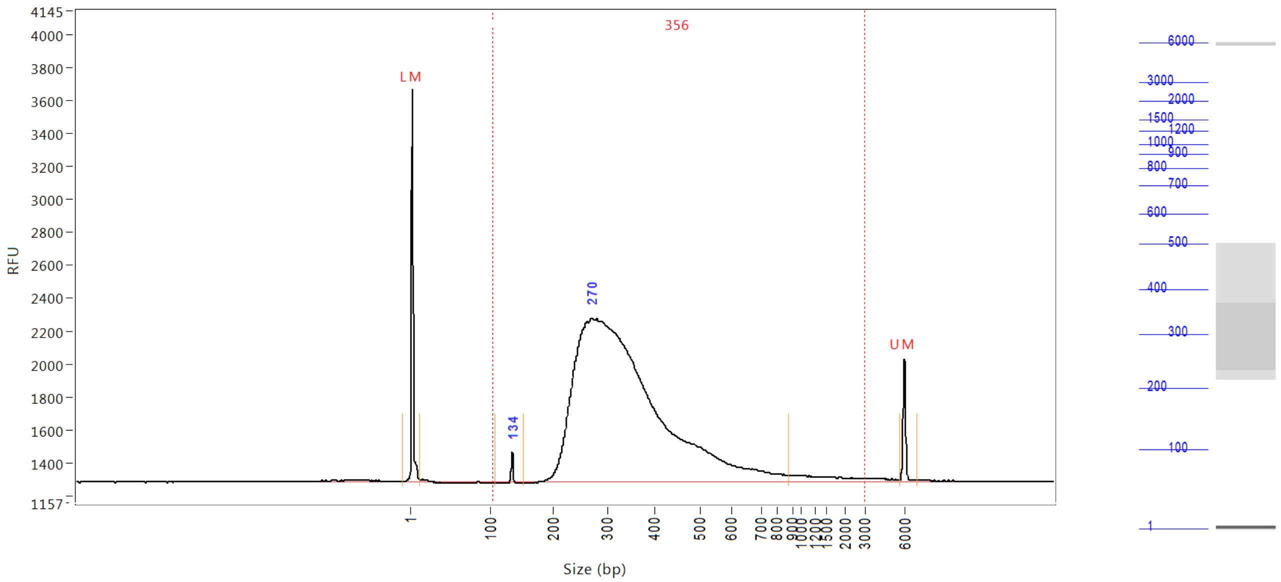

Size (bp)	Molarity (nmole/L)	Conc. (ng/uL)	From (bp)	To (bp)	Avg. Size (bp)	CV%	RFU	Corr. Peak Area
1 (LM)	17.054	0.0131	0	12	1	212.58	2382	14.036
134	0.119	0.0097	107	152	133	1.03	184	0.866
270	11.499	1.8888	152	884	339	30.58	990	169.274
6000 (UM)	0.001	0.0025	5653	6912	5998	2.42	747	2.691

TIC:	1.8985	ng/uL
TIM:	11.617	nmole/L
Total Conc.:	1.9380	ng/uL

Smear Analysis 100 bp to 3000 bp 1.9280 ng/ul 99.5 %Total 8.913 nmole/L 356 Avg. Size (b.p.) 53.33 %CV

Sample Peak Width (sec): 50 Sample Min Peak Height: 25 Sample Baseline V to V?: Y Sample Baseline V to V pts: 3
Sample Filter: Binomial # of Pts for Filter: 3 Sample Start Region (min): 0 Sample End Region (min): 50
Manual Baseline Start (min): 10 Manual Baseline End (min): 48
Marker Peak Width (sec): 5 Marker Min Peak Height: 200 Marker Baseline V to V?: Y Marker Baseline V to V pts: 3
Lower Marker Selection: First Peak > 200 RFU Upper Marker Selection: Last Peak > 200 RFU
Ladder Size (bp): 1, 100, 200, 300, 400, 500, 600, 700, 800, 900, 1000, 1200, 1500, 2000, 3000, 6000
Quantification Using: Ladder Final Concentration (ng/uL): 0.0830 Dilution Factor: 12.0

 Copyright 2015 Advanced Analytical Technologies, Inc.

Data File: 2017 07 25 18H 46M.raw
Sample: HBLAK+vector3
Well Location: E2

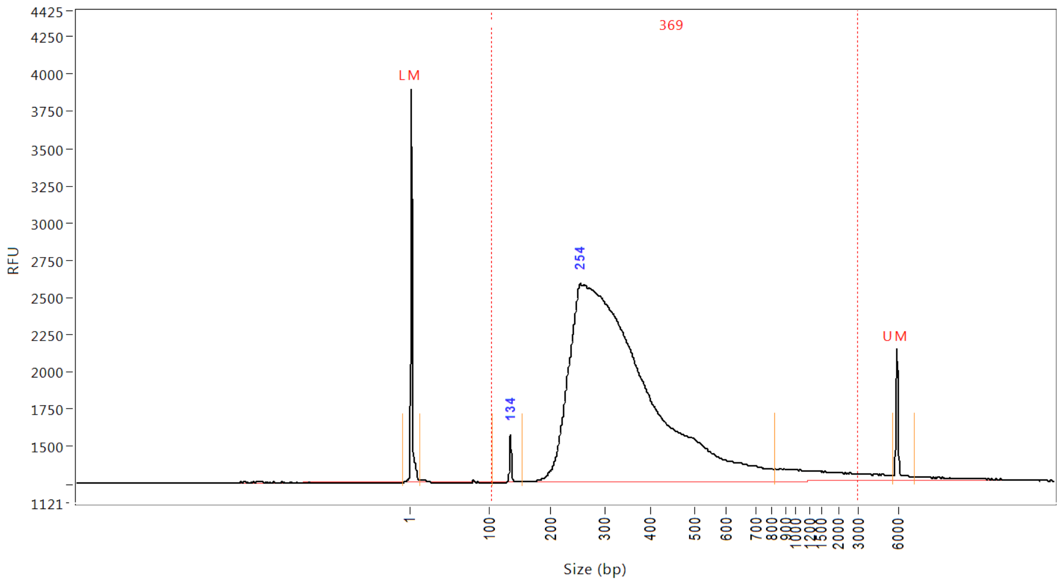

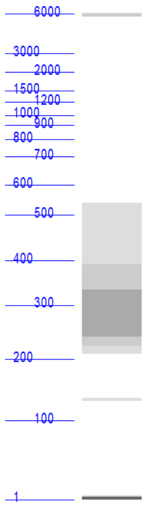

Size (bp)	Molarity (nmole/L)	Conc. (ng/uL)	From (bp)	To (bp)	Avg. Size (bp)	CV%	RFU	Corr. Peak Area
1 (LM)	17.054	0.0131	0	13	1	183.78	2634	15.115
134	0.241	0.0196	104	154	133	1.24	319	1.888
254	14.718	2.2738	154	824	338	30.94	1328	219.435
6000 (UM)	0.001	0.0031	5703	7306	6065	4.88	882	3.602

TIC:	2.2933	ng/uL
TIM:	14.959	nmole/L
Total Conc.:	2.3832	ng/uL

Smear Analysis 100 bp to 3000 bp 2.3633 ng/ul 99.2 %Total 10.542 nmole/L 369 Avg. Size (b.p.) 63.80 %CV

```
Sample Peak Width (sec): 50   Sample Min Peak Height: 25    Sample Baseline V to V?: Y    Sample Baseline V to V pts: 3
Sample Filter: Binomial       # of Pts for Filter: 3        Sample Start Region (min): 0  Sample End Region (min): 50
Manual Baseline Start (min): 10     Manual Baseline End (min): 48
Marker Peak Width (sec): 5    Marker Min Peak Height: 200   Marker Baseline V to V?: Y    Marker Baseline V to V pts: 3
Lower Marker Selection: First Peak > 200 RFU                Upper Marker Selection: Last Peak > 200 RFU
Ladder Size (bp): 1, 100, 200, 300, 400, 500, 600, 700, 800, 900, 1000, 1200, 1500, 2000, 3000, 6000
Quantification Using: Ladder          Final Concentration (ng/uL): 0.0830          Dilution Factor: 12.0
```

 Copyright 2015 Advanced Analytical Technologies, Inc.

Data File: 2017 07 25 18H 46M.raw
Sample: HBLAK+vector4
Well Location: E3

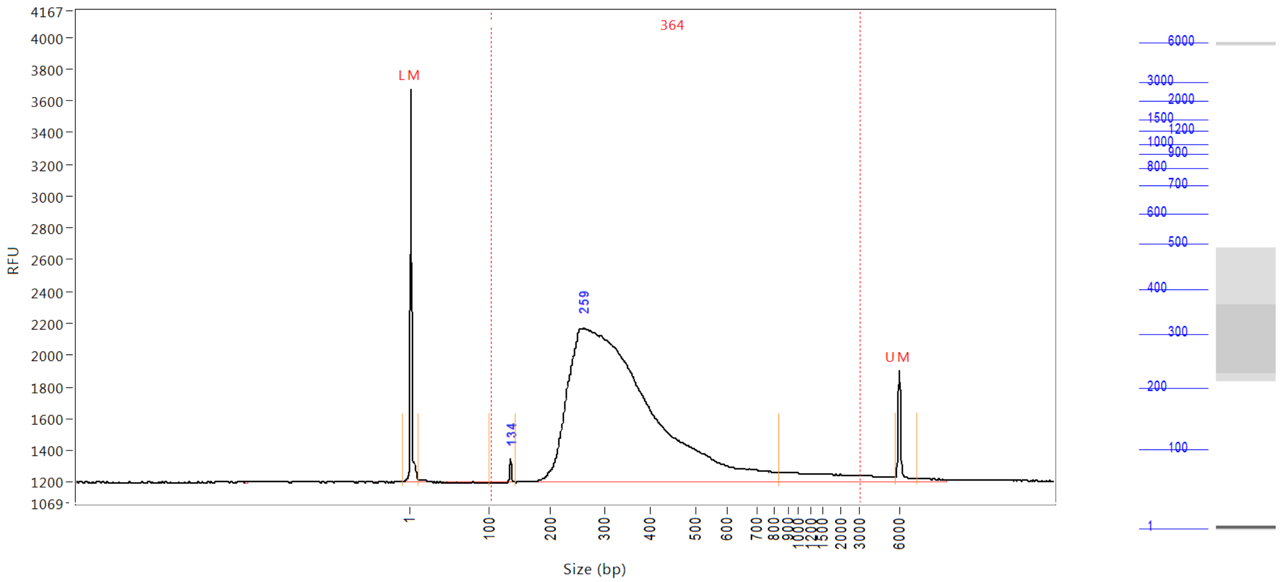

Size (bp)	Molarity (nmole/L)	Conc. (ng/uL)	From (bp)	To (bp)	Avg. Size (bp)	CV%	RFU	Corr. Peak Area
1 (LM)	17.054	0.0131	0	12	1	195.17	2468	14.250
134	0.102	0.0083	99	142	133	1.09	140	0.759
259	11.709	1.8427	142	840	336	30.74	965	167.664
6000 (UM)	0.001	0.0026	5678	7331	6065	4.45	697	2.841

TIC:	1.8511	ng/uL
TIM:	11.811	nmole/L
Total Conc.:	1.9148	ng/uL

Smear Analysis 100 bp to 3000 bp 1.9000 ng/ul 99.2 %Total 8.582 nmole/L 364 Avg. Size (b.p.) 62.31 %CV

```
Sample Peak Width (sec): 50   Sample Min Peak Height: 25    Sample Baseline V to V?: Y    Sample Baseline V to V pts: 3
Sample Filter: Binomial       # of Pts for Filter: 3        Sample Start Region (min): 0  Sample End Region (min): 50
Manual Baseline Start (min): 10     Manual Baseline End (min): 48
Marker Peak Width (sec): 5    Marker Min Peak Height: 200   Marker Baseline V to V?: Y    Marker Baseline V to V pts: 3
Lower Marker Selection: First Peak > 200 RFU                Upper Marker Selection: Last Peak > 200 RFU
Ladder Size (bp): 1, 100, 200, 300, 400, 500, 600, 700, 800, 900, 1000, 1200, 1500, 2000, 3000, 6000
Quantification Using: Ladder          Final Concentration (ng/uL): 0.0830          Dilution Factor: 12.0
```

 Copyright 2015 Advanced Analytical Technologies, Inc.

Data File: 2017 07 25 18H 46M.raw
Sample: Ladder
Well Location: E12

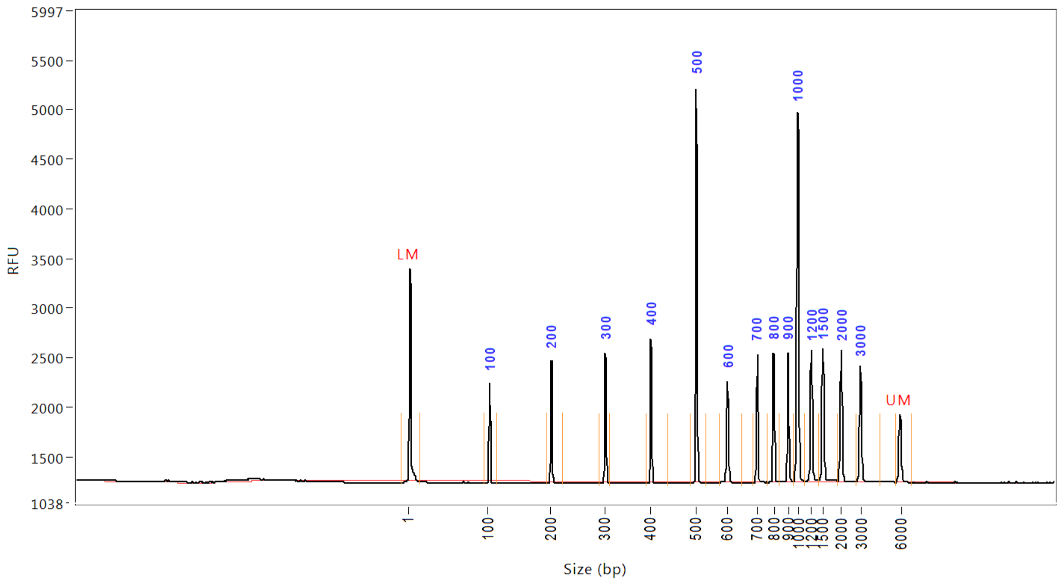

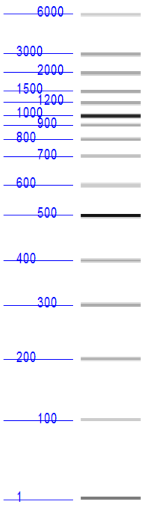

Size (bp)	Molarity (nmole/L)	Conc. (ng/uL)	From (bp)	To (bp)	Avg. Size (bp)	CV%	RFU	Corr. Peak Area
1 (LM)	17.054	0.0131	0	14	1	160.45	2133	11.730
100	0.808	0.0492	94	112	100	0.90	977	3.685
200	0.456	0.0555	193	222	200	0.59	1209	4.157
300	0.306	0.0559	290	311	300	0.46	1295	4.186
400	0.248	0.0604	391	438	399	0.40	1438	4.522
500	0.549	0.1668	486	531	500	0.38	3948	12.494
600	0.145	0.0529	573	648	598	0.53	1012	3.958
700	0.125	0.0533	689	764	699	0.46	1277	3.988
800	0.113	0.0549	764	840	798	0.63	1296	4.110
900	0.103	0.0561	840	949	899	0.80	1302	4.198
1000	0.269	0.1636	949	1098	995	1.10	3726	12.252
1200	0.079	0.0577	1098	1380	1195	2.00	1321	4.325
1500	0.067	0.0611	1380	1898	1496	3.94	1340	4.580
2000	0.048	0.0582	1898	2793	2004	3.77	1325	4.361
3000	0.028	0.0504	2793	4513	2988	3.63	1174	3.776
6000 (UM)	0.001	0.0026	5628	6912	5980	1.43	687	2.363

TIC:	0.9960	ng/uL
TIM:	3.345	nmole/L
Total Conc.:	0.9960	ng/uL

Sample Peak Width (sec): 10   Sample Min Peak Height: 100   Sample Baseline V to V?: Y   Sample Baseline V to V pts: 3
Sample Filter: Binomial   # of Pts for Filter: 3   Sample Start Region (min): 0   Sample End Region (min): 50
Manual Baseline Start (min): 10   Manual Baseline End (min): 48
Marker Peak Width (sec): 5   Marker Min Peak Height: 200   Marker Baseline V to V?: Y   Marker Baseline V to V pts: 3
Lower Marker Selection: First Peak > 200 RFU   Upper Marker Selection: Last Peak > 200 RFU
Ladder Size (bp): 1, 100, 200, 300, 400, 500, 600, 700, 800, 900, 1000, 1200, 1500, 2000, 3000, 6000
Quantification Using: Ladder   Final Concentration (ng/uL): 0.0830   Dilution Factor: 12.0

 Copyright 2015 Advanced Analytical Technologies, Inc.
