# Supplementary material for: Proteomic and transcriptomic profiles of human urothelial cancer cells with histone deacetylase 5 overexpression
Source: Sci Data. 2022 May 27;9:240. doi: 10.1038/s41597-022-01319-0 (PMC9142574; doi:10.1038/s41597-022-01319-0)
Supplement: Supplementary file 4 — QC report of RNA samples [file 41597_2022_1319_MOESM4_ESM.pdf]

Number		VM-Cub-1+vector vs VM-Cub-1+HD5				Annotation	
12,536	Feature ID	P-value	FC	Bonferroni	FDR	Uniprot ID	gene symbol
1	A1CF	0.00756622	2.90	1	0.02697042	Q9NQ94	A1CF
1	A4GNT	7.9155E-19	-12.35	4.6048E-14	5.8982E-18	Q9UNA3	A4GNT
1	AADAC	7.8616E-97	37.80	4.5734E-92	1.3222E-95	P22760	AADAC
1	AADACL2	1.1757E-41	62.40	6.8398E-37	1.258E-40	Q6P093	AADACL2
1	AADACL2-AS1	2.628E-250	78.91	1.529E-245	7.18E-249		
1	AADACP1	6.786E-159	128.87	3.948E-154	1.504E-157		
1	AADAT	0	-1.82	0	0	Q8N5Z0	AADAT
1	AAED1	1.9168E-42	2.42	1.1151E-37	2.0731E-41	Q7RTV5	AAED1
1	AANAT	0.00166977	-2.50	1	0.00636965	Q16613	AANAT
1	AAR2	4.619E-88	1.84	2.6871E-83	7.3098E-87	Q9Y312	AAR2
1	AARS	0	-1.72	0	0	P49588	AARS
1	AASDH	0	-1.54	0	0	Q4L235	AASDH
1	AATBC	4.9872E-35	-6.86	2.9013E-30	4.9199E-34		
1	AATF	4.8199E-41	1.65	2.8039E-36	5.1073E-40	Q9NY61	AATF
1	AATK	2.5842E-17	-5.28	1.5033E-12	1.8661E-16	Q6ZMQ8	AATK
1	AB015752.3	1.3015E-08	-7.89	0.00075712	6.8998E-08		
1	ABBA0100093	0.01219299	6.77	1	0.04233704		
1	ABC13-47488	8.1532E-79	16.74	4.7431E-74	1.198E-77		
1	ABC7-424044	7.464E-52	-54.66	4.3421E-47	8.8833E-51		
1	ABCA1	2.068E-158	-4.62	1.203E-153	4.577E-157	O95477	ABCA1
1	ABCA10	8.5922E-10	1.95	4.9984E-05	4.8002E-09	Q8WWZ4	ABCA10
1	ABCA12	6.475E-135	-10.49	3.767E-130	1.329E-133	Q86UK0	ABCA12
1	ABCA13	1.5979E-05	-3.96	0.92953437	7.1207E-05	Q86UQ4	ABCA13
1	ABCA2	0	-2.44	0	0	Q9BZC7	ABCA2
1	ABCA3	1.201E-239	123.41	6.985E-235	3.252E-238	Q99758	ABCA3
1	ABCA4	7.2116E-09	-3.98	0.00041953	3.8666E-08	P78363	ABCA4
1	ABCA5	2.6472E-17	1.70	1.54E-12	1.9109E-16	Q8WWZ7	ABCA5
1	ABCA7	0	-4.01	0	0	Q8IZY2	ABCA7
1	ABCA9-AS1	1.764E-33	-26.93	1.0262E-28	1.7071E-32		
1	ABCB1	5.1065E-18	-4.57	2.9707E-13	3.7424E-17	P08183	ABCB1
1	ABCB10	0	-1.57	0	0	Q9NRK6	ABCB10
1	ABCB10P1	4.6128E-08	-53.38	0.00268344	2.3779E-07		
1	ABCB10P3	0.00445449	-17.85	1	0.0162927		
1	ABCB4	7.5722E-05	-3.65	1	0.00032241	P21439	ABCB4
1	ABCB6	0	-1.61	0	0	Q9NP58	ABCB6
1	ABCB8	9.056E-76	2.18	5.2682E-71	1.2966E-74	Q9NUT2	ABCB8
1	ABCC10	0	-2.54	0	0	Q5T3U5	ABCC10
1	ABCC11	7.0231E-14	-4.06	4.0856E-09	4.5787E-13	Q96J66	ABCC11
1	ABCC12	0.00081675	13.52	1	0.00320561	Q96J65	ABCC12
1	ABCC2	2.8705E-14	1.87	1.6699E-09	1.8948E-13	Q92887	ABCC2
1	ABCC3	5.897E-165	-5.38	3.431E-160	1.328E-163	O15438	ABCC3
1	ABCC4	1.0481E-39	1.69	6.097E-35	1.0921E-38	O15439	ABCC4
1	ABCC5	0	-1.54	0	0	O15440	ABCC5
1	ABCC6	4.8427E-05	-1.85	1	0.00020891	O95255	ABCC6

1	ABCD2	0.00163247	-8.19	1	0.00623269	Q9UBJ2	ABCD2
1	ABCF2	1.595E-142	2.46	9.28E-138	3.372E-141	Q9UG63	ABCF2
1	ABCG1	6.803E-216	-9.89	3.958E-211	1.782E-214	P45844	ABCG1
1	ABCG2	2.184E-139	-5.58	1.271E-134	4.571E-138	Q9UNQ0	ABCG2
1	ABCG4	2.2325E-09	4.17	0.00012988	1.225E-08	Q9H172	ABCG4
1	ABHD10	5.1261E-33	1.56	2.9821E-28	4.9315E-32	Q9NUJ1	ABHD10
1	ABHD11-AS1	4.155E-10	-66.42	2.4171E-05	2.3515E-09		
1	ABHD12B	0.00632999	-2.08	1	0.02274214	Q7Z5M8	ABHD12B
1	ABHD14B	8.58E-122	3.29	4.991E-117	1.677E-120	Q96IU4	ABHD14B
1	ABHD15-AS1	0.0003755	-4.94	1	0.00151538		
1	ABHD16B	3.5454E-16	2.07	2.0625E-11	2.4898E-15	Q9H3Z7	ABHD16B
1	ABHD17A	5.3221E-26	1.55	3.0961E-21	4.5417E-25	Q96GS6	ABHD17A
1	ABHD17C	0	-2.01	0	0	Q6PCB6	ABHD17C
1	ABHD18	1.9784E-13	-1.57	1.1509E-08	1.2716E-12	Q0P651	ABHD18
1	ABHD3	0	-1.70	0	0	Q8WU67	ABHD3
1	ABHD5	3.32E-141	2.94	1.932E-136	6.981E-140	Q8WTS1	ABHD5
1	ABI3BP	5.6759E-08	-1.65	0.00330192	2.9115E-07	Q7Z7G0	ABI3BP
1	ABL1	1.0637E-49	1.52	6.188E-45	1.2418E-48	P00519	ABL1
1	ABL2	5.4042E-17	1.50	3.1438E-12	3.8726E-16	P42684	ABL2
1	ABLIM1	0	-9.50	0	0	O14639	ABLIM1
1	ABLIM2	6.7732E-27	-2.96	3.9402E-22	5.8766E-26	Q6H8Q1	ABLIM2
1	ABLIM3	9.847E-206	-4.76	5.728E-201	2.521E-204	O94929	ABLIM3
1	ABR	5.411E-111	1.70	3.148E-106	9.981E-110	Q12979	ABR
1	ABTB1	2.67E-53	2.47	1.5532E-48	3.2265E-52	Q969K4	ABTB1
1	AC000120.7	0.01119138	-2.08	1	0.03904567		
1	AC002066.1	5.7692E-41	-8.72	3.3562E-36	6.1055E-40		
1	AC002116.7	3.0829E-07	-1.56	0.01793431	1.5201E-06		
1	AC002117.1	1.3016E-62	-5.15	7.5718E-58	1.6932E-61		
1	AC002128.5	0.00040657	-14.09	1	0.00163544		
1	AC002310.11	1.229E-07	2.86	0.00714929	6.1958E-07		
1	AC002310.12	1.0131E-05	-1.82	0.5893827	4.5752E-05		
1	AC002310.13	5.6983E-08	-1.78	0.00331494	2.9217E-07		
1	AC002310.7	4.2021E-09	2.91	0.00024445	2.2763E-08		
1	AC002398.9	1.2383E-06	1.89	0.07203941	5.9185E-06		
1	AC002454.1	4.4827E-05	1.87	1	0.00019396		
1	AC002456.2	0.00051045	-6.10	1	0.00203807		
1	AC002463.3	6.6076E-09	-11.29	0.00038439	3.547E-08		
1	AC002472.8	0.00113164	-5.01	1	0.00438706		
1	AC002519.6	0.00246054	-19.72	1	0.00922233		
1	AC002539.1	3.4073E-09	15.00	0.00019822	1.8549E-08		
1	AC002985.3	1.2783E-05	8.24	0.74362148	5.7338E-05		
1	AC003002.4	0.01088319	-3.40	1	0.03803201		
1	AC003002.6	0.00358986	-3.14	1	0.01325441		
1	AC003092.1	9.216E-224	2823.82	5.362E-219	2.452E-222		
1	AC003092.2	2.9655E-10	69.53	1.7251E-05	1.6893E-09		
1	AC003958.2	1.2628E-16	-8.19	7.3463E-12	8.9819E-16		
1	AC003984.1	8.2514E-14	-7.94	4.8002E-09	5.3687E-13		

1	AC003988.1	0.00159392	-23.41	1	0.00608989		
1	AC004012.1	2.9074E-05	-16.22	1	0.00012737		
1	AC004014.3	6.2169E-05	-5.71	1	0.0002663		
1	AC004069.2	8.7968E-16	-4.07	5.1175E-11	6.0944E-15		
1	AC004112.4	5.3843E-08	1.55	0.00313227	2.7651E-07		
1	AC004156.3	0.00021499	4.43	1	0.00088555		
1	AC004237.1	5.5796E-08	3.75	0.0032459	2.8631E-07		
1	AC004538.3	0.00012263	2.92	1	0.00051408		
1	AC004754.3	6.9851E-06	-55.06	0.40635044	3.1871E-05		
1	AC004870.3	0.00011775	-5.24	1	0.00049463		
1	AC004870.4	2.14E-07	-7.74	0.01244924	1.0649E-06		
1	AC004893.11	8.5511E-08	-1.94	0.0049745	4.3434E-07		
1	AC004895.4	0.005546	-3.44	1	0.02007798		
1	AC004951.5	5.7089E-05	-3.71	1	0.00024533		
1	AC004951.6	4.4144E-06	-2.44	0.25680311	2.0354E-05		
1	AC004967.7	1.5857E-10	-1.61	9.2247E-06	9.1443E-10		
1	AC004980.10	0.00431946	-6.40	1	0.01583266		
1	AC004980.7	0	-2.47	0	0		
1	AC004980.9	3.8533E-08	-2.80	0.00224161	1.9943E-07		
1	AC004985.12	5.465E-05	-7.14	1	0.00023505		
1	AC005062.2	5.3984E-12	-3.87	3.1405E-07	3.2985E-11		
1	AC005076.5	3.6875E-10	-2.99	2.1451E-05	2.0932E-09		
1	AC005083.1	1.3082E-29	-13.55	7.6106E-25	1.1949E-28		
1	AC005104.3	0.01122443	-1.62	1	0.03915159		
1	AC005154.6	0	-3.51	0	0		
1	AC005197.2	0.00295628	21.65	1	0.01100452		
1	AC005256.1	9.9061E-06	-2.93	0.5762795	4.4767E-05		
1	AC005262.2	2.1977E-05	-40.71	1	9.7063E-05		
1	AC005262.3	0.00020795	-15.46	1	0.00085766		
1	AC005301.9	0.00106855	-3.06	1	0.00415186		
1	AC005329.7	0.01128476	1.83	1	0.03935256		
1	AC005336.4	1.188E-154	-27.61	6.914E-150	2.593E-153		
1	AC005481.5	1.0365E-22	176.37	6.0295E-18	8.3338E-22		
1	AC005537.2	2.8149E-28	-3.96	1.6375E-23	2.5166E-27		
1	AC005540.3	0.01193197	3.67	1	0.04150648		
1	AC005592.2	3.544E-13	2.10	2.0617E-08	2.2556E-12		
1	AC005609.16	0.00025632	-34.57	1	0.001048		
1	AC005618.6	9.9363E-10	-8.81	5.7804E-05	5.5362E-09		
1	AC005682.5	1.0985E-25	1.59	6.3906E-21	9.3267E-25		
1	AC005722.4	0.00117013	-11.91	1	0.00453113		
1	AC005753.1	9.4947E-05	-32.57	1	0.00040168		
1	AC005754.7	8.9457E-10	-6.59	5.2041E-05	4.9934E-09		
1	AC005932.1	0.00576914	1.75	1	0.02083264		
1	AC005943.2	5.8814E-20	1.91	3.4214E-15	4.4824E-19		
1	AC006042.6	2.8389E-06	-17.47	0.16515143	1.3262E-05		
1	AC006116.20	0.01224499	-3.99	1	0.04251253		
1	AC006159.4	6.3823E-06	-6.30	0.37128165	2.92E-05		

1	AC006160.5	1.9331E-06	-2.10	0.11245349	9.1307E-06		
1	AC006262.6	3.305E-139	-10.33	1.923E-134	6.904E-138		
1	AC006273.4	0.00510661	2.57	1	0.01856119		
1	AC006277.2	7.8867E-09	2.11	0.0004588	4.2193E-08		
1	AC006369.2	5.7785E-11	5.26	3.3616E-06	3.3866E-10		
1	AC006460.2	5.3915E-09	-7.00	0.00031365	2.9074E-08		
1	AC006548.28	2.7644E-08	-10.49	0.00160816	1.4429E-07		
1	AC006994.2	3.1308E-06	-4.94	0.18213296	1.4582E-05		
1	AC007036.5	0.00080678	-4.33	1	0.00316864		
1	AC007036.6	0.00476785	-19.67	1	0.01738316		
1	AC007040.8	2.0634E-11	-7.39	1.2003E-06	1.2334E-10		
1	AC007128.1	1.107E-183	-16.39	6.437E-179	2.635E-182		
1	AC007192.4	0.0097682	-2.65	1	0.03429835		
1	AC007193.6	9.9093E-98	-9.73	5.7646E-93	1.6792E-96		
1	AC007204.2	2.4095E-46	155.10	1.4017E-41	2.7207E-45		
1	AC007255.8	2.3791E-32	-7.52	1.384E-27	2.2652E-31		
1	AC007285.6	0.00048992	-1.80	1	0.00195881		
1	AC007326.11	1.7486E-18	-63.70	1.0172E-13	1.2929E-17		
1	AC007326.13	2.5318E-11	-3.39	1.4729E-06	1.5086E-10		
1	AC007349.4	0.00197502	-8.83	1	0.00747575		
1	AC007386.4	2.9008E-06	2.37	0.16874878	1.3541E-05		
1	AC007389.3	4.2178E-06	-12.17	0.24536704	1.9466E-05		
1	AC007405.4	2.8532E-05	-2.05	1	0.00012503		
1	AC007405.6	1.3479E-11	-2.54	7.8415E-07	8.1251E-11		
1	AC007405.8	0.00489781	-4.81	1	0.01783234		
1	AC007566.10	2.6935E-06	-1.88	0.15669142	1.2601E-05		
1	AC007682.1	1.5406E-22	-6.70	8.9622E-18	1.2348E-21		
1	AC007731.1	7.6723E-06	-40.33	0.44632586	3.4913E-05		
1	AC007743.1	7.7036E-10	-5.76	4.4815E-05	4.3108E-09		
1	AC007773.2	2.0345E-14	-2.38	1.1835E-09	1.3488E-13		
1	AC007879.1	4.7523E-33	-6.20	2.7646E-28	4.5726E-32		
1	AC007879.2	7.4322E-07	-6.06	0.04323607	3.5943E-06		
1	AC007879.3	2.6269E-06	-6.99	0.15281851	1.2298E-05		
1	AC007879.5	5.331E-174	-3.94	3.101E-169	1.235E-172		
1	AC007966.1	4.0011E-15	-12.55	2.3276E-10	2.7131E-14		
1	AC007969.5	2.5497E-05	2.80	1	0.00011209		
1	AC008132.13	3.3014E-06	6.04	0.19205679	1.5345E-05		
1	AC008440.10	7.9742E-05	40.97	1	0.00033903		
1	AC008746.12	0.00177863	-2.22	1	0.00676452		
1	AC008937.3	0.00054969	3.17	1	0.00219056		
1	AC008984.2	0.00041765	5.75	1	0.00167887		
1	AC009133.12	4.6427E-09	1.54	0.00027008	2.5105E-08		
1	AC009133.22	1.3067E-06	29.32	0.07601691	6.235E-06		
1	AC009237.8	2.818E-130	9.10	1.639E-125	5.686E-129		
1	AC009264.1	3.4767E-95	-8.72	2.0225E-90	5.7936E-94		
1	AC009299.2	1.2398E-81	-9.43	7.2123E-77	1.8646E-80		
1	AC009336.19	4.3237E-19	52.72	2.5153E-14	3.2384E-18		

1	AC009404.2	2.9304E-32	3.32	1.7047E-27	2.7864E-31		
1	AC009478.1	1.9859E-14	-8.27	1.1553E-09	1.3171E-13		
1	AC009495.2	4.2683E-15	-21.97	2.4831E-10	2.89E-14		
1	AC009501.4	1.4816E-06	-2.00	0.08619222	7.0453E-06		
1	AC009502.4	9.0802E-10	-3.65	5.2823E-05	5.0655E-09		
1	AC009506.1	0.00062966	-1.82	1	0.0024959		
1	AC009784.3	4.6655E-09	-12.50	0.00027141	2.5226E-08		
1	AC009950.2	6.0083E-14	-2.98	3.4953E-09	3.9264E-13		
1	AC010091.1	0.00820473	-15.99	1	0.02911444		
1	AC010105.1	3.5181E-06	-10.14	0.20465937	1.6322E-05		
1	AC010127.3	1.9519E-57	-22.08	1.1355E-52	2.4388E-56		
1	AC010148.1	7.9246E-06	-2.01	0.46100464	3.603E-05		
1	AC010226.4	0.01229532	-2.97	1	0.04266691		
1	AC010492.2	4.7548E-06	12.94	0.27660321	2.1887E-05		
1	AC010504.2	1.1929E-09	-5.19	6.9395E-05	6.621E-09		
1	AC010642.1	1.8196E-08	3.34	0.00105856	9.5936E-08		
1	AC010733.5	0.00027684	-2.91	1	0.00112833		
1	AC010883.5	3.0729E-14	-4.64	1.7876E-09	2.0261E-13		
1	AC010890.1	1.5518E-22	173.20	9.0273E-18	1.2436E-21		
1	AC010967.2	9.947E-45	-12.50	5.7866E-40	1.103E-43		
1	AC011288.2	8.371E-99	-8.98	4.8695E-94	1.4297E-97		
1	AC011290.4	0.00301162	-3.14	1	0.01120406		
1	AC011380.8	6.541E-08	-11.27	0.00380518	3.3432E-07		
1	AC011380.9	0.00046726	-5.87	1	0.00187155		
1	AC011738.4	0.00310834	-4.37	1	0.01154691		
1	AC011997.1	5.7561E-18	-2.62	3.3486E-13	4.2115E-17		
1	AC012074.2	9.4379E-07	-18.44	0.05490414	4.539E-06		
1	AC012146.7	5.0242E-45	3.94	2.9228E-40	5.6002E-44		
1	AC012358.8	9.7326E-11	-3.01	5.6618E-06	5.659E-10		
1	AC012442.5	1.0133E-06	-2.15	0.05894779	4.8629E-06		
1	AC012462.1	0.00291132	23.81	1	0.0108427		
1	AC012462.2	1.4759E-05	40.57	0.85859264	6.5954E-05		
1	AC012494.1	7.4594E-11	-7.72	4.3394E-06	4.3547E-10		
1	AC012501.2	1.3631E-70	-11.22	7.9299E-66	1.8782E-69		
1	AC012668.2	0.0073533	6.77	1	0.026242		
1	AC013268.3	6.077E-48	-12.85	3.5353E-43	6.9825E-47		
1	AC013268.4	0.00090267	-29.02	1	0.00352857		
1	AC013271.3	0.00383599	-21.56	1	0.01412551		
1	AC015849.16	1.8396E-09	-5.04	0.00010702	1.013E-08		
1	AC015922.5	0.01277743	-7.66	1	0.04425805		
1	AC016582.2	0.0003786	-5.33	1	0.00152747		
1	AC016722.4	4.042E-10	2.94	2.3514E-05	2.2896E-09		
1	AC016738.4	7.6357E-57	54.99	4.442E-52	9.4976E-56		
1	AC016745.3	0.01052778	-2.82	1	0.03684755		
1	AC016747.3	1.935E-124	51.40	1.126E-119	3.82E-123		
1	AC016831.6	0.00157775	-10.06	1	0.00603127		
1	AC016995.3	5.1473E-19	-3.22	2.9944E-14	3.8483E-18		

1	AC016999.2	0.007941	8.29	1	0.02823023		
1	AC017002.1	0.00999484	2.74	1	0.03504973		
1	AC017002.4	1.3244E-06	-10.32	0.0770467	6.3158E-06		
1	AC017074.2	1.3448E-05	1.88	0.78232172	6.0239E-05		
1	AC017104.6	3.4725E-08	3.04	0.00202007	1.8015E-07		
1	AC018641.7	3.4289E-24	-12.24	1.9947E-19	2.8322E-23		
1	AC018647.3	0.00380456	-4.13	1	0.01401156		
1	AC018755.16	2.6552E-06	4.76	0.15446079	1.2426E-05		
1	AC018890.6	1.637E-13	-1.73	9.5233E-09	1.0543E-12		
1	AC021218.2	5.0582E-08	1.72	0.00294253	2.601E-07		
1	AC022007.5	4.2987E-12	-2.58	2.5007E-07	2.6368E-11		
1	AC023347.1	1.0813E-44	-14.05	6.2903E-40	1.1984E-43		
1	AC024084.1	0.00526711	-5.63	1	0.01911354		
1	AC024560.2	0.00639158	-3.39	1	0.02294499		
1	AC024560.3	0	-2.24	0	0		
1	AC024592.12	0.00189542	2.08	1	0.00719178		
1	AC024592.9	9.7454E-15	-38.05	5.6693E-10	6.5254E-14		
1	AC024937.4	0.00302571	-3.89	1	0.01125218		
1	AC024937.6	3.0256E-16	-5.42	1.7601E-11	2.129E-15		
1	AC025165.8	2.5E-08	-3.24	0.00145437	1.3079E-07		
1	AC025263.3	0.01280385	-4.44	1	0.04433903		
1	AC026150.8	0.00377362	-2.47	1	0.01389938		
1	AC026188.1	0.00048976	-6.58	1	0.00195832		
1	AC027612.6	3.242E-209	190.77	1.886E-204	8.361E-208		
1	AC034220.3	2.5551E-08	-1.55	0.0014864	1.336E-07		
1	AC046143.3	6.2008E-10	1.58	3.6073E-05	3.4863E-09		
1	AC058791.1	2.6896E-69	2.66	1.5646E-64	3.6634E-68		
1	AC060834.3	8.9189E-62	-26.04	5.1885E-57	1.152E-60		
1	AC061992.2	1.9746E-08	-2.59	0.00114873	1.0384E-07		
1	AC062017.1	3.8832E-10	3.20	2.259E-05	2.2018E-09		
1	AC062029.1	5.4463E-19	-2.86	3.1684E-14	4.0703E-18		
1	AC067945.4	1.4008E-27	-10.69	8.1487E-23	1.2354E-26		
1	AC067956.1	8.2753E-06	-4.99	0.48140599	3.7601E-05		
1	AC068138.1	1.2756E-11	94.14	7.4209E-07	7.6957E-11		
1	AC068491.2	0.00140517	-26.48	1	0.00539816		
1	AC068533.7	1.5202E-05	-1.83	0.88433494	6.7843E-05		
1	AC068580.6	1.0382E-05	-1.73	0.60395397	4.6854E-05		
1	AC068580.7	6.2545E-11	-6.63	3.6385E-06	3.6615E-10		
1	AC068831.10	3.8375E-14	-3.61	2.2325E-09	2.5223E-13		
1	AC068831.15	3.8277E-05	-44.40	1	0.0001664		
1	AC068831.6	0.00701776	-1.79	1	0.02508917		
1	AC069277.2	2.1374E-13	-3.86	1.2434E-08	1.3715E-12		
1	AC069513.4	0.00375831	2.62	1	0.01384562		
1	AC072062.1	2.9751E-08	-2.19	0.00173072	1.5501E-07		
1	AC073043.1	9.5164E-07	-2.70	0.05536043	4.5756E-06		
1	AC073071.1	0.00088507	-10.06	1	0.00346254		
1	AC073072.5	0.00560808	3.31	1	0.02029641		

1	AC073115.7	8.606E-08	-7.78	0.00500647	4.3706E-07		
1	AC073130.1	0.00112194	-2.03	1	0.00435146		
1	AC073130.3	3.483E-11	-7.18	2.0262E-06	2.0613E-10		
1	AC073218.1	0.00460619	-2.98	1	0.01682851		
1	AC073254.1	3.3973E-08	2.00	0.00197634	1.7635E-07		
1	AC073342.12	0.00587097	-1.57	1	0.02118115		
1	AC073343.13	9.3913E-12	-3.05	5.4633E-07	5.6933E-11		
1	AC073834.3	0.00158237	-2.71	1	0.00604736		
1	AC073957.15	0.00410033	-21.63	1	0.01506647		
1	AC074011.2	0.0018213	-6.36	1	0.00692138		
1	AC074183.4	0.00083106	12.03	1	0.00325893		
1	AC074286.1	2.5842E-38	-6.16	1.5033E-33	2.6509E-37		
1	AC074289.1	8.8401E-12	-11.37	5.1426E-07	5.3608E-11		
1	AC074391.1	2.9552E-11	-3.21	1.7192E-06	1.756E-10		
1	AC078882.1	0.00435057	-7.15	1	0.01593666		
1	AC079305.11	1.6986E-07	-11.99	0.00988136	8.4972E-07		
1	AC079305.8	1.6441E-06	-1.81	0.09564254	7.7986E-06		
1	AC079354.5	3.0572E-12	14.26	1.7785E-07	1.8868E-11		
1	AC079610.1	1.295E-20	-8.90	7.5337E-16	9.9784E-20		
1	AC079613.1	2.3146E-05	-5.07	1	0.00010208		
1	AC079779.4	6.759E-07	3.20	0.0393196	3.275E-06		
1	AC079922.3	5.2838E-05	1.70	1	0.0002275		
1	AC083843.1	1.3033E-37	-2.60	7.5815E-33	1.3241E-36		
1	AC083843.4	5.4584E-07	-2.35	0.03175377	2.6586E-06		
1	AC084809.2	0.00572721	-2.72	1	0.02070536		
1	AC090587.4	0.00046493	5.14	1	0.00186259		
1	AC090952.5	0.00666659	-5.10	1	0.02388803		
1	AC091729.9	1.802E-129	-4.29	1.048E-124	3.632E-128		
1	AC091814.2	1.0391E-06	-12.87	0.0604491	4.9834E-06		
1	AC092013.1	0.00028807	-11.24	1	0.00117258		
1	AC092162.1	0.00017756	2.23	1	0.0007358		
1	AC092573.2	6.8801E-08	-3.24	0.00400243	3.5124E-07		
1	AC092625.1	1.9898E-06	-20.27	0.11575206	9.3939E-06		
1	AC093162.5	1.0178E-27	-5.28	5.9212E-23	9.0084E-27		
1	AC093323.3	2.0406E-93	4.41	1.1871E-88	3.359E-92		
1	AC093388.3	2.2887E-08	-2.96	0.00133144	1.1993E-07		
1	AC093616.4	8.8507E-20	2.56	5.1488E-15	6.727E-19		
1	AC093627.10	0.00200846	21.76	1	0.00759688		
1	AC093627.12	1.1495E-27	75.65	6.6872E-23	1.0157E-26		
1	AC093627.9	2.364E-109	128.74	1.376E-104	4.312E-108		
1	AC093690.1	0.0030276	-2.87	1	0.01125848		
1	AC093818.1	1.0383E-25	-2.99	6.0401E-21	8.8216E-25		
1	AC093901.1	2.74E-42	-4.20	1.594E-37	2.9595E-41		
1	AC097662.2	0.00189411	-1.71	1	0.00718725		
1	AC097713.3	0.00028075	-27.20	1	0.00114355		
1	AC097721.2	0.00052834	-1.58	1	0.00210678		
1	AC097724.3	3.6488E-16	-3.49	2.1226E-11	2.5617E-15		

1	AC098820.3	6.1792E-28	-5.37	3.5947E-23	5.4956E-27			
1	AC098820.4	1.0256E-06	-10.59	0.05966307	4.9194E-06			
1	AC098826.5	0.00011139	32.03	1	0.0004688			
1	AC098828.2	1.6412E-09	-4.98	9.5473E-05	9.0513E-09			
1	AC098828.3	0.00025784	-11.31	1	0.00105386			
1	AC100830.3	7.3748E-06	-4.50	0.42902358	3.3599E-05			
1	AC102953.4	1.7501E-06	-5.83	0.10181151	8.2881E-06			
1	AC104135.2	4.0225E-11	-19.17	2.3401E-06	2.3728E-10			
1	AC104389.28	0.00068185	-2.93	1	0.00269233			
1	AC104794.4	2.1646E-05	-36.54	1	9.5619E-05			
1	AC105760.2	6.2052E-06	-1.91	0.36098016	2.8408E-05			
1	AC107218.3	2.2893E-16	4.29	1.3318E-11	1.6146E-15			
1	AC107399.2	2.2093E-06	1.72	0.12852176	1.04E-05			
1	AC108142.1	4.4602E-58	-9.98	2.5947E-53	5.6028E-57			
1	AC108463.1	4.9488E-19	140.34	2.8789E-14	3.7023E-18			
1	AC108463.2	1.061E-24	41.33	6.1723E-20	8.8555E-24			
1	AC112198.1	1.1483E-07	-15.51	0.00668007	5.7987E-07			
1	AC112229.1	5.9247E-34	-9.12	3.4466E-29	5.78E-33			
1	AC112229.6	0.00030554	-27.16	1	0.0012414			
1	AC112497.1	4.4053E-05	-6.58	1	0.00019071			
1	AC112518.3	0.00040131	10.96	1	0.00161528			
1	AC113189.5	4.3442E-15	-2.12	2.5272E-10	2.9403E-14			
1	AC114271.2	2.3559E-05	3.54	1	0.00010385			
1	AC114765.1	2.3876E-33	-5.73	1.389E-28	2.3069E-32			
1	AC115522.3	2.6233E-10	-9.94	1.5261E-05	1.4982E-09			
1	AC115617.2	0.00105626	-10.74	1	0.00410551			
1	AC116050.1	2.5837E-07	65.14	0.01503036	1.2797E-06			
1	AC116366.5	0.00049425	-7.90	1	0.00197531			
1	AC116366.6	2.6085E-13	-7.55	1.5175E-08	1.6672E-12			
1	AC116609.3	0.00162461	11.17	1	0.00620428			
1	AC118754.4	9.6375E-11	31.47	5.6065E-06	5.6054E-10			
1	AC124789.1	1.714E-188	-7.68	9.969E-184	4.147E-187			
1	AC124861.1	5.9374E-92	-14.13	3.454E-87	9.6724E-91			
1	AC124997.1	0.0030363	-3.54	1	0.01128939			
1	AC127904.2	0.00476786	-4.30	1	0.01738316			
1	AC128709.2	4.4105E-06	-4.58	0.25657832	2.0338E-05			
1	AC128709.3	1.0241E-06	-3.73	0.05957702	4.9136E-06			
1	AC128709.4	5.3043E-11	-35.87	3.0857E-06	3.115E-10			
1	AC129778.2	0.00262098	3.03	1	0.00980282			
1	AC131056.5	0.00751105	19.73	1	0.02678196			
1	AC133785.1	7.9313E-13	-6.04	4.614E-08	4.9913E-12			
1	AC135048.13	0.00064661	-2.17	1	0.00256032			
1	AC137932.4	6.8267E-06	-1.76	0.39713468	3.1172E-05			
1	AC137932.6	1.4203E-76	20.39	8.2624E-72	2.0462E-75			
1	AC138035.2	0.000907	-2.09	1	0.00354501			
1	AC139099.5	1.5515E-07	-53.68	0.00902588	7.7823E-07			
1	AC139143.2	0.00031734	-10.66	1	0.00128747			

1	AC139712.4	0.00978382	-7.60	1	0.0343451		
1	AC141586.5	2.7942E-24	2.14	1.6255E-19	2.3112E-23		
1	AC141930.2	0.00573247	22.07	1	0.02070536		
1	AC144450.1	5.936E-217	568.32	3.453E-212	1.56E-215		
1	AC144450.2	5.1536E-49	390.62	2.998E-44	5.9781E-48		
1	AC144568.2	0.00246391	21.73	1	0.00923436		
1	AC145124.2	4.9992E-63	32.16	2.9082E-58	6.5192E-62		
1	AC145343.2	7.6992E-08	-2.36	0.00447895	3.921E-07		
1	AC147651.4	1.3303E-07	22.09	0.00773867	6.6944E-07		
1	AC156455.1	4.282E-167	20.36	2.491E-162	9.719E-166		
1	AC159540.1	5.4316E-08	1.79	0.00315977	2.7881E-07		
1	AC159540.2	0.0024133	2.46	1	0.00905401		
1	ACAA1	3.3447E-38	1.58	1.9457E-33	3.4256E-37	P09110	ACAA1
1	ACAA2	1.529E-141	24.20	8.895E-137	3.221E-140	P42765	ACAA2
1	ACACA	0	-1.73	0	0	Q13085	ACACA
1	ACACB	2.3798E-24	2.15	1.3845E-19	1.9727E-23	O00763	ACACB
1	ACAD8	2.9755E-30	1.54	1.731E-25	2.7467E-29	Q9UKU7	ACAD8
1	ACADL	8.6864E-37	-8.76	5.0532E-32	8.7487E-36	P28330	ACADL
1	ACADM	8.5122E-27	1.51	4.9519E-22	7.3699E-26	P11310	ACADM
1	ACADSB	1.1219E-28	3.90	6.5263E-24	1.0098E-27	P45954	ACADSB
1	ACAP2	0	-1.58	0	0	Q15057	ACAP2
1	ACAT1	1.7033E-19	1.53	9.909E-15	1.2859E-18	P24752	ACAT1
1	ACAT2	4.6006E-80	2.07	2.6764E-75	6.8327E-79	Q9BWD1	ACAT2
1	ACBD3	0	-1.63	0	0	Q9H3P7	ACBD3
1	ACBD3-AS1	0.001491	-7.05	1	0.00571094		
1	ACCS	0	-3.06	0	0	Q96QU6	ACCS
1	ACE	3.5605E-40	-10.83	2.0713E-35	3.7328E-39	P12821	ACE
1	ACE2	3.3249E-07	-49.48	0.01934226	1.6367E-06	Q9BYF1	ACE2
1	ACEA_U3_1	0.00088378	29.94	1	0.00345795		
1	ACER2	2.106E-23	-3.59	1.2251E-18	1.7142E-22	Q5QJU3	ACER2
1	ACER3	0	-1.65	0	0	Q9NUN7	ACER3
1	ACHE	8.5922E-07	-2.57	0.0499841	4.1398E-06	P22303	ACHE
1	ACKR3	2.0942E-09	-60.86	0.00012183	1.15E-08	P25106	ACKR3
1	ACLY	0	-2.31	0	0	P53396	ACLY
1	ACO1	4.7116E-79	4.57	2.7409E-74	6.9374E-78	P21399	ACO1
1	ACOT11	3.55E-174	-6.21	2.065E-169	8.23E-173	Q8WXI4	ACOT11
1	ACOT4	1.7311E-32	-7.00	1.007E-27	1.6522E-31	Q8N9L9	ACOT4
1	ACOT6	4.7858E-05	-6.35	1	0.00020661	Q3I5F7	ACOT6
1	ACOT7	4.027E-71	4.86	2.3427E-66	5.5659E-70	O00154	ACOT7
1	ACOX2	0.00061712	3.32	1	0.00244752	Q99424	ACOX2
1	ACOXL	6.3655E-18	-12.45	3.7031E-13	4.6539E-17	Q9NUZ1	ACOXL
1	ACP5	4.8502E-15	-4.66	2.8216E-10	3.2759E-14	P13686	ACP5
1	ACP6	0	-2.04	0	0	Q9NPH0	ACP6
1	ACP7	7.7644E-29	-5.59	4.5169E-24	7.004E-28	Q6ZNF0	ACP7
1	ACPP	5.256E-189	-42.76	3.058E-184	1.274E-187	P15309	ACPP
1	ACR	0.00773177	-7.62	1	0.02753693	P10323	ACR
1	ACRBP	4.7294E-05	-2.39	1	0.00020428	Q8NEB7	ACRBP

1	ACSF2	0	-2.37	0	0	Q96CM8	ACSF2
1	ACSL1	0	-10.85	0	0	P33121	ACSL1
1	ACSL3	0	-2.30	0	0	O95573	ACSL3
1	ACSL4	1.531E-101	2.42	8.9091E-97	2.661E-100	O60488	ACSL4
1	ACSL5	2.5619E-36	-5.98	1.4904E-31	2.5687E-35	Q9ULC5	ACSL5
1	ACSL6	3.06E-05	-36.30	1	0.00013389	Q9UKU0	ACSL6
1	ACSM1	4.9435E-05	-34.84	1	0.00021317	Q08AH1	ACSM1
1	ACSM2A	2.3981E-06	42.52	0.13950463	1.1257E-05	Q08AH3	ACSM2A
1	ACSM3	6.0841E-20	-2.81	3.5394E-15	4.6345E-19	Q53FZ2	ACSM3
1	ACSM4	2.3764E-25	-7.26	1.3824E-20	2.0079E-24	P0C7M7	ACSM4
1	ACSM6	0.00011101	-3.65	1	0.0004672	Q6P461	ACSM6
1	ACSS1	7.478E-100	3.72	4.3503E-95	1.2844E-98	Q9NUB1	ACSS1
1	ACSS2	0	-3.42	0	0	Q9NR19	ACSS2
1	ACSS3	3.451E-199	-12.63	2.007E-194	8.675E-198	Q9H6R3	ACSS3
1	ACTA2	3.614E-129	-8.86	2.102E-124	7.272E-128	P62736	ACTA2
1	ACTBL2	0.00483013	4.52	1	0.01759888	Q562R1	ACTBL2
1	ACTBP1	8.28E-54	-25.80	4.8168E-49	1.0069E-52		
1	ACTG1	1.7091E-77	1.92	9.9425E-73	2.48E-76	P63261	ACTG1
1	ACTG1P15	9.1459E-05	-7.12	1	0.00038723		
1	ACTG1P17	0.00066103	-1.68	1	0.00261397		
1	ACTG2	0.01352573	-16.15	1	0.04674148	P63267	ACTG2
1	ACTL10	1.1273E-05	1.63	0.65581254	5.0732E-05		
1	ACTN1	3.1336E-15	2.11	1.8229E-10	2.1343E-14	P12814	ACTN1
1	ACTN3	0.00091988	-3.95	1	0.00359389	Q08043	ACTN3
1	ACTN4	0	-1.52	0	0	O43707	ACTN4
1	ACTR10	1.1865E-32	1.78	6.9025E-28	1.1353E-31	Q9NZ32	ACTR10
1	ACTR1A	2.005E-100	1.91	1.1663E-95	3.455E-99	P61163	ACTR1A
1	ACTR3	7.1365E-28	1.51	4.1516E-23	6.3363E-27	P61158	ACTR3
1	ACTR3C	3.6819E-84	-3.10	2.1419E-79	5.6544E-83	Q9C0K3	ACTR3C
1	ACTR5	5.0552E-82	1.89	2.9408E-77	7.6266E-81	Q9H9F9	ACTR5
1	ACTR8	2.6274E-56	1.90	1.5285E-51	3.259E-55	Q9H981	ACTR8
1	ACTRT3	9.1073E-21	-2.53	5.2981E-16	7.0463E-20	Q9BYD9	ACTRT3
1	ACVR1B	0	-1.67	0	0	P36896	ACVR1B
1	ACVR1C	4.11E-175	-10.64	2.391E-170	9.563E-174	Q8NER5	ACVR1C
1	ACVRL1	0.00517986	-8.22	1	0.01880748	P37023	ACVRL1
1	ACY1	0	-1.60	0	0	Q03154	ACY1
1	ACY3	6.0175E-09	-8.81	0.00035006	3.238E-08	Q96HD9	ACY3
1	ACYP1	1.2706E-28	1.60	7.3915E-24	1.1422E-27	P07311	ACYP1
1	ACYP2	4.0248E-41	-2.24	2.3414E-36	4.271E-40	P14621	ACYP2
1	ADA	1.2134E-33	3.83	7.0588E-29	1.1776E-32	P00813	ADA
1	ADAD2	3.893E-49	-12.00	2.2647E-44	4.5236E-48	Q8NCV1	ADAD2
1	ADAM10	0	-2.12	0	0	O14672	ADAM10
1	ADAM12	5.22E-181	29.86	3.036E-176	1.235E-179	O43184	ADAM12
1	ADAM15	0	-2.50	0	0	Q13444	ADAM15
1	ADAM17	0	-2.15	0	0	P78536	ADAM17
1	ADAM19	1.902E-114	70.42	1.106E-109	3.569E-113	Q9H013	ADAM19
1	ADAM20P1	0.00408789	2.87	1	0.01502457		

1	ADAM22	0	-1.86	0	0	Q9P0K1	ADAM22
1	ADAM23	7.4867E-17	-1.85	4.3553E-12	5.3525E-16	O75077	ADAM23
1	ADAM28	2.7425E-07	-4.60	0.015954	1.3569E-06	Q9UKQ2	ADAM28
1	ADAM32	1.7831E-08	2.10	0.00103729	9.4034E-08	Q8TC27	ADAM32
1	ADAM5	0.00960734	8.30	1	0.03376615	Q6NVV9	ADAM5
1	ADAM8	7.6978E-42	-6.17	4.4781E-37	8.2622E-41	P78325	ADAM8
1	ADAMTS12	7.8108E-73	23.24	4.5438E-68	1.0941E-71	P58397	ADAMTS12
1	ADAMTS13	4.7846E-22	-2.06	2.7834E-17	3.7916E-21	Q76LX8	ADAMTS13
1	ADAMTS15	2.6146E-51	-3.97	1.521E-46	3.0947E-50	Q8TE58	ADAMTS15
1	ADAMTS16	2.321E-111	85.73	1.35E-106	4.29E-110	Q8TE57	ADAMTS16
1	ADAMTS18	2.5572E-07	8.70	0.01487642	1.2674E-06	Q8TE60	ADAMTS18
1	ADAMTS19	3.7597E-27	-9.17	2.1872E-22	3.2837E-26	Q8TE59	ADAMTS19
1	ADAMTS19-AS	6.4802E-05	-30.94	1	0.00027728		
1	ADAMTS2	1.0855E-05	3.47	0.63150052	4.8912E-05	O95450	ADAMTS2
1	ADAMTS20	5.1657E-62	-10.14	3.0051E-57	6.6884E-61	P59510	ADAMTS20
1	ADAMTS3	6.119E-201	-5.35	3.559E-196	1.549E-199	O15072	ADAMTS3
1	ADAMTS5	0.00015219	12.64	1	0.0006333	Q9UNA0	ADAMTS5
1	ADAMTS6	1.197E-85	3.07	6.9635E-81	1.8574E-84	Q9UKP5	ADAMTS6
1	ADAMTS7	4.2037E-38	-3.22	2.4454E-33	4.3001E-37	Q9UKP4	ADAMTS7
1	ADAMTS7P4	3.574E-07	-4.33	0.02079148	1.7575E-06		
1	ADAMTS8	0.00434327	-17.87	1	0.01591491	Q9UP79	ADAMTS8
1	ADAMTSL1	2.5571E-06	-2.65	0.14875503	1.1984E-05	Q8N6G6	ADAMTSL1
1	ADAMTSL3	2.6935E-12	-6.84	1.5669E-07	1.6657E-11	P82987	ADAMTSL3
1	ADAMTSL4	0	-33.27	0	0	Q6UY14	ADAMTSL4
1	ADAMTSL4-AS	8.3659E-25	-5.30	4.8668E-20	6.9985E-24		
1	ADAMTSL5	1.357E-131	4.05	7.894E-127	2.749E-130	Q6ZMM2	ADAMTSL5
1	ADAP1	2.4444E-78	-7.82	1.422E-73	3.5701E-77	O75689	ADAP1
1	ADAP2	7.1714E-08	-2.50	0.00417188	3.656E-07	Q9NPF8	ADAP2
1	ADARB1	3.672E-22	1.95	2.1362E-17	2.9151E-21	P78563	ADARB1
1	ADARB2	0.0042188	-2.58	1	0.01547541	Q9NS39	ADARB2
1	ADAT3	3.7508E-25	2.86	2.182E-20	3.1572E-24	Q96EY9	ADAT3
1	ADCK1	3.3119E-25	1.83	1.9266E-20	2.791E-24	Q86TW2	ADCK1
1	ADCK2	4.534E-67	2.28	2.6376E-62	6.0887E-66	Q7Z695	ADCK2
1	ADCK5	0	-1.76	0	0	Q3MIX3	ADCK5
1	ADCY1	4.0458E-05	-6.34	1	0.00017555	Q08828	ADCY1
1	ADCY10P1	0	-2.46	0	0		
1	ADCY5	7.825E-05	1.92	1	0.00033298	O95622	ADCY5
1	ADCY7	0	-3.89	0	0	P51828	ADCY7
1	ADCY9	2.0387E-49	2.26	1.186E-44	2.3739E-48	O60503	ADCY9
1	ADD2	0	-9.99	0	0	P35612	ADD2
1	ADD3	0	-5.00	0	0	Q9UEY8	ADD3
1	ADD3-AS1	7.396E-06	-6.95	0.43025354	3.3685E-05		
1	ADGRA3	0	-3.62	0	0	Q8IWK6	ADGRA3
1	ADGRB1	5.801E-13	3.79	3.3746E-08	3.6693E-12		
1	ADGRB2	2.2571E-35	-2.84	1.3131E-30	2.2361E-34	O60241	ADGRB2
1	ADGRB3	1.6378E-22	-2.51	9.5276E-18	1.3114E-21	O60242	ADGRB3
1	ADGRD1	0.00050454	4.35	1	0.0020152	Q6QNK2	ADGRD1

1	ADGRD2	5.8177E-27	-12.79	3.3844E-22	5.0559E-26	Q7Z7M1	ADGRD2
1	ADGRE1	1.2557E-07	-4.59	0.00730476	6.3278E-07	Q14246	ADGRE1
1	ADGRE3	1.9553E-11	-1.53	1.1375E-06	1.1708E-10	Q9BY15	ADGRE3
1	ADGRF1	0	-19.99	0	0	Q5T601	ADGRF1
1	ADGRF2	5.0504E-24	-6.46	2.938E-19	4.1568E-23	Q8IZF7	ADGRF2
1	ADGRF4	9.7292E-36	-5.17	5.6599E-31	9.68E-35	Q8IZF3	ADGRF4
1	ADGRF5P1	3.2047E-06	-40.27	0.18642776	1.4915E-05		
1	ADGRG1	0	-9.74	0	0	Q9Y653	ADGRG1
1	ADGRG2	3.1824E-19	-4.23	1.8513E-14	2.3888E-18	Q8IZP9	ADGRG2
1	ADGRG3	0.00137326	-10.67	1	0.00528569	Q86Y34	ADGRG3
1	ADGRG5	1.3947E-05	-6.17	0.81132596	6.2414E-05	Q8IZF4	ADGRG5
1	ADGRG7	7.8104E-05	-5.33	1	0.00033238	Q96K78	ADGRG7
1	ADGRL1	3.929E-16	1.56	2.2856E-11	2.7574E-15	O94910	ADGRL1
1	ADGRL2	0	-8.21	0	0	O95490	ADGRL2
1	ADGRL3	7.1244E-98	-5.68	4.1446E-93	1.2087E-96	Q9HAR2	ADGRL3
1	ADGRV1	1.511E-235	-8.92	8.792E-231	4.07E-234		
1	ADH6	0.01106152	-4.60	1	0.03862039	P28332	ADH6
1	ADHFE1	2.012E-107	-6.71	1.171E-102	3.621E-106	Q8IWW8	ADHFE1
1	ADI1P2	0.00573247	-21.39	1	0.02070536		
1	ADIPOR1	0	-1.80	0	0	Q96A54	ADIPOR1
1	ADIRF	0	-4.86	0	0	Q15847	ADIRF
1	ADM	2.0855E-14	1.51	1.2132E-09	1.3823E-13	P35318	ADM
1	ADM2	1.6049E-14	-4.24	9.3363E-10	1.0674E-13	Q7Z4H4	ADM2
1	ADM5	4.0157E-49	5.06	2.3361E-44	4.6638E-48	C9JUS6	ADM5
1	ADO	1.2659E-50	2.23	7.3645E-46	1.4893E-49	Q96SZ5	ADO
1	ADORA1	0.0130625	5.47	1	0.0452024	P30542	ADORA1
1	ADPRH	5.3852E-07	-2.43	0.03132794	2.6236E-06	P54922	ADPRH
1	ADPRHL1	4.505E-108	-4.09	2.621E-103	8.14E-107	Q8NDY3	ADPRHL1
1	ADPRHL2	3.2785E-50	1.65	1.9072E-45	3.8445E-49	Q9NX46	ADPRHL2
1	ADRA1D	0.00117384	10.47	1	0.0045443	P25100	ADRA1D
1	ADRB1	0.0001453	-3.77	1	0.00060594	P08588	ADRB1
1	ADRB2	4.428E-216	-4.34	2.576E-211	1.161E-214	P07550	ADRB2
1	ADRM1	3.071E-99	1.90	1.7866E-94	5.2578E-98	Q16186	ADRM1
1	ADSL	2.0413E-59	2.99	1.1875E-54	2.5906E-58	P30566	ADSL
1	ADSSL1	2.261E-224	-10.21	1.315E-219	6.019E-223	Q8N142	ADSSL1
1	AEN	5.643E-120	2.39	3.283E-115	1.091E-118	Q8WTP8	AEN
1	AES	7.2783E-76	3.84	4.2341E-71	1.0431E-74	Q08117	AES
1	AF001548.5	0.00068635	1.91	1	0.00270935		
1	AF011889.5	2.8075E-17	2.08	1.6333E-12	2.0241E-16		
1	AF064858.10	2.4036E-05	-19.55	1	0.00010587		
1	AF064858.11	4.9714E-18	-137.70	2.892E-13	3.6447E-17		
1	AF064858.6	1.0459E-33	-16.95	6.0842E-29	1.0164E-32		
1	AF064858.8	1.1005E-55	-50.49	6.4021E-51	1.3598E-54		
1	AF121898.3	2.275E-66	-15.02	1.3234E-61	3.0424E-65		
1	AF131217.1	0.0077335	-2.20	1	0.0275414		
1	AF146191.4	2.1422E-33	-7.75	1.2462E-28	2.0708E-32		
1	AF196972.3	9.9203E-08	-8.34	0.00577102	5.0222E-07		

1	AF196972.9	5.6864E-08	-3.07	0.00330803	2.9164E-07		
1	AFAP1	2.8628E-28	1.69	1.6654E-23	2.559E-27	Q8N556	AFAP1
1	AFAP1-AS1	1.872E-110	-7.53	1.089E-105	3.44E-109		
1	AFAP1L2	0	-12.56	0	0	Q8N4X5	AFAP1L2
1	AFDN-AS1	0.00026085	-2.76	1	0.00106578	Q9Y6Z5	AFDN-AS1
1	AFF2	0.00224883	-4.34	1	0.00846205	P51816	AFF2
1	AFF4	9.7707E-24	1.51	5.684E-19	8.0012E-23	Q9UHB7	AFF4
1	AGA	0	-2.79	0	0	P20933	AGA
1	AGAP11	9.8501E-47	-6.93	5.7302E-42	1.1168E-45	Q8TF27	AGAP11
1	AGAP12P	4.6957E-40	-7.97	2.7317E-35	4.9192E-39		
1	AGAP14	3.2785E-21	10.79	1.9072E-16	2.559E-20	A8MT82	AGAP14
1	AGAP2	1.283E-174	-7.06	7.466E-170	2.979E-173	Q99490	AGAP2
1	AGAP2-AS1	1.2741E-51	2.44	7.4118E-47	1.5123E-50		
1	AGAP4	1.6911E-10	1.69	9.8381E-06	9.7417E-10	Q96P64	AGAP4
1	AGAP6	1.0782E-15	1.67	6.2726E-11	7.439E-15	Q5VW22	AGAP6
1	AGAP7P	3.0192E-37	-4.79	1.7564E-32	3.0562E-36	Q5VUJ5	AGAP7P
1	AGAP9	5.502E-215	6.50	3.201E-210	1.438E-213	Q5VTM2	AGAP9
1	AGBL1	0.00916641	-3.74	1	0.03233171	Q96MI9	AGBL1
1	AGBL2	1.0577E-24	-2.65	6.1532E-20	8.8294E-24	Q5U5Z8	AGBL2
1	AGBL4	0.0073282	-2.38	1	0.02615722	Q5VU57	AGBL4
1	AGFG2	0	-1.84	0	0	O95081	AGFG2
1	AGK	1.2908E-49	1.59	7.5092E-45	1.5061E-48	Q53H12	AGK
1	AGMAT	6.6895E-67	-7.21	3.8916E-62	8.9771E-66	Q9BSE5	AGMAT
1	AGMO	8.2096E-44	-21.64	4.7758E-39	9.0417E-43	Q6ZNB7	AGMO
1	AGO4	2.2204E-16	-1.53	1.2917E-11	1.5667E-15	Q9HCK5	AGO4
1	AGPAT3	4.6109E-62	1.87	2.6823E-57	5.9727E-61	Q9NRZ7	AGPAT3
1	AGPAT4	0	-1.76	0	0	Q9NRZ5	AGPAT4
1	AGPAT5	0	-1.89	0	0	Q9NUQ2	AGPAT5
1	AGRN	0	-1.84	0	0	O00468	AGRN
1	AGTPBP1	0	-2.56	0	0	Q9UPW5	AGTPBP1
1	AGTR1	2.7206E-07	-20.36	0.01582658	1.3464E-06	P30556	AGTR1
1	AHCTF1	0	-1.70	0	0	Q8WYP5	AHCTF1
1	AHDC1	0	-2.59	0	0	Q5TGY3	AHDC1
1	AHNAK	0	-2.79	0	0	Q09666	AHNAK
1	AHRR	8.7381E-11	-1.70	5.0833E-06	5.093E-10	A9YTQ3	AHRR
1	AHSA1	1.4098E-52	2.68	8.2013E-48	1.69E-51	O95433	AHSA1
1	AHSA2	0	-1.82	0	0	Q719I0	AHSA2
1	AIF1L	1.329E-256	-11.97	7.729E-252	3.649E-255	Q9BQI0	AIF1L
1	AIFM3	1.0538E-22	-10.36	6.1305E-18	8.4711E-22	Q96NN9	AIFM3
1	AIG1	0	-1.60	0	0	Q9NVV5	AIG1
1	AIM1	0	-9.53	0	0		
1	AIM1L	3.3418E-84	-14.84	1.9441E-79	5.1335E-83		
1	AIM2	1.0843E-30	-3.83	6.308E-26	1.008E-29	O14862	AIM2
1	AJ003147.8	0.00012041	-12.50	1	0.00050541		
1	AJUBA	0	-1.53	0	0	Q96IF1	AJUBA
1	AK5	6.046E-114	39.65	3.517E-109	1.131E-112	Q9Y6K8	AK5
1	AK6	7.8669E-38	1.90	4.5765E-33	8.0191E-37	Q9Y3D8	AK6

1	AK7	0.00879686	1.53	1	0.03109231	Q96M32	AK7
1	AK8	8.8455E-14	-2.93	5.1458E-09	5.7495E-13	Q96MA6	AK8
1	AKAP12	0	38.29	0	0	Q02952	AKAP12
1	AKAP17BP	0.00229784	7.21	1	0.00863644		
1	AKAP2	0	118.45	0	0		
1	AKAP5	4.5947E-05	1.86	1	0.00019868	P24588	AKAP5
1	AKAP8P1	0.00032464	-29.01	1	0.00131607		
1	AKAP9	7.0025E-10	-1.53	4.0736E-05	3.9264E-09	Q99996	AKAP9
1	AKIP1	5.852E-115	2.76	3.404E-110	1.101E-113	Q9NQ31	AKIP1
1	AKNA	6.2902E-92	-3.95	3.6592E-87	1.0238E-90	Q7Z591	AKNA
1	AKNAD1	0.00969625	2.87	1	0.03405807		
1	AKR1B10	2.8983E-23	-13.12	1.6861E-18	2.3529E-22	O60218	AKR1B10
1	AKR1B15	2.349E-10	-14.37	1.3665E-05	1.3443E-09	C9JRZ8	AKR1B15
1	AKR1C1	0	-2.81	0	0	Q04828	AKR1C1
1	AKR1C2	2.2474E-48	-3.60	1.3074E-43	2.5931E-47	P52895	AKR1C2
1	AKR1C3	5.639E-119	-5.83	3.28E-114	1.086E-117	P42330	AKR1C3
1	AKR1C7P	1.5704E-08	-7.59	0.00091358	8.2985E-08		
1	AKR7A2	3.7161E-38	1.74	2.1618E-33	3.8034E-37	O43488	AKR7A2
1	AKR7L	0.00328118	1.96	1	0.01216335	Q8NHP1	AKR7L
1	AKT1	1.2101E-49	2.81	7.0399E-45	1.4122E-48	P31749	AKT1
1	AKT1S1	5.2703E-42	1.61	3.0659E-37	5.6682E-41	Q96B36	AKT1S1
1	AKT3	1.3622E-69	5.43	7.9247E-65	1.8607E-68	Q9Y243	AKT3
1	AL021546.6	0.00209838	3.72	1	0.00792362		
1	AL022341.3	0.00026342	-4.16	1	0.00107555		
1	AL022344.7	0.00153155	-23.50	1	0.00586237		
1	AL078471.5	1.5053E-54	-15.16	8.7567E-50	1.8427E-53		
1	AL136419.6	1.8155E-05	1.63	1	8.0628E-05		
1	AL157871.2	7.8774E-06	3.48	0.45825819	3.5821E-05		
1	AL163953.3	3.6785E-11	-2.96	2.14E-06	2.1743E-10		
1	AL450992.2	1.753E-27	-8.48	1.0198E-22	1.5433E-26		
1	AL591893.1	3.4674E-23	-8.30	2.0172E-18	2.809E-22		
1	AL672294.1	3.0078E-08	-7.43	0.00174978	1.5666E-07		
1	AL928654.7	1.6586E-07	18.25	0.00964866	8.2999E-07		
1	ALB	1.4221E-06	-8.43	0.08273016	6.769E-06	P02768	ALB
1	ALDH1A2	3.7987E-39	-7.72	2.2099E-34	3.9385E-38	O94788	ALDH1A2
1	ALDH1A3	0	-5.03	0	0	P47895	ALDH1A3
1	ALDH1B1	5.6809E-40	3.71	3.3048E-35	5.9375E-39	P30837	ALDH1B1
1	ALDH1L1	6.7715E-12	-10.28	3.9392E-07	4.1231E-11	O75891	ALDH1L1
1	ALDH1L1-AS2	8.3112E-05	-30.92	1	0.00035281		
1	ALDH1L2	6.223E-258	-10.48	3.62E-253	1.715E-256	Q3SY69	ALDH1L2
1	ALDH2	1.801E-301	-11.29	1.048E-296	5.06E-300	P05091	ALDH2
1	ALDH3A1	0	-15.39	0	0	P30838	ALDH3A1
1	ALDH3A2	0	-1.75	0	0	P51648	ALDH3A2
1	ALDH3B1	0	-4.64	0	0	P43353	ALDH3B1
1	ALDH3B2	3.1095E-76	-52.19	1.8089E-71	4.4742E-75	P48448	ALDH3B2
1	ALDH4A1	0	-1.57	0	0	P30038	ALDH4A1
1	ALDH6A1	3.3698E-57	2.11	1.9603E-52	4.2004E-56	Q02252	ALDH6A1

1	ALDH7A1	0	-1.57	0	0	P49419	ALDH7A1
1	ALDH8A1	1.8301E-11	3.26	1.0646E-06	1.0972E-10	Q9H2A2	ALDH8A1
1	ALDOC	0	-2.62	0	0	P09972	ALDOC
1	ALG10B	0	-2.53	0	0	Q5I7T1	ALG10B
1	ALG1L	2.7853E-07	-1.91	0.0162034	1.3775E-06	Q6GMV1	ALG1L
1	ALG1L11P	0.01258215	3.37	1	0.04362841		
1	ALG1L12P	1.4089E-07	3.36	0.00819591	7.0844E-07		
1	ALG1L2	0.00554889	-1.53	1	0.0200872	C9J202	ALG1L2
1	ALG1L6P	1.257E-105	310.43	7.31E-101	2.239E-104		
1	ALG1L8P	2.8447E-05	-2.93	1	0.00012467		
1	ALG1L9P_1	6.5569E-08	-1.67	0.00381439	3.3507E-07		
1	ALK	4.412E-15	-5.89	2.5666E-10	2.9855E-14	Q9UM73	ALK
1	ALKBH1	3.8145E-61	2.04	2.2191E-56	4.9018E-60	Q13686	ALKBH1
1	ALKBH2	1.1229E-24	1.68	6.5326E-20	9.3671E-24	Q6NS38	ALKBH2
1	ALKBH3	1.6432E-43	1.81	9.5593E-39	1.8033E-42	Q96Q83	ALKBH3
1	ALKBH7	5.3993E-38	2.26	3.141E-33	5.5163E-37	Q9BT30	ALKBH7
1	ALKBH8	5.6587E-80	2.26	3.2919E-75	8.3891E-79	Q96BT7	ALKBH8
1	ALOX12-AS1	5.3291E-15	-1.87	3.1001E-10	3.5939E-14		
1	ALOX12B	6.0854E-11	-9.42	3.5401E-06	3.564E-10	O75342	ALOX12B
1	ALOX12P2	3.3101E-74	-21.57	1.9256E-69	4.6806E-73		
1	ALOX15	0.00437502	-5.64	1	0.01601908	P16050	ALOX15
1	ALOX5	0.00021593	-2.71	1	0.00088923	P09917	ALOX5
1	ALOXE3	1.3538E-30	-12.30	7.8758E-26	1.2563E-29	Q9BYJ1	ALOXE3
1	ALPK1	0	-6.75	0	0	Q96QP1	ALPK1
1	ALPK2	5.396E-78	53.68	3.1391E-73	7.8614E-77	Q86TB3	ALPK2
1	ALPP	5.3721E-23	-15.90	3.1252E-18	4.3399E-22	P05187	ALPP
1	ALPPL2	0.01197491	-6.41	1	0.04164425	P10696	ALPPL2
1	ALS2CL	0	-11.97	0	0	Q60I27	ALS2CL
1	ALS2CR11	3.9402E-18	-5.35	2.2922E-13	2.8934E-17		
1	ALS2CR12	7.7639E-10	1.93	4.5166E-05	4.3436E-09	Q96Q35	ALS2CR12
1	ALX1	2.5744E-19	-7.67	1.4976E-14	1.9354E-18	Q15699	ALX1
1	ALX4	2.1948E-06	2.10	0.12768288	1.0335E-05	Q9H161	ALX4
1	ALYREF	3.765E-121	2.92	2.19E-116	7.333E-120	Q86V81	ALYREF
1	AMD1	1.08E-83	2.12	6.283E-79	1.6495E-82	P17707	AMD1
1	AMDHD1	4.9143E-13	-2.32	2.8589E-08	3.1139E-12	Q96NU7	AMDHD1
1	AMDHD2	5.5579E-53	2.96	3.2332E-48	6.6955E-52	Q9Y303	AMDHD2
1	AMFR	0	-1.65	0	0	Q9UKV5	AMFR
1	AMH	1.5523E-20	2.32	9.0303E-16	1.1948E-19	P03971	AMH
1	AMIGO2	1.91E-111	1.98	1.111E-106	3.533E-110	Q86SJ2	AMIGO2
1	AMN	3.3067E-77	-11.52	1.9237E-72	4.7876E-76	Q9BXJ7	AMN
1	AMOT	6.974E-124	22.24	4.057E-119	1.374E-122	Q4VCS5	AMOT
1	AMOTL1	0	-1.78	0	0	Q8IY63	AMOTL1
1	AMPD2	7.52E-87	1.69	4.3747E-82	1.1776E-85	Q01433	AMPD2
1	AMPD3	6.6613E-16	-1.55	3.8752E-11	4.6304E-15	Q01432	AMPD3
1	AMT	2.1733E-06	-2.82	0.12642826	1.0238E-05	P48728	AMT
1	AMTN	5.8882E-11	107.72	3.4254E-06	3.4499E-10	Q6UX39	AMTN
1	AMY2B	4.3049E-39	-3.11	2.5043E-34	4.4593E-38	P19961	AMY2B

1	AMZ1	3.355E-09	-1.64	0.00019517	1.8273E-08	Q400G9	AMZ1
1	AMZ2	1.0061E-92	2.33	5.853E-88	1.6473E-91	Q86W34	AMZ2
1	AMZ2P1	1.8597E-37	2.19	1.0819E-32	1.8871E-36		
1	ANG	2.1238E-22	3.00	1.2355E-17	1.6945E-21	P03950	ANG
1	ANGEL2	0	-1.70	0	0	Q5VTE6	ANGEL2
1	ANGPT1	1.391E-158	-6.53	8.093E-154	3.081E-157	Q15389	ANGPT1
1	ANGPTL2	1.2013E-06	-4.52	0.06988499	5.7438E-06	Q9UKU9	ANGPTL2
1	ANGPTL4	2.407E-13	-1.54	1.4002E-08	1.5402E-12	Q9BY76	ANGPTL4
1	ANK1	1.969E-135	-5.20	1.145E-130	4.049E-134	P16157	ANK1
1	ANK2	1.4446E-18	-1.75	8.404E-14	1.0703E-17	Q01484	ANK2
1	ANK3	1.9539E-72	-12.46	1.1367E-67	2.7284E-71	Q12955	ANK3
1	ANKDD1B	9.8869E-12	2.93	5.7516E-07	5.9869E-11	A6NHY2	ANKDD1B
1	ANKFN1	0.00025458	2.15	1	0.00104143		
1	ANKH	0	-1.86	0	0	Q9HCJ1	ANKH
1	ANKIB1	0	-1.67	0	0	Q9P2G1	ANKIB1
1	ANKLE1	1.3819E-12	3.70	8.0393E-08	8.6193E-12	Q8NAG6	ANKLE1
1	ANKMY2	6.0481E-58	1.88	3.5184E-53	7.5894E-57	Q8IV38	ANKMY2
1	ANKRD1	0	148.32	0	0	Q15327	ANKRD1
1	ANKRD13D	0	-2.99	0	0	Q6ZTN6	ANKRD13D
1	ANKRD18A	7.377E-214	132.95	4.292E-209	1.922E-212		
1	ANKRD18B	6.5933E-10	1.62	3.8356E-05	3.702E-09		
1	ANKRD18CP	1.7827E-08	-3.08	0.00103709	9.4025E-08		
1	ANKRD2	3.792E-168	-8.91	2.206E-163	8.634E-167	Q9GZV1	ANKRD2
1	ANKRD20A17	0.00107742	-2.51	1	0.00418466		
1	ANKRD20A18	2.3913E-29	-18.44	1.3911E-24	2.1736E-28		
1	ANKRD20A21	2.8105E-07	-1.89	0.0163499	1.3896E-06		
1	ANKRD20A4	2.5814E-07	7.83	0.01501726	1.2788E-06		
1	ANKRD20A9P	3.176E-121	40.68	1.848E-116	6.19E-120		
1	ANKRD22	4.843E-229	-26.23	2.817E-224	1.296E-227		
1	ANKRD28	1.2593E-21	1.61	7.3259E-17	9.9105E-21	O15084	ANKRD28
1	ANKRD29	5.0292E-07	-2.11	0.02925676	2.4548E-06		
1	ANKRD31	1.5938E-13	-2.82	9.2718E-09	1.0267E-12		
1	ANKRD33B	0	-3.14	0	0		
1	ANKRD36C	1.2944E-26	2.32	7.5303E-22	1.1179E-25	Q5JPF3	ANKRD36C
1	ANKRD37	4.4441E-31	-3.61	2.5853E-26	4.1545E-30	Q7Z713	ANKRD37
1	ANKRD39	1.1612E-32	2.09	6.7553E-28	1.1114E-31	Q53RE8	ANKRD39
1	ANKRD50	2.169E-120	3.22	1.262E-115	4.202E-119	Q9ULJ7	ANKRD50
1	ANKRD54	4.4384E-30	1.55	2.582E-25	4.0854E-29	Q6NXT1	ANKRD54
1	ANKRD62	0.00352075	-6.15	1	0.01302072		
1	ANKRD65	1.529E-160	-17.69	8.892E-156	3.402E-159		
1	ANKRD7	3.9857E-26	-16.44	2.3186E-21	3.4123E-25	Q92527	ANKRD7
1	ANKS1A	0	-1.76	0	0	Q92625	ANKS1A
1	ANKS1B	0.0041764	2.11	1	0.0153334	Q7Z6G8	ANKS1B
1	ANKS3	8.6309E-31	1.64	5.0209E-26	8.036E-30	Q6ZW76	ANKS3
1	ANKS6	0	-1.90	0	0	Q68DC2	ANKS6
1	ANLN	0	-2.46	0	0	Q9NQW6	ANLN
1	ANO1	0	-7.41	0	0	Q5XXA6	ANO1

1	ANO1-AS2	3.0484E-06	-5.24	0.17734032	1.4208E-05		
1	ANO10	7.6146E-81	2.14	4.4297E-76	1.1379E-79	Q9NW15	ANO10
1	ANO2	0.00503954	-3.03	1	0.01832886	Q9NQ90	ANO2
1	ANO3	9.1187E-06	-7.38	0.53047298	4.1301E-05	Q9BYT9	ANO3
1	ANO6	0	-1.62	0	0	Q4KMQ2	ANO6
1	ANO7	0.00013044	-1.59	1	0.00054571	Q6IWH7	ANO7
1	ANO7L1	3.2917E-14	-3.80	1.9149E-09	2.1684E-13		
1	ANO8	1.112E-11	1.62	6.469E-07	6.721E-11	Q9HCE9	ANO8
1	ANO9	8.05E-295	-15.88	4.683E-290	2.258E-293	A1A5B4	ANO9
1	ANOS1	4.5225E-14	7.63	2.6309E-09	2.9664E-13	P23352	ANOS1
1	ANPEP	2.2905E-39	-8.72	1.3324E-34	2.3811E-38	P15144	ANPEP
1	ANTXR2	2.2708E-25	1.66	1.321E-20	1.9192E-24	P58335	ANTXR2
1	ANXA1	0	-2.04	0	0	P04083	ANXA1
1	ANXA13	7.2464E-05	-8.19	1	0.00030894	P27216	ANXA13
1	ANXA4	0	-1.55	0	0	P09525	ANXA4
1	ANXA5	2.1395E-64	2.06	1.2447E-59	2.8217E-63	P08758	ANXA5
1	ANXA6	0	56.48	0	0	P08133	ANXA6
1	ANXA7	6.1139E-34	1.74	3.5567E-29	5.9616E-33	P20073	ANXA7
1	ANXA8	0	-4.14	0	0	P13928	ANXA8
1	ANXA8L1	0	-5.70	0	0	Q5VT79	ANXA8L1
1	ANXA9	1.102E-194	-5.13	6.412E-190	2.733E-193	O76027	ANXA9
1	AOC2	1.4278E-50	3.87	8.306E-46	1.6787E-49	O75106	AOC2
1	AOC3	5.0271E-62	6.42	2.9245E-57	6.5104E-61	Q16853	AOC3
1	AOX1	1.6064E-25	8.64	9.3451E-21	1.3603E-24	Q06278	AOX1
1	AP000230.1	0.00361295	-4.12	1	0.01333121		
1	AP000251.3	2.2752E-21	15.54	1.3236E-16	1.7809E-20		
1	AP000266.7	0.00011659	40.13	1	0.00048997		
1	AP000275.65	3.8532E-06	6.83	0.22415593	1.7834E-05		
1	AP000318.2	1.9296E-08	-97.91	0.00112252	1.0152E-07		
1	AP000347.4	0.00127587	-2.04	1	0.00492517		
1	AP000350.10	6.3362E-16	-3.07	3.686E-11	4.4128E-15		
1	AP000350.5	4.1486E-22	-15.19	2.4134E-17	3.2898E-21		
1	AP000350.8	1.1609E-08	-4.81	0.00067537	6.1689E-08		
1	AP000351.13	0.00942764	-18.16	1	0.03316063		
1	AP000351.3	4.4205E-17	-5.43	2.5716E-12	3.1728E-16		
1	AP000351.4	4.3262E-15	-11.76	2.5167E-10	2.9288E-14		
1	AP000432.1	1.1661E-06	-3.43	0.06783956	5.5807E-06		
1	AP000439.3	0.00056894	-25.34	1	0.00226385		
1	AP000442.1	5.0444E-09	-2.21	0.00029345	2.7237E-08		
1	AP000487.5	1.2883E-23	-2.68	7.4947E-19	1.0529E-22		
1	AP000487.6	1.7782E-05	-6.52	1	7.9008E-05		
1	AP000640.2	7.0621E-05	-4.23	1	0.00030126		
1	AP000688.14	0.00834404	-1.67	1	0.02959073		
1	AP000692.9	0.00894074	-2.98	1	0.03157019		
1	AP000695.6	0.00068021	1.91	1	0.00268622		
1	AP000721.4	1.8175E-19	-3.38	1.0573E-14	1.3712E-18		
1	AP001042.1	0.00232718	-7.68	1	0.00874104		

1	AP001065.15	7.5653E-46	121.92	4.401E-41	8.5027E-45		
1	AP001258.4	4.2894E-81	-5.08	2.4953E-76	6.4196E-80		
1	AP001347.6	2.2965E-19	3.33	1.3359E-14	1.7287E-18		
1	AP001372.2	2.1217E-28	-3.12	1.2343E-23	1.8992E-27		
1	AP001432.14	4.188E-05	-1.80	1	0.00018148		
1	AP001437.1	0.01043412	-2.73	1	0.03653732		
1	AP001468.58	5.4442E-14	-11.21	3.1671E-09	3.5614E-13		
1	AP001469.5	0.00019027	-3.61	1	0.00078754		
1	AP001469.7	0.00126038	-2.66	1	0.00486701		
1	AP001469.9	3.2317E-21	-2.57	1.88E-16	2.5228E-20		
1	AP001505.10	6.1812E-08	2.47	0.00359583	3.1628E-07		
1	AP001631.10	0.01045711	-4.50	1	0.03661123		
1	AP003419.11	0.01263023	-2.45	1	0.04377167		
1	AP003774.1	0.00038535	-7.47	1	0.00155352		
1	AP003774.4	9.7661E-05	-5.21	1	0.00041271		
1	AP003900.6	5.4058E-08	-2.85	0.00314477	2.7754E-07		
1	AP004372.1	4.8621E-08	91.20	0.00282847	2.5024E-07		
1	AP006222.2	4.078E-109	6.30	2.372E-104	7.419E-108		
1	AP1M1	2.5965E-22	2.55	1.5105E-17	2.0678E-21	Q9BXS5	AP1M1
1	AP1M2	0	-9.63	0	0	Q9Y6Q5	AP1M2
1	AP1S2	4.077E-134	5.91	2.372E-129	8.331E-133	P56377	AP1S2
1	AP2A2	2.994E-74	1.93	1.7417E-69	4.2378E-73	O94973	AP2A2
1	AP2S1	1.2881E-35	1.87	7.4934E-31	1.2807E-34	P53680	AP2S1
1	AP3S2	0	-1.64	0	0	P59780	AP3S2
1	AP4B1	6.683E-127	2.39	3.888E-122	1.334E-125	Q9Y6B7	AP4B1
1	AP4B1-AS1	1.5924E-09	2.40	9.2638E-05	8.7892E-09		
1	AP4S1	1.5284E-47	2.30	8.8916E-43	1.7476E-46	Q9Y587	AP4S1
1	AP5S1	2.182E-18	1.53	1.2694E-13	1.6094E-17	Q9NUS5	AP5S1
1	APBA1	5.0254E-21	-3.51	2.9235E-16	3.9068E-20	Q02410	APBA1
1	APBA2	2.718E-119	141.45	1.581E-114	5.243E-118	Q99767	APBA2
1	APBB1	2.059E-157	50.59	1.198E-152	4.537E-156	O00213	APBB1
1	APBB3	3.6901E-15	1.53	2.1467E-10	2.5069E-14	O95704	APBB3
1	APCDD1L-AS1	2.4919E-07	2.60	0.01449649	1.2355E-06		
1	APEX1	1.268E-108	2.37	7.378E-104	2.301E-107	P27695	APEX1
1	APH1A	0	-1.88	0	0	Q96BI3	APH1A
1	APITD1-CORT	1.8386E-06	2.07	0.10695601	8.6984E-06		
1	APLF	2.4909E-32	-2.62	1.4491E-27	2.3709E-31	Q8IW19	APLF
1	APLN	6.482E-137	-7.16	3.771E-132	1.342E-135	Q9ULZ1	APLN
1	APLP1	4.0419E-23	2.39	2.3513E-18	3.2712E-22	P51693	APLP1
1	APOA2	0.00277121	-6.72	1	0.01034276	P02652	APOA2
1	APOBEC3A	3.3734E-13	-4.69	1.9625E-08	2.1488E-12	P31941	APOBEC3A
1	APOBEC3B	0	-1.73	0	0	Q9UH17	APOBEC3B
1	APOBEC3D	1.2606E-21	-5.63	7.3336E-17	9.9196E-21	Q96AK3	APOBEC3D
1	APOBEC3F	1.8663E-50	-3.00	1.0857E-45	2.1938E-49	Q8IUX4	APOBEC3F
1	APOBEC3G	2.9207E-94	-16.39	1.6991E-89	4.8352E-93	Q9HC16	APOBEC3G
1	APOBEC3H	3.6809E-07	-11.03	0.02141315	1.8087E-06	Q6NTF7	APOBEC3H
1	APOBR	0.01323927	-3.53	1	0.04578691	Q0VD83	APOBR

1	APOC1	1.3153E-06	-2.58	0.07651467	6.2743E-06	P02654	APOC1
1	APOE	1.1719E-13	-2.72	6.8174E-09	7.5833E-13	P02649	APOE
1	APOF	0.002041	-23.38	1	0.00771246	Q13790	APOF
1	APOL1	1.69E-214	-10.81	9.829E-210	4.412E-213	O14791	APOL1
1	APOL3	1.6528E-69	-37.65	9.6151E-65	2.2555E-68	O95236	APOL3
1	APOL6	2.0548E-61	-8.78	1.1954E-56	2.647E-60	Q9BWW8	APOL6
1	APOPT1	1.1609E-86	3.03	6.7537E-82	1.817E-85	Q96IL0	APOPT1
1	APTX	4.1064E-48	1.63	2.3888E-43	4.7295E-47	Q7Z2E3	APTX
1	AQP1	3.4272E-17	-4.67	1.9937E-12	2.4669E-16	P29972	AQP1
1	AQP11	0.00013887	1.93	1	0.00057982	Q8NBQ7	AQP11
1	AQP3	0	-8.23	0	0	Q92482	AQP3
1	AQP4-AS1	1.2911E-06	-6.66	0.0751086	6.164E-06		
1	AQP6	0.01397778	2.41	1	0.04825492	Q13520	AQP6
1	AQP7	6.5299E-14	-11.59	3.7987E-09	4.261E-13	O14520	AQP7
1	AQP7P1	1.7068E-12	19.33	9.929E-08	1.0624E-11		
1	AQP7P3	0.00272001	4.55	1	0.01015623	A6NL99	AQP7P3
1	ARAF	0	-1.63	0	0	P10398	ARAF
1	ARAP1	0	-2.60	0	0	Q96P48	ARAP1
1	ARAP1-AS1	0.0035383	-22.37	1	0.01307236		
1	ARAP2	2.84E-234	-12.04	1.652E-229	7.642E-233	Q8WZ64	ARAP2
1	ARAP3	0	-1.73	0	0	Q8WWN8	ARAP3
1	ARC	1.5547E-09	5.63	9.0444E-05	8.5867E-09	Q7LC44	ARC
1	AREG	0	-5.16	0	0	P15514	AREG
1	ARF4	1.3178E-24	1.64	7.6664E-20	1.0985E-23	P18085	ARF4
1	ARF6	2.511E-153	2.48	1.46E-148	5.452E-152	P62330	ARF6
1	ARFGAP1	4.8608E-80	2.30	2.8277E-75	7.2136E-79	Q8N6T3	ARFGAP1
1	ARFGAP3	3.6846E-92	2.00	2.1435E-87	6.0092E-91	Q9NP61	ARFGAP3
1	ARFGEF3	2.4141E-39	2.08	1.4044E-34	2.5064E-38	Q5TH69	ARFGEF3
1	ARFIP2	1.1813E-15	2.31	6.8719E-11	8.1353E-15	P53365	ARFIP2
1	ARFRP1	8.1187E-39	2.84	4.723E-34	8.3771E-38	Q13795	ARFRP1
1	ARG2	1.0914E-35	2.14	6.3489E-31	1.0855E-34	P78540	ARG2
1	ARHGAP11B	0	-2.31	0	0	Q3KRB8	ARHGAP11B
1	ARHGAP17	1.1701E-46	2.49	6.8069E-42	1.3251E-45	Q68EM7	ARHGAP17
1	ARHGAP18	2.4629E-63	6.17	1.4328E-58	3.2212E-62	Q8N392	ARHGAP18
1	ARHGAP21	0	-1.67	0	0	Q5T5U3	ARHGAP21
1	ARHGAP22	7.767E-108	3.00	4.519E-103	1.402E-106	Q7Z5H3	ARHGAP22
1	ARHGAP23	0	-4.83	0	0	Q9P227	ARHGAP23
1	ARHGAP23P1	0.00360227	-10.03	1	0.0132935		
1	ARHGAP24	2.4732E-24	-1.94	1.4388E-19	2.0481E-23	Q8N264	ARHGAP24
1	ARHGAP25	6.4655E-28	-5.57	3.7613E-23	5.7459E-27	P42331	ARHGAP25
1	ARHGAP27	0	-2.13	0	0	Q6ZUM4	ARHGAP27
1	ARHGAP29	1.824E-131	8.54	1.061E-126	3.694E-130	Q52LW3	ARHGAP29
1	ARHGAP30	6.9896E-40	-4.55	4.0661E-35	7.2948E-39	Q7Z6I6	ARHGAP30
1	ARHGAP31	6.495E-109	2.92	3.778E-104	1.181E-107	Q2M1Z3	ARHGAP31
1	ARHGAP32	0	-2.55	0	0	A7KAX9	ARHGAP32
1	ARHGAP4	3.4825E-06	-2.12	0.20259059	1.6162E-05	P98171	ARHGAP4
1	ARHGAP40	3.001E-130	-17.10	1.746E-125	6.052E-129	Q5TG30	ARHGAP40

1	ARHGAP44	5.674E-142	19.97	3.301E-137	1.197E-140	Q17R89	ARHGAP44
1	ARHGAP45	0	-1.69	0	0	Q92619	ARHGAP45
1	ARHGAP5	4.0362E-67	2.43	2.348E-62	5.4226E-66	Q13017	ARHGAP5
1	ARHGAP5-AS1	1.051E-26	3.66	6.1142E-22	9.089E-26		
1	ARHGAP6	0.0120166	3.79	1	0.04176946	O43182	ARHGAP6
1	ARHGAP8	2.1361E-60	-9.32	1.2427E-55	2.7287E-59	P85298	ARHGAP8
1	ARHGAP9	0.00059756	8.51	1	0.00237279	Q9BRR9	ARHGAP9
1	ARHGDIA	3.7951E-28	2.78	2.2077E-23	3.3871E-27	P52565	ARHGDIA
1	ARHGEF10L	2.9072E-17	-1.85	1.6913E-12	2.0952E-16	Q9HCE6	ARHGEF10L
1	ARHGEF16	9.3781E-67	-9.91	5.4556E-62	1.2565E-65	Q5VV41	ARHGEF16
1	ARHGEF18	4.0226E-49	1.61	2.3401E-44	4.6709E-48	Q6ZSZ5	ARHGEF18
1	ARHGEF19	0	-1.94	0	0	Q8IW93	ARHGEF19
1	ARHGEF25	4.0517E-16	-1.77	2.357E-11	2.8426E-15	Q86VW2	ARHGEF25
1	ARHGEF26	3.4776E-27	2.02	2.023E-22	3.0403E-26	Q96DR7	ARHGEF26
1	ARHGEF26-AS	5.0869E-06	-3.33	0.29592711	2.3382E-05		
1	ARHGEF28	2.0268E-30	3.18	1.1791E-25	1.8754E-29	Q8N1W1	ARHGEF28
1	ARHGEF3	0	-5.71	0	0	Q9NR81	ARHGEF3
1	ARHGEF35	1.0622E-27	-2.12	6.1795E-23	9.3956E-27		
1	ARHGEF37	3.1802E-45	-4.36	1.85E-40	3.5503E-44	A1IGU5	ARHGEF37
1	ARHGEF4	2.852E-243	-7.37	1.659E-238	7.768E-242	Q9NR80	ARHGEF4
1	ARHGEF40	2.5392E-53	3.10	1.4771E-48	3.0691E-52	Q8TER5	ARHGEF40
1	ARHGEF5	0	-2.19	0	0	Q12774	ARHGEF5
1	ARHGEF9	0	-2.51	0	0	O43307	ARHGEF9
1	ARID3A	3.349E-173	3.50	1.948E-168	7.738E-172	Q99856	ARID3A
1	ARID4B	0	-1.91	0	0	Q4LE39	ARID4B
1	ARID5A	3.5214E-18	1.89	2.0485E-13	2.5878E-17	Q03989	ARID5A
1	ARID5B	1.0909E-36	1.82	6.3463E-32	1.0984E-35	Q14865	ARID5B
1	ARL10	1.7145E-78	-5.50	9.974E-74	2.5073E-77	Q8N8L6	ARL10
1	ARL11	5.4943E-45	-14.39	3.1962E-40	6.1172E-44	Q969Q4	ARL11
1	ARL14	1.9825E-37	-4.78	1.1533E-32	2.0103E-36	Q8N4G2	ARL14
1	ARL14EP	1.5028E-28	1.71	8.7422E-24	1.3485E-27	Q8N8R7	ARL14EP
1	ARL15	2.0559E-18	1.95	1.196E-13	1.5174E-17	Q9NXU5	ARL15
1	ARL16	3.2128E-26	1.54	1.869E-21	2.7558E-25	Q0P5N6	ARL16
1	ARL17A	1.4154E-10	-1.79	8.2339E-06	8.1816E-10	Q8IVW1	ARL17A
1	ARL17B	1.1478E-06	1.59	0.06677198	5.4938E-06		
1	ARL2	5.0936E-52	1.90	2.9631E-47	6.0757E-51	P36404	ARL2
1	ARL3	6.7209E-50	2.10	3.9098E-45	7.8621E-49	P36405	ARL3
1	ARL4A	1.8674E-29	2.25	1.0863E-24	1.7019E-28	P40617	ARL4A
1	ARL4C	1.6136E-51	3.26	9.3872E-47	1.9138E-50	P56559	ARL4C
1	ARL4D	3.051E-178	-4.67	1.775E-173	7.168E-177	P49703	ARL4D
1	ARL6IP1	0	-1.76	0	0	Q15041	ARL6IP1
1	ARL6IP4	8.1366E-42	1.73	4.7334E-37	8.73E-41	Q66PJ3	ARL6IP4
1	ARMC5	3.3127E-32	1.80	1.9272E-27	3.1479E-31	Q96C12	ARMC5
1	ARMC6	3.4556E-36	3.24	2.0102E-31	3.4581E-35	Q6NXE6	ARMC6
1	ARMC7	3.9342E-25	1.79	2.2887E-20	3.3102E-24	Q9H6L4	ARMC7
1	ARMC9	1.1024E-52	1.98	6.4133E-48	1.324E-51	Q7Z3E5	ARMC9
1	ARMCX1	0.00094999	-4.03	1	0.00370754	Q9P291	ARMCX1

1	ARMCX2	2.9706E-64	-4.48	1.7281E-59	3.9124E-63	Q7L311	ARMCX2
1	ARMCX4	5.2438E-21	-3.05	3.0505E-16	4.0749E-20	Q5H9R4	ARMCX4
1	ARMCX5	8.1061E-20	1.55	4.7157E-15	6.1635E-19	Q6P1M9	ARMCX5
1	ARMCX6	2.271E-35	1.66	1.3211E-30	2.2495E-34	Q7L4S7	ARMCX6
1	ARNT	0	-1.63	0	0	P27540	ARNT
1	ARNT2	5.617E-115	4.41	3.267E-110	1.057E-113	Q9HBZ2	ARNT2
1	ARNTL	2.4677E-26	3.12	1.4355E-21	2.1211E-25	O00327	ARNTL
1	ARNTL2-AS1	0.00076895	-1.73	1	0.00302578		
1	ARPC1B	0	-1.54	0	0	O15143	ARPC1B
1	ARPC3	1.5992E-20	1.63	9.3033E-16	1.2306E-19	O15145	ARPC3
1	ARPC4-TTLL3	1.4844E-09	2.03	8.6354E-05	8.207E-09	A0A0A6YYG9	ARPC4-TTLL3
1	ARPIN	0	-1.81	0	0	Q7Z6K5	ARPIN
1	ARRB1	0.0051366	-1.77	1	0.01866204	P49407	ARRB1
1	ARRB2	1.4117E-47	4.92	8.2122E-43	1.6153E-46	P32121	ARRB2
1	ARRDC1	0	-1.56	0	0	Q8N5I2	ARRDC1
1	ARRDC3-AS1	9.4241E-06	-1.64	0.54823595	4.2638E-05		
1	ARRDC4	7.516E-85	3.79	4.3724E-80	1.1607E-83	Q8NCT1	ARRDC4
1	ARSD	8.2776E-31	23.36	4.8154E-26	7.7084E-30	P51689	ARSD
1	ARSE	1.2779E-07	48.76	0.0074343	6.4366E-07	P51690	ARSE
1	ARSG	1.37E-111	3.99	7.968E-107	2.537E-110	Q96EG1	ARSG
1	ARSI	6.1045E-34	-3.17	3.5512E-29	5.9534E-33	Q5FYB1	ARSI
1	ARSJ	9.418E-102	7.73	5.4785E-97	1.639E-100	Q5FYB0	ARSJ
1	ARTN	0	-18.98	0	0	Q5T4W7	ARTN
1	ARV1	0	-1.77	0	0	Q9H2C2	ARV1
1	ARVCF	0	-2.68	0	0	O00192	ARVCF
1	AS3MT	5.2871E-09	-110.41	0.00030757	2.8521E-08	Q9HBK9	AS3MT
1	ASAH1	0	-2.36	0	0	Q13510	ASAH1
1	ASAP1	5.2228E-28	1.55	3.0383E-23	4.6536E-27	Q9ULH1	ASAP1
1	ASB1	1.1052E-23	2.59	6.4296E-19	9.0393E-23	Q9Y576	ASB1
1	ASB13	0	-1.98	0	0	Q8WXK3	ASB13
1	ASB15	0.00505933	-19.70	1	0.01839511	Q8WXK1	ASB15
1	ASB16	0.00170634	-1.52	1	0.0065019	Q96NS5	ASB16
1	ASB16-AS1	1.7764E-15	-1.58	1.0334E-10	1.2183E-14		
1	ASB2	2.945E-147	-5.25	1.713E-142	6.295E-146	Q96Q27	ASB2
1	ASB4	3.3293E-05	-8.91	1	0.00014539	Q9Y574	ASB4
1	ASB9	2.313E-29	-6.50	1.3456E-24	2.1038E-28	Q96DX5	ASB9
1	ASCL1	1.2586E-10	-9.95	7.3217E-06	7.2911E-10	P50553	ASCL1
1	ASCL5	1.2129E-20	-4.77	7.0557E-16	9.3564E-20	Q7RTU5	ASCL5
1	ASGR1	1.2434E-11	8.28	7.2335E-07	7.5052E-11	P07306	ASGR1
1	ASH1L	0	-1.54	0	0	Q9NR48	ASH1L
1	ASH1L-AS1	2.1719E-29	-2.76	1.2635E-24	1.9769E-28		
1	ASH2L	0	-1.60	0	0	Q9UBL3	ASH2L
1	ASIC3	3.8896E-08	2.21	0.00226276	2.0126E-07	Q9UHC3	ASIC3
1	ASIC5	2.8333E-06	-9.66	0.16482658	1.3239E-05	Q9NY37	ASIC5
1	ASNS	0	-1.74	0	0	P08243	ASNS
1	ASNSD1	2.9788E-65	2.51	1.7329E-60	3.9536E-64	Q9NWL6	ASNSD1
1	ASNSP1	5.837E-14	-92.72	3.3956E-09	3.8153E-13		

1	ASPG	0.00140517	-26.98	1	0.00539816	Q86U10	ASPG
1	ASPHD1	3.4832E-63	11.37	2.0263E-58	4.5484E-62	Q5U4P2	ASPHD1
1	ASPHD2	1.9646E-08	1.64	0.00114289	1.0334E-07	Q6ICH7	ASPHD2
1	ASPM	0	-2.09	0	0	Q8IZT6	ASPM
1	ASPSCR1	1.9459E-24	1.56	1.132E-19	1.6163E-23	Q9BZE9	ASPSCR1
1	ASRGL1	0	-5.17	0	0	Q7L266	ASRGL1
1	ASS1	4.549E-156	-8.92	2.646E-151	9.989E-155	P00966	ASS1
1	ASS1P12	1.0478E-12	4.69	6.0955E-08	6.5663E-12		
1	ASXL2	5.8587E-61	2.31	3.4083E-56	7.5171E-60	Q76L83	ASXL2
1	ATAD2	0	-1.54	0	0	Q6PL18	ATAD2
1	ATAD3A	1.2547E-78	1.91	7.2991E-74	1.8376E-77	Q9NVI7	ATAD3A
1	ATAD3B	6.853E-100	2.23	3.9864E-95	1.1773E-98	Q5T9A4	ATAD3B
1	ATAD3C	1.2736E-41	-6.60	7.4093E-37	1.3625E-40	Q5T2N8	ATAD3C
1	ATAT1	4.26E-193	-8.76	2.478E-188	1.05E-191	Q5SQI0	ATAT1
1	ATE1-AS1	0.00256046	-1.56	1	0.00958386		
1	ATF3	1.4829E-39	-4.57	8.6266E-35	1.5424E-38	P18847	ATF3
1	ATF4	9.7237E-15	1.55	5.6567E-10	6.5116E-14	P18848	ATF4
1	ATF4P2	0.01262781	-2.99	1	0.04376588		
1	ATF5	3.733E-108	2.49	2.172E-103	6.749E-107	Q9Y2D1	ATF5
1	ATF6B	0	-1.84	0	0	Q99941	ATF6B
1	ATF7IP	1.2545E-84	2.05	7.2976E-80	1.9357E-83	Q6VMQ6	ATF7IP
1	ATF7IP2	7.041E-132	45.65	4.096E-127	1.427E-130	Q5U623	ATF7IP2
1	ATG14	6.9501E-37	1.67	4.0431E-32	7.0048E-36	Q6ZNE5	ATG14
1	ATG16L2	2.7324E-11	-1.71	1.5895E-06	1.6263E-10	Q8NAA4	ATG16L2
1	ATG2B	1.3454E-60	1.94	7.827E-56	1.7217E-59	Q96BY7	ATG2B
1	ATG7	1.1898E-20	1.51	6.9217E-16	9.1799E-20	O95352	ATG7
1	ATG9B	0.0059917	-2.44	1	0.0215961	Q674R7	ATG9B
1	ATIC	2.935E-33	1.62	1.7074E-28	2.8296E-32	P31939	ATIC
1	ATL1	5.9874E-07	-1.51	0.03483088	2.9101E-06	Q8WXF7	ATL1
1	ATL2	0	-1.63	0	0	Q8NHH9	ATL2
1	ATOH7	6.638E-06	-4.36	0.38615647	3.0339E-05	Q8N100	ATOH7
1	ATOH8	0.00263868	-4.80	1	0.00986522	Q96SQ7	ATOH8
1	ATOX1	2.1672E-38	1.74	1.2608E-33	2.2255E-37	O00244	ATOX1
1	ATP10D	0	-2.27	0	0	Q9P241	ATP10D
1	ATP11B	0	-2.25	0	0	Q9Y2G3	ATP11B
1	ATP11C	5.883E-75	5.59	3.4224E-70	8.3697E-74	Q8NB49	ATP11C
1	ATP13A2	5.6983E-33	1.66	3.3149E-28	5.4801E-32	Q9NQ11	ATP13A2
1	ATP13A3	0	-1.92	0	0	Q9H7F0	ATP13A3
1	ATP13A4	5.3775E-11	-6.59	3.1283E-06	3.157E-10	Q4VNC1	ATP13A4
1	ATP1A3	1.5294E-22	-17.68	8.8974E-18	1.226E-21	P13637	ATP1A3
1	ATP1B1	0	-2.15	0	0	P05026	ATP1B1
1	ATP1B3	0	-2.84	0	0	P54709	ATP1B3
1	ATP1B3-AS1	5.4181E-06	-5.08	0.31519433	2.4877E-05		
1	ATP2A1-AS1	5.6739E-47	4.38	3.3007E-42	6.4492E-46		
1	ATP2A2	0	-2.21	0	0	P16615	ATP2A2
1	ATP2A3	0.00012508	-5.34	1	0.00052404	Q93084	ATP2A3
1	ATP2B1	0	-1.52	0	0	P20020	ATP2B1

1	ATP2B1-AS1	0.01424553	-2.14	1	0.04913841		
1	ATP2B4	0	-12.53	0	0	P23634	ATP2B4
1	ATP2C1	0	-2.05	0	0	P98194	ATP2C1
1	ATP2C2	1.5665E-42	-4.00	9.1127E-38	1.696E-41	O75185	ATP2C2
1	ATP4A	0.00628958	19.62	1	0.02261092	P20648	ATP4A
1	ATP5A1P1	7.97E-06	40.42	0.46364824	3.6234E-05		
1	ATP5A1P10	2.0573E-08	61.34	0.00119681	1.0809E-07		
1	ATP5A1P7	0.0035383	23.76	1	0.01307236		
1	ATP5A1P8	9.0224E-20	150.52	5.2487E-15	6.8548E-19		
1	ATP5E	1.5308E-27	2.10	8.9054E-23	1.3493E-26	P56381	ATP5E
1	ATP5J2	0	-1.57	0	0	P56134	ATP5J2
1	ATP6AP2	0	-1.80	0	0	A0A1C7CYW4	ATP6AP2
1	ATP6V0D2	2.4877E-05	-14.32	1	0.00010945	Q8N8Y2	ATP6V0D2
1	ATP6V0E1	4.4212E-26	1.61	2.572E-21	3.7818E-25	O15342	ATP6V0E1
1	ATP6V0E2	0	42.73	0	0	Q8NHE4	ATP6V0E2
1	ATP6V0E2-AS	3.642E-188	81.02	2.119E-183	8.792E-187		
1	ATP6V1B1	1.0601E-31	-3.16	6.1668E-27	9.9915E-31	P15313	ATP6V1B1
1	ATP6V1B1-AS	7.6533E-29	4.08	4.4522E-24	6.9059E-28		
1	ATP6V1C2	6.3284E-12	-1.82	3.6815E-07	3.8574E-11	Q8NEY4	ATP6V1C2
1	ATP6V1D	7.6315E-81	2.93	4.4395E-76	1.1401E-79	Q9Y5K8	ATP6V1D
1	ATP6V1G2	2.408E-06	2.08	0.14008288	1.1301E-05	O95670	ATP6V1G2
1	ATP6V1G2-DD	1.6828E-41	3.07	9.7895E-37	1.7956E-40	F2Z307	ATP6V1G2-DD
1	ATP7B	6.757E-205	6.97	3.931E-200	1.726E-203	P35670	ATP7B
1	ATP8A1	8.4787E-13	2.96	4.9324E-08	5.3306E-12	Q9Y2Q0	ATP8A1
1	ATP8A2	4.842E-05	6.97	1	0.0002089	Q9NTI2	ATP8A2
1	ATP8B1	3.8137E-93	5.31	2.2186E-88	6.2602E-92	O43520	ATP8B1
1	ATP8B2	1.801E-123	16.95	1.048E-118	3.545E-122	P98198	ATP8B2
1	ATP8B3	2.7533E-13	1.53	1.6017E-08	1.758E-12	O60423	ATP8B3
1	ATP8B5P	8.257E-05	2.49	1	0.00035054		
1	ATP9A	8.172E-115	2.27	4.754E-110	1.537E-113	O75110	ATP9A
1	ATR	0	-1.77	0	0	Q13535	ATR
1	ATRAID	0	-1.59	0	0	Q6UW56	ATRAID
1	ATRX	3.0265E-13	-1.51	1.7606E-08	1.9297E-12	P46100	ATRX
1	ATXN3	1.7998E-28	1.68	1.047E-23	1.6131E-27	P54252	ATXN3
1	ATXN7L1	1.975E-10	-1.56	1.149E-05	1.1342E-09	Q9ULK2	ATXN7L1
1	AUH	0	-1.65	0	0	Q13825	AUH
1	AURKAIP1	8.2749E-61	2.39	4.8139E-56	1.0601E-59	Q9NWT8	AURKAIP1
1	AVEN	1.7176E-35	2.05	9.9919E-31	1.7048E-34	Q9NQS1	AVEN
1	AVIL	2.1399E-15	2.11	1.2449E-10	1.4647E-14	O75366	AVIL
1	AVPI1	2.4059E-12	-1.57	1.3996E-07	1.4903E-11	Q5T686	AVPI1
1	AXIN2	3.6802E-73	26.89	2.1409E-68	5.1626E-72	Q9Y2T1	AXIN2
1	AZIN1-AS1	6.585E-113	-11.36	3.83E-108	1.227E-111		
1	AZIN2	4.4191E-24	2.31	2.5707E-19	3.6397E-23	Q96A70	AZIN2
1	AZU1	0.00010842	-5.32	1	0.00045675	P20160	AZU1
1	B3GALNT2	0	-2.32	0	0	Q8NCR0	B3GALNT2
1	B3GALT4	1.999E-198	-11.56	1.163E-193	5.014E-197	O96024	B3GALT4
1	B3GALT5	5.7358E-12	-5.04	3.3367E-07	3.5009E-11	Q9Y2C3	B3GALT5

1	B3GALT6	3.9292E-26	1.72	2.2857E-21	3.3644E-25	Q96L58	B3GALT6
1	B3GNT3	2.4182E-87	-8.67	1.4068E-82	3.8011E-86	Q9Y2A9	B3GNT3
1	B3GNT5	0	-1.78	0	0	Q9BYG0	B3GNT5
1	B3GNT7	4.1143E-81	-20.95	2.3935E-76	6.1592E-80	Q8NFL0	B3GNT7
1	B3GNT8	1.2336E-10	-4.00	7.1763E-06	7.1506E-10	Q7Z7M8	B3GNT8
1	B3GNT9	8.8669E-30	-2.15	5.1583E-25	8.1309E-29	Q6UX72	B3GNT9
1	B3GNTL1	1.8822E-29	2.14	1.095E-24	1.7152E-28	Q67FW5	B3GNTL1
1	B4GALNT1	0	-3.95	0	0	Q00973	B4GALNT1
1	B4GALNT4	1.549E-280	61.94	9.009E-276	4.321E-279	Q76KP1	B4GALNT4
1	B4GALT1	0	-1.65	0	0	P15291	B4GALT1
1	B4GALT3	0	-2.06	0	0	O60512	B4GALT3
1	B4GALT6	8.6587E-35	2.43	5.0371E-30	8.5273E-34	Q9UBX8	B4GALT6
1	B4GALT7	2.2581E-80	2.04	1.3136E-75	3.3648E-79	Q9UBV7	B4GALT7
1	B4GAT1	3.0301E-56	1.92	1.7627E-51	3.7553E-55	O43505	B4GAT1
1	BAAT	0.01420976	2.18	1	0.04902665	Q14032	BAAT
1	BABAM1	2.7037E-65	1.69	1.5729E-60	3.5902E-64	Q9NWV8	BABAM1
1	BACE1	0	-1.78	0	0	P56817	BACE1
1	BACE2	1.2702E-21	-1.80	7.3892E-17	9.9922E-21	Q9Y5Z0	BACE2
1	BACH1	3.6049E-89	2.44	2.0971E-84	5.7487E-88	O14867	BACH1
1	BAG1	6.303E-133	2.77	3.667E-128	1.284E-131	Q99933	BAG1
1	BAG3	0	-2.90	0	0	O95817	BAG3
1	BAG5	8.1565E-96	2.14	4.745E-91	1.3627E-94	Q9UL15	BAG5
1	BAGE2	9.947E-127	-12.52	5.787E-122	1.982E-125	Q86Y30	BAGE2
1	BAHCC1	2.16E-214	18.78	1.257E-209	5.636E-213	Q9P281	BAHCC1
1	BAIAP2	0	-1.85	0	0	Q9UQB8	BAIAP2
1	BAIAP2L1	0	-2.65	0	0	Q9UHR4	BAIAP2L1
1	BAIAP2L2	8.9545E-14	-2.64	5.2092E-09	5.8184E-13	Q6UXY1	BAIAP2L2
1	BAIAP3	4.0748E-24	-2.99	2.3705E-19	3.3586E-23	O94812	BAIAP3
1	BAMBI	1.3157E-43	18.75	7.6538E-39	1.4463E-42	Q13145	BAMBI
1	BANK1	2.0054E-50	-5.36	1.1666E-45	2.3558E-49	Q8NDB2	BANK1
1	BARX1	0.00010517	-5.53	1	0.00044329	Q9HBU1	BARX1
1	BARX2	1.2928E-67	-6.81	7.5207E-63	1.7429E-66	Q9UMQ3	BARX2
1	BASP1	3.74E-107	8.64	2.176E-102	6.72E-106	P80723	BASP1
1	BATF	0.00031338	-4.04	1	0.00127212	Q16520	BATF
1	BATF2	3.1539E-08	-5.47	0.00183475	1.6408E-07	Q8N1L9	BATF2
1	BAX	1.4474E-46	1.73	8.42E-42	1.6369E-45	Q07812	BAX
1	BAZ2A	0	-2.24	0	0	Q9UIF9	BAZ2A
1	BAZ2B	0	-2.41	0	0	Q9UIF8	BAZ2B
1	BBOF1	1.0166E-60	4.59	5.9141E-56	1.3012E-59	Q8ND07	BBOF1
1	BBOX1	3.3012E-06	-4.90	0.19204233	1.5345E-05	O75936	BBOX1
1	BBOX1-AS1	4.6702E-12	-1.69	2.7168E-07	2.8598E-11		
1	BBS1	0	-3.11	0	0	Q8NFJ9	BBS1
1	BBS5	8.3378E-12	1.75	4.8504E-07	5.0615E-11	Q8N3I7	BBS5
1	BCAM	0	-10.62	0	0	P50895	BCAM
1	BCAN	4.822E-103	2.13	2.8053E-98	8.473E-102	Q96GW7	BCAN
1	BCAP29	0	-1.62	0	0	Q9UHQ4	BCAP29
1	BCAR3	1.3771E-52	1.69	8.0113E-48	1.6515E-51	O75815	BCAR3

1	BCAS1	2.6447E-06	-6.00	0.15385155	1.2378E-05	O75363	BCAS1
1	BCAS2	4.5112E-71	2.42	2.6243E-66	6.2321E-70	O75934	BCAS2
1	BCAS4	2.1935E-71	2.34	1.276E-66	3.0382E-70	Q8TDM0	BCAS4
1	BCAT1	1.238E-113	4.17	7.203E-109	2.313E-112	P54687	BCAT1
1	BCKDHA	0	-2.55	0	0	P12694	BCKDHA
1	BCKDHB	0	-1.67	0	0	P21953	BCKDHB
1	BCKDK	3.8418E-48	1.83	2.2349E-43	4.4256E-47	O14874	BCKDK
1	BCL10	2.124E-117	2.37	1.235E-112	4.053E-116	O95999	BCL10
1	BCL11B	2.0369E-07	2.28	0.01184932	1.0145E-06	Q9C0K0	BCL11B
1	BCL2	2.644E-176	5.12	1.538E-171	6.172E-175	P10415	BCL2
1	BCL2A1	2.034E-35	17.19	1.1832E-30	2.0164E-34	Q16548	BCL2A1
1	BCL2L1	7.444E-129	1.82	4.331E-124	1.496E-127	Q07817	BCL2L1
1	BCL2L10	0.00077249	-5.54	1	0.00303928	Q9HD36	BCL2L10
1	BCL2L14	7.9856E-11	-2.22	4.6455E-06	4.659E-10	Q9BZR8	BCL2L14
1	BCL2L15	0.00013082	2.81	1	0.00054714	Q5TBC7	BCL2L15
1	BCL2L2-PABPI	4.2959E-17	2.03	2.4991E-12	3.0845E-16		
1	BCL3	0	-4.56	0	0	P20749	BCL3
1	BCL6	0	-2.11	0	0	P41182	BCL6
1	BCL9	0	-1.50	0	0	O00512	BCL9
1	BCL9L	0	-2.12	0	0	Q86UU0	BCL9L
1	BCLAF1	2.9904E-30	1.51	1.7397E-25	2.7596E-29	Q9NYF8	BCLAF1
1	BCR	0	-3.23	0	0	P11274	BCR
1	BCRP2	7.8027E-12	-2.75	4.5391E-07	4.7411E-11		
1	BCRP3_2	2.8519E-09	-2.48	0.00016591	1.558E-08		
1	BCRP9	0.00229352	-4.32	1	0.00862073		
1	BCS1L	6.6777E-91	1.81	3.8847E-86	1.0788E-89	Q9Y276	BCS1L
1	BDKRB1	1.3053E-78	-10.73	7.5934E-74	1.9108E-77	P46663	BDKRB1
1	BDKRB2	3.2602E-58	-6.88	1.8966E-53	4.1016E-57	P30411	BDKRB2
1	BDNF	5.03E-156	12.09	2.926E-151	1.104E-154	P23560	BDNF
1	BDNF-AS	4.2378E-21	-3.00	2.4653E-16	3.2993E-20		
1	BDP1	5.3979E-13	1.50	3.1402E-08	3.417E-12	A6H8Y1	BDP1
1	BEAN1	0.00024712	-2.71	1	0.00101205	Q3B7T3	BEAN1
1	BEGAIN	2.6841E-05	-1.66	1	0.00011787	Q9BUH8	BEGAIN
1	BEND3	3.3649E-16	1.62	1.9575E-11	2.3656E-15	Q5T5X7	BEND3
1	BEND6	1.8838E-38	-4.39	1.0959E-33	1.9358E-37	Q5SZJ8	BEND6
1	BEND7	3.749E-217	102.47	2.181E-212	9.859E-216	Q8N7W2	BEND7
1	BEST1	2.4554E-10	-4.27	1.4284E-05	1.4041E-09	O76090	BEST1
1	BEST4	5.2128E-26	-8.10	3.0325E-21	4.4504E-25	Q8NFU0	BEST4
1	BET1L	4.09E-43	3.05	2.3793E-38	4.4589E-42	Q9NYM9	BET1L
1	BEX2	1.8784E-20	-9.12	1.0927E-15	1.4435E-19	Q9BXY8	BEX2
1	BEX3	2.0476E-93	2.22	1.1912E-88	3.3697E-92	Q00994	BEX3
1	BFSP1	6.201E-101	-3.44	3.6072E-96	1.072E-99	Q12934	BFSP1
1	BGLAP	0.00023833	-4.49	1	0.00097775	P02818	BGLAP
1	BHLHA15	0.00022296	-4.84	1	0.00091716	Q7RTS1	BHLHA15
1	BHLHE41	1.257E-189	-5.60	7.313E-185	3.053E-188	Q9C0J9	BHLHE41
1	BHMT	3.8306E-15	-36.19	2.2284E-10	2.5996E-14	Q93088	BHMT
1	BICC1	0	79.20	0	0	Q9H694	BICC1

1	BICD1	3.493E-111	2.26	2.032E-106	6.448E-110	Q96G01	BICD1
1	BICD2	0	-1.59	0	0	Q8TD16	BICD2
1	BICDL1	5.437E-138	-5.76	3.163E-133	1.13E-136	Q6ZP65	BICDL1
1	BICDL2	2.117E-177	-19.36	1.232E-172	4.957E-176	A1A5D9	BICDL2
1	BIK	5.4156E-22	-4.18	3.1505E-17	4.2881E-21	Q13323	BIK
1	BIN1	1.3742E-94	2.16	7.9943E-90	2.2789E-93	O00499	BIN1
1	BIRC3	3.269E-18	-2.19	1.9017E-13	2.4045E-17	Q13489	BIRC3
1	BIRC7	4.3938E-19	-8.54	2.5561E-14	3.2897E-18	Q96CA5	BIRC7
1	BISPR	3.7657E-87	25.53	2.1906E-82	5.9143E-86		
1	BLCAP	0	-2.05	0	0	P62952	BLCAP
1	BLK	6.087E-78	-27.14	3.5411E-73	8.8615E-77	P51451	BLK
1	BLNK	2.2736E-10	-8.28	1.3226E-05	1.3019E-09	Q8WV28	BLNK
1	BLOC1S2	9.086E-32	1.65	5.2857E-27	8.5724E-31	Q6QNY1	BLOC1S2
1	BLOC1S5	5.4742E-21	1.72	3.1846E-16	4.2523E-20	Q8TDH9	BLOC1S5
1	BLVRB	0	-2.14	0	0	P30043	BLVRB
1	BLZF1	0	-1.70	0	0	Q9H2G9	BLZF1
1	BMP1	0	-3.88	0	0	P13497	BMP1
1	BMP2	1.379E-199	-17.62	8.023E-195	3.47E-198	P12643	BMP2
1	BMP4	4.5753E-09	-1.57	0.00026616	2.4755E-08	P12644	BMP4
1	BMP6	2.2904E-97	6.92	1.3324E-92	3.8688E-96	P22004	BMP6
1	BMP8A	1.8695E-08	-5.82	0.00108754	9.8447E-08	Q7Z5Y6	BMP8A
1	BMP8B	0.00022228	-2.02	1	0.00091441	P34820	BMP8B
1	BMPER	6.709E-103	9.57	3.9027E-98	1.177E-101	Q8N8U9	BMPER
1	BMS1	2.9627E-77	2.02	1.7235E-72	4.2917E-76	Q14692	BMS1
1	BMS1P10	3.608E-118	7.62	2.099E-113	6.913E-117		
1	BMS1P11	3.7647E-24	616.34	2.1901E-19	3.1052E-23		
1	BMS1P12	3.5905E-05	8.33	1	0.00015642		
1	BMS1P14	1.0826E-06	8.67	0.06298163	5.1879E-06		
1	BMS1P17	4.2178E-06	-11.95	0.24536704	1.9466E-05		
1	BMS1P2	5.8747E-06	2.27	0.3417561	2.6927E-05		
1	BMS1P4_1	2.2083E-05	1.51	1	9.7506E-05		
1	BMS1P7	1.622E-05	-7.10	0.94356166	7.2243E-05		
1	BMS1P8	0.00112127	3.44	1	0.00434917		
1	BMS1P9	0.00312064	8.56	1	0.01159036		
1	BMX	8.6173E-06	-14.31	0.50130215	3.9106E-05	P51813	BMX
1	BNC1	1.5657E-66	9.40	9.1082E-62	2.0958E-65	Q01954	BNC1
1	BNC2	5.4192E-77	41.66	3.1526E-72	7.8364E-76	Q6ZN30	BNC2
1	BNIP1	6.0812E-20	1.62	3.5377E-15	4.6329E-19	Q12981	BNIP1
1	BNIP3	2.393E-133	14.82	1.392E-128	4.879E-132	Q12983	BNIP3
1	BNIP3P30	5.4789E-09	31.50	0.00031873	2.9537E-08		
1	BNIP3P4	6.0354E-11	116.57	3.511E-06	3.5351E-10		
1	BNIPL	8.9664E-06	-2.34	0.52161361	4.0637E-05	Q7Z465	BNIPL
1	BOD1	3.2236E-96	2.06	1.8753E-91	5.4013E-95	Q96IK1	BOD1
1	BOK	1.9795E-82	2.03	1.1516E-77	2.9965E-81	Q9UMX3	BOK
1	BOLA1	2.9396E-38	-2.77	1.7101E-33	3.0133E-37	Q9Y3E2	BOLA1
1	BOLA2B	3.8225E-10	1.55	2.2237E-05	2.168E-09		
1	BOLA3	6.0239E-13	1.58	3.5043E-08	3.807E-12	Q53S33	BOLA3

1	BOLL	3.1635E-05	-7.02	1	0.00013831	Q8N9W6	BOLL
1	BORCS7	2.8407E-08	1.56	0.00165258	1.4811E-07	Q96B45	BORCS7
1	BORCS8-MEF2	1.8776E-36	2.05	1.0923E-31	1.8858E-35	H3BNR1	BORCS8-MEF2
1	bP-21201H5.1	0.00437454	1.77	1	0.0160184		
1	bP-21264C1.1	1.1516E-37	-4.45	6.6995E-33	1.1708E-36		
1	bP-21264C1.2	0	-3.24	0	0		
1	bP-2189O9.2	2.5466E-07	-1.76	0.01481478	1.2622E-06		
1	BPHL	0	-1.57	0	0	Q86WA6	BPHL
1	BPNT1	0	-1.60	0	0	O95861	BPNT1
1	BRCA2	6.5068E-10	-1.56	3.7853E-05	3.6541E-09	P51587	BRCA2
1	BRCC3	9.9546E-47	1.67	5.791E-42	1.1284E-45	P46736	BRCC3
1	BRD9	0	-2.13	0	0	Q9H8M2	BRD9
1	BRF1	1.6297E-83	2.60	9.4807E-79	2.4851E-82	Q92994	BRF1
1	BRI3	0	-1.71	0	0	O95415	BRI3
1	BRINP3	9.5043E-42	-11.09	5.529E-37	1.0186E-40	Q76B58	BRINP3
1	BRIP1	2.0963E-14	1.52	1.2195E-09	1.3893E-13	Q9BX63	BRIP1
1	BRIX1	0	-1.54	0	0	Q8TDN6	BRIX1
1	BRMS1L	9.6799E-26	1.66	5.6312E-21	8.2303E-25	Q5PSV4	BRMS1L
1	BRPF1	4.1242E-34	1.67	2.3992E-29	4.031E-33	P55201	BRPF1
1	BRSK1	1.411E-57	7.28	8.2083E-53	1.7649E-56	Q8TDC3	BRSK1
1	BRSK2	2.138E-188	45.78	1.244E-183	5.17E-187	Q8IWQ3	BRSK2
1	BSCL2	2.946E-182	-9.59	1.714E-177	6.987E-181	Q96G97	BSCL2
1	BSN	2.0178E-07	1.97	0.01173823	1.0051E-06	Q9UPA5	BSN
1	BSN-AS2	0.00266728	2.40	1	0.00996699		
1	BSPRY	8.0981E-28	-41.58	4.711E-23	7.1748E-27	Q5W0U4	BSPRY
1	BST1	2.1468E-08	-4.15	0.00124886	1.1265E-07	Q10588	BST1
1	BST2	4.111E-142	59.18	2.392E-137	8.678E-141	Q10589	BST2
1	BTBD10	2.1819E-28	1.54	1.2693E-23	1.9528E-27	Q9BSF8	BTBD10
1	BTBD16	2.8916E-63	-7.35	1.6821E-58	3.7784E-62	Q32M84	BTBD16
1	BTBD19	3.2889E-10	1.97	1.9133E-05	1.8695E-09		
1	BTBD2	1.199E-14	-2.34	6.9753E-10	8.0056E-14	Q9BX70	BTBD2
1	BTBD3	5.1781E-45	1.91	3.0123E-40	5.7685E-44	Q9Y2F9	BTBD3
1	BTBD6	6.2537E-97	2.51	3.638E-92	1.053E-95	Q96KE9	BTBD6
1	BTBD7P1	3.1892E-07	-7.00	0.0185526	1.5715E-06		
1	BTC	6.756E-85	-5.41	3.9302E-80	1.0442E-83	P35070	BTC
1	BTD	1.2825E-31	1.71	7.4606E-27	1.2072E-30	P43251	BTD
1	BTF3	6.5697E-54	2.07	3.8219E-49	7.9972E-53	P20290	BTF3
1	BTF3L4	4.6616E-21	1.55	2.7118E-16	3.6264E-20	Q96K17	BTF3L4
1	BTG2	4.778E-185	-8.71	2.78E-180	1.142E-183	P78543	BTG2
1	BTN3A1	1.9135E-83	-4.50	1.1131E-78	2.9155E-82	O00481	BTN3A1
1	BTN3A2	2.5607E-64	-4.84	1.4897E-59	3.3757E-63	P78410	BTN3A2
1	BTN3A3	1.543E-173	-4.97	8.974E-169	3.568E-172	O00478	BTN3A3
1	BTNL8	0.00285033	-21.55	1	0.01062578	Q6UX41	BTNL8
1	BTNL9	2.6653E-19	45.92	1.5505E-14	2.0032E-18	Q6UXG8	BTNL9
1	BTRC	2.294E-109	1.91	1.334E-104	4.184E-108	Q9Y297	BTRC
1	BVES	6.4475E-54	-10.23	3.7508E-49	7.8501E-53	Q8NE79	BVES
1	BX322557.10	5.0498E-78	5.81	2.9377E-73	7.3589E-77		

1	BX842568.2	6.354E-08	-10.82	0.0036964	3.2484E-07		
1	BZW1	1.6894E-92	2.91	9.8281E-88	2.7615E-91	Q7L1Q6	BZW1
1	C10orf10	4.862E-186	-7.67	2.828E-181	1.167E-184		
1	C10orf105	0.005325	-9.50	1	0.01931036	Q8TEF2	C10orf105
1	C10orf11	4.6381E-08	-3.01	0.00269818	2.3905E-07		
1	C10orf111	0.01318482	2.14	1	0.04560943	Q8N326	C10orf111
1	C10orf25	7.3661E-13	2.69	4.2852E-08	4.6416E-12	Q5T742	C10orf25
1	C10orf35	1.9719E-25	1.87	1.1471E-20	1.6686E-24	Q96D05	C10orf35
1	C10orf55	1.7941E-28	-3.11	1.0437E-23	1.6082E-27		
1	C10orf67	1.4358E-05	-6.55	0.83526622	6.4202E-05	Q8IYJ2	C10orf67
1	C10orf91	4.5787E-12	-5.03	2.6636E-07	2.8053E-11		
1	C10orf95	0.00012076	-1.73	1	0.00050674	Q9H7T3	C10orf95
1	C11orf16	4.4544E-16	4.60	2.5913E-11	3.116E-15		
1	C11orf42	2.7426E-09	2.22	0.00015955	1.4997E-08		
1	C11orf45	1.2999E-38	-2.29	7.5619E-34	1.3379E-37	Q8TAV5	C11orf45
1	C11orf52	6.9229E-33	-7.72	4.0273E-28	6.6501E-32	Q96A22	C11orf52
1	C11orf57	5.1408E-40	1.64	2.9906E-35	5.3816E-39	Q6ZUT1	C11orf57
1	C11orf58	1.1274E-56	2.75	6.5584E-52	1.4008E-55		
1	C11orf63	1.0752E-78	48.83	6.2549E-74	1.5759E-77	Q6NUN7	C11orf63
1	C11orf65	1.0003E-07	-2.46	0.00581888	5.0621E-07	Q8NCR3	C11orf65
1	C11orf68	0	-2.18	0	0	Q9H3H3	C11orf68
1	C11orf71	6.1135E-38	-2.55	3.5565E-33	6.2416E-37		
1	C11orf72	2.6572E-05	-4.78	1	0.00011673		
1	C11orf80	0	-1.81	0	0		
1	C11orf84	0	-1.79	0	0		
1	C11orf94	3.3099E-06	18.46	0.19255277	1.5382E-05	C9JXX5	C11orf94
1	C12orf10	1.6853E-22	1.52	9.804E-18	1.3484E-21	Q9HB07	C12orf10
1	C12orf29	8.8652E-15	1.55	5.1572E-10	5.9436E-14	Q8N999	C12orf29
1	C12orf4	1.7369E-80	2.35	1.0104E-75	2.5908E-79	Q9NQ89	C12orf4
1	C12orf42	9.8263E-35	-10.21	5.7163E-30	9.6723E-34		
1	C12orf43	1.6418E-23	1.54	9.5509E-19	1.3388E-22	Q96C57	C12orf43
1	C12orf49	6.1234E-47	1.59	3.5622E-42	6.9588E-46	Q9H741	C12orf49
1	C12orf54	1.882E-06	-15.54	0.10948587	8.8977E-06		
1	C12orf56	7.8275E-33	-10.06	4.5536E-28	7.5117E-32		
1	C12orf66	3.468E-80	-2.43	2.0175E-75	5.1597E-79	Q96MD2	C12orf66
1	C12orf75	1.1162E-96	7.60	6.4932E-92	1.875E-95		
1	C14orf1	7.6452E-37	1.65	4.4475E-32	7.7026E-36	Q9UKR5	C14orf1
1	C14orf105	3.9648E-11	10.13	2.3065E-06	2.339E-10	Q9NVL8	C14orf105
1	C14orf119	7.8199E-27	1.72	4.5492E-22	6.7756E-26	Q9NWQ9	C14orf119
1	C14orf132	2.8743E-17	117.25	1.6721E-12	2.0717E-16	Q9NPU4	C14orf132
1	C14orf159	0	-1.66	0	0	Q7Z3D6	C14orf159
1	C14orf166	8.2949E-67	2.23	4.8255E-62	1.1124E-65	Q9Y224	C14orf166
1	C14orf178	6.8072E-05	2.67	1	0.00029073		
1	C14orf2	5.7183E-39	2.19	3.3266E-34	5.9149E-38		
1	C14orf39	0.01114735	1.86	1	0.03890139		
1	C14orf79	1.3635E-81	2.52	7.9323E-77	2.0502E-80		
1	C14orf80	2.7714E-83	3.30	1.6122E-78	4.2193E-82		

1	C14orf93	0	-1.64	0	0	Q9H972	C14orf93
1	C15orf56	0.00069967	-2.92	1	0.00276006		
1	C15orf65	0.01339609	1.62	1	0.04630996		
1	C16orf45	3.169E-177	14.32	1.844E-172	7.416E-176	Q96MC5	C16orf45
1	C16orf52	1.5646E-51	2.56	9.1016E-47	1.856E-50		
1	C16orf54	3.0537E-05	-6.87	1	0.00013363	Q6UWD8	C16orf54
1	C16orf59	1.167E-24	1.65	6.7886E-20	9.7314E-24	Q7L2K0	C16orf59
1	C16orf62	1.2043E-79	5.06	7.0057E-75	1.7808E-78	Q7Z3J2	C16orf62
1	C16orf71	0.00016437	1.97	1	0.00068279		
1	C16orf74	0	-2.67	0	0		
1	C16orf86	1.5019E-05	2.36	0.87371989	6.7054E-05		
1	C16orf87	9.7807E-12	1.55	5.6898E-07	5.9244E-11	Q6PH81	C16orf87
1	C17orf50	3.0904E-09	-27.68	0.00017978	1.6855E-08		
1	C17orf51	3.9465E-15	-35.00	2.2959E-10	2.6774E-14		
1	C17orf58	7.0705E-19	1.68	4.1132E-14	5.272E-18		
1	C17orf80	1.9432E-50	1.87	1.1304E-45	2.2832E-49	Q9BSJ5	C17orf80
1	C17orf97	5.9843E-12	2.45	3.4813E-07	3.6507E-11		
1	C17orf99	2.3003E-05	-4.65	1	0.00010146	Q6UX52	C17orf99
1	C18orf21	5.8039E-29	1.86	3.3764E-24	5.2493E-28		
1	C18orf32	4.2478E-32	3.23	2.4711E-27	4.0279E-31	Q8TCD1	C18orf32
1	C18orf54	4.167E-36	2.07	2.4241E-31	4.1652E-35		
1	C18orf65	0.00067881	25.96	1	0.00268106		
1	C18orf8	2.0005E-36	1.81	1.1637E-31	2.0082E-35	Q96DM3	C18orf8
1	C19orf12	3.5362E-80	2.35	2.0572E-75	5.2599E-79	Q9NSK7	C19orf12
1	C19orf24	5.9879E-37	1.95	3.4834E-32	6.0423E-36	Q9BVV8	C19orf24
1	C19orf25	4.0584E-42	1.72	2.3609E-37	4.3713E-41	Q9UFG5	C19orf25
1	C19orf44	9.7929E-22	1.62	5.6969E-17	7.7225E-21	Q9H6X5	C19orf44
1	C19orf47	3.1462E-24	1.54	1.8303E-19	2.6009E-23	Q8N9M1	C19orf47
1	C19orf48	3.9904E-68	1.91	2.3214E-63	5.3935E-67		
1	C19orf53	2.6415E-33	1.99	1.5367E-28	2.5492E-32	Q9UNZ5	C19orf53
1	C19orf57	3.4601E-11	2.18	2.0129E-06	2.0483E-10	Q0VDD7	C19orf57
1	C19orf66	2.3571E-70	-2.63	1.3712E-65	3.2417E-69		
1	C19orf70	1.2533E-62	2.33	7.2911E-58	1.6308E-61		
1	C19orf71	0.00074723	-1.84	1	0.00294327		
1	C1GALT1	0	-1.89	0	0	Q9NS00	C1GALT1
1	C1GALT1C1L	1.7598E-19	142.16	1.0238E-14	1.3282E-18	P0DN25	C1GALT1C1L
1	C1orf101	5.3744E-14	-2.97	3.1265E-09	3.5169E-13		
1	C1orf105	0.00067856	-10.05	1	0.00268023	O95561	C1orf105
1	C1orf106	0	-4.21	0	0		
1	C1orf109	2.3492E-57	1.96	1.3666E-52	2.9327E-56	Q9NX04	C1orf109
1	C1orf115	2.2116E-06	-1.58	0.1286552	1.0408E-05	Q9H7X2	C1orf115
1	C1orf116	0	-12.81	0	0		
1	C1orf122	5.2697E-43	2.28	3.0656E-38	5.7397E-42		
1	C1orf123	1.49E-26	1.61	8.6681E-22	1.2851E-25	Q9NWV4	C1orf123
1	C1orf131	0	-1.80	0	0	Q8NDD1	C1orf131
1	C1orf132	2.436E-14	-2.26	1.4171E-09	1.6112E-13		
1	C1orf137	7.8675E-13	-28.56	4.5769E-08	4.9528E-12		

1	C1orf143	0.00037404	13.28	1	0.0015096		
1	C1orf145	2.1162E-05	-3.28	1	9.3584E-05		
1	C1orf147	1.853E-08	-3.31	0.00107796	9.7614E-08		
1	C1orf168	5.9789E-08	11.45	0.00347815	3.062E-07		
1	C1orf186	6.8572E-05	2.78	1	0.00029278	Q6ZWK4	C1orf186
1	C1orf194	5.9822E-06	-2.92	0.34800916	2.7409E-05		
1	C1orf204	0.00196425	-1.70	1	0.00743837		
1	C1orf21	2.7632E-09	-2.08	0.00016075	1.5106E-08	Q9H246	C1orf21
1	C1orf210	1.486E-212	-12.11	8.647E-208	3.864E-211	Q8IVY1	C1orf210
1	C1orf216	1.4458E-45	1.71	8.4107E-41	1.6202E-44	Q8TAB5	C1orf216
1	C1orf226	8.6947E-81	-6.40	5.058E-76	1.2983E-79		
1	C1orf228	2.0054E-21	-3.82	1.1666E-16	1.5718E-20		
1	C1orf234	1.7346E-07	-3.28	0.01009107	8.673E-07		
1	C1orf27	0	-1.88	0	0		
1	C1orf53	1.0583E-06	-2.02	0.06156317	5.0728E-06		
1	C1orf54	2.1575E-19	-3.55	1.2551E-14	1.6245E-18	Q8WWF1	C1orf54
1	C1orf56	4.4167E-52	-3.19	2.5694E-47	5.2738E-51		
1	C1orf61	0.00195933	-9.45	1	0.00742024	Q13536	C1orf61
1	C1orf74	3.5377E-46	-2.39	2.058E-41	3.9892E-45		
1	C1QB	0.00126424	-10.68	1	0.00488126	P02746	C1QB
1	C1QBP	8.3417E-79	1.92	4.8527E-74	1.2251E-77	Q07021	C1QBP
1	C1QL3	1.1253E-16	-4.77	6.5462E-12	8.0095E-16	Q5VWW1	C1QL3
1	C1QL4	1.1945E-26	4.79	6.949E-22	1.0327E-25	Q86Z23	C1QL4
1	C1QTNF1	0.00448276	4.28	1	0.01639301	Q9BXJ1	C1QTNF1
1	C1QTNF3	3.2321E-06	-2.90	0.18802625	1.504E-05	Q9BXJ4	C1QTNF3
1	C1QTNF3-AM.	2.5465E-22	-4.00	1.4814E-17	2.0282E-21	E9PGA6	C1QTNF3-AM.
1	C1QTNF4	1.6793E-33	-4.33	9.7694E-29	1.6261E-32	Q9BXJ3	C1QTNF4
1	C1QTNF6	0	-9.15	0	0	Q9BXI9	C1QTNF6
1	C1R	2.974E-139	-3.60	1.73E-134	6.214E-138	P00736	C1R
1	C1RL	0	-2.38	0	0	Q9NZP8	C1RL
1	C1S	9.5434E-07	1.64	0.05551749	4.5878E-06	P09871	C1S
1	C2	2.6717E-08	1.81	0.00155424	1.3956E-07	P06681	C2
1	C20orf197	4.154E-129	41.88	2.416E-124	8.352E-128		
1	C20orf203	0.00926422	17.61	1	0.03266085	Q8NBC4	C20orf203
1	C20orf27	1.0109E-42	1.61	5.8809E-38	1.0972E-41	Q9GZN8	C20orf27
1	C20orf96	8.534E-128	3.54	4.965E-123	1.709E-126	Q9NUD7	C20orf96
1	C21orf59	1.2016E-37	1.59	6.9903E-33	1.221E-36	P57076	C21orf59
1	C21orf62-AS1	2.8612E-11	-2.33	1.6645E-06	1.7017E-10		
1	C22orf23	0	-4.57	0	0	Q9BZE7	C22orf23
1	C22orf24	0.00341515	3.91	1	0.01264626	Q9Y442	C22orf24
1	C22orf29	1.4012E-81	1.93	8.1512E-77	2.1063E-80		
1	C2CD2	2.3653E-80	2.24	1.376E-75	3.5236E-79	Q9Y426	C2CD2
1	C2CD2L	1.365E-30	1.70	7.9408E-26	1.2665E-29	O14523	C2CD2L
1	C2CD4D	0.00094086	-5.54	1	0.00367315	B7Z1M9	C2CD4D
1	C2CD5	9.5475E-36	1.63	5.5542E-31	9.5008E-35	Q86YS7	C2CD5
1	C2orf16	2.0788E-17	-2.15	1.2093E-12	1.5038E-16	Q68DN1	C2orf16
1	C2orf27A	3.7224E-32	1.77	2.1654E-27	3.5331E-31		

1	C2orf27AP3	0.00942764	-19.46	1	0.03316063		
1	C2orf27B	0.00291024	3.14	1	0.01083937		
1	C2orf54	2.6743E-68	-95.01	1.5557E-63	3.6188E-67		
1	C2orf68	0	-1.75	0	0		
1	C2orf72	0.00796586	-15.96	1	0.02831345		
1	C2orf73	0.00021319	-3.87	1	0.00087848		
1	C2orf74	5.3837E-95	26.89	3.1319E-90	8.9585E-94	A8MZ97	C2orf74
1	C2orf78	0.00062004	-8.52	1	0.00245859		
1	C2orf82	2.237E-13	1.75	1.3013E-08	1.4338E-12	Q6UX34	C2orf82
1	C3	3.186E-169	-7.06	1.853E-164	7.277E-168	P01024	C3
1	C3orf14	2.122E-213	92.22	1.235E-208	5.524E-212		
1	C3orf18	2.7456E-81	4.93	1.5972E-76	4.1186E-80	Q9UK00	C3orf18
1	C3orf58	0	-3.24	0	0	Q8NDZ4	C3orf58
1	C3orf80	0.00315616	-6.01	1	0.01171856	F5H4A9	C3orf80
1	C3orf84	5.1237E-69	-16.89	2.9807E-64	6.9626E-68		
1	C4A	9.9859E-05	1.96	1	0.00042166	P0C0L4	C4A
1	C4B	1.797E-12	-3.75	1.0454E-07	1.1175E-11	P0C0L5	C4B
1	C4BPAP1	0.00977274	-17.79	1	0.03431223		
1	C4orf22	8.8179E-07	2.36	0.05129746	4.2465E-06		
1	C4orf3	0	-1.56	0	0	Q8WVX3	C4orf3
1	C4orf32	2.0039E-18	-1.76	1.1657E-13	1.4797E-17	Q8N8J7	C4orf32
1	C4orf33	4.7758E-43	-2.40	2.7783E-38	5.2028E-42	Q8N1A6	C4orf33
1	C4orf36	1.6705E-34	-2.40	9.7178E-30	1.641E-33		
1	C4orf45	0.00568981	3.06	1	0.02058324		
1	C4orf47	3.3973E-13	-3.72	1.9764E-08	2.1635E-12	A7E2U8	C4orf47
1	C4orf48	0.00011491	1.54	1	0.00048323	Q5BLP8	C4orf48
1	C4orf51	0.00147457	-3.70	1	0.00564947		
1	C5	8.4712E-79	-3.91	4.928E-74	1.2438E-77	P01031	C5
1	C5AR1	4.8424E-59	-7.73	2.817E-54	6.1333E-58	P21730	C5AR1
1	C5orf22	0	-1.63	0	0	Q49AR2	C5orf22
1	C5orf24	4.9412E-29	1.75	2.8745E-24	4.4725E-28	Q7Z6I8	C5orf24
1	C5orf30	6.7203E-92	2.36	3.9094E-87	1.0936E-90	Q96GV9	C5orf30
1	C5orf34	0	-1.67	0	0		
1	C5orf38	1.7757E-80	149.25	1.033E-75	2.6481E-79	Q86SI9	C5orf38
1	C5orf46	2.581E-186	692.84	1.501E-181	6.199E-185	Q6UWT4	C5orf46
1	C5orf56	1.3796E-52	-8.40	8.0258E-48	1.6541E-51		
1	C5orf58	0.00182553	-6.38	1	0.00693699		
1	C5orf63	6.8274E-69	-4.56	3.9718E-64	9.2691E-68	A6NC05	C5orf63
1	C5orf66	0.00359344	-1.55	1	0.01326678		
1	C6orf1	2.3337E-39	2.39	1.3576E-34	2.4256E-38		
1	C6orf118	0.0001214	3.41	1	0.00050923		
1	C6orf132	0	-4.45	0	0		
1	C6orf141	1.7644E-45	-2.96	1.0264E-40	1.9754E-44		
1	C6orf15	3.5625E-12	-5.27	2.0725E-07	2.1921E-11	Q6UXA7	C6orf15
1	C6orf163	0.00424087	-1.61	1	0.01555243	Q5TEZ5	C6orf163
1	C6orf223	5.617E-255	-24.51	3.268E-250	1.54E-253		
1	C6orf226	2.0503E-06	-1.88	0.11927352	9.6734E-06	Q5I0X4	C6orf226

1	C6orf48	2.508E-108	2.16	1.459E-103	4.543E-107		
1	C6orf62	8.7492E-88	1.89	5.0898E-83	1.3805E-86	Q9GZU0	C6orf62
1	C6orf89	0	-1.56	0	0	Q6UWU4	C6orf89
1	C6orf99	0.00355829	-2.44	1	0.01314204		
1	C7	0.00333921	4.76	1	0.01237056	P10643	C7
1	C7orf31	2.8517E-55	13.83	1.6589E-50	3.5102E-54	Q8N865	C7orf31
1	C7orf55-LUC7	9.2623E-24	1.53	5.3882E-19	7.588E-23		
1	C7orf57	3.3374E-18	-6.17	1.9415E-13	2.4539E-17		
1	C7orf61	7.5262E-06	-2.11	0.4378291	3.4262E-05	Q8IZ16	C7orf61
1	C8orf31	1.6395E-91	-6.70	9.5377E-87	2.6627E-90		
1	C8orf34	0.0029994	-4.18	1	0.01116217		
1	C8orf37	1.6986E-24	-2.08	9.8816E-20	1.4133E-23	Q96NL8	C8orf37
1	C8orf37-AS1	1.0672E-30	-3.34	6.2085E-26	9.9225E-30		
1	C8orf44	0	-3.20	0	0	Q96CB5	C8orf44
1	C8orf46	5.9401E-21	-6.00	3.4556E-16	4.6099E-20		
1	C8orf48	4.4281E-75	119.82	2.576E-70	6.3107E-74	Q96LL4	C8orf48
1	C8orf74	5.8943E-22	-30.69	3.4289E-17	4.6614E-21	Q6P047	C8orf74
1	C8orf88	7.0758E-64	-5.27	4.1163E-59	9.2981E-63	P0DMB2	C8orf88
1	C9	0.00014763	-6.59	1	0.00061511	P02748	C9
1	C9orf116	2.1311E-56	3.29	1.2398E-51	2.6451E-55	Q5BN46	C9orf116
1	C9orf139	3.703E-23	29.98	2.1542E-18	2.999E-22		
1	C9orf142	3.8119E-31	1.80	2.2175E-26	3.5669E-30	Q9BUH6	C9orf142
1	C9orf147	2.3285E-08	-1.54	0.0013546	1.2197E-07		
1	C9orf152	7.0997E-09	7.73	0.00041302	3.8077E-08		
1	C9orf16	0	-1.53	0	0	Q9BUW7	C9orf16
1	C9orf3	0	-2.44	0	0		
1	C9orf40	2.7453E-27	1.69	1.5971E-22	2.4056E-26		
1	C9orf41-AS1	8.6178E-05	-1.72	1	0.0003654		
1	C9orf43	9.7647E-05	-1.70	1	0.00041268	Q8TAL5	C9orf43
1	C9orf47	0.00022565	34.28	1	0.00092772	Q6ZRZ4	C9orf47
1	C9orf72	3.5666E-17	1.61	2.0748E-12	2.566E-16	Q96LT7	C9orf72
1	C9orf84	1.184E-125	-9.03	6.886E-121	2.347E-124		
1	C9orf92	2.851E-61	104.40	1.6585E-56	3.6677E-60		
1	CA1	5.0929E-07	-5.35	0.02962743	2.4851E-06	P00915	CA1
1	CA11	1.9532E-14	-1.69	1.1363E-09	1.2962E-13	O75493	CA11
1	CA12	3.0912E-91	-3.43	1.7983E-86	5.0049E-90	O43570	CA12
1	CA13	7.112E-45	-2.54	4.1373E-40	7.9047E-44	Q8N1Q1	CA13
1	CA14	9.5201E-33	-4.80	5.5382E-28	9.1209E-32	Q9ULX7	CA14
1	CA2	5.211E-179	-5.71	3.031E-174	1.226E-177	P00918	CA2
1	CA3	9.4241E-27	-5.12	5.4824E-22	8.1547E-26	P07451	CA3
1	CA3-AS1	2.776E-29	-7.41	1.6149E-24	2.5217E-28		
1	CA5B	1.2206E-08	-1.61	0.00071006	6.4775E-08	Q9Y2D0	CA5B
1	CA5BP1	2.277E-20	-1.87	1.3246E-15	1.747E-19		
1	CA9	4.035E-101	-14.26	2.3474E-96	6.997E-100	Q16790	CA9
1	CABLES1	3.6188E-11	-1.78	2.1052E-06	2.1403E-10	Q8TDN4	CABLES1
1	CABP1	7.1276E-08	6.88	0.00414641	3.635E-07	Q9NZU7	CABP1
1	CABP4	7.969E-183	-12.92	4.636E-178	1.893E-181	P57796	CABP4

1	CABP7	6.0828E-05	3.11	1	0.00026077	Q86V35	CABP7
1	CABYR	7.058E-145	-4.83	4.106E-140	1.5E-143	O75952	CABYR
1	CACNA1D	2.695E-41	-9.93	1.5678E-36	2.8677E-40	Q01668	CACNA1D
1	CACNA1F	3.233E-07	-20.85	0.01880741	1.5924E-06	O60840	CACNA1F
1	CACNA1I	0.00020323	-6.10	1	0.0008388	Q9P0X4	CACNA1I
1	CACNA2D1	0	-2.90	0	0	P54289	CACNA2D1
1	CACNA2D2	9.4179E-16	-4.42	5.4788E-11	6.5107E-15	Q9NY47	CACNA2D2
1	CACNA2D4	1.568E-222	14.28	9.123E-218	4.166E-221	Q7Z3S7	CACNA2D4
1	CACNB4	2.259E-12	-3.00	1.3142E-07	1.4013E-11	O00305	CACNB4
1	CACNG4	3.8261E-27	-7.80	2.2258E-22	3.3411E-26	Q9UBN1	CACNG4
1	CACNG7	1.086E-229	154.72	6.32E-225	2.909E-228	P62955	CACNG7
1	CACNG8	1.4257E-05	-3.39	0.82940811	6.3761E-05	Q8WXS5	CACNG8
1	CACTIN	2.9385E-50	1.61	1.7094E-45	3.4472E-49	Q8WUQ7	CACTIN
1	CACTIN-AS1	0.00503409	1.53	1	0.01831135		
1	CADM1	8.5672E-17	39.40	4.9839E-12	6.1182E-16	Q9BY67	CADM1
1	CADM2	1.016E-179	-10.75	5.91E-175	2.393E-178	Q8N3J6	CADM2
1	CADM4	3.2544E-52	-3.10	1.8932E-47	3.8899E-51	Q8NFZ8	CADM4
1	CADPS2	1.107E-141	-13.46	6.441E-137	2.334E-140	Q86UW7	CADPS2
1	CAHM	0.00807712	-1.72	1	0.02868261		
1	CALB1	0.00022338	-1.78	1	0.00091875	P05937	CALB1
1	CALCA	0.01193309	-7.74	1	0.04150648	P01258	CALCA
1	CALCB	0.01319674	-6.42	1	0.04564525	P10092	CALCB
1	CALCRL	9.5256E-25	-3.28	5.5414E-20	7.9595E-24	Q16602	CALCRL
1	CALD1	0	-2.45	0	0	Q05682	CALD1
1	CALHM2	2.9653E-37	-2.18	1.725E-32	3.0021E-36	Q9HA72	CALHM2
1	CALHM3	0.00859914	-15.98	1	0.03043577	Q86XJ0	CALHM3
1	CALM1	1.7178E-65	3.21	9.9932E-61	2.2868E-64	P0DP23	CALM1
1	CALML3	0	-3.71	0	0	P27482	CALML3
1	CALML3-AS1	9.082E-146	-7.44	5.284E-141	1.935E-144		
1	CALML5	0.00142033	-3.54	1	0.00545246	Q9NZT1	CALML5
1	CALR4P	4.0484E-08	-8.63	0.0023551	2.0929E-07		
1	CAMK1	5.725E-167	6.74	3.33E-162	1.299E-165	Q14012	CAMK1
1	CAMK1D	4.0949E-16	2.21	2.3821E-11	2.8718E-15	Q8IU85	CAMK1D
1	CAMK2A	8.8434E-14	-5.38	5.1446E-09	5.7488E-13	Q9UQM7	CAMK2A
1	CAMK2D	1.185E-33	1.77	6.8933E-29	1.1502E-32	Q13557	CAMK2D
1	CAMK2N1	3.4195E-10	-1.59	1.9892E-05	1.9426E-09	Q7Z7J9	CAMK2N1
1	CAMK2N2	2.628E-135	24.36	1.529E-130	5.399E-134	Q96S95	CAMK2N2
1	CAMK4	3.3072E-56	-3.75	1.9239E-51	4.097E-55	Q16566	CAMK4
1	CAMKK1	2.6984E-47	-2.33	1.5698E-42	3.0756E-46	Q8N5S9	CAMKK1
1	CAMLG	3.4803E-36	1.72	2.0246E-31	3.4817E-35	P49069	CAMLG
1	CAMSAP1	1.9707E-60	1.75	1.1464E-55	2.5185E-59	Q5T5Y3	CAMSAP1
1	CAMSAP3	2.7826E-80	-2.60	1.6187E-75	4.1442E-79	Q9P1Y5	CAMSAP3
1	CAMTA1	1.0027E-33	1.81	5.8329E-29	9.7492E-33	Q9Y6Y1	CAMTA1
1	CANX	2.7964E-43	1.53	1.6268E-38	3.0596E-42	P27824	CANX
1	CAP2	7.3272E-27	1.59	4.2625E-22	6.3516E-26	P40123	CAP2
1	CAPG	1.82E-99	2.49	1.0589E-94	3.1217E-98	P40121	CAPG
1	CAPN1	0	-3.76	0	0	P07384	CAPN1

1	CAPN11	9.9745E-08	-6.88	0.00580254	5.0483E-07	Q9UMQ6	CAPN11
1	CAPN12	4.3492E-55	-8.80	2.5301E-50	5.3434E-54	Q6ZSI9	CAPN12
1	CAPN13	1.1802E-06	-3.07	0.06865566	5.646E-06	Q6MZZ7	CAPN13
1	CAPN14	0.00229035	-2.00	1	0.00860939	A8MX76	CAPN14
1	CAPN15	2.0736E-27	1.72	1.2063E-22	1.8233E-26	O75808	CAPN15
1	CAPN3	1.3646E-14	-2.89	7.9384E-10	9.0891E-14	P20807	CAPN3
1	CAPN5	3.394E-191	5.70	1.974E-186	8.303E-190	O15484	CAPN5
1	CAPN8	4.5423E-31	-19.53	2.6424E-26	4.2449E-30	A6NHC0	CAPN8
1	CAPN9	0.00295482	23.91	1	0.01099978	O14815	CAPN9
1	CAPNS1	3.3057E-24	1.56	1.9231E-19	2.732E-23	P04632	CAPNS1
1	CAPRIN2	4.5279E-49	1.95	2.634E-44	5.2534E-48	Q6IMN6	CAPRIN2
1	CAPS	1.4135E-10	-1.51	8.2227E-06	8.1712E-10	Q13938	CAPS
1	CAPS2	1.027E-151	-4.99	5.972E-147	2.218E-150	Q9BXY5	CAPS2
1	CARD10	0	-2.97	0	0	Q9BWT7	CARD10
1	CARD11	0	66.86	0	0	Q9BXL7	CARD11
1	CARD16	6.352E-119	-25.77	3.695E-114	1.223E-117	Q5EG05	CARD16
1	CARD17	2.1834E-06	-44.44	0.12701832	1.0283E-05	Q5XLA6	CARD17
1	CARD18	1.8942E-27	-12.44	1.1019E-22	1.6671E-26	P57730	CARD18
1	CARD6	1.1437E-73	-5.14	6.6536E-69	1.6114E-72	Q9BX69	CARD6
1	CARD8	2.3043E-38	1.63	1.3405E-33	2.3646E-37	Q9Y2G2	CARD8
1	CARD8-AS1	3.875E-21	3.04	2.2542E-16	3.0201E-20		
1	CARD9	3.1189E-12	2.57	1.8144E-07	1.9243E-11	Q9H257	CARD9
1	CARF	3.2969E-21	-2.05	1.9179E-16	2.573E-20	Q8N187	CARF
1	CARHSP1	5.5333E-84	1.77	3.2189E-79	8.4865E-83	Q9Y2V2	CARHSP1
1	CARM1	1.4322E-89	1.88	8.3319E-85	2.2896E-88	Q86X55	CARM1
1	CARMIL3	5.5283E-12	8.23	3.216E-07	3.376E-11	Q8ND23	CARMIL3
1	CARNS1	1.3688E-11	-3.97	7.9629E-07	8.2492E-11	A5YM72	CARNS1
1	CARS	1.0451E-20	1.50	6.0795E-16	8.0737E-20	P49589	CARS
1	CASC11	3.1249E-07	-3.09	0.01817852	1.5407E-06		
1	CASC19	0.00879881	-3.86	1	0.03109733		
1	CASC2	2.1566E-09	-1.72	0.00012546	1.1839E-08	Q6XLA1	CASC2
1	CASC20	2.2902E-11	-15.34	1.3323E-06	1.3665E-10		
1	CASC6	1.1701E-21	-181.82	6.8071E-17	9.215E-21		
1	CASC8	1.4211E-10	-1.68	8.2669E-06	8.2127E-10		
1	CASC9	3.343E-159	-7.75	1.945E-154	7.418E-158		
1	CASK	0	-1.96	0	0	O14936	CASK
1	CASKIN1	0.00639801	-2.63	1	0.02296524	Q8WXD9	CASKIN1
1	CASKIN2	8.758E-38	1.66	5.0949E-33	8.9164E-37	Q8WXE0	CASKIN2
1	CASP1	9.437E-193	-14.55	5.49E-188	2.323E-191	P29466	CASP1
1	CASP10	1.01E-226	-8.36	5.876E-222	2.702E-225	Q92851	CASP10
1	CASP1P1	0.00090267	-28.64	1	0.00352857		
1	CASP1P2	0.00034157	-10.67	1	0.00138314		
1	CASP5	2.8125E-06	-21.36	0.16361577	1.3146E-05	P51878	CASP5
1	CASQ1	0.00619566	-2.62	1	0.02228983	P31415	CASQ1
1	CASZ1	3.1768E-08	-1.69	0.00184806	1.6523E-07	Q86V15	CASZ1
1	CATSPER1	2.1003E-10	-1.88	1.2218E-05	1.205E-09	Q8NEC5	CATSPER1
1	CATSPER2	0	-2.07	0	0	Q96P56	CATSPER2

1	CATSPER2P1	1.729E-35	-2.92	1.0058E-30	1.7159E-34		
1	CATSPERB	1.0947E-06	-3.64	0.06368412	5.2454E-06	Q9H7T0	CATSPERB
1	CAV1	0	-3.94	0	0	Q03135	CAV1
1	CAV2	0	-3.44	0	0	P51636	CAV2
1	CBARP	7.5764E-50	2.88	4.4075E-45	8.8575E-49	Q8N350	CBARP
1	CBFA2T3	3.0541E-08	-7.32	0.00177666	1.5897E-07	O75081	CBFA2T3
1	CBLC	0	-12.87	0	0	Q9ULV8	CBLC
1	CBLN3	0.00017123	2.43	1	0.00071043	Q6UW01	CBLN3
1	CBR3	3.1151E-35	2.44	1.8122E-30	3.0809E-34	O75828	CBR3
1	CBR3-AS1	2.2274E-10	-2.05	1.2958E-05	1.276E-09		
1	CBR4	0	-1.84	0	0	Q8N4T8	CBR4
1	CBSL	1.236E-26	12.37	7.1903E-22	1.0682E-25	P0DN79	CBSL
1	CBWD1	1.881E-58	1.94	1.0942E-53	2.3706E-57	Q9BRT8	CBWD1
1	CBWD5	3.183E-109	2.39	1.852E-104	5.798E-108	Q5RIA9	CBWD5
1	CBWD7	2.0601E-12	2.31	1.1985E-07	1.2793E-11		
1	CBX2	9.7217E-73	11.76	5.6555E-68	1.3602E-71	Q14781	CBX2
1	CBX3P2	2.279E-09	-3.31	0.00013258	1.25E-08		
1	CBX3P4	0.00038576	-31.76	1	0.00155475		
1	CBX4	4.1365E-32	1.63	2.4063E-27	3.9236E-31	O00257	CBX4
1	CBX5	0	-1.98	0	0	P45973	CBX5
1	CBX6	6.222E-70	1.77	3.6196E-65	8.5207E-69	O95503	CBX6
1	CBX7	1.9761E-90	-2.92	1.1496E-85	3.1809E-89	O95931	CBX7
1	CBX8	2.1337E-27	1.90	1.2412E-22	1.8753E-26	Q9HC52	CBX8
1	CC2D1B	6.759E-51	1.51	3.932E-46	7.9692E-50	Q5T0F9	CC2D1B
1	CC2D2A	0	-2.29	0	0	Q9P2K1	CC2D2A
1	CCBE1	3.356E-116	98.90	1.953E-111	6.362E-115	Q6UXH8	CCBE1
1	CCDC102A	3.1166E-19	2.13	1.813E-14	2.3397E-18	Q96A19	CCDC102A
1	CCDC102B	6.1766E-07	-1.66	0.03593197	2.9998E-06	Q68D86	CCDC102B
1	CCDC103	2.6419E-07	-2.21	0.01536883	1.3082E-06	Q8IW40	CCDC103
1	CCDC106	1.0765E-50	1.95	6.2625E-46	1.2672E-49	Q9BWC9	CCDC106
1	CCDC107	1.3052E-95	3.90	7.5927E-91	2.1787E-94	Q8WV48	CCDC107
1	CCDC112	3.3115E-79	2.83	1.9264E-74	4.8807E-78	Q8NEF3	CCDC112
1	CCDC113	2.594E-114	-5.36	1.509E-109	4.864E-113	Q9H0I3	CCDC113
1	CCDC114	1.7912E-36	-6.12	1.042E-31	1.7994E-35	Q96M63	CCDC114
1	CCDC116	3.6258E-20	4.06	2.1093E-15	2.7746E-19	Q8IYX3	CCDC116
1	CCDC117	5.0753E-87	2.13	2.9525E-82	7.9561E-86	Q8IWD4	CCDC117
1	CCDC120	5.9427E-96	-8.34	3.4571E-91	9.9428E-95	Q96HB5	CCDC120
1	CCDC122	6.745E-110	-7.03	3.924E-105	1.234E-108		
1	CCDC124	1.305E-55	1.93	7.5916E-51	1.6111E-54	Q96CT7	CCDC124
1	CCDC127	0	-2.25	0	0		
1	CCDC13	4.4641E-14	4.62	2.597E-09	2.9288E-13	Q8IYE1	CCDC13
1	CCDC13-AS1	5.8106E-05	7.13	1	0.00024947		
1	CCDC134	5.5682E-11	1.51	3.2392E-06	3.2657E-10	Q9H6E4	CCDC134
1	CCDC136	2.7891E-17	2.02	1.6225E-12	2.0113E-16	Q96JN2	CCDC136
1	CCDC137	3.6884E-97	2.04	2.1457E-92	6.2193E-96	Q6PK04	CCDC137
1	CCDC141	0.00099573	-9.45	1	0.00388214	Q6ZP82	CCDC141
1	CCDC144A	0.00353645	-2.90	1	0.01307236		

1	CCDC144CP	8.8935E-12	-10.19	5.1737E-07	5.3926E-11		
1	CCDC146	1.0733E-23	-2.89	6.2436E-19	8.779E-23	Q8IYE0	CCDC146
1	CCDC148	9.3436E-10	-2.49	5.4356E-05	5.211E-09	Q8NFR7	CCDC148
1	CCDC149	1.1973E-12	-2.61	6.9654E-08	7.4881E-12		
1	CCDC15	1.9209E-24	1.86	1.1175E-19	1.5957E-23	Q0P6D6	CCDC15
1	CCDC151	1.423E-19	4.42	8.2782E-15	1.0756E-18	A5D8V7	CCDC151
1	CCDC152	5.561E-128	-8.68	3.235E-123	1.115E-126		
1	CCDC153	9.9831E-09	-3.39	0.00058076	5.3198E-08	Q494R4	CCDC153
1	CCDC154	0.00079594	-4.72	1	0.00312773	A6NI56	CCDC154
1	CCDC157	1.1086E-12	1.67	6.4494E-08	6.9423E-12		
1	CCDC158	1.0196E-08	-12.00	0.00059317	5.4305E-08		
1	CCDC168	3.7742E-10	4.19	2.1956E-05	2.1416E-09		
1	CCDC169	1.2873E-43	-4.76	7.4887E-39	1.4154E-42		
1	CCDC17	1.0502E-14	-2.19	6.1097E-10	7.0258E-14	Q96LX7	CCDC17
1	CCDC170	0.00574431	2.42	1	0.02074686	Q8IYT3	CCDC170
1	CCDC171	8.8891E-07	1.65	0.05171151	4.2797E-06		
1	CCDC173	5.4E-141	7.67	3.142E-136	1.134E-139		
1	CCDC18-AS1	1.999E-155	-4.69	1.163E-150	4.38E-154		
1	CCDC181	1.5822E-06	-2.93	0.0920441	7.5114E-06	Q5TID7	CCDC181
1	CCDC183	0.00226553	1.63	1	0.00851993	Q5T5S1	CCDC183
1	CCDC183-AS1	1.9116E-06	1.62	0.11120675	9.0317E-06		
1	CCDC186	0	-1.74	0	0		
1	CCDC187	0.00577935	-3.32	1	0.02086562	A0A096LP49	CCDC187
1	CCDC189	1.2507E-17	1.74	7.2757E-13	9.0947E-17		
1	CCDC190	3.483E-20	25.86	2.0262E-15	2.6657E-19		
1	CCDC192	3.0622E-32	-2.87	1.7814E-27	2.9108E-31		
1	CCDC24	0	-2.22	0	0	Q8N4L8	CCDC24
1	CCDC28A	8.5113E-24	1.68	4.9514E-19	6.9777E-23	Q8IWP9	CCDC28A
1	CCDC3	8.5556E-07	-2.80	0.04977141	4.1232E-06	Q9BQI4	CCDC3
1	CCDC33	6.434E-112	-9.13	3.743E-107	1.194E-110	Q8N5R6	CCDC33
1	CCDC34	1.0585E-50	2.18	6.1574E-46	1.2462E-49		
1	CCDC36	1.669E-14	-7.86	9.7092E-10	1.1094E-13	Q8IYA8	CCDC36
1	CCDC38	3.1763E-06	-2.54	0.18477912	1.4787E-05	Q502W7	CCDC38
1	CCDC40	2.305E-191	10.36	1.341E-186	5.64E-190	Q4G0X9	CCDC40
1	CCDC57	1.6407E-12	-1.52	9.5445E-08	1.0215E-11	Q2TAC2	CCDC57
1	CCDC58	1.0014E-23	1.65	5.8254E-19	8.1967E-23	Q4VC31	CCDC58
1	CCDC59	5.2771E-14	1.53	3.0699E-09	3.4547E-13	Q9P031	CCDC59
1	CCDC6	2.2036E-79	1.81	1.2819E-74	3.252E-78	Q16204	CCDC6
1	CCDC60	0.00040164	-10.61	1	0.00161649	Q8IWA6	CCDC60
1	CCDC62	2.1369E-17	3.23	1.2431E-12	1.5454E-16	Q6P9F0	CCDC62
1	CCDC68	2.0624E-22	-2.72	1.1998E-17	1.646E-21	Q9H2F9	CCDC68
1	CCDC69	7.2823E-11	1.72	4.2364E-06	4.256E-10	A6NI79	CCDC69
1	CCDC70	0.00048276	28.04	1	0.00193127	Q6NSX1	CCDC70
1	CCDC74A	5.0047E-20	1.87	2.9114E-15	3.8198E-19		
1	CCDC77	2.2728E-43	1.72	1.3222E-38	2.49E-42	Q9BR77	CCDC77
1	CCDC78	6.0431E-16	1.90	3.5155E-11	4.2112E-15	A2IDD5	CCDC78
1	CCDC84	8.5725E-13	1.63	4.987E-08	5.3884E-12		

1	CCDC85A	3.7771E-07	-45.87	0.02197268	1.8547E-06		
1	CCDC87	0.00433509	-1.68	1	0.01588796	Q9NVE4	CCDC87
1	CCDC88B	5.418E-208	-9.50	3.152E-203	1.391E-206	A6NC98	CCDC88B
1	CCDC88C	1.868E-129	14.72	1.087E-124	3.763E-128	Q9P219	CCDC88C
1	CCDC89	3.2879E-20	-6.45	1.9127E-15	2.5177E-19	Q8N998	CCDC89
1	CCDC94	7.9907E-57	2.07	4.6485E-52	9.9369E-56	Q9BW85	CCDC94
1	CCDC96	2.9954E-08	3.11	0.00174255	1.5606E-07	Q2M329	CCDC96
1	CCER2	2.0504E-09	-4.51	0.00011928	1.1267E-08	I3L3R5	CCER2
1	CCL17	0.0110027	-2.52	1	0.03842425	Q92583	CCL17
1	CCL28	1.4368E-58	-11.25	8.3583E-54	1.8139E-57	Q9NRJ3	CCL28
1	CCL5	1.8524E-07	-9.46	0.0107761	9.2443E-07	P13501	CCL5
1	CCNA1	1.23E-211	161.34	7.154E-207	3.191E-210	P78396	CCNA1
1	CCNB1IP1	5.2565E-87	2.08	3.0579E-82	8.2378E-86	Q9NPC3	CCNB1IP1
1	CCNB3	3.3128E-37	-12.90	1.9272E-32	3.3516E-36	Q8WWL7	CCNB3
1	CCND1	0	-2.00	0	0	P24385	CCND1
1	CCNE1	8.023E-187	6.35	4.667E-182	1.93E-185	P24864	CCNE1
1	CCNE2	7.0332E-28	1.97	4.0915E-23	6.2475E-27	O96020	CCNE2
1	CCNG1	1.2467E-20	1.65	7.2526E-16	9.6138E-20	P51959	CCNG1
1	CCNG2	0	-3.12	0	0	Q16589	CCNG2
1	CCNI2	5.463E-18	-5.08	3.178E-13	4E-17		
1	CCNJ	3.49E-120	3.70	2.03E-115	6.751E-119	Q5T5M9	CCNJ
1	CCNJL	3.207E-10	1.53	1.8656E-05	1.8237E-09	Q8IV13	CCNJL
1	CCNK	4.035E-119	1.77	2.347E-114	7.775E-118	O75909	CCNK
1	CCNL1	0	-1.52	0	0	Q9UK58	CCNL1
1	CCNO	1.1121E-59	2.54	6.4697E-55	1.4144E-58	P22674	CCNO
1	CCNY	1.0686E-68	3.67	6.2165E-64	1.4484E-67	Q8ND76	CCNY
1	CCNYL2	9.427E-178	80.51	5.484E-173	2.209E-176	Q5T2Q4	CCNYL2
1	CCR1	0.00030263	-25.36	1	0.00122984	P32246	CCR1
1	CCR10	1.2469E-14	3.84	7.2537E-10	8.3175E-14	P46092	CCR10
1	CCR3	6.4882E-94	-6.84	3.7745E-89	1.0714E-92	P51677	CCR3
1	CCR4	1.74E-05	12.45	1	7.733E-05	P51679	CCR4
1	CCR7	0.00592622	-2.24	1	0.02136937	P32248	CCR7
1	CCRL2	1.8564E-10	-6.62	1.0799E-05	1.0674E-09	O00421	CCRL2
1	CCS	0	-1.81	0	0	O14618	CCS
1	CCSER1	8.2456E-18	-2.15	4.7968E-13	6.0148E-17		
1	CCT2	5.9241E-86	2.46	3.4463E-81	9.2246E-85	P78371	CCT2
1	CCT6B	3.6859E-05	-1.69	1	0.00016046	Q92526	CCT6B
1	CCT8	1.2056E-86	2.51	7.0134E-82	1.8863E-85	P50990	CCT8
1	CCZ1	0	-3.04	0	0	P86791	CCZ1
1	CD101	3.0935E-86	-8.76	1.7996E-81	4.8221E-85	Q93033	CD101
1	CD109	0	-3.49	0	0	Q6YHK3	CD109
1	CD14	3.8935E-49	-12.11	2.265E-44	4.5236E-48	P08571	CD14
1	CD151	9.787E-35	1.62	5.6935E-30	9.6353E-34	P48509	CD151
1	CD160	0.00043683	1.93	1	0.00175341	O95971	CD160
1	CD163L1	6.0002E-07	-1.69	0.03490573	2.9159E-06	Q9NR16	CD163L1
1	CD164L2	2.2605E-06	-10.73	0.13150362	1.063E-05	Q6UWJ8	CD164L2
1	CD200R1	8.449E-22	-12.44	4.9151E-17	6.6691E-21	Q8TD46	CD200R1

1	CD200R1L	5.9935E-30	-10.68	3.4866E-25	5.5055E-29	Q6Q8B3	CD200R1L
1	CD24	0	-6.58	0	0	P25063	CD24
1	CD247	1.594E-07	-5.00	0.00927297	7.9857E-07	P20963	CD247
1	CD27-AS1	3.721E-206	-6.59	2.165E-201	9.535E-205		
1	CD274	1.6739E-49	1.98	9.7375E-45	1.9502E-48	Q9NZQ7	CD274
1	CD2AP	0	-1.66	0	0	Q9Y5K6	CD2AP
1	CD2BP2	2.7736E-76	2.13	1.6135E-71	3.9929E-75	O95400	CD2BP2
1	CD302	7.0152E-08	-1.62	0.00408101	3.5798E-07	Q8IX05	CD302
1	CD320	2.195E-99	2.77	1.2771E-94	3.7639E-98	Q9NPF0	CD320
1	CD33	4.8061E-10	4.37	2.7959E-05	2.7126E-09	P20138	CD33
1	CD36	0.00091026	-5.31	1	0.00355704	P16671	CD36
1	CD37	0.00011674	1.96	1	0.00049056	P11049	CD37
1	CD38	1.4842E-18	-10.62	8.634E-14	1.0986E-17	P28907	CD38
1	CD3EAP	1.3559E-37	1.94	7.888E-33	1.3773E-36	O15446	CD3EAP
1	CD40	0	96.43	0	0	P25942	CD40
1	CD46	0	-3.54	0	0	P15529	CD46
1	CD47	0	-4.43	0	0	Q08722	CD47
1	CD5	2.9024E-10	-14.27	1.6885E-05	1.6545E-09	P06127	CD5
1	CD53	1.3502E-10	2.39	7.8546E-06	7.8124E-10	P19397	CD53
1	CD55	0	-3.99	0	0	P08174	CD55
1	CD59	1.6753E-41	1.64	9.7458E-37	1.7879E-40	P13987	CD59
1	CD6	1.3996E-15	-18.45	8.142E-11	9.6196E-15	P30203	CD6
1	CD68	0	-4.29	0	0	P34810	CD68
1	CD70	2.645E-169	10.67	1.539E-164	6.044E-168	P32970	CD70
1	CD72	0.0010206	-1.69	1	0.00397513	P21854	CD72
1	CD74	1.129E-208	-19.35	6.569E-204	2.907E-207	P04233	CD74
1	CD81-AS1	1.482E-32	4.93	8.6214E-28	1.4154E-31		
1	CD82	3.2249E-75	-9.49	1.8761E-70	4.6016E-74	P27701	CD82
1	CD83	2.728E-165	-4.68	1.587E-160	6.15E-164	Q01151	CD83
1	CD86	1.3806E-09	-6.96	8.0318E-05	7.6457E-09	P42081	CD86
1	CD8A	2.3689E-13	-6.20	1.3781E-08	1.5165E-12	P01732	CD8A
1	CD8B	6.1783E-05	-12.48	1	0.00026471	P10966	CD8B
1	CD9	0	-3.95	0	0	P21926	CD9
1	CD93	1.0209E-24	-41.63	5.9392E-20	8.5248E-24	Q9NPY3	CD93
1	CD96	2.6301E-23	-3.13	1.53E-18	2.1363E-22	P40200	CD96
1	CD99	8.2145E-73	1.99	4.7787E-68	1.1504E-71	P14209	CD99
1	CD99L2	1.5655E-63	3.35	9.1071E-59	2.0507E-62	Q8TCZ2	CD99L2
1	CDA	7.2326E-33	-6.80	4.2075E-28	6.9465E-32	P32320	CDA
1	CDC16	1.0993E-94	1.76	6.3951E-90	1.8251E-93	Q13042	CDC16
1	CDC20B	3.5717E-06	1.95	0.2077799	1.656E-05	Q86Y33	CDC20B
1	CDC34	8.2758E-52	1.91	4.8144E-47	9.8453E-51	P49427	CDC34
1	CDC37	1.9194E-48	2.19	1.1166E-43	2.2151E-47	Q16543	CDC37
1	CDC37L1-AS1	6.214E-14	-2.68	3.615E-09	4.0577E-13		
1	CDC37P1	0.00221501	26.07	1	0.00834127		
1	CDC42BPB	6.0228E-77	2.30	3.5037E-72	8.707E-76	Q9Y5S2	CDC42BPB
1	CDC42BPG	5.423E-237	-9.62	3.155E-232	1.466E-235	Q6DT37	CDC42BPG
1	CDC42EP2	1.0682E-62	2.13	6.2143E-58	1.3908E-61	O14613	CDC42EP2

1	CDC42EP3	8.309E-117	4.71	4.833E-112	1.581E-115	Q9UKI2	CDC42EP3
1	CDC42EP5	5.839E-110	-11.33	3.397E-105	1.068E-108	Q6NZY7	CDC42EP5
1	CDC42SE1	0	-2.90	0	0	Q9NRR8	CDC42SE1
1	CDC45	3.4371E-92	1.75	1.9995E-87	5.6087E-91	O75419	CDC45
1	CDC6	5.5269E-88	2.32	3.2152E-83	8.7394E-87	Q99741	CDC6
1	CDCA2	0	-1.70	0	0	Q69YH5	CDCA2
1	CDCA4	6.639E-31	3.00	3.8621E-26	6.1923E-30	Q9BXL8	CDCA4
1	CDH1	0	-20.20	0	0	P12830	CDH1
1	CDH10	6.7466E-21	-4.94	3.9248E-16	5.231E-20	Q9Y6N8	CDH10
1	CDH12	3.7432E-06	-1.65	0.21775941	1.7338E-05	P55289	CDH12
1	CDH15	0.00046951	2.17	1	0.00188029	P55291	CDH15
1	CDH17	2.8395E-06	-3.16	0.1651833	1.3263E-05	Q12864	CDH17
1	CDH18	1.279E-176	-10.28	7.441E-172	2.988E-175	Q13634	CDH18
1	CDH19	4.5406E-20	-13.95	2.6415E-15	3.4692E-19	Q9H159	CDH19
1	CDH2	3.446E-120	8.55	2.004E-115	6.668E-119	P19022	CDH2
1	CDH23	3.469E-11	-2.99	2.0181E-06	2.0534E-10	Q9H251	CDH23
1	CDH24	0	-2.06	0	0	Q86UP0	CDH24
1	CDH26	5.4321E-16	2.58	3.1601E-11	3.7914E-15	Q8IXH8	CDH26
1	CDH3	0	-18.45	0	0	P22223	CDH3
1	CDH4	4.084E-199	15.04	2.376E-194	1.026E-197	P55283	CDH4
1	CDH5	5.8678E-71	-8.10	3.4135E-66	8.0985E-70	P33151	CDH5
1	CDH6	3.0314E-52	125.44	1.7635E-47	3.6249E-51	P55285	CDH6
1	CDH7	7.0892E-12	-10.57	4.1241E-07	4.3121E-11	Q9ULB5	CDH7
1	CDH8	1.9702E-30	-2.86	1.1462E-25	1.8239E-29	P55286	CDH8
1	CDHR3	1.1477E-05	-1.51	0.66763772	5.1623E-05	Q6ZTQ4	CDHR3
1	CDHR5	7.9355E-05	-3.03	1	0.00033755	Q9HBB8	CDHR5
1	CDIP1	4.104E-180	7.44	2.388E-175	9.678E-179	Q9H305	CDIP1
1	CDK10	0	-2.03	0	0	Q15131	CDK10
1	CDK11A	2.7897E-20	1.58	1.6229E-15	2.1379E-19	Q9UQ88	CDK11A
1	CDK11B	1.0558E-85	1.76	6.1418E-81	1.6391E-84	P21127	CDK11B
1	CDK13	0	-1.87	0	0	Q14004	CDK13
1	CDK14	6.8221E-47	-7.23	3.9687E-42	7.7483E-46	O94921	CDK14
1	CDK15	0.00016982	-8.17	1	0.00070476	Q96Q40	CDK15
1	CDK16	0	-1.81	0	0	Q00536	CDK16
1	CDK17	7.0343E-62	4.14	4.0921E-57	9.0956E-61	Q00537	CDK17
1	CDK18	1.1087E-97	-15.75	6.4496E-93	1.8776E-96	Q07002	CDK18
1	CDK20	1.0321E-93	97.81	6.0041E-89	1.7018E-92	Q8IZL9	CDK20
1	CDK5	5.435E-123	3.64	3.162E-118	1.066E-121	Q00535	CDK5
1	CDK5R1	3.4094E-11	1.81	1.9834E-06	2.0191E-10	Q15078	CDK5R1
1	CDK5RAP1	5.957E-36	1.51	3.4654E-31	5.9421E-35	Q96SZ6	CDK5RAP1
1	CDK7	2.2106E-15	1.56	1.286E-10	1.5121E-14	P50613	CDK7
1	CDKL1	5.162E-12	1.69	3.0029E-07	3.1573E-11	Q00532	CDKL1
1	CDKL2	2.6594E-25	-9.67	1.5471E-20	2.2448E-24	Q92772	CDKL2
1	CDKL3	6.5768E-05	1.62	1	0.00028128	Q8IVW4	CDKL3
1	CDKN1A	0	-3.55	0	0	P38936	CDKN1A
1	CDKN1B	3.5809E-28	1.57	2.0831E-23	3.1969E-27	P46527	CDKN1B
1	CDKN1C	1.3707E-12	4.94	7.9736E-08	8.5499E-12	P49918	CDKN1C

1	CDKN2A	0	-2.51	0	0	P42771	CDKN2A
1	CDKN2AIPNL	1.2568E-40	1.77	7.3111E-36	1.3242E-39	Q96HQ2	CDKN2AIPNL
1	CDKN2D	9.4864E-19	1.82	5.5186E-14	7.058E-18	P55273	CDKN2D
1	CDKN3	1.2481E-33	1.87	7.2609E-29	1.2112E-32	Q16667	CDKN3
1	CDNF	9.5016E-06	-1.78	0.55274375	4.2982E-05	Q49AH0	CDNF
1	CDON	3.7689E-60	3.51	2.1925E-55	4.8082E-59	Q4KMG0	CDON
1	CDPF1	4.7018E-55	2.10	2.7352E-50	5.7753E-54	Q6NVV7	CDPF1
1	CDR1	3.5479E-30	303.77	2.0639E-25	3.2714E-29	P51861	CDR1
1	CDRT1	2.767E-135	-6.67	1.61E-130	5.682E-134	O95170	CDRT1
1	CDRT15P1	0.00166276	-4.13	1	0.00634375		
1	CDRT4	0.01197711	-2.54	1	0.04164714		
1	CDS1	0	-8.65	0	0	Q92903	CDS1
1	CDT1	1.1254E-25	1.58	6.5471E-21	9.5537E-25	Q9H211	CDT1
1	CDV3	8.8056E-32	1.50	5.1226E-27	8.3118E-31	Q9UKY7	CDV3
1	CDYL	0	-2.34	0	0	Q9Y232	CDYL
1	CDYL2	3.16E-141	29.94	1.838E-136	6.647E-140	Q8N8U2	CDYL2
1	CEACAM1	8.5962E-13	-7.37	5.0007E-08	5.4027E-12	P13688	CEACAM1
1	CEACAM19	8.0425E-50	-3.22	4.6786E-45	9.3968E-49	Q7Z692	CEACAM19
1	CEACAM22P	2.7754E-08	-3.26	0.00161458	1.4484E-07		
1	CEACAM6	8.9517E-22	17.94	5.2076E-17	7.0621E-21	P40199	CEACAM6
1	CEACAMP5	0.00144125	-21.63	1	0.00552692		
1	CEBPA	4.9688E-58	-6.18	2.8906E-53	6.2404E-57	P49715	CEBPA
1	CEBPB-AS1	3.7288E-05	-1.76	1	0.00016224		
1	CEBPD	5.0557E-59	-3.77	2.9411E-54	6.4021E-58	P49716	CEBPD
1	CECR1	0.00411104	-19.69	1	0.01510487		
1	CECR6	0.00048634	3.42	1	0.00194501		
1	CECR7	6.1908E-11	27.22	3.6015E-06	3.6247E-10		
1	CEL	8.135E-165	-6.41	4.732E-160	1.831E-163	P19835	CEL
1	CELA2B	1.7044E-05	-1.99	0.99151163	7.5809E-05	P08218	CELA2B
1	CELF3	0.00216295	-23.60	1	0.0081563	Q5SZQ8	CELF3
1	CELF5	2.8441E-07	6.38	0.01654555	1.4055E-06	Q8N6W0	CELF5
1	CELP	1.5841E-13	-11.45	9.2156E-09	1.0208E-12		
1	CELSR1	0	-3.83	0	0	Q9NYQ6	CELSR1
1	CELSR2	0	-2.88	0	0	Q9HCU4	CELSR2
1	CELSR3-AS1	2.5044E-07	1.88	0.01456905	1.2416E-06		
1	CEMIP	4.111E-06	-2.34	0.23915547	1.8984E-05	Q8WUJ3	CEMIP
1	CENPB	1.81E-129	2.54	1.053E-124	3.647E-128	P07199	CENPB
1	CENPC	0	-1.72	0	0	Q03188	CENPC
1	CENPF	0	-1.88	0	0	P49454	CENPF
1	CENPI	1.4312E-36	1.79	8.3257E-32	1.4394E-35	Q92674	CENPI
1	CENPK	3.1609E-25	1.56	1.8388E-20	2.665E-24	Q9BS16	CENPK
1	CENPM	1.1833E-39	1.80	6.8838E-35	1.2323E-38	Q9NSP4	CENPM
1	CENPO	1.4122E-31	1.51	8.2156E-27	1.3289E-30	Q9BU64	CENPO
1	CENPS	1.9123E-14	1.61	1.1124E-09	1.2693E-13	Q8N2Z9	CENPS
1	CENPU	0	-2.38	0	0	Q71F23	CENPU
1	CENPV	8.1855E-79	7.95	4.7618E-74	1.2025E-77	Q7Z7K6	CENPV
1	CENPW	3.8326E-14	1.88	2.2296E-09	2.5193E-13	Q5EE01	CENPW

1	CENPX	1.828E-125	2.73	1.063E-120	3.62E-124	A8MT69	CENPX
1	CEP112	2.675E-51	2.79	1.5561E-46	3.1654E-50	Q8N8E3	CEP112
1	CEP126	2.5988E-27	-3.07	1.5118E-22	2.2796E-26	Q9P2H0	CEP126
1	CEP128	1.2282E-98	2.46	7.145E-94	2.0953E-97	Q6ZU80	CEP128
1	CEP131	8.5255E-23	1.59	4.9596E-18	6.8664E-22	Q9UPN4	CEP131
1	CEP152	4.4985E-11	-1.52	2.617E-06	2.6487E-10	O94986	CEP152
1	CEP162	1.0944E-19	1.64	6.3668E-15	8.2987E-19	Q5TB80	CEP162
1	CEP164P1	4.016E-14	3.52	2.3363E-09	2.6384E-13		
1	CEP170	6.4932E-89	2.95	3.7773E-84	1.0343E-87	Q5SW79	CEP170
1	CEP170B	1.3141E-30	1.81	7.6447E-26	1.2198E-29	Q9Y4F5	CEP170B
1	CEP170P1	3.1622E-05	4.33	1	0.00013826	Q96L14	CEP170P1
1	CEP350	0	-1.83	0	0	Q5VT06	CEP350
1	CEP41	4.7461E-38	1.60	2.761E-33	4.8506E-37	Q9BYV8	CEP41
1	CEP44	0	-3.02	0	0	Q9C0F1	CEP44
1	CEP70	0	-2.57	0	0	Q8NHQ1	CEP70
1	CEP72	0	-2.30	0	0	Q9P209	CEP72
1	CEP83-AS1	2.7976E-08	-2.02	0.00162745	1.4595E-07		
1	CEP85L	1.4196E-30	2.43	8.2582E-26	1.3169E-29	Q5SZL2	CEP85L
1	CERCAM	0	-1.50	0	0	Q5T4B2	CERCAM
1	CERK	5.7681E-53	1.58	3.3555E-48	6.9444E-52	Q8TCT0	CERK
1	CERKL	3.2875E-14	-13.78	1.9125E-09	2.1659E-13	Q49MI3	CERKL
1	CERNA1	1.3844E-14	-6.39	8.0538E-10	9.218E-14		
1	CERS1	2.1644E-41	5.45	1.2591E-36	2.3069E-40	P27544	CERS1
1	CERS2	0	-1.92	0	0	Q96G23	CERS2
1	CERS3	0.00663719	-2.77	1	0.02378856	Q8IU89	CERS3
1	CERS4	1.621E-162	96.36	9.43E-158	3.624E-161	Q9HA82	CERS4
1	CERS6	0	-1.59	0	0	Q6ZMG9	CERS6
1	CES2	0	-1.79	0	0	O00748	CES2
1	CES3	2.7157E-13	-2.94	1.5798E-08	1.7345E-12	Q6UWW8	CES3
1	CES4A	1.457E-27	-8.64	8.4762E-23	1.2847E-26	Q5XG92	CES4A
1	CFAP157	2.2597E-06	1.91	0.13145689	1.0627E-05	Q5JU67	CFAP157
1	CFAP161	2.6799E-09	-3.29	0.0001559	1.4666E-08		
1	CFAP36	2.3608E-27	1.54	1.3734E-22	2.0727E-26	Q96G28	CFAP36
1	CFAP43	7.4501E-13	-2.91	4.334E-08	4.6941E-12	Q8NDM7	CFAP43
1	CFAP46	0.0002196	-3.75	1	0.00090393	Q8IYW2	CFAP46
1	CFAP47	0.00011097	-3.51	1	0.00046706		
1	CFAP52	2.1592E-30	-3.47	1.2561E-25	1.9966E-29	Q8N1V2	CFAP52
1	CFAP53	1.3702E-23	3.07	7.9711E-19	1.1194E-22	Q96M91	CFAP53
1	CFAP54	1.0511E-11	1.86	6.1146E-07	6.3594E-11	Q96N23	CFAP54
1	CFAP57	3.2404E-15	-2.83	1.8851E-10	2.2058E-14		
1	CFAP58	2.0314E-05	-1.78	1	8.9915E-05	Q5T655	CFAP58
1	CFAP58-AS1	0.00024547	-3.42	1	0.00100578		
1	CFAP70	2.1078E-07	1.78	0.01226214	1.0492E-06	Q5T0N1	CFAP70
1	CFAP74	0.00155899	-2.67	1	0.00596349	Q9C0B2	CFAP74
1	CFAP97	0	-2.24	0	0		
1	CFAP99	0.00650631	-4.53	1	0.02333815	D6REC4	CFAP99
1	CFB	2.448E-106	-7.17	1.424E-101	4.375E-105	P00751	CFB

1	CFD	4.3833E-12	-4.19	2.55E-07	2.6878E-11	P00746	CFD
1	CFH	0	-5.88	0	0	P08603	CFH
1	CFHR1	4.3127E-10	-12.05	2.5089E-05	2.4394E-09	Q03591	CFHR1
1	CFHR3	5.1496E-11	-12.53	2.9957E-06	3.0269E-10	Q02985	CFHR3
1	CFHR4	4.883E-193	-2084.66	2.841E-188	1.203E-191	Q92496	CFHR4
1	CFHR5	2.4835E-06	-48.29	0.14447686	1.1647E-05	Q9BXR6	CFHR5
1	CFL2	7.014E-101	4.96	4.08E-96	1.212E-99	Q9Y281	CFL2
1	CFTR	1.0249E-06	-12.45	0.05962201	4.9169E-06	P13569	CFTR
1	CGB7	3.5334E-27	-4.15	2.0555E-22	3.0882E-26	P0DN87	CGB7
1	CGN	0	-4.09	0	0	Q9P2M7	CGN
1	CGNL1	0.00012197	-4.38	1	0.00051146	Q0VF96	CGNL1
1	CGREF1	0.00024868	6.12	1	0.00101821	Q99674	CGREF1
1	CGRRF1	3.9667E-80	3.06	2.3076E-75	5.8943E-79	Q99675	CGRRF1
1	CH17-125A10	0.00859959	-6.24	1	0.03043577		
1	CH17-140K24	2.9882E-30	-4.18	1.7383E-25	2.758E-29		
1	CH17-140K24	0.00119036	-8.75	1	0.00460639		
1	CH17-260O16	0.00433874	-3.04	1	0.01589933		
1	CH17-268I9.2	6.8719E-45	-15.72	3.9977E-40	7.6408E-44		
1	CH17-296N19	2.519E-19	15.58	1.4654E-14	1.8943E-18		
1	CH17-302M23	0.00073067	-2.71	1	0.00287902		
1	CH17-335B8.4	5.2256E-05	-3.18	1	0.0002251		
1	CH17-353B19	1.9303E-10	-5.73	1.1229E-05	1.1091E-09		
1	CH17-360D5.2	0	-2.60	0	0		
1	CH17-360D5.3	1.4546E-88	-11.24	8.4623E-84	2.3096E-87		
1	CH17-408M7.	1.6372E-09	-10.17	9.5241E-05	9.031E-09		
1	CH17-437K3.1	1.1272E-07	3.32	0.0065573	5.6936E-07		
1	CH17-472G23	1.6818E-11	8.55	9.7834E-07	1.01E-10		
1	CH17-472G23	2.6988E-29	-2.41	1.57E-24	2.4524E-28		
1	CH17-472G23	1.1558E-34	2.33	6.724E-30	1.137E-33		
1	CH17-78J1.1	7.1614E-08	-7.02	0.00416605	3.6512E-07		
1	CH17-80A12.1	9.1005E-05	2.67	1	0.00038537		
1	CH25H	0.00890563	-8.32	1	0.03145194	O95992	CH25H
1	CH507-145C2	0.00060128	1.74	1	0.00238683		
1	CH507-145C2	0.00041977	1.61	1	0.00168702		
1	CH507-145C2	0.00049112	4.80	1	0.00196333		
1	CH507-152C1	0.00178118	-3.09	1	0.0067729		
1	CH507-210P1	5.5283E-21	-4.49	3.2161E-16	4.2932E-20		
1	CH507-236L2	0.0049639	2.85	1	0.01806055		
1	CH507-24F1.2	0.00107865	3.35	1	0.00418914		
1	CH507-254M2	9.3554E-06	1.90	0.54424084	4.2343E-05		
1	CH507-254M2	1.0039E-47	3.51	5.8401E-43	1.1503E-46		
1	CH507-39O4.1	0.0001493	8.54	1	0.00062181		
1	CH507-39O4.2	0.00023166	2.45	1	0.00095154		
1	CH507-42P11	2.0491E-05	-3.44	1	9.0664E-05		
1	CH507-42P11	4.2249E-12	-17.65	2.4578E-07	2.5921E-11		
1	CH507-42P11	1.3422E-28	-11.28	7.808E-24	1.2057E-27		
1	CH507-42P11	0	-4.35	0	0		

1	CH507-9B2.4	0.00140301	-3.54	1	0.00539451		
1	CH507-9B2.8	1.6279E-11	-30.04	9.4703E-07	9.7844E-11		
1	CHAC2	3.1054E-10	1.63	1.8065E-05	1.7671E-09	Q8WUX2	CHAC2
1	CHAD	3.7435E-05	-3.39	1	0.00016287	O15335	CHAD
1	CHADL	1.9119E-13	-2.98	1.1122E-08	1.2293E-12	Q6NUI6	CHADL
1	CHAF1B	1.2318E-65	2.12	7.1658E-61	1.6413E-64	Q13112	CHAF1B
1	CHAMP1	1.6538E-33	1.58	9.6211E-29	1.6016E-32	Q96JM3	CHAMP1
1	CHCHD1	3.5475E-24	1.93	2.0637E-19	2.9289E-23	Q96BP2	CHCHD1
1	CHCHD4	8.9478E-30	1.69	5.2053E-25	8.2038E-29	Q8N4Q1	CHCHD4
1	CHD1	4.8536E-26	1.58	2.8235E-21	4.1467E-25	O14646	CHD1
1	CHD1L	0	-1.58	0	0	Q86WJ1	CHD1L
1	CHD7	0	-2.07	0	0	Q9P2D1	CHD7
1	CHFR	2.959E-117	2.24	1.721E-112	5.643E-116	Q96EP1	CHFR
1	CHKA	0	-4.31	0	0	P35790	CHKA
1	CHKB-AS1	0.00111451	1.93	1	0.0043238		
1	CHMP1B2P	1.954E-140	-18.00	1.137E-135	4.097E-139		
1	CHMP2A	0	-2.50	0	0	O43633	CHMP2A
1	CHMP4A	4.8768E-48	2.73	2.837E-43	5.6101E-47	Q9BY43	CHMP4A
1	CHMP4B	5.8801E-39	1.62	3.4207E-34	6.079E-38	Q9H444	CHMP4B
1	CHMP4C	0	-3.63	0	0	Q96CF2	CHMP4C
1	CHMP5	4.0397E-17	1.53	2.3501E-12	2.902E-16	Q9NZZ3	CHMP5
1	CHMP6	2.161E-111	2.29	1.257E-106	3.997E-110	Q96FZ7	CHMP6
1	CHN1	2.936E-95	3.21	1.708E-90	4.8954E-94	P15882	CHN1
1	CHN2	1.4822E-94	-8.39	8.6223E-90	2.4572E-93	P52757	CHN2
1	CHODL	1.672E-06	-9.13	0.09726501	7.9271E-06	Q9H9P2	CHODL
1	CHPF2	5.228E-110	2.16	3.041E-105	9.569E-109	Q9P2E5	CHPF2
1	CHPT1	1.017E-47	1.91	5.9162E-43	1.1648E-46	Q8WUD6	CHPT1
1	CHRDL1	0.01186113	7.58	1	0.04128343	Q9BU40	CHRDL1
1	CHRDL2	0.00019369	2.30	1	0.00080116	Q6WN34	CHRDL2
1	CHRFAM7A	3.1812E-25	25.77	1.8506E-20	2.6816E-24	Q494W8	CHRFAM7A
1	CHRM1	0.00221894	-19.72	1	0.00835552	P11229	CHRM1
1	CHRM3	7.3838E-63	-11.23	4.2955E-58	9.6203E-62	P20309	CHRM3
1	CHRNA10	6.3734E-12	2.14	3.7077E-07	3.8836E-11	Q9GZZ6	CHRNA10
1	CHRNA2	0.00942764	-19.34	1	0.03316063	Q15822	CHRNA2
1	CHRNA6	0.00725479	-3.95	1	0.02590156	Q15825	CHRNA6
1	CHRNA7	1.289E-47	46.76	7.4984E-43	1.4752E-46	P36544	CHRNA7
1	CHRNB2	0.00023783	3.33	1	0.00097605	P17787	CHRNB2
1	CHRNB4	6.7464E-18	-8.44	3.9247E-13	4.9305E-17	P30926	CHRNB4
1	CHRNE	8.6105E-10	9.55	5.0091E-05	4.8099E-09	Q04844	CHRNE
1	CHST10	2.007E-138	193.31	1.167E-133	4.174E-137	O43529	CHST10
1	CHST11	0	-2.80	0	0	Q9NPF2	CHST11
1	CHST15	1.4774E-64	-7.50	8.5944E-60	1.9515E-63	Q7LFX5	CHST15
1	CHST2	5.054E-118	5.47	2.94E-113	9.672E-117	Q9Y4C5	CHST2
1	CHST3	0	-3.35	0	0	Q7LGC8	CHST3
1	CHSY1	4.306E-119	2.08	2.505E-114	8.294E-118	Q86X52	CHSY1
1	CHSY3	6.7499E-49	-4.72	3.9267E-44	7.8205E-48	Q70JA7	CHSY3
1	CHURC1	2.0843E-19	1.62	1.2125E-14	1.5702E-18	Q8WUH1	CHURC1

1	CIAO1	7.828E-64	1.54	4.5538E-59	1.0282E-62	O76071	CIAO1
1	CIART	7.2883E-96	-9.34	4.2399E-91	1.2184E-94	Q8N365	CIART
1	CIB2	3.1503E-07	5.52	0.01832632	1.5529E-06	O75838	CIB2
1	CICP16	6.6847E-06	-3.36	0.38887444	3.0543E-05		
1	CICP6	0.004673	-3.44	1	0.01706084		
1	CIDEA	2.6739E-08	-4.81	0.00155552	1.3966E-07	O60543	CIDEA
1	CILP	0.00129442	23.79	1	0.00499479	O75339	CILP
1	CILP2	0.00051885	2.53	1	0.00207115	Q8IUL8	CILP2
1	CINP	1.1257E-71	2.39	6.5486E-67	1.5618E-70	Q9BW66	CINP
1	CIPC	2.2326E-54	1.83	1.2988E-49	2.7257E-53	Q9C0C6	CIPC
1	CISD1	6.2375E-46	2.02	3.6286E-41	7.0145E-45	Q9NZ45	CISD1
1	CIT	0	-1.68	0	0	O14578	CIT
1	CITED1	0.00075594	2.76	1	0.00297639	Q99966	CITED1
1	CITED2	7.1592E-56	3.18	4.1648E-51	8.8556E-55	Q99967	CITED2
1	CITED4	1.5252E-29	-2.58	8.8727E-25	1.392E-28	Q96RK1	CITED4
1	CKB	0	-14.78	0	0	P12277	CKB
1	CKLF	9.9269E-70	2.05	5.7749E-65	1.3562E-68	Q9UBR5	CKLF
1	CKMT1A	0	-12.34	0	0	P12532	CKMT1A
1	CKMT1B	8.609E-200	-10.90	5.008E-195	2.17E-198		
1	CLCA2	8.6299E-07	-6.33	0.05020351	4.1576E-06	Q9UQC9	CLCA2
1	CLCA3P	0.00014601	-29.07	1	0.00060876	Q9Y6N3	CLCA3P
1	CLCA4	0.00023025	-7.46	1	0.00094586	Q14CN2	CLCA4
1	CLCF1	2.797E-188	-4.38	1.627E-183	6.76E-187	Q9UBD9	CLCF1
1	CLCN1	2.4698E-34	-39.25	1.4368E-29	2.4188E-33	P35523	CLCN1
1	CLCN2	2.8866E-15	-1.53	1.6792E-10	1.9679E-14	P51788	CLCN2
1	CLCN3	0	-2.35	0	0	P51790	CLCN3
1	CLCN3P1	0.00026159	-7.10	1	0.00106868		
1	CLCN5	0	-1.89	0	0	P51795	CLCN5
1	CLCNKA	0.00032111	-29.07	1	0.00130229	P51800	CLCNKA
1	CLCNKB	9.3586E-26	-66.09	5.4443E-21	7.9583E-25	P51801	CLCNKB
1	CLDN10	4.9559E-12	5.32	2.8831E-07	3.0332E-11	P78369	CLDN10
1	CLDN11	0	-3.09	0	0	O75508	CLDN11
1	CLDN12	0	-2.47	0	0	P56749	CLDN12
1	CLDN16	5.7093E-09	-2.80	0.00033213	3.075E-08	Q9Y5I7	CLDN16
1	CLDN2	2.4866E-53	11.30	1.4465E-48	3.0073E-52	P57739	CLDN2
1	CLDN22	0.00140517	-26.35	1	0.00539816	Q8N7P3	CLDN22
1	CLDN23	1.0511E-31	-5.97	6.1146E-27	9.9102E-31	Q96B33	CLDN23
1	CLDN24	6.0812E-14	-6.86	3.5377E-09	3.9722E-13	A6NM45	CLDN24
1	CLDN3	0.00043461	-7.09	1	0.00174487	O15551	CLDN3
1	CLDN4	0	-18.58	0	0	O14493	CLDN4
1	CLDN7	0	-17.12	0	0	O95471	CLDN7
1	CLDN9	6.3485E-16	-7.39	3.6932E-11	4.4208E-15	O95484	CLDN9
1	CLDND2	1.1177E-14	3.32	6.502E-10	7.471E-14	Q8NHS1	CLDND2
1	CLEC11A	7.73E-221	53.39	4.497E-216	2.047E-219	Q9Y240	CLEC11A
1	CLEC20A	1.5359E-10	-6.10	8.9347E-06	8.8612E-10	Q6ZU45	CLEC20A
1	CLEC2B	2.1469E-22	-2.19	1.2489E-17	1.7127E-21	Q92478	CLEC2B
1	CLEC4A	0.00171388	1.73	1	0.00652978	Q9UMR7	CLEC4A

1	CLEC4F	0.00052183	-5.41	1	0.00208193	Q8N1N0	CLEC4F
1	CLGN	1.3773E-98	-4.12	8.0126E-94	2.349E-97	O14967	CLGN
1	CLIC1	1.0623E-42	1.79	6.1801E-38	1.1526E-41	O00299	CLIC1
1	CLIC3	5.655E-120	-21.31	3.29E-115	1.093E-118	O95833	CLIC3
1	CLIC4	4.0906E-75	2.63	2.3797E-70	5.8311E-74	Q9Y696	CLIC4
1	CLIC5	0.00057531	-2.78	1	0.00228812	Q9NZA1	CLIC5
1	CLIP3	2.5758E-08	-2.19	0.00149846	1.3464E-07	Q96DZ5	CLIP3
1	CLIP4	0	-4.28	0	0	Q8N3C7	CLIP4
1	CLK2	0	-1.53	0	0	P49760	CLK2
1	CLK3	0	-1.63	0	0	P49761	CLK3
1	CLK4	3.4688E-51	1.97	2.0179E-46	4.0982E-50	Q9HAZ1	CLK4
1	CLMN	1.7632E-71	10.97	1.0257E-66	2.4434E-70	Q96JQ2	CLMN
1	CLMP	1.1437E-92	6.40	6.6534E-88	1.8716E-91	Q9H6B4	CLMP
1	CLN8	0	-2.19	0	0	Q9UBY8	CLN8
1	CLOCK	0	-2.08	0	0	O15516	CLOCK
1	CLPP	6.1971E-90	2.12	3.6051E-85	9.9424E-89	Q16740	CLPP
1	CLPTM1L	0	-2.11	0	0	Q96KA5	CLPTM1L
1	CLRN1-AS1_1	1.0672E-12	-6.50	6.2081E-08	6.6855E-12		
1	CLSPN	3.467E-142	2.36	2.017E-137	7.32E-141	Q9HAW4	CLSPN
1	CLSTN1	0	-2.52	0	0	O94985	CLSTN1
1	CLSTN2	4.6135E-11	-14.78	2.6839E-06	2.7151E-10	Q9H4D0	CLSTN2
1	CLSTN2-AS1	0.00058679	-30.45	1	0.0023317		
1	CLTA	3.353E-99	2.35	1.9508E-94	5.7375E-98	P09496	CLTA
1	CLTCL1	5.3963E-46	2.11	3.1392E-41	6.0755E-45	P53675	CLTCL1
1	CLU	0	-2.39	0	0	P10909	CLU
1	CLUAP1	2.0105E-55	1.89	1.1696E-50	2.4779E-54	Q96AJ1	CLUAP1
1	CLUHP3	0	-1.79	0	0		
1	CLUL1	1.293E-14	-6.22	7.5219E-10	8.6191E-14	Q15846	CLUL1
1	CLYBL	1.1806E-71	-2.84	6.868E-67	1.6376E-70	Q8N0X4	CLYBL
1	CMAS	3.8463E-38	1.77	2.2375E-33	3.9358E-37	Q8NFW8	CMAS
1	CMB9-22P13.	7.6326E-49	3.07	4.4402E-44	8.8344E-48		
1	CMC4	2.5921E-06	1.57	0.15079324	1.2141E-05	P56277	CMC4
1	CMIP	2.8803E-49	1.53	1.6756E-44	3.3511E-48	Q8IY22	CMIP
1	CMSS1	4.4229E-57	1.98	2.573E-52	5.512E-56	Q9BQ75	CMSS1
1	CMTM3	5.008E-100	2.77	2.9135E-95	8.61E-99	Q96MX0	CMTM3
1	CMTM4	0	-2.08	0	0	Q8IZR5	CMTM4
1	CMTM6	0	-1.86	0	0	Q9NX76	CMTM6
1	CMTM8	2.0054E-54	-6.36	1.1666E-49	2.4494E-53	Q8IZV2	CMTM8
1	CNBD1	1.2563E-55	-9.52	7.3086E-51	1.5514E-54		
1	CNDP2	4.355E-148	1.81	2.533E-143	9.334E-147	Q96KP4	CNDP2
1	CNFN	0.00138135	-1.87	1	0.00531543	Q9BYD5	CNFN
1	CNGB1	4.1309E-23	-3.40	2.4031E-18	3.3418E-22	Q14028	CNGB1
1	CNGB3	0.00390798	-1.61	1	0.01438422	Q9NQW8	CNGB3
1	CNIH3	2.3769E-11	-1.78	1.3827E-06	1.4179E-10	Q8TBE1	CNIH3
1	CNKSR1	1.025E-277	-11.81	5.96E-273	2.856E-276	Q969H4	CNKSR1
1	CNKSR2	0.00118829	2.83	1	0.00459901	Q8WXI2	CNKSR2
1	CNKSR3	6.091E-200	-6.92	3.543E-195	1.536E-198	Q6P9H4	CNKSR3

1	CNN2P2	0.00327014	-2.64	1	0.01212475		
1	CNN2P3	0.00435057	-7.22	1	0.01593666		
1	CNN3	1.3628E-45	3.90	7.9278E-41	1.5281E-44	Q15417	CNN3
1	CNNM4	0	-1.60	0	0	Q6P4Q7	CNNM4
1	CNOT1	0	-1.71	0	0	A5YKK6	CNOT1
1	CNOT6	4.4861E-33	1.56	2.6098E-28	4.3179E-32	Q9ULM6	CNOT6
1	CNOT8	5.3023E-38	1.51	3.0846E-33	5.4182E-37	Q9UFF9	CNOT8
1	CNPPD1	2.2453E-59	1.77	1.3062E-54	2.8482E-58	Q9BV87	CNPPD1
1	CNRIP1	5.357E-300	83.54	3.116E-295	1.504E-298	Q96F85	CNRIP1
1	CNTN1	0	-12.29	0	0	Q12860	CNTN1
1	CNTN5	8.9108E-10	-2.31	5.1838E-05	4.9744E-09	O94779	CNTN5
1	CNTNAP1	5.168E-53	2.40	3.0064E-48	6.2284E-52	P78357	CNTNAP1
1	CNTNAP2	1.8031E-05	1.70	1	8.0084E-05	Q9UHC6	CNTNAP2
1	CNTNAP3	4.649E-243	23.25	2.705E-238	1.264E-241	Q9BZ76	CNTNAP3
1	CNTNAP3B	0	143.13	0	0	Q96NU0	CNTNAP3B
1	CNTNAP3P2	6.397E-162	138.99	3.722E-157	1.428E-160		
1	CNTNAP4	2.953E-192	-11.35	1.718E-187	7.252E-191	Q9C0A0	CNTNAP4
1	CNTRL	0	-1.97	0	0	Q7Z7A1	CNTRL
1	COA7	1.752E-126	2.40	1.019E-121	3.49E-125	Q96BR5	COA7
1	COASY	0	-1.59	0	0	Q13057	COASY
1	COBLL1	9.9003E-67	-2.65	5.7594E-62	1.3261E-65	Q53SF7	COBLL1
1	COCH	1.3963E-34	2.08	8.1228E-30	1.3723E-33	O43405	COCH
1	COG1	5.9056E-67	1.66	3.4355E-62	7.9269E-66	Q8WTW3	COG1
1	COG4	0	-1.96	0	0	Q9H9E3	COG4
1	COG5	0	-1.53	0	0	Q9UP83	COG5
1	COG6	0	-1.57	0	0	Q9Y2V7	COG6
1	COL11A1	1.7179E-07	-5.37	0.00999398	8.5911E-07	P12107	COL11A1
1	COL12A1	7.7644E-72	5.04	4.5169E-67	1.0793E-70	Q99715	COL12A1
1	COL13A1	2.059E-226	36.05	1.198E-221	5.502E-225	Q5TAT6	COL13A1
1	COL14A1	4.1616E-07	-4.69	0.02420988	2.0399E-06	Q05707	COL14A1
1	COL16A1	1.869E-296	-10.85	1.087E-291	5.244E-295	Q07092	COL16A1
1	COL17A1	0	-11.63	0	0	Q9UMD9	COL17A1
1	COL18A1	2.806E-75	3.43	1.6324E-70	4.0068E-74	P39060	COL18A1
1	COL19A1	1.4194E-06	-3.65	0.08257178	6.7565E-06	Q14993	COL19A1
1	COL1A1	4.984E-127	-9.06	2.899E-122	9.949E-126	P02452	COL1A1
1	COL1A2	2.6419E-22	-7.78	1.5369E-17	2.103E-21	P08123	COL1A2
1	COL20A1	0.01065736	2.87	1	0.03727641	Q9P218	COL20A1
1	COL21A1	0.00387266	-2.72	1	0.01425783	Q96P44	COL21A1
1	COL24A1	2.1103E-25	-7.93	1.2277E-20	1.7847E-24	Q17RW2	COL24A1
1	COL28A1	1.0017E-05	-4.82	0.58275162	4.5241E-05	Q2UY09	COL28A1
1	COL4A1	0	176.15	0	0	P02462	COL4A1
1	COL4A2	0	268.88	0	0	P08572	COL4A2
1	COL4A2-AS1	4.4648E-07	55.46	0.02597337	2.1843E-06		
1	COL4A4	1.8226E-24	-5.26	1.0603E-19	1.5147E-23	P53420	COL4A4
1	COL4A5	0	-2.56	0	0	P29400	COL4A5
1	COL4A6	0	-3.05	0	0	Q14031	COL4A6
1	COL5A1	0.00173793	3.00	1	0.00661622	P20908	COL5A1

1	COL5A2	9.036E-76	5.04	5.2566E-71	1.2941E-74	P05997	COL5A2
1	COL6A1	7.4554E-89	10.94	4.3371E-84	1.1873E-87	P12109	COL6A1
1	COL6A2	2.703E-171	8.12	1.573E-166	6.208E-170	P12110	COL6A2
1	COL6A4P1	2.2737E-11	-1.91	1.3227E-06	1.3569E-10		
1	COL6A6	0.00511829	-1.96	1	0.01859899	A6NMZ7	COL6A6
1	COL7A1	0	-6.37	0	0	Q02388	COL7A1
1	COL8A1	1.721E-23	7.36	1.0012E-18	1.4024E-22	P27658	COL8A1
1	COL8A2	3.8058E-22	-6.74	2.214E-17	3.0192E-21	P25067	COL8A2
1	COL9A2	2.6517E-36	-6.60	1.5426E-31	2.6583E-35	Q14055	COL9A2
1	COL9A3	6.336E-40	-3.44	3.6859E-35	6.6163E-39	Q14050	COL9A3
1	COLCA1	5.3809E-22	-50.20	3.1303E-17	4.2612E-21	Q6ZS62	COLCA1
1	COLCA2	2.6129E-16	-39.91	1.52E-11	1.8413E-15	A8K830	COLCA2
1	COLEC11	9.9991E-29	-14.32	5.8169E-24	9.01E-28	Q9BWP8	COLEC11
1	COLGALT2	0.00047048	-3.07	1	0.00188406	Q8IYK4	COLGALT2
1	COLQ	1.1987E-06	1.92	0.06973196	5.7317E-06	Q9Y215	COLQ
1	COMMD1	9.066E-39	1.92	5.2741E-34	9.3445E-38	Q8N668	COMMD1
1	COMMD10	6.6953E-15	1.65	3.8949E-10	4.5033E-14	Q9Y6G5	COMMD10
1	COMMD3-BM	2.2276E-05	1.59	1	9.8324E-05	R4GMX3	COMMD3-BM
1	COMMD4	1.545E-87	2.11	8.9881E-83	2.4325E-86	Q9H0A8	COMMD4
1	COMMD7	7.0571E-83	8.88	4.1054E-78	1.0719E-81	Q86VX2	COMMD7
1	COMMD9	1.4341E-88	2.80	8.3426E-84	2.2775E-87	Q9P000	COMMD9
1	COPB1	2.2806E-51	1.85	1.3267E-46	2.702E-50	P53618	COPB1
1	COPE	1.127E-53	2.88	6.5563E-49	1.3688E-52	O14579	COPE
1	COPRS	2.1096E-18	1.56	1.2273E-13	1.5565E-17	Q9NQ92	COPRS
1	COPS3	4.2266E-49	1.91	2.4588E-44	4.9058E-48	Q9UNS2	COPS3
1	COPS8	2.4434E-25	1.57	1.4214E-20	2.0639E-24	Q99627	COPS8
1	COPS9	7.9676E-65	2.29	4.6351E-60	1.0544E-63	Q8WXC6	COPS9
1	COPZ2	2.5895E-12	-1.93	1.5064E-07	1.6024E-11	Q9P299	COPZ2
1	COQ10A	6.5178E-21	1.73	3.7916E-16	5.0555E-20	Q96MF6	COQ10A
1	COQ8B	0	-1.89	0	0	Q96D53	COQ8B
1	CORO1A	1.104E-28	2.11	6.4224E-24	9.9402E-28	P31146	CORO1A
1	CORO1B	0	-1.52	0	0	Q9BR76	CORO1B
1	CORO2A	0	-3.08	0	0	Q92828	CORO2A
1	CORO2B	1.5542E-40	13.94	9.0412E-36	1.6358E-39	Q9UQ03	CORO2B
1	CORO6	5.909E-281	-18.87	3.437E-276	1.649E-279	Q6QEF8	CORO6
1	CORO7-PAM1	5.38E-07	7.25	0.0312978	2.6213E-06		
1	COX16	2.6307E-23	1.90	1.5304E-18	2.1365E-22	Q9P0S2	COX16
1	COX17	0	-1.64	0	0	Q14061	COX17
1	COX18	1.5556E-21	-1.63	9.0496E-17	1.2223E-20	Q8N8Q8	COX18
1	COX19	0	-2.40	0	0	Q49B96	COX19
1	COX6B2	7.2681E-62	-10.88	4.2281E-57	9.3958E-61	Q6YFQ2	COX6B2
1	COX6C	0	-1.79	0	0	P09669	COX6C
1	COX7A2	4.1633E-13	-1.58	2.422E-08	2.6449E-12	P14406	COX7A2
1	COX8A	0	-2.15	0	0	P10176	COX8A
1	CP	0.01199704	-1.90	1	0.04171131	P00450	CP
1	CPA2	0.00079745	2.66	1	0.00313324	P48052	CPA2
1	CPA4	4.5268E-75	8.22	2.6334E-70	6.4497E-74	Q9UI42	CPA4

1	CPA6	4.1141E-09	-4.78	0.00023933	2.2297E-08	Q8N4T0	CPA6
1	CPAMD8	4.6571E-06	-2.87	0.27092253	2.1447E-05	Q8IZJ3	CPAMD8
1	CPB2	0.00059109	-3.37	1	0.00234783	Q96IY4	CPB2
1	CPE	4.265E-233	-14.15	2.481E-228	1.147E-231	P16870	CPE
1	CPEB2	8.9355E-89	3.82	5.1981E-84	1.4218E-87	Q7Z5Q1	CPEB2
1	CPEB4	4.009E-158	10.57	2.332E-153	8.861E-157	Q17RY0	CPEB4
1	CPED1	2.969E-123	-4.13	1.727E-118	5.839E-122	A4D0V7	CPED1
1	CPHL1P	1.2061E-12	-7.37	7.0166E-08	7.5423E-12		
1	CPLX1	0.01223735	-1.65	1	0.04248855	O14810	CPLX1
1	CPM	0.00034768	-1.86	1	0.00140713	P14384	CPM
1	CPNE2	0	-1.57	0	0	Q96FN4	CPNE2
1	CPNE5	2.1339E-25	-8.12	1.2414E-20	1.8043E-24	Q9HCH3	CPNE5
1	CPNE7	8.495E-126	131.29	4.942E-121	1.686E-124	Q9UBL6	CPNE7
1	CPNE8	0	-2.38	0	0	Q86YQ8	CPNE8
1	CPO	0.00095458	8.01	1	0.00372495	Q8IVL8	CPO
1	CPQ	0.00722606	1.53	1	0.02580375	Q9Y646	CPQ
1	CPS1	3.2857E-61	2.01	1.9114E-56	4.2232E-60	P31327	CPS1
1	CPSF1	0	-1.61	0	0	Q10570	CPSF1
1	CPSF1P1	5.681E-09	23.49	0.00033049	3.0603E-08		
1	CPSF7	0	-1.95	0	0	Q8N684	CPSF7
1	CPT1A	0	-2.23	0	0	P50416	CPT1A
1	CPT1C	2.7025E-70	-6.73	1.5721E-65	3.7149E-69	Q8TCG5	CPT1C
1	CPT2	0	-2.36	0	0	P23786	CPT2
1	CPTP	2.1473E-36	1.79	1.2492E-31	2.1545E-35	Q5TA50	CPTP
1	CPXM2	0.01041133	-7.09	1	0.03645971	Q8N436	CPXM2
1	CR1	2.2377E-07	-9.95	0.01301788	1.1125E-06	P17927	CR1
1	CR1L	2.3736E-07	-4.85	0.01380833	1.1782E-06	Q2VPA4	CR1L
1	CR2	9.239E-240	-33.47	5.375E-235	2.503E-238	P20023	CR2
1	CRABP2	1.556E-212	-10.02	9.052E-208	4.043E-211	P29373	CRABP2
1	CRACR2A	4.842E-115	3.29	2.817E-110	9.123E-114	Q9BSW2	CRACR2A
1	CRACR2B	2.5823E-24	-3.45	1.5022E-19	2.1375E-23	Q8N4Y2	CRACR2B
1	CRADD	4.6068E-11	1.60	2.68E-06	2.7117E-10	P78560	CRADD
1	CRAT	4.3428E-38	1.57	2.5264E-33	4.4408E-37	P43155	CRAT
1	CRB1	2.3506E-24	-9.25	1.3674E-19	1.949E-23	P82279	CRB1
1	CRB2	0.00340217	-4.79	1	0.01260014	Q5IJ48	CRB2
1	CRB3	2.26E-174	-9.82	1.314E-169	5.241E-173	Q9BUF7	CRB3
1	CREB3	1.3229E-49	1.77	7.6961E-45	1.5429E-48	O43889	CREB3
1	CREB3L2	3.6601E-78	5.47	2.1292E-73	5.3391E-77	Q70SY1	CREB3L2
1	CREB3L4	0	-2.31	0	0	Q8TEY5	CREB3L4
1	CREB5	4.006E-179	27.34	2.331E-174	9.432E-178	Q02930	CREB5
1	CREBBP	1.9984E-19	1.51	1.1625E-14	1.5057E-18	Q92793	CREBBP
1	CREBL2	2.1308E-68	2.63	1.2396E-63	2.8841E-67	O60519	CREBL2
1	CREBRF	3.4218E-09	-1.55	0.00019906	1.8623E-08	Q8IUR6	CREBRF
1	CREBZF	0	-2.08	0	0	Q9NS37	CREBZF
1	CREG1	0	-5.04	0	0	O75629	CREG1
1	CREG2	2.5641E-19	-2.03	1.4916E-14	1.9279E-18	Q8IUH2	CREG2
1	CREM	1.1467E-72	6.81	6.6707E-68	1.6024E-71	Q03060	CREM

1	CRHR2	3.0487E-06	-2.23	0.1773552	1.4208E-05	Q13324	CRHR2
1	CRIM1	7.1159E-95	2.41	4.1396E-90	1.1824E-93	Q9NZV1	CRIM1
1	CRIP2	8.1483E-62	4.55	4.7402E-57	1.0529E-60	P52943	CRIP2
1	CRIP3	2.9202E-14	-13.41	1.6988E-09	1.9269E-13	Q6Q6R5	CRIP3
1	CRIPAK	1.3613E-35	-3.13	7.919E-31	1.353E-34	Q8N1N5	CRIPAK
1	CRISP2	0.00793762	-8.76	1	0.02821995	P16562	CRISP2
1	CRISP3	4.4151E-27	-48.94	2.5684E-22	3.8495E-26	P54108	CRISP3
1	CRISPLD1	1.015E-189	-12.49	5.903E-185	2.467E-188	Q9H336	CRISPLD1
1	CRISPLD2	8.8513E-33	-5.54	5.1491E-28	8.4857E-32	Q9H0B8	CRISPLD2
1	CRLF1	7.3119E-14	2.04	4.2536E-09	4.7649E-13	O75462	CRLF1
1	CRLS1	1.748E-34	1.60	1.0169E-29	1.7168E-33	Q9UJA2	CRLS1
1	CRMP1	2.4018E-96	3.53	1.3972E-91	4.0289E-95	Q14194	CRMP1
1	CROCC	2.8277E-27	1.65	1.645E-22	2.4763E-26	Q5TZA2	CROCC
1	CROCCP3	2.1397E-09	1.63	0.00012448	1.1747E-08	Q8IVE0	CROCCP3
1	CRTC1	7.8067E-75	2.47	4.5415E-70	1.1098E-73	Q6UUV9	CRTC1
1	CRTC3-AS1	1.6669E-16	-3.37	9.6968E-12	1.1823E-15		
1	CRX	0.0061124	-5.63	1	0.02199982	O43186	CRX
1	CRY1	1.2005E-98	3.09	6.984E-94	2.0487E-97	Q16526	CRY1
1	CRYAB	0.00045208	-3.67	1	0.00181298	P02511	CRYAB
1	CRYBB2	2.046E-05	-4.65	1	9.0532E-05	P43320	CRYBB2
1	CRYBB3	5.048E-46	-5.95	2.9366E-41	5.6845E-45	P26998	CRYBB3
1	CRYGS	1.748E-07	1.59	0.0101687	8.7375E-07	P22914	CRYGS
1	CRYM-AS1	0.00013517	1.80	1	0.00056471	A6NIL9	CRYM-AS1
1	CSAD	2.6867E-14	-1.74	1.563E-09	1.7753E-13	Q9Y600	CSAD
1	CSAG1	1.098E-142	-9.44	6.389E-138	2.323E-141		
1	CSAG2	0.00942764	-17.91	1	0.03316063	Q9Y5P2	CSAG2
1	CSAG3	7.597E-247	-11.49	4.419E-242	2.072E-245		
1	CSAG4	2.2182E-09	-4.74	0.00012904	1.2174E-08		
1	CSDE1	5.7089E-39	1.60	3.3211E-34	5.9063E-38	O75534	CSDE1
1	CSE1L	1.1513E-44	1.82	6.6974E-40	1.275E-43	P55060	CSE1L
1	CSF1	2.467E-107	-4.97	1.435E-102	4.437E-106	P09603	CSF1
1	CSF1R	9.1489E-09	-2.86	0.00053223	4.8837E-08	P07333	CSF1R
1	CSF2	1.7933E-14	-6.59	1.0432E-09	1.1913E-13	P04141	CSF2
1	CSF3	6.843E-146	40.12	3.981E-141	1.459E-144	P09919	CSF3
1	CSGALNACT1	9.5205E-23	-2.78	5.5384E-18	7.6582E-22	Q8TDX6	CSGALNACT1
1	CSGALNACT2	9.5848E-47	4.08	5.5758E-42	1.0871E-45	Q8N6G5	CSGALNACT2
1	CSMD1	2.8934E-14	-4.52	1.6832E-09	1.9097E-13	Q96PZ7	CSMD1
1	CSMD3	7.5956E-83	-8.39	4.4187E-78	1.1534E-81	Q7Z407	CSMD3
1	CSNK2A3	0.00120383	3.42	1	0.00465727	Q8NEV1	CSNK2A3
1	CSNK2B	4.4113E-77	1.85	2.5662E-72	6.3821E-76	P67870	CSNK2B
1	CSPG4	2.315E-163	-9.60	1.347E-158	5.189E-162	Q6UVK1	CSPG4
1	CSPG4P11	4.3886E-06	2.22	0.25529964	2.0243E-05		
1	CSPG5	4.2246E-05	-1.80	1	0.00018303	O95196	CSPG5
1	CSRNP1	1.2861E-28	2.49	7.4817E-24	1.156E-27	Q96S65	CSRNP1
1	CSRNP3	3.1049E-31	-3.38	1.8063E-26	2.9091E-30	Q8WYN3	CSRNP3
1	CSRP1	1.0054E-33	1.55	5.8488E-29	9.774E-33	P21291	CSRP1
1	CST3	0	-2.65	0	0	P01034	CST3

1	CST6	2.086E-186	-20.59	1.213E-181	5.011E-185	Q15828	CST6
1	CST9LP2	5.5789E-10	-9.34	3.2454E-05	3.1412E-09		
1	CSTA	2.191E-174	-15.66	1.275E-169	5.085E-173	P01040	CSTA
1	CSTF1	3.4257E-22	1.54	1.9929E-17	2.7214E-21	Q05048	CSTF1
1	CSTF2T	3.5461E-42	1.62	2.0629E-37	3.8223E-41	Q9H0L4	CSTF2T
1	CSTF3-AS1	2.43E-06	-1.80	0.1413609	1.1402E-05		
1	CSTL1	0.00368502	-5.61	1	0.01358594	Q9H114	CSTL1
1	CT83	2.9657E-56	-9.04	1.7253E-51	3.6763E-55	Q5H943	CT83
1	CTA-126B4.7	1.4994E-06	-5.84	0.08722691	7.1275E-06		
1	CTA-14H9.5	3.0272E-11	-11.87	1.7611E-06	1.7974E-10		
1	CTA-221G9.7	0.01214249	-3.21	1	0.0421869		
1	CTA-223H9.9	0.00170016	-7.08	1	0.00647964		
1	CTA-246H3.8	0.00242297	-3.03	1	0.0090891		
1	CTA-292E10.6	1.4082E-05	-1.81	0.8192269	6.3008E-05		
1	CTA-293F17.1	1.4035E-11	-5.28	8.1646E-07	8.4554E-11		
1	CTA-363E6.2	0.00441874	-8.22	1	0.016166		
1	CTA-373H7.7	0.00082173	11.22	1	0.00322407		
1	CTA-384D8.31	6.9824E-08	-22.19	0.00406193	3.5634E-07		
1	CTA-384D8.35	2.5022E-09	-29.83	0.00014556	1.371E-08		
1	CTA-392C11.1	2.3913E-06	-42.14	0.13911124	1.1227E-05		
1	CTA-392C11.2	7.6521E-90	-12.77	4.4515E-85	1.227E-88		
1	CTA-392E5.1	0.0060263	-3.86	1	0.0217141		
1	CTA-445C9.14	1.2178E-12	-1.68	7.0843E-08	7.6126E-12		
1	CTA-941F9.10	0.00220719	-1.97	1	0.00831774		
1	CTA-963H5.5	0.00301527	2.38	1	0.0112162		
1	CTAGE15	3.0885E-14	-3.76	1.7967E-09	2.0362E-13	A4D2H0	CTAGE15
1	CTAGE16P	0.00022104	-6.09	1	0.00090956		
1	CTAGE4	1.1022E-48	-4.00	6.4121E-44	1.2745E-47	Q8IX94	CTAGE4
1	CTB-109A12.1	4.6894E-05	39.99	1	0.00020266		
1	CTB-111H14.1	1.0841E-09	-2.79	6.3065E-05	6.0297E-09		
1	CTB-113P19.1	0.00014383	-3.94	1	0.00059995		
1	CTB-113P19.4	9.2919E-60	-9.39	5.4055E-55	1.1828E-58		
1	CTB-118P15.2	0.00015722	3.53	1	0.00065371		
1	CTB-129O4.1	3.6211E-32	2.56	2.1065E-27	3.4381E-31		
1	CTB-131B5.5	1.3696E-18	2.85	7.9675E-14	1.015E-17		
1	CTB-133G6.1	1.0408E-15	-4.95	6.0547E-11	7.1841E-15		
1	CTB-133P21.1	0.00101687	-4.53	1	0.00396165		
1	CTB-147C22.9	0.00014196	-10.74	1	0.00059247		
1	CTB-171A8.1	1.2082E-09	-2.41	7.0284E-05	6.7033E-09		
1	CTB-179K24.3	1.5126E-05	-1.89	0.87992854	6.7521E-05		
1	CTB-25B13.5	0.00109227	2.14	1	0.00424006		
1	CTB-25B13.9	0.00224985	3.99	1	0.00846535		
1	CTB-31N19.2	6.3006E-07	2.47	0.03665315	3.0583E-06		
1	CTB-31N19.5	0.00397572	6.36	1	0.0146271		
1	CTB-31O20.6	0.00536961	3.42	1	0.01946605		
1	CTB-32O4.2	1.405E-26	-6.25	8.1735E-22	1.2123E-25		
1	CTB-32O4.3	6.192E-57	-5.77	3.6022E-52	7.7084E-56		

1	CTB-32O4.4	3.8015E-31	-6.42	2.2115E-26	3.5578E-30		
1	CTB-35F21.3	0.00107503	-25.27	1	0.00417592		
1	CTB-39G8.3	4.0574E-05	-2.64	1	0.00017604		
1	CTB-47B11.3	0.00010058	32.15	1	0.00042461		
1	CTB-50L17.16	0.00332586	-3.14	1	0.01232424		
1	CTB-50L17.7	0.00022967	-6.08	1	0.0009437		
1	CTB-52I2.4	3.9418E-09	-3.22	0.00022931	2.1387E-08		
1	CTB-54O9.9	3.9933E-08	3.08	0.00232304	2.0649E-07		
1	CTB-58E17.1	7.4984E-07	-2.01	0.04362134	3.6254E-06		
1	CTB-58E17.5	0.00034446	-2.03	1	0.00139438		
1	CTB-5E10.3	0.00238307	3.65	1	0.00894693		
1	CTB-60B18.10	1.5053E-07	-3.37	0.00875667	7.5573E-07		
1	CTB-60B18.22	1.5479E-06	-8.26	0.09004845	7.3539E-06		
1	CTB-60B18.23	2.5439E-23	-7.50	1.4799E-18	2.0671E-22		
1	CTB-60B18.6	0.00054205	-4.39	1	0.00216071		
1	CTB-73N10.1	0.00588597	-3.27	1	0.02123081		
1	CTB-88F18.5	2.0654E-17	-7.09	1.2015E-12	1.4944E-16		
1	CTB-89H12.4	4.4224E-10	-1.54	2.5727E-05	2.5002E-09		
1	CTB-91J4.1	1.9071E-05	-2.70	1	8.453E-05		
1	CTB-92J24.3	5.6717E-32	15.84	3.2994E-27	5.3693E-31		
1	CTB-96E2.3	2.7736E-06	7.92	0.16134928	1.2968E-05		
1	CTBP1-AS2	7.1877E-39	1.77	4.1814E-34	7.4256E-38		
1	CTBP2P1	7.4525E-06	-5.94	0.43354201	3.3931E-05		
1	CTC-1337H24	0.00139182	-3.97	1	0.00535358		
1	CTC-203F4.2	0.00073881	-2.48	1	0.00291053		
1	CTC-241F20.4	1.0618E-32	-13.25	6.1772E-28	1.017E-31		
1	CTC-241N9.1	0.00045774	1.61	1	0.00183442		
1	CTC-260E6.6	1.052E-99	40.61	6.1191E-95	1.8051E-98		
1	CTC-260F20.3	0.00275728	1.71	1	0.01029206		
1	CTC-261N6.1	1.047E-63	-16.44	6.0909E-59	1.3734E-62		
1	CTC-281F24.5	1.1834E-09	2.59	6.8844E-05	6.5697E-09		
1	CTC-295J13.3	1.828E-05	-5.91	1	8.1175E-05		
1	CTC-296K1.3	1.1589E-14	12.11	6.7417E-10	7.7419E-14		
1	CTC-296K1.4	2.004E-20	9.95	1.1658E-15	1.5397E-19		
1	CTC-301O7.4	1.0265E-11	2.00	5.9717E-07	6.214E-11		
1	CTC-303L1.2	0.00541425	3.39	1	0.01961933		
1	CTC-308K20.1	3.5695E-16	4.01	2.0765E-11	2.5063E-15		
1	CTC-325H20.4	4.202E-139	7.11	2.444E-134	8.774E-138		
1	CTC-325H20.8	6.262E-06	16.26	0.36428651	2.8661E-05		
1	CTC-325J23.2	0.0006832	-3.74	1	0.00269747		
1	CTC-326K19.6	0.00288889	6.25	1	0.0107626		
1	CTC-338M12.[illegible]	2.7169E-05	-4.21	1	0.00011928		
1	CTC-338M12.[illegible]	6.1019E-08	-8.62	0.00354974	3.1234E-07		
1	CTC-338M12.[illegible]	1.9177E-33	-8.35	1.1156E-28	1.8547E-32		
1	CTC-339O9.1	2.1489E-08	25.65	0.00125011	1.1275E-07		
1	CTC-347C20.1	0.0046889	-3.55	1	0.01711242		
1	CTC-360G5.8	5.0138E-17	14.09	2.9167E-12	3.5947E-16		

1	CTC-360J11.4	8.0199E-05	35.92	1	0.00034089		
1	CTC-441N14.4	0.00078844	-2.51	1	0.00309912		
1	CTC-444N24.6	2.7158E-12	-2.24	1.5799E-07	1.6793E-11		
1	CTC-454I21.3	0.00707379	3.60	1	0.02528016		
1	CTC-455F18.3	0.00140517	27.68	1	0.00539816		
1	CTC-457E21.9	4.4684E-91	60.30	2.5995E-86	7.2308E-90		
1	CTC-463A16.1	0.00270328	3.59	1	0.01009634		
1	CTC-465D4.1	6.9851E-06	-55.15	0.40635044	3.1871E-05		
1	CTC-467M3.1	0.00063939	-25.31	1	0.0025331		
1	CTC-471F3.5	9.9525E-06	-2.67	0.57897423	4.4955E-05		
1	CTC-471J1.8	0.00495861	2.28	1	0.01804354		
1	CTC-479C5.10	1.879E-05	-6.95	1	8.3339E-05		
1	CTC-487M23.8	7.3915E-06	3.29	0.42999186	3.3669E-05		
1	CTC-490E21.1	1.3025E-11	-2.03	7.577E-07	7.8567E-11		
1	CTC-490E21.1	0.00026716	-4.42	1	0.00109019		
1	CTC-492K19.4	0.00654916	2.83	1	0.0234846		
1	CTC-497E21.3	6.5997E-30	-16.69	3.8393E-25	6.0614E-29		
1	CTC-497E21.5	1.6727E-15	-7.02	9.7309E-11	1.1481E-14		
1	CTC-498J12.3	0.00664729	2.15	1	0.02382328		
1	CTC-499B15.5	1.8332E-19	1.98	1.0665E-14	1.3827E-18		
1	CTC-499J9.1	2.0825E-47	-16.55	1.2115E-42	2.3773E-46		
1	CTC-510F12.2	0.00160914	-3.14	1	0.00614643		
1	CTC-512J12.6	0.00077215	8.99	1	0.00303816		
1	CTC-513N18.7	9.6727E-15	73.16	5.627E-10	6.4782E-14		
1	CTC-523E23.1	1.3537E-05	38.34	0.78747918	6.0613E-05		
1	CTC-523E23.3	1.8432E-07	8.96	0.01072259	9.2E-07		
1	CTC-523E23.5	1.3213E-06	18.43	0.07686251	6.3012E-06		
1	CTC-529P8.1	4.4832E-09	-4.39	0.00026081	2.4266E-08		
1	CTC-534A2.2	4.3129E-26	2.98	2.509E-21	3.6908E-25		
1	CTC-535M15.1	1.0521E-14	-5.07	6.1202E-10	7.0364E-14		
1	CTC-543D15.3	0.00052735	8.04	1	0.00210311		
1	CTC-550B14.6	8.2202E-05	-8.73	1	0.00034903		
1	CTC-550B14.7	3.0211E-07	-2.29	0.01757479	1.4907E-06		
1	CTC-551A13.1	0.00143309	-7.56	1	0.00549888		
1	CTC-551A13.2	5.2441E-10	-7.69	3.0507E-05	2.9564E-09		
1	CTC-559E9.12	0.00263365	9.77	1	0.00984703		
1	CTC-559E9.4	0.00090267	29.34	1	0.00352857		
1	CTC-559E9.5	0.00093626	2.59	1	0.00365569		
1	CTC-559E9.6	6.6367E-19	4.12	3.8608E-14	4.9542E-18		
1	CTC-559E9.8	7.2834E-51	134.82	4.237E-46	8.584E-50		
1	CTC-806A22.1	0.00389914	-1.79	1	0.01435259		
1	CTD-2003C8.2	1.539E-14	-5.35	8.9528E-10	1.024E-13		
1	CTD-2005H7.1	5.7652E-13	-5.14	3.3538E-08	3.6471E-12		
1	CTD-2005H7.2	2.0258E-31	-5.19	1.1785E-26	1.9026E-30		
1	CTD-2006C1.2	1.6482E-21	2.32	9.5884E-17	1.2942E-20		
1	CTD-2007L18.	1.1986E-06	-3.20	0.06972641	5.7317E-06		
1	CTD-2008L17.	1.0717E-07	-7.44	0.00623473	5.4163E-07		

1	CTD-2008L17.	1.0625E-19	-7.10	6.1812E-15	8.061E-19		
1	CTD-2008P7.9	0.00214989	-19.72	1	0.00811024		
1	CTD-2012J19.	2.9812E-11	-3.65	1.7343E-06	1.7706E-10		
1	CTD-2012K14.	0.00407258	-1.55	1	0.0149702		
1	CTD-2012K14.	0.01249918	-2.22	1	0.04335106		
1	CTD-2013N24	1.0547E-05	-1.60	0.61357613	4.7568E-05		
1	CTD-2017D11	4.9865E-08	-2.03	0.00290084	2.5657E-07		
1	CTD-2020K17.	6.7359E-08	-2.20	0.00391853	3.4406E-07		
1	CTD-2020K17.	6.5091E-19	-4.82	3.7866E-14	4.8603E-18		
1	CTD-2020K17.	1.9285E-08	-5.75	0.0011219	1.0147E-07		
1	CTD-2021H9.1	2.6475E-05	-16.53	1	0.00011632		
1	CTD-2021H9.2	0.0117853	-15.94	1	0.0410341		
1	CTD-2021H9.3	9.3848E-72	-16.49	5.4595E-67	1.3033E-70		
1	CTD-2021J15.	2.962E-115	6.54	1.723E-110	5.586E-114		
1	CTD-2022H16	0.0035383	-21.85	1	0.01307236		
1	CTD-2027I19.1	0.00619126	6.34	1	0.02227538		
1	CTD-2033A16	0.00573247	-21.40	1	0.02070536		
1	CTD-2033A16	1.3699E-34	-3.03	7.969E-30	1.3468E-33		
1	CTD-2033C11	0.0119043	-2.01	1	0.04142774		
1	CTD-2034I4.2	1.6596E-21	-875.06	9.6547E-17	1.3029E-20		
1	CTD-2035E11.	0.00024032	-32.61	1	0.00098557		
1	CTD-2066L21.	7.7495E-18	-2.65	4.5082E-13	5.6564E-17		
1	CTD-2083E4.4	1.5458E-05	-1.85	0.89926988	6.8962E-05		
1	CTD-2083E4.5	0.00494989	-3.01	1	0.01801295		
1	CTD-2083E4.6	2.8864E-05	-4.51	1	0.00012646		
1	CTD-2095E4.5	4.965E-10	-3.61	2.8883E-05	2.8007E-09		
1	CTD-2104P17.	7.1759E-14	-8.31	4.1745E-09	4.6778E-13		
1	CTD-2105E13.	1.4818E-11	-14.85	8.6205E-07	8.9193E-11		
1	CTD-2116N17	1.0377E-15	1.54	6.0365E-11	7.1633E-15		
1	CTD-2130O13	2.0183E-30	23.55	1.1741E-25	1.8678E-29		
1	CTD-2132N18	2.9372E-27	-3.41	1.7087E-22	2.5718E-26		
1	CTD-2132N18	0.00914059	3.84	1	0.03224648		
1	CTD-2134A5.3	0.0035383	-22.37	1	0.01307236		
1	CTD-2134A5.4	1.6948E-53	-9.78	9.8594E-49	2.0536E-52		
1	CTD-2135J3.3	4.8676E-09	-10.69	0.00028317	2.6297E-08		
1	CTD-2139B15	0.00066065	-25.33	1	0.00261269		
1	CTD-2152M20	6.2536E-09	-3.27	0.00036379	3.3604E-08		
1	CTD-2162K18.	0.00123702	5.81	1	0.0047806		
1	CTD-2165H16	1.6429E-19	-3.61	9.5573E-15	1.2412E-18		
1	CTD-2175A23	0.01075632	-1.86	1	0.03760672		
1	CTD-2184C24.	9.16E-05	-13.02	1	0.0003878		
1	CTD-2184D3.5	3.7556E-08	-3.50	0.00218475	1.9453E-07		
1	CTD-2186M15	8.0692E-08	-2.26	0.00469415	4.1044E-07		
1	CTD-2192J16.	0.00405068	2.75	1	0.0148925		
1	CTD-2192J16.	0.00434797	1.64	1	0.01593015		
1	CTD-2199O4.3	0.00579002	-2.43	1	0.02090286		
1	CTD-2201E18.	1.8554E-12	-1.51	1.0794E-07	1.1537E-11		

1	CTD-2203K17.	1.836E-108	-3.02	1.068E-103	3.328E-107		
1	CTD-2206N4.4	5.6949E-39	-8.73	3.313E-34	5.8928E-38		
1	CTD-2207O23	0.00058679	31.44	1	0.0023317		
1	CTD-2207P18.	1.451E-160	13.26	8.439E-156	3.23E-159		
1	CTD-2245F17.	2.605E-167	143.19	1.515E-162	5.917E-166		
1	CTD-2256P15.	6.1859E-14	-2.59	3.5986E-09	4.0397E-13		
1	CTD-2260A17	9.4532E-12	-2.07	5.4993E-07	5.7296E-11		
1	CTD-2263F21.	2.2984E-05	15.52	1	0.00010138		
1	CTD-2267D19	2.3916E-41	-3.43	1.3913E-36	2.5467E-40		
1	CTD-2269F5.1	1.237E-58	-4.58	7.1959E-54	1.5623E-57		
1	CTD-2270L9.4	2.9675E-11	3.49	1.7263E-06	1.763E-10		
1	CTD-2270P14.	6.3074E-10	-2.46	3.6692E-05	3.5441E-09		
1	CTD-2284J15.	8.7015E-05	-3.36	1	0.00036885		
1	CTD-2287O16	0.00143646	-1.65	1	0.00551036		
1	CTD-2288F12.	0.00079864	-1.79	1	0.00313751		
1	CTD-2288O8.1	0.00569854	-2.47	1	0.02061224		
1	CTD-2291D10	2.0174E-47	121.91	1.1736E-42	2.3044E-46		
1	CTD-2292P10.	1.3434E-21	-2.52	7.8148E-17	1.0561E-20		
1	CTD-2298J14.	9.9942E-41	-15.68	5.814E-36	1.0548E-39		
1	CTD-2302E22.	0.00022358	2.75	1	0.00091944		
1	CTD-2303H24	3.6329E-11	-6.16	2.1134E-06	2.1482E-10		
1	CTD-2306M5.	3.8277E-05	-45.48	1	0.0001664		
1	CTD-2307P3.1	1.8972E-13	-9.93	1.1037E-08	1.22E-12		
1	CTD-2311M21	0.00942764	-17.79	1	0.03316063		
1	CTD-2311M21	2.8516E-19	-141.20	1.6589E-14	2.1422E-18		
1	CTD-2311M21	0.00573247	-21.30	1	0.02070536		
1	CTD-2311M21	1.8241E-19	-54.34	1.0612E-14	1.376E-18		
1	CTD-2314G24	1.7159E-07	-12.18	0.00998231	8.5818E-07		
1	CTD-2315E11.	0.01219299	-5.87	1	0.04233704		
1	CTD-2316B1.1	0.00027872	-7.45	1	0.00113577		
1	CTD-2318O12	3.4968E-05	-3.33	1	0.00015248		
1	CTD-2319I12.1	5.8875E-07	-7.65	0.0342498	2.8627E-06		
1	CTD-2319I12.4	1.7152E-16	-2.36	9.9782E-12	1.2163E-15		
1	CTD-2325M2.	3.8379E-15	-3.18	2.2327E-10	2.6043E-14		
1	CTD-2325P2.3	0.0082236	3.02	1	0.02917783		
1	CTD-2330K9.3	1.0527E-06	-4.96	0.06124243	5.0472E-06		
1	CTD-2331H12	1.7755E-47	58.88	1.0329E-42	2.0288E-46		
1	CTD-2331H12	2.7078E-25	6.00	1.5752E-20	2.2853E-24		
1	CTD-2334D19	5.1366E-41	306.46	2.9881E-36	5.4389E-40		
1	CTD-2340E1.2	0.0003665	-5.75	1	0.00148032		
1	CTD-2341M24	1.1866E-20	-3.71	6.9029E-16	9.1563E-20		
1	CTD-2349B8.1	4.5445E-09	-2.52	0.00026437	2.4593E-08		
1	CTD-2350C19.	0.01249357	3.32	1	0.0433342		
1	CTD-2353F22.	2.7852E-08	-19.81	0.00162027	1.4533E-07		
1	CTD-2354A18	7.039E-138	24.08	4.095E-133	1.462E-136		
1	CTD-2368P22.	9.8103E-07	-4.84	0.05707063	4.7123E-06		
1	CTD-2371O3.3	9.691E-09	1.71	0.00056377	5.1674E-08		

1	CTD-2376I4.1	0.00787154	-5.62	1	0.02799728		
1	CTD-2383M3.	1.2407E-05	-4.32	0.72173734	5.5711E-05		
1	CTD-2384A14	2.2595E-36	-6.84	1.3144E-31	2.2662E-35		
1	CTD-2384B11	0.00268432	22.09	1	0.01002811		
1	CTD-2410N18	3.5504E-06	2.38	0.2065438	1.6471E-05		
1	CTD-2501E16.	5.9536E-06	-49.36	0.34634666	2.7282E-05		
1	CTD-2506P8.6	0.01180156	-1.53	1	0.04108581		
1	CTD-2509G16	0.00080956	26.01	1	0.00317912		
1	CTD-2509G16	8.039E-100	18.57	4.6767E-95	1.3804E-98		
1	CTD-2510F5.4	3.2443E-08	3.19	0.00188734	1.686E-07		
1	CTD-2510F5.6	2.6928E-14	1.60	1.5665E-09	1.7791E-13		
1	CTD-2514C3.1	2.9606E-57	544.69	1.7223E-52	3.6935E-56		
1	CTD-2515C13.	0.0035383	-23.17	1	0.01307236		
1	CTD-2515C13.	1.0485E-25	-20.77	6.0994E-21	8.9056E-25		
1	CTD-2516F10.	8.2051E-11	-4.78	4.7733E-06	4.7852E-10		
1	CTD-2517O10	6.951E-15	-3.98	4.0437E-10	4.6737E-14		
1	CTD-2521M24	2.3535E-19	133.88	1.3691E-14	1.7712E-18		
1	CTD-2523D13	5.238E-75	-10.58	3.0471E-70	7.4593E-74		
1	CTD-2524L6.3	0.00024138	2.61	1	0.00098964		
1	CTD-2527I21.	0.00019385	8.54	1	0.00080178		
1	CTD-2528L19.	4.1632E-07	5.62	0.02421884	2.0405E-06		
1	CTD-2528L19.	5.0625E-07	1.95	0.02945061	2.4705E-06		
1	CTD-2529P6.3	0.00897135	3.33	1	0.03167443		
1	CTD-2530N21	2.1596E-08	-1.62	0.00125633	1.1331E-07		
1	CTD-2531D15	8.563E-12	-23.13	4.9815E-07	5.1939E-11		
1	CTD-2535I10.	0.00221969	-1.90	1	0.00835746		
1	CTD-2537I9.1	0.00010856	-1.82	1	0.00045725		
1	CTD-2538C1.2	4.0947E-06	2.06	0.2382052	1.8911E-05		
1	CTD-2541J13.	0.00102977	2.33	1	0.0040084		
1	CTD-2541J13.	4.2744E-11	16.84	2.4866E-06	2.5186E-10		
1	CTD-2542C24.	6.6873E-06	40.43	0.3890274	3.0553E-05		
1	CTD-2542C24.	1.0013E-06	53.47	0.05825059	4.8069E-06		
1	CTD-2547L16.	0.00014177	-1.96	1	0.00059172		
1	CTD-2547L24.	0.0002136	-4.53	1	0.00088009		
1	CTD-2550O8.5	0.00751696	2.87	1	0.0268014		
1	CTD-2554C21	0.00047685	-10.69	1	0.00190889		
1	CTD-2555A7.2	0.00074927	-11.30	1	0.00295073		
1	CTD-2555C10.	6.4051E-27	-7.67	3.7261E-22	5.5589E-26		
1	CTD-2555K7.2	0.00201628	-1.52	1	0.0076245		
1	CTD-2555O16	0.00513555	-3.47	1	0.01865938		
1	CTD-2555O16	0.00421199	2.76	1	0.01545238		
1	CTD-2561F5.1	0.00011929	10.24	1	0.00050095		
1	CTD-2561J22.	5.6742E-87	21.26	3.3009E-82	8.8877E-86		
1	CTD-2561J22.	8.946E-106	194.40	5.204E-101	1.595E-104		
1	CTD-2561J22.	1.416E-205	657.53	8.236E-201	3.623E-204		
1	CTD-2583A14	0.00342318	1.51	1	0.01267311		
1	CTD-2587H24	5.0865E-05	-12.50	1	0.00021919		

1	CTD-2587H24	4.4012E-06	1.96	0.25603266	2.0299E-05		
1	CTD-2587H24	8.9214E-20	28.07	5.1899E-15	6.7789E-19		
1	CTD-2588J6.2	8.4158E-08	14.62	0.00489578	4.2777E-07		
1	CTD-2589M5.	4.4629E-12	9.60	2.5963E-07	2.7355E-11		
1	CTD-2591A6.2	5.5608E-23	-9.11	3.2349E-18	4.4904E-22		
1	CTD-2609K8.3	0.01396944	-17.83	1	0.04823182		
1	CTD-2616J11.	0.00025012	8.73	1	0.00102383		
1	CTD-2616J11.	0.00127668	-3.19	1	0.00492765		
1	CTD-2619J13.	4.9224E-06	-9.45	0.28635486	2.2639E-05		
1	CTD-2619J13.	3.1782E-05	2.23	1	0.00013891		
1	CTD-2619J13.	0.0002738	1.75	1	0.00111626		
1	CTD-2620I22.1	2.252E-106	95.98	1.31E-101	4.027E-105		
1	CTD-2630F21.	9.7022E-05	1.99	1	0.00041013		
1	CTD-2636A23	5.7652E-10	-7.98	3.3539E-05	3.2452E-09		
1	CTD-2647L4.4	0.00010008	-2.45	1	0.00042257		
1	CTD-2651B20	0.00398494	-3.04	1	0.01465822		
1	CTD-2651B20	3.7652E-05	4.36	1	0.00016375		
1	CTD-2655K5.1	8.8114E-15	-13.90	5.126E-10	5.9082E-14		
1	CTD-2666L21.	3.4689E-06	3.28	0.20179851	1.61E-05		
1	CTD-3006G17	6.2026E-52	-9.14	3.6083E-47	7.391E-51		
1	CTD-3012A18	0.0035383	23.37	1	0.01307236		
1	CTD-3035D6.2	1.2389E-10	28.54	7.207E-06	7.1797E-10		
1	CTD-3051D23	1.1091E-05	-9.35	0.64519401	4.9938E-05		
1	CTD-3064H18	0.00027133	30.04	1	0.00110657		
1	CTD-3064M3.	1.3681E-05	-36.60	0.79585393	6.1238E-05		
1	CTD-3064M3.	0.00038576	-32.26	1	0.00155475		
1	CTD-3074O7.5	9.4318E-07	-1.57	0.05486875	4.5365E-06		
1	CTD-3088G3.8	5.2434E-37	-7.57	3.0503E-32	5.2929E-36		
1	CTD-3099C6.1	6.337E-14	15.58	3.6865E-09	4.137E-13		
1	CTD-3105H18	0.01136155	2.30	1	0.03961086		
1	CTD-3105H18	2.9713E-06	1.80	0.17285272	1.3858E-05		
1	CTD-3105H18	1.4932E-13	6.51	8.6865E-09	9.6335E-13		
1	CTD-3105H18	1.3067E-06	29.19	0.07601691	6.235E-06		
1	CTD-3126B10	2.19E-06	-4.50	0.12740099	1.0313E-05		
1	CTD-3128G10	1.5781E-11	-5.33	9.1804E-07	9.4878E-11		
1	CTD-3131K8.2	6.4641E-25	3.09	3.7604E-20	5.4177E-24		
1	CTD-3157E16.	5.0103E-08	-3.89	0.00291467	2.5773E-07		
1	CTD-3157E16.	3.5655E-22	-10.84	2.0742E-17	2.8313E-21		
1	CTD-3162L10.	3.6661E-14	10.96	2.1327E-09	2.412E-13		
1	CTD-3184A7.4	8.0236E-47	2.27	4.6676E-42	9.1094E-46		
1	CTD-3185P2.1	0.00757928	-1.53	1	0.02701531		
1	CTD-3214H19	0.00033754	-4.35	1	0.00136705		
1	CTD-3214H19	0.0131932	-2.14	1	0.04563569		
1	CTD-3220F14.	0.00057037	5.21	1	0.0022694		
1	CTD-3234P18.	5.4847E-09	8.24	0.00031907	2.9562E-08		
1	CTD-3252C9.4	0	-2.13	0	0		
1	CTF1	1.1294E-98	-2.96	6.5705E-94	1.9285E-97	Q16619	CTF1

1	CTGF	9.717E-183	58.19	5.653E-178	2.307E-181	P29279	CTGF
1	CTHRC1	6.9502E-22	-4.09	4.0432E-17	5.4928E-21	Q96CG8	CTHRC1
1	CTIF	2.7789E-35	3.94	1.6166E-30	2.7507E-34	O43310	CTIF
1	CTNNA3	5.669E-05	-2.41	1	0.00024366	Q9UI47	CTNNA3
1	CTNNAL1	1.0908E-46	1.90	6.3455E-42	1.236E-45	Q9UBT7	CTNNAL1
1	CTNNB1	9.6974E-88	1.75	5.6414E-83	1.5292E-86	P35222	CTNNB1
1	CTNNBIP1	0	-2.20	0	0	Q9NSA3	CTNNBIP1
1	CTNNBL1	1.8446E-62	1.60	1.0731E-57	2.3963E-61	Q8WYA6	CTNNBL1
1	CTNND1	0	-3.03	0	0	O60716	CTNND1
1	CTPS1	1.2194E-88	1.70	7.0936E-84	1.9387E-87	P17812	CTPS1
1	CTR9	4.283E-115	1.99	2.492E-110	8.074E-114	Q6PD62	CTR9
1	CTRB2	0.00139055	-23.40	1	0.00534907	Q6GPI1	CTRB2
1	CTRC	6.1324E-13	-6.91	3.5674E-08	3.8747E-12	Q99895	CTRC
1	CTRL	0.00087917	1.57	1	0.00344062	P40313	CTRL
1	CTSA	9.282E-77	1.77	5.3997E-72	1.3395E-75	P10619	CTSA
1	CTSD	0	-2.89	0	0	P07339	CTSD
1	CTSF	4.0893E-86	-6.85	2.3789E-81	6.3692E-85	Q9UBX1	CTSF
1	CTSH	0	-7.55	0	0	P09668	CTSH
1	CTSK	2.1585E-12	-1.87	1.2557E-07	1.3397E-11	P43235	CTSK
1	CTSL	0	169.41	0	0	P07711	CTSL
1	CTSO	1.93E-157	-14.94	1.123E-152	4.255E-156	P43234	CTSO
1	CTSS	1.2453E-28	-8.04	7.2443E-24	1.1197E-27	P25774	CTSS
1	CTSV	0	-2.28	0	0	O60911	CTSV
1	CTSZ	1.7263E-73	3.31	1.0042E-68	2.4287E-72	Q9UBR2	CTSZ
1	CTTN	0	-2.79	0	0	Q14247	CTTN
1	CTTNBP2	2.8728E-35	-6.52	1.6712E-30	2.8432E-34	Q8WZ74	CTTNBP2
1	CTTNBP2NL	7.5347E-58	4.17	4.3832E-53	9.4466E-57	Q9P2B4	CTTNBP2NL
1	CTXN1	9.067E-178	24.59	5.275E-173	2.125E-176	P60606	CTXN1
1	CUBN	9.3686E-32	14.94	5.4501E-27	8.8375E-31	O60494	CUBN
1	CUEDC1	5.2168E-27	2.24	3.0348E-22	4.5418E-26		
1	CUL2	6.7836E-76	5.29	3.9463E-71	9.7271E-75	Q13617	CUL2
1	CUL4B	1.0405E-61	1.74	6.053E-57	1.3433E-60	Q13620	CUL4B
1	CUL7	0	-1.95	0	0	Q14999	CUL7
1	CUL9	0	-1.56	0	0	Q8IWT3	CUL9
1	CUTC	2.1418E-82	2.20	1.246E-77	3.2413E-81	Q9NTM9	CUTC
1	CUZD1	3.7091E-43	-4.40	2.1577E-38	4.0483E-42	Q86UP6	CUZD1
1	CWF19L1	8.584E-64	1.67	4.9937E-59	1.127E-62	Q69YN2	CWF19L1
1	CXADR	3.133E-243	-10.28	1.823E-238	8.53E-242	P78310	CXADR
1	CXCL1	0	37.34	0	0	P09341	CXCL1
1	CXCL12	0.00049525	25.92	1	0.00197918	P48061	CXCL12
1	CXCL13	0.00671201	-1.92	1	0.02404338	O43927	CXCL13
1	CXCL16	7.5825E-96	-5.76	4.4111E-91	1.2672E-94	Q9H2A7	CXCL16
1	CXCL2	3.4534E-15	1.86	2.009E-10	2.348E-14	P19875	CXCL2
1	CXCL3	5.141E-102	5.35	2.9906E-97	8.97E-101	P19876	CXCL3
1	CXCL5	3.4541E-68	-7.69	2.0094E-63	4.6697E-67	P42830	CXCL5
1	CXCL6	1.143E-110	49.48	6.647E-106	2.101E-109	P80162	CXCL6
1	CXCL8	0	65.49	0	0	P10145	CXCL8

1	CXCR2	1.2603E-08	-21.01	0.00073314	6.685E-08	P25025	CXCR2
1	CXCR5	2.4348E-24	-1329.27	1.4164E-19	2.0171E-23	P32302	CXCR5
1	CXorf23	3.8842E-10	-1.60	2.2596E-05	2.2021E-09	A2AJT9	CXorf23
1	CXorf38	0	-2.33	0	0		
1	CXorf40A	3.1838E-17	1.61	1.8521E-12	2.2931E-16	Q8TE69	CXorf40A
1	CXorf49	3.4042E-11	-75.82	1.9804E-06	2.0163E-10		
1	CXorf49B	4.8293E-09	-66.71	0.00028094	2.6095E-08		
1	CXorf56	2.6976E-27	1.57	1.5693E-22	2.3641E-26		
1	CXorf57	5.56E-186	-10.57	3.234E-181	1.333E-184	Q6NSI4	CXorf57
1	CXXC1	2.7981E-43	2.40	1.6278E-38	3.0608E-42	Q9P0U4	CXXC1
1	CXXC4	2.8E-111	-23.88	1.629E-106	5.172E-110	Q9H2H0	CXXC4
1	CXXC4-AS1	3.8116E-37	-11.75	2.2174E-32	3.8543E-36		
1	CXXC5	1.841E-195	-5.33	1.071E-190	4.581E-194	Q7LFL8	CXXC5
1	CYB561	0	-2.36	0	0	P49447	CYB561
1	CYB561A3	0	-1.87	0	0	Q8NBI2	CYB561A3
1	CYB561D2_1	4.3966E-47	2.11	2.5577E-42	5.0033E-46		
1	CYB5A	8.3097E-90	86.71	4.8341E-85	1.3313E-88	P00167	CYB5A
1	CYB5D1	5.2503E-67	2.06	3.0543E-62	7.049E-66	Q6P9G0	CYB5D1
1	CYB5D2	0	-2.31	0	0	Q8WUJ1	CYB5D2
1	CYB5R1	0	-1.50	0	0	Q9UHQ9	CYB5R1
1	CYB5R2	3.6757E-15	6.41	2.1383E-10	2.4974E-14	Q6BCY4	CYB5R2
1	CYBA	0	-1.84	0	0	P13498	CYBA
1	CYCSP6	5.8924E-12	-8.40	3.4278E-07	3.5954E-11		
1	CYFIP2	2.157E-213	43.21	1.255E-208	5.613E-212	Q96F07	CYFIP2
1	CYGB	2.1312E-08	-6.75	0.00123983	1.1186E-07	Q8WWM9	CYGB
1	CYHR1	0	-1.70	0	0	Q6ZMK1	CYHR1
1	CYMP	3.6863E-07	-5.79	0.0214449	1.8112E-06		
1	CYMP-AS1	4.757E-11	-6.75	2.7673E-06	2.7984E-10		
1	CYP11A1	3.7434E-09	-59.04	0.00021777	2.0343E-08	P05108	CYP11A1
1	CYP17A1	7.9322E-08	-4.22	0.00461449	4.0365E-07	P05093	CYP17A1
1	CYP19A1	5.9587E-15	-3.23	3.4664E-10	4.0134E-14	P11511	CYP19A1
1	CYP1A1	1.9635E-54	-4.79	1.1422E-49	2.3992E-53	P04798	CYP1A1
1	CYP1B1	5.2858E-87	-5.08	3.075E-82	8.2817E-86	Q16678	CYP1B1
1	CYP1B1-AS1	1.8872E-14	-5.26	1.0979E-09	1.2528E-13		
1	CYP20A1	4.9998E-39	1.83	2.9086E-34	5.1782E-38	Q6UW02	CYP20A1
1	CYP24A1	1.1097E-10	-2.33	6.4556E-06	6.4402E-10	Q07973	CYP24A1
1	CYP26A1	6.3849E-84	5.70	3.7143E-79	9.7849E-83	O43174	CYP26A1
1	CYP26B1	6.7766E-36	2.29	3.9422E-31	6.7527E-35	Q9NR63	CYP26B1
1	CYP27C1	8.373E-103	-4.67	4.8708E-98	1.468E-101	Q4G0S4	CYP27C1
1	CYP2AB1P	3.2663E-15	-13.38	1.9002E-10	2.2229E-14		
1	CYP2B6	0.00024034	-7.46	1	0.00098558	P20813	CYP2B6
1	CYP2C8	0.00284735	-3.56	1	0.01061602	P10632	CYP2C8
1	CYP2D6	8.7032E-20	-3.98	5.063E-15	6.6157E-19	P10635	CYP2D6
1	CYP2J2	8.7136E-90	-16.51	5.0691E-85	1.3953E-88	P51589	CYP2J2
1	CYP2S1	2.8825E-64	-3.34	1.6769E-59	3.7973E-63	Q96SQ9	CYP2S1
1	CYP2T1P	5.8599E-05	-18.02	1	0.00025145		
1	CYP2U1	4.9444E-29	-2.43	2.8764E-24	4.4747E-28	Q7Z449	CYP2U1

1	CYP2W1	7.292E-127	-37.19	4.242E-122	1.455E-125	Q8TAV3	CYP2W1
1	CYP39A1	8.0995E-41	-8.80	4.7118E-36	8.5575E-40	Q9NYL5	CYP39A1
1	CYP4A22-AS1	9.0735E-23	-4.42	5.2784E-18	7.3037E-22		
1	CYP4F11	0	-20.33	0	0	Q9HBI6	CYP4F11
1	CYP4F12	1.5522E-47	-50.08	9.0295E-43	1.7743E-46	Q9HCS2	CYP4F12
1	CYP4F2	0.00670241	-7.62	1	0.02401047	P78329	CYP4F2
1	CYP4F29P	0	-2.08	0	0		
1	CYP4F3	2.575E-205	-35.67	1.498E-200	6.581E-204	Q08477	CYP4F3
1	CYP4F34P	1.536E-137	178.81	8.936E-133	3.188E-136		
1	CYP4V2	8.3082E-45	-2.49	4.8332E-40	9.2237E-44	Q6ZWL3	CYP4V2
1	CYP4X1	4.1466E-09	-13.06	0.00024123	2.2469E-08	Q8N118	CYP4X1
1	CYP4Z1	8.4185E-09	-5.86	0.00048974	4.4996E-08	Q86W10	CYP4Z1
1	CYP4Z2P	0.0045097	-5.64	1	0.01648636	Q8N1L4	CYP4Z2P
1	CYR61	1.3704E-49	2.88	7.9724E-45	1.598E-48	O00622	CYR61
1	CYSRT1	5.7982E-32	-2.43	3.373E-27	5.4882E-31	A8MQ03	CYSRT1
1	CYSTM1	1.1573E-79	2.45	6.7325E-75	1.7122E-78	Q9H1C7	CYSTM1
1	CYTH1	0	-1.77	0	0	Q15438	CYTH1
1	CYTOR	3.0853E-80	2.08	1.7949E-75	4.5916E-79		
1	CYYR1	0.00393992	-19.70	1	0.01449903	Q96J86	CYYR1
1	CYYR1-AS1	1.1233E-12	-26.07	6.5346E-08	7.031E-12		
1	D2HGDH	0	-2.20	0	0	Q8N465	D2HGDH
1	DAAM1	0	-1.74	0	0	Q9Y4D1	DAAM1
1	DAB1	3.0385E-07	-2.17	0.0176762	1.4987E-06	O75553	DAB1
1	DAB2	1.0627E-85	4.11	6.1823E-81	1.6495E-84	P98082	DAB2
1	DAB2IP	0	-3.54	0	0	Q5VWQ8	DAB2IP
1	DACH1	1.8751E-17	-7.91	1.0908E-12	1.3579E-16	Q9UI36	DACH1
1	DACH2	8.9561E-31	-12.01	5.2101E-26	8.3336E-30	Q96NX9	DACH2
1	DACT1	1.694E-208	-43.65	9.855E-204	4.357E-207	Q9NYF0	DACT1
1	DACT3	0.00322309	2.70	1	0.01195563	Q96B18	DACT3
1	DAD1	3.6621E-23	1.71	2.1304E-18	2.9663E-22	P61803	DAD1
1	DAG1	0	-1.53	0	0	Q14118	DAG1
1	DAGLA	4.057E-125	-4.16	2.36E-120	8.025E-124	Q9Y4D2	DAGLA
1	DAGLB	0	-1.99	0	0	Q8NCG7	DAGLB
1	DANCR	9.2963E-23	1.71	5.4081E-18	7.48E-22		
1	DANT1	2.5563E-10	-9.63	1.4871E-05	1.4608E-09		
1	DAO	1.3977E-09	-8.43	8.131E-05	7.7365E-09	P14920	DAO
1	DAP	1.0689E-62	3.11	6.218E-58	1.3914E-61	P51397	DAP
1	DAPK3	7.0693E-75	2.00	4.1125E-70	1.0053E-73	O43293	DAPK3
1	DAPP1	5.718E-113	-16.09	3.326E-108	1.066E-111	Q9UN19	DAPP1
1	DARS-AS1	1.6374E-23	-2.65	9.5256E-19	1.3354E-22		
1	DARS2	0	-1.97	0	0	Q6PI48	DARS2
1	DAW1	1.331E-31	18.52	7.7427E-27	1.2527E-30	Q8N136	DAW1
1	DBF4	0	-1.64	0	0	Q9UBU7	DBF4
1	DBN1	0	21.79	0	0	Q16643	DBN1
1	DBNDD1	8.9153E-39	2.42	5.1864E-34	9.1908E-38	Q9H9R9	DBNDD1
1	DBNDD2	1.3982E-49	2.22	8.134E-45	1.6297E-48	Q9BQY9	DBNDD2
1	DBP	2.892E-192	-5.07	1.682E-187	7.105E-191	Q10586	DBP

1	DCAF12	1.3725E-43	1.55	7.9841E-39	1.5081E-42	Q5T6F0	DCAF12
1	DCAF12L2	2.2446E-14	-10.94	1.3057E-09	1.4858E-13		
1	DCAF13P1	5.5942E-08	-51.51	0.00325435	2.87E-07		
1	DCAF4	2.114E-106	5.54	1.23E-101	3.781E-105	Q8WV16	DCAF4
1	DCAF4L2	0.00702386	-5.32	1	0.02510946		
1	DCAF6	0	-1.63	0	0	Q58WW2	DCAF6
1	DCAKD	0	-2.53	0	0	Q8WVC6	DCAKD
1	DCBLD1	3.195E-56	1.99	1.8587E-51	3.9588E-55	Q8N8Z6	DCBLD1
1	DCBLD2	5.823E-278	9.25	3.387E-273	1.624E-276	Q96PD2	DCBLD2
1	DCDC1	2.9368E-10	-3.23	1.7085E-05	1.6737E-09	P59894	DCDC1
1	DCDC2	4.874E-177	61.86	2.836E-172	1.14E-175	Q9UHG0	DCDC2
1	DCHS1	2.3435E-12	2.53	1.3633E-07	1.4525E-11	Q96JQ0	DCHS1
1	DCLK1	3.2431E-22	3.52	1.8866E-17	2.5784E-21	O15075	DCLK1
1	DCLK2	4.7813E-76	9.27	2.7815E-71	6.8678E-75	Q8N568	DCLK2
1	DCLRE1B	4.33E-113	2.80	2.519E-108	8.079E-112	Q9H816	DCLRE1B
1	DCP1B	4.397E-196	104.71	2.558E-191	1.095E-194	Q8IZD4	DCP1B
1	DCP2	3.9508E-28	1.56	2.2984E-23	3.5251E-27	Q8IU60	DCP2
1	DCST1	1.4249E-32	-3.60	8.2894E-28	1.3616E-31	Q5T197	DCST1
1	DCST1-AS1	1.3945E-13	-2.81	8.1121E-09	9.0064E-13		
1	DCST2	8.2779E-52	-10.20	4.8156E-47	9.8458E-51	Q5T1A1	DCST2
1	DCT	0.01226301	-7.01	1	0.04256746	P40126	DCT
1	DCTD	0	-2.59	0	0	P32321	DCTD
1	DCTN3	1.0632E-20	1.59	6.1848E-16	8.2124E-20	O75935	DCTN3
1	DCTN4	6.1946E-48	1.66	3.6036E-43	7.1162E-47	Q9UJW0	DCTN4
1	DCTN5	3.8915E-63	1.53	2.2639E-58	5.0782E-62	Q9BTE1	DCTN5
1	DCTN6	0	-1.62	0	0	O00399	DCTN6
1	DCTPP1	9.2573E-96	2.59	5.3854E-91	1.5462E-94	Q9H773	DCTPP1
1	DCUN1D4	0	-1.54	0	0	Q92564	DCUN1D4
1	DCXR	2.419E-33	1.86	1.4072E-28	2.3368E-32	Q7Z4W1	DCXR
1	DDAH1	5.1812E-35	3.20	3.0141E-30	5.1095E-34	O94760	DDAH1
1	DDAH2	5.9863E-54	4.44	3.4825E-49	7.2916E-53	O95865	DDAH2
1	DDB1	0	-1.63	0	0	Q16531	DDB1
1	DDHD1	2.9947E-14	1.53	1.7422E-09	1.9752E-13	Q8NEL9	DDHD1
1	DDHD2	0	-1.65	0	0	O94830	DDHD2
1	DDIT4	0	-7.92	0	0	Q9NX09	DDIT4
1	DDN	8.285E-141	-4.94	4.82E-136	1.738E-139	O94850	DDN
1	DDO	7.2285E-09	-8.95	0.00042051	3.8753E-08	Q99489	DDO
1	DDR1	0	-8.92	0	0	Q08345	DDR1
1	DDR1-AS1	0.00420156	-4.81	1	0.01541604		
1	DDR2	1.1004E-40	3.95	6.4016E-36	1.1603E-39	Q16832	DDR2
1	DDRGK1	4.8246E-27	1.50	2.8067E-22	4.2029E-26	Q96HY6	DDRGK1
1	DDT	3.2046E-51	2.27	1.8642E-46	3.7876E-50	P30046	DDT
1	DDTL	2.785E-33	2.23	1.6202E-28	2.6868E-32	A6NHG4	DDTL
1	DDX1	5.0472E-50	1.84	2.9361E-45	5.9101E-49	Q92499	DDX1
1	DDX10	9.5954E-86	1.93	5.582E-81	1.4901E-84	Q13206	DDX10
1	DDX11	8.4181E-26	1.61	4.8972E-21	7.1648E-25	Q96FC9	DDX11
1	DDX11L2	4.6669E-09	1.72	0.00027149	2.5232E-08		

1	DDX12P	2.4852E-10	1.65	1.4458E-05	1.4208E-09	Q92771	DDX12P
1	DDX18	8.8943E-23	1.51	5.1742E-18	7.1605E-22	Q9NVP1	DDX18
1	DDX20	2.0426E-46	2.04	1.1883E-41	2.3082E-45	Q9UHI6	DDX20
1	DDX21	4.9336E-50	2.33	2.8701E-45	5.7795E-49	Q9NR30	DDX21
1	DDX24	4.351E-108	1.85	2.531E-103	7.863E-107	Q9GZR7	DDX24
1	DDX27	6.389E-145	1.93	3.717E-140	1.359E-143	Q96GQ7	DDX27
1	DDX39A	1.775E-36	1.50	1.0326E-31	1.7834E-35	O00148	DDX39A
1	DDX41	2.0413E-39	1.50	1.1875E-34	2.1225E-38	Q9UJV9	DDX41
1	DDX46	7.2307E-66	1.56	4.2064E-61	9.641E-65	Q7L014	DDX46
1	DDX47	4.8418E-26	1.64	2.8167E-21	4.1373E-25	Q9H0S4	DDX47
1	DDX49	8.742E-116	2.78	5.086E-111	1.654E-114	Q9Y6V7	DDX49
1	DDX50	1.5606E-33	1.74	9.0784E-29	1.5121E-32	Q9BQ39	DDX50
1	DDX51	5.8197E-33	1.55	3.3855E-28	5.5959E-32	Q8N8A6	DDX51
1	DDX55	1.5902E-48	1.63	9.2508E-44	1.8362E-47	Q8NHQ9	DDX55
1	DDX58	1.1039E-56	3.47	6.422E-52	1.3722E-55	O95786	DDX58
1	DDX59	0	-1.95	0	0	Q5T1V6	DDX59
1	DDX6	1.9215E-82	1.72	1.1178E-77	2.9117E-81	P26196	DDX6
1	DDX60	1.292E-189	-7.46	7.517E-185	3.137E-188	Q8IY21	DDX60
1	DDX60L	5.1528E-80	-3.95	2.9976E-75	7.643E-79	Q5H9U9	DDX60L
1	DEAF1	9.3551E-46	2.51	5.4423E-41	1.0506E-44	O75398	DEAF1
1	DEC1	1.2945E-06	-2.54	0.07530673	6.1793E-06	Q9P2X7	DEC1
1	DECR1	0	-2.03	0	0	Q16698	DECR1
1	DECR2	8.5104E-83	2.71	4.9508E-78	1.2916E-81	Q9NUI1	DECR2
1	DEDD	0	-1.50	0	0	O75618	DEDD
1	DEFB107B	5.4701E-18	-21.76	3.1822E-13	4.0048E-17		
1	DEFB133	4.838E-18	-148.67	2.8144E-13	3.5477E-17	Q30KQ1	DEFB133
1	DENND1B	1.6706E-13	-1.92	9.7188E-09	1.0757E-12	Q6P3S1	DENND1B
1	DENND1C	2.336E-202	-13.16	1.359E-197	5.935E-201	Q8IV53	DENND1C
1	DENND2A	7.779E-140	68.31	4.525E-135	1.629E-138	Q9ULE3	DENND2A
1	DENND2C	6.3539E-95	-4.39	3.6963E-90	1.0567E-93	Q68D51	DENND2C
1	DENND2D	2.826E-166	-12.71	1.644E-161	6.395E-165	Q9H6A0	DENND2D
1	DENND4C	0	-1.56	0	0	Q5VZ89	DENND4C
1	DENND5A	9.5964E-80	3.72	5.5826E-75	1.4212E-78	Q6IQ26	DENND5A
1	DENND5B	1.7653E-74	3.54	1.0269E-69	2.5041E-73	Q6ZUT9	DENND5B
1	DENND5B-AS1	1.6002E-14	-4.95	9.3088E-10	1.0644E-13		
1	DENND6A	0	-1.64	0	0	Q8IWF6	DENND6A
1	DENND6B	3.2218E-24	-2.66	1.8742E-19	2.663E-23	Q8NEG7	DENND6B
1	DENR	3.8992E-58	1.68	2.2683E-53	4.9023E-57	O43583	DENR
1	DEPDC7	1.552E-134	-9.14	9.028E-130	3.177E-133	Q96QD5	DEPDC7
1	DERL1	0	-1.76	0	0	Q9BUN8	DERL1
1	DERL3	1.0272E-23	20.86	5.9756E-19	8.4045E-23	Q96Q80	DERL3
1	DET1	1.4838E-11	-1.54	8.6321E-07	8.9304E-11	Q7L5Y6	DET1
1	DEXI	1.299E-26	1.58	7.5567E-22	1.1217E-25		
1	DFFB	1.0401E-64	2.31	6.0506E-60	1.3751E-63	O76075	DFFB
1	DFNA5	1.7892E-52	2.94	1.0409E-47	2.1439E-51	O60443	DFNA5
1	DFNB59	5.6365E-06	-1.58	0.32789548	2.5867E-05	Q0ZLH3	DFNB59
1	DGAT1	0	-1.81	0	0	O75907	DGAT1

1	DGAT2	4.2183E-11	2.27	2.4539E-06	2.486E-10	Q96PD7	DGAT2
1	DGCR14	1.114E-126	2.12	6.481E-122	2.22E-125	Q96DF8	DGCR14
1	DGCR6	2.0323E-05	1.81	1	8.9946E-05	Q14129	DGCR6
1	DGCR6L	9.367E-118	2.81	5.449E-113	1.79E-116	Q9BY27	DGCR6L
1	DGKA	0	-5.81	0	0	P23743	DGKA
1	DGKG	2.352E-129	-8.59	1.368E-124	4.735E-128	P49619	DGKG
1	DGKH	7.1054E-15	-1.69	4.1335E-10	4.7753E-14	Q86XP1	DGKH
1	DGUOK-AS1	1.8229E-28	-3.90	1.0604E-23	1.6332E-27		
1	DHCR7	0	-2.76	0	0	Q9UBM7	DHCR7
1	DHDH	2.8117E-06	-3.48	0.1635654	1.3143E-05	Q9UQ10	DHDH
1	DHFR2	4.885E-15	-1.50	2.8418E-10	3.2983E-14	Q86XF0	DHFR2
1	DHRS12	1.416E-96	-4.27	8.2374E-92	2.3773E-95	A0PJE2	DHRS12
1	DHRS2	2.8148E-86	4.69	1.6375E-81	4.3888E-85	Q13268	DHRS2
1	DHRS3	0	-3.88	0	0	O75911	DHRS3
1	DHRS4	8.8401E-45	1.89	5.1426E-40	9.8104E-44	Q9BTZ2	DHRS4
1	DHRS4-AS1	1.6973E-21	1.51	9.8738E-17	1.3323E-20		
1	DHRS4L2	6.7009E-23	1.64	3.8982E-18	5.4051E-22	Q6PKH6	DHRS4L2
1	DHRS7B	0	-1.87	0	0	Q6IAN0	DHRS7B
1	DHRS9	7.5864E-37	-11.61	4.4133E-32	7.6447E-36	Q9BPW9	DHRS9
1	DHTKD1	0	-2.01	0	0	Q96HY7	DHTKD1
1	DHX29	3.569E-81	2.63	2.0763E-76	5.347E-80	Q7Z478	DHX29
1	DHX32	0	-1.56	0	0	Q7L7V1	DHX32
1	DHX33	1.0453E-93	1.82	6.0809E-89	1.7231E-92	Q9H6R0	DHX33
1	DHX37	1.1844E-48	1.60	6.8904E-44	1.3693E-47	Q8IY37	DHX37
1	DHX58	2.219E-199	-23.67	1.291E-194	5.581E-198	Q96C10	DHX58
1	DIMT1	9.9725E-20	1.52	5.8014E-15	7.5697E-19	Q9UNQ2	DIMT1
1	DIO1	0.00289871	1.83	1	0.01079852	P49895	DIO1
1	DIO2	2.4296E-21	2.97	1.4134E-16	1.901E-20	Q92813	DIO2
1	DIO2-AS1	1.712E-24	3.14	9.9594E-20	1.4242E-23		
1	DIP2A	0	-1.80	0	0	Q14689	DIP2A
1	DIRAS3	0.01399811	1.88	1	0.04832221	O95661	DIRAS3
1	DISC1FP1	2.5949E-20	4.51	1.5095E-15	1.9889E-19		
1	DISP2	8.906E-111	6.01	5.181E-106	1.641E-109	A7MBM2	DISP2
1	DIXDC1	5.4929E-11	1.77	3.1954E-06	3.2225E-10	Q155Q3	DIXDC1
1	DKC1	4.501E-99	1.75	2.6184E-94	7.6923E-98	O60832	DKC1
1	DKFZP434I071	3.5289E-11	-1.64	2.0529E-06	2.0882E-10		
1	DKFZP434K02	4.7573E-05	-32.82	1	0.00020543		
1	DKK1	2.842E-112	4.74	1.653E-107	5.281E-111	O94907	DKK1
1	DKK3	0	-1.72	0	0	Q9UBP4	DKK3
1	DLAT	1.3494E-55	1.75	7.8502E-51	1.6657E-54	P10515	DLAT
1	DLC1	0	168.48	0	0	Q96QB1	DLC1
1	DLEC1	6.2989E-07	-3.14	0.03664301	3.0577E-06	Q9Y238	DLEC1
1	DLEU1	0	-1.88	0	0	O43261	DLEU1
1	DLEU7	1.2756E-24	-11.76	7.4205E-20	1.0634E-23		
1	DLEU7-AS1	0.00026159	-7.07	1	0.00106868		
1	DLG1	0.00015336	-1.88	1	0.00063802	Q12959	DLG1
1	DLG1-AS1	1.3455E-56	-5.29	7.8275E-52	1.6711E-55		

1	DLGAP4	9.1806E-52	3.37	5.3407E-47	1.0908E-50	Q9Y2H0	DLGAP4
1	DLGAP5	1.0686E-21	1.61	6.2166E-17	8.4202E-21	Q15398	DLGAP5
1	DLK2	5.8498E-74	-5.22	3.403E-69	8.2598E-73	Q6UY11	DLK2
1	DLL1	0	-9.77	0	0	O00548	DLL1
1	DLL3	1.506E-60	41.66	8.7608E-56	1.9254E-59	Q9NYJ7	DLL3
1	DLST	3.0445E-12	2.11	1.7711E-07	1.8792E-11	P36957	DLST
1	DLX1	3.1178E-67	5.38	1.8138E-62	4.1947E-66	P56177	DLX1
1	DLX2-AS1	3.7634E-05	-3.53	1	0.00016369		
1	DLX3	0.0044581	-5.64	1	0.01630488	O60479	DLX3
1	DLX4	0.00540108	-1.68	1	0.01957525	Q92988	DLX4
1	DLX5	1.3155E-06	-16.14	0.07652631	6.2747E-06	P56178	DLX5
1	DLX6	1.173E-42	-12.04	6.8239E-38	1.2717E-41	P56179	DLX6
1	DLX6-AS1	8.0485E-90	-17.25	4.6821E-85	1.2898E-88		
1	DMBT1	0.00025709	4.71	1	0.00105102	Q9UGM3	DMBT1
1	DMBX1	5.3984E-12	-3.41	3.1405E-07	3.2985E-11	Q8NFW5	DMBX1
1	DMC1	0.00205845	-2.69	1	0.00777638	Q14565	DMC1
1	DMD	1.743E-211	7.94	1.014E-206	4.52E-210	P11532	DMD
1	DMGDH	1.8165E-06	-1.88	0.10567236	8.5968E-06	Q9UI17	DMGDH
1	DMKN	3.4018E-11	-2.13	1.979E-06	2.015E-10	Q6E0U4	DMKN
1	DMP1	0.00040657	-14.09	1	0.00163544	Q13316	DMP1
1	DMRTA1	7.3151E-33	-2.82	4.2555E-28	7.0246E-32	Q5VZB9	DMRTA1
1	DMTF1	0	-1.54	0	0	Q9Y222	DMTF1
1	DNA2	5.2695E-48	1.61	3.0655E-43	6.0582E-47	P51530	DNA2
1	DNAAF2	2.0766E-32	1.82	1.2081E-27	1.9794E-31	Q9NVR5	DNAAF2
1	DNAAF3	6.2772E-39	2.61	3.6517E-34	6.4873E-38	Q8N9W5	DNAAF3
1	DNAAF5	0	-1.66	0	0	Q86Y56	DNAAF5
1	DNAH10	0.00048661	-3.58	1	0.00194599	Q8IVF4	DNAH10
1	DNAH10OS	2.9805E-06	-4.52	0.17338944	1.3898E-05		
1	DNAH12	0.00019432	-1.89	1	0.00080351	Q6ZR08	DNAH12
1	DNAH14	0	-2.89	0	0	Q0VDD8	DNAH14
1	DNAH17-AS1	6.839E-06	-47.98	0.39785181	3.1224E-05		
1	DNAH2	1.9748E-14	-4.65	1.1488E-09	1.31E-13	Q9P225	DNAH2
1	DNAH3	3.7156E-72	-5.56	2.1615E-67	5.1766E-71	Q8TD57	DNAH3
1	DNAH5	2.3314E-52	-3.84	1.3563E-47	2.7907E-51	Q8TE73	DNAH5
1	DNAH6	1.6232E-07	-2.85	0.00944306	8.128E-07	Q9C0G6	DNAH6
1	DNAI1	0.01421275	1.87	1	0.04903406	Q9UI46	DNAI1
1	DNAJA1	1.031E-103	3.55	5.995E-99	1.818E-102	P31689	DNAJA1
1	DNAJA1P3	0.00853159	-7.62	1	0.03021717		
1	DNAJA4	0	-9.50	0	0	Q8WW22	DNAJA4
1	DNAJB13	0.00457014	1.84	1	0.01670311	P59910	DNAJB13
1	DNAJB14	0	-1.80	0	0	Q8TBM8	DNAJB14
1	DNAJB5	5.7083E-46	2.44	3.3208E-41	6.4244E-45	O75953	DNAJB5
1	DNAJB6	1.1199E-11	2.21	6.515E-07	6.7681E-11	O75190	DNAJB6
1	DNAJB7	0.00050045	3.90	1	0.00199924	Q7Z6W7	DNAJB7
1	DNAJB9	2.2939E-24	-1.97	1.3344E-19	1.9025E-23	Q9UBS3	DNAJB9
1	DNAJC11	2.814E-120	2.05	1.637E-115	5.449E-119	Q9NVH1	DNAJC11
1	DNAJC12	1.9668E-21	-6.49	1.1442E-16	1.5418E-20	Q9UKB3	DNAJC12

1	DNAJC13	0	-1.73	0	0	O75165	DNAJC13
1	DNAJC15	1.3388E-21	1.91	7.7886E-17	1.0527E-20	Q9Y5T4	DNAJC15
1	DNAJC18	1.0161E-62	2.92	5.9112E-58	1.3233E-61	Q9H819	DNAJC18
1	DNAJC21	0	-1.72	0	0	Q5F1R6	DNAJC21
1	DNAJC27-AS1	8.011E-07	-2.10	0.04660328	3.8672E-06		
1	DNAJC3-AS1	8.9739E-38	-2.80	5.2205E-33	9.1347E-37		
1	DNAJC4	0	-1.85	0	0	Q9NNZ3	DNAJC4
1	DNAJC5	5.2868E-69	1.77	3.0755E-64	7.1825E-68	Q9H3Z4	DNAJC5
1	DNAJC9	3.1469E-96	2.84	1.8307E-91	5.2742E-95	Q8WXX5	DNAJC9
1	DNAL1	1.326E-87	2.50	7.714E-83	2.0883E-86	Q4LDG9	DNAL1
1	DNAL4	2.9134E-14	-1.58	1.6949E-09	1.9227E-13	O96015	DNAL4
1	DNASE1L2	8.1648E-24	3.49	4.7498E-19	6.6993E-23	Q92874	DNASE1L2
1	DNASE1L3	0.00022342	-4.38	1	0.00091886	Q13609	DNASE1L3
1	DND1P1	0.00012081	2.45	1	0.00050693		
1	DNER	4.322E-121	97.38	2.514E-116	8.415E-120	Q8NFT8	DNER
1	DNHD1	1.3906E-07	-1.62	0.00808963	6.9943E-07	Q96M86	DNHD1
1	DNLZ	1.3275E-52	2.50	7.7229E-48	1.5923E-51	Q5SXM8	DNLZ
1	DNM1	0	-5.67	0	0	Q05193	DNM1
1	DNM1P28	0.00738517	-2.60	1	0.02635414		
1	DNM1P35	7.9063E-31	-3.50	4.5994E-26	7.3649E-30		
1	DNM1P46	0.00103855	23.85	1	0.00404072	Q6ZS02	DNM1P46
1	DNM1P51	3.0363E-06	3.01	0.176635	1.4152E-05		
1	DNM2	1.1826E-53	1.66	6.8797E-49	1.436E-52	P50570	DNM2
1	DNM3	1.08E-19	-2.63	6.2827E-15	8.1912E-19	Q9UQ16	DNM3
1	DNMBP	1.534E-132	5.71	8.924E-128	3.119E-131	Q6XZF7	DNMBP
1	DNMBP-AS1	0.00914832	17.64	1	0.03226986		
1	DNTTIP1	2.274E-100	2.63	1.3231E-95	3.917E-99	Q9H147	DNTTIP1
1	DNTTIP2	1.4798E-31	1.72	8.6086E-27	1.3919E-30	Q5QJE6	DNTTIP2
1	DOC2A	5.022E-118	18.48	2.921E-113	9.613E-117	Q14183	DOC2A
1	DOC2B	8.2117E-08	-8.18	0.00477709	4.1747E-07	Q14184	DOC2B
1	DOC2GP	6.9684E-09	-3.31	0.00040538	3.739E-08		
1	DOCK10	3.2679E-23	10.82	1.9011E-18	2.6496E-22	Q96BY6	DOCK10
1	DOCK11	2.1187E-60	5.94	1.2325E-55	2.7071E-59	Q5JSL3	DOCK11
1	DOCK2	1.101E-181	36.85	6.403E-177	2.609E-180	Q92608	DOCK2
1	DOCK3	2.748E-122	6.99	1.599E-117	5.381E-121	Q8IZD9	DOCK3
1	DOCK4	1.533E-191	19.63	8.915E-187	3.754E-190	Q8N1I0	DOCK4
1	DOCK6	0	-1.55	0	0	Q96HP0	DOCK6
1	DOCK8	5.2955E-15	-6.90	3.0806E-10	3.5721E-14	Q8NF50	DOCK8
1	DOCK9-AS2	7.4148E-07	-2.59	0.04313505	3.5865E-06		
1	DOHH	1.0057E-43	2.10	5.8508E-39	1.1066E-42	Q9BU89	DOHH
1	DOK1	1.8191E-24	1.69	1.0583E-19	1.512E-23	Q99704	DOK1
1	DOK3	5.7875E-42	3.15	3.3668E-37	6.221E-41	Q7L591	DOK3
1	DOK4	6.0386E-92	-3.14	3.5129E-87	9.8346E-91	Q8TEW6	DOK4
1	DOK7	4.9267E-98	-6.32	2.866E-93	8.3705E-97	Q18PE1	DOK7
1	DOLK	0	-1.87	0	0	Q9UPQ8	DOLK
1	DOPEY2	2.179E-27	2.00	1.2676E-22	1.9145E-26	Q9Y3R5	DOPEY2
1	DPCD	1.8524E-35	1.79	1.0776E-30	1.8374E-34	Q9BVM2	DPCD

1	DPF1	4.6502E-69	2.44	2.7052E-64	6.3206E-68	Q92782	DPF1
1	DPF3	8.769E-126	70.73	5.101E-121	1.74E-124	Q92784	DPF3
1	DPH2	2.1765E-55	1.74	1.2662E-50	2.682E-54	Q9BQC3	DPH2
1	DPH3	6.5562E-18	1.54	3.814E-13	4.7926E-17	Q96FX2	DPH3
1	DPH6-AS1	1.1756E-06	-2.01	0.06838774	5.6245E-06		
1	DPM1	7.7043E-28	2.82	4.4819E-23	6.8312E-27	O60762	DPM1
1	DPM3	0	-2.05	0	0	Q9P2X0	DPM3
1	DPP7	1.778E-136	11.16	1.034E-131	3.674E-135	Q9UHL4	DPP7
1	DPP9	9.5144E-42	2.08	5.5349E-37	1.0195E-40	Q86TI2	DPP9
1	DPPA2	5.104E-05	-10.75	1	0.00021989	Q7Z7J5	DPPA2
1	DPY19L1P1	0	-2.48	0	0		
1	DPY19L1P2	1.3773E-06	-2.30	0.08012497	6.5639E-06		
1	DPY19L2	1.09E-132	-9.21	6.342E-128	2.219E-131	Q6NUT2	DPY19L2
1	DPY19L2P1	5.7586E-75	3.26	3.35E-70	8.1968E-74	Q6NXN4	DPY19L2P1
1	DPY19L2P3	2.116E-11	-2.49	1.231E-06	1.2645E-10		
1	DPY19L2P4	0.00011751	-6.04	1	0.00049373		
1	DPY19L3	2.1152E-21	1.53	1.2305E-16	1.6565E-20	Q6ZPD9	DPY19L3
1	DPY19L4	0	-2.18	0	0	Q7Z388	DPY19L4
1	DPYD	2.5341E-89	3.16	1.4742E-84	4.0444E-88	Q12882	DPYD
1	DPYSL2	1.8713E-77	4.96	1.0886E-72	2.7134E-76	Q16555	DPYSL2
1	DPYSL3	0	203.18	0	0	Q14195	DPYSL3
1	DPYSL4	0.0118934	-7.01	1	0.04139315	O14531	DPYSL4
1	DQX1	2.2949E-72	-5.88	1.335E-67	3.2015E-71	Q8TE96	DQX1
1	DRAM1	6.076E-129	3.34	3.535E-124	1.221E-127	Q8N682	DRAM1
1	DRC3	2.6373E-07	-1.69	0.01534234	1.3061E-06	Q9H069	DRC3
1	DRC7	0.00026378	-7.45	1	0.00107684	Q8IY82	DRC7
1	DRD4	0.00071647	-1.69	1	0.00282519	P21917	DRD4
1	DRG1	6.3478E-72	2.35	3.6928E-67	8.8301E-71	Q9Y295	DRG1
1	DRICH1	1.2861E-09	3.43	7.4817E-05	7.1322E-09	Q6PGQ1	DRICH1
1	DROSHA	0	-1.64	0	0	Q9NRR4	DROSHA
1	DRP2	3.7314E-40	7.70	2.1707E-35	3.9105E-39	Q13474	DRP2
1	DSC1	1.26E-05	-48.37	0.73298454	5.654E-05	Q08554	DSC1
1	DSC2	0	-11.95	0	0	Q02487	DSC2
1	DSC3	0	-13.50	0	0	Q14574	DSC3
1	DSCAM	0.01294316	-4.02	1	0.04481078	O60469	DSCAM
1	DSCAS	2.7285E-12	-10.52	1.5873E-07	1.687E-11		
1	DSCR10	0.00573247	-21.64	1	0.02070536	P59022	DSCR10
1	DSCR4	2.2832E-83	-15.64	1.3282E-78	3.4779E-82	P56555	DSCR4
1	DSCR4-IT1	0.0001469	-5.94	1	0.00061231		
1	DSCR8	2.019E-117	-8.14	1.174E-112	3.854E-116	Q96T75	DSCR8
1	DSE	0	-8.24	0	0	Q9UL01	DSE
1	DSEL	8.9802E-67	5.29	5.2241E-62	1.2034E-65	Q8IZU8	DSEL
1	DSG1	2.4138E-31	-10.26	1.4042E-26	2.2645E-30	Q02413	DSG1
1	DSG1-AS1	8.721E-06	-12.14	0.50733596	3.9561E-05		
1	DSG2	0	-3.40	0	0	Q14126	DSG2
1	DSG2-AS1	2.2014E-10	-5.86	1.2806E-05	1.2617E-09		
1	DSG3	0	-9.63	0	0	P32926	DSG3

1	DSP	0	-10.23	0	0	P15924	DSP
1	DST	1.3189E-16	1.60	7.6728E-12	9.3765E-16	Q03001	DST
1	DTD1	3.4512E-53	1.84	2.0077E-48	4.1662E-52	Q8TEA8	DTD1
1	DTD2	4.8557E-55	2.25	2.8247E-50	5.9631E-54	Q96FN9	DTD2
1	DTHD1	3.6969E-11	-30.22	2.1506E-06	2.1849E-10	Q6ZMT9	DTHD1
1	DTNA	6.377E-153	-7.47	3.71E-148	1.382E-151	Q9Y4J8	DTNA
1	DTNB	0	-1.75	0	0	O60941	DTNB
1	DTNBP1	2.1501E-20	1.57	1.2508E-15	1.6506E-19	Q96EV8	DTNBP1
1	DTX2	0	-1.92	0	0	Q86UW9	DTX2
1	DTX2P1	1.7948E-10	-7.56	1.0441E-05	1.033E-09		
1	DTX2P1-UPK3	0	-2.58	0	0		
1	DTX3	9.573E-221	-9.05	5.569E-216	2.533E-219	Q8N9I9	DTX3
1	DTX3L	3.056E-41	-4.99	1.7778E-36	3.2501E-40	Q8TDB6	DTX3L
1	DTX4	1.0896E-58	-3.53	6.3388E-54	1.3768E-57	Q9Y2E6	DTX4
1	DUBR	4.1948E-14	1.58	2.4403E-09	2.7543E-13		
1	DUOX1	1.372E-182	-10.53	7.98E-178	3.255E-181	Q9NRD9	DUOX1
1	DUOX2	1.0456E-90	-38.57	6.0826E-86	1.6859E-89	Q9NRD8	DUOX2
1	DUOXA1	3.541E-108	-57.32	2.06E-103	6.404E-107	Q1HG43	DUOXA1
1	DUOXA2	9.9346E-13	-83.26	5.7793E-08	6.2324E-12	Q1HG44	DUOXA2
1	DUS3L	3.0709E-38	1.63	1.7865E-33	3.1463E-37	Q96G46	DUS3L
1	DUS4L	1.7764E-15	-1.59	1.0334E-10	1.2183E-14	O95620	DUS4L
1	DUSP1	2.6767E-73	9.73	1.5572E-68	3.7585E-72	P28562	DUSP1
1	DUSP10	3.0934E-88	-17.06	1.7996E-83	4.9022E-87	Q9Y6W6	DUSP10
1	DUSP13	4.7848E-05	-8.55	1	0.00020658	Q9UII6	DUSP13
1	DUSP18	1.6957E-36	2.79	9.8647E-32	1.7046E-35	Q8NEJ0	DUSP18
1	DUSP19	0.00045795	-1.60	1	0.00183515	Q8WTR2	DUSP19
1	DUSP23	3.4685E-28	8.16	2.0178E-23	3.098E-27	Q9BVJ7	DUSP23
1	DUSP3	1.1789E-40	1.56	6.8582E-36	1.2426E-39	P51452	DUSP3
1	DUSP4	0	20.95	0	0	Q13115	DUSP4
1	DUSP5P1	0.00023358	-1.87	1	0.00095917		
1	DUSP6	2.514E-115	4.34	1.462E-110	4.744E-114	Q16828	DUSP6
1	DUSP8	2.141E-19	4.88	1.2455E-14	1.6123E-18	Q13202	DUSP8
1	DUSP9	9.026E-16	-7.57	5.2508E-11	6.2413E-15	Q99956	DUSP9
1	DUX4L50	4.5883E-13	1.64	2.6692E-08	2.9108E-12		
1	DUXAP10	2.1409E-24	1.63	1.2454E-19	1.7767E-23		
1	DUXAP8_1	1.0695E-48	2.09	6.2219E-44	1.237E-47		
1	DVL1	9.5479E-84	1.87	5.5544E-79	1.4598E-82	O14640	DVL1
1	DXO	2.453E-32	1.77	1.427E-27	2.3351E-31	O77932	DXO
1	DYM	2.3312E-24	2.91	1.3561E-19	1.9332E-23	Q7RTS9	DYM
1	DYNC1H1	1.6901E-59	2.40	9.8321E-55	2.1472E-58	Q14204	DYNC1H1
1	DYNC1I1	3.659E-194	-9.72	2.129E-189	9.05E-193	O14576	DYNC1I1
1	DYNC1LI1	3.3729E-26	1.63	1.9621E-21	2.8927E-25	Q9Y6G9	DYNC1LI1
1	DYNLT3	1.2714E-12	-1.85	7.3962E-08	7.9435E-12	P51808	DYNLT3
1	DYRK4	6.137E-101	2.70	3.5701E-96	1.062E-99	Q9NR20	DYRK4
1	DYSF	4.8974E-27	-2.61	2.849E-22	4.2657E-26	O75923	DYSF
1	DYX1C1	2.192E-08	1.89	0.00127516	1.1499E-07		
1	DYX1C1-CCPG	3.1613E-10	5.91	1.8391E-05	1.7982E-09		

1	DZANK1	1.6341E-11	1.65	9.5063E-07	9.8196E-11	Q9NVP4	DZANK1
1	DZIP1	8.6752E-47	5.02	5.0467E-42	9.8453E-46	Q86YF9	DZIP1
1	DZIP3	0	-1.53	0	0	Q86Y13	DZIP3
1	E2F1	1.479E-135	3.08	8.606E-131	3.046E-134	Q01094	E2F1
1	E2F2	1.169E-108	3.22	6.799E-104	2.122E-107	Q14209	E2F2
1	E2F6P4	0.00089295	-3.35	1	0.00349222		
1	EAF2	4.1088E-21	2.66	2.3903E-16	3.2007E-20	Q96CJ1	EAF2
1	EAPP	3.0737E-36	1.74	1.7881E-31	3.0776E-35	Q56P03	EAPP
1	EARS2	2.1267E-87	1.95	1.2372E-82	3.3456E-86	Q5JPH6	EARS2
1	EBAG9	0	-1.51	0	0	O00559	EBAG9
1	EBF4	6.0608E-14	4.29	3.5258E-09	3.9598E-13	Q9BQW3	EBF4
1	EBLN3P	1.836E-115	2.23	1.068E-110	3.468E-114		
1	EBNA1BP2	2.773E-119	2.31	1.613E-114	5.349E-118	Q99848	EBNA1BP2
1	EBP	0	-4.05	0	0	Q15125	EBP
1	ECD	5.7548E-53	1.91	3.3478E-48	6.9298E-52	O95905	ECD
1	ECHDC1	9.3708E-49	1.85	5.4513E-44	1.084E-47	Q9NTX5	ECHDC1
1	ECHDC2	0	-1.65	0	0	Q86YB7	ECHDC2
1	ECHDC3	1.2043E-08	-10.75	0.00070058	6.3938E-08	Q96DC8	ECHDC3
1	ECM1	3.597E-210	-8.26	2.092E-205	9.299E-209	Q16610	ECM1
1	ECT2L	0.006752	-1.67	1	0.02417619	Q008S8	ECT2L
1	EDA	2.1571E-41	5.38	1.2549E-36	2.2996E-40	Q92838	EDA
1	EDAR	2.2047E-28	-3.51	1.2826E-23	1.9729E-27	Q9UNE0	EDAR
1	EDARADD	7.234E-113	-8.03	4.209E-108	1.348E-111	Q8WWZ3	EDARADD
1	EDEM3	0	-1.76	0	0	Q9BZQ6	EDEM3
1	EDF1	4.0027E-37	1.97	2.3285E-32	4.0454E-36	O60869	EDF1
1	EDIL3	0	-2.98	0	0	O43854	EDIL3
1	EDN1	1.0013E-74	3.38	5.8249E-70	1.4224E-73	P05305	EDN1
1	EDN2	2.5635E-08	-10.23	0.0014913	1.3403E-07	P20800	EDN2
1	EDNRA	2.8337E-65	-7.84	1.6485E-60	3.7619E-64	P25101	EDNRA
1	EDNRB	3.167E-147	-14.22	1.842E-142	6.766E-146	P24530	EDNRB
1	EEF1A1	1.2713E-15	1.56	7.3955E-11	8.751E-15	P68104	EEF1A1
1	EEF1A2	0	73.79	0	0	Q05639	EEF1A2
1	EEF1B2	2.249E-41	2.10	1.3083E-36	2.3966E-40	P24534	EEF1B2
1	EEF1DP3	0.01352393	4.55	1	0.04673802	Q658K8	EEF1DP3
1	EEF1E1	9.0647E-69	1.92	5.2733E-64	1.2297E-67	O43324	EEF1E1
1	EEF1E1P1	2.3368E-06	-4.06	0.13593896	1.0981E-05		
1	EEF1G	0	-1.52	0	0	P26641	EEF1G
1	EEF1GP1	4.4053E-06	-50.06	0.25627142	2.0316E-05		
1	EEF2	2.0445E-50	1.65	1.1893E-45	2.4013E-49	P13639	EEF2
1	EEF2KMT	3.1029E-27	1.87	1.8051E-22	2.7157E-26	Q96G04	EEF2KMT
1	EEPD1	1.1658E-62	-3.08	6.7817E-58	1.5171E-61	Q7L9B9	EEPD1
1	EFCAB1	7.0635E-22	-5.94	4.1091E-17	5.5808E-21	Q9HAE3	EFCAB1
1	EFCAB11	1.895E-50	2.17	1.1024E-45	2.227E-49	Q9BUY7	EFCAB11
1	EFCAB2	4.6222E-80	-3.02	2.6889E-75	6.863E-79	Q5VUJ9	EFCAB2
1	EFCAB5	4.1924E-21	-3.22	2.4389E-16	3.2649E-20	A4FU69	EFCAB5
1	EFCAB8	1.0254E-15	7.16	5.9651E-11	7.0811E-15	A8MWE9	EFCAB8
1	EFCAB9	0.00319607	6.78	1	0.01186222	A8MZ26	EFCAB9

1	EFEMP2	3.5896E-13	1.52	2.0882E-08	2.2842E-12	O95967	EFEMP2
1	EFHC2	1.7728E-13	-5.82	1.0313E-08	1.1408E-12	Q5JST6	EFHC2
1	EFHD1	1.7001E-14	10.23	9.89E-10	1.1298E-13	Q9BUP0	EFHD1
1	EFHD2	0	-2.42	0	0	Q96C19	EFHD2
1	EFNA1	3.372E-188	-3.97	1.962E-183	8.144E-187	P20827	EFNA1
1	EFNA5	4.1602E-58	1.72	2.4201E-53	5.2282E-57	P52803	EFNA5
1	EFNB1	0	-5.07	0	0	P98172	EFNB1
1	EFNB3	7.578E-21	4.72	4.4084E-16	5.8724E-20	Q15768	EFNB3
1	EFR3A	0	-1.96	0	0	Q14156	EFR3A
1	EFS	1.6886E-20	-11.95	9.8232E-16	1.2987E-19	O43281	EFS
1	EFTUD1P1	0.00017204	-36.00	1	0.00071346		
1	EFTUD1P2	4.6629E-08	-7.48	0.00271257	2.4028E-07		
1	EGF	1.1743E-30	-14.51	6.8314E-26	1.0911E-29	P01133	EGF
1	EGFEM1P	2.9729E-35	-6.23	1.7294E-30	2.9417E-34		
1	EGFL6	2.2372E-15	-14.58	1.3015E-10	1.5293E-14	Q8IUX8	EGFL6
1	EGFL7	3.48E-186	24.48	2.025E-181	8.356E-185	Q9UHF1	EGFL7
1	EGFL8	1.3514E-10	1.64	7.8618E-06	7.8188E-10	Q99944	EGFL8
1	EGFLAM	1.273E-208	85.90	7.403E-204	3.274E-207	Q63HQ2	EGFLAM
1	EGFR	0	-3.10	0	0	P00533	EGFR
1	EGFR-AS1	2.6075E-06	-3.82	0.15168918	1.221E-05		
1	EGLN3	7.495E-127	-5.16	4.36E-122	1.495E-125	Q9H6Z9	EGLN3
1	EGR1	0	-2.80	0	0	P18146	EGR1
1	EGR3	0.00056758	1.84	1	0.00225858	Q06889	EGR3
1	EHBP1L1	0	-1.82	0	0	Q8N3D4	EHBP1L1
1	EHD4	0	-1.59	0	0	Q9H223	EHD4
1	EHF	0	-15.50	0	0	Q9NZC4	EHF
1	EHHADH	0	-1.79	0	0	Q08426	EHHADH
1	EHMT2	1.2139E-74	1.55	7.0617E-70	1.7241E-73	Q96KQ7	EHMT2
1	EID2B	1.045E-10	1.74	6.0792E-06	6.0677E-10	Q96D98	EID2B
1	EID3	2.3544E-47	7.36	1.3697E-42	2.6851E-46	Q8N140	EID3
1	EIF1	4.2006E-21	1.52	2.4436E-16	3.2708E-20	P41567	EIF1
1	EIF1AX	1.7003E-53	2.06	9.8913E-49	2.0594E-52	P47813	EIF1AX
1	EIF1B	2.8649E-37	1.78	1.6666E-32	2.9015E-36	O60739	EIF1B
1	EIF1B-AS1	2.1631E-15	-2.51	1.2584E-10	1.4803E-14		
1	EIF2B2	1.08E-38	3.36	6.283E-34	1.1122E-37	P49770	EIF2B2
1	EIF2B3	2.9499E-27	1.64	1.7161E-22	2.5825E-26	Q9NR50	EIF2B3
1	EIF2B4	8.6968E-78	1.98	5.0593E-73	1.2642E-76	Q9UI10	EIF2B4
1	EIF2D	0	-1.61	0	0	P41214	EIF2D
1	EIF2S1	4.9989E-28	2.96	2.9081E-23	4.4548E-27	P05198	EIF2S1
1	EIF2S2	1.2612E-27	1.58	7.3368E-23	1.1137E-26	P20042	EIF2S2
1	EIF3C	1.0937E-78	2.11	6.3625E-74	1.6026E-77	Q99613	EIF3C
1	EIF3CL	1.077E-08	1.88	0.00062655	5.7292E-08	B5ME19	EIF3CL
1	EIF3F	8.596E-81	2.19	5.0006E-76	1.2839E-79	O00303	EIF3F
1	EIF3G	4.454E-118	2.27	2.591E-113	8.529E-117	O75821	EIF3G
1	EIF3I	8.0185E-79	2.33	4.6647E-74	1.1785E-77	Q13347	EIF3I
1	EIF3L	4.4834E-34	1.79	2.6082E-29	4.3813E-33	Q9Y262	EIF3L
1	EIF3M	9.6027E-18	1.57	5.5863E-13	6.9968E-17	Q7L2H7	EIF3M

1	EIF4A1	5.2277E-95	2.52	3.0411E-90	8.7014E-94	P60842	EIF4A1
1	EIF4A3	1.1245E-66	1.98	6.5416E-62	1.5056E-65	P38919	EIF4A3
1	EIF4E3	7.946E-11	2.23	4.6225E-06	4.6364E-10	Q8N5X7	EIF4E3
1	EIF4EBP3	2.4321E-05	-5.97	1	0.00010711	O60516	EIF4EBP3
1	EIF4G2	3.8797E-97	2.65	2.2569E-92	6.54E-96	P78344	EIF4G2
1	EIF5	8.1416E-69	3.09	4.7363E-64	1.1051E-67	P55010	EIF5
1	EIF6	2.3959E-40	1.80	1.3938E-35	2.5177E-39	P56537	EIF6
1	ELAVL2	1.7445E-09	-5.75	0.00010149	9.615E-09	Q12926	ELAVL2
1	ELAVL3	0.00436115	-8.80	1	0.0159734	Q14576	ELAVL3
1	ELDR	1.9925E-11	-5.57	1.1591E-06	1.1925E-10		
1	ELF1	0	-2.42	0	0	P32519	ELF1
1	ELF3	0	-13.58	0	0	P78545	ELF3
1	ELF3-AS1	2.0054E-20	-3.81	1.1666E-15	1.5405E-19		
1	ELF4	0	-2.31	0	0	Q99607	ELF4
1	ELF5	9.145E-167	-23.83	5.32E-162	2.073E-165	Q9UKW6	ELF5
1	ELFN1	2.6111E-26	-4.98	1.519E-21	2.243E-25	P0C7U0	ELFN1
1	ELFN2_1	0.0135886	2.44	1	0.04695037		
1	ELFN2_2	1.062E-155	22.08	6.177E-151	2.327E-154		
1	ELK3	2.6803E-62	1.92	1.5592E-57	3.4773E-61	P41970	ELK3
1	ELK4	0	-2.06	0	0	P28324	ELK4
1	ELL2	6.5219E-81	5.56	3.794E-76	9.7558E-80	O00472	ELL2
1	ELL3	2.601E-143	-5.04	1.513E-138	5.516E-142	Q9HB65	ELL3
1	ELMO1	0	-9.42	0	0	Q92556	ELMO1
1	ELMO2	1.5787E-24	2.64	9.1837E-20	1.3146E-23	Q96JJ3	ELMO2
1	ELMO3	0	-5.73	0	0	Q96BJ8	ELMO3
1	ELMOD1	0.00162157	2.44	1	0.00619311	Q8N336	ELMOD1
1	ELN	1.5432E-26	-30.45	8.9776E-22	1.3306E-25	P15502	ELN
1	ELOA	1.1722E-49	1.55	6.8192E-45	1.3682E-48	Q14241	ELOA
1	ELOA-AS1	1.3198E-05	-1.50	0.76779361	5.9152E-05		
1	ELOA2	0.00577568	-19.67	1	0.02085369	Q8IYF1	ELOA2
1	ELOF1	1.4808E-48	2.08	8.6143E-44	1.7102E-47	P60002	ELOF1
1	ELOVL3	4.3208E-07	3.68	0.02513563	2.116E-06	Q9HB03	ELOVL3
1	ELOVL4	3.965E-144	-3.67	2.306E-139	8.417E-143	Q9GZR5	ELOVL4
1	ELOVL5	0	-2.74	0	0	Q9NYP7	ELOVL5
1	ELOVL6	0	-2.25	0	0	Q9H5J4	ELOVL6
1	ELOVL7	0	-1.66	0	0	A1L3X0	ELOVL7
1	ELP6	3.475E-55	1.65	2.0216E-50	4.273E-54	Q0PNE2	ELP6
1	EMBP1	3.7314E-64	-4.78	2.1707E-59	4.9122E-63		
1	EMC10	0	-1.66	0	0	Q5UCC4	EMC10
1	EMC2	0	-2.15	0	0	Q15006	EMC2
1	EMC3-AS1	2.2044E-06	-1.67	0.12823952	1.0379E-05		
1	EMC9	4.2976E-64	2.07	2.5001E-59	5.655E-63	Q9Y3B6	EMC9
1	EMD	2.458E-107	2.24	1.43E-102	4.421E-106	P50402	EMD
1	EMG1_1	6.1095E-54	1.73	3.5542E-49	7.4401E-53		
1	EMG1_2	8.5953E-60	3.58	5.0002E-55	1.0949E-58		
1	EMID1	1.897E-28	-9.67	1.1036E-23	1.6991E-27	Q96A84	EMID1
1	EMILIN2	9.4908E-19	-3.85	5.5212E-14	7.0604E-18	Q9BXX0	EMILIN2

1	EML1	0.00978387	2.43	1	0.0343451	O00423	EML1
1	EML2	0	-2.01	0	0	O95834	EML2
1	EML3	0	-1.99	0	0	Q32P44	EML3
1	EML5	6.8969E-83	6.26	4.0122E-78	1.0478E-81	Q05BV3	EML5
1	EMP2	9.556E-168	5.80	5.559E-163	2.173E-166	P54851	EMP2
1	EMP3	7.395E-61	6.07	4.302E-56	9.4778E-60	P54852	EMP3
1	EMX1	1.5305E-10	-2.85	8.9036E-06	8.833E-10	Q04741	EMX1
1	EN1	3.703E-148	195.02	2.154E-143	7.939E-147	Q05925	EN1
1	ENAH	0	-2.61	0	0	Q8N8S7	ENAH
1	ENDOD1	0	-3.03	0	0	O94919	ENDOD1
1	ENDOG	9.9463E-06	-1.62	0.57861743	4.4931E-05	Q14249	ENDOG
1	ENDOU	7.1355E-08	-12.44	0.004151	3.6387E-07	P21128	ENDOU
1	ENG	3.7032E-32	1.81	2.1543E-27	3.5155E-31	P17813	ENG
1	ENHO	1.332E-110	38.80	7.749E-106	2.448E-109	Q6UWT2	ENHO
1	ENKD1	4.2582E-48	1.98	2.4771E-43	4.9014E-47	Q9H0I2	ENKD1
1	ENKUR	9.513E-30	-3.37	5.5341E-25	8.7178E-29	Q8TC29	ENKUR
1	ENO1	4.2155E-96	2.43	2.4523E-91	7.0611E-95	P06733	ENO1
1	ENO2	5.214E-144	2.90	3.033E-139	1.107E-142	P09104	ENO2
1	ENO3	2.8445E-09	1.69	0.00016548	1.5542E-08	P13929	ENO3
1	ENOX2	0	-1.80	0	0	Q16206	ENOX2
1	ENPEP	1.431E-135	-8.81	8.322E-131	2.947E-134	Q07075	ENPEP
1	ENPP4	3.5759E-19	1.79	2.0803E-14	2.6825E-18	Q9Y6X5	ENPP4
1	ENPP5	6.416E-197	-15.70	3.733E-192	1.605E-195	Q9UJA9	ENPP5
1	ENPP6	2.5372E-08	-15.01	0.00147597	1.3268E-07	Q6UWR7	ENPP6
1	ENPP7P1	1.2401E-06	9.81	0.07214426	5.9261E-06		
1	ENPP7P6	7.4175E-07	3.97	0.04315041	3.5875E-06		
1	ENPP7P7	5.2682E-36	-11.00	3.0647E-31	5.2577E-35		
1	ENPP7P8	0.00172484	-2.34	1	0.00656898		
1	ENSA	0	-1.63	0	0	O43768	ENSA
1	ENTPD1	1.0406E-12	1.68	6.0537E-08	6.5234E-12	P49961	ENTPD1
1	ENTPD1-AS1	4.0873E-15	1.53	2.3778E-10	2.7697E-14		
1	ENTPD2	9.7273E-17	-4.83	5.6588E-12	6.9399E-16	Q9Y5L3	ENTPD2
1	ENTPD3	6.9123E-27	-5.47	4.0212E-22	5.9946E-26	O75355	ENTPD3
1	ENTPD4	0	-1.79	0	0	Q9Y227	ENTPD4
1	ENTPD6	3.1001E-60	1.62	1.8034E-55	3.9584E-59	O75354	ENTPD6
1	ENTPD7	4.4878E-66	2.62	2.6108E-61	5.9907E-65	Q9NQZ7	ENTPD7
1	ENTPD8	1.1137E-32	-5.33	6.4787E-28	1.0663E-31	Q5MY95	ENTPD8
1	EOMES	1.1207E-36	-10.69	6.5196E-32	1.1282E-35	O95936	EOMES
1	EP300-AS1	0.00105319	1.71	1	0.0040941		
1	EPAS1	0	-1.62	0	0	Q99814	EPAS1
1	EPB41L1	5.8162E-78	-3.35	3.3835E-73	8.4693E-77	Q9H4G0	EPB41L1
1	EPB41L2	4.3015E-39	3.02	2.5024E-34	4.4566E-38	O43491	EPB41L2
1	EPB41L3	1.2019E-29	17.60	6.9922E-25	1.0985E-28	Q9Y2J2	EPB41L3
1	EPB41L4A	0.00055789	-1.55	1	0.00222218	Q9HCS5	EPB41L4A
1	EPB41L4A-AS1	1.827E-33	1.81	1.0628E-28	1.7678E-32		
1	EPB41L4B	1.6305E-78	-2.71	9.4851E-74	2.3856E-77	Q9H329	EPB41L4B
1	EPC2	7.1914E-24	1.64	4.1835E-19	5.9056E-23	Q52LR7	EPC2

1	EPCAM	0	-11.34	0	0	P16422	EPCAM
1	EPDR1	1.999E-104	-11.57	1.163E-99	3.543E-103	Q9UM22	EPDR1
1	EPGN	0.01260342	-2.82	1	0.04369435	Q6UW88	EPGN
1	EPHA1	0	-13.69	0	0	P21709	EPHA1
1	EPHA3	3.617E-32	-6.99	2.1042E-27	3.4348E-31	P29320	EPHA3
1	EPHA4	5.9476E-08	3.53	0.00345993	3.0471E-07	P54764	EPHA4
1	EPHA5-AS1	0.00463117	-8.21	1	0.01691554		
1	EPHA6	2.01E-153	-14.30	1.169E-148	4.368E-152	Q9UF33	EPHA6
1	EPHA7	1.821E-114	-7.47	1.059E-109	3.418E-113	Q15375	EPHA7
1	EPHA8	0.00166194	-7.09	1	0.00634186	P29322	EPHA8
1	EPHB1	5.5736E-16	3.39	3.2424E-11	3.8882E-15	P54762	EPHB1
1	EPHB3	1.998E-100	-4.62	1.1624E-95	3.444E-99	P54753	EPHB3
1	EPHB4	0	-2.04	0	0	P54760	EPHB4
1	EPHB6	1.0289E-26	4.41	5.9857E-22	8.8993E-26	O15197	EPHB6
1	EPHX1	0	-3.94	0	0	P07099	EPHX1
1	EPHX2	6.732E-194	-14.41	3.916E-189	1.662E-192	P34913	EPHX2
1	EPHX4	1.3163E-41	-5.16	7.6575E-37	1.4079E-40	Q8IUS5	EPHX4
1	EPN3	4.9395E-82	-8.62	2.8735E-77	7.4539E-81	Q9H201	EPN3
1	EPOP	6.4106E-22	1.87	3.7293E-17	5.0691E-21	A6NHQ4	EPOP
1	EPPK1	7.509E-150	-8.97	4.369E-145	1.616E-148	P58107	EPPK1
1	EPRS	0	-1.72	0	0	P07814	EPRS
1	EPS8	3.2928E-37	2.99	1.9156E-32	3.332E-36	Q12929	EPS8
1	EPS8L1	2.951E-107	-14.29	1.717E-102	5.305E-106	Q8TE68	EPS8L1
1	EPS8L2	0	-1.79	0	0	Q9H6S3	EPS8L2
1	EPSTI1	1.3325E-13	-15.67	7.7517E-09	8.6092E-13		
1	EPX	0.00066581	-2.81	1	0.00263185	P11678	EPX
1	ERAP1	0	-3.39	0	0	Q9NZ08	ERAP1
1	ERAP2	5.9623E-66	-3.17	3.4685E-61	7.9534E-65	Q6P179	ERAP2
1	ERBB2	0	-1.59	0	0	P04626	ERBB2
1	ERBB3	0	-11.96	0	0	P21860	ERBB3
1	ERBB4	4.4459E-05	-2.07	1	0.00019242	Q15303	ERBB4
1	ERBIN	4.9896E-27	1.51	2.9027E-22	4.3453E-26	Q96RT1	ERBIN
1	ERC1	2.3629E-41	2.39	1.3746E-36	2.5172E-40	Q8IUD2	ERC1
1	ERCC2	7.3101E-90	2.22	4.2526E-85	1.1725E-88	P18074	ERCC2
1	ERCC4	5.1622E-49	2.04	3.0031E-44	5.987E-48	Q92889	ERCC4
1	ERCC5	0	-1.98	0	0	P28715	ERCC5
1	ERCC6L	3.0404E-50	2.06	1.7687E-45	3.566E-49	Q2NKX8	ERCC6L
1	ERFE	3.3686E-24	2.26	1.9597E-19	2.7836E-23	Q4G0M1	ERFE
1	ERG	0.00015724	-5.78	1	0.00065374	P11308	ERG
1	ERGIC1	1.0278E-15	2.32	5.9793E-11	7.0971E-15	Q969X5	ERGIC1
1	ERH	6.9645E-91	2.52	4.0515E-86	1.1245E-89	P84090	ERH
1	ERICD	1.4739E-16	4.11	8.5743E-12	1.0462E-15		
1	ERICH1	0	-1.97	0	0		
1	ERICH2	2.7743E-12	-8.14	1.6139E-07	1.7146E-11	A1L162	ERICH2
1	ERICH3	5.1207E-06	-6.49	0.29788976	2.3534E-05		
1	ERICH3-AS1	1.9725E-08	-7.69	0.00114751	1.0374E-07		
1	ERICH5	1.4905E-05	-4.17	0.86708642	6.6581E-05		

1	ERICH6	1.1956E-07	-2.88	0.00695533	6.0313E-07		
1	ERICH6-AS1	5.5504E-16	-2.44	3.2289E-11	3.8725E-15		
1	ERLIN2	0	-1.59	0	0	O94905	ERLIN2
1	ERMAP	0	-2.04	0	0	Q96PL5	ERMAP
1	ERMN	1.3234E-21	-59.88	7.6989E-17	1.0407E-20	Q8TAM6	ERMN
1	ERMP1	0	-2.94	0	0	Q7Z2K6	ERMP1
1	ERO1A	0	-1.56	0	0	Q96HE7	ERO1A
1	ERP44	0	-2.34	0	0	Q9BS26	ERP44
1	ERRFI1	2.46E-211	15.54	1.431E-206	6.377E-210	Q9UJM3	ERRFI1
1	ERV3-1	1.123E-19	-1.73	6.5331E-15	8.5133E-19	Q14264	ERV3-1
1	ERVH48-1	0.00012579	-3.37	1	0.00052693	M5A8F1	ERVH48-1
1	ERVMER34-1	2.792E-37	-9.04	1.6242E-32	2.8281E-36	Q9H9K5	ERVMER34-1
1	ERVW-1	0.00416957	-3.52	1	0.01531025	Q9UQF0	ERVW-1
1	ESAM	3.4373E-07	-2.14	0.01999613	1.6911E-06	Q96AP7	ESAM
1	ESM1	6.536E-226	32.15	3.802E-221	1.745E-224	Q9NQ30	ESM1
1	ESPN	2.365E-217	-7.86	1.376E-212	6.224E-216	B1AK53	ESPN
1	ESPNL	4.2797E-05	-3.21	1	0.00018535	Q6ZVH7	ESPNL
1	ESPNP	5.3651E-25	-14.31	3.1211E-20	4.5018E-24		
1	ESRP1	0	-12.02	0	0	Q6NXG1	ESRP1
1	ESRP2	0	-10.75	0	0	Q9H6T0	ESRP2
1	ESRRA	0	-2.39	0	0	P11474	ESRRA
1	ESRRAP2	2.4357E-08	-3.33	0.00141697	1.2747E-07		
1	ESRRB	5.522E-155	17.99	3.212E-150	1.207E-153	O95718	ESRRB
1	ESRRG	1.8396E-14	-5.28	1.0702E-09	1.2218E-13	P62508	ESRRG
1	ESYT3	7.9225E-15	-9.76	4.6088E-10	5.3189E-14	A0FGR9	ESYT3
1	ETF1	2.7242E-73	1.88	1.5848E-68	3.8243E-72	P62495	ETF1
1	ETFB	3.9414E-36	1.56	2.2929E-31	3.9403E-35	P38117	ETFB
1	ETNK1	2.5729E-64	2.56	1.4968E-59	3.391E-63	Q9HBU6	ETNK1
1	ETNPPL	1.8969E-05	-49.35	1	8.4095E-05	Q8TBG4	ETNPPL
1	ETS1	3.3604E-22	2.56	1.9549E-17	2.671E-21	P14921	ETS1
1	ETS2	0	-2.27	0	0	P15036	ETS2
1	ETV1	2.063E-80	10.28	1.2002E-75	3.0757E-79	P50549	ETV1
1	ETV2	2.7886E-08	2.49	0.00162225	1.4549E-07	O00321	ETV2
1	ETV3	0	-1.50	0	0	P41162	ETV3
1	ETV4	1.2632E-86	4.03	7.3487E-82	1.9755E-85	P43268	ETV4
1	ETV5	9.358E-167	28.82	5.444E-162	2.121E-165	P41161	ETV5
1	ETV6	7.9811E-61	1.81	4.6429E-56	1.0227E-59	P41212	ETV6
1	ETV7	3.3732E-19	-4.44	1.9623E-14	2.5317E-18	Q9Y603	ETV7
1	EVA1A	7.44E-188	61.08	4.328E-183	1.795E-186	Q9H8M9	EVA1A
1	EVA1C	2.7483E-71	9.67	1.5988E-66	3.8021E-70	P58658	EVA1C
1	EVC	5.216E-173	5.13	3.034E-168	1.205E-171	P57679	EVC
1	EVI2B	0.01082422	2.87	1	0.03783275	P34910	EVI2B
1	EVI5	1.2577E-52	2.07	7.3167E-48	1.5092E-51	O60447	EVI5
1	EVL	0	112.62	0	0	Q9UI08	EVL
1	EVPL	0	-2.25	0	0	Q92817	EVPL
1	EVPLL	0.00102621	-23.44	1	0.00399591		
1	EXO1	2.059E-47	1.77	1.1978E-42	2.3514E-46	Q9UQ84	EXO1

1	EXOC1	0	-1.58	0	0	Q9NV70	EXOC1
1	EXOC3	0	-2.44	0	0	O60645	EXOC3
1	EXOC3-AS1	1.9603E-22	-2.65	1.1404E-17	1.5654E-21	Q8N2X6	EXOC3-AS1
1	EXOC5	1.0588E-22	1.59	6.1597E-18	8.5103E-22	O00471	EXOC5
1	EXOC6	6.0964E-43	1.85	3.5465E-38	6.6364E-42	Q8TAG9	EXOC6
1	EXOSC3	1.5414E-58	1.99	8.9668E-54	1.9451E-57	Q9NQT5	EXOSC3
1	EXOSC4	7.1362E-23	1.56	4.1514E-18	5.7531E-22	Q9NPD3	EXOSC4
1	EXPH5	3.0534E-84	-3.99	1.7763E-79	4.6929E-83	Q8NEV8	EXPH5
1	EXTL3	0	-1.92	0	0	O43909	EXTL3
1	EXTL3-AS1	1.809E-30	-6.06	1.0524E-25	1.6755E-29		
1	EYA4	1.0019E-38	-7.97	5.8287E-34	1.032E-37	O95677	EYA4
1	EYS	2.536E-137	-3.99	1.475E-132	5.261E-136	Q5T1H1	EYS
1	F11-AS1	0.0046821	-8.26	1	0.01709191		
1	F11R	0	-12.13	0	0	Q9Y624	F11R
1	F12	5.7131E-11	1.88	3.3235E-06	3.3493E-10	P00748	F12
1	F13A1	0.00088673	-5.71	1	0.00346857	P00488	F13A1
1	F2R	1.6941E-82	12.20	9.8552E-78	2.5678E-81	P25116	F2R
1	F2RL2	3.8712E-45	40.50	2.252E-40	4.3175E-44	O00254	F2RL2
1	F3	0	-2.55	0	0	P13726	F3
1	F5	0.00042442	-7.09	1	0.00170477	P12259	F5
1	F8A1	1.3486E-42	1.99	7.8452E-38	1.4618E-41	P23610	F8A1
1	F8A3	1.829E-34	-5.78	1.064E-29	1.7942E-33		
1	FA2H	1.7563E-36	-15.37	1.0217E-31	1.7649E-35	Q7L5A8	FA2H
1	FAAH	1.5431E-41	-2.35	8.9765E-37	1.6474E-40	O00519	FAAH
1	FAAH2	6.029E-145	-12.82	3.507E-140	1.283E-143	Q6GMR7	FAAH2
1	FAAHP1	0.00200526	-3.06	1	0.00758579		
1	FAAP100	9.8195E-91	1.93	5.7124E-86	1.5837E-89	Q0VG06	FAAP100
1	FAAP20	6.2445E-89	3.45	3.6327E-84	9.9498E-88	Q6NZ36	FAAP20
1	FAAP24	4.268E-24	2.18	2.4828E-19	3.5163E-23	Q9BTP7	FAAP24
1	FABP3	1.4622E-16	-2.15	8.5063E-12	1.0384E-15	P05413	FABP3
1	FABP4	5.4729E-20	1.94	3.1838E-15	4.1733E-19	P15090	FABP4
1	FABP5	9.1209E-77	4.78	5.306E-72	1.3166E-75	Q01469	FABP5
1	FABP6	3.5415E-09	-14.36	0.00020602	1.926E-08	P51161	FABP6
1	FADS1	0	-2.07	0	0	O60427	FADS1
1	FADS2	0	-1.52	0	0	O95864	FADS2
1	FADS3	0	-4.68	0	0	Q9Y5Q0	FADS3
1	FAHD1	4.0659E-62	2.10	2.3653E-57	5.2703E-61	Q6P587	FAHD1
1	FAHD2B	5.7297E-18	-1.75	3.3332E-13	4.1927E-17	Q6P2I3	FAHD2B
1	FAHD2CP	1.0635E-24	-2.46	6.1868E-20	8.875E-24		
1	FAIM	0	-1.68	0	0	Q9NVQ4	FAIM
1	FALEC	0.0015161	-2.29	1	0.00580475		
1	FAM102B	1.4117E-29	1.84	8.2123E-25	1.289E-28		
1	FAM104A	8.7306E-39	1.62	5.079E-34	9.0021E-38	Q969W3	FAM104A
1	FAM105A	2.3049E-65	-2.96	1.3408E-60	3.0633E-64		
1	FAM107B	5.0552E-14	1.56	2.9408E-09	3.3102E-13		
1	FAM109A	5.125E-63	2.97	2.9814E-58	6.6819E-62	Q8N4B1	FAM109A
1	FAM109B	1.0551E-40	-7.15	6.1379E-36	1.1129E-39	Q6ICB4	FAM109B

1	FAM110A	0	-1.59	0	0	Q9BQ89	FAM110A
1	FAM110C	3.0265E-59	-8.14	1.7607E-54	3.8358E-58	Q1W6H9	FAM110C
1	FAM111A	0	-1.52	0	0	Q96PZ2	FAM111A
1	FAM111B	2.5651E-39	1.92	1.4922E-34	2.6623E-38	Q6SJ93	FAM111B
1	FAM114A1	6.067E-37	1.66	3.5294E-32	6.1211E-36	Q8IWE2	FAM114A1
1	FAM117B	1.5027E-10	1.56	8.742E-06	8.6744E-10	Q6P1L5	FAM117B
1	FAM118A	5.62E-20	1.59	3.2694E-15	4.2837E-19	Q9NWS6	FAM118A
1	FAM118B	5.449E-28	1.87	3.1699E-23	4.8514E-27	Q9BPY3	FAM118B
1	FAM122B	2.3694E-19	1.53	1.3784E-14	1.7829E-18		
1	FAM124A	1.1264E-19	4.79	6.5527E-15	8.5377E-19	Q86V42	FAM124A
1	FAM127A	7.1411E-93	2.92	4.1543E-88	1.1702E-91		
1	FAM127B	2.4135E-39	1.72	1.404E-34	2.5063E-38		
1	FAM127C	1.105E-132	78.45	6.426E-128	2.248E-131		
1	FAM129A	0	-4.26	0	0	Q9BZQ8	FAM129A
1	FAM131B	1.1268E-05	-2.97	0.65552627	5.0716E-05	Q86XD5	FAM131B
1	FAM131C	4.9289E-25	-4.86	2.8673E-20	4.1393E-24	Q96AQ9	FAM131C
1	FAM133A	0.00067952	2.05	1	0.00268368	Q8N9E0	FAM133A
1	FAM135A	0	-3.00	0	0	Q9P2D6	FAM135A
1	FAM136A	6.3286E-48	2.50	3.6816E-43	7.2673E-47	Q96C01	FAM136A
1	FAM13A	1.4933E-86	-3.36	8.6874E-82	2.3316E-85	O94988	FAM13A
1	FAM13B	3.2398E-35	1.63	1.8847E-30	3.2031E-34	Q9NYF5	FAM13B
1	FAM149A	4.8411E-06	-2.22	0.28162344	2.2272E-05		
1	FAM151B	1.2838E-18	3.66	7.4684E-14	9.52E-18	Q6UXP7	FAM151B
1	FAM153B	0.00536232	3.99	1	0.01944204		
1	FAM156A	5.7389E-23	2.45	3.3386E-18	4.6324E-22	Q8NDB6	FAM156A
1	FAM157A	6.967E-70	-6.56	4.053E-65	9.5296E-69		
1	FAM157C	0	-4.19	0	0		
1	FAM159B	0.00070778	-3.04	1	0.00279109	A6NKW6	FAM159B
1	FAM160A1	2.1816E-56	-5.19	1.2691E-51	2.7072E-55		
1	FAM160B2	0	-2.93	0	0		
1	FAM161A	5.635E-17	1.74	3.2781E-12	4.0366E-16	Q3B820	FAM161A
1	FAM161B	1.206E-106	3.90	7.013E-102	2.161E-105		
1	FAM162B	2.382E-09	-8.19	0.00013857	1.306E-08	Q5T6X4	FAM162B
1	FAM167A	4.1053E-05	2.11	1	0.00017801	Q96KS9	FAM167A
1	FAM167B	6.6064E-14	-2.99	3.8432E-09	4.3104E-13		
1	FAM169A	1.891E-23	1.78	1.1001E-18	1.54E-22	Q9Y6X4	FAM169A
1	FAM171A1	0	133.24	0	0	Q5VUB5	FAM171A1
1	FAM171A2	8.0428E-31	2.39	4.6788E-26	7.4909E-30	A8MVW0	FAM171A2
1	FAM173A	3.0885E-06	1.51	0.17967077	1.439E-05	Q9BQD7	FAM173A
1	FAM173B	0	-1.72	0	0	Q6P4H8	FAM173B
1	FAM174B	2.76E-99	-3.68	1.6055E-94	4.7289E-98	Q3ZCQ3	FAM174B
1	FAM175A	2.4938E-19	1.70	1.4508E-14	1.8758E-18		
1	FAM180B	3.6349E-05	-5.06	1	0.00015833	Q6P0A1	FAM180B
1	FAM181A	7.2204E-29	-5.03	4.2004E-24	6.5213E-28		
1	FAM181A-AS1	0.00099294	-4.31	1	0.00387154		
1	FAM182B	3.638E-200	23.03	2.116E-195	9.177E-199		
1	FAM183A	1.8539E-13	-2.64	1.0785E-08	1.1924E-12		

1	FAM184A	0.00064072	-1.63	1	0.00253802	Q8NB25	FAM184A
1	FAM185BP	3.6659E-06	-3.34	0.21326282	1.6989E-05		
1	FAM186A	0.0054627	-1.94	1	0.01978503		
1	FAM186B	0.00378367	-2.20	1	0.01393549	Q8IYM0	FAM186B
1	FAM187A	4.7225E-05	-1.53	1	0.000204	A6NFU0	FAM187A
1	FAM196B	1.526E-187	38.36	8.875E-183	3.675E-186		
1	FAM198B	2.393E-139	-13.31	1.392E-134	5.006E-138	Q6UWH4	FAM198B
1	FAM19A2	1.3736E-06	-1.62	0.07990668	6.547E-06	Q8N3H0	FAM19A2
1	FAM201A	3.488E-236	90.52	2.029E-231	9.41E-235		
1	FAM207A	5.8846E-93	2.39	3.4233E-88	9.654E-92	Q9NSI2	FAM207A
1	FAM20B	0	-1.71	0	0	O75063	FAM20B
1	FAM20C	0	341.88	0	0	Q8IXL6	FAM20C
1	FAM210B	1.1686E-90	2.19	6.7983E-86	1.8837E-89	Q96KR6	FAM210B
1	FAM212A	3.7661E-22	-2.50	2.1909E-17	2.9885E-21	Q96EL1	FAM212A
1	FAM212B-AS1	0.00962107	-3.94	1	0.03381238		
1	FAM213A	0	-6.95	0	0	Q9BRX8	FAM213A
1	FAM213B	1.6746E-91	6.15	9.7419E-87	2.7189E-90	Q8TBF2	FAM213B
1	FAM214A	0	-1.56	0	0	Q32MH5	FAM214A
1	FAM214B	2.5445E-22	1.65	1.4802E-17	2.0269E-21	Q7L5A3	FAM214B
1	FAM216A	2.6268E-33	1.82	1.5281E-28	2.5359E-32		
1	FAM217B	1.2494E-48	2.04	7.2685E-44	1.4439E-47	Q9NTX9	FAM217B
1	FAM218A	9.9581E-09	-7.81	0.0005793	5.3069E-08	Q96MZ4	FAM218A
1	FAM219A	2.6415E-57	2.22	1.5367E-52	3.2961E-56		
1	FAM220CP	1.197E-06	-4.97	0.0696362	5.7248E-06		
1	FAM221A	1.4601E-22	2.72	8.4937E-18	1.1711E-21		
1	FAM222A	2.6064E-26	3.31	1.5162E-21	2.2393E-25		
1	FAM222A-AS1	0.00226732	-3.03	1	0.00852558		
1	FAM223A	0.00194318	-3.21	1	0.00736243		
1	FAM225A	5.9738E-34	3.63	3.4752E-29	5.8269E-33		
1	FAM227A	2.5281E-26	2.19	1.4707E-21	2.1727E-25		
1	FAM227B	9.9617E-15	-2.18	5.7951E-10	6.6664E-14		
1	FAM228B	7.3872E-18	-1.88	4.2974E-13	5.3933E-17		
1	FAM229A	2.8625E-33	2.55	1.6652E-28	2.7602E-32		
1	FAM230A	3.1826E-07	-11.08	0.01851443	1.5685E-06		
1	FAM230C	4.262E-149	-6.68	2.479E-144	9.155E-148		
1	FAM234A	0	-2.45	0	0	Q9H0X4	FAM234A
1	FAM24B	1.8589E-98	-4.67	1.0814E-93	3.1676E-97	Q8N5W8	FAM24B
1	FAM25A	0.01436522	-2.47	1	0.04952486		
1	FAM25BP	0.0035383	-23.18	1	0.01307236		
1	FAM26F	3.499E-06	-11.06	0.20354879	1.6235E-05	Q5R3K3	FAM26F
1	FAM27C	1.3591E-71	4.54	7.9066E-67	1.8848E-70		
1	FAM27E3	2.003E-153	3.75	1.165E-148	4.354E-152	Q08E93	FAM27E3
1	FAM32A	2.7656E-82	2.12	1.6089E-77	4.18E-81	Q9Y421	FAM32A
1	FAM35A	2.4085E-71	2.23	1.4011E-66	3.3328E-70		
1	FAM35BP	3.6291E-07	-2.63	0.02111208	1.784E-06		
1	FAM35DP	0.00019424	2.46	1	0.00080322		
1	FAM3C	0	-4.70	0	0	Q92520	FAM3C

1	FAM3C2	0	-3.53	0	0		
1	FAM43A	1.8883E-59	3.80	1.0985E-54	2.3979E-58		
1	FAM46A	4.5653E-80	2.59	2.6558E-75	6.782E-79	Q96IP4	FAM46A
1	FAM46B	1.3395E-56	-6.48	7.7921E-52	1.6639E-55	Q96A09	FAM46B
1	FAM46C	0.00415452	-1.84	1	0.01525884	Q5VWP2	FAM46C
1	FAM47E	2.1639E-05	-7.12	1	9.5597E-05	Q6ZV65	FAM47E
1	FAM47E-STBD	1.0958E-18	-2.60	6.3746E-14	8.1371E-18		
1	FAM49A	1.1614E-08	-10.49	0.00067561	6.1705E-08	Q9H0Q0	FAM49A
1	FAM50A	1.6509E-69	2.16	9.6039E-65	2.2534E-68	Q14320	FAM50A
1	FAM50B	1.3398E-64	-10.14	7.7942E-60	1.7702E-63	Q9Y247	FAM50B
1	FAM53A	3.2225E-14	3.38	1.8746E-09	2.1233E-13	Q6NSI3	FAM53A
1	FAM57B	4.8315E-05	2.39	1	0.00020847	Q71RH2	FAM57B
1	FAM58A	1.4057E-73	2.21	8.1775E-69	1.9781E-72	Q8N1B3	FAM58A
1	FAM64A	6.9767E-97	2.20	4.0586E-92	1.1744E-95		
1	FAM65B	0.00064849	1.85	1	0.00256738		
1	FAM65C	3.329E-120	-7.73	1.937E-115	6.445E-119		
1	FAM66D	0.00110493	-1.60	1	0.00428777		
1	FAM69A	1.1649E-73	-2.80	6.7768E-69	1.6409E-72	Q5T7M9	FAM69A
1	FAM69B	6.72E-124	4.43	3.909E-119	1.324E-122	Q5VUD6	FAM69B
1	FAM71F2	7.4877E-09	3.09	0.00043559	4.012E-08	Q6NXP2	FAM71F2
1	FAM72A	0	-2.05	0	0	Q5TYM5	FAM72A
1	FAM72B	1.078E-284	43.19	6.27E-280	3.014E-283	Q86X60	FAM72B
1	FAM72C	7.9927E-50	4.82	4.6497E-45	9.3405E-49	H0Y354	FAM72C
1	FAM72D	0.0011867	1.63	1	0.00459316	Q6L9T8	FAM72D
1	FAM78A	1.6709E-17	2.33	9.7203E-13	1.2117E-16		
1	FAM78B	0.01268268	2.18	1	0.04394297		
1	FAM83A	0	-5.02	0	0	Q86UY5	FAM83A
1	FAM83A-AS1	2.291E-32	-4.10	1.3328E-27	2.1824E-31		
1	FAM83B	5.115E-97	-3.65	2.9756E-92	8.6174E-96	Q5T0W9	FAM83B
1	FAM83C	0	-7.92	0	0	Q9BQN1	FAM83C
1	FAM83C-AS1	0.00439885	3.02	1	0.01610033		
1	FAM83E	3.3405E-25	-8.95	1.9433E-20	2.8148E-24	Q2M2I3	FAM83E
1	FAM83H	0	-3.90	0	0	Q6ZRV2	FAM83H
1	FAM83H-AS1	0	-8.08	0	0		
1	FAM84A	5.6165E-12	-9.25	3.2674E-07	3.4292E-11		
1	FAM84B	0	-5.12	0	0	Q96KN1	FAM84B
1	FAM85B	0.00074545	3.42	1	0.00293646		
1	FAM86B1	2.956E-135	3.27	1.72E-130	6.068E-134	Q8N7N1	FAM86B1
1	FAM86B2	9.0531E-11	3.20	5.2666E-06	5.2702E-10	P0C5J1	FAM86B2
1	FAM86B3P	5.3094E-17	1.92	3.0887E-12	3.8052E-16		
1	FAM86C2P	3.9951E-49	-2.29	2.3241E-44	4.6408E-48	A6NEL3	FAM86C2P
1	FAM86DP	4.426E-174	72.71	2.575E-169	1.025E-172		
1	FAM86GP	0.00425089	-3.07	1	0.01558723		
1	FAM86HP	3.0145E-12	-2.14	1.7536E-07	1.861E-11		
1	FAM86JP	1.0818E-19	-1.79	6.2934E-15	8.2041E-19		
1	FAM87B	0.00897921	-2.58	1	0.03170025		
1	FAM89A	1.049E-09	-1.73	6.1026E-05	5.8415E-09		

1	FAM95B1	0.00017769	1.96	1	0.00073632		
1	FAM95C	2.636E-236	114.44	1.533E-231	7.116E-235		
1	FAM98A	8.9689E-93	1.88	5.2176E-88	1.4689E-91	Q8NCA5	FAM98A
1	FAM9B	2.381E-116	981.54	1.385E-111	4.516E-115	Q8IZU0	FAM9B
1	FANCF	1.8879E-41	2.15	1.0983E-36	2.0137E-40	Q9NPI8	FANCF
1	FANCG	6.3493E-78	1.71	3.6937E-73	9.2411E-77	O15287	FANCG
1	FANCL	0	-1.71	0	0	Q9NW38	FANCL
1	FANK1	5.618E-32	-2.67	3.2682E-27	5.3194E-31	Q8TC84	FANK1
1	FARSA	4.5738E-47	1.74	2.6608E-42	5.2019E-46	Q9Y285	FARSA
1	FARSB	5.994E-45	1.66	3.4869E-40	6.6685E-44	Q9NSD9	FARSB
1	FAS	6.6675E-86	-8.14	3.8788E-81	1.0374E-84	P25445	FAS
1	FASN	0	-4.70	0	0	P49327	FASN
1	FAT1	0	-3.11	0	0	Q14517	FAT1
1	FAT2	0	-15.50	0	0	Q9NYQ8	FAT2
1	FAT4	5.1347E-98	-4.03	2.9871E-93	8.7188E-97	Q6V0I7	FAT4
1	FAXC	1.9725E-15	-2.87	1.1475E-10	1.3517E-14	Q5TGI0	FAXC
1	FBLIM1	0	-4.60	0	0	Q8WUP2	FBLIM1
1	FBLN1	1.125E-240	-30.85	6.545E-236	3.051E-239	P23142	FBLN1
1	FBLN2	5.2756E-06	-14.92	0.30690167	2.4232E-05	P98095	FBLN2
1	FBLN7	1.4229E-30	-2.98	8.2777E-26	1.3198E-29	Q53RD9	FBLN7
1	FBN1	2.225E-248	69.88	1.294E-243	6.071E-247	P35555	FBN1
1	FBN2	0	-13.59	0	0	P35556	FBN2
1	FBP2	6.635E-122	-6.04	3.86E-117	1.298E-120	O00757	FBP2
1	FBXL13	1.397E-86	3.79	8.1271E-82	2.183E-85	Q8NEE6	FBXL13
1	FBXL14	1.5023E-22	1.54	8.7392E-18	1.2044E-21	Q8N1E6	FBXL14
1	FBXL15	2.1983E-18	1.85	1.2788E-13	1.6212E-17	Q9H469	FBXL15
1	FBXL16	1.2547E-64	16.95	7.2989E-60	1.6581E-63	Q8N461	FBXL16
1	FBXL19-AS1	7.0629E-17	2.27	4.1088E-12	5.0538E-16		
1	FBXL22	2.3068E-06	-2.87	0.1341933	1.0842E-05	Q6P050	FBXL22
1	FBXL7	0.01193988	2.34	1	0.04152758	Q9UJT9	FBXL7
1	FBXO10	3.685E-116	3.14	2.144E-111	6.982E-115	Q9UK96	FBXO10
1	FBXO15	0.01254885	1.86	1	0.04351815	Q8NCQ5	FBXO15
1	FBXO17	5.96E-175	26.36	3.467E-170	1.386E-173	Q96EF6	FBXO17
1	FBXO2	3.1714E-64	-11.24	1.8449E-59	4.1759E-63	Q9UK22	FBXO2
1	FBXO24	1.5685E-11	-2.94	9.1248E-07	9.4313E-11	O75426	FBXO24
1	FBXO27	0	-2.43	0	0	Q8NI29	FBXO27
1	FBXO30	1.5975E-23	1.54	9.2932E-19	1.3034E-22	Q8TB52	FBXO30
1	FBXO32	9.0662E-69	-11.03	5.2741E-64	1.2297E-67	Q969P5	FBXO32
1	FBXO33	4.0938E-60	2.28	2.3815E-55	5.2203E-59	Q7Z6M2	FBXO33
1	FBXO36	8.4791E-14	-1.80	4.9326E-09	5.5132E-13		
1	FBXO4	0	-1.99	0	0	Q9UKT5	FBXO4
1	FBXO41	0	-1.99	0	0	Q8TF61	FBXO41
1	FBXO43	3.4247E-08	1.51	0.00199226	1.7772E-07	Q4G163	FBXO43
1	FBXO44	4.1886E-15	1.53	2.4367E-10	2.8377E-14	Q9H4M3	FBXO44
1	FBXO47	4.3692E-10	-25.28	2.5418E-05	2.4706E-09		
1	FBXO5	3.716E-72	1.92	2.1618E-67	5.1766E-71	Q9UKT4	FBXO5
1	FBXO6	5.3851E-28	3.68	3.1327E-23	4.7967E-27	Q9NRD1	FBXO6

1	FBXO7	1.6336E-34	1.53	9.503E-30	1.605E-33	Q9Y3I1	FBXO7
1	FBXO8	1.1103E-27	-2.03	6.4592E-23	9.815E-27	Q9NRD0	FBXO8
1	FBXW11	4.3388E-72	1.73	2.5241E-67	6.0413E-71	Q9UKB1	FBXW11
1	FBXW12	3.4209E-06	-2.26	0.19900859	1.5881E-05	Q6X9E4	FBXW12
1	FCF1	1.2509E-89	2.05	7.2769E-85	2.0002E-88	Q9Y324	FCF1
1	FCGR1A	1.6674E-23	-19.62	9.6998E-19	1.3595E-22	P12314	FCGR1A
1	FCGR1B	2.9216E-06	-45.63	0.16995893	1.3635E-05	Q92637	FCGR1B
1	FCGR2A	1.8413E-89	100.72	1.0712E-84	2.9419E-88	P12318	FCGR2A
1	FCGR2C	0.00028982	16.11	1	0.00117934	P31995	FCGR2C
1	FCGRT	3.332E-166	-5.03	1.939E-161	7.535E-165	P55899	FCGRT
1	FCHO1	3.4764E-25	-4.87	2.0224E-20	2.928E-24	O14526	FCHO1
1	FCHSD1	0	-1.55	0	0	Q86WN1	FCHSD1
1	FCMR	1.5174E-06	-5.78	0.08827562	7.2113E-06	O60667	FCMR
1	FCN3	2.558E-06	-6.65	0.14881119	1.1986E-05	O75636	FCN3
1	FDFT1	0	-1.75	0	0	P37268	FDFT1
1	FDX1	1.1507E-14	1.56	6.6939E-10	7.688E-14	P10109	FDX1
1	FDX2	2.219E-65	2.54	1.2909E-60	2.9499E-64	Q6P4F2	FDX2
1	FDXACB1	1.1194E-12	1.70	6.5118E-08	7.0087E-12	Q9BRP7	FDXACB1
1	FECH	2.826E-142	2.46	1.644E-137	5.97E-141	P22830	FECH
1	FEM1A	2.4594E-09	2.14	0.00014307	1.348E-08	Q9BSK4	FEM1A
1	FEM1C	6.1548E-25	1.54	3.5805E-20	5.16E-24	Q96JP0	FEM1C
1	FER	1.5808E-54	2.23	9.1963E-50	1.934E-53	P16591	FER
1	FER1L4	0	-2.40	0	0	A9Z1Z3	FER1L4
1	FER1L6	4.2308E-26	-5.90	2.4612E-21	3.6211E-25	Q2WGJ9	FER1L6
1	FERMT1	0	-2.03	0	0	Q9BQL6	FERMT1
1	FERMT2	2.3963E-71	4.36	1.394E-66	3.3167E-70	Q96AC1	FERMT2
1	FERMT3	6.4456E-15	-2.15	3.7497E-10	4.3374E-14	Q86UX7	FERMT3
1	FES	2.1416E-32	-10.67	1.2458E-27	2.0407E-31	P07332	FES
1	FEZ1	1.4559E-23	-2.98	8.4695E-19	1.1889E-22	Q99689	FEZ1
1	FEZ2	1.515E-88	1.94	8.8132E-84	2.4047E-87	Q9UHY8	FEZ2
1	FEZF1	1.1043E-28	-22.30	6.4243E-24	9.9417E-28	A0PJY2	FEZF1
1	FEZF1-AS1	1.81E-132	-16.96	1.053E-127	3.679E-131		
1	FGD1	5.2184E-90	2.35	3.0358E-85	8.3791E-89	P98174	FGD1
1	FGD2	1.507E-144	-33.77	8.768E-140	3.202E-143	Q7Z6J4	FGD2
1	FGD3	2.2815E-48	-29.25	1.3273E-43	2.6319E-47	Q5JSP0	FGD3
1	FGD4	0	-2.58	0	0	Q96M96	FGD4
1	FGD5	0.00028296	-10.66	1	0.00115242	Q6ZNL6	FGD5
1	FGD5-AS1	6.539E-101	1.70	3.8037E-96	1.13E-99		
1	FGF11	2.09E-262	-9.70	1.216E-257	5.784E-261	Q92914	FGF11
1	FGF12	2.227E-133	-9.85	1.295E-128	4.542E-132	P61328	FGF12
1	FGF13	3.1166E-10	-2.86	1.8131E-05	1.7733E-09	Q92913	FGF13
1	FGF14	2.6785E-27	-3.71	1.5582E-22	2.3481E-26	Q92915	FGF14
1	FGF18	0.00122967	2.91	1	0.00475378	O76093	FGF18
1	FGF19	8.7126E-16	-9.15	5.0685E-11	6.0375E-15	O95750	FGF19
1	FGF2	3.093E-214	8.00	1.799E-209	8.064E-213	P09038	FGF2
1	FGF20	0.00055538	11.91	1	0.00221263	Q9NP95	FGF20
1	FGF5	1.75E-172	4.16	1.018E-167	4.034E-171	P12034	FGF5

1	FGF7P3	0.00222337	1.93	1	0.00836897		
1	FGF8	0.00747604	2.15	1	0.02666205	P55075	FGF8
1	FGFBP1	0	-7.79	0	0	Q14512	FGFBP1
1	FGFBP3	1.5021E-25	2.45	8.7381E-21	1.2723E-24	Q8TAT2	FGFBP3
1	FGFR1OP2	1.1738E-27	1.58	6.8282E-23	1.0369E-26	Q9NVK5	FGFR1OP2
1	FGFR2	0	-12.98	0	0	P21802	FGFR2
1	FGFR3	0	-12.01	0	0	P22607	FGFR3
1	FGFR4	3.2124E-75	-6.77	1.8688E-70	4.5848E-74	P22455	FGFR4
1	FGR	0.0001506	-7.82	1	0.00062705	P09769	FGR
1	FHAD1	4.416E-110	-11.26	2.569E-105	8.09E-109		
1	FHDC1	4.1147E-44	-3.14	2.3937E-39	4.5403E-43		
1	FHIT	9.4454E-22	-5.28	5.4947E-17	7.4495E-21	P49789	FHIT
1	FHL1	5.907E-201	17.45	3.436E-196	1.496E-199	Q13642	FHL1
1	FHOD3	0	-2.52	0	0	Q2V2M9	FHOD3
1	FIBCD1	0.00020852	2.47	1	0.00085981	Q8N539	FIBCD1
1	FIBIN	0.00030646	-32.80	1	0.00124495	Q8TAL6	FIBIN
1	FIGN	0	-1.94	0	0	Q5HY92	FIGN
1	FILIP1	1.8227E-17	-5.72	1.0604E-12	1.3205E-16	Q7Z7B0	FILIP1
1	FITM1	8.0977E-05	4.26	1	0.00034408	A5D6W6	FITM1
1	FITM2	3.7664E-49	1.99	2.191E-44	4.3777E-48	Q8N6M3	FITM2
1	FJX1	2.278E-78	2.66	1.3252E-73	3.3296E-77	Q86VR8	FJX1
1	FKBP11	0	-2.36	0	0	Q9NYL4	FKBP11
1	FKBP14	0	-1.73	0	0	Q9NWM8	FKBP14
1	FKBP2	0	-2.20	0	0	P26885	FKBP2
1	FKBP3	7.3805E-30	1.63	4.2936E-25	6.7732E-29	Q00688	FKBP3
1	FKBP4	1.713E-135	2.37	9.963E-131	3.524E-134	Q02790	FKBP4
1	FKBP5	0	-1.53	0	0	Q13451	FKBP5
1	FKBP6	8.3363E-05	-4.72	1	0.00035383	O75344	FKBP6
1	FKBP7	5.6916E-22	1.95	3.311E-17	4.5036E-21	Q9Y680	FKBP7
1	FKBP9	0	-3.66	0	0	O95302	FKBP9
1	FKBP9P1	0.00036725	2.70	1	0.00148324	Q75LS8	FKBP9P1
1	FKBPL	1.1213E-12	1.58	6.5233E-08	7.0203E-12	Q9UIM3	FKBPL
1	FKTN	0	-1.82	0	0	O75072	FKTN
1	FLG	3.7083E-69	-13.34	2.1573E-64	5.0463E-68	P20930	FLG
1	FLG-AS1	4.2155E-92	-18.73	2.4523E-87	6.8731E-91		
1	FLG2	1.1948E-19	-49.64	6.9508E-15	9.0493E-19	Q5D862	FLG2
1	FLI1	5.592E-150	3.65	3.253E-145	1.204E-148	Q01543	FLI1
1	FLJ21408	6.0098E-26	-5.98	3.4962E-21	5.1233E-25		
1	FLJ22447	0	-2.75	0	0		
1	FLJ27354	7.4992E-10	1.78	4.3626E-05	4.2E-09		
1	FLJ31104	0.00040993	-2.92	1	0.00164863		
1	FLJ31356	2.7658E-11	-5.54	1.609E-06	1.6459E-10		
1	FLJ37035	7.2454E-14	-2.60	4.215E-09	4.7226E-13		
1	FLJ37453	7.2182E-28	-2.59	4.1991E-23	6.4059E-27		
1	FLJ42969	1.4772E-10	-2.41	8.5934E-06	8.5303E-10		
1	FLJ46284	9.8647E-08	-3.72	0.00573869	4.9945E-07		
1	FLNA	1.0843E-63	1.89	6.3079E-59	1.422E-62	P21333	FLNA

1	FLNC	1.979E-203	6.57	1.151E-198	5.032E-202	Q14315	FLNC
1	FLOT1	7.8972E-64	1.69	4.5941E-59	1.0371E-62	O75955	FLOT1
1	FLOT2	0	-1.61	0	0	Q14254	FLOT2
1	FLRT2	8.367E-262	110.94	4.867E-257	2.31E-260	O43155	FLRT2
1	FLT1	5.1981E-08	-4.80	0.00302393	2.6708E-07	P17948	FLT1
1	FLT3LG	2.6851E-17	-1.87	1.562E-12	1.9375E-16	P49771	FLT3LG
1	FLVCR1	0	-2.18	0	0	Q9Y5Y0	FLVCR1
1	FLVCR1-AS1	3.6559E-11	-1.77	2.1268E-06	2.1614E-10		
1	FLYWCH1	6.1516E-56	1.70	3.5787E-51	7.6109E-55	Q4VC44	FLYWCH1
1	FLYWCH2	1.1283E-14	1.51	6.5637E-10	7.5401E-14	Q96CP2	FLYWCH2
1	FMN1	4.689E-165	22.95	2.728E-160	1.056E-163	Q68DA7	FMN1
1	FMNL1	3.6838E-39	2.20	2.143E-34	3.82E-38	O95466	FMNL1
1	FMNL3	5.4079E-50	6.36	3.146E-45	6.33E-49	Q8IVF7	FMNL3
1	FMO4	4.258E-15	-2.63	2.4771E-10	2.8837E-14	P31512	FMO4
1	FMO5	9.4851E-56	-5.18	5.5179E-51	1.1723E-54	P49326	FMO5
1	FMO9P	3.8595E-05	-4.93	1	0.00016773		
1	FMOD	0.00057681	-27.27	1	0.00229376	Q06828	FMOD
1	FN1	0	305.50	0	0	P02751	FN1
1	FN3K	7.4139E-56	5.73	4.313E-51	9.1668E-55	Q9H479	FN3K
1	FN3KRP	3.2411E-95	1.84	1.8855E-90	5.4025E-94	Q9HA64	FN3KRP
1	FNBP1	7.9545E-29	3.15	4.6274E-24	7.1732E-28	Q96RU3	FNBP1
1	FNBP1L	1.0167E-41	1.66	5.9146E-37	1.0888E-40	Q5T0N5	FNBP1L
1	FNBP1P1	5.3783E-21	-3.54	3.1288E-16	4.1789E-20		
1	FNDC10	5.0867E-05	2.23	1	0.00021919	F2Z333	FNDC10
1	FNDC11	0.00668771	2.39	1	0.02396078	Q9BVV2	FNDC11
1	FNDC3A	2.0299E-57	1.68	1.1808E-52	2.5356E-56	Q9Y2H6	FNDC3A
1	FNDC4	1.3303E-35	9.18	7.7387E-31	1.3224E-34	Q9H6D8	FNDC4
1	FNDC7	0.0087302	3.47	1	0.03086985	Q5VTL7	FNDC7
1	FNDC8	0.0014343	2.93	1	0.00550279	Q8TC99	FNDC8
1	FNTA	0	-1.67	0	0	P49354	FNTA
1	FNTB	1.26E-127	2.51	7.33E-123	2.521E-126	P49356	FNTB
1	FOLH1B	0.00019398	-5.72	1	0.00080226	Q9HBA9	FOLH1B
1	FOLR1	2.8582E-16	-9.52	1.6628E-11	2.0125E-15	P15328	FOLR1
1	FOLR3	2.3088E-15	-11.04	1.3431E-10	1.5777E-14	P41439	FOLR3
1	FOSL2	1.6435E-58	1.55	9.5607E-54	2.0735E-57	P15408	FOSL2
1	FOXA1	3.817E-132	10.04	2.221E-127	7.757E-131	P55317	FOXA1
1	FOXC2	1.802E-284	303.91	1.048E-279	5.037E-283	Q99958	FOXC2
1	FOXC2-AS1	9.1422E-40	77.97	5.3184E-35	9.5312E-39		
1	FOXD1	2.0468E-58	2.25	1.1907E-53	2.5784E-57	Q16676	FOXD1
1	FOXD2-AS1	1.5282E-40	2.40	8.8902E-36	1.6088E-39		
1	FOXD3-AS1	5.6122E-19	-10.31	3.2648E-14	4.1932E-18		
1	FOXD4L4	0.00260925	6.19	1	0.00976145	Q8WXT5	FOXD4L4
1	FOXD4L6	0.00225089	8.89	1	0.00846762	Q3SYB3	FOXD4L6
1	FOXE1	1.0504E-22	-36.52	6.1108E-18	8.4449E-22	O00358	FOXE1
1	FOXF2	2.5166E-55	3.66	1.464E-50	3.0991E-54	Q12947	FOXF2
1	FOXG1	0.00030142	-5.14	1	0.00122517	P55316	FOXG1
1	FOXJ2	2.0177E-37	3.41	1.1738E-32	2.0457E-36	Q9P0K8	FOXJ2

1	FOXL1	1.871E-144	80.54	1.089E-139	3.974E-143	Q12952	FOXL1
1	FOXL2NB	1.175E-28	-2.52	6.8356E-24	1.057E-27	Q6ZUU3	FOXL2NB
1	FOXM1	2.7956E-42	1.61	1.6263E-37	3.019E-41	Q08050	FOXM1
1	FOXN1	1.1573E-06	-3.88	0.06732623	5.539E-06	O15353	FOXN1
1	FOXN2	3.0692E-11	1.57	1.7855E-06	1.8217E-10	P32314	FOXN2
1	FOXN3	1.8377E-92	3.75	1.069E-87	3.0021E-91	O00409	FOXN3
1	FOXO3	0	-1.68	0	0	O43524	FOXO3
1	FOXO4	7.5225E-14	1.61	4.3761E-09	4.901E-13	P98177	FOXO4
1	FOXO6	2.6535E-15	-2.99	1.5436E-10	1.8111E-14	A8MYZ6	FOXO6
1	FOXP1	0	29.76	0	0	Q9H334	FOXP1
1	FOXP2	1.326E-157	-12.76	7.711E-153	2.924E-156	O15409	FOXP2
1	FOXP4-AS1	1.0817E-09	-2.07	6.2929E-05	6.0179E-09		
1	FOXQ1	8.9321E-21	-1.86	5.1962E-16	6.9117E-20	Q9C009	FOXQ1
1	FOXRED1	3.202E-109	2.15	1.863E-104	5.829E-108	Q96CU9	FOXRED1
1	FOXRED2	0	-2.18	0	0	Q8IWF2	FOXRED2
1	FOXS1	0.00400756	-19.71	1	0.01473956	O43638	FOXS1
1	FPGT	0	-1.57	0	0	O14772	FPGT
1	FPGT-TNNI3K	6.0419E-14	-4.69	3.5148E-09	3.9479E-13	V9GXZ4	FPGT-TNNI3K
1	FRAS1	0	-10.97	0	0	Q86XX4	FRAS1
1	FREM1	6.8197E-07	-43.96	0.03967307	3.3042E-06	Q5H8C1	FREM1
1	FREM2	5.87E-124	-30.48	3.415E-119	1.157E-122	Q5SZK8	FREM2
1	FREM3	2.094E-07	-7.86	0.01218146	1.0425E-06	P0C091	FREM3
1	FRG1	0	-2.01	0	0	Q14331	FRG1
1	FRG1DP	2.8E-202	-10.30	1.629E-197	7.108E-201		
1	FRG1EP	2.4909E-74	-6.62	1.449E-69	3.5273E-73		
1	FRG1FP	7.484E-103	-12.89	4.3537E-98	1.313E-101		
1	FRG1GP	5.392E-153	-8.45	3.137E-148	1.169E-151		
1	FRG1HP	3.6277E-66	2.09	2.1104E-61	4.8459E-65		
1	FRG1JP	1.7672E-16	-3.13	1.028E-11	1.2526E-15		
1	FRG1KP	6.9309E-31	-5.18	4.032E-26	6.4605E-30		
1	FRGCA	1.0488E-05	-7.49	0.61010718	4.7306E-05		
1	FRK	1.913E-214	-11.70	1.113E-209	4.993E-213	P42685	FRK
1	FRMD4A	2.2926E-82	3.06	1.3337E-77	3.4668E-81	Q9P2Q2	FRMD4A
1	FRMD4B	2.0149E-70	-10.49	1.1722E-65	2.7724E-69	Q9Y2L6	FRMD4B
1	FRMD5	9.4338E-17	1.97	5.488E-12	6.7321E-16	Q7Z6J6	FRMD5
1	FRMPD2	1.0218E-89	-12.91	5.9442E-85	1.6348E-88	Q68DX3	FRMPD2
1	FRMPD3	2.4852E-15	-6.72	1.4458E-10	1.6971E-14	Q5JV73	FRMPD3
1	FRRS1	2.929E-173	-4.44	1.704E-168	6.771E-172	Q6ZNA5	FRRS1
1	FRYL	0	-1.91	0	0	O94915	FRYL
1	FSCN1	0	-2.24	0	0	Q16658	FSCN1
1	FSCN2	0.00240816	-1.72	1	0.0090353	O14926	FSCN2
1	FSD1	2.3671E-63	7.17	1.377E-58	3.0965E-62	Q9BTV5	FSD1
1	FSD1L	1.1679E-20	2.07	6.7943E-16	9.0158E-20	Q9BXM9	FSD1L
1	FSIP2	1.1467E-31	-6.82	6.6706E-27	1.0803E-30	Q5CZC0	FSIP2
1	FSIP2-AS1	1.3097E-10	-16.22	7.6193E-06	7.5829E-10		
1	FSTL1	1.166E-222	13.82	6.782E-218	3.098E-221	Q12841	FSTL1
1	FSTL3	2.635E-74	6.95	1.5329E-69	3.7306E-73	O95633	FSTL3

1	FSTL5	2.4795E-10	-7.66	1.4424E-05	1.4176E-09	Q8N475	FSTL5
1	FTCD	1.3838E-26	-12.54	8.0504E-22	1.1942E-25	O95954	FTCD
1	FTCDNL1	7.3994E-33	-9.22	4.3045E-28	7.1043E-32	E5RQL4	FTCDNL1
1	FTH1	0	-3.61	0	0	P02794	FTH1
1	FTH1P2	2.4108E-08	-4.15	0.00140243	1.2617E-07		
1	FTH1P8	0.00068883	-3.40	1	0.00271859		
1	FTSJ3	1.264E-39	2.00	7.3531E-35	1.3154E-38	Q8IY81	FTSJ3
1	FURIN	0	-2.43	0	0	P09958	FURIN
1	FUT1	2.5667E-91	-4.01	1.4931E-86	4.158E-90	P19526	FUT1
1	FUT11	4.091E-116	2.90	2.38E-111	7.75E-115	Q495W5	FUT11
1	FUT2	2.1952E-29	-3.08	1.2771E-24	1.9979E-28	Q10981	FUT2
1	FUT3	1.086E-257	-28.91	6.316E-253	2.987E-256	P21217	FUT3
1	FUT8	1.257E-235	11.91	7.312E-231	3.387E-234	Q9BYC5	FUT8
1	FUT9	1.165E-116	-12.25	6.78E-112	2.216E-115	Q9Y231	FUT9
1	FUZ	5.047E-18	1.59	2.936E-13	3.6996E-17	Q9BT04	FUZ
1	FXN	7.5199E-39	2.11	4.3747E-34	7.762E-38	Q16595	FXN
1	FXYD2	6.7737E-18	121.38	3.9405E-13	4.9498E-17	P54710	FXYD2
1	FXYD3	0	-22.63	0	0	Q14802	FXYD3
1	FXYD6-FXYD2	0.00818319	17.61	1	0.02904153	A0A087WZ82	FXYD6-FXYD2
1	FYB	6.2957E-12	-17.99	3.6625E-07	3.8383E-11		
1	FYCO1	2.2484E-31	1.61	1.308E-26	2.11E-30	Q9BQS8	FYCO1
1	FYN	7.4176E-94	2.23	4.3151E-89	1.2245E-92	P06241	FYN
1	FZD1	3.2068E-67	-2.58	1.8655E-62	4.3134E-66	Q9UP38	FZD1
1	FZD5	0	-1.81	0	0	Q13467	FZD5
1	FZD6	0	-2.60	0	0	O60353	FZD6
1	FZD7	0	-1.72	0	0	O75084	FZD7
1	FZD8	0	87.15	0	0	Q9H461	FZD8
1	G0S2	8.1246E-13	-1.62	4.7264E-08	5.1107E-12	P27469	G0S2
1	G3BP2	0	-1.72	0	0	Q9UN86	G3BP2
1	G6PD	0	-1.54	0	0	P11413	G6PD
1	GAB2	1.2601E-19	1.77	7.3304E-15	9.5374E-19	Q9UQC2	GAB2
1	GABARAP	1.7389E-22	1.60	1.0116E-17	1.3903E-21	H6UMI1, O95	GABARAP
1	GABARAPL1	0	-1.83	0	0	Q9H0R8	GABARAPL1
1	GABBR1	3.32E-216	7.49	1.931E-211	8.708E-215	Q9UBS5	GABBR1
1	GABPB1-AS1	0	-6.89	0	0		
1	GABRA3	1.672E-141	-10.37	9.727E-137	3.519E-140	P34903	GABRA3
1	GABRB1	0.00229007	-3.56	1	0.00860887	P18505	GABRB1
1	GABRE	2.047E-196	-7.87	1.191E-191	5.103E-195	P78334	GABRE
1	GABRG1	4.788E-07	-11.81	0.02785344	2.3394E-06	Q8N1C3	GABRG1
1	GABRG2	1.811E-194	-7.40	1.054E-189	4.486E-193	P18507	GABRG2
1	GABRG3	0.00090242	-4.37	1	0.00352857	Q99928	GABRG3
1	GABRP	0.00367243	-8.23	1	0.0135421	O00591	GABRP
1	GABRQ	2.6791E-21	-4.05	1.5585E-16	2.0945E-20	Q9UN88	GABRQ
1	GABRR1	1.1468E-15	-9.07	6.6713E-11	7.8997E-15	P24046	GABRR1
1	GABRR2	8.8113E-07	3.06	0.05125877	4.2436E-06	P28476	GABRR2
1	GACAT2	5.251E-137	77.90	3.054E-132	1.088E-135		
1	GAD1	1.1778E-61	-3.95	6.8515E-57	1.5198E-60	Q99259	GAD1

1	GADD45B	1.6978E-25	1.85	9.8765E-21	1.4374E-24	O75293	GADD45B
1	GADD45GIP1	4.3664E-24	1.90	2.5401E-19	3.5969E-23	Q8TAE8	GADD45GIP1
1	GADL1	0.01437271	17.75	1	0.04954772	Q6ZQY3	GADL1
1	GAK	0	-1.73	0	0	O14976	GAK
1	GAL	1.617E-162	-17.57	9.405E-158	3.616E-161	P22466	GAL
1	GAL3ST4	2.5828E-27	-6.62	1.5025E-22	2.2659E-26	Q96RP7	GAL3ST4
1	GALC	4.9672E-61	-22.66	2.8896E-56	6.3746E-60	P54803	GALC
1	GALK1	1.9819E-30	1.74	1.153E-25	1.8345E-29	P51570	GALK1
1	GALK2	0	-1.76	0	0	Q01415	GALK2
1	GALM	3.2553E-67	-3.16	1.8938E-62	4.3766E-66	Q96C23	GALM
1	GALNT1	1.132E-107	2.36	6.588E-103	2.041E-106	Q10472	GALNT1
1	GALNT10	2.1913E-93	2.07	1.2748E-88	3.6041E-92	Q86SR1	GALNT10
1	GALNT11	1.3945E-45	1.62	8.1125E-41	1.5631E-44	Q8NCW6	GALNT11
1	GALNT12	7.141E-181	-6.35	4.154E-176	1.688E-179	Q8IXK2	GALNT12
1	GALNT13	1.813E-218	-9.42	1.055E-213	4.778E-217	Q8IUC8	GALNT13
1	GALNT16	1.753E-109	18.69	1.02E-104	3.2E-108	Q8N428	GALNT16
1	GALNT18	0	-4.24	0	0	Q6P9A2	GALNT18
1	GALNT2	0	-2.94	0	0	Q10471	GALNT2
1	GALNT3	0	-10.85	0	0	Q14435	GALNT3
1	GALNT5	0	-17.01	0	0	Q7Z7M9	GALNT5
1	GALNT6	0	-3.79	0	0	Q8NCL4	GALNT6
1	GALNT8	0.00601472	5.16	1	0.02167503	Q9NY28	GALNT8
1	GALNT9	7.2592E-06	-4.00	0.42229954	3.3088E-05	Q9HCQ5	GALNT9
1	GALNTL6	5.0292E-66	-8.43	2.9257E-61	6.7119E-65	Q49A17	GALNTL6
1	GAMT	1.2709E-11	2.26	7.3934E-07	7.6687E-11	Q14353	GAMT
1	GAP43	5.745E-22	-7.75	3.3421E-17	4.5446E-21	P17677	GAP43
1	GAPDH	2.2967E-86	2.66	1.3361E-81	3.5839E-85	P04406	GAPDH
1	GAPDHP21	1.1709E-08	-5.36	0.00068118	6.2202E-08		
1	GAPDHP35	0.00475472	2.84	1	0.0173385		
1	GAREM2	1.0731E-19	-4.85	6.2429E-15	8.1405E-19	Q75VX8	GAREM2
1	GARS	3.4975E-43	2.18	2.0346E-38	3.8202E-42	P41250	GARS
1	GAS1	1.7162E-37	21.57	9.9838E-33	1.7421E-36	P54826	GAS1
1	GAS2L1	1.1687E-82	2.29	6.7985E-78	1.7732E-81	Q99501	GAS2L1
1	GAS2L2	4.4004E-21	-23.00	2.5599E-16	3.4255E-20	Q8NHY3	GAS2L2
1	GAS5	0	-1.64	0	0		
1	GAS6	0	-2.31	0	0	Q14393	GAS6
1	GAS6-AS1	2.802E-155	-15.44	1.63E-150	6.132E-154		
1	GAS6-AS2	5.4851E-40	-3.51	3.1909E-35	5.7359E-39		
1	GATA1	0.00584746	-5.63	1	0.02110105	P15976	GATA1
1	GATA2	5.8362E-16	-1.64	3.3951E-11	4.0695E-15	P23769	GATA2
1	GATA3	6.494E-106	3.53	3.778E-101	1.159E-104	P23771	GATA3
1	GATA3-AS1	3.1438E-76	37.44	1.8289E-71	4.5225E-75		
1	GATA6	3.1789E-18	-1.72	1.8493E-13	2.3385E-17	Q92908	GATA6
1	GATA6-AS1	0.00411422	-2.30	1	0.01511562		
1	GATAD1	0	-1.68	0	0	Q8WUU5	GATAD1
1	GATAD2A	8.8631E-84	1.87	5.156E-79	1.3561E-82	Q86YP4	GATAD2A
1	GATS	1.59E-138	-3.97	9.253E-134	3.309E-137		

1	GBA2	1.5279E-75	1.67	8.8886E-71	2.1839E-74	Q9HCG7	GBA2
1	GBAP1	0	-1.88	0	0		
1	GBAS	9.537E-25	1.50	5.5481E-20	7.9679E-24		
1	GBGT1	1.9775E-08	54.97	0.00115037	1.0397E-07	Q8N5D6	GBGT1
1	GBP1	3.13E-180	-17.70	1.821E-175	7.388E-179	P32455	GBP1
1	GBP1P1	8.5058E-98	-13.72	4.9481E-93	1.4418E-96		
1	GBP2	2.272E-165	-12.80	1.321E-160	5.122E-164	P32456	GBP2
1	GBP3	3.4793E-20	1.64	2.024E-15	2.6632E-19	Q9H0R5	GBP3
1	GBP4	5.796E-121	-129.33	3.372E-116	1.126E-119	Q96PP9	GBP4
1	GBP6	1.741E-207	-12.79	1.013E-202	4.468E-206	Q6ZN66	GBP6
1	GBP7	5.4167E-28	-16.31	3.1511E-23	4.8234E-27	Q8N8V2	GBP7
1	GCA	2.364E-189	-5.24	1.375E-184	5.732E-188	P28676	GCA
1	GCAT	5.3763E-51	1.86	3.1276E-46	6.3453E-50	O75600	GCAT
1	GCC2-AS1	0.00215659	-1.67	1	0.00813496		
1	GCGR	3.261E-25	-25.63	1.897E-20	2.7486E-24	P47871	GCGR
1	GCLC	0	-6.35	0	0	P48506	GCLC
1	GCM1	3.0032E-08	8.12	0.00174708	1.5645E-07	Q9NP62	GCM1
1	GCNT1	0	-5.16	0	0	Q02742	GCNT1
1	GCNT2	1.8945E-25	-1.99	1.1021E-20	1.6033E-24	Q8N0V5	GCNT2
1	GCNT4	0.00354074	-1.60	1	0.01307969	Q9P109	GCNT4
1	GCNT7	0.00862903	5.92	1	0.03052695	Q6ZNI0	GCNT7
1	GCOM1	1.0239E-06	-6.05	0.05956605	4.9131E-06	H8Y6P7	GCOM1
1	GCSAM	1.3777E-06	-1.62	0.08014636	6.5645E-06	Q8N6F7	GCSAM
1	GDA	0.00055882	-4.73	1	0.00222541	Q9Y2T3	GDA
1	GDAP1	2.6876E-48	2.00	1.5635E-43	3.0984E-47	Q8TB36	GDAP1
1	GDF11	0	-1.50	0	0	O95390	GDF11
1	GDF15	2.6601E-09	-1.80	0.00015475	1.4562E-08	Q99988	GDF15
1	GDF5	0.00016008	-1.81	1	0.00066524	P43026	GDF5
1	GDI1	2.075E-135	1.85	1.207E-130	4.265E-134	P31150	GDI1
1	GDPD2	1.5851E-53	-7.81	9.221E-49	1.921E-52	Q9HCC8	GDPD2
1	GDPD3	2.1334E-10	-1.64	1.2411E-05	1.2232E-09	Q7L5L3	GDPD3
1	GEM	1.6007E-07	1.60	0.00931163	8.0176E-07	P55040	GEM
1	GEMIN2	1.7204E-22	1.89	1.0008E-17	1.3757E-21	O14893	GEMIN2
1	GEMIN5	1.2013E-90	2.02	6.9885E-86	1.9359E-89	Q8TEQ6	GEMIN5
1	GEMIN7	1.3003E-12	1.50	7.5646E-08	8.1191E-12	Q9H840	GEMIN7
1	GEMIN8	3.474E-100	-2.92	2.0208E-95	5.975E-99	Q9NWZ8	GEMIN8
1	GET4	0	-1.69	0	0	Q7L5D6	GET4
1	GFER	1.3991E-55	2.22	8.1392E-51	1.7266E-54	P55789	GFER
1	GFI1	4.8868E-95	-4.28	2.8428E-90	8.1363E-94	Q99684	GFI1
1	GFPT2	5.735E-100	41.20	3.3364E-95	9.856E-99	O94808	GFPT2
1	GFRA1	9.1008E-09	22.76	0.00052943	4.8599E-08	P56159	GFRA1
1	GFRAL	0.001689	-11.11	1	0.00643837	Q6UXV0	GFRAL
1	GFY	2.7641E-34	-17.64	1.608E-29	2.7057E-33	I3L273	GFY
1	GGCT	0	-1.69	0	0	O75223	GGCT
1	GGCX	0	-1.60	0	0	P38435	GGCX
1	GGN	2.7708E-06	1.91	0.16118678	1.2956E-05	Q86UU5	GGN
1	GGPS1	0	-2.08	0	0	O95749	GGPS1

1	GGT1	2.1459E-10	-1.66	1.2483E-05	1.2303E-09	P19440	GGT1
1	GGT4P	1.7137E-06	-8.94	0.09969121	8.1195E-06		
1	GGT6	4.1508E-24	-160.05	2.4147E-19	3.4202E-23	Q6P531	GGT6
1	GGT7	7.8707E-83	5.75	4.5787E-78	1.1949E-81	Q9UJ14	GGT7
1	GHDC	0	-1.68	0	0	Q8N2G8	GHDC
1	GHRLOS_1	0.00042328	1.60	1	0.00170042		
1	GIGYF1	0	-1.67	0	0	O75420	GIGYF1
1	GIMAP2	3.5181E-16	-15.20	2.0466E-11	2.4708E-15	Q9UG22	GIMAP2
1	GINS1	1.5382E-61	1.58	8.9481E-57	1.9841E-60	Q14691	GINS1
1	GINS2	6.3925E-21	1.58	3.7188E-16	4.9597E-20	Q9Y248	GINS2
1	GINS3	4.6212E-53	1.83	2.6883E-48	5.5728E-52	Q9BRX5	GINS3
1	GIPC2	4.1846E-52	-5.06	2.4343E-47	4.9976E-51	Q8TF65	GIPC2
1	GIPR	5.367E-101	-7.57	3.1221E-96	9.289E-100	P48546	GIPR
1	GIT1	0	-1.74	0	0	Q9Y2X7	GIT1
1	GJA1	1.005E-50	1.91	5.8467E-46	1.1835E-49	P17302	GJA1
1	GJA3	1.7104E-30	-3.09	9.9501E-26	1.5852E-29	Q9Y6H8	GJA3
1	GJA9	0.00600475	-4.19	1	0.02164179	P57773	GJA9
1	GJB2	0	-8.92	0	0	P29033	GJB2
1	GJB3	0	-8.73	0	0	O75712	GJB3
1	GJB4	2.481E-16	-6.21	1.4433E-11	1.7488E-15	Q9NTQ9	GJB4
1	GJB5	3.051E-195	-9.24	1.775E-190	7.585E-194	O95377	GJB5
1	GJB6	2.19E-248	-12.01	1.274E-243	5.978E-247	O95452	GJB6
1	GJC1	1.5487E-43	4.26	9.0093E-39	1.7005E-42	P36383	GJC1
1	GJC2	4.5921E-07	-2.06	0.02671414	2.2456E-06	Q5T442	GJC2
1	GJC3	0.00467599	-1.97	1	0.01707067	Q8NFK1	GJC3
1	GK5	0	-2.06	0	0	Q6ZS86	GK5
1	GLB1L	1.3889E-18	-1.61	8.0796E-14	1.0291E-17	Q6UWU2	GLB1L
1	GLDC	0.0007744	-1.67	1	0.00304638	P23378	GLDC
1	GLDN	6.2576E-23	-3.74	3.6403E-18	5.0497E-22	Q6ZMI3	GLDN
1	GLI2	9.338E-162	85.53	5.432E-157	2.083E-160	P10070	GLI2
1	GLI3	3.3813E-19	2.69	1.967E-14	2.5374E-18	P10071	GLI3
1	GLI4	0	-3.04	0	0	P10075	GLI4
1	GLIDR	1.506E-70	4.02	8.7613E-66	2.0742E-69		
1	GLIPR1	2.7001E-22	1.59	1.5707E-17	2.149E-21	P48060	GLIPR1
1	GLIPR1L1	1.2389E-08	-102.22	0.00072071	6.574E-08	Q6UWM5	GLIPR1L1
1	GLIPR2	6.909E-172	10.90	4.02E-167	1.589E-170	Q9H4G4	GLIPR2
1	GLIS1	6.9872E-73	51.60	4.0647E-68	9.7922E-72	Q8NBF1	GLIS1
1	GLIS3	5.159E-154	3.59	3.001E-149	1.124E-152	Q8NEA6	GLIS3
1	GLMP	0	-1.98	0	0	Q8WWB7	GLMP
1	GLRA1	3.0221E-11	2.49	1.758E-06	1.7945E-10	P23415	GLRA1
1	GLRA3	1.9697E-29	-8.42	1.1458E-24	1.794E-28	O75311	GLRA3
1	GLRB	2.3318E-50	-2.68	1.3565E-45	2.7382E-49	P48167	GLRB
1	GLRX	7.4652E-39	2.21	4.3428E-34	7.7082E-38	P35754	GLRX
1	GLRX5	2.2199E-70	2.11	1.2914E-65	3.0537E-69	Q86SX6	GLRX5
1	GLS	1.601E-119	2.41	9.312E-115	3.09E-118	O94925	GLS
1	GLS2	1.2807E-26	-5.17	7.4502E-22	1.1062E-25	Q9UI32	GLS2
1	GLT8D2	1.755E-157	-5.08	1.021E-152	3.87E-156	Q9H1C3	GLT8D2

1	GLTPD2	6.7734E-34	5.16	3.9404E-29	6.5992E-33	A6NH11	GLTPD2
1	GLTSCR1L	0	-2.05	0	0		
1	GLUD1P3	8.6731E-38	2.56	5.0455E-33	8.8316E-37		
1	GLUL	0	-7.68	0	0	P15104	GLUL
1	GLYAT	4.7103E-15	6.68	2.7402E-10	3.1833E-14	Q6IB77	GLYAT
1	GMDS-AS1	1.9549E-31	-2.36	1.1373E-26	1.8364E-30		
1	GMFB	2.8557E-69	3.07	1.6613E-64	3.8888E-68	P60983	GMFB
1	GML	3.2296E-09	-33.96	0.00018788	1.7605E-08	Q99445	GML
1	GMNC	2.6685E-09	-6.93	0.00015524	1.4606E-08	A6NCL1	GMNC
1	GMPPA	5.2953E-57	1.78	3.0805E-52	6.5949E-56	Q96IJ6	GMPPA
1	GMPPB	2.7522E-66	1.85	1.6011E-61	3.6781E-65	Q9Y5P6	GMPPB
1	GMPR2	8.2843E-35	1.60	4.8193E-30	8.1628E-34	Q9P2T1	GMPR2
1	GNA11	1.2733E-47	2.26	7.4075E-43	1.4576E-46	P29992	GNA11
1	GNA12	1.085E-120	1.98	6.31E-116	2.106E-119	Q03113	GNA12
1	GNA15	0	-1.93	0	0	P30679	GNA15
1	GNAI1	3.0719E-70	-4.56	1.7871E-65	4.2217E-69	P63096	GNAI1
1	GNAI3	3.0826E-47	1.75	1.7933E-42	3.5114E-46	P08754	GNAI3
1	GNAO1	6.1925E-74	-16.55	3.6025E-69	8.7375E-73	P09471	GNAO1
1	GNAS-AS1	2.8621E-10	-7.64	1.665E-05	1.6322E-09		
1	GNAZ	4.2542E-82	28.90	2.4749E-77	6.4215E-81	P19086	GNAZ
1	GNB1	3.6865E-94	1.80	2.1446E-89	6.0995E-93	P62873	GNB1
1	GNB1L	1.5037E-80	2.51	8.7474E-76	2.2435E-79	Q9BYB4	GNB1L
1	GNB3	1.587E-145	16.58	9.232E-141	3.38E-144	P16520	GNB3
1	GNE	1.4088E-71	5.50	8.1956E-67	1.9532E-70	Q9Y223	GNE
1	GNG11	7.3561E-78	9.94	4.2793E-73	1.0698E-76	P61952	GNG11
1	GNG12	1.0369E-78	2.22	6.0323E-74	1.5202E-77	Q9UBI6	GNG12
1	GNG2	0.00209401	-2.88	1	0.00790812	P59768	GNG2
1	GNG4	0.01190817	-2.31	1	0.04143712	P50150	GNG4
1	GNG5	4.6022E-36	1.65	2.6773E-31	4.5994E-35	P63218	GNG5
1	GNG7	0.00025188	-2.23	1	0.00103072	O60262	GNG7
1	GNGT1	0	-2.59	0	0	P63211	GNGT1
1	GNL3	2.355E-102	1.90	1.37E-97	4.113E-101	Q9BVP2	GNL3
1	GNL3L	2.4135E-31	2.11	1.404E-26	2.2645E-30	Q9NVN8	GNL3L
1	GNLY	0.00077585	-25.34	1	0.00305126	P22749	GNLY
1	GNPAT	0	-1.66	0	0	O15228	GNPAT
1	GNPNAT1	2.6864E-51	1.87	1.5628E-46	3.1783E-50	Q96EK6	GNPNAT1
1	GNRH1	1.3939E-12	-2.05	8.1091E-08	8.6924E-12	P01148	GNRH1
1	GOLGA1	0	-1.56	0	0	Q92805	GOLGA1
1	GOLGA2	0	-1.51	0	0	Q08379	GOLGA2
1	GOLGA2P10	4.3071E-16	1.51	2.5056E-11	3.0192E-15		
1	GOLGA2P7	0	-2.12	0	0		
1	GOLGA3	4.3898E-46	1.58	2.5537E-41	4.9453E-45	Q08378	GOLGA3
1	GOLGA5	5.0823E-30	1.55	2.9566E-25	4.6715E-29	Q8TBA6	GOLGA5
1	GOLGA6L10	1.2531E-08	-2.19	0.000729	6.6484E-08	A6NI86	GOLGA6L10
1	GOLGA6L9	0	-2.15	0	0	A6NEM1	GOLGA6L9
1	GOLGA8A	7.0086E-10	1.55	4.0772E-05	3.9294E-09	A7E2F4	GOLGA8A
1	GOLGA8B	6.51E-52	4.17	3.7871E-47	7.7557E-51	A8MQT2	GOLGA8B

1	GOLGA8F	1.8044E-28	-580.47	1.0497E-23	1.6169E-27	Q08AF8	GOLGA8F
1	GOLGA8G	7.2146E-28	-572.30	4.197E-23	6.4038E-27		
1	GOLGA8H	1.1229E-08	2.11	0.00065325	5.9695E-08	P0CJ92	GOLGA8H
1	GOLGA8J	6.8818E-05	-5.25	1	0.00029379	A6NMD2	GOLGA8J
1	GOLGA8K	0.00016708	4.98	1	0.0006936	D6RF30	GOLGA8K
1	GOLGA8N	4.0769E-08	-1.75	0.00237171	2.1073E-07	F8WBI6	GOLGA8N
1	GOLGA8Q	0.00251562	-4.17	1	0.00942146	A0A0J9YX86	GOLGA8Q
1	GOLGA8R	4.3285E-08	-3.92	0.00251806	2.2343E-07	I6L899	GOLGA8R
1	GOLGA8S	0.0086661	2.61	1	0.03065065	H3BPF8	GOLGA8S
1	GOLGA8T	0.00039591	-6.23	1	0.001594	H3BQL2	GOLGA8T
1	GOLIM4	0	-1.60	0	0	O00461	GOLIM4
1	GOLM1	0	-1.55	0	0	Q8NBJ4	GOLM1
1	GOLPH3	0	-1.58	0	0	Q9H4A6	GOLPH3
1	GOLPH3L	0	-1.59	0	0	Q9H4A5	GOLPH3L
1	GOLT1A	1.6574E-15	-101.96	9.6415E-11	1.1376E-14	Q6ZVE7	GOLT1A
1	GOLT1B	3.3354E-77	2.20	1.9403E-72	4.8279E-76	Q9Y3E0	GOLT1B
1	GON4L	0	-1.61	0	0	Q3T8J9	GON4L
1	GON7	1.7172E-60	2.47	9.9899E-56	2.1951E-59	Q9BXV9	GON7
1	GP1BA	3.2093E-08	2.17	0.00186696	1.6681E-07	P07359	GP1BA
1	GPAA1	0	-1.80	0	0	O43292	GPAA1
1	GPAT2P2	0.00117013	-11.91	1	0.00453113		
1	GPAT3	4.8643E-17	1.88	2.8298E-12	3.4888E-16	Q53EU6	GPAT3
1	GPATCH11	7.9999E-32	1.76	4.6538E-27	7.5586E-31	Q8N954	GPATCH11
1	GPATCH2	0	-1.83	0	0	Q9NW75	GPATCH2
1	GPATCH4	1.2078E-22	1.55	7.0265E-18	9.6985E-22	Q5T3I0	GPATCH4
1	GPBAR1	0.00176992	2.09	1	0.00673225	Q8TDU6	GPBAR1
1	GPC1	0	-6.27	0	0	P35052	GPC1
1	GPC2	2.5062E-22	3.31	1.4579E-17	1.9966E-21	Q8N158	GPC2
1	GPC5	1.2491E-07	-5.44	0.00726641	6.2956E-07	P78333	GPC5
1	GPC6	0.00020116	-2.88	1	0.00083072	Q9Y625	GPC6
1	GPD2	0	-2.08	0	0	P43304	GPD2
1	GPER1	1.6127E-16	-5.14	9.3817E-12	1.1442E-15	Q99527	GPER1
1	GPHN	5.7555E-76	2.10	3.3482E-71	8.2591E-75	Q9NQX3	GPHN
1	GPLD1	9.7326E-17	3.22	5.6618E-12	6.9428E-16	P80108	GPLD1
1	GPM6A	2.298E-117	-17.15	1.337E-112	4.385E-116	P51674	GPM6A
1	GPM6B	4.5312E-16	-2.43	2.636E-11	3.169E-15	Q13491	GPM6B
1	GPN3	2.2612E-21	1.65	1.3154E-16	1.7702E-20	Q9UHW5	GPN3
1	GPNMB	8.6102E-70	-7.59	5.0089E-65	1.1772E-68	Q14956	GPNMB
1	GPR1	7.4472E-34	-63.12	4.3323E-29	7.252E-33	P46091	GPR1
1	GPR107	0	-1.63	0	0	Q5VW38	GPR107
1	GPR108	0	-1.83	0	0	Q9NPR9	GPR108
1	GPR132	1.0093E-33	-9.86	5.8713E-29	9.81E-33	Q9UNW8	GPR132
1	GPR135	5.3952E-40	2.99	3.1386E-35	5.645E-39	Q8IZ08	GPR135
1	GPR137C	1.4349E-07	1.94	0.00834712	7.2126E-07	Q8N3F9	GPR137C
1	GPR150	9.8932E-05	-5.35	1	0.00041786	Q8NGU9	GPR150
1	GPR153	0	-2.02	0	0	Q6NV75	GPR153
1	GPR155	5.4684E-12	-1.75	3.1812E-07	3.3409E-11	Q7Z3F1	GPR155

1	GPR158	1.9712E-74	-3.85	1.1467E-69	2.7942E-73	Q5T848	GPR158
1	GPR160	3.285E-163	-4.57	1.911E-158	7.359E-162	Q9UJ42	GPR160
1	GPR162	2.5405E-79	37.25	1.4779E-74	3.7454E-78	Q16538	GPR162
1	GPR17	0.01149211	-4.15	1	0.04004926	Q13304	GPR17
1	GPR173	1.9658E-91	37.63	1.1436E-86	3.1881E-90	Q9NS66	GPR173
1	GPR179	3.2734E-06	-5.46	0.19042904	1.5222E-05	Q6PRD1	GPR179
1	GPR19	2.8221E-32	-2.48	1.6417E-27	2.6839E-31	Q15760	GPR19
1	GPR20	6.8325E-19	-31.03	3.9748E-14	5.0965E-18	Q99678	GPR20
1	GPR3	2.255E-150	9.52	1.312E-145	4.858E-149	P46089	GPR3
1	GPR37	2.9785E-24	-20.46	1.7327E-19	2.463E-23	O15354	GPR37
1	GPR37L1	7.4305E-08	-4.99	0.00432259	3.7868E-07	O60883	GPR37L1
1	GPR39	8.0296E-45	-2.44	4.6711E-40	8.9177E-44	O43194	GPR39
1	GPR52	0.00573247	-19.83	1	0.02070536	Q9Y2T5	GPR52
1	GPR61	4.8743E-14	-2.95	2.8356E-09	3.1936E-13	Q9BZJ8	GPR61
1	GPR63	6.2025E-27	-2.09	3.6082E-22	5.3854E-26	Q9BZJ6	GPR63
1	GPR68	1.9063E-20	-3.40	1.109E-15	1.4647E-19	Q15743	GPR68
1	GPR75	5.9433E-16	2.30	3.4574E-11	4.1431E-15	O95800	GPR75
1	GPR83	5.5519E-11	5.00	3.2297E-06	3.2568E-10	Q9NYM4	GPR83
1	GPR84	1.197E-08	-21.99	0.00069632	6.3556E-08	Q9NQS5	GPR84
1	GPR85	5.192E-137	-10.43	3.02E-132	1.076E-135	P60893	GPR85
1	GPR87	0	-10.54	0	0	Q9BY21	GPR87
1	GPR89B	0	-2.17	0	0	P0CG08	GPR89B
1	GPRASP1	6.2093E-05	-1.68	1	0.00026599	Q5JY77	GPRASP1
1	GPRC5C	9.797E-125	-6.59	5.7E-120	1.936E-123	Q9NQ84	GPRC5C
1	GPRC6A	4.4382E-43	-7.36	2.5819E-38	4.8359E-42	Q5T6X5	GPRC6A
1	GPRIN1	2.0608E-88	12.24	1.1989E-83	3.2694E-87	Q7Z2K8	GPRIN1
1	GPRIN2	1.5994E-25	-33.04	9.3044E-21	1.3546E-24	O60269	GPRIN2
1	GPRIN3	3.0285E-11	-17.26	1.7618E-06	1.7979E-10		
1	GPS1	7.3027E-39	1.71	4.2483E-34	7.5417E-38	Q13098	GPS1
1	GPS2	7.6381E-76	2.03	4.4434E-71	1.0944E-74	Q13227	GPS2
1	GPT	0.00058783	-1.84	1	0.00233567	P24298	GPT
1	GPT2	1.2637E-16	1.70	7.3514E-12	8.987E-16	Q8TD30	GPT2
1	GPX2	2.302E-189	-6.73	1.339E-184	5.584E-188	P18283	GPX2
1	GPX3	8.433E-195	-6.25	4.906E-190	2.092E-193	P22352	GPX3
1	GPX7	0.00472727	-5.82	1	0.01724381	Q96SL4	GPX7
1	GPX8	5.731E-101	2.96	3.3342E-96	9.917E-100	Q8TED1	GPX8
1	GRAMD1A	9.699E-176	29.92	5.642E-171	2.26E-174	Q96CP6	GRAMD1A
1	GRAMD1C	1.248E-121	-4.15	7.26E-117	2.435E-120	Q8IYS0	GRAMD1C
1	GRAMD2	1.973E-169	-8.40	1.148E-164	4.511E-168		
1	GRAMD4	0	-4.14	0	0	Q6IC98	GRAMD4
1	GRASP	1.1639E-18	4.49	6.7708E-14	8.6395E-18	Q7Z6J2	GRASP
1	GRB10	0	87.94	0	0	Q13322	GRB10
1	GRB14	3.156E-184	-12.55	1.836E-179	7.526E-183	Q14449	GRB14
1	GRB7	1.0353E-94	-16.28	6.0227E-90	1.7193E-93	Q14451	GRB7
1	GREB1L	6.1081E-52	-3.98	3.5534E-47	7.28E-51	Q9C091	GREB1L
1	GRHL1	2.8647E-64	-6.77	1.6665E-59	3.7746E-63	Q9NZI5	GRHL1
1	GRHL2	0	-12.61	0	0	Q6ISB3	GRHL2

1	GRHL3	1.087E-197	-21.84	6.322E-193	2.72E-196	Q8TE85	GRHL3
1	GRHPR	5.0706E-80	1.83	2.9498E-75	7.523E-79	Q9UBQ7	GRHPR
1	GRIA2	3.5384E-53	-10.59	2.0584E-48	4.2705E-52	P42262	GRIA2
1	GRIA4	0.00754249	-1.50	1	0.02689077	P48058	GRIA4
1	GRID2IP	2.2117E-10	-3.51	1.2866E-05	1.2675E-09	A4D2P6	GRID2IP
1	GRIK1	5.976E-42	20.01	3.4765E-37	6.4225E-41	P39086	GRIK1
1	GRIK1-AS1	9.8987E-06	20.47	0.5758488	4.4737E-05		
1	GRIK2	8.4338E-09	-2.61	0.00049063	4.5074E-08	Q13002	GRIK2
1	GRIN2D	0	-9.93	0	0	O15399	GRIN2D
1	GRIN3A	1.4528E-08	2.61	0.00084516	7.6903E-08	Q8TCU5	GRIN3A
1	GRIN3B	2.6729E-09	-4.98	0.0001555	1.4629E-08	O60391	GRIN3B
1	GRIP1	1.077E-182	-4.32	6.263E-178	2.555E-181	Q9Y3R0	GRIP1
1	GRIPAP1	0	-2.01	0	0	Q4V328	GRIPAP1
1	GRK2	0	-3.50	0	0	P25098	GRK2
1	GRK3	8.1625E-32	1.74	4.7484E-27	7.7098E-31	P35626	GRK3
1	GRK5	4.5265E-08	1.56	0.00263327	2.3345E-07	P34947	GRK5
1	GRK6	9.0911E-72	1.90	5.2887E-67	1.2628E-70	P43250	GRK6
1	GRK7	2.765E-25	-6.19	1.6085E-20	2.3332E-24	Q8WTQ7	GRK7
1	GRM1	2.1515E-05	-4.76	1	9.5088E-05	Q13255	GRM1
1	GRM3	1.6654E-06	-2.06	0.09688539	7.8974E-06	Q14832	GRM3
1	GRM5	7.7713E-12	-6.82	4.5209E-07	4.7225E-11	P41594	GRM5
1	GRM5-AS1	0.00072079	-5.57	1	0.00284087		
1	GRM8	0.00901451	-1.86	1	0.03182101	O00222	GRM8
1	GRPEL2	2.8242E-16	1.66	1.643E-11	1.9891E-15	Q8TAA5	GRPEL2
1	GRPR	1.512E-188	8.42	8.796E-184	3.66E-187	P30550	GRPR
1	GRTP1	1.054E-219	-6.35	6.129E-215	2.778E-218	Q5TC63	GRTP1
1	GRTP1-AS1	0.00140517	-26.91	1	0.00539816		
1	GRWD1	1.71E-106	2.04	9.947E-102	3.062E-105	Q9BQ67	GRWD1
1	GRXCR2	2.016E-07	-8.14	0.01172771	1.0043E-06	A6NFK2	GRXCR2
1	GS1-114I9.3	1.398E-196	-5.49	8.133E-192	3.49E-195		
1	GS1-124K5.10	3.0441E-05	1.89	1	0.00013322		
1	GS1-124K5.12	0	-1.67	0	0		
1	GS1-259H13.2	5.5341E-16	3.07	3.2194E-11	3.8616E-15		
1	GS1-279B7.1	0.00623659	-3.20	1	0.02242873		
1	GS1-358P8.4	5.5227E-27	-2.69	3.2128E-22	4.8017E-26		
1	GS1-393G12.1	0.00928825	-7.05	1	0.03274162		
1	GS1-393G12.1	6.5099E-11	4.25	3.787E-06	3.8091E-10		
1	GS1-590J6.3	0.00181951	-3.75	1	0.00691547		
1	GSAP	1.9323E-40	-3.36	1.1241E-35	2.0324E-39	A4D1B5	GSAP
1	GSDMB	0	-2.25	0	0	Q8TAX9	GSDMB
1	GSDMC	1.517E-06	-6.17	0.08825285	7.2102E-06	Q9BYG8	GSDMC
1	GSDMD	0	-1.94	0	0	P57764	GSDMD
1	GSE1	0	-2.48	0	0	Q14687	GSE1
1	GSG1L	3.2804E-09	3.89	0.00019083	1.7878E-08	Q6UXU4	GSG1L
1	GSG2	5.3435E-18	1.61	3.1085E-13	3.9135E-17	Q8TF76	GSG2
1	GSK3A	3.1222E-44	1.53	1.8163E-39	3.4471E-43	P49840	GSK3A
1	GSKIP	4.8925E-63	2.54	2.8462E-58	6.3815E-62	Q9P0R6	GSKIP

1	GSN	0	-4.29	0	0	P06396	GSN
1	GSR	0	-2.74	0	0	P00390	GSR
1	GSTCD	0	-1.76	0	0	Q8NEC7	GSTCD
1	GSTM1	1.7306E-44	3.15	1.0067E-39	1.9147E-43	P09488	GSTM1
1	GSTM2	0.00724425	-2.33	1	0.02586711	P28161	GSTM2
1	GSTM3	2.5319E-18	2.19	1.4729E-13	1.8659E-17	P21266	GSTM3
1	GSTO1	2.5896E-25	1.71	1.5065E-20	2.1861E-24	P78417	GSTO1
1	GSTO2	0	-9.41	0	0	Q9H4Y5	GSTO2
1	GSTP1	0	-2.82	0	0	P09211	GSTP1
1	GSTT2	1.093E-121	19.12	6.359E-117	2.134E-120	P0CG29	GSTT2
1	GSTT2B	4.0194E-60	4.05	2.3382E-55	5.1266E-59	P0CG30	GSTT2B
1	GSTZ1	6.6609E-55	2.24	3.8749E-50	8.1698E-54	O43708	GSTZ1
1	GTF2A1	8.0149E-54	2.26	4.6626E-49	9.7503E-53	P52655	GTF2A1
1	GTF2E2	0	-1.55	0	0	P29084	GTF2E2
1	GTF2H2	1.016E-121	4.22	5.909E-117	1.984E-120	Q13888	GTF2H2
1	GTF2H2B	5.4261E-82	3.47	3.1566E-77	8.1819E-81		
1	GTF2H2C	6.355E-113	2.25	3.697E-108	1.184E-111	Q6P1K8	GTF2H2C
1	GTF2H5	4.9924E-27	1.68	2.9043E-22	4.3471E-26	Q6ZYL4	GTF2H5
1	GTF2I	0	-1.66	0	0	P78347	GTF2I
1	GTF2IP1	1.4937E-24	2.39	8.6895E-20	1.2444E-23		
1	GTF2IP13	1.0319E-17	-2.62	6.0029E-13	7.513E-17		
1	GTF2IP14	1.5676E-08	-3.91	0.00091195	8.2845E-08		
1	GTF2IP20	0	-2.05	0	0		
1	GTF2IP23	3.0337E-05	-2.84	1	0.00013278		
1	GTF2IP5	0.00085316	-2.86	1	0.00334243		
1	GTF2IP6	0.00039151	-5.13	1	0.00157693		
1	GTF2IRD1	0	-1.67	0	0	Q9UHL9	GTF2IRD1
1	GTF2IRD2	5.9669E-05	-1.71	1	0.00025591	Q86UP8	GTF2IRD2
1	GTF2IRD2B	2.673E-13	-1.96	1.555E-08	1.7078E-12	Q6EKJ0	GTF2IRD2B
1	GTF3A	2.4754E-73	1.88	1.44E-68	3.4775E-72	Q92664	GTF3A
1	GTF3C6	9.3724E-87	2.65	5.4523E-82	1.4672E-85	Q969F1	GTF3C6
1	GTPBP3	1.8539E-33	1.67	1.0785E-28	1.7933E-32	Q969Y2	GTPBP3
1	GTSE1	1.2458E-40	1.76	7.2473E-36	1.3129E-39	Q9NYZ3	GTSE1
1	GTSE1-AS1	0.00710699	-1.75	1	0.02539413		
1	GUCY1A3	1.4853E-85	-21.74	8.6404E-81	2.3029E-84	Q02108	GUCY1A3
1	GUCY1B2	2.5891E-21	-2.88	1.5062E-16	2.025E-20	O75343	GUCY1B2
1	GUCY1B3	4.467E-101	-15.07	2.5985E-96	7.738E-100	Q02153	GUCY1B3
1	GUCY2C	2.336E-123	-11.17	1.359E-118	4.595E-122	P25092	GUCY2C
1	GUCY2D	2.9702E-06	-4.94	0.17278631	1.3854E-05	Q02846	GUCY2D
1	GUCY2EP	1.2108E-21	-50.20	7.044E-17	9.5331E-21		
1	GULP1	3.7307E-55	2.86	2.1703E-50	4.5854E-54	Q9UBP9	GULP1
1	GUSBP1	0	-2.15	0	0	Q15486	GUSBP1
1	GUSBP2	5.8554E-05	-1.71	1	0.00025132		
1	GUSBP5	1.2767E-21	-13.43	7.427E-17	1.0042E-20		
1	GVINP1	1.2908E-08	4.17	0.0007509	6.8444E-08	Q7Z2Y8	GVINP1
1	GVQW1	1.2433E-07	1.91	0.00723259	6.2669E-07	Q8N7I0	GVQW1
1	GVQW2	9.7868E-07	-1.83	0.05693378	4.7018E-06		

1	GXYLT1P4	3.068E-10	-3.89	1.7848E-05	1.7465E-09		
1	GXYLT2	0.00042669	-1.53	1	0.00171367	A0PJZ3	GXYLT2
1	GYG1	0	-1.63	0	0	P46976	GYG1
1	GYPE	0.00409511	-8.82	1	0.0150492	P15421	GYPE
1	GYS2	2.2731E-16	7.80	1.3223E-11	1.6036E-15	P54840	GYS2
1	GZF1	0	-1.64	0	0	Q9H116	GZF1
1	H19	2.624E-200	-19.33	1.527E-195	6.627E-199		
1	H1F0	0	-2.45	0	0	P07305	H1F0
1	H1FX	0	-1.67	0	0	Q92522	H1FX
1	H2AFB1	0.00221501	24.19	1	0.00834127	P0C5Y9	H2AFB1
1	H2AFV	0	-1.55	0	0	Q71UI9	H2AFV
1	H2AFX	4.5967E-19	1.59	2.6741E-14	3.4402E-18	P16104	H2AFX
1	H2BFS	4.8138E-05	-3.15	1	0.00020777	P57053	H2BFS
1	H3F3A	0	-1.72	0	0	P84243	H3F3A
1	H6PD	0	-5.00	0	0	O95479	H6PD
1	HAAO	1.4197E-10	-3.89	8.2588E-06	8.2055E-10	P46952	HAAO
1	HABP4	2.184E-131	3.39	1.27E-126	4.42E-130	Q5JVS0	HABP4
1	HACD4	5.27E-208	-7.16	3.066E-203	1.354E-206	Q5VWC8	HACD4
1	HACL1	1.3908E-33	1.83	8.0908E-29	1.3491E-32	Q9UJ83	HACL1
1	HADH	0	-1.65	0	0	Q16836	HADH
1	HAGH	1.3709E-32	1.62	7.9748E-28	1.3104E-31	Q16775	HAGH
1	HAL	2.206E-168	-6.32	1.284E-163	5.026E-167	P42357	HAL
1	HAP1	2.787E-225	-17.05	1.621E-220	7.424E-224	P54257	HAP1
1	HAPLN1	1.309E-62	-6.83	7.6147E-58	1.7024E-61	P10915	HAPLN1
1	HAPLN3	4.1322E-09	-1.65	0.00024039	2.2393E-08	Q96S86	HAPLN3
1	HARS	2.226E-05	1.91	1	9.8258E-05	P12081	HARS
1	HAS2	0	116.11	0	0	Q92819	HAS2
1	HAS2-AS1	1.597E-134	34.85	9.29E-130	3.267E-133		
1	HAS3	0	-2.48	0	0	O00219	HAS3
1	HAT1	2.7262E-14	1.59	1.586E-09	1.8008E-13	O14929	HAT1
1	HAUS1	4.6863E-14	1.53	2.7262E-09	3.0728E-13	Q96CS2	HAUS1
1	HAUS2	6.7257E-32	1.62	3.9126E-27	6.3614E-31	Q9NVX0	HAUS2
1	HAUS7	7.534E-108	2.52	4.383E-103	1.36E-106	Q99871	HAUS7
1	HAVCR1	8.023E-09	57.06	0.00046673	4.2906E-08	Q96D42	HAVCR1
1	HBE1	2.829E-134	-19.03	1.646E-129	5.784E-133	P02100	HBE1
1	HBEGF	1.9163E-37	3.90	1.1148E-32	1.9438E-36	Q99075	HBEGF
1	HBG2	2.1848E-09	-5.33	0.0001271	1.1992E-08	P69892	HBG2
1	HBP1	0	-1.98	0	0	O60381	HBP1
1	HBS1L	6.1524E-28	1.61	3.5791E-23	5.4727E-27	Q9Y450	HBS1L
1	HCAR1	1.099E-237	-11.55	6.392E-233	2.972E-236	Q9BXC0	HCAR1
1	HCAR2	4.597E-214	-13.76	2.674E-209	1.198E-212	Q8TDS4	HCAR2
1	HCAR3	4.167E-123	-9.76	2.424E-118	8.181E-122	P49019	HCAR3
1	HCFC1R1	3.6655E-27	1.67	2.1324E-22	3.2028E-26	Q9NWW0	HCFC1R1
1	HCFC2	6.7268E-13	1.51	3.9133E-08	4.2452E-12	Q9Y5Z7	HCFC2
1	HCG11	2.6005E-82	-13.34	1.5128E-77	3.9315E-81		
1	HCG14	0.00108952	-7.15	1	0.00422997		
1	HCG20	0.01432174	-2.71	1	0.04938664		

1	HCG25	5.818E-05	-3.26	1	0.00024977		
1	HCG27	1.3623E-08	-1.92	0.00079248	7.2188E-08		
1	HCG4B	4.0962E-09	-5.97	0.00023829	2.2206E-08		
1	HCG4P7	0.00012812	-3.12	1	0.00053633		
1	HCG9	5.2743E-05	4.84	1	0.00022711		
1	HCLS1	0.00123011	1.65	1	0.00475516	P14317	HCLS1
1	HCN2	2.625E-183	23.92	1.527E-178	6.243E-182	Q9UL51	HCN2
1	HCP5	1.8318E-61	-2.95	1.0656E-56	2.3602E-60	Q6MZN7	HCP5
1	HCRTR2	2.5702E-20	-15.75	1.4952E-15	1.9702E-19	O43614	HCRTR2
1	HDAC11	5.3748E-45	2.65	3.1268E-40	5.9865E-44	Q96DB2	HDAC11
1	HDAC5	0	185.99	0	0	Q9UQL6	HDAC5
1	HDAC7	0	-1.64	0	0	Q8WUI4	HDAC7
1	HDAC9	3.741E-100	-17.04	2.1762E-95	6.433E-99	Q9UKV0	HDAC9
1	HDGF	0	-1.82	0	0	P51858	HDGF
1	HDGFP1	7.3086E-20	-16.00	4.2517E-15	5.5592E-19		
1	HDHD5	1.2507E-88	1.81	7.2757E-84	1.9879E-87	Q9BXW7	HDHD5
1	HEATR4	1.8031E-38	-4.30	1.0489E-33	1.8539E-37		
1	HEATR5A	9.75E-25	1.62	5.672E-20	8.1435E-24		
1	HEBP1	3.4376E-97	2.35	1.9998E-92	5.7999E-96	Q9NRV9	HEBP1
1	HECA	0	-1.64	0	0	Q9UBI9	HECA
1	HECTD3	3.6718E-98	1.83	2.136E-93	6.2403E-97	Q5T447	HECTD3
1	HECW1	2.223E-158	-7.69	1.293E-153	4.918E-157	Q76N89	HECW1
1	HECW2	6.5053E-26	-6.01	3.7844E-21	5.5441E-25	Q9P2P5	HECW2
1	HEG1	1.7867E-22	1.64	1.0394E-17	1.4278E-21	Q9ULI3	HEG1
1	HEIH	5.4295E-45	2.42	3.1586E-40	6.0462E-44		
1	HELLPAR	0.0011281	1.56	1	0.00437421		
1	HEMGN	0.00418885	-17.84	1	0.01537332	Q9BXL5	HEMGN
1	HENMT1	1.644E-193	-8.98	9.564E-189	4.056E-192	Q5T8I9	HENMT1
1	HEPHL1	5.1082E-41	-12.34	2.9716E-36	5.4099E-40	Q6MZM0	HEPHL1
1	HERC2P10	4.7212E-05	1.93	1	0.00020398		
1	HERC2P7	0.00048998	2.34	1	0.00195889		
1	HERC6	6.2728E-96	-4.20	3.6491E-91	1.0492E-94	Q8IVU3	HERC6
1	HERPUD2	0	-2.71	0	0	Q9BSE4	HERPUD2
1	HES1	0	-2.02	0	0	Q14469	HES1
1	HES2	2.633E-52	-8.23	1.5317E-47	3.1504E-51	Q9Y543	HES2
1	HES6	1.152E-111	6.17	6.703E-107	2.135E-110	Q96HZ4	HES6
1	HEXA-AS1	0.00414786	-2.12	1	0.01523537		
1	HEYL	1.6749E-05	-12.50	0.97435543	7.4532E-05	Q9NQ87	HEYL
1	HFE	0	-2.73	0	0	Q30201	HFE
1	HGD	0.00779617	2.55	1	0.02775098	Q93099	HGD
1	HGF	5.4814E-33	-17.39	3.1887E-28	5.2724E-32	P14210	HGF
1	HGS	2.3635E-93	1.89	1.3749E-88	3.8862E-92	O14964	HGS
1	HGSNAT	0	-2.54	0	0	Q68CP4	HGSNAT
1	HHAT	1.9594E-18	2.40	1.1399E-13	1.4477E-17	Q5VTY9	HHAT
1	HHIP	6.3094E-10	-3.70	3.6704E-05	3.5449E-09	Q96QV1	HHIP
1	HHIP-AS1	4.5798E-15	-5.17	2.6642E-10	3.0976E-14		
1	HHIPL1	5.407E-09	-15.46	0.00031454	2.9154E-08	Q96JK4	HHIPL1

1	HHIPL2	1.1072E-24	-4.12	6.4411E-20	9.2372E-24	Q6UWX4	HHIPL2
1	HHLA1	3.6675E-06	-3.87	0.213356	1.6995E-05	C9JL84	HHLA1
1	HHLA2	0.00016102	-6.60	1	0.00066908	Q9UM44	HHLA2
1	HHLA3	4.548E-41	-2.40	2.6457E-36	4.8218E-40	Q9XRX5	HHLA3
1	HIC1	2.5351E-26	3.27	1.4748E-21	2.1784E-25	Q14526	HIC1
1	HIC2	6.9726E-84	3.75	4.0563E-79	1.0683E-82	Q96JB3	HIC2
1	HID1	4.2111E-48	3.59	2.4497E-43	4.8491E-47	Q8IV36	HID1
1	HIGD1A	1.8719E-31	2.20	1.089E-26	1.7592E-30	Q9Y241	HIGD1A
1	HIGD2A	6.4085E-32	1.74	3.7281E-27	6.0639E-31	Q9BW72	HIGD2A
1	HINT1	1.0809E-42	1.99	6.2881E-38	1.1725E-41	P49773	HINT1
1	HINT2	2.1125E-14	1.56	1.2289E-09	1.3997E-13	Q9BX68	HINT2
1	HIP1R	0	-2.75	0	0	O75146	HIP1R
1	HIPK1	2.451E-74	1.86	1.4258E-69	3.4717E-73	Q86Z02	HIPK1
1	HIPK1-AS1	2.8197E-06	-2.95	0.16403529	1.3177E-05		
1	HIRA	1.0779E-83	1.73	6.2705E-79	1.6467E-82	P54198	HIRA
1	HIST1H2AE	0.00014641	-2.16	1	0.0006104		
1	HIST1H2AG	4.5416E-06	-1.72	0.26420047	2.0927E-05	P0C0S8	HIST1H2AG
1	HIST1H2BC	2.1154E-08	-1.83	0.00123059	1.1106E-07	P62807	HIST1H2BC
1	HIST1H2BD	9.6212E-08	-1.54	0.00559701	4.8746E-07	P58876	HIST1H2BD
1	HIST1H2BJ	4.6769E-10	-1.76	2.7207E-05	2.6412E-09	P06899	HIST1H2BJ
1	HIST1H2BK	0	-2.16	0	0	O60814	HIST1H2BK
1	HIST1H2BN	9.4332E-09	-2.10	0.00054876	5.0322E-08	Q99877	HIST1H2BN
1	HIST1H3F	0.0008266	3.82	1	0.0032421		
1	HIST1H3G	0.00035467	-1.59	1	0.00143451		
1	HIST1H3H	3.2996E-09	-1.77	0.00019195	1.7982E-08		
1	HIST1H4C	2.0884E-05	6.28	1	9.2367E-05		
1	HIST1H4I	2.1419E-05	-2.01	1	9.4675E-05		
1	HIST1H4J	6.1396E-09	1.99	0.00035716	3.3014E-08		
1	HIST2H2AA3	0.00236824	-3.03	1	0.00889297	Q6FI13	HIST2H2AA3
1	HIST2H2BD	2.1734E-26	-2.73	1.2644E-21	1.8698E-25	Q6DRA6	HIST2H2BD
1	HIST2H2BE	9.1567E-38	-3.94	5.3268E-33	9.3191E-37	Q16778	HIST2H2BE
1	HIST2H2BF	6.3496E-70	-4.56	3.6938E-65	8.6934E-69	Q5QNW6	HIST2H2BF
1	HIST2H3C	0.00197937	-2.10	1	0.00749171		
1	HIST2H4B	0.00346968	-2.91	1	0.01283922		
1	HIST3H2A	1.1706E-58	-3.05	6.8097E-54	1.4788E-57	Q7L7L0	HIST3H2A
1	HIST3H2BB	2.7621E-21	-4.63	1.6068E-16	2.1591E-20	Q8N257	HIST3H2BB
1	HIVEP1	8.6548E-28	1.72	5.0349E-23	7.6669E-27	P15822	HIVEP1
1	HIVEP2	0	-1.75	0	0	P31629	HIVEP2
1	HIVEP3	2.118E-201	8.40	1.232E-196	5.368E-200	Q5T1R4	HIVEP3
1	HK1	1.5097E-64	1.69	8.7827E-60	1.9938E-63	P19367	HK1
1	HK2	4.6621E-51	1.54	2.7121E-46	5.5046E-50	P52789	HK2
1	HKDC1	7.9498E-09	-3.28	0.00046247	4.2526E-08	Q2TB90	HKDC1
1	HLA-B	0	-2.70	0	0	Q31612	HLA-B
1	HLA-C	0	-3.03	0	0	Q95604	HLA-C
1	HLA-DMA	1.1147E-45	-3.31	6.4848E-41	1.2514E-44	P28067	HLA-DMA
1	HLA-DMB	1.1644E-14	-7.18	6.7738E-10	7.7779E-14	P28068	HLA-DMB
1	HLA-DOB	4.7221E-05	-2.28	1	0.000204	P13765	HLA-DOB

1	HLA-DPA1	1.2634E-17	35.36	7.3494E-13	9.1845E-17	P20036	HLA-DPA1
1	HLA-DPB1	1.1781E-94	39.53	6.8535E-90	1.9554E-93	P04440	HLA-DPB1
1	HLA-DQB1	1.409E-105	-28.09	8.2E-101	2.51E-104	P01920	HLA-DQB1
1	HLA-F	6.445E-99	6.13	3.7494E-94	1.1011E-97	P30511	HLA-F
1	HLA-F-AS1	1.624E-130	42.05	9.445E-126	3.279E-129		
1	HLA-G	0.00411679	-19.71	1	0.01512336	P17693	HLA-G
1	HLA-H	3.7157E-19	-1.97	2.1615E-14	2.7859E-18	P01893	HLA-H
1	HLA-K	4.465E-127	-7.64	2.598E-122	8.917E-126		
1	HLA-V	3.3274E-08	-5.47	0.00193568	1.728E-07		
1	HLX	8.1479E-15	-2.80	4.74E-10	5.4696E-14	Q14774	HLX
1	HLX-AS1	5.0234E-10	-5.18	2.9223E-05	2.8334E-09		
1	HMBOX1	0	-2.17	0	0	Q6NT76	HMBOX1
1	HMCN1	4.1227E-53	-3.33	2.3983E-48	4.9737E-52	Q96RW7	HMCN1
1	HMGA1P4	2.566E-10	-4.29	1.4928E-05	1.4662E-09		
1	HMGB1P38	0.005325	-9.42	1	0.01931036		
1	HMGB3	4.209E-101	2.29	2.4484E-96	7.296E-100	O15347	HMGB3
1	HMGCLL1	1.709E-11	-18.34	9.9421E-07	1.026E-10	Q8TB92	HMGCLL1
1	HMGCS1	0	-3.36	0	0	Q01581	HMGCS1
1	HMGN2	2.1701E-47	1.93	1.2624E-42	2.4763E-46	P05204	HMGN2
1	HMGN2P39	0.00039245	-30.91	1	0.00158051		
1	HMGN2P46	0.0016291	-1.76	1	0.00622062		
1	HMGN3-AS1	4.5364E-08	-2.03	0.00263903	2.3394E-07		
1	HMGN5	1.9086E-54	-3.76	1.1103E-49	2.3326E-53	P82970	HMGN5
1	HMOX1	1.1547E-30	3.38	6.7173E-26	1.0731E-29	P09601	HMOX1
1	HMSD	3.7751E-26	-17.84	2.1961E-21	3.2348E-25	A8MTL9	HMSD
1	HN1L	2.294E-104	1.66	1.334E-99	4.062E-103		
1	HNF1A-AS1	2.7175E-07	-9.46	0.01580851	1.3449E-06		
1	HNF1B	6.7195E-38	1.77	3.909E-33	6.8554E-37	P35680	HNF1B
1	HNF4G	6.2782E-37	-3.47	3.6523E-32	6.3331E-36	Q14541	HNF4G
1	HNMT	2.072E-126	-7.47	1.205E-121	4.125E-125	P50135	HNMT
1	HNRNPA0	1.9942E-56	1.62	1.1601E-51	2.4762E-55	Q13151	HNRNPA0
1	HNRNPA1	2.1698E-47	1.64	1.2623E-42	2.4763E-46	P09651	HNRNPA1
1	HNRNPA1P46	9.6088E-13	-6.32	5.5898E-08	6.0319E-12		
1	HNRNPA3	1.919E-106	1.63	1.116E-101	3.434E-105	P51991	HNRNPA3
1	HNRNPAB	6.0774E-14	2.24	3.5355E-09	3.9702E-13	Q99729	HNRNPAB
1	HNRNPF	1.055E-106	1.99	6.135E-102	1.891E-105	P52597	HNRNPF
1	HNRNPH1	4.153E-114	2.25	2.416E-109	7.781E-113	P31943	HNRNPH1
1	HNRNPM	6.2532E-58	1.52	3.6377E-53	7.8433E-57	P52272	HNRNPM
1	HNRNPUL2	0	-1.76	0	0	Q1KMD3	HNRNPUL2
1	HOGA1	6.5727E-53	-9.74	3.8236E-48	7.9114E-52	Q86XE5	HOGA1
1	HOMER2	0	-2.51	0	0	Q9NSB8	HOMER2
1	HOMER2P1	1.3067E-06	-26.91	0.07601691	6.235E-06		
1	HOMER3	1.6077E-71	2.06	9.3528E-67	2.2285E-70	Q9NSC5	HOMER3
1	HOMEZ	0	-2.41	0	0	Q8IX15	HOMEZ
1	HOOK1	1.521E-225	-9.99	8.848E-221	4.057E-224	Q9UJC3	HOOK1
1	HOOK2	0	-2.82	0	0	Q96ED9	HOOK2
1	HOPX	4.4357E-06	-4.04	0.25804048	2.0449E-05	Q9BPY8	HOPX

1	HORMAD1	2.078E-110	-18.31	1.209E-105	3.816E-109	Q86X24	HORMAD1
1	HORMAD2-AS	0.0001236	2.12	1	0.00051803		
1	HOTAIRM1	0	-1.87	0	0		
1	HOTTIP	1.9295E-19	-3.18	1.1225E-14	1.4545E-18		
1	HOXA-AS2	5.435E-146	-3.96	3.162E-141	1.159E-144		
1	HOXA1	8.0451E-43	-2.25	4.6802E-38	8.7447E-42	P49639	HOXA1
1	HOXA10	0	-1.96	0	0	P31260	HOXA10
1	HOXA11	4.0099E-53	-6.15	2.3327E-48	4.8386E-52	P31270	HOXA11
1	HOXA11-AS	5.19E-155	-8.17	3.019E-150	1.135E-153		
1	HOXA2	5.5277E-17	-2.23	3.2157E-12	3.9602E-16	O43364	HOXA2
1	HOXA4	7.4739E-17	1.96	4.3479E-12	5.344E-16	Q00056	HOXA4
1	HOXA7	7.5388E-50	-2.62	4.3856E-45	8.8153E-49	P31268	HOXA7
1	HOXB-AS3	1.1976E-26	3.81	6.9669E-22	1.0352E-25		
1	HOXB1	1.6972E-32	-14.56	9.8732E-28	1.6202E-31	P14653	HOXB1
1	HOXB13	3.7023E-49	4.53	2.1538E-44	4.3041E-48	Q92826	HOXB13
1	HOXB2	1.0539E-14	1.60	6.131E-10	7.0479E-14	P14652	HOXB2
1	HOXB3	2.313E-35	2.21	1.3455E-30	2.2907E-34	P14651	HOXB3
1	HOXB4	1.0655E-46	6.03	6.1983E-42	1.2075E-45	P17483	HOXB4
1	HOXB5	1.3184E-41	2.73	7.6696E-37	1.4099E-40	P09067	HOXB5
1	HOXB6	4.521E-116	4.83	2.63E-111	8.562E-115	P17509	HOXB6
1	HOXB7	7.276E-22	1.51	4.2328E-17	5.7471E-21	P09629	HOXB7
1	HOXB8	1.9071E-70	24.22	1.1094E-65	2.6246E-69	P17481	HOXB8
1	HOXB9	4.9449E-53	4.23	2.8767E-48	5.9608E-52	P17482	HOXB9
1	HOXC-AS1	3.3085E-21	25.86	1.9247E-16	2.5814E-20		
1	HOXC-AS2	0.00219707	8.05	1	0.0082807		
1	HOXC10	1.3295E-25	24.11	7.734E-21	1.1266E-24	Q9NYD6	HOXC10
1	HOXC4	6.806E-202	21.91	3.959E-197	1.727E-200	P09017	HOXC4
1	HOXC5	1.1372E-36	727.41	6.6153E-32	1.1443E-35	Q00444	HOXC5
1	HOXC6	3.3382E-79	16.98	1.942E-74	4.9189E-78	P09630	HOXC6
1	HOXC8	1.497E-112	44.41	8.709E-108	2.786E-111	P31273	HOXC8
1	HOXC9	3.27E-198	15.47	1.902E-193	8.196E-197	P31274	HOXC9
1	HOXD-AS2	2.033E-109	21.59	1.183E-104	3.711E-108		
1	HOXD1	0.00023863	2.17	1	0.00097891	Q9GZZ0	HOXD1
1	HOXD10	2.0158E-07	-3.64	0.01172644	1.0042E-06	P28358	HOXD10
1	HOXD13	6.597E-07	-5.78	0.0383776	3.1981E-06	P35453	HOXD13
1	HOXD3	5.2478E-18	42.27	3.0529E-13	3.8444E-17	P31249	HOXD3
1	HOXD4	3.943E-243	302.06	2.294E-238	1.073E-241	P09016	HOXD4
1	HOXD8	7.313E-158	8.16	4.254E-153	1.615E-156	P13378	HOXD8
1	HOXD9	1.352E-125	4.46	7.867E-121	2.68E-124	P28356	HOXD9
1	HPCAL1	2.4232E-72	2.24	1.4097E-67	3.3797E-71	P37235	HPCAL1
1	HPDL	3.9038E-26	-7.39	2.271E-21	3.3431E-25	Q96IR7	HPDL
1	HPGD	1.4016E-24	-22.23	8.1534E-20	1.1678E-23	P15428	HPGD
1	HPN-AS1	3.4307E-07	-12.16	0.01995759	1.6882E-06		
1	HPS3	0	-2.19	0	0	Q969F9	HPS3
1	HPS5	7.4125E-28	1.54	4.3122E-23	6.5754E-27	Q9UPZ3	HPS5
1	HPSE	5.144E-191	-10.09	2.992E-186	1.257E-189	Q9Y251	HPSE
1	HPSE2	0.00504947	-2.78	1	0.0183627	Q8WWQ2	HPSE2

1	HPX	2.2805E-06	2.64	0.13266699	1.0721E-05	P02790	HPX
1	HR	0	-12.11	0	0	O43593	HR
1	HRAS	1.1193E-33	3.26	6.5111E-29	1.0874E-32	P01112	HRAS
1	HRASLS	6.052E-06	-7.65	0.35207144	2.7724E-05	Q9HDD0	HRASLS
1	HRAT92	1.0424E-12	-6.74	6.0638E-08	6.5336E-12		
1	HRCT1	5.1479E-11	-4.38	2.9947E-06	3.0262E-10	Q6UXD1	HRCT1
1	HRH4	0.00940099	3.63	1	0.03312495	Q9H3N8	HRH4
1	HRK	1.3038E-06	-7.01	0.07584956	6.2233E-06	O00198	HRK
1	HRNR	1.245E-32	-13.86	7.2427E-28	1.1906E-31	Q86YZ3	HRNR
1	HS1BP3-IT1	2.1651E-05	-2.51	1	9.5635E-05		
1	HS3ST1	0	-9.45	0	0	O14792	HS3ST1
1	HS3ST5	0.00283011	-6.72	1	0.01055377	Q8IZT8	HS3ST5
1	HS6ST1	0	-2.38	0	0	O60243	HS6ST1
1	HS6ST2	9.082E-160	-12.30	5.283E-155	2.019E-158	Q96MM7	HS6ST2
1	HSCB	1.5701E-44	1.94	9.1338E-40	1.7375E-43	Q8IWL3	HSCB
1	HSD11B1	0.00073291	-26.93	1	0.00288767	P28845	HSD11B1
1	HSD11B1L	3.7832E-09	2.24	0.00022008	2.0551E-08	Q7Z5J1	HSD11B1L
1	HSD11B2	2.3808E-45	-3.32	1.385E-40	2.6599E-44	P80365	HSD11B2
1	HSD17B10	6.2108E-93	2.51	3.6131E-88	1.0183E-91	Q99714	HSD17B10
1	HSD17B11	0	-2.27	0	0	Q8NBQ5	HSD17B11
1	HSD17B14	0.0108219	-1.99	1	0.0378269	Q9BPX1	HSD17B14
1	HSD17B3	2.4694E-06	-3.24	0.14365499	1.1581E-05	P37058	HSD17B3
1	HSD17B7	0	-2.25	0	0	P56937	HSD17B7
1	HSD17B7P2	1.7965E-34	2.09	1.0451E-29	1.7636E-33		
1	HSD17B8	4.464E-154	-6.86	2.597E-149	9.734E-153	Q92506	HSD17B8
1	HSD3B7	2.149E-20	1.70	1.2502E-15	1.65E-19	Q9H2F3	HSD3B7
1	HSD3BP5	1.584E-05	-2.49	0.92146226	7.0599E-05		
1	HSF1	0	-1.79	0	0	Q00613	HSF1
1	HSF2BP	3.4622E-14	-2.57	2.0141E-09	2.2792E-13	O75031	HSF2BP
1	HSF4	2.5375E-21	-2.22	1.4762E-16	1.9849E-20	Q9ULV5	HSF4
1	HSFX1	1.5204E-08	-3.23	0.0008845	8.0424E-08	Q9UBD0	HSFX1
1	HSFX2	0.00019471	-6.87	1	0.00080499		
1	HSH2D	1.556E-153	-31.61	9.052E-149	3.386E-152	Q96JZ2	HSH2D
1	HSP90AA1	5.1386E-82	2.52	2.9893E-77	7.7503E-81	P07900	HSP90AA1
1	HSP90AA5P	0.00176883	-10.08	1	0.00672857	Q58FG0	HSP90AA5P
1	HSPA12B	0.00040484	-1.70	1	0.00162927	Q96MM6	HSPA12B
1	HSPA1A	2.0299E-98	2.38	1.1809E-93	3.4549E-97	P0DMV8	HSPA1A
1	HSPA4	5.3657E-39	1.67	3.1215E-34	5.5542E-38	P34932	HSPA4
1	HSPA5	0	-1.60	0	0	P11021	HSPA5
1	HSPA6	5.257E-222	17.14	3.058E-217	1.394E-220	P17066	HSPA6
1	HSPA7	0.00065446	25.88	1	0.00258981	P48741	HSPA7
1	HSPA8	3.7175E-76	2.52	2.1626E-71	5.3451E-75	P11142	HSPA8
1	HSPA9	2.2087E-54	1.64	1.2849E-49	2.6971E-53	P38646	HSPA9
1	HSPB1	0	-2.57	0	0	P04792	HSPB1
1	HSPB6	0.00130661	-1.86	1	0.00503885	O14558	HSPB6
1	HSPB7	0.0006344	-25.31	1	0.00251433	Q9UBY9	HSPB7
1	HSPB8	7.8633E-14	-9.95	4.5744E-09	5.1202E-13	Q9UJY1	HSPB8

1	HSPD1	2.7583E-58	1.74	1.6046E-53	3.4724E-57	P10809	HSPD1
1	HSPD1P4	0.00010049	4.77	1	0.00042428		
1	HSPE1	1.711E-20	1.68	9.9537E-16	1.3156E-19	P61604	HSPE1
1	HSPG2	0	-2.68	0	0	P98160	HSPG2
1	HTATIP2	0	-2.65	0	0	Q9BUP3	HTATIP2
1	HTATSF1	6.2421E-29	1.60	3.6313E-24	5.643E-28	O43719	HTATSF1
1	HTR1B	1.784E-13	-8.80	1.0378E-08	1.1478E-12	P28222	HTR1B
1	HTR1D	3.8631E-75	23.63	2.2473E-70	5.5082E-74	P28221	HTR1D
1	HTR2C	8.326E-155	-8.14	4.843E-150	1.817E-153	P28335	HTR2C
1	HTR4	0.00041658	-7.63	1	0.00167465	Q13639	HTR4
1	HTR7	4.0608E-69	14.93	2.3623E-64	5.5246E-68	P34969	HTR7
1	HTRA1	0	-2.15	0	0	Q92743	HTRA1
1	HTRA2	8.2753E-34	1.76	4.8141E-29	8.0571E-33	O43464	HTRA2
1	HTRA3	0	-11.65	0	0	P83110	HTRA3
1	HTRA4	1.8825E-11	-11.92	1.0951E-06	1.1281E-10	P83105	HTRA4
1	HTT	0	-1.62	0	0	P42858	HTT
1	HTT-AS	0.00329963	-3.67	1	0.01222861		
1	HUS1	0	-1.90	0	0	O60921	HUS1
1	HVCN1	8.0502E-08	-3.29	0.00468314	4.0951E-07	Q96D96	HVCN1
1	HYAL1	1.235E-189	-7.70	7.184E-185	3.001E-188	Q12794	HYAL1
1	HYAL3	2.0868E-21	1.88	1.214E-16	1.635E-20	O43820	HYAL3
1	HYAL4	2.132E-11	-18.35	1.2403E-06	1.2738E-10	Q2M3T9	HYAL4
1	HYDIN	2.0773E-22	-10.95	1.2085E-17	1.6577E-21	Q4G0P3	HYDIN
1	HYDIN2	1.5739E-08	-4.60	0.0009156	8.316E-08		
1	HYI	1.949E-135	3.71	1.134E-130	4.009E-134	Q5T013	HYI
1	HYKK	4.2835E-20	1.99	2.4919E-15	3.2757E-19	A2RU49	HYKK
1	HYLS1	7.3995E-38	2.26	4.3046E-33	7.5439E-37	Q96M11	HYLS1
1	IAH1	0	-1.70	0	0	Q2TAA2	IAH1
1	IAPP	0.00942764	-18.12	1	0.03316063	P10997	IAPP
1	IARS2	0	-2.05	0	0	Q9NSE4	IARS2
1	IBA57	8.3284E-14	1.59	4.845E-09	5.4164E-13	Q5T440	IBA57
1	IBSP	3.8011E-06	-8.67	0.22112419	1.7601E-05	P21815	IBSP
1	ICA1	6.897E-288	-11.03	4.012E-283	1.931E-286	Q05084	ICA1
1	ICA1L	1.422E-33	3.36	8.2722E-29	1.3789E-32	Q8NDH6	ICA1L
1	ICAM1	5.6251E-16	1.62	3.2724E-11	3.9237E-15	P05362	ICAM1
1	ICAM2	9.466E-148	-12.55	5.507E-143	2.026E-146	P13598	ICAM2
1	ICAM3	1.329E-105	3.57	7.734E-101	2.368E-104	P32942	ICAM3
1	ICAM5	0	-1.71	0	0	Q9UMF0	ICAM5
1	ICK	4.369E-27	1.73	2.5416E-22	3.81E-26	Q9UPZ9	ICK
1	ICOSLG	8.5599E-20	-3.04	4.9796E-15	6.5076E-19	O75144	ICOSLG
1	ID1	0	-4.31	0	0	P41134	ID1
1	ID2	1.8977E-05	-1.72	1	8.4123E-05	Q02363	ID2
1	ID3	7.9565E-80	2.47	4.6286E-75	1.179E-78	Q02535	ID3
1	ID4	0.00042415	-7.09	1	0.00170379	P47928	ID4
1	IDH1-AS1	0.00860972	-1.55	1	0.03046974		
1	IDH2	0	-3.55	0	0	P48735	IDH2
1	IDI1	0	-2.06	0	0	Q13907	IDI1

1	IDNK	8.2823E-14	-1.68	4.8181E-09	5.3882E-13	Q5T6J7	IDNK
1	IDO1	0.00013177	-33.02	1	0.00055104	P14902	IDO1
1	IDS	6.4944E-76	4.70	3.7781E-71	9.3147E-75	B3KWA1, P22	IDS
1	IDUA	1.9526E-26	-2.18	1.1359E-21	1.6823E-25	P35475	IDUA
1	IER3	0	-1.51	0	0	P46695	IER3
1	IER3IP1	1.5857E-59	2.83	9.2246E-55	2.015E-58	Q9Y5U9	IER3IP1
1	IFFO1	1.2276E-94	13.69	7.1412E-90	2.0369E-93	Q0D2I5	IFFO1
1	IFFO2	0	-3.16	0	0	Q5TF58	IFFO2
1	IFI16	0	-3.61	0	0	Q16666	IFI16
1	IFI27	6.301E-126	-4.95	3.666E-121	1.251E-124	P40305	IFI27
1	IFI27L2	0	-1.74	0	0	Q9H2X8	IFI27L2
1	IFI30	0	-5.85	0	0	P13284	IFI30
1	IFI35	1.1102E-14	-1.54	6.4586E-10	7.4228E-14	P80217	IFI35
1	IFI44	0	-8.88	0	0	Q8TCB0	IFI44
1	IFI44L	8.354E-123	-12.95	4.86E-118	1.639E-121	Q53G44	IFI44L
1	IFI6	1.6895E-05	-1.52	0.98285601	7.5176E-05	P09912	IFI6
1	IFIH1	2.393E-172	-14.38	1.392E-167	5.514E-171	Q9BYX4	IFIH1
1	IFIT1	0	-2.90	0	0	P09914	IFIT1
1	IFIT3	1.1601E-33	-3.01	6.7489E-29	1.1267E-32	O14879	IFIT3
1	IFITM1	4.2128E-28	-18.89	2.4508E-23	3.7582E-27	P13164	IFITM1
1	IFITM10	1.6034E-35	-12.79	9.3274E-31	1.5928E-34	A6NMD0	IFITM10
1	IFNA1	0.01154484	-3.37	1	0.04022579	P01562	IFNA1
1	IFNA20P	0.00575073	-5.66	1	0.02076875		
1	IFNE	8.6056E-53	-4.14	5.0062E-48	1.0354E-51	Q86WN2	IFNE
1	IFNLR1	4.26E-37	-2.96	2.4782E-32	4.3047E-36	Q8IU57	IFNLR1
1	IFT27	0	-1.64	0	0	Q9BW83	IFT27
1	IFT43	1.8163E-30	1.50	1.0566E-25	1.682E-29	Q96FT9	IFT43
1	IFT52	7.5062E-81	2.29	4.3667E-76	1.122E-79	Q9Y366	IFT52
1	IFT74	1.6707E-19	1.59	9.7188E-15	1.2619E-18	Q96LB3	IFT74
1	IFT80	0	-1.94	0	0	Q9P2H3	IFT80
1	IGDCC4	1.2425E-10	-4.41	7.2279E-06	7.1998E-10	Q8TDY8	IGDCC4
1	IGF1R	0	-2.58	0	0	P08069	IGF1R
1	IGF2	3.35E-166	23.19	1.949E-161	7.571E-165	P01344	IGF2
1	IGF2BP1	1.701E-194	151.35	9.893E-190	4.213E-193	Q9NZI8	IGF2BP1
1	IGF2BP2-AS1	0.00053857	3.55	1	0.00214697		
1	IGFBP1	0.00418879	-19.71	1	0.01537332	P08833	IGFBP1
1	IGFBP2	0.00012549	-7.82	1	0.00052573	P18065	IGFBP2
1	IGFBP3	0	-3.12	0	0	P17936	IGFBP3
1	IGFBP6	0	-1.76	0	0	P24592	IGFBP6
1	IGFBP7	0	-1.64	0	0	Q16270	IGFBP7
1	IGFL1	4.9862E-20	-159.22	2.9007E-15	3.8062E-19	Q6UW32	IGFL1
1	IGFL1P1	7.102E-20	-8.91	4.1315E-15	5.4042E-19		
1	IGFL2-AS1	0	-11.46	0	0		
1	IGFN1	7.8796E-30	8.38	4.5839E-25	7.229E-29	Q86VF2	IGFN1
1	IGHV1-45	0.00231536	-21.55	1	0.00869891	A0A0A0MS14	IGHV1-45
1	IGIP	3.9016E-08	-1.73	0.00226972	2.0186E-07	A6NJ69	IGIP
1	IGKV1OR-2	2.702E-16	154.39	1.5718E-11	1.9032E-15		

1	IGKV1OR-3	0.00225089	8.71	1	0.00846762		
1	IGLL1	3.3383E-12	88.12	1.942E-07	2.0568E-11	P15814	IGLL1
1	IGLON5	0.01016941	3.14	1	0.03564684	A6NGN9	IGLON5
1	IGSF1	2.751E-159	-9.67	1.6E-154	6.106E-158	Q8N6C5	IGSF1
1	IGSF10	4.537E-191	6.18	2.64E-186	1.11E-189	Q6WRI0	IGSF10
1	IGSF11	6.4511E-63	-9.17	3.7529E-58	8.4089E-62	Q5DX21	IGSF11
1	IGSF23	2.1736E-13	-5.90	1.2645E-08	1.3944E-12	A1L1A6	IGSF23
1	IGSF3	0	-14.58	0	0	O75054	IGSF3
1	IGSF8	0	-1.63	0	0	Q969P0	IGSF8
1	IGSF9	0	-18.99	0	0	Q9P2J2	IGSF9
1	IHH	0.0018205	11.96	1	0.00691878	Q14623	IHH
1	IK	9.1945E-84	1.94	5.3488E-79	1.4061E-82	Q13123	IK
1	IKBIP	1.2298E-93	3.05	7.1541E-89	2.0261E-92	Q70UQ0	IKBIP
1	IKBKE	3.2164E-68	-3.11	1.8711E-63	4.3504E-67	Q14164	IKBKE
1	IKZF2	4.1573E-59	-6.55	2.4185E-54	5.2667E-58	Q9UKS7	IKZF2
1	IL10RB	0	-1.83	0	0	Q08334	IL10RB
1	IL11	1.742E-165	23.38	1.014E-160	3.93E-164	P20809	IL11
1	IL12A	1.9271E-05	1.77	1	8.5395E-05	P29459	IL12A
1	IL15	3.61E-40	-4.16	2.1001E-35	3.784E-39	P40933	IL15
1	IL15RA	5.2182E-83	-10.44	3.0356E-78	7.9363E-82	Q13261	IL15RA
1	IL16	1.3869E-25	-6.53	8.0679E-21	1.175E-24	Q14005	IL16
1	IL17D	7.5846E-11	-1.89	4.4123E-06	4.4269E-10	Q8TAD2	IL17D
1	IL17RB	7.7263E-13	1.60	4.4947E-08	4.8649E-12	Q9NRM6	IL17RB
1	IL17RC	0	-1.54	0	0	Q8NAC3	IL17RC
1	IL17RD	1.551E-53	-2.82	9.0227E-49	1.8801E-52	Q8NFM7	IL17RD
1	IL17RE	6.552E-183	-16.40	3.811E-178	1.557E-181	Q8NFR9	IL17RE
1	IL18BP	3.9382E-09	-1.89	0.0002291	2.1369E-08	O95998	IL18BP
1	IL18R1	4.0313E-16	-5.47	2.3452E-11	2.8286E-15	Q13478	IL18R1
1	IL1A	4.203E-306	-39.30	2.445E-301	1.183E-304	P01583	IL1A
1	IL1B	1.1569E-13	-1.76	6.7299E-09	7.4876E-13	P01584	IL1B
1	IL1R1	8.7022E-07	-2.34	0.05062421	4.1918E-06	P14778	IL1R1
1	IL1R2	0.010391	1.98	1	0.03639949	P27930	IL1R2
1	IL1RAP	0	-3.70	0	0	Q9NPH3	IL1RAP
1	IL1RAPL1	1.498E-103	6.12	8.713E-99	2.636E-102	Q9NZN1	IL1RAPL1
1	IL1RL2	2.563E-119	-10.50	1.491E-114	4.947E-118	Q9HB29	IL1RL2
1	IL1RN	7.495E-157	-44.71	4.36E-152	1.65E-155	P18510	IL1RN
1	IL20RA	1.197E-134	-11.31	6.964E-130	2.452E-133	Q9UHF4	IL20RA
1	IL22RA1	1.235E-163	-40.09	7.186E-159	2.771E-162	Q8N6P7	IL22RA1
1	IL23A	1.5284E-05	-1.80	0.8891248	6.8205E-05	Q9NPF7	IL23A
1	IL24	0.00644874	-7.62	1	0.02314164	Q13007	IL24
1	IL27RA	5.1034E-76	2.95	2.9689E-71	7.3287E-75	Q6UWB1	IL27RA
1	IL2RB	3.2258E-34	-56.49	1.8766E-29	3.1555E-33	P14784	IL2RB
1	IL2RG	0.00035619	-5.35	1	0.00144037	P31785	IL2RG
1	IL31RA	1.9677E-22	7.81	1.1447E-17	1.5711E-21	Q8NI17	IL31RA
1	IL32	2.0045E-18	-4.70	1.1661E-13	1.48E-17	P24001	IL32
1	IL33	1.0572E-29	-23.37	6.1502E-25	9.6807E-29	O95760	IL33
1	IL34	1.3793E-20	-9.28	8.0242E-16	1.0625E-19	Q6ZMJ4	IL34

1	IL36G	7.5263E-14	-5.54	4.3783E-09	4.903E-13	Q9NZH8	IL36G
1	IL36RN	3.0814E-05	-34.69	1	0.00013478	Q9UBH0	IL36RN
1	IL4R	0	-7.90	0	0	P24394	IL4R
1	IL6	4.2996E-27	6.15	2.5013E-22	3.7511E-26	P05231	IL6
1	IL6R	6.4518E-94	-22.32	3.7533E-89	1.0657E-92	P08887	IL6R
1	IL7R	1.563E-198	124.48	9.094E-194	3.922E-197	P16871	IL7R
1	IL9R	5.5902E-25	-8.45	3.2521E-20	4.688E-24	Q01113	IL9R
1	IL9RP3	2.3521E-23	-6.06	1.3683E-18	1.9129E-22		
1	ILDR1	1.1388E-55	-13.77	6.6247E-51	1.4065E-54	Q86SU0	ILDR1
1	ILF3	2.1863E-26	1.61	1.2719E-21	1.8806E-25	Q12906	ILF3
1	ILK	5.498E-154	2.73	3.198E-149	1.198E-152	Q13418	ILK
1	ILKAP	2.248E-35	1.58	1.3077E-30	2.2274E-34	Q9H0C8	ILKAP
1	IMMP2L	1.0741E-52	-2.88	6.2486E-48	1.2902E-51	Q96T52	IMMP2L
1	IMP4	6.8352E-86	2.61	3.9763E-81	1.0629E-84	Q96G21	IMP4
1	IMPA2	0	-2.17	0	0	O14732	IMPA2
1	IMPDH1P8	0.00516212	-1.52	1	0.01875008		
1	IMPDH2	8.3177E-58	2.01	4.8387E-53	1.0419E-56	P12268	IMPDH2
1	IMPG1	0.00277942	-1.66	1	0.01037138	Q17R60	IMPG1
1	INA	5.7297E-16	-8.56	3.3332E-11	3.9957E-15	Q16352	INA
1	INCA1	8.1129E-23	-2.02	4.7196E-18	6.5351E-22	Q0VD86	INCA1
1	ING1	6.9697E-12	1.52	4.0545E-07	4.242E-11	Q9UK53	ING1
1	INHA	6.9219E-08	6.88	0.00402672	3.5335E-07	P05111	INHA
1	INHBA	3.064E-211	30.09	1.782E-206	7.929E-210	P08476	INHBA
1	INHBB	0.00101477	23.83	1	0.00395427	P09529	INHBB
1	INHBC	3.6339E-05	-4.54	1	0.00015829	P55103	INHBC
1	INHBE	2.2087E-06	-2.75	0.12848714	1.0398E-05	P58166	INHBE
1	INO80E	1.887E-120	1.88	1.097E-115	3.657E-119	Q8NBZ0	INO80E
1	INPP1	0	-1.57	0	0	P49441	INPP1
1	INPP4B	0	-2.21	0	0	O15327	INPP4B
1	INPP5D	4.9474E-09	-6.33	0.00028781	2.6718E-08	Q92835	INPP5D
1	INPP5J	1.5824E-28	2.51	9.2056E-24	1.4195E-27	Q15735	INPP5J
1	INPP5K	1.263E-106	2.87	7.346E-102	2.262E-105	Q9BT40	INPP5K
1	INSC	0.00267308	-8.94	1	0.00998675	Q1MX18	INSC
1	INSIG1	0	-2.01	0	0	O15503	INSIG1
1	INSL3	0.00027031	-7.46	1	0.00110266	P51460	INSL3
1	INSR	1.027E-164	7.10	5.973E-160	2.309E-163	P06213	INSR
1	INTS1	0	-2.11	0	0	Q8N201	INTS1
1	INTS10	0	-1.65	0	0	Q9NVR2	INTS10
1	INTS13	3.8727E-41	1.73	2.2529E-36	4.1134E-40	Q9NVM9	INTS13
1	INTS2	7.7052E-39	1.56	4.4824E-34	7.9518E-38	Q9H0H0	INTS2
1	INTS4P2	5.8667E-11	162.65	3.4129E-06	3.438E-10		
1	INTS6-AS1	4.3069E-23	-2.74	2.5055E-18	3.4822E-22		
1	INTS6L	0	-1.95	0	0		
1	INTS7	0	-1.84	0	0	Q9NVH2	INTS7
1	INTS8	0	-1.59	0	0	Q75QN2	INTS8
1	INTS9	0	-1.71	0	0	Q9NV88	INTS9
1	INTU	6.9371E-41	-2.38	4.0356E-36	7.3348E-40	Q9ULD6	INTU

1	IP6K1	6.1573E-84	1.80	3.582E-79	9.4386E-83	Q92551	IP6K1
1	IP6K3	0.00020155	-2.28	1	0.00083221	Q96PC2	IP6K3
1	IPCEF1	2.5042E-25	-15.17	1.4568E-20	2.1146E-24	Q8WWN9	IPCEF1
1	IPO11	5.9196E-21	1.59	3.4437E-16	4.5946E-20	Q9UI26	IPO11
1	IPO4	1.1801E-28	2.65	6.8651E-24	1.0614E-27	Q8TEX9	IPO4
1	IPO5	4.213E-39	1.65	2.4509E-34	4.3656E-38	O00410	IPO5
1	IPO7	6.6664E-62	2.68	3.8781E-57	8.6238E-61	O95373	IPO7
1	IPO9	0.00012253	-1.89	1	0.00051368	Q96P70	IPO9
1	IQCA1	2.5746E-13	-5.97	1.4977E-08	1.6461E-12	Q86XH1	IQCA1
1	IQCB1	1.84E-54	1.65	1.0704E-49	2.2497E-53	Q15051	IQCB1
1	IQCB2P	2.9245E-05	-9.95	1	0.0001281		
1	IQCE	0	-1.53	0	0	Q6IPM2	IQCE
1	IQCH	2.2903E-87	-3.76	1.3324E-82	3.602E-86		
1	IQCH-AS1	6.8658E-14	-2.02	3.9941E-09	4.4772E-13		
1	IQCJ-SCHIP1	0	-1.52	0	0		
1	IQGAP1	0	-1.54	0	0	P46940	IQGAP1
1	IQGAP2	4.1555E-05	4.35	1	0.00018012	Q13576	IQGAP2
1	IQSEC2	3.5218E-98	-3.29	2.0488E-93	5.9871E-97	Q5JU85	IQSEC2
1	IQSEC3	5.2034E-12	-79.59	3.027E-07	3.1823E-11	Q9UPP2	IQSEC3
1	IRAK1	8.2955E-43	2.07	4.8258E-38	9.0151E-42	P51617	IRAK1
1	IRAK3	4.1072E-21	-5.81	2.3893E-16	3.1998E-20	Q9Y616	IRAK3
1	IRF1	0	-3.44	0	0	P10914	IRF1
1	IRF2	0	-3.12	0	0	P14316	IRF2
1	IRF2BP1	2.465E-11	1.52	1.434E-06	1.4696E-10	Q8IU81	IRF2BP1
1	IRF5	9.96E-184	-6.27	5.794E-179	2.373E-182	Q13568	IRF5
1	IRF6	0	-26.28	0	0	O14896	IRF6
1	IRF7	9.6081E-84	4.56	5.5894E-79	1.4686E-82	Q92985	IRF7
1	IRGQ	5.8286E-34	1.58	3.3907E-29	5.6872E-33	Q8WZA9	IRGQ
1	IRS1	1.8045E-59	4.18	1.0497E-54	2.292E-58	P35568	IRS1
1	IRS2	6.746E-173	77.16	3.925E-168	1.557E-171	Q9Y4H2	IRS2
1	IRS4	0.00015107	-29.08	1	0.00062886	O14654	IRS4
1	IRX2	5.07E-177	98.47	2.949E-172	1.185E-175	Q9BZI1	IRX2
1	IRX3	1.1602E-77	3.31	6.7494E-73	1.6848E-76	P78415	IRX3
1	IRX5	9.3592E-14	2.63	5.4446E-09	6.08E-13	P78411	IRX5
1	ISCA2	1.1107E-31	2.16	6.4615E-27	1.0466E-30	Q86U28	ISCA2
1	ISCU	0	-1.66	0	0	Q9H1K1	ISCU
1	ISG15	1.5797E-33	2.39	9.1896E-29	1.5303E-32	P05161	ISG15
1	ISL1	1.8313E-07	-12.17	0.01065366	9.1432E-07	P61371	ISL1
1	ISL2	4.5844E-87	-8.97	2.6669E-82	7.1943E-86	Q96A47	ISL2
1	ISM1	2.4062E-13	-2.41	1.3998E-08	1.5399E-12	B1AKI9	ISM1
1	IST1	0	-1.57	0	0	P53990	IST1
1	ISY1	1.6193E-19	1.66	9.4199E-15	1.2235E-18	Q9ULR0	ISY1
1	ISYNA1	1.755E-127	-3.84	1.021E-122	3.508E-126	Q9NPH2	ISYNA1
1	ITFG1	0	-1.76	0	0	Q8TB96	ITFG1
1	ITGA1	1.5666E-35	-2.26	9.1137E-31	1.5566E-34	P56199	ITGA1
1	ITGA10	2.634E-39	4.08	1.5323E-34	2.7333E-38	O75578	ITGA10
1	ITGA11	0.00076263	2.53	1	0.00300149	Q9UKX5	ITGA11

1	ITGA2B	0.0005243	-2.17	1	0.00209121	P08514	ITGA2B
1	ITGA3	0	-4.64	0	0	P26006	ITGA3
1	ITGA6	0	-4.80	0	0	P23229	ITGA6
1	ITGA7	8.292E-10	-1.92	4.8238E-05	4.6338E-09	Q13683	ITGA7
1	ITGAM	1.3399E-11	-11.59	7.7945E-07	8.0781E-11	P11215	ITGAM
1	ITGAV	0	-2.03	0	0	P06756	ITGAV
1	ITGB1BP1	2.2828E-37	1.51	1.328E-32	2.314E-36	O14713	ITGB1BP1
1	ITGB2-AS1	0.00440319	-2.99	1	0.01611319		
1	ITGB3	1.888E-123	30.57	1.099E-118	3.716E-122	P05106	ITGB3
1	ITGB4	0	-14.57	0	0	P16144	ITGB4
1	ITGB5	1.017E-110	2.00	5.915E-106	1.872E-109	P18084	ITGB5
1	ITGB6	0	-9.25	0	0	P18564	ITGB6
1	ITGB7	0.00013877	-3.29	1	0.00057945	P26010	ITGB7
1	ITGBL1	0	-11.74	0	0	O95965	ITGBL1
1	ITIH2	3.6473E-11	7.75	2.1218E-06	2.1565E-10	P19823	ITIH2
1	ITIH3	0.00942764	-19.74	1	0.03316063	Q06033	ITIH3
1	ITIH4	0.00355675	-2.36	1	0.01313717	Q14624	ITIH4
1	ITIH6	4.8333E-09	-3.23	0.00028117	2.6114E-08	Q6UXX5	ITIH6
1	ITM2A	1.7794E-16	-144.16	1.0352E-11	1.261E-15	O43736	ITM2A
1	ITM2B	0	-2.86	0	0	Q9Y287	ITM2B
1	ITPKA	1.0726E-31	3.25	6.2395E-27	1.0108E-30	P23677	ITPKA
1	ITPKC	0	-2.58	0	0	Q96DU7	ITPKC
1	ITPR1	7.8484E-27	2.21	4.5657E-22	6.7983E-26	Q14643	ITPR1
1	ITPR1-AS1	0.0002563	-2.28	1	0.001048		
1	ITPR3	0	-2.01	0	0	Q14573	ITPR3
1	ITPRIPL1	2.7881E-78	-6.61	1.622E-73	4.0702E-77	Q6GPH6	ITPRIPL1
1	ITPRIPL2	4.7566E-50	1.85	2.7671E-45	5.5743E-49	Q3MIP1	ITPRIPL2
1	ITSN2	0	-1.66	0	0	Q9NZM3	ITSN2
1	IVL	3.2456E-87	-31.25	1.8881E-82	5.1003E-86	P07476	IVL
1	IVNS1ABP	0	-1.52	0	0	Q9Y6Y0	IVNS1ABP
1	IZUMO1	8.8863E-08	-5.14	0.0051695	4.5101E-07	Q8IYV9	IZUMO1
1	JADE2	7.9816E-80	1.66	4.6432E-75	1.1824E-78	Q9NQC1	JADE2
1	JAG2	0	-13.53	0	0	Q9Y219	JAG2
1	JAK2	1.4131E-15	1.58	8.2205E-11	9.7112E-15	O60674	JAK2
1	JAK3	5.9907E-10	-15.44	3.485E-05	3.3704E-09	P52333	JAK3
1	JAKMIP2	2.4612E-08	-5.34	0.00143179	1.2878E-07	Q96AA8	JAKMIP2
1	JAKMIP3	1.2589E-14	-5.88	7.3236E-10	8.3957E-14	Q5VZ66	JAKMIP3
1	JAM2	0.00098022	-1.92	1	0.00382246	P57087	JAM2
1	JAM3	2.8142E-13	2.05	1.6371E-08	1.7959E-12	Q9BX67	JAM3
1	JARID2	0	-1.73	0	0	Q92833	JARID2
1	JAZF1	3.5095E-22	2.15	2.0416E-17	2.7872E-21	Q86VZ6	JAZF1
1	JDP2	4.8357E-64	-5.06	2.8131E-59	6.3617E-63	Q8WYK2	JDP2
1	JHDM1D-AS1	8.5686E-17	-2.85	4.9847E-12	6.1184E-16		
1	JMJD1C	4.917E-40	1.84	2.8604E-35	5.1493E-39	Q15652	JMJD1C
1	JMJD7	1.094E-29	-2.25	6.364E-25	1.0016E-28	P0C870	JMJD7
1	JMJD7-PLA2G	0	-3.73	0	0		
1	JPH1	3.101E-192	-10.41	1.804E-187	7.611E-191	Q9HDC5	JPH1

1	JPH2	0.00031492	-1.66	1	0.00127819	Q9BR39	JPH2
1	JPH3	2.7539E-17	2.05	1.602E-12	1.9867E-16	Q8WXH2	JPH3
1	JUN	1.765E-181	15.48	1.027E-176	4.18E-180	P05412	JUN
1	JUNB	0	-5.49	0	0	P17275	JUNB
1	JUP	0	-10.39	0	0	P14923	JUP
1	KANK2	1.1377E-44	3.46	6.6183E-40	1.2602E-43	Q63ZY3	KANK2
1	KANK3	0.00063841	1.83	1	0.00252936		
1	KANSL1-AS1	1.2456E-12	-6.43	7.2459E-08	7.783E-12		
1	KANSL1L	0	-1.81	0	0	A0AUZ9	KANSL1L
1	KANSL3	7.1831E-38	1.51	4.1787E-33	7.3246E-37	Q9P2N6	KANSL3
1	KAT2B	4.2692E-14	1.52	2.4836E-09	2.8025E-13	Q92831	KAT2B
1	KAT6A	0	-1.59	0	0	Q92794	KAT6A
1	KATNA1	2.4608E-22	1.54	1.4315E-17	1.9613E-21	O75449	KATNA1
1	KATNAL1	6.7095E-59	2.47	3.9032E-54	8.4889E-58	Q9BW62	KATNAL1
1	KATNAL2	1.008E-164	-5.40	5.864E-160	2.268E-163	Q8IYT4	KATNAL2
1	KAZALD1	1.3742E-50	2.50	7.9941E-46	1.6159E-49	Q96I82	KAZALD1
1	KAZN	3.4285E-19	-3.47	1.9945E-14	2.5722E-18	Q674X7	KAZN
1	KB-1042C11.5	6.56E-143	-5.00	3.816E-138	1.389E-141		
1	KB-1460A1.1	3.3184E-21	-5.94	1.9304E-16	2.5888E-20		
1	KB-1507C5.4	3.7706E-08	-12.88	0.0021935	1.9526E-07		
1	KB-1562D12.1	5.3655E-79	-18.46	3.1213E-74	7.8961E-78		
1	KB-1572G7.3	0.00024565	3.63	1	0.00100636		
1	KB-1615E4.2	5.0414E-37	-31.95	2.9328E-32	5.0908E-36		
1	KB-173C10.2	0.00019937	-9.29	1	0.00082357		
1	KB-1930G5.4	1.201E-28	-26.26	6.987E-24	1.0801E-27		
1	KB-1980E6.3	0.00804235	-15.97	1	0.02856436		
1	KB-1991G8.1	2.485E-103	-58.78	1.4456E-98	4.37E-102		
1	KB-68A7.1	0.01039621	-15.95	1	0.03641552		
1	KBTBD2	0	-1.74	0	0	Q8IY47	KBTBD2
1	KBTBD3	4.419E-15	-1.84	2.5707E-10	2.9899E-14	Q8NAB2	KBTBD3
1	KBTBD4	3.6055E-17	1.59	2.0974E-12	2.5936E-16	Q9NVX7	KBTBD4
1	KBTBD6	9.6855E-31	1.87	5.6344E-26	9.0065E-30	Q86V97	KBTBD6
1	KBTBD8	1.4639E-38	3.24	8.5162E-34	1.5062E-37	Q8NFY9	KBTBD8
1	KCCAT198	1.6708E-11	83.78	9.7198E-07	1.0036E-10		
1	KCNA7	5.3705E-14	-9.20	3.1243E-09	3.5151E-13	Q96RP8	KCNA7
1	KCNAB2	0	-3.42	0	0	Q13303	KCNAB2
1	KCNAB3	5.6012E-22	2.91	3.2584E-17	4.4338E-21	O43448	KCNAB3
1	KCNC1	0.00472524	2.53	1	0.01723856	P48547	KCNC1
1	KCND1	7.5335E-21	-6.64	4.3825E-16	5.8387E-20	Q9NSA2	KCND1
1	KCND2	3.1505E-09	-5.02	0.00018328	1.718E-08	Q9NZV8	KCND2
1	KCND3	6.5284E-05	4.84	1	0.00027925	Q9UK17	KCND3
1	KCNE1	0.00044211	3.70	1	0.00177375	P15382	KCNE1
1	KCNE3	0.0002331	-6.10	1	0.00095724	Q9Y6H6	KCNE3
1	KCNG3	0.00036882	-7.46	1	0.00148936	Q8TAE7	KCNG3
1	KCNH1	0.00603836	2.19	1	0.02175352	O95259	KCNH1
1	KCNH3	0.00909096	-1.72	1	0.03208114	Q9ULD8	KCNH3
1	KCNH4	0.00656462	-7.62	1	0.02353569	Q9UQ05	KCNH4

1	KCNH7	2.934E-09	-7.16	0.00017068	1.6019E-08	Q9NS40	KCNH7
1	KCNH8	1.1553E-25	-13.82	6.7207E-21	9.8042E-25	Q96L42	KCNH8
1	KCNIP1	0.00423644	6.34	1	0.01553817	Q9NZI2	KCNIP1
1	KCNIP3	1.789E-18	11.38	1.0408E-13	1.3226E-17	Q9Y2W7	KCNIP3
1	KCNJ1	0.00785339	-1.82	1	0.02793899	P48048	KCNJ1
1	KCNJ10	3.1522E-14	-1.97	1.8338E-09	2.0777E-13	P78508	KCNJ10
1	KCNJ11	1.7408E-23	8.26	1.0127E-18	1.4183E-22	Q14654	KCNJ11
1	KCNJ15	3.8815E-43	-4.31	2.258E-38	4.2341E-42	Q99712	KCNJ15
1	KCNJ16	2.1996E-26	179.58	1.2796E-21	1.8917E-25	Q9NPI9	KCNJ16
1	KCNJ2	0	793.00	0	0	P63252	KCNJ2
1	KCNJ2-AS1	1.6682E-82	147.66	9.7047E-78	2.5292E-81		
1	KCNJ3	0.01307938	-7.01	1	0.04525814	P48549	KCNJ3
1	KCNJ5	1.5243E-31	-8.33	8.8676E-27	1.433E-30	P48544	KCNJ5
1	KCNJ6	0.00587734	-8.25	1	0.021201	P48051	KCNJ6
1	KCNJ8	3.2598E-06	2.01	0.18963283	1.5161E-05	Q15842	KCNJ8
1	KCNK1	0	-6.74	0	0	O00180	KCNK1
1	KCNK5	6.876E-305	-13.87	4E-300	1.934E-303	O95279	KCNK5
1	KCNK6	7.5622E-29	-1.86	4.3993E-24	6.8269E-28	Q9Y257	KCNK6
1	KCNK7	0.00046552	-2.28	1	0.00186484	Q9Y2U2	KCNK7
1	KCNMA1	3.4182E-67	9.10	1.9885E-62	4.5945E-66	Q12791	KCNMA1
1	KCNMB2	4.8539E-05	-5.61	1	0.00020935	Q9Y691	KCNMB2
1	KCNMB2-AS1	3.5481E-43	-6.52	2.0641E-38	3.8747E-42		
1	KCNMB3	7.296E-154	-6.33	4.244E-149	1.588E-152	Q9NPA1	KCNMB3
1	KCNMB4	9.772E-105	-8.92	5.685E-100	1.736E-103	Q86W47	KCNMB4
1	KCNN4	3.179E-157	-5.39	1.85E-152	7E-156	O15554	KCNN4
1	KCNQ2	3.8031E-07	-12.12	0.02212414	1.8673E-06	O43526	KCNQ2
1	KCNQ5	8.813E-158	-9.94	5.127E-153	1.945E-156	Q9NR82	KCNQ5
1	KCNQ5-IT1	6.7428E-05	-12.47	1	0.00028806		
1	KCNS3	1.487E-166	-10.48	8.65E-162	3.366E-165	Q9BQ31	KCNS3
1	KCNT1	1.2707E-07	3.65	0.00739205	6.4012E-07	Q5JUK3	KCNT1
1	KCNT2	5.383E-143	-16.70	3.131E-138	1.14E-141	Q6UVM3	KCNT2
1	KCNU1	0.01403388	5.90	1	0.04844283	A8MYU2	KCNU1
1	KCNV1	5.574E-156	-15.37	3.242E-151	1.223E-154	Q6PIU1	KCNV1
1	KCP	6.448E-149	-10.96	3.751E-144	1.385E-147	Q6ZWJ8	KCP
1	KCTD14	2.243E-115	5.87	1.305E-110	4.235E-114	Q9BQ13	KCTD14
1	KCTD15	1.117E-104	4.44	6.5E-100	1.984E-103	Q96SI1	KCTD15
1	KCTD17	2.5868E-84	3.29	1.5048E-79	3.9789E-83	Q8N5Z5	KCTD17
1	KCTD2	8.1756E-32	1.57	4.756E-27	7.7209E-31	Q14681	KCTD2
1	KCTD3	0	-1.90	0	0	Q9Y597	KCTD3
1	KCTD6	8.2094E-17	1.77	4.7757E-12	5.8655E-16	Q8NC69	KCTD6
1	KCTD9	0	-1.63	0	0	Q7L273	KCTD9
1	KDELC1	4.7568E-70	2.75	2.7672E-65	6.525E-69	Q6UW63	KDELC1
1	KDELR2	0	-1.62	0	0	P33947	KDELR2
1	KDELR3	4.9169E-70	2.24	2.8603E-65	6.7429E-69	O43731	KDELR3
1	KDF1	9.279E-188	-10.09	5.398E-183	2.237E-186	Q8NAX2	KDF1
1	KDM1A	1.089E-119	1.70	6.336E-115	2.104E-118	O60341	KDM1A
1	KDM2A	0	-3.24	0	0	Q9Y2K7	KDM2A

1	KDM4A-AS1	0.01200457	-1.51	1	0.04173012		
1	KDM4C	0	-1.75	0	0	Q9H3R0	KDM4C
1	KDM5B	0	-1.64	0	0	Q9UGL1	KDM5B
1	KDM5C	1.2188E-63	1.51	7.09E-59	1.5979E-62	P41229	KDM5C
1	KDM6A	0	-5.02	0	0	O15550	KDM6A
1	KDM7A	6.9771E-16	-1.73	4.0588E-11	4.8464E-15	Q6ZMT4	KDM7A
1	KDR	4.8381E-07	-17.36	0.02814541	2.3632E-06	P35968	KDR
1	KHDC1	9.0047E-22	2.12	5.2384E-17	7.1029E-21	Q4VXA5	KHDC1
1	KHDC1L	0.00013929	-2.01	1	0.00058149		
1	KHDRBS3	2.0498E-84	12.38	1.1924E-79	3.1571E-83	O75525	KHDRBS3
1	KHK	3.229E-172	10.94	1.879E-167	7.431E-171	P50053	KHK
1	KHNYN	1.3126E-51	1.55	7.636E-47	1.5574E-50	O15037	KHNYN
1	KHSRPP1	0.0144535	-4.02	1	0.04980855		
1	KIAA0040	7E-102	-15.66	4.0722E-97	1.22E-100	Q15053	KIAA0040
1	KIAA0319L	0	-1.67	0	0	Q8IZA0	KIAA0319L
1	KIAA0355	8.5217E-30	1.64	4.9574E-25	7.8156E-29		
1	KIAA0391	1.0385E-27	1.79	6.0416E-23	9.1888E-27	O15091	KIAA0391
1	KIAA0408	1.7532E-34	4.09	1.0199E-29	1.7217E-33	Q6ZU52	KIAA0408
1	KIAA0513	2.8529E-33	-2.20	1.6596E-28	2.7514E-32	O60268	KIAA0513
1	KIAA0753	8.2641E-39	1.79	4.8075E-34	8.5225E-38	Q2KHM9	KIAA0753
1	KIAA0825	3.6323E-07	-1.58	0.02113054	1.7854E-06		
1	KIAA0907	0	-1.55	0	0	Q7Z7F0	KIAA0907
1	KIAA1024	2.512E-11	2.01	1.4613E-06	1.4973E-10	Q9UPX6	KIAA1024
1	KIAA1107	7.6262E-09	-3.53	0.00044365	4.0837E-08		
1	KIAA1109	0	-1.77	0	0	Q2LD37	KIAA1109
1	KIAA1143	9.4448E-28	1.51	5.4944E-23	8.3629E-27	Q96AT1	KIAA1143
1	KIAA1191	3.8031E-80	1.73	2.2124E-75	5.6525E-79	Q96A73	KIAA1191
1	KIAA1211	6.4324E-41	-2.71	3.742E-36	6.8036E-40		
1	KIAA1211L	1.105E-101	-91.71	6.4263E-97	1.922E-100		
1	KIAA1217	3.361E-221	-7.91	1.955E-216	8.903E-220	Q5T5P2	KIAA1217
1	KIAA1257	2.0075E-12	-16.65	1.1678E-07	1.247E-11		
1	KIAA1324	1.4372E-17	-4.28	8.3609E-13	1.0437E-16	Q6UXG2	KIAA1324
1	KIAA1324L	0	-2.88	0	0	A8MWY0	KIAA1324L
1	KIAA1456	0.00347495	9.03	1	0.01285787	Q9P272	KIAA1456
1	KIAA1462	1.1711E-93	-6.03	6.8128E-89	1.93E-92	Q9P266	KIAA1462
1	KIAA1468	8.3623E-54	1.65	4.8647E-49	1.0167E-52		
1	KIAA1522	0	-5.92	0	0	Q9P206	KIAA1522
1	KIAA1549	1.1512E-42	2.23	6.6971E-38	1.2483E-41	Q9HCM3	KIAA1549
1	KIAA1549L	4.5091E-88	3.96	2.6231E-83	7.1377E-87	Q6ZVL6	KIAA1549L
1	KIAA1614	6.6312E-37	-2.87	3.8576E-32	6.6857E-36		
1	KIAA1614-AS1	0.00014836	-2.69	1	0.00061793		
1	KIAA1671	0	-2.68	0	0		
1	KIAA1683	6.3236E-24	-7.37	3.6787E-19	5.1959E-23	Q9H0B3	KIAA1683
1	KIAA1755	7.1475E-07	-1.66	0.0415798	3.4604E-06		
1	KIAA2012	1.6052E-05	2.48	0.93379762	7.1528E-05		
1	KIAA2022	0.00475028	-9.43	1	0.0173234	Q5QGS0	KIAA2022
1	KIF14	0	-2.12	0	0	Q15058	KIF14

1	KIF17	2.026E-130	9.56	1.179E-125	4.09E-129	Q9P2E2	KIF17
1	KIF1BP	1.6952E-53	2.69	9.8619E-49	2.0537E-52	Q96EK5	KIF1BP
1	KIF21A	0	-2.01	0	0	Q7Z4S6	KIF21A
1	KIF21B	1.2767E-07	-2.59	0.00742726	6.4311E-07	O75037	KIF21B
1	KIF24	3.5807E-46	1.97	2.083E-41	4.0368E-45	Q5T7B8	KIF24
1	KIF26B	1.3186E-32	-3.80	7.6707E-28	1.2608E-31	Q2KJY2	KIF26B
1	KIF2A	7.3661E-59	1.73	4.2852E-54	9.3176E-58	O00139	KIF2A
1	KIF3B	9.0884E-73	1.75	5.2871E-68	1.2718E-71	O15066	KIF3B
1	KIF3C	5.441E-121	2.72	3.165E-116	1.058E-119	O14782	KIF3C
1	KIF5C	1.034E-165	10.26	6.017E-161	2.334E-164	O60282	KIF5C
1	KIF7	2.672E-109	3.34	1.554E-104	4.869E-108	Q2M1P5	KIF7
1	KIF9	3.7632E-19	1.80	2.1892E-14	2.8212E-18	Q9HAQ2	KIF9
1	KIRREL	0	-2.04	0	0	Q96J84	KIRREL
1	KISS1	0.00986876	-16.01	1	0.03463264	Q15726	KISS1
1	KIT	1.0341E-25	-6.12	6.0159E-21	8.7875E-25	P10721	KIT
1	KITLG	1.257E-43	5.08	7.3128E-39	1.3826E-42	P21583	KITLG
1	KL	0.00468576	-2.82	1	0.0171031	Q9UEF7	KL
1	KLC3	8.061E-235	-8.63	4.689E-230	2.17E-233	Q6P597	KLC3
1	KLF10	0	-2.14	0	0	Q13118	KLF10
1	KLF13	6.811E-38	1.62	3.9622E-33	6.9476E-37	Q9Y2Y9	KLF13
1	KLF14	1.1709E-05	-3.35	0.68117983	5.2641E-05	Q8TD94	KLF14
1	KLF15	2.705E-08	-4.73	0.00157358	1.4126E-07	Q9UIH9	KLF15
1	KLF2	1.4321E-17	5.27	8.3311E-13	1.0401E-16	Q9Y5W3	KLF2
1	KLF3	0	-2.81	0	0	P57682	KLF3
1	KLF3-AS1	1.0237E-13	-2.83	5.9555E-09	6.6393E-13		
1	KLF4	4.092E-129	-3.41	2.38E-124	8.231E-128	O43474	KLF4
1	KLF5	0	-4.32	0	0	Q13887	KLF5
1	KLF6	7.232E-19	2.41	4.2071E-14	5.3903E-18	Q99612	KLF6
1	KLF7	0	-3.64	0	0	O75840	KLF7
1	KLF8	5.4926E-27	-7.48	3.1952E-22	4.7769E-26	O95600	KLF8
1	KLF9	1.4665E-10	-1.70	8.5313E-06	8.472E-10	Q13886	KLF9
1	KLHDC1	2.1575E-10	-2.82	1.2551E-05	1.2367E-09	Q8N7A1	KLHDC1
1	KLHDC7B	4.5873E-53	-42.53	2.6686E-48	5.5331E-52	Q96G42	KLHDC7B
1	KLHDC8B	4.6389E-30	2.30	2.6986E-25	4.2673E-29	Q8IXV7	KLHDC8B
1	KLHDC9	4.3687E-51	-9.40	2.5415E-46	5.1593E-50	Q8NEP7	KLHDC9
1	KLHL12	0	-2.49	0	0	Q53G59	KLHL12
1	KLHL14	0.00085543	-3.14	1	0.00335085	Q9P2G3	KLHL14
1	KLHL15	2.6432E-14	-1.62	1.5377E-09	1.7473E-13	Q96M94	KLHL15
1	KLHL17	1.3976E-82	2.43	8.1304E-78	2.12E-81	Q6TDP4	KLHL17
1	KLHL18	7.185E-104	2.13	4.18E-99	1.269E-102	O94889	KLHL18
1	KLHL2	0	-2.03	0	0	O95198	KLHL2
1	KLHL22	2.6666E-31	1.82	1.5513E-26	2.5004E-30	Q53GT1	KLHL22
1	KLHL23	5.2785E-30	1.66	3.0707E-25	4.851E-29	Q8NBE8	KLHL23
1	KLHL26	2.242E-77	2.54	1.3043E-72	3.2501E-76	Q53HC5	KLHL26
1	KLHL28	2.2748E-17	1.89	1.3234E-12	1.6443E-16	Q9NXS3	KLHL28
1	KLHL2P1	0.00129494	-5.00	1	0.00499646		
1	KLHL3	2.56E-105	-12.58	1.489E-100	4.553E-104	Q9UH77	KLHL3

1	KLHL30	5.7419E-07	-2.44	0.03340309	2.7936E-06	Q0D2K2	KLHL30
1	KLHL31	3.6592E-76	-8.49	2.1287E-71	5.2626E-75	Q9H511	KLHL31
1	KLHL32	1.8217E-11	-5.37	1.0598E-06	1.0923E-10	Q96NJ5	KLHL32
1	KLHL36	0	-1.85	0	0	Q8N4N3	KLHL36
1	KLHL4	9.7307E-13	-5.44	5.6607E-08	6.1065E-12	Q9C0H6	KLHL4
1	KLHL42	4.5242E-77	2.56	2.6319E-72	6.5437E-76	Q9P2K6	KLHL42
1	KLHL7-AS1	2.4333E-14	-2.18	1.4155E-09	1.6097E-13		
1	KLK1	0.00059758	-25.30	1	0.00237279	P06870	KLK1
1	KLK10	0.00010169	-11.86	1	0.00042914	O43240	KLK10
1	KLK14	9.7418E-14	-6.23	5.6672E-09	6.3243E-13	Q9P0G3	KLK14
1	KLRA1P	1.8541E-07	1.90	0.010786	9.252E-07		
1	KLRC4-KLRK1	1.5315E-12	-20.13	8.9095E-08	9.5441E-12	H3BQV0	KLRC4-KLRK1
1	KLRD1	2.9717E-05	-9.26	1	0.00013014	Q13241	KLRD1
1	KLRF1	1.1428E-11	-9.64	6.6482E-07	6.9058E-11	Q9NZS2	KLRF1
1	KLRG1	1.2659E-19	3.14	7.3645E-15	9.5805E-19	Q96E93	KLRG1
1	KLRK1	5.382E-13	-17.25	3.1309E-08	3.4076E-12	P26718	KLRK1
1	KMT2C	7.5321E-12	1.54	4.3818E-07	4.5782E-11	Q8NEZ4	KMT2C
1	KMT2E	0	-1.81	0	0	Q8IZD2	KMT2E
1	KMT2E-AS1	4.4637E-11	-2.22	2.5967E-06	2.6288E-10		
1	KMT5B	0	-1.88	0	0	Q4FZB7	KMT5B
1	KNDC1	3.8389E-15	18.36	2.2332E-10	2.6047E-14	Q76NI1	KNDC1
1	KNOP1	1.172E-32	1.50	6.8178E-28	1.1215E-31	Q1ED39	KNOP1
1	KPNA1	9.0846E-81	1.59	5.2849E-76	1.3561E-79	P52294	KPNA1
1	KPNA2P3	0.00022985	-1.93	1	0.00094438		
1	KPNA3	5.1196E-50	1.86	2.9783E-45	5.9937E-49	O00505	KPNA3
1	KPNA7	3.6051E-26	-4.44	2.0973E-21	3.0896E-25	A9QM74	KPNA7
1	KRBA1	2.6746E-38	1.85	1.5559E-33	2.7432E-37	A5PL33	KRBA1
1	KRBOX1	0.00025632	-34.65	1	0.001048	C9JBD0	KRBOX1
1	KRBOX4	3.3307E-15	-1.61	1.9376E-10	2.2662E-14	Q5JUW0	KRBOX4
1	KRCC1	0	-4.09	0	0	Q9NPI7	KRCC1
1	KREMEN1	0	-8.31	0	0	Q96MU8	KREMEN1
1	KREMEN2	3.669E-289	-8.95	2.134E-284	1.028E-287	Q8NCW0	KREMEN2
1	KRI1	8.761E-106	2.29	5.097E-101	1.563E-104	Q8N9T8	KRI1
1	KRIT1	0	-1.51	0	0	O00522	KRIT1
1	KRT13	0	-6.97	0	0	P13646	KRT13
1	KRT14	1.678E-175	-6.80	9.761E-171	3.908E-174	P02533	KRT14
1	KRT15	0	-7.10	0	0	P19012	KRT15
1	KRT16	3.181E-103	-9.37	1.8503E-98	5.59E-102	P08779	KRT16
1	KRT16P6	6.7363E-11	-7.68	3.9188E-06	3.9404E-10		
1	KRT17	0	-9.91	0	0	Q04695	KRT17
1	KRT17P4	6.4093E-06	-40.11	0.37285324	2.9319E-05		
1	KRT17P7	0.00573247	-20.01	1	0.02070536		
1	KRT18	1.292E-122	2.86	7.513E-118	2.531E-121	P05783	KRT18
1	KRT18P34	5.1007E-05	-4.10	1	0.00021978		
1	KRT18P8	0.00097251	-25.11	1	0.00379316		
1	KRT19	0	-10.48	0	0	P08727	KRT19
1	KRT222	0.00013638	-3.15	1	0.00056958	Q8N1A0	KRT222

1	KRT223P	0.00064468	25.86	1	0.002553		
1	KRT3	1.7783E-17	-207.78	1.0345E-12	1.289E-16	P12035	KRT3
1	KRT31	5.3637E-24	-7.98	3.1203E-19	4.4128E-23	Q15323	KRT31
1	KRT32	3.3127E-06	-10.73	0.19271085	1.5393E-05	Q14532	KRT32
1	KRT4	8.7342E-72	-107.40	5.081E-67	1.2135E-70	P19013	KRT4
1	KRT42P	9.7615E-19	-19.14	5.6787E-14	7.2571E-18		
1	KRT5	0	-14.88	0	0	P13647	KRT5
1	KRT6A	0	-5.64	0	0	P02538	KRT6A
1	KRT6B	4.4034E-17	-5.87	2.5616E-12	3.161E-16	P04259	KRT6B
1	KRT6C	1.5673E-08	-3.84	0.00091174	8.2833E-08	P48668	KRT6C
1	KRT7-AS	0	-2.12	0	0		
1	KRT71	0.00056268	-27.32	1	0.00223973	Q3SY84	KRT71
1	KRT74	2.2463E-08	-6.94	0.00130678	1.1778E-07	Q7RTS7	KRT74
1	KRT75	0.00573247	-20.19	1	0.02070536	O95678	KRT75
1	KRT77	0.00140517	-27.26	1	0.00539816	Q7Z794	KRT77
1	KRT78	6.1017E-17	-129.79	3.5496E-12	4.3693E-16	Q8N1N4	KRT78
1	KRT8	9.732E-39	1.77	5.6615E-34	1.0026E-37	P05787	KRT8
1	KRT81	4.2298E-07	-12.21	0.02460627	2.0723E-06	Q14533	KRT81
1	KRT86	0.00225952	-2.48	1	0.00849844	O43790	KRT86
1	KRT87P	0.00197428	-3.16	1	0.00747344	A6NCN2	KRT87P
1	KRT89P	0.00747132	-6.35	1	0.02664684		
1	KRT8P12	1.4614E-62	-2.47	8.5014E-58	1.9002E-61		
1	KRT8P14	0.00360227	11.04	1	0.0132935		
1	KRT9	0.00108738	-10.66	1	0.0042222	P35527	KRT9
1	KRTAP2-3	2.128E-225	134.13	1.238E-220	5.673E-224	P0C7H8	KRTAP2-3
1	KRTAP3-1	2.2307E-11	79.95	1.2977E-06	1.3313E-10	Q9BYR8	KRTAP3-1
1	KRTAP4-1	1.0628E-05	-10.35	0.61829148	4.7926E-05	Q9BYQ7	KRTAP4-1
1	KRTAP4-7	0.00029261	28.00	1	0.0011903	Q9BYR0	KRTAP4-7
1	KRTAP4-8	0.00450842	21.71	1	0.0164827	A0A0G2JLE6,	KRTAP4-8
1	KRTAP4-9	3.9266E-05	14.37	1	0.00017054	Q9BYQ8	KRTAP4-9
1	KRTAP5-7	0.00221501	-25.08	1	0.00834127	Q6L8G8	KRTAP5-7
1	KRTAP5-9	2.7178E-09	-10.33	0.00015811	1.4865E-08	P26371	KRTAP5-9
1	KRTCAP3	1.553E-218	-8.47	9.036E-214	4.094E-217	Q53RY4	KRTCAP3
1	KRTDAP	0.00130105	2.50	1	0.00501872	P60985	KRTDAP
1	KSR1	4.9924E-15	2.01	2.9043E-10	3.3696E-14	Q8IVT5	KSR1
1	KTN1	1.0679E-32	1.55	6.2123E-28	1.0226E-31	Q86UP2	KTN1
1	KTN1-AS1	3.9173E-23	1.95	2.2789E-18	3.1717E-22		
1	KXD1	3.02E-109	2.14	1.757E-104	5.503E-108	Q9BQD3	KXD1
1	KYAT3	3.2509E-32	1.76	1.8912E-27	3.0896E-31	Q6YP21	KYAT3
1	KYNU	6.6931E-33	-6.62	3.8936E-28	6.4305E-32	Q16719	KYNU
1	L1CAM	3.337E-208	27.52	1.942E-203	8.579E-207	P32004	L1CAM
1	L34079.2	0.00140517	27.77	1	0.00539816		
1	L34079.4	3.5549E-06	26.19	0.20680059	1.6487E-05		
1	L3MBTL2	2.8722E-52	1.55	1.6709E-47	3.4359E-51	Q969R5	L3MBTL2
1	LA16c-313D11	0.00022537	4.90	1	0.00092661		
1	LA16c-325D7.	3.4168E-16	-5.69	1.9877E-11	2.4009E-15		
1	LA16c-325D7.	7.0392E-05	-2.07	1	0.00030035		

1	LA16c-358B7.	3.6917E-07	1.64	0.02147591	1.8137E-06		
1	LA16c-361A3.	0.00840841	-2.57	1	0.02979903		
1	LA16c-380A1.	0.00543189	-19.62	1	0.01968081		
1	LA16c-380A1.	0.00829455	-15.96	1	0.02941879		
1	LA16c-380H5.	6.3725E-06	-4.37	0.3707119	2.916E-05		
1	LA16c-444G7.	4.871E-179	38.93	2.834E-174	1.146E-177		
1	LA16c-60G3.6	4.8815E-07	9.26	0.02839761	2.3841E-06		
1	LACC1	4.638E-143	-7.18	2.698E-138	9.829E-142	Q8IV20	LACC1
1	LAD1	0	-8.10	0	0	O00515	LAD1
1	LAG3	7.3317E-11	-4.37	4.2651E-06	4.2836E-10	P18627	LAG3
1	LAGE3	8.5134E-35	2.63	4.9526E-30	8.3857E-34	Q14657	LAGE3
1	LAMA1	8.948E-159	36.50	5.205E-154	1.983E-157	P25391	LAMA1
1	LAMA2	1.4833E-86	-2.89	8.629E-82	2.3165E-85	P24043	LAMA2
1	LAMA3	0	-7.45	0	0	Q16787	LAMA3
1	LAMA4	6.087E-126	-7.48	3.541E-121	1.209E-124	Q16363	LAMA4
1	LAMA5	0	-2.79	0	0	O15230	LAMA5
1	LAMA5-AS1	0.00122795	-6.08	1	0.00474745		
1	LAMB1	1.4236E-84	1.95	8.2819E-80	2.1956E-83	P07942	LAMB1
1	LAMB3	0	-3.30	0	0	Q13751	LAMB3
1	LAMB4	0.01298087	-3.53	1	0.04493597	A4D0S4	LAMB4
1	LAMC1	0	-4.26	0	0	P11047	LAMC1
1	LAMC2	0	-8.64	0	0	Q13753	LAMC2
1	LAMP3	4.7862E-61	-19.93	2.7843E-56	6.1437E-60	Q9UQV4	LAMP3
1	LAMTOR4	0	-1.65	0	0	Q0VGL1	LAMTOR4
1	LANCL2	1.5144E-42	1.91	8.8099E-38	1.6403E-41	Q9NS86	LANCL2
1	LANCL3	1.4145E-37	-7.47	8.2288E-33	1.4366E-36	Q6ZV70	LANCL3
1	LAPTM5	6.6586E-19	-2.53	3.8736E-14	4.9699E-18	Q13571	LAPTM5
1	LARGE1	1.056E-160	12.12	6.143E-156	2.352E-159	O95461	LARGE1
1	LARGE2	2.3364E-23	-8.01	1.3592E-18	1.9004E-22	Q8N3Y3	LARGE2
1	LARP6	1.4357E-67	2.88	8.352E-63	1.9347E-66	Q9BRS8	LARP6
1	LARS	3.0826E-84	1.63	1.7933E-79	4.7365E-83	Q9P2J5	LARS
1	LAS1L	2.235E-99	1.80	1.3E-94	3.8304E-98	Q9Y4W2	LAS1L
1	LAT	1.1269E-05	1.77	0.65555639	5.0716E-05	O43561	LAT
1	LAT2	8.0369E-13	1.94	4.6754E-08	5.0561E-12	Q9GZY6	LAT2
1	LAX1	0.00049613	-11.25	1	0.00198239	Q8IWV1	LAX1
1	LAYN	2.5661E-32	16.47	1.4928E-27	2.4416E-31	Q6UX15	LAYN
1	LBHD1	0	-2.38	0	0		
1	LBR	0	-1.92	0	0	Q14739	LBR
1	LBX1	1.6161E-83	127.28	9.4015E-79	2.465E-82	P52954	LBX1
1	LBX1-AS1	7.662E-148	74.39	4.457E-143	1.64E-146		
1	LBX2	3.9741E-41	2.03	2.3119E-36	4.2188E-40	Q6XYB7	LBX2
1	LCN2	3.861E-303	-18.61	2.246E-298	1.085E-301	P80188	LCN2
1	LCORL	0	-1.69	0	0	Q8N3X6	LCORL
1	LCP1	0	-9.67	0	0	P13796	LCP1
1	LDB2	7.013E-102	-15.35	4.0796E-97	1.221E-100	O43679	LDB2
1	LDB3	1.8841E-15	-3.04	1.0961E-10	1.2916E-14	O75112	LDB3
1	LDHA	1.9959E-34	1.74	1.1611E-29	1.957E-33	P00338	LDHA

1	LDHAP4	0.00092729	-1.51	1	0.00362189		
1	LDHB	4.671E-111	3.44	2.717E-106	8.62E-110	P07195	LDHB
1	LDHD	3.2237E-12	-3.60	1.8753E-07	1.9876E-11	Q86WU2	LDHD
1	LDLR	0	-8.60	0	0	P01130	LDLR
1	LDLRAD1	2.7531E-15	-5.63	1.6016E-10	1.8778E-14	Q5T700	LDLRAD1
1	LDLRAD2	6.8946E-07	-3.08	0.04010869	3.3402E-06	Q5SZI1	LDLRAD2
1	LDOC1	0	142.19	0	0	O95751	LDOC1
1	LDOC1L	1.2285E-97	7.70	7.1465E-93	2.0793E-96		
1	LEF1	1.8645E-12	-1.62	1.0847E-07	1.1592E-11	Q9UJU2	LEF1
1	LEF1-AS1	2.441E-07	-6.07	0.01420022	1.2109E-06		
1	LEFTY1	0.00276892	-2.57	1	0.01033486	O75610	LEFTY1
1	LEKR1	2.3328E-48	-4.71	1.3571E-43	2.6905E-47		
1	LEMD1	2.507E-131	-16.96	1.458E-126	5.07E-130	Q68G75	LEMD1
1	LEMD2	0	-1.99	0	0	Q8NC56	LEMD2
1	LEMD3	0	-1.73	0	0	Q9Y2U8	LEMD3
1	LENG1	1.1718E-12	1.64	6.817E-08	7.3301E-12	Q96BZ8	LENG1
1	LENG8-AS1	1.3376E-36	-2.50	7.7814E-32	1.3458E-35		
1	LENG9	3.3901E-86	-5.87	1.9722E-81	5.2831E-85	Q96B70	LENG9
1	LEPR	3.1341E-74	-2.58	1.8232E-69	4.435E-73	P48357	LEPR
1	LEPROT	0	-1.56	0	0	O15243	LEPROT
1	LETM2	1.3647E-12	1.75	7.939E-08	8.5155E-12	Q2VYF4	LETM2
1	LFNG	0	-3.65	0	0	Q8NES3	LFNG
1	LGALS1	8.8798E-65	3.05	5.1658E-60	1.1746E-63	P09382	LGALS1
1	LGALS2	1.0519E-14	-4.55	6.1195E-10	7.0364E-14	P05162	LGALS2
1	LGALS3	0	-1.58	0	0	P17931	LGALS3
1	LGALS3BP	3.518E-112	3.08	2.047E-107	6.537E-111	Q08380	LGALS3BP
1	LGALS4	5.5433E-10	-9.60	3.2248E-05	3.1217E-09	P56470	LGALS4
1	LGALS8	0	-1.65	0	0	O00214	LGALS8
1	LGALS9	4.561E-07	-3.75	0.02653298	2.2306E-06	O00182	LGALS9
1	LGALS9C	0.00794968	-17.84	1	0.02825937	Q6DKI2	LGALS9C
1	LGI4	2.965E-12	-5.06	1.7249E-07	1.8311E-11	Q8N135	LGI4
1	LGMN	1.6109E-36	1.61	9.3711E-32	1.6196E-35	Q99538	LGMN
1	LGR4	0	-1.60	0	0	Q9BXB1	LGR4
1	LGR6	2.7775E-29	-4.83	1.6158E-24	2.5227E-28	Q9HBX8	LGR6
1	LGSN	1.096E-106	-16.65	6.376E-102	1.965E-105	Q5TDP6	LGSN
1	LHB	0.00084042	-2.23	1	0.00329429	P01229	LHB
1	LHFPL2	1.5205E-70	1.94	8.8451E-66	2.0935E-69	Q6ZUX7	LHFPL2
1	LHFPL3-AS2	9.8959E-06	-2.64	0.57568315	4.4727E-05		
1	LHFPL4	5.7228E-06	5.87	0.3329183	2.6249E-05	Q7Z7J7	LHFPL4
1	LHFPL5	0.00016549	-3.99	1	0.00068723	Q8TAF8	LHFPL5
1	LHPP	4.6137E-68	-2.73	2.684E-63	6.2346E-67	Q9H008	LHPP
1	LHX1	7.238E-36	2.27	4.2106E-31	7.2112E-35	P48742	LHX1
1	LHX4	8.128E-10	-2.25	4.7284E-05	4.5443E-09	Q969G2	LHX4
1	LHX5	4.9088E-15	-8.71	2.8556E-10	3.3136E-14	Q9H2C1	LHX5
1	LHX6	0.00372038	-1.69	1	0.01371023	Q9UPM6	LHX6
1	LHX8	3.9166E-12	-12.96	2.2784E-07	2.4055E-11	Q68G74	LHX8
1	LHX9	6.4814E-14	-5.14	3.7705E-09	4.2299E-13	Q9NQ69	LHX9

1	LIF	4.9651E-74	4.36	2.8884E-69	7.0141E-73	P15018	LIF
1	LIFR-AS1	2.8105E-06	-2.32	0.16349629	1.3139E-05		
1	LILRA6	1.1943E-07	4.24	0.00694801	6.0255E-07	Q6PI73	LILRA6
1	LILRB3	3.2003E-08	3.61	0.00186177	1.6641E-07	O75022	LILRB3
1	LIMA1	0	-2.14	0	0	Q9UHB6	LIMA1
1	LIMCH1	1.8435E-97	19.67	1.0724E-92	3.1166E-96	Q9UPQ0	LIMCH1
1	LIMD1	3.5573E-92	2.22	2.0694E-87	5.8032E-91	Q9UGP4	LIMD1
1	LIMD1-AS1	5.2893E-12	2.37	3.077E-07	3.2332E-11		
1	LIMD2	4.1944E-44	2.10	2.4401E-39	4.6266E-43	Q9BT23	LIMD2
1	LIME1	1.314E-138	3.43	7.641E-134	2.736E-137	Q9H400	LIME1
1	LIMK1	0	-1.53	0	0	P53667	LIMK1
1	LIMS2	9.8095E-21	-3.31	5.7066E-16	7.5845E-20	Q7Z4I7	LIMS2
1	LIMS3	3.3683E-27	27.44	1.9595E-22	2.9457E-26	P0CW19	LIMS3
1	LIN28B	0.00787623	-7.62	1	0.02801027	Q6ZN17	LIN28B
1	LIN52	3.1026E-52	1.90	1.8049E-47	3.7092E-51	Q52LA3	LIN52
1	LIN7A	3.9504E-31	-5.53	2.2981E-26	3.6953E-30	O14910	LIN7A
1	LIN7B	6.3614E-15	1.72	3.7007E-10	4.2822E-14	Q9HAP6	LIN7B
1	LIN9	2.2204E-16	-1.72	1.2917E-11	1.5667E-15	Q5TKA1	LIN9
1	LINC-ROR	2.0619E-05	11.20	1	9.1222E-05		
1	LINC00032	0.00573247	-21.47	1	0.02070536		
1	LINC00094	1.5696E-47	1.78	9.1312E-43	1.7939E-46		
1	LINC00111	3.7411E-15	-18.43	2.1763E-10	2.5413E-14		
1	LINC00165	0.00109164	1.67	1	0.0042379		
1	LINC00173	4.7812E-10	-7.05	2.7814E-05	2.6993E-09		
1	LINC00174	5.1002E-20	-2.59	2.967E-15	3.8922E-19		
1	LINC00176	0.00138867	1.53	1	0.00534217		
1	LINC00184	4.706E-117	-10.65	2.738E-112	8.97E-116		
1	LINC00205	5.012E-190	5.44	2.916E-185	1.22E-188		
1	LINC00211	0.0103682	17.66	1	0.03632617		
1	LINC00221	6.985E-209	-8.33	4.064E-204	1.8E-207		
1	LINC00222	0.00096621	-2.30	1	0.00376908		
1	LINC00235	2.8273E-14	2.86	1.6447E-09	1.8669E-13		
1	LINC00240	4.2297E-31	-2.84	2.4606E-26	3.9553E-30		
1	LINC00243	8.7829E-08	-1.94	0.00510936	4.4588E-07		
1	LINC00265	5.4247E-21	-2.89	3.1558E-16	4.2144E-20		
1	LINC00266-1	0.00028349	-3.69	1	0.0011543		
1	LINC00271	2.78E-29	-4.79	1.6172E-24	2.5245E-28		
1	LINC00284	1.4186E-06	-10.78	0.08252381	6.7537E-06		
1	LINC00304	6.5913E-07	-6.66	0.03834451	3.1956E-06		
1	LINC00308	0.00188432	-1.77	1	0.00715337		
1	LINC00313	1.8736E-41	-6.52	1.0899E-36	1.9988E-40		
1	LINC00315	0.00257073	21.88	1	0.00962106		
1	LINC00319	0.00013505	-4.25	1	0.00056424		
1	LINC00324	9.2624E-08	-2.75	0.00538834	4.6969E-07		
1	LINC00342	1.4612E-13	2.17	8.5002E-09	9.43E-13		
1	LINC00355	3.378E-166	-7.91	1.965E-161	7.631E-165		
1	LINC00371	0.00126846	-2.06	1	0.00489721		

1	LINC00393	1.6837E-24	-5.44	9.7946E-20	1.401E-23		
1	LINC00408	4.5077E-07	22.60	0.02622336	2.2051E-06		
1	LINC00410	0.00166287	-25.06	1	0.00634375		
1	LINC00431	5.8145E-07	-2.19	0.03382533	2.8282E-06		
1	LINC00441	9.2805E-09	-4.47	0.00053989	4.9531E-08		
1	LINC00461	1.16E-103	-12.25	6.747E-99	2.044E-102		
1	LINC00467	6.0143E-85	-3.35	3.4988E-80	9.3052E-84		
1	LINC00470	1.1602E-31	-22.38	6.7491E-27	1.0928E-30	Q9BZP3	LINC00470
1	LINC00472	1.077E-16	-4.89	6.2654E-12	7.6716E-16		
1	LINC00476	9.7567E-96	-2.82	5.6759E-91	1.6291E-94		
1	LINC00479	5.2587E-40	-34.12	3.0592E-35	5.5031E-39		
1	LINC00482	4.6132E-05	-9.55	1	0.00019947	Q8N8I6	LINC00482
1	LINC00491	2.7909E-43	-9.51	1.6236E-38	3.0542E-42		
1	LINC00492	1.5327E-84	-8.19	8.9166E-80	2.3626E-83		
1	LINC00501	7.3919E-06	-6.00	0.43001492	3.3669E-05		
1	LINC00506	4.0928E-32	-10.79	2.3809E-27	3.8828E-31		
1	LINC00511	7.14E-104	2.36	4.154E-99	1.262E-102		
1	LINC00513	3.466E-147	-3.40	2.016E-142	7.399E-146		
1	LINC00518	9.2763E-15	-12.25	5.3964E-10	6.2171E-14	Q8N0U6	LINC00518
1	LINC00521	0	-4.53	0	0		
1	LINC00528	1.7003E-42	-7.32	9.8911E-38	1.8399E-41		
1	LINC00534	1.8485E-42	-8.26	1.0753E-37	1.9995E-41		
1	LINC00535	1.1962E-07	-2.56	0.00695873	6.0338E-07		
1	LINC00539	2.8129E-19	-3.42	1.6364E-14	2.1134E-18		
1	LINC00565	1.6019E-07	9.57	0.00931903	8.0233E-07		
1	LINC00571	2.3371E-07	-3.07	0.01359582	1.1603E-06		
1	LINC00574	1.2888E-05	-7.27	0.74974433	5.7793E-05		
1	LINC00578	1.59E-107	-9.81	9.247E-103	2.862E-106		
1	LINC00589	7.8042E-05	2.54	1	0.00033216		
1	LINC00592	0.00031712	-2.36	1	0.00128665		
1	LINC00598	3.5706E-08	-2.41	0.00207714	1.8513E-07		
1	LINC00601	0.00080397	3.06	1	0.00315824		
1	LINC00607	1.5357E-19	23.68	8.9337E-15	1.1605E-18		
1	LINC00612	0.0048356	-3.68	1	0.01761483		
1	LINC00618	3.6279E-09	1.93	0.00021105	1.9722E-08		
1	LINC00620	1.1046E-15	-27.65	6.4257E-11	7.6188E-15		
1	LINC00621	0.00190333	9.75	1	0.00721944		
1	LINC00622	8.1014E-05	-3.49	1	0.00034421		
1	LINC00623	1.4049E-28	-2.30	8.1727E-24	1.2612E-27		
1	LINC00624	4.4347E-14	-4.08	2.5799E-09	2.9102E-13		
1	LINC00632	4.5057E-57	156.21	2.6212E-52	5.614E-56		
1	LINC00634	0.00156631	-1.92	1	0.00598953		
1	LINC00636	1.6375E-09	-7.91	9.5259E-05	9.0318E-09		
1	LINC00637	0.00013501	-4.51	1	0.00056411		
1	LINC00641	1.0335E-39	2.32	6.0123E-35	1.0771E-38		
1	LINC00645	4.8865E-09	-81.54	0.00028427	2.6397E-08		
1	LINC00648	1.639E-111	-14.81	9.533E-107	3.033E-110		

1	LINC00654	7.0585E-08	-8.15	0.00410619	3.6013E-07		
1	LINC00656	4.4916E-22	-17.49	2.6129E-17	3.5608E-21		
1	LINC00659	6.6544E-11	5.46	3.8711E-06	3.8929E-10		
1	LINC00663	1.6742E-19	3.60	9.7393E-15	1.2644E-18		
1	LINC00664	2.0937E-07	15.12	0.01218003	1.0425E-06		
1	LINC00665	0	87.66	0	0		
1	LINC00672	1.6161E-18	-3.67	9.4013E-14	1.1956E-17	A0A1B0GWH6	LINC00672
1	LINC00677	0.00014685	-6.58	1	0.00061212		
1	LINC00685	1.0537E-07	-2.73	0.00612952	5.3263E-07		
1	LINC00691	2.0125E-06	-18.06	0.11707453	9.4989E-06		
1	LINC00698	7.2432E-30	-18.15	4.2136E-25	6.6493E-29		
1	LINC00702	1.7887E-24	2.46	1.0406E-19	1.4874E-23		
1	LINC00704	3.313E-14	1.60	1.9273E-09	2.1822E-13		
1	LINC00705	0.01227835	1.54	1	0.04261564		
1	LINC00707	5.3688E-26	2.25	3.1233E-21	4.5809E-25		
1	LINC00824	1.9391E-07	-6.25	0.01128081	9.6682E-07		
1	LINC00839	5.623E-241	184.23	3.271E-236	1.526E-239		
1	LINC00847	4.8544E-11	1.53	2.824E-06	2.8551E-10		
1	LINC00853	0.0111371	-2.05	1	0.03886794		
1	LINC00857	4.2741E-62	-2.20	2.4864E-57	5.5389E-61		
1	LINC00862	9.3465E-09	-2.55	0.00054373	4.9869E-08	A6NCI5	LINC00862
1	LINC00865	4.369E-118	-9.71	2.541E-113	8.368E-117		
1	LINC00866	0.00477244	-3.27	1	0.01739767		
1	LINC00869	2.7132E-86	-5.07	1.5784E-81	4.2316E-85		
1	LINC00871	2.1579E-30	-9.88	1.2553E-25	1.9958E-29		
1	LINC00879	9.2532E-19	-12.16	5.383E-14	6.888E-18		
1	LINC00880	4.1098E-14	-17.70	2.3909E-09	2.6994E-13		
1	LINC00881	0.00808262	-4.44	1	0.02869864		
1	LINC00882	5.1784E-07	-2.29	0.03012497	2.526E-06		
1	LINC00884	8.2365E-39	-16.88	4.7915E-34	8.4956E-38		
1	LINC00885	1.2841E-20	-50.17	7.4701E-16	9.8968E-20		
1	LINC00886	1.869E-148	-10.17	1.088E-143	4.01E-147		
1	LINC00887	3.9563E-46	-31.96	2.3015E-41	4.4586E-45		
1	LINC00888	1.4197E-22	2.13	8.2587E-18	1.1388E-21		
1	LINC00899	5.618E-18	-1.79	3.2682E-13	4.112E-17		
1	LINC00907	0.00166271	2.19	1	0.00634375		
1	LINC00909	4.5381E-13	1.52	2.64E-08	2.8802E-12		
1	LINC00910	6.462E-145	-4.56	3.759E-140	1.374E-143		
1	LINC00921	0.00023828	1.85	1	0.00097768		
1	LINC00926	1.226E-54	6.32	7.1323E-50	1.5019E-53		
1	LINC00928	7.9742E-05	-39.78	1	0.00033903		
1	LINC00933	3.0568E-06	-3.43	0.17782704	1.4244E-05		
1	LINC00941	1.2372E-57	2.09	7.1975E-53	1.5482E-56		
1	LINC00942	0.00116156	2.48	1	0.00449946		
1	LINC00943	1.5591E-06	-18.65	0.09069864	7.4058E-06		
1	LINC00944	1.7713E-49	-14.83	1.0305E-44	2.0634E-48		
1	LINC00950	4.2675E-07	-4.22	0.02482554	2.0902E-06		

1	LINC00957	0.00376756	-2.15	1	0.01387791		
1	LINC00958	7.488E-118	18.84	4.356E-113	1.432E-116		
1	LINC00959	1.1558E-12	-1.67	6.7238E-08	7.2322E-12		
1	LINC00960	8.0612E-12	1.90	4.6895E-07	4.8957E-11		
1	LINC00963	0	-2.95	0	0		
1	LINC00964	2.1666E-25	-20.30	1.2604E-20	1.8317E-24		
1	LINC00970	0.00393674	-8.78	1	0.01448826		
1	LINC00972	0.00913143	-15.97	1	0.03221615		
1	LINC00973	2.917E-204	42.92	1.697E-199	7.433E-203		
1	LINC00974	0.00103483	-11.14	1	0.00402702		
1	LINC00997	8.9917E-11	-3.85	5.2308E-06	5.2382E-10		
1	LINC00998	0	-2.21	0	0	A4D0T7	LINC00998
1	LINC01001	7.6297E-10	-2.18	4.4385E-05	4.2702E-09		
1	LINC01002	0	-3.51	0	0		
1	LINC01004	0	-3.84	0	0		
1	LINC01006	1.9568E-55	18.67	1.1383E-50	2.4127E-54	Q8NI28	LINC01006
1	LINC01010	2.2103E-06	-14.69	0.1285845	1.0403E-05		
1	LINC01011	2.0485E-13	-2.17	1.1917E-08	1.3152E-12		
1	LINC01012	6.1184E-09	-2.26	0.00035593	3.2905E-08		
1	LINC01023	2.8971E-06	-2.17	0.16853768	1.3525E-05		
1	LINC01032	7.6515E-07	-3.12	0.04451155	3.6973E-06		
1	LINC01036	3.3244E-16	-6.43	1.9339E-11	2.3376E-15		
1	LINC01037	1.1548E-29	-18.29	6.7178E-25	1.0563E-28		
1	LINC01058	1.2204E-07	-7.62	0.00709976	6.1539E-07		
1	LINC01063	1.113E-09	-7.64	6.475E-05	6.1867E-09		
1	LINC01075	8.2293E-32	-10.04	4.7873E-27	7.7704E-31		
1	LINC01081	0.00345277	-6.33	1	0.01277906		
1	LINC01085	3.4086E-38	275.33	1.9829E-33	3.4904E-37		
1	LINC01088	3.8857E-30	-5.86	2.2605E-25	3.579E-29		
1	LINC01091	6.1714E-12	-11.53	3.5901E-07	3.7632E-11		
1	LINC01094	0.00030969	-2.71	1	0.00125755		
1	LINC01098	1.4771E-08	-5.78	0.00085928	7.8166E-08		
1	LINC01099	1.3621E-27	-70.22	7.9239E-23	1.2017E-26		
1	LINC01106	0	-4.61	0	0		
1	LINC01107	3.0826E-07	4.91	0.01793253	1.5201E-06		
1	LINC01111	7.2263E-28	13.65	4.2038E-23	6.4122E-27		
1	LINC01115_2	2.9844E-11	4.90	1.7362E-06	1.7723E-10		
1	LINC01116	3.098E-61	3.52	1.8022E-56	3.9846E-60		
1	LINC01117	7.5367E-09	2.96	0.00043844	4.0372E-08		
1	LINC01121	0.01051676	-1.74	1	0.0368112		
1	LINC01122	8.6128E-06	-3.83	0.50104284	3.9089E-05		
1	LINC01123	1.313E-255	-7.06	7.636E-251	3.6E-254		
1	LINC01124	0.00222167	-2.88	1	0.00836366		
1	LINC01125	7.3425E-11	-1.97	4.2714E-06	4.2894E-10	A0A1B0GVN3	LINC01125
1	LINC01126	7.1439E-08	-3.46	0.00415589	3.6426E-07		
1	LINC01127	2.1043E-31	54.94	1.2242E-26	1.9758E-30		
1	LINC01128	6.2796E-27	1.80	3.6531E-22	5.4507E-26		

1	LINC01132	4.3794E-30	-10.85	2.5477E-25	4.0318E-29		
1	LINC01133	2.3922E-05	-14.99	1	0.00010539		
1	LINC01134	8.3688E-11	-3.10	4.8684E-06	4.8787E-10		
1	LINC01137	3.1353E-26	-2.50	1.8239E-21	2.6905E-25		
1	LINC01138	1.403E-207	-6.92	8.164E-203	3.603E-206		
1	LINC01152	1.6373E-22	-7.54	9.5249E-18	1.3112E-21		
1	LINC01158	0.00787587	17.61	1	0.02801027		
1	LINC01169	0.01102969	2.63	1	0.03851388		
1	LINC01173	0.00134921	-3.06	1	0.00519589		
1	LINC01176	5.2966E-58	-8.16	3.0813E-53	6.6507E-57		
1	LINC01182	2.7224E-10	7.50	1.5838E-05	1.5538E-09		
1	LINC01185	9.5722E-06	-5.14	0.5568539	4.3298E-05		
1	LINC01186	4.6071E-38	-7.49	2.6801E-33	4.7094E-37		
1	LINC01189	1.1808E-05	-12.53	0.68694221	5.3079E-05		
1	LINC01191	2.9613E-11	4.56	1.7227E-06	1.7595E-10		
1	LINC01192	4.314E-106	-24.85	2.51E-101	7.706E-105		
1	LINC01194	2.6754E-15	-3.48	1.5564E-10	1.8255E-14		
1	LINC01202	8.9702E-11	-17.62	5.2183E-06	5.2262E-10		
1	LINC01204	1.9765E-32	3.90	1.1498E-27	1.8849E-31		
1	LINC01205	4.834E-12	-15.46	2.8121E-07	2.9595E-11		
1	LINC01208	0.00010385	-11.83	1	0.00043792		
1	LINC01219	7.7919E-24	-24.22	4.5328E-19	6.3951E-23		
1	LINC01224	1.5995E-27	74.02	9.3049E-23	1.4094E-26		
1	LINC01226	8.798E-200	-5.96	5.118E-195	2.217E-198		
1	LINC01232	7.9742E-60	-2.49	4.6389E-55	1.016E-58		
1	LINC01234	1.124E-28	11.11	6.5389E-24	1.0116E-27		
1	LINC01237	1.078E-05	-2.32	0.62710194	4.8575E-05		
1	LINC01238_1	9.0595E-06	-3.89	0.52702951	4.1046E-05		
1	LINC01239	6.7696E-47	-4.16	3.9381E-42	7.6902E-46		
1	LINC01242	9.4021E-12	11.99	5.4696E-07	5.6993E-11		
1	LINC01249	0.00517666	-19.68	1	0.01879818		
1	LINC01250	0.0093374	-4.28	1	0.03290688		
1	LINC01251	1.3562E-05	16.91	0.7889415	6.0716E-05		
1	LINC01252	1.4831E-31	-8.25	8.6277E-27	1.3947E-30		
1	LINC01267	0.005325	-9.33	1	0.01931036		
1	LINC01268	0.00511218	-4.54	1	0.01857963		
1	LINC01270	1.391E-134	-3.65	8.091E-130	2.848E-133		
1	LINC01271	1.8302E-71	-6.08	1.0647E-66	2.5356E-70		
1	LINC01277	0.00249784	-2.71	1	0.00935789		
1	LINC01285	4.5305E-20	-18.19	2.6356E-15	3.4619E-19		
1	LINC01287	2.565E-239	-11.37	1.492E-234	6.94E-238		
1	LINC01291	1.244E-194	-8.11	7.237E-190	3.084E-193		
1	LINC01293	0.00055053	-5.70	1	0.00219362		
1	LINC01297_2	0.00886704	-8.27	1	0.03132706		
1	LINC01301	6.6594E-16	-3.23	3.8741E-11	4.6304E-15		
1	LINC01305	3.9698E-82	-19.10	2.3094E-77	5.9968E-81		
1	LINC01311	5.3841E-15	2.21	3.1321E-10	3.6302E-14		

1	LINC01315	8.4887E-08	2.79	0.0049382	4.3132E-07		
1	LINC01320	0.00199373	21.76	1	0.00754462		
1	LINC01322	3.1339E-17	1.71	1.8231E-12	2.2575E-16		
1	LINC01324	4.7545E-05	-10.01	1	0.00020532		
1	LINC01337	0.0002379	1.90	1	0.00097627		
1	LINC01339	8.1625E-14	-12.66	4.7484E-09	5.312E-13		
1	LINC01340	3.8712E-08	-4.35	0.00225204	2.0032E-07		
1	LINC01344	0.00163631	-3.81	1	0.00624691		
1	LINC01348	2.0243E-14	-2.99	1.1776E-09	1.3422E-13		
1	LINC01351	1.0437E-08	-5.85	0.00060717	5.5576E-08		
1	LINC01352	0.0002798	-27.22	1	0.00113986		
1	LINC01353	0.01193309	-8.25	1	0.04150648		
1	LINC01356	2.836E-26	-9.67	1.6498E-21	2.4355E-25		
1	LINC01358	2E-147	25.31	1.163E-142	4.275E-146		
1	LINC01359	0.00828125	-2.06	1	0.02937521		
1	LINC01361	7.014E-13	-90.90	4.0803E-08	4.4236E-12		
1	LINC01362	8.644E-137	-11.54	5.029E-132	1.787E-135		
1	LINC01372	1.75E-07	-2.85	0.01018065	8.747E-07		
1	LINC01374	0.00762497	-7.62	1	0.02717652		
1	LINC01375	0.00011622	-6.85	1	0.00048855		
1	LINC01376	2.3173E-20	-2.70	1.348E-15	1.7777E-19		
1	LINC01378	3.4718E-35	-6.88	2.0197E-30	3.4302E-34		
1	LINC01385	0.00273235	-7.05	1	0.01020033		
1	LINC01386	0.00071681	-3.24	1	0.00282634		
1	LINC01389	4.8316E-08	-2.69	0.00281071	2.4878E-07		
1	LINC01391	3.6473E-18	-4.63	2.1218E-13	2.6797E-17		
1	LINC01392	9.0535E-05	-7.62	1	0.00038346		
1	LINC01393	1.7958E-21	-43.62	1.0447E-16	1.4091E-20		
1	LINC01397	1.1876E-22	-7.50	6.9086E-18	9.5383E-22		
1	LINC01410	1.949E-108	3.06	1.134E-103	3.531E-107		
1	LINC01419	3.881E-42	-11.99	2.2577E-37	4.181E-41		
1	LINC01426	2.75E-104	12.90	1.6E-99	4.868E-103		
1	LINC01429	0.00014801	2.56	1	0.00061656		
1	LINC01431	5.8129E-15	-3.83	3.3816E-10	3.9161E-14		
1	LINC01433	0.00024996	4.38	1	0.00102329		
1	LINC01435	0.00738731	-7.62	1	0.02636014		
1	LINC01446	2.9135E-40	-4.17	1.6949E-35	3.0572E-39		
1	LINC01451	9.7744E-07	-5.15	0.05686145	4.6962E-06		
1	LINC01460	4.262E-10	-8.55	2.4794E-05	2.4111E-09		
1	LINC01468	2.9378E-14	11.29	1.709E-09	1.9384E-13		
1	LINC01470	1.3257E-05	-3.45	0.77119154	5.9409E-05		
1	LINC01478	0.00360227	-9.92	1	0.0132935		
1	LINC01481_1	8.5513E-26	-2.16	4.9746E-21	7.2771E-25		
1	LINC01482	0.00019503	-5.97	1	0.0008062		
1	LINC01483	1.224E-267	481.22	7.122E-263	3.4E-266		
1	LINC01493	0.00901532	-17.80	1	0.03182193		
1	LINC01494	9.6545E-21	-16.18	5.6164E-16	7.4657E-20		

1	LINC01497	4.7381E-27	236.53	2.7563E-22	4.1293E-26		
1	LINC01500	3.7244E-08	-30.31	0.00216664	1.9295E-07		
1	LINC01503	4.785E-262	-17.91	2.784E-257	1.324E-260		
1	LINC01504	3.78E-35	15.49	2.199E-30	3.7334E-34		
1	LINC01510	0.01051235	-5.67	1	0.03679797		
1	LINC01529	0.00031622	1.98	1	0.00128308		
1	LINC01535	1.7186E-33	21.17	9.9977E-29	1.6635E-32		
1	LINC01537	4.7151E-26	44.10	2.7429E-21	4.0308E-25		
1	LINC01551	0.00287763	-9.37	1	0.01072341		
1	LINC01560	4.2229E-07	-1.99	0.02456632	2.0691E-06		
1	LINC01564	2.61E-10	-11.80	1.5183E-05	1.4909E-09		
1	LINC01572	0	-2.38	0	0		
1	LINC01583	9.8275E-09	57.07	0.00057171	5.2388E-08		
1	LINC01585	0.00808262	-4.44	1	0.02869864		
1	LINC01588	1.8022E-58	2.45	1.0484E-53	2.2723E-57		
1	LINC01589	4.754E-08	-4.81	0.00276557	2.4487E-07		
1	LINC01597	2.8727E-06	-8.68	0.16711548	1.3415E-05		
1	LINC01598_1	0.00435057	7.94	1	0.01593666		
1	LINC01598_2	0.00458246	-3.44	1	0.01674602		
1	LINC01600	0.00014788	-2.34	1	0.00061612		
1	LINC01605	2.3241E-43	-4.54	1.352E-38	2.5452E-42		
1	LINC01607	2.8263E-08	-6.53	0.00164419	1.4739E-07		
1	LINC01608	4.877E-159	-15.28	2.837E-154	1.082E-157		
1	LINC01609	5.5846E-31	-10.22	3.2488E-26	5.2139E-30		
1	LINC01611	1.506E-53	-10.61	8.7613E-49	1.826E-52		
1	LINC01615	5.2077E-08	7.69	0.00302953	2.6756E-07		
1	LINC01618	1.4404E-10	-4.57	8.3793E-06	8.3227E-10		
1	LINC01621	3.0634E-05	-17.25	1	0.00013403		
1	LINC01625	0.00438725	-19.70	1	0.0160619		
1	LINC01629	6.673E-154	-13.23	3.882E-149	1.453E-152		
1	LINC01630	0.01431565	-3.52	1	0.0493686		
1	LINC01635	0.00524032	3.73	1	0.01902104		
1	LINC01637	2.0916E-51	-7.51	1.2168E-46	2.4792E-50		
1	LINC01655	5.2007E-18	-128.41	3.0254E-13	3.8108E-17		
1	LINC01664	0.00134288	7.64	1	0.00517357		
1	LINC01666	6.0151E-10	-4.32	3.4992E-05	3.3838E-09		
1	LINC01667	7.0762E-09	-4.81	0.00041165	3.7954E-08		
1	LINC01669	0.00012138	-4.91	1	0.0005092		
1	LINC01679	1.3919E-22	-8.53	8.0971E-18	1.1168E-21		
1	LINC01680	0.00489731	-21.42	1	0.01783163		
1	LINC01687	3.7587E-22	-2.25	2.1866E-17	2.9831E-21		
1	LINC01691	9.1257E-20	-3.41	5.3088E-15	6.9323E-19		
1	LINC01695	1.0584E-11	-15.11	6.1573E-07	6.4025E-11		
1	LINC01697	6.9972E-45	-9.12	4.0705E-40	7.7786E-44		
1	LINC01698	1.9714E-07	-7.13	0.01146823	9.8246E-07		
1	LINC01700	0.01039832	-17.77	1	0.03642073		
1	LINC01701	2.5355E-07	-6.32	0.01474981	1.2569E-06		

1	LINC01703	3.6526E-15	-4.05	2.1249E-10	2.482E-14		
1	LINC01704	0.0025432	-6.00	1	0.00952292		
1	LINC01707	4.8894E-07	-5.27	0.02844367	2.3878E-06		
1	LINC01715	0.0011116	-1.74	1	0.00431311		
1	LINC01719	2.2833E-34	-2.73	1.3283E-29	2.2373E-33		
1	LINC01725	1.1711E-12	-6.95	6.8128E-08	7.3264E-12		
1	LINC01732	0.00328006	-21.40	1	0.01215998		
1	LINC01747	5.9621E-13	-15.95	3.4684E-08	3.7683E-12		
1	LINC01748	2.2577E-24	-3.78	1.3134E-19	1.8728E-23		
1	LINC01759	3.2029E-07	-17.36	0.01863254	1.5781E-06		
1	LINC01762	3.1654E-06	-4.24	0.18414349	1.4737E-05		
1	LINC01764	1.1357E-24	-10.79	6.6069E-20	9.4723E-24		
1	LINC01768	1.0759E-06	-3.81	0.06259019	5.1565E-06		
1	LINC01770	6.2853E-15	-9.64	3.6564E-10	4.2315E-14		
1	LINC01771	1.0683E-07	-2.39	0.00621446	5.3996E-07		
1	LINC01772	3.9065E-05	-1.56	1	0.0001697		
1	LINC01775	1.7849E-05	2.59	1	7.9292E-05		
1	LINC01776	7.6598E-15	3.02	4.456E-10	5.1455E-14		
1	LINC01783	0.00861968	8.34	1	0.03050054		
1	LINC01786	0.00118889	2.71	1	0.00460101		
1	LINC01800	1.4716E-06	-21.09	0.08560991	6.9994E-06		
1	LINC01801	0.00889711	17.64	1	0.0314242		
1	LINC01806	1.629E-114	-5.38	9.475E-110	3.06E-113		
1	LINC01807	2.2779E-45	-11.20	1.3252E-40	2.5455E-44		
1	LINC01812	0.00038576	33.60	1	0.00155475		
1	LINC01816	6.8857E-05	-3.34	1	0.00029393		
1	LINC01828	3.5388E-05	8.94	1	0.00015427		
1	LINC01830	2.0628E-05	-14.34	1	9.1254E-05		
1	LINC01833	5.8717E-31	-6.83	3.4158E-26	5.4802E-30		
1	LINC01835	4.4923E-25	-31.17	2.6134E-20	3.7755E-24		
1	LINC01836	5.3118E-13	1.94	3.0901E-08	3.3639E-12		
1	LINC01842	0.00215745	1.86	1	0.00813768		
1	LINC01843	7.4047E-12	-9.37	4.3076E-07	4.5016E-11		
1	LINC01850	6.8331E-16	-11.46	3.9751E-11	4.7481E-15		
1	LINC01864	0.00528288	-3.34	1	0.01916836		
1	LINC01873	3.6897E-09	-13.98	0.00021464	2.0055E-08		
1	LINC01876	3.3855E-23	7.27	1.9695E-18	2.7437E-22		
1	LINC01882	7.9748E-10	-8.34	4.6393E-05	4.4604E-09		
1	LINC01899	0.00060574	-25.31	1	0.00240403		
1	LINC01901	5.7592E-13	-7.66	3.3503E-08	3.6436E-12		
1	LINC01902	0.00360227	-9.90	1	0.0132935		
1	LINC01905	0.0040867	-19.71	1	0.01502114		
1	LINC01909	7.091E-08	2.42	0.00412513	3.617E-07		
1	LINC01910	1.4071E-10	6.06	8.1854E-06	8.135E-10		
1	LINC01929	1.3373E-20	193.59	7.7798E-16	1.0303E-19		
1	LINC01930	2.9448E-16	117.36	1.7131E-11	2.0727E-15		
1	LINC01933	7.7965E-07	3.32	0.0453551	3.7658E-06		

1	LINC01941	0.00022876	-5.99	1	0.00094015		
1	LINC01943	3.22E-07	3.35	0.01873209	1.5863E-06		
1	LINC01948	3.3498E-11	-14.35	1.9487E-06	1.9848E-10		
1	LINC01952	1.1203E-06	-3.63	0.06517191	5.3648E-06		
1	LINC01956	3.8847E-34	296.41	2.2599E-29	3.7988E-33		
1	LINC01958	3.8629E-05	-32.78	1	0.00016785		
1	LINC01967	7.2895E-81	-12.27	4.2406E-76	1.0898E-79		
1	LINC01968	5.7778E-08	-6.97	0.00336118	2.9622E-07		
1	LINC01970	2.5737E-06	-2.57	0.14972335	1.2057E-05		
1	LINC01977	5.6775E-08	-6.32	0.00330282	2.912E-07		
1	LINC01980	4.099E-141	-11.35	2.384E-136	8.614E-140		
1	LINC01981	0.00032948	-14.04	1	0.00133503		
1	LINC01983	2.4661E-15	-9.31	1.4346E-10	1.6844E-14		
1	LINC01989	0.00439279	-2.11	1	0.01608119		
1	LINC01994	0.000508	-4.10	1	0.0020286		
1	LINC02004	8.7265E-24	-48.69	5.0766E-19	7.1531E-23		
1	LINC02006	0.00103701	1.55	1	0.00403498		
1	LINC02008	1.878E-08	-4.65	0.00109249	9.8886E-08		
1	LINC02009	0.00102811	-9.45	1	0.00400248		
1	LINC02012	2.5294E-05	-3.64	1	0.00011121		
1	LINC02014	2.103E-06	-8.77	0.12233863	9.9156E-06		
1	LINC02015	0.0001451	-3.42	1	0.00060512		
1	LINC02018	9.3028E-08	11.14	0.00541181	4.7162E-07		
1	LINC02021	2.7635E-12	3.46	1.6077E-07	1.7081E-11		
1	LINC02027	0.00081675	-12.78	1	0.00320561		
1	LINC02028	0.00290345	-3.71	1	0.01081547		
1	LINC02035	2.2112E-20	-1.98	1.2863E-15	1.697E-19		
1	LINC02041	0.00031359	-30.87	1	0.00127287		
1	LINC02042	0.01219299	-5.95	1	0.04233704		
1	LINC02043	5.5247E-26	10.19	3.2139E-21	4.7125E-25		
1	LINC02050	7.0258E-08	-9.31	0.0040872	3.5849E-07		
1	LINC02053	1.8146E-06	57.89	0.10556076	8.5885E-06		
1	LINC02055	2.274E-116	-12.48	1.323E-111	4.315E-115		
1	LINC02057	5.8524E-16	2.14	3.4046E-11	4.0803E-15		
1	LINC02062	4.9133E-10	-1.57	2.8583E-05	2.7726E-09		
1	LINC02066	1.0658E-06	12.47	0.0619994	5.1083E-06		
1	LINC02068	2.3834E-07	6.28	0.01386517	1.1828E-06		
1	LINC02076	1.2065E-19	135.92	7.0185E-15	9.1351E-19		
1	LINC02081	2.0813E-23	-2.09	1.2108E-18	1.6944E-22		
1	LINC02082	6.5389E-06	-5.35	0.38039681	2.9891E-05		
1	LINC02084	4.447E-19	-6.36	2.587E-14	3.329E-18		
1	LINC02086	1.9031E-11	-1.61	1.1071E-06	1.14E-10		
1	LINC02095	1.9769E-06	-20.26	0.11500529	9.3356E-06		
1	LINC02098	1.3799E-06	-8.73	0.0802767	6.5741E-06		
1	LINC02099	0.00066266	2.93	1	0.00262009		
1	LINC02100	1.314E-128	-18.95	7.646E-124	2.639E-127		
1	LINC02102	8.5325E-08	-14.33	0.00496369	4.3351E-07		

1	LINC02104	1.3204E-12	86.12	7.6815E-08	8.2411E-12		
1	LINC02118	0.00278808	21.81	1	0.01040169		
1	LINC02137	0.00269985	2.86	1	0.01008419		
1	LINC02139	0.00140729	-6.73	1	0.00540559		
1	LINC02141	1.5975E-05	-6.65	0.92934217	7.1198E-05		
1	LINC02145	3.8145E-07	15.54	0.02219026	1.8728E-06		
1	LINC02152	9.731E-117	-28.10	5.661E-112	1.851E-115		
1	LINC02156	1.3588E-60	-6.28	7.905E-56	1.7385E-59		
1	LINC02161	2.4059E-10	-13.32	1.3996E-05	1.3764E-09		
1	LINC02175	0.00486584	-3.85	1	0.01772149		
1	LINC02198	9.133E-05	-5.01	1	0.00038671		
1	LINC02203	0.00951191	3.34	1	0.03344289		
1	LINC02206	0.00022476	-7.20	1	0.00092423		
1	LINC02210-CR	0.00631479	5.06	1	0.02269175		
1	LINC02211	3.1893E-07	-3.57	0.01855322	1.5715E-06		
1	LINC02220	2.2113E-29	-11.58	1.2864E-24	2.0122E-28		
1	LINCR-0001	2.1621E-05	11.63	1	9.5526E-05		
1	LINGO2	4.0645E-08	-2.46	0.0023645	2.101E-07	Q7L985	LINGO2
1	LINP1	4.0487E-09	1.54	0.00023553	2.1953E-08		
1	LIPA	0	-1.94	0	0	P38571	LIPA
1	LIPE	5.395E-28	7.16	3.1385E-23	4.8048E-27	Q05469	LIPE
1	LIPG	8.175E-201	8.31	4.756E-196	2.069E-199	Q9Y5X9	LIPG
1	LIPH	6.871E-189	-9.56	3.997E-184	1.665E-187	Q8WWY8	LIPH
1	LIPJ	0.00125802	-23.39	1	0.00485853	Q5W064	LIPJ
1	LIPM	6.9115E-19	-19.36	4.0207E-14	5.1541E-18	Q5VYY2	LIPM
1	LIPT2	7.8068E-08	-1.85	0.00454151	3.9744E-07	A6NK58	LIPT2
1	LITAF	0	-2.50	0	0	Q99732	LITAF
1	LIX1L	2.061E-32	3.53	1.199E-27	1.9649E-31	Q8IVB5	LIX1L
1	LL09NC01-139	0.00840389	-2.65	1	0.0297848		
1	LL09NC01-251	0.000139	-2.69	1	0.0005803		
1	LL0XNC01-116	3.8966E-16	16.75	2.2668E-11	2.735E-15		
1	LL0XNC01-116	3.5867E-36	18.40	2.0865E-31	3.587E-35		
1	LL0XNC01-240	5.9788E-17	40.11	3.4781E-12	4.2818E-16		
1	LL21NC02-21A	0.00250693	6.78	1	0.00938954		
1	LL22NC03-80A	4.9045E-84	2.25	2.8532E-79	7.5262E-83		
1	LLfos-48D6.2	0.00584139	-2.98	1	0.02108048		
1	LLGL1	9.6886E-43	1.59	5.6363E-38	1.0519E-41	Q15334	LLGL1
1	LLGL2	0	-3.47	0	0	Q6P1M3	LLGL2
1	LLNLF-173C4.1	0.00521516	-3.24	1	0.01893208		
1	LLPH	4.5027E-25	1.74	2.6194E-20	3.7836E-24	Q9BRT6	LLPH
1	LMAN1	5.863E-139	2.12	3.411E-134	1.223E-137	P49257	LMAN1
1	LMAN1L	5.8599E-05	-17.94	1	0.00025145	Q9HAT1	LMAN1L
1	LMBRD1	0	-2.24	0	0	Q9NUN5	LMBRD1
1	LMBRD2	0	-3.41	0	0	Q68DH5	LMBRD2
1	LMCD1-AS1	8.1007E-10	-2.39	4.7125E-05	4.5295E-09		
1	LMF1	0	-1.69	0	0	Q96S06	LMF1
1	LMF2	1.3276E-46	1.81	7.7233E-42	1.5029E-45	Q9BU23	LMF2

1	LMLN	0	-1.53	0	0	Q96KR4	LMLN
1	LMNA	0	-2.24	0	0	P02545	LMNA
1	LMNTD1	4.7727E-13	3.69	2.7765E-08	3.0258E-12	Q8N9Z9	LMNTD1
1	LMO2	1.6367E-85	-12.57	9.5211E-81	2.5362E-84	P25791	LMO2
1	LMO3	4.6817E-13	-5.44	2.7236E-08	2.9694E-12	Q8TAP4	LMO3
1	LMOD1	4.344E-13	-7.30	2.5271E-08	2.7591E-12	P29536	LMOD1
1	LMOD3	0.00214933	-4.33	1	0.00810864	Q0VAK6	LMOD3
1	LMTK2	0	-2.33	0	0	Q8IWU2	LMTK2
1	LMTK3	3.754E-171	-5.30	2.184E-166	8.612E-170	Q96Q04	LMTK3
1	LNCSRLR	0.00368054	2.56	1	0.01357026		
1	LNX1	9.799E-14	-2.37	5.7004E-09	6.36E-13	Q8TBB1	LNX1
1	LNX2	0	-2.01	0	0	Q8N448	LNX2
1	LONP1	1.322E-53	1.54	7.6905E-49	1.6042E-52	P36776	LONP1
1	LONRF1	0	-1.75	0	0	Q17RB8	LONRF1
1	LOX	0	-2.06	0	0	P28300	LOX
1	LOXL1	2.0212E-06	-2.78	0.11758026	9.5392E-06	Q08397	LOXL1
1	LOXL1-AS1	3.3985E-23	7.52	1.9771E-18	2.754E-22		
1	LOXL2	9.1154E-79	7.06	5.3028E-74	1.3377E-77	Q9Y4K0	LOXL2
1	LOXL4	0	-12.23	0	0	Q96JB6	LOXL4
1	LPAL2	1.0853E-07	-4.19	0.00631369	5.484E-07	Q16609	LPAL2
1	LPAR2	2.1358E-43	-2.12	1.2425E-38	2.3412E-42	Q9HBW0	LPAR2
1	LPAR3	5.7208E-51	-12.81	3.328E-46	6.7492E-50	Q9UBY5	LPAR3
1	LPAR5	2.76E-202	-15.99	1.605E-197	7.008E-201	Q9H1C0	LPAR5
1	LPAR6	1.6021E-23	-6.06	9.3202E-19	1.307E-22	P43657	LPAR6
1	LPCAT1	0	-1.84	0	0	Q8NF37	LPCAT1
1	LPCAT3	0	-1.94	0	0	Q6P1A2	LPCAT3
1	LPCAT4	0	-1.50	0	0	Q643R3	LPCAT4
1	LPGAT1	0	-1.73	0	0	Q92604	LPGAT1
1	LPIN2	1.008E-102	2.68	5.8635E-98	1.765E-101	Q92539	LPIN2
1	LPO	4.8603E-14	-19.59	2.8274E-09	3.1848E-13	P22079	LPO
1	LPP-AS2	3.7825E-17	-2.76	2.2005E-12	2.72E-16		
1	LRBA	0	-1.95	0	0	P50851	LRBA
1	LRCH2	5.4E-140	6.01	3.141E-135	1.132E-138	Q5VUJ6	LRCH2
1	LRCH4	0	-1.93	0	0	O75427	LRCH4
1	LRCOL1	0.00695484	-4.53	1	0.02487339	A6NCL2	LRCOL1
1	LRFN1	0.00250455	1.87	1	0.00938181	Q9P244	LRFN1
1	LRFN4	0	-1.87	0	0	Q6PJG9	LRFN4
1	LRFN5	4.7166E-31	-7.87	2.7439E-26	4.4071E-30	Q96NI6	LRFN5
1	LRG1	1.55E-133	-38.61	9.014E-129	3.162E-132	P02750	LRG1
1	LRIF1	6.3443E-79	2.42	3.6907E-74	9.3294E-78	Q5T3J3	LRIF1
1	LRIG1	2.1612E-82	5.20	1.2573E-77	3.269E-81	Q96JA1	LRIG1
1	LRIG3	1.9043E-73	-3.13	1.1078E-68	2.6778E-72	Q6UXM1	LRIG3
1	LRIT3	0.00017186	2.67	1	0.00071295	Q3SXY7	LRIT3
1	LRMP	5.772E-45	-5.82	3.3578E-40	6.4227E-44	Q12912	LRMP
1	LRP1	0	-5.99	0	0	Q07954	LRP1
1	LRP11	0	-3.68	0	0	Q86VZ4	LRP11
1	LRP12	0	-2.03	0	0	Q9Y561	LRP12

1	LRP1B	3.6004E-26	-2.73	2.0945E-21	3.0861E-25	Q9NZR2	LRP1B
1	LRP2	2.1214E-26	-7.19	1.2341E-21	1.8259E-25	P98164	LRP2
1	LRP2BP	9.791E-27	-2.98	5.6958E-22	8.4696E-26	Q9P2M1	LRP2BP
1	LRP3	4.527E-196	35.37	2.634E-191	1.127E-194	O75074	LRP3
1	LRP4	0	-5.53	0	0	O75096	LRP4
1	LRP4-AS1	1.5646E-44	-4.43	9.102E-40	1.7317E-43		
1	LRP5	0	-4.54	0	0	O75197	LRP5
1	LRP6	6.3068E-49	1.91	3.6689E-44	7.3086E-48	O75581	LRP6
1	LRR1	1.2509E-68	2.41	7.2772E-64	1.6951E-67	Q96L50	LRR1
1	LRRC1	0	-3.59	0	0	Q9BTT6	LRRC1
1	LRRC14B	0.00135409	-10.55	1	0.00521399		
1	LRRC17	1.0818E-08	-6.78	0.00062935	5.7538E-08	Q8N6Y2	LRRC17
1	LRRC2	1.3188E-09	-6.12	7.6718E-05	7.3092E-09		
1	LRRC2-AS1	0.00221501	-25.36	1	0.00834127		
1	LRRC24	5.6407E-12	3.52	3.2814E-07	3.4436E-11	Q50LG9	LRRC24
1	LRRC29	3.16E-07	-1.70	0.01838317	1.5576E-06	Q8WV35	LRRC29
1	LRRC3	2.8506E-48	-10.46	1.6583E-43	3.2857E-47	Q9BY71	LRRC3
1	LRRC31	1.8198E-07	-10.98	0.01058633	9.0869E-07		
1	LRRC32	0.00505681	-5.63	1	0.01838822	Q14392	LRRC32
1	LRRC34	3.2955E-88	-11.13	1.9171E-83	5.2209E-87	Q8IZ02	LRRC34
1	LRRC37A17P	1.3155E-11	-1.89	7.6525E-07	7.9334E-11		
1	LRRC37A2	4.3274E-12	-1.59	2.5174E-07	2.6538E-11	A6NM11	LRRC37A2
1	LRRC37A3	2.7129E-15	-1.98	1.5782E-10	1.8506E-14	O60309	LRRC37A3
1	LRRC37A4P	1.3465E-32	2.63	7.833E-28	1.2873E-31		
1	LRRC37A5P	1.8878E-05	-2.04	1	8.3711E-05		
1	LRRC37A7P	0.00012398	-6.11	1	0.00051953		
1	LRRC37A9P	0.00143892	-3.09	1	0.00551835		
1	LRRC4	0.00650219	-19.56	1	0.02332625	Q9HBW1	LRRC4
1	LRRC45	2.755E-110	2.39	1.603E-105	5.057E-109	Q96CN5	LRRC45
1	LRRC47	7.978E-102	1.82	4.6413E-97	1.389E-100	Q8N1G4	LRRC47
1	LRRC49	7.2938E-20	1.80	4.2431E-15	5.5487E-19	Q8IUZ0	LRRC49
1	LRRC56	1.7521E-09	-1.95	0.00010193	9.6548E-09		
1	LRRC59	6.8304E-75	1.74	3.9735E-70	9.7152E-74	Q96AG4	LRRC59
1	LRRC6	6.2644E-60	8.26	3.6443E-55	7.9831E-59	Q86X45	LRRC6
1	LRRC61	9.8362E-74	2.62	5.7221E-69	1.3865E-72		
1	LRRC63	9.018E-11	-6.24	5.2461E-06	5.2524E-10		
1	LRRC66	3.0692E-09	3.81	0.00017855	1.6746E-08	Q68CR7	LRRC66
1	LRRC69	4.8939E-13	-1.92	2.847E-08	3.1016E-12		
1	LRRC70	0.00288889	-5.61	1	0.0107626	Q7Z2Q7	LRRC70
1	LRRC73	5.9579E-09	-2.87	0.00034659	3.2065E-08		
1	LRRC75B	1.876E-21	-4.41	1.0914E-16	1.4716E-20		
1	LRRC8A	0	-3.28	0	0	Q8IWT6	LRRC8A
1	LRRC8B	6.9693E-52	-2.49	4.0543E-47	8.2979E-51	Q6P9F7	LRRC8B
1	LRRC8C	4.2841E-92	2.17	2.4923E-87	6.9831E-91	Q8TDW0	LRRC8C
1	LRRC8D	2.9461E-76	1.81	1.7138E-71	4.2401E-75	Q7L1W4	LRRC8D
1	LRRC9	4.6193E-17	-8.39	2.6873E-12	3.3143E-16		
1	LRRCC1	0	-2.67	0	0	Q9C099	LRRCC1

1	LRRIQ1	8.3412E-29	-8.07	4.8524E-24	7.5196E-28		
1	LRRIQ3	1.4327E-33	-6.56	8.3344E-29	1.3891E-32	A6PVS8	LRRIQ3
1	LRRIQ4	1.7322E-17	-6.70	1.0077E-12	1.2558E-16	A6NIV6	LRRIQ4
1	LRRK2	2.6244E-61	-9.43	1.5267E-56	3.3784E-60	Q5S007	LRRK2
1	LRRN1	1.3375E-09	-3.72	7.781E-05	7.4112E-09	Q6UXK5	LRRN1
1	LRRN2	1.7746E-10	-4.62	1.0323E-05	1.0217E-09	O75325	LRRN2
1	LRRN4	5.292E-184	20.47	3.079E-179	1.261E-182	Q8WUT4	LRRN4
1	LRRTM4	1.6288E-07	-5.18	0.00947562	8.1546E-07	Q86VH4	LRRTM4
1	LRTM2	3.1397E-08	35.48	0.00182649	1.6336E-07	Q8N967	LRTM2
1	LRTOMT	0	-1.78	0	0	Q8WZ04	LRTOMT
1	LSM10	9.2513E-79	2.65	5.3818E-74	1.3573E-77	Q969L4	LSM10
1	LSM11	2.0765E-66	2.55	1.208E-61	2.7783E-65	P83369	LSM11
1	LSM14B	4.1173E-82	2.24	2.3952E-77	6.2165E-81	Q9BX40	LSM14B
1	LSM4	1.5972E-20	1.56	9.2914E-16	1.2292E-19	Q9Y4Z0	LSM4
1	LSMEM1	2.0178E-10	-2.00	1.1738E-05	1.1583E-09	Q8N8F7	LSMEM1
1	LSP1	1.0024E-07	-2.76	0.00583119	5.0724E-07	P33241	LSP1
1	LSR	0	-2.53	0	0	Q86X29	LSR
1	LSS	0	-2.47	0	0	P48449	LSS
1	LTA4H	0	-1.62	0	0	P09960	LTA4H
1	LTB4R	3.6312E-42	2.39	2.1124E-37	3.9133E-41	Q15722	LTB4R
1	LTB4R2	3.4561E-36	2.65	2.0106E-31	3.4581E-35	Q9NPC1	LTB4R2
1	LTBP1	8.3219E-47	3.47	4.8412E-42	9.4462E-46	Q14766	LTBP1
1	LTBP2	2.8813E-80	2.91	1.6762E-75	4.2902E-79	Q14767	LTBP2
1	LTBP3	9.493E-39	1.95	5.5224E-34	9.7811E-38	Q9NS15	LTBP3
1	LTBP4	2.9253E-31	1.75	1.7018E-26	2.7412E-30	Q8N2S1	LTBP4
1	LTC4S	0.00066106	-4.04	1	0.00261397	Q16873	LTC4S
1	LTK	4.6835E-09	-6.22	0.00027246	2.5319E-08	P29376	LTK
1	LTV1	1.3035E-58	2.65	7.5832E-54	1.646E-57	Q96GA3	LTV1
1	LUC7L3	0	-2.20	0	0	O95232	LUC7L3
1	LUCAT1	2.644E-109	-4.45	1.538E-104	4.821E-108		
1	LUM	8.8891E-73	-7.42	5.1711E-68	1.2443E-71	P51884	LUM
1	LURAP1	3.4318E-24	4.74	1.9964E-19	2.8342E-23	Q96LR2	LURAP1
1	LURAP1L	1.105E-209	7.02	6.43E-205	2.854E-208		
1	LURAP1L-AS1	0.00023528	-4.03	1	0.00096591		
1	LUZP1	2.8798E-75	1.81	1.6753E-70	4.1111E-74	Q86V48	LUZP1
1	LUZP2	2.6501E-08	-4.98	0.00154168	1.3847E-07	Q86TE4	LUZP2
1	LVCAT1	6.6309E-38	-33.19	3.8575E-33	6.7663E-37		
1	LVCAT5	1.3463E-91	-11.23	7.8321E-87	2.1871E-90		
1	LXN	9.8674E-19	-2.62	5.7403E-14	7.3349E-18	Q9BS40	LXN
1	LY6D	0	-16.48	0	0	Q14210	LY6D
1	LY6E	0	-2.75	0	0	Q16553	LY6E
1	LY6G5C	0.00022033	1.60	1	0.00090673	Q5SRR4	LY6G5C
1	LY6K	5.5546E-27	1.68	3.2313E-22	4.8287E-26	Q17RY6	LY6K
1	LY75	4.8459E-25	-20.38	2.819E-20	4.0702E-24	O60449	LY75
1	LY75-CD302	4.4893E-06	-5.70	0.26115928	2.0688E-05		
1	LYG1	2.0562E-07	-1.88	0.01196173	1.024E-06	Q8N1E2	LYG1
1	LYL1	5.986E-57	13.07	3.4823E-52	7.4535E-56	P12980	LYL1

1	LYN	0	-3.22	0	0	P07948	LYN
1	LYNX1_1	1.0799E-57	-9.96	6.2824E-53	1.3516E-56		
1	LYPD1	3.6849E-91	35.30	2.1437E-86	5.9645E-90	Q8N2G4	LYPD1
1	LYPD2	2.7131E-09	-16.24	0.00015783	1.4841E-08	Q6UXB3	LYPD2
1	LYPD3	0	-17.14	0	0	O95274	LYPD3
1	LYPD5	3.9917E-55	-6.08	2.3221E-50	4.9052E-54	Q6UWN5	LYPD5
1	LYPD6B	8.196E-118	-24.81	4.768E-113	1.567E-116	Q8NI32	LYPD6B
1	LYPLA1P2	8.1552E-05	-3.84	1	0.00034639		
1	LYPLAL1	0	-2.60	0	0	Q5VWZ2	LYPLAL1
1	LYRM2	2.1215E-27	1.54	1.2341E-22	1.8648E-26	Q9NU23	LYRM2
1	LYSMD2	1.7187E-78	4.38	9.9983E-74	2.5128E-77		
1	LYSMD3	5.4971E-27	2.01	3.1979E-22	4.7801E-26	Q7Z3D4	LYSMD3
1	LYST	1.4588E-55	-2.77	8.4861E-51	1.7998E-54	Q99698	LYST
1	LZIC	9.6594E-52	1.85	5.6192E-47	1.1475E-50	Q8WZA0	LZIC
1	LZTFL1	3.698E-14	1.59	2.1513E-09	2.4327E-13	Q9NQ48	LZTFL1
1	LZTR1	4.5081E-72	2.27	2.6225E-67	6.2755E-71	Q8N653	LZTR1
1	LZTS3	1.258E-133	3.30	7.316E-129	2.568E-132	O60299	LZTS3
1	M6PR	3.3939E-52	1.62	1.9743E-47	4.0558E-51	P20645	M6PR
1	MAATS1	4.2507E-06	-5.35	0.2472794	1.9614E-05	Q7Z4T9	MAATS1
1	MAB21L1	1.9004E-11	-5.17	1.1055E-06	1.1386E-10	Q13394	MAB21L1
1	MAB21L3	9.8591E-05	-4.16	1	0.00041649	Q8N8X9	MAB21L3
1	MACC1	1.157E-262	-33.46	6.731E-258	3.204E-261	Q6ZN28	MACC1
1	MACF1	2.0717E-27	1.77	1.2052E-22	1.8219E-26	Q9UPN3	MACF1
1	MACROD1	0	-2.56	0	0	Q9BQ69	MACROD1
1	MACROD2	8.6221E-55	11.10	5.0158E-50	1.0571E-53	A1Z1Q3	MACROD2
1	MAD2L2	8.3603E-59	2.24	4.8635E-54	1.0568E-57	Q9UI95	MAD2L2
1	MADCAM1	0.00586112	2.12	1	0.02114772	Q13477	MADCAM1
1	MAFA	4.1848E-05	-3.86	1	0.00018135	Q8NHW3	MAFA
1	MAFG-AS1	2.7113E-24	3.00	1.5773E-19	2.243E-23		
1	MAGEA10	0	-10.53	0	0	P43363	MAGEA10
1	MAGEA12	4.883E-186	-6.90	2.84E-181	1.171E-184	P43365	MAGEA12
1	MAGEA2	4.3011E-25	-8.77	2.5021E-20	3.6168E-24	P43356	MAGEA2
1	MAGEA2B	6.3819E-06	-5.59	0.37126079	2.92E-05		
1	MAGEA3	3.241E-200	-7.13	1.886E-195	8.181E-199	P43357	MAGEA3
1	MAGEA4	1.212E-172	-10.27	7.049E-168	2.795E-171	P43358	MAGEA4
1	MAGEA5	0.00045385	-4.93	1	0.00181948	P43359	MAGEA5
1	MAGEA6	2.588E-211	-7.15	1.505E-206	6.703E-210	P43360	MAGEA6
1	MAGEB6P1	4.8669E-13	99.00	2.8313E-08	3.0849E-12		
1	MAGEC1	0	-9.47	0	0	O60732	MAGEC1
1	MAGEC3	9.0356E-06	46.91	0.52563642	4.0944E-05		
1	MAGED1	1.7783E-81	4.71	1.0345E-76	2.6697E-80	Q9Y5V3	MAGED1
1	MAGED2	5.0177E-52	1.87	2.919E-47	5.9864E-51	Q9UNF1	MAGED2
1	MAGEE1	6.7511E-13	1.93	3.9274E-08	4.2601E-12	Q9HCI5	MAGEE1
1	MAGEH1	2.3299E-12	1.98	1.3554E-07	1.4445E-11	Q9H213	MAGEH1
1	MAGI1	2.0454E-59	5.68	1.1899E-54	2.5952E-58	Q96QZ7	MAGI1
1	MAGI2	1.0977E-28	-3.60	6.3858E-24	9.8867E-28	Q86UL8	MAGI2
1	MAGI2-AS3	3.5088E-28	-3.32	2.0412E-23	3.1331E-27		

1	MAGIX	1.8022E-19	-3.52	1.0484E-14	1.3598E-18		
1	MAGOHB	4.3197E-65	2.31	2.5129E-60	5.7255E-64	Q96A72	MAGOHB
1	MAIP1	9.8323E-41	1.99	5.7198E-36	1.0379E-39	Q8WWC4	MAIP1
1	MAK	1.91E-09	-3.74	0.00011111	1.0507E-08	P20794	MAK
1	MAL2_1	0	-27.69	0	0		
1	MAL2_2	3.7079E-06	-58.21	0.21570331	1.7179E-05		
1	MALAT1_1	0	-2.62	0	0		
1	MALL	0	-1.96	0	0	Q13021	MALL
1	MALRD1	0.00124589	-4.42	1	0.00481298	Q5VYJ5	MALRD1
1	MALT1	9.0552E-58	4.15	5.2678E-53	1.1338E-56	Q9UDY8	MALT1
1	MAMDC2-AS1	0.00626293	-2.12	1	0.02252069		
1	MAML1	2.2464E-61	1.59	1.3068E-56	2.8931E-60	Q92585	MAML1
1	MAML2	7.6203E-49	-2.89	4.433E-44	8.8219E-48	Q8IZL2	MAML2
1	MAML3	2.1128E-69	2.63	1.2291E-64	2.8818E-68	Q96JK9	MAML3
1	MAMSTR	4.5527E-07	1.98	0.02648491	2.2269E-06	Q6ZN01	MAMSTR
1	MAN2B2	0	-1.86	0	0	Q9Y2E5	MAN2B2
1	MAN2C1	0	-1.69	0	0	Q9NTJ4	MAN2C1
1	MANBA	0	-1.85	0	0	O00462	MANBA
1	MANEA-AS1	2.9162E-07	-2.60	0.01696461	1.4404E-06		
1	MANEAL	1.9679E-91	2.28	1.1448E-86	3.1906E-90	Q5VSG8	MANEAL
1	MANSC1	9.7469E-14	1.74	5.6702E-09	6.3269E-13	Q9H8J5	MANSC1
1	MAOA	1.5896E-98	-8.15	9.2471E-94	2.7094E-97	P21397	MAOA
1	MAOB	1.25E-215	-6.28	7.27E-211	3.272E-214	P27338	MAOB
1	MAP10	3.4191E-40	-9.11	1.9891E-35	3.5865E-39	Q9P2G4	MAP10
1	MAP1B	7.455E-277	62.63	4.337E-272	2.075E-275	P46821	MAP1B
1	MAP1LC3A	4.9216E-41	-6.70	2.8631E-36	5.2141E-40	Q9H492	MAP1LC3A
1	MAP1S	2.4316E-49	1.93	1.4145E-44	2.8296E-48	Q66K74	MAP1S
1	MAP2	3.657E-256	-19.45	2.127E-251	1.004E-254	P11137	MAP2
1	MAP2K1	0	-1.86	0	0	Q02750	MAP2K1
1	MAP2K4	2.8619E-31	1.55	1.6649E-26	2.6823E-30	P45985	MAP2K4
1	MAP2K6	9.6529E-30	3.31	5.6155E-25	8.8446E-29	P52564	MAP2K6
1	MAP3K12	7.4952E-73	2.67	4.3603E-68	1.0502E-71	Q12852	MAP3K12
1	MAP3K14-AS1	5.445E-60	-3.83	3.1676E-55	6.9403E-59		
1	MAP3K15	6.887E-25	2.96	4.0064E-20	5.7696E-24	Q6ZN16	MAP3K15
1	MAP3K20	1.7143E-48	1.77	9.9725E-44	1.9787E-47	Q9NYL2	MAP3K20
1	MAP3K21	0	-1.52	0	0	Q5TCX8	MAP3K21
1	MAP3K3	6.7755E-48	1.64	3.9416E-43	7.7789E-47	Q99759	MAP3K3
1	MAP3K5	3.1891E-23	1.58	1.8552E-18	2.5864E-22	Q99683	MAP3K5
1	MAP3K7CL	1.5252E-24	-2.27	8.8726E-20	1.2704E-23	P57077	MAP3K7CL
1	MAP3K8	2.088E-139	-11.75	1.214E-134	4.37E-138	P41279	MAP3K8
1	MAP4K4	1.943E-43	2.78	1.1303E-38	2.1307E-42	O95819	MAP4K4
1	MAP4K5	4.891E-156	2.07	2.846E-151	1.074E-154	Q9Y4K4	MAP4K5
1	MAP7	0	-6.35	0	0	Q14244	MAP7
1	MAP7D3	1.9146E-84	2.15	1.1138E-79	2.9497E-83	Q8IWC1	MAP7D3
1	MAPK10	2.515E-211	-12.01	1.463E-206	6.518E-210	P53779	MAPK10
1	MAPK11	1.282E-12	1.52	7.458E-08	8.0073E-12	Q15759	MAPK11
1	MAPK12	2.8493E-41	1.58	1.6575E-36	3.0308E-40	P53778	MAPK12

1	MAPK13	3.3333E-54	-6.74	1.9391E-49	4.067E-53	O15264	MAPK13
1	MAPK15	6.8322E-43	-11.82	3.9746E-38	7.4333E-42	Q8TD08	MAPK15
1	MAPK1IP1L	9.0706E-53	2.60	5.2767E-48	1.0909E-51	Q8NDC0	MAPK1IP1L
1	MAPK3	6.7977E-31	1.56	3.9545E-26	6.3373E-30	P27361	MAPK3
1	MAPK7	1.312E-88	2.09	7.6324E-84	2.0848E-87	Q13164	MAPK7
1	MAPK8	1.5636E-57	1.90	9.0962E-53	1.9549E-56	P45983	MAPK8
1	MAPK8IP1	8.6826E-74	23.47	5.051E-69	1.2245E-72	Q9UQF2	MAPK8IP1
1	MAPK8IP2	0.00366475	1.76	1	0.01351548	Q13387	MAPK8IP2
1	MAPK9	3.2981E-21	2.39	1.9186E-16	2.5736E-20	P45984	MAPK9
1	MAPKAPK2	0	-1.77	0	0	P49137	MAPKAPK2
1	MAPKAPK3	2.9379E-51	1.75	1.7091E-46	3.4731E-50	Q16644	MAPKAPK3
1	MAPRE1	1.3539E-42	1.68	7.876E-38	1.4672E-41	Q15691	MAPRE1
1	MAPRE3	1.8796E-22	2.18	1.0934E-17	1.5014E-21	Q9UPY8	MAPRE3
1	MAPT	2.5544E-28	2.03	1.486E-23	2.2844E-27	P10636	MAPT
1	MARC1	9.203E-217	-7.41	5.353E-212	2.417E-215	Q5VT66	MARC1
1	MARC2	0	-2.21	0	0	Q969Z3	MARC2
1	MARCH1	2.1021E-40	-2.85	1.2229E-35	2.2094E-39	Q8TCQ1	MARCH1
1	MARCH2	9.8047E-92	2.60	5.7038E-87	1.5932E-90	Q9P0N8	MARCH2
1	MARCH3	5.24E-72	3.29	3.0483E-67	7.2909E-71	Q86UD3	MARCH3
1	MARCH4	3.227E-146	92.02	1.877E-141	6.883E-145	Q9P2E8	MARCH4
1	MARCH6	0	-2.14	0	0	O60337	MARCH6
1	MARCH8	6.3921E-55	5.00	3.7185E-50	7.8433E-54	Q5T0T0	MARCH8
1	MARCH9	7.3013E-35	1.65	4.2475E-30	7.1967E-34	Q86YJ5	MARCH9
1	MARCKSL1	5.9301E-68	4.92	3.4498E-63	8.0079E-67	P49006	MARCKSL1
1	MARK1	1.958E-170	-4.42	1.139E-165	4.485E-169	Q9P0L2	MARK1
1	MARK2	0	-3.45	0	0	Q7KZI7	MARK2
1	MARK2P9	1.2463E-14	13.32	7.25E-10	8.3142E-14		
1	MARK3	1.578E-141	1.68	9.18E-137	3.323E-140	P27448	MARK3
1	MARS2	6.6892E-19	1.66	3.8914E-14	4.9915E-18	Q96GW9	MARS2
1	MARVELD2	1.017E-45	-5.11	5.9164E-41	1.1419E-44	Q8N4S9	MARVELD2
1	MARVELD3	9.8533E-73	-9.72	5.7321E-68	1.3779E-71	Q96A59	MARVELD3
1	MASP1	1.6089E-05	-13.71	0.93597457	7.1684E-05	P48740	MASP1
1	MASP2	0.00012926	1.60	1	0.00054088	O00187	MASP2
1	MAST3	2.057E-204	4.46	1.197E-199	5.243E-203	O60307	MAST3
1	MAST4	0	-2.37	0	0	O15021	MAST4
1	MAST4-AS1	2.8208E-10	-3.47	1.641E-05	1.6091E-09		
1	MAT2B	3.9382E-23	1.54	2.291E-18	3.1882E-22	Q9NZL9	MAT2B
1	MATN1	0.00012719	6.25	1	0.00053268	P21941	MATN1
1	MATN1-AS1	1.0997E-07	-2.27	0.00639725	5.5561E-07		
1	MATN2	3.82E-125	-7.12	2.222E-120	7.558E-124	O00339	MATN2
1	MATN3	2.6209E-43	3.93	1.5247E-38	2.8686E-42	O15232	MATN3
1	MAX	2.7074E-68	1.86	1.575E-63	3.6628E-67	P61244	MAX
1	MAZ	7.8806E-80	1.78	4.5845E-75	1.168E-78	P56270	MAZ
1	MB	1.436E-124	-17.66	8.353E-120	2.835E-123	P02144	MB
1	MB21D2	3.2522E-39	1.84	1.892E-34	3.3737E-38	Q8IYB1	MB21D2
1	MBD2	1.1675E-33	1.55	6.7916E-29	1.1336E-32	Q9UBB5	MBD2
1	MBD3	1.8462E-90	1.95	1.074E-85	2.9727E-89	O95983	MBD3

1	MBD4	0	-1.59	0	0	O95243	MBD4
1	MBD6	0	-1.75	0	0	Q96DN6	MBD6
1	MBIP	1.6076E-45	1.92	9.3522E-41	1.8013E-44	Q9NS73	MBIP
1	MBNL1	1.8539E-43	1.69	1.0785E-38	2.0337E-42	Q9NR56	MBNL1
1	MBNL1-AS1	0.00547793	1.60	1	0.01983523		
1	MBNL2	1.2554E-72	2.52	7.303E-68	1.7538E-71	Q5VZF2	MBNL2
1	MBNL3	7.09E-210	-8.53	4.125E-205	1.832E-208	Q9NUK0	MBNL3
1	MBP	0	-2.85	0	0	P02686	MBP
1	MBTPS1	0	-1.93	0	0	Q14703	MBTPS1
1	MBTPS2	0	-2.03	0	0	O43462	MBTPS2
1	MCAM	4E-271	-9.79	2.327E-266	1.111E-269	P43121	MCAM
1	MCAT	1.4818E-43	1.88	8.6203E-39	1.6274E-42	Q8IVS2	MCAT
1	MCCC1	0	-2.18	0	0	Q96RQ3	MCCC1
1	MCF2	3.9185E-13	8.15	2.2796E-08	2.491E-12	P10911	MCF2
1	MCF2L	7.1977E-21	-6.50	4.1872E-16	5.58E-20	O15068	MCF2L
1	MCF2L2	1.5167E-19	-2.99	8.8234E-15	1.1463E-18	Q86YR7	MCF2L2
1	MCFD2	1.4332E-15	2.29	8.3377E-11	9.8461E-15	Q8NI22	MCFD2
1	MCHR2-AS1	1.6106E-07	-7.47	0.00936932	8.0659E-07		
1	MCIDAS	5.032E-87	4.56	2.9273E-82	7.8903E-86	D6RGH6	MCIDAS
1	MCM10	1.6712E-28	1.63	9.7222E-24	1.4987E-27	Q7L590	MCM10
1	MCM3AP-AS1	0	-2.32	0	0		
1	MCM5	3.5371E-60	1.73	2.0577E-55	4.5135E-59	P33992	MCM5
1	MCM8	3.3724E-53	1.71	1.9619E-48	4.0719E-52	Q9UJA3	MCM8
1	MCM8-AS1	1.7804E-10	1.65	1.0357E-05	1.0249E-09		
1	MCMDC2	3.587E-131	-12.57	2.087E-126	7.253E-130	Q4G0Z9	MCMDC2
1	MCOLN2	4.314E-114	-6.50	2.509E-109	8.079E-113	Q8IZK6	MCOLN2
1	MCOLN3	3.3119E-93	-5.38	1.9267E-88	5.441E-92	Q8TDD5	MCOLN3
1	MCPH1-AS1	1.2255E-29	-2.85	7.1289E-25	1.1198E-28		
1	MCRIP1	8.5083E-84	2.59	4.9496E-79	1.3022E-82	C9JLW8	MCRIP1
1	MCRIP2	1.2803E-63	1.83	7.4478E-59	1.6782E-62	Q9BUT9	MCRIP2
1	MCTP1	2.8917E-69	-2.77	1.6822E-64	3.9369E-68	Q6DN14	MCTP1
1	MCTP2	2.53E-252	-14.29	1.472E-247	6.933E-251	Q6DN12	MCTP2
1	MCTS1	7.2137E-32	1.77	4.1965E-27	6.8191E-31	Q9ULC4	MCTS1
1	MCU	6.086E-128	2.06	3.54E-123	1.22E-126	Q8NE86	MCU
1	MCUB	5.906E-38	2.03	3.4358E-33	6.0319E-37	Q9NWR8	MCUB
1	MDFI	0	-5.78	0	0	Q99750	MDFI
1	MDFIC	1.893E-196	-10.95	1.101E-191	4.722E-195	Q9P1T7	MDFIC
1	MDGA2	1.158E-102	-10.03	6.7385E-98	2.027E-101	Q7Z553	MDGA2
1	MDK	0	-1.87	0	0	P21741	MDK
1	MDM2	5.7073E-53	1.77	3.3202E-48	6.8741E-52	Q00987	MDM2
1	MDM4	0	-1.72	0	0	O15151	MDM4
1	MDP1	9.8647E-10	1.91	5.7387E-05	5.4974E-09	Q86V88	MDP1
1	ME1	0	-3.91	0	0	P48163	ME1
1	ME3	2.8225E-38	-4.56	1.6419E-33	2.8943E-37	Q16798	ME3
1	MEAF6	3.5609E-31	1.56	2.0715E-26	3.3342E-30	Q9HAF1	MEAF6
1	MECOM	0	-1.91	0	0	Q03112	MECOM
1	MED12L	1.157E-33	-2.84	6.7307E-29	1.1238E-32	Q86YW9	MED12L

1	MED14	0	-2.04	0	0	O60244	MED14
1	MED28	0	-1.58	0	0	Q9H204	MED28
1	MED30	0	-2.44	0	0	Q96HR3	MED30
1	MED6	1.8075E-79	1.83	1.0515E-74	2.6695E-78	O75586	MED6
1	MED8	4.8334E-42	2.02	2.8118E-37	5.2022E-41	Q96G25	MED8
1	MEF2A	0	-1.87	0	0	Q02078	MEF2A
1	MEF2C	7.905E-136	-12.87	4.599E-131	1.63E-134	Q06413	MEF2C
1	MEF2C-AS1	2.2962E-34	-4.06	1.3358E-29	2.2495E-33		
1	MEF2D	0	-1.79	0	0	Q14814	MEF2D
1	MEGF10	1.5265E-08	-14.69	0.00088805	8.0732E-08	Q96KG7	MEGF10
1	MEGF11	5.7987E-05	-1.82	1	0.00024897	A6BM72	MEGF11
1	MEGF6	2.121E-28	-4.64	1.2338E-23	1.8988E-27	O75095	MEGF6
1	MEGF9	2.0643E-26	3.38	1.2009E-21	1.7777E-25	Q9H1U4	MEGF9
1	MEI1	3.4737E-11	-4.98	2.0208E-06	2.056E-10	Q5TIA1	MEI1
1	MEI4	1.6457E-07	-9.32	0.00957356	8.2382E-07	A8MW99	MEI4
1	MEIS1	1.3495E-19	1.73	7.8506E-15	1.0205E-18	O00470	MEIS1
1	MEIS1-AS3	1.0102E-10	4.98	5.8765E-06	5.8683E-10		
1	MEIS2	1.378E-120	2.27	8.014E-116	2.673E-119	O14770	MEIS2
1	MEIS3	5.8966E-56	13.72	3.4303E-51	7.297E-55	Q99687	MEIS3
1	MEIS3P1	1.7734E-23	-5.43	1.0316E-18	1.4447E-22	A6NDR6	MEIS3P1
1	MELTF	7.786E-232	3.47	4.529E-227	2.089E-230	P08582	MELTF
1	MELTF-AS1	3.6295E-12	-1.96	2.1114E-07	2.2324E-11		
1	MEOX1	1.0965E-05	-42.12	0.63788093	4.9395E-05	P50221	MEOX1
1	MEOX2	0.01262752	-5.26	1	0.04376588	P50222	MEOX2
1	MEP1A	1.1913E-05	-7.65	0.69303541	5.3529E-05	Q16819	MEP1A
1	MEPE	9.2911E-06	-6.95	0.54050092	4.2062E-05	Q9NQ76	MEPE
1	MESP1	1.9541E-29	-7.18	1.1368E-24	1.7801E-28	Q9BRJ9	MESP1
1	MESP2	4.5879E-34	-14.27	2.669E-29	4.4827E-33	Q0VG99	MESP2
1	MEST	0	-7.82	0	0	Q5EB52	MEST
1	MESTIT1	0.00800968	-7.62	1	0.02845181		
1	METAP2	2.4545E-33	1.66	1.4279E-28	2.3707E-32	P50579	METAP2
1	METRN	6.305E-262	9.63	3.668E-257	1.742E-260	Q9UJH8	METRN
1	METTL10	0	-1.77	0	0		
1	METTL13	9.109E-103	1.71	5.299E-98	1.597E-101	Q8N6R0	METTL13
1	METTL15P1	0.00104295	-3.25	1	0.00405673	P0C7V9	METTL15P1
1	METTL21EP	0.00263019	-1.78	1	0.00983536	A6NDL7	METTL21EP
1	METTL25	1.2988E-18	2.24	7.5558E-14	9.6301E-18	Q8N6Q8	METTL25
1	METTL26	8.4596E-59	1.90	4.9213E-54	1.0691E-57		
1	METTL3	6.674E-112	1.82	3.882E-107	1.238E-110	Q86U44	METTL3
1	METTL7A	1.229E-266	-19.62	7.152E-262	3.41E-265	Q9H8H3	METTL7A
1	METTL7B	6.379E-102	-19.28	3.7109E-97	1.112E-100	Q6UX53	METTL7B
1	METTL9	3.357E-65	1.81	1.9529E-60	4.4526E-64	Q9H1A3	METTL9
1	MEX3B	3.4965E-35	3.63	2.034E-30	3.454E-34	Q6ZN04	MEX3B
1	MEX3C	6.14E-102	2.49	3.5718E-97	1.071E-100	Q5U5Q3	MEX3C
1	MEX3D	8.7608E-82	2.83	5.0965E-77	1.3186E-80	Q86XN8	MEX3D
1	MFAP3L	2.421E-47	5.16	1.4084E-42	2.7605E-46	O75121	MFAP3L
1	MFN1	0	-1.64	0	0	Q8IWA4	MFN1

1	MFNG	1.444E-101	-11.49	8.4018E-97	2.51E-100	O00587	MFNG
1	MFSD12	1.9378E-36	1.62	1.1273E-31	1.9456E-35	Q6NUT3	MFSD12
1	MFSD14A	0	-1.95	0	0	Q96MC6	MFSD14A
1	MFSD14B	0	-1.70	0	0	Q5SR56	MFSD14B
1	MFSD14C	2.2382E-13	-1.61	1.3021E-08	1.4345E-12	Q5VZR4	MFSD14C
1	MFSD3	0	-2.12	0	0	Q96ES6	MFSD3
1	MFSD4A	0.00161443	-1.50	1	0.00616623	Q8N468	MFSD4A
1	MFSD6	0	-2.52	0	0	Q6ZSS7	MFSD6
1	MFSD7	4.9857E-25	-4.78	2.9004E-20	4.1864E-24	Q6UXD7	MFSD7
1	MFSD8	0	-1.59	0	0	Q8NHS3	MFSD8
1	MGAT3	0.01206465	-3.53	1	0.04192397	Q09327	MGAT3
1	MGAT4A	0.00560094	-1.79	1	0.02027181	Q9UM21	MGAT4A
1	MGAT4B	5.253E-56	2.07	3.0559E-51	6.5033E-55	Q9UQ53	MGAT4B
1	MGAT5B	1.017E-140	10.33	5.915E-136	2.132E-139	Q3V5L5	MGAT5B
1	MGC27382	0.00472423	-17.85	1	0.01723598		
1	MGC32805	0.00134406	-5.49	1	0.00517777		
1	MGLL	0	35.71	0	0	Q99685	MGLL
1	MGMT	5.17E-210	93.98	3.008E-205	1.336E-208	P16455	MGMT
1	MGRN1	6.6617E-50	1.82	3.8754E-45	7.7944E-49	O60291	MGRN1
1	MGST2	0	-2.30	0	0	Q99735	MGST2
1	MIA3	0	-2.04	0	0	Q5JRA6	MIA3
1	MIAT	0.00026302	1.74	1	0.00107427		
1	MICAL1	3.2062E-42	1.79	1.8652E-37	3.4579E-41	Q8TDZ2	MICAL1
1	MICAL2	4.8474E-48	3.68	2.82E-43	5.5774E-47	O94851	MICAL2
1	MICAL3	4.8546E-15	1.86	2.8241E-10	3.2785E-14	Q7RTP6	MICAL3
1	MICALCL	1.5498E-10	-1.64	9.0159E-06	8.9399E-10	Q6ZW33	MICALCL
1	MICALL1	0	-2.55	0	0	Q8N3F8	MICALL1
1	MICALL2	0	-7.63	0	0	Q8IY33	MICALL2
1	MICB	3.7291E-58	1.82	2.1694E-53	4.6895E-57	Q29980	MICB
1	MICE	1.0775E-08	17.67	0.00062684	5.7314E-08		
1	MID1	2.7698E-32	1.94	1.6113E-27	2.635E-31	O15344	MID1
1	MID1IP1	0	-1.88	0	0	Q9NPA3	MID1IP1
1	MID2	3.5858E-24	1.88	2.086E-19	2.9597E-23	Q9UJV3	MID2
1	MIEF1	1.3881E-85	1.80	8.075E-81	2.1528E-84	Q9NQG6	MIEF1
1	MIER2	4.2121E-48	1.89	2.4503E-43	4.8492E-47	Q8N344	MIER2
1	MIER3	1.844E-112	2.84	1.073E-107	3.431E-111	Q7Z3K6	MIER3
1	MIF	8.2435E-24	1.83	4.7956E-19	6.7629E-23	P14174	MIF
1	MIF-AS1	7.6718E-16	-3.12	4.463E-11	5.3226E-15		
1	MIIP	1.4283E-53	1.80	8.3088E-49	1.7321E-52	Q5JXC2	MIIP
1	MILR1	1.2924E-30	-4.84	7.5186E-26	1.1999E-29	Q7Z6M3	MILR1
1	MINCR	6.5576E-22	-1.73	3.8148E-17	5.1838E-21		
1	MINDY1	4.0769E-90	-2.57	2.3717E-85	6.5516E-89	Q8N5J2	MINDY1
1	MINDY2	0	-1.94	0	0	Q8NBR6	MINDY2
1	MINDY4	2.3455E-08	-1.50	0.00136448	1.2284E-07	Q4G0A6	MINDY4
1	MIOX	1.5699E-07	-5.61	0.00913253	7.8688E-07	Q9UGB7	MIOX
1	MIPEP	0	-1.51	0	0	Q99797	MIPEP
1	MIPEPP3	0.00014535	-1.57	1	0.00060611		

1	MIPOL1	6.6703E-21	1.68	3.8804E-16	5.1725E-20	Q8TD10	MIPOL1
1	MIR100HG	1.6411E-66	4.11	9.5471E-62	2.1962E-65		
1	MIR137HG	0	109.68	0	0		
1	MIR155HG	2.3966E-28	4.33	1.3942E-23	2.1439E-27		
1	MIR17HG	9.887E-09	-1.65	0.00057516	5.27E-08	Q75NE6	MIR17HG
1	MIR2052HG	1.8013E-10	-2.07	1.0479E-05	1.0365E-09		
1	MIR205HG	0	-19.41	0	0		
1	MIR210HG	0	-2.13	0	0		
1	MIR2117HG	0.00017145	-3.78	1	0.00071129		
1	MIR222HG	0	-1.85	0	0		
1	MIR22HG	1.7822E-21	1.61	1.0368E-16	1.3986E-20		
1	MIR31HG	0	-2.54	0	0		
1	MIR34AHG	3.248E-99	-24.77	1.8897E-94	5.5596E-98		
1	MIR3681HG	5.4454E-36	-2.82	3.1678E-31	5.4336E-35		
1	MIR4300HG	6.0237E-16	1.72	3.5042E-11	4.1982E-15		
1	MIR4435-2HG	3.7266E-29	2.00	2.1679E-24	3.3789E-28		
1	MIR4500HG	9.1387E-41	-16.22	5.3164E-36	9.6521E-40		
1	MIR503HG	3.9425E-25	2.33	2.2935E-20	3.3167E-24		
1	MIR548XHG	6.663E-199	-18.09	3.876E-194	1.673E-197		
1	MIR5689HG	4.4413E-06	-4.16	0.25836728	2.0473E-05		
1	MIR646HG	5.152E-152	49.57	2.997E-147	1.115E-150		
1	MIR663AHG	0.00210899	-4.92	1	0.00796214		
1	MIR762HG	0.0006983	-1.63	1	0.00275503		
1	MIR9-3HG	2.5028E-24	-37.99	1.456E-19	2.072E-23		
1	MIR99AHG	3.6586E-11	-3.38	2.1284E-06	2.1627E-10		
1	MIRLET7DHG	5.3812E-53	-2.14	3.1305E-48	6.484E-52		
1	MIS12	5.9051E-46	3.99	3.4353E-41	6.6446E-45	Q9H081	MIS12
1	MISP3	7.8765E-42	3.24	4.5821E-37	8.4525E-41		
1	MITF	1.3442E-28	3.67	7.8199E-24	1.2073E-27	O75030	MITF
1	MKKS	8.8604E-47	1.54	5.1545E-42	1.0054E-45	Q9NPJ1	MKKS
1	MKL1	2.6556E-65	1.79	1.5449E-60	3.5279E-64	Q969V6	MKL1
1	MKNK1	0	-1.54	0	0	Q9BUB5	MKNK1
1	MKNK1-AS1	1.4064E-07	-6.83	0.00818158	7.0726E-07		
1	MKNK2	0	-2.02	0	0	Q9HBH9	MKNK2
1	MKRN2	1.1911E-45	1.67	6.9294E-41	1.3367E-44	Q9H000	MKRN2
1	MKRN2OS	0.00010625	-1.58	1	0.00044783		
1	MKRN3	0	-1.81	0	0	Q13064	MKRN3
1	MLF1	5.148E-26	1.87	2.9948E-21	4.3964E-25	P58340	MLF1
1	MLH3	1.4219E-46	1.69	8.2716E-42	1.6083E-45	Q9UHC1	MLH3
1	MLLT1	4.3893E-61	1.69	2.5535E-56	5.6368E-60	Q03111	MLLT1
1	MLLT11	0	-6.80	0	0	Q13015	MLLT11
1	MLLT3	1.6805E-61	2.56	9.7759E-57	2.1657E-60	P42568	MLLT3
1	MLLT6	0	-1.80	0	0	P55198	MLLT6
1	MLPH	4.241E-101	36.70	2.4671E-96	7.349E-100	Q9BV36	MLPH
1	MLXIP	1.4628E-16	1.56	8.5098E-12	1.0387E-15	Q9HAP2	MLXIP
1	MLXIPL	8.4605E-75	-10.71	4.9218E-70	1.2022E-73	Q9NP71	MLXIPL
1	MME	0	-2.33	0	0	P08473	MME

1	MMP1	1.0083E-15	3.37	5.8658E-11	6.9649E-15	P03956	MMP1
1	MMP10	6.3643E-77	17.37	3.7024E-72	9.1961E-76	P09238	MMP10
1	MMP11	7.8879E-13	-2.22	4.5887E-08	4.965E-12	P24347	MMP11
1	MMP12	4.8214E-06	5.54	0.28047893	2.2188E-05	P39900	MMP12
1	MMP13	5.9642E-10	2.04	3.4696E-05	3.3562E-09	P45452	MMP13
1	MMP15	0	-6.71	0	0	P51511	MMP15
1	MMP16	8.362E-105	5.18	4.864E-100	1.486E-103	P51512	MMP16
1	MMP17	1.934E-163	-9.87	1.125E-158	4.338E-162	Q9ULZ9	MMP17
1	MMP2	9.9452E-06	-4.31	0.57855146	4.4931E-05	P08253	MMP2
1	MMP25	0.00057717	2.23	1	0.00229503	Q9NPA2	MMP25
1	MMP25-AS1	4.8125E-15	2.13	2.7996E-10	3.2516E-14		
1	MMP28	0	-28.27	0	0	Q9H239	MMP28
1	MMP7	3.9416E-18	-5.57	2.293E-13	2.8941E-17	P09237	MMP7
1	MMRN2	1.988E-102	-4.80	1.1567E-97	3.474E-101	Q9H8L6	MMRN2
1	MN1	2.647E-139	35.32	1.54E-134	5.533E-138	Q10571	MN1
1	MNAT1	1.5795E-17	1.64	9.1886E-13	1.1458E-16	P51948	MNAT1
1	MNX1	6.2194E-42	4.16	3.6181E-37	6.6828E-41	P50219	MNX1
1	MOAP1	2.8878E-90	2.50	1.6799E-85	4.6433E-89	Q96BY2	MOAP1
1	MOB2	1.2706E-43	2.00	7.3917E-39	1.3973E-42	Q70IA6	MOB2
1	MOB3A	3.2153E-89	2.10	1.8705E-84	5.1288E-88	Q96BX8	MOB3A
1	MOB3B	4.0197E-41	-4.76	2.3384E-36	4.2664E-40	Q86TA1	MOB3B
1	MOB3C	1.0086E-54	-2.15	5.8676E-50	1.2358E-53	Q70IA8	MOB3C
1	MOCS1	1.527E-172	107.61	8.885E-168	3.522E-171	Q9NZB8	MOCS1
1	MOCS2	1.0877E-75	2.26	6.3274E-71	1.5562E-74	O96007	MOCS2
1	MOCS3	1.4652E-47	2.19	8.5237E-43	1.6759E-46	O95396	MOCS3
1	MOGAT3	0.00239001	-8.84	1	0.00897126	Q86VF5	MOGAT3
1	MOK	4.4564E-30	3.19	2.5925E-25	4.1007E-29	Q9UQ07	MOK
1	MON1A	2.4297E-15	1.60	1.4135E-10	1.6598E-14	Q86VX9	MON1A
1	MON2	0	-1.66	0	0	Q7Z3U7	MON2
1	MORC4	6.2573E-86	1.84	3.6401E-81	9.7408E-85	Q8TE76	MORC4
1	MORN1	5.9706E-59	2.08	3.4734E-54	7.5573E-58		
1	MORN2	2.4858E-14	1.97	1.4461E-09	1.6438E-13	Q502X0	MORN2
1	MORN3	6.0156E-05	-2.85	1	0.00025795	Q6PF18	MORN3
1	MORN5	9.2791E-08	-4.34	0.00539803	4.705E-07		
1	MOSPD1	5.3035E-83	3.11	3.0853E-78	8.064E-82	Q9UJG1	MOSPD1
1	MOSPD2	0	-1.88	0	0	Q8NHP6	MOSPD2
1	MOXD1	8.8888E-44	-11.29	5.171E-39	9.7843E-43	Q6UVY6	MOXD1
1	MPHOSPH10	9.4855E-27	1.59	5.5181E-22	8.2066E-26	O00566	MPHOSPH10
1	MPHOSPH8	0	-1.57	0	0	Q99549	MPHOSPH8
1	MPL	0.00104145	-2.02	1	0.00405143	P40238	MPL
1	MPLKIP	0	-1.66	0	0	Q8TAP9	MPLKIP
1	MPP1	3.4488E-93	4.93	2.0063E-88	5.6644E-92	Q00013	MPP1
1	MPP2	2.3551E-30	2.42	1.37E-25	2.1767E-29	Q14168	MPP2
1	MPP3	1.7265E-14	1.60	1.0043E-09	1.147E-13	Q13368	MPP3
1	MPP4	2.037E-191	84.61	1.185E-186	4.987E-190	Q96JB8	MPP4
1	MPP5	1.127E-44	1.74	6.5562E-40	1.2486E-43	Q8N3R9	MPP5
1	MPP7	2.2715E-38	-4.18	1.3214E-33	2.3318E-37	Q5T2T1	MPP7

1	MPPE1	0	-1.69	0	0	Q53F39	MPPE1
1	MPRIP	0	-2.05	0	0	Q6WCQ1	MPRIP
1	MPST	1.711E-149	2.57	9.955E-145	3.68E-148	P25325	MPST
1	MPV17	5.9095E-58	1.69	3.4378E-53	7.417E-57	P39210	MPV17
1	MPV17L2	1.4269E-49	1.95	8.3011E-45	1.6629E-48	Q567V2	MPV17L2
1	MPZ	8.3588E-11	-6.22	4.8626E-06	4.8734E-10	P25189	MPZ
1	MPZL1	0	-2.09	0	0	O95297	MPZL1
1	MPZL2	0	-13.50	0	0	O60487	MPZL2
1	MPZL3	6.3571E-94	-3.67	3.6982E-89	1.0503E-92	Q6UWV2	MPZL3
1	MR1	0	-12.43	0	0	Q95460	MR1
1	MRAP	0.00271841	2.09	1	0.01015091	Q8TCY5	MRAP
1	MRAP2	4.2543E-17	5.75	2.4749E-12	3.0551E-16	Q96G30	MRAP2
1	MRAS	5.2711E-27	-3.08	3.0664E-22	4.5884E-26	O14807	MRAS
1	MRGBP	1.402E-118	2.64	8.154E-114	2.695E-117	Q9NV56	MRGBP
1	MRGPRD	0.00011144	-8.17	1	0.00046896	Q8TDS7	MRGPRD
1	MRGPRF	8.2849E-14	-6.89	4.8197E-09	5.3893E-13	Q96AM1	MRGPRF
1	MRI1	6.4618E-95	1.79	3.7591E-90	1.0743E-93	Q9BV20	MRI1
1	MRLN	0.00423686	2.64	1	0.01553873	P0DMT0	MRLN
1	MROH1	0	-3.24	0	0		
1	MROH3P	2.5051E-09	-6.43	0.00014573	1.3722E-08		
1	MROH5	0.00540167	-2.02	1	0.01957617		
1	MROH6	0	-8.29	0	0		
1	MROH7	3.6261E-08	-15.48	0.00210944	1.8796E-07	Q68CQ1	MROH7
1	MROH7-TTC4	0.00023065	-9.01	1	0.00094744		
1	MRPL1	1.5057E-21	1.67	8.7591E-17	1.1832E-20	Q9BYD6	MRPL1
1	MRPL12	2.0215E-59	2.47	1.176E-54	2.5659E-58	P52815	MRPL12
1	MRPL15	1.0645E-75	2.68	6.1923E-71	1.5233E-74	Q9P015	MRPL15
1	MRPL17	5.5682E-83	2.66	3.2392E-78	8.4642E-82	Q9NRX2	MRPL17
1	MRPL18	5.954E-46	1.69	3.4637E-41	6.6983E-45	Q9H0U6	MRPL18
1	MRPL19	4.6409E-36	1.60	2.6998E-31	4.6372E-35	P49406	MRPL19
1	MRPL20	3.985E-137	2.87	2.318E-132	8.265E-136	Q9BYC9	MRPL20
1	MRPL22	1.617E-23	1.71	9.4065E-19	1.3189E-22	Q9NWU5	MRPL22
1	MRPL23	2.6218E-23	2.71	1.5252E-18	2.1299E-22	Q16540	MRPL23
1	MRPL23-AS1	4.9299E-28	-3.22	2.8679E-23	4.3946E-27		
1	MRPL27	2.3545E-22	1.62	1.3697E-17	1.8768E-21	Q9P0M9	MRPL27
1	MRPL28	3.8509E-31	1.84	2.2402E-26	3.6028E-30	Q13084	MRPL28
1	MRPL30	1.7252E-24	1.61	1.0036E-19	1.435E-23	Q8TCC3	MRPL30
1	MRPL34	1.0332E-40	1.99	6.0107E-36	1.0903E-39	Q9BQ48	MRPL34
1	MRPL38	3.355E-27	1.54	1.9517E-22	2.9345E-26	Q96DV4	MRPL38
1	MRPL4	1.5399E-23	1.55	8.9581E-19	1.2567E-22	Q9BYD3	MRPL4
1	MRPL40	4.4334E-29	2.05	2.5791E-24	4.016E-28	Q9NQ50	MRPL40
1	MRPL41	7.014E-20	1.86	4.0803E-15	5.3379E-19	Q8IXM3	MRPL41
1	MRPL44	1.9227E-38	1.73	1.1185E-33	1.9751E-37	Q9H9J2	MRPL44
1	MRPL49	1.8413E-38	1.66	1.0711E-33	1.8928E-37	Q13405	MRPL49
1	MRPL51	6.387E-44	2.04	3.7156E-39	7.0384E-43	Q4U2R6	MRPL51
1	MRPL52	1.0531E-31	1.58	6.1263E-27	9.9276E-31	Q86TS9	MRPL52
1	MRPL53	6.8314E-18	1.58	3.9741E-13	4.9901E-17	Q96EL3	MRPL53

1	MRPL54	8.7948E-22	1.86	5.1163E-17	6.9402E-21	Q6P161	MRPL54
1	MRPS12	1.9756E-45	2.10	1.1493E-40	2.2106E-44	O15235	MRPS12
1	MRPS14	0	-1.84	0	0	O60783	MRPS14
1	MRPS15	5.4148E-20	1.53	3.15E-15	4.1296E-19	P82914	MRPS15
1	MRPS16	2.9367E-89	2.40	1.7084E-84	4.6857E-88	Q9Y3D3	MRPS16
1	MRPS17	3.2348E-11	1.56	1.8818E-06	1.9179E-10	Q9Y2R5	MRPS17
1	MRPS2	2.225E-136	2.27	1.294E-131	4.595E-135	Q9Y399	MRPS2
1	MRPS21	0	-2.30	0	0	P82921	MRPS21
1	MRPS23	6.1571E-29	1.97	3.5818E-24	5.567E-28	Q9Y3D9	MRPS23
1	MRPS26	2.8503E-33	1.77	1.6581E-28	2.7493E-32	Q9BYN8	MRPS26
1	MRPS27	7.8736E-43	1.63	4.5804E-38	8.5615E-42	Q92552	MRPS27
1	MRPS31P4	4.8008E-05	1.77	1	0.00020723		
1	MRPS33	3.1572E-12	1.50	1.8367E-07	1.9473E-11	Q9Y291	MRPS33
1	MRPS34	4.7369E-25	1.72	2.7556E-20	3.9792E-24	P82930	MRPS34
1	MRPS35	1.4455E-48	2.08	8.409E-44	1.6698E-47	P82673	MRPS35
1	MRPS36	2.8447E-14	1.50	1.6549E-09	1.8782E-13	P82909	MRPS36
1	MRTO4	1.039E-102	2.07	6.0443E-98	1.819E-101	Q9UKD2	MRTO4
1	MRVI1-AS1	0.00112229	-23.46	1	0.00435255		
1	MSANTD1	4.1542E-07	-3.03	0.0241665	2.0364E-06		
1	MSANTD3	3.3619E-34	1.51	1.9558E-29	3.2881E-33	Q96H12	MSANTD3
1	MSC	7.0163E-25	-5.68	4.0817E-20	5.8754E-24	O60682	MSC
1	MSC-AS1	3.7832E-72	-16.11	2.2009E-67	5.269E-71		
1	MSH3	2.0695E-42	1.91	1.2039E-37	2.2377E-41	P20585	MSH3
1	MSL3P1	5.822E-145	-8.06	3.387E-140	1.239E-143	P0C860	MSL3P1
1	MSLN	1.7514E-15	-14.19	1.0189E-10	1.2017E-14	Q13421	MSLN
1	MSMO1	0	-3.57	0	0	Q15800	MSMO1
1	MSN	5.1138E-70	1.64	2.9749E-65	7.0097E-69	P26038	MSN
1	MSR1	2.0236E-11	1.85	1.1772E-06	1.2103E-10	P21757	MSR1
1	MSRA	6.9237E-58	4.64	4.0278E-53	8.6824E-57	Q9UJ68	MSRA
1	MSRB1	4.4764E-67	2.49	2.6041E-62	6.0127E-66	Q9NZV6	MSRB1
1	MSRB2	3.414E-105	-3.63	1.986E-100	6.07E-104	Q9Y3D2	MSRB2
1	MSRB3	9.811E-103	2.35	5.7077E-98	1.719E-101	Q8IXL7	MSRB3
1	MST1P2	0.00143733	-1.52	1	0.00551296		
1	MST1R	0	-3.82	0	0	Q04912	MST1R
1	MSTN	1.126E-06	8.46	0.06550385	5.3913E-06	O14793	MSTN
1	MSX1	8.18E-156	77.20	4.759E-151	1.794E-154	P28360	MSX1
1	MSX2	5.5809E-85	38.69	3.2467E-80	8.637E-84	P35548	MSX2
1	MT-ATP6	0	-3.68	0	0	P00846	MT-ATP6
1	MT-ATP8	0	-3.71	0	0	P03928	MT-ATP8
1	MT-CO1	0	-2.15	0	0	P00395	MT-CO1
1	MT-CO2	0	-3.68	0	0	P00403	MT-CO2
1	MT-CO3	0	-2.51	0	0	P00414	MT-CO3
1	MT-CYB	0	-2.95	0	0	P00156	MT-CYB
1	MT-ND1	0	-1.72	0	0	P03886	MT-ND1
1	MT-ND2	0	-3.11	0	0	P03891	MT-ND2
1	MT-ND3	0	-3.43	0	0	P03897	MT-ND3
1	MT-ND4	0	-2.91	0	0	P03905	MT-ND4

1	MT-ND4L	0	-3.08	0	0	P03901	MT-ND4L
1	MT-ND5	0	-2.56	0	0	P03915	MT-ND5
1	MT-ND6	0	-3.13	0	0	P03923	MT-ND6
1	MT-RNR1	0	-2.11	0	0	A0A0C5B5G6	MT-RNR1
1	MT-RNR2	2.6179E-11	-1.52	1.5229E-06	1.5591E-10	Q8IVG9	MT-RNR2
1	MT-TY	0.0078449	-2.80	1	0.02791591		
1	MT1B	0.00058546	25.92	1	0.00232703	P07438	MT1B
1	MT1E	1.4767E-28	2.40	8.5908E-24	1.3253E-27	P04732	MT1E
1	MT1F	5.9767E-33	-5.84	3.4769E-28	5.746E-32	P04733	MT1F
1	MT1L	2.5354E-18	-7.25	1.475E-13	1.8682E-17	Q93083	MT1L
1	MT1X	0	-1.94	0	0	P80297	MT1X
1	MT2A	2.691E-112	3.00	1.566E-107	5.004E-111	P02795	MT2A
1	MTA1	1.0156E-78	1.72	5.9083E-74	1.4893E-77	Q13330	MTA1
1	MTA2	0	-1.84	0	0	O94776	MTA2
1	MTBP	0	-1.72	0	0	Q96DY7	MTBP
1	MTCL1	9.889E-136	8.96	5.753E-131	2.038E-134	Q9Y4B5	MTCL1
1	MTCO1P47	7.609E-06	-9.19	0.44264491	3.4633E-05		
1	MTCP1	1.14E-16	1.54	6.6316E-12	8.1131E-16	P56278	MTCP1
1	MTCYBP21	0.00964709	-17.76	1	0.03389768		
1	MTDH	0	-1.82	0	0	Q86UE4	MTDH
1	MTERF1	0	-1.51	0	0	Q99551	MTERF1
1	MTFP1	3.482E-41	2.20	2.0256E-36	3.6997E-40	Q9UDX5	MTFP1
1	MTG1	0	-1.61	0	0	Q9BT17	MTG1
1	MTG2	2.653E-21	2.53	1.5434E-16	2.0744E-20	Q9H4K7	MTG2
1	MTHFD1	1.408E-106	2.04	8.189E-102	2.521E-105	P11586	MTHFD1
1	MTHFR	0	-1.80	0	0	P42898	MTHFR
1	MTHFS	1.0145E-23	2.01	5.9018E-19	8.303E-23	P49914	MTHFS
1	MTHFSD	0	-1.70	0	0	Q2M296	MTHFSD
1	MTMR11	0	-2.72	0	0	A4FU01	MTMR11
1	MTMR4	5.942E-108	1.93	3.457E-103	1.073E-106	Q9NYA4	MTMR4
1	MTMR9LP	2.8931E-11	1.69	1.683E-06	1.7203E-10		
1	MTND5P30	0.00102528	-25.44	1	0.00399255		
1	MTRF1	0	-1.55	0	0	O75570	MTRF1
1	MTRF1L	4.2372E-61	1.74	2.4649E-56	5.4426E-60	Q9UGC7	MTRF1L
1	MTRNR2L12	3.9808E-06	-1.64	0.23158053	1.841E-05	P0DMP1	MTRNR2L12
1	MTSS1	1.375E-76	-3.95	7.999E-72	1.9814E-75	O43312	MTSS1
1	MTSS1L	0	-3.40	0	0	Q765P7	MTSS1L
1	MTTP	0.00111737	-3.48	1	0.00433462	P55157	MTTP
1	MTURN	0	-6.20	0	0	Q8N3F0	MTURN
1	MTUS1	0	-14.69	0	0	Q9ULD2	MTUS1
1	MTX1P1	7.8305E-33	-1.92	4.5553E-28	7.5133E-32		
1	MUC1	1.572E-200	-10.37	9.145E-196	3.976E-199	P15941	MUC1
1	MUC12	8.4991E-07	-2.92	0.04944271	4.0963E-06	Q9UKN1	MUC12
1	MUC16	1.368E-142	-27.56	7.958E-138	2.893E-141	Q8WXI7	MUC16
1	MUC19	0.00800412	-15.97	1	0.02843379	Q7Z5P9	MUC19
1	MUC20	1.736E-118	-8.25	1.01E-113	3.336E-117	Q8N307	MUC20
1	MUC20P1	8.0904E-18	-6.12	4.7065E-13	5.9038E-17		

1	MUC3A	1.6712E-10	-16.52	9.7221E-06	9.6316E-10	Q02505	MUC3A
1	MUC4	7.679E-171	-48.39	4.467E-166	1.76E-169	Q99102	MUC4
1	MUC5AC	0.00079536	-3.37	1	0.00312587	P98088	MUC5AC
1	MUC6	0.00029435	-3.45	1	0.0011972	Q6W4X9	MUC6
1	MUM1	5.137E-55	1.74	2.9884E-50	6.3073E-54	Q2TAK8	MUM1
1	MUM1L1	1.1061E-05	-3.78	0.64345004	4.9814E-05	Q5H9M0	MUM1L1
1	MUT	0	-1.57	0	0	P22033	MUT
1	MVB12A	1.9741E-28	1.58	1.1484E-23	1.7679E-27	Q96EY5	MVB12A
1	MVB12B	5.646E-194	34.67	3.285E-189	1.395E-192	Q9H7P6	MVB12B
1	MVD	0	-2.24	0	0	P53602	MVD
1	MVK	0	-1.53	0	0	Q03426	MVK
1	MX1	1.783E-116	-10.44	1.037E-111	3.388E-115	P20591	MX1
1	MX2	2.0779E-26	-29.39	1.2088E-21	1.7889E-25	P20592	MX2
1	MXD1	0	-2.08	0	0	Q05195	MXD1
1	MXD3	1.1102E-56	1.83	6.4585E-52	1.3797E-55	Q9BW11	MXD3
1	MXRA7	1.909E-116	2.46	1.111E-111	3.625E-115	P84157	MXRA7
1	MXRA8	2.7644E-11	-1.91	1.6082E-06	1.6452E-10	Q9BRK3	MXRA8
1	MYADM	0	134.51	0	0	Q96S97	MYADM
1	MYADML2	3.3602E-05	6.24	1	0.00014668	A6NDP7	MYADML2
1	MYB	2.2433E-14	-1.78	1.305E-09	1.4852E-13	P10242	MYB
1	MYBL2	1.729E-153	2.31	1.006E-148	3.763E-152	P10244	MYBL2
1	MYCBPAP	4.6033E-14	-6.79	2.6779E-09	3.0188E-13	Q8TBZ2	MYCBPAP
1	MYCL	9.6043E-25	-4.07	5.5872E-20	8.023E-24	P12524	MYCL
1	MYEF2	8.1358E-06	38.38	0.47329485	3.6979E-05	Q9P2K5	MYEF2
1	MYEOV	0	-3.15	0	0		
1	MYH11	0.00016884	1.58	1	0.00070079	P35749	MYH11
1	MYH14	1.7333E-54	-20.77	1.0083E-49	2.1197E-53	Q7Z406	MYH14
1	MYH16	0	-2.61	0	0	Q9H6N6	MYH16
1	MYH7B	2.2475E-21	-4.60	1.3074E-16	1.7599E-20	A7E2Y1	MYH7B
1	MYL6B	8.2283E-27	1.71	4.7867E-22	7.1263E-26	P14649	MYL6B
1	MYL9	1.0166E-63	3.57	5.9138E-59	1.3337E-62	P24844	MYL9
1	MYLIP	2.95E-118	-4.97	1.716E-113	5.664E-117	Q8WY64	MYLIP
1	MYLK	0	-5.82	0	0	Q15746	MYLK
1	MYLK-AS1	3.7657E-10	-1.62	2.1907E-05	2.137E-09		
1	MYLK-AS2	0.01045918	-15.95	1	0.03661625		
1	MYLK2	1.0308E-08	3.20	0.00059968	5.4896E-08	Q9H1R3	MYLK2
1	MYLK3	0.00534691	1.83	1	0.01938739	Q32MK0	MYLK3
1	MYLK4	2.0201E-16	-4.83	1.1752E-11	1.4311E-15	Q86YV6	MYLK4
1	MYNN	0	-1.69	0	0	Q9NPC7	MYNN
1	MYO15A	3.5501E-12	-5.78	2.0653E-07	2.1848E-11	Q9UKN7	MYO15A
1	MYO15B	8.2091E-10	-2.52	4.7756E-05	4.5884E-09	Q96JP2	MYO15B
1	MYO18A	0	-3.90	0	0	Q92614	MYO18A
1	MYO1D	0	-5.67	0	0	O94832	MYO1D
1	MYO1F	0.00062425	1.53	1	0.00247495	O00160	MYO1F
1	MYO1G	0.00103467	-4.19	1	0.0040267	B0I1T2	MYO1G
1	MYO5A	8.6032E-79	2.21	5.0048E-74	1.2629E-77	Q9Y4I1	MYO5A
1	MYO5B	1.6592E-61	2.55	9.6525E-57	2.1388E-60	Q9ULV0	MYO5B

1	MYO5C	5.8368E-08	-1.82	0.00339551	2.9916E-07	Q9NQX4	MYO5C
1	MYO7A	5.7251E-52	-7.24	3.3305E-47	6.8263E-51	Q13402	MYO7A
1	MYO7B	0.00321513	-2.71	1	0.01192915	Q6PIF6	MYO7B
1	MYOM1	1.6254E-08	-3.88	0.00094555	8.5834E-08	P52179	MYOM1
1	MYOM2	4.5114E-14	94.49	2.6245E-09	2.9595E-13	P54296	MYOM2
1	MYOM3	0.00136814	-3.44	1	0.00526635	Q5VTT5	MYOM3
1	MYOSLID	8.2208E-62	-6.00	4.7824E-57	1.062E-60		
1	MYOT	3.5341E-05	-8.83	1	0.00015407	Q9UBF9	MYOT
1	MYOZ3	0.00177933	-9.42	1	0.00676672	Q8TDC0	MYOZ3
1	MYRF	6.1844E-74	-3.71	3.5977E-69	8.7281E-73	Q9Y2G1	MYRF
1	MYZAP	0.00954664	-2.83	1	0.03355687	P0CAP1	MYZAP
1	MZF1	0	-1.94	0	0	P28698	MZF1
1	MZT2B	6.0726E-40	1.99	3.5327E-35	6.3434E-39	Q6NZ67	MZT2B
1	N4BP1	0	-1.52	0	0	O75113	N4BP1
1	N4BP2L2	0	-1.62	0	0	Q92802	N4BP2L2
1	N4BP3	0.0002419	-1.52	1	0.00099162	O15049	N4BP3
1	NAA10	4.7268E-84	1.89	2.7498E-79	7.2553E-83	P41227	NAA10
1	NAA11	8.368E-232	-10.01	4.868E-227	2.244E-230	Q9BSU3	NAA11
1	NAA30	1.7691E-32	1.91	1.0292E-27	1.6883E-31	Q147X3	NAA30
1	NAA38	1.368E-42	1.81	7.9583E-38	1.4823E-41	Q9BRA0	NAA38
1	NAA40	0	-2.38	0	0	Q86UY6	NAA40
1	NAAA	0	-4.53	0	0	Q02083	NAAA
1	NAALADL1	1.3969E-27	-13.19	8.1263E-23	1.2322E-26	Q9UQQ1	NAALADL1
1	NAALADL2	2.3597E-18	-2.40	1.3727E-13	1.7394E-17	Q58DX5	NAALADL2
1	NAB1	0	-1.91	0	0	Q13506	NAB1
1	NAB2	0	-2.46	0	0	Q15742	NAB2
1	NABP2	6.9625E-57	1.98	4.0503E-52	8.662E-56	Q9BQ15	NABP2
1	NACAD	4.3464E-31	-10.08	2.5284E-26	4.0637E-30	O15069	NACAD
1	NACAP1	3.0757E-05	-8.92	1	0.00013455	Q9BZK3	NACAP1
1	NACC2	2.0227E-72	3.34	1.1767E-67	2.8238E-71	Q96BF6	NACC2
1	NADK2	0	-2.02	0	0	Q4G0N4	NADK2
1	NADK2-AS1	0.00931389	-2.69	1	0.03282999		
1	NADSYN1	0	-2.88	0	0	Q6IA69	NADSYN1
1	NAGA	9.1058E-92	2.82	5.2972E-87	1.4805E-90	P17050	NAGA
1	NAGK	2.1222E-90	2.05	1.2346E-85	3.4151E-89	Q9UJ70	NAGK
1	NAGLU	0	-2.00	0	0	P54802	NAGLU
1	NAGPA	2.2027E-71	2.15	1.2814E-66	3.0503E-70	Q9UK23	NAGPA
1	NALCN	7.866E-113	20.50	4.576E-108	1.465E-111	Q8IZF0	NALCN
1	NAMPT	0	-1.83	0	0	P43490	NAMPT
1	NAMPTP1	2.0802E-32	-7.72	1.2102E-27	1.9826E-31		
1	NANOS1	9.9815E-06	-2.48	0.58066359	4.5083E-05	Q8WY41	NANOS1
1	NANS	3.2013E-23	1.60	1.8623E-18	2.5959E-22	Q9NR45	NANS
1	NAP1L1	6.1657E-85	1.99	3.5869E-80	9.5345E-84	P55209	NAP1L1
1	NAP1L2	2.646E-58	-7.46	1.5393E-53	3.3317E-57	Q9ULW6	NAP1L2
1	NAP1L3	5.381E-14	-7.27	3.1303E-09	3.5208E-13	Q99457	NAP1L3
1	NAP1L5	2.4791E-55	-8.45	1.4422E-50	3.0535E-54	Q96NT1	NAP1L5
1	NAPA-AS1	8.9217E-06	-1.73	0.51900996	4.0453E-05		

1	NAPEPLD	0	-2.35	0	0	Q6IQ20	NAPEPLD
1	NAPRT	0	-4.32	0	0	Q6XQN6	NAPRT
1	NAPSA	3.155E-06	-2.27	0.18353628	1.469E-05	O96009	NAPSA
1	NARF	1.204E-30	1.52	7.0044E-26	1.1182E-29	Q9UHQ1	NARF
1	NARS	2.5203E-45	3.47	1.4661E-40	2.8152E-44	O43776	NARS
1	NAT1	1.9153E-22	-2.15	1.1142E-17	1.5297E-21	P18440	NAT1
1	NAT8L	2.8309E-88	9.44	1.6469E-83	4.4874E-87	Q8N9F0	NAT8L
1	NAT9	5.7293E-40	1.57	3.333E-35	5.987E-39	Q9BTE0	NAT9
1	NATD1	1.6897E-17	2.01	9.8295E-13	1.2252E-16	Q8N6N6	NATD1
1	NAV1	8.449E-157	29.97	4.915E-152	1.859E-155	Q8NEY1	NAV1
1	NAV3	1.379E-138	3.99	8.019E-134	2.869E-137	Q8IVL0	NAV3
1	NBEA	6.9616E-70	3.09	4.0498E-65	9.5245E-69	Q8NFP9	NBEA
1	NBEAL2	0	-2.16	0	0	Q6ZNJ1	NBEAL2
1	NBEAP3	1.1521E-51	2.52	6.7023E-47	1.3678E-50		
1	NBPF1	3.16E-113	2.91	1.839E-108	5.9E-112	Q3BBV0	NBPF1
1	NBPF15	2.505E-67	2.61	1.4573E-62	3.3718E-66	Q8N660	NBPF15
1	NBPF19	8.8818E-16	-1.66	5.1669E-11	6.1437E-15	A0A087WUL8	NBPF19
1	NBPF20	7.154E-13	1.80	4.1618E-08	4.5099E-12	Q3BBV1	NBPF20
1	NBPF22P	0.00637367	-5.70	1	0.02288776		
1	NBPF25P	3.4658E-05	1.64	1	0.00015119		
1	NBPF6	0.00029695	-32.39	1	0.00120751	Q5VWK0	NBPF6
1	NBPF8	3.7599E-27	1.56	2.1873E-22	3.2837E-26	Q3BBV2	NBPF8
1	NBPF9	9.3501E-30	1.78	5.4393E-25	8.57E-29	Q3BBW0	NBPF9
1	NBR2	9.8807E-14	-4.24	5.748E-09	6.4123E-13	O15453	NBR2
1	NCALD	1.2394E-09	-7.05	7.2099E-05	6.8751E-09	P61601	NCALD
1	NCAM2	1.99E-104	-9.95	1.158E-99	3.527E-103	O15394	NCAM2
1	NCAPGP2	0.0028144	-19.79	1	0.01049719		
1	NCAPH2	3.0305E-78	1.78	1.763E-73	4.4229E-77	Q6IBW4	NCAPH2
1	NCBP2-AS2	0	-1.88	0	0		
1	NCCRP1	0.00813587	-15.97	1	0.02888065	Q6ZVX7	NCCRP1
1	NCF1	6.3338E-11	-8.61	3.6846E-06	3.7068E-10	P14598	NCF1
1	NCF1B	0.00245685	-10.53	1	0.00920965	A6NI72	NCF1B
1	NCF2	0.00189898	-2.45	1	0.00720341	P19878	NCF2
1	NCK1-AS1	1.1905E-22	-2.20	6.9257E-18	9.5605E-22		
1	NCK2	6.2478E-48	1.88	3.6346E-43	7.1759E-47	O43639	NCK2
1	NCKAP5	8.279E-125	6.03	4.816E-120	1.637E-123	O14513	NCKAP5
1	NCL	6.7851E-54	1.63	3.9472E-49	8.2559E-53	P19338	NCL
1	NCOA5	5.83E-44	2.38	3.3916E-39	6.4258E-43	Q9HCD5	NCOA5
1	NCOR1	2.0169E-58	1.74	1.1733E-53	2.5413E-57	O75376	NCOR1
1	NCOR2	1.271E-111	2.88	7.393E-107	2.354E-110	Q9Y618	NCOR2
1	NCR3LG1	7.229E-186	6.92	4.205E-181	1.732E-184	Q68D85	NCR3LG1
1	NCSTN	0	-2.59	0	0	Q92542	NCSTN
1	NDC80	1.1249E-29	1.65	6.544E-25	1.0294E-28	O14777	NDC80
1	NDFIP2	0	-2.97	0	0	Q9NV92	NDFIP2
1	NDNF	0.00040587	-5.83	1	0.0016331	Q8TB73	NDNF
1	NDOR1	4.2799E-18	1.67	2.4898E-13	3.1413E-17	Q9UHB4	NDOR1
1	NDRG1	0	-4.59	0	0	Q92597	NDRG1

1	NDRG2	5.8821E-05	-2.81	1	0.00025239	Q9UN36	NDRG2
1	NDRG4	3.347E-194	-6.21	1.947E-189	8.283E-193	Q9ULP0	NDRG4
1	NDST1	0	-2.77	0	0	P52848	NDST1
1	NDST1-AS1	1.1008E-27	-6.84	6.4036E-23	9.732E-27		
1	NDST2	3.3073E-46	1.66	1.924E-41	3.7309E-45	P52849	NDST2
1	NDST3	7.6015E-07	-2.36	0.04422116	3.6738E-06	O95803	NDST3
1	NDST4	8.1447E-08	-6.71	0.00473809	4.1417E-07	Q9H3R1	NDST4
1	NDUFA11	2.4415E-90	2.48	1.4203E-85	3.9269E-89	Q86Y39	NDUFA11
1	NDUFA13	1.2096E-12	1.53	7.0369E-08	7.5633E-12	Q9P0J0	NDUFA13
1	NDUFA4L2	8.311E-16	-2.23	4.8348E-11	5.7633E-15	Q9NRX3	NDUFA4L2
1	NDUFA6	2.097E-41	2.22	1.2199E-36	2.2359E-40	P56556	NDUFA6
1	NDUFA6-AS1	4.832E-06	-1.71	0.28109405	2.2233E-05		
1	NDUFA9	3.149E-18	1.54	1.8319E-13	2.3171E-17	Q16795	NDUFA9
1	NDUFAF2	1.9599E-21	1.73	1.1402E-16	1.5366E-20	Q8N183	NDUFAF2
1	NDUFAF6	0	-1.60	0	0	Q330K2	NDUFAF6
1	NDUFAF8	1.2747E-15	1.55	7.4155E-11	8.7736E-15	A1L188	NDUFAF8
1	NDUFB1	5.7524E-24	1.62	3.3464E-19	4.7285E-23	O75438	NDUFB1
1	NDUFB10	2.1143E-73	2.65	1.23E-68	2.9723E-72	O96000	NDUFB10
1	NDUFB2	3.3011E-30	1.57	1.9204E-25	3.0443E-29	O95178	NDUFB2
1	NDUFB2-AS1	0.00014314	2.17	1	0.00059712		
1	NDUFB5	0	-1.63	0	0	O43674	NDUFB5
1	NDUFB6	6.3688E-69	2.42	3.705E-64	8.6505E-68	O95139	NDUFB6
1	NDUFB9	0	-1.89	0	0	Q9Y6M9	NDUFB9
1	NDUFC2-KCTD	0.01406111	2.49	1	0.04853394	E9PQ53	NDUFC2-KCTD
1	NDUFS2	0	-1.83	0	0	O75306	NDUFS2
1	NDUFS4	5.8369E-31	2.04	3.3956E-26	5.4486E-30	O43181	NDUFS4
1	NDUFS5	5.7742E-19	1.74	3.3591E-14	4.3131E-18	O43920	NDUFS5
1	NDUFV1	0	-1.71	0	0	P49821	NDUFV1
1	NDUFV2P1	1.7307E-11	3.30	1.0068E-06	1.0386E-10		
1	NEAT1	0	-3.07	0	0		
1	NEB	2.6908E-61	-4.76	1.5654E-56	3.4632E-60	P20929	NEB
1	NEBL	1.2488E-77	-15.54	7.265E-73	1.8126E-76	O76041	NEBL
1	NEBL-AS1	6.0157E-05	-32.89	1	0.00025795		
1	NECAB1	2.1892E-23	-6.22	1.2736E-18	1.7812E-22	Q8N987	NECAB1
1	NECAB2	2.1615E-05	-7.37	1	9.5506E-05	Q7Z6G3	NECAB2
1	NECAP1	4.8274E-30	1.63	2.8083E-25	4.4379E-29	Q8NC96	NECAP1
1	NECTIN1	0	-11.01	0	0	Q15223	NECTIN1
1	NECTIN2	0	-1.98	0	0	Q92692	NECTIN2
1	NECTIN4	2.143E-277	-40.40	1.247E-272	5.971E-276	Q96NY8	NECTIN4
1	NEDD4	1.2667E-84	2.45	7.3691E-80	1.9541E-83	P46934	NEDD4
1	NEDD4L	9.4486E-68	3.68	5.4966E-63	1.2747E-66	Q96PU5	NEDD4L
1	NEDD8	5.0176E-23	1.75	2.9189E-18	4.0552E-22	Q15843	NEDD8
1	NEDD8-MDP1	0.00129788	3.32	1	0.00500714	E9PL57	NEDD8-MDP1
1	NEDD9	1.263E-173	8.94	7.347E-169	2.923E-172	Q14511	NEDD9
1	NEFH	5.7352E-06	5.01	0.33364121	2.6304E-05	P12036	NEFH
1	NEFL	0.00100571	23.84	1	0.00392	P07196	NEFL
1	NEGR1	1.604E-105	3.83	9.331E-101	2.855E-104	Q7Z3B1	NEGR1

1	NEIL1	2.413E-10	-1.58	1.4037E-05	1.3803E-09	Q96FI4	NEIL1
1	NEK1	0	-1.61	0	0	Q96PY6	NEK1
1	NEK10	6.1187E-62	8.94	3.5595E-57	7.9188E-61	Q6ZWH5	NEK10
1	NEK2	0	-2.25	0	0	P51955	NEK2
1	NEK3	0	-1.66	0	0	P51956	NEK3
1	NEK4	5.1113E-26	1.51	2.9735E-21	4.3657E-25	P51957	NEK4
1	NEK8	0	-1.63	0	0	Q86SG6	NEK8
1	NELL2	1.552E-184	-8.13	9.028E-180	3.704E-183	Q99435	NELL2
1	NEMF	8.2119E-51	1.63	4.7772E-46	9.6763E-50	O60524	NEMF
1	NEMP1	0	-1.75	0	0	O14524	NEMP1
1	NEMP2	0	-2.16	0	0	A6NFY4	NEMP2
1	NEO1	0	-2.17	0	0	Q92859	NEO1
1	NEPNP	0.00800166	-2.38	1	0.02843025		
1	NES	0.00018128	-1.97	1	0.00075075	P48681	NES
1	NET1	0	-2.91	0	0	Q7Z628	NET1
1	NETO1	1.458E-251	73.51	8.48E-247	3.99E-250	Q8TDF5	NETO1
1	NETO2	2.1751E-35	3.36	1.2653E-30	2.156E-34	Q8NC67	NETO2
1	NEURL1	2.814E-99	-8.29	1.6369E-94	4.8202E-98	O76050	NEURL1
1	NEURL1B	8.3438E-76	-3.02	4.8539E-71	1.1953E-74	A8MQ27	NEURL1B
1	NEURL4	2.6876E-27	1.60	1.5635E-22	2.3557E-26	Q96JN8	NEURL4
1	NEXN	3.131E-156	10.70	1.821E-151	6.878E-155	Q0ZGT2	NEXN
1	NEXN-AS1	9.9172E-53	10.73	5.7692E-48	1.192E-51		
1	NF1	0	-1.62	0	0	P21359	NF1
1	NF2	4.4636E-69	4.21	2.5967E-64	6.0684E-68	P35240	NF2
1	NFAM1	0.00605308	-5.62	1	0.0218025	Q8NET5	NFAM1
1	NFASC	1.1433E-76	-6.34	6.651E-72	1.6487E-75	O94856	NFASC
1	NFATC1	2.5416E-07	-3.49	0.01478565	1.2599E-06	O95644	NFATC1
1	NFATC2	4.4316E-25	3.93	2.578E-20	3.7255E-24	Q13469	NFATC2
1	NFATC3	0	-1.66	0	0	Q12968	NFATC3
1	NFATC4	1.111E-147	-15.17	6.463E-143	2.376E-146	Q14934	NFATC4
1	NFE2L2	0	-1.59	0	0	Q16236	NFE2L2
1	NFE2L3	2.0035E-24	2.58	1.1655E-19	1.6634E-23	Q9Y4A8	NFE2L3
1	NFE4	7.097E-125	154.44	4.129E-120	1.403E-123	Q86UQ8	NFE4
1	NFIB	0	-1.59	0	0	O00712	NFIB
1	NFIC	1.6128E-46	1.73	9.3821E-42	1.8235E-45	P08651	NFIC
1	NFIX	3.8408E-87	-3.24	2.2344E-82	6.029E-86	Q14938	NFIX
1	NFKB1	0	-1.77	0	0	P19838	NFKB1
1	NFKBIA	0	-2.24	0	0	P25963	NFKBIA
1	NFKBIB	1.3678E-43	1.76	7.9569E-39	1.5033E-42	Q15653	NFKBIB
1	NFKBIE	1.7458E-12	-1.59	1.0156E-07	1.0862E-11	O00221	NFKBIE
1	NFKBIZ	1.1989E-30	1.78	6.9746E-26	1.1138E-29	Q9BYH8	NFKBIZ
1	NFX1	3.263E-164	1.78	1.898E-159	7.331E-163	Q12986	NFX1
1	NFYAP1	2.9777E-11	-7.19	1.7323E-06	1.7687E-10		
1	NFYB	3.3818E-16	1.50	1.9673E-11	2.3769E-15	P25208	NFYB
1	NGDN	1.6102E-61	2.56	9.3672E-57	2.0765E-60	Q8NEJ9	NGDN
1	NGEF	2.5934E-84	-7.07	1.5087E-79	3.9881E-83	Q8N5V2	NGEF
1	NGFR	4.136E-180	-7.49	2.406E-175	9.75E-179	P08138	NGFR

1	NHEJ1	2.302E-156	3.66	1.339E-151	5.061E-155	Q9H9Q4	NHEJ1
1	NHLH2	0.00108187	-3.00	1	0.0042011	Q02577	NHLH2
1	NHLRC1	3.0762E-19	-5.27	1.7895E-14	2.3103E-18	Q6VVB1	NHLRC1
1	NHLRC3	0	-2.81	0	0	Q5JS37	NHLRC3
1	NHP2	4.683E-132	2.88	2.724E-127	9.503E-131	Q9NX24	NHP2
1	NHS	2.1678E-84	-5.58	1.2611E-79	3.3362E-83	Q6T4R5	NHS
1	NID1	1.5886E-87	36.23	9.2415E-83	2.5004E-86	P14543	NID1
1	NID2	1.076E-96	-7.09	6.2597E-92	1.8081E-95	Q14112	NID2
1	NIFK	2.4513E-94	2.61	1.426E-89	4.0616E-93	Q9BYG3	NIFK
1	NIFKP6	0.01346667	2.88	1	0.04654567		
1	NINJ1	0	-1.71	0	0	Q92982	NINJ1
1	NINJ2	2.6483E-42	-5.47	1.5406E-37	2.8609E-41	Q9NZG7	NINJ2
1	NIPAL2	5.7664E-84	-8.85	3.3545E-79	8.8417E-83	Q9H841	NIPAL2
1	NIPAL3	0	-1.75	0	0	Q6P499	NIPAL3
1	NIPAL4	9.9292E-07	-42.17	0.05776218	4.7688E-06	Q0D2K0	NIPAL4
1	NIPBL	0	-2.32	0	0	Q6KC79	NIPBL
1	NIT1	0	-1.96	0	0	Q86X76	NIT1
1	NIT2	0	-1.60	0	0	Q9NQR4	NIT2
1	NKAIN2	2.1938E-45	-7.48	1.2762E-40	2.4523E-44	Q5VXU1	NKAIN2
1	NKAIN4	0.01353552	1.76	1	0.04677043	Q8IVV8	NKAIN4
1	NKD2	5.926E-176	-14.88	3.447E-171	1.383E-174	Q969F2	NKD2
1	NKILA	3.9494E-09	2.08	0.00022975	2.1424E-08		
1	NKPD1	6.331E-08	-8.55	0.00368301	3.237E-07	Q17RQ9	NKPD1
1	NKTR	1.7798E-08	1.53	0.00103538	9.3878E-08	P30414	NKTR
1	NKX1-2	8.1857E-11	-5.46	4.762E-06	4.7744E-10	Q9UD57	NKX1-2
1	NKX2-1	3.5905E-05	-8.96	1	0.00015642	P43699	NKX2-1
1	NKX2-5	2.323E-118	326.52	1.352E-113	4.462E-117	P52952	NKX2-5
1	NKX2-8	3.541E-21	-4.59	2.0599E-16	2.7617E-20	O15522	NKX2-8
1	NKX6-1	1.1398E-29	-6.94	6.6307E-25	1.0429E-28	P78426	NKX6-1
1	NLE1	4.4434E-96	2.43	2.5849E-91	7.4386E-95	Q9NVX2	NLE1
1	NLGN1	5.117E-220	-11.04	2.977E-215	1.351E-218	Q8N2Q7	NLGN1
1	NLGN2	8.9309E-48	2.03	5.1954E-43	1.0237E-46	Q8NFZ4	NLGN2
1	NLGN3	1.8609E-09	-2.57	0.00010826	1.0246E-08	Q9NZ94	NLGN3
1	NLGN4X	0.01299626	-16.06	1	0.04498389	Q8N0W4	NLGN4X
1	NLN	2.2152E-65	1.71	1.2887E-60	2.9456E-64	Q9BYT8	NLN
1	NLRC3	0.00136425	1.76	1	0.00525242	Q7RTR2	NLRC3
1	NLRC5	6.646E-175	-7.70	3.866E-170	1.545E-173	Q86WI3	NLRC5
1	NLRP1	9.37E-104	8.73	5.451E-99	1.653E-102	Q9C000	NLRP1
1	NLRP14	5.5028E-21	6.28	3.2012E-16	4.274E-20	Q86W24	NLRP14
1	NLRP2	0	-9.65	0	0	Q9NX02	NLRP2
1	NLRP3P1	1.3541E-17	-3.98	7.8771E-13	9.8378E-17		
1	NLRP6	0.00035404	-8.24	1	0.00143207	P59044	NLRP6
1	NMB	0	-3.48	0	0	P08949	NMB
1	NME1	8.4922E-67	2.56	4.9403E-62	1.1386E-65	P15531	NME1
1	NME1-NME2	6.1201E-33	2.97	3.5603E-28	5.8819E-32		
1	NME4	4.6308E-25	2.84	2.6939E-20	3.8907E-24	O00746	NME4
1	NME5	0.00111924	-5.32	1	0.00434159	P56597	NME5

1	NME6	2.2241E-53	2.00	1.2938E-48	2.6915E-52	O75414	NME6
1	NMI	1.7222E-77	-2.88	1.0019E-72	2.4984E-76	Q13287	NMI
1	NMNAT2	1.1625E-78	-10.26	6.7625E-74	1.703E-77	Q9BZQ4	NMNAT2
1	NMNAT3	1.1954E-19	-7.36	6.9539E-15	9.0523E-19	Q96T66	NMNAT3
1	NMRAL2P	1.865E-204	-18.40	1.085E-199	4.757E-203		
1	NMRK1	0	-4.44	0	0	Q9NWW6	NMRK1
1	NMT2	1.428E-102	6.85	8.3093E-98	2.498E-101	O60551	NMT2
1	NMU	9.712E-233	-17.85	5.65E-228	2.609E-231	P48645	NMU
1	NNAT	1.923E-08	-2.36	0.00111871	1.012E-07	Q16517	NNAT
1	NNMT	6.4585E-34	229.40	3.7572E-29	6.2955E-33	P40261	NNMT
1	NNT	0	-2.44	0	0	Q13423	NNT
1	NNT-AS1	0	-2.05	0	0		
1	NOC2L	1.2026E-79	1.99	6.9959E-75	1.7788E-78	Q9Y3T9	NOC2L
1	NOC3L	7.8534E-58	1.72	4.5686E-53	9.8419E-57	Q8WTT2	NOC3L
1	NOCT	5.5492E-12	1.65	3.2282E-07	3.3885E-11	Q9UK39	NOCT
1	NOD1	0	-2.50	0	0	Q9Y239	NOD1
1	NOD2	3.8734E-10	-5.53	2.2533E-05	2.1964E-09	Q9HC29	NOD2
1	NODAL	1.8926E-09	-4.56	0.0001101	1.0416E-08	Q96S42	NODAL
1	NOG	8.746E-135	138.47	5.088E-130	1.793E-133	Q13253	NOG
1	NOL11	1.0938E-25	1.58	6.3631E-21	9.2879E-25	Q9H8H0	NOL11
1	NOL3	0	-2.03	0	0	O60936	NOL3
1	NOL4	3.9034E-10	-18.23	2.2707E-05	2.2128E-09	O94818	NOL4
1	NOL4L	1.0018E-54	3.29	5.8276E-50	1.2276E-53	Q96MY1	NOL4L
1	NOL6	1.0361E-28	2.83	6.0275E-24	9.3334E-28	Q9H6R4	NOL6
1	NOL7	5.8637E-51	1.87	3.4112E-46	6.9164E-50	Q9UMY1	NOL7
1	NOL9	6.301E-112	3.23	3.666E-107	1.17E-110	Q5SY16	NOL9
1	NOLC1	1.0482E-51	1.53	6.0978E-47	1.245E-50	Q14978	NOLC1
1	NOM1	4.1543E-27	1.54	2.4167E-22	3.6249E-26	Q5C9Z4	NOM1
1	NOMO2	0	-1.57	0	0	Q5JPE7	NOMO2
1	NOMO3	0	-2.83	0	0	P69849	NOMO3
1	NONO	1.741E-159	1.58	1.013E-154	3.868E-158	Q15233	NONO
1	NONOP2	8.5012E-05	-2.27	1	0.00036064		
1	NOP10	4.5814E-13	1.58	2.6652E-08	2.9067E-12	Q9NPE3	NOP10
1	NOP16	1.091E-107	2.72	6.35E-103	1.968E-106	Q9Y3C1	NOP16
1	NOP2	4.474E-131	2.38	2.603E-126	9.044E-130	P46087	NOP2
1	NOP56	8.265E-137	1.92	4.808E-132	1.709E-135	O00567	NOP56
1	NOP58	1.2192E-27	1.52	7.0928E-23	1.0768E-26	Q9Y2X3	NOP58
1	NOP9	3.3567E-55	1.62	1.9527E-50	4.1293E-54	Q86U38	NOP9
1	NOS1AP	2.3773E-21	-4.21	1.383E-16	1.8606E-20	O75052	NOS1AP
1	NOS3	1.3966E-05	-7.58	0.81243572	6.2495E-05	P29474	NOS3
1	NOSIP	6.6E-102	2.62	3.8395E-97	1.15E-100	Q9Y314	NOSIP
1	NOSTRIN	4.3445E-32	3.84	2.5274E-27	4.1189E-31	Q8IVI9	NOSTRIN
1	NOTCH2	3.0391E-33	1.81	1.7679E-28	2.9285E-32	Q04721	NOTCH2
1	NOTCH3	7.048E-176	-10.10	4.1E-171	1.644E-174	Q9UM47	NOTCH3
1	NOV	1.397E-261	-16.70	8.126E-257	3.855E-260	P48745	NOV
1	NOVA1	4.3639E-96	-10.47	2.5387E-91	7.3076E-95	P51513	NOVA1
1	NOVA1-AS1	1.5681E-90	-13.44	9.122E-86	2.5262E-89		

1	NOX3	4.7969E-08	56.93	0.00279057	2.4704E-07	Q9HBY0	NOX3
1	NOX4	1.8142E-21	-4.95	1.0554E-16	1.4233E-20	Q9NPH5	NOX4
1	NOXA1	3.0885E-23	-2.05	1.7967E-18	2.5059E-22	Q86UR1	NOXA1
1	NPAS1	0.00307299	-1.56	1	0.01142068	Q99742	NPAS1
1	NPAS2	0	61.81	0	0	Q99743	NPAS2
1	NPC1	0	-1.53	0	0	O15118	NPC1
1	NPDC1	0	173.77	0	0	Q9NQX5	NPDC1
1	NPEPL1	4.4154E-41	1.71	2.5686E-36	4.683E-40	Q8NDH3	NPEPL1
1	NPFFR2	3.6569E-49	-11.59	2.1274E-44	4.2522E-48	Q9Y5X5	NPFFR2
1	NPIPA1	1.4636E-13	1.60	8.5143E-09	9.4445E-13	Q9UND3	NPIPA1
1	NPIPA2	0.00102186	2.36	1	0.00397977	E9PIF3	NPIPA2
1	NPIPA5	5.2922E-14	-3.01	3.0787E-09	3.4642E-13	E9PKD4	NPIPA5
1	NPIPA8	6.8919E-17	67.81	4.0093E-12	4.9327E-16	P0DM63	NPIPA8
1	NPIPB13	0.00135383	-1.69	1	0.00521334	A6NJU9	NPIPB13
1	NPIPB14P	1.5381E-13	-2.81	8.948E-09	9.919E-13		
1	NPIPB15	1.048E-102	-8.91	6.0943E-98	1.833E-101	A6NHN6	NPIPB15
1	NPIPB2	9.6498E-19	2.64	5.6137E-14	7.1749E-18		
1	NPIPB4	0.00043722	-1.57	1	0.00175486	C9JG80	NPIPB4
1	NPIPB6	0.00221988	-3.08	1	0.00835746	E9PJ23	NPIPB6
1	NPL	2.9145E-54	-4.08	1.6955E-49	3.5567E-53	Q9BXD5	NPL
1	NPM1	1.3278E-67	2.51	7.7246E-63	1.7898E-66	P06748	NPM1
1	NPM1P9	0.00149014	-2.31	1	0.00570801		
1	NPM2	4.4745E-16	-8.72	2.603E-11	3.1297E-15	Q86SE8	NPM2
1	NPNT	8.199E-120	-28.64	4.77E-115	1.584E-118	Q6UXI9	NPNT
1	NPPA-AS1	1.0943E-37	2.66	6.366E-33	1.1127E-36		
1	NPR3	3.4003E-05	-2.79	1	0.00014838	P17342	NPR3
1	NPRL2	1.052E-100	2.81	6.1176E-96	1.815E-99	Q8WTW4	NPRL2
1	NPTN	0	-1.94	0	0	Q9Y639	NPTN
1	NPTXR	5.705E-114	-7.40	3.319E-109	1.068E-112	O95502	NPTXR
1	NPW	8.9407E-07	-1.85	0.0520116	4.3035E-06	Q8N729	NPW
1	NPY1R	9.1808E-82	-9.01	5.3409E-77	1.3815E-80	P25929	NPY1R
1	NPY4R	0.00512599	-3.83	1	0.01862583	P50391	NPY4R
1	NPY6R	1.9768E-08	-28.58	0.00114998	1.0395E-07	Q99463	NPY6R
1	NQO1	0	-2.09	0	0	P15559	NQO1
1	NQO2	0	-1.53	0	0	P16083	NQO2
1	NR1D1	0	-3.12	0	0	P20393	NR1D1
1	NR1H3	7.7354E-17	-1.61	4.5E-12	5.5289E-16	Q13133	NR1H3
1	NR1I2	0.00110007	-4.73	1	0.00426951	O75469	NR1I2
1	NR1I3	8.8363E-07	-2.24	0.05140455	4.2546E-06	Q14994	NR1I3
1	NR2C2AP	1.5113E-57	2.18	8.7916E-53	1.8899E-56	Q86WQ0	NR2C2AP
1	NR2E1	0.00173555	-4.93	1	0.00660837	Q9Y466	NR2E1
1	NR2E3	2.1572E-22	4.15	1.2549E-17	1.7207E-21	Q9Y5X4	NR2E3
1	NR2F1	4.826E-160	11.65	2.807E-155	1.073E-158	P10589	NR2F1
1	NR2F1-AS1	1.217E-110	4.69	7.081E-106	2.238E-109		
1	NR2F2	0	-1.83	0	0	P24468	NR2F2
1	NR2F2-AS1	2.9945E-57	-6.19	1.742E-52	3.735E-56		
1	NR2F6	0	-1.72	0	0	P10588	NR2F6

1	NR3C1	9.1904E-36	1.51	5.3464E-31	9.1485E-35	P04150	NR3C1
1	NR4A2	8.0143E-25	-1.99	4.6623E-20	6.7064E-24	P43354	NR4A2
1	NR5A2	0.00352939	1.62	1	0.01305184	O00482	NR5A2
1	NR6A1	0	-2.28	0	0	Q15406	NR6A1
1	NRARP	8.2555E-50	-3.87	4.8026E-45	9.6437E-49	Q7Z6K4	NRARP
1	NRAS	3.2112E-81	2.19	1.8681E-76	4.8122E-80	P01111	NRAS
1	NRAV	1.7012E-23	1.65	9.8967E-19	1.3865E-22		
1	NRBF2	1.2806E-23	1.75	7.45E-19	1.0468E-22	Q96F24	NRBF2
1	NRCAM	2.217E-200	-9.96	1.29E-195	5.6E-199	Q92823	NRCAM
1	NRDE2	1.9125E-38	1.66	1.1126E-33	1.965E-37	Q9H7Z3	NRDE2
1	NREP	1.5763E-52	-2.68	9.1698E-48	1.8891E-51	Q16612	NREP
1	NRG1	0	-2.02	0	0	Q02297	NRG1
1	NRG2	2.179E-125	28.27	1.268E-120	4.314E-124	O14511	NRG2
1	NRG3	4.663E-05	-3.80	1	0.00020155	P56975	NRG3
1	NRG4	3.3392E-87	-4.94	1.9426E-82	5.2459E-86	Q8WWG1	NRG4
1	NRGN	5.759E-212	13.54	3.35E-207	1.495E-210	Q92686	NRGN
1	NRIP3	4.8145E-30	-4.86	2.8008E-25	4.4267E-29	Q9NQ35	NRIP3
1	NRK	7.0121E-18	157.87	4.0792E-13	5.1208E-17	Q7Z2Y5	NRK
1	NRL	1.2321E-06	2.34	0.07167767	5.8902E-06	P54845	NRL
1	NRN1L	7.8725E-06	-4.30	0.45797717	3.5802E-05	Q496H8	NRN1L
1	NRP1	1.066E-240	18.30	6.199E-236	2.891E-239	O14786	NRP1
1	NRP2	0	-2.19	0	0	O60462	NRP2
1	NRROS	3.7972E-06	-15.51	0.22089599	1.7584E-05	Q86YC3	NRROS
1	NRSN2	2.1099E-49	1.68	1.2274E-44	2.4558E-48	Q9GZP1	NRSN2
1	NRSN2-AS1	1.5459E-14	1.87	8.9931E-10	1.0285E-13		
1	NRTN	1.0899E-18	-18.00	6.3407E-14	8.0958E-18	Q99748	NRTN
1	NRXN3	1.28E-101	19.88	7.4489E-97	2.227E-100	Q9Y4C0	NRXN3
1	NSA2	1.5585E-63	2.52	9.0665E-59	2.042E-62	O95478	NSA2
1	NSD3	0	-1.59	0	0	Q9BZ95	NSD3
1	NSFP1	3.8797E-43	31.14	2.257E-38	4.2329E-42		
1	NSMF	1.718E-105	1.81	9.992E-101	3.057E-104	Q6X4W1	NSMF
1	NSUN3	2.2204E-16	-1.58	1.2917E-11	1.5667E-15	Q9H649	NSUN3
1	NSUN5P1	2.2204E-15	-1.65	1.2917E-10	1.5182E-14	Q3KNT7	NSUN5P1
1	NSUN7	3.0907E-71	-20.32	1.798E-66	4.2748E-70	Q8NE18	NSUN7
1	NT5C1B	4.636E-11	-3.67	2.6969E-06	2.728E-10	Q96P26	NT5C1B
1	NT5C2	9.589E-115	1.67	5.578E-110	1.803E-113	P49902	NT5C2
1	NT5C3A	0	-1.82	0	0	Q9H0P0	NT5C3A
1	NT5DC3	1.234E-113	3.14	7.182E-109	2.307E-112	Q86UY8	NT5DC3
1	NT5M	0.00427004	-1.50	1	0.01565545	Q9NPB1	NT5M
1	NTAN1	1.7052E-93	3.01	9.9198E-89	2.8077E-92	Q96AB6	NTAN1
1	NTAN1P2	0.01033597	-1.95	1	0.03621541		
1	NTF3	0.00014737	-12.56	1	0.00061414	P20783	NTF3
1	NTF4	1.673E-257	-12.25	9.734E-253	4.603E-256	P34130	NTF4
1	NTHL1	3.3025E-97	2.96	1.9212E-92	5.5735E-96	P78549	NTHL1
1	NTM	1.4423E-15	13.76	8.3903E-11	9.9071E-15	Q9P121	NTM
1	NTMT1	7.8874E-97	2.12	4.5884E-92	1.3261E-95	Q9BV86	NTMT1
1	NTN4	2.6291E-57	5.27	1.5295E-52	3.2814E-56	Q9HB63	NTN4

1	NTNG1	2.6185E-72	12.14	1.5233E-67	3.6512E-71	Q9Y2I2	NTNG1
1	NTNG2	1.1935E-19	6.91	6.9428E-15	9.0402E-19	Q96CW9	NTNG2
1	NTS	2.8865E-07	-7.16	0.01679174	1.4258E-06	P30990	NTS
1	NTSR1	5.3102E-09	4.27	0.00030892	2.864E-08	P30989	NTSR1
1	NUAK1	1.6438E-35	2.30	9.5627E-31	1.6324E-34	O60285	NUAK1
1	NUAK2	6.4449E-23	1.69	3.7493E-18	5.1993E-22	Q9H093	NUAK2
1	NUB1	1.1916E-39	1.62	6.9319E-35	1.2407E-38	Q9Y5A7	NUB1
1	NUBP2	7.7705E-47	1.77	4.5204E-42	8.8237E-46	Q9Y5Y2	NUBP2
1	NUCB2	2.2267E-97	2.81	1.2954E-92	3.7623E-96	P80303	NUCB2
1	NUDC	5.246E-103	2.22	3.0515E-98	9.211E-102	Q9Y266	NUDC
1	NUDCD3	0	-1.62	0	0	Q8IVD9	NUDCD3
1	NUDT16P1	3.9984E-44	-2.20	2.326E-39	4.4129E-43		
1	NUDT17	1.8952E-05	-1.58	1	8.4035E-05	P0C025	NUDT17
1	NUDT18	2.584E-91	-3.96	1.5032E-86	4.185E-90	Q6ZVK8	NUDT18
1	NUDT19	2.6266E-27	1.62	1.528E-22	2.3033E-26	A8MXV4	NUDT19
1	NUDT2	1.6768E-35	1.79	9.7546E-31	1.6649E-34	P50583	NUDT2
1	NUDT4	1.548E-33	1.69	9.0052E-29	1.5004E-32	Q9NZJ9	NUDT4
1	NUDT4P1	1.9305E-29	2.60	1.1231E-24	1.7589E-28		
1	NUDT6	3.1157E-15	-1.61	1.8125E-10	2.1226E-14	P53370	NUDT6
1	NUDT7	1.0658E-23	2.35	6.2004E-19	8.7194E-23	P0C024	NUDT7
1	NUFIP1	3.7981E-30	1.68	2.2095E-25	3.4994E-29	Q9UHK0	NUFIP1
1	NUFIP1P	0.001689	-11.24	1	0.00643837		
1	NUMA1	0	-1.72	0	0	Q14980	NUMA1
1	NUP107	3.6471E-58	1.61	2.1216E-53	4.5873E-57	P57740	NUP107
1	NUP133	0	-1.66	0	0	Q8WUM0	NUP133
1	NUP153	1.0084E-72	2.06	5.8663E-68	1.4095E-71	P49790	NUP153
1	NUP210	1.0021E-21	-9.85	5.8293E-17	7.8999E-21	Q8TEM1	NUP210
1	NUP35	7.7989E-23	1.64	4.5369E-18	6.2847E-22	Q8NFH5	NUP35
1	NUP43	5.4994E-49	1.61	3.1992E-44	6.3755E-48	Q8NFH3	NUP43
1	NUP50	3.591E-101	1.72	2.0889E-96	6.228E-100	Q9UKX7	NUP50
1	NUP62CL	1.042E-193	-9.71	6.064E-189	2.573E-192	Q9H1M0	NUP62CL
1	NUP85	1.3E-52	1.55	7.5624E-48	1.5596E-51	Q9BW27	NUP85
1	NUP98	1.152E-103	1.75	6.703E-99	2.032E-102	P52948	NUP98
1	NUTM2B-AS1	3.7852E-14	1.55	2.202E-09	2.4893E-13		
1	NUTM2E	1.4934E-05	7.34	0.86874796	6.6693E-05		
1	NUTM2HP	0.00021587	-6.35	1	0.00088915		
1	NXF2B	0.00186906	23.73	1	0.00709914		
1	NXN	0	-2.36	0	0	Q6DKJ4	NXN
1	NXNL2	1.3016E-05	5.91	0.75719162	5.8353E-05	Q5VZ03	NXNL2
1	NXPH1	1.3273E-05	-4.18	0.77212234	5.9476E-05	P58417	NXPH1
1	NXPH4	1.1173E-41	-2.25	6.4998E-37	1.1959E-40	O95158	NXPH4
1	NYAP1	1.1564E-05	-3.00	0.6727402	5.2001E-05	Q6ZVC0	NYAP1
1	NYNRIN	1.2724E-11	-9.79	7.402E-07	7.6768E-11	Q9P2P1	NYNRIN
1	OAF	1.2651E-83	-3.02	7.3596E-79	1.9311E-82	Q86UD1	OAF
1	OAS1	1.152E-120	-32.56	6.703E-116	2.236E-119	P00973	OAS1
1	OAS2	1.0816E-59	-18.11	6.2921E-55	1.3759E-58	P29728	OAS2
1	OAS3	0	-6.03	0	0	Q9Y6K5	OAS3

1	OBSL1	2.3271E-71	9.10	1.3537E-66	3.2217E-70	O75147	OBSL1
1	OCIAD2	0	-1.61	0	0	Q56VL3	OCIAD2
1	OCLM	0.00888163	-2.27	1	0.03137478	Q9Y5M6	OCLM
1	OCLN	0	-3.27	0	0	Q16625	OCLN
1	ODC1	2.3012E-57	5.29	1.3387E-52	2.8734E-56	P11926	ODC1
1	ODF2-AS1	0.00482576	-4.04	1	0.01758544		
1	ODF3B	6.5772E-31	-3.97	3.8262E-26	6.1357E-30		
1	ODF3L1	1.9842E-05	-6.96	1	8.7866E-05		
1	ODF3L2	0.00057775	-7.46	1	0.002297	Q3SX64	ODF3L2
1	OFCC1	3.27E-107	-13.09	1.902E-102	5.877E-106	Q8IZS5	OFCC1
1	OFD1	0	-2.22	0	0	O75665	OFD1
1	OGDH	0	-2.14	0	0	Q02218	OGDH
1	OGDHL	1.6371E-12	-3.95	9.5238E-08	1.0194E-11	Q9ULD0	OGDHL
1	OGFOD1	9.9533E-50	1.64	5.7902E-45	1.1622E-48	Q8N543	OGFOD1
1	OGFR	7.3455E-41	3.13	4.2732E-36	7.7638E-40	Q9NZT2	OGFR
1	OGFR-AS1	0.00624294	1.79	1	0.02245021		
1	OGG1	2.3533E-27	1.61	1.369E-22	2.0668E-26	O15527	OGG1
1	OLA1	7.31E-82	2.02	4.2525E-77	1.1014E-80	Q9NTK5	OLA1
1	OLFM2	1.7249E-07	-2.28	0.01003459	8.6252E-07	O95897	OLFM2
1	OLFM3	4.1323E-11	-5.62	2.4039E-06	2.4363E-10	Q96PB7	OLFM3
1	OLFM5P	6.1778E-08	-87.74	0.00359387	3.1614E-07		
1	OLFML2A	0	-15.99	0	0	Q68BL7	OLFML2A
1	OLFML2B	0.0017336	-4.03	1	0.00660146	Q68BL8	OLFML2B
1	OLMALINC	0	-3.34	0	0		
1	OLR1	1.3026E-26	-175.03	7.578E-22	1.1247E-25	P78380	OLR1
1	OMA1	0	-1.52	0	0	Q96E52	OMA1
1	ONECUT1	1.1011E-09	6.76	6.4057E-05	6.1223E-09	Q9UBC0	ONECUT1
1	ONECUT2	7.915E-193	10.73	4.605E-188	1.949E-191	O95948	ONECUT2
1	ONECUT3	0.00098829	-23.45	1	0.00385364	O60422	ONECUT3
1	OOSP1	1.3745E-06	8.93	0.07995893	6.5508E-06	A8MZH6	OOSP1
1	OPA1	0	-1.62	0	0	O60313	OPA1
1	OPLAH	3.802E-127	-5.43	2.212E-122	7.595E-126	O14841	OPLAH
1	OPN1SW	1.2564E-20	2.15	7.3089E-16	9.6871E-20	P03999	OPN1SW
1	OPN3	0	-1.63	0	0	Q9H1Y3	OPN3
1	OPRL1	3.7218E-17	-5.69	2.1651E-12	2.6766E-16	P41146	OPRL1
1	OPRM1	0.00241456	-5.37	1	0.00905816	P35372	OPRM1
1	OPTN	0	-1.59	0	0	Q96CV9	OPTN
1	OR1H1P	0.00077916	-10.04	1	0.00306344		
1	OR2A1-AS1	3.3403E-10	-1.53	1.9432E-05	1.8984E-09		
1	OR2A7	1.2164E-14	-2.88	7.076E-10	8.1194E-14	Q96R45	OR2A7
1	OR2B6	0.00102664	-2.49	1	0.0039973	P58173	OR2B6
1	OR4C6	0.00645128	-3.94	1	0.02314932	Q8NH72	OR4C6
1	OR51B4	1.5764E-06	-16.71	0.09170605	7.4862E-06	Q9Y5P0	OR51B4
1	OR8A1	8.2342E-07	-55.95	0.04790168	3.9726E-06	Q8NGG7	OR8A1
1	OR8G3P	1.1032E-10	-39.87	6.4177E-06	6.4029E-10	P0DMU2	OR8G3P
1	OR8G5	1.4146E-05	-7.19	0.82293235	6.3278E-05	Q8NG78	OR8G5
1	OR8R1P	1.2734E-17	-24.99	7.4076E-13	9.2561E-17		

1	OR8S1	0.01199772	-5.26	1	0.04171131	Q8NH09	OR8S1
1	OR9K1P	6.0886E-08	-12.04	0.00354199	3.1169E-07		
1	ORAI2	5.9373E-32	1.61	3.454E-27	5.619E-31	Q96SN7	ORAI2
1	ORAI3	4.538E-113	-3.24	2.64E-108	8.463E-112	Q9BRQ5	ORAI3
1	ORAOV1	0	-2.60	0	0	Q8WV07	ORAOV1
1	ORC2	6.272E-106	2.09	3.649E-101	1.12E-104	Q13416	ORC2
1	ORMDL2	0	-1.72	0	0	Q53FV1	ORMDL2
1	OS9	0	-1.61	0	0	Q13438	OS9
1	OSBP	0	-1.90	0	0	P22059	OSBP
1	OSBP2	2.9763E-25	2.27	1.7314E-20	2.5097E-24	Q969R2	OSBP2
1	OSBPL10	8.3682E-65	1.63	4.8681E-60	1.1071E-63	Q9BXB5	OSBPL10
1	OSBPL11	8.8294E-42	1.74	5.1364E-37	9.468E-41	Q9BXB4	OSBPL11
1	OSBPL5	6.7636E-67	4.98	3.9347E-62	9.0744E-66	Q9H0X9	OSBPL5
1	OSBPL6	8.7207E-33	-1.92	5.0732E-28	8.3633E-32	Q9BZF3	OSBPL6
1	OSBPL7	0	-2.21	0	0	Q9BZF2	OSBPL7
1	OSCAR	9.662E-258	20.56	5.621E-253	2.66E-256	Q8IYS5	OSCAR
1	OSCP1	1.8672E-20	2.35	1.0862E-15	1.4351E-19	Q8WVF1	OSCP1
1	OSGEP	3.0786E-43	2.31	1.7909E-38	3.3652E-42	Q9NPF4	OSGEP
1	OSGEPL1-AS1	0.00836919	-1.86	1	0.02967448		
1	OSGIN1	0	-2.05	0	0	Q9UJX0	OSGIN1
1	OSMR	0	-1.84	0	0	Q99650	OSMR
1	OSMR-AS1	3.9096E-21	-3.32	2.2744E-16	3.0467E-20		
1	OSR1	2.277E-69	20.05	1.3246E-64	3.1044E-68	Q8TAX0	OSR1
1	OSR2	2.1023E-14	-1.82	1.223E-09	1.3931E-13	Q8N2R0	OSR2
1	OSTF1	0	-1.86	0	0	Q92882	OSTF1
1	OTOGL	2.46E-09	-5.09	0.00014311	1.3482E-08	Q3ZCN5	OTOGL
1	OTUB1	0	-2.23	0	0	Q96FW1	OTUB1
1	OTUD3	5.151E-26	1.84	2.9966E-21	4.3983E-25	Q5T2D3	OTUD3
1	OTUD6B-AS1	9.4813E-14	-1.58	5.5157E-09	6.1572E-13		
1	OTUD7A	1.4766E-29	5.90	8.5902E-25	1.3481E-28	Q8TE49	OTUD7A
1	OTULIN	0	-1.75	0	0	Q96BN8	OTULIN
1	OTX2-AS1	8.5506E-08	-4.90	0.00497421	4.3434E-07		
1	OVCA2	4.1692E-12	2.29	2.4254E-07	2.559E-11	Q8WZ82	OVCA2
1	OVCH1	1.0453E-15	-8.29	6.0812E-11	7.2146E-15	Q7RTY7	OVCH1
1	OVCH1-AS1	8.827E-107	-4.19	5.135E-102	1.584E-105		
1	OVGP1	2.2261E-09	1.65	0.0001295	1.2216E-08	Q12889	OVGP1
1	OVOL1	4.93E-240	-16.56	2.868E-235	1.336E-238	O14753	OVOL1
1	OVOL1-AS1	5.5829E-08	-13.59	0.00324778	2.8645E-07		
1	OVOL2	2.2367E-90	-8.10	1.3012E-85	3.5984E-89	Q9BRP0	OVOL2
1	OVOL3	0.00032357	-1.87	1	0.00131199	O00110	OVOL3
1	OVOS2	1.008E-127	-9.34	5.862E-123	2.017E-126	Q6IE36	OVOS2
1	OXCT2	1.7659E-19	-8.19	1.0273E-14	1.3326E-18	Q9BYC2	OXCT2
1	OXER1	0.00065106	-7.82	1	0.00257739	Q8TDS5	OXER1
1	OXGR1	0.00277741	-4.13	1	0.01036456	Q96P68	OXGR1
1	OXR1	0	-2.31	0	0	Q8N573	OXR1
1	OXTR	1.8789E-10	2.02	1.093E-05	1.0801E-09	P30559	OXTR
1	P2RX5	1.013E-242	292.36	5.896E-238	2.754E-241	Q93086	P2RX5

1	P2RX5-TAX1B	7.7453E-08	2.94	0.00450575	3.9438E-07		
1	P2RX6	4.3131E-18	-7.52	2.5091E-13	3.1653E-17	O15547	P2RX6
1	P2RY1	1.5301E-64	-3.56	8.9013E-60	2.0203E-63	P47900	P2RY1
1	P2RY11	3.6837E-30	2.15	2.1429E-25	3.3956E-29	Q96G91	P2RY11
1	P2RY2	0	-8.09	0	0	P41231	P2RY2
1	P2RY6	8.1867E-75	-18.93	4.7626E-70	1.1636E-73	Q15077	P2RY6
1	P3H2	1.1665E-46	3.59	6.7861E-42	1.3213E-45	Q8IVL5	P3H2
1	P3H3	0	118.64	0	0	Q8IVL6	P3H3
1	P4HA2-AS1	9.6817E-05	-1.68	1	0.00040938		
1	P4HA3	2.7595E-05	-4.22	1	0.00012103	Q7Z4N8	P4HA3
1	P4HTM	0	-1.56	0	0	Q9NXG6	P4HTM
1	PA2G4	7.5025E-39	1.59	4.3645E-34	7.7453E-38	Q9UQ80	PA2G4
1	PAAF1	0	-1.56	0	0	Q9BRP4	PAAF1
1	PABPC1	0	-1.61	0	0	P11940	PABPC1
1	PABPC1L	1.7238E-11	1.65	1.0028E-06	1.0346E-10	Q4VXU2	PABPC1L
1	PABPC1P4	2.3048E-07	-6.40	0.01340803	1.1448E-06		
1	PABPC4L	1.9617E-18	-3.61	1.1412E-13	1.449E-17	P0CB38	PABPC4L
1	PABPC5	4.1399E-14	-5.91	2.4084E-09	2.7185E-13	Q96DU9	PABPC5
1	PABPN1	7.1033E-68	1.59	4.1323E-63	9.5854E-67	Q86U42	PABPN1
1	PACERR	4.712E-14	-6.91	2.7412E-09	3.089E-13		
1	PACS1	0	-2.04	0	0	Q6VY07	PACS1
1	PACSIN1	2.4523E-05	-6.13	1	0.00010795	Q9BY11	PACSIN1
1	PADI1	4.4267E-41	-2.52	2.5752E-36	4.6941E-40	Q9ULC6	PADI1
1	PADI3	1.0286E-12	-1.90	5.9835E-08	6.4505E-12	Q9ULW8	PADI3
1	PADI4	4.8756E-20	-6.08	2.8363E-15	3.7232E-19	Q9UM07	PADI4
1	PAGR1	2.138E-141	2.58	1.244E-136	4.499E-140	Q9BTK6	PAGR1
1	PAH	2.1875E-30	-17.64	1.2726E-25	2.0225E-29	P00439	PAH
1	PAICSP1	0.00021591	4.00	1	0.00088921		
1	PAK1IP1	5.5112E-70	2.32	3.2061E-65	7.5527E-69	Q9NWT1	PAK1IP1
1	PAK2	0	-1.52	0	0	Q13177	PAK2
1	PAK3	4.96E-151	317.31	2.886E-146	1.071E-149	O75914	PAK3
1	PAK6	0	-4.30	0	0	Q9NQU5	PAK6
1	PALM2	0.0008428	7.23	1	0.00330295	Q8IXS6	PALM2
1	PALM2-AKAP2	1.0314E-12	7.25	5.9999E-08	6.4675E-12	B1ALY0	PALM2-AKAP2
1	PALM3	1.2581E-05	-13.71	0.73189565	5.646E-05	A6NDB9	PALM3
1	PALMD	7.4705E-13	-3.19	4.3459E-08	4.7064E-12	Q9NP74	PALMD
1	PAM	0	-1.67	0	0	P19021	PAM
1	PAMR1	5.8335E-37	-32.68	3.3936E-32	5.8875E-36	Q6UXH9	PAMR1
1	PAN2	0	-2.07	0	0	Q504Q3	PAN2
1	PANCR	1.8508E-08	-8.75	0.00107667	9.7525E-08		
1	PANK3	2.7723E-26	1.66	1.6128E-21	2.3812E-25	Q9H999	PANK3
1	PANK4	1.1568E-97	1.96	6.7297E-93	1.9586E-96	Q9NVE7	PANK4
1	PANO1	1.5645E-13	3.34	9.1011E-09	1.0085E-12	I0J062	PANO1
1	PANX2	1.8173E-52	-3.55	1.0572E-47	2.1771E-51	Q96RD6	PANX2
1	PANX3	0.01102609	-5.62	1	0.03850362	Q96QZ0	PANX3
1	PAPD7	0	-1.70	0	0	Q5XG87	PAPD7
1	PAPLN	4.049E-147	-6.55	2.355E-142	8.64E-146	O95428	PAPLN

1	PAPOLA	2.3606E-46	1.61	1.3732E-41	2.666E-45	P51003	PAPOLA
1	PAPPA	5.8526E-06	4.74	0.34047085	2.6832E-05	Q13219	PAPPA
1	PAPPA2	3.5724E-27	-5.00	2.0782E-22	3.1218E-26	Q9BXP8	PAPPA2
1	PAPSS2	2.4078E-67	4.88	1.4007E-62	3.2416E-66	O95340	PAPSS2
1	PAQR5	1.1913E-06	-1.85	0.06930079	5.6981E-06	Q9NXK6	PAQR5
1	PAQR6	4.6583E-54	-4.02	2.7099E-49	5.6776E-53	Q6TCH4	PAQR6
1	PAQR7	0	-1.61	0	0	Q86WK9	PAQR7
1	PAQR9	2.3852E-42	-27.58	1.3875E-37	2.5776E-41	Q6ZVX9	PAQR9
1	PAQR9-AS1	3.9256E-17	-5.44	2.2837E-12	2.8218E-16		
1	PARD3	2.9361E-26	2.73	1.708E-21	2.5203E-25	Q8TEW0	PARD3
1	PARD3-AS1	1.6752E-10	4.68	9.7451E-06	9.6524E-10		
1	PARD3B	1.8385E-85	4.28	1.0695E-80	2.8482E-84	Q8TEW8	PARD3B
1	PARD6A	4.8103E-68	4.60	2.7983E-63	6.4986E-67	Q9NPB6	PARD6A
1	PARD6B	6.4521E-06	1.51	0.37534669	2.9511E-05	Q9BYG5	PARD6B
1	PARD6G	2.2169E-11	-1.51	1.2897E-06	1.3234E-10	Q9BYG4	PARD6G
1	PARD6G-AS1	2.2675E-10	-2.35	1.3191E-05	1.2986E-09		
1	PARG	2.6881E-30	1.51	1.5638E-25	2.4822E-29	Q86W56	PARG
1	PARGP1	0.00046415	1.52	1	0.00185973		
1	PARK2	3.9941E-27	2.97	2.3235E-22	3.4872E-26		
1	PARK7	6.648E-64	2.03	3.8674E-59	8.7399E-63	Q99497	PARK7
1	PARM1	7.504E-10	-2.06	4.3654E-05	4.2023E-09	Q6UWI2	PARM1
1	PARN	9.4515E-43	1.51	5.4983E-38	1.0268E-41	O95453	PARN
1	PARP1	0	-1.50	0	0	P09874	PARP1
1	PARP10	0	-3.44	0	0	Q53GL7	PARP10
1	PARP11	5.5758E-66	3.42	3.2437E-61	7.4396E-65	Q9NR21	PARP11
1	PARP12	0	-2.41	0	0	Q9H0J9	PARP12
1	PARP14	0	-3.43	0	0	Q460N5	PARP14
1	PARP15	1.6141E-22	-11.35	9.3898E-18	1.2928E-21	Q460N3	PARP15
1	PARP16	1.4748E-21	1.76	8.5793E-17	1.1592E-20	Q8N5Y8	PARP16
1	PARP2	6.125E-50	1.89	3.5632E-45	7.1679E-49	Q9UGN5	PARP2
1	PARP3	2.3617E-53	1.93	1.3739E-48	2.8576E-52	Q9Y6F1	PARP3
1	PARP4	0	-2.04	0	0	Q9UKK3	PARP4
1	PARP9	2.3806E-78	-5.97	1.3849E-73	3.4779E-77	Q8IXQ6	PARP9
1	PART1	1.9186E-21	-5.86	1.1161E-16	1.5046E-20		
1	PARVA	1.1897E-45	3.77	6.9208E-41	1.3353E-44	Q9NVD7	PARVA
1	PATJ	0	-2.93	0	0	Q8NI35	PATJ
1	PATL2	6.3103E-36	-5.32	3.671E-31	6.2923E-35	C9JE40	PATL2
1	PATZ1	7.871E-109	2.23	4.579E-104	1.43E-107	Q9HBE1	PATZ1
1	PAX5	2.879E-218	81.06	1.675E-213	7.581E-217	Q02548	PAX5
1	PAX8	5.78E-172	52.93	3.363E-167	1.33E-170	Q06710	PAX8
1	PAX8-AS1	1.0939E-18	2.63	6.3635E-14	8.124E-18		
1	PAX9	4.2185E-32	-2.00	2.454E-27	4.0007E-31	P55771	PAX9
1	PAXIP1	1.4742E-63	1.63	8.576E-59	1.932E-62	Q6ZW49	PAXIP1
1	PBDC1	3.5011E-16	1.57	2.0368E-11	2.4595E-15	Q9BVG4	PBDC1
1	PBK	4.4409E-16	-1.51	2.5834E-11	3.1073E-15	Q96KB5	PBK
1	PBX1	0.00686827	-1.70	1	0.02457288	P40424	PBX1
1	PBX3	8.7759E-70	2.17	5.1053E-65	1.1996E-68	P40426	PBX3

1	PBX4	6.8456E-70	6.51	3.9824E-65	9.3681E-69	Q9BYU1	PBX4
1	PBXIP1	1.8761E-30	1.60	1.0914E-25	1.7371E-29	Q96AQ6	PBXIP1
1	PC	0	-2.03	0	0	P11498	PC
1	PCAT1	1.1418E-16	-6.24	6.6426E-12	8.1255E-16		
1	PCAT14	1.8282E-05	-2.94	1	8.1179E-05		
1	PCAT2	0.00846649	-16.00	1	0.02999387		
1	PCAT5	1.2361E-06	46.66	0.07190692	5.9081E-06		
1	PCAT6	6.8249E-14	-6.46	3.9703E-09	4.451E-13		
1	PCAT7	2.0105E-26	-3.73	1.1696E-21	1.7317E-25		
1	PCBD1	3.0726E-47	4.29	1.7875E-42	3.5007E-46	P61457	PCBD1
1	PCBP4	6.8121E-76	5.33	3.9629E-71	9.7656E-75	P57723	PCBP4
1	PCCB	0	-1.53	0	0	P05166	PCCB
1	PCDH1	0	-14.62	0	0	Q08174	PCDH1
1	PCDH10	8.359E-132	-9.95	4.863E-127	1.694E-130	Q9P2E7	PCDH10
1	PCDH11X	2.337E-187	8.21	1.36E-182	5.625E-186	Q9BZA7	PCDH11X
1	PCDH11Y	2.4823E-12	-2.53	1.444E-07	1.5369E-11	Q9BZA8	PCDH11Y
1	PCDH12	1.023E-19	4.26	5.9512E-15	7.7631E-19	Q9NPG4	PCDH12
1	PCDH15	3.247E-123	-6.75	1.889E-118	6.384E-122	Q96QU1	PCDH15
1	PCDH17	0.00035799	-5.84	1	0.00144732	O14917	PCDH17
1	PCDH18	1.5945E-34	-12.27	9.2758E-30	1.5669E-33	Q9HCL0	PCDH18
1	PCDH20	3.045E-246	-9.12	1.772E-241	8.298E-245	Q8N6Y1	PCDH20
1	PCDH7	3.059E-118	-9.74	1.78E-113	5.872E-117	O60245	PCDH7
1	PCDH9	1.3957E-46	-4.64	8.1193E-42	1.5793E-45	Q9HC56	PCDH9
1	PCDHA2	1.7196E-11	-22.64	1.0004E-06	1.0322E-10	Q9Y5H9	PCDHA2
1	PCDHA3	1.9948E-11	-14.86	1.1605E-06	1.1938E-10	Q9Y5H8	PCDHA3
1	PCDHA4	2.9697E-44	-12.14	1.7276E-39	3.2795E-43	Q9UN74	PCDHA4
1	PCDHA5	0.00052746	-12.00	1	0.00210341	Q9Y5H7	PCDHA5
1	PCDHA7	0.00143322	-9.43	1	0.00549903	Q9UN72	PCDHA7
1	PCDHB10	2.5757E-40	-8.09	1.4984E-35	2.7047E-39	Q9UN67	PCDHB10
1	PCDHB11	1.2182E-30	-4.12	7.0869E-26	1.1312E-29	Q9Y5F2	PCDHB11
1	PCDHB12	2.5426E-27	-4.17	1.4791E-22	2.2317E-26	Q9Y5F1	PCDHB12
1	PCDHB13	1.1326E-27	-2.67	6.5888E-23	1.0009E-26	Q9Y5F0	PCDHB13
1	PCDHB14	1.4937E-30	-3.52	8.6894E-26	1.3852E-29	Q9Y5E9	PCDHB14
1	PCDHB15	0.00844521	-2.63	1	0.02992214	Q9Y5E8	PCDHB15
1	PCDHB16	9.5353E-81	-17.30	5.547E-76	1.423E-79	Q9NRJ7	PCDHB16
1	PCDHB4	1.527E-09	-4.05	8.8829E-05	8.4374E-09	Q9Y5E5	PCDHB4
1	PCDHB5	4.449E-124	-9.04	2.588E-119	8.773E-123	Q9Y5E4	PCDHB5
1	PCDHB6	0.00157911	-6.37	1	0.00603569	Q9Y5E3	PCDHB6
1	PCDHB7	5.0485E-25	-9.08	2.9369E-20	4.2386E-24	Q9Y5E2	PCDHB7
1	PCDHB8	1.491E-133	-8.54	8.673E-129	3.043E-132	Q9UN66	PCDHB8
1	PCDHB9	7.1183E-16	-2.37	4.141E-11	4.9427E-15	Q9Y5E1	PCDHB9
1	PCDHGA1	1.0721E-09	-3.30	6.2368E-05	5.9665E-09	Q9Y5H4	PCDHGA1
1	PCDHGA4	0.00087738	-3.10	1	0.00343432	Q9Y5G9	PCDHGA4
1	PCDHGA6	4.0447E-05	-2.10	1	0.00017551	Q9Y5G7	PCDHGA6
1	PCDHGA9	1.0481E-05	-6.73	0.60970225	4.7278E-05	Q9Y5G4	PCDHGA9
1	PCDHGB1	0.00104555	-2.60	1	0.00406602	Q9Y5G3	PCDHGB1
1	PCDHGB2	1.2915E-09	-1.81	7.513E-05	7.1614E-09	Q9Y5G2	PCDHGB2

1	PCDHGB3	0.00082053	-2.92	1	0.0032198	Q9Y5G1	PCDHGB3
1	PCDHGB5	1.2534E-06	42.58	0.07291496	5.9879E-06	Q9Y5G0	PCDHGB5
1	PCED1B	0	-2.86	0	0	Q96HM7	PCED1B
1	PCED1B-AS1	7.1463E-09	-8.59	0.00041573	3.8323E-08		
1	PCGF5	1.0149E-71	2.40	5.9043E-67	1.4091E-70	Q86SE9	PCGF5
1	PCGF6	2.4412E-95	2.36	1.4201E-90	4.0727E-94	Q9BYE7	PCGF6
1	PCLO	0	-2.09	0	0	Q9Y6V0	PCLO
1	PCMTD2	2.3056E-32	1.58	1.3413E-27	2.1956E-31	Q9NV79	PCMTD2
1	PCNP	2.9497E-38	1.77	1.716E-33	3.0232E-37	Q8WW12	PCNP
1	PCNX3	0	-1.92	0	0	Q9H6A9	PCNX3
1	PCNX4	7.9315E-94	1.69	4.6141E-89	1.309E-92	Q63HM2	PCNX4
1	PCOLCE2	5.3946E-88	-13.41	3.1382E-83	8.5347E-87	Q9UKZ9	PCOLCE2
1	PCP4L1	1.0754E-10	-14.33	6.2562E-06	6.2425E-10		
1	PCSK4	3.0358E-08	1.60	0.00176602	1.5808E-07	Q6UW60	PCSK4
1	PCSK5	4.409E-114	13.23	2.565E-109	8.255E-113	Q92824	PCSK5
1	PCSK6	2.7939E-44	4.14	1.6253E-39	3.0858E-43	P29122	PCSK6
1	PCSK9	0	-22.14	0	0	Q8NBP7	PCSK9
1	PCTP	2.2204E-16	-1.57	1.2917E-11	1.5667E-15	Q9UKL6	PCTP
1	PCYOX1	0	-1.65	0	0	Q9UHG3	PCYOX1
1	PCYOX1L	0	-2.23	0	0	Q8NBM8	PCYOX1L
1	PCYT1A	0	-1.75	0	0	P49585	PCYT1A
1	PCYT1B	4.628E-216	-12.25	2.693E-211	1.213E-214	Q9Y5K3	PCYT1B
1	PDC	0.00221501	-25.23	1	0.00834127	P20941	PDC
1	PDCD10	0	-1.57	0	0	Q9BUL8	PDCD10
1	PDCD11	1.5104E-40	1.63	8.7868E-36	1.5904E-39	Q14690	PDCD11
1	PDCD1LG2	2.8022E-22	2.73	1.6301E-17	2.2291E-21	Q9BQ51	PDCD1LG2
1	PDCD2L	1.1298E-20	1.71	6.5726E-16	8.7239E-20	Q9BRP1	PDCD2L
1	PDCD4	0	-1.78	0	0	Q53EL6	PDCD4
1	PDCD5	4.761E-20	1.50	2.7697E-15	3.6371E-19	O14737	PDCD5
1	PDCD6	0	-2.63	0	0	O75340	PDCD6
1	PDCD6IPP2	7.9253E-07	-2.71	0.04610453	3.8271E-06		
1	PDCL2	9.8404E-08	-13.27	0.00572454	4.9831E-07	Q8N4E4	PDCL2
1	PDCL3	9.49E-57	2.24	5.5207E-52	1.1799E-55	Q9H2J4	PDCL3
1	PDCL3P4	1.4335E-06	-8.94	0.08338987	6.8207E-06		
1	PDDC1	8.4928E-52	1.74	4.9406E-47	1.0097E-50		
1	PDE10A	0	192.29	0	0	Q9Y233	PDE10A
1	PDE11A	4.6574E-32	-4.90	2.7094E-27	4.4135E-31	Q9HCR9	PDE11A
1	PDE12	6.7752E-43	1.82	3.9414E-38	7.374E-42	Q6L8Q7	PDE12
1	PDE1A	1.3692E-28	-14.57	7.9655E-24	1.2296E-27	P54750	PDE1A
1	PDE2A	5.6042E-06	1.91	0.32601853	2.5723E-05	O00408	PDE2A
1	PDE3A	2.8893E-36	2.48	1.6808E-31	2.8945E-35	Q14432	PDE3A
1	PDE3B	2.833E-119	-9.98	1.648E-114	5.463E-118	Q13370	PDE3B
1	PDE4A	4.2692E-23	-8.29	2.4835E-18	3.4522E-22	P27815	PDE4A
1	PDE4B	5.2758E-12	-4.08	3.0692E-07	3.2253E-11	Q07343	PDE4B
1	PDE4D	4.3433E-47	-3.11	2.5267E-42	4.9436E-46	Q08499	PDE4D
1	PDE5A	1.6172E-81	-3.87	9.4081E-77	2.4291E-80	O76074	PDE5A
1	PDE6A	2.0406E-40	-6.94	1.1871E-35	2.1455E-39	P16499	PDE6A

1	PDE7B	1.5563E-11	2.51	9.0537E-07	9.3588E-11	Q9NP56	PDE7B
1	PDE8B	4.763E-21	-2.81	2.7708E-16	3.7038E-20	O95263	PDE8B
1	PDE9A	2.7662E-10	-10.16	1.6092E-05	1.5786E-09	O76083	PDE9A
1	PDF	4.7064E-08	1.58	0.00273793	2.4249E-07	Q9HBH1	PDF
1	PDGFA	0	-2.53	0	0	P04085	PDGFA
1	PDGFB	1.2E-87	-3.64	6.9811E-83	1.8904E-86	P01127	PDGFB
1	PDGFC	3.4342E-26	2.88	1.9978E-21	2.9445E-25	Q9NRA1	PDGFC
1	PDGFD	2.199E-170	-13.14	1.279E-165	5.035E-169	Q9GZP0	PDGFD
1	PDGFRA	9.2172E-08	5.79	0.005362	4.6744E-07	P16234	PDGFRA
1	PDGFRB	9.3032E-09	-4.71	0.0005412	4.9647E-08	P09619	PDGFRB
1	PDGFRL	2.5256E-63	3.62	1.4693E-58	3.3025E-62	Q15198	PDGFRL
1	PDIA2	0.01012577	-8.32	1	0.03549602	Q13087	PDIA2
1	PDK1	0	-1.50	0	0	Q15118	PDK1
1	PDK2	1.4671E-16	1.64	8.5348E-12	1.0415E-15	Q15119	PDK2
1	PDK4	1.8202E-11	-7.40	1.0589E-06	1.0915E-10	Q16654	PDK4
1	PDLIM1	0	-4.54	0	0	O00151	PDLIM1
1	PDLIM2	0	-1.91	0	0	Q96JY6	PDLIM2
1	PDLIM3	5.2599E-07	-44.03	0.03059908	2.5647E-06	Q53GG5	PDLIM3
1	PDLIM4	2.6784E-14	1.87	1.5581E-09	1.77E-13	P50479	PDLIM4
1	PDLIM5	2.6239E-42	2.27	1.5264E-37	2.8352E-41	Q96HC4	PDLIM5
1	PDPN	3.2165E-10	-2.11	1.8712E-05	1.8289E-09	Q86YL7	PDPN
1	PDRG1	3.221E-104	2.66	1.874E-99	5.7E-103	Q9NUG6	PDRG1
1	PDS5B	0	-1.66	0	0	Q9NTI5	PDS5B
1	PDXDC2P_1	0	-2.42	0	0		
1	PDXK	0	-1.94	0	0	O00764	PDXK
1	PDXP	6.638E-135	19.84	3.861E-130	1.362E-133	Q96GD0	PDXP
1	PDZD11	3.4217E-17	1.54	1.9905E-12	2.4632E-16	Q5EBL8	PDZD11
1	PDZD4	3.3829E-08	5.64	0.00196796	1.7562E-07	Q76G19	PDZD4
1	PDZD7	7.6509E-10	-1.91	4.4508E-05	4.2817E-09	Q9H5P4	PDZD7
1	PDZK1IP1	1.48E-05	-2.41	0.86099004	6.6128E-05	Q13113	PDZK1IP1
1	PDZK1P1	0.00058679	-29.92	1	0.0023317		
1	PDZPH1P	0.00084229	28.40	1	0.00330116		
1	PEA15	3.682E-99	2.11	2.1417E-94	6.2955E-98	Q15121	PEA15
1	PEAR1	0	-1.75	0	0	Q5VY43	PEAR1
1	PEBP1	4.5547E-57	4.66	2.6497E-52	5.6738E-56	P30086	PEBP1
1	PEBP4	3.7532E-05	-6.59	1	0.00016328	Q96S96	PEBP4
1	PEF1	1.8971E-49	1.76	1.1036E-44	2.2095E-48	Q9UBV8	PEF1
1	PEG10	0	187.70	0	0	Q86TG7	PEG10
1	PELI2	2.0774E-11	1.96	1.2085E-06	1.2417E-10	Q9HAT8	PELI2
1	PELI3	7.3139E-15	-1.51	4.2548E-10	4.9143E-14	Q8N2H9	PELI3
1	PELO	1.87E-43	2.00	1.0879E-38	2.0511E-42	Q9BRX2	PELO
1	PEPD	1.9761E-37	1.63	1.1496E-32	2.0041E-36	P12955	PEPD
1	PER1	0	-1.89	0	0	O15534	PER1
1	PER3	1.5086E-16	1.68	8.7759E-12	1.0706E-15	P56645	PER3
1	PERP	0	-4.04	0	0	Q96FX8	PERP
1	PES1	7.4978E-48	2.43	4.3618E-43	8.6014E-47	O00541	PES1
1	PET117	7.4414E-48	2.47	4.3289E-43	8.5383E-47	Q6UWS5	PET117

1	PEX1	0	-1.57	0	0	O43933	PEX1
1	PEX10	1.9168E-98	2.71	1.1151E-93	3.2644E-97	O60683	PEX10
1	PEX11G	3.1435E-30	-4.03	1.8287E-25	2.8999E-29	Q96HA9	PEX11G
1	PEX16	1.17E-18	1.56	6.8065E-14	8.684E-18	Q9Y5Y5	PEX16
1	PEX3	9.0236E-30	1.63	5.2494E-25	8.272E-29	P56589	PEX3
1	PEX5	4.428E-121	2.12	2.576E-116	8.618E-120	P50542	PEX5
1	PEX6	0	-2.88	0	0	Q13608	PEX6
1	PFAS	3.002E-81	2.73	1.7464E-76	4.501E-80	O15067	PFAS
1	PFDN1	3.6749E-97	3.70	2.1378E-92	6.1984E-96	O60925	PFDN1
1	PFDN4	6.7262E-32	2.13	3.9129E-27	6.3614E-31	Q9NQP4	PFDN4
1	PFKFB1	5.6245E-91	12.48	3.272E-86	9.0914E-90	P16118	PFKFB1
1	PFKFB2	0	-8.32	0	0	O60825	PFKFB2
1	PFN1	2.9727E-84	2.42	1.7293E-79	4.5701E-83	P07737	PFN1
1	PGA5	0.00963605	-3.22	1	0.03386093	P0DJD9	PGA5
1	PGAM1P5	0.00270601	2.84	1	0.01010587		
1	PGAM1P8	1.4528E-18	-4.09	8.4517E-14	1.0761E-17		
1	PGAM2	1.7338E-05	48.47	1	7.7058E-05	P15259	PGAM2
1	PGAP1	4.5553E-11	-1.59	2.65E-06	2.6819E-10	Q75T13	PGAP1
1	PGAP3	0	-2.03	0	0	Q96FM1	PGAP3
1	PGBD1	2.563E-100	2.61	1.491E-95	4.411E-99	Q96JS3	PGBD1
1	PGBD4	2.9376E-34	2.09	1.7089E-29	2.875E-33		
1	PGCP1	0.01270839	7.58	1	0.0440268		
1	PGF	2.2169E-13	1.93	1.2896E-08	1.4213E-12	P49763	PGF
1	PGM1	0	-1.86	0	0	P36871	PGM1
1	PGM2	0	-1.67	0	0	Q96G03	PGM2
1	PGM3	1.946E-82	1.97	1.1321E-77	2.9474E-81	O95394	PGM3
1	PGM5	2.7656E-27	9.90	1.6088E-22	2.423E-26	Q15124	PGM5
1	PGM5P2	5.3274E-28	81.36	3.0991E-23	4.746E-27		
1	PGPEP1	4.9287E-94	2.37	2.8672E-89	8.1524E-93	Q9NXJ5	PGPEP1
1	PGR	0.00079574	-11.32	1	0.00312717	P06401	PGR
1	PHACTR1	7.1732E-28	4.32	4.1729E-23	6.3679E-27	Q9C0D0	PHACTR1
1	PHAX	7.3068E-42	1.79	4.2506E-37	7.8469E-41	Q9H814	PHAX
1	PHB	2.2288E-44	1.81	1.2966E-39	2.464E-43	P35232	PHB
1	PHB2	1.4738E-75	2.11	8.5737E-71	2.1071E-74	Q99623	PHB2
1	PHC1	3.42E-109	2.99	1.989E-104	6.224E-108	P78364	PHC1
1	PHC1P1	7.371E-16	2.76	4.288E-11	5.1151E-15		
1	PHC2	9.273E-134	1.79	5.395E-129	1.894E-132	Q8IXK0	PHC2
1	PHC3	0	-1.99	0	0	Q8NDX5	PHC3
1	PHF1	0	-1.80	0	0	O43189	PHF1
1	PHF10	7.952E-141	1.82	4.626E-136	1.669E-139	Q8WUB8	PHF10
1	PHF11	7.5078E-82	-3.10	4.3676E-77	1.1309E-80	Q9UIL8	PHF11
1	PHF13	1.145E-108	2.16	6.66E-104	2.079E-107	Q86YI8	PHF13
1	PHF14	2.5675E-88	2.81	1.4936E-83	4.072E-87	O94880	PHF14
1	PHF19	2.2234E-46	1.72	1.2934E-41	2.5115E-45	Q5T6S3	PHF19
1	PHF21B	5.192E-195	72.02	3.021E-190	1.29E-193	Q96EK2	PHF21B
1	PHF23	1.008E-99	2.14	5.8635E-95	1.7302E-98	Q9BUL5	PHF23
1	PHF24	0.00585578	5.18	1	0.02112976	Q9UPV7	PHF24

1	PHKA2	0	-2.73	0	0	P46019	PHKA2
1	PHKA2-AS1	0.00167989	-2.00	1	0.00640657		
1	PHLDA1	2.1482E-82	2.36	1.2497E-77	3.2501E-81	Q8WV24	PHLDA1
1	PHLDA2	4.9326E-28	2.07	2.8695E-23	4.3963E-27	Q53GA4	PHLDA2
1	PHLDA3	0	-9.66	0	0	Q9Y5J5	PHLDA3
1	PHLDB3	4.6366E-47	-2.13	2.6973E-42	5.2723E-46	Q6NSJ2	PHLDB3
1	PHLPP2	8.3797E-24	1.63	4.8748E-19	6.8727E-23	Q6ZVD8	PHLPP2
1	PHRF1	8.6578E-90	2.23	5.0366E-85	1.3867E-88	Q9P1Y6	PHRF1
1	PHYHD1	5.914E-151	-9.81	3.44E-146	1.277E-149	Q5SRE7	PHYHD1
1	PHYHIP	4.5862E-10	-2.53	2.668E-05	2.5908E-09		
1	PI15	5.4662E-27	-4.05	3.1799E-22	4.7553E-26	O43692	PI15
1	PI16	8.3302E-07	-11.83	0.04846008	4.0179E-06	Q6UXB8	PI16
1	PI3	0.00218966	-3.70	1	0.00825381	P19957	PI3
1	PI4KAP1	1.312E-107	9.18	7.631E-103	2.364E-106	Q8N8J0	PI4KAP1
1	PIANP	0.00067129	1.95	1	0.00265296	Q8IYJ0	PIANP
1	PIAS1	0	-1.56	0	0	O75925	PIAS1
1	PICSAR	3.1473E-40	2.25	1.8309E-35	3.3019E-39		
1	PID1	1.011E-106	38.13	5.881E-102	1.814E-105	Q7Z2X4	PID1
1	PIDD1	2.171E-25	1.60	1.2629E-20	1.8351E-24	Q9HB75	PIDD1
1	PIEZO1	1.9984E-15	-1.56	1.1626E-10	1.3688E-14	Q92508	PIEZO1
1	PIGB	0	-2.14	0	0	Q92521	PIGB
1	PIGC	0	-1.84	0	0	Q92535	PIGC
1	PIGG	0	-1.78	0	0	Q5H8A4	PIGG
1	PIGH	6.093E-70	3.25	3.5445E-65	8.3479E-69	Q14442	PIGH
1	PIGL	1.0895E-22	1.56	6.3383E-18	8.7557E-22	Q9Y2B2	PIGL
1	PIGM	0	-1.62	0	0	Q9H3S5	PIGM
1	PIGR	1.3025E-12	-35.76	7.5772E-08	8.1318E-12	P01833	PIGR
1	PIGS	0	-2.09	0	0	Q96S52	PIGS
1	PIGV	0	-2.58	0	0	Q9NUD9	PIGV
1	PIGX	0	-1.88	0	0	Q8TBF5	PIGX
1	PIGZ	3.315E-123	-4.19	1.929E-118	6.516E-122	Q86VD9	PIGZ
1	PIK3C2B	2.6024E-94	-3.72	1.5139E-89	4.3108E-93	O00750	PIK3C2B
1	PIK3C2G	2.662E-23	-11.41	1.5486E-18	2.1613E-22	O75747	PIK3C2G
1	PIK3CB	0	-2.09	0	0	P42338	PIK3CB
1	PIK3CD	1.176E-109	3.19	6.84E-105	2.149E-108	O00329	PIK3CD
1	PIK3CD-AS2	3.6314E-11	3.60	2.1125E-06	2.1475E-10		
1	PIK3CG	3.2519E-12	-5.09	1.8917E-07	2.0048E-11	P48736	PIK3CG
1	PIK3IP1	1.7833E-69	-2.76	1.0374E-64	2.433E-68	Q96FE7	PIK3IP1
1	PIK3IP1-AS1	4.7407E-11	-2.91	2.7578E-06	2.7891E-10		
1	PIK3R1	5.2846E-24	1.61	3.0743E-19	4.3483E-23	P27986	PIK3R1
1	PIK3R4	0	-2.02	0	0	Q99570	PIK3R4
1	PIK3R5	0.00077339	27.93	1	0.00304263	Q8WYR1	PIK3R5
1	PIK3R6	0.00117013	12.86	1	0.00453113	Q5UE93	PIK3R6
1	PILRB	0	-2.29	0	0	Q9UKJ0	PILRB
1	PIM1	0	-5.06	0	0	P11309	PIM1
1	PIN1	8.7395E-48	1.77	5.0841E-43	1.002E-46	Q13526	PIN1
1	PINLYP	7.1295E-30	20.61	4.1475E-25	6.547E-29	A6NC86	PINLYP

1	PIP4K2C	0	-2.03	0	0	Q8TBX8	PIP4K2C
1	PIP5K1C	3.8829E-54	1.75	2.2589E-49	4.7346E-53	O60331	PIP5K1C
1	PIP5KL1	1.586E-265	-8.26	9.224E-261	4.397E-264	Q5T9C9	PIP5KL1
1	PIPOX	1.617E-06	-2.60	0.09406681	7.6727E-06	Q9P0Z9	PIPOX
1	PIPSL	0.00048366	-2.32	1	0.00193472	A2A3N6	PIPSL
1	PIR	0	-5.38	0	0	O00625	PIR
1	PITPNC1	8.3861E-61	2.42	4.8785E-56	1.0741E-59	Q9UKF7	PITPNC1
1	PITPNM1	0	-2.67	0	0	O00562	PITPNM1
1	PITPNM2	0	-2.39	0	0	Q9BZ72	PITPNM2
1	PITX2	3.79E-233	-9.42	2.205E-228	1.019E-231	Q99697	PITX2
1	PITX3	2.9699E-07	-17.68	0.01727739	1.4658E-06	O75364	PITX3
1	PIWIL4	0.00386353	-2.13	1	0.01422514	Q7Z3Z4	PIWIL4
1	PKD1	2.5301E-17	1.86	1.4719E-12	1.8277E-16	P98161	PKD1
1	PKD1L2	2.968E-167	18.05	1.726E-162	6.738E-166	Q7Z442	PKD1L2
1	PKD1L3	0.00609812	-2.25	1	0.02195111	Q7Z443	PKD1L3
1	PKD1P1	2.0702E-17	-34.81	1.2043E-12	1.4977E-16		
1	PKD1P5	1.2588E-13	3.08	7.3231E-09	8.1377E-13		
1	PKD1P6	9.8922E-48	3.26	5.7547E-43	1.1337E-46		
1	PKD1P6-NPIPI	2.4982E-19	2.89	1.4533E-14	1.8789E-18		
1	PKD2	0	-1.80	0	0	Q13563	PKD2
1	PKHD1	1.1657E-23	-5.12	6.7813E-19	9.531E-23	P08F94	PKHD1
1	PKHD1L1	7.5903E-10	-25.48	4.4156E-05	4.2486E-09	Q86WI1	PKHD1L1
1	PKIA	6.166E-103	4.26	3.5871E-98	1.082E-101	P61925	PKIA
1	PKIA-AS1	0.00566932	-2.23	1	0.02051039		
1	PKIB	4.0653E-76	-5.55	2.3649E-71	5.8436E-75	Q9C010	PKIB
1	PKIG	2.7248E-98	76.31	1.5851E-93	4.6335E-97	Q9Y2B9	PKIG
1	PKN1	2.8959E-43	3.27	1.6846E-38	3.1666E-42	Q16512	PKN1
1	PKN3	9.1294E-44	1.68	5.311E-39	1.0047E-42	Q6P5Z2	PKN3
1	PKP1	4.642E-136	-8.07	2.701E-131	9.58E-135	Q13835	PKP1
1	PKP2	0	-1.91	0	0	Q99959	PKP2
1	PLA2G10	1.2086E-19	-4.88	7.0311E-15	9.1503E-19	O15496	PLA2G10
1	PLA2G12A	0	-1.87	0	0	Q9BZM1	PLA2G12A
1	PLA2G16	6.1782E-68	13.66	3.5941E-63	8.339E-67	P53816	PLA2G16
1	PLA2G4A	1.081E-176	-8.33	6.289E-172	2.527E-175	P47712	PLA2G4A
1	PLA2G4B	0.00131232	-2.56	1	0.00505902	P0C869	PLA2G4B
1	PLA2G4C	0.00083322	-1.81	1	0.00326675	Q9UP65	PLA2G4C
1	PLA2G4D	1.0259E-22	-7.94	5.9683E-18	8.2504E-22	Q86XP0	PLA2G4D
1	PLA2G4E	0.00020321	-3.40	1	0.00083877	Q3MJ16	PLA2G4E
1	PLA2G4E-AS1	2.8E-12	-7.64	1.6289E-07	1.7303E-11		
1	PLA2G4F	7.23E-205	-10.58	4.206E-200	1.846E-203	Q68DD2	PLA2G4F
1	PLA2G6	1.7527E-42	-2.80	1.0196E-37	1.8963E-41	O60733	PLA2G6
1	PLA2G7	3.1043E-20	-6.41	1.8059E-15	2.378E-19	Q13093	PLA2G7
1	PLA2R1	4.683E-110	-9.67	2.724E-105	8.574E-109	Q13018	PLA2R1
1	PLAA	5.0875E-70	1.75	2.9596E-65	6.9752E-69	Q9Y263	PLAA
1	PLAC1	0.01450999	-1.69	1	0.04999728	Q9HBJ0	PLAC1
1	PLAC8	1.7666E-22	-5.43	1.0277E-17	1.4121E-21	Q9NZF1	PLAC8
1	PLAC8L1	1.3512E-06	1.86	0.07860595	6.441E-06		

1	PLAC9	2.2474E-24	-7.71	1.3074E-19	1.8645E-23	Q5JTB6	PLAC9
1	PLAGL1	1.472E-256	-11.79	8.564E-252	4.042E-255	Q9UM63	PLAGL1
1	PLAGL2	4.6165E-52	1.74	2.6856E-47	5.5112E-51	Q9UPG8	PLAGL2
1	PLAT	5.5358E-74	7.92	3.2204E-69	7.8184E-73	P00750	PLAT
1	PLAU	0	-1.65	0	0	P00749	PLAU
1	PLAUR	4.6717E-78	4.18	2.7177E-73	6.8113E-77	Q03405	PLAUR
1	PLB1	1.0555E-57	-5.44	6.14E-53	1.3213E-56	Q6P1J6	PLB1
1	PLBD1	0	-10.93	0	0	Q6P4A8	PLBD1
1	PLBD1-AS1	3.9932E-29	-12.87	2.323E-24	3.619E-28		
1	PLCB2	1.3519E-05	-2.93	0.78644322	6.0542E-05	Q00722	PLCB2
1	PLCB3	0	-2.71	0	0	Q01970	PLCB3
1	PLCB4	4.9996E-07	8.94	0.02908454	2.4408E-06	Q15147	PLCB4
1	PLCD1	1.6001E-78	-2.78	9.3085E-74	2.3418E-77	P51178	PLCD1
1	PLCD3	0	-3.31	0	0	Q8N3E9	PLCD3
1	PLCD4	1.7797E-12	1.58	1.0353E-07	1.1069E-11	Q9BRC7	PLCD4
1	PLCE1	1.1501E-15	1.65	6.6906E-11	7.9216E-15	Q9P212	PLCE1
1	PLCE1-AS1	4.9973E-12	103.32	2.9071E-07	3.0579E-11		
1	PLCG1	1.7655E-62	2.77	1.027E-57	2.294E-61	P19174	PLCG1
1	PLCG1-AS1	4.6209E-10	3.31	2.6882E-05	2.6101E-09		
1	PLCG2	6.2284E-33	3.48	3.6233E-28	5.985E-32	P16885	PLCG2
1	PLCH1	9.513E-176	-7.17	5.534E-171	2.217E-174	Q4KWH8	PLCH1
1	PLCL1	1.6545E-22	-6.26	9.6247E-18	1.3243E-21	Q15111	PLCL1
1	PLCL2	9.353E-179	6.30	5.441E-174	2.199E-177	Q9UPR0	PLCL2
1	PLCXD1	0	-1.69	0	0	Q9NUJ7	PLCXD1
1	PLCZ1	6.5655E-10	-5.98	3.8194E-05	3.6867E-09	Q86YW0	PLCZ1
1	PLD1	0	-1.67	0	0	Q13393	PLD1
1	PLD3	0	-2.36	0	0	Q8IV08	PLD3
1	PLD6	1.0273E-13	1.69	5.9764E-09	6.6612E-13	Q8N2A8	PLD6
1	PLEC	0	-1.88	0	0	Q15149	PLEC
1	PLEK2	1.6741E-23	1.55	9.7387E-19	1.3647E-22	Q9NYT0	PLEK2
1	PLEKHA1	0	-1.98	0	0	Q9HB21	PLEKHA1
1	PLEKHA2	0	-2.18	0	0	Q9HB19	PLEKHA2
1	PLEKHA3	3.0352E-33	2.09	1.7657E-28	2.9253E-32	Q9HB20	PLEKHA3
1	PLEKHA4	1.043E-174	-10.57	6.068E-170	2.423E-173	Q9H4M7	PLEKHA4
1	PLEKHA6	2.095E-168	-10.45	1.219E-163	4.775E-167		
1	PLEKHA8	0	-1.63	0	0	Q96JA3	PLEKHA8
1	PLEKHA8P1	1.3483E-10	-1.56	7.8434E-06	7.802E-10	O95397	PLEKHA8P1
1	PLEKHB1	2.178E-65	-5.35	1.267E-60	2.8967E-64	Q9UF11	PLEKHB1
1	PLEKHD1	1.4723E-10	6.43	8.5651E-06	8.5047E-10		
1	PLEKHF1	3.5021E-16	2.28	2.0373E-11	2.4599E-15	Q96S99	PLEKHF1
1	PLEKHG1	0.00013174	-1.63	1	0.00055095	Q9ULL1	PLEKHG1
1	PLEKHG2	3.0204E-27	1.69	1.7571E-22	2.6438E-26	Q9H7P9	PLEKHG2
1	PLEKHG4B	4.356E-243	-12.09	2.534E-238	1.185E-241	Q96PX9	PLEKHG4B
1	PLEKHG6	1.756E-181	-10.02	1.021E-176	4.161E-180	Q3KR16	PLEKHG6
1	PLEKHH2	5.0346E-50	-3.43	2.9288E-45	5.8966E-49	Q8IVE3	PLEKHH2
1	PLEKHH3	0	-1.70	0	0	Q7Z736	PLEKHH3
1	PLEKHJ1	4.5655E-27	1.52	2.656E-22	3.9796E-26	Q9NW61	PLEKHJ1

1	PLEKHO2	3.41E-36	1.74	1.9837E-31	3.4132E-35	Q8TD55	PLEKHO2
1	PLGLA	0.00025632	-34.27	1	0.001048	Q15195	PLGLA
1	PLGLB1	1.7327E-05	-2.06	1	7.7015E-05	Q02325	PLGLB1
1	PLGLB2	0.00036927	-2.37	1	0.00149107		
1	PLIN2	1.3444E-70	9.88	7.8207E-66	1.8528E-69	Q99541	PLIN2
1	PLIN4	2.1035E-52	-7.57	1.2237E-47	2.5184E-51	Q96Q06	PLIN4
1	PLIN5	3.4088E-06	-5.36	0.19830187	1.5829E-05	Q00G26	PLIN5
1	PLK2	6.9501E-31	1.57	4.0432E-26	6.4774E-30	Q9NYY3	PLK2
1	PLK4	0	-1.51	0	0	O00444	PLK4
1	PLLP	1.4895E-17	-1.94	8.6648E-13	1.0812E-16	Q9Y342	PLLP
1	PLPP1	1.34E-19	1.57	7.7955E-15	1.0135E-18	O14494	PLPP1
1	PLPP2	0	-2.10	0	0	O43688	PLPP2
1	PLPP3	1.4729E-26	2.20	8.5686E-22	1.2706E-25	O14495	PLPP3
1	PLPP5	0	-1.58	0	0	Q8NEB5	PLPP5
1	PLPP6	0	-2.64	0	0	Q8IY26	PLPP6
1	PLPPR1	2.6397E-05	-6.14	1	0.00011598	Q8TBJ4	PLPPR1
1	PLPPR2	6.5745E-78	6.37	3.8247E-73	9.5641E-77	Q96GM1	PLPPR2
1	PLPPR4	5.4731E-05	-13.10	1	0.00023537	Q7Z2D5	PLPPR4
1	PLS1	0	-3.00	0	0	Q14651	PLS1
1	PLS3	1.9117E-27	1.66	1.1121E-22	1.6822E-26	P13797	PLS3
1	PLS3-AS1	2.3626E-06	-1.98	0.13743995	1.1097E-05		
1	PLSCR1	0	-2.46	0	0	O15162	PLSCR1
1	PLSCR2	1.39E-28	-14.58	8.0864E-24	1.2481E-27	Q9NRY7	PLSCR2
1	PLSCR4	1.213E-114	-4.56	7.057E-110	2.279E-113	Q9NRQ2	PLSCR4
1	PLTP	0	-13.81	0	0	P55058	PLTP
1	PLXDC1	4.3574E-16	-6.00	2.5349E-11	3.0537E-15	Q8IUK5	PLXDC1
1	PLXDC2	9.5786E-06	3.57	0.55722813	4.3324E-05	Q6UX71	PLXDC2
1	PLXNA1	0	-1.66	0	0	Q9UIW2	PLXNA1
1	PLXNA2	0	-5.50	0	0	O75051	PLXNA2
1	PLXNB1	0	-2.00	0	0	O43157	PLXNB1
1	PLXNB2	0	-2.05	0	0	O15031	PLXNB2
1	PLXNB3	4.7065E-14	1.67	2.738E-09	3.0857E-13	Q9ULL4	PLXNB3
1	PLXNC1	2.2689E-07	-1.55	0.01319913	1.1273E-06	O60486	PLXNC1
1	PM20D1	0.00140721	-3.50	1	0.00540559	Q6GTS8	PM20D1
1	PM20D2	2.5195E-32	1.54	1.4657E-27	2.3976E-31	Q8IYS1	PM20D2
1	PMAIP1	1.79E-177	8.98	1.041E-172	4.193E-176	Q13794	PMAIP1
1	PMEL	9.0304E-95	-7.08	5.2533E-90	1.5001E-93	P40967	PMEL
1	PMF1	0	-1.63	0	0	Q6P1K2	PMF1
1	PMF1-BGLAP	0.00202519	-1.56	1	0.00765468	U3KQ54	PMF1-BGLAP
1	PMFBP1	1.2762E-18	-3.90	7.4241E-14	9.4647E-18	Q8TBY8	PMFBP1
1	PMM2	6.1644E-70	2.17	3.5861E-65	8.4437E-69	O15305	PMM2
1	PMP22	3.6E-162	15.44	2.094E-157	8.038E-161	Q01453	PMP22
1	PMS2P2	4.1321E-05	4.56	1	0.00017913	O95744	PMS2P2
1	PMS2P4	0.00045461	1.56	1	0.00182227		
1	PMS2P6_1	5.0821E-11	-1.96	2.9565E-06	2.9884E-10		
1	PMS2P6_2	0.01204964	-2.80	1	0.04187929		
1	PMS2P9	0.00942764	-19.44	1	0.03316063		

1	PMVK	8.586E-48	1.91	4.9948E-43	9.846E-47	Q15126	PMVK
1	PNCK	0.00127154	-2.94	1	0.0049088	Q6P2M8	PNCK
1	PNISR	0	-1.58	0	0	Q8TF01	PNISR
1	PNLDC1	1.1457E-06	4.14	0.06665096	5.4843E-06	Q8NA58	PNLDC1
1	PNMA1	1.6774E-38	3.60	9.7583E-34	1.7256E-37	Q8ND90	PNMA1
1	PNN	1.0459E-52	1.57	6.0845E-48	1.2569E-51	Q9H307	PNN
1	PNO1	3.838E-42	1.92	2.2327E-37	4.1354E-41	Q9NRX1	PNO1
1	PNP	1.793E-29	1.68	1.0431E-24	1.6347E-28	P00491	PNP
1	PNPLA2	2.643E-86	2.39	1.5375E-81	4.1232E-85	Q96AD5	PNPLA2
1	PNPLA4	3.8943E-11	-1.53	2.2654E-06	2.299E-10	P41247	PNPLA4
1	PNPLA8	0	-1.83	0	0	Q9NP80	PNPLA8
1	POC1B-AS1	3.8984E-06	1.56	0.22678534	1.804E-05		
1	PODNL1	1.0932E-05	-1.74	0.63594573	4.9252E-05	Q6PEZ8	PODNL1
1	PODXL	0	-1.73	0	0	O00592	PODXL
1	POF1B	7.461E-197	-13.92	4.34E-192	1.865E-195	Q8WVV4	POF1B
1	POFUT1	1.4289E-60	1.60	8.3123E-56	1.8277E-59	Q9H488	POFUT1
1	POFUT2	9.0319E-25	1.75	5.2542E-20	7.5524E-24	Q9Y2G5	POFUT2
1	POGLUT1	6.0191E-38	1.63	3.5016E-33	6.1464E-37	Q8NBL1	POGLUT1
1	POGZ	0	-1.52	0	0	Q7Z3K3	POGZ
1	POLD4	0	-2.95	0	0	Q9HCU8	POLD4
1	POLDIP2	3.9813E-39	1.58	2.3161E-34	4.1263E-38	Q9Y2S7	POLDIP2
1	POLE3	1.467E-44	1.72	8.5339E-40	1.624E-43	Q9NRF9	POLE3
1	POLI	9.5916E-32	1.78	5.5798E-27	9.0449E-31	Q9UNA4	POLI
1	POLM	1.7366E-73	2.00	1.0102E-68	2.4426E-72	Q9NP87	POLM
1	POLN	6.1365E-12	1.91	3.5699E-07	3.7424E-11	Q7Z5Q5	POLN
1	POLR1B	6.5411E-42	1.59	3.8052E-37	7.0272E-41	Q9H9Y6	POLR1B
1	POLR1E	2.0286E-41	3.41	1.1801E-36	2.1634E-40	Q9GZS1	POLR1E
1	POLR2F	1.4167E-33	1.62	8.2417E-29	1.3741E-32	P61218	POLR2F
1	POLR2I	1.0077E-47	2.09	5.8625E-43	1.1545E-46	P36954	POLR2I
1	POLR2J2_1	1.2044E-08	-1.52	0.00070063	6.3938E-08		
1	POLR2J3	0	-3.41	0	0	Q9H1A7	POLR2J3
1	POLR2J4_1	0	-2.34	0	0		
1	POLR2L	1.3918E-96	2.88	8.0969E-92	2.3374E-95	P62875	POLR2L
1	POLR3A	5.2444E-81	2.45	3.0509E-76	7.8469E-80	O14802	POLR3A
1	POLR3B	1.1708E-33	1.70	6.8112E-29	1.1367E-32	Q9NW08	POLR3B
1	POLR3C	4.5116E-76	2.31	2.6246E-71	6.4821E-75	Q9BUI4	POLR3C
1	POLR3G	4.049E-132	3.78	2.356E-127	8.222E-131	O15318	POLR3G
1	POLR3H	1.2186E-77	1.89	7.0891E-73	1.7692E-76	Q9Y535	POLR3H
1	POLR3K	2.4723E-51	2.20	1.4382E-46	2.9274E-50	Q9Y2Y1	POLR3K
1	POLRMT	5.7734E-44	1.57	3.3586E-39	6.3646E-43	O00411	POLRMT
1	POLRMTP1	6.7139E-29	3.69	3.9058E-24	6.0658E-28		
1	POM121B	1.2531E-21	21.46	7.2899E-17	9.8632E-21	A6NF01	POM121B
1	POM121L4P	1.1777E-08	11.11	0.00068509	6.2554E-08		
1	POMGNT2	3.3044E-62	5.67	1.9223E-57	4.2841E-61	Q8NAT1	POMGNT2
1	POMT2	4.333E-110	2.68	2.52E-105	7.938E-109	Q9UKY4	POMT2
1	PON3	1.0074E-53	-11.04	5.8607E-49	1.2243E-52	Q15166	PON3
1	POP4	1.9979E-34	1.95	1.1623E-29	1.9586E-33	O95707	POP4

1	POP5	1.8806E-21	1.56	1.094E-16	1.475E-20	Q969H6	POP5
1	POPDC3	9.7408E-33	1.96	5.6666E-28	9.3308E-32	Q9HBV1	POPDC3
1	PORCN	0	-2.36	0	0	Q9H237	PORCN
1	POSTN	3.9426E-67	27.00	2.2936E-62	5.2982E-66	Q15063	POSTN
1	POT1-AS1	8.803E-114	-7.97	5.121E-109	1.646E-112		
1	POU2F1	0	-1.88	0	0	P14859	POU2F1
1	POU2F3	1.5551E-11	-9.12	9.0468E-07	9.3527E-11	Q9UKI9	POU2F3
1	POU3F2	6.6797E-20	7.28	3.8859E-15	5.0849E-19	P20265	POU3F2
1	POU3F4	0.00016587	-7.04	1	0.00068868	P49335	POU3F4
1	POU4F1	8.3803E-38	-8.71	4.8752E-33	8.5365E-37	Q01851	POU4F1
1	POU5F1	2.1394E-80	9.13	1.2446E-75	3.1888E-79	Q01860	POU5F1
1	POU5F1B	2.0246E-08	-5.74	0.00117782	1.0642E-07	Q06416	POU5F1B
1	POU6F1	4.1636E-23	2.00	2.4222E-18	3.3679E-22	Q14863	POU6F1
1	POU6F2	3.432E-170	-10.28	1.996E-165	7.854E-169	P78424	POU6F2
1	POU6F2-AS2	5.6914E-15	-27.04	3.3109E-10	3.8356E-14		
1	PP14571	8.3963E-14	-6.65	4.8845E-09	5.4599E-13		
1	PP7080	0	-1.67	0	0		
1	PPA1	9.56E-131	2.83	5.561E-126	1.932E-129	Q15181	PPA1
1	PPA2	0	-1.53	0	0	Q9H2U2	PPA2
1	PPAN	5.8756E-48	1.82	3.418E-43	6.7524E-47	Q9NQ55	PPAN
1	PPARA	4.8865E-53	2.14	2.8427E-48	5.8915E-52	Q07869	PPARA
1	PPARG	3.8594E-54	8.34	2.2452E-49	4.7069E-53	P37231	PPARG
1	PPDPF	1.0541E-61	2.32	6.1323E-57	1.3606E-60	Q9H3Y8	PPDPF
1	PPFIA1	0	-2.06	0	0	Q13136	PPFIA1
1	PPFIA2	0.00137361	-4.39	1	0.00528668	O75334	PPFIA2
1	PPFIA3	1.4052E-93	2.35	8.1748E-89	2.3145E-92	O75145	PPFIA3
1	PPFIA4	4.939E-111	-8.20	2.873E-106	9.113E-110	O75335	PPFIA4
1	PPFIBP1	3.5755E-48	1.59	2.08E-43	4.1204E-47	Q86W92	PPFIBP1
1	PPIC	1.3773E-41	2.24	8.0126E-37	1.4718E-40	P45877	PPIC
1	PPIEL	1.8295E-24	-5.63	1.0643E-19	1.52E-23		
1	PPIF	1.8414E-33	2.91	1.0712E-28	1.7815E-32	P30405	PPIF
1	PPIL4	1.0491E-40	1.66	6.1029E-36	1.1068E-39	Q8WUA2	PPIL4
1	PPIL6	4.6148E-08	-1.69	0.00268463	2.3787E-07	Q8IXY8	PPIL6
1	PPL	0	-10.60	0	0	O60437	PPL
1	PPM1A	2.0655E-26	1.56	1.2016E-21	1.7785E-25	P35813	PPM1A
1	PPM1AP1	0.00545604	9.02	1	0.01976212		
1	PPM1D	2.3533E-39	2.06	1.369E-34	2.4451E-38	O15297	PPM1D
1	PPM1F	6.7716E-69	1.73	3.9393E-64	9.1954E-68	P49593	PPM1F
1	PPM1H	1.562E-29	-2.39	9.0866E-25	1.4254E-28	Q9ULR3	PPM1H
1	PPM1J	1.416E-124	-4.15	8.236E-120	2.797E-123	Q5JR12	PPM1J
1	PPM1K	1.224E-21	1.75	7.1206E-17	9.6355E-21	Q8N3J5	PPM1K
1	PPM1L	1.0692E-11	1.56	6.2199E-07	6.4669E-11	Q5SGD2	PPM1L
1	PPM1M	0	-1.95	0	0	Q96MI6	PPM1M
1	PPP1CA	0	-1.61	0	0	P62136	PPP1CA
1	PPP1R10	1.1345E-40	1.57	6.5996E-36	1.196E-39	Q96QC0	PPP1R10
1	PPP1R12B	0	-2.52	0	0	O60237	PPP1R12B
1	PPP1R13B	1.353E-102	2.40	7.8711E-98	2.367E-101	Q96KQ4	PPP1R13B

1	PPP1R13L	0	-2.02	0	0	Q8WUF5	PPP1R13L
1	PPP1R14B	0	-1.54	0	0	Q96C90	PPP1R14B
1	PPP1R14C	4.5538E-07	5.06	0.02649144	2.2273E-06	Q8TAE6	PPP1R14C
1	PPP1R15A	1.4609E-60	2.27	8.4986E-56	1.8682E-59	O75807	PPP1R15A
1	PPP1R18	6.139E-153	1.72	3.571E-148	1.331E-151	Q6NYC8	PPP1R18
1	PPP1R1A	0.00784933	17.61	1	0.02792826	Q13522	PPP1R1A
1	PPP1R1C	6.0613E-16	-4.29	3.5261E-11	4.2234E-15	Q8WVI7	PPP1R1C
1	PPP1R26-AS1	2.4215E-20	1.83	1.4087E-15	1.8567E-19		
1	PPP1R32	5.4838E-09	-1.95	0.00031901	2.956E-08	Q7Z5V6	PPP1R32
1	PPP1R35	0	-1.77	0	0	Q8TAP8	PPP1R35
1	PPP1R36	0.00678844	-1.55	1	0.02429919	Q96LQ0	PPP1R36
1	PPP1R3B	1.0276E-87	6.62	5.9777E-83	1.6195E-86	Q86XI6	PPP1R3B
1	PPP1R3C	1.0899E-22	2.64	6.3405E-18	8.7576E-22	Q9UQK1	PPP1R3C
1	PPP1R3F	8.0887E-59	-2.71	4.7055E-54	1.0229E-57	Q6ZSY5	PPP1R3F
1	PPP1R3G	4.4484E-73	-6.37	2.5878E-68	6.2388E-72		
1	PPP1R9A	1.4963E-67	6.75	8.7045E-63	2.0159E-66	Q9ULJ8	PPP1R9A
1	PPP2CA	4.6729E-41	1.72	2.7184E-36	4.9525E-40	P67775	PPP2CA
1	PPP2CB	0	-1.52	0	0	P62714	PPP2CB
1	PPP2R2A	0	-1.93	0	0	P63151	PPP2R2A
1	PPP2R3A	1.5493E-27	1.67	9.0131E-23	1.3654E-26	Q06190	PPP2R3A
1	PPP2R3B	0	-1.50	0	0	Q9Y5P8	PPP2R3B
1	PPP2R5A	0	-2.27	0	0	Q15172	PPP2R5A
1	PPP2R5B	2.1647E-23	1.76	1.2593E-18	1.7615E-22	Q15173	PPP2R5B
1	PPP2R5C	6.7623E-64	1.86	3.9339E-59	8.8881E-63	Q13362	PPP2R5C
1	PPP2R5E	8.6145E-49	1.82	5.0114E-44	9.9669E-48	Q16537	PPP2R5E
1	PPP4R1-AS1	5.7145E-05	-2.56	1	0.00024554		
1	PPP4R3A	8.5465E-63	1.64	4.9719E-58	1.1133E-61	Q6IN85	PPP4R3A
1	PPP4R4	2.752E-19	-2.24	1.601E-14	2.0682E-18	Q6NUP7	PPP4R4
1	PPP6R1	1.6128E-64	1.54	9.3824E-60	2.129E-63	Q9UPN7	PPP6R1
1	PPP6R3	0	-2.27	0	0	Q5H9R7	PPP6R3
1	PPRC1	3.2223E-46	1.59	1.8745E-41	3.6356E-45	Q5VV67	PPRC1
1	PQLC1	4.0747E-30	1.60	2.3704E-25	3.7524E-29	Q8N2U9	PQLC1
1	PQLC2	1.1175E-42	1.95	6.5012E-38	1.212E-41	Q6ZP29	PQLC2
1	PRAME	4.289E-170	-12.47	2.495E-165	9.813E-169	P78395	PRAME
1	PRB1	8.8185E-05	-4.30	1	0.00037364	P04280	PRB1
1	PRB2	2.1229E-06	-2.86	0.12349844	1.0008E-05	P02812	PRB2
1	PRB3	1.7727E-07	-3.84	0.01031239	8.8579E-07	Q04118	PRB3
1	PRC1-AS1	0.00496161	-3.38	1	0.01805333		
1	PRCD	5.1497E-07	-3.25	0.02995759	2.5122E-06	Q00LT1	PRCD
1	PRCP	0	-1.50	0	0	P42785	PRCP
1	PRDM1	1.0274E-28	-8.38	5.9765E-24	9.2559E-28	O75626	PRDM1
1	PRDM13	3.2306E-05	-3.97	1	0.00014115	Q9H4Q3	PRDM13
1	PRDM5	6.8545E-91	-6.70	3.9875E-86	1.107E-89	Q9NQX1	PRDM5
1	PRDM6	1.4791E-11	-3.68	8.6046E-07	8.9047E-11	Q9NQX0	PRDM6
1	PRDM8	4.1846E-09	-1.79	0.00024344	2.2671E-08	Q9NQV8	PRDM8
1	PRDX2	8.1467E-18	1.53	4.7392E-13	5.9441E-17	P32119	PRDX2
1	PRDX5	0	-2.43	0	0	P30044	PRDX5

1	PRELID1	4.6796E-95	2.53	2.7223E-90	7.7935E-94	Q9Y255	PRELID1
1	PRELID3B	1.7262E-57	2.16	1.0042E-52	2.1573E-56	Q9Y3B1	PRELID3B
1	PREX1	6.2753E-62	10.45	3.6506E-57	8.1196E-61	Q8TCU6	PREX1
1	PRICKLE1	4.7294E-96	-2.63	2.7513E-91	7.9151E-95	Q96MT3	PRICKLE1
1	PRICKLE3	0	-2.50	0	0	O43900	PRICKLE3
1	PRICKLE4	3.4347E-13	-3.15	1.9981E-08	2.1863E-12	Q2TBC4	PRICKLE4
1	PRIMPOL	0	-3.21	0	0	Q96LW4	PRIMPOL
1	PRKAA1	0	-1.97	0	0	Q13131	PRKAA1
1	PRKAA2	3.901E-153	18.33	2.269E-148	8.468E-152	P54646	PRKAA2
1	PRKAB1	5.0963E-89	1.96	2.9647E-84	8.1226E-88	Q9Y478	PRKAB1
1	PRKAB2	0	-2.80	0	0	O43741	PRKAB2
1	PRKAG2	9.6141E-97	4.39	5.5929E-92	1.616E-95	Q9UGJ0	PRKAG2
1	PRKAG3	1.023E-14	-23.08	5.9513E-10	6.8453E-14	Q9UGI9	PRKAG3
1	PRKAR2A	3.5132E-44	1.56	2.0438E-39	3.8782E-43	P13861	PRKAR2A
1	PRKAR2A-AS1	9.308E-09	-1.87	0.00054148	4.9668E-08		
1	PRKCA	2.2618E-63	4.02	1.3158E-58	2.9595E-62	P17252	PRKCA
1	PRKCD	0	-3.20	0	0	Q05655	PRKCD
1	PRKCDBP	1.19E-103	4.58	6.922E-99	2.096E-102		
1	PRKCG	3.055E-210	165.24	1.777E-205	7.902E-209	P05129	PRKCG
1	PRKCH	0	-4.27	0	0	P24723	PRKCH
1	PRKCI	0	-1.91	0	0	P41743	PRKCI
1	PRKCQ-AS1	1.4319E-16	3.81	8.3299E-12	1.0172E-15		
1	PRKD1	2.0583E-46	1.92	1.1974E-41	2.3255E-45	Q15139	PRKD1
1	PRKD2	0	-1.60	0	0	Q9BZL6	PRKD2
1	PRKG1	2.386E-17	-3.31	1.388E-12	1.7245E-16	Q13976	PRKG1
1	PRKG2	3.6092E-19	-3.70	2.0996E-14	2.7071E-18	Q13237	PRKG2
1	PRKXP1	8.9562E-09	-2.85	0.00052102	4.784E-08		
1	PRMT2	0	-1.59	0	0	P55345	PRMT2
1	PRMT5	3.072E-115	2.48	1.787E-110	5.792E-114	O14744	PRMT5
1	PRMT6	2.9029E-52	1.78	1.6887E-47	3.4719E-51	Q96LA8	PRMT6
1	PRMT8	0.008403	-4.87	1	0.02978347	Q9NR22	PRMT8
1	PROB1	6.9444E-14	1.62	4.0398E-09	4.5279E-13		
1	PROC	2.9798E-23	-2.95	1.7335E-18	2.4187E-22	P04070	PROC
1	PROCA1	2.295E-192	-3.96	1.335E-187	5.641E-191	Q8NCQ7	PROCA1
1	PROCR	2.4647E-23	1.57	1.4338E-18	2.0031E-22	Q9UNN8	PROCR
1	PRODH	3.4409E-12	-4.85	2.0017E-07	2.1193E-11	O43272	PRODH
1	PROM2	1.661E-184	-23.59	9.661E-180	3.963E-183	Q8N271	PROM2
1	PRORSD1P	2.7393E-06	-4.92	0.15935492	1.2811E-05	A6NEY8	PRORSD1P
1	PROS1	0	-3.90	0	0	P07225	PROS1
1	PROSER2	0	-4.30	0	0	Q86WR7	PROSER2
1	PROSER2-AS1	1.1915E-81	-8.03	6.9315E-77	1.7925E-80		
1	PROX1	1.3433E-05	2.88	0.78146202	6.0177E-05	Q92786	PROX1
1	PROX2	0.00063072	2.60	1	0.00249992	Q3B8N5	PROX2
1	PROZ	2.959E-10	2.67	1.7214E-05	1.6858E-09	P22891	PROZ
1	PRPF40B	0	-3.04	0	0	Q6NWY9	PRPF40B
1	PRPF6	1.5429E-18	2.41	8.9758E-14	1.1418E-17	O94906	PRPF6
1	PRPH2	0.00082162	-3.76	1	0.00322386	P23942	PRPH2

1	PRPSAP1	7.8441E-36	1.56	4.5633E-31	7.8111E-35	Q14558	PRPSAP1
1	PRR14	1.9153E-45	1.64	1.1142E-40	2.1435E-44	Q9BWN1	PRR14
1	PRR15	5.547E-136	-7.92	3.227E-131	1.144E-134	Q8IV56	PRR15
1	PRR15L	2.4484E-07	-3.25	0.01424325	1.2145E-06		
1	PRR16	6.051E-288	87.67	3.52E-283	1.695E-286	Q569H4	PRR16
1	PRR19	1.9017E-08	2.18	0.00110628	1.0012E-07	A6NJB7	PRR19
1	PRR20C	0.00228424	-23.62	1	0.00858753	P86479	PRR20C
1	PRR22	1.3368E-45	3.83	7.7767E-41	1.4996E-44		
1	PRR29	1.9849E-13	-5.83	1.1547E-08	1.2755E-12		
1	PRR3	1.0854E-71	2.07	6.3142E-67	1.5062E-70	P79522	PRR3
1	PRR33	1.2264E-05	-14.30	0.71345709	5.5076E-05		
1	PRR34-AS1	4.0297E-15	1.73	2.3442E-10	2.7316E-14		
1	PRR5	2.9352E-32	-2.34	1.7075E-27	2.7905E-31	P85299	PRR5
1	PRR5-ARHGAI	4.7777E-16	-13.15	2.7794E-11	3.3406E-15	B1AHC4	PRR5-ARHGAI
1	PRR5L	7.899E-158	8.61	4.595E-153	1.744E-156	Q6MZQ0	PRR5L
1	PRR7	5.3992E-19	1.76	3.1409E-14	4.0356E-18	Q8TB68	PRR7
1	PRR7-AS1	4.0798E-23	2.51	2.3734E-18	3.3009E-22		
1	PRRC1	9.4284E-33	1.53	5.4849E-28	9.0346E-32	Q96M27	PRRC1
1	PRRC2B	0	-1.87	0	0	Q5JSZ5	PRRC2B
1	PRRG1	6.6993E-83	-3.01	3.8973E-78	1.0181E-81	O14668	PRRG1
1	PRRG2	8.806E-200	-6.40	5.123E-195	2.218E-198	O14669	PRRG2
1	PRRG4	1.264E-264	-14.25	7.354E-260	3.504E-263	Q9BZD6	PRRG4
1	PRRT2	0.0005502	1.84	1	0.00219243	Q7Z6L0	PRRT2
1	PRRT3-AS1	7.4538E-29	-7.26	4.3362E-24	6.7301E-28		
1	PRRT4	3.0375E-21	-52.62	1.7671E-16	2.3732E-20	C9JH25	PRRT4
1	PRSS1	2.148E-103	812.74	1.2498E-98	3.78E-102	P07477	PRSS1
1	PRSS12	1.3113E-61	4.94	7.6282E-57	1.6918E-60	P56730	PRSS12
1	PRSS16	6.2195E-92	-3.84	3.6181E-87	1.0126E-90	Q9NQE7	PRSS16
1	PRSS2	0	14029.81	0	0	P07478	PRSS2
1	PRSS21	1.299E-199	-8.32	7.555E-195	3.269E-198	Q9Y6M0	PRSS21
1	PRSS22	0	-8.76	0	0	Q9GZN4	PRSS22
1	PRSS23	0	-1.71	0	0	O95084	PRSS23
1	PRSS3	2.363E-103	11.32	1.3745E-98	4.156E-102	P35030	PRSS3
1	PRSS35	1.4496E-11	-5.58	8.4326E-07	8.7294E-11	Q8N3Z0	PRSS35
1	PRSS36	9.2674E-13	-3.21	5.3912E-08	5.8189E-12	Q5K4E3	PRSS36
1	PRSS38	1.9116E-32	-31.33	1.1121E-27	1.8236E-31	A1L453	PRSS38
1	PRSS41	5.7608E-38	-8.86	3.3513E-33	5.8846E-37	Q7RTY9	PRSS41
1	PRSS42	0.00242609	-21.56	1	0.00909962	Q7Z5A4	PRSS42
1	PRSS45	0.00573247	-21.70	1	0.02070536	Q7RTY3	PRSS45
1	PRSS50_1	0.0035383	-22.05	1	0.01307236		
1	PRSS50_2	1.1377E-10	-11.70	6.6185E-06	6.5987E-10		
1	PRSS51	0.00014824	2.41	1	0.00061746	A0A1B0GVH4	PRSS51
1	PRSS53	1.2459E-11	2.28	7.2478E-07	7.5193E-11	Q2L4Q9	PRSS53
1	PRSS8	0	-20.65	0	0	Q16651	PRSS8
1	PRTFDC1	6.292E-198	-7.33	3.66E-193	1.576E-196	Q9NRG1	PRTFDC1
1	PRTG	3.7097E-11	-1.60	2.1581E-06	2.1919E-10	Q2VWP7	PRTG
1	PRUNE1	0	-1.55	0	0	Q86TP1	PRUNE1

1	PRUNE2	1.0964E-16	-5.19	6.3782E-12	7.8068E-16	Q8WUY3	PRUNE2
1	PRX	5.4456E-14	1.65	3.168E-09	3.5619E-13	Q9BXM0	PRX
1	PSCA	2.553E-143	-4.57	1.485E-138	5.417E-142	O43653	PSCA
1	PSD	1.5238E-09	2.12	8.8644E-05	8.4214E-09	A5PKW4	PSD
1	PSD2	5.9671E-07	-17.32	0.03471274	2.9005E-06	Q9BQI7	PSD2
1	PSD3	0	-4.66	0	0	Q9NYI0	PSD3
1	PSD4	0	-10.83	0	0	Q8NDX1	PSD4
1	PSG5	7.3038E-07	-18.57	0.04248905	3.5334E-06	Q15238	PSG5
1	PSG9	5.9559E-05	-3.12	1	0.00025546	Q00887	PSG9
1	PSKH1	0	-1.93	0	0	P11801	PSKH1
1	PSMA1	1.0551E-29	1.69	6.1382E-25	9.6634E-29	P25786	PSMA1
1	PSMA5	2.4921E-53	1.94	1.4498E-48	3.0134E-52	P28066	PSMA5
1	PSMA6	8.5673E-16	1.61	4.984E-11	5.9375E-15	P60900	PSMA6
1	PSMA7	6.2163E-46	1.96	3.6163E-41	6.9921E-45	O14818	PSMA7
1	PSMA8	5.3716E-84	3.48	3.1249E-79	8.2407E-83	Q8TAA3	PSMA8
1	PSMB5	2.4472E-23	1.56	1.4237E-18	1.9892E-22	P28074	PSMB5
1	PSMB8	2.56E-216	-8.22	1.489E-211	6.717E-215	P28062	PSMB8
1	PSMB8-AS1	2.3217E-73	-8.58	1.3506E-68	3.2624E-72		
1	PSMB9	6.644E-164	-6.47	3.865E-159	1.492E-162	P28065	PSMB9
1	PSMC1	2.9014E-95	2.40	1.6878E-90	4.839E-94	P62191	PSMC1
1	PSMC3	2.9957E-53	1.89	1.7427E-48	3.6186E-52	P17980	PSMC3
1	PSMC3IP	1.7235E-36	1.68	1.0026E-31	1.7322E-35	Q9P2W1	PSMC3IP
1	PSMC4	1.5459E-62	1.82	8.9931E-58	2.0096E-61	P43686	PSMC4
1	PSMC5	3.578E-32	1.57	2.0815E-27	3.3989E-31	P62195	PSMC5
1	PSMC6	4.5228E-23	1.68	2.6311E-18	3.6558E-22	P62333	PSMC6
1	PSMD13	3.2104E-63	1.87	1.8676E-58	4.1941E-62	Q9UNM6	PSMD13
1	PSMD2	2.2533E-45	1.57	1.3109E-40	2.5185E-44	Q13200	PSMD2
1	PSMD6	8.8897E-34	1.71	5.1715E-29	8.6466E-33	Q15008	PSMD6
1	PSMD6-AS2	8.1671E-05	-1.65	1	0.00034687		
1	PSMD7	1.3282E-53	1.84	7.7266E-49	1.6114E-52	P51665	PSMD7
1	PSMD9	4.769E-61	2.00	2.7743E-56	6.123E-60	O00233	PSMD9
1	PSMG2	8.2469E-24	1.52	4.7975E-19	6.7647E-23	Q969U7	PSMG2
1	PSMG3-AS1	2.0996E-74	-3.49	1.2214E-69	2.9748E-73		
1	PSORS1C1	2.123E-115	-11.39	1.235E-110	4.008E-114		
1	PSORS1C2	1.2103E-11	-20.22	7.0408E-07	7.3099E-11	Q9UIG4	PSORS1C2
1	PSORS1C3	0.00963133	17.61	1	0.03384638		
1	PSPN	6.5945E-05	1.55	1	0.000282	O60542	PSPN
1	PTAFR	6.842E-222	-8.71	3.98E-217	1.814E-220	P25105	PTAFR
1	PTAR1	0	-1.71	0	0	Q7Z6K3	PTAR1
1	PTBP1P	0.00719698	8.30	1	0.02570464		
1	PTBP3	0	-1.86	0	0	O95758	PTBP3
1	PTCD1	0	-1.69	0	0	O75127	PTCD1
1	PTCH2	5.5366E-09	2.84	0.00032209	2.984E-08	Q9Y6C5	PTCH2
1	PTCHD1-AS	8.3757E-05	-2.63	1	0.0003554		
1	PTCHD4	8.6293E-06	-5.68	0.50199854	3.9151E-05	Q6ZW05	PTCHD4
1	PTDSS1	0	-2.33	0	0	P48651	PTDSS1
1	PTDSS2	1.4791E-37	3.33	8.6045E-33	1.5019E-36	Q9BVG9	PTDSS2

1	PTENP1	0.00873436	2.81	1	0.03088268		
1	PTENP1-AS	0.00134893	4.53	1	0.00519514		
1	PTER	2.487E-43	1.72	1.4468E-38	2.7226E-42	Q96BW5	PTER
1	PTGER1	0.00680599	-7.63	1	0.02435754	P34995	PTGER1
1	PTGER2	7.0512E-11	-7.77	4.102E-06	4.1226E-10	P43116	PTGER2
1	PTGER4	5.599E-122	6.33	3.257E-117	1.096E-120	P35408	PTGER4
1	PTGER4P2-CD	0.00305439	1.93	1	0.01135518		
1	PTGES	2.6E-162	-5.32	1.512E-157	5.808E-161	O14684	PTGES
1	PTGES2-AS1	3.0258E-16	-3.22	1.7602E-11	2.129E-15		
1	PTGES3L	7.4212E-30	-6.28	4.3172E-25	6.8095E-29		
1	PTGFR	8.6326E-05	-5.74	1	0.000366	P43088	PTGFR
1	PTGFRN	0	-5.82	0	0	Q9P2B2	PTGFRN
1	PTGS1	0	-12.13	0	0	P23219	PTGS1
1	PTGS2	8.9684E-08	-2.24	0.0052173	4.5506E-07	P35354	PTGS2
1	PTH1R	0.00676857	1.74	1	0.02423405	Q03431	PTH1R
1	PTH2R	0.0071006	-1.91	1	0.02537441	P49190	PTH2R
1	PTK2	0	-1.63	0	0	Q05397	PTK2
1	PTK2B	0	-8.99	0	0	Q14289	PTK2B
1	PTK6	0	-1.86	0	0	Q13882	PTK6
1	PTK7	0	-2.13	0	0	Q13308	PTK7
1	PTMAP5	0.00612664	-1.75	1	0.0220497		
1	PTMS	0	-1.57	0	0	P20962	PTMS
1	PTN	0.00202496	2.81	1	0.00765468	P21246	PTN
1	PTP4A2	8.525E-36	1.54	4.9593E-31	8.4876E-35	Q12974	PTP4A2
1	PTP4A2P1	0.00035937	-3.37	1	0.0014525		
1	PTP4A3	7.774E-186	-11.68	4.522E-181	1.862E-184	O75365	PTP4A3
1	PTPDC1	0	-2.42	0	0	A2A3K4	PTPDC1
1	PTPN13	0	-4.16	0	0	Q12923	PTPN13
1	PTPN21	8.6431E-88	2.84	5.028E-83	1.3645E-86	Q16825	PTPN21
1	PTPN22	9.4076E-06	36.32	0.54727667	4.2566E-05	Q9Y2R2	PTPN22
1	PTPN3	0	-1.90	0	0	P26045	PTPN3
1	PTPN6	0	-7.94	0	0	P29350	PTPN6
1	PTPRB	5.576E-191	-9.92	3.244E-186	1.362E-189	P23467	PTPRB
1	PTPRD	2.2467E-15	-4.48	1.307E-10	1.5357E-14	P23468	PTPRD
1	PTPRE	0	-2.22	0	0	P23469	PTPRE
1	PTPRF	0	-1.52	0	0	P10586	PTPRF
1	PTPRG	9.725E-103	28.08	5.6575E-98	1.704E-101	P23470	PTPRG
1	PTPRG-AS1	2.539E-114	209.39	1.477E-109	4.763E-113		
1	PTPRM	1.096E-166	12.30	6.373E-162	2.481E-165	P28827	PTPRM
1	PTPRN2	1.151E-162	26.45	6.697E-158	2.577E-161	Q92932	PTPRN2
1	PTPRQ	1.3051E-09	-3.55	7.5925E-05	7.2358E-09	Q9UMZ3	PTPRQ
1	PTPRR	0.00165691	-3.55	1	0.0063235	Q15256	PTPRR
1	PTPRU	0	-9.23	0	0	Q92729	PTPRU
1	PTPRZ1	1.512E-122	-4.49	8.796E-118	2.963E-121	P23471	PTPRZ1
1	PTRF	1.885E-136	1.98	1.097E-131	3.895E-135		
1	PTRH1	5.2304E-25	1.67	3.0427E-20	4.39E-24	Q86Y79	PTRH1
1	PTS	5.2957E-55	1.93	3.0807E-50	6.5008E-54	Q03393	PTS

1	PTX3	7.564E-139	144.48	4.4E-134	1.577E-137	P26022	PTX3
1	PUDP	7.4597E-36	1.91	4.3396E-31	7.4296E-35	Q08623	PUDP
1	PURA	7.6874E-16	1.53	4.4721E-11	5.3328E-15	Q00577	PURA
1	PURB	0	-1.65	0	0	Q96QR8	PURB
1	PURG	1.8343E-18	21.81	1.0671E-13	1.3559E-17	Q9UJV8	PURG
1	PUSL1	6.7605E-85	2.71	3.9329E-80	1.0446E-83	Q8N0Z8	PUSL1
1	PVRIG	1.0664E-05	-7.67	0.62038018	4.808E-05	Q6DKI7	PVRIG
1	PVRL3-AS1	4.7071E-37	-5.27	2.7383E-32	4.754E-36		
1	PWP1	1.122E-20	1.50	6.527E-16	8.6645E-20	Q13610	PWP1
1	PWWP2A	1.3275E-41	1.69	7.7228E-37	1.4191E-40	Q96N64	PWWP2A
1	PWWP2B	2.185E-181	-5.39	1.271E-176	5.173E-180	Q6NUJ5	PWWP2B
1	PXDC1	5.031E-115	17.55	2.927E-110	9.475E-114	Q5TGL8	PXDC1
1	PXDN	0	607.37	0	0	Q92626	PXDN
1	PXDNL	1.7339E-06	-1.79	0.10086911	8.2134E-06	A1KZ92	PXDNL
1	PXK	6.432E-108	3.45	3.742E-103	1.161E-106	Q7Z7A4	PXK
1	PXMP4	6.5572E-77	2.75	3.8146E-72	9.4725E-76	Q9Y6I8	PXMP4
1	PXN	0	-1.74	0	0	P49023	PXN
1	PXYLP1	0	-3.40	0	0	Q8TE99	PXYLP1
1	PYCARD	2.775E-100	-10.36	1.6142E-95	4.774E-99	Q9ULZ3	PYCARD
1	PYCARD-AS1	0.00793762	-8.79	1	0.02821995		
1	PYCR1	4.38E-145	10.57	2.548E-140	9.326E-144	P32322	PYCR1
1	PYCR2	0	-2.67	0	0	Q96C36	PYCR2
1	PYGB	8.5281E-52	2.09	4.9611E-47	1.0137E-50	P11216	PYGB
1	PYGO1	4.551E-121	29.83	2.648E-116	8.855E-120	Q9Y3Y4	PYGO1
1	PYM1	0	-1.54	0	0	Q9BRP8	PYM1
1	PYROXD1	3.8773E-30	1.95	2.2556E-25	3.5718E-29	Q8WU10	PYROXD1
1	PYY	3.0026E-05	-2.74	1	0.00013145	P10082	PYY
1	PYY2	1.1335E-13	9.27	6.5938E-09	7.3387E-13	Q9NRI6	PYY2
1	QKI	7.1443E-31	1.55	4.1561E-26	6.6562E-30	Q96PU8	QKI
1	QPCT	1.7688E-29	-13.47	1.029E-24	1.6128E-28	Q16769	QPCT
1	QPRT	0	198.79	0	0	Q15274	QPRT
1	QRFPR	2.5862E-48	-20.99	1.5045E-43	2.9822E-47	Q96P65	QRFPR
1	QRICH2	9.7468E-40	4.27	5.6701E-35	1.016E-38		
1	QSOX1	3.9344E-81	1.84	2.2888E-76	5.8913E-80	O00391	QSOX1
1	QTRT1	5.515E-117	2.52	3.208E-112	1.05E-115	Q9BXR0	QTRT1
1	R3HDM2	0	-1.91	0	0	Q9Y2K5	R3HDM2
1	R3HDM4	1.4032E-32	1.51	8.1632E-28	1.3411E-31	Q96D70	R3HDM4
1	RAB11FIP1	0	-2.34	0	0	Q6WKZ4	RAB11FIP1
1	RAB11FIP2	3.6033E-24	1.82	2.0962E-19	2.9738E-23	Q7L804	RAB11FIP2
1	RAB11FIP3	2.977E-110	5.34	1.732E-105	5.464E-109	O75154	RAB11FIP3
1	RAB11FIP4	3.3555E-70	-14.14	1.952E-65	4.6092E-69	Q86YS3	RAB11FIP4
1	RAB11FIP5	8.346E-46	1.53	4.8552E-41	9.3784E-45	Q9BXF6	RAB11FIP5
1	RAB17	9.748E-187	-9.66	5.671E-182	2.343E-185	Q9H0T7	RAB17
1	RAB19	3.5638E-06	-16.08	0.20732304	1.6525E-05	A4D1S5	RAB19
1	RAB20	2.0762E-64	-3.58	1.2078E-59	2.7387E-63	Q9NX57	RAB20
1	RAB24	8.5861E-48	2.09	4.9949E-43	9.846E-47	Q969Q5	RAB24
1	RAB25	0	-13.57	0	0	P57735	RAB25

1	RAB26	0	-5.17	0	0	Q9ULW5	RAB26
1	RAB27B	3.7765E-50	-4.25	2.197E-45	4.4267E-49	O00194	RAB27B
1	RAB28	0	-1.65	0	0	P51157	RAB28
1	RAB29	0	-1.59	0	0	O14966	RAB29
1	RAB30	8.962E-152	6.22	5.214E-147	1.937E-150	Q15771	RAB30
1	RAB32	3.1055E-19	1.67	1.8066E-14	2.3317E-18	Q13637	RAB32
1	RAB33A	0.00022878	-8.63	1	0.00094017	Q14088	RAB33A
1	RAB36	3.064E-133	10.21	1.783E-128	6.246E-132	O95755	RAB36
1	RAB37	6.1178E-07	-3.04	0.03558957	2.9715E-06	Q96AX2	RAB37
1	RAB38	2.119E-190	-4.83	1.233E-185	5.172E-189	P57729	RAB38
1	RAB39B	2.272E-13	10.61	1.3217E-08	1.4556E-12	Q96DA2	RAB39B
1	RAB3A	6.22E-05	1.80	1	0.00026641	P20336	RAB3A
1	RAB3B	1.4762E-98	2.50	8.5874E-94	2.5168E-97	P20337	RAB3B
1	RAB3D	3.965E-164	4.49	2.307E-159	8.906E-163	O95716	RAB3D
1	RAB3GAP2	0	-1.91	0	0	Q9H2M9	RAB3GAP2
1	RAB3IL1	2.5966E-34	-2.71	1.5105E-29	2.5425E-33	Q8TBN0	RAB3IL1
1	RAB40A	6.0467E-09	-2.35	0.00035176	3.2532E-08	Q8WXH6	RAB40A
1	RAB40B	4.712E-116	4.47	2.741E-111	8.92E-115	Q12829	RAB40B
1	RAB42	5.8711E-06	-2.77	0.34154335	2.6914E-05	Q8N4Z0	RAB42
1	RAB43	0	-2.38	0	0	Q86YS6	RAB43
1	RAB4B	0	-1.81	0	0	P61018	RAB4B
1	RAB6A	0	-1.55	0	0	P20340	RAB6A
1	RAB6B	5.1454E-08	1.77	0.00299331	2.6445E-07	Q9NRW1	RAB6B
1	RAB7B	5.4985E-05	-3.43	1	0.00023643	Q96AH8	RAB7B
1	RAB8A	7.1338E-32	1.51	4.15E-27	6.7447E-31	P61006	RAB8A
1	RAB8B	1.9044E-96	4.48	1.1079E-91	3.1955E-95	Q92930	RAB8B
1	RABAC1	9.0129E-40	1.90	5.2431E-35	9.398E-39	Q9UI14	RABAC1
1	RABGAP1	3.3022E-09	-2.05	0.0001921	1.7994E-08	Q9Y3P9	RABGAP1
1	RABGAP1L	0	-3.30	0	0	Q5R372	RABGAP1L
1	RABGGTB	3.6002E-75	1.59	2.0944E-70	5.1345E-74	P53611	RABGGTB
1	RABL3	1.982E-16	1.53	1.153E-11	1.4043E-15	Q5HYI8	RABL3
1	RAC1	0	-1.50	0	0	P63000	RAC1
1	RAC3	2.5693E-23	1.81	1.4947E-18	2.0875E-22	P60763	RAC3
1	RACK1	1.1506E-98	2.79	6.6934E-94	1.964E-97	P63244	RACK1
1	RAD17	4.1322E-31	1.58	2.4039E-26	3.8648E-30	O75943	RAD17
1	RAD21	0	-1.90	0	0	O60216	RAD21
1	RAD21-AS1	2.7691E-05	-3.60	1	0.00012143		
1	RAD51-AS1	0	-2.85	0	0		
1	RAD51AP1	1.7547E-35	1.67	1.0208E-30	1.7408E-34	Q96B01	RAD51AP1
1	RAD51AP2	1.4835E-14	-21.99	8.6301E-10	9.8742E-14	Q09MP3	RAD51AP2
1	RAD51C	5.338E-27	1.66	3.1053E-22	4.6459E-26	O43502	RAD51C
1	RAD51D	1.0702E-53	1.85	6.226E-49	1.3001E-52	O75771	RAD51D
1	RAD52	2.757E-36	1.92	1.6039E-31	2.7629E-35	P43351	RAD52
1	RAD54L	1.8745E-87	2.23	1.0905E-82	2.9496E-86	Q92698	RAD54L
1	RAD9A	0	-3.93	0	0	Q99638	RAD9A
1	RAD9B	0.00019739	1.76	1	0.00081565	Q6WBX8	RAD9B
1	RAE1	3.169E-147	1.95	1.843E-142	6.767E-146	P78406	RAE1

1	RAET1E	2.378E-149	-43.78	1.383E-144	5.112E-148	Q8TD07	RAET1E
1	RAET1E-AS1_1	4.2529E-35	-7.57	2.4741E-30	4.1984E-34		
1	RAET1G	1.123E-168	-15.23	6.53E-164	2.561E-167	Q6H3X3	RAET1G
1	RAET1K	8.0937E-59	4.52	4.7084E-54	1.0234E-57		
1	RAET1L	0.00104677	-5.39	1	0.0040705	Q5VY80	RAET1L
1	RAG1	1.6432E-92	-8.88	9.5593E-88	2.6875E-91	P15918	RAG1
1	RAI14	5.3937E-98	2.36	3.1377E-93	9.1559E-97	Q9P0K7	RAI14
1	RAI2	0.00665072	-8.75	1	0.02383409	Q9Y5P3	RAI2
1	RALGAPA2	0	-2.86	0	0	Q2PPJ7	RALGAPA2
1	RALGDS	0	-2.27	0	0	Q12967	RALGDS
1	RALGPS2	0	-1.86	0	0	Q86X27	RALGPS2
1	RALY-AS1	5.5122E-34	-2.32	3.2067E-29	5.3803E-33		
1	RALYL	3.1415E-10	-4.56	1.8275E-05	1.7873E-09	Q86SE5	RALYL
1	RAN	1.9661E-52	1.99	1.1438E-47	2.3544E-51	P62826	RAN
1	RANBP1	2.976E-136	2.38	1.731E-131	6.144E-135	P43487	RANBP1
1	RANBP17	9.6281E-84	24.61	5.601E-79	1.4712E-82	Q9H2T7	RANBP17
1	RANBP3L	1.2997E-05	-3.64	0.75608526	5.8272E-05	Q86VV4	RANBP3L
1	RANBP6	2.8654E-14	1.51	1.6669E-09	1.8916E-13	O60518	RANBP6
1	RANGAP1	1.348E-123	1.61	7.84E-119	2.654E-122	P46060	RANGAP1
1	RANGRF	4.1552E-70	2.23	2.4173E-65	5.7024E-69	Q9HD47	RANGRF
1	RANP1	0.01037276	1.70	1	0.03633995		
1	RAP1B	5.1719E-42	1.86	3.0087E-37	5.5645E-41	P61224	RAP1B
1	RAP1GAP	5.2364E-25	-3.71	3.0462E-20	4.3944E-24	P47736	RAP1GAP
1	RAP1GAP2	1.7223E-53	1.69	1.002E-48	2.0857E-52	Q684P5	RAP1GAP2
1	RAP2A	1.616E-27	1.75	9.4009E-23	1.4237E-26	P10114	RAP2A
1	RAP2C-AS1	9.5336E-08	-2.40	0.00554609	4.8319E-07		
1	RAPGEF2	1.65E-41	1.96	9.5986E-37	1.7612E-40	Q9Y4G8	RAPGEF2
1	RAPGEF3	3.722E-305	-10.24	2.165E-300	1.047E-303	O95398	RAPGEF3
1	RAPGEF5	1.303E-115	-5.69	7.582E-111	2.463E-114	Q92565	RAPGEF5
1	RAPGEFL1	0	-6.85	0	0	Q9UHV5	RAPGEFL1
1	RAPSN	5.1847E-37	16.18	3.0161E-32	5.2345E-36	Q13702	RAPSN
1	RARA-AS1	0.00269753	-1.66	1	0.01007681		
1	RARB	1.8154E-07	4.08	0.01056088	9.0659E-07	P10826	RARB
1	RARG	0	-4.15	0	0	P13631	RARG
1	RARRES2	3.9272E-11	3.32	2.2846E-06	2.3178E-10	Q99969	RARRES2
1	RARRES3	1.2699E-21	-7.25	7.3877E-17	9.9914E-21	Q9UL19	RARRES3
1	RARS	2.972E-103	1.85	1.729E-98	5.225E-102	P54136	RARS
1	RASA3	3.787E-109	7.10	2.203E-104	6.89E-108	Q14644	RASA3
1	RASA4	1.0605E-19	2.37	6.1694E-15	8.0466E-19	O43374	RASA4
1	RASA4B	5.3602E-07	2.39	0.03118219	2.6118E-06	C9J798	RASA4B
1	RASAL1	5.6953E-06	-3.97	0.33131833	2.6127E-05	O95294	RASAL1
1	RASAL2	0	-2.28	0	0	Q9UJF2	RASAL2
1	RASAL2-AS1	1.9004E-15	-3.08	1.1055E-10	1.3026E-14		
1	RASD2	8.262E-187	-23.84	4.806E-182	1.987E-185	Q96D21	RASD2
1	RASEF	6.567E-151	-11.64	3.82E-146	1.417E-149	Q8IZ41	RASEF
1	RASGEF1A	8.8879E-13	-10.09	5.1704E-08	5.5836E-12	Q8N9B8	RASGEF1A
1	RASGEF1B	3.3514E-05	-2.98	1	0.00014631	Q0VAM2	RASGEF1B

1	RASGRF1	7.6382E-33	-7.67	4.4435E-28	7.3325E-32	Q13972	RASGRF1
1	RASGRF2	2.833E-09	-1.80	0.0001648	1.5482E-08	O14827	RASGRF2
1	RASGRP2	4.0785E-10	-7.91	2.3726E-05	2.3093E-09	Q7LDG7	RASGRP2
1	RASIP1	2.336E-190	-7.80	1.359E-185	5.698E-189	Q5U651	RASIP1
1	RASL10A	2.5161E-05	2.65	1	0.00011065	Q92737	RASL10A
1	RASL10B	3.1316E-05	-2.75	1	0.00013693	Q96S79	RASL10B
1	RASL11A	4.1059E-08	2.76	0.00238857	2.1217E-07	Q6T310	RASL11A
1	RASL11B	6.4854E-06	-5.12	0.3772812	2.9651E-05	Q9BPW5	RASL11B
1	RASSF1	3.1138E-27	1.57	1.8114E-22	2.7244E-26	Q9NS23	RASSF1
1	RASSF10	1.3416E-94	-9.98	7.8047E-90	2.2255E-93	A6NK89	RASSF10
1	RASSF2	3.3485E-29	27.04	1.948E-24	3.0375E-28	P50749	RASSF2
1	RASSF3	1.8752E-98	2.38	1.0909E-93	3.1945E-97	Q86WH2	RASSF3
1	RASSF4	9.2694E-26	-1.89	5.3924E-21	7.8848E-25	Q9H2L5	RASSF4
1	RASSF5	3.558E-195	-15.56	2.07E-190	8.842E-194	Q8WWW0	RASSF5
1	RASSF6	1.123E-44	-11.73	6.5331E-40	1.2444E-43	Q6ZTQ3	RASSF6
1	RASSF7	6.0551E-40	1.71	3.5225E-35	6.3263E-39	Q02833	RASSF7
1	RASSF8	1.5986E-48	5.90	9.2996E-44	1.8455E-47	Q8NHQ8	RASSF8
1	RASSF8-AS1	5.1386E-45	2.67	2.9893E-40	5.7255E-44		
1	RASSF9	2.277E-111	-10.36	1.325E-106	4.21E-110	O75901	RASSF9
1	RAVER1	6.3356E-57	1.83	3.6857E-52	7.8838E-56	Q8IY67	RAVER1
1	RBBP8NL	1.775E-106	-16.29	1.032E-101	3.176E-105	Q8NC74	RBBP8NL
1	RBBP9	1.6233E-64	2.05	9.4437E-60	2.1424E-63	O75884	RBBP9
1	RBFADN	5.9991E-05	-1.59	1	0.00025727		
1	RBFOX3	0.00119973	3.03	1	0.00464173	A6NFN3	RBFOX3
1	RBL2	0	-1.91	0	0	Q08999	RBL2
1	RBM11	6.3493E-29	-7.32	3.6936E-24	5.7381E-28	P57052	RBM11
1	RBM14-RBM4	0	-2.57	0	0	A0A0A0MSL8	RBM14-RBM4
1	RBM20	0.00271045	-4.13	1	0.01012184	Q5T481	RBM20
1	RBM22	3.6728E-80	1.67	2.1366E-75	5.4604E-79	Q9NW64	RBM22
1	RBM23	0	-2.33	0	0	Q86U06	RBM23
1	RBM24	2.1248E-08	-3.73	0.00123609	1.1154E-07	Q9BX46	RBM24
1	RBM25	5.58E-42	1.51	3.2461E-37	5.9991E-41	P49756	RBM25
1	RBM26-AS1	1.8328E-15	-1.81	1.0662E-10	1.2568E-14		
1	RBM3	1.776E-99	2.13	1.0331E-94	3.0466E-98	P98179	RBM3
1	RBM38	7.4826E-97	2.35	4.3529E-92	1.2588E-95	Q9H0Z9	RBM38
1	RBM4	0	-1.88	0	0	Q9BWF3	RBM4
1	RBM42	1.2076E-32	1.60	7.0251E-28	1.1553E-31	Q9BTD8	RBM42
1	RBM43	1.3796E-09	-1.52	8.0258E-05	7.6407E-09	Q6ZSC3	RBM43
1	RBM44	3.6132E-45	3.86	2.102E-40	4.0306E-44	Q6ZP01	RBM44
1	RBM47	2.152E-184	-13.31	1.252E-179	5.133E-183	A0AV96	RBM47
1	RBM4B	0	-3.01	0	0	Q9BQ04	RBM4B
1	RBM8A	7.4509E-41	1.61	4.3345E-36	7.8738E-40	Q9Y5S9	RBM8A
1	RBMS1	0	-1.94	0	0	P29558	RBMS1
1	RBMS2	2.973E-119	2.07	1.73E-114	5.731E-118	Q15434	RBMS2
1	RBMS3	5.555E-237	6.92	3.232E-232	1.501E-235	Q6XE24	RBMS3
1	RBMS3-AS3	6.5991E-12	19.46	3.839E-07	4.019E-11		
1	RBMX2	3.3968E-27	1.58	1.9761E-22	2.9702E-26	Q9Y388	RBMX2

1	RBMXL1	6.2004E-57	1.80	3.607E-52	7.7172E-56	Q96E39	RBMXL1
1	RBMXP2	0.00918485	-2.45	1	0.03239478		
1	RBP1	7.5962E-25	-5.75	4.419E-20	6.3583E-24	P09455	RBP1
1	RBP3	6.0696E-06	-6.01	0.35309354	2.7798E-05	P10745	RBP3
1	RCAN1	1.1486E-83	3.02	6.6816E-79	1.7537E-82	P53805	RCAN1
1	RCAN2	1.5817E-06	-3.77	0.09201091	7.5093E-06	Q14206	RCAN2
1	RCBTB2	6.7983E-12	-1.70	3.9548E-07	4.139E-11	O95199	RCBTB2
1	RCC1	7.2212E-92	1.79	4.2009E-87	1.1747E-90	P18754	RCC1
1	RCCD1	0	-4.09	0	0	A6NED2	RCCD1
1	RCE1	0	-3.24	0	0	Q9Y256	RCE1
1	RCL1	2.5765E-35	1.69	1.4989E-30	2.5508E-34	Q9Y2P8	RCL1
1	RCN3	1.1324E-08	-3.86	0.00065876	6.0193E-08	Q96D15	RCN3
1	RCOR2	8.994E-236	-10.09	5.232E-231	2.425E-234	Q8IZ40	RCOR2
1	RCOR3	0	-1.82	0	0	Q9P2K3	RCOR3
1	RCSD1	9.5104E-90	-73.75	5.5326E-85	1.5224E-88	Q6JBY9	RCSD1
1	RDH11	5.288E-97	1.84	3.0762E-92	8.9063E-96	Q8TC12	RDH11
1	RDH12	1.5785E-06	-4.43	0.09182659	7.4954E-06	Q96NR8	RDH12
1	RDH14	3.7694E-15	1.55	2.1928E-10	2.5596E-14	Q9HBH5	RDH14
1	RDH16	4.3699E-13	-3.26	2.5421E-08	2.775E-12	O75452	RDH16
1	RDH5	1.271E-16	-2.49	7.3939E-12	9.0379E-16	Q92781	RDH5
1	RDM1	2.9553E-14	-2.02	1.7192E-09	1.9495E-13	Q8NG50	RDM1
1	REC114	3.979E-08	-2.61	0.00231474	2.0579E-07	Q7Z4M0	REC114
1	RECK	5.8331E-82	2.90	3.3934E-77	8.7934E-81	O95980	RECK
1	RECQL	3.8247E-87	2.94	2.225E-82	6.0053E-86	P46063	RECQL
1	REEP1	0.00058457	-3.06	1	0.00232383	Q9H902	REEP1
1	REEP2	1.0231E-16	2.22	5.9521E-12	7.2915E-16	Q9BRK0	REEP2
1	REEP3	1.4262E-23	1.51	8.2967E-19	1.1649E-22	Q6NUK4	REEP3
1	REEP4	0	-2.91	0	0	Q9H6H4	REEP4
1	REL	0	-2.36	0	0	Q04864	REL
1	RELB	1.414E-191	-4.44	8.227E-187	3.466E-190	Q01201	RELB
1	RELN	7.4366E-98	43.50	4.3262E-93	1.2613E-96	P78509	RELN
1	REN	2.7323E-07	-18.59	0.01589466	1.3519E-06	P00797	REN
1	REP15	5.7897E-09	5.44	0.00033681	3.1175E-08	Q6BDI9	REP15
1	REPS2	9.46E-101	-22.71	5.5033E-96	1.634E-99	Q8NFH8	REPS2
1	RER1	7.687E-65	1.61	4.4718E-60	1.0175E-63	O15258	RER1
1	RERE	6.7933E-43	1.69	3.9519E-38	7.3923E-42	Q9P2R6	RERE
1	RETNLB	9.6634E-15	-11.81	5.6216E-10	6.4727E-14	Q9BQ08	RETNLB
1	RFC5	9.5768E-43	1.51	5.5712E-38	1.0402E-41	P40937	RFC5
1	RFK	4.3386E-41	2.34	2.5239E-36	4.6032E-40	Q969G6	RFK
1	RFLNA	1.1163E-10	-3.53	6.4939E-06	6.4771E-10	Q6ZTI6	RFLNA
1	RFLNB	7.1453E-88	3.83	4.1567E-83	1.1286E-86	Q8N5W9	RFLNB
1	RFNG	2.0695E-43	1.78	1.2039E-38	2.2689E-42	Q9Y644	RFNG
1	RFPL3S	1.1439E-06	2.80	0.06654455	5.476E-06		
1	RFTN1	2.0186E-81	2.08	1.1743E-76	3.0289E-80	Q14699	RFTN1
1	RFTN2	0.00236985	-1.75	1	0.00889844	Q52LD8	RFTN2
1	RFWD2	0	-1.63	0	0	Q8NHY2	RFWD2
1	RFX2	0	-1.82	0	0	P48378	RFX2

1	RFX3-AS1	2.704E-159	-4.81	1.573E-154	6.004E-158		
1	RFX5	0	-2.36	0	0	P48382	RFX5
1	RFX6	4.3759E-26	-12.29	2.5457E-21	3.7442E-25	Q8HWS3	RFX6
1	RFXANK	3.1052E-27	1.59	1.8064E-22	2.7173E-26	O14593	RFXANK
1	RGCC	3.8203E-25	-5.24	2.2224E-20	3.2153E-24	Q9H4X1	RGCC
1	RGL1	5.6596E-07	1.55	0.03292423	2.754E-06	Q9NZL6	RGL1
1	RGL2	0	-1.85	0	0	O15211	RGL2
1	RGMB	1.66E-120	7.87	9.656E-116	3.219E-119	Q6NW40	RGMB
1	RGMB-AS1	1.52E-185	30.89	8.842E-181	3.639E-184		
1	RGN	2.3411E-13	-8.90	1.3619E-08	1.4989E-12	Q15493	RGN
1	RGP1	2.9504E-72	1.64	1.7164E-67	4.1131E-71	Q92546	RGP1
1	RGPD1	7.9186E-13	-8.30	4.6066E-08	4.9839E-12	P0DJD0	RGPD1
1	RGPD2	2.5588E-11	-14.36	1.4886E-06	1.5244E-10	P0DJD1	RGPD2
1	RGPD3	0.00038728	2.16	1	0.00156034	A6NKT7	RGPD3
1	RGPD5	1.5304E-08	5.23	0.00089031	8.0915E-08	Q99666	RGPD5
1	RGPD8	7.4529E-17	6.67	4.3357E-12	5.3296E-16	O14715	RGPD8
1	RGS10	4.885E-15	-1.50	2.8418E-10	3.2983E-14	O43665	RGS10
1	RGS12	0	-1.66	0	0	O14924	RGS12
1	RGS14	2.6603E-17	1.63	1.5476E-12	1.9199E-16	O43566	RGS14
1	RGS16	2.234E-09	-4.30	0.00012996	1.2257E-08	O15492	RGS16
1	RGS17	3.667E-178	5.25	2.133E-173	8.608E-177	Q9UGC6	RGS17
1	RGS18	4.7479E-08	-16.52	0.00276205	2.4458E-07	Q9NS28	RGS18
1	RGS19	2.1683E-14	1.56	1.2614E-09	1.436E-13	P49795	RGS19
1	RGS2	2.419E-12	-2.04	1.4073E-07	1.4984E-11	P41220	RGS2
1	RGS20	0	-2.35	0	0	O76081	RGS20
1	RGS21	0.00017511	-6.34	1	0.00072585	Q2M5E4	RGS21
1	RGS3	0	-2.83	0	0	P49796	RGS3
1	RGS4	1.195E-115	94.97	6.95E-111	2.259E-114	P49798	RGS4
1	RGS5_1	1.8009E-17	-1.82	1.0477E-12	1.3049E-16		
1	RGS5_2	0.00164674	-3.59	1	0.00628509		
1	RGS6	0.00065997	-2.11	1	0.00261017	P49758	RGS6
1	RGS7	0.00013082	4.49	1	0.00054714	P49802	RGS7
1	RGS9	5.7887E-07	1.79	0.03367494	2.8159E-06	O75916	RGS9
1	RHBDD2	0	-1.63	0	0	Q6NTF9	RHBDD2
1	RHBDF1	0	-1.81	0	0	Q96CC6	RHBDF1
1	RHBDF2	0	-2.06	0	0	Q6PJF5	RHBDF2
1	RHBDL1	1.43E-09	-2.02	8.3189E-05	7.9122E-09	O75783	RHBDL1
1	RHBDL2	1.585E-150	-21.78	9.221E-146	3.416E-149	Q9NX52	RHBDL2
1	RHBDL3	0.00025874	-5.14	1	0.0010573	P58872	RHBDL3
1	RHBG	3.4034E-10	-24.04	1.9799E-05	1.9337E-09	Q9H310	RHBG
1	RHCE	2.9053E-06	-2.92	0.16901349	1.3561E-05	P18577	RHCE
1	RHCG	9.4107E-11	-85.47	5.4746E-06	5.4757E-10	Q9UBD6	RHCG
1	RHNO1	8.514E-104	2.31	4.953E-99	1.503E-102	Q9BSD3	RHNO1
1	RHOBTB1	8.6581E-73	-6.92	5.0367E-68	1.2122E-71	O94844	RHOBTB1
1	RHOBTB3	0	-4.19	0	0	O94955	RHOBTB3
1	RHOC	1.959E-104	2.27	1.14E-99	3.474E-103	P08134	RHOC
1	RHOD	0	-4.70	0	0	O00212	RHOD

1	RHOF	1.7046E-38	1.71	9.9164E-34	1.7533E-37	Q9HBH0	RHOF
1	RHOG	2.132E-112	2.65	1.24E-107	3.965E-111	P84095	RHOG
1	RHOJ	3.776E-123	18.39	2.197E-118	7.416E-122	Q9H4E5	RHOJ
1	RHOU	1.9947E-15	-2.40	1.1604E-10	1.3666E-14	Q7L0Q8	RHOU
1	RHOV	4.329E-128	-14.11	2.518E-123	8.683E-127	Q96L33	RHOV
1	RHOXF1-AS1	2.2712E-08	-7.79	0.00132122	1.1903E-07		
1	RHOXF1P3	0.00106006	-2.84	1	0.00411945		
1	RHPN2	0	-1.54	0	0	Q8IUC4	RHPN2
1	RIBC1	0.00022302	1.66	1	0.00091735	Q8N443	RIBC1
1	RIBC2	6.2E-142	8.56	3.607E-137	1.308E-140	Q9H4K1	RIBC2
1	RIC8A	4.723E-54	2.57	2.7475E-49	5.7552E-53	Q9NPQ8	RIC8A
1	RICTOR	3.2734E-12	-1.51	1.9043E-07	2.0174E-11	Q6R327	RICTOR
1	RILP	7.1182E-13	-2.00	4.141E-08	4.4884E-12	Q96NA2	RILP
1	RILPL2	3.5949E-25	1.62	2.0913E-20	3.0269E-24	Q969X0	RILPL2
1	RIMBP3	5.0286E-08	2.57	0.00292532	2.5863E-07	Q9UFD9	RIMBP3
1	RIMBP3B	0.01120472	2.75	1	0.03908985		
1	RIMKLA	0.00065399	4.31	1	0.00258841	Q8IXN7	RIMKLA
1	RIMKLB	1.4969E-96	7.89	8.7081E-92	2.5124E-95	Q9ULI2	RIMKLB
1	RIMS1	1.419E-246	45.70	8.252E-242	3.867E-245	Q86UR5	RIMS1
1	RIMS2	0	-2.16	0	0	Q9UQ26	RIMS2
1	RIN3	1.0077E-44	2.28	5.8622E-40	1.1173E-43	Q8TB24	RIN3
1	RINL	9.6181E-06	-2.04	0.55952527	4.3499E-05	Q6ZS11	RINL
1	RIOK2	2.3772E-91	2.76	1.3829E-86	3.852E-90	Q9BVS4	RIOK2
1	RIPK4	0	-7.38	0	0	P57078	RIPK4
1	RIT1	0	-2.09	0	0	Q92963	RIT1
1	RITA1	4.6033E-29	1.81	2.6779E-24	4.1686E-28	Q96K30	RITA1
1	RLIM	3.4998E-33	1.54	2.036E-28	3.3708E-32	Q9NVW2	RLIM
1	RMDN1	0	-1.80	0	0	Q96DB5	RMDN1
1	RMND5A	0	-1.60	0	0	Q9H871	RMND5A
1	RMRP_1	0.00678784	3.57	1	0.02429856		
1	RN7SL636P	0.00140517	-26.99	1	0.00539816		
1	RNASE4	5.0742E-06	2.59	0.29518473	2.3326E-05	P34096	RNASE4
1	RNASEH1	3.4751E-65	1.77	2.0216E-60	4.6072E-64	O60930	RNASEH1
1	RNASEH1-AS1	8.436E-152	6.34	4.907E-147	1.824E-150		
1	RNASEH2A	9.1964E-31	1.91	5.3499E-26	8.5544E-30	O75792	RNASEH2A
1	RNASEH2B-AS	4.6947E-05	-2.86	1	0.00020286		
1	RNASEK	1.3355E-28	1.61	7.7692E-24	1.1999E-27	Q6P5S7	RNASEK
1	RNASEK-C17o	2.0845E-06	7.43	0.12126137	9.8299E-06	H0YIS7	RNASEK-C17o
1	RNASEL	9.955E-209	-8.28	5.791E-204	2.564E-207	Q05823	RNASEL
1	RNASET2	0	-1.69	0	0	O00584	RNASET2
1	RND2	0.00041443	-4.94	1	0.0016663	P52198	RND2
1	RNF11	1.4548E-32	1.63	8.4632E-28	1.3897E-31	Q9Y3C5	RNF11
1	RNF113A	2.5712E-17	1.67	1.4958E-12	1.857E-16	O15541	RNF113A
1	RNF115	1.4909E-69	1.74	8.6729E-65	2.0359E-68	Q9Y4L5	RNF115
1	RNF125	1.4558E-05	-1.51	0.84689138	6.508E-05	Q96EQ8	RNF125
1	RNF128	2.243E-155	-16.07	1.305E-150	4.911E-154	Q8TEB7	RNF128
1	RNF13	0	-2.00	0	0	O43567	RNF13

1	RNF130	1.4546E-86	11.13	8.4622E-82	2.2723E-85	Q86XS8	RNF130
1	RNF135	6.2382E-25	-1.73	3.629E-20	5.2292E-24	Q8IUD6	RNF135
1	RNF138	5.5288E-20	1.63	3.2163E-15	4.2154E-19	Q8WVD3	RNF138
1	RNF139-AS1	5.1658E-11	-1.63	3.0051E-06	3.0358E-10		
1	RNF14	7.1246E-28	1.68	4.1447E-23	6.3268E-27	Q9UBS8	RNF14
1	RNF141	2.233E-23	1.62	1.299E-18	1.8166E-22	Q8WVD5	RNF141
1	RNF144A	2.3191E-12	5.10	1.3491E-07	1.438E-11	P50876	RNF144A
1	RNF148	0.00997763	-15.94	1	0.03499782	Q8N7C7	RNF148
1	RNF150	2.4409E-23	-5.80	1.42E-18	1.9843E-22	Q9ULK6	RNF150
1	RNF151	0.00317413	5.79	1	0.01178453	Q2KHN1	RNF151
1	RNF152	1.6273E-06	-1.54	0.09466799	7.7211E-06	Q8N8N0	RNF152
1	RNF157	1.8199E-07	-3.17	0.0105872	9.0869E-07	Q96PX1	RNF157
1	RNF157-AS1	1.3281E-16	-10.38	7.7261E-12	9.4394E-16		
1	RNF165	5.643E-181	-10.89	3.283E-176	1.335E-179	Q6ZSG1	RNF165
1	RNF17	3.8485E-24	-9.48	2.2388E-19	3.1734E-23	Q9BXT8	RNF17
1	RNF170	4.2504E-60	-2.20	2.4726E-55	5.4189E-59	Q96K19	RNF170
1	RNF175	2.9044E-11	-17.62	1.6896E-06	1.7264E-10	Q8N4F7	RNF175
1	RNF180	2.6904E-05	-3.78	1	0.00011814	Q86T96	RNF180
1	RNF185	7.0235E-31	1.55	4.0858E-26	6.5447E-30	Q96GF1	RNF185
1	RNF19A	0	-2.45	0	0	Q9NV58	RNF19A
1	RNF19B	0	-1.89	0	0	Q6ZMZ0	RNF19B
1	RNF207	2.9439E-10	-1.64	1.7126E-05	1.6774E-09	Q6ZRF8	RNF207
1	RNF212B	2.3391E-22	-7.62	1.3608E-17	1.8648E-21	A8MTL3	RNF212B
1	RNF215	1.5973E-62	1.94	9.292E-58	2.076E-61	Q9Y6U7	RNF215
1	RNF217-AS1	1.3876E-06	-2.13	0.08072397	6.6097E-06		
1	RNF219-AS1	4.1706E-54	-16.67	2.4262E-49	5.0842E-53		
1	RNF223	8.883E-58	-5.65	5.1676E-53	1.1125E-56	E7ERA6	RNF223
1	RNF224	4.2423E-07	-2.68	0.02467888	2.078E-06	P0DH78	RNF224
1	RNF225	2.1379E-06	-43.86	0.12437011	1.0076E-05	M0QZC1	RNF225
1	RNF25	3.0499E-38	1.94	1.7743E-33	3.1254E-37	Q96BH1	RNF25
1	RNF31	1.3313E-83	1.81	7.7448E-79	2.0317E-82	Q96EP0	RNF31
1	RNF34	3.129E-118	2.10	1.82E-113	6.001E-117	Q969K3	RNF34
1	RNF38	6.4696E-78	1.99	3.7637E-73	9.4138E-77	Q9H0F5	RNF38
1	RNF39	7.702E-32	-3.08	4.4806E-27	7.2796E-31	Q9H2S5	RNF39
1	RNF40	8.7615E-86	1.64	5.0969E-81	1.361E-84	O75150	RNF40
1	RNF43	6.1718E-27	-40.46	3.5904E-22	5.3596E-26	Q68DV7	RNF43
1	RNF5P1	0.005325	-9.57	1	0.01931036		
1	RNFT2	1.668E-200	7.53	9.705E-196	4.218E-199	Q96EX2	RNFT2
1	RNH1	4.007E-132	2.42	2.331E-127	8.138E-131	P13489	RNH1
1	RNLS	1.8523E-43	-4.38	1.0776E-38	2.0324E-42	Q5VYX0	RNLS
1	RNPC3	0	-1.63	0	0	Q96LT9	RNPC3
1	RNPEP	0	-1.99	0	0	Q9H4A4	RNPEP
1	ROBO1	0	-7.89	0	0	Q9Y6N7	ROBO1
1	ROBO3	2.319E-118	101.96	1.349E-113	4.454E-117	Q96MS0	ROBO3
1	ROBO4	3.285E-249	44.49	1.911E-244	8.971E-248	Q8WZ75	ROBO4
1	ROM1	1.8529E-38	-2.89	1.0779E-33	1.9044E-37	Q03395	ROM1
1	ROPN1B	0.00666382	-3.07	1	0.0238796	Q9BZX4	ROPN1B

1	ROR1	2.1747E-69	-3.96	1.2651E-64	2.9656E-68	Q01973	ROR1
1	RORA-AS1	2.2336E-20	-4.92	1.2994E-15	1.714E-19		
1	RORB	2.1774E-43	-25.80	1.2667E-38	2.3863E-42	Q92753	RORB
1	RORB-AS1	0.00020874	-5.34	1	0.00086066		
1	RORC	0.00869179	-17.83	1	0.03073776	P51449	RORC
1	RP1-101K10.6	3.6957E-12	-1.69	2.15E-07	2.2727E-11		
1	RP1-102D24.5	6.2426E-08	5.08	0.00363157	3.1937E-07		
1	RP1-102K2.9	0.00066444	-5.29	1	0.0026268		
1	RP1-111C20.4	3.2195E-34	-4.56	1.8729E-29	3.1499E-33		
1	RP1-12G14.7	0.00311268	3.82	1	0.0115623		
1	RP1-130H16.1	0.00049723	-3.09	1	0.00198667		
1	RP1-138A5.3	2.433E-194	-16.24	1.416E-189	6.024E-193		
1	RP1-13D10.2	1.2739E-05	-5.83	0.74105459	5.7145E-05		
1	RP1-13D10.3	0.00023754	-7.82	1	0.000975		
1	RP1-140K8.5	1.3131E-08	10.83	0.00076388	6.9609E-08		
1	RP1-149A16.1	0.00081675	13.49	1	0.00320561		
1	RP1-150O5.3	2.3766E-06	-4.52	0.13825916	1.1162E-05		
1	RP1-151B14.6	4.2969E-07	-9.79	0.02499689	2.1045E-06		
1	RP1-151F17.2	3.8819E-05	-1.83	1	0.00016865		
1	RP1-154K9.2	3.6296E-14	-7.58	2.1115E-09	2.3886E-13		
1	RP1-159A19.4	2.388E-10	-12.47	1.3892E-05	1.3662E-09		
1	RP1-15D23.2	1.704E-192	22.14	9.912E-188	4.191E-191		
1	RP1-161P9.5	5.0315E-07	-10.28	0.02927029	2.4558E-06		
1	RP1-170O19.1	7.8865E-08	-4.45	0.00458787	4.0139E-07		
1	RP1-178F15.4	2.1659E-06	-1.64	0.12599861	1.0204E-05		
1	RP1-179N16.6	5.8453E-06	-2.10	0.34004535	2.6801E-05		
1	RP1-193H18.2	2.1129E-07	5.66	0.01229166	1.0516E-06		
1	RP1-197B17.3	4.002E-138	-7.98	2.328E-133	8.321E-137		
1	RP1-198K11.5	0.00851647	1.82	1	0.03016542		
1	RP1-199J3.6	0.00630229	5.16	1	0.02265243		
1	RP1-200K18.1	0.00573247	-21.56	1	0.02070536		
1	RP1-205F14P.	2.9752E-11	77.79	1.7308E-06	1.7674E-10		
1	RP1-206D15.6	0.00326703	-2.29	1	0.01211397		
1	RP1-20C7.6	0.0001215	-3.97	1	0.00050962		
1	RP1-225E12.3	0.00081675	-12.87	1	0.00320561		
1	RP1-228H13.5	2.4561E-12	2.18	1.4288E-07	1.521E-11		
1	RP1-239B22.5	3.202E-192	5.70	1.863E-187	7.856E-191		
1	RP1-261D10.2	3.596E-118	4.01	2.092E-113	6.893E-117		
1	RP1-261G23.5	1.69E-149	-21.48	9.831E-145	3.636E-148		
1	RP1-267D11.6	1.0765E-08	-1.56	0.00062623	5.7268E-08		
1	RP1-272L16.1	1.2724E-24	-14.13	7.4018E-20	1.0609E-23		
1	RP1-27K12.2	8.8444E-42	-39.94	5.1451E-37	9.4824E-41		
1	RP1-27K12.4	4.4955E-05	-6.88	1	0.00019448		
1	RP1-283E3.4	0	-2.71	0	0		
1	RP1-28O10.1	2.1553E-44	-11.08	1.2538E-39	2.3837E-43		
1	RP1-309F20.3	6.6911E-07	-4.37	0.03892471	3.2426E-06		
1	RP1-30E17.2	7.0387E-05	-15.44	1	0.00030035		

1	RP1-313I6.12	1.796E-17	-3.91	1.0448E-12	1.3016E-16		
1	RP1-34B20.4	0.00685086	-2.96	1	0.0245121		
1	RP1-34M23.5	0.00196669	-8.84	1	0.00744712		
1	RP1-35C21.2	0.00573247	-19.74	1	0.02070536		
1	RP1-37E16.12	0.00734741	4.10	1	0.0262226		
1	RP1-39G22.7	4.0963E-11	-1.81	2.383E-06	2.4154E-10		
1	RP1-45I4.3	0.00157855	-4.71	1	0.00603395		
1	RP1-4G17.5	7.9961E-08	-24.42	0.00465167	4.0679E-07		
1	RP1-59D14.5	0.00309071	1.84	1	0.01148361		
1	RP1-60O19.1	7.8057E-05	2.30	1	0.0003322		
1	RP1-69D17.4	8.8233E-10	4.78	5.1329E-05	4.9264E-09		
1	RP1-78O14.1	2.5725E-08	4.15	0.0014965	1.3448E-07		
1	RP1-80N2.2	0.00862104	-4.21	1	0.03050054		
1	RP1-80N2.3	1.8042E-25	-8.61	1.0496E-20	1.5271E-24		
1	RP1-80N2.4	4.9227E-18	-9.00	2.8637E-13	3.6094E-17		
1	RP1-84O15.2	5.2619E-10	-13.65	3.061E-05	2.9661E-09		
1	RP1-89D4.1	0.00173814	-4.04	1	0.00661657		
1	RP1-8B1.4	1.8012E-07	4.99	0.01047811	8.9972E-07		
1	RP1-90G24.10	1.9908E-11	12.59	1.1581E-06	1.1916E-10		
1	RP1-95L4.4	0.00216272	1.61	1	0.00815596		
1	RP11-1000B6.	8.4138E-13	-3.25	4.8946E-08	5.2903E-12		
1	RP11-1000B6.	8.9105E-07	-2.50	0.05183606	4.2896E-06		
1	RP11-1007I13	5.4572E-93	-7.97	3.1747E-88	8.9553E-92		
1	RP11-1007O2	2.547E-10	-1.94	1.4817E-05	1.4556E-09		
1	RP11-100E13.	0.00013379	12.68	1	0.00055911		
1	RP11-100N20	0.00327902	-4.80	1	0.01215688		
1	RP11-1012A1.	0.00023832	1.97	1	0.00097775		
1	RP11-1017G2	1.0369E-53	4.18	6.0321E-49	1.2598E-52		
1	RP11-101E7.2	0.00018686	3.30	1	0.00077364		
1	RP11-1020A1	3.4879E-09	-2.46	0.00020291	1.8974E-08		
1	RP11-1020M1	2.7554E-05	-34.70	1	0.00012087		
1	RP11-1023L17	6.7963E-31	-2.99	3.9537E-26	6.3371E-30		
1	RP11-1024P1	8.8488E-40	41.34	5.1477E-35	9.2286E-39		
1	RP11-1029J19	0.00238746	3.32	1	0.00896224		
1	RP11-1035H1	0.0001024	3.96	1	0.00043203		
1	RP11-1038A1	3.7902E-26	-34.71	2.2049E-21	3.2468E-25		
1	RP11-1038A1	1.4292E-32	-9.88	8.3142E-28	1.3655E-31		
1	RP11-103H7.5	0.00793762	-8.78	1	0.02821995		
1	RP11-103J17.	0.00942764	-18.06	1	0.03316063		
1	RP11-103J17.	1.2657E-27	-2.37	7.3629E-23	1.1175E-26		
1	RP11-103J8.1	9.5418E-12	-2.43	5.5508E-07	5.7827E-11		
1	RP11-104D21	0.0023996	21.79	1	0.00900493		
1	RP11-104H15	2.8349E-06	-8.71	0.16491771	1.3245E-05		
1	RP11-104H15	3.2515E-20	-4.71	1.8915E-15	2.4905E-19		
1	RP11-104L21.	0.00058679	-30.86	1	0.0023317		
1	RP11-104N10	0.00439882	-2.19	1	0.01610033		
1	RP11-104O19	0.00452822	-2.25	1	0.01655301		

1	RP11-1055B8.	0.0046432	-21.23	1	0.0169552		
1	RP11-1055B8.	0.00190382	2.01	1	0.00722083		
1	RP11-1055B8.	0.00057498	3.39	1	0.0022871		
1	RP11-1060J15	4.7464E-19	4.51	2.7612E-14	3.5513E-18		
1	RP11-106M3.1	3.3253E-09	-26.30	0.00019344	1.8116E-08		
1	RP11-1070N1	1.3087E-76	92.18	7.613E-72	1.8863E-75		
1	RP11-1070N1	5.0735E-06	56.98	0.29514394	2.3324E-05		
1	RP11-1070N1	7.7413E-10	71.62	4.5034E-05	4.3315E-09		
1	RP11-107M16	7.9725E-05	-17.08	1	0.00033903		
1	RP11-1080G1	7.0766E-26	-5.94	4.1167E-21	6.0292E-25		
1	RP11-1082L8.	1.2337E-20	-14.12	7.1771E-16	9.5149E-20		
1	RP11-108H9.1	1.5141E-05	-36.62	0.88082223	6.7584E-05		
1	RP11-108K14.	0.00041444	-2.98	1	0.0016663		
1	RP11-108K3.1	0.00039638	-2.77	1	0.00159578		
1	RP11-108K3.2	0.0001772	-4.62	1	0.00073443		
1	RP11-108M12	0.000806	2.13	1	0.00316579		
1	RP11-108M9.1	3.2693E-05	9.86	1	0.0001428		
1	RP11-108O10	0.00431839	2.70	1	0.01582974		
1	RP11-108O10	0.0140647	2.70	1	0.04854345		
1	RP11-108P20.	6.8444E-06	10.73	0.39816463	3.1246E-05		
1	RP11-1094H2	1.934E-192	-7.93	1.125E-187	4.754E-191		
1	RP11-109A6.3	8.1301E-05	36.35	1	0.00034538		
1	RP11-109A6.5	9.3756E-23	220.58	5.4542E-18	7.5428E-22		
1	RP11-109E12.	0.00035823	-6.09	1	0.00144811		
1	RP11-109M17	0.00942764	-17.89	1	0.03316063		
1	RP11-109N23	1.0333E-35	3.90	6.0111E-31	1.0279E-34		
1	RP11-10K16.1	1.4529E-31	-2.45	8.4518E-27	1.3669E-30		
1	RP11-10N16.3	1.5029E-41	-56.89	8.7427E-37	1.6051E-40		
1	RP11-10N23.2	0.00012494	-2.15	1	0.0005235		
1	RP11-10N23.5	0.01108447	-2.42	1	0.03869356		
1	RP11-10O17.1	5.5612E-14	-3.32	3.2352E-09	3.6371E-13		
1	RP11-10O17.3	1.6969E-22	-3.51	9.8717E-18	1.3573E-21		
1	RP11-10O22.1	1.0525E-05	-7.90	0.61229852	4.7472E-05		
1	RP11-1100L3.	0.00043945	-4.69	1	0.00176344		
1	RP11-1101K5.	0.0063115	-3.86	1	0.02268413		
1	RP11-110G21	5.3168E-18	-1.92	3.093E-13	3.8945E-17		
1	RP11-110I1.12	5.6329E-07	1.61	0.03276905	2.7417E-06		
1	RP11-1113L8.	9.6436E-05	-11.87	1	0.00040786		
1	RP11-1114A5.	8.8399E-11	1.91	5.1425E-06	5.1508E-10		
1	RP11-1114I9.1	7.6689E-71	-10.50	4.4613E-66	1.0574E-69		
1	RP11-111A22.	2.7307E-07	-1.82	0.01588544	1.3513E-06		
1	RP11-111F5.2	0.0012452	4.19	1	0.00481094		
1	RP11-111F5.4	6.9911E-62	5.27	4.067E-57	9.0418E-61		
1	RP11-111K18.	0.00647606	-3.26	1	0.02323682		
1	RP11-111M22	0	-1.63	0	0		
1	RP11-111M22	6.6488E-29	-2.18	3.8679E-24	6.0079E-28		
1	RP11-112L6.2	1.1401E-06	-3.69	0.06632339	5.4583E-06		

1	RP11-112L6.3	5.7655E-05	-3.57	1	0.00024764			
1	RP11-1134I14	1.0328E-16	-8.66	6.0085E-12	7.3597E-16			
1	RP11-1143G9	3.155E-08	-21.55	0.00183541	1.6413E-07			
1	RP11-1144P2	2.6989E-13	-12.62	1.57E-08	1.724E-12			
1	RP11-1148L6.	6.6562E-28	2.19	3.8722E-23	5.9145E-27			
1	RP11-1148L6.	0.0080035	-2.24	1	0.02843333			
1	RP11-1149O2	0.00011381	-2.01	1	0.00047871			
1	RP11-114B7.6	4.7701E-26	-7.04	2.775E-21	4.0766E-25			
1	RP11-114G22	0.00027958	-3.17	1	0.00113905			
1	RP11-114H23	1.531E-135	4.83	8.907E-131	3.152E-134			
1	RP11-114H23	2.1187E-91	13.78	1.2326E-86	3.4343E-90			
1	RP11-114M1.	1.7342E-09	-13.18	0.00010089	9.5591E-09			
1	RP11-115C21.	3.3754E-14	-1.83	1.9636E-09	2.2225E-13			
1	RP11-115D19	1.675E-20	-15.53	9.744E-16	1.2885E-19			
1	RP11-115E19.	0.00014225	-12.45	1	0.00059362			
1	RP11-115J23.	1.612E-101	37.80	9.3794E-97	2.8E-100			
1	RP11-115N4.1	9.9827E-07	-2.20	0.05807332	4.7939E-06			
1	RP11-1166P1	2.6394E-10	-4.56	1.5354E-05	1.5071E-09			
1	RP11-116G8.5	1.2859E-07	-3.15	0.00748058	6.4756E-07			
1	RP11-116O18	3.7549E-12	-7.42	2.1844E-07	2.3083E-11			
1	RP11-1198D2	0.00076332	-4.62	1	0.003004			
1	RP11-119B16.	1.2551E-05	-1.99	0.73016896	5.6336E-05			
1	RP11-119D9.1	4.0448E-23	-11.83	2.353E-18	3.2731E-22			
1	RP11-119F7.5	1.619E-08	-3.93	0.00094185	8.5529E-08			
1	RP11-11M20.	2.0062E-13	3.06	1.1671E-08	1.2886E-12			
1	RP11-11N5.1	1.0357E-17	-9.41	6.0253E-13	7.5401E-17			
1	RP11-11N5.3	0.00027549	-7.81	1	0.00112302			
1	RP11-11N9.4	5.3044E-11	-2.14	3.0858E-06	3.115E-10			
1	RP11-120D5.1	6.8087E-27	-3.31	3.9609E-22	5.9065E-26			
1	RP11-1212A2	7.6367E-82	3.89	4.4426E-77	1.15E-80			
1	RP11-1212A2	0.00221501	-25.08	1	0.00834127			
1	RP11-121C2.3	5.9595E-07	-1.61	0.03466866	2.897E-06			
1	RP11-121C6.5	0.01193309	8.86	1	0.04150648			
1	RP11-121L10.	4.817E-15	-4.32	2.8023E-10	3.2543E-14			
1	RP11-121M22	3.6128E-10	-7.26	2.1017E-05	2.0514E-09			
1	RP11-1221G1	7.9742E-05	41.43	1	0.00033903			
1	RP11-1223D1	2.814E-07	-76.83	0.01637009	1.3911E-06			
1	RP11-1223D1	4.946E-14	-49.55	2.8773E-09	3.2398E-13			
1	RP11-1223D1	3.5871E-25	-52.92	2.0867E-20	3.0208E-24			
1	RP11-1223D1	1.9274E-21	-81.62	1.1212E-16	1.5113E-20			
1	RP11-1228E1	3.5795E-06	-2.87	0.20823228	1.6595E-05			
1	RP11-122C21.	0.01217707	-2.54	1	0.04229439			
1	RP11-122G18	3.3859E-16	-3.30	1.9697E-11	2.3794E-15			
1	RP11-122G18	3.3951E-05	-7.85	1	0.00014818			
1	RP11-122G18	4.5067E-12	-3.62	2.6218E-07	2.7615E-11			
1	RP11-122K13.	1.3078E-06	-2.18	0.07607884	6.2396E-06			
1	RP11-122M14	1.2792E-10	-6.79	7.4416E-06	7.4076E-10			

1	RP11-123O10	3.5575E-17	-13.45	2.0695E-12	2.5597E-16		
1	RP11-123O10	3.2418E-35	-4.02	1.8859E-30	3.2046E-34		
1	RP11-1246C1	3.4342E-71	-5.80	1.9978E-66	4.7487E-70		
1	RP11-124N14	8.8351E-11	42.51	5.1397E-06	5.1485E-10		
1	RP11-124N3.3	1.7221E-30	-31.65	1.0018E-25	1.5958E-29		
1	RP11-1252I4.2	0.00733638	2.62	1	0.02618483		
1	RP11-125O5.2	0.00469303	-2.60	1	0.01712641		
1	RP11-126K1.2	0.00261695	-1.69	1	0.00978963		
1	RP11-1275H2	3.0705E-09	-2.06	0.00017862	1.6752E-08		
1	RP11-1275H2	0.00018125	-2.73	1	0.00075066		
1	RP11-1275H2	2.1651E-09	-2.43	0.00012595	1.1885E-08		
1	RP11-1277A3.	3.818E-168	10.09	2.221E-163	8.691E-167		
1	RP11-1277A3.	1.507E-117	105.62	8.764E-113	2.877E-116		
1	RP11-127B20.	0.00887755	-2.17	1	0.03136229		
1	RP11-127L20.	0.00119767	-27.44	1	0.00463406		
1	RP11-1281K2	6.5949E-19	-8.54	3.8365E-14	4.9237E-18		
1	RP11-1281K2	3.6754E-07	-75.44	0.02138142	1.8062E-06		
1	RP11-128L5.1	6.6459E-25	-4.72	3.8662E-20	5.5685E-24		
1	RP11-129M6.	5.8684E-17	-13.07	3.4139E-12	4.2033E-16		
1	RP11-12A20.4	2.9631E-09	2.28	0.00017238	1.6176E-08		
1	RP11-12A20.7	1.4756E-06	2.13	0.08584339	7.0174E-06		
1	RP11-12G12.7	6.5798E-21	2.17	3.8277E-16	5.1029E-20		
1	RP11-12J10.3	8.4561E-11	-2.96	4.9193E-06	4.9291E-10		
1	RP11-12M5.4	8.0441E-06	-4.18	0.46795516	3.6568E-05		
1	RP11-130F10.	7.0106E-25	-4.52	4.0783E-20	5.8715E-24		
1	RP11-1319K7.	1.0992E-09	3.51	6.3947E-05	6.1123E-09		
1	RP11-131L12.	1.8726E-05	-5.08	1	8.3068E-05		
1	RP11-132A1.4	2.3732E-29	-3.81	1.3806E-24	2.1575E-28		
1	RP11-133F8.2	0.01228717	-6.41	1	0.04264116		
1	RP11-134G8.5	2.8287E-12	-4.47	1.6455E-07	1.7476E-11		
1	RP11-134G8.7	1.148E-20	-3.39	6.6786E-16	8.8634E-20		
1	RP11-135A1.2	0.00063741	-3.99	1	0.0025256		
1	RP11-135F9.4	2.951E-07	3.79	0.01716728	1.4571E-06		
1	RP11-135J2.3	0.00134178	-3.46	1	0.00516966		
1	RP11-1365D1	0.01346521	-1.74	1	0.04654338		
1	RP11-136C24.	1.7196E-05	-9.48	1	7.645E-05		
1	RP11-136O12	4.3273E-11	-11.18	2.5174E-06	2.5493E-10		
1	RP11-1376P1	0.00239117	-1.88	1	0.00897444		
1	RP11-137H2.4	7.752E-27	7.59	4.5096E-22	6.7178E-26		
1	RP11-138A9.1	1.8281E-09	-8.80	0.00010635	1.0069E-08		
1	RP11-1391J7.	0.00019775	1.80	1	0.0008171		
1	RP11-13J10.1	0.00552221	-2.24	1	0.0199931		
1	RP11-13K12.2	3.5573E-06	-11.73	0.20694137	1.6497E-05		
1	RP11-13N12.1	3.115E-21	36.17	1.8121E-16	2.4327E-20		
1	RP11-13N12.2	3.0891E-06	-16.73	0.1797059	1.4391E-05		
1	RP11-140K17.	9.3453E-19	-5.02	5.4365E-14	6.9557E-18		
1	RP11-141C7.3	1.9385E-06	-3.10	0.11276928	9.1556E-06		

1	RP11-141C7.5	0.00308526	-1.58	1	0.0114641		
1	RP11-141M1.:	2.6038E-33	-7.44	1.5147E-28	2.5141E-32		
1	RP11-141M3.(	0.00038094	-29.25	1	0.00153638		
1	RP11-142A23.	6.9985E-13	-85.21	4.0713E-08	4.4143E-12		
1	RP11-142L1.1	0.00016476	-6.61	1	0.00068433		
1	RP11-143J24.:	2.3171E-13	-34.32	1.348E-08	1.4839E-12		
1	RP11-143K11.	0.0109965	1.50	1	0.03840491		
1	RP11-144F15.	0.00083265	1.82	1	0.00326492		
1	RP11-144G6.1	9.4574E-19	2.80	5.5017E-14	7.0373E-18		
1	RP11-144G6.4	1.1422E-43	2.62	6.6448E-39	1.2566E-42		
1	RP11-145A3.1	1.3536E-19	-10.11	7.8747E-15	1.0235E-18		
1	RP11-145A3.2	0.0035383	-22.79	1	0.01307236		
1	RP11-145G20	4.602E-41	-13.68	2.6772E-36	4.8783E-40		
1	RP11-146E13.	8.1405E-07	1.68	0.04735644	3.929E-06		
1	RP11-146I2.1	2.3993E-07	-4.51	0.0139574	1.1906E-06		
1	RP11-146N23	0.00606409	-19.72	1	0.0218408		
1	RP11-147I3.1	7.0883E-06	-2.00	0.41235732	3.2332E-05		
1	RP11-147L13.	6.0371E-33	3.37	3.512E-28	5.8031E-32		
1	RP11-148B18.	1.6333E-30	16.57	9.5014E-26	1.5139E-29		
1	RP11-148B18.	4.8144E-27	24.00	2.8007E-22	4.1946E-26		
1	RP11-148E17.	4.4669E-38	-15.80	2.5986E-33	4.5669E-37		
1	RP11-148I19.:	0.00450415	-2.96	1	0.01646917		
1	RP11-148L24.	4.291E-126	-9.89	2.496E-121	8.53E-125		
1	RP11-149I23.3	1.1654E-07	4.47	0.00677965	5.8831E-07		
1	RP11-14O19.2	1.5382E-06	-48.13	0.08948297	7.3083E-06		
1	RP11-150C16.	5.9566E-09	-9.74	0.00034652	3.2061E-08		
1	RP11-150D5.2	1.0652E-34	-11.94	6.1969E-30	1.048E-33		
1	RP11-150O12	4.2874E-16	-9.45	2.4942E-11	3.0058E-15		
1	RP11-151A6.4	0.01070383	2.19	1	0.03743219		
1	RP11-152F13.	0.00090804	-2.01	1	0.00354861		
1	RP11-152H18	1.5E-05	2.23	0.87258866	6.6978E-05		
1	RP11-153F1.1	0.01331127	-1.59	1	0.04602222		
1	RP11-154D17	0.00210281	-2.13	1	0.00793984		
1	RP11-154D6.1	7.9918E-07	-2.36	0.04649149	3.8585E-06		
1	RP11-154H23	8.9933E-24	28.10	5.2318E-19	7.3687E-23		
1	RP11-156E8.1	0.00437527	-3.41	1	0.01601908		
1	RP11-156K13.	3.111E-108	-21.76	1.81E-103	5.63E-107		
1	RP11-156K23.	0.00028767	-1.66	1	0.00117101		
1	RP11-156P1.3	0	-1.81	0	0		
1	RP11-157F20.	2.6024E-06	-5.90	0.15139044	1.2188E-05		
1	RP11-157L3.1	2.8961E-09	3.30	0.00016848	1.5815E-08		
1	RP11-157P1.4	3.8587E-08	-1.76	0.00224475	1.9969E-07		
1	RP11-158I9.5	0.00069987	-10.67	1	0.00276067		
1	RP11-158I9.8	2.6474E-08	-2.87	0.00154007	1.3835E-07		
1	RP11-158K1.3	1.746E-06	-1.88	0.10157128	8.2693E-06		
1	RP11-158M2.4	1.4834E-05	-3.32	0.86295415	6.6274E-05		
1	RP11-158M2.:	0.00524225	-3.16	1	0.01902689		

1	RP11-159D12	3.3386E-75	3.03	1.9422E-70	4.7626E-74		
1	RP11-159D12	9.992E-11	1.57	5.8128E-06	5.8058E-10		
1	RP11-159F24.	0.00087963	-2.45	1	0.0034422		
1	RP11-159F24.	1.3702E-23	-6.85	7.9709E-19	1.1194E-22		
1	RP11-159F24.	3.0657E-21	-8.58	1.7834E-16	2.3945E-20		
1	RP11-15B17.1	2.2241E-07	-2.34	0.01293871	1.1059E-06		
1	RP11-15B24.5	1.1951E-17	-4.32	6.9526E-13	8.6918E-17		
1	RP11-15E1.5	0.00020955	-2.34	1	0.00086387		
1	RP11-15E18.1	3.732E-11	-2.42	2.1711E-06	2.2048E-10		
1	RP11-15F12.6	0.00060588	-11.91	1	0.00240443		
1	RP11-15H20.6	2.5299E-15	2.23	1.4718E-10	1.727E-14		
1	RP11-15H20.7	1.668E-28	2.65	9.7033E-24	1.496E-27		
1	RP11-15H20.8	2.8993E-16	2.53	1.6866E-11	2.0409E-15		
1	RP11-15I11.2	0.00017068	-3.20	1	0.00070823		
1	RP11-15I11.3	9.3314E-05	-12.47	1	0.000395		
1	RP11-15K19.2	3.1355E-14	2.55	1.8241E-09	2.0669E-13		
1	RP11-15K3.1	2.2466E-79	17.83	1.3069E-74	3.3138E-78		
1	RP11-160H22	4.8213E-07	-7.76	0.02804731	2.3555E-06		
1	RP11-161D15	1.0711E-08	-20.75	0.00062313	5.699E-08		
1	RP11-161D15	9.5098E-09	-17.36	0.00055322	5.0717E-08		
1	RP11-161D15	1.3757E-41	-48.11	8.003E-37	1.4703E-40		
1	RP11-161I10.1	0.00140934	-6.34	1	0.00541277		
1	RP11-161I6.2	9.271E-111	-10.37	5.393E-106	1.708E-109		
1	RP11-162P23.	0.00037055	-4.88	1	0.00149594		
1	RP11-163E9.2	1.6732E-06	-2.44	0.09733941	7.9318E-06		
1	RP11-164J13.1	2.2881E-07	-2.46	0.01331106	1.1367E-06		
1	RP11-164N3.3	3.2406E-43	-5.19	1.8852E-38	3.5416E-42		
1	RP11-164P12.	0.00038996	-3.00	1	0.00157104		
1	RP11-164P12.	6.264E-198	-6.38	3.644E-193	1.569E-196		
1	RP11-165A20.	5.0127E-05	-6.69	1	0.00021609		
1	RP11-166O4.6	0.00672266	-1.79	1	0.02407857		
1	RP11-166P13.	1.51E-37	-4.77	8.7841E-33	1.533E-36		
1	RP11-168K11.	0.00028811	27.96	1	0.00117261		
1	RP11-169E6.1	5.92E-20	-3.97	3.4439E-15	4.5113E-19		
1	RP11-169F17.	0	-11.60	0	0		
1	RP11-16B13.1	0.01058708	-1.83	1	0.03704619		
1	RP11-16D22.2	6.7978E-06	-5.91	0.39545502	3.1045E-05		
1	RP11-16E12.1	0.00792818	2.73	1	0.02819156		
1	RP11-16K12.1	0.01120824	-15.98	1	0.03909979		
1	RP11-170M17	4.0386E-15	-2.23	2.3494E-10	2.7373E-14		
1	RP11-171L9.1	6.2071E-06	-21.25	0.3610895	2.8414E-05		
1	RP11-172F10.	5.3704E-25	32.83	3.1242E-20	4.5056E-24		
1	RP11-172H24	0.00094014	-5.31	1	0.00367057		
1	RP11-173A6.3	0.00859959	-6.39	1	0.03043577		
1	RP11-173D3.1	4.1438E-06	2.63	0.24106224	1.9129E-05		
1	RP11-173M1.	9.8111E-05	-1.76	1	0.00041458		
1	RP11-173M1.	0.00266616	-1.76	1	0.00996347		

1	RP11-174G6.1	1.0221E-13	-4.25	5.9459E-09	6.6294E-13		
1	RP11-174O3.3	4.4773E-19	-2.94	2.6046E-14	3.3513E-18		
1	RP11-175I6.5	2.0732E-49	-12.29	1.2061E-44	2.4136E-48		
1	RP11-175P19.	0.0035383	24.02	1	0.01307236		
1	RP11-177B4.1	0.0006784	3.65	1	0.00267997		
1	RP11-177C12.	3.8919E-09	-2.68	0.00022641	2.1128E-08		
1	RP11-177F15.	0.00081551	-3.84	1	0.00320182		
1	RP11-177G23	2.1232E-05	-4.01	1	9.3877E-05		
1	RP11-177H13	7.5241E-05	3.81	1	0.00032043		
1	RP11-178C3.1	0.00117093	4.85	1	0.00453332		
1	RP11-178C3.2	0.00122315	1.91	1	0.00472981		
1	RP11-178L8.7	5.2445E-06	-3.24	0.30509604	2.4092E-05		
1	RP11-179K3.2	0.00064224	-7.50	1	0.00254369		
1	RP11-17E2.2	0.00014125	2.50	1	0.00058965		
1	RP11-17M16.1	1.0255E-13	7.36	5.9657E-09	6.65E-13		
1	RP11-180K7.1	2.9884E-05	-9.19	1	0.00013085		
1	RP11-180P8.1	0.0005895	4.17	1	0.00234205		
1	RP11-181C3.1	3.0355E-10	-5.69	1.7659E-05	1.7285E-09		
1	RP11-182L21.	6.7492E-10	2.16	3.9263E-05	3.7862E-09		
1	RP11-184A2.3	0.00621609	-2.14	1	0.02235919		
1	RP11-184D12	1.1534E-05	-7.65	0.67096254	5.1876E-05		
1	RP11-184M15	2.3085E-27	-11.25	1.343E-22	2.0277E-26		
1	RP11-186B7.4	5.0236E-14	2.05	2.9224E-09	3.2899E-13		
1	RP11-186N15	0.00632865	-1.58	1	0.02273874		
1	RP11-187C18.	5.1443E-16	4.07	2.9926E-11	3.5922E-15		
1	RP11-187C18.	0.00573247	22.10	1	0.02070536		
1	RP11-187E13.	0.00103503	7.65	1	0.00402756		
1	RP11-18B16.2	1.6464E-20	29.38	9.5779E-16	1.2668E-19		
1	RP11-18H21.1	6.5308E-06	-8.67	0.37992081	2.9856E-05		
1	RP11-18H7.1	4.5919E-08	-1.52	0.00267129	2.3673E-07		
1	RP11-190A12.	0.00232357	1.62	1	0.00872862		
1	RP11-190J1.3	9.2057E-26	-14.98	5.3553E-21	7.8317E-25		
1	RP11-190P13.	0.01264626	-17.83	1	0.04382461		
1	RP11-191G24	4.0853E-09	-4.45	0.00023766	2.2149E-08		
1	RP11-191L17.	8.3855E-05	-2.76	1	0.00035579		
1	RP11-192H23	4.8406E-14	-1.86	2.816E-09	3.1725E-13		
1	RP11-192H23	4.2757E-20	-3.44	2.4873E-15	3.2702E-19		
1	RP11-195C7.1	9.9207E-18	-5.28	5.7713E-13	7.2267E-17		
1	RP11-195C7.3	0.00024672	-29.04	1	0.00101048		
1	RP11-195E2.1	1.9775E-09	19.11	0.00011504	1.0871E-08		
1	RP11-195M16	1.5215E-07	-22.28	0.00885107	7.6368E-07		
1	RP11-196G18	2.0579E-86	-4.94	1.1972E-81	3.2122E-85		
1	RP11-196G18	3.8519E-74	-5.04	2.2408E-69	5.4441E-73		
1	RP11-196G18	4.5645E-71	-26.61	2.6554E-66	6.3043E-70		
1	RP11-1L9.1	1.0315E-17	4.51	6.0007E-13	7.5112E-17		
1	RP11-206L10.	5.746E-07	-1.69	0.03342676	2.7953E-06		
1	RP11-206L10.	0.01092972	-2.59	1	0.03817855		

1	RP11-20E24.1	0.00969779	-1.69	1	0.0340614		
1	RP11-20G13.1	3.6767E-63	-15.19	2.1389E-58	4.7989E-62		
1	RP11-20I23.1	0.0001361	-4.85	1	0.00056846		
1	RP11-210L7.3	6.3859E-13	-2.69	3.7149E-08	4.0323E-12		
1	RP11-210M15	2.3798E-07	13.34	0.01384441	1.1812E-06		
1	RP11-211G23	0.00246679	-21.55	1	0.00924393		
1	RP11-211G23	0	-2.91	0	0		
1	RP11-211N8.2	1.757E-29	8.25	1.0221E-24	1.6024E-28		
1	RP11-212D19	8.9269E-09	3.42	0.00051931	4.7688E-08		
1	RP11-212P7.2	4.694E-10	-1.66	2.7307E-05	2.6507E-09		
1	RP11-213H15	9.1763E-05	-4.66	1	0.00038846		
1	RP11-214F16.	0.00176435	-1.53	1	0.00671286		
1	RP11-214N9.1	0.00198057	-1.71	1	0.0074958		
1	RP11-215P8.3	0.0002009	3.18	1	0.00082972		
1	RP11-216B9.6	1.5993E-07	-1.98	0.0093038	8.0115E-07		
1	RP11-216L13.	1.6771E-07	-1.75	0.00975637	8.3911E-07		
1	RP11-216L13.	3.2111E-17	2.59	1.868E-12	2.3125E-16		
1	RP11-216N14	8.7383E-15	-15.60	5.0834E-10	5.8605E-14		
1	RP11-217B7.3	2.0578E-06	-5.17	0.11971008	9.7081E-06		
1	RP11-217C7.1	1.319E-212	-12.65	7.672E-208	3.43E-211		
1	RP11-217E13.	0.00124709	-4.92	1	0.00481727		
1	RP11-217E22.	6.1089E-98	1235.60	3.5538E-93	1.0367E-96		
1	RP11-218M22	2.48E-111	-6.15	1.442E-106	4.582E-110		
1	RP11-219A15.	2.9828E-10	-78.41	1.7352E-05	1.6989E-09		
1	RP11-219A15.	1.1264E-17	-6.52	6.5529E-13	8.1962E-17		
1	RP11-219B4.7	2.2422E-06	-3.88	0.13043688	1.0546E-05		
1	RP11-21J7.1	0.00120723	-3.14	1	0.0046692		
1	RP11-21L19.1	1.3652E-14	4.01	7.9418E-10	9.0919E-14		
1	RP11-21L23.2	1.3493E-50	-5.25	7.8496E-46	1.5871E-49		
1	RP11-21L23.3	7.7181E-15	-8.42	4.4899E-10	5.1841E-14		
1	RP11-21L23.4	3.2613E-61	-19.24	1.8972E-56	4.1927E-60		
1	RP11-220I1.5	0.00579494	3.71	1	0.02091933		
1	RP11-221J22.1	4.3309E-05	20.02	1	0.00018756		
1	RP11-221N13	2.224E-29	5.39	1.2938E-24	2.0234E-28		
1	RP11-223A3.1	0.00010877	-1.95	1	0.00045814		
1	RP11-225N10	2.0313E-08	-7.87	0.00118167	1.0676E-07		
1	RP11-227D13	3.3461E-21	27.83	1.9466E-16	2.6101E-20		
1	RP11-227D13	0.00249887	2.93	1	0.00936114		
1	RP11-228B15.	1.3263E-27	-4.38	7.7154E-23	1.1704E-26		
1	RP11-229P13.	1.3337E-05	5.41	0.77586641	5.9751E-05		
1	RP11-229P13.	2.2382E-42	119.59	1.3021E-37	2.4198E-41		
1	RP11-22B23.1	5.6319E-15	1.95	3.2763E-10	3.7959E-14		
1	RP11-22C11.2	1.656E-107	-17.08	9.635E-103	2.981E-106		
1	RP11-22H5.2	0.01091281	2.02	1	0.03812406		
1	RP11-230B22.	0.00170395	-1.74	1	0.00649324		
1	RP11-230G5.2	5.5028E-12	-1.80	3.2012E-07	3.3608E-11		
1	RP11-231C18.	0.00128229	1.83	1	0.00494864		

1	RP11-231D20	1.2032E-14	-10.07	6.9994E-10	8.0324E-14		
1	RP11-231E6.1	0.00545029	-1.71	1	0.01974252		
1	RP11-231N3.1	0.00223441	-5.86	1	0.00840999		
1	RP11-232D9.2	6.2523E-09	-10.95	0.00036372	3.36E-08		
1	RP11-232D9.3	8.2601E-26	-8.64	4.8052E-21	7.0324E-25		
1	RP11-235C23.	0.00311733	-3.53	1	0.01157881		
1	RP11-235E17.	5.2448E-05	-2.03	1	0.00022589		
1	RP11-236F9.2	2.0885E-27	7.14	1.215E-22	1.8362E-26		
1	RP11-236L14.	0.00017204	-36.99	1	0.00071346		
1	RP11-237N19	0.00042132	-6.57	1	0.00169292		
1	RP11-23F23.2	0.0065567	2.44	1	0.02350873		
1	RP11-23N2.4	0.00095724	-3.44	1	0.00373511		
1	RP11-241F15.	4.6521E-11	-5.64	2.7063E-06	2.7372E-10		
1	RP11-241F15.	0.00389906	-4.13	1	0.01435259		
1	RP11-241F15.	0.00097836	-3.46	1	0.00381546		
1	RP11-241F15.	1.7451E-11	-5.11	1.0152E-06	1.047E-10		
1	RP11-241G9.3	3.1675E-07	-7.95	0.01842645	1.5612E-06		
1	RP11-242D8.1	1.5422E-10	-1.70	8.9719E-06	8.8971E-10		
1	RP11-244F12.	0.00566412	3.91	1	0.02049285		
1	RP11-244F12.	1.8624E-05	4.62	1	8.2648E-05		
1	RP11-244H3.1	3.1486E-12	-1.69	1.8317E-07	1.9422E-11		
1	RP11-244H3.4	1.2125E-08	1.93	0.00070537	6.4359E-08		
1	RP11-244M2.	2.2969E-08	-1.54	0.00133618	1.2034E-07		
1	RP11-244O19	7.6211E-09	-1.88	0.00044335	4.0813E-08		
1	RP11-245D16	1.3953E-06	-2.18	0.08116746	6.6444E-06		
1	RP11-245P10.	1.0758E-05	-5.12	0.62583554	4.8492E-05		
1	RP11-247A12.	0.00018098	-2.37	1	0.0007496		
1	RP11-248N22	0.00573247	-19.82	1	0.02070536		
1	RP11-249C24.	4.4842E-23	17.49	2.6087E-18	3.6251E-22		
1	RP11-24F11.2	9.4723E-05	-6.27	1	0.00040076		
1	RP11-24M17.	2.3247E-07	-4.88	0.01352399	1.1544E-06		
1	RP11-250B2.3	0.0023755	-1.63	1	0.0089191		
1	RP11-250B2.6	0.00140516	-1.90	1	0.00539816		
1	RP11-251G23	4.0146E-15	-10.71	2.3354E-10	2.722E-14		
1	RP11-252A24.	3.3197E-31	3.09	1.9312E-26	3.1088E-30		
1	RP11-252A24.	0.00748422	-2.89	1	0.02668959		
1	RP11-252K23.	4.5272E-05	3.49	1	0.00019582		
1	RP11-253E3.3	4.0664E-15	1.61	2.3656E-10	2.7558E-14		
1	RP11-254F7.2	0.00036619	-2.83	1	0.00147916		
1	RP11-255E6.6	7.357E-05	-30.96	1	0.0003135		
1	RP11-255G12	0.00059003	-6.03	1	0.00234392		
1	RP11-255H23	8.5971E-06	5.69	0.50013043	3.9021E-05		
1	RP11-256I9.2	7.248E-107	14.16	4.216E-102	1.301E-105		
1	RP11-256I9.3	3.5492E-09	-3.37	0.00020647	1.93E-08		
1	RP11-256L11.	0.00862104	3.81	1	0.03050054		
1	RP11-256L6.3	0.00115796	-6.88	1	0.00448609		
1	RP11-257K9.8	6.2209E-17	8.62	3.619E-12	4.4541E-16		

1	RP11-257P3.3	1.8476E-08	-4.03	0.00107481	9.7365E-08		
1	RP11-258F22.	0.00028431	29.97	1	0.00115757		
1	RP11-259G18	0.00077759	-2.36	1	0.00305769		
1	RP11-259N19	6.515E-13	2.08	3.79E-08	4.1125E-12		
1	RP11-259O2.3	0.00078697	-7.10	1	0.00309352		
1	RP11-25D10.2	0.01173018	-5.23	1	0.04085198		
1	RP11-25H12.1	4.4709E-89	-15.15	2.6009E-84	7.1278E-88		
1	RP11-25I15.3	3.8834E-19	-28.51	2.2591E-14	2.9105E-18		
1	RP11-25K21.6	3.4018E-47	25.46	1.9789E-42	3.8727E-46		
1	RP11-260N14	6.6671E-06	-3.96	0.38785242	3.0468E-05		
1	RP11-261C10.	0.00787849	2.92	1	0.02801658		
1	RP11-261C10.	2.9218E-06	-2.19	0.16997547	1.3635E-05		
1	RP11-262H14	2.5206E-08	-1.83	0.00146631	1.3184E-07		
1	RP11-262H14	8.8396E-40	6.17	5.1423E-35	9.2206E-39		
1	RP11-262H14	1.8119E-06	4.03	0.10540454	8.5764E-06		
1	RP11-264B17.	0.00032146	1.71	1	0.00130364		
1	RP11-264B17.	2.2295E-06	2.00	0.12969875	1.0489E-05		
1	RP11-264J4.1	7.425E-05	-32.72	1	0.00031628		
1	RP11-265N7.1	1.432E-06	-8.19	0.08330801	6.8151E-06		
1	RP11-266A24.	9.856E-05	-2.47	1	0.0004164		
1	RP11-266K4.9	0.00618572	1.93	1	0.02225681		
1	RP11-266L9.1	0.0045063	-19.68	1	0.01647598		
1	RP11-266L9.6	0.0012584	-23.39	1	0.00485967		
1	RP11-267M23	2.3254E-07	-2.13	0.01352758	1.1546E-06		
1	RP11-268G12	8.3688E-16	2.03	4.8685E-11	5.802E-15		
1	RP11-268G12	9.2285E-07	2.02	0.05368596	4.4398E-06		
1	RP11-268J15.	1.9561E-10	-1.86	1.1379E-05	1.1234E-09		
1	RP11-269C23.	1.2533E-09	-9.31	7.2908E-05	6.9516E-09		
1	RP11-269F20.	0.0008329	-5.58	1	0.00326568		
1	RP11-269F21.	9.6062E-19	-10.61	5.5883E-14	7.1443E-18		
1	RP11-269G24	0.00945742	-2.60	1	0.03326135		
1	RP11-26H16.4	0.00433402	-8.23	1	0.01588503		
1	RP11-273G15	1.9901E-40	-18.77	1.1577E-35	2.0928E-39		
1	RP11-274B21.	0.00201246	1.94	1	0.00761105		
1	RP11-274B21.	1.4701E-07	14.64	0.00855209	7.3871E-07		
1	RP11-275N1.1	3.1302E-08	-13.60	0.00182095	1.6288E-07		
1	RP11-276H19	3.7918E-09	-1.59	0.00022058	2.0596E-08		
1	RP11-276H7.2	0.00704879	-1.61	1	0.02519236		
1	RP11-276H7.3	1.1194E-06	-2.69	0.06512258	5.3612E-06		
1	RP11-277A4.4	0.011537	1.86	1	0.04020087		
1	RP11-277P12.	2.163E-128	-17.33	1.258E-123	4.341E-127		
1	RP11-278H7.1	0.00054791	8.50	1	0.00218375		
1	RP11-278L15.	6.2145E-16	-132.35	3.6152E-11	4.3286E-15		
1	RP11-279O17	2.3568E-27	-14.03	1.3711E-22	2.0695E-26		
1	RP11-279O17	6.1544E-09	-9.18	0.00035802	3.3086E-08		
1	RP11-27G14.4	6.743E-16	-6.08	3.9227E-11	4.686E-15		
1	RP11-27K13.3	1.2206E-25	-2.55	7.101E-21	1.0348E-24		

1	RP11-27M24.	0.00942764	19.85	1	0.03316063		
1	RP11-280G9.1	2.012E-12	-7.53	1.1705E-07	1.2497E-11		
1	RP11-281A20.	4.8091E-11	105.16	2.7977E-06	2.8288E-10		
1	RP11-281A20.	2.2236E-30	40.09	1.2935E-25	2.0555E-29		
1	RP11-281P23.	1.3043E-11	-20.07	7.5875E-07	7.8668E-11		
1	RP11-281P23.	8.5844E-44	-6.35	4.9939E-39	9.451E-43		
1	RP11-282A11.	1.6931E-10	-10.96	9.8492E-06	9.7516E-10		
1	RP11-283G6.3	0.01396173	-2.27	1	0.04821093		
1	RP11-284E5.1	5.1197E-75	-9.33	2.9783E-70	7.2926E-74		
1	RP11-284F21.	9.4684E-16	2.66	5.5082E-11	6.5441E-15		
1	RP11-284F21.	1.089E-127	-5.22	6.337E-123	2.18E-126		
1	RP11-284H19	0.00402264	6.73	1	0.01479318		
1	RP11-284M14	6.8814E-27	-5.26	4.0032E-22	5.9687E-26		
1	RP11-285E9.6	3.7197E-08	-2.85	0.0021639	1.9272E-07		
1	RP11-285F7.2	4.8744E-17	-10.73	2.8357E-12	3.4956E-16		
1	RP11-285G1.1	5.9522E-08	10.86	0.00346264	3.0492E-07		
1	RP11-286B14.	4.6238E-95	-11.15	2.6898E-90	7.7029E-94		
1	RP11-286B14.	9.4648E-09	-12.82	0.00055061	5.0487E-08		
1	RP11-286H14	0.01435397	3.55	1	0.04949194		
1	RP11-286N22	0	-2.27	0	0		
1	RP11-286O1.1	8.6011E-05	11.04	1	0.00036477		
1	RP11-287D1.3	0.00231117	-1.91	1	0.00868428		
1	RP11-287D1.4	2.5017E-33	-18.62	1.4553E-28	2.4159E-32		
1	RP11-288I21.1	9.6746E-05	-2.62	1	0.00040914		
1	RP11-289H16	5.4784E-12	2.36	3.187E-07	3.3466E-11		
1	RP11-290F24.	2.8146E-06	2.17	0.16373724	1.3154E-05		
1	RP11-290F5.2	2.036E-16	-11.62	1.1844E-11	1.4422E-15		
1	RP11-290H9.2	1.1211E-06	8.50	0.06522003	5.3683E-06		
1	RP11-290K4.2	1.2778E-06	-4.08	0.07433277	6.1019E-06		
1	RP11-290L1.3	5.4948E-24	141.23	3.1966E-19	4.5188E-23		
1	RP11-291L22.	3.9639E-10	1.81	2.306E-05	2.2467E-09		
1	RP11-292E2.1	9.5794E-09	-10.70	0.00055727	5.1084E-08		
1	RP11-292F9.1	3.3458E-05	5.42	1	0.00014609		
1	RP11-293A21.	6.8777E-08	-7.13	0.00400103	3.5115E-07		
1	RP11-293M10	0.00026326	-7.45	1	0.0010751		
1	RP11-294C11.	0.00942764	19.87	1	0.03316063		
1	RP11-295G12	1.8119E-11	-15.63	1.0541E-06	1.0868E-10		
1	RP11-295G24	3.8421E-06	-4.85	0.22350905	1.7784E-05		
1	RP11-295K2.3	7.3317E-05	-2.06	1	0.00031244		
1	RP11-295K3.1	3.301E-158	-6.85	1.92E-153	7.299E-157		
1	RP11-296E7.1	5.2671E-07	3.15	0.03064069	2.5679E-06		
1	RP11-296O14	3.9325E-18	-2.40	2.2877E-13	2.8881E-17		
1	RP11-297A16.	1.1818E-06	-2.82	0.06874902	5.6532E-06		
1	RP11-297A16.	1.0284E-05	-3.10	0.59828473	4.6429E-05		
1	RP11-297D21	5.4119E-34	-3.83	3.1483E-29	5.2842E-33		
1	RP11-297N6.4	1.7006E-07	-10.55	0.00989284	8.5063E-07		
1	RP11-297P16.	5.1172E-12	-5.78	2.9769E-07	3.1302E-11		

1	RP11-298A10.	5.412E-07	21.75	0.03148402	2.6364E-06		
1	RP11-298A8.2	0.0031767	21.69	1	0.01179255		
1	RP11-298I3.4	3.037E-05	-1.89	1	0.00013292		
1	RP11-298J20.4	0.00900485	1.55	1	0.03178883		
1	RP11-298J23.1	0	-3.29	0	0		
1	RP11-299G20	0.01215349	-1.62	1	0.04222257		
1	RP11-29H23.5	1.896E-217	-10.09	1.103E-212	4.992E-216		
1	RP11-2B6.3	4.4294E-07	-2.80	0.02576746	2.1684E-06		
1	RP11-2E11.5	0.00127664	-23.50	1	0.00492765		
1	RP11-2F9.4	0.00090267	30.14	1	0.00352857		
1	RP11-2I17.4	5.7372E-05	-3.72	1	0.00024648		
1	RP11-2L4.1	2.4372E-11	30.72	1.4178E-06	1.4533E-10		
1	RP11-2N1.2	2.6328E-17	147.65	1.5316E-12	1.9007E-16		
1	RP11-300E4.2	0.00172334	-9.46	1	0.00656369		
1	RP11-301G19	0.00011564	34.11	1	0.00048626		
1	RP11-302M6.4	0.00189111	1.93	1	0.00717727		
1	RP11-304F15.	1.552E-10	2.96	9.0288E-06	8.9518E-10		
1	RP11-304L19.	0.00596413	5.17	1	0.02150338		
1	RP11-304M2.1	0.01173219	-3.52	1	0.04085654		
1	RP11-305B6.3	1.9567E-38	-10.82	1.1383E-33	2.0097E-37		
1	RP11-305L7.7	6.1342E-11	-6.38	3.5685E-06	3.5919E-10		
1	RP11-306O13	2.1529E-05	2.23	1	9.5142E-05		
1	RP11-307B6.3	0.0033264	-6.74	1	0.01232549		
1	RP11-307P5.1	2.4889E-06	-4.06	0.14478867	1.167E-05		
1	RP11-308B16.	8.882E-119	-9.17	5.167E-114	1.709E-117		
1	RP11-309H21	3.8836E-11	-11.26	2.2592E-06	2.2929E-10		
1	RP11-309L24.	3.4754E-41	-4.63	2.0218E-36	3.6935E-40		
1	RP11-30K9.6	7.5877E-24	-3.05	4.4141E-19	6.2284E-23		
1	RP11-30L15.4	0.00360055	-2.72	1	0.01329135		
1	RP11-30O15.1	2.8389E-06	-17.44	0.16515143	1.3262E-05		
1	RP11-310E22.	0.00336358	6.80	1	0.01246005		
1	RP11-310K10.	0.00010301	-31.00	1	0.00043457		
1	RP11-311F12.	2.189E-155	-12.31	1.273E-150	4.794E-154		
1	RP11-311F12.	6.2035E-16	-12.47	3.6088E-11	4.3214E-15		
1	RP11-313E19.	5.6304E-22	7.29	3.2754E-17	4.4564E-21		
1	RP11-313I2.13	2.0026E-05	-17.86	1	8.8661E-05		
1	RP11-313I2.5	2.8781E-09	-22.83	0.00016743	1.5721E-08		
1	RP11-313J2.1	9.045E-232	200.91	5.262E-227	2.424E-230		
1	RP11-313M3.	4.4565E-12	-7.12	2.5925E-07	2.7319E-11		
1	RP11-314A20.	0.00188241	-1.58	1	0.00714845		
1	RP11-314D7.1	0.00149278	-9.42	1	0.00571736		
1	RP11-314D7.2	4.1507E-19	-12.15	2.4146E-14	3.1096E-18		
1	RP11-314N13	1.9654E-07	-5.07	0.01143379	9.7976E-07		
1	RP11-314N14	2.0148E-34	237.82	1.1721E-29	1.9749E-33		
1	RP11-314P15.	1.1549E-05	-3.03	0.67186168	5.1941E-05		
1	RP11-315D16	7.4137E-12	-3.74	4.3129E-07	4.5067E-11		
1	RP11-315O6.1	8.0707E-19	-6.91	4.695E-14	6.0131E-18		

1	RP11-316M1.	3.9558E-09	-2.91	0.00023012	2.1457E-08		
1	RP11-316M21	0.00257014	-2.36	1	0.00961945		
1	RP11-317J19.:	2.598E-17	-5.45	1.5114E-12	1.8758E-16		
1	RP11-317J9.1	1.8659E-05	-17.26	1	8.2784E-05		
1	RP11-317L10.	0.00942764	-19.69	1	0.03316063		
1	RP11-317N12	9.067E-121	-13.67	5.275E-116	1.761E-119		
1	RP11-318A15.	0.00040578	-2.09	1	0.00163285		
1	RP11-318G21	2.6865E-55	-12.63	1.5629E-50	3.3076E-54		
1	RP11-319G6.1	1.0165E-41	-3.30	5.9137E-37	1.0888E-40		
1	RP11-320M2.	0.00491861	1.85	1	0.0179036		
1	RP11-320N21	0.0015724	-5.77	1	0.00601201		
1	RP11-321G12	0.00212691	-8.82	1	0.00802716		
1	RP11-323I15.!	0.00181798	-2.84	1	0.0069101		
1	RP11-323J4.1	2.4989E-06	-1.90	0.14537033	1.1716E-05		
1	RP11-324E6.6	4.8969E-15	-2.29	2.8487E-10	3.3059E-14		
1	RP11-324I22.4	6.7E-155	7.74	3.898E-150	1.464E-153		
1	RP11-324L17.	4.8143E-05	7.50	1	0.00020778		
1	RP11-325B23.	1.1225E-36	-18.10	6.5301E-32	1.1298E-35		
1	RP11-325F22.	4.6996E-12	-4.68	2.7339E-07	2.8775E-11		
1	RP11-325N19	4.5431E-15	-4.57	2.6429E-10	3.0731E-14		
1	RP11-325P15.	0.00119078	-23.48	1	0.00460773		
1	RP11-326A19.	0.00096562	-2.55	1	0.00376703		
1	RP11-326C3.2	2.871E-10	-4.90	1.6702E-05	1.637E-09		
1	RP11-326C3.7	8.3058E-17	9.96	4.8318E-12	5.933E-16		
1	RP11-326I11.3	6.1575E-93	-7.44	3.5821E-88	1.0099E-91		
1	RP11-326I11.4	7.332E-17	-2.90	4.2653E-12	5.2445E-16		
1	RP11-327J17.:	0.00140517	-25.74	1	0.00539816		
1	RP11-327P2.5	1.7056E-08	-2.71	0.00099224	9.0007E-08		
1	RP11-328C8.4	1.2544E-05	-9.18	0.72975386	5.6308E-05		
1	RP11-328K4.1	2.086E-100	-12.64	1.2135E-95	3.593E-99		
1	RP11-328P23.	3.3493E-37	264.63	1.9484E-32	3.388E-36		
1	RP11-329B9.4	3.2031E-05	-4.94	1	0.00013998		
1	RP11-329E24.	2.4009E-17	-14.55	1.3967E-12	1.7351E-16		
1	RP11-32K4.1	2.94E-42	-9.84	1.7103E-37	3.1737E-41		
1	RP11-332J15.:	9.9464E-06	-2.72	0.57862171	4.4931E-05		
1	RP11-332J15.:	1.0387E-07	-4.30	0.00604239	5.2533E-07		
1	RP11-332J15.4	0.008403	-4.78	1	0.02978347		
1	RP11-333E1.1	4.5017E-05	-1.79	1	0.00019474		
1	RP11-334C17.	0.00155474	-1.55	1	0.00594763		
1	RP11-336A10.	1.0635E-25	-3.22	6.1867E-21	9.0317E-25		
1	RP11-336A10.	1.229E-138	-6.82	7.151E-134	2.561E-137		
1	RP11-336K24.	2.3053E-11	-2.35	1.3411E-06	1.3753E-10		
1	RP11-33A14.1	0.00527363	4.29	1	0.019136		
1	RP11-33E12.2	0.00016565	-1.64	1	0.00068783		
1	RP11-33N16.3	1.1135E-05	3.64	0.64778301	5.013E-05		
1	RP11-33O4.1	3.1914E-08	3.89	0.00185656	1.6597E-07		
1	RP11-33O4.2	2.3044E-06	2.38	0.1340589	1.0832E-05		

1	RP11-341D18	0.005325	-9.36	1	0.01931036		
1	RP11-341G23	3.9604E-17	3.78	2.3039E-12	2.8461E-16		
1	RP11-342D14	0.01083312	2.73	1	0.03786156		
1	RP11-342M1.:	0.00483036	-1.59	1	0.01759888		
1	RP11-343C2.9	0.00932424	2.11	1	0.03286448		
1	RP11-343H5.6	0.0125726	-5.32	1	0.0435979		
1	RP11-344P13.	0.00031059	-4.68	1	0.00126111		
1	RP11-344P13.	1.366E-10	-4.05	7.9464E-06	7.9014E-10		
1	RP11-345F18.	1.3161E-07	-10.41	0.00765625	6.6248E-07		
1	RP11-345P4.6	0.01422469	2.55	1	0.04906945		
1	RP11-346D19	1.0045E-16	-6.53	5.8436E-12	7.1621E-16		
1	RP11-346J10.:	0.00047717	-4.81	1	0.00190992		
1	RP11-347C12.	0.00204821	1.59	1	0.00773868		
1	RP11-347C12.	2.731E-14	-13.00	1.5887E-09	1.8037E-13		
1	RP11-347C18.	4.8825E-05	-3.42	1	0.00021057		
1	RP11-347E10.	0.00493138	-4.48	1	0.01794896		
1	RP11-347H15	2.4146E-05	10.29	1	0.00010634		
1	RP11-347I19.8	0.01292871	1.76	1	0.04476342		
1	RP11-347J14.8	0.01115118	17.62	1	0.03891242		
1	RP11-348J24.:	8.5279E-12	-9.07	4.961E-07	5.1731E-11		
1	RP11-34A14.3	6.5158E-34	-14.61	3.7905E-29	6.3503E-33		
1	RP11-34D15.2	0.00781502	-2.77	1	0.02781639		
1	RP11-34F13.2	7.6799E-06	-42.00	0.44677106	3.4945E-05		
1	RP11-34P13.1	6.1704E-05	-2.82	1	0.00026439		
1	RP11-34P13.1	0.00038163	-28.79	1	0.00153907		
1	RP11-34P13.8	0.00530003	3.37	1	0.01922817		
1	RP11-350G8.5	0.00888422	-15.99	1	0.03138202		
1	RP11-351D16	0.00524747	-1.67	1	0.01904461		
1	RP11-351M16	0.0030171	-4.71	1	0.01122231		
1	RP11-351M8.	3.0679E-10	-139.86	1.7847E-05	1.7465E-09		
1	RP11-351M8.:	4.1971E-25	-1485.94	2.4416E-20	3.5299E-24		
1	RP11-352D13	0.00011659	38.46	1	0.00048997		
1	RP11-352M15	0	-3.02	0	0		
1	RP11-353N4.5	6.4046E-19	-26.79	3.7258E-14	4.7828E-18		
1	RP11-353N4.6	0.00051893	-4.03	1	0.00207125		
1	RP11-354P17.	0.00140517	-26.84	1	0.00539816		
1	RP11-355B11.	7.5722E-13	-2.84	4.4051E-08	4.77E-12		
1	RP11-355F16.	0.01078027	-2.05	1	0.03768592		
1	RP11-356C4.5	3.8277E-05	-44.60	1	0.0001664		
1	RP11-356I2.4	1.2394E-11	-2.37	7.2099E-07	7.483E-11		
1	RP11-356J5.1:	2.116E-18	-4.51	1.231E-13	1.5609E-17		
1	RP11-356O9.1	5.0482E-09	3.48	0.00029368	2.7255E-08		
1	RP11-357H14	7.2151E-66	2.63	4.1973E-61	9.6225E-65		
1	RP11-359B12.	4.1982E-38	3.28	2.4422E-33	4.2952E-37		
1	RP11-359D14	6.9851E-06	-53.77	0.40635044	3.1871E-05		
1	RP11-359G22	9.5945E-41	-40.82	5.5815E-36	1.013E-39		
1	RP11-35G9.3	2.0788E-26	3.58	1.2093E-21	1.7895E-25		

1	RP11-35G9.5	1.7032E-29	6.92	9.9085E-25	1.5535E-28		
1	RP11-360L9.7	0.0104389	-2.86	1	0.03655186		
1	RP11-360O19	1.706E-07	-55.21	0.00992436	8.5327E-07		
1	RP11-360P21.	0.00035886	-6.87	1	0.00145054		
1	RP11-361H10	8.6123E-05	2.64	1	0.0003652		
1	RP11-361H10	4.3428E-05	5.88	1	0.00018806		
1	RP11-361L15.	5.8977E-08	-3.66	0.00343093	3.0223E-07		
1	RP11-362F19.	1.4807E-07	-7.62	0.00861354	7.4377E-07		
1	RP11-363E6.3	6.7121E-06	4.38	0.39046699	3.0661E-05		
1	RP11-363E6.4	0.00155272	-2.35	1	0.00594027		
1	RP11-363E7.4	4.8192E-18	-4.26	2.8035E-13	3.5345E-17		
1	RP11-363J20.1	1.5694E-05	-4.19	0.91297561	6.9976E-05		
1	RP11-363J20.1	0.00540623	2.25	1	0.01959147		
1	RP11-363L24.	4.7375E-09	-2.20	0.0002756	2.5609E-08		
1	RP11-364B6.2	6.1043E-68	-3.97	3.5511E-63	8.2411E-67		
1	RP11-365N19	2.7517E-05	3.38	1	0.00012072		
1	RP11-366F6.2	1.222E-108	-20.49	7.106E-104	2.217E-107		
1	RP11-366H4.1	2.0883E-45	94.23	1.2148E-40	2.3353E-44		
1	RP11-366L5.1	2.5428E-05	2.76	1	0.00011179		
1	RP11-367G18	1.4274E-79	-15.49	8.3037E-75	2.1102E-78		
1	RP11-367H1.1	1.473E-11	-8.38	8.5692E-07	8.8699E-11		
1	RP11-367N14	7.7605E-07	-2.56	0.04514618	3.7494E-06		
1	RP11-368L12.	0.00026874	-3.58	1	0.00109643		
1	RP11-368P15.	0.00263306	-6.54	1	0.00984547		
1	RP11-369G6.2	7.1341E-05	36.67	1	0.00030424		
1	RP11-370B11.	0.01228032	-2.71	1	0.04261994		
1	RP11-370B11.	0.00039581	-1.97	1	0.00159371		
1	RP11-370I10.6	8.803E-21	-10.13	5.121E-16	6.8135E-20		
1	RP11-371E8.4	6.1792E-06	1.91	0.35947129	2.8291E-05		
1	RP11-373N22	1.478E-18	-3.86	8.5982E-14	1.0942E-17		
1	RP11-374M1.1	8.5832E-05	2.85	1	0.00036404		
1	RP11-378J18.9	5.198E-06	-2.03	0.30239113	2.3884E-05		
1	RP11-379B8.1	2.039E-08	-3.56	0.00118614	1.0714E-07		
1	RP11-379F4.4	5.6302E-07	-2.71	0.03275302	2.7406E-06		
1	RP11-379H18	2.4552E-78	-3.38	1.4283E-73	3.5851E-77		
1	RP11-379I19.1	0.0001215	-11.86	1	0.00050962		
1	RP11-379J13.1	0.00591461	-4.54	1	0.02133014		
1	RP11-379K17.	4.809E-06	-3.26	0.27975843	2.2133E-05		
1	RP11-37B2.1	1.6962E-55	-3.20	9.8676E-51	2.0919E-54		
1	RP11-37N22.1	0.00045351	3.52	1	0.0018183		
1	RP11-380I10.4	0.00884018	-15.96	1	0.03123783		
1	RP11-380J14.1	1.4037E-57	14.70	8.1657E-53	1.7561E-56		
1	RP11-380O24	0.01122328	-1.98	1	0.03914992		
1	RP11-380P13.	0.00046119	-5.17	1	0.00184801		
1	RP11-381E24.	6.2779E-11	-2.44	3.6521E-06	3.6745E-10		
1	RP11-381K20.	3.2387E-67	-7.73	1.8841E-62	4.3553E-66		
1	RP11-381O7.3	2.4938E-22	3.91	1.4507E-17	1.987E-21		

1	RP11-382A20.	0	-2.57	0	0		
1	RP11-382A20.	2.4476E-50	-3.20	1.4239E-45	2.873E-49		
1	RP11-382A20.	0.00345151	-2.31	1	0.0127752		
1	RP11-382A20.	8.4174E-07	-68.26	0.04896756	4.058E-06		
1	RP11-383C5.3	0.01193309	-8.26	1	0.04150648		
1	RP11-383I23.2	0.0062066	-2.13	1	0.02232779		
1	RP11-383J24.1	0.01176061	4.84	1	0.04095304		
1	RP11-384J4.2	8.4902E-21	-8.74	4.9391E-16	6.5741E-20		
1	RP11-384K6.8	1.3917E-10	-3.80	8.0963E-06	8.0488E-10		
1	RP11-384P7.7	0.00154123	7.22	1	0.00589827		
1	RP11-385D13	8.8527E-34	-10.59	5.15E-29	8.612E-33		
1	RP11-385F7.1	1.5712E-07	-5.22	0.00914013	7.8747E-07		
1	RP11-385J1.3	0.0035383	-23.37	1	0.01307236		
1	RP11-385M4.1	0.0022983	-21.78	1	0.00863761		
1	RP11-386B13.	2.5758E-14	-177.85	1.4984E-09	1.7032E-13		
1	RP11-386G11	0.00100675	-3.31	1	0.0039238		
1	RP11-386G11	1.2916E-21	-4.05	7.5135E-17	1.0158E-20		
1	RP11-387H17	1.3236E-41	58.42	7.7001E-37	1.4152E-40		
1	RP11-388M20	0.0139188	2.98	1	0.04806839		
1	RP11-38H17.1	5.1245E-07	-12.14	0.02981107	2.5003E-06		
1	RP11-38L15.3	2.3733E-61	-7.20	1.3806E-56	3.0558E-60		
1	RP11-38L15.8	1.2177E-08	-4.42	0.00070836	6.4625E-08		
1	RP11-390E23.	4.7901E-20	-2.58	2.7866E-15	3.6589E-19		
1	RP11-390F4.1	5.6503E-07	-8.13	0.03287029	2.7497E-06		
1	RP11-390F4.3	6.763E-141	-12.25	3.934E-136	1.42E-139		
1	RP11-390F4.6	8.6506E-22	-11.44	5.0324E-17	6.8273E-21		
1	RP11-390P24.	8.0313E-13	-2.07	4.6721E-08	5.0531E-12		
1	RP11-392E22.	2.0336E-08	30.69	0.00118303	1.0687E-07		
1	RP11-392E22.	9.1002E-26	1638.73	5.2939E-21	7.7431E-25		
1	RP11-392E22.	8.2959E-22	53.10	4.8261E-17	6.5491E-21		
1	RP11-392O17	1.7944E-22	-4.32	1.0438E-17	1.4337E-21		
1	RP11-392O17	3.2201E-14	-4.05	1.8733E-09	2.122E-13		
1	RP11-393I2.4	8.1403E-05	-4.66	1	0.00034579		
1	RP11-394A14.	1.5409E-13	-10.70	8.964E-09	9.9358E-13		
1	RP11-394I13.2	1.2495E-15	-4.67	7.269E-11	8.6023E-15		
1	RP11-395B7.4	1.6723E-06	-3.04	0.09728596	7.9281E-06		
1	RP11-395I14.2	4.9167E-06	-2.43	0.28602282	2.2614E-05		
1	RP11-395N3.2	7.5311E-45	5.27	4.3811E-40	8.3673E-44		
1	RP11-396B14.	2.1506E-15	-4.07	1.2511E-10	1.4719E-14		
1	RP11-396F22.	2.4732E-28	-4.38	1.4387E-23	2.2121E-27		
1	RP11-397A16.	2.8985E-16	-2.70	1.6861E-11	2.0406E-15		
1	RP11-398G24	7.6633E-42	23.98	4.4581E-37	8.2267E-41		
1	RP11-398H6.1	4.6983E-06	-6.91	0.27331938	2.1634E-05		
1	RP11-398K22.	6.583E-174	-7.40	3.83E-169	1.524E-172		
1	RP11-399B17.	1.8625E-09	-15.08	0.00010835	1.0254E-08		
1	RP11-399E6.1	2.0319E-11	-10.44	1.182E-06	1.2151E-10		
1	RP11-399K21.	3.9571E-66	-8.86	2.302E-61	5.2847E-65		

1	RP11-399K21.	0.00194573	-2.85	1	0.00737161		
1	RP11-399K21.	1.0248E-24	-3.09	5.9618E-20	8.556E-24		
1	RP11-399O19	5.1985E-19	-13.70	3.0242E-14	3.8861E-18		
1	RP11-3B12.2	1.419E-103	-6.38	8.255E-99	2.498E-102		
1	RP11-3B12.5	9.9302E-08	-5.79	0.00577679	5.0268E-07		
1	RP11-3J1.1	3.5273E-45	-21.03	2.052E-40	3.9355E-44		
1	RP11-400F19.	3.4935E-08	-1.80	0.00203231	1.812E-07		
1	RP11-400G3.5	0.00195128	-3.09	1	0.0073912		
1	RP11-400K9.4	1.5026E-18	-6.27	8.7411E-14	1.1121E-17		
1	RP11-400N13	0.00111427	-4.81	1	0.00432315		
1	RP11-400N13	0.00047713	-3.89	1	0.00190988		
1	RP11-401G5.1	2.8392E-08	-10.48	0.00165165	1.4804E-07		
1	RP11-402P6.1	0.00038576	-32.68	1	0.00155475		
1	RP11-402P6.1	0.00942764	-18.06	1	0.03316063		
1	RP11-403I13.5	0.00029655	-8.19	1	0.00120598		
1	RP11-403I13.7	5.4962E-26	-2.63	3.1973E-21	4.6889E-25		
1	RP11-403I13.8	0	-3.18	0	0		
1	RP11-404F10.	7.2728E-06	-5.49	0.42309003	3.3147E-05		
1	RP11-404P21.	3.3226E-45	-12.30	1.9329E-40	3.7085E-44		
1	RP11-405A12.	5.4225E-10	-4.02	3.1545E-05	3.0549E-09		
1	RP11-405F3.4	0.00081001	-10.63	1	0.00318064		
1	RP11-405K6.1	0.00020258	-13.03	1	0.00083635		
1	RP11-405M12	1.8767E-05	5.80	1	8.3245E-05		
1	RP11-406A9.2	3.9323E-34	-7.60	2.2876E-29	3.8447E-33		
1	RP11-406O16	2.1117E-13	-96.63	1.2284E-08	1.3553E-12		
1	RP11-407A16.	0.00995289	-7.59	1	0.03491313		
1	RP11-407N17	3.674E-05	3.76	1	0.00015996		
1	RP11-408B11.	1.7849E-12	-7.67	1.0383E-07	1.11E-11		
1	RP11-40A7.2	0.00411685	-6.36	1	0.01512336		
1	RP11-40E6.1	1.0978E-11	82.15	6.3863E-07	6.6371E-11		
1	RP11-40F6.1	1.9448E-08	-6.50	0.00113139	1.0231E-07		
1	RP11-410L14.	2.114E-34	-3.14	1.2298E-29	2.0718E-33		
1	RP11-411K7.1	0.00090267	-28.20	1	0.00352857		
1	RP11-412D9.4	9.0345E-11	4.53	5.2557E-06	5.261E-10		
1	RP11-412H8.2	3.677E-283	29.30	2.139E-278	1.028E-281		
1	RP11-413N10	9.0776E-05	2.41	1	0.00038442		
1	RP11-414J4.2	9.7307E-07	-19.43	0.05660722	4.676E-06		
1	RP11-415G4.1	7.5967E-08	-10.67	0.00441929	3.8701E-07		
1	RP11-415J8.5	0.00034803	-11.79	1	0.00140834		
1	RP11-416I2.1	2.5963E-40	-8.92	1.5104E-35	2.7258E-39		
1	RP11-417E7.2	5.6079E-06	40.46	0.32623617	2.5739E-05		
1	RP11-417F21.	0.00376021	4.14	1	0.01385173		
1	RP11-417L14.	0.001768	-7.90	1	0.00672583		
1	RP11-417L19.	0.00033124	1.60	1	0.00134198		
1	RP11-419I17.1	1.327E-191	10.81	7.718E-187	3.252E-190		
1	RP11-420A23.	5.7084E-43	-5.31	3.3208E-38	6.2164E-42		
1	RP11-420L9.5	1.4646E-42	9.15	8.5202E-38	1.5866E-41		

1	RP11-421L21.	0	-2.28	0	0		
1	RP11-422J15.1	8.313E-35	-8.56	4.836E-30	8.1897E-34		
1	RP11-422P24.	4.9151E-10	-2.07	2.8593E-05	2.7733E-09		
1	RP11-423H2.3	2.4849E-07	5.10	0.01445559	1.2322E-06		
1	RP11-425D10	0.00211023	-2.38	1	0.00796631		
1	RP11-426C22.	1.011E-114	100.41	5.882E-110	1.9E-113		
1	RP11-426C22.	1.0388E-12	2.89	6.0428E-08	6.5124E-12		
1	RP11-426C22.	0.0011242	5.95	1	0.00435935		
1	RP11-426C22.	7.0667E-10	112.90	4.111E-05	3.9616E-09		
1	RP11-426L16.	0.00016361	-5.50	1	0.00067977		
1	RP11-427M20	4.4762E-05	-5.93	1	0.00019371		
1	RP11-428C19.	6.561E-36	-4.23	3.8168E-31	6.539E-35		
1	RP11-428C19.	6.3031E-12	-12.56	3.6668E-07	3.8424E-11		
1	RP11-428J1.4	1.6884E-22	-2.62	9.8223E-18	1.3507E-21		
1	RP11-428J1.5	2.8749E-26	-4.69	1.6725E-21	2.4686E-25		
1	RP11-428O18	1.8526E-05	1.61	1	8.2224E-05		
1	RP11-428P16.	1.9463E-09	-3.47	0.00011322	1.0701E-08		
1	RP11-429G19	0.00107721	1.81	1	0.00418411		
1	RP11-429J17.1	1.9533E-05	-1.98	1	8.6524E-05		
1	RP11-429J17.7	2.0607E-36	-13.73	1.1988E-31	2.068E-35		
1	RP11-42I10.1	0.00051887	-2.86	1	0.00207115		
1	RP11-430C7.4	4.6626E-05	-6.33	1	0.00020155		
1	RP11-430C7.5	0.00010947	-5.76	1	0.00046092		
1	RP11-430H12	1.4864E-05	-8.20	0.86469052	6.6402E-05		
1	RP11-430K21.	3.9293E-19	-14.54	2.2858E-14	2.9441E-18		
1	RP11-431J24.1	1.0023E-89	2.90	5.8305E-85	1.604E-88		
1	RP11-431K24.	2.0897E-21	3.21	1.2157E-16	1.637E-20		
1	RP11-431M3.	1.4441E-08	-21.57	0.00084012	7.6465E-08		
1	RP11-431M3.1	0.00221501	-25.23	1	0.00834127		
1	RP11-431M7.1	3.4304E-06	-40.25	0.19955916	1.5923E-05		
1	RP11-431M7.1	3.7079E-06	-57.86	0.21570331	1.7179E-05		
1	RP11-432I5.2	1.4057E-20	-5.76	8.1774E-16	1.0827E-19		
1	RP11-432J24.1	0.00075246	-7.09	1	0.00296307		
1	RP11-432J24.5	0.00136615	-25.43	1	0.00525937		
1	RP11-433J20.1	4.8927E-07	-47.96	0.02846307	2.3892E-06		
1	RP11-434D11	1.3093E-05	-2.91	0.76167924	5.869E-05		
1	RP11-434D9.1	0.00064181	-4.20	1	0.00254216		
1	RP11-434D9.2	5.5076E-05	-6.09	1	0.0002368		
1	RP11-434E6.4	4.0397E-09	2.85	0.00023501	2.1906E-08		
1	RP11-434E6.5	2.3479E-06	2.39	0.13658452	1.1032E-05		
1	RP11-434P11.	0.00991361	-5.26	1	0.03477954		
1	RP11-436D10	0.00019879	2.08	1	0.00082127		
1	RP11-436D23	5.2863E-51	-8.28	3.0753E-46	6.2404E-50		
1	RP11-437J19.1	6.2595E-05	-2.94	1	0.00026803		
1	RP11-438B23.	0.00203984	-6.36	1	0.00770858		
1	RP11-438J1.1	1.8191E-11	2.58	1.0582E-06	1.091E-10		
1	RP11-439E19.	2.75E-05	3.40	1	0.00012066		

1	RP11-439K3.1	0.00100048	-6.04	1	0.0039004		
1	RP11-43F13.1	5.107E-155	-4.42	2.971E-150	1.117E-153		
1	RP11-440D17	4.9959E-08	12.03	0.00290633	2.5702E-07		
1	RP11-440D17	6.6096E-05	7.69	1	0.0002826		
1	RP11-440I14.2	0.00025254	-27.19	1	0.0010332		
1	RP11-440L14.	9.1241E-08	-1.59	0.00530783	4.6276E-07		
1	RP11-441O15	8.3001E-16	-1.89	4.8285E-11	5.7565E-15		
1	RP11-442G21	0.00242376	-5.06	1	0.00909147		
1	RP11-442J17.1	3.4635E-09	61.18	0.00020149	1.8846E-08		
1	RP11-444E17.	0.00323497	-3.03	1	0.01199816		
1	RP11-445O16	0.0007582	26.00	1	0.00298486		
1	RP11-445P17.	2.4004E-05	-8.12	1	0.00010574		
1	RP11-445P17.	0.00020578	-4.15	1	0.00084895		
1	RP11-445P19.	7.1716E-06	-17.70	0.41719932	3.2698E-05		
1	RP11-446F17.	0.0048799	-4.34	1	0.01777046		
1	RP11-446H18	6.8523E-06	-4.63	0.39862303	3.1279E-05		
1	RP11-446J8.1	8.3907E-29	-10.69	4.8812E-24	7.5631E-28		
1	RP11-448G15	1.5908E-06	-1.95	0.09254134	7.5507E-06		
1	RP11-449H3.3	1.92E-09	1.74	0.00011169	1.056E-08		
1	RP11-449P15.	6.2476E-09	-43.92	0.00036345	3.3581E-08		
1	RP11-44F14.1	0.00221501	-25.68	1	0.00834127		
1	RP11-44F14.1	9.8828E-16	-15.42	5.7492E-11	6.8272E-15		
1	RP11-44F14.2	1.3383E-30	-8.23	7.7851E-26	1.242E-29		
1	RP11-44F14.8	2.1997E-38	-22.93	1.2797E-33	2.2585E-37		
1	RP11-44F14.9	7.8863E-15	-12.58	4.5878E-10	5.2952E-14		
1	RP11-44F21.5	3.3527E-47	-8.42	1.9504E-42	3.8175E-46		
1	RP11-44M6.7	4.3088E-27	-2.74	2.5066E-22	3.7586E-26		
1	RP11-44N12.2	0.01323553	-7.02	1	0.04577667		
1	RP11-44N12.5	0.00907811	-4.19	1	0.03203774		
1	RP11-44N21.1	1.2824E-31	-3.10	7.4603E-27	1.2072E-30		
1	RP11-44N21.4	2.1048E-08	-28.24	0.00122443	1.1053E-07		
1	RP11-44N22.3	0.00805031	-3.55	1	0.02859088		
1	RP11-451G4.2	0.00874858	-17.82	1	0.0309273		
1	RP11-452C8.1	1.709E-05	-11.06	0.99419824	7.6003E-05		
1	RP11-452H21	0.0007224	2.47	1	0.00284701		
1	RP11-452I5.2	1.5175E-06	-1.81	0.08828014	7.2113E-06		
1	RP11-452K12.	7.9742E-05	-39.74	1	0.00033903		
1	RP11-452L6.6	0.00606559	1.66	1	0.02184484		
1	RP11-452L6.7	0.00086951	-3.15	1	0.00340417		
1	RP11-454P21.	7.156E-07	-6.05	0.04162908	3.4642E-06		
1	RP11-455F5.3	0.00969535	-1.61	1	0.03405696		
1	RP11-455G16	3.3646E-09	-3.31	0.00019573	1.832E-08		
1	RP11-455I9.1	0.00284763	-3.82	1	0.01061639		
1	RP11-456K23.	3.6198E-06	2.84	0.21057709	1.6779E-05		
1	RP11-456N14	5.3948E-08	-5.15	0.00313836	2.7702E-07		
1	RP11-457K10.	9.7944E-24	-10.60	5.6978E-19	8.0183E-23		
1	RP11-457M11	8.7096E-05	2.09	1	0.00036916		

1	RP11-458D21	0.00088457	3.77	1	0.0034608			
1	RP11-458F8.1	2.9256E-07	-3.29	0.0170191	1.4449E-06			
1	RP11-458F8.4	7.5755E-29	-3.23	4.4069E-24	6.8378E-28			
1	RP11-458I7.1	3.4037E-06	-7.80	0.19800443	1.5806E-05			
1	RP11-459E5.1	9.8612E-08	2.63	0.00573663	4.9931E-07			
1	RP11-459O1.2	0.00368845	-4.08	1	0.01359773			
1	RP11-45A17.3	4.8564E-14	-11.51	2.8251E-09	3.1825E-13			
1	RP11-45M22.4	0.00067833	5.99	1	0.00267988			
1	RP11-460B17.	0.00017204	37.22	1	0.00071346			
1	RP11-460B17.	2.6743E-24	15.07	1.5558E-19	2.213E-23			
1	RP11-460E7.8	0.0038618	6.36	1	0.01421966			
1	RP11-460N11	7.1605E-61	33.93	4.1655E-56	9.1792E-60			
1	RP11-460N20	0.00126211	-2.13	1	0.00487336			
1	RP11-462G12	0.00029071	28.02	1	0.0011829			
1	RP11-462G12	0.00397614	4.76	1	0.01462771			
1	RP11-462G2.1	3.5541E-07	-7.06	0.02067589	1.748E-06			
1	RP11-462G22	0.001689	-11.35	1	0.00643837			
1	RP11-462L8.1	0.0002352	2.18	1	0.00096568			
1	RP11-463D19	5.876E-11	3.59	3.4183E-06	3.4431E-10			
1	RP11-463O9.1	0.00484806	-17.84	1	0.01765893			
1	RP11-463O9.9	0.00041214	-11.23	1	0.0016573			
1	RP11-464F9.1	0.00440217	2.19	1	0.01611046			
1	RP11-464F9.2	7.1071E-05	-1.89	1	0.00030314			
1	RP11-464F9.2	0.00038254	-1.81	1	0.00154251			
1	RP11-465B22.	1.4897E-85	2.96	8.666E-81	2.3091E-84			
1	RP11-465B22.	6.909E-169	-10.97	4.019E-164	1.577E-167			
1	RP11-465I4.3	0.00017204	-37.09	1	0.00071346			
1	RP11-466A19.	0.00010321	-14.15	1	0.0004354			
1	RP11-466F5.6	3.0696E-05	-3.51	1	0.0001343			
1	RP11-466F5.8	1.4273E-12	-4.23	8.3032E-08	8.8985E-12			
1	RP11-466G12	3.3601E-09	7.43	0.00019547	1.8297E-08			
1	RP11-466I1.1	1.3443E-10	30.59	7.8201E-06	7.7796E-10			
1	RP11-467D6.1	9.3545E-05	-1.91	1	0.00039595			
1	RP11-467H10	1.3032E-05	-3.40	0.7581329	5.8421E-05			
1	RP11-467J12.4	2.5018E-15	-4.24	1.4554E-10	1.7082E-14			
1	RP11-467L13.	0.0062648	-3.95	1	0.02252603			
1	RP11-467L13.	5.4944E-10	-1.88	3.1963E-05	3.0948E-09			
1	RP11-468E2.4	1.5751E-14	2.95	9.163E-10	1.0478E-13			
1	RP11-468E2.6	1.2683E-05	3.52	0.73780966	5.6903E-05			
1	RP11-468N14	3.6285E-07	-7.94	0.02110839	1.7839E-06			
1	RP11-469N6.1	1.8421E-18	-6.86	1.0716E-13	1.3615E-17			
1	RP11-46A10.5	3.9069E-33	-3.93	2.2728E-28	3.7617E-32			
1	RP11-46C24.7	5.4423E-07	-1.62	0.03165975	2.6509E-06			
1	RP11-46D6.1	0.00011369	-1.61	1	0.00047827			
1	RP11-46F15.2	8.3663E-08	2.82	0.00486702	4.2529E-07			
1	RP11-470L19.	5.8365E-08	-70.11	0.00339535	2.9916E-07			
1	RP11-471B22.	2.8964E-11	-2.92	1.6849E-06	1.722E-10			

1	RP11-472G21	0.01200132	-7.01	1	0.04172134		
1	RP11-472I20.4	1.5697E-09	1.91	9.1317E-05	8.668E-09		
1	RP11-473M20	2.2347E-29	-11.28	1.3E-24	2.0328E-28		
1	RP11-474O21	1.1039E-08	-3.04	0.0006422	5.8702E-08		
1	RP11-475E11.	0.01397543	3.17	1	0.04824967		
1	RP11-475I24.3	2.6636E-59	26.73	1.5495E-54	3.3773E-58		
1	RP11-476K15.	1.6791E-08	-5.91	0.00097682	8.8641E-08		
1	RP11-477D19	3.0951E-06	-1.63	0.18005724	1.4418E-05		
1	RP11-477I4.4	0.00031268	3.38	1	0.00126952		
1	RP11-478P10.	0.00185696	-4.35	1	0.00705458		
1	RP11-479G22	1.1698E-37	3.30	6.8054E-33	1.1891E-36		
1	RP11-479O17	0.00059702	-1.93	1	0.0023709		
1	RP11-479O9.3	0.01232625	-3.00	1	0.04276657		
1	RP11-47G4.2	7.2926E-17	121.44	4.2424E-12	5.2169E-16		
1	RP11-47I22.2	4.047E-25	-4.24	2.3543E-20	3.4041E-24		
1	RP11-47I22.4	0.00307816	-3.34	1	0.01143918		
1	RP11-47P18.1	2.9653E-07	-8.81	0.01725056	1.4638E-06		
1	RP11-480A16.	2.8036E-08	-1.61	0.00163098	1.4625E-07		
1	RP11-480D4.2	0.0032405	-5.14	1	0.01201789		
1	RP11-481E4.2	0.00157615	23.75	1	0.00602594		
1	RP11-481J13.1	7.1996E-20	-13.39	4.1883E-15	5.4777E-19		
1	RP11-482D24	0.00093427	8.10	1	0.00364841		
1	RP11-482G13	4.457E-07	-7.29	0.02592806	2.1808E-06		
1	RP11-482H16	0.00097459	-4.75	1	0.003801		
1	RP11-482M8.	0.00333211	1.61	1	0.01234506		
1	RP11-483I13.2	0.00087258	-8.97	1	0.00341597		
1	RP11-484N16	4.4432E-06	-10.63	0.25848082	2.048E-05		
1	RP11-485G7.5	0.00649989	2.19	1	0.02331944		
1	RP11-486A14.	2.1468E-10	-7.03	1.2489E-05	1.2307E-09		
1	RP11-486A14.	7.7051E-09	-5.61	0.00044823	4.1255E-08		
1	RP11-486L19.	7.443E-209	-22.17	4.33E-204	1.918E-207		
1	RP11-486M23	0.01033476	-2.79	1	0.03621337		
1	RP11-488C13.	5.686E-09	1.50	0.00033078	3.0628E-08		
1	RP11-488C13.	0.0049012	4.75	1	0.01784358		
1	RP11-488C13.	0.00043587	4.45	1	0.00174969		
1	RP11-488L18.	5.4573E-16	-4.31	3.1747E-11	3.8084E-15		
1	RP11-488L18.	0	-3.16	0	0		
1	RP11-48B3.3	8.2202E-06	7.00	0.4782048	3.7354E-05		
1	RP11-48B3.4	1.0062E-07	20.63	0.00585326	5.0911E-07		
1	RP11-48B3.5	0.00031538	8.52	1	0.00127988		
1	RP11-490G2.2	3.4886E-12	90.26	2.0295E-07	2.148E-11		
1	RP11-490M8.	5.2593E-15	3.79	3.0596E-10	3.5481E-14		
1	RP11-493E12.	4.7139E-05	3.10	1	0.00020367		
1	RP11-493L12.	4.8591E-36	-5.30	2.8267E-31	4.8528E-35		
1	RP11-493L12.	4.9552E-26	-14.39	2.8826E-21	4.233E-25		
1	RP11-493L12.	1.6343E-13	-16.65	9.5073E-09	1.0526E-12		
1	RP11-494M8.4	1.903E-11	-33.49	1.1071E-06	1.14E-10		

1	RP11-495P10.	9.7822E-24	-7.67	5.6907E-19	8.0094E-23		
1	RP11-495P10.	2.5086E-15	-22.09	1.4593E-10	1.7126E-14		
1	RP11-496B10.	0.00687228	-2.23	1	0.02458568		
1	RP11-496D24	0.00951806	-16.08	1	0.03346246		
1	RP11-496H1.1	1.5922E-42	-11.08	9.2627E-38	1.7236E-41		
1	RP11-496H1.2	0.00147618	-4.34	1	0.00565527		
1	RP11-496I9.1	3.8164E-16	2.51	2.2201E-11	2.6791E-15		
1	RP11-496N12	0.00247872	-6.00	1	0.00928805		
1	RP11-497E19.	0.00015213	30.09	1	0.00063311		
1	RP11-498C9.1	3.1585E-10	2.59	1.8374E-05	1.7968E-09		
1	RP11-498C9.1	9.4003E-18	2.63	5.4686E-13	6.8503E-17		
1	RP11-498E11.	0.00480685	-5.64	1	0.0175198		
1	RP11-499E18.	4.2241E-28	-7.49	2.4573E-23	3.7677E-27		
1	RP11-499F3.1	2.2496E-27	-6.64	1.3087E-22	1.9763E-26		
1	RP11-499F3.2	2.124E-11	-3.21	1.2356E-06	1.2691E-10		
1	RP11-49I11.1	4.7803E-18	-3.35	2.7809E-13	3.5068E-17		
1	RP11-49I11.3	0.00942764	-19.45	1	0.03316063		
1	RP11-49K24.6	5.8447E-27	2.92	3.4001E-22	5.0785E-26		
1	RP11-4L24.4	1.1281E-27	3.60	6.5628E-23	9.9709E-27		
1	RP11-4O1.2	1.3239E-28	-5.10	7.7015E-24	1.1896E-27		
1	RP11-501E14.	2.605E-06	-12.64	0.15154581	1.22E-05		
1	RP11-502I4.3	1.0065E-06	-1.87	0.05854933	4.8304E-06		
1	RP11-504P24.	0.00028635	-1.92	1	0.00116582		
1	RP11-505K9.4	1.2532E-13	-3.21	7.2902E-09	8.102E-13		
1	RP11-505P4.7	0.00012888	-2.82	1	0.00053937		
1	RP11-506B6.6	2.3605E-05	-14.32	1	0.00010404		
1	RP11-506M12	0.00065504	-2.21	1	0.00259157		
1	RP11-506M13	0.00348644	-1.78	1	0.01289958		
1	RP11-507K12.	0.00630116	3.65	1	0.02264977		
1	RP11-50B3.2	0.00228045	-1.95	1	0.00857384		
1	RP11-50D16.4	0.00030393	-2.07	1	0.00123504		
1	RP11-50I19.2	0.01157349	3.53	1	0.04032294		
1	RP11-510I5.4	1.022E-111	11.24	5.945E-107	1.894E-110		
1	RP11-510N19	2.5018E-21	-32.90	1.4554E-16	1.9573E-20		
1	RP11-511B23.	0.000251	-6.09	1	0.00102727		
1	RP11-511B23.	0.00021021	-1.88	1	0.00086647		
1	RP11-511P7.6	0.00010855	-1.53	1	0.00045725		
1	RP11-513O13	4.4286E-06	6.97	0.25762889	2.0418E-05		
1	RP11-514O12	3.032E-09	-4.34	0.00017638	1.6545E-08		
1	RP11-514P8.8	0.00031305	-3.03	1	0.00127088		
1	RP11-515E23.	1.7463E-28	-21.80	1.0159E-23	1.5658E-27		
1	RP11-516J2.1	2.47E-14	8.16	1.4369E-09	1.6336E-13		
1	RP11-517C16.	0	-2.88	0	0		
1	RP11-517C16.	0.00628732	-2.09	1	0.02260419		
1	RP11-517I3.2	1.5861E-07	3.20	0.00922724	7.9477E-07		
1	RP11-517O13	5.6802E-14	273.64	3.3044E-09	3.714E-13		
1	RP11-517O13	9.6765E-05	3.62	1	0.00040919		

1	RP11-517P14.	3.199E-11	3.52	1.861E-06	1.8968E-10		
1	RP11-519G16	1.5573E-05	-5.13	0.90594958	6.9443E-05		
1	RP11-519M16	1.4157E-20	-14.30	8.2355E-16	1.0902E-19		
1	RP11-51M24.	1.581E-13	-9.81	9.1973E-09	1.0189E-12		
1	RP11-520B13.	0.0028667	-2.92	1	0.01068542		
1	RP11-520H14	0.00071948	-2.71	1	0.00283611		
1	RP11-521B24.	3.7764E-51	8.58	2.1969E-46	4.4606E-50		
1	RP11-521D12	0.00232773	-2.72	1	0.00874253		
1	RP11-521D12	2.3923E-39	-9.92	1.3917E-34	2.4848E-38		
1	RP11-522B15.	6.2519E-27	-3.75	3.637E-22	5.4275E-26		
1	RP11-522I20.3	2.1101E-07	-2.14	0.01227535	1.0503E-06		
1	RP11-524H19	7.9953E-05	-8.57	1	0.0003399		
1	RP11-524K22.	0.00012168	-11.88	1	0.00051027		
1	RP11-525G13	0.0062341	-2.34	1	0.02242119		
1	RP11-525K10.	0.0002396	6.00	1	0.00098267		
1	RP11-526A4.1	1.1437E-14	-8.93	6.6533E-10	7.6422E-14		
1	RP11-526I2.5	0.00016451	-2.10	1	0.00068334		
1	RP11-527N22	8.1253E-11	-3.78	4.7268E-06	4.7396E-10		
1	RP11-528B10.	0.00160612	-3.91	1	0.00613568		
1	RP11-528I4.2	0.00076875	-5.10	1	0.00302519		
1	RP11-529F4.1	5.9038E-05	-5.58	1	0.00025326		
1	RP11-529G21	7.2501E-08	-3.81	0.00421769	3.6958E-07		
1	RP11-529H2.2	1.694E-08	-4.85	0.00098545	8.9416E-08		
1	RP11-530N7.3	0.0035383	-23.21	1	0.01307236		
1	RP11-531F16.	0.00143874	-3.09	1	0.00551802		
1	RP11-532F12.	1.57E-203	-14.30	9.131E-199	3.994E-202		
1	RP11-533E19.	1.8737E-06	-7.72	0.10900077	8.8597E-06		
1	RP11-533E19.	1.2652E-10	-5.35	7.3601E-06	7.3279E-10		
1	RP11-535A19.	1.7941E-05	-2.94	1	7.9692E-05		
1	RP11-536C5.2	0.00883741	-19.61	1	0.03122997		
1	RP11-536G4.2	3.0719E-07	-3.32	0.01787041	1.515E-06		
1	RP11-536K7.3	0.00123656	-1.59	1	0.00477914		
1	RP11-538I12.2	1.6006E-17	4.48	9.3116E-13	1.161E-16		
1	RP11-538I12.3	0.00547512	19.67	1	0.01982629		
1	RP11-539E17.	1.8646E-05	-3.23	1	8.2741E-05		
1	RP11-539G18	0.00131108	-1.55	1	0.00505472		
1	RP11-539I5.1	1.0826E-38	-5.82	6.2977E-34	1.1146E-37		
1	RP11-539L10.	2.8129E-05	2.81	1	0.0001233		
1	RP11-53O19.1	3.4309E-29	-5.91	1.9959E-24	3.1118E-28		
1	RP11-53O19.3	5.9829E-10	-1.84	3.4805E-05	3.3664E-09		
1	RP11-540A21.	1.904E-06	2.10	0.1107614	8.9977E-06		
1	RP11-540D14	0.0084241	-3.13	1	0.02985279		
1	RP11-540O11	8.8878E-20	-3.16	5.1704E-15	6.7543E-19		
1	RP11-541F9.2	0.00266614	-9.41	1	0.00996347		
1	RP11-541M12	2.7205E-05	3.76	1	0.00011942		
1	RP11-541N10	1.026E-06	-2.17	0.05968588	4.9209E-06		
1	RP11-541P9.3	7.8181E-12	-3.36	4.5481E-07	4.75E-11		

1	RP11-542C16.	0.00027285	-4.70	1	0.00111247			
1	RP11-542G1.1	9.378E-08	-16.30	0.00545558	4.7535E-07			
1	RP11-542K23.	1.3698E-05	-4.11	0.79688071	6.1313E-05			
1	RP11-542K23.	8.0298E-05	-7.27	1	0.00034129			
1	RP11-544M22	2.5551E-17	-8.98	1.4864E-12	1.8456E-16			
1	RP11-545D19	2.8547E-07	-7.69	0.01660693	1.4105E-06			
1	RP11-545I5.3	4.077E-05	-1.52	1	0.00017685			
1	RP11-547D13	0.00940694	2.09	1	0.03314392			
1	RP11-548L20.	3.4903E-70	-11.57	2.0305E-65	4.7933E-69			
1	RP11-54A4.2	0.00048139	-3.64	1	0.00192603			
1	RP11-54A9.1	2.2954E-79	9.32	1.3353E-74	3.3848E-78			
1	RP11-54D18.2	7.918E-07	-19.90	0.0460621	3.8238E-06			
1	RP11-54H7.4	0	-3.02	0	0			
1	RP11-54O7.16	2.0427E-06	11.60	0.11883384	9.6393E-06			
1	RP11-54O7.17	7.4074E-26	2.63	4.3092E-21	6.3083E-25			
1	RP11-54O7.18	2.2346E-15	6.56	1.2999E-10	1.5277E-14			
1	RP11-552F3.9	1.1186E-08	-4.10	0.00065076	5.9473E-08			
1	RP11-553A10.	1.0907E-40	-5.77	6.345E-36	1.1503E-39			
1	RP11-553A21.	1.4191E-36	-9.95	8.2557E-32	1.4276E-35			
1	RP11-554F20.	1.3005E-14	-2.12	7.5653E-10	8.6678E-14			
1	RP11-555M1.1	7.4578E-05	-2.80	1	0.00031763			
1	RP11-556I14.2	3.4729E-42	-9.32	2.0203E-37	3.7448E-41			
1	RP11-556N21	0.00017204	-36.44	1	0.00071346			
1	RP11-557H15	0.00191423	-4.91	1	0.00725698			
1	RP11-55L3.1	1.161E-09	-28.51	6.7543E-05	6.4486E-09			
1	RP11-55L4.2	1.3057E-09	-4.00	7.5959E-05	7.2383E-09			
1	RP11-560J1.2	6.5551E-05	-2.20	1	0.00028037			
1	RP11-561B11.	1.5708E-05	2.03	0.91381878	7.0035E-05			
1	RP11-561C5.4	4.5736E-05	-4.09	1	0.00019779			
1	RP11-561N12	4.8522E-80	75.41	2.8227E-75	7.2026E-79			
1	RP11-561O23	5.9162E-08	-7.17	0.00344169	3.0313E-07			
1	RP11-562I5.1	0.00140856	2.66	1	0.00541013			
1	RP11-562I5.2	9.4436E-17	2.92	5.4937E-12	6.7383E-16			
1	RP11-562L8.1	1.9315E-06	-12.22	0.11236508	9.1242E-06			
1	RP11-563D10	2.9916E-09	-17.62	0.00017404	1.6328E-08			
1	RP11-563J2.2	2.4715E-07	-2.32	0.01437789	1.2256E-06			
1	RP11-563J2.3	0.00167896	-3.55	1	0.00640346			
1	RP11-563P16.	2.7585E-08	-7.64	0.00160471	1.4402E-07			
1	RP11-564D11	2.098E-65	-2.71	1.2205E-60	2.7909E-64			
1	RP11-565A3.2	0.00187516	-4.78	1	0.00712187			
1	RP11-566K11.	5.9379E-10	-37.08	3.4543E-05	3.3417E-09			
1	RP11-566K11.	0.00254936	5.37	1	0.00954354			
1	RP11-567J20.1	0.01225337	-16.02	1	0.0425391			
1	RP11-567M16	3.5766E-09	-1.67	0.00020806	1.9445E-08			
1	RP11-567N4.2	0.00254936	-4.99	1	0.00954354			
1	RP11-567N4.3	1.1185E-21	-23.98	6.5069E-17	8.8122E-21			
1	RP11-568K15.	2.921E-185	-13.41	1.699E-180	6.988E-184			

1	RP11-569A11.	0.00085807	-7.59	1	0.00336075		
1	RP11-571L19.	4.2384E-09	-6.70	0.00024656	2.2953E-08		
1	RP11-572M11	9.1252E-09	-3.06	0.00053085	4.872E-08		
1	RP11-573D15	8.9784E-10	4.80	5.2231E-05	5.0111E-09		
1	RP11-573D15	3.9305E-18	-6.73	2.2865E-13	2.887E-17		
1	RP11-573D15	0.00029921	-2.20	1	0.00121645		
1	RP11-573G6.4	8.3738E-07	-6.81	0.04871368	4.0383E-06		
1	RP11-574E24.	7.155E-10	19.47	4.1623E-05	4.0096E-09		
1	RP11-574F21.	0.00483556	-2.00	1	0.01761483		
1	RP11-574F21.	3.6551E-05	2.29	1	0.00015917		
1	RP11-574K11.	0.00077557	2.07	1	0.00305059		
1	RP11-574M7.:	3.5966E-78	-14.41	2.0923E-73	5.2478E-77		
1	RP11-575F12.	3.4215E-85	-7.54	1.9905E-80	5.2994E-84		
1	RP11-575F12.	5.8594E-12	-4.76	3.4086E-07	3.5756E-11		
1	RP11-575F12.	1.3857E-43	-7.79	8.0614E-39	1.5224E-42		
1	RP11-575L7.4	0.01063508	-2.72	1	0.03720072		
1	RP11-576I22.:	0.00889724	-2.28	1	0.0314242		
1	RP11-578F21.	0.00151674	-5.68	1	0.00580684		
1	RP11-57A19.2	1.3385E-06	-11.09	0.07786502	6.3819E-06		
1	RP11-57A19.4	9.4165E-15	2.53	5.478E-10	6.3096E-14		
1	RP11-57C13.3	0.00216025	7.64	1	0.0081473		
1	RP11-57C13.6	0.00086652	2.51	1	0.00339317		
1	RP11-57H14.4	5.7579E-14	-1.73	3.3496E-09	3.7644E-13		
1	RP11-57K17.1	0.0105912	4.85	1	0.03705838		
1	RP11-580I16.:	2.8839E-06	-1.54	0.16776595	1.3465E-05		
1	RP11-583F2.1	0.00022919	-2.05	1	0.00094179		
1	RP11-583F2.7	7.4352E-11	-2.79	4.3254E-06	4.3414E-10		
1	RP11-585P4.5	5.4055E-41	5.26	3.1446E-36	5.7216E-40		
1	RP11-588H23	3.3252E-06	-42.28	0.19343872	1.545E-05		
1	RP11-588K22.	4.1342E-16	-25.60	2.405E-11	2.899E-15		
1	RP11-588L15.	0.00516933	-19.71	1	0.01877508		
1	RP11-58A12.3	1.0301E-71	135.97	5.9928E-67	1.4299E-70		
1	RP11-58C22.1	2.2235E-05	-4.86	1	9.8154E-05		
1	RP11-58O9.2	6.2541E-40	-7.47	3.6382E-35	6.5319E-39		
1	RP11-593F23.	0.01205964	-3.46	1	0.04190905		
1	RP11-594N15	2.2785E-05	3.90	1	0.00010053		
1	RP11-597D13	0.00130733	-4.93	1	0.00504093		
1	RP11-597D13	1.435E-116	-14.76	8.348E-112	2.727E-115		
1	RP11-598D12	2.9066E-11	-21.89	1.6909E-06	1.7275E-10		
1	RP11-598F7.4	0.00025549	-27.19	1	0.00104497		
1	RP11-598F7.5	4.9145E-11	-73.82	2.859E-06	2.8902E-10		
1	RP11-598F7.6	1.7579E-09	-79.18	0.00010227	9.6862E-09		
1	RP11-599J14.:	4.3829E-15	-3.23	2.5497E-10	2.9662E-14		
1	RP11-59D5__	2.7831E-22	167.04	1.6191E-17	2.2142E-21		
1	RP11-59H7.3	3.8749E-07	-2.10	0.02254164	1.9021E-06		
1	RP11-5O24.1	3.2685E-05	-3.08	1	0.00014278		
1	RP11-5P18.10	0.00012854	-6.24	1	0.000538		

1	RP11-600F24.	1.3046E-12	-1.87	7.5891E-08	8.1437E-12		
1	RP11-606D9.1	0.01055455	-7.61	1	0.03693458		
1	RP11-608O21	4.9766E-40	-14.07	2.8951E-35	5.2108E-39		
1	RP11-60A24.3	0.00954577	2.65	1	0.03355585		
1	RP11-60C6.5	0.00942764	-19.65	1	0.03316063		
1	RP11-610P16.	4.7173E-17	-14.88	2.7442E-12	3.3842E-16		
1	RP11-611O2.5	0.00141194	1.55	1	0.00542202		
1	RP11-613D13	7.0835E-71	2.83	4.1207E-66	9.7694E-70		
1	RP11-613M10	5.5743E-45	2.90	3.2428E-40	6.2051E-44		
1	RP11-613M10	0.00032496	3.38	1	0.00131729		
1	RP11-615I2.1	1.2391E-06	-2.50	0.0720808	5.9214E-06		
1	RP11-615I2.2	1.1325E-10	-75.98	6.5883E-06	6.5705E-10		
1	RP11-617F23.	0.00195891	-2.07	1	0.00741914		
1	RP11-618L22.	5.6887E-06	-6.84	0.33093256	2.6099E-05		
1	RP11-618P17.	2.1633E-07	-1.61	0.01258478	1.0764E-06		
1	RP11-619A14.	2.3616E-06	-8.65	0.1373814	1.1093E-05		
1	RP11-61J19.5	8.8539E-18	-7.03	5.1506E-13	6.4561E-17		
1	RP11-61K9.3	3.034E-20	-5.69	1.765E-15	2.3248E-19		
1	RP11-61O11.1	1.886E-100	-12.96	1.0973E-95	3.254E-99		
1	RP11-620J15.1	1.3064E-22	-14.54	7.5998E-18	1.0487E-21		
1	RP11-620J15.1	5.1615E-36	3.07	3.0026E-31	5.153E-35		
1	RP11-621L6.3	0.01282355	-4.29	1	0.04440459		
1	RP11-624L4.1	3.152E-49	-3.79	1.8336E-44	3.6658E-48		
1	RP11-624M8.	8.6726E-10	-3.53	5.0452E-05	4.8442E-09		
1	RP11-626G11	3.0294E-05	-5.11	1	0.00013261		
1	RP11-626G11	0.0068041	-1.60	1	0.02435225		
1	RP11-626G11	3.3643E-06	-2.88	0.19571317	1.563E-05		
1	RP11-626H12	0.00028982	-14.57	1	0.00117934		
1	RP11-626P14.	9.8392E-05	-3.70	1	0.00041574		
1	RP11-626P14.	4.3353E-65	-77.92	2.522E-60	5.7449E-64		
1	RP11-627G18	0.00356241	-2.37	1	0.01315557		
1	RP11-62F24.2	1.5536E-05	40.22	0.90378202	6.9292E-05		
1	RP11-637O19	3.8508E-05	2.78	1	0.00016736		
1	RP11-638I2.6	7.7953E-07	9.58	0.0453481	3.7655E-06		
1	RP11-638I2.9	0.00103978	4.52	1	0.00404524		
1	RP11-63G10.2	0.00573247	22.17	1	0.02070536		
1	RP11-63G10.4	8.751E-232	13.31	5.091E-227	2.346E-230		
1	RP11-642C5.1	6.2068E-07	-8.55	0.03610722	3.0142E-06		
1	RP11-642D21	0.00123993	-23.43	1	0.00479154		
1	RP11-643C9.2	0.00020795	-15.44	1	0.00085766		
1	RP11-644C3.1	5.996E-08	-8.17	0.0034881	3.0705E-07		
1	RP11-644F5.1	6.3033E-13	-2.12	3.6669E-08	3.9805E-12		
1	RP11-645C24.	2.6618E-05	-4.32	1	0.00011692		
1	RP11-646E18.	4.2331E-08	-59.30	0.00246257	2.1855E-07		
1	RP11-649A16.	2.4662E-69	-17.07	1.4347E-64	3.3615E-68		
1	RP11-649A18.	3.8005E-10	-1.72	2.2109E-05	2.1562E-09		
1	RP11-649A18.	0.00056434	3.22	1	0.00224618		

1	RP11-649E7.5	3.8399E-08	-92.68	0.00223384	1.9876E-07		
1	RP11-649G15	0.0002821	-8.58	1	0.00114898		
1	RP11-64C12.3	2.9538E-49	-6.94	1.7183E-44	3.436E-48		
1	RP11-64C12.5	0.00052168	-27.33	1	0.00208163		
1	RP11-64C12.8	8.9512E-06	-4.74	0.5207294	4.0574E-05		
1	RP11-64D22.5	2.1631E-20	19.96	1.2584E-15	1.6603E-19		
1	RP11-64K12.4	0.00352075	5.44	1	0.01302072		
1	RP11-64P14.7	2.0105E-08	-5.94	0.00116961	1.057E-07		
1	RP11-650K20.	0.00651833	3.89	1	0.02337982		
1	RP11-650P15.	0.01129485	-4.78	1	0.0393854		
1	RP11-653J6.1	0.00010375	-2.80	1	0.00043755		
1	RP11-654A16.	8.8065E-07	-3.62	0.05123099	4.2417E-06		
1	RP11-654C22.	0.00290766	5.49	1	0.01083045		
1	RP11-655G22	1.8405E-05	-14.47	1	8.17E-05		
1	RP11-656D10	0.00245672	1.96	1	0.00920965		
1	RP11-656G20	9.9687E-08	-14.55	0.00579916	5.0458E-07		
1	RP11-657O9.1	8.1125E-11	-4.95	4.7193E-06	4.7326E-10		
1	RP11-659O3.1	4.3859E-37	-15.03	2.5515E-32	4.4304E-36		
1	RP11-65B7.2	9.9008E-15	-6.35	5.7597E-10	6.6272E-14		
1	RP11-65E22.2	0.00081675	-12.83	1	0.00320561		
1	RP11-65N13.8	0.00487314	-3.08	1	0.01774697		
1	RP11-660L16.	4.9183E-50	-3.03	2.8612E-45	5.7627E-49		
1	RP11-661A12.	0.00328379	-2.02	1	0.01217225		
1	RP11-661A12.	6.23E-05	-1.81	1	0.00026682		
1	RP11-662B19.	0.00884519	-17.81	1	0.03125364		
1	RP11-664H17	4.4076E-32	-19.59	2.5641E-27	4.1781E-31		
1	RP11-665C16.	0.00170013	-6.37	1	0.00647964		
1	RP11-665G4.1	0.01259321	-2.01	1	0.04366418		
1	RP11-665J16.1	0.00011761	-16.07	1	0.00049405		
1	RP11-666A8.1	2.7351E-94	-12.85	1.5911E-89	4.5292E-93		
1	RP11-666A8.8	1.4553E-12	-2.66	8.4659E-08	9.072E-12		
1	RP11-667F14.	0.00142819	-6.70	1	0.00548155		
1	RP11-669M16	0.00025773	32.22	1	0.00105348		
1	RP11-669N7.2	0.00012379	4.50	1	0.00051875		
1	RP11-66B24.1	0.00016267	-3.39	1	0.00067587		
1	RP11-66B24.2	1.8676E-11	-1.79	1.0864E-06	1.1193E-10		
1	RP11-66B24.4	0	-5.94	0	0		
1	RP11-66B24.8	0.01443689	-2.79	1	0.0497572		
1	RP11-66D17.3	0.00890968	-5.27	1	0.03146432		
1	RP11-66D17.5	0.00517891	3.09	1	0.0188052		
1	RP11-672A2.3	0.00037006	-28.83	1	0.00149406		
1	RP11-672L10.	5.0894E-06	-3.05	0.29607234	2.3392E-05		
1	RP11-675F6.3	0.00026315	-7.19	1	0.00107473		
1	RP11-676B18.	1.5212E-06	-9.52	0.0884953	7.2282E-06		
1	RP11-676J12.1	1.0832E-47	41.22	6.3014E-43	1.2402E-46		
1	RP11-676J15.1	4.0449E-13	88.20	2.3531E-08	2.5703E-12		
1	RP11-676M6.	0.00638277	4.04	1	0.02291705		

1	RP11-677M14	2.7718E-05	-36.55	1	0.00012154		
1	RP11-678G14	9.7654E-06	15.55	0.56808975	4.4151E-05		
1	RP11-679B17.	0.01049483	17.59	1	0.03673886		
1	RP11-679C8.2	9.7461E-12	-2.14	5.6697E-07	5.9041E-11		
1	RP11-680A11.	0.00342363	1.62	1	0.01267366		
1	RP11-680F8.1	8.9029E-27	-8.51	5.1792E-22	7.7059E-26		
1	RP11-680G24	7.8212E-09	5.01	0.00045499	4.1861E-08		
1	RP11-680H20	8.8018E-08	-4.34	0.00512034	4.4676E-07		
1	RP11-681B3.4	1.0086E-08	-2.74	0.00058672	5.3724E-08		
1	RP11-681L8.1	3.6994E-07	-10.76	0.02152073	1.8173E-06		
1	RP11-683L23.	0.00561048	3.17	1	0.02030382		
1	RP11-685F15.	4.094E-11	-87.70	2.3816E-06	2.4145E-10		
1	RP11-685G9.2	2.4402E-05	-9.76	1	0.00010744		
1	RP11-685G9.4	8.7657E-05	-30.93	1	0.00037143		
1	RP11-685M7.:	0.00017045	-1.77	1	0.0007073		
1	RP11-685M7.!	0.00239472	2.89	1	0.0089872		
1	RP11-685N10	1.0876E-18	5.92	6.3272E-14	8.0796E-18		
1	RP11-686D16	0.00114408	-10.10	1	0.00443379		
1	RP11-686D22	0.00043311	2.57	1	0.00173909		
1	RP11-686D22	0.00027136	4.71	1	0.00110661		
1	RP11-686D22	4.7042E-08	2.13	0.00273661	2.4239E-07		
1	RP11-686D22	5.3925E-24	2.34	3.137E-19	4.4358E-23		
1	RP11-686O6.2	6.7069E-13	-4.15	3.9017E-08	4.2331E-12		
1	RP11-687F6.5	0.00282258	7.22	1	0.01052705		
1	RP11-688G15	1.0083E-07	-84.07	0.00586543	5.1008E-07		
1	RP11-689C9.1	5.0088E-05	-7.12	1	0.00021593		
1	RP11-689P11.	5.1049E-29	-8.77	2.9697E-24	4.6193E-28		
1	RP11-68E19.2	0.00409726	-2.16	1	0.01505615		
1	RP11-68L18.1	0.00065441	-2.56	1	0.00258978		
1	RP11-690D19	5.7179E-06	-1.82	0.33263195	2.6229E-05		
1	RP11-692D12	6.429E-36	-3.49	3.74E-31	6.4086E-35		
1	RP11-693N9.2	1.0168E-05	-4.88	0.59151528	4.5914E-05		
1	RP11-694I15.7	4.9247E-31	-4.87	2.8649E-26	4.6001E-30		
1	RP11-696N14	5.8012E-59	-3.97	3.3748E-54	7.3445E-58		
1	RP11-697E2.1	1.4545E-06	-1.73	0.08461486	6.9198E-06		
1	RP11-697E22.	0.00476979	2.66	1	0.01738909		
1	RP11-697M17	2.5681E-07	-6.61	0.01493941	1.2725E-06		
1	RP11-69E11.4	3.5292E-27	-4.72	2.0531E-22	3.0851E-26		
1	RP11-69H14.6	8.1621E-45	56.38	4.7482E-40	9.0632E-44		
1	RP11-69I8.2	0.00070149	2.89	1	0.00276686		
1	RP11-6J21.2	0.00024652	-4.54	1	0.00100974		
1	RP11-6N13.1	0.01160277	-1.98	1	0.04041552		
1	RP11-6N17.4	7.1569E-05	-1.59	1	0.00030517		
1	RP11-6N17.9	8.4888E-12	-1.50	4.9383E-07	5.1499E-11		
1	RP11-700E23.	8.182E-07	-11.45	0.04759787	3.9481E-06		
1	RP11-700N1.1	2.6861E-06	-14.74	0.15626082	1.2567E-05		
1	RP11-701H24	7.8777E-08	87.31	0.00458278	4.0098E-07		

1	RP11-702C7.1	2.3766E-05	-7.69	1	0.00010472		
1	RP11-702H23	0.00146619	-3.57	1	0.00561924		
1	RP11-703M24	0.00631479	5.10	1	0.02269175		
1	RP11-705C15.	0.00176569	-5.16	1	0.0067175		
1	RP11-705O1.8	0.00013236	2.04	1	0.00055341		
1	RP11-705O24	0.0094633	-3.73	1	0.03328003		
1	RP11-706C16.	0.00460298	-19.68	1	0.01681784		
1	RP11-706C16.	4.7272E-63	-19.20	2.75E-58	6.1673E-62		
1	RP11-706D8.3	0.00776947	-7.61	1	0.02766442		
1	RP11-706O15	1.456E-168	19.47	8.473E-164	3.32E-167		
1	RP11-706O15	0.00799213	17.61	1	0.02839986		
1	RP11-707A18.	2.2625E-07	-47.76	0.0131618	1.1244E-06		
1	RP11-707G18	0.00026739	-5.39	1	0.00109106		
1	RP11-707P17.	1.2774E-06	-7.26	0.07431345	6.1008E-06		
1	RP11-707P17.	0.00010411	-2.83	1	0.00043898		
1	RP11-708J19.1	4.5989E-07	1.80	0.02675348	2.2488E-06		
1	RP11-709P2.1	0.00942764	-19.59	1	0.03316063		
1	RP11-70C1.3	1.8741E-06	6.29	0.10902277	8.8608E-06		
1	RP11-70P17.1	0.00436602	-3.24	1	0.01598921		
1	RP11-711C17.	2.1984E-07	-51.43	0.01278902	1.0935E-06		
1	RP11-711K1.7	8.0483E-09	-105.58	0.0004682	4.3033E-08		
1	RP11-712B9.2	4.4797E-12	-5.05	2.606E-07	2.7455E-11		
1	RP11-712L6.5	6.067E-07	2.06	0.03529396	2.9475E-06		
1	RP11-718O11	0.01071083	2.74	1	0.03745443		
1	RP11-719K4.3	0.01219299	-6.08	1	0.04233704		
1	RP11-719K4.6	0.00942764	-17.83	1	0.03316063		
1	RP11-71N10.1	1.084E-09	-13.57	6.3063E-05	6.0297E-09		
1	RP11-720L8.1	5.93E-13	-10.26	3.4497E-08	3.7493E-12		
1	RP11-722E23.	7.4751E-08	-2.64	0.00434859	3.8085E-07		
1	RP11-722G7.1	4.195E-106	114.51	2.44E-101	7.494E-105		
1	RP11-722M1.	6.3696E-24	-12.07	3.7055E-19	5.2322E-23		
1	RP11-723O4.2	0.00284393	1.61	1	0.01060394		
1	RP11-725M22	1.4188E-06	-16.73	0.08253946	6.7545E-06		
1	RP11-726G1.1	8.4096E-34	-4.08	4.8922E-29	8.1851E-33		
1	RP11-727A23.	0.00108147	-2.19	1	0.00419981		
1	RP11-727A23.	0.00173964	-2.18	1	0.00662185		
1	RP11-728G15	5.1865E-09	16.40	0.00030172	2.7989E-08		
1	RP11-728K20.	0.00947802	-5.27	1	0.03332774		
1	RP11-729M20	0.00563571	-5.23	1	0.02039262		
1	RP11-72I8.2	7.4327E-05	3.53	1	0.00031658		
1	RP11-730K11.	0.00055835	-25.32	1	0.00222368		
1	RP11-731D1.4	2.1204E-14	-8.52	1.2335E-09	1.4048E-13		
1	RP11-732A19.	0.01173018	5.71	1	0.04085198		
1	RP11-734I18.1	8.531E-175	-13.36	4.963E-170	1.983E-173		
1	RP11-734K21.	6.904E-49	-7.83	4.0163E-44	7.9975E-48		
1	RP11-734K21.	0.0031992	-8.83	1	0.0118723		
1	RP11-734K21.	9.872E-193	-9.61	5.743E-188	2.429E-191		

1	RP11-735G4.1	0.00037752	-27.15	1	0.00152324		
1	RP11-736E3.1	2.2128E-06	-5.06	0.12872794	1.0413E-05		
1	RP11-736E3.3	3.998E-06	-46.28	0.23257784	1.8483E-05		
1	RP11-736G13	5.2651E-11	-77.84	3.0629E-06	3.0932E-10		
1	RP11-736K20.	6.6983E-06	-1.85	0.38966756	3.0601E-05		
1	RP11-739L10.	0.0093746	-2.01	1	0.03303597		
1	RP11-739N10	5.6008E-09	-8.90	0.00032582	3.018E-08		
1	RP11-739N20	1.9763E-22	-2.15	1.1497E-17	1.5778E-21		
1	RP11-739P1.2	0.00367131	-5.05	1	0.01353883		
1	RP11-73K9.2	1.6256E-12	-3.28	9.4569E-08	1.0123E-11		
1	RP11-73M18.:	8.872E-15	2.20	5.1612E-10	5.9475E-14		
1	RP11-73M7.1	6.9467E-12	-5.36	4.0411E-07	4.2285E-11		
1	RP11-73M7.6	0.00063717	-2.53	1	0.00252497		
1	RP11-742B18.	2.033E-72	-12.56	1.1827E-67	2.8375E-71		
1	RP11-744N12	1.9528E-08	-15.80	0.00113603	1.0272E-07		
1	RP11-745C15.	0.00142234	-3.55	1	0.00545979		
1	RP11-745L13.	0.00970221	-2.39	1	0.03407489		
1	RP11-746B8.1	4.3589E-05	-8.91	1	0.00018874		
1	RP11-746P2.3	2.8865E-06	46.79	0.16791643	1.3476E-05		
1	RP11-747E23.	0.00022166	-31.24	1	0.00091196		
1	RP11-74J13.8	1.1844E-16	-2.63	6.89E-12	8.4261E-16		
1	RP11-74M13.:	1.172E-06	-5.19	0.06817912	5.6078E-06		
1	RP11-752G15	0.00125742	-1.97	1	0.00485653		
1	RP11-752L20.	0.00942764	-19.52	1	0.03316063		
1	RP11-753H16	0.00359891	-3.38	1	0.01328614		
1	RP11-753H16	2.7261E-06	-60.50	0.15858661	1.275E-05		
1	RP11-754B17.	0.00607689	-3.11	1	0.02187876		
1	RP11-755F10.	4.3678E-07	-1.84	0.02540936	2.1388E-06		
1	RP11-756H6.1	0.01081624	-5.30	1	0.03780938		
1	RP11-756P10.	1.6565E-16	9.73	9.6366E-12	1.1751E-15		
1	RP11-756P10.	1.3512E-15	3.43	7.8603E-11	9.2922E-15		
1	RP11-757F18.	4.9411E-14	-2.61	2.8744E-09	3.237E-13		
1	RP11-757G1.5	3.4108E-11	-7.15	1.9842E-06	2.0198E-10		
1	RP11-757G1.6	5.835E-12	-11.97	3.3944E-07	3.5611E-11		
1	RP11-758H9.2	2.3861E-24	-4.36	1.3881E-19	1.9773E-23		
1	RP11-758I14.2	0.00466893	-8.24	1	0.01704703		
1	RP11-758M4.4	3.027E-07	-2.54	0.01760956	1.4933E-06		
1	RP11-759A24.	2.4342E-16	-42.67	1.4161E-11	1.7162E-15		
1	RP11-761I4.4	0.00025632	-34.65	1	0.001048		
1	RP11-766N7.4	6.634E-05	13.82	1	0.00028362		
1	RP11-770E5.1	7.1122E-19	-2.67	4.1374E-14	5.3024E-18		
1	RP11-770G2.2	8.8613E-24	-5.41	5.155E-19	7.2626E-23		
1	RP11-770J1.3	0.00013559	-3.32	1	0.00056635		
1	RP11-770J1.5	5.1407E-08	-9.37	0.00299057	2.6423E-07		
1	RP11-771K4.1	8.44E-06	-10.80	0.49098907	3.8323E-05		
1	RP11-774D14	1.06E-09	-2.85	6.1665E-05	5.9009E-09		
1	RP11-774O3.1	0.00740209	-4.13	1	0.02640963		

1	RP11-774O3.2	0.00159038	-4.57	1	0.00607715		
1	RP11-774O3.3	9.0026E-11	-3.35	5.2372E-06	5.244E-10		
1	RP11-775B15.	0.00573247	-21.61	1	0.02070536		
1	RP11-775C24.	6.4998E-09	-13.35	0.00037812	3.4911E-08		
1	RP11-775C24.	3.9448E-16	-4.70	2.2948E-11	2.7682E-15		
1	RP11-775H9.2	6.05E-07	-18.69	0.03519501	2.9395E-06		
1	RP11-778D9.9	0.00459822	-17.84	1	0.01680255		
1	RP11-779O18	0.00026703	4.35	1	0.00108975		
1	RP11-77I22.2	2.0096E-11	-2.43	1.169E-06	1.2024E-10		
1	RP11-77K12.1	3.2539E-15	-3.10	1.8929E-10	2.2147E-14		
1	RP11-77K12.5	0.00025814	1.71	1	0.00105501		
1	RP11-77K12.7	3.6326E-07	5.55	0.02113255	1.7854E-06		
1	RP11-783K16.	1.9397E-18	-2.22	1.1284E-13	1.4332E-17		
1	RP11-783K16.	0.0018855	-2.71	1	0.00715716		
1	RP11-783K16.	9.404E-10	-1.97	5.4707E-05	5.2437E-09		
1	RP11-789C1.2	0.00762582	-7.62	1	0.02717789		
1	RP11-789C2.1	0.005375	-3.93	1	0.01948316		
1	RP11-78F17.1	9.817E-122	-14.49	5.711E-117	1.918E-120		
1	RP11-78L16.1	1.3102E-22	-7.32	7.6221E-18	1.0516E-21		
1	RP11-78O22.1	0.01193309	-8.27	1	0.04150648		
1	RP11-78O7.2	0.01019764	2.15	1	0.03574365		
1	RP11-791G15	0.00511232	2.32	1	0.01857963		
1	RP11-793H13	0.00020163	1.51	1	0.00083249		
1	RP11-796E2.4	0.00088963	-2.62	1	0.00347967		
1	RP11-797H7.1	0.00199502	-2.15	1	0.00754899		
1	RP11-797H7.5	1.0767E-26	-17.77	6.2637E-22	9.3099E-26		
1	RP11-797M17	0.01441237	2.61	1	0.04967561		
1	RP11-798G7.5	0.00014703	-4.93	1	0.0006128		
1	RP11-798K3.2	2.6868E-69	-4.66	1.563E-64	3.6605E-68		
1	RP11-798K3.4	0.00875422	-2.79	1	0.03094538		
1	RP11-798M19	2.9808E-22	-2.83	1.734E-17	2.3705E-21		
1	RP11-799D4.4	8.2729E-26	-3.46	4.8127E-21	7.0423E-25		
1	RP11-79E3.3	3.7468E-52	-15.96	2.1797E-47	4.4757E-51		
1	RP11-79H23.3	6.1673E-05	-5.48	1	0.00026427		
1	RP11-79N23.1	2.6685E-47	-6.31	1.5524E-42	3.0421E-46		
1	RP11-79P5.9	0.00067845	-1.73	1	0.00267997		
1	RP11-7F17.1	1.0283E-47	-16.15	5.982E-43	1.1776E-46		
1	RP11-7F17.5	5.2519E-05	-4.01	1	0.00022616		
1	RP11-7F18.2	0.00201543	1.83	1	0.00762176		
1	RP11-7K24.3	1.53E-109	-14.01	8.901E-105	2.795E-108		
1	RP11-800A3.2	1.277E-12	-10.36	7.4285E-08	7.9765E-12		
1	RP11-800A3.4	0	-10.28	0	0		
1	RP11-802E16.	6.5646E-09	-1.79	0.00038189	3.5243E-08		
1	RP11-804N13	0.00167007	-2.59	1	0.00637036		
1	RP11-806L2.2	0.00861975	-2.01	1	0.03050054		
1	RP11-806O11	4.0762E-14	-32.72	2.3713E-09	2.6776E-13		
1	RP11-807H17	0.00130369	-23.47	1	0.00502855		

1	RP11-807H22	7.188E-07	-45.88	0.04181551	3.4791E-06		
1	RP11-807H22	1.9337E-10	-9.63	1.1249E-05	1.1109E-09		
1	RP11-809O17	3.9649E-09	-2.61	0.00023065	2.1504E-08		
1	RP11-80A15.1	0.00555486	1.84	1	0.02010756		
1	RP11-80H18.3	0.00104284	1.69	1	0.00405657		
1	RP11-80H5.9	1.4997E-11	-2.13	8.7243E-07	9.0248E-11		
1	RP11-80P20.3	3.8149E-10	-4.58	2.2193E-05	2.1639E-09		
1	RP11-810P12.	3.8114E-28	-4.86	2.2172E-23	3.4012E-27		
1	RP11-810P8.1	6.0746E-49	-19.33	3.5338E-44	7.0409E-48		
1	RP11-813I20.2	0.01110217	4.85	1	0.03875301		
1	RP11-815M8.	8.2073E-06	-4.26	0.4774538	3.7298E-05		
1	RP11-816J6.3	3.7046E-09	-4.39	0.00021551	2.0134E-08		
1	RP11-818F20.	6.7085E-19	4.88	3.9026E-14	5.0053E-18		
1	RP11-81A22.4	2.8706E-18	3.30	1.6699E-13	2.1133E-17		
1	RP11-81A22.5	0.00534087	1.90	1	0.01936667		
1	RP11-81H3.2	5.392E-183	-9.17	3.137E-178	1.282E-181		
1	RP11-82L18.2	5.395E-143	22.31	3.139E-138	1.143E-141		
1	RP11-82O18.2	0.00024087	-4.17	1	0.00098763		
1	RP11-832A4.7	9.8407E-73	-12.11	5.7247E-68	1.3765E-71		
1	RP11-834C11.	1.9007E-31	68.52	1.1057E-26	1.7857E-30		
1	RP11-834C11.	6.022E-05	14.09	1	0.0002582		
1	RP11-834C11.	8.472E-258	100.49	4.929E-253	2.334E-256		
1	RP11-837J7.3	0.00230043	-1.76	1	0.00864503		
1	RP11-838N2.5	5.0572E-21	-25.64	2.942E-16	3.9311E-20		
1	RP11-839D17	4.5531E-10	26.36	2.6487E-05	2.5726E-09		
1	RP11-83A24.2	4.6225E-15	-2.05	2.6891E-10	3.1254E-14		
1	RP11-845M18	2.2055E-05	-17.22	1	9.7391E-05		
1	RP11-845M18	9.7307E-07	-19.27	0.05660722	4.676E-06		
1	RP11-847H18	0.00698722	2.77	1	0.02498307		
1	RP11-848G14	1.7953E-24	-7.86	1.0444E-19	1.4926E-23		
1	RP11-848P1.4	0.00171944	3.54	1	0.00655011		
1	RP11-848P1.9	6.3496E-37	-4.71	3.6938E-32	6.404E-36		
1	RP11-849H4.2	0	-2.70	0	0		
1	RP11-849H4.4	1.6143E-06	-2.59	0.0939092	7.6607E-06		
1	RP11-849N15	0.00010718	-2.25	1	0.00045167		
1	RP11-84A14.7	9.9974E-14	-5.80	5.8159E-09	6.4866E-13		
1	RP11-84G21.1	6.2279E-08	-1.61	0.00362301	3.1865E-07		
1	RP11-85G18.6	0.00019514	-2.37	1	0.00080661		
1	RP11-861A13.	0.00216339	-4.33	1	0.00815743		
1	RP11-861E21.	0.00303073	-2.10	1	0.01126939		
1	RP11-861E21.	4.1151E-14	-2.54	2.3939E-09	2.7025E-13		
1	RP11-863K10.	1.6801E-10	30.69	9.7737E-06	9.6788E-10		
1	RP11-863K10.	1.8491E-10	9.65	1.0757E-05	1.0634E-09		
1	RP11-863P13.	1.9121E-16	119.44	1.1123E-11	1.3549E-15		
1	RP11-863P13.	1.016E-221	59.71	5.91E-217	2.693E-220		
1	RP11-864I4.1	6.8302E-06	-1.51	0.39734067	3.1186E-05		
1	RP11-867G2.6	1.0083E-07	86.70	0.00586543	5.1008E-07		

1	RP11-867G2.8	0.00627522	2.86	1	0.02256209		
1	RP11-867G23	1.1756E-20	-3.85	6.8389E-16	9.0725E-20		
1	RP11-867O8.5	5.9586E-13	3.58	3.4664E-08	3.767E-12		
1	RP11-86A5.1	0.00942764	19.42	1	0.03316063		
1	RP11-874J12.4	5.0051E-13	-2.73	2.9117E-08	3.1707E-12		
1	RP11-875O11	2.9461E-43	-6.04	1.7139E-38	3.221E-42		
1	RP11-875O11	4.6114E-11	-3.78	2.6827E-06	2.7141E-10		
1	RP11-876N24	0.00065571	2.21	1	0.00259385		
1	RP11-87G24.3	0.00030968	8.52	1	0.00125755		
1	RP11-881M11	0.00025196	-3.08	1	0.00103099		
1	RP11-883A18.	2.5554E-12	13.59	1.4866E-07	1.5815E-11		
1	RP11-885B4.1	3.9549E-05	-2.26	1	0.00017173		
1	RP11-886H22	0.00082034	3.74	1	0.00321926		
1	RP11-888D10	7.7201E-15	23.00	4.4911E-10	5.1848E-14		
1	RP11-88E10.5	1.0214E-09	-3.66	5.9418E-05	5.6903E-09		
1	RP11-890B15.	2.1399E-17	-8.61	1.2449E-12	1.5474E-16		
1	RP11-894J14.5	8.4283E-08	-1.59	0.00490306	4.2836E-07		
1	RP11-894P9.1	1.808E-06	-1.58	0.10517719	8.5586E-06		
1	RP11-895M11	3.9792E-12	22.43	2.3149E-07	2.4431E-11		
1	RP11-89K10.1	2.617E-27	-6.94	1.5224E-22	2.2952E-26		
1	RP11-89K11.1	4.5765E-10	27.79	2.6624E-05	2.5856E-09		
1	RP11-8L2.1	7.9558E-23	-4.32	4.6282E-18	6.4094E-22		
1	RP11-900F13.	1.7785E-16	-9.74	1.0346E-11	1.2605E-15		
1	RP11-902B17.	3.5438E-42	-14.90	2.0616E-37	3.8206E-41		
1	RP11-907D1.1	5.7765E-05	38.83	1	0.00024807		
1	RP11-907D1.3	2.0696E-05	40.88	1	9.1548E-05		
1	RP11-90H3.2	0.00462584	-17.87	1	0.01689712		
1	RP11-90L1.8	1.9462E-07	-3.26	0.01132184	9.7025E-07		
1	RP11-927P21.	0.0007594	-3.47	1	0.00298922		
1	RP11-927P21.	0.00279155	-8.78	1	0.01041397		
1	RP11-92G12.3	1.5247E-26	-5.62	8.8696E-22	1.3148E-25		
1	RP11-932A10.	0.00942764	19.77	1	0.03316063		
1	RP11-932O9.7	3.7623E-12	-2.32	2.1887E-07	2.3127E-11		
1	RP11-93B14.1	5.8937E-05	9.41	1	0.00025285		
1	RP11-93B14.6	0.01329295	7.57	1	0.04596709		
1	RP11-93G5.1	0.00010744	-1.95	1	0.00045273		
1	RP11-93I21.3	3.629E-38	9.00	2.1111E-33	3.7148E-37		
1	RP11-944L7.4	2.9857E-16	1.64	1.7369E-11	2.1013E-15		
1	RP11-945A11.	3.0789E-37	-7.07	1.7911E-32	3.1161E-36		
1	RP11-945A11.	0.00921317	-17.82	1	0.03248483		
1	RP11-946L16.	3.7249E-05	-4.76	1	0.00016208		
1	RP11-949J7.8	2.0493E-25	-3.70	1.1922E-20	1.7336E-24		
1	RP11-94B19.7	0.00062471	-25.33	1	0.00247662		
1	RP11-94I2.4	0.00418626	-1.69	1	0.01536573		
1	RP11-94P11.4	4.5745E-62	-10.72	2.6612E-57	5.9268E-61		
1	RP11-950C14.	0.00479592	-1.70	1	0.01748106		
1	RP11-956E11.	0.00143555	-11.15	1	0.00550722		

1	RP11-958N24	1.4238E-06	1.68	0.08282906	6.7765E-06		
1	RP11-958N24	2.6688E-65	10.13	1.5525E-60	3.5446E-64		
1	RP11-95D17.1	6.6495E-10	-1.84	3.8683E-05	3.7332E-09		
1	RP11-95I16.2	0.00045398	-5.82	1	0.00181984		
1	RP11-95M15.	0.00048277	-7.10	1	0.00193127		
1	RP11-962G15	1.6543E-24	-9.79	9.6236E-20	1.377E-23		
1	RP11-964E11.	3.784E-26	-13.36	2.2013E-21	3.2419E-25		
1	RP11-964E11.	1.7975E-17	-4.32	1.0457E-12	1.3026E-16		
1	RP11-967K21.	1.6308E-06	-3.51	0.09486775	7.7367E-06		
1	RP11-968A15.	0.00052565	-3.46	1	0.00209649		
1	RP11-96B2.1	0.00262724	-21.53	1	0.00982559		
1	RP11-96C23.1	2.1393E-06	-5.33	0.12445294	1.0082E-05		
1	RP11-96C23.1	1.622E-08	-5.46	0.00094357	8.5678E-08		
1	RP11-96C23.1	1.201E-39	-3.40	6.9867E-35	1.2503E-38		
1	RP11-96C23.1	8.4174E-07	-67.83	0.04896756	4.058E-06		
1	RP11-96K19.2	0.0015031	1.83	1	0.00575614		
1	RP11-977B10.	0.00167466	-1.87	1	0.00638746		
1	RP11-977G19	0.00122636	3.37	1	0.00474192		
1	RP11-977G19	5.7511E-05	-1.82	1	0.00024704		
1	RP11-982M15	0.00189757	-2.61	1	0.00719897		
1	RP11-983P16.	8.7459E-86	-6.01	5.0878E-81	1.3589E-84		
1	RP11-989E6.1	2.929E-11	-2.56	1.7039E-06	1.7406E-10		
1	RP11-98F14.1	0.00090772	2.50	1	0.0035476		
1	RP11-98G7.1	2.6384E-10	-3.34	1.5349E-05	1.5067E-09		
1	RP11-98J9.2	1.731E-05	-12.02	1	7.6948E-05		
1	RP11-996F15.	5.4514E-18	-3.14	3.1713E-13	3.9921E-17		
1	RP11-999E24.	0.00854582	-17.81	1	0.03026387		
1	RP11-9G1.3	2.0818E-45	-14.44	1.211E-40	2.3285E-44		
1	RP13-1032I1.1	4.3796E-08	2.14	0.00254777	2.2601E-07		
1	RP13-1032I1.1	0.00969224	1.74	1	0.0340481		
1	RP13-143G15	0.00022762	4.62	1	0.00093561		
1	RP13-188A5.1	2.4493E-05	-3.46	1	0.00010784		
1	RP13-192B19.	0.00978585	-17.75	1	0.03434996		
1	RP13-317D12	1.5278E-07	3.06	0.00888785	7.6672E-07		
1	RP13-395E19.	1.3799E-06	21.93	0.08027672	6.5741E-06		
1	RP13-395E19.	4.5551E-42	11.67	2.6499E-37	4.9036E-41		
1	RP13-401N8.1	1.8907E-06	4.83	0.10998982	8.9364E-06		
1	RP13-415G19	2.5033E-09	-10.34	0.00014563	1.3715E-08		
1	RP13-463N16	4.0428E-27	-2.85	2.3519E-22	3.5292E-26		
1	RP13-476E20.	7.3981E-12	-6.33	4.3038E-07	4.4986E-11		
1	RP13-578N3.3	1.9564E-14	-18.32	1.1381E-09	1.2982E-13		
1	RP13-580B18.	0.00218118	-2.34	1	0.00822294		
1	RP13-582O9.6	4.2037E-05	-2.72	1	0.00018214		
1	RP13-60P5.2	1.9055E-09	76.24	0.00011085	1.0485E-08		
1	RP13-631K18.	0.00525763	-2.80	1	0.01908033		
1	RP13-631K18.	2.5155E-05	-3.42	1	0.00011063		
1	RP13-672B3.2	1.202E-09	-1.86	6.9922E-05	6.6694E-09		

1	RP13-726E6.2	3.0316E-17	-5.63	1.7636E-12	2.1843E-16		
1	RP13-735L24.	1.3992E-07	-3.34	0.00813954	7.0369E-07		
1	RP13-890H12	7.1265E-26	-6.02	4.1458E-21	6.0709E-25		
1	RP13-923O23	7.4577E-10	-3.34	4.3385E-05	4.1776E-09		
1	RP13-942N8.1	1.7404E-07	-1.71	0.01012454	8.7003E-07		
1	RP13-968A2.1	9.2099E-25	-3.01	5.3578E-20	7.699E-24		
1	RP13-977J11.1	8.4845E-24	6.08	4.9358E-19	6.9574E-23		
1	RP1L1	8.987E-251	-9.07	5.228E-246	2.457E-249	Q8IWN7	RP1L1
1	RP2	2.7756E-14	-1.51	1.6147E-09	1.833E-13	O75695	RP2
1	RP3-322G13.7	0.00061547	-2.16	1	0.00244132		
1	RP3-323P24.3	0.00631479	-4.66	1	0.02269175		
1	RP3-324O17.8	0.00673838	-5.63	1	0.0241319		
1	RP3-325F22.5	1.6028E-18	-4.97	9.3242E-14	1.186E-17		
1	RP3-326L13.2	0.00214415	-21.57	1	0.00809119		
1	RP3-331H24.5	2.5081E-06	-6.61	0.14590367	1.1758E-05		
1	RP3-388N13.3	0.00047845	2.27	1	0.00191453		
1	RP3-395C13.1	1.0051E-06	-44.06	0.05846919	4.8243E-06		
1	RP3-395M20.	2.2826E-05	1.88	1	0.0001007		
1	RP3-395M20.8	3.9956E-14	-11.60	2.3244E-09	2.6252E-13		
1	RP3-399L15.3	2.0303E-11	-2.93	1.1811E-06	1.2143E-10		
1	RP3-400B16.1	7.0533E-05	-2.42	1	0.00030093		
1	RP3-405J10.3	0.00425891	-2.81	1	0.01561563		
1	RP3-406A7.7	0.00017739	-8.52	1	0.00073517		
1	RP3-406P24.3	0.00244802	-2.11	1	0.00917835		
1	RP3-406P24.5	1.4959E-07	-3.64	0.00870232	7.5124E-07		
1	RP3-407E4.3	0.00012109	-30.84	1	0.00050801		
1	RP3-407E4.4	1.2673E-14	-12.98	7.3723E-10	8.4497E-14		
1	RP3-425C14.5	1.8649E-05	-2.45	1	8.2744E-05		
1	RP3-428L16.2	3.996E-05	1.84	1	0.00017348		
1	RP3-430N8.11	0.00942764	19.96	1	0.03316063		
1	RP3-439F8.1	4.818E-155	-11.30	2.803E-150	1.054E-153		
1	RP3-449O17.1	1.4926E-06	-1.52	0.08682773	7.0961E-06		
1	RP3-454B23.1	0.00942764	-19.50	1	0.03316063		
1	RP3-460G2.2	3.035E-188	19.73	1.766E-183	7.333E-187		
1	RP3-467K16.2	1.6829E-14	-27.90	9.7899E-10	1.1185E-13		
1	RP3-467K16.4	6.6174E-18	-8.02	3.8496E-13	4.8368E-17		
1	RP3-467K16.7	1.3669E-05	-16.62	0.79516605	6.119E-05		
1	RP3-467N11.1	1.4451E-08	-1.92	0.00084069	7.651E-08		
1	RP3-468K18.5	0.00013019	2.55	1	0.00054473		
1	RP3-488M23.1	5.8693E-08	14.65	0.00341443	3.008E-07		
1	RP3-512B11.3	6.3466E-12	-9.03	3.6921E-07	3.868E-11		
1	RP3-514A23.2	3.6657E-05	34.22	1	0.00015961		
1	RP3-522D1.1	1.8783E-08	-7.44	0.0010927	9.8896E-08		
1	RP3-523E19.2	0.00110019	-3.22	1	0.00426966		
1	RP3-523K23.2	3.0289E-77	-5.69	1.762E-72	4.3864E-76		
1	RP3-525N10.2	8.9893E-09	-4.28	0.00052294	4.8007E-08		
1	RP4-533D7.5	1.0718E-10	-6.41	6.235E-06	6.222E-10		

1	RP4-536B24.2	1.6708E-07	-81.61	0.00971971	8.3603E-07		
1	RP4-536B24.3	2.4671E-41	-12.79	1.4352E-36	2.6266E-40		
1	RP4-536B24.4	3.0931E-13	-5.06	1.7994E-08	1.9717E-12		
1	RP4-539M6.14	0.00200652	-19.72	1	0.00759003		
1	RP4-539M6.19	0.01436202	2.45	1	0.04951674		
1	RP4-541C22.5	8.399E-16	3.61	4.8861E-11	5.8223E-15		
1	RP4-545L17.1	0.00772953	-1.87	1	0.02753063		
1	RP4-550H1.4	7.3219E-06	-16.68	0.42594197	3.3365E-05		
1	RP4-550H1.5	0.00942764	-19.35	1	0.03316063		
1	RP4-555D20.1	9.0474E-18	2.56	5.2632E-13	6.5947E-17		
1	RP4-555D20.2	7.1226E-52	2.69	4.1435E-47	8.4787E-51		
1	RP4-555D20.4	3.8006E-05	-1.75	1	0.00016527		
1	RP4-559A3.7	2.934E-13	-2.74	1.7068E-08	1.8715E-12		
1	RP4-564M11.1	0.00258699	2.94	1	0.00967942		
1	RP4-565E6.1	1.6354E-55	-12.70	9.514E-51	2.0174E-54		
1	RP4-568C11.4	3.45E-60	-8.93	2.007E-55	4.4033E-59		
1	RP4-569D19.5	0.0003575	-13.94	1	0.00144547		
1	RP4-569M23.1	0.00701269	-1.97	1	0.02507259		
1	RP4-575N6.2	1.7536E-16	-17.23	1.0201E-11	1.2432E-15		
1	RP4-580N22.2	7.7684E-13	-30.39	4.5192E-08	4.8909E-12		
1	RP4-583P15.1	0.00573247	21.35	1	0.02070536		
1	RP4-583P15.1	2.8219E-29	3.09	1.6416E-24	2.5622E-28		
1	RP4-584D14.7	0.01090851	2.42	1	0.03811131		
1	RP4-591C20.9	1.1743E-29	1.66	6.8312E-25	1.0736E-28		
1	RP4-592A1.2	0.00011452	32.06	1	0.00048163		
1	RP4-594A5.1	7.9735E-14	-10.38	4.6385E-09	5.1914E-13		
1	RP4-607I7.1	1.4227E-09	-3.66	8.2763E-05	7.8732E-09		
1	RP4-608O15.3	2.5758E-10	-2.81	1.4985E-05	1.4717E-09		
1	RP4-612B15.3	2.8904E-13	-3.81	1.6814E-08	1.8439E-12		
1	RP4-613B23.5	1.188E-05	2.13	0.69111186	5.3388E-05		
1	RP4-614C10.1	1.2112E-07	75.82	0.00704623	6.1086E-07		
1	RP4-614C10.2	0	187.47	0	0		
1	RP4-614O4.11	4.5872E-11	2.66	2.6686E-06	2.7004E-10		
1	RP4-621F18.2	1.2611E-11	3.39	7.3361E-07	7.6101E-11		
1	RP4-625H18.2	8.2248E-38	-8.79	4.7847E-33	8.3795E-37		
1	RP4-635E18.6	3.4501E-05	2.74	1	0.00015052		
1	RP4-646N3.1	3.5318E-11	-4.89	2.0546E-06	2.0897E-10		
1	RP4-647C14.2	0.00356073	-3.08	1	0.0131502		
1	RP4-651E10.4	0.00029162	-5.81	1	0.00118649		
1	RP4-660H19.1	9.4338E-25	-5.48	5.488E-20	7.884E-24		
1	RP4-663N10.1	0.00859959	-6.39	1	0.03043577		
1	RP4-665J23.1	1.2287E-18	-2.04	7.148E-14	9.115E-18		
1	RP4-669L17.1	2.9516E-07	-2.11	0.0171705	1.4572E-06		
1	RP4-669L17.8	5.857E-39	-3.98	3.4072E-34	6.0562E-38		
1	RP4-669P10.1	0.00202815	-4.03	1	0.00766539		
1	RP4-676J13.2	0.00221091	1.80	1	0.00833069		
1	RP4-680D5.2	2.421E-10	-3.47	1.4084E-05	1.3847E-09		

1	RP4-681N20.5	8.3593E-05	-2.03	1	0.00035478		
1	RP4-682C21.5	0.00294853	-3.66	1	0.01097777		
1	RP4-724E13.2	9.9126E-13	8.44	5.7666E-08	6.2193E-12		
1	RP4-726N1.2	0.01044109	-5.64	1	0.03655731		
1	RP4-734P14.4	3.5427E-10	2.20	2.0609E-05	2.0122E-09		
1	RP4-737E23.2	1.4437E-40	-18.47	8.3986E-36	1.5204E-39		
1	RP4-737E23.5	2.164E-225	-10.29	1.259E-220	5.766E-224		
1	RP4-737E23.6	0.00310583	-21.52	1	0.01153834		
1	RP4-737E23.7	5.624E-78	-7.98	3.2717E-73	8.1915E-77		
1	RP4-739H11.3	6.846E-40	-2.93	3.9826E-35	7.1462E-39		
1	RP4-753M9.1	2.2907E-07	29.41	0.01332581	1.1379E-06		
1	RP4-758J18.7	1.9194E-06	-18.06	0.11165938	9.0677E-06		
1	RP4-760C5.3	1.6875E-18	24.72	9.8167E-14	1.2482E-17		
1	RP4-761J14.8	0.00998147	2.58	1	0.03500881		
1	RP4-764O22.1	0.00029623	-5.21	1	0.00120478		
1	RP4-771M4.3	0.00703248	7.14	1	0.0251387		
1	RP4-777O23.3	5.6698E-08	-2.63	0.00329837	2.9086E-07		
1	RP4-781K5.4	3.6167E-54	-5.96	2.104E-49	4.4118E-53		
1	RP4-781K5.5	1.731E-30	-5.14	1.007E-25	1.6037E-29		
1	RP4-781K5.6	5.1931E-20	-25.38	3.0211E-15	3.962E-19		
1	RP4-781K5.7	2.2515E-28	-8.22	1.3098E-23	2.0144E-27		
1	RP4-781K5.9	1.0922E-16	-15.58	6.3536E-12	7.7777E-16		
1	RP4-790G17.7	0.00194156	-5.32	1	0.00735674		
1	RP4-791C19.1	3.9897E-06	-17.96	0.23209768	1.8448E-05		
1	RP4-794H19.1	2.429E-142	6.53	1.413E-137	5.133E-141		
1	RP4-794I6.4	4.5868E-35	-7.07	2.6683E-30	4.5257E-34		
1	RP4-798A10.7	5.1011E-10	-3.54	2.9675E-05	2.8766E-09		
1	RP4-798P15.3	8.3209E-29	-12.59	4.8406E-24	7.5024E-28		
1	RP4-803A2.1	0.00741849	-6.02	1	0.02646491		
1	RP4-806M20.[illegible]	0.00226614	-21.59	1	0.0085217		
1	RP4-806M20.[illegible]	0.00257652	-6.01	1	0.0096415		
1	RP5-1004I9.1	2.025E-271	-10.54	1.178E-266	5.628E-270		
1	RP5-1011O1.2	2.8745E-07	-5.20	0.0167223	1.4202E-06		
1	RP5-1021I20.2	0.00101677	10.47	1	0.00396152		
1	RP5-1021I20.5	0.00061999	5.13	1	0.00245859		
1	RP5-1024G6.2	0.00617795	-3.29	1	0.02223023		
1	RP5-1024G6.5	0.00011145	-2.27	1	0.00046896		
1	RP5-1029K10.	2.3927E-06	-3.82	0.13919437	1.1233E-05		
1	RP5-1033H22	1.4625E-05	7.71	0.85080396	6.5376E-05		
1	RP5-1042I8.7	0.00077646	2.10	1	0.00305346		
1	RP5-1042K10.	0.00190439	2.95	1	0.00722241		
1	RP5-1050D4.2	0.00342327	3.64	1	0.01267311		
1	RP5-1050D4.5	8.9663E-06	-2.43	0.52160551	4.0637E-05		
1	RP5-1052I5.2	5.1943E-09	4.25	0.00030217	2.8028E-08		
1	RP5-1056H1.2	7.9605E-12	-3.00	4.631E-07	4.8355E-11		
1	RP5-1057I20.2	0.00040657	-13.90	1	0.00163544		
1	RP5-1061H20	1.6633E-11	-1.75	9.6758E-07	9.9926E-11		

1	RP5-1063M23	6.2578E-11	18.66	3.6404E-06	3.6631E-10		
1	RP5-1063M23	7.9604E-91	16.63	4.6309E-86	1.2846E-89		
1	RP5-1071N3.1	9.6756E-08	-5.56	0.0056287	4.9018E-07		
1	RP5-1085F17.	1.0281E-17	2.69	5.9807E-13	7.4872E-17		
1	RP5-1085F17.	2.2014E-05	2.92	1	9.7216E-05		
1	RP5-1086L22.	6.2708E-07	-6.98	0.03648	3.0446E-06		
1	RP5-1112D6.4	0.00040764	-1.73	1	0.00163965		
1	RP5-1120P11.	2.061E-171	-9.99	1.199E-166	4.736E-170		
1	RP5-1121A15.	0.00124831	-7.09	1	0.00482167		
1	RP5-1125A11.	6.3027E-08	-6.73	0.00366652	3.2236E-07		
1	RP5-1132H15	8.8127E-34	2.08	5.1267E-29	8.5745E-33		
1	RP5-1132H15	0.00453149	-8.21	1	0.01656392		
1	RP5-1142A6.2	0.00244394	1.58	1	0.00916541		
1	RP5-1142J19.1	0.00507392	-19.87	1	0.01844469		
1	RP5-1157M23	0.00087759	-1.69	1	0.0034349		
1	RP5-1158E12.	8.2378E-09	-4.16	0.00047922	4.4038E-08		
1	RP5-1159O4.1	6.7336E-27	-5.00	3.9172E-22	5.8431E-26		
1	RP5-1159O4.2	3.2482E-11	-13.60	1.8896E-06	1.9256E-10		
1	RP5-1165K10.	0.00139309	-2.14	1	0.00535813		
1	RP5-1173A5.1	1.2174E-07	52.96	0.00708199	6.139E-07		
1	RP5-1174N9.2	1.6295E-09	-7.80	9.4796E-05	8.9896E-09		
1	RP5-1184F4.5	3.9969E-07	5.46	0.02325163	1.9608E-06		
1	RP5-1198O20	1.3621E-14	-3.53	7.9236E-10	9.0732E-14		
1	RP5-827C21.2	3.7805E-12	81.93	2.1993E-07	2.3236E-11		
1	RP5-827C21.4	4.0918E-12	-2.79	2.3804E-07	2.512E-11		
1	RP5-851M4.1	1.015E-16	-6.00	5.9047E-12	7.2344E-16		
1	RP5-856G1.2	0.00766008	-3.73	1	0.0272933		
1	RP5-857K21.2	0.00942764	20.03	1	0.03316063		
1	RP5-857K21.4	0	-2.77	0	0		
1	RP5-858B6.3	0.00079076	-3.88	1	0.00310801		
1	RP5-864K19.4	0.00169925	-1.67	1	0.00647703		
1	RP5-874C20.8	0.00433613	2.45	1	0.01589076		
1	RP5-875H18.9	4.7138E-07	-3.92	0.02742232	2.3036E-06		
1	RP5-875O13.7	2.3547E-08	-1.62	0.00136983	1.2331E-07		
1	RP5-877J2.1	0.00038652	-4.11	1	0.00155738		
1	RP5-881P19.7	0.01066133	-17.79	1	0.03728634		
1	RP5-882C2.2	5.094E-24	-3.05	2.9634E-19	4.192E-23		
1	RP5-884G6.2	3.915E-12	-4.24	2.2775E-07	2.4047E-11		
1	RP5-884M6.1	2.251E-193	-6.89	1.31E-188	5.552E-192		
1	RP5-906C1.1	0.00081849	25.93	1	0.00321222		
1	RP5-908M14.	0.00139948	-5.29	1	0.00538128		
1	RP5-908M14.9	0.00010937	1.61	1	0.00046057		
1	RP5-921G16.1	2.9257E-06	-6.40	0.1701972	1.3652E-05		
1	RP5-928E24.2	9.2078E-06	-42.54	0.53565732	4.1695E-05		
1	RP5-940J5.6	1.7176E-05	-2.54	0.99919212	7.6373E-05		
1	RP5-958B11.1	4.2928E-08	-62.22	0.00249727	2.216E-07		
1	RP5-963E22.4	0.00360227	11.23	1	0.0132935		

1	RP5-965G21.4	1.652E-05	-1.76	0.96100925	7.3533E-05		
1	RP5-968J1.1	0.01395886	-4.02	1	0.04820387		
1	RP5-968P14.2	1.6544E-07	-3.21	0.00962441	8.2798E-07		
1	RP5-972B16.2	1.3738E-15	-3.04	7.9919E-11	9.4444E-15		
1	RP6-114E22.1	5.8657E-27	-19.52	3.4123E-22	5.0961E-26		
1	RP6-191P20.4	2.5267E-11	1.68	1.4699E-06	1.5057E-10		
1	RP6-1O2.1	0.00162864	-3.69	1	0.00621927		
1	RP6-201G10.2	0.01190476	2.80	1	0.04142774		
1	RP6-24A23.7	0.001689	-11.23	1	0.00643837		
1	RP6-65G23.3	0	-2.12	0	0		
1	RP6-74O6.2	2.5695E-07	-3.23	0.01494769	1.2731E-06		
1	RP6-91H8.3	3.402E-18	157.73	1.9791E-13	2.5007E-17		
1	RPA2	1.8823E-83	2.32	1.095E-78	2.8688E-82	P15927	RPA2
1	RPAP1	0	-1.85	0	0	Q9BWH6	RPAP1
1	RPAP2	1.4408E-25	1.63	8.3817E-21	1.2206E-24	Q8IXW5	RPAP2
1	RPE65	6.2401E-15	2.57	3.6301E-10	4.202E-14	Q16518	RPE65
1	RPF1	1.175E-27	1.65	6.8354E-23	1.0379E-26	Q9H9Y2	RPF1
1	RPF2	6.1669E-36	1.81	3.5875E-31	6.1504E-35	Q9H7B2	RPF2
1	RPH3AL	3.6921E-19	-1.99	2.1478E-14	2.7685E-18	Q9UNE2	RPH3AL
1	RPIA	3.3932E-35	1.54	1.974E-30	3.3537E-34	P49247	RPIA
1	RPL10	5.4428E-92	3.15	3.1663E-87	8.8692E-91	P27635	RPL10
1	RPL10P16	0.00016489	2.30	1	0.00068476		
1	RPL10P19	3.834E-05	-3.65	1	0.00016666		
1	RPL11	5.6152E-21	1.63	3.2666E-16	4.3601E-20	P62913	RPL11
1	RPL12P14	0.00093264	7.66	1	0.00364226		
1	RPL12P45	0.0001104	-12.49	1	0.00046476		
1	RPL13A	3.8402E-46	2.32	2.234E-41	4.3286E-45	P40429	RPL13A
1	RPL13AP20	0.01181642	1.99	1	0.04113263		
1	RPL13AP25	0.00953374	2.13	1	0.03351557		
1	RPL13P12	3.4335E-68	-6.86	1.9974E-63	4.643E-67		
1	RPL14	2.694E-66	2.15	1.5672E-61	3.6011E-65	P50914	RPL14
1	RPL14P1	1.1889E-05	2.47	0.69163582	5.3425E-05		
1	RPL15	1.0398E-20	1.72	6.0491E-16	8.0355E-20	P61313	RPL15
1	RPL17	1.038E-273	4.52	6.04E-269	2.888E-272	P18621	RPL17
1	RPL17-C18orf	3.6232E-38	2.97	2.1078E-33	3.7095E-37	A0A0A6YYL6	RPL17-C18orf
1	RPL18A	1.8888E-54	2.26	1.0988E-49	2.3088E-53	Q02543	RPL18A
1	RPL21	2.0187E-11	1.50	1.1744E-06	1.2076E-10	P46778	RPL21
1	RPL22	1.0394E-25	1.68	6.0464E-21	8.8294E-25	P35268	RPL22
1	RPL22L1	0	-1.85	0	0	Q6P5R6	RPL22L1
1	RPL23AP82	2.371E-87	2.22	1.3793E-82	3.7278E-86		
1	RPL23AP87	1.4758E-11	5.54	8.5853E-07	8.8856E-11		
1	RPL24	1.4762E-20	1.75	8.5875E-16	1.1365E-19	P83731	RPL24
1	RPL26	1.2202E-35	2.22	7.0985E-31	1.2134E-34	P61254	RPL26
1	RPL26L1	3.8169E-37	2.24	2.2204E-32	3.8589E-36	Q9UNX3	RPL26L1
1	RPL27A	4.9633E-94	3.12	2.8873E-89	8.2074E-93	P46776	RPL27A
1	RPL28	1.3036E-51	2.19	7.5834E-47	1.547E-50	P46779	RPL28
1	RPL29	8.8051E-31	1.96	5.1223E-26	8.1944E-30	P47914	RPL29

1	RPL31	3.8499E-17	1.70	2.2396E-12	2.7677E-16	P62899	RPL31
1	RPL32	3.4826E-28	1.94	2.026E-23	3.1102E-27	P62910	RPL32
1	RPL32P3	0	-2.35	0	0		
1	RPL34-AS1	4.9552E-31	-4.74	2.8826E-26	4.6278E-30		
1	RPL35	1.7153E-11	1.52	9.9787E-07	1.0297E-10	P42766	RPL35
1	RPL36	1.5258E-54	2.39	8.876E-50	1.8674E-53	Q9Y3U8	RPL36
1	RPL36A	2.6532E-19	1.70	1.5435E-14	1.9944E-18	P83881	RPL36A
1	RPL36AL	1.8257E-28	1.84	1.0621E-23	1.6355E-27	Q969Q0	RPL36AL
1	RPL38	6.3187E-24	1.83	3.6759E-19	5.1926E-23	P63173	RPL38
1	RPL39	5.3652E-15	1.66	3.1212E-10	3.6179E-14	P62891	RPL39
1	RPL39L	2.0913E-09	-1.64	0.00012166	1.1486E-08	Q96EH5	RPL39L
1	RPL5	9.706E-47	2.18	5.6463E-42	1.1007E-45	P46777	RPL5
1	RPL5P19	0.00942764	-19.41	1	0.03316063		
1	RPL6	4.4846E-25	1.78	2.6089E-20	3.7695E-24	Q02878	RPL6
1	RPL7	2.4726E-15	1.62	1.4384E-10	1.6887E-14	P18124	RPL7
1	RPL9P29	1.4646E-09	-3.18	8.5203E-05	8.0984E-09		
1	RPLP2	7.002E-133	3.40	4.073E-128	1.426E-131	P05387	RPLP2
1	RPN2	0	-1.60	0	0	P04844	RPN2
1	RPP14	1.2344E-85	2.20	7.1809E-81	1.9149E-84	O95059	RPP14
1	RPP25	7.0825E-07	-1.82	0.04120147	3.4297E-06	Q9BUL9	RPP25
1	RPP30	2.7154E-34	1.85	1.5796E-29	2.6584E-33	P78346	RPP30
1	RPP40	1.9048E-23	1.85	1.1081E-18	1.551E-22	O75818	RPP40
1	RPRD2	0	-1.78	0	0	Q5VT52	RPRD2
1	RPRM	1.7904E-07	-15.10	0.01041557	8.945E-07	Q9NS64	RPRM
1	RPS11P6	4.2999E-14	-4.53	2.5014E-09	2.8223E-13		
1	RPS12	7.6594E-29	1.96	4.4558E-24	6.9103E-28	P25398	RPS12
1	RPS13	1.016E-132	3.44	5.912E-128	2.069E-131	P62277	RPS13
1	RPS14	8.7964E-32	2.10	5.1172E-27	8.3044E-31	P62263	RPS14
1	RPS15	3.2622E-31	2.00	1.8978E-26	3.0555E-30	P62841	RPS15
1	RPS15A	1.3043E-18	1.71	7.5877E-14	9.6695E-18	P62244	RPS15A
1	RPS16	2.698E-16	1.66	1.5695E-11	1.9006E-15	P62249	RPS16
1	RPS19	7.8295E-18	1.58	4.5547E-13	5.7141E-17	P39019	RPS19
1	RPS19BP1	1.0552E-75	2.68	6.1385E-71	1.5105E-74	Q86WX3	RPS19BP1
1	RPS2	1.1798E-31	1.74	6.8633E-27	1.1111E-30	P15880	RPS2
1	RPS21	4.1587E-58	2.56	2.4193E-53	5.2275E-57	P63220	RPS21
1	RPS23	1.8838E-36	2.18	1.0959E-31	1.8917E-35	P62266	RPS23
1	RPS23P8	0.00688368	2.74	1	0.02462497		
1	RPS24	1.781E-45	2.17	1.0361E-40	1.9936E-44	P62847	RPS24
1	RPS25	1.0538E-21	1.79	6.1303E-17	8.3045E-21	P62851	RPS25
1	RPS26	1.5563E-22	1.76	9.0538E-18	1.2471E-21	P62854	RPS26
1	RPS27L	1.2886E-65	2.45	7.4965E-61	1.7166E-64	Q71UM5	RPS27L
1	RPS28	3.5595E-26	1.75	2.0707E-21	3.0515E-25	P62857	RPS28
1	RPS29	9.8784E-10	1.83	5.7467E-05	5.5045E-09	P62273	RPS29
1	RPS2P32	9.5988E-19	-6.82	5.584E-14	7.1397E-18		
1	RPS4X	5.8292E-48	2.09	3.3911E-43	6.7004E-47	P62701	RPS4X
1	RPS4XP16	0.00060791	-3.86	1	0.00241214		
1	RPS4XP22	1.6867E-06	-7.36	0.09812443	7.9945E-06		

1	RPS6KA1	0	-1.73	0	0	Q15418	RPS6KA1
1	RPS6KA2	2.6605E-08	1.94	0.00154775	1.3899E-07	Q15349	RPS6KA2
1	RPS6KA3	0	-1.55	0	0	P51812	RPS6KA3
1	RPS6KA4	0	-2.25	0	0	O75676	RPS6KA4
1	RPS6KA5	2.0024E-97	2.87	1.1649E-92	3.3844E-96	O75582	RPS6KA5
1	RPS6KA6	3.7963E-14	1.90	2.2085E-09	2.4963E-13	Q9UK32	RPS6KA6
1	RPS6KB2	0	-1.56	0	0	Q9UBS0	RPS6KB2
1	RPS6KC1	0	-2.09	0	0	Q96S38	RPS6KC1
1	RPS6KL1	3.9034E-85	5.41	2.2708E-80	6.0441E-84	Q9Y6S9	RPS6KL1
1	RPS7P9	0.00011616	-14.36	1	0.00048834		
1	RPS8	4.3539E-19	1.64	2.5328E-14	3.2604E-18	P62241	RPS8
1	RPS9	5.4819E-24	1.56	3.189E-19	4.5087E-23	P46781	RPS9
1	RPSA	2.3643E-20	1.72	1.3754E-15	1.8134E-19	P08865	RPSA
1	RPSAP52	6.4484E-09	1.70	0.00037513	3.4644E-08		
1	RPSAP69	2.5891E-30	-48.40	1.5062E-25	2.3919E-29		
1	RPTN	5.2822E-07	-20.54	0.03072873	2.5749E-06	Q6XPR3	RPTN
1	RPTOR	3.6851E-43	1.57	2.1438E-38	4.0229E-42	Q8N122	RPTOR
1	RPUSD1	1.0981E-79	3.28	6.3879E-75	1.6254E-78	Q9UJJ7	RPUSD1
1	RPUSD4	6.4381E-73	1.75	3.7453E-68	9.0248E-72	Q96CM3	RPUSD4
1	RRAGD	0.00056263	1.76	1	0.00223966	Q9NQL2	RRAGD
1	RRAS2	4.0435E-85	2.21	2.3523E-80	6.2594E-84	P62070	RRAS2
1	RRH	3.4964E-05	3.85	1	0.00015247	O14718	RRH
1	RRM1	1.6599E-58	2.07	9.6565E-54	2.0938E-57	P23921	RRM1
1	RRM2	1.501E-121	2.13	8.73E-117	2.927E-120	P31350	RRM2
1	RRN3	1.2894E-53	1.75	7.5007E-49	1.5649E-52	Q9NYV6	RRN3
1	RRN3P1	4.216E-74	-6.78	2.4526E-69	5.9573E-73	Q2M238	RRN3P1
1	RRN3P2	0.00763843	-2.95	1	0.02721783		
1	RRNAD1	0	-3.85	0	0	Q96FB5	RRNAD1
1	RRP1	3.076E-42	1.53	1.7894E-37	3.3186E-41	P56182	RRP1
1	RRP12	6.7667E-88	1.71	3.9364E-83	1.0691E-86	Q5JTH9	RRP12
1	RRP36	2.8382E-43	1.81	1.6511E-38	3.1041E-42	Q96EU6	RRP36
1	RRP7BP	9.8309E-42	1.66	5.719E-37	1.0532E-40	Q9NSQ0	RRP7BP
1	RRP8	1.649E-114	3.12	9.591E-110	3.096E-113	O43159	RRP8
1	RRP9	5.0178E-65	3.15	2.9191E-60	6.6463E-64	O43818	RRP9
1	RRS1	6.9728E-65	1.98	4.0564E-60	9.2316E-64	Q15050	RRS1
1	RSAD1	1.6634E-08	2.10	0.00096767	8.7826E-08	Q9HA92	RSAD1
1	RSAD2	0.00028305	-7.45	1	0.0011527	Q8WXG1	RSAD2
1	RSG1	2.1866E-06	1.52	0.12720528	1.0298E-05	Q9BU20	RSG1
1	RSL1D1	1.3833E-49	1.79	8.0475E-45	1.6127E-48	O76021	RSL1D1
1	RSPH10B2	1.7757E-11	4.43	1.033E-06	1.0653E-10		
1	RSPH14	2.0125E-07	-51.42	0.0117074	1.0027E-06	Q9UHP6	RSPH14
1	RSPH3	3.0616E-20	-1.83	1.781E-15	2.3456E-19	Q86UC2	RSPH3
1	RSPH9	8.5659E-05	-2.09	1	0.00036333	Q9H1X1	RSPH9
1	RSPO3	0.00026815	2.65	1	0.0010941	Q9BXY4	RSPO3
1	RSPO4	8.6101E-05	-30.87	1	0.00036513	Q2I0M5	RSPO4
1	RSU1	1.7565E-76	2.03	1.0218E-71	2.53E-75	Q15404	RSU1
1	RTBDN	9.3067E-06	-4.31	0.5414107	4.2127E-05	Q9BSG5	RTBDN

1	RTCA-AS1	8.1615E-08	-2.02	0.00474788	4.1499E-07		
1	RTCB	7.1307E-97	2.54	4.1482E-92	1.1999E-95	Q9Y3I0	RTCB
1	RTEL1	7.2429E-45	2.11	4.2135E-40	8.0487E-44	Q9NZ71	RTEL1
1	RTEL1-TNFRSF	3.4225E-09	1.82	0.0001991	1.8625E-08	F6WH68	RTEL1-TNFRSF
1	RTFDC1	1.279E-112	2.16	7.443E-108	2.382E-111	Q9BY42	RTFDC1
1	RTKN	0	-2.42	0	0	Q9BST9	RTKN
1	RTKN2	2.265E-106	4.62	1.318E-101	4.05E-105	Q8IZC4	RTKN2
1	RTN1	0.00857528	-3.03	1	0.03036265	Q16799	RTN1
1	RTN2	3.5319E-77	2.74	2.0547E-72	5.1111E-76	O75298	RTN2
1	RTN3	0	-1.99	0	0	O95197	RTN3
1	RTP4	5.9609E-13	-20.52	3.4677E-08	3.768E-12	Q96DX8	RTP4
1	RTTN	6.6714E-54	1.79	3.881E-49	8.1193E-53	Q86VV8	RTTN
1	RUBCNL	0.00551005	-5.65	1	0.01995032		
1	RUFY1	3.1808E-42	1.53	1.8504E-37	3.4311E-41	Q96T51	RUFY1
1	RUFY2	4.6808E-21	1.53	2.723E-16	3.6409E-20	Q8WXA3	RUFY2
1	RUFY3	0	-1.69	0	0	Q7L099	RUFY3
1	RUNDC3B	8.1668E-08	-2.27	0.00475096	4.1522E-07		
1	RUNX2	0	-2.69	0	0	Q13950	RUNX2
1	RUSC1-AS1	7.1476E-13	-1.85	4.1581E-08	4.5064E-12	Q66K80	RUSC1-AS1
1	RUSC2	4.3484E-90	2.78	2.5297E-85	6.9842E-89	Q8N2Y8	RUSC2
1	RWDD1	1.2372E-52	2.11	7.1974E-48	1.4852E-51	Q9H446	RWDD1
1	RWDD2A	1.9357E-82	2.90	1.1261E-77	2.9325E-81		
1	RWDD4	0	-2.18	0	0		
1	RXFP1	1.8706E-12	-7.70	1.0882E-07	1.1629E-11	Q9HBX9	RXFP1
1	RXRA	0	-4.52	0	0	P19793	RXRA
1	RYK	3.2837E-07	-1.98	0.01910275	1.617E-06	P34925	RYK
1	RYR1	6.8539E-05	-5.54	1	0.00029266	P21817	RYR1
1	RYR2	0.00371913	-2.44	1	0.01370646	Q92736	RYR2
1	S100A10	0	-2.04	0	0	P60903	S100A10
1	S100A11	0	-2.15	0	0	P31949	S100A11
1	S100A14	0	-18.99	0	0	Q9HCY8	S100A14
1	S100A16	2.6309E-33	1.77	1.5305E-28	2.5394E-32	Q96FQ6	S100A16
1	S100A2	0	-4.77	0	0	P29034	S100A2
1	S100A3	6.1E-188	-5.76	3.549E-183	1.472E-186	P33764	S100A3
1	S100A4	2.858E-172	-5.82	1.662E-167	6.581E-171	P26447	S100A4
1	S100A5	1.2475E-10	-5.25	7.2573E-06	7.2284E-10	P33763	S100A5
1	S100A8	0.00103329	-23.45	1	0.0040216	P05109	S100A8
1	S100A9	8.4823E-94	-141.56	4.9345E-89	1.3995E-92	P06702	S100A9
1	S100P	1.1028E-28	-10.95	6.4153E-24	9.9308E-28	P25815	S100P
1	S1PR1	5.894E-91	-16.96	3.4288E-86	9.5244E-90	P21453	S1PR1
1	S1PR3	8.6195E-60	20.76	5.0143E-55	1.0977E-58	Q99500	S1PR3
1	S1PR4	1E-06	-4.29	0.05817439	4.8015E-06	O95977	S1PR4
1	S1PR5	1.2737E-40	-2.83	7.4096E-36	1.3418E-39	Q9H228	S1PR5
1	SAA1	3.2221E-13	-7.57	1.8744E-08	2.0535E-12	P0DJI8	SAA1
1	SAA2	7.5333E-05	-4.16	1	0.0003208	P0DJI9	SAA2
1	SAAL1	8.7577E-66	1.97	5.0947E-61	1.1674E-64	Q96ER3	SAAL1
1	SAC3D1	5.5043E-56	2.12	3.2021E-51	6.8129E-55	A6NKF1	SAC3D1

1	SACS	3.801E-165	3.76	2.211E-160	8.564E-164	Q9NZJ4	SACS
1	SACS-AS1	0.00146074	26.02	1	0.00559906		
1	SAE1	8.4765E-41	1.53	4.9311E-36	8.9543E-40	Q9UBE0	SAE1
1	SALL1	0.00049218	25.91	1	0.00196729	Q9NSC2	SALL1
1	SALL2	2.2665E-12	10.92	1.3185E-07	1.4057E-11	Q9Y467	SALL2
1	SALL4	2.2627E-14	-2.18	1.3163E-09	1.4975E-13	Q9UJQ4	SALL4
1	SALRNA1	9.9219E-05	2.73	1	0.00041905		
1	SAMD12	2.514E-162	-10.74	1.462E-157	5.617E-161		
1	SAMD12-AS1	8.1986E-05	-4.30	1	0.00034819		
1	SAMD13	9.0191E-08	-1.82	0.00524676	4.5751E-07		
1	SAMD15	8.938E-122	8.27	5.2E-117	1.747E-120	Q9P1V8	SAMD15
1	SAMD4A	9.8495E-79	2.49	5.7298E-74	1.4447E-77	Q9UPU9	SAMD4A
1	SAMD5	3.615E-66	-10.00	2.103E-61	4.83E-65		
1	SAMD8	0	-2.05	0	0	Q96LT4	SAMD8
1	SAMD9	0	-7.39	0	0	Q5K651	SAMD9
1	SAMD9L	1.754E-286	-16.30	1.021E-281	4.909E-285	Q8IVG5	SAMD9L
1	SAMHD1	1.0562E-76	2.09	6.1445E-72	1.5239E-75	Q9Y3Z3	SAMHD1
1	SAMM50	1.9658E-55	1.82	1.1436E-50	2.4233E-54	Q9Y512	SAMM50
1	SAMMSON	7.8011E-05	-5.19	1	0.00033206		
1	SAP25	2.7602E-05	-6.21	1	0.00012105	Q8TEE9	SAP25
1	SAPCD2	6.117E-262	4.92	3.558E-257	1.69E-260	Q86UD0	SAPCD2
1	SAR1A	3.8554E-88	2.11	2.2428E-83	6.1062E-87	Q9NR31	SAR1A
1	SARM1	1.3343E-45	5.73	7.7619E-41	1.497E-44	Q6SZW1	SARM1
1	SASH3	0.01422412	-2.71	1	0.04906945	O75995	SASH3
1	SAT1	0	-2.91	0	0	P21673	SAT1
1	SAT2	1.7623E-63	2.45	1.0252E-58	2.3074E-62	Q96F10	SAT2
1	SATB1	6.6732E-61	-12.08	3.8821E-56	8.5565E-60	Q01826	SATB1
1	SATB1-AS1	1.5036E-41	-11.42	8.7473E-37	1.6056E-40		
1	SAXO1	1.8772E-10	-4.74	1.092E-05	1.0792E-09	Q8IYX7	SAXO1
1	SAXO2	2.6773E-05	-1.56	1	0.00011759	Q658L1	SAXO2
1	SAYSD1	9.0332E-11	-1.53	5.255E-06	5.2608E-10	Q9NPB0	SAYSD1
1	SBDSP1	8.4846E-44	2.07	4.9358E-39	9.3429E-43		
1	SBF2	2.8168E-62	1.74	1.6386E-57	3.6528E-61	Q86WG5	SBF2
1	SBK1	9.6349E-16	8.90	5.605E-11	6.6568E-15	Q52WX2	SBK1
1	SBNO2	0	-1.54	0	0	Q9Y2G9	SBNO2
1	SC22CB-56B3.	6.4308E-16	-9.00	3.741E-11	4.4765E-15		
1	SCAMP5	3.8057E-11	1.62	2.2139E-06	2.2476E-10	Q8TAC9	SCAMP5
1	SCAND1	8.0911E-12	1.60	4.7069E-07	4.9133E-11	P57086	SCAND1
1	SCARA3	2.7002E-75	3.25	1.5708E-70	3.8567E-74	Q6AZY7	SCARA3
1	SCARB1	0	-2.48	0	0	Q8WTV0	SCARB1
1	SCARB2	0	-1.67	0	0	Q14108	SCARB2
1	SCARF1	0.0053095	-2.14	1	0.01926016	Q14162	SCARF1
1	SCARF2	0.00065402	1.71	1	0.00258841	Q96GP6	SCARF2
1	SCART1	4.5916E-09	-1.84	0.00026711	2.4838E-08		
1	SCCPDH	1.3855E-73	2.57	8.0602E-69	1.9502E-72	Q8NBX0	SCCPDH
1	SCD	0	-4.19	0	0	O00767	SCD
1	SCD5	0	-2.98	0	0	Q86SK9	SCD5

1	SCEL	9.2257E-70	-8.43	5.367E-65	1.2607E-68	O95171	SCEL
1	SCG2	7.5455E-14	7.84	4.3895E-09	4.9149E-13	P13521	SCG2
1	SCG5	2.6149E-12	1.84	1.5212E-07	1.6176E-11	P05408	SCG5
1	SCGB1A1	4.0023E-64	-12.28	2.3283E-59	5.2676E-63	P11684	SCGB1A1
1	SCGB2B2	1.6662E-06	-2.27	0.09693111	7.9005E-06	Q4G0G5	SCGB2B2
1	SCGB3A2	2.0403E-13	47.01	1.1869E-08	1.3101E-12	Q96PL1	SCGB3A2
1	SCHLAP1	6.5558E-52	-9.15	3.8138E-47	7.8087E-51		
1	SCIN	8.7855E-08	-4.30	0.00511086	4.4597E-07	Q9Y6U3	SCIN
1	SCLY	4.3555E-41	1.77	2.5337E-36	4.6203E-40	Q96I15	SCLY
1	SCML2	4.3168E-37	6.05	2.5113E-32	4.3614E-36	Q9UQR0	SCML2
1	SCN1B	2.6256E-63	168.27	1.5274E-58	3.4325E-62	Q07699	SCN1B
1	SCN2A	8.9097E-05	1.71	1	0.00037745	Q99250	SCN2A
1	SCN2B	0.00172143	-9.45	1	0.00655682	O60939	SCN2B
1	SCN3A	2.1844E-07	-2.91	0.01270756	1.0868E-06	Q9NY46	SCN3A
1	SCN4B	3.3014E-08	-6.45	0.00192056	1.7148E-07	Q8IWT1	SCN4B
1	SCN8A	0.00998712	-2.22	1	0.03502477	Q9UQD0	SCN8A
1	SCNN1A	0	-17.95	0	0	P37088	SCNN1A
1	SCNN1D	4.8343E-15	3.42	2.8123E-10	3.2656E-14	P51172	SCNN1D
1	SCO1	5.0369E-48	1.62	2.9301E-43	5.7931E-47	O75880	SCO1
1	SCO2_1	3.8952E-19	2.09	2.266E-14	2.9189E-18		
1	SCO2_2	1.5487E-07	8.32	0.00900938	7.7687E-07		
1	SCPEP1	0	-1.52	0	0	Q9HB40	SCPEP1
1	SCTR	7.7532E-06	-9.63	0.4510322	3.5273E-05	P47872	SCTR
1	SCUBE2	9.5719E-24	7.08	5.5684E-19	7.8406E-23	Q9NQ36	SCUBE2
1	SCUBE3	0.00084161	1.53	1	0.00329872	Q8IX30	SCUBE3
1	SCX	7.4907E-18	-4.92	4.3577E-13	5.4683E-17	Q7RTU7	SCX
1	SDAD1P1	0.00013055	-1.89	1	0.00054616		
1	SDC1	0	-2.86	0	0	P18827	SDC1
1	SDC2	2.0695E-09	12.70	0.00012039	1.1369E-08	P34741	SDC2
1	SDC4	0	-2.96	0	0	P31431	SDC4
1	SDCBP2	1.0049E-10	1.99	5.846E-06	5.8384E-10	Q9H190	SDCBP2
1	SDCBP2-AS1	0	-2.95	0	0		
1	SDE2	0	-1.55	0	0	Q6IQ49	SDE2
1	SDF2L1	6.4676E-70	3.17	3.7624E-65	8.8528E-69	Q9HCN8	SDF2L1
1	SDHA	0	-2.80	0	0	P31040	SDHA
1	SDHAF2	0	-1.60	0	0	Q9NX18	SDHAF2
1	SDHAF4	4.4245E-43	-2.54	2.5739E-38	4.8219E-42	Q5VUM1	SDHAF4
1	SDHAP1	0	-1.62	0	0		
1	SDHAP2	3.2187E-06	-10.05	0.18724428	1.4978E-05		
1	SDHAP3	3.327E-123	-6.10	1.935E-118	6.536E-122		
1	SDHD_2	0.0030008	2.33	1	0.01116665		
1	SDK1	4.0121E-70	12.19	2.334E-65	5.5073E-69	Q7Z5N4	SDK1
1	SDPR	1.1749E-25	-7.66	6.8346E-21	9.9673E-25		
1	SDR16C5	9.6243E-06	-7.64	0.55988453	4.3523E-05	Q8N3Y7	SDR16C5
1	SDR39U1	1.1269E-17	1.50	6.5555E-13	8.1985E-17	Q9NRG7	SDR39U1
1	SDR42E1	0	-2.83	0	0	Q8WUS8	SDR42E1
1	SDR42E2	1.0002E-06	-4.64	0.0581829	4.8018E-06	A6NKP2	SDR42E2

1	SDR9C7	0.00065394	-25.27	1	0.00258841	Q8NEX9	SDR9C7
1	SEC11C	6.0663E-58	5.26	3.529E-53	7.6106E-57	Q9BY50	SEC11C
1	SEC13	3.994E-122	1.84	2.324E-117	7.818E-121	P55735	SEC13
1	SEC14L1	4.4281E-12	2.09	2.576E-07	2.7147E-11	Q92503	SEC14L1
1	SEC14L2	5.9144E-64	2.25	3.4407E-59	7.7773E-63	O76054	SEC14L2
1	SEC14L4	0.0005339	-10.06	1	0.00212879	Q9UDX3	SEC14L4
1	SEC14L5	1.0424E-07	-19.18	0.00606378	5.271E-07	O43304	SEC14L5
1	SEC14L6	0.00033146	-4.93	1	0.00134279	B5MCN3	SEC14L6
1	SEC16B	2.4778E-53	-21.63	1.4415E-48	2.9974E-52	Q96JE7	SEC16B
1	SEC1P	0.00607179	-1.79	1	0.02186176		
1	SEC22B	6.3865E-14	2.26	3.7153E-09	4.1684E-13	O75396	SEC22B
1	SEC22C	3.4417E-86	2.10	2.0022E-81	5.3621E-85	Q9BRL7	SEC22C
1	SEC23A	2.3922E-37	3.14	1.3916E-32	2.4245E-36	Q15436	SEC23A
1	SEC24A	2.1281E-45	2.02	1.238E-40	2.3794E-44	O95486	SEC24A
1	SEC24B-AS1	2.807E-07	-1.77	0.01632973	1.388E-06		
1	SEC24C	1.6196E-35	2.52	9.4217E-31	1.6086E-34	P53992	SEC24C
1	SEC62	0	-2.67	0	0	Q99442	SEC62
1	SECTM1	6.7038E-10	1.88	3.8999E-05	3.7611E-09	Q8WVN6	SECTM1
1	SEH1L	1.3402E-53	1.73	7.7963E-49	1.6256E-52	Q96EE3	SEH1L
1	SEL1L2	5.8599E-05	19.23	1	0.00025145	Q5TEA6	SEL1L2
1	SEL1L3	0	-11.88	0	0	Q68CR1	SEL1L3
1	SELENBP1	1.842E-230	-16.06	1.071E-225	4.932E-229	Q13228	SELENBP1
1	SELENOI	0	-1.68	0	0	Q9C0D9	SELENOI
1	SELENOK	1.5189E-10	1.54	8.8359E-06	8.7667E-10	Q9Y6D0	SELENOK
1	SELENOM	9.6601E-54	-5.76	5.6197E-49	1.1742E-52	Q8WWX9	SELENOM
1	SELENON	6.8845E-41	1.56	4.005E-36	7.2805E-40	Q9NZV5	SELENON
1	SELENOP	8.8979E-45	-6.71	5.1763E-40	9.8728E-44	P49908	SELENOP
1	SELL	2.7496E-50	-24.81	1.5995E-45	3.2262E-49	P14151	SELL
1	SELPLG	2.3302E-08	3.14	0.00135557	1.2205E-07	Q14242	SELPLG
1	SEM1	0	-1.84	0	0		
1	SEMA3A	4.5008E-42	3.52	2.6183E-37	4.846E-41	Q14563	SEMA3A
1	SEMA3B	5.568E-186	-5.64	3.239E-181	1.335E-184	Q13214	SEMA3B
1	SEMA3D	4.7978E-16	1.76	2.7911E-11	3.3539E-15	O95025	SEMA3D
1	SEMA3E	1.4721E-81	-9.62	8.564E-77	2.2123E-80	O15041	SEMA3E
1	SEMA3F	0	-19.53	0	0	Q13275	SEMA3F
1	SEMA3G	7.6931E-11	-6.68	4.4754E-06	4.4897E-10	Q9NS98	SEMA3G
1	SEMA4A	0	-13.30	0	0	Q9H3S1	SEMA4A
1	SEMA4B	0	-4.16	0	0	Q9NPR2	SEMA4B
1	SEMA4C	2.8082E-31	1.53	1.6337E-26	2.6324E-30	Q9C0C4	SEMA4C
1	SEMA4D	2.8132E-25	1.76	1.6366E-20	2.3729E-24	Q92854	SEMA4D
1	SEMA4G	8.8777E-10	-2.10	5.1645E-05	4.9563E-09	Q9NTN9	SEMA4G
1	SEMA5A	1.3212E-28	2.13	7.6857E-24	1.1874E-27	Q13591	SEMA5A
1	SEMA6A	6.0084E-07	1.83	0.03495333	2.9196E-06	Q9H2E6	SEMA6A
1	SEMA6B	3.7021E-11	2.44	2.1536E-06	2.1878E-10	Q9H3T3	SEMA6B
1	SEMA6C	2.1852E-15	1.91	1.2712E-10	1.495E-14	Q9H3T2	SEMA6C
1	SEMA7A	6.591E-292	-8.94	3.834E-287	1.848E-290	O75326	SEMA7A
1	SENP5	0	-1.61	0	0	Q96HI0	SENP5

1	SEPHS1	1.347E-100	1.82	7.8334E-96	2.324E-99	P49903	SEPHS1
1	SEPHS2	5.3984E-40	1.74	3.1405E-35	5.6473E-39	Q99611	SEPHS2
1	SEPSECS	1.9323E-34	-2.12	1.1241E-29	1.8953E-33	Q9HD40	SEPSECS
1	SEPSECS-AS1	5.3311E-13	-3.00	3.1013E-08	3.3758E-12		
1	SEPT14P19	0.00134832	-3.74	1	0.00519315		
1	SEPT4	0.00138403	2.41	1	0.00532538	O43236	SEPT4
1	SEPT4-AS1	0.00082295	2.38	1	0.00322821		
1	SEPT5	5.86E-07	-1.72	0.03408975	2.8498E-06	Q99719	SEPT5
1	SEPT7	0	-1.57	0	0	Q16181	SEPT7
1	SEPT7-AS1	1.7512E-45	-4.10	1.0187E-40	1.961E-44		
1	SEPT7P2	0	-1.74	0	0		
1	SEPT8	1.3763E-35	1.97	8.0064E-31	1.3677E-34	Q92599	SEPT8
1	SERAC1	7.7673E-50	2.53	4.5186E-45	9.0789E-49	Q96JX3	SERAC1
1	SERINC2	0	-7.58	0	0	Q96SA4	SERINC2
1	SERINC5	0	-2.35	0	0	Q86VE9	SERINC5
1	SERP2	6.912E-170	-7.55	4.021E-165	1.581E-168	Q8N6R1	SERP2
1	SERPINA10	4.7662E-16	-6.27	2.7727E-11	3.3329E-15	Q9UK55	SERPINA10
1	SERPINA3	4.2016E-10	-4.68	2.4443E-05	2.3775E-09	P01011	SERPINA3
1	SERPINA5	4.2211E-08	7.72	0.0024556	2.18E-07	P05154	SERPINA5
1	SERPINB1	0	-4.27	0	0	P30740	SERPINB1
1	SERPINB11	0.00030255	-27.25	1	0.00122962	Q96P15	SERPINB11
1	SERPINB13	0	-66.42	0	0	Q9UIV8	SERPINB13
1	SERPINB2	3.698E-223	-28.39	2.151E-218	9.831E-222	P05120	SERPINB2
1	SERPINB3	1.1962E-37	-20.46	6.9588E-33	1.2157E-36	P29508	SERPINB3
1	SERPINB4	3.2504E-06	-40.27	0.18908865	1.5119E-05	P48594	SERPINB4
1	SERPINB5	0	-4.94	0	0	P36952	SERPINB5
1	SERPINB8	1.2596E-86	2.99	7.3277E-82	1.9703E-85	P50452	SERPINB8
1	SERPINB9	9.133E-176	-16.99	5.313E-171	2.13E-174	P50453	SERPINB9
1	SERPINB9P1	1.314E-138	-15.05	7.645E-134	2.736E-137		
1	SERPINE1	0	-1.99	0	0	P05121	SERPINE1
1	SERPINE2	8.128E-155	7.31	4.729E-150	1.775E-153	P07093	SERPINE2
1	SERPINE3	3.0215E-06	1.86	0.17577541	1.4085E-05	A8MV23	SERPINE3
1	SERPINF1	3.8062E-09	1.56	0.00022142	2.067E-08	P36955	SERPINF1
1	SERPINF2	2.3328E-78	-10.25	1.3571E-73	3.4089E-77	P08697	SERPINF2
1	SERPING1	0.00103025	-23.46	1	0.00401	P05155	SERPING1
1	SERPINI1	3.076E-157	-4.65	1.789E-152	6.775E-156	Q99574	SERPINI1
1	SERTAD1	4.6142E-29	1.91	2.6842E-24	4.1778E-28	Q9UHV2	SERTAD1
1	SERTAD4	1.4366E-84	-10.85	8.3576E-80	2.2151E-83	Q9NUC0	SERTAD4
1	SESN3	1.383E-202	-7.08	8.047E-198	3.515E-201	P58005	SESN3
1	SETBP1	4.4771E-76	2.62	2.6045E-71	6.4341E-75	Q9Y6X0	SETBP1
1	SETD1A	3.4044E-41	1.62	1.9805E-36	3.6187E-40	O15047	SETD1A
1	SETD3	9.647E-123	2.23	5.612E-118	1.892E-121	Q86TU7	SETD3
1	SETD9	1.8E-189	6.40	1.047E-184	4.368E-188	Q8NE22	SETD9
1	SETDB1	0	-2.21	0	0	Q15047	SETDB1
1	SETMAR	4.4059E-21	1.82	2.5631E-16	3.4293E-20	Q53H47	SETMAR
1	SEZ6L2	9.4635E-64	7.54	5.5053E-59	1.2419E-62	Q6UXD5	SEZ6L2
1	SF3A1	2.3022E-73	1.66	1.3393E-68	3.2358E-72	Q15459	SF3A1

1	SF3A3	1.0486E-65	1.88	6.0999E-61	1.3975E-64	Q12874	SF3A3
1	SF3B5	1.1808E-18	1.73	6.8691E-14	8.7616E-18	Q9BWJ5	SF3B5
1	SFMBT2	9.757E-185	129.29	5.676E-180	2.33E-183	Q5VUG0	SFMBT2
1	SFN	0	-3.56	0	0	P31947	SFN
1	SFRP4	2.5703E-07	-6.47	0.0149525	1.2734E-06	Q6FHJ7	SFRP4
1	SFT2D2	0	-1.83	0	0	O95562	SFT2D2
1	SFTA1P	4.4321E-42	2.45	2.5783E-37	4.7729E-41		
1	SFTPD	0.00019294	-7.59	1	0.00079817	P35247	SFTPD
1	SFXN2	8.3443E-77	2.05	4.8542E-72	1.2048E-75	Q96NB2	SFXN2
1	SFXN3	3.6332E-71	1.64	2.1136E-66	5.0227E-70	Q9BWM7	SFXN3
1	SFXN4	0	-1.93	0	0	Q6P4A7	SFXN4
1	SGCD	1.3098E-07	-7.10	0.00761963	6.5937E-07	Q92629	SGCD
1	SGCE	6.4172E-65	2.22	3.7332E-60	8.498E-64	O43556	SGCE
1	SGCG	2.8527E-07	6.71	0.01659558	1.4096E-06	Q13326	SGCG
1	SGF29	2.0834E-56	2.23	1.212E-51	2.5864E-55	Q96ES7	SGF29
1	SGIP1	2.8117E-56	29.41	1.6357E-51	3.4861E-55	Q9BQI5	SGIP1
1	SGK494	2.65E-27	-3.84	1.5416E-22	2.3235E-26	L7N484, Q96L	SGK494
1	SGMS1-AS1	1.032E-07	-1.60	0.0060037	5.2202E-07		
1	SGMS2	1.0929E-77	-2.82	6.3577E-73	1.5878E-76	Q8NHU3	SGMS2
1	SGO1-AS1	7.0589E-05	-2.23	1	0.00030115		
1	SGSH	7.925E-104	2.63	4.61E-99	1.4E-102	P51688	SGSH
1	SGSM2	1.009E-79	2.16	5.87E-75	1.494E-78	O43147	SGSM2
1	SGTA	1.3212E-19	2.41	7.6862E-15	9.9964E-19	O43765	SGTA
1	SGTB	2.13E-113	3.10	1.239E-108	3.978E-112	Q96EQ0	SGTB
1	SH2B2	2.2839E-16	2.14	1.3287E-11	1.6111E-15	O14492	SH2B2
1	SH2B3	7.6398E-58	2.89	4.4444E-53	9.5763E-57	Q9UQQ2	SH2B3
1	SH2D1B	0.00011632	-12.51	1	0.00048892	O14796	SH2D1B
1	SH2D2A	1.6643E-17	-8.20	9.6818E-13	1.2071E-16	Q9NP31	SH2D2A
1	SH2D3A	0	-4.97	0	0	Q9BRG2	SH2D3A
1	SH2D3C	1.8725E-37	-28.66	1.0893E-32	1.8998E-36	Q8N5H7	SH2D3C
1	SH2D4A	0	-2.59	0	0	Q9H788	SH2D4A
1	SH2D4B	6.2429E-28	-4.40	3.6317E-23	5.5505E-27		
1	SH2D5	1.2967E-86	2.87	7.5433E-82	2.0272E-85	Q6ZV89	SH2D5
1	SH3BGR	2.4312E-11	-2.43	1.4144E-06	1.4499E-10	P55822	SH3BGR
1	SH3BGRL3	1.7771E-81	2.46	1.0338E-76	2.6686E-80	Q9H299	SH3BGRL3
1	SH3BP1	0	-3.37	0	0	Q9Y3L3	SH3BP1
1	SH3BP5	5.482E-110	3.20	3.189E-105	1.003E-108	O60239	SH3BP5
1	SH3BP5L	0	-1.69	0	0	Q7L8J4	SH3BP5L
1	SH3D19	0	-1.78	0	0	Q5HYK7	SH3D19
1	SH3GL3	4.59E-148	-10.65	2.67E-143	9.835E-147	Q99963	SH3GL3
1	SH3KBP1	5.7284E-45	1.74	3.3324E-40	6.3754E-44	Q96B97	SH3KBP1
1	SH3PXD2A	0	-9.75	0	0	Q5TCZ1	SH3PXD2A
1	SH3PXD2A-AS	4.262E-190	-7.24	2.479E-185	1.038E-188		
1	SH3PXD2B	1.191E-115	32.91	6.929E-111	2.252E-114	A1X283	SH3PXD2B
1	SH3RF1	5.424E-31	1.75	3.1554E-26	5.0648E-30	Q7Z6J0	SH3RF1
1	SH3RF2	4.624E-263	-9.61	2.69E-258	1.281E-261	Q8TEC5	SH3RF2
1	SH3RF3	8.939E-175	23.38	5.2E-170	2.077E-173	Q8TEJ3	SH3RF3

1	SH3RF3-AS1	8.1605E-10	25.65	4.7473E-05	4.5617E-09		
1	SH3TC1	3.1886E-94	-6.82	1.855E-89	5.2773E-93	Q8TE82	SH3TC1
1	SH3TC2	4.2827E-69	-3.62	2.4914E-64	5.8251E-68	Q8TF17	SH3TC2
1	SH3YL1	0	-4.68	0	0	Q96HL8	SH3YL1
1	SHANK1	3.556E-121	25.39	2.069E-116	6.928E-120	Q9Y566	SHANK1
1	SHANK2	4.427E-130	-3.96	2.576E-125	8.927E-129	Q9UPX8	SHANK2
1	SHARPIN	0	-1.57	0	0	Q9H0F6	SHARPIN
1	SHC1	0	-1.75	0	0	P29353	SHC1
1	SHC1P2	1.0313E-05	-21.34	0.59997039	4.6553E-05		
1	SHC3	7.568E-120	49.63	4.403E-115	1.463E-118	Q92529	SHC3
1	SHC4	1.1965E-08	2.53	0.00069603	6.3535E-08	Q6S5L8	SHC4
1	SHF	9.517E-07	-2.96	0.05536445	4.5756E-06	Q7M4L6	SHF
1	SHISA2	1.9896E-47	41.46	1.1574E-42	2.2731E-46	Q6UWI4	SHISA2
1	SHISA3	9.213E-135	8.43	5.359E-130	1.888E-133	A0PJX4	SHISA3
1	SHMT1	2.3594E-12	2.22	1.3726E-07	1.462E-11	P34896	SHMT1
1	SHOX2	1.6999E-27	-3.71	9.8892E-23	1.4968E-26	O60902	SHOX2
1	SHPK	8.354E-128	2.28	4.86E-123	1.673E-126	Q9UHJ6	SHPK
1	SHROOM1	1.1653E-39	2.02	6.7793E-35	1.2138E-38	Q2M3G4	SHROOM1
1	SHROOM2	3.154E-124	-3.27	1.835E-119	6.222E-123	Q13796	SHROOM2
1	SHROOM3	1.331E-107	-17.52	7.74E-103	2.397E-106	Q8TF72	SHROOM3
1	SHROOM4	8.776E-34	13.42	5.1054E-29	8.5403E-33	Q9ULL8	SHROOM4
1	SHTN1	7.5873E-13	-1.59	4.4138E-08	4.7789E-12	A0MZ66	SHTN1
1	SIGIRR	0	-2.21	0	0	Q6IA17	SIGIRR
1	SIGLEC1	0.01431263	-3.26	1	0.0493611	Q9BZZ2	SIGLEC1
1	SIGLEC10	0.00057217	-2.60	1	0.00227609	Q96LC7	SIGLEC10
1	SIGLEC15	2.643E-211	5.90	1.537E-206	6.842E-210	Q6ZMC9	SIGLEC15
1	SIM1	5.5078E-23	-12.49	3.2041E-18	4.4483E-22	P81133	SIM1
1	SIM2	1.761E-105	3.74	1.024E-100	3.132E-104	Q14190	SIM2
1	SIMC1	3.166E-166	51.16	1.842E-161	7.161E-165	Q8NDZ2	SIMC1
1	SIPA1L1	1.263E-104	2.21	7.347E-100	2.241E-103	O43166	SIPA1L1
1	SIRPA	2.5315E-53	-2.24	1.4727E-48	3.0604E-52	P78324	SIRPA
1	SIRPB1	2.1247E-08	-3.86	0.00123602	1.1154E-07	O00241	SIRPB1
1	SIRPB2	1.1307E-22	-22.83	6.578E-18	9.0831E-22	Q5JXA9	SIRPB2
1	SIRT1	6.1042E-71	1.93	3.551E-66	8.4208E-70	Q96EB6	SIRT1
1	SIRT2	8.1824E-39	1.54	4.76E-34	8.4412E-38	Q8IXJ6	SIRT2
1	SIRT3	1.394E-83	2.33	8.1094E-79	2.1268E-82	Q9NTG7	SIRT3
1	SIRT4	0.0060712	-1.59	1	0.02186097	Q9Y6E7	SIRT4
1	SIRT6	8.2247E-38	1.64	4.7846E-33	8.3795E-37	Q8N6T7	SIRT6
1	SIVA1	1.537E-107	2.86	8.94E-103	2.768E-106	O15304	SIVA1
1	SIX1	1.6427E-29	1.78	9.5562E-25	1.4988E-28	Q15475	SIX1
1	SIX2	2.2732E-32	-6.10	1.3224E-27	2.1658E-31	Q9NPC8	SIX2
1	SIX5	2.4802E-27	2.02	1.4428E-22	2.1772E-26	Q8N196	SIX5
1	SKA1	2.223E-110	3.04	1.293E-105	4.082E-109	Q96BD8	SKA1
1	SKA3	1.1435E-29	1.60	6.6521E-25	1.0461E-28	Q8IX90	SKA3
1	SKI	3.2068E-81	2.91	1.8655E-76	4.8068E-80	P12755	SKI
1	SKIDA1	4.3804E-08	-2.46	0.00254824	2.2603E-07		
1	SKIV2L2	1.9547E-82	2.18	1.1371E-77	2.9598E-81	P42285	SKIV2L2

1	SKP1	1.0704E-16	1.52	6.2272E-12	7.6258E-16	P63208	SKP1
1	SKP2	0	-2.41	0	0	Q13309	SKP2
1	SLAIN1	6.446E-104	-16.35	3.75E-99	1.139E-102	Q8ND83	SLAIN1
1	SLC10A3	1.8242E-53	1.77	1.0612E-48	2.2086E-52	P09131	SLC10A3
1	SLC10A4	0.00638251	3.79	1	0.02291705	Q96EP9	SLC10A4
1	SLC10A5	9.729E-09	-3.74	0.00056598	5.1872E-08	Q5PT55	SLC10A5
1	SLC10A7	1.174E-108	-2.85	6.828E-104	2.131E-107	Q0GE19	SLC10A7
1	SLC12A7	0	-12.34	0	0	Q9Y666	SLC12A7
1	SLC12A8	5.177E-192	-12.12	3.012E-187	1.27E-190	A0AV02	SLC12A8
1	SLC12A9	0	-1.71	0	0	Q9BXP2	SLC12A9
1	SLC13A3	6.147E-166	-4.95	3.576E-161	1.388E-164	Q8WWT9	SLC13A3
1	SLC13A4	3.3267E-08	-2.14	0.0019353	1.7278E-07	Q9UKG4	SLC13A4
1	SLC14A2	4.8492E-32	-6.48	2.821E-27	4.5944E-31	Q15849	SLC14A2
1	SLC15A1	2.2516E-13	-9.48	1.3098E-08	1.4427E-12	P46059	SLC15A1
1	SLC15A2	1.2013E-92	-7.23	6.9883E-88	1.9652E-91	Q16348	SLC15A2
1	SLC15A3	4.886E-194	-23.41	2.842E-189	1.208E-192	Q8IY34	SLC15A3
1	SLC16A12	5.913E-148	-24.20	3.44E-143	1.266E-146	Q6ZSM3	SLC16A12
1	SLC16A14	3.873E-197	-4.78	2.253E-192	9.692E-196	Q7RTX9	SLC16A14
1	SLC16A2	2.771E-108	25.63	1.612E-103	5.019E-107	P36021	SLC16A2
1	SLC16A3	9.0819E-69	3.21	5.2833E-64	1.2315E-67	O15427	SLC16A3
1	SLC16A5	0	-1.99	0	0	O15375	SLC16A5
1	SLC16A6	2.0248E-33	7.16	1.1779E-28	1.958E-32	O15403	SLC16A6
1	SLC16A6P1	2.7433E-09	-2.08	0.00015959	1.4999E-08		
1	SLC16A7	3.382E-206	-10.68	1.968E-201	8.672E-205	O60669	SLC16A7
1	SLC16A8	3.7567E-06	-2.39	0.21854065	1.7398E-05	O95907	SLC16A8
1	SLC16A9	2.147E-135	-30.12	1.249E-130	4.412E-134	Q7RTY1	SLC16A9
1	SLC17A5	0	-1.61	0	0	Q9NRA2	SLC17A5
1	SLC17A9	3.2109E-97	3.18	1.8679E-92	5.4206E-96	Q9BYT1	SLC17A9
1	SLC18A2	4.765E-217	6.63	2.772E-212	1.253E-215	Q05940	SLC18A2
1	SLC18B1	0	-2.18	0	0	Q6NT16	SLC18B1
1	SLC19A1	7.2892E-64	2.13	4.2404E-59	9.5763E-63	P41440	SLC19A1
1	SLC19A2	8.8818E-16	-1.50	5.1669E-11	6.1437E-15	O60779	SLC19A2
1	SLC1A3	5.3375E-80	-22.19	3.105E-75	7.9149E-79	P43003	SLC1A3
1	SLC1A4	4.071E-220	23.24	2.368E-215	1.076E-218	P43007	SLC1A4
1	SLC1A5	0	-1.70	0	0	Q15758	SLC1A5
1	SLC1A6	0.00346341	-8.23	1	0.01281682	P48664	SLC1A6
1	SLC20A2	0	-2.20	0	0	Q08357	SLC20A2
1	SLC22A1	6.4429E-07	1.78	0.03748094	3.1258E-06	O15245	SLC22A1
1	SLC22A11	0.00094269	-5.56	1	0.00368003	Q9NSA0	SLC22A11
1	SLC22A14	0.00100835	-1.98	1	0.00392955	Q9Y267	SLC22A14
1	SLC22A17	9.038E-11	16.85	5.2578E-06	5.262E-10	Q8WUG5	SLC22A17
1	SLC22A18AS	1.0524E-07	-2.96	0.00612202	5.3207E-07	Q8N1D0	SLC22A18AS
1	SLC22A20	6.9764E-91	-10.78	4.0584E-86	1.1261E-89	A6NK97	SLC22A20
1	SLC22A23	3.5911E-83	-3.13	2.0891E-78	5.4631E-82	A1A5C7	SLC22A23
1	SLC23A3	7.1561E-17	2.61	4.163E-12	5.1199E-16	Q6PIS1	SLC23A3
1	SLC24A2	2.6781E-16	-8.68	1.558E-11	1.8871E-15	Q9UI40	SLC24A2
1	SLC24A3	6.5726E-08	-5.58	0.00382353	3.3581E-07	Q9HC58	SLC24A3

1	SLC25A10	2.524E-126	2.76	1.468E-121	5.023E-125	Q9UBX3	SLC25A10
1	SLC25A11	6.3733E-46	1.81	3.7076E-41	7.1659E-45	Q02978	SLC25A11
1	SLC25A14	4.0628E-27	1.68	2.3635E-22	3.5461E-26	O95258	SLC25A14
1	SLC25A17	1.715E-35	1.55	9.9771E-31	1.7026E-34	O43808	SLC25A17
1	SLC25A19	3.7496E-48	1.88	2.1813E-43	4.3202E-47	Q9HC21	SLC25A19
1	SLC25A20	3.9397E-29	1.96	2.2919E-24	3.571E-28	O43772	SLC25A20
1	SLC25A22	2.7625E-51	1.75	1.6071E-46	3.2678E-50	Q9H936	SLC25A22
1	SLC25A24	2.322E-29	1.61	1.3508E-24	2.1116E-28	Q6NUK1	SLC25A24
1	SLC25A27	1.0226E-89	-5.49	5.9486E-85	1.6356E-88	O95847	SLC25A27
1	SLC25A29	2.7625E-61	5.70	1.6071E-56	3.5547E-60	Q8N8R3	SLC25A29
1	SLC25A3	1.8167E-32	1.65	1.0569E-27	1.7334E-31	Q00325	SLC25A3
1	SLC25A33	5.9653E-30	1.78	3.4702E-25	5.4805E-29	Q9BSK2	SLC25A33
1	SLC25A34	2.1418E-26	-6.59	1.246E-21	1.8432E-25	Q6PIV7	SLC25A34
1	SLC25A35	0	-1.81	0	0	Q3KQZ1	SLC25A35
1	SLC25A36	0	-2.91	0	0	Q96CQ1	SLC25A36
1	SLC25A38	2.0208E-35	1.63	1.1756E-30	2.0037E-34	Q96DW6	SLC25A38
1	SLC25A4	0	-4.10	0	0	P12235	SLC25A4
1	SLC25A40	0	-2.20	0	0	Q8TBP6	SLC25A40
1	SLC25A41	0.00045751	3.91	1	0.00183363	Q8N5S1	SLC25A41
1	SLC25A42	2.0878E-66	3.06	1.2146E-61	2.7927E-65	Q86VD7	SLC25A42
1	SLC25A44	0	-1.93	0	0	Q96H78	SLC25A44
1	SLC25A45	3.1919E-30	3.67	1.8569E-25	2.9442E-29	Q8N413	SLC25A45
1	SLC25A46	2.8985E-58	1.86	1.6862E-53	3.6482E-57	Q96AG3	SLC25A46
1	SLC25A5	1.739E-20	1.61	1.0116E-15	1.3369E-19	P05141	SLC25A5
1	SLC25A51	1.2074E-67	2.07	7.0237E-63	1.6281E-66	Q9H1U9	SLC25A51
1	SLC25A6	1.6435E-78	2.21	9.5609E-74	2.4041E-77	P12236	SLC25A6
1	SLC26A11	8.1533E-42	2.55	4.7431E-37	8.7462E-41	Q86WA9	SLC26A11
1	SLC26A7	1.5124E-11	-2.40	8.7982E-07	9.0994E-11	Q8TE54	SLC26A7
1	SLC26A9	4.4944E-12	-95.90	2.6146E-07	2.7542E-11	Q7LBE3	SLC26A9
1	SLC27A3	6.079E-206	-5.05	3.536E-201	1.557E-204	Q5K4L6	SLC27A3
1	SLC27A5	4.4876E-13	-2.12	2.6106E-08	2.8485E-12	Q9Y2P5	SLC27A5
1	SLC27A6	1.1303E-07	-5.20	0.00657519	5.7086E-07	Q9Y2P4	SLC27A6
1	SLC28A1	0.00068767	-7.10	1	0.0027142	O00337	SLC28A1
1	SLC29A2	0	-10.81	0	0	Q14542	SLC29A2
1	SLC29A3	1.4731E-10	-1.54	8.5697E-06	8.5085E-10	Q9BZD2	SLC29A3
1	SLC2A1	0	-4.86	0	0	P11166	SLC2A1
1	SLC2A1-AS1	2.855E-54	-10.30	1.6609E-49	3.4849E-53		
1	SLC2A12	5.2911E-39	-2.74	3.0781E-34	5.478E-38	Q8TD20	SLC2A12
1	SLC2A13	7.391E-139	-3.85	4.299E-134	1.541E-137	Q96QE2	SLC2A13
1	SLC2A3	2.566E-201	230.64	1.493E-196	6.501E-200	P11169	SLC2A3
1	SLC2A4	9.248E-14	-3.49	5.3799E-09	6.0084E-13	P14672	SLC2A4
1	SLC2A4RG	2.7676E-51	1.71	1.61E-46	3.2731E-50	Q9NR83	SLC2A4RG
1	SLC2A8	2.7649E-96	3.46	1.6085E-91	4.6367E-95	Q9NY64	SLC2A8
1	SLC2A9	1.393E-121	-11.51	8.106E-117	2.718E-120	Q9NRM0	SLC2A9
1	SLC30A10	2.9405E-06	-3.73	0.17106012	1.3719E-05	Q6XR72	SLC30A10
1	SLC30A3	0.01196371	-7.01	1	0.04160799	Q99726	SLC30A3
1	SLC30A4	2.1582E-06	1.56	0.12555235	1.0169E-05	O14863	SLC30A4

1	SLC30A8	4.567E-30	-10.13	2.6568E-25	4.2018E-29	Q8IWU4	SLC30A8
1	SLC33A1	0	-1.90	0	0	O00400	SLC33A1
1	SLC34A1	2.2449E-06	-8.44	0.13059364	1.0558E-05	Q06495	SLC34A1
1	SLC34A2	2.2567E-26	30.30	1.3128E-21	1.9403E-25	O95436	SLC34A2
1	SLC34A3	1.8319E-05	-2.36	1	8.1339E-05	Q8N130	SLC34A3
1	SLC35A3	2.2204E-16	-1.75	1.2917E-11	1.5667E-15	Q9Y2D2	SLC35A3
1	SLC35B4	5.5277E-34	1.63	3.2157E-29	5.3946E-33	Q969S0	SLC35B4
1	SLC35C1	1.6167E-31	1.73	9.4052E-27	1.5197E-30	Q96A29	SLC35C1
1	SLC35E2B	3.0833E-53	1.79	1.7937E-48	3.7237E-52	P0CK96	SLC35E2B
1	SLC35E3	2.7732E-25	1.52	1.6133E-20	2.3397E-24	Q7Z769	SLC35E3
1	SLC35E4	9.398E-89	3.80	5.4672E-84	1.495E-87	Q6ICL7	SLC35E4
1	SLC35F2	2.2758E-41	1.55	1.3239E-36	2.4248E-40	Q8IXU6	SLC35F2
1	SLC35F3	3.3929E-22	-7.29	1.9738E-17	2.6964E-21	Q8IY50	SLC35F3
1	SLC36A1	8.8818E-16	-1.51	5.1669E-11	6.1437E-15	Q7Z2H8	SLC36A1
1	SLC36A4	4.4584E-21	1.61	2.5936E-16	3.4697E-20	Q6YBV0	SLC36A4
1	SLC37A1	1.743E-185	-11.11	1.014E-180	4.172E-184	P57057	SLC37A1
1	SLC37A2	0	-5.33	0	0	Q8TED4	SLC37A2
1	SLC38A10	0	-1.61	0	0	Q9HBR0	SLC38A10
1	SLC38A4	1.91E-239	-17.68	1.111E-234	5.169E-238	Q969I6	SLC38A4
1	SLC38A5	0	-8.82	0	0	Q8WUX1	SLC38A5
1	SLC39A1	0	-1.78	0	0	Q9NY26	SLC39A1
1	SLC39A11	0	-1.69	0	0	Q8N1S5	SLC39A11
1	SLC39A13	1.1221E-79	2.21	6.5275E-75	1.6605E-78	Q96H72	SLC39A13
1	SLC39A3	2.2667E-51	2.13	1.3186E-46	2.6861E-50	Q9BRY0	SLC39A3
1	SLC39A4	0	-1.82	0	0	Q6P5W5	SLC39A4
1	SLC39A8	0	-3.29	0	0	Q9C0K1	SLC39A8
1	SLC3A2	0	-7.01	0	0	P08195	SLC3A2
1	SLC40A1	5.5299E-25	-10.54	3.217E-20	4.6381E-24	Q9NP59	SLC40A1
1	SLC41A1	0	-1.94	0	0	Q8IVJ1	SLC41A1
1	SLC41A2	4.1102E-21	-1.91	2.3911E-16	3.2013E-20	Q96JW4	SLC41A2
1	SLC43A1	1.5517E-36	5.49	9.0269E-32	1.5604E-35	O75387	SLC43A1
1	SLC43A2	3.1735E-61	2.51	1.8462E-56	4.0808E-60	Q8N370	SLC43A2
1	SLC43A3	2.0401E-62	-6.18	1.1868E-57	2.6492E-61	Q8NBI5	SLC43A3
1	SLC44A1	0	-2.53	0	0	Q8WWI5	SLC44A1
1	SLC44A2	0	-4.04	0	0	Q8IWA5	SLC44A2
1	SLC44A3	1.1076E-75	-19.09	6.4433E-71	1.5843E-74	Q8N4M1	SLC44A3
1	SLC44A3-AS1	1.724E-196	-6.73	1.003E-191	4.303E-195		
1	SLC44A4	3.654E-134	-24.38	2.126E-129	7.469E-133	Q53GD3	SLC44A4
1	SLC44A5	0	-5.62	0	0	Q8NCS7	SLC44A5
1	SLC45A1	8.4949E-74	21.71	4.9418E-69	1.1983E-72	Q9Y2W3	SLC45A1
1	SLC45A4	0	-2.11	0	0	Q5BKX6	SLC45A4
1	SLC46A2	9.1926E-10	-3.24	5.3477E-05	5.1277E-09	Q9BY10	SLC46A2
1	SLC47A1	3.5206E-12	-7.09	2.0481E-07	2.1671E-11	Q96FL8	SLC47A1
1	SLC47A2	0	-13.65	0	0	Q86VL8	SLC47A2
1	SLC48A1	0	-6.85	0	0	Q6P1K1	SLC48A1
1	SLC4A10	1.4377E-08	-2.43	0.00083636	7.6129E-08	Q6U841	SLC4A10
1	SLC4A11	0	-2.96	0	0	Q8NBS3	SLC4A11

1	SLC4A2	5.7115E-94	2.06	3.3226E-89	9.4419E-93	P04920	SLC4A2
1	SLC4A4	0.00968051	1.82	1	0.03401099	Q9Y6R1	SLC4A4
1	SLC4A5	1.9846E-13	-2.68	1.1545E-08	1.2754E-12	Q9BY07	SLC4A5
1	SLC4A7	1.0161E-68	2.39	5.9112E-64	1.3776E-67	Q9Y6M7	SLC4A7
1	SLC4A8	3.4774E-45	4.77	2.023E-40	3.8806E-44	Q2Y0W8	SLC4A8
1	SLC4A9	7.6861E-13	-18.89	4.4713E-08	4.8402E-12	Q96Q91	SLC4A9
1	SLC51A	1.8719E-14	-2.77	1.089E-09	1.243E-13	Q86UW1	SLC51A
1	SLC52A1	5.8463E-94	-22.22	3.401E-89	9.6621E-93	Q9NWF4	SLC52A1
1	SLC52A3	2.7441E-92	-14.02	1.5963E-87	4.4803E-91	Q9NQ40	SLC52A3
1	SLC5A10	3.2233E-12	-5.32	1.8751E-07	1.9876E-11	A0PJK1	SLC5A10
1	SLC5A11	6.7507E-05	-3.31	1	0.00028838	Q8WWX8	SLC5A11
1	SLC5A12	1.0013E-84	-12.19	5.8251E-80	1.5455E-83	Q1EHB4	SLC5A12
1	SLC5A2	1.7941E-05	-3.77	1	7.9692E-05	P31639	SLC5A2
1	SLC5A4	1.0465E-10	38.54	6.0878E-06	6.0756E-10	Q9NY91	SLC5A4
1	SLC5A6	2.8005E-27	1.52	1.6292E-22	2.4528E-26	Q9Y289	SLC5A6
1	SLC6A12	4.0098E-05	-2.49	1	0.00017405	P48065	SLC6A12
1	SLC6A13	7.2219E-26	15.84	4.2013E-21	6.1512E-25	Q9NSD5	SLC6A13
1	SLC6A14	6.7485E-22	-35.32	3.9258E-17	5.334E-21	Q9UN76	SLC6A14
1	SLC6A15	0	-12.73	0	0	Q9H2J7	SLC6A15
1	SLC6A16	6.1581E-16	2.46	3.5824E-11	4.2903E-15	Q9GZN6	SLC6A16
1	SLC6A17	1.7178E-06	5.14	0.0999315	8.1384E-06	Q9H1V8	SLC6A17
1	SLC6A6	0	-1.93	0	0	P31641	SLC6A6
1	SLC6A7	1.7042E-19	-12.57	9.9141E-15	1.2864E-18	Q99884	SLC6A7
1	SLC6A8	0	-1.54	0	0	P48029	SLC6A8
1	SLC6A9	2.187E-257	-7.24	1.272E-252	6.011E-256	P48067	SLC6A9
1	SLC7A1	0	-1.75	0	0	P30825	SLC7A1
1	SLC7A11	5.9511E-07	-1.51	0.03461988	2.8932E-06	Q9UPY5	SLC7A11
1	SLC7A5	0	-10.43	0	0	Q01650	SLC7A5
1	SLC7A8	2.2773E-58	-7.50	1.3248E-53	2.8681E-57	Q9UHI5	SLC7A8
1	SLC8A1-AS1	7.3032E-11	-8.77	4.2486E-06	4.2674E-10		
1	SLC8A2	0.00756546	-5.28	1	0.02697042	Q9UPR5	SLC8A2
1	SLC8B1	6.9027E-43	1.87	4.0155E-38	7.5085E-42	Q6J4K2	SLC8B1
1	SLC9A1	0	-1.82	0	0	P19634	SLC9A1
1	SLC9A2	2.583E-16	-6.10	1.5026E-11	1.8205E-15	Q9UBY0	SLC9A2
1	SLC9A3R1	0	-2.32	0	0	O14745	SLC9A3R1
1	SLC9A3R2	4.4189E-35	3.56	2.5707E-30	4.3615E-34	Q15599	SLC9A3R2
1	SLC9A5	5.481E-12	1.87	3.1885E-07	3.3479E-11	Q14940	SLC9A5
1	SLC9A9	1.0386E-37	-2.46	6.042E-33	1.0563E-36	Q8IVB4	SLC9A9
1	SLC9B1	3.0778E-05	-2.02	1	0.00013463	Q4ZJI4	SLC9B1
1	SLC9C1	5.3391E-22	-4.88	3.106E-17	4.2287E-21	Q4G0N8	SLC9C1
1	SLCO1A2	4.112E-171	-10.07	2.392E-166	9.428E-170	P46721	SLCO1A2
1	SLCO1B1	5.7539E-23	-3.60	3.3472E-18	4.6438E-22	Q9Y6L6	SLCO1B1
1	SLCO1B3	0	-2.03	0	0	Q9NPD5	SLCO1B3
1	SLCO1B7	3.7507E-07	-2.49	0.02181944	1.8419E-06	G3V0H7	SLCO1B7
1	SLCO2B1	0.0077075	4.86	1	0.02745552	O94956	SLCO2B1
1	SLCO3A1	9.9458E-25	10.40	5.7859E-20	8.3059E-24	Q9UIG8	SLCO3A1
1	SLCO4A1	2.532E-139	71.58	1.473E-134	5.295E-138	Q96BD0	SLCO4A1

1	SLCO4A1-AS1	7.5394E-24	65.03	4.386E-19	6.1896E-23		
1	SLCO4C1	6.569E-194	-20.67	3.822E-189	1.623E-192	Q6ZQN7	SLCO4C1
1	SLCO5A1	8.5411E-05	4.74	1	0.00036231	Q9H2Y9	SLCO5A1
1	SLCO6A1	7.8858E-37	-13.96	4.5875E-32	7.9437E-36	Q86UG4	SLCO6A1
1	SLF2	3.7466E-31	1.71	2.1795E-26	3.5069E-30	Q8IX21	SLF2
1	SLFN11	0	131.07	0	0	Q7Z7L1	SLFN11
1	SLFN12	8.4908E-25	1.73	4.9394E-20	7.102E-24	Q8IYM2	SLFN12
1	SLFN13	8.1801E-38	-2.45	4.7587E-33	8.3369E-37	Q68D06	SLFN13
1	SLFN14	0.00716617	-5.62	1	0.02559775	P0C7P3	SLFN14
1	SLFN5	0	-2.97	0	0	Q08AF3	SLFN5
1	SLIRP	9.2955E-43	2.33	5.4075E-38	1.01E-41	Q9GZT3	SLIRP
1	SLIT1	2.4389E-05	2.53	1	0.0001074	O75093	SLIT1
1	SLIT2	5.2769E-79	7.06	3.0698E-74	7.7677E-78	O94813	SLIT2
1	SLITRK3	2.9895E-46	-11.88	1.7391E-41	3.3737E-45	O94933	SLITRK3
1	SLITRK4	3.3051E-60	422.27	1.9227E-55	4.2192E-59	Q8IW52	SLITRK4
1	SLITRK5	1.84E-226	-12.94	1.07E-221	4.919E-225	O94991	SLITRK5
1	SLITRK6	2.3625E-66	-8.35	1.3743E-61	3.1587E-65	Q9H5Y7	SLITRK6
1	SLPI	3.389E-118	-16.87	1.972E-113	6.499E-117	P03973	SLPI
1	SLU7	1.5686E-54	1.91	9.1253E-50	1.9195E-53	O95391	SLU7
1	SLX1A-SULT1A	9.3684E-10	1.55	5.45E-05	5.2243E-09		
1	SMAD1	2.0261E-18	1.52	1.1787E-13	1.4958E-17	Q15797	SMAD1
1	SMAD3	0	-1.89	0	0	P84022	SMAD3
1	SMAD4	1.8609E-84	2.04	1.0826E-79	2.8678E-83	Q13485	SMAD4
1	SMAD6	8.6022E-50	-3.48	5.0042E-45	1.0047E-48	O43541	SMAD6
1	SMAD7	5.9806E-51	2.26	3.4792E-46	7.0528E-50	O15105	SMAD7
1	SMAD9	0.00105075	-1.76	1	0.0040849	O15198	SMAD9
1	SMAGP	5.241E-18	1.67	3.0489E-13	3.8399E-17	Q0VAQ4	SMAGP
1	SMARCA2	0	-1.59	0	0	P51531	SMARCA2
1	SMARCA4	1.4867E-29	1.77	8.6486E-25	1.3571E-28	P51532	SMARCA4
1	SMARCB1	1.1174E-32	1.55	6.5002E-28	1.0696E-31	Q12824	SMARCB1
1	SMARCD3	6.1241E-77	2.43	3.5626E-72	8.8512E-76	Q6STE5	SMARCD3
1	SMC1B	3.7556E-05	1.99	1	0.00016337	Q8NDV3	SMC1B
1	SMC2-AS1	1.6038E-10	-2.96	9.3299E-06	9.2467E-10		
1	SMC4	0	-1.58	0	0	Q9NTJ3	SMC4
1	SMC5-AS1	3.1137E-16	-3.29	1.8114E-11	2.1903E-15		
1	SMCO2	6.7637E-05	-1.74	1	0.00028891	A6NFE2	SMCO2
1	SMCR8	8.2357E-78	1.89	4.791E-73	1.1975E-76	Q8TEV9	SMCR8
1	SMG1P5	2.7685E-10	1.62	1.6106E-05	1.5798E-09		
1	SMG1P7	1.5725E-59	-2.33	9.1477E-55	1.9986E-58		
1	SMG7	0	-1.84	0	0	Q92540	SMG7
1	SMG7-AS1	1.2564E-08	-2.59	0.00073089	6.6651E-08		
1	SMILR	0.01408839	-7.02	1	0.04861367		
1	SMIM10	8.9E-84	58.59	5.1775E-79	1.3614E-82	Q96HG1	SMIM10
1	SMIM10L1	5.8042E-68	2.18	3.3765E-63	7.8397E-67	P0DMW3	SMIM10L1
1	SMIM10L2B	4.1994E-06	16.26	0.24429462	1.9384E-05		
1	SMIM11A	0.00847068	2.69	1	0.03000689	P58511	SMIM11A
1	SMIM13	1.4904E-70	3.48	8.6704E-66	2.0531E-69	P0DJ93	SMIM13

1	SMIM14	1.1189E-29	-2.24	6.5093E-25	1.0241E-28	Q96QK8	SMIM14
1	SMIM15	4.451E-47	2.09	2.5893E-42	5.0632E-46	Q7Z3B0	SMIM15
1	SMIM15-AS1	1.4734E-10	1.56	8.5716E-06	8.5094E-10		
1	SMIM17	5.2352E-12	21.24	3.0455E-07	3.2007E-11	P0DL12	SMIM17
1	SMIM19	1.094E-164	-4.74	6.363E-160	2.459E-163	Q96E16	SMIM19
1	SMIM2-AS1	7.1644E-05	-1.69	1	0.00030547		
1	SMIM22	1.9241E-07	-4.36	0.01119312	9.5955E-07	K7EJ46	SMIM22
1	SMIM23	7.9742E-05	42.30	1	0.00033903	A6NLE4	SMIM23
1	SMIM25	0.00287472	-8.83	1	0.01071327		
1	SMIM3	1.1277E-05	1.53	0.65604487	5.0746E-05	Q9BZL3	SMIM3
1	SMIM5	0.00053846	-2.68	1	0.00214669	Q71RC9	SMIM5
1	SMLR1	6.3957E-13	5.87	3.7206E-08	4.038E-12	H3BR10	SMLR1
1	SMO	1.8185E-90	3.10	1.0579E-85	2.9288E-89	Q99835	SMO
1	SMOC1	2.2603E-21	4.01	1.3149E-16	1.7697E-20	Q9H4F8	SMOC1
1	SMOX	4.8117E-44	4.89	2.7992E-39	5.3065E-43	Q9NWM0	SMOX
1	SMPD1	5.5121E-88	3.42	3.2066E-83	8.7184E-87	P17405	SMPD1
1	SMPD3	8.7689E-31	-3.00	5.1012E-26	8.162E-30	Q9NY59	SMPD3
1	SMPD4	8.2483E-53	1.60	4.7984E-48	9.9263E-52	Q9NXE4	SMPD4
1	SMPDL3A	1.4076E-46	-2.76	8.1887E-42	1.5925E-45	Q92484	SMPDL3A
1	SMPDL3B	5.983E-185	-12.68	3.481E-180	1.429E-183	Q92485	SMPDL3B
1	SMR3B	8.6523E-21	152.80	5.0334E-16	6.6987E-20	P02814	SMR3B
1	SMTN	1.7522E-35	3.04	1.0193E-30	1.7386E-34	P53814	SMTN
1	SMTNL1	2.1713E-12	-15.62	1.2631E-07	1.3475E-11	A8MU46	SMTNL1
1	SMTNL2	0.00112848	-4.53	1	0.00437536		
1	SMU1	1.5178E-82	1.88	8.8295E-78	2.3018E-81	Q2TAY7	SMU1
1	SMURF2	2.132E-109	4.97	1.24E-104	3.891E-108	Q9HAU4	SMURF2
1	SMYD2	0	-1.95	0	0	Q9NRG4	SMYD2
1	SMYD3	0	-1.53	0	0	Q9H7B4	SMYD3
1	SMYD4	6.2113E-26	1.53	3.6134E-21	5.2943E-25	Q8IYR2	SMYD4
1	SNAI1	1.8577E-35	8.12	1.0807E-30	1.8423E-34	O95863	SNAI1
1	SNAI2	0	-1.94	0	0	O43623	SNAI2
1	SNAI3	1.2874E-07	-3.20	0.00748953	6.4828E-07	Q3KNW1	SNAI3
1	SNAP25	3.63E-171	457.94	2.112E-166	8.33E-170	P60880	SNAP25
1	SNAP25-AS1	3.8146E-09	3.87	0.00022191	2.0712E-08		
1	SNAP29	1.4762E-89	2.13	8.5876E-85	2.3592E-88	O95721	SNAP29
1	SNAP91	2.5254E-13	-7.35	1.4692E-08	1.6148E-12	O60641	SNAP91
1	SNAPC2	6.0555E-85	3.49	3.5227E-80	9.3664E-84	Q13487	SNAPC2
1	SNAPC4	3.0535E-13	1.55	1.7764E-08	1.9467E-12	Q5SXM2	SNAPC4
1	SNCA	1.435E-125	-15.67	8.347E-121	2.843E-124	P37840	SNCA
1	SNCA-AS1	0.00221501	-25.61	1	0.00834127		
1	SNCAIP	5.784E-191	-7.70	3.365E-186	1.413E-189	Q9Y6H5	SNCAIP
1	SNCG	0	-12.86	0	0	O76070	SNCG
1	SNHG1	0	-1.52	0	0		
1	SNHG14	1.046E-147	87.38	6.086E-143	2.238E-146		
1	SNHG16	3.5797E-31	3.00	2.0825E-26	3.3513E-30		
1	SNHG22	0.00026643	5.55	1	0.00108737		
1	SNHG3	1.9346E-27	1.50	1.1254E-22	1.7021E-26		

1	SNHG4	8.3659E-30	1.76	4.8668E-25	7.6738E-29		
1	SNHG7	7.0718E-85	2.19	4.1139E-80	1.0924E-83		
1	SNHG8	1.1844E-18	1.73	6.8899E-14	8.787E-18		
1	SNN	0	-3.39	0	0	O75324	SNN
1	SNPH	4.365E-132	8.67	2.539E-127	8.86E-131	O15079	SNPH
1	SNRK	4.4465E-49	2.10	2.5867E-44	5.16E-48	Q9NRH2	SNRK
1	SNRK-AS1	0.00065481	6.71	1	0.00259083		
1	SNRNP25	1.095E-110	2.95	6.368E-106	2.015E-109	Q9BV90	SNRNP25
1	SNRPB2	4.4092E-32	1.79	2.565E-27	4.1789E-31	P08579	SNRPB2
1	SNRPD1	8.637E-23	1.60	5.0245E-18	6.9543E-22	P62314	SNRPD1
1	SNRPF	8.9637E-16	1.51	5.2146E-11	6.1997E-15	P62306	SNRPF
1	SNRPN	0	145.27	0	0	P63162	SNRPN
1	SNTA1	2.294E-129	2.76	1.335E-124	4.62E-128	Q13424	SNTA1
1	SNTG1	0.00279361	-2.64	1	0.010421	Q9NSN8	SNTG1
1	SNTG2	1.548E-236	101.22	9.006E-232	4.181E-235	Q9NY99	SNTG2
1	SNURF	3.7723E-13	20.12	2.1945E-08	2.3989E-12	Q9Y675	SNURF
1	SNW1	1.453E-41	1.79	8.4525E-37	1.5523E-40	Q13573	SNW1
1	SNX10	1.391E-14	-2.20	8.0918E-10	9.2604E-14	Q9Y5X0	SNX10
1	SNX15	6.6404E-72	2.10	3.863E-67	9.235E-71	Q9NRS6	SNX15
1	SNX16	7.7154E-26	-1.82	4.4883E-21	6.5696E-25	P57768	SNX16
1	SNX18	9.8685E-38	1.71	5.7409E-33	1.0038E-36	Q96RF0	SNX18
1	SNX18P26	6.855E-10	20.32	3.9878E-05	3.8444E-09		
1	SNX18P7	0.00212355	2.14	1	0.00801502		
1	SNX19P2	0.00035808	32.08	1	0.0014476		
1	SNX2	3.6661E-33	1.74	2.1327E-28	3.5304E-32	O60749	SNX2
1	SNX21	1.0986E-67	2.12	6.391E-63	1.4818E-66	Q969T3	SNX21
1	SNX24	2.2706E-33	1.73	1.3209E-28	2.1942E-32	Q9Y343	SNX24
1	SNX25	0	-2.34	0	0	Q9H3E2	SNX25
1	SNX30	0	-1.63	0	0	Q5VWJ9	SNX30
1	SNX31	0.00041187	-7.09	1	0.00165632	Q8N9S9	SNX31
1	SNX33	0	-2.46	0	0	Q8WV41	SNX33
1	SNX4	8.5765E-40	1.79	4.9893E-35	8.9478E-39	O95219	SNX4
1	SNX6	3.6499E-52	2.06	2.1233E-47	4.3608E-51	Q9UNH7	SNX6
1	SNX9	5.7978E-64	1.63	3.3728E-59	7.6256E-63	Q9Y5X1	SNX9
1	SOAT2	3.9558E-06	-3.02	0.23012489	1.8299E-05	O75908	SOAT2
1	SOBP	8.823E-189	4.97	5.132E-184	2.137E-187	A7XYQ1	SOBP
1	SOCS2	1.9281E-98	12.11	1.1217E-93	3.2826E-97	O14508	SOCS2
1	SOCS2-AS1	1.175E-183	12.99	6.837E-179	2.797E-182		
1	SOCS3	2.7075E-88	3.14	1.5751E-83	4.2929E-87	O14543	SOCS3
1	SOCS5	5.9053E-71	2.33	3.4354E-66	8.1484E-70	O75159	SOCS5
1	SOCS6	3.6E-126	4.40	2.094E-121	7.16E-125	O14544	SOCS6
1	SOD2	1.8078E-83	1.95	1.0517E-78	2.7559E-82	P04179	SOD2
1	SOD3	1.5871E-21	-14.54	9.2328E-17	1.2468E-20	P08294	SOD3
1	SOGA3_1	2.8052E-51	4.73	1.6319E-46	3.3168E-50		
1	SOHLH2	1.9364E-05	-9.69	1	8.5801E-05	Q9NX45	SOHLH2
1	SON	0	-1.51	0	0	P18583	SON
1	SORBS1	1.0135E-66	-4.01	5.8958E-62	1.3572E-65	Q9BX66	SORBS1

1	SORBS2	1.2563E-39	-2.91	7.3085E-35	1.3077E-38	O94875	SORBS2
1	SORCS1	0.00341845	-4.13	1	0.01265757	Q8WY21	SORCS1
1	SORCS2	9.636E-12	3.33	5.6056E-07	5.8392E-11	Q96PQ0	SORCS2
1	SORL1	0	-15.57	0	0	Q92673	SORL1
1	SORT1	2.9847E-35	3.11	1.7363E-30	2.9529E-34	Q99523	SORT1
1	SOSTDC1	1.1215E-12	-10.78	6.524E-08	7.0203E-12	Q6X4U4	SOSTDC1
1	SOWAHB	1.448E-68	-43.90	8.4236E-64	1.9612E-67		
1	SOWAHC	5.882E-52	1.84	3.4218E-47	7.0119E-51		
1	SOWAHD	0.004205	-2.46	1	0.01542772		
1	SOX12	6.8326E-52	4.42	3.9748E-47	8.1367E-51	O15370	SOX12
1	SOX13	0	-3.81	0	0	Q9UN79	SOX13
1	SOX18	6.4156E-16	16.99	3.7322E-11	4.4665E-15	P35713	SOX18
1	SOX2	9.9327E-06	-1.51	0.57782202	4.4879E-05	P48431	SOX2
1	SOX2-OT	8.5072E-06	-1.91	0.49490012	3.8622E-05		
1	SOX4	1.5654E-97	15.02	9.1063E-93	2.6479E-96	Q06945	SOX4
1	SOX5	4.7458E-06	-3.30	0.27608392	2.1847E-05	P35711	SOX5
1	SOX5P1	0.00942764	-19.48	1	0.03316063		
1	SOX7	0	-9.44	0	0	Q9BT81	SOX7
1	SOX9	9.062E-60	4.95	5.2717E-55	1.1538E-58	P48436	SOX9
1	SOX9-AS1	2.3264E-08	-2.85	0.00135335	1.2187E-07		
1	SP1	0	-1.60	0	0	P08047	SP1
1	SP140L	0	-1.92	0	0	Q9H930	SP140L
1	SP4	2.4234E-20	1.78	1.4098E-15	1.8579E-19	Q02446	SP4
1	SP5	0.00015388	2.83	1	0.00064015	Q6BEB4	SP5
1	SP6	6.3517E-34	-6.79	3.695E-29	6.1925E-33	Q3SY56	SP6
1	SP8	0.00034176	-10.66	1	0.00138385	Q8IXZ3	SP8
1	SP9	6.0243E-05	-32.80	1	0.00025828	P0CG40	SP9
1	SPACA6	2.264E-08	1.59	0.00131707	1.1868E-07	W5XKT8	SPACA6
1	SPACA9	8.665E-21	-1.78	5.0408E-16	6.7076E-20	Q96E40	SPACA9
1	SPAG1	0	-1.87	0	0	Q07617	SPAG1
1	SPAG17	3.87E-35	-6.12	2.2513E-30	3.821E-34	Q6Q759	SPAG17
1	SPAG4	0	-1.58	0	0	Q9NPE6	SPAG4
1	SPAG5-AS1	0.01167707	-1.77	1	0.04067189		
1	SPAG6	5.5105E-07	-1.75	0.03205676	2.6832E-06	O75602	SPAG6
1	SPANXA2-OT1	2.5578E-06	12.04	0.14879707	1.1986E-05	Q8N9U9	SPANXA2-OT1
1	SPARC	0	26.37	0	0	P09486	SPARC
1	SPARCL1	1.1104E-18	-5.23	6.4597E-14	8.2446E-18	Q14515	SPARCL1
1	SPATA12	7.1267E-07	-6.50	0.04145883	3.4509E-06		
1	SPATA13_1	1.0754E-13	-1.57	6.2562E-09	6.9692E-13		
1	SPATA17	1.5777E-12	-2.24	9.1783E-08	9.829E-12	Q96L03	SPATA17
1	SPATA18	1.931E-32	-15.44	1.1233E-27	1.8419E-31	Q8TC71	SPATA18
1	SPATA2L	8.4332E-18	1.51	4.9059E-13	6.1509E-17		
1	SPATA32	1.9777E-07	-8.59	0.0115052	9.8554E-07	Q96LK8	SPATA32
1	SPATA4	0.00704038	-3.13	1	0.02516542	Q8NEY3	SPATA4
1	SPATA46	0.00919032	-15.96	1	0.03240987	Q5T0L3	SPATA46
1	SPATA6L	2.7644E-40	-5.48	1.6081E-35	2.9017E-39		
1	SPATA7	9.4189E-25	1.98	5.4793E-20	7.8726E-24	Q9P0W8	SPATA7

1	SPATC1	2.3536E-07	-5.96	0.01369187	1.1683E-06	Q76KD6	SPATC1
1	SPATC1L	7.6546E-27	-2.08	4.453E-22	6.6344E-26	Q9H0A9	SPATC1L
1	SPC25	2.3883E-51	1.84	1.3893E-46	2.8285E-50	Q9HBM1	SPC25
1	SPCS2	0	-1.51	0	0	Q15005	SPCS2
1	SPCS3	0	-1.84	0	0	P61009	SPCS3
1	SPDL1	9.2401E-38	1.66	5.3753E-33	9.4024E-37	Q96EA4	SPDL1
1	SPDYA	3.9841E-10	2.09	2.3177E-05	2.2575E-09	Q5MJ70	SPDYA
1	SPDYC	0.00021692	-6.59	1	0.00089319	Q5MJ68	SPDYC
1	SPDYE1	2.492E-05	-3.92	1	0.00010963	Q8NFV5	SPDYE1
1	SPDYE12P	2.1269E-06	-2.83	0.12373285	1.0025E-05		
1	SPDYE16	2.7464E-13	-5.80	1.5977E-08	1.7539E-12	A6NNV3	SPDYE16
1	SPDYE2	3.8114E-08	-3.59	0.00221727	1.9732E-07	Q495Y8	SPDYE2
1	SPDYE2B	0.0071814	-2.72	1	0.02565056	A6NHP3	SPDYE2B
1	SPDYE3	9.4347E-32	-2.22	5.4885E-27	8.8984E-31	A6NKU9	SPDYE3
1	SPDYE5	0.00041885	-1.93	1	0.00168354	A6NIY4	SPDYE5
1	SPDYE6	1.8931E-11	-4.99	1.1013E-06	1.1343E-10	P0CI01	SPDYE6
1	SPDYE8P	0.00315502	-3.02	1	0.01171509		
1	SPECC1	0	-8.30	0	0	Q5M775	SPECC1
1	SPECC1L	8.152E-104	2.19	4.742E-99	1.439E-102	Q69YQ0	SPECC1L
1	SPECC1L-ADO	0.00189239	3.81	1	0.00718153		
1	SPEF1	3.4911E-08	4.35	0.00203092	1.8109E-07	Q9Y4P9	SPEF1
1	SPEF2	1.3578E-07	-1.51	0.00789915	6.832E-07		
1	SPEG	8.4574E-08	1.73	0.00491999	4.2981E-07	Q15772	SPEG
1	SPERT	0.00027061	-3.91	1	0.00110379	Q8NA61	SPERT
1	SPESP1	2.3096E-12	-2.11	1.3436E-07	1.4323E-11	Q6UW49	SPESP1
1	SPG11	0	-1.92	0	0	Q96JI7	SPG11
1	SPHK1	1.248E-109	2.94	7.262E-105	2.281E-108	Q9NYA1	SPHK1
1	SPHK2	5.5369E-30	1.61	3.2211E-25	5.0878E-29	Q9NRA0	SPHK2
1	SPIN2A	0.00503679	-1.55	1	0.01832001	Q99865	SPIN2A
1	SPINK5	5.58E-152	-11.50	3.246E-147	1.207E-150	Q9NQ38	SPINK5
1	SPINT1	0	-14.83	0	0	O43278	SPINT1
1	SPINT2	0	-9.42	0	0	O43291	SPINT2
1	SPIRE1	1.43E-162	2.15	8.32E-158	3.2E-161	Q08AE8	SPIRE1
1	SPIRE2	7.6594E-21	1.55	4.4558E-16	5.9347E-20	Q8WWL2	SPIRE2
1	SPNS2	6.133E-220	7.74	3.568E-215	1.619E-218	Q8IVW8	SPNS2
1	SPNS3	6.3636E-12	3.71	3.7019E-07	3.878E-11	Q6ZMD2	SPNS3
1	SPOCK1	0	94.34	0	0	Q08629	SPOCK1
1	SPOCK3	3.3397E-11	-2.43	1.9428E-06	1.9793E-10	Q9BQ16	SPOCK3
1	SPON2	3.1614E-23	-3.73	1.8391E-18	2.5643E-22	Q9BUD6	SPON2
1	SPP1	0	-6.88	0	0	P10451	SPP1
1	SPPL2A	0	-1.85	0	0	Q8TCT8	SPPL2A
1	SPR	4.0947E-40	1.73	2.3821E-35	4.2904E-39	P35270	SPR
1	SPRED1	4.0194E-78	2.56	2.3382E-73	5.8617E-77	Q7Z699	SPRED1
1	SPRED3	9.617E-180	10.77	5.595E-175	2.266E-178	Q2MJR0	SPRED3
1	SPRR1A	0.00010227	-8.19	1	0.00043154	P35321	SPRR1A
1	SPRR1B	9.8554E-45	-5.41	5.7333E-40	1.0931E-43	P22528	SPRR1B
1	SPRR2A	2.5231E-08	-14.01	0.00146777	1.3196E-07	P35326	SPRR2A

1	SPRR3	4.0111E-13	-9.10	2.3334E-08	2.5491E-12	Q9UBC9	SPRR3
1	SPRY1	9.9799E-60	-6.75	5.8057E-55	1.2701E-58	O43609	SPRY1
1	SPRY2	6.1608E-79	4.14	3.584E-74	9.0619E-78	O43597	SPRY2
1	SPRY3	5.1208E-10	-2.48	2.979E-05	2.8874E-09	O43610	SPRY3
1	SPRY4	8.827E-251	22.38	5.135E-246	2.414E-249	Q9C004	SPRY4
1	SPRYD4	0	-1.83	0	0	Q8WW59	SPRYD4
1	SPSB2	2.4949E-51	2.16	1.4514E-46	2.9535E-50	Q99619	SPSB2
1	SPTB	1.5836E-09	-2.11	9.2125E-05	8.7422E-09	P11277	SPTB
1	SPTBN2	0	-6.02	0	0	O15020	SPTBN2
1	SPTBN5	2.774E-22	-3.61	1.6137E-17	2.2073E-21	Q9NRC6	SPTBN5
1	SPTLC1	0	-1.54	0	0	O15269	SPTLC1
1	SPTLC3	1.955E-216	-13.91	1.137E-211	5.132E-215	Q9NUV7	SPTLC3
1	SPTSSA	3.5515E-55	1.75	2.066E-50	4.3661E-54	Q969W0	SPTSSA
1	SPTSSB	2.0275E-17	-14.22	1.1795E-12	1.4676E-16	Q8NFR3	SPTSSB
1	SPTY2D1-AS1	7.644E-08	-1.55	0.00444681	3.8932E-07	A0A0U1RR47	SPTY2D1-AS1
1	SPX	7.8276E-09	12.08	0.00045536	4.1892E-08	Q9BT56	SPX
1	SQLE	0	-2.16	0	0	Q14534	SQLE
1	SRA1	2.1791E-92	2.18	1.2677E-87	3.5589E-91	Q9HD15	SRA1
1	SRD5A1	0	-2.40	0	0	P18405	SRD5A1
1	SRD5A3	2.939E-97	-3.45	1.7097E-92	4.9629E-96	Q9H8P0	SRD5A3
1	SRD5A3-AS1	9.2336E-06	-3.16	0.53715316	4.1808E-05		
1	SREBF1	0	-6.34	0	0	P36956	SREBF1
1	SREBF2	0	-1.85	0	0	Q12772	SREBF2
1	SRFBP1	9.0489E-33	1.66	5.2641E-28	8.6723E-32	Q8NEF9	SRFBP1
1	SRGAP2	0	-1.50	0	0	O75044	SRGAP2
1	SRGAP2B	5.647E-90	2.81	3.2851E-85	9.0622E-89	P0DMP2	SRGAP2B
1	SRGAP2C	7.253E-226	50.14	4.219E-221	1.936E-224	P0DJJ0	SRGAP2C
1	SRGAP2D	3.3881E-55	11.09	1.971E-50	4.167E-54		
1	SRGAP3	4.3404E-70	-6.11	2.525E-65	5.9552E-69	O43295	SRGAP3
1	SRGN	0	100.66	0	0	P10124	SRGN
1	SRM	9.0045E-31	3.33	5.2383E-26	8.3772E-30	P19623	SRM
1	SRMS	0.00085686	2.00	1	0.00335623	Q9H3Y6	SRMS
1	SRP14-AS1	1.4468E-23	-2.51	8.4165E-19	1.1816E-22		
1	SRP19	1.3564E-23	1.65	7.8907E-19	1.1084E-22	P09132	SRP19
1	SRPK2	0	-1.74	0	0	P78362	SRPK2
1	SRPK3	3.2712E-20	2.82	1.903E-15	2.5052E-19	Q9UPE1	SRPK3
1	SRPX	5.798E-126	-7.69	3.373E-121	1.152E-124	P78539	SRPX
1	SRR	3.0372E-35	1.68	1.7669E-30	3.0044E-34	Q9GZT4	SRR
1	SRRD	4.4489E-31	1.87	2.5881E-26	4.1583E-30	Q9UH36	SRRD
1	SRRM2-AS1	6.6925E-05	-1.67	1	0.00028604		
1	SRRM3	4.3746E-16	-10.66	2.5449E-11	3.0654E-15		
1	SRSF6	5.2688E-11	2.01	3.0651E-06	3.0951E-10	Q13247	SRSF6
1	SRSF8	3.4203E-24	1.62	1.9897E-19	2.8255E-23	Q9BRL6	SRSF8
1	SS18	3.782E-99	1.60	2.1999E-94	6.4647E-98	Q15532	SS18
1	SSBP1	4.0117E-45	1.98	2.3338E-40	4.4734E-44	Q04837	SSBP1
1	SSBP2	2.444E-125	-7.14	1.422E-120	4.837E-124	P81877	SSBP2
1	SSBP4	1.537E-101	3.76	8.9409E-97	2.67E-100	Q9BWG4	SSBP4

1	SSC4D	2.9939E-18	-3.82	1.7417E-13	2.2035E-17	Q8WTU2	SSC4D
1	SSC5D	9.1054E-07	-2.62	0.05296992	4.3813E-06	A1L4H1	SSC5D
1	SSFA2	3.1657E-74	3.28	1.8416E-69	4.4786E-73	P28290	SSFA2
1	SSH1	0	-2.71	0	0	Q8WYL5	SSH1
1	SSH3	0	-7.58	0	0	Q8TE77	SSH3
1	SSNA1	1.596E-32	1.81	9.2843E-28	1.5238E-31	O43805	SSNA1
1	SSPN	6.282E-114	-6.96	3.655E-109	1.175E-112	Q14714	SSPN
1	SSR3	6.5117E-79	1.75	3.7881E-74	9.5732E-78	Q9UNL2	SSR3
1	SSR4	5.5801E-62	2.00	3.2462E-57	7.2234E-61	P51571	SSR4
1	SSR4P1	2.649E-08	2.33	0.00154102	1.3842E-07		
1	SSSCA1-AS1	4.3086E-20	-3.05	2.5065E-15	3.2941E-19		
1	SSX2IP	3.6716E-63	2.32	2.1359E-58	4.7933E-62	Q9Y2D8	SSX2IP
1	ST14	0	-11.53	0	0	Q9Y5Y6	ST14
1	ST20	8.304E-12	-1.68	4.8308E-07	5.0415E-11		
1	ST3GAL2	1.8857E-65	1.93	1.097E-60	2.5092E-64	Q16842	ST3GAL2
1	ST3GAL3	0	-2.13	0	0	Q11203	ST3GAL3
1	ST3GAL5	1.3304E-05	1.68	0.77395833	5.9613E-05	Q9UNP4	ST3GAL5
1	ST3GAL6	1.8091E-34	2.42	1.0524E-29	1.7757E-33	Q9Y274	ST3GAL6
1	ST3GAL6-AS1	0.00015808	3.41	1	0.00065709		
1	ST5	0	-2.00	0	0	P78524	ST5
1	ST6GALNAC1	8.3331E-07	-6.51	0.04847674	4.019E-06	Q9NSC7	ST6GALNAC1
1	ST6GALNAC2	4.0719E-82	-11.90	2.3688E-77	6.1495E-81	Q9UJ37	ST6GALNAC2
1	ST6GALNAC3	0.00227866	-3.20	1	0.00856767	Q8NDV1	ST6GALNAC3
1	ST6GALNAC5	3.434E-24	-4.99	1.9977E-19	2.8356E-23	Q9BVH7	ST6GALNAC5
1	ST6GALNAC6	3.538E-99	1.95	2.0584E-94	6.0524E-98	Q969X2	ST6GALNAC6
1	ST7-AS1	2.3295E-10	-2.67	1.3552E-05	1.3333E-09		
1	ST7L	5.5624E-32	1.59	3.2359E-27	5.2676E-31	Q8TDW4	ST7L
1	ST8SIA4	3.3725E-74	-14.72	1.9619E-69	4.7677E-73	Q92187	ST8SIA4
1	ST8SIA5	2.099E-18	13.60	1.2211E-13	1.5488E-17	O15466	ST8SIA5
1	STAB2	0.0001331	3.14	1	0.00055633	Q8WWQ8	STAB2
1	STAC	1.2289E-19	-8.41	7.1487E-15	9.3022E-19	Q99469	STAC
1	STAG3	1.8843E-27	-3.72	1.0962E-22	1.6586E-26	Q9UJ98	STAG3
1	STAG3L1	6.039E-08	1.72	0.00351315	3.0917E-07	P0CL83	STAG3L1
1	STAG3L2	0	-2.36	0	0	P0CL84	STAG3L2
1	STAG3L5P	0	-2.52	0	0		
1	STAG3L5P-PVI	0	-2.56	0	0		
1	STAMBPL1	0	-1.51	0	0	Q96FJ0	STAMBPL1
1	STAP2	0	-2.44	0	0	Q9UGK3	STAP2
1	STARD10	0	-1.81	0	0	Q9Y365	STARD10
1	STARD13	3.346E-73	2.15	1.9465E-68	4.6949E-72	Q9Y3M8	STARD13
1	STARD4	0	-1.53	0	0	Q96DR4	STARD4
1	STARD4-AS1	7.6863E-20	-3.53	4.4714E-15	5.8458E-19		
1	STARD5	5.4285E-47	-6.41	3.158E-42	6.1716E-46	Q9NSY2	STARD5
1	STARD8	1.1701E-13	-4.33	6.8071E-09	7.5727E-13	Q92502	STARD8
1	STARD9	2.476E-100	5.04	1.4405E-95	4.263E-99	Q9P2P6	STARD9
1	STAT1	0	-1.67	0	0	P42224	STAT1
1	STAT2	0	-1.72	0	0	P52630	STAT2

1	STAT4	8.4175E-61	-6.41	4.8968E-56	1.0779E-59	Q14765	STAT4
1	STAT5A	6.8323E-37	-7.26	3.9746E-32	6.8872E-36	P42229	STAT5A
1	STAT6	0	-3.23	0	0	P42226	STAT6
1	STAU1	3.5422E-21	2.49	2.0606E-16	2.7623E-20	O95793	STAU1
1	STBD1	0.00020037	-1.52	1	0.00082763	O95210	STBD1
1	STC1	0.00036268	4.51	1	0.00146549	P52823	STC1
1	STC2	3.395E-171	33.40	1.975E-166	7.795E-170	O76061	STC2
1	STEAP1	6.437E-226	-6.52	3.745E-221	1.719E-224	Q9UHE8	STEAP1
1	STEAP2	6.871E-283	-11.00	3.997E-278	1.919E-281	Q8NFT2	STEAP2
1	STEAP2-AS1	7.7659E-07	-4.89	0.04517717	3.7516E-06		
1	STEAP3	0	-2.21	0	0	Q658P3	STEAP3
1	STEAP4	2.122E-124	-25.64	1.234E-119	4.187E-123	Q687X5	STEAP4
1	STIM1	3.093E-118	2.49	1.799E-113	5.935E-117	Q13586	STIM1
1	STIM2	0	-1.74	0	0	Q9P246	STIM2
1	STIP1	0	-1.52	0	0	P31948	STIP1
1	STK10	2.5445E-69	1.82	1.4802E-64	3.4674E-68	O94804	STK10
1	STK17A	1.313E-103	1.94	7.639E-99	2.313E-102	Q9UEE5	STK17A
1	STK24-AS1	4.5785E-13	-3.27	2.6635E-08	2.9052E-12		
1	STK3	0	-2.19	0	0	Q13188	STK3
1	STK31	1.5211E-81	-8.15	8.8486E-77	2.2853E-80	Q9BXU1	STK31
1	STK32C	1.9205E-74	2.35	1.1173E-69	2.723E-73	Q86UX6	STK32C
1	STK33	1.5836E-43	-10.11	9.2127E-39	1.7386E-42	Q9BYT3	STK33
1	STK38L	1.4896E-23	1.56	8.6653E-19	1.2162E-22	Q9Y2H1	STK38L
1	STK4	1.8702E-70	1.97	1.088E-65	2.5745E-69	Q13043	STK4
1	STMN1	1.0441E-34	1.82	6.074E-30	1.0276E-33	P16949	STMN1
1	STMN3	1.363E-196	12.84	7.932E-192	3.406E-195	Q9NZ72	STMN3
1	STOM	2.203E-89	77.94	1.2816E-84	3.5179E-88	P27105	STOM
1	STOML2	8.6234E-51	2.00	5.0166E-46	1.0157E-49	Q9UJZ1	STOML2
1	STON1-GTF2A	0.01209226	-2.99	1	0.04201487		
1	STON2	0	-5.02	0	0	Q8WXE9	STON2
1	STOX2	8.456E-151	-10.35	4.919E-146	1.823E-149	Q9P2F5	STOX2
1	STPG2	1.0986E-29	-7.53	6.3908E-25	1.0056E-28		
1	STRA6	0	-12.13	0	0	Q9BX79	STRA6
1	STRA8	1.4719E-18	-6.68	8.5627E-14	1.09E-17	Q7Z7C7	STRA8
1	STRADB	1.5869E-79	2.99	9.2318E-75	2.3449E-78	Q9C0K7	STRADB
1	STRAP	6.5615E-96	2.28	3.8171E-91	1.0972E-94	Q9Y3F4	STRAP
1	STRC	6.6779E-05	-3.48	1	0.00028546	Q7RTU9	STRC
1	STRCP1	1.0708E-11	-32.48	6.229E-07	6.4758E-11	A6NGW2	STRCP1
1	STRIP2	5.5976E-15	1.60	3.2563E-10	3.7733E-14	Q9ULQ0	STRIP2
1	STRN3	4.3361E-43	1.74	2.5225E-38	4.7264E-42	Q13033	STRN3
1	STT3B	1.6875E-92	2.08	9.8168E-88	2.7591E-91	Q8TCJ2	STT3B
1	STUB1	9.908E-101	2.27	5.7638E-96	1.711E-99	Q9UNE7	STUB1
1	STX10	7.8222E-27	1.58	4.5505E-22	6.7766E-26	O60499	STX10
1	STX11	7.0185E-05	-1.59	1	0.00029951	O75558	STX11
1	STX16	6.579E-61	1.78	3.8272E-56	8.4375E-60	O14662	STX16
1	STX16-NPEPL1	0.01259564	1.75	1	0.04366998	H3BU86	STX16-NPEPL1
1	STX17-AS1	0.00072693	-4.03	1	0.0028645		

1	STX18-AS1	4.948E-17	-2.02	2.8785E-12	3.548E-16		
1	STX19	1.2325E-06	-6.99	0.07169901	5.8915E-06	Q8N4C7	STX19
1	STX1A	2.8323E-77	4.34	1.6477E-72	4.1038E-76	Q16623	STX1A
1	STX1B	1.4642E-16	2.85	8.5179E-12	1.0395E-15	P61266	STX1B
1	STX2	3.2835E-74	5.96	1.9102E-69	4.6442E-73	P32856	STX2
1	STX6	0	-2.33	0	0	O43752	STX6
1	STXBP1	1.5221E-65	1.88	8.8548E-61	2.0267E-64	P61764	STXBP1
1	STXBP5L	6.053E-09	3.70	0.00035213	3.2562E-08	Q9Y2K9	STXBP5L
1	STXBP6	0.00428064	2.58	1	0.01569334	Q8NFX7	STXBP6
1	STYK1	3.8297E-79	-3.55	2.2279E-74	5.6402E-78	Q6J9G0	STYK1
1	STYX	3.3751E-33	1.75	1.9634E-28	3.2513E-32	Q8WUJ0	STYX
1	SUCLA2-AS1	0.01232801	-1.76	1	0.04277013		
1	SUCLG2-AS1	5.0715E-45	2.57	2.9503E-40	5.6519E-44		
1	SUCNR1	1.9058E-07	48.80	0.01108659	9.5058E-07	Q9BXA5	SUCNR1
1	SUGP2	1.0014E-27	1.56	5.8254E-23	8.864E-27	Q8IX01	SUGP2
1	SUGT1P1	7.8314E-09	-2.70	0.00045558	4.1908E-08		
1	SULF1	0.00572804	-2.71	1	0.02070536	Q8IWU6	SULF1
1	SULF2	6.9862E-17	3.09	4.0642E-12	4.9996E-16	Q8IWU5	SULF2
1	SULT1A1	1.816E-153	-16.21	1.057E-148	3.95E-152	P50225	SULT1A1
1	SULT1A2	1.3196E-54	-26.16	7.6768E-50	1.6162E-53	P50226	SULT1A2
1	SULT1A4	0.01216973	-2.49	1	0.04227396	P0DMN0	SULT1A4
1	SULT1B1	0.00012159	4.08	1	0.00050992	O43704	SULT1B1
1	SULT2B1	3.4933E-06	-6.01	0.20321846	1.6211E-05	O00204	SULT2B1
1	SUMF1	0	-3.20	0	0	Q8NBK3	SUMF1
1	SUMF2	0	-2.06	0	0	Q8NBJ7	SUMF2
1	SUN1	0	-2.61	0	0	O94901	SUN1
1	SUN3	1.9993E-29	-11.56	1.1631E-24	1.8207E-28	Q8TAQ9	SUN3
1	SUPT4H1	3.4355E-40	1.97	1.9986E-35	3.603E-39	P63272	SUPT4H1
1	SUPV3L1	9.2011E-45	1.68	5.3526E-40	1.0207E-43	Q8IYB8	SUPV3L1
1	SURF2	1.9686E-24	1.72	1.1452E-19	1.6349E-23	Q15527	SURF2
1	SURF6	1.2361E-31	1.58	7.1912E-27	1.164E-30	O75683	SURF6
1	SUSD1	7.8765E-89	-5.06	4.5821E-84	1.254E-87	Q6UWL2	SUSD1
1	SUSD5	3.863E-107	6.93	2.247E-102	6.938E-106	O60279	SUSD5
1	SUV39H1	0	-1.52	0	0	O43463	SUV39H1
1	SUZ12P1	3.4195E-14	-1.60	1.9893E-09	2.2513E-13		
1	SV2A	0	-3.82	0	0	Q7L0J3	SV2A
1	SVEP1	5.34E-233	-9.98	3.106E-228	1.435E-231	Q4LDE5	SVEP1
1	SVIL	0	-1.77	0	0	O95425	SVIL
1	SVILP1	2.2528E-09	-1.81	0.00013105	1.2358E-08		
1	SVIP	7.601E-197	4.75	4.422E-192	1.899E-195	Q8NHG7	SVIP
1	SVOP	0.00123296	-4.36	1	0.00476585	Q8N4V2	SVOP
1	SWAP70	7.88E-122	2.13	4.584E-117	1.541E-120	Q9UH65	SWAP70
1	SYBU	4.412E-174	-8.75	2.566E-169	1.022E-172	Q9NX95	SYBU
1	SYCE1L	5.5535E-11	2.11	3.2307E-06	3.2574E-10	A8MT33	SYCE1L
1	SYCE2	7.9662E-25	4.36	4.6343E-20	6.6671E-24	Q6PIF2	SYCE2
1	SYCE3	6.0642E-06	-9.62	0.35277987	2.7776E-05	A1L190	SYCE3
1	SYCP1	3.4214E-05	-5.50	1	0.00014929	Q15431	SYCP1

1	SYCP2L	3.4533E-11	4.41	2.0089E-06	2.0445E-10	Q5T4T6	SYCP2L
1	SYDE1	6.303E-132	12.84	3.667E-127	1.278E-130	Q6ZW31	SYDE1
1	SYK	0.00026429	-3.57	1	0.00107874	P43405	SYK
1	SYN1	1.1017E-16	-5.77	6.409E-12	7.8427E-16	P17600	SYN1
1	SYN2	3.2699E-29	-5.47	1.9022E-24	2.9667E-28	Q92777	SYN2
1	SYNC	7.934E-32	-5.15	4.6155E-27	7.4976E-31	Q9H7C4	SYNC
1	SYNDIG1	3.9966E-05	-4.40	1	0.00017349	Q9H7V2	SYNDIG1
1	SYNE1	6.391E-109	3.12	3.718E-104	1.162E-107	Q8NF91	SYNE1
1	SYNE3	5.1907E-20	10.37	3.0196E-15	3.9607E-19	Q6ZMZ3	SYNE3
1	SYNE4	2.8625E-81	-9.11	1.6652E-76	4.2929E-80	Q8N205	SYNE4
1	SYNGAP1	0	-2.49	0	0	Q96PV0	SYNGAP1
1	SYNGR1	1.942E-175	12.03	1.13E-170	4.521E-174	O43759	SYNGR1
1	SYNGR2	3.4934E-43	2.18	2.0323E-38	3.8164E-42	O43760	SYNGR2
1	SYNGR3	3.1502E-21	5.45	1.8326E-16	2.4599E-20	O43761	SYNGR3
1	SYNJ2	1.385E-101	1.82	8.0564E-97	2.408E-100	O15056	SYNJ2
1	SYNJ2BP-COX	0.00457617	1.92	1	0.0167241	A0A087WUM	SYNJ2BP-COX
1	SYNM	9.2983E-55	4.62	5.4092E-50	1.1397E-53	O15061	SYNM
1	SYNPO	3.3517E-65	-10.45	1.9498E-60	4.4466E-64	Q8N3V7	SYNPO
1	SYNPO2	4.795E-36	-11.19	2.7895E-31	4.7896E-35	Q9UMS6	SYNPO2
1	SYNPO2L	3.2071E-08	-6.80	0.00186567	1.6672E-07	Q9H987	SYNPO2L
1	SYP	1.2798E-05	-1.54	0.74451738	5.7399E-05	P08247	SYP
1	SYPL1	0	-3.78	0	0	Q16563	SYPL1
1	SYPL2	0.0083953	3.02	1	0.02976161	Q5VXT5	SYPL2
1	SYS1	6.0052E-41	1.65	3.4934E-36	6.3529E-40	Q8N2H4	SYS1
1	SYS1-DBNDD2	0.00472659	2.84	1	0.0172424	H3BUS1	SYS1-DBNDD2
1	SYT1	8.3301E-72	-5.61	4.846E-67	1.1577E-70	P21579	SYT1
1	SYT11	9.4083E-10	-2.40	5.4732E-05	5.2455E-09	Q9BT88	SYT11
1	SYT12	0	-6.28	0	0	Q8IV01	SYT12
1	SYT14	9.9892E-18	-7.25	5.8111E-13	7.2757E-17	Q8NB59	SYT14
1	SYT15	5.4374E-91	-9.81	3.1631E-86	8.7938E-90	Q9BQS2	SYT15
1	SYT17	3.097E-23	-6.43	1.8016E-18	2.5124E-22	Q9BSW7	SYT17
1	SYT2	6.3215E-38	-6.06	3.6775E-33	6.4517E-37	Q8N9I0	SYT2
1	SYT5	8.8798E-16	26.93	5.1657E-11	6.1437E-15	O00445	SYT5
1	SYT7	8.9397E-33	-3.48	5.2006E-28	8.5691E-32	O43581	SYT7
1	SYT8	0	-9.75	0	0	Q8NBV8	SYT8
1	SYTL1	0	-11.46	0	0	Q8IYJ3	SYTL1
1	SYTL3	7.1241E-28	-3.02	4.1443E-23	6.3268E-27	Q4VX76	SYTL3
1	SYTL4	1.0234E-27	1.76	5.9538E-23	9.0566E-27	Q96C24	SYTL4
1	SYTL5	0.01029971	-2.42	1	0.03609273	Q8TDW5	SYTL5
1	SYVN1	0	-1.60	0	0	Q86TM6	SYVN1
1	SZT2-AS1	6.1502E-45	12.91	3.5778E-40	6.841E-44		
1	TAB3	0	-3.63	0	0	Q8N5C8	TAB3
1	TAC4	8.9925E-08	1.86	0.00523129	4.5624E-07	Q86UU9	TAC4
1	TACC2	0	-1.55	0	0	O95359	TACC2
1	TACO1	9.612E-31	1.80	5.5917E-26	8.9396E-30	Q9BSH4	TACO1
1	TACR1	4.8315E-07	-2.26	0.02810693	2.3603E-06	P25103	TACR1
1	TACR3	0.00182869	-3.67	1	0.00694853	P29371	TACR3

1	TACSTD2	0	-14.16	0	0	P09758	TACSTD2
1	TAF10	6.664E-114	2.41	3.876E-109	1.246E-112	Q12962	TAF10
1	TAF13	5.6348E-71	2.74	3.278E-66	7.7788E-70	Q15543	TAF13
1	TAF1A	0	-1.72	0	0	Q15573	TAF1A
1	TAF1A-AS1	3.1189E-05	-1.77	1	0.0001364		
1	TAF1B	0	-1.56	0	0	Q53T94	TAF1B
1	TAF1C	2.2204E-16	-1.53	1.2917E-11	1.5667E-15	Q15572	TAF1C
1	TAF4	4.4802E-48	1.73	2.6063E-43	5.1559E-47	O00268	TAF4
1	TAF4B	1.1272E-30	1.87	6.5576E-26	1.0477E-29	Q92750	TAF4B
1	TAF7	3.312E-116	8.62	1.926E-111	6.279E-115	Q15545	TAF7
1	TAF7L	3.763E-15	5.74	2.1891E-10	2.5559E-14	Q5H9L4	TAF7L
1	TAF9	5.5402E-24	1.69	3.223E-19	4.5554E-23	Q16594	TAF9
1	TAF9B	3.9594E-43	1.81	2.3033E-38	4.3174E-42	Q9HBM6	TAF9B
1	TAGAP	6.2497E-09	-72.12	0.00036357	3.3589E-08	Q8N103	TAGAP
1	TAGLN	9.3554E-61	4.87	5.4424E-56	1.1977E-59	Q01995	TAGLN
1	TAGLN3	3.9254E-07	-3.15	0.02283585	1.9261E-06	Q9UI15	TAGLN3
1	TALDO1	1.0499E-42	1.85	6.1074E-38	1.1392E-41	P37837	TALDO1
1	TANC1	0	-2.29	0	0	Q9C0D5	TANC1
1	TANC2	6.404E-111	4.11	3.726E-106	1.181E-109	Q9HCD6	TANC2
1	TAOK3	3.2763E-41	1.79	1.9059E-36	3.4837E-40	Q9H2K8	TAOK3
1	TAP1	0	-1.96	0	0	Q03518	TAP1
1	TAP2	0	-2.37	0	0	Q03519	TAP2
1	TAPBP	0	-4.77	0	0	O15533	TAPBP
1	TAPBPL	1.947E-100	-4.20	1.1326E-95	3.358E-99	Q9BX59	TAPBPL
1	TAPT1	0	-1.78	0	0	Q6NXT6	TAPT1
1	TAPT1-AS1	2.0576E-93	-8.90	1.197E-88	3.3851E-92		
1	TARBP1	0	-2.93	0	0	Q13395	TARBP1
1	TARID	1.0154E-10	-3.88	5.9071E-06	5.8982E-10		
1	TARM1	1.0072E-08	6.99	0.00058593	5.3657E-08	B6A8C7	TARM1
1	TARS2	0	-2.28	0	0	Q9BW92	TARS2
1	TARSL2	3.9319E-36	2.23	2.2873E-31	3.9315E-35	A2RTX5	TARSL2
1	TAS1R3	0.00204728	-2.05	1	0.00773567	Q7RTX0	TAS1R3
1	TAS2R20	3.0246E-05	-2.45	1	0.00013241	P59543	TAS2R20
1	TASP1	0	-1.97	0	0	Q9H6P5	TASP1
1	TBC1D1	0	-1.63	0	0	Q86TI0	TBC1D1
1	TBC1D12	1.8619E-14	1.51	1.0831E-09	1.2365E-13	O60347	TBC1D12
1	TBC1D16	1.2608E-78	2.00	7.3347E-74	1.8461E-77	Q8TBP0	TBC1D16
1	TBC1D2	0	-2.75	0	0	Q9BYX2	TBC1D2
1	TBC1D22B	0	-1.89	0	0	Q9NU19	TBC1D22B
1	TBC1D29	0.00015494	-4.11	1	0.00064439		
1	TBC1D2B	2.6334E-63	1.80	1.5319E-58	3.4418E-62	Q9UPU7	TBC1D2B
1	TBC1D3	1.8969E-05	49.43	1	8.4095E-05	Q8IZP1	TBC1D3
1	TBC1D30	1.7353E-30	-2.48	1.0095E-25	1.6075E-29	Q9Y2I9	TBC1D30
1	TBC1D31	0	-1.60	0	0	Q96DN5	TBC1D31
1	TBC1D32	0	-1.74	0	0	Q96NH3	TBC1D32
1	TBC1D3B	6.3926E-12	-2.48	3.7188E-07	3.8949E-11	A6NDS4	TBC1D3B
1	TBC1D3C	1.405E-17	61.47	8.1736E-13	1.0206E-16	Q6IPX1	TBC1D3C

1	TBC1D3D	7.5062E-10	131.33	4.3666E-05	4.2031E-09	A0A087WVF3	TBC1D3D
1	TBC1D3F	3.4213E-08	11.89	0.0019903	1.7756E-07	A6NER0	TBC1D3F
1	TBC1D3H	1.8748E-58	66.39	1.0906E-53	2.3633E-57	P0C7X1	TBC1D3H
1	TBC1D3L	1.251E-213	-7.81	7.28E-209	3.259E-212	B9A6J9	TBC1D3L
1	TBC1D4	0	-1.65	0	0	O60343	TBC1D4
1	TBC1D5	0	-1.86	0	0	Q92609	TBC1D5
1	TBC1D8-AS1	1.4715E-07	-2.14	0.00856043	7.3931E-07		
1	TBC1D8B	0	-1.92	0	0	Q0IIM8	TBC1D8B
1	TBCB	2.3253E-62	1.81	1.3527E-57	3.0175E-61	Q99426	TBCB
1	TBCE	0	-1.78	0	0	Q15813	TBCE
1	TBCK	0	-1.52	0	0	Q8TEA7	TBCK
1	TBKBP1	2.2137E-13	-1.74	1.2878E-08	1.4194E-12	A7MCY6	TBKBP1
1	TBL1X	1.871E-127	1.95	1.088E-122	3.739E-126	O60907	TBL1X
1	TBL1XR1	0	-3.34	0	0	Q9BZK7	TBL1XR1
1	TBL3	3.2421E-29	1.60	1.8861E-24	2.9419E-28	Q12788	TBL3
1	TBX1	3.6752E-13	-2.59	2.138E-08	2.3379E-12	O43435	TBX1
1	TBX15	5.5723E-09	7.55	0.00032416	3.0029E-08	Q96SF7	TBX15
1	TBX19	1.2061E-34	-3.40	7.0163E-30	1.1862E-33	O60806	TBX19
1	TBX2	2.1006E-17	1.77	1.222E-12	1.5193E-16	Q13207	TBX2
1	TBX20	1.539E-09	3.25	8.9532E-05	8.5017E-09	Q9UMR3	TBX20
1	TBX3	3.427E-161	79.95	1.994E-156	7.639E-160	O15119	TBX3
1	TBX6	5.2369E-91	-6.08	3.0465E-86	8.4719E-90	O95947	TBX6
1	TBXA2R	1.685E-68	5.86	9.8022E-64	2.2812E-67	P21731	TBXA2R
1	TBXAS1	1.627E-187	-5.30	9.463E-183	3.917E-186	P24557	TBXAS1
1	TC2N	1.0035E-97	-12.13	5.838E-93	1.7001E-96	Q8N9U0	TC2N
1	TCAF1	5.4506E-91	1.94	3.1708E-86	8.8128E-90	Q9Y4C2	TCAF1
1	TCAF1P1	6.6259E-40	3.76	3.8546E-35	6.9177E-39		
1	TCAF2	2.0207E-10	1.54	1.1755E-05	1.1597E-09	A6NFQ2	TCAF2
1	TCAF2P1	0.00010213	-8.79	1	0.00043098		
1	TCEA2	1.7496E-79	2.14	1.0178E-74	2.5847E-78	Q15560	TCEA2
1	TCEA3	4.6782E-18	-5.50	2.7215E-13	3.4328E-17	O75764	TCEA3
1	TCEAL3	1.7935E-74	2.22	1.0433E-69	2.5435E-73	Q969E4	TCEAL3
1	TCEAL4	3.7336E-29	1.74	2.172E-24	3.3847E-28	Q96EI5	TCEAL4
1	TCEAL8	3.7591E-27	1.94	2.1868E-22	3.2837E-26	Q8IYN2	TCEAL8
1	TCEAL9	5.72E-111	146.14	3.328E-106	1.055E-109	Q9UHQ7	TCEAL9
1	TCF19	1.6715E-27	2.78	9.7237E-23	1.4724E-26	Q9Y242	TCF19
1	TCF24	3.3709E-10	-6.62	1.961E-05	1.9156E-09	Q7RTU0	TCF24
1	TCF3	5.7303E-75	2.02	3.3336E-70	8.1585E-74	P15923	TCF3
1	TCF4	1.121E-187	5.96	6.523E-183	2.702E-186	P15884	TCF4
1	TCF7	1.8578E-19	-1.97	1.0807E-14	1.401E-18	P36402	TCF7
1	TCF7L1	5.7351E-22	-1.93	3.3364E-17	4.5374E-21	Q9HCS4	TCF7L1
1	TCFL5	1.3455E-45	1.65	7.8275E-41	1.5091E-44	Q9UL49	TCFL5
1	TCHH	2.5738E-27	-3.65	1.4973E-22	2.2584E-26	Q07283	TCHH
1	TCIRG1	0	-2.11	0	0	Q13488	TCIRG1
1	TCL6	5.439E-132	123.50	3.164E-127	1.103E-130		
1	TCN2	3.6049E-93	-5.14	2.0971E-88	5.9191E-92	P20062	TCN2
1	TCP1	3.8718E-43	1.65	2.2524E-38	4.225E-42	P17987	TCP1

1	TCP11X1	0.00090421	25.84	1	0.00353433		
1	TCTA	0	-1.84	0	0	P57738	TCTA
1	TCTEX1D2	0	-2.36	0	0	Q8WW35	TCTEX1D2
1	TCTEX1D4	0.00909816	-4.34	1	0.0321046	Q5JR98	TCTEX1D4
1	TCTN1	1.8201E-34	1.68	1.0588E-29	1.7862E-33	Q2MV58	TCTN1
1	TCTN3	8.3679E-34	1.58	4.8679E-29	8.1458E-33	Q6NUS6	TCTN3
1	TDO2	4.112E-110	-13.63	2.392E-105	7.537E-109	P48775	TDO2
1	TDP1	8.3962E-45	3.50	4.8844E-40	9.3197E-44	Q9NUW8	TDP1
1	TDP2	3.7566E-30	1.55	2.1854E-25	3.4617E-29	O95551	TDP2
1	TDRD6	2.0626E-07	-8.34	0.01199906	1.0271E-06	O60522	TDRD6
1	TDRD7	0	-1.84	0	0	Q8NHU6	TDRD7
1	TDRP	5.745E-121	71.96	3.342E-116	1.117E-119	Q86YL5	TDRP
1	TEAD1	3.469E-111	3.40	2.018E-106	6.407E-110	P28347	TEAD1
1	TEAD3	0	-2.23	0	0	Q99594	TEAD3
1	TEAD4	2.852E-102	2.41	1.6588E-97	4.979E-101	Q15561	TEAD4
1	TEC	2.1686E-26	-1.99	1.2616E-21	1.8659E-25	P42680	TEC
1	TECR	0	-2.10	0	0	Q9NZ01	TECR
1	TECRL	0.00573247	-19.83	1	0.02070536	Q5HYJ1	TECRL
1	TECTA	9.4572E-19	5.59	5.5016E-14	7.0373E-18	O75443	TECTA
1	TEF	0	-3.14	0	0	Q10587	TEF
1	TEK	0.0024451	-3.86	1	0.00916799	Q02763	TEK
1	TEKT2	0.00937555	3.17	1	0.03303732	Q9UIF3	TEKT2
1	TELO2	2.4901E-29	1.56	1.4486E-24	2.2631E-28	Q9Y4R8	TELO2
1	TEN1	3.2791E-22	1.77	1.9076E-17	2.6067E-21	Q86WV5	TEN1
1	TEN1-CDK3	3.2468E-07	-1.82	0.01888805	1.5991E-06		
1	TENM1	2.0465E-72	-14.43	1.1905E-67	2.8556E-71	Q9UKZ4	TENM1
1	TENM3	0	-20.30	0	0	Q9P273	TENM3
1	TES	0	-2.13	0	0	Q9UGI8	TES
1	TESC	1.7194E-05	8.00	1	7.6449E-05	Q96BS2	TESC
1	TESK1	1.5999E-43	1.65	9.3073E-39	1.7561E-42	Q15569	TESK1
1	TESK2	2.2316E-35	-2.37	1.2982E-30	2.2116E-34	Q96S53	TESK2
1	TESMIN	2.6596E-83	-11.10	1.5472E-78	4.0503E-82	Q9Y4I5	TESMIN
1	TET1	1.6214E-24	-2.98	9.4324E-20	1.3498E-23	Q8NFU7	TET1
1	TEX11	5.3966E-12	-6.61	3.1394E-07	3.2981E-11	Q8IYF3	TEX11
1	TEX19	2.6275E-30	-5.01	1.5285E-25	2.427E-29	Q8NA77	TEX19
1	TEX21P	3.0957E-06	-2.26	0.18008659	1.442E-05		
1	TEX22	1.4144E-88	3.07	8.2282E-84	2.2469E-87	C9J3V5	TEX22
1	TEX261	2.2236E-47	1.57	1.2936E-42	2.5369E-46	Q6UWH6	TEX261
1	TEX29	0.01029241	1.58	1	0.03606933	Q8N6K0	TEX29
1	TEX30	6.9015E-32	1.89	4.0149E-27	6.5261E-31	Q5JUR7	TEX30
1	TEX35	9.3147E-11	-4.96	5.4187E-06	5.4203E-10	Q5T0J7	TEX35
1	TEX40	1.109E-05	-3.15	0.64513315	4.9938E-05		
1	TF	0.0012049	-7.05	1	0.00466111	P02787	TF
1	TFAM	8.1031E-98	2.84	4.7139E-93	1.3739E-96	Q00059	TFAM
1	TFAP2A	0	-1.84	0	0	P05549	TFAP2A
1	TFAP2C	2.327E-153	25.72	1.354E-148	5.055E-152	Q92754	TFAP2C
1	TFAP2E	1.0575E-08	-1.88	0.00061517	5.6288E-08	Q6VUC0	TFAP2E

1	TFAP4	2.9507E-33	1.73	1.7165E-28	2.8443E-32	Q01664	TFAP4
1	TFDP2	0	-3.10	0	0	Q14188	TFDP2
1	TFEB	9.005E-215	-5.28	5.239E-210	2.352E-213	P19484	TFEB
1	TFF3	0.00140517	27.77	1	0.00539816	Q07654	TFF3
1	TFIP11	1.6923E-46	1.68	9.8449E-42	1.9131E-45	Q9UBB9	TFIP11
1	TFP1	2.0027E-06	-1.59	0.1165024	9.4541E-06		
1	TFPI	2.029E-171	8.37	1.18E-166	4.664E-170	P10646	TFPI
1	TFPI2	1.2069E-73	6.11	7.0208E-69	1.6991E-72	P48307	TFPI2
1	TFPT	2.0679E-45	1.81	1.203E-40	2.3134E-44	P0C1Z6	TFPT
1	TFR2	5.19E-126	-10.31	3.019E-121	1.032E-124	Q9UP52	TFR2
1	TFRC	0	-1.97	0	0	P02786	TFRC
1	TGFA	0	-5.38	0	0	P01135	TGFA
1	TGFB1I1	8.216E-56	5.23	4.7796E-51	1.0156E-54	O43294	TGFB1I1
1	TGFB2	2.25E-43	4.86	1.3089E-38	2.4655E-42	P61812	TGFB2
1	TGFB2-AS1	5.2757E-07	2.49	0.03069101	2.5719E-06		
1	TGFBI	0	-5.80	0	0	Q15582	TGFBI
1	TGFBR2	0	12.10	0	0	P37173	TGFBR2
1	TGFBR3	8.6521E-27	-2.33	5.0333E-22	7.49E-26	Q03167	TGFBR3
1	TGFBR3L	5.4719E-40	4.76	3.1832E-35	5.7232E-39	H3BV60	TGFBR3L
1	TGFBRAP1	1.7081E-57	1.86	9.9365E-53	2.1351E-56	Q8WUH2	TGFBRAP1
1	TGIF2	5.9338E-39	1.73	3.4519E-34	6.1335E-38	Q9GZN2	TGIF2
1	TGIF2LX	2.0079E-07	1.75	0.01168099	1.0005E-06	Q8IUE1	TGIF2LX
1	TGM1	5.8215E-76	-3.91	3.3866E-71	8.3516E-75	P22735	TGM1
1	TGM4	2.1408E-06	2.64	0.12453643	1.0088E-05	P49221	TGM4
1	TGM5	9.5847E-53	-33.81	5.5758E-48	1.1523E-51	O43548	TGM5
1	THAP3	1.0647E-41	2.32	6.194E-37	1.1401E-40	Q8WTV1	THAP3
1	THAP6	0	-1.90	0	0	Q8TBB0	THAP6
1	THAP7	5.6135E-39	1.70	3.2656E-34	5.8096E-38	Q9BT49	THAP7
1	THAP7-AS1	1.0902E-16	-2.02	6.3422E-12	7.7646E-16		
1	THBD	0	-4.42	0	0	P07204	THBD
1	THBS1	0	-6.06	0	0	P07996	THBS1
1	THBS3	0	-2.54	0	0	P49746	THBS3
1	THCAT158	1.065E-13	259.92	6.1954E-09	6.9021E-13		
1	THEM4	9.2033E-53	-2.35	5.3539E-48	1.1066E-51	Q5T1C6	THEM4
1	THEM5	1.4166E-47	-9.72	8.2412E-43	1.6207E-46	Q8N1Q8	THEM5
1	THEM6	0	-2.08	0	0	Q8WUY1	THEM6
1	THEMIS	2.5099E-06	-8.29	0.14601095	1.1766E-05	Q8N1K5	THEMIS
1	THEMIS2	1.5087E-79	-5.50	8.7769E-75	2.2299E-78	Q5TEJ8	THEMIS2
1	THG1L	6.5373E-85	2.91	3.803E-80	1.0106E-83	Q9NWX6	THG1L
1	THNSL1	4.2028E-23	-1.86	2.4449E-18	3.399E-22	Q8IYQ7	THNSL1
1	THNSL2	1.2849E-70	-13.14	7.4749E-66	1.7713E-69	Q86YJ6	THNSL2
1	THOC1	2.3312E-55	1.83	1.3561E-50	2.8719E-54	Q96FV9	THOC1
1	THOC5	2.53E-122	2.58	1.472E-117	4.955E-121	Q13769	THOC5
1	THOP1	4.8894E-87	2.25	2.8444E-82	7.6688E-86	P52888	THOP1
1	THRB	1.48E-118	-5.80	8.611E-114	2.846E-117	P10828	THRB
1	THRB-AS1	6.3307E-08	-6.75	0.00368284	3.237E-07		
1	THSD1	4.819E-123	27.00	2.803E-118	9.458E-122	Q9NS62	THSD1

1	THSD7A	9.3691E-05	-3.76	1	0.00039651	Q9UPZ6	THSD7A
1	THSD7B	1.5551E-26	-9.08	9.0467E-22	1.3406E-25	Q9C0I4	THSD7B
1	THTPA	1.2493E-14	1.69	7.2675E-10	8.3324E-14	Q9BU02	THTPA
1	THUMPD1	2.003E-104	2.17	1.165E-99	3.549E-103	Q9NXG2	THUMPD1
1	TIAF1	0.00035353	-1.74	1	0.0014301	O95411	TIAF1
1	TIAL1	0	-1.73	0	0	Q01085	TIAL1
1	TIAM1	2.551E-173	20.32	1.484E-168	5.898E-172	Q13009	TIAM1
1	TICAM1	0	-1.57	0	0	Q8IUC6	TICAM1
1	TICAM2	7.293E-05	-4.79	1	0.00031084	Q86XR7	TICAM2
1	TIGAR	9.571E-117	2.96	5.568E-112	1.821E-115	Q9NQ88	TIGAR
1	TIGD3	3.3798E-10	2.99	1.9661E-05	1.9204E-09	Q6B0B8	TIGD3
1	TIGIT	7.4121E-11	-10.48	4.3119E-06	4.3288E-10	Q495A1	TIGIT
1	TIMM10	2.6928E-24	1.75	1.5665E-19	2.228E-23	P62072	TIMM10
1	TIMM10B	1.598E-111	2.47	9.298E-107	2.959E-110	Q9Y5J6	TIMM10B
1	TIMM22	4.7801E-56	2.17	2.7808E-51	5.919E-55	Q9Y584	TIMM22
1	TIMM23	6.224E-136	2.18	3.621E-131	1.283E-134	O14925	TIMM23
1	TIMM23B	1.1582E-28	1.84	6.7377E-24	1.042E-27	Q5SRD1	TIMM23B
1	TIMM29	2.2186E-31	1.73	1.2906E-26	2.0823E-30	Q9BSF4	TIMM29
1	TIMM44	1.9845E-59	1.71	1.1545E-54	2.5196E-58	O43615	TIMM44
1	TIMM50	7.3841E-34	1.50	4.2957E-29	7.1918E-33	Q3ZCQ8	TIMM50
1	TIMM8A	2.0668E-62	2.20	1.2023E-57	2.6832E-61	O60220	TIMM8A
1	TIMM9	6.9901E-34	2.09	4.0664E-29	6.8091E-33	Q9Y5J7	TIMM9
1	TIMP1	7.5571E-77	5.71	4.3963E-72	1.0914E-75	P01033	TIMP1
1	TIMP2	1.735E-24	1.51	1.0093E-19	1.4429E-23	P16035	TIMP2
1	TIMP3	7.3313E-08	-4.08	0.00426492	3.7366E-07	P35625	TIMP3
1	TIMP4	1.6154E-45	-10.31	9.3974E-41	1.8093E-44	Q99727	TIMP4
1	TINAG	0.00787162	17.61	1	0.02799728	Q9UJW2	TINAG
1	TINAGL1	0	-6.98	0	0	Q9GZM7	TINAGL1
1	TINF2	7.4596E-28	1.68	4.3395E-23	6.6151E-27	Q9BSI4	TINF2
1	TIPARP	0	-2.61	0	0	Q7Z3E1	TIPARP
1	TIPARP-AS1	3.8264E-12	-3.60	2.226E-07	2.3513E-11		
1	TIPIN	5.0533E-15	1.56	2.9397E-10	3.4099E-14	Q9BVW5	TIPIN
1	TJP1	0	-2.25	0	0	Q07157	TJP1
1	TJP3	9.5388E-35	-7.24	5.5491E-30	9.3925E-34	O95049	TJP3
1	TKFC	0	-3.21	0	0	Q3LXA3	TKFC
1	TKT	0	-1.93	0	0	P29401	TKT
1	TLCD1	2.5979E-14	-1.52	1.5113E-09	1.7176E-13	Q96CP7	TLCD1
1	TLCD2	1.0534E-15	-1.79	6.1279E-11	7.2692E-15	A6NGC4	TLCD2
1	TLDC1	0	-2.47	0	0	Q6P9B6	TLDC1
1	TLE1	9.7952E-92	2.14	5.6982E-87	1.5921E-90	Q04724	TLE1
1	TLE2	3.781E-199	53.10	2.199E-194	9.5E-198	Q04725	TLE2
1	TLE3	0	-1.76	0	0	Q04726	TLE3
1	TLE4	1.5338E-69	5.42	8.9224E-65	2.094E-68	Q04727	TLE4
1	TLE6	3.32E-149	35.25	1.931E-144	7.134E-148	Q9H808	TLE6
1	TLL1	7.7362E-08	-1.80	0.00450044	3.9395E-07	O43897	TLL1
1	TLN1	1.943E-88	2.03	1.1303E-83	3.0833E-87	Q9Y490	TLN1
1	TLR1	1.743E-150	-8.33	1.014E-145	3.755E-149	Q15399	TLR1

1	TLR10	0.00245685	-10.55	1	0.00920965	Q9BXR5	TLR10
1	TLR2	5.3E-195	-13.86	3.083E-190	1.316E-193	O60603	TLR2
1	TLR3	2.472E-209	-17.95	1.438E-204	6.378E-208	O15455	TLR3
1	TLR4	0	125.91	0	0	O00206	TLR4
1	TLR5	8.9508E-06	-4.73	0.52070469	4.0574E-05	O60602	TLR5
1	TLR6	1.2917E-74	-5.51	7.5145E-70	1.8337E-73	Q9Y2C9	TLR6
1	TM2D1	0	-1.67	0	0	Q9BX74	TM2D1
1	TM4SF1	0	-2.61	0	0	P30408	TM4SF1
1	TM4SF1-AS1	6.842E-112	-26.40	3.981E-107	1.269E-110		
1	TM4SF19	4.1015E-10	-1.98	2.386E-05	2.3219E-09	Q96DZ7	TM4SF19
1	TM4SF19-AS1	3.4201E-06	-2.35	0.1989603	1.5879E-05		
1	TM4SF19-TCT	3.9757E-08	20.63	0.00231282	2.0564E-07		
1	TMBIM1	0	-1.86	0	0	Q969X1	TMBIM1
1	TMBIM4	0	-1.67	0	0	Q9HC24	TMBIM4
1	TMBIM6	0	-2.84	0	0	P55061	TMBIM6
1	TMBIM7P	0.00010157	-32.64	1	0.00042869		
1	TMC1	3.323E-152	-31.82	1.933E-147	7.191E-151	Q8TDI8	TMC1
1	TMC2	2.465E-06	-3.43	0.14339755	1.1562E-05	Q8TDI7	TMC2
1	TMC3	0.00011583	-11.87	1	0.00048697	Q7Z5M5	TMC3
1	TMC3-AS1	2.3718E-39	-13.75	1.3797E-34	2.4638E-38		
1	TMC4	0	-9.89	0	0	Q7Z404	TMC4
1	TMC5	2.2335E-79	-10.54	1.2993E-74	3.2952E-78	Q6UXY8	TMC5
1	TMC7	8.716E-78	-3.28	5.0704E-73	1.2667E-76	Q7Z402	TMC7
1	TMC8	7.0028E-07	-3.03	0.04073808	3.3914E-06	Q8IU68	TMC8
1	TMCC1	0	-1.57	0	0	O94876	TMCC1
1	TMCC2	9.2807E-11	-1.60	5.3989E-06	5.4011E-10	O75069	TMCC2
1	TMCC3	6.3403E-14	1.73	3.6884E-09	4.1387E-13	Q9ULS5	TMCC3
1	TMCO1	0	-1.66	0	0	Q9UM00	TMCO1
1	TMCO4	0	-2.90	0	0	Q5TGY1	TMCO4
1	TMED10P2	2.0015E-05	-14.33	1	8.8616E-05		
1	TMED4	0	-1.81	0	0	Q7Z7H5	TMED4
1	TMED7-TICAM	1.2412E-11	4.38	7.2207E-07	7.4934E-11	A0A0A6YYA0	TMED7-TICAM
1	TMED8	6.8195E-86	2.66	3.9672E-81	1.0607E-84	Q6PL24	TMED8
1	TMEFF1	9.0792E-06	7.22	0.52817053	4.1128E-05	Q8IYR6	TMEFF1
1	TMEFF2	1.6318E-06	-2.55	0.09493005	7.7412E-06	Q9UIK5	TMEFF2
1	TMEM104	6.4387E-28	1.58	3.7456E-23	5.7229E-27	Q8NE00	TMEM104
1	TMEM105	1.1353E-10	-5.01	6.6043E-06	6.5853E-10	Q8N8V8	TMEM105
1	TMEM106A	3.4453E-43	6.95	2.0043E-38	3.7646E-42	Q96A25	TMEM106A
1	TMEM106C	2.552E-41	1.74	1.4846E-36	2.7165E-40	Q9BVX2	TMEM106C
1	TMEM108	1.9078E-06	4.38	0.11098599	9.0145E-06	Q6UXF1	TMEM108
1	TMEM108-AS	2.5147E-75	643.19	1.4629E-70	3.5926E-74		
1	TMEM117	1.014E-105	-4.73	5.9E-101	1.808E-104	Q9H0C3	TMEM117
1	TMEM121	1.0443E-59	23.25	6.0749E-55	1.3287E-58	Q9BTD3	TMEM121
1	TMEM125	2.2775E-94	-26.92	1.3249E-89	3.7747E-93	Q96AQ2	TMEM125
1	TMEM126B	0	-1.51	0	0	Q8IUX1	TMEM126B
1	TMEM129	0	-1.96	0	0	A0AVI4	TMEM129
1	TMEM130	0.00041633	-10.67	1	0.00167379	Q8N3G9	TMEM130

1	TMEM132A	0	-2.60	0	0	Q24JP5	TMEM132A
1	TMEM132B	2.489E-05	-3.45	1	0.0001095	Q14DG7	TMEM132B
1	TMEM134	0	-2.51	0	0	Q9H6X4	TMEM134
1	TMEM135	0	-1.82	0	0	Q86UB9	TMEM135
1	TMEM136	2.2288E-56	13.02	1.2966E-51	2.7651E-55	Q6ZRR5	TMEM136
1	TMEM138	0	-2.32	0	0	Q9NPI0	TMEM138
1	TMEM143	0	-2.13	0	0	Q96AN5	TMEM143
1	TMEM144	1.1147E-88	-4.55	6.4847E-84	1.7727E-87	Q7Z5S9	TMEM144
1	TMEM145	4.4564E-30	4.80	2.5925E-25	4.1007E-29	Q8NBT3	TMEM145
1	TMEM14B	2.3367E-29	1.60	1.3594E-24	2.1247E-28	Q9NUH8	TMEM14B
1	TMEM150A	2.0361E-30	2.13	1.1845E-25	1.8834E-29	Q86TG1	TMEM150A
1	TMEM150C	0.01303138	-1.97	1	0.04509742	B9EJG8	TMEM150C
1	TMEM151A	0.00932946	17.55	1	0.03288088	Q8N4L1	TMEM151A
1	TMEM154	5.5498E-42	1.73	3.2285E-37	5.9677E-41	Q6P9G4	TMEM154
1	TMEM156	2.123E-196	16.14	1.235E-191	5.291E-195	Q8N614	TMEM156
1	TMEM158	4.4026E-66	5.63	2.5612E-61	5.8783E-65	Q8WZ71	TMEM158
1	TMEM159	0	-2.46	0	0	Q96B96	TMEM159
1	TMEM161A	1.6526E-54	1.82	9.614E-50	2.0214E-53	Q9NX61	TMEM161A
1	TMEM161B-A	0	-2.41	0	0		
1	TMEM163	4.9659E-20	-5.08	2.8888E-15	3.7916E-19	Q8TC26	TMEM163
1	TMEM167B	2.9721E-18	1.52	1.729E-13	2.1878E-17	Q9NRX6	TMEM167B
1	TMEM168	0	-4.44	0	0	Q9H0V1	TMEM168
1	TMEM169	0.00128035	2.03	1	0.00494149	Q96HH4	TMEM169
1	TMEM17	6.8436E-16	-2.23	3.9812E-11	4.7548E-15	Q86X19	TMEM17
1	TMEM170B	4.538E-143	19.84	2.64E-138	9.622E-142	Q5T4T1	TMEM170B
1	TMEM173	0	-1.85	0	0	Q86WV6	TMEM173
1	TMEM175	0	-1.72	0	0	Q9BSA9	TMEM175
1	TMEM178A	0.00030034	-2.66	1	0.00122097	Q8NBL3	TMEM178A
1	TMEM178B	5.4447E-23	-6.77	3.1674E-18	4.3979E-22	H3BS89	TMEM178B
1	TMEM184A	3.717E-277	-9.55	2.162E-272	1.035E-275	Q6ZMB5	TMEM184A
1	TMEM187	0	-2.15	0	0	Q14656	TMEM187
1	TMEM189	0	-1.76	0	0	A5PLL7	TMEM189
1	TMEM19	0	-1.54	0	0	Q96HH6	TMEM19
1	TMEM191A	1.7203E-06	1.70	0.10007832	8.1497E-06	Q9H0A3	TMEM191A
1	TMEM191B	2.6702E-36	21.42	1.5534E-31	2.6764E-35	P0C7N4	TMEM191B
1	TMEM191C	2.7545E-12	4.06	1.6024E-07	1.7027E-11	A6NGB0	TMEM191C
1	TMEM192	0	-1.68	0	0	Q8IY95	TMEM192
1	TMEM2	2.4996E-60	3.44	1.4541E-55	3.1924E-59	Q9UHN6	TMEM2
1	TMEM201	1.7334E-17	1.59	1.0084E-12	1.2566E-16	Q5SNT2	TMEM201
1	TMEM205	5.6833E-23	1.67	3.3062E-18	4.5888E-22	Q6UW68	TMEM205
1	TMEM216	5.81E-115	-3.87	3.38E-110	1.093E-113	Q9P0N5	TMEM216
1	TMEM217	8.7805E-15	-3.06	5.108E-10	5.8881E-14	Q8N7C4	TMEM217
1	TMEM223	0	-2.18	0	0	A0PJW6	TMEM223
1	TMEM229B	0.00086872	-1.90	1	0.00340131	Q8NBD8	TMEM229B
1	TMEM236	1.0838E-08	8.04	0.0006305	5.7638E-08	Q5W0B7	TMEM236
1	TMEM238	9.1526E-23	-3.12	5.3244E-18	7.3654E-22	C9JI98	TMEM238
1	TMEM240	2.8532E-08	2.81	0.0016598	1.4874E-07	Q5SV17	TMEM240

1	TMEM243	0	-1.94	0	0	Q9BU79	TMEM243
1	TMEM246	0	-5.56	0	0	Q9BRR3	TMEM246
1	TMEM246-AS	4.9286E-34	-8.12	2.8672E-29	4.8139E-33		
1	TMEM249	3.0953E-19	5.19	1.8007E-14	2.3243E-18	Q2WGJ8	TMEM249
1	TMEM25	2.6767E-46	-2.75	1.5571E-41	3.0212E-45	Q86YD3	TMEM25
1	TMEM251	1.7643E-26	1.94	1.0263E-21	1.5203E-25	Q8N6I4	TMEM251
1	TMEM254	1.4122E-38	1.72	8.2156E-34	1.4533E-37	Q8TBM7	TMEM254
1	TMEM254-AS	9.4284E-20	-4.50	5.4849E-15	7.1595E-19		
1	TMEM255A	1.8517E-44	-7.21	1.0772E-39	2.0483E-43	Q5JRV8	TMEM255A
1	TMEM255B	9.7064E-26	2.67	5.6466E-21	8.2517E-25	Q8WV15	TMEM255B
1	TMEM258	0	-1.91	0	0	P61165	TMEM258
1	TMEM263	2.0938E-76	2.58	1.218E-71	3.0149E-75	Q8WUH6	TMEM263
1	TMEM265	2.2847E-25	-1543.81	1.3291E-20	1.9307E-24	A0A087WTH1	TMEM265
1	TMEM266	0.00023267	-1.74	1	0.00095563	Q2M3C6	TMEM266
1	TMEM267	0	-2.22	0	0	Q0VDI3	TMEM267
1	TMEM268	3.3709E-24	1.70	1.961E-19	2.7851E-23	Q5VZI3	TMEM268
1	TMEM269	7.2469E-14	-4.88	4.2158E-09	4.7231E-13	A0A1B0GVZ9	TMEM269
1	TMEM27	1.0444E-12	-7.25	6.0757E-08	6.5457E-12	Q9HBJ8	TMEM27
1	TMEM30B	5.562E-303	-9.51	3.236E-298	1.563E-301	Q3MIR4	TMEM30B
1	TMEM33	0	-1.73	0	0	P57088	TMEM33
1	TMEM35B	0	-1.95	0	0	Q8NCS4	TMEM35B
1	TMEM37	9.4968E-06	-2.83	0.55246885	4.2964E-05	Q8WXS4	TMEM37
1	TMEM38A	3.8555E-12	1.93	2.2429E-07	2.3684E-11	Q9H6F2	TMEM38A
1	TMEM40	2.95E-190	-10.31	1.716E-185	7.189E-189	Q8WWA1	TMEM40
1	TMEM43	1.1392E-46	1.53	6.627E-42	1.2906E-45	Q9BTV4	TMEM43
1	TMEM44	0	-1.85	0	0	Q2T9K0	TMEM44
1	TMEM44-AS1	1.7216E-65	-2.95	1.0015E-60	2.2912E-64		
1	TMEM45A	1.45E-156	-4.84	8.435E-152	3.189E-155	Q9NWC5	TMEM45A
1	TMEM45B	0.0006374	-4.73	1	0.0025256	Q96B21	TMEM45B
1	TMEM47	6.9223E-27	-12.97	4.027E-22	6.0024E-26	Q9BQJ4	TMEM47
1	TMEM51	0	-4.08	0	0	Q9NW97	TMEM51
1	TMEM51-AS1	5.966E-116	-16.11	3.47E-111	1.129E-114		
1	TMEM52	9.4552E-16	2.67	5.5004E-11	6.5357E-15	Q8NDY8	TMEM52
1	TMEM52B	1.7305E-77	-83.78	1.0067E-72	2.5099E-76	Q4KMG9	TMEM52B
1	TMEM54	0	-2.28	0	0	Q969K7	TMEM54
1	TMEM55A	6.2789E-13	-1.62	3.6527E-08	3.9656E-12	Q8N4L2	TMEM55A
1	TMEM56	4.999E-141	-9.46	2.908E-136	1.05E-139	Q96MV1	TMEM56
1	TMEM56-RWI	0.00262778	-1.58	1	0.00982697	S4R434	TMEM56-RWI
1	TMEM57	7.6562E-45	1.65	4.4539E-40	8.5047E-44	Q8N5G2	TMEM57
1	TMEM59	0	-1.52	0	0	Q9BXS4	TMEM59
1	TMEM59L	3.0804E-24	-6.25	1.792E-19	2.5469E-23	Q9UK28	TMEM59L
1	TMEM60	0	-1.76	0	0	Q9H2L4	TMEM60
1	TMEM61	2.9669E-10	-12.36	1.726E-05	1.69E-09	Q8N0U2	TMEM61
1	TMEM63A	0	-3.37	0	0	O94886	TMEM63A
1	TMEM63B	0	-3.80	0	0	Q5T3F8	TMEM63B
1	TMEM63C	5.7902E-05	-2.34	1	0.00024863	Q9P1W3	TMEM63C
1	TMEM67	0	-1.61	0	0	Q5HYA8	TMEM67

1	TMEM68	0	-2.53	0	0	Q96MH6	TMEM68
1	TMEM69	0	-1.71	0	0	Q5SWH9	TMEM69
1	TMEM70	1.8204E-34	1.87	1.059E-29	1.7862E-33	Q9BUB7	TMEM70
1	TMEM72-AS1	3.2462E-06	2.18	0.18884458	1.5103E-05		
1	TMEM74B	0.00711431	2.13	1	0.02541874	Q9NUR3	TMEM74B
1	TMEM79	0	-2.07	0	0	Q9BSE2	TMEM79
1	TMEM80	0	-1.73	0	0	Q96HE8	TMEM80
1	TMEM81	0.00033599	-1.67	1	0.00136105	Q6P7N7	TMEM81
1	TMEM86A	0.01150365	-2.09	1	0.04008704	Q8N2M4	TMEM86A
1	TMEM86B	1.6889E-13	1.79	9.8251E-09	1.0873E-12	Q8N661	TMEM86B
1	TMEM87A	0	-2.01	0	0	Q8NBN3	TMEM87A
1	TMEM88B	2.2103E-06	-14.66	0.1285845	1.0403E-05	A6NKF7	TMEM88B
1	TMEM89	0.00878096	3.78	1	0.03103802	A2RUT3	TMEM89
1	TMEM92	5.1699E-16	-7.03	3.0076E-11	3.6096E-15	Q6UXU6	TMEM92
1	TMEM97	2.754E-101	2.11	1.602E-96	4.779E-100	Q5BJF2	TMEM97
1	TMEM98	2.354E-163	4.36	1.37E-158	5.275E-162	Q9Y2Y6	TMEM98
1	TMEM9B-AS1	2.0455E-06	1.50	0.11899426	9.6516E-06		
1	TMIE	2.4962E-17	-14.75	1.4522E-12	1.8037E-16	Q8NEW7	TMIE
1	TMOD1	0.00067517	-2.51	1	0.00266795	P28289	TMOD1
1	TMOD2	3.315E-178	8.24	1.928E-173	7.784E-177	Q9NZR1	TMOD2
1	TMPO	8.75E-53	1.64	5.0902E-48	1.0526E-51	P42166	TMPO
1	TMPOP1	0.00862026	17.60	1	0.03050054		
1	TMPPE	1.102E-05	1.82	0.64106813	4.9634E-05	Q6ZT21	TMPPE
1	TMPRSS11A	6.6436E-07	-6.98	0.03864853	3.2202E-06	Q6ZMR5	TMPRSS11A
1	TMPRSS11F	0.000409	-5.84	1	0.00164499	Q6ZWK6	TMPRSS11F
1	TMPRSS13	2.5845E-08	-10.23	0.00150353	1.3509E-07	Q9BYE2	TMPRSS13
1	TMPRSS15	1.9669E-17	-13.41	1.1442E-12	1.4239E-16	P98073	TMPRSS15
1	TMPRSS3	1.1673E-08	-59.07	0.00067909	6.2017E-08	P57727	TMPRSS3
1	TMPRSS4	0	-6.54	0	0	Q9NRS4	TMPRSS4
1	TMPRSS4-AS1	0.00018704	-13.09	1	0.00077428	A0A1B0GVV1	TMPRSS4-AS1
1	TMPRSS5	9.8343E-11	2.67	5.721E-06	5.7153E-10	Q9H3S3	TMPRSS5
1	TMPRSS6	0.00043963	2.15	1	0.00176402	Q8IU80	TMPRSS6
1	TMPRSS9	1.1522E-16	-3.38	6.7027E-12	8.198E-16	Q7Z410	TMPRSS9
1	TMSB10	8.9893E-42	1.97	5.2294E-37	9.6359E-41	P63313	TMSB10
1	TMSB15B	1.134E-220	41.90	6.595E-216	2.998E-219	P0CG35, A0A(	TMSB15B
1	TMTC1	6.746E-08	3.86	0.00392444	3.4455E-07	Q8IUR5	TMTC1
1	TMTC2	9.4628E-85	-2.69	5.5049E-80	1.461E-83	Q8N394	TMTC2
1	TMUB1	1.9541E-17	1.55	1.1368E-12	1.4148E-16	Q9BVT8	TMUB1
1	TMX3	6.6172E-37	1.53	3.8495E-32	6.6727E-36	Q96JJ7	TMX3
1	TNC	0	-3.29	0	0	P24821	TNC
1	TNF	0.00435565	-5.64	1	0.01595425	P01375	TNF
1	TNFAIP2	0	-5.17	0	0	Q03169	TNFAIP2
1	TNFAIP3	0	-1.57	0	0	P21580	TNFAIP3
1	TNFAIP8	1.3048E-14	1.56	7.5903E-10	8.6955E-14	O95379	TNFAIP8
1	TNFAIP8L1	0	-1.50	0	0	Q8WVP5	TNFAIP8L1
1	TNFAIP8L3	0.01110796	-2.49	1	0.03877089	Q5GJ75	TNFAIP8L3
1	TNFRSF10B	0	-2.44	0	0	O14763	TNFRSF10B

1	TNFRSF10C	1.6407E-40	3.62	9.5446E-36	1.7266E-39	O14798	TNFRSF10C
1	TNFRSF11A	3.3833E-25	2.02	1.9682E-20	2.8504E-24	Q9Y6Q6	TNFRSF11A
1	TNFRSF11B	1.0296E-10	-6.97	5.9897E-06	5.9802E-10	O00300	TNFRSF11B
1	TNFRSF12A	3.0897E-65	2.17	1.7974E-60	4.0999E-64	Q9NP84	TNFRSF12A
1	TNFRSF13C	2.1267E-08	1.74	0.0012372	1.1163E-07	Q96RJ3	TNFRSF13C
1	TNFRSF14	2.535E-161	-3.92	1.475E-156	5.653E-160	Q92956	TNFRSF14
1	TNFRSF18	1.1424E-19	-5.94	6.6456E-15	8.6554E-19	Q9Y5U5	TNFRSF18
1	TNFRSF19	2.5651E-39	11.76	1.4922E-34	2.6623E-38	Q9NS68	TNFRSF19
1	TNFRSF21	0	-13.24	0	0	O75509	TNFRSF21
1	TNFRSF25	3.2749E-08	-1.57	0.00190515	1.7015E-07	Q93038	TNFRSF25
1	TNFSF10	6.255E-101	-7.72	3.6389E-96	1.081E-99	P50591	TNFSF10
1	TNFSF12	7.2252E-24	2.38	4.2032E-19	5.9325E-23	O43508	TNFSF12
1	TNFSF12-TNFS	0.00132852	2.71	1	0.00511959	A0A0A6YY99	TNFSF12-TNFS
1	TNFSF13	1.122E-125	-3.66	6.53E-121	2.226E-124	O75888	TNFSF13
1	TNFSF15	8.218E-108	-13.24	4.781E-103	1.482E-106	O95150	TNFSF15
1	TNFSF18	0.00010369	8.98	1	0.00043732	Q9UNG2	TNFSF18
1	TNFSF4	0.00778798	-2.62	1	0.02772524	P23510	TNFSF4
1	TNFSF8	0.00036348	4.34	1	0.00146831	P32971	TNFSF8
1	TNIP1	0	-1.88	0	0	Q15025	TNIP1
1	TNK1	4.3279E-88	-2.77	2.5177E-83	6.8528E-87	Q13470	TNK1
1	TNK2	0	-2.90	0	0	Q07912	TNK2
1	TNKS	0	-1.94	0	0	O95271	TNKS
1	TNKS1BP1	0	-2.98	0	0	Q9C0C2	TNKS1BP1
1	TNNC1	3.7626E-05	-2.22	1	0.00016367	P63316	TNNC1
1	TNNI1	7.911E-137	-12.72	4.602E-132	1.637E-135	P19237	TNNI1
1	TNNI2	9.361E-190	-12.92	5.446E-185	2.277E-188	P48788	TNNI2
1	TNNI3K	0.00606731	-3.57	1	0.02184967	Q59H18	TNNI3K
1	TNNT1	1.271E-132	10.79	7.395E-128	2.586E-131	P13805	TNNT1
1	TNNT2	3.9299E-17	-13.01	2.2862E-12	2.8245E-16	P45379	TNNT2
1	TNNT3	1.6307E-61	-25.71	9.4867E-57	2.1025E-60	P45378	TNNT3
1	TNPO1	2.0288E-30	1.64	1.1802E-25	1.8769E-29	Q92973	TNPO1
1	TNRC18	0	-1.86	0	0	O15417	TNRC18
1	TNRC6A	1.1484E-28	1.58	6.6806E-24	1.0333E-27	Q8NDV7	TNRC6A
1	TNRC6C	3.6285E-13	1.60	2.1109E-08	2.3087E-12	Q9HCJ0	TNRC6C
1	TNS1	4.2596E-12	-3.94	2.478E-07	2.6131E-11	Q9HBL0	TNS1
1	TNS2	5.7783E-55	2.11	3.3615E-50	7.0917E-54	Q63HR2	TNS2
1	TNS3	0	-4.81	0	0	Q68CZ2	TNS3
1	TNS4	0	-10.66	0	0	Q8IZW8	TNS4
1	TNXB	8.3219E-05	-3.17	1	0.00035324	P22105	TNXB
1	TOB1	0	-2.07	0	0	P50616	TOB1
1	TOB1-AS1	2.7229E-09	-3.06	0.0001584	1.4892E-08		
1	TOE1	2.8822E-96	2.53	1.6767E-91	4.8319E-95	Q96GM8	TOE1
1	TOGARAM1	6.9824E-28	1.65	4.0619E-23	6.2033E-27	Q9Y4F4	TOGARAM1
1	TOGARAM2	2.7292E-17	-4.84	1.5877E-12	1.9691E-16		
1	TOLLIP	4.0071E-97	2.18	2.3311E-92	6.7529E-96	Q9H0E2	TOLLIP
1	TOLLIP-AS1	4.1408E-11	-3.20	2.4089E-06	2.4411E-10		
1	TOM1L2	0	-3.47	0	0	Q6ZVM7	TOM1L2

1	TOMM20	0	-2.32	0	0	Q15388	TOMM20
1	TOMM22	1.7367E-38	1.90	1.0103E-33	1.7859E-37	Q9NS69	TOMM22
1	TOMM34	5.3389E-76	2.05	3.1059E-71	7.665E-75	Q15785	TOMM34
1	TOMM40	2.7262E-33	1.53	1.586E-28	2.6306E-32	O96008	TOMM40
1	TOMM5	2.2365E-57	2.28	1.3011E-52	2.7932E-56	Q8N4H5	TOMM5
1	TOMM6	2.0292E-17	1.60	1.1805E-12	1.4686E-16	Q96B49	TOMM6
1	TOP1	2.0799E-28	1.83	1.2099E-23	1.8623E-27	P11387	TOP1
1	TOP3A	2.9574E-73	2.09	1.7204E-68	4.1507E-72	Q13472	TOP3A
1	TOPORS	7.3024E-62	1.77	4.2481E-57	9.4381E-61	Q9NS56	TOPORS
1	TOPORS-AS1	1.0832E-15	1.81	6.3017E-11	7.4727E-15		
1	TOR1AIP1	0	-1.70	0	0	Q5JTV8	TOR1AIP1
1	TOR1AIP2	0	-1.67	0	0	Q8NFQ8	TOR1AIP2
1	TOR2A	6.6613E-16	-1.50	3.8752E-11	4.6304E-15	Q5JU69	TOR2A
1	TOR4A	1.3021E-29	1.60	7.5751E-25	1.1896E-28	Q9NXH8	TOR4A
1	TOX	4.2683E-05	3.01	1	0.00018489	O94900	TOX
1	TOX2	8.83E-89	25.86	5.1368E-84	1.4054E-87	Q96NM4	TOX2
1	TOX4	3.3116E-41	1.66	1.9265E-36	3.5206E-40	O94842	TOX4
1	TP53	0	-3.07	0	0	P04637	TP53
1	TP53AIP1	2.747E-232	-7.87	1.598E-227	7.375E-231	Q9HCN2	TP53AIP1
1	TP53I13	0	-1.78	0	0	Q8NBR0	TP53I13
1	TP53I3	7.9396E-44	2.03	4.6188E-39	8.746E-43	Q53FA7	TP53I3
1	TP53INP1	2.41E-128	-3.80	1.402E-123	4.836E-127	Q96A56	TP53INP1
1	TP53RK	1.1367E-77	2.31	6.6129E-73	1.6512E-76	Q96S44	TP53RK
1	TP53TG1	1.016E-105	-3.80	5.912E-101	1.811E-104	Q9Y2A0	TP53TG1
1	TP63	0	-7.34	0	0	Q9H3D4	TP63
1	TP73	2.0457E-74	14.30	1.1901E-69	2.899E-73	O15350	TP73
1	TP73-AS1_1	4.564E-190	134.80	2.655E-185	1.111E-188		
1	TPCN2	0	-2.28	0	0	Q8NHX9	TPCN2
1	TPD52L1	0	-9.53	0	0	Q16890	TPD52L1
1	TPD52L2	5.4068E-08	1.94	0.00314534	2.7756E-07	O43399	TPD52L2
1	TPGS1	1.1232E-05	1.59	0.6534223	5.0563E-05	Q6ZTW0	TPGS1
1	TPI1	1.3023E-49	2.04	7.5758E-45	1.5191E-48	P60174	TPI1
1	TPI1P2	0.00988323	-1.53	1	0.03467713		
1	TPK1	2.6603E-45	4.32	1.5476E-40	2.971E-44	Q9H3S4	TPK1
1	TPM1	6.1743E-59	3.13	3.5919E-54	7.8135E-58	P09493	TPM1
1	TPM2	1.6351E-33	2.93	9.5121E-29	1.5838E-32	P07951	TPM2
1	TPM3	8.1139E-32	1.61	4.7202E-27	7.6651E-31	P06753	TPM3
1	TPM3P9	2.7016E-09	1.68	0.00015716	1.478E-08		
1	TPO	2.9708E-58	169.57	1.7282E-53	3.7383E-57	P07202	TPO
1	TPPP	2.2204E-16	-2.03	1.2917E-11	1.5667E-15	O94811	TPPP
1	TPRG1	6.243E-137	-8.65	3.632E-132	1.293E-135	Q6ZUI0	TPRG1
1	TPRG1-AS1	5.1832E-07	-3.27	0.03015257	2.5281E-06		
1	TPRKB	4.1985E-18	1.58	2.4425E-13	3.082E-17	Q9Y3C4	TPRKB
1	TPRN	2.0763E-84	2.64	1.2079E-79	3.1972E-83	Q4KMQ1	TPRN
1	TPRXL	0.0039599	-19.71	1	0.0145698		
1	TPSP2	0.00216836	-21.57	1	0.00817567		
1	TPT1	5.0465E-16	1.61	2.9358E-11	3.5247E-15	P13693	TPT1

1	TPTE	1.186E-141	-11.22	6.901E-137	2.5E-140	P56180	TPTE
1	TPTE2	1.4593E-09	-5.57	8.4892E-05	8.0703E-09	Q6XPS3	TPTE2
1	TPTE2P1	2.318E-15	-11.47	1.3485E-10	1.5838E-14		
1	TPTE2P6	7.7913E-05	-2.80	1	0.00033166		
1	TPTEP1	3.8599E-05	-4.98	1	0.00016773		
1	TRABD2A	2.1706E-31	-3.41	1.2627E-26	2.0376E-30	Q86V40	TRABD2A
1	TRADD	0	-1.77	0	0	Q15628	TRADD
1	TRAF1	3.3124E-27	-2.49	1.927E-22	2.8977E-26	Q13077	TRAF1
1	TRAF3	3.9276E-41	1.56	2.2849E-36	4.171E-40	Q13114	TRAF3
1	TRAF3IP1	1.5853E-24	1.52	9.2221E-20	1.3199E-23	Q8TDR0	TRAF3IP1
1	TRAF3IP2	0	-1.81	0	0	O43734	TRAF3IP2
1	TRAF3IP3	4.7638E-18	-10.69	2.7713E-13	3.4952E-17	Q9Y228	TRAF3IP3
1	TRAF5	1.9367E-91	3.43	1.1267E-86	3.1419E-90	O00463	TRAF5
1	TRAFD1	0	-1.57	0	0	O14545	TRAFD1
1	TRAK2	2.0807E-84	2.19	1.2105E-79	3.2031E-83	O60296	TRAK2
1	TRAM1L1	4.3082E-46	-4.40	2.5063E-41	4.8543E-45	Q8N609	TRAM1L1
1	TRANK1	4.141E-163	-8.23	2.409E-158	9.274E-162		
1	TRAPPC11	0	-3.00	0	0	Q7Z392	TRAPPC11
1	TRAPPC12-AS	0.00721219	-2.02	1	0.02575738		
1	TRAPPC2	9.7284E-29	-1.81	5.6594E-24	8.7674E-28	P0DI81	TRAPPC2
1	TRAPPC2L	2.3903E-30	1.69	1.3905E-25	2.209E-29	H3BP13, Q9U	TRAPPC2L
1	TRAPPC4	2.123E-33	1.79	1.235E-28	2.0526E-32	Q9Y296	TRAPPC4
1	TRAPPC5	3.9815E-32	2.51	2.3162E-27	3.7779E-31	Q8IUR0	TRAPPC5
1	TRAPPC6A	1.279E-118	-5.29	7.438E-114	2.46E-117	O75865	TRAPPC6A
1	TRBC2	1.8878E-06	52.33	0.10982313	8.9244E-06	A0A5B9	TRBC2
1	TRBV12-4	0.00054827	25.89	1	0.00218506		
1	TRDMT1	4.5867E-72	3.60	2.6683E-67	6.3834E-71	O14717	TRDMT1
1	TRDN	1.859E-19	-7.74	1.0815E-14	1.4018E-18	Q13061	TRDN
1	TREH	3.2733E-09	-6.04	0.00019042	1.7842E-08	O43280	TREH
1	TRERF1	0	-2.41	0	0	Q96PN7	TRERF1
1	TREX2	0.00402596	-1.64	1	0.01480444	Q9BQ50	TREX2
1	TRHDE	4.3616E-08	-3.97	0.00253733	2.2511E-07	Q9UKU6	TRHDE
1	TRHDE-AS1	6.9405E-08	-4.03	0.00403757	3.5424E-07		
1	TRIAP1	4.3032E-28	1.93	2.5033E-23	3.8377E-27	O43715	TRIAP1
1	TRIB1	6.1659E-27	3.30	3.587E-22	5.3553E-26	Q96RU8	TRIB1
1	TRIB2	2.195E-195	30.50	1.277E-190	5.459E-194	Q92519	TRIB2
1	TRIB3	3.625E-108	3.22	2.109E-103	6.555E-107	Q96RU7	TRIB3
1	TRIM11	0	-1.50	0	0	Q96F44	TRIM11
1	TRIM15	0.01438331	-4.02	1	0.04958135	Q9C019	TRIM15
1	TRIM16	0	-4.74	0	0	O95361	TRIM16
1	TRIM16L	0	-3.70	0	0	Q309B1	TRIM16L
1	TRIM17	9.3987E-36	-4.47	5.4676E-31	9.3543E-35	Q9Y577	TRIM17
1	TRIM22	9.615E-19	-20.58	5.5934E-14	7.15E-18	Q8IYM9	TRIM22
1	TRIM23	7.8499E-35	1.92	4.5666E-30	7.7361E-34	P36406	TRIM23
1	TRIM24	1.1573E-29	1.57	6.7327E-25	1.0584E-28	O15164	TRIM24
1	TRIM27	7.447E-110	1.86	4.332E-105	1.361E-108	P14373	TRIM27
1	TRIM29	0	-8.90	0	0	Q14134	TRIM29

1	TRIM3	7.1893E-84	4.17	4.1823E-79	1.1012E-82	O75382	TRIM3
1	TRIM31	1.3016E-17	-25.97	7.5721E-13	9.4592E-17	Q9BZY9	TRIM31
1	TRIM31-AS1	0.00469726	-5.83	1	0.01714079		
1	TRIM34	1.337E-152	-38.57	7.781E-148	2.898E-151	Q9BYJ4	TRIM34
1	TRIM36	1.8647E-79	2.73	1.0848E-74	2.7532E-78	Q9NQ86	TRIM36
1	TRIM38	0	-1.74	0	0	O00635	TRIM38
1	TRIM39	3.5436E-32	1.71	2.0615E-27	3.3667E-31	Q9HCM9	TRIM39
1	TRIM44	9.404E-111	1.80	5.471E-106	1.732E-109	Q96DX7	TRIM44
1	TRIM46	3.8379E-24	-5.43	2.2326E-19	3.1651E-23	Q7Z4K8	TRIM46
1	TRIM47	0	-5.60	0	0	Q96LD4	TRIM47
1	TRIM51BP	6.4085E-10	-17.76	3.7281E-05	3.5999E-09		
1	TRIM51EP	2.2528E-09	-47.52	0.00013105	1.2358E-08		
1	TRIM52-AS1	2.871E-11	1.66	1.6702E-06	1.7074E-10		
1	TRIM55	1.2963E-08	-57.07	0.00075413	6.8732E-08	Q9BYV6	TRIM55
1	TRIM56	0	-3.22	0	0	Q9BRZ2	TRIM56
1	TRIM59	2.4585E-64	2.10	1.4302E-59	3.2417E-63	Q8IWR1	TRIM59
1	TRIM6	1.047E-256	-18.62	6.094E-252	2.878E-255	Q9C030	TRIM6
1	TRIM6-TRIM34	1.303E-07	-8.72	0.00758014	6.56E-07	B2RNG4	TRIM6-TRIM34
1	TRIM60P18	0.00216028	-1.64	1	0.0081473		
1	TRIM61	0.00186693	-23.57	1	0.00709153	Q5EBN2	TRIM61
1	TRIM68	2.328E-51	2.00	1.3543E-46	2.7577E-50	Q6AZZ1	TRIM68
1	TRIM73	9.0832E-19	2.26	5.2841E-14	6.7623E-18	Q86UV7	TRIM73
1	TRIM77	0.00020795	-15.19	1	0.00085766	I1YAP6	TRIM77
1	TRIM8	2.7439E-67	1.81	1.5963E-62	3.6925E-66	Q9BZR9	TRIM8
1	TRIM9	1.4825E-10	2.30	8.6244E-06	8.5594E-10	Q9C026	TRIM9
1	TRIML2	5.624E-185	67.22	3.272E-180	1.344E-183	Q8N7C3	TRIML2
1	TRIO	0	-2.12	0	0	O75962	TRIO
1	TRIOBP	0	-2.64	0	0	Q9H2D6	TRIOBP
1	TRIP12	3.1929E-70	1.70	1.8575E-65	4.387E-69	Q14669	TRIP12
1	TRIP13	0	-1.70	0	0	Q15645	TRIP13
1	TRIP6	0	-1.69	0	0	Q15654	TRIP6
1	TRIQK	2.8082E-11	-1.55	1.6336E-06	1.6704E-10	Q629K1	TRIQK
1	TRMT1	8.225E-143	2.09	4.785E-138	1.74E-141	Q9NXH9	TRMT1
1	TRMT10C	8.9657E-17	1.70	5.2157E-12	6.3997E-16	Q7L0Y3	TRMT10C
1	TRMT112	0	-1.79	0	0	Q9UI30	TRMT112
1	TRMT12	0	-1.82	0	0	Q53H54	TRMT12
1	TRMT2A	7.2818E-56	1.76	4.2361E-51	9.0054E-55	Q8IZ69	TRMT2A
1	TRMT5	2.2931E-32	1.73	1.334E-27	2.184E-31	Q32P41	TRMT5
1	TRMT61A	7.4046E-49	4.02	4.3075E-44	8.5756E-48	Q96FX7	TRMT61A
1	TRNT1	6.5332E-47	1.90	3.8007E-42	7.4231E-46	Q96Q11	TRNT1
1	TRO	1.1495E-09	-2.28	6.687E-05	6.3862E-09	Q12816	TRO
1	TRPA1	4.1462E-44	-28.85	2.412E-39	4.5743E-43	O75762	TRPA1
1	TRPC1	0	-2.49	0	0	P48995	TRPC1
1	TRPC3	1.9453E-27	-18.81	1.1316E-22	1.7112E-26	Q13507	TRPC3
1	TRPC4	4.6867E-07	-5.64	0.02726465	2.2908E-06	Q9UBN4	TRPC4
1	TRPC6	1.2281E-13	-7.59	7.1442E-09	7.9424E-13	Q9Y210	TRPC6
1	TRPC7	0.00061286	2.12	1	0.00243147	Q9HCX4	TRPC7

1	TRPM2	4.5705E-84	-6.62	2.6588E-79	7.0173E-83	O94759	TRPM2
1	TRPM2-AS	7.185E-81	-38.01	4.1798E-76	1.0745E-79		
1	TRPM3	0.00025123	-2.76	1	0.00102814	Q9HCF6	TRPM3
1	TRPM4	0	-2.07	0	0	Q8TD43	TRPM4
1	TRPM6	3.1208E-15	-4.73	1.8155E-10	2.1258E-14	Q9BX84	TRPM6
1	TRPS1	8.4625E-52	-2.49	4.923E-47	1.0063E-50	Q9UHF7	TRPS1
1	TRPT1	0	-1.60	0	0	Q86TN4	TRPT1
1	TRPV2	7.085E-06	-3.79	0.41216178	3.2319E-05	Q9Y5S1	TRPV2
1	TRPV4	1.1124E-92	-28.38	6.4716E-88	1.8209E-91	Q9HBA0	TRPV4
1	TSC1	0	-1.60	0	0	Q92574	TSC1
1	TSC22D3	2.614E-215	-5.75	1.52E-210	6.836E-214	Q99576	TSC22D3
1	TSC22D4	0	-1.52	0	0	Q9Y3Q8	TSC22D4
1	TSEN15	0	-2.05	0	0	Q8WW01	TSEN15
1	TSEN2	6.7171E-31	1.71	3.9076E-26	6.2642E-30	Q8NCE0	TSEN2
1	TSHZ2	0.00153738	2.05	1	0.00588431	Q9NRE2	TSHZ2
1	TSKU	0	-1.92	0	0	Q8WUA8	TSKU
1	TSLP	7.9618E-08	-26.69	0.00463169	4.0508E-07	Q969D9	TSLP
1	TSN	1.1331E-73	1.85	6.5915E-69	1.5968E-72	Q15631	TSN
1	TSNAX	0	-1.52	0	0	Q99598	TSNAX
1	TSNAX-DISC1	0.00220425	2.03	1	0.0083072		
1	TSNAXIP1	7.9845E-49	3.70	4.6449E-44	9.2399E-48	Q2TAA8	TSNAXIP1
1	TSPAN1	0	-7.27	0	0	O60635	TSPAN1
1	TSPAN11	4.3542E-19	-6.36	2.533E-14	3.2604E-18	A1L157	TSPAN11
1	TSPAN12	6.0255E-12	-1.70	3.5053E-07	3.6754E-11	O95859	TSPAN12
1	TSPAN13	8.205E-142	-4.24	4.773E-137	1.73E-140	O95857	TSPAN13
1	TSPAN15	0	-3.60	0	0	O95858	TSPAN15
1	TSPAN16	0.01430582	2.52	1	0.04934053	Q9UKR8	TSPAN16
1	TSPAN17	8.4872E-42	2.12	4.9373E-37	9.1028E-41	Q96FV3	TSPAN17
1	TSPAN19	2.81E-06	-7.98	0.16346779	1.3137E-05	P0C672	TSPAN19
1	TSPAN2	2.6314E-65	-18.64	1.5308E-60	3.4966E-64	O60636	TSPAN2
1	TSPAN31	0	-1.65	0	0	Q12999	TSPAN31
1	TSPAN33	1.7106E-33	2.78	9.9512E-29	1.656E-32	Q86UF1	TSPAN33
1	TSPAN4	5.1106E-98	5.07	2.973E-93	8.6804E-97	O14817	TSPAN4
1	TSPAN5	3.987E-110	2.83	2.319E-105	7.309E-109	P62079	TSPAN5
1	TSPAN6	4.1717E-26	1.65	2.4269E-21	3.571E-25	O43657	TSPAN6
1	TSPAN7	7.7654E-10	-5.15	4.5174E-05	4.3437E-09	P41732	TSPAN7
1	TSPAN8	0.00798163	-7.61	1	0.02836428	P19075	TSPAN8
1	TSPAN9	1.175E-101	3.13	6.8364E-97	2.044E-100	O75954	TSPAN9
1	TSPEAR	2.1107E-10	7.39	1.2279E-05	1.2106E-09	Q8WU66	TSPEAR
1	TSPEAR-AS1	4.3212E-06	5.54	0.25137899	1.9935E-05		
1	TSPO	6.7455E-19	1.56	3.9241E-14	5.0322E-18	P30536	TSPO
1	TSPY26P	2.5461E-09	-3.47	0.00014812	1.3944E-08	Q9H489	TSPY26P
1	TSPYL2	1.5398E-58	1.73	8.9574E-54	1.9435E-57	Q9H2G4	TSPYL2
1	TSPYL5	0.00196801	-21.58	1	0.00745165	Q86VY4	TSPYL5
1	TSR1	5.5149E-54	1.61	3.2082E-49	6.7188E-53	Q2NL82	TSR1
1	TSR3	1.175E-46	1.79	6.8352E-42	1.3303E-45	Q9UJK0	TSR3
1	TSSC1	0	-1.63	0	0		

1	TSSC4	2.1367E-62	3.31	1.243E-57	2.7733E-61	Q9Y5U2	TSSC4
1	TSSK2	0.0035383	-22.30	1	0.01307236	Q96PF2	TSSK2
1	TSSK3	0.00845163	1.60	1	0.02994308	Q96PN8	TSSK3
1	TSSK6	6.102E-12	1.85	3.5498E-07	3.7217E-11	Q9BXA6	TSSK6
1	TST	1.2547E-29	1.80	7.2989E-25	1.1464E-28	Q16762	TST
1	TSTD1	6.551E-190	-12.24	3.811E-185	1.594E-188	Q8NFU3	TSTD1
1	TTBK1	0.01415564	-7.01	1	0.04884283	Q5TCY1	TTBK1
1	TTC1	5.8082E-75	2.31	3.3789E-70	8.2653E-74	Q99614	TTC1
1	TTC13	0	-3.78	0	0		
1	TTC21A	7.0413E-15	2.06	4.0962E-10	4.7338E-14	Q8NDW8	TTC21A
1	TTC22	9.486E-196	-7.92	5.518E-191	2.361E-194		
1	TTC23L	1.7873E-08	-1.92	0.00103973	9.4246E-08	Q6PF05	TTC23L
1	TTC25	1.736E-07	-1.83	0.01009903	8.6791E-07	Q96NG3	TTC25
1	TTC26	4.748E-46	2.07	2.7621E-41	5.3477E-45	A0AVF1	TTC26
1	TTC28	2.0701E-40	4.94	1.2043E-35	2.1762E-39	Q96AY4	TTC28
1	TTC28-AS1	5.9171E-25	1.60	3.4422E-20	4.9614E-24		
1	TTC30B	4.0371E-24	-2.10	2.3485E-19	3.3279E-23	Q8N4P2	TTC30B
1	TTC32	0	-1.81	0	0	Q5I0X7	TTC32
1	TTC34	1.2498E-05	2.21	0.72707475	5.6114E-05		
1	TTC38	1.2809E-74	2.56	7.4513E-70	1.8187E-73	Q5R3I4	TTC38
1	TTC39A	4.776E-121	-7.05	2.779E-116	9.29E-120	Q5SRH9	TTC39A
1	TTC39B	1.683E-102	-3.80	9.7908E-98	2.942E-101		
1	TTC39C	2.7522E-29	-1.87	1.6011E-24	2.5005E-28		
1	TTC3P1	4.7905E-05	-1.68	1	0.0002068		
1	TTC4	3.5547E-36	1.54	2.0679E-31	3.5555E-35		
1	TTC41P	0.00581221	-1.51	1	0.02097908	Q6P2S7	TTC41P
1	TTC6	4.37E-178	11.31	2.542E-173	1.025E-176		
1	TTC7B	3.019E-114	10.42	1.756E-109	5.658E-113	Q86TV6	TTC7B
1	TTC8	2.1892E-89	2.26	1.2735E-84	3.4968E-88	Q8TAM2	TTC8
1	TTC9	4.4548E-07	-2.10	0.02591509	2.1801E-06		
1	TTI2	1.1242E-12	-1.56	6.54E-08	7.036E-12	Q6NXR4	TTI2
1	TTL	6.2721E-61	1.58	3.6488E-56	8.0458E-60	Q8NG68	TTL
1	TTLL10	6.9677E-27	-13.54	4.0534E-22	6.0408E-26	Q6ZVT0	TTLL10
1	TTLL10-AS1	4.3623E-10	-137.26	2.5377E-05	2.4669E-09		
1	TTLL12	1.1492E-75	2.10	6.6851E-71	1.6433E-74	Q14166	TTLL12
1	TTLL3	2.1094E-19	1.90	1.2271E-14	1.5887E-18	Q9Y4R7	TTLL3
1	TTLL5	4.3594E-69	1.74	2.536E-64	5.9281E-68	Q6EMB2	TTLL5
1	TTLL7	3.6048E-22	2.34	2.0971E-17	2.8621E-21	Q6ZT98	TTLL7
1	TTLL8	0.00364692	-3.15	1	0.01345314	A6PVC2	TTLL8
1	TTN	5.306E-153	27.57	3.087E-148	1.151E-151	Q8WZ42	TTN
1	TTN-AS1	1.685E-273	96.96	9.801E-269	4.685E-272		
1	TTPAL	3.9399E-43	2.32	2.292E-38	4.297E-42	Q9BTX7	TTPAL
1	TTYH1	0.006556	2.14	1	0.02350767	Q9H313	TTYH1
1	TTYH2	2.3836E-13	5.10	1.3866E-08	1.5258E-12	Q9BSA4	TTYH2
1	TTYH3	7.1396E-42	1.72	4.1534E-37	7.6687E-41	Q9C0H2	TTYH3
1	TUBA1A	0	58.16	0	0	Q71U36	TUBA1A
1	TUBA1B	2.5593E-41	1.81	1.4888E-36	2.7238E-40	P68363	TUBA1B

1	TUBA3FP	1.5767E-05	-4.65	0.91724517	7.0287E-05		
1	TUBA4A	0	-1.56	0	0	P68366	TUBA4A
1	TUBA8	7.2684E-05	1.90	1	0.00030983	Q9NY65	TUBA8
1	TUBAL3	1.4929E-05	-4.04	0.86850753	6.6685E-05	A6NHL2	TUBAL3
1	TUBB	8.4799E-51	1.80	4.9331E-46	9.9901E-50	P07437	TUBB
1	TUBB1	2.2554E-07	2.10	0.0131203	1.1211E-06	Q9H4B7	TUBB1
1	TUBB2A	1.3263E-86	5.52	7.7157E-82	2.073E-85	Q13885	TUBB2A
1	TUBB3	3.3179E-55	5.16	1.9302E-50	4.0824E-54	Q13509	TUBB3
1	TUBB4A	3.0397E-16	6.41	1.7683E-11	2.1385E-15	P04350	TUBB4A
1	TUBB6	2.0151E-25	2.86	1.1723E-20	1.7049E-24	Q9BUF5	TUBB6
1	TUBB8P12	0.00575356	19.88	1	0.02077768		
1	TUBBP5	0.00248433	2.24	1	0.00930786		
1	TUBE1	0	-1.75	0	0	Q9UJT0	TUBE1
1	TUBG1	8.5968E-38	1.57	5.0011E-33	8.7554E-37	P23258	TUBG1
1	TULP1	3.5092E-41	-16.70	2.0414E-36	3.728E-40	O00294	TULP1
1	TULP3	2.987E-108	2.17	1.738E-103	5.409E-107	O75386	TULP3
1	TULP4	1.2865E-25	1.67	7.4842E-21	1.0904E-24	Q9NRJ4	TULP4
1	TUSC1	2.224E-167	3.92	1.294E-162	5.055E-166		
1	TUSC2	6.0765E-28	1.63	3.5349E-23	5.4067E-27	O75896	TUSC2
1	TUT1	0	-2.31	0	0	Q9H6E5	TUT1
1	TVP23C	0.00418427	-1.57	1	0.01535941	Q96ET8	TVP23C
1	TWF2	1.329E-46	1.69	7.7315E-42	1.5042E-45	Q6IBS0	TWF2
1	TWIST1	8.4854E-24	-13.03	4.9363E-19	6.9574E-23	Q15672	TWIST1
1	TWNK	1.571E-134	2.23	9.142E-130	3.216E-133	Q96RR1	TWNK
1	TWSG1	1.728E-127	2.87	1.006E-122	3.457E-126	Q9GZX9	TWSG1
1	TXK	4.9352E-07	1.89	0.02870984	2.4098E-06	P42681	TXK
1	TXLNB	8.8569E-16	-19.88	5.1524E-11	6.1353E-15	Q8N3L3	TXLNB
1	TXN2	1.2487E-48	1.82	7.2639E-44	1.4433E-47	Q99757	TXN2
1	TXNDC15	1.7919E-28	3.09	1.0424E-23	1.6064E-27	Q96J42	TXNDC15
1	TXNDC16	1.6284E-15	1.51	9.4728E-11	1.1179E-14	Q9P2K2	TXNDC16
1	TXNDC17	6.1487E-82	2.51	3.5769E-77	9.2667E-81	Q9BRA2	TXNDC17
1	TXNDC5	1.9366E-46	1.69	1.1266E-41	2.1888E-45	Q8NBS9	TXNDC5
1	TXNDC9	1.3871E-24	1.85	8.0693E-20	1.1559E-23	O14530	TXNDC9
1	TXNL1	3.632E-112	3.47	2.113E-107	6.745E-111	O43396	TXNL1
1	TXNL4A	3.2459E-47	1.60	1.8882E-42	3.6966E-46	P83876	TXNL4A
1	TXNRD1	1.5926E-42	3.03	9.2646E-38	1.7236E-41	Q16881	TXNRD1
1	TXNRD3NB_1	1.5917E-21	-4.50	9.2593E-17	1.2502E-20		
1	TYK2	2.2734E-38	1.51	1.3225E-33	2.3333E-37	P29597	TYK2
1	TYMP	5.5364E-71	-4.30	3.2207E-66	7.6447E-70	P19971	TYMP
1	TYMS	1.5847E-63	3.20	9.2189E-59	2.0754E-62	P04818	TYMS
1	TYMSOS	6.1441E-09	-1.60	0.00035743	3.3034E-08	Q8TAI1	TYMSOS
1	TYRO3	0	-2.18	0	0	Q06418	TYRO3
1	TYRP1	8.2E-118	-10.09	4.77E-113	1.568E-116	P17643	TYRP1
1	TYSND1	0	-1.53	0	0	Q2T9J0	TYSND1
1	TYW1B	2.673E-190	-4.35	1.555E-185	6.517E-189	Q6NUM6	TYW1B
1	TYW5	1.8297E-17	1.59	1.0644E-12	1.3254E-16	A2RUC4	TYW5
1	U2AF1	1.1231E-41	3.58	6.5334E-37	1.2019E-40	Q01081	U2AF1

1	U2SURP	0	-2.34	0	0	O15042	U2SURP
1	U3_18	0.00045353	-5.47	1	0.0018183		
1	U47924.27	1.5299E-75	-11.06	8.9001E-71	2.1862E-74		
1	U47924.29	8.6486E-08	-14.31	0.00503123	4.3918E-07		
1	U52111.14	7.437E-151	27.51	4.327E-146	1.604E-149		
1	U91328.19	4.6999E-06	-1.51	0.27341159	2.1639E-05		
1	UAP1	8.4651E-86	2.38	4.9245E-81	1.3157E-84	Q16222	UAP1
1	UAP1L1	7.052E-164	59.45	4.102E-159	1.583E-162	Q3KQV9	UAP1L1
1	UBA5	0	-1.54	0	0	Q9GZZ9	UBA5
1	UBA52	4.2E-113	2.95	2.443E-108	7.838E-112	P62987	UBA52
1	UBA6-AS1	0	-1.65	0	0		
1	UBA7	1.6623E-26	-3.01	9.6705E-22	1.4327E-25	P41226	UBA7
1	UBAC2	0	-1.54	0	0	Q8NBM4	UBAC2
1	UBAC2-AS1	1.368E-12	-1.96	7.9579E-08	8.5349E-12		
1	UBALD1	7.086E-109	2.60	4.122E-104	1.288E-107		
1	UBALD2	0	-1.69	0	0		
1	UBAP1	5.3359E-52	1.54	3.1041E-47	6.3635E-51	Q9NZ09	UBAP1
1	UBAP1L	3.7209E-06	1.65	0.21646133	1.7237E-05	F5GYI3	UBAP1L
1	UBAP2	1.154E-103	1.86	6.713E-99	2.034E-102	Q5T6F2	UBAP2
1	UBASH3B	0	115.64	0	0	Q8TF42	UBASH3B
1	UBB	2.2223E-36	1.88	1.2928E-31	2.2293E-35	P0CG47	UBB
1	UBBP4	0.00019568	2.27	1	0.00080879		
1	UBE2C	9.0449E-37	1.77	5.2618E-32	9.1082E-36	O00762	UBE2C
1	UBE2D1	5.0125E-16	1.69	2.916E-11	3.5018E-15	P51668	UBE2D1
1	UBE2E2	0	-2.40	0	0	Q96LR5	UBE2E2
1	UBE2H	0	-1.94	0	0	P62256	UBE2H
1	UBE2I	1.1277E-55	1.56	6.5601E-51	1.3931E-54	P63279	UBE2I
1	UBE2J1	5.8372E-39	3.43	3.3957E-34	6.0368E-38	Q9Y385	UBE2J1
1	UBE2J2	1.826E-115	2.06	1.062E-110	3.45E-114	Q8N2K1	UBE2J2
1	UBE2L6	8.691E-135	-5.09	5.056E-130	1.782E-133	O14933	UBE2L6
1	UBE2N	3.8435E-25	1.55	2.2359E-20	3.2344E-24	P61088	UBE2N
1	UBE2Q2L	0.00032078	2.68	1	0.00130104		
1	UBE2Q2P2	3.8116E-06	-2.25	0.2217351	1.7648E-05		
1	UBE2R2	2.2336E-95	1.88	1.2994E-90	3.7274E-94	Q712K3	UBE2R2
1	UBE2R2-AS1	1.7597E-22	2.41	1.0237E-17	1.4067E-21		
1	UBE2T	1.9207E-13	-1.50	1.1173E-08	1.2348E-12	Q9NPD8	UBE2T
1	UBE2V1	1.0873E-41	2.17	6.3251E-37	1.164E-40	Q13404	UBE2V1
1	UBE3B	0	-1.61	0	0	Q7Z3V4	UBE3B
1	UBE3D	2.747E-18	1.72	1.598E-13	2.0231E-17	Q7Z6J8	UBE3D
1	UBIAD1	6.7817E-24	1.60	3.9452E-19	5.5699E-23	Q9Y5Z9	UBIAD1
1	UBL4A	4.521E-115	2.50	2.63E-110	8.521E-114	P11441	UBL4A
1	UBL4B	1.3503E-05	-49.95	0.78550358	6.0475E-05	Q8N7F7	UBL4B
1	UBL7	1.3396E-39	1.82	7.7929E-35	1.3936E-38	Q96S82	UBL7
1	UBLCP1	4.2752E-38	1.76	2.487E-33	4.3724E-37	Q8WVY7	UBLCP1
1	UBQLNL	5.2557E-22	-160.07	3.0574E-17	4.1632E-21		
1	UBR2	0	-1.70	0	0	Q8IWV8	UBR2
1	UBR5	0	-1.77	0	0	O95071	UBR5

1	UBR5-AS1	3.366E-16	-2.05	1.9581E-11	2.366E-15		
1	UBR7	2.8469E-53	3.65	1.6561E-48	3.4396E-52	Q8N806	UBR7
1	UBTD2	2.2944E-84	3.28	1.3348E-79	3.5302E-83	Q8WUN7	UBTD2
1	UCA1_1	0	-17.60	0	0		
1	UCHL1	0	-8.93	0	0	P09936	UCHL1
1	UCHL3	1.6793E-22	1.62	9.7694E-18	1.3438E-21	P15374	UCHL3
1	UCKL1	2.0214E-29	1.57	1.1759E-24	1.8405E-28	Q9NWZ5	UCKL1
1	UCN	6.6183E-12	5.10	3.8502E-07	4.0303E-11	P55089	UCN
1	UCN2	8.9338E-67	-4.44	5.1972E-62	1.1975E-65	Q96RP3	UCN2
1	UCP1	0.0003632	-3.65	1	0.00146739	P25874	UCP1
1	UCP2	7.792E-137	18.44	4.533E-132	1.613E-135	P55851	UCP2
1	UFD1L	1.964E-100	1.71	1.1424E-95	3.386E-99		
1	UFL1-AS1	9.3092E-38	-3.90	5.4156E-33	9.4711E-37		
1	UFSP1	0.00363634	-1.65	1	0.01341495	Q6NVU6	UFSP1
1	UFSP2	0	-1.69	0	0	Q9NUQ7	UFSP2
1	UGCG	0	-2.44	0	0	Q16739	UGCG
1	UGDH	5.4946E-46	3.72	3.1964E-41	6.1851E-45	O60701	UGDH
1	UGDH-AS1	1.1785E-25	-1.84	6.856E-21	9.9971E-25		
1	UGT1A1	4.9502E-06	-7.90	0.28797052	2.2763E-05	P22309	UGT1A1
1	UGT1A10	0.00225089	-7.71	1	0.00846762	Q9HAW8	UGT1A10
1	UGT1A4	0.00090267	-28.90	1	0.00352857	P22310	UGT1A4
1	UGT1A6	8.145E-138	-17.40	4.738E-133	1.691E-136	P19224	UGT1A6
1	UGT1A7	0.00803247	-17.83	1	0.02853101	Q9HAW7	UGT1A7
1	UGT2B7	6.4983E-21	31.73	3.7803E-16	5.0411E-20	P16662	UGT2B7
1	UGT3A2	0.0001584	-30.74	1	0.00065837	Q3SY77	UGT3A2
1	UGT8	0	-10.27	0	0	Q16880	UGT8
1	UHRF1	5.501E-117	2.25	3.2E-112	1.048E-115	Q96T88	UHRF1
1	UIMC1	9.8938E-65	1.80	5.7556E-60	1.3084E-63	Q96RL1	UIMC1
1	ULBP1	1.4437E-11	3.10	8.3986E-07	8.696E-11	Q9BZM6	ULBP1
1	ULBP2	1.9224E-52	2.35	1.1184E-47	2.3026E-51	Q9BZM5	ULBP2
1	ULBP3	2.5223E-52	3.85	1.4673E-47	3.0186E-51	Q9BZM4	ULBP3
1	ULK2	1.6819E-27	-14.60	9.7844E-23	1.4811E-26	Q8IYT8	ULK2
1	ULK4	9.0017E-62	3.23	5.2366E-57	1.1624E-60	Q96C45	ULK4
1	ULK4P3	1.4425E-05	5.85	0.83913626	6.4494E-05		
1	UMAD1	6.6613E-16	-1.50	3.8752E-11	4.6304E-15		
1	UMODL1	7.8437E-09	-21.62	0.0004563	4.197E-08	Q5DID0	UMODL1
1	UMPS	6.2959E-29	1.61	3.6626E-24	5.6908E-28	P11172	UMPS
1	UNC13B	3.9851E-61	1.81	2.3183E-56	5.1199E-60	O14795	UNC13B
1	UNC13D	0	-10.65	0	0	Q70J99	UNC13D
1	UNC5B	7.2789E-84	-10.41	4.2344E-79	1.1146E-82	Q8IZJ1	UNC5B
1	UNC5B-AS1	3.1574E-18	-14.56	1.8368E-13	2.323E-17		
1	UNC5C	0.00050636	-1.78	1	0.00202219	O95185	UNC5C
1	UNC5CL	1.971E-05	1.78	1	8.7293E-05	Q8IV45	UNC5CL
1	UNC79	0.00232497	-1.52	1	0.00873333	Q9P2D8	UNC79
1	UNC80	3.982E-05	-32.79	1	0.00017288	Q8N2C7	UNC80
1	UNC93B1	0	-5.32	0	0	Q9H1C4	UNC93B1
1	UNC93B2	0.01254323	-4.29	1	0.04350124		

1	UNKL	0	-1.82	0	0	Q9H9P5	UNKL
1	UPB1	0.01298231	3.79	1	0.04493829	Q9UBR1	UPB1
1	UPK1B	2.7624E-31	-10.62	1.607E-26	2.5899E-30	O75841	UPK1B
1	UPK2	6.9212E-95	-5.99	4.0263E-90	1.1504E-93	O00526	UPK2
1	UPK3B	0	-16.49	0	0	Q9BT76	UPK3B
1	UPK3BL	8.7525E-88	-5.97	5.0917E-83	1.3806E-86		
1	UPP1	0	-5.64	0	0	Q16831	UPP1
1	UPRT	8.6574E-19	1.78	5.0364E-14	6.4478E-18	Q96BW1	UPRT
1	UQCC2	0	-1.58	0	0	Q9BRT2	UQCC2
1	UQCRHL	0.00145034	-2.77	1	0.00556029	A0A096LP55	UQCRHL
1	UQCRQ	1.705E-11	1.53	9.9184E-07	1.0237E-10	O14949	UQCRQ
1	UROD	1.8765E-79	3.61	1.0917E-74	2.77E-78	P06132	UROD
1	USE1	2.4631E-13	1.70	1.4329E-08	1.5754E-12	Q9NZ43	USE1
1	USO1	0	-1.87	0	0	O60763	USO1
1	USP1	2.6737E-50	1.75	1.5554E-45	3.1378E-49	O94782	USP1
1	USP12-AS2	5.0561E-05	-14.29	1	0.00021792		
1	USP14	5.462E-117	2.27	3.177E-112	1.041E-115	P54578	USP14
1	USP16	8.4709E-70	1.96	4.9279E-65	1.1584E-68	Q9Y5T5	USP16
1	USP2-AS1	3.9605E-05	2.18	1	0.00017197		
1	USP21	0	-1.98	0	0	Q9UK80	USP21
1	USP26	3.413E-110	-23.42	1.985E-105	6.261E-109	Q9BXU7	USP26
1	USP27X	1.8394E-06	1.54	0.10700436	8.7017E-06	A6NNY8	USP27X
1	USP3-AS1	8.2393E-13	-2.15	4.7931E-08	5.1817E-12		
1	USP32P1	0.00092715	-2.66	1	0.00362157		
1	USP32P2	4.8347E-07	-6.80	0.02812539	2.3617E-06		
1	USP32P3	1.1835E-25	-9.46	6.885E-21	1.0038E-24		
1	USP37	4.0118E-21	1.51	2.3338E-16	3.126E-20	Q86T82	USP37
1	USP39	2.7927E-62	1.75	1.6247E-57	3.6224E-61	Q53GS9	USP39
1	USP40	0	-1.64	0	0	Q9NVE5	USP40
1	USP42	0	-1.54	0	0	Q9H9J4	USP42
1	USP43	1.5077E-13	-1.52	8.7708E-09	9.7237E-13	Q70EL4	USP43
1	USP44	3.0394E-10	-4.17	1.7681E-05	1.7306E-09	Q9H0E7	USP44
1	USP46-AS1	1.7845E-05	-1.69	1	7.9284E-05		
1	USP47	2.9907E-47	1.66	1.7398E-42	3.408E-46	Q96K76	USP47
1	USP51	0.00205959	-1.68	1	0.00778018	Q70EK9	USP51
1	USP6	0.00028336	-2.76	1	0.00115389	P35125	USP6
1	USP9X	0	-2.18	0	0	Q93008	USP9X
1	UST	1.1016E-16	-1.69	6.4082E-12	7.8427E-16	Q9Y2C2	UST
1	UTP11	2.2584E-47	1.84	1.3138E-42	2.5761E-46	Q9Y3A2	UTP11
1	UTP14A	7.7014E-72	1.95	4.4802E-67	1.0708E-70	Q9BVJ6	UTP14A
1	UTP15	1.734E-104	2.64	1.009E-99	3.076E-103	Q8TED0	UTP15
1	UTP20	7.1624E-44	1.82	4.1666E-39	7.8914E-43	O75691	UTP20
1	UVSSA	0	-2.85	0	0	Q2YD98	UVSSA
1	VAMP5	1.6974E-08	-1.87	0.00098747	8.9591E-08	O95183	VAMP5
1	VAMP8	0	-3.42	0	0	Q9BV40	VAMP8
1	VANGL2	9.311E-205	-9.73	5.416E-200	2.376E-203	Q9ULK5	VANGL2
1	VAPB	7.2856E-41	2.08	4.2383E-36	7.7018E-40	O95292	VAPB

1	VARS2	2.6361E-98	1.96	1.5335E-93	4.484E-97	Q5ST30	VARS2
1	VASH1	7.636E-199	12.03	4.442E-194	1.916E-197	Q7L8A9	VASH1
1	VAV1	0.00022661	-4.66	1	0.0009316	P15498	VAV1
1	VAV2	0	-1.63	0	0	P52735	VAV2
1	VAV3	1.9015E-07	4.08	0.01106196	9.4855E-07	Q9UKW4	VAV3
1	VAX2	1.2957E-09	-1.57	7.5373E-05	7.1839E-09	Q9UIW0	VAX2
1	VCAM1	6.386E-112	828.63	3.715E-107	1.185E-110	P19320	VCAM1
1	VCAN	0	-1.66	0	0	P13611	VCAN
1	VCP	5.71E-104	1.84	3.322E-99	1.01E-102	P55072	VCP
1	VCX	2.8408E-06	-5.24	0.16525972	1.3269E-05	Q9H320	VCX
1	VDAC1	2.3772E-59	1.82	1.3829E-54	3.0149E-58	P21796	VDAC1
1	VDR	0	-5.74	0	0	P11473	VDR
1	VEGFA	0	-6.87	0	0	P15692	VEGFA
1	VEGFC	2.081E-158	16.05	1.211E-153	4.605E-157	P49767	VEGFC
1	VEGFD	1.3409E-07	-3.70	0.0078008	6.7475E-07	O43915	VEGFD
1	VEPH1	3.4182E-50	6.77	1.9885E-45	4.0074E-49	Q14D04	VEPH1
1	VEZF1	1.141E-104	1.86	6.636E-100	2.025E-103	Q14119	VEZF1
1	VGF	1.3553E-15	2.93	7.8843E-11	9.3195E-15	O15240	VGF
1	VGLL1	6.2348E-05	-30.94	1	0.00026701	Q99990	VGLL1
1	VILL	1.2402E-59	-6.93	7.2148E-55	1.577E-58	O15195	VILL
1	VIM	1.2516E-52	197.37	7.2813E-48	1.5022E-51	P08670	VIM
1	VIM-AS1	3.4334E-35	17.89	1.9974E-30	3.3928E-34		
1	VIPAS39	1.2247E-52	1.74	7.1243E-48	1.4704E-51	Q9H9C1	VIPAS39
1	VIPR1	8.7269E-88	-12.30	5.0768E-83	1.3773E-86	P32241	VIPR1
1	VIPR1-AS1	0.00506535	-2.36	1	0.01841583		
1	VKORC1	2.413E-101	2.73	1.4039E-96	4.189E-100	Q9BQB6	VKORC1
1	VLDLR	6.362E-137	-3.19	3.701E-132	1.318E-135	P98155	VLDLR
1	VLDLR-AS1	2.1439E-08	-2.75	0.00124719	1.1251E-07		
1	VMO1	2.7564E-59	21.56	1.6035E-54	3.4942E-58	Q7Z5L0	VMO1
1	VN1R51P	0.00022994	-4.39	1	0.00094467		
1	VN1R83P	0.0002028	4.36	1	0.00083713		
1	VN1R85P	4.6252E-12	94.51	2.6907E-07	2.8332E-11		
1	VN1R87P	0.00836288	17.61	1	0.02965408		
1	VNN1	2.2819E-15	-9.28	1.3274E-10	1.5595E-14	O95497	VNN1
1	VPREB3	0.00656674	-2.72	1	0.02354184	Q9UKI3	VPREB3
1	VPS13D	0	-1.58	0	0	Q5THJ4	VPS13D
1	VPS18	5.664E-117	2.17	3.295E-112	1.078E-115	Q9P253	VPS18
1	VPS26A	2.8054E-32	1.64	1.632E-27	2.6684E-31	O75436	VPS26A
1	VPS33A	3.8778E-24	1.77	2.2559E-19	3.1971E-23	Q96AX1	VPS33A
1	VPS33B	0	-1.53	0	0	Q9H267	VPS33B
1	VPS37C	0	-2.02	0	0	A5D8V6	VPS37C
1	VPS37D	1.55E-42	8.84	9.0172E-38	1.6786E-41	Q86XT2	VPS37D
1	VPS39	0	-1.73	0	0	Q96JC1	VPS39
1	VPS41	0	-1.73	0	0	P49754	VPS41
1	VPS45	0	-1.64	0	0	Q9NRW7	VPS45
1	VPS50	0	-1.67	0	0	Q96JG6	VPS50
1	VSIG1	1.4916E-17	-4.48	8.677E-13	1.0826E-16	Q86XK7	VSIG1

1	VSIG10	3.4102E-65	1.83	1.9839E-60	4.5221E-64	Q8N0Z9	VSIG10
1	VSIG10L	3.1897E-29	2.78	1.8556E-24	2.8957E-28	Q86VR7	VSIG10L
1	VSIG2	1.1964E-09	-3.13	6.96E-05	6.6399E-09	Q96IQ7	VSIG2
1	VSIR	8.966E-221	-5.40	5.216E-216	2.373E-219	Q9H7M9	VSIR
1	VSNL1	5.306E-161	-14.39	3.087E-156	1.182E-159	P62760	VSNL1
1	VSTM1	5.801E-262	92.74	3.374E-257	1.604E-260	Q6UX27	VSTM1
1	VSTM5	2.1866E-07	-3.51	0.01272018	1.0878E-06	A8MXK1	VSTM5
1	VTA1	6.2338E-18	1.50	3.6264E-13	4.5587E-17	Q9NP79	VTA1
1	VTI1B	3.4381E-90	2.80	2.0001E-85	5.5267E-89	Q9UEU0	VTI1B
1	VWA1	1.1397E-87	-11.24	6.6304E-83	1.7959E-86	Q6PCB0	VWA1
1	VWA2	4.6126E-26	-16.67	2.6834E-21	3.9438E-25	Q5GFL6	VWA2
1	VWA3A	0.00049543	2.55	1	0.00197974	A6NCI4	VWA3A
1	VWA3B	1.9831E-06	-43.83	0.11536588	9.3641E-06	Q502W6	VWA3B
1	VWA5A	7.4418E-36	3.87	4.3292E-31	7.413E-35		
1	VWA5B2	7.4134E-06	3.60	0.43126569	3.3756E-05		
1	VWA7	1.0201E-19	-3.19	5.9341E-15	7.7418E-19	Q9Y334	VWA7
1	VWA8	0	-1.53	0	0	A3KMH1	VWA8
1	VWA8-AS1	9.0585E-08	-3.67	0.00526968	4.5947E-07		
1	VWC2L	1.0678E-08	31.87	0.0006212	5.6824E-08	B2RUY7	VWC2L
1	VWCE	5.7917E-41	-3.87	3.3693E-36	6.1282E-40	Q96DN2	VWCE
1	VWDE	1.217E-203	-10.67	7.08E-199	3.098E-202	Q8N2E2	VWDE
1	VWF	1.168E-168	-20.56	6.794E-164	2.663E-167	P04275	VWF
1	WAC-AS1	7.675E-127	-3.02	4.465E-122	1.53E-125		
1	WARS	2.2904E-89	2.13	1.3324E-84	3.6565E-88	P23381	WARS
1	WARS2	2.6829E-18	1.51	1.5608E-13	1.9761E-17	Q9UGM6	WARS2
1	WARS2-IT1	0.00269806	-9.49	1	0.01007814		
1	WAS	0.00060031	-1.86	1	0.00238312	P42768	WAS
1	WASF3	2.121E-102	59.94	1.2337E-97	3.705E-101	Q9UPY6	WASF3
1	WASH2P	2.1087E-10	-1.54	1.2267E-05	1.2095E-09	Q6VEQ5	WASH2P
1	WASH3P	0	-1.85	0	0	C4AMC7	WASH3P
1	WASH5P	8.8818E-16	-2.01	5.1669E-11	6.1437E-15		
1	WASH7P	0	-2.74	0	0		
1	WASHC2A	8.9452E-47	2.33	5.2038E-42	1.0148E-45	Q641Q2	WASHC2A
1	WASHC2C	2.3999E-23	2.54	1.3961E-18	1.9513E-22	Q9Y4E1	WASHC2C
1	WASIR2	0.00025705	-27.19	1	0.00105093		
1	WBP11	1.0502E-52	1.65	6.1095E-48	1.2618E-51	Q9Y2W2	WBP11
1	WBP1LP2	4.4785E-05	3.07	1	0.00019379		
1	WBP2	0	-1.58	0	0	Q969T9	WBP2
1	WBSCR22	0	-1.50	0	0		
1	WBSCR27	1.9875E-54	4.25	1.1562E-49	2.428E-53		
1	WBSCR28	3.6906E-05	-7.69	1	0.00016066		
1	WDCP	2.3173E-16	1.55	1.3481E-11	1.6342E-15	Q9H6R7	WDCP
1	WDFY1	6.8517E-45	1.63	3.9859E-40	7.6197E-44	Q8IWB7	WDFY1
1	WDFY3-AS2	1.1405E-21	-2.31	6.6348E-17	8.9841E-21		
1	WDR11	0	-1.64	0	0	Q9BZH6	WDR11
1	WDR11-AS1	6.5008E-09	-3.87	0.00037818	3.4913E-08		
1	WDR12	2.5732E-31	1.57	1.497E-26	2.4137E-30	Q9GZL7	WDR12

1	WDR17	3.553E-126	-13.88	2.067E-121	7.068E-125	Q8IZU2	WDR17
1	WDR20	3.6263E-79	1.95	2.1096E-74	5.342E-78	Q8TBZ3	WDR20
1	WDR24	1.4364E-26	1.57	8.3562E-22	1.2392E-25	Q96S15	WDR24
1	WDR25	1.1255E-76	3.47	6.5476E-72	1.6235E-75	Q64LD2	WDR25
1	WDR3	1.7652E-91	1.93	1.0269E-86	2.8652E-90	Q9UNX4	WDR3
1	WDR31	1.4881E-12	-1.57	8.657E-08	9.2747E-12		
1	WDR34	2.6529E-46	1.62	1.5433E-41	2.995E-45	Q96EX3	WDR34
1	WDR35	2.9613E-80	2.93	1.7227E-75	4.4081E-79	Q9P2L0	WDR35
1	WDR36	1.4925E-59	1.91	8.6827E-55	1.8974E-58	Q8NI36	WDR36
1	WDR37	0	-1.55	0	0	Q9Y2I8	WDR37
1	WDR4	8.432E-21	1.52	4.9052E-16	6.5307E-20	P57081	WDR4
1	WDR41	3.5082E-70	1.83	2.0409E-65	4.8167E-69	Q9HAD4	WDR41
1	WDR46	9.33E-34	1.50	5.4276E-29	9.0733E-33	O15213	WDR46
1	WDR47	1.2813E-68	1.99	7.4536E-64	1.7358E-67	O94967	WDR47
1	WDR55	8.4027E-82	1.68	4.8882E-77	1.2651E-80	Q9H6Y2	WDR55
1	WDR59	0	-1.68	0	0	Q6PJI9	WDR59
1	WDR60	6.6616E-34	1.92	3.8753E-29	6.4913E-33	Q8WVS4	WDR60
1	WDR63	1.6987E-18	-3.62	9.882E-14	1.2561E-17	Q8IWG1	WDR63
1	WDR66	0	-1.55	0	0	Q8TBY9	WDR66
1	WDR7	1.6179E-67	3.09	9.4118E-63	2.1792E-66	Q9Y4E6	WDR7
1	WDR72	0	-2.73	0	0	Q3MJ13	WDR72
1	WDR76	2.4167E-44	1.65	1.4059E-39	2.6707E-43	Q9H967	WDR76
1	WDR77	3.23E-108	1.85	1.879E-103	5.845E-107	Q9BQA1	WDR77
1	WDR78	4.4271E-47	-2.97	2.5754E-42	5.037E-46	Q5VTH9	WDR78
1	WDR81	2.072E-31	1.59	1.2054E-26	1.9457E-30	Q562E7	WDR81
1	WDR82P1	6.6598E-10	-9.29	3.8743E-05	3.7382E-09		
1	WDR83	5.4889E-27	1.71	3.1931E-22	4.7744E-26	Q9BRX9	WDR83
1	WDR88	9.8681E-73	-3.06	5.7407E-68	1.3796E-71		
1	WDR89	3.2321E-57	2.20	1.8803E-52	4.0297E-56		
1	WDR93	8.5976E-15	-3.47	5.0016E-10	5.7688E-14	Q6P2C0	WDR93
1	WDTC1	6.5273E-32	1.55	3.7972E-27	6.1753E-31	Q8N5D0	WDTC1
1	WEE1	6.8945E-55	1.69	4.0108E-50	8.4545E-54	P30291	WEE1
1	WFDC2	2.0779E-47	-6.42	1.2088E-42	2.3725E-46	Q14508	WFDC2
1	WFDC21P	0.00065683	-2.14	1	0.0025981		
1	WFIKKN2	0.00186638	-3.97	1	0.0070899	Q8TEU8	WFIKKN2
1	WHAMMP3	2.3621E-20	2.91	1.3741E-15	1.8119E-19	Q1A5X7	WHAMMP3
1	WI2-87327B8	9.3574E-26	25.34	5.4436E-21	7.9583E-25		
1	WIPF1	9.2639E-21	-4.24	5.3892E-16	7.1665E-20	O43516	WIPF1
1	WIPF3	9.6969E-43	-6.12	5.6411E-38	1.0526E-41	A6NGB9	WIPF3
1	WIPI1	2.918E-152	3.98	1.698E-147	6.318E-151	Q5MNZ9	WIPI1
1	WISP3	2.081E-180	-11.09	1.21E-175	4.912E-179	O95389	WISP3
1	WNK1	2.9828E-55	1.75	1.7352E-50	3.6709E-54	Q9H4A3	WNK1
1	WNK2	4.2372E-07	-5.91	0.02464971	2.0758E-06	Q9Y3S1	WNK2
1	WNK3	0.00050309	-3.10	1	0.00200952	Q9BYP7	WNK3
1	WNK4	1.2217E-76	15.87	7.1072E-72	1.7614E-75	Q96J92	WNK4
1	WNT10A	3.4779E-82	-29.74	2.0232E-77	5.2552E-81	Q9GZT5	WNT10A
1	WNT10B	1.1612E-29	2.64	6.7553E-25	1.0618E-28	O00744	WNT10B

1	WNT11	5.0156E-16	-4.15	2.9178E-11	3.5036E-15	O96014	WNT11
1	WNT16	0	-4.25	0	0	Q9UBV4	WNT16
1	WNT2B	8.586E-33	2.40	4.9948E-28	8.2355E-32	Q93097	WNT2B
1	WNT3	4.6333E-15	-1.75	2.6954E-10	3.1323E-14	P56703	WNT3
1	WNT4	1.7916E-37	-8.69	1.0422E-32	1.8183E-36	P56705	WNT4
1	WNT5B	0	55.07	0	0	Q9H1J7	WNT5B
1	WNT6	1.0379E-44	-16.13	6.038E-40	1.1505E-43	Q9Y6F9	WNT6
1	WNT7A	0	-10.97	0	0	O00755	WNT7A
1	WNT9A	0	-9.28	0	0	O14904	WNT9A
1	WRAP53	8.8985E-69	1.92	5.1766E-64	1.2075E-67	Q9BUR4	WRAP53
1	WRAP73	1.9836E-81	2.14	1.1539E-76	2.9771E-80	Q9P2S5	WRAP73
1	WRN	0	-2.17	0	0	Q14191	WRN
1	WSB1	0	-1.91	0	0	Q9Y6I7	WSB1
1	WSCD1	0	251.47	0	0	Q658N2	WSCD1
1	WSCD2	0.00786082	-15.97	1	0.02796228	Q2TBF2	WSCD2
1	WT1	1.9362E-19	-11.00	1.1264E-14	1.4594E-18	P19544	WT1
1	WT1-AS	3.6491E-08	-19.78	0.00212281	1.891E-07	Q06250	WT1-AS
1	WTAPP1	7.1669E-25	-5.24	4.1693E-20	6.0007E-24		
1	WTIP	5.6152E-40	2.99	3.2666E-35	5.871E-39	A6NIX2	WTIP
1	WWC2	0	-1.53	0	0	Q6AWC2	WWC2
1	WWC2-AS2	5.2496E-13	-4.55	3.0539E-08	3.3249E-12		
1	WWC3	0	-3.66	0	0	Q9ULE0	WWC3
1	WWP1	0	-1.90	0	0	Q9H0M0	WWP1
1	WWTR1	1.2517E-64	1.77	7.2814E-60	1.6545E-63	Q9GZV5	WWTR1
1	WWTR1-AS1	2.7564E-07	-3.01	0.01603531	1.3637E-06		
1	XAB2	7.0048E-51	1.63	4.0749E-46	8.2572E-50	Q9HCS7	XAB2
1	XAF1	2.2462E-22	-12.28	1.3067E-17	1.7913E-21	Q6GPH4	XAF1
1	XBP1	4.8136E-27	1.69	2.8003E-22	4.1945E-26	P17861	XBP1
1	XG	8.54E-220	-21.96	4.968E-215	2.253E-218	P55808	XG
1	XIST	6.2577E-93	-138.76	3.6404E-88	1.0257E-91		
1	XK	6.0023E-26	-11.23	3.4918E-21	5.1176E-25	P51811	XK
1	XKR6	4.101E-222	110.28	2.386E-217	1.088E-220	Q5GH73	XKR6
1	XKR8	1.0375E-13	1.58	6.0356E-09	6.7264E-13	Q9H6D3	XKR8
1	XPO6	6.6977E-88	1.53	3.8963E-83	1.0585E-86	Q96QU8	XPO6
1	XPR1	0	-2.64	0	0	Q9UBH6	XPR1
1	XRCC3	1.7125E-58	1.87	9.9621E-54	2.1596E-57	O43542	XRCC3
1	XRCC5	5.2298E-29	1.61	3.0424E-24	4.7316E-28	P13010	XRCC5
1	XRN1	0	-2.51	0	0	Q8IZH2	XRN1
1	XRRA1	0	-2.30	0	0	Q6P2D8	XRRA1
1	XX-C2158C6.1	0.0035383	22.11	1	0.01307236		
1	XX-FW83128A	0.00885946	17.59	1	0.03130216		
1	XXbac-B444P2	0.00039136	3.25	1	0.00157643		
1	XXbac-B461K1	2.1967E-07	1.54	0.01277928	1.0927E-06		
1	XXbac-BPG11(	7.9742E-05	-40.15	1	0.00033903		
1	XXbac-BPG15	6.4866E-05	4.02	1	0.00027752		
1	XXbac-BPG18	6.0185E-11	1.94	3.5012E-06	3.5255E-10		
1	XXbac-BPG24	0.00514482	-17.89	1	0.01868842		

1	XXbac-BPG29	6.3558E-07	-70.81	0.03697405	3.084E-06		
1	XXbac-BPG30	0.00678414	2.93	1	0.0242868		
1	XXbac-BPGBP	5.0885E-19	-2.48	2.9602E-14	3.8053E-18		
1	XXbac-BPGBP	0.00491011	-2.22	1	0.01787489		
1	XXcos-LUCA1	4.9654E-19	-4.07	2.8886E-14	3.7143E-18		
1	XXyac-YX65C7	5.136E-11	2.92	2.9878E-06	3.0195E-10		
1	XXyac-YX65C7	0.00226469	21.77	1	0.00851732		
1	XXYLT1	0	-1.81	0	0	Q8NBI6	XXYLT1
1	XYLB	4.298E-29	2.10	2.5003E-24	3.8946E-28	O75191	XYLB
1	XYLT2	0	-1.91	0	0	Q9H1B5	XYLT2
1	YARS2	4.8805E-40	1.80	2.8392E-35	5.1119E-39	Q9Y2Z4	YARS2
1	YBX2	6.9126E-12	-14.35	4.0213E-07	4.2082E-11	Q9Y2T7	YBX2
1	YBX3	6.0693E-73	1.61	3.5307E-68	8.5099E-72	P16989	YBX3
1	YDJC	4.772E-52	2.18	2.7761E-47	5.6957E-51	A8MPS7	YDJC
1	YIPF3	0	-1.75	0	0	Q9GZM5	YIPF3
1	YIPF7	0.00341288	-1.99	1	0.01263868	Q8N8F6	YIPF7
1	YOD1	0	-1.72	0	0	Q5VVQ6	YOD1
1	YPEL1	1.4259E-20	5.97	8.2947E-16	1.0979E-19	O60688	YPEL1
1	YPEL2	6.8068E-18	-2.11	3.9598E-13	4.9727E-17	Q96QA6	YPEL2
1	YPEL3	1.8446E-10	-1.71	1.0731E-05	1.061E-09	P61236	YPEL3
1	YPEL5	0	-1.81	0	0	P62699	YPEL5
1	YRDC	2.5361E-77	2.12	1.4753E-72	3.6755E-76	Q86U90	YRDC
1	YTHDF1	1.479E-120	1.85	8.604E-116	2.869E-119	Q9BYJ9	YTHDF1
1	YTHDF3-AS1	4.8461E-08	-2.26	0.00281916	2.4948E-07		
1	YWHAB	1.0215E-87	2.09	5.9423E-83	1.6104E-86	P31946	YWHAB
1	YWHAEP7	6.8419E-48	-14.35	3.9802E-43	7.8536E-47		
1	YWHAZ	0	-1.53	0	0	P63104	YWHAZ
1	Z83844.1	0.000677	-2.92	1	0.00267478		
1	Z83851.4	0.01217926	-1.75	1	0.04229949		
1	Z84812.4	5.1826E-10	-4.67	3.0149E-05	2.922E-09		
1	Z97634.3	0.00075883	-2.36	1	0.00298717		
1	ZACN	0.00021868	2.12	1	0.00090025	Q401N2	ZACN
1	ZBBX	0.00024299	-5.13	1	0.00099587	A8MT70	ZBBX
1	ZBED1	1.1489E-25	1.59	6.6834E-21	9.7511E-25	O96006	ZBED1
1	ZBED2	3.7681E-15	1.69	2.1921E-10	2.559E-14	Q9BTP6	ZBED2
1	ZBED5	3.6555E-43	1.59	2.1265E-38	3.9912E-42	Q49AG3	ZBED5
1	ZBED5-AS1	5.1967E-42	-2.64	3.0231E-37	5.5901E-41		
1	ZBED6	8.7907E-13	-3.09	5.1139E-08	5.5238E-12	P86452	ZBED6
1	ZBED6CL	2.9254E-36	1.93	1.7018E-31	2.9302E-35		
1	ZBTB10	1.202E-167	10.57	6.994E-163	2.733E-166	Q96DT7	ZBTB10
1	ZBTB11-AS1	1.8603E-18	2.63	1.0822E-13	1.3748E-17		
1	ZBTB12	5.8579E-35	2.21	3.4078E-30	5.775E-34	Q9Y330	ZBTB12
1	ZBTB18	5.14E-104	4.72	2.99E-99	9.094E-103	Q99592	ZBTB18
1	ZBTB2	7.9017E-90	2.10	4.5967E-85	1.2667E-88	Q8N680	ZBTB2
1	ZBTB20	4.2998E-10	-1.73	2.5014E-05	2.4323E-09	Q9HC78	ZBTB20
1	ZBTB21	2.2313E-44	1.96	1.2981E-39	2.4664E-43	Q9ULJ3	ZBTB21
1	ZBTB24	3.859E-35	1.64	2.2449E-30	3.8107E-34	O43167	ZBTB24

1	ZBTB26	0	-1.77	0	0	Q9HCK0	ZBTB26
1	ZBTB32	5.538E-05	-2.31	1	0.00023806	Q9Y2Y4	ZBTB32
1	ZBTB37	9.4607E-07	-1.54	0.05503664	4.5496E-06	Q5TC79	ZBTB37
1	ZBTB41	0	-2.08	0	0	Q5SVQ8	ZBTB41
1	ZBTB43	2.5061E-73	2.32	1.4579E-68	3.5198E-72	O43298	ZBTB43
1	ZBTB45P1	4.6237E-12	-3.96	2.6898E-07	2.8325E-11		
1	ZBTB45P2	0.00032154	-3.97	1	0.00130387		
1	ZBTB46	3.697E-102	3.34	2.1505E-97	6.452E-101	Q86UZ6	ZBTB46
1	ZBTB48	3.5507E-37	1.78	2.0656E-32	3.5911E-36	P10074	ZBTB48
1	ZBTB7B	0	-2.13	0	0	O15156	ZBTB7B
1	ZBTB8B	1.9409E-05	4.05	1	8.5996E-05	Q8NAP8	ZBTB8B
1	ZC2HC1C	1.5226E-14	1.95	8.8578E-10	1.0132E-13	Q53FD0	ZC2HC1C
1	ZC3H11A	0	-1.71	0	0	O75152	ZC3H11A
1	ZC3H12A	0	-3.95	0	0	Q5D1E8	ZC3H12A
1	ZC3H12B	1.0251E-06	1.74	0.05963656	4.9177E-06	Q5HYM0	ZC3H12B
1	ZC3H14	6.5951E-46	2.93	3.8366E-41	7.4137E-45	Q6PJT7	ZC3H14
1	ZC3H15	1.4842E-27	1.59	8.6339E-23	1.3084E-26	Q8WU90	ZC3H15
1	ZC3H6	5.184E-104	-5.24	3.016E-99	9.169E-103	P61129	ZC3H6
1	ZC3H7B	2.6654E-44	1.50	1.5506E-39	2.9445E-43	Q9UGR2	ZC3H7B
1	ZC3HAV1L	5.031E-101	-12.24	2.9265E-96	8.71E-100	Q96H79	ZC3HAV1L
1	ZC4H2	7.624E-162	20.25	4.435E-157	1.701E-160	Q9NQZ6	ZC4H2
1	ZCCHC18	4.2403E-90	46.60	2.4667E-85	6.8123E-89	P0CG32	ZCCHC18
1	ZCCHC2	1.428E-48	1.88	8.3071E-44	1.6499E-47	Q9C0B9	ZCCHC2
1	ZCCHC24	2.2165E-10	1.75	1.2894E-05	1.2701E-09	Q8N2G6	ZCCHC24
1	ZCCHC7	5.7061E-76	3.11	3.3195E-71	8.1901E-75	Q8N3Z6	ZCCHC7
1	ZCWPW1	1.0119E-16	-1.93	5.8869E-12	7.2143E-16	Q9H0M4	ZCWPW1
1	ZCWPW2	0.00031847	-2.30	1	0.00129185	A0A1B0GU75,	ZCWPW2
1	ZDBF2	2.022E-159	88.95	1.176E-154	4.491E-158	Q9HCK1	ZDBF2
1	ZDHHC1	8.0149E-88	12.14	4.6626E-83	1.2656E-86	Q8WTX9	ZDHHC1
1	ZDHHC11	3.3922E-46	-6.22	1.9734E-41	3.8258E-45	Q9H8X9	ZDHHC11
1	ZDHHC11B	0.00629998	-1.68	1	0.02264691	P0C7U3	ZDHHC11B
1	ZDHHC12	0	-2.05	0	0	Q96GR4	ZDHHC12
1	ZDHHC13	0	-1.96	0	0	Q8IUH4	ZDHHC13
1	ZDHHC14	2.3957E-21	1.54	1.3937E-16	1.8747E-20	Q8IZN3	ZDHHC14
1	ZDHHC19	0.00156543	-5.56	1	0.00598732	Q8WVZ1	ZDHHC19
1	ZDHHC2	1.1928E-50	2.16	6.9391E-46	1.4035E-49	Q9UIJ5	ZDHHC2
1	ZDHHC20	5.2252E-36	1.68	3.0397E-31	5.2157E-35	Q5W0Z9	ZDHHC20
1	ZDHHC22	0.01279942	7.57	1	0.04432897	Q8N966	ZDHHC22
1	ZDHHC23	0	-4.15	0	0	Q8IYP9	ZDHHC23
1	ZDHHC24	0	-2.68	0	0	Q6UX98	ZDHHC24
1	ZDHHC4	0	-1.80	0	0	Q9NPG8	ZDHHC4
1	ZDHHC5	0	-1.90	0	0	Q9C0B5	ZDHHC5
1	ZDHHC7	0	-1.56	0	0	Q9NXF8	ZDHHC7
1	ZDHHC8	2.2917E-42	1.80	1.3332E-37	2.4771E-41	Q9ULC8	ZDHHC8
1	ZDHHC9	0	-2.05	0	0	Q9Y397	ZDHHC9
1	ZEB1	4.734E-252	26.87	2.754E-247	1.296E-250	P37275	ZEB1
1	ZEB2	5.4344E-79	25.98	3.1614E-74	7.9955E-78	O60315	ZEB2

1	ZEB2P1	1.1677E-17	-4.29	6.7928E-13	8.4942E-17		
1	ZER1	0	-1.65	0	0	Q7Z7L7	ZER1
1	ZFAND1	0	-1.69	0	0	Q8TCF1	ZFAND1
1	ZFAND4	7.1928E-39	3.31	4.1843E-34	7.4296E-38	Q86XD8	ZFAND4
1	ZFAS1	1.8092E-63	1.96	1.0525E-58	2.3683E-62		
1	ZFAT	2.2649E-14	-1.51	1.3176E-09	1.4988E-13	Q9P243	ZFAT
1	ZFHX2	8.4485E-46	14.96	4.9148E-41	9.4918E-45	Q9C0A1	ZFHX2
1	ZFHX4	3.7168E-81	3.18	2.1622E-76	5.5669E-80	Q86UP3	ZFHX4
1	ZFHX4-AS1	6.223E-21	3.54	3.6201E-16	4.8288E-20		
1	ZFP1	7.3301E-28	1.68	4.2642E-23	6.5033E-27	Q6P2D0	ZFP1
1	ZFP2	3.6857E-30	5.81	2.1441E-25	3.3969E-29	Q6ZN57	ZFP2
1	ZFP30	1.888E-258	147.04	1.098E-253	5.204E-257	Q9Y2G7	ZFP30
1	ZFP36	0	-3.82	0	0	P26651	ZFP36
1	ZFP36L1	1.201E-30	2.65	6.987E-26	1.1156E-29	Q07352	ZFP36L1
1	ZFP69B	3.3196E-28	2.34	1.9311E-23	2.9655E-27	Q9UJL9	ZFP69B
1	ZFP82	7.339E-202	6.68	4.269E-197	1.861E-200	Q8N141	ZFP82
1	ZFPM2	6.66E-195	-4.56	3.875E-190	1.653E-193	Q8WW38	ZFPM2
1	ZFPM2-AS1	1.0081E-93	-4.50	5.8646E-89	1.6628E-92		
1	ZFYVE1	1.3735E-45	1.84	7.99E-41	1.5398E-44	Q9HBF4	ZFYVE1
1	ZFYVE26	2.618E-70	1.94	1.523E-65	3.5997E-69	Q68DK2	ZFYVE26
1	ZFYVE28	9.1022E-28	5.38	5.2951E-23	8.0607E-27	Q9HCC9	ZFYVE28
1	ZGPAT	2.3196E-43	1.69	1.3494E-38	2.5407E-42	Q8N5A5	ZGPAT
1	ZHX1	0	-1.61	0	0	Q9UKY1	ZHX1
1	ZHX2	2.9047E-25	1.83	1.6898E-20	2.4497E-24	Q9Y6X8	ZHX2
1	ZHX3	1.2053E-73	2.09	7.0116E-69	1.6973E-72	Q9H4I2	ZHX3
1	ZIC1	1.2154E-12	-33.10	7.0707E-08	7.5988E-12	Q15915	ZIC1
1	ZIC2	6.4529E-31	1.91	3.7539E-26	6.0207E-30	O95409	ZIC2
1	ZIC4	0.00163247	-8.47	1	0.00623269	Q8N9L1	ZIC4
1	ZIK1	2.793E-101	63.10	1.6249E-96	4.846E-100	Q3SY52	ZIK1
1	ZKSCAN1	0	-1.99	0	0	P17029	ZKSCAN1
1	ZKSCAN2	4.8518E-78	2.80	2.8225E-73	7.0721E-77	Q63HK3	ZKSCAN2
1	ZKSCAN7	3.4381E-72	-6.48	2.0001E-67	4.7918E-71	Q9P0L1	ZKSCAN7
1	ZMAT1	0.01230206	-7.01	1	0.04268774	Q5H9K5	ZMAT1
1	ZMAT3	9.6144E-11	-1.51	5.5931E-06	5.5925E-10	Q9HA38	ZMAT3
1	ZMAT5	2.8675E-30	1.87	1.6681E-25	2.6474E-29	Q9UDW3	ZMAT5
1	ZMIZ1	0	-2.93	0	0	Q9ULJ6	ZMIZ1
1	ZMIZ1-AS1	1.0307E-15	-2.89	5.9958E-11	7.1159E-15		
1	ZMYM2	0	-2.51	0	0	Q9UBW7	ZMYM2
1	ZMYND10	4.751E-07	3.26	0.02763866	2.3216E-06	O75800	ZMYND10
1	ZMYND12	0.00117481	-3.70	1	0.00454775	Q9H0C1	ZMYND12
1	ZMYND8	2.0592E-51	1.58	1.1979E-46	2.4412E-50	Q9ULU4	ZMYND8
1	ZNF10	9.915E-167	5.71	5.768E-162	2.246E-165	P21506	ZNF10
1	ZNF100	2.858E-114	6.55	1.662E-109	5.358E-113	Q8IYN0	ZNF100
1	ZNF101	1.996E-67	2.31	1.1612E-62	2.6879E-66	Q8IZC7	ZNF101
1	ZNF107	2.8078E-36	1.95	1.6334E-31	2.8133E-35	Q9UII5	ZNF107
1	ZNF114	3.704E-181	-8.86	2.155E-176	8.766E-180	Q8NC26	ZNF114
1	ZNF12	0	-2.12	0	0	P17014	ZNF12

1	ZNF121	1.374E-22	2.75	7.9929E-18	1.1026E-21	P58317	ZNF121
1	ZNF134	1.0504E-27	1.92	6.1108E-23	9.2926E-27	P52741	ZNF134
1	ZNF136	1.4506E-18	2.04	8.4389E-14	1.0746E-17	P52737	ZNF136
1	ZNF137P	4.608E-101	14.67	2.6808E-96	7.981E-100	P52743	ZNF137P
1	ZNF14	1.575E-212	13.56	9.163E-208	4.091E-211	P17017	ZNF14
1	ZNF140	1.537E-180	15.02	8.94E-176	3.63E-179	P52738	ZNF140
1	ZNF142	3.1008E-28	1.67	1.8039E-23	2.7705E-27	P52746	ZNF142
1	ZNF146	9.344E-107	2.13	5.436E-102	1.677E-105	Q15072	ZNF146
1	ZNF165	1.8886E-31	-2.44	1.0987E-26	1.7746E-30	P49910	ZNF165
1	ZNF174	1.9818E-32	1.88	1.1529E-27	1.8897E-31	Q15697	ZNF174
1	ZNF175	5.96E-168	6.48	3.467E-163	1.356E-166	Q9Y473	ZNF175
1	ZNF177	3.0176E-30	36.80	1.7555E-25	2.7842E-29	Q13360	ZNF177
1	ZNF180	1.5514E-33	1.85	9.0249E-29	1.5034E-32	Q9UJW8	ZNF180
1	ZNF181	8.3591E-16	1.71	4.8628E-11	5.796E-15	Q2M3W8	ZNF181
1	ZNF184	2.3654E-24	1.75	1.376E-19	1.961E-23	Q99676	ZNF184
1	ZNF185	0	-1.84	0	0	O15231	ZNF185
1	ZNF192P1	7.6665E-16	1.82	4.4599E-11	5.3196E-15		
1	ZNF195	3.2761E-83	2.16	1.9058E-78	4.9852E-82	O14628	ZNF195
1	ZNF20	0.00024793	2.09	1	0.00101527	P17024	ZNF20
1	ZNF202	5.3714E-49	1.86	3.1247E-44	6.2283E-48	O95125	ZNF202
1	ZNF204P	1.3881E-13	-1.82	8.0751E-09	8.9664E-13		
1	ZNF205	9.0901E-39	1.89	5.288E-34	9.3677E-38	O95201	ZNF205
1	ZNF208	4.33E-224	254.40	2.519E-219	1.152E-222	O43345	ZNF208
1	ZNF212	2.5613E-27	1.58	1.49E-22	2.2477E-26	Q9UDV6	ZNF212
1	ZNF22	2.378E-120	3.63	1.383E-115	4.606E-119	P17026	ZNF22
1	ZNF221	0.00405204	-1.52	1	0.01489659	Q9UK13	ZNF221
1	ZNF223	3.847E-10	-1.86	2.238E-05	2.1817E-09	Q9UK11	ZNF223
1	ZNF229	8.5491E-08	-9.71	0.00497333	4.3431E-07	Q9UJW7	ZNF229
1	ZNF232	5.1754E-40	2.21	3.0107E-35	5.4169E-39	Q9UNY5	ZNF232
1	ZNF239	5.619E-139	34.97	3.269E-134	1.173E-137	Q16600	ZNF239
1	ZNF248	8.0164E-43	1.87	4.6635E-38	8.7151E-42	Q8NDW4	ZNF248
1	ZNF25	5.7794E-58	3.37	3.3621E-53	7.2552E-57	P17030	ZNF25
1	ZNF252P-AS1	0.00037193	-1.66	1	0.0015013		
1	ZNF253	1.842E-116	214.26	1.072E-111	3.499E-115	O75346	ZNF253
1	ZNF254	8.3207E-91	3.13	4.8405E-86	1.3423E-89	O75437	ZNF254
1	ZNF256	1.8124E-07	1.89	0.01054361	9.0519E-07	Q9Y2P7	ZNF256
1	ZNF257	8.694E-167	207.23	5.058E-162	1.972E-165	Q9Y2Q1	ZNF257
1	ZNF260	3.9859E-20	1.55	2.3187E-15	3.049E-19	Q3ZCT1	ZNF260
1	ZNF267	2.4599E-40	2.02	1.431E-35	2.584E-39	Q14586	ZNF267
1	ZNF268	3.661E-259	141.56	2.13E-254	1.01E-257	Q14587	ZNF268
1	ZNF271P	1.3732E-17	1.66	7.9885E-13	9.9756E-17		
1	ZNF273	0	-2.42	0	0	Q14593	ZNF273
1	ZNF275	7.9726E-92	2.60	4.638E-87	1.2966E-90	Q9NSD4	ZNF275
1	ZNF28	6.946E-204	9.16	4.041E-199	1.769E-202	P17035	ZNF28
1	ZNF280B	8.8856E-24	-8.66	5.1691E-19	7.2815E-23	Q86YH2	ZNF280B
1	ZNF282	8.2949E-33	1.51	4.8255E-28	7.9576E-32	Q9UDV7	ZNF282
1	ZNF284	7.699E-06	1.52	0.44788094	3.5029E-05	Q2VY69	ZNF284

1	ZNF285	6.144E-05	1.63	1	0.00026337	Q96NJ3	ZNF285
1	ZNF285B	3.6498E-07	3.27	0.02123258	1.7937E-06		
1	ZNF286B	1.465E-130	7.05	8.522E-126	2.959E-129	P0CG31	ZNF286B
1	ZNF287	1.0168E-25	-11.77	5.9152E-21	8.6417E-25	Q9HBT7	ZNF287
1	ZNF30	1.544E-205	17.89	8.984E-201	3.951E-204	P17039	ZNF30
1	ZNF300P1	0.0017631	-6.37	1	0.00670853		
1	ZNF316	0	-1.71	0	0	A6NFI3	ZNF316
1	ZNF317	1.3169E-40	1.53	7.661E-36	1.3871E-39	Q96PQ6	ZNF317
1	ZNF32	3.204E-172	4.57	1.864E-167	7.375E-171	P17041	ZNF32
1	ZNF32-AS2	1.1994E-09	6.00	6.9776E-05	6.6561E-09		
1	ZNF32-AS3	2.3812E-09	7.60	0.00013852	1.3057E-08		
1	ZNF320	5.5316E-88	31.40	3.218E-83	8.7445E-87	A2RRD8	ZNF320
1	ZNF326	4.6568E-36	1.62	2.709E-31	4.6523E-35	Q5BKZ1	ZNF326
1	ZNF331	3.1202E-34	3.78	1.8151E-29	3.0532E-33	Q9NQX6	ZNF331
1	ZNF335	3.5605E-30	1.65	2.0713E-25	3.2825E-29	Q9H4Z2	ZNF335
1	ZNF337-AS1	1.3241E-65	-5.18	7.7027E-61	1.7634E-64		
1	ZNF33A	7.1995E-67	2.03	4.1882E-62	9.6569E-66	Q06730	ZNF33A
1	ZNF34	5.1082E-09	1.55	0.00029717	2.7577E-08	Q8IZ26	ZNF34
1	ZNF341-AS1	0.0002452	-2.66	1	0.00100479		
1	ZNF343	3.0572E-36	1.72	1.7785E-31	3.0616E-35	Q6P1L6	ZNF343
1	ZNF346	7.9662E-28	1.62	4.6342E-23	7.059E-27	Q9UL40	ZNF346
1	ZNF347	3.602E-166	137.89	2.095E-161	8.135E-165	Q96SE7	ZNF347
1	ZNF35	2.3904E-20	1.61	1.3906E-15	1.8331E-19	P13682	ZNF35
1	ZNF350	5.022E-158	65.53	2.921E-153	1.109E-156	Q9GZX5	ZNF350
1	ZNF350-AS1	1.1893E-17	144.05	6.9188E-13	8.6507E-17		
1	ZNF354A	1.6185E-74	7.22	9.4155E-70	2.2965E-73	O60765	ZNF354A
1	ZNF354B	9.5547E-74	2.55	5.5584E-69	1.3472E-72	Q96LW1	ZNF354B
1	ZNF358	9.162E-41	1.97	5.3299E-36	9.6749E-40	Q9NW07	ZNF358
1	ZNF365	1.4843E-31	6.10	8.635E-27	1.3957E-30	Q70YC5	ZNF365
1	ZNF367	5.2064E-22	1.50	3.0288E-17	4.1247E-21	Q7RTV3	ZNF367
1	ZNF37A	2.7064E-56	3.45	1.5744E-51	3.3563E-55	P17032	ZNF37A
1	ZNF37BP	1.4519E-27	2.16	8.446E-23	1.2803E-26		
1	ZNF382	6.2441E-15	9.42	3.6324E-10	4.2042E-14	Q96SR6	ZNF382
1	ZNF383	1.2528E-17	1.87	7.2878E-13	9.1086E-17	Q8NA42	ZNF383
1	ZNF385A	0	-10.92	0	0	Q96PM9	ZNF385A
1	ZNF385B	5.943E-26	-6.83	3.4573E-21	5.0679E-25	Q569K4	ZNF385B
1	ZNF385C	1.1899E-23	-14.08	6.9224E-19	9.7279E-23	Q66K41	ZNF385C
1	ZNF385D	6.2907E-39	-8.80	3.6596E-34	6.5001E-38	Q9H6B1	ZNF385D
1	ZNF395	0	-2.14	0	0	Q9H8N7	ZNF395
1	ZNF396	0.00221979	-1.64	1	0.00835746	Q96N95	ZNF396
1	ZNF408	7.0277E-16	1.50	4.0883E-11	4.881E-15	Q9H9D4	ZNF408
1	ZNF41	0	-1.85	0	0	P51814	ZNF41
1	ZNF410	7.201E-139	2.19	4.189E-134	1.502E-137	Q86VK4	ZNF410
1	ZNF415	3.8268E-15	24.29	2.2262E-10	2.5974E-14	Q09FC8	ZNF415
1	ZNF418	3.2673E-08	10.84	0.00190073	1.6978E-07	Q8TF45	ZNF418
1	ZNF420	4.7782E-16	1.77	2.7797E-11	3.3406E-15	Q8TAQ5	ZNF420
1	ZNF426	1.8005E-64	2.31	1.0474E-59	2.3757E-63	Q9BUY5	ZNF426

1	ZNF428	4.7181E-26	1.79	2.7447E-21	4.0328E-25	Q96B54	ZNF428
1	ZNF429	2.188E-205	102.23	1.273E-200	5.595E-204	Q86V71	ZNF429
1	ZNF43	4.268E-178	71.15	2.483E-173	1.001E-176	P17038	ZNF43
1	ZNF430	3.0787E-11	1.61	1.791E-06	1.8265E-10	Q9H8G1	ZNF430
1	ZNF431	5.6687E-11	1.53	3.2977E-06	3.3243E-10	Q8TF32	ZNF431
1	ZNF432	9.6812E-43	1.99	5.6319E-38	1.0513E-41	O94892	ZNF432
1	ZNF433	3.4213E-13	2.41	1.9903E-08	2.1783E-12	Q8N7K0	ZNF433
1	ZNF438	6.44E-140	6.17	3.747E-135	1.349E-138	Q7Z4V0	ZNF438
1	ZNF439	2.329E-131	46.32	1.355E-126	4.712E-130	Q8NDP4	ZNF439
1	ZNF44	3.963E-110	3.02	2.306E-105	7.269E-109	P15621	ZNF44
1	ZNF441	1.0632E-51	2.74	6.1849E-47	1.2625E-50	Q8N8Z8	ZNF441
1	ZNF442	1.7593E-34	3.75	1.0235E-29	1.7274E-33	Q9H7R0	ZNF442
1	ZNF443	1.8899E-17	1.86	1.0994E-12	1.3684E-16	Q9Y2A4	ZNF443
1	ZNF462	2.7802E-27	1.80	1.6174E-22	2.4355E-26	Q96JM2	ZNF462
1	ZNF467	1.9153E-08	-3.24	0.00111421	1.0081E-07	Q7Z7K2	ZNF467
1	ZNF468	6.3936E-55	2.78	3.7194E-50	7.8435E-54	Q5VIY5	ZNF468
1	ZNF48	3.8716E-37	1.84	2.2523E-32	3.9136E-36	Q96MX3	ZNF48
1	ZNF480	1.1744E-50	2.19	6.8319E-46	1.3821E-49	Q8WV37	ZNF480
1	ZNF485	2.044E-178	3.70	1.189E-173	4.804E-177	Q8NCK3	ZNF485
1	ZNF486	5.6665E-16	106.90	3.2964E-11	3.9521E-15	Q96H40	ZNF486
1	ZNF487	3.741E-11	1.74	2.1763E-06	2.2099E-10	B1APH4	ZNF487
1	ZNF488	5.4146E-25	-1.93	3.1499E-20	4.542E-24	Q96MN9	ZNF488
1	ZNF490	4.1038E-09	1.73	0.00023874	2.2243E-08	Q9ULM2	ZNF490
1	ZNF491	1.3146E-27	3.55	7.6476E-23	1.1603E-26	Q8N8L2	ZNF491
1	ZNF492	5.598E-173	289.74	3.257E-168	1.292E-171	Q9P255	ZNF492
1	ZNF493	9.448E-242	107.63	5.496E-237	2.566E-240	Q6ZR52	ZNF493
1	ZNF496	0	-1.58	0	0	Q96IT1	ZNF496
1	ZNF497	9.6409E-05	-1.57	1	0.00040777	Q6ZNH5	ZNF497
1	ZNF501	1.0944E-12	-7.57	6.3665E-08	6.8538E-12	Q96CX3	ZNF501
1	ZNF503	9.2681E-20	1.59	5.3916E-15	7.0396E-19	Q96F45	ZNF503
1	ZNF503-AS1	5.2502E-05	-8.46	1	0.00022611		
1	ZNF510	7.8569E-43	1.78	4.5707E-38	8.5449E-42	Q9Y2H8	ZNF510
1	ZNF511-PRAP	0.00074915	-2.23	1	0.00295066		
1	ZNF512	0	-1.76	0	0	Q96ME7	ZNF512
1	ZNF512B	1.133E-110	2.25	6.59E-106	2.084E-109	Q96KM6	ZNF512B
1	ZNF516	7.1317E-63	10.18	4.1488E-58	9.2939E-62	Q92618	ZNF516
1	ZNF518A	0	-3.41	0	0	Q6AHZ1	ZNF518A
1	ZNF518B	1.6581E-97	19.89	9.6461E-93	2.8041E-96	Q9C0D4	ZNF518B
1	ZNF527	1.2642E-97	3.47	7.3542E-93	2.1391E-96	Q8NB42	ZNF527
1	ZNF528	1.237E-209	212.19	7.194E-205	3.192E-208	Q3MIS6	ZNF528
1	ZNF528-AS1	1.7687E-91	151.45	1.0289E-86	2.8701E-90		
1	ZNF529	3.2481E-17	1.79	1.8896E-12	2.3389E-16	Q6P280	ZNF529
1	ZNF530	2.74E-09	-1.52	0.0001594	1.4984E-08	Q6P9A1	ZNF530
1	ZNF532	1.379E-117	2.79	8.021E-113	2.634E-116	Q9HCE3	ZNF532
1	ZNF540	3.4165E-63	7.34	1.9875E-58	4.4624E-62	Q8NDQ6	ZNF540
1	ZNF546	1.1952E-06	-1.60	0.06952681	5.7163E-06	Q86UE3	ZNF546
1	ZNF548	2.833E-16	-1.64	1.648E-11	1.995E-15	Q8NEK5	ZNF548

1	ZNF549	1.2956E-74	4.63	7.5373E-70	1.8388E-73	Q6P9A3	ZNF549
1	ZNF551	1.2674E-26	2.02	7.3732E-22	1.0951E-25	Q7Z340	ZNF551
1	ZNF555	1.9103E-40	2.83	1.1113E-35	2.0096E-39	Q8NEP9	ZNF555
1	ZNF557	2.3634E-41	2.20	1.3749E-36	2.5172E-40	Q8N988	ZNF557
1	ZNF559	1.693E-203	11.67	9.847E-199	4.306E-202	Q9BR84	ZNF559
1	ZNF559-ZNF1	2.1219E-14	33.22	1.2344E-09	1.4056E-13	A0A0A6YYI6	ZNF559-ZNF1
1	ZNF561	5.292E-105	2.06	3.079E-100	9.406E-104	Q8N587	ZNF561
1	ZNF562	3.248E-51	2.81	1.8895E-46	3.8381E-50	Q6V9R5	ZNF562
1	ZNF563	1.6879E-19	2.85	9.8194E-15	1.2744E-18	Q8TA94	ZNF563
1	ZNF565	2.4589E-24	2.26	1.4304E-19	2.0368E-23	Q8N9K5	ZNF565
1	ZNF566	2.1358E-20	1.98	1.2425E-15	1.64E-19	Q969W8	ZNF566
1	ZNF568	7.4692E-49	44.15	4.3451E-44	8.6487E-48	Q3ZCX4	ZNF568
1	ZNF569	2.87E-205	5.85	1.669E-200	7.331E-204	Q5MCW4	ZNF569
1	ZNF57	6.8513E-23	1.82	3.9857E-18	5.525E-22	Q68EA5	ZNF57
1	ZNF570	5.935E-138	4.45	3.453E-133	1.233E-136	Q96NI8	ZNF570
1	ZNF571	4.7996E-52	3.21	2.7921E-47	5.7274E-51	Q7Z3V5	ZNF571
1	ZNF571-AS1	1.6572E-10	6.64	9.6409E-06	9.552E-10		
1	ZNF572	2.3774E-23	-2.99	1.383E-18	1.9332E-22	Q7Z3I7	ZNF572
1	ZNF573	1.582E-16	1.89	9.2029E-12	1.1226E-15	Q86YE8	ZNF573
1	ZNF577	1.473E-170	206.51	8.568E-166	3.374E-169	Q9BSK1	ZNF577
1	ZNF578	3.0269E-10	3.86	1.7609E-05	1.7238E-09	Q96N58	ZNF578
1	ZNF579	3.1225E-38	2.49	1.8165E-33	3.1986E-37	Q8NAF0	ZNF579
1	ZNF580	7.2143E-30	1.72	4.1968E-25	6.6238E-29	Q9UK33	ZNF580
1	ZNF581	7.7944E-52	1.84	4.5343E-47	9.2745E-51	Q9P0T4	ZNF581
1	ZNF585A	7.1283E-10	-2.93	4.1468E-05	3.9954E-09	Q6P3V2	ZNF585A
1	ZNF585B	1.656E-108	3.63	9.631E-104	3.002E-107	Q52M93	ZNF585B
1	ZNF589	1.4624E-43	1.66	8.5075E-39	1.6064E-42	Q86UQ0	ZNF589
1	ZNF593	3.5869E-80	2.95	2.0866E-75	5.3339E-79	O00488	ZNF593
1	ZNF594	2.7798E-28	-1.97	1.6171E-23	2.4856E-27	Q96JF6	ZNF594
1	ZNF595	0	-2.35	0	0	Q8IYB9	ZNF595
1	ZNF597	8.8853E-64	-5.37	5.1689E-59	1.1663E-62	Q96LX8	ZNF597
1	ZNF598	4.4425E-58	2.02	2.5844E-53	5.5819E-57	Q86UK7	ZNF598
1	ZNF599	2.9561E-83	16.25	1.7197E-78	4.4994E-82	Q96NL3	ZNF599
1	ZNF600	5.011E-103	2.69	2.9148E-98	8.801E-102	Q6ZNG1	ZNF600
1	ZNF602P	0.00028029	4.12	1	0.00114178		
1	ZNF605	5.1279E-48	-2.36	2.9831E-43	5.8967E-47	Q86T29	ZNF605
1	ZNF606	6.603E-128	-12.89	3.841E-123	1.323E-126	Q8WXB4	ZNF606
1	ZNF607	6.003E-74	3.99	3.4922E-69	8.4741E-73	Q96SK3	ZNF607
1	ZNF608	5.228E-102	-10.90	3.0414E-97	9.12E-101	Q9ULD9	ZNF608
1	ZNF610	7.5067E-07	7.70	0.04366934	3.6291E-06	Q8N9Z0	ZNF610
1	ZNF611	1.421E-72	2.60	8.2668E-68	1.9848E-71	Q8N823	ZNF611
1	ZNF613	4.461E-160	154.68	2.595E-155	9.924E-159	Q6PF04	ZNF613
1	ZNF614	8.5855E-52	2.91	4.9945E-47	1.0203E-50	Q8N883	ZNF614
1	ZNF615	2.6434E-30	2.23	1.5378E-25	2.4413E-29	Q8N8J6	ZNF615
1	ZNF620	1.0671E-15	1.78	6.2078E-11	7.3631E-15	Q6ZNG0	ZNF620
1	ZNF625	6.35E-106	31.81	3.694E-101	1.133E-104	Q96I27	ZNF625
1	ZNF625-ZNF2	1.3487E-18	2.37	7.8458E-14	9.9959E-18		

1	ZNF626	1.125E-148	681.68	6.545E-144	2.414E-147	Q68DY1	ZNF626
1	ZNF627	4.6019E-26	1.90	2.6771E-21	3.9352E-25	Q7L945	ZNF627
1	ZNF628	2.3189E-10	1.52	1.349E-05	1.3274E-09	Q5EBL2	ZNF628
1	ZNF630	5.1282E-35	-5.51	2.9833E-30	5.0581E-34	Q2M218	ZNF630
1	ZNF641	0	-2.15	0	0	Q96N77	ZNF641
1	ZNF644	2.5227E-30	1.59	1.4676E-25	2.331E-29	Q9H582	ZNF644
1	ZNF649	5.848E-95	67.13	3.402E-90	9.7284E-94	Q9BS31	ZNF649
1	ZNF658	5.7293E-28	2.22	3.3329E-23	5.1001E-27	Q5TYW1	ZNF658
1	ZNF658B	9.9412E-30	3.63	5.7832E-25	9.106E-29	Q4V348	ZNF658B
1	ZNF66	1.382E-104	360.11	8.038E-100	2.451E-103	Q6ZN08	ZNF66
1	ZNF660	1.3503E-06	-9.19	0.07855523	6.4374E-06	Q6AZW8	ZNF660
1	ZNF662	3.8969E-39	-14.73	2.267E-34	4.0395E-38	Q6ZS27	ZNF662
1	ZNF665	2.862E-152	58.05	1.665E-147	6.199E-151	Q9H7R5	ZNF665
1	ZNF668	9.8424E-28	1.90	5.7257E-23	8.7136E-27	Q96K58	ZNF668
1	ZNF670-ZNF6!	1.9196E-10	-2.14	1.1167E-05	1.103E-09	F2Z2N8	ZNF670-ZNF6!
1	ZNF671	5.3042E-41	11.47	3.0857E-36	5.6154E-40	Q8TAW3	ZNF671
1	ZNF674	4.1686E-09	-1.76	0.0002425	2.2586E-08	Q2M3X9	ZNF674
1	ZNF674-AS1	2.9441E-15	-1.91	1.7127E-10	2.0067E-14		
1	ZNF675	3.4977E-56	3.60	2.0348E-51	4.3321E-55	Q8TD23	ZNF675
1	ZNF676	2.418E-176	184.85	1.407E-171	5.647E-175	Q8N7Q3	ZNF676
1	ZNF677	7.6718E-17	29.13	4.463E-12	5.4842E-16	Q86XU0	ZNF677
1	ZNF679	2.0944E-08	24.80	0.0012184	1.1001E-07	Q8IYX0	ZNF679
1	ZNF681	2.3887E-93	4.02	1.3896E-88	3.9265E-92	Q96N22	ZNF681
1	ZNF682	4.096E-220	195.56	2.383E-215	1.082E-218	O95780	ZNF682
1	ZNF684	6.4834E-09	1.56	0.00037717	3.4826E-08	Q5T5D7	ZNF684
1	ZNF687	0	-1.63	0	0	Q8N1G0	ZNF687
1	ZNF688	1.9961E-10	1.52	1.1612E-05	1.146E-09	P0C7X2	ZNF688
1	ZNF689	8.3916E-93	3.30	4.8817E-88	1.3748E-91	Q96CS4	ZNF689
1	ZNF69	2.8563E-15	1.69	1.6616E-10	1.9477E-14	Q9UC07	ZNF69
1	ZNF692	0	-1.70	0	0	Q9BU19	ZNF692
1	ZNF697	2.1391E-98	3.03	1.2444E-93	3.6396E-97	Q5TEC3	ZNF697
1	ZNF699	2.475E-183	6.84	1.44E-178	5.89E-182	Q32M78	ZNF699
1	ZNF70	3.4704E-69	2.77	2.0189E-64	4.7236E-68	Q9UC06	ZNF70
1	ZNF701	9.888E-169	4.30	5.752E-164	2.257E-167	M0R085, Q9N	ZNF701
1	ZNF702P	9.145E-103	76.12	5.32E-98	1.603E-101	Q9H963	ZNF702P
1	ZNF703	4.9541E-65	2.22	2.882E-60	6.5635E-64	Q9H7S9	ZNF703
1	ZNF705E	1.8715E-06	3.90	0.10887411	8.8508E-06	A8MWA4	ZNF705E
1	ZNF706	0	-1.56	0	0	Q9Y5V0	ZNF706
1	ZNF708	5.6834E-36	2.41	3.3062E-31	5.6701E-35	P17019	ZNF708
1	ZNF709	3.5936E-10	13.08	2.0905E-05	2.0407E-09	Q8N972	ZNF709
1	ZNF71	2.4121E-40	4.18	1.4032E-35	2.5343E-39	Q9NQZ8	ZNF71
1	ZNF711	2.122E-53	-12.91	1.2345E-48	2.5686E-52	Q9Y462	ZNF711
1	ZNF713	8.1366E-13	2.35	4.7334E-08	5.1177E-12	Q8N859	ZNF713
1	ZNF714	1.1385E-39	1.88	6.6233E-35	1.1861E-38	Q96N38	ZNF714
1	ZNF716	5.1579E-25	297.83	3.0005E-20	4.3298E-24	A6NP11	ZNF716
1	ZNF718	6.3557E-86	-2.71	3.6974E-81	9.8913E-85	Q3SXZ3	ZNF718
1	ZNF724	1.806E-190	5.53	1.05E-185	4.408E-189	A8MTY0	ZNF724

1	ZNF726	3.418E-215	6.67	1.988E-210	8.937E-214	A6NNF4	ZNF726
1	ZNF727	1.9087E-62	472.35	1.1104E-57	2.4791E-61	A8MUV8	ZNF727
1	ZNF730	2.44E-222	72.38	1.419E-217	6.477E-221	Q6ZMV8	ZNF730
1	ZNF737	1.778E-215	228.82	1.034E-210	4.652E-214	O75373	ZNF737
1	ZNF738	8.1358E-54	4.77	4.7329E-49	9.8953E-53	Q8NE65	ZNF738
1	ZNF74	1.7971E-92	2.48	1.0455E-87	2.9367E-91	Q16587	ZNF74
1	ZNF746	2.089E-116	2.13	1.215E-111	3.965E-115	Q6NUN9	ZNF746
1	ZNF747	7.4134E-21	1.88	4.3127E-16	5.7464E-20	Q9BV97	ZNF747
1	ZNF749	4.2062E-14	1.57	2.4469E-09	2.7614E-13	O43361	ZNF749
1	ZNF750	3.24E-180	-112.32	1.885E-175	7.642E-179	Q32MQ0	ZNF750
1	ZNF76	0	-2.15	0	0	P36508	ZNF76
1	ZNF761	1.0756E-09	1.55	6.2574E-05	5.9851E-09	Q86XN6	ZNF761
1	ZNF764	3.1657E-26	2.02	1.8416E-21	2.7162E-25	Q96H86	ZNF764
1	ZNF77	8.6663E-17	1.92	5.0415E-12	6.1867E-16	Q15935	ZNF77
1	ZNF771	1.3777E-54	2.73	8.0144E-50	1.6869E-53	Q7L3S4	ZNF771
1	ZNF772	2.588E-156	23.86	1.506E-151	5.689E-155	Q68DY9	ZNF772
1	ZNF773	7.5394E-15	1.91	4.386E-10	5.0652E-14	Q6PK81	ZNF773
1	ZNF774	1.4236E-12	-1.80	8.2818E-08	8.8765E-12	Q6NX45	ZNF774
1	ZNF775	2.1482E-27	2.98	1.2497E-22	1.8877E-26	Q96BV0	ZNF775
1	ZNF777	4.7706E-87	2.11	2.7753E-82	7.4845E-86	Q9ULD5	ZNF777
1	ZNF778	3.1957E-33	1.63	1.859E-28	3.0789E-32	Q96MU6	ZNF778
1	ZNF783	3.1583E-59	2.17	1.8373E-54	4.002E-58	Q6ZMS7	ZNF783
1	ZNF785	8.5219E-13	1.52	4.9575E-08	5.3572E-12	A8K8V0	ZNF785
1	ZNF786	2.6484E-17	1.73	1.5407E-12	1.9115E-16	Q8N393	ZNF786
1	ZNF788	3.822E-178	63.80	2.223E-173	8.969E-177	Q6ZQV5	ZNF788
1	ZNF789	0	-1.87	0	0	Q5FWF6	ZNF789
1	ZNF790	5.3309E-29	2.69	3.1012E-24	4.8223E-28	Q6PG37	ZNF790
1	ZNF790-AS1	4.0011E-08	2.52	0.00232757	2.0688E-07		
1	ZNF792	2.207E-121	38.37	1.284E-116	4.303E-120	Q3KQV3	ZNF792
1	ZNF793	1.423E-180	61.48	8.281E-176	3.364E-179	Q6ZN11	ZNF793
1	ZNF793-AS1	4.2884E-30	77.64	2.4947E-25	3.9486E-29		
1	ZNF799	9.1891E-25	2.23	5.3457E-20	7.6828E-24	Q96GE5	ZNF799
1	ZNF804A	9.509E-207	8.05	5.531E-202	2.439E-205	Q7Z570	ZNF804A
1	ZNF804B	5.1658E-32	-24.39	3.0051E-27	4.8936E-31	A4D1E1	ZNF804B
1	ZNF808	5.1731E-21	1.87	3.0094E-16	4.0206E-20	Q8N4W9	ZNF808
1	ZNF813	7.9432E-84	3.78	4.6209E-79	1.216E-82	Q6ZN06	ZNF813
1	ZNF816	1.3958E-34	2.23	8.1197E-30	1.372E-33	Q0VGE8	ZNF816
1	ZNF823	4.0269E-17	1.68	2.3426E-12	2.8932E-16	P16415	ZNF823
1	ZNF826P	4.915E-236	181.42	2.859E-231	1.326E-234	Q6ZT77	ZNF826P
1	ZNF829	2.627E-44	43.67	1.5282E-39	2.9026E-43	Q3KNS6	ZNF829
1	ZNF83	2.667E-154	12.51	1.552E-149	5.818E-153	P51522	ZNF83
1	ZNF833P	0.01090593	3.10	1	0.03810461	Q6ZTB9	ZNF833P
1	ZNF837	6.6691E-19	-2.88	3.8797E-14	4.9772E-18	Q96EG3	ZNF837
1	ZNF839	2.4276E-43	1.91	1.4122E-38	2.658E-42	A8K0R7	ZNF839
1	ZNF844	9.092E-159	7.49	5.289E-154	2.014E-157	Q08AG5	ZNF844
1	ZNF847P	0.00367311	3.19	1	0.01354373		
1	ZNF85	1.971E-251	110.02	1.147E-246	5.393E-250	Q03923	ZNF85

1	ZNF850	1.1843E-53	2.65	6.8893E-49	1.4377E-52	A8MQ14	ZNF850
1	ZNF853	2.9221E-38	11.29	1.6999E-33	2.9959E-37	P0CG23	ZNF853
1	ZNF862	3.7038E-28	-1.94	2.1547E-23	3.3062E-27	O60290	ZNF862
1	ZNF878	2.0931E-18	4.70	1.2176E-13	1.5446E-17	C9JN71	ZNF878
1	ZNF883	2.0936E-10	2.99	1.2179E-05	1.2012E-09	P0CG24	ZNF883
1	ZNF887P	5.3068E-12	2.32	3.0872E-07	3.2435E-11		
1	ZNF888	2.6435E-23	1.74	1.5378E-18	2.1466E-22	P0CJ79	ZNF888
1	ZNF890P	0.00514173	-3.51	1	0.01867952		
1	ZNF891	5.3065E-90	4.13	3.087E-85	8.5183E-89	A8MT65	ZNF891
1	ZNF90	1.883E-188	68.86	1.095E-183	4.554E-187	Q03938	ZNF90
1	ZNF91	1.709E-200	7.19	9.943E-196	4.319E-199	Q05481	ZNF91
1	ZNF92	3.5954E-32	2.19	2.0916E-27	3.4148E-31	Q03936	ZNF92
1	ZNF93	1.268E-267	188.76	7.379E-263	3.52E-266	P35789	ZNF93
1	ZNF98	2.98E-99	119.86	1.7338E-94	5.1039E-98	A6NK75	ZNF98
1	ZNF99	3.2301E-93	89.57	1.8791E-88	5.3081E-92	A8MXY4	ZNF99
1	ZNRD1ASP	9.1572E-11	-1.67	5.3271E-06	5.3298E-10		
1	ZNRF2	3.1664E-92	-3.89	1.842E-87	5.1684E-91	Q8NHG8	ZNRF2
1	ZNRF2P2	1.8086E-29	-1.86	1.0521E-24	1.6486E-28		
1	ZPR1	8.2794E-58	2.04	4.8165E-53	1.0374E-56	O75312	ZPR1
1	ZRANB2-AS2	4.2215E-09	-2.54	0.00024558	2.2866E-08		
1	ZSCAN12	1.1714E-38	-2.24	6.8145E-34	1.2059E-37	O43309	ZSCAN12
1	ZSCAN12P1	4.748E-31	-4.88	2.7621E-26	4.4357E-30		
1	ZSCAN16	3.6953E-24	2.17	2.1497E-19	3.0488E-23	Q9H4T2	ZSCAN16
1	ZSCAN2	1.3867E-15	1.55	8.0673E-11	9.5324E-15	Q7Z7L9	ZSCAN2
1	ZSCAN23	5.3403E-35	-14.44	3.1067E-30	5.2655E-34	Q3MJ62	ZSCAN23
1	ZSCAN26	1.5068E-47	1.72	8.7654E-43	1.7231E-46	Q16670	ZSCAN26
1	ZSCAN31	0	-2.55	0	0	Q96LW9	ZSCAN31
1	ZSWIM3	5.6702E-21	1.76	3.2986E-16	4.4022E-20	Q96MP5	ZSWIM3
1	ZSWIM5	2.6814E-10	-1.83	1.5599E-05	1.5306E-09	Q9P217	ZSWIM5
1	ZSWIM5P2	2.1253E-06	-8.42	0.12363931	1.0019E-05		
1	ZSWIM6	2.7421E-41	1.80	1.5952E-36	2.9174E-40	Q9HCJ5	ZSWIM6
1	ZWINT	9.907E-129	2.40	5.763E-124	1.99E-127	O95229	ZWINT
1	ZXDA	8.2959E-12	2.15	4.8261E-07	5.0371E-11	P98168	ZXDA
1	ZXDB	6.9768E-50	2.56	4.0587E-45	8.1598E-49	P98169	ZXDB
1	ZYG11A	3.135E-117	-18.62	1.824E-112	5.977E-116	Q6WRX3	ZYG11A
1	ZYG11B	6.7632E-23	1.51	3.9344E-18	5.4546E-22	Q9C0D3	ZYG11B
1	ZYX	1.092E-148	1.98	6.352E-144	2.344E-147	Q15942	ZYX

GO biological p	GO cellular com	GO molecular f	object name	Synonym
0006397 // m	0005737 // cy	0005515 // pr	APOBEC1 com	A1CF_HUMAN|A1CF|ACF|ASP
0016266 // O-	0016021 // int	0008375 // ac	Alpha-1,4-N-a	A4GCT_HUMAN|A4GNT
0009056 // ca	0016021 // int	0019213 // de	Arylacetamide	AAAD_HUMAN|AADAC|DAC
0009056 // ca	0005576 // ex	0016787 // hy	Arylacetamide	ADCL2_HUMAN|AADACL2
0070189 // ky	0005759 // m	0016212 // ky	Kynurenine/a	AADAT_HUMAN|AADAT|KAT2
0098869 // ce		0016209 // an	Thioredoxin-li	AAED1_HUMAN|AAED1|C9orf21
0051412 // re	0048471 // pe	0004059 // ar	Serotonin N-a	SNAT_HUMAN|AANAT|SNAT
0000244 // sp			Protein AAR2	AAR2_HUMAN|AAR2|C20orf4|CGI-23|PRO0225
0008033 // tR	0005829 // cy	0016597 // an	Alanine--tRNA	SYAC_HUMAN|AARS
0006631 // fa		0005524 // AT	Acyl-CoA synt	ACSF4_HUMAN|AASDH|ACSF4|U26|HSPC318
0032929 // ne	0005634 // nu	0003723 // RN	Protein AATF	AATF_HUMAN|AATF|CHE1|DED|HSPC277
0006468 // pr	0016021 // int	0004674 // pr	Serine/threon	LMTK1_HUMAN|AATK|AATYK|KIAA0641|LMR1|LMTK1
0060155 // pl	0005886 // pl	0005515 // pr	ATP-binding c	ABCA1_HUMAN|ABCA1|ABC1|CERP
0006869 // lip	0016021 // int	0005524 // AT	ATP-binding c	ABCAA_HUMAN|ABCA10
2000010 // po	0005743 // m	0005102 // re	ATP-binding c	ABCAC_HUMAN|ABCA12|ABC12
0006869 // lip	0016021 // int	0005524 // AT	ATP-binding c	ABCAD_HUMAN|ABCA13
0006810 // tra	0016021 // int	0000166 // nu	ATP-binding c	ABCA2_HUMAN|ABCA2|ABC2|KIAA1062
0042493 // re	0097208 // al	0042626 // AT	ATP-binding c	ABCA3_HUMAN|ABCA3|ABC3
0045494 // ph	0043231 // int	0005395 // ey	Retinal-specif	ABCA4_HUMAN|ABCA4|ABCR
0006869 // lip	0005770 // lat	0005319 // lip	ATP-binding c	ABCA5_HUMAN|ABCA5|KIAA1888
0033344 // ch	0005794 // Go	0090554 // ph	ATP-binding c	ABCA7_HUMAN|ABCA7
0042493 // re	0016020 // m	0008559 // xe	Multidrug resi	MDR1_HUMAN|ABCB1|MDR1|PGY1
0055085 // tra	0005743 // m	0042803 // pr	ATP-binding c	ABCBA_HUMAN|ABCB10
0032376 // po	0005886 // pl	0042626 // AT	Phosphatidylc	MDR3_HUMAN|ABCB4|MDR3|PGY3
0015886 // he	0005794 // Go	0005524 // AT	ATP-binding c	ABCB6_HUMAN|ABCB6|MTABC3|PRP|UMAT
0055085 // tra	0005740 // m	0005215 // tra	ATP-binding c	ABCB8_HUMAN|ABCB8|MABC1
0099133 /// 0	0005887 // int	0042626 // AT	Multidrug resi	MRP7_HUMAN|ABCC10|MRP7|SIMRP7
0099133 /// 0	0005887 // int	0008514 // or	ATP-binding c	ABCCB_HUMAN|ABCC11|MRP8
0055085 // tra	0016021 // int	0042626 // AT	Multidrug resi	MRP9_HUMAN|ABCC12|MRP9
0071222 // ce	0016324 // ap	0005515 // pr	Canalicular m	MRP2_HUMAN|ABCC2|CMOAT|CMOAT1|CMRP|MRP2
0099133 /// 0	0005887 // int	0008514 // or	Canalicular m	MRP3_HUMAN|ABCC3|CMOAT2|MLP2|MRP3
0042493 // re	0031088 // pl	0042626 // AT	Multidrug resi	MRP4_HUMAN|ABCC4|MRP4
0099133 /// 0	0005886 // pl	0042626 // AT	Multidrug resi	MRP5_HUMAN|ABCC5|MRP5
0099133 /// 0	0016021 // int	0005215 // tra	Multidrug resi	MRP6_HUMAN|ABCC6|ARA|MRP6

0000038 // ve	0005829 // cy	0005515 // pr	ATP-binding c	ABCD2_HUMAN|ABCD2|ALD1|ALDL1|ALDR|ALDRP
0006810 // tra	0005740 // m	0005215 // tra	ATP-binding c	ABCF2_HUMAN|ABCF2|HUSSY-18
0010872 // re	0055037 // re	0005524 // AT	ATP-binding c	ABCG1_HUMAN|ABCG1|ABC8|WHT1
0006879 // ce	0016021 // int	0042803 // pr	ATP-binding c	ABCG2_HUMAN|ABCG2|ABCP|BCRP|BCRP1|MXR
1990830 // ce	0016021 // int	0017127 // ch	ATP-binding c	ABCG4_HUMAN|ABCG4|WHITE2
0052695 // ce	0005829 // cy	0004553 // hy	Mycophenolic	ABHDA_HUMAN|ABHD10
		0016787 // hy	Protein ABHD	AB12B_HUMAN|ABHD12B|C14orf29
0045944 // po	0005829 // cy	0005515 // pr	Protein ABHD	ABHEB_HUMAN|ABHD14B|CIB
		0016787 // hy	Protein ABHD	ABHGB_HUMAN|ABHD16B|C20orf135
0008150 // bi	0005576 // ex	0016787 // hy	Protein ABHD	AB17A_HUMAN|ABHD17A|C19orf27|FAM108A1
		0016787 // hy	Protein ABHD	AB17C_HUMAN|ABHD17C|FAM108C1
	0005576 // ex		Protein ABHD	ABD18_HUMAN|ABHD18|C4orf29
0006656 // ph	0016021 // int	0102568 /// 0	Phospholipase	ABHD3_HUMAN|ABHD3
0006631 // fa	0005811 // lip	0004806 // tri	1-acylglycerol	ABHD5_HUMAN|ABHD5|NCIE2|CGI-58
0030198 // ex	0005614 // int	0005518 // co	Target of Nesl	TARSH_HUMAN|ABI3BP|NESHBP|TARSH
0006468 // pr	0005634 // nu	0005515 // pr	Tyrosine-prot	ABL1_HUMAN|ABL1|ABL|JTK7
0038083 // pe	0005829 // cy	0005515 // pr	Abelson tyros	ABL2_HUMAN|ABL2|ABLL|ARG
0007010 // cy	0030027 // la	0005515 // pr	Actin-binding	ABLM1_HUMAN|ABLIM1|ABLIM|KIAA0059|LIMAB1
0030036 // ac	0015629 // ac	0003779 // ac	Actin-binding	ABLM2_HUMAN|ABLIM2|KIAA1808
0006351 // tra	0030027 // la	0005515 // pr	Actin-binding	ABLM3_HUMAN|ABLIM3|KIAA0843|HMFN1661
0007264 // sn	0005886 // pl	0005089 // Rh	Active breakp	ABR_HUMAN|ABR
0043161 // pr	0005737 // cy	0005515 // pr	Ankyrin repea	ABTB1_HUMAN|ABTB1|BPOZ|PP2259

0036109 // al	0005782 // pe	0003988 // ac	3-ketoacyl-Co	THIK_HUMAN|ACAA1|ACAA|PTHIO
0006695 // ch	0005739 // m	0003723 // RN	3-ketoacyl-Co	THIM_HUMAN|ACAA2
0071380 // ce	0001650 // fib	0003989 // ac	Acetyl-CoA ca	ACACA_HUMAN|ACACA|ACAC|ACC1|ACCA
0006633 // fa	0012505 // en	0005524 // AT	Acetyl-CoA ca	ACACB_HUMAN|ACACB|ACC2|ACCB
0006574 // va	0005759 // m	0003995 // ac	Isobutyryl-Co/	ACAD8_HUMAN|ACAD8|ARC42|IBD
0001659 // te	0031966 // m	0003995 // ac	Long-chain sp	ACADL_HUMAN|ACADL
0055114 // ox	0005759 // m	0003995 // ac	Medium-chair	ACADM_HUMAN|ACADM
0006631 // fa	0005739 // m	0003995 // ac	Short/branche	ACDSB_HUMAN|ACADSB
1990090 // ce	0016020 // m	0017137 // Ra	Arf-GAP with	ACAP2_HUMAN|ACAP2|CENTB2|KIAA0041
0009725 // re	0005739 // m	0003985 // ac	Acetyl-CoA ac	THIL_HUMAN|ACAT1|ACAT|MAT
0006629 // lip	0005829 // cy	0005515 // pr	Acetyl-CoA ac	THIC_HUMAN|ACAT2|ACTL
0006694 // st	0005794 // G	0005515 // pr	Golgi resident	GCP60_HUMAN|ACBD3|GCP60|GOCAP1|GOLPH1
0009058 // bi		0005515 // pr	1-aminocyclo	1A1L1_HUMAN|ACCS|PHACS
0003081 // re	0005576 // ex	0031711 // br	Angiotensin-c	ACE_HUMAN|ACE|DCP|DCP1
0001817 // re	0009986 // ce	0008270 // zir	Angiotensin-c	ACE2_HUMAN|ACE2|UNQ868/PRO1885
0008284 // po	0030173 // in	0017040 // ce	Alkaline ceran	ACER2_HUMAN|ACER2|ASAH3L|PP11646
0071602 // ph	0030176 // in	0070774 // ph	Alkaline ceran	ACER3_HUMAN|ACER3|APHC|PHCA
0060041 // re	0009986 // ce	0001540 // be	Acetylcholines	ACES_HUMAN|ACHE
0007155 // ce	0005886 // pl	0019958 // C-	Atypical chem	ACKR3_HUMAN|ACKR3|CMKOR1|CXCR7|GPR159|RDC1
0006633 // fa	0070062 // ex	0003878 // AT	ATP-citrate sy	ACLY_HUMAN|ACLY
0006879 // ce	0005737 // cy	0003723 // RN	Cytoplasmic a	ACOC_HUMAN|ACO1|IREB1
0009266 // re	0005829 // cy	0047617 // ac	Acyl-coenzym	ACO11_HUMAN|ACOT11|BFIT|KIAA0707|THEA
0046459 // sh	0005777 // pe	0005102 // re	Acyl-coenzym	ACOT4_HUMAN|ACOT4|PTE2B|PTEIB
0006631 // fa	0005829 // cy	0047617 // ac	Putative acyl-	ACOT6_HUMAN|ACOT6|C14orf42
0015937 // co	0005829 // cy	0005515 // pr	Cytosolic acyl	BACH_HUMAN|ACOT7|BACH
0033540 // fa	0005739 // m	0016402 // pr	Peroxisomal a	ACOX2_HUMAN|ACOX2
0033540 // fa	0005782 // pe	0016402 // pr	Acyl-coenzym	ACOXL_HUMAN|ACOXL
0050830 // de	0005829 // cy	0003993 // ac	Tartrate-resist	PPA5_HUMAN|ACP5
0016311 // de	0005737 // cy	0052642 // lys	Lysophosphat	PPA6_HUMAN|ACP6|ACPL1|LPAP|UNQ205/PRO231
0016311 // de	0005576 // ex	0003993 // ac	Acid phosphat	ACP7_HUMAN|ACP7|PAPL|PAPL1
0006144 // pu	0005615 // ex	0005515 // pr	Prostatic acid	PPAP_HUMAN|ACPP
0007340 // ac	0043159 // ac	0003677 // DI	Acrosin	ACRO_HUMAN|ACR|ACRS
0008150 // bi	0002080 // ac	0003674 // m	Acrosin-bindir	ACRBP_HUMAN|ACRBP

0006631 // fa	0005759 // m	0005515 // pr	Acyl-CoA synt	ACSF2_HUMAN	ACSF2|UNQ493/PRO1009
0043651 // lir	0005789 // er	0004467 // lo	Long-chain-fa	ACSL1_HUMAN	ACSL1|FACL1|FACL2|LACS|LACS1|LACS
0007584 // re	0005789 // er	0019904 // pr	Long-chain-fa	ACSL3_HUMAN	ACSL3|ACS3|FACL3|LACS3
0001676 // lo	0005737 // cy	0004467 // lo	Long-chain-fa	ACSL4_HUMAN	ACSL4|ACS4|FACL4|LACS4
0001676 // lo	0005739 // m	0005524 // AT	Long-chain-fa	ACSL5_HUMAN	ACSL5|ACS5|FACL5|UNQ633/PRO1250
0001676 // lo	0005789 // er	0005524 // AT	Long-chain-fa	ACSL6_HUMAN	ACSL6|ACS2|FACL6|KIAA0837|LACS5
0006633 // fa	0005759 // m	0004321 // fa	Acyl-coenzym	ACSM1_HUMAN	ACSM1|BUCS1|LAE|MACS1
0006633 // fa	0005759 // m	0004321 // fa	Acyl-coenzym	ACS2A_HUMAN	ACSM2A|ACSM2|MACS2
0006633 // fa	0005759 // m	0004321 // fa	Acyl-coenzym	ACSM3_HUMAN	ACSM3|SAH
0006633 // fa	0005759 // m	0004321 // fa	Acyl-coenzym	ACSM4_HUMAN	ACSM4
0006633 // fa	0005759 // m	0004321 // fa	Acyl-coenzym	ACSM6_HUMAN	ACSM6|C10orf129
0006085 // ac	0005759 // m	0016208 // AI	Acetyl-coenzy	ACS2L_HUMAN	ACSS1|ACAS2L|KIAA1846
0019427 // ac	0005759 // m	0003987 // ac	Acetyl-coenzy	ACSA_HUMAN	ACSS2|ACAS2
0046951 // ke	0005759 // m	0005524 // AT	Acyl-CoA synt	ACSS3_HUMAN	ACSS3
0090131 // m	0030175 // fil	0019901 // pr	Actin, aortic s	ACTA_HUMAN	ACTA2|ACTSA|ACTVS|GIG46
0008150 // bi	0005737 // cy	0005524 // AT	Beta-actin-like	ACTBL_HUMAN	ACTBL2
0045214 // sa	0005886 // pl	0005515 // pr	Actin, cytopla	ACTG_HUMAN	ACTG1|ACTG
0090131 // m	0030175 // fil	0005524 // AT	Actin, gamma	ACTH_HUMAN	ACTG2|ACTA3|ACTL3|ACTSG
0002576 // pl	0005916 // fa	0044325 // io	Alpha-actinin-	ACTN1_HUMAN	ACTN1
0070885 // ne	0005829 // cy	0005178 // in	Alpha-actinin-	ACTN3_HUMAN	ACTN3
1903506 // re	0005634 // nu	0005515 // pr	Alpha-actinin-	ACTN4_HUMAN	ACTN4
0006888 // EF	0005576 // ex	0005515 // pr	Actin-related	ARP10_HUMAN	ACTR10|ACTR11|ARP11
0000086 // G2	0005829 // cy	0005515 // pr	Alpha-centrac	ACTZ_HUMAN	ACTR1A|CTRN1
0007163 // es	0005829 // cy	0051015 // ac	Actin-related	ARP3_HUMAN	ACTR3|ARP3
0008150 // bi	0005885 // Ar	0005524 // AT	Actin-related	ARP3C_HUMAN	ACTR3C|ARP11
0006310 // DI	0005634 // nu	0005515 // pr	Actin-related	ARP5_HUMAN	ACTR5|ARP5
0006310 // DI	0005634 // nu	0005515 // pr	Actin-related	ARP8_HUMAN	ACTR8|ARP8|INO80N
	0005856 // cy		Actin-related	ACTT3_HUMAN	ACTRT3|ARPM1
0030308 // ne	0048179 // ac	0046332 // SN	Activin recept	ACV1B_HUMAN	ACVR1B|ACVRLK4|ALK4
0046676 // ne	0048179 // ac	0004702 // re	Activin recept	ACV1C_HUMAN	ACVR1C|ALK7
0035912 // do	0005886 // pl	0046332 // SN	Serine/threon	ACVL1_HUMAN	ACVRL1|ACVRLK1|ALK1
0006520 // ce	0070062 // ex	0004046 // ar	Aminoacylase	ACY1_HUMAN	ACY1
0016032 // vi	0070062 // ex	0004046 // ar	N-acyl-aromat	ACY3_HUMAN	ACY3|ASPA2
0006796 // ph	0070062 // ex	0003998 // ac	Acylphosphat	ACYP1_HUMAN	ACYP1|ACYPE
0006796 // ph		0003998 // ac	Acylphosphat	ACYP2_HUMAN	ACYP2|ACYP
0001821 // hi	0043025 // ne	0004000 // ac	Adenosine de	ADA_HUMAN	ADA|ADA1
0006396 // RI		0003723 // RI	Adenosine de	ADAD2_HUMAN	ADAD2|TENRL
0007162 // ne	0035579 // sp	0004222 // m	Disintegrin an	ADA10_HUMAN	ADAM10|KUZ|MADM
0006508 // pr	0005615 // ex	0008237 // m	Disintegrin an	ADA12_HUMAN	ADAM12|MLTN|UNQ346/PRO545
0006508 // pr	0016021 // in	0004222 // m	Disintegrin an	ADA15_HUMAN	ADAM15|MDC15
0006509 // m	0045121 // m	0004222 // m	Disintegrin an	ADA17_HUMAN	ADAM17|CSVP|TACE
0007507 // he	0016021 // in	0005515 // pr	Disintegrin an	ADA19_HUMAN	ADAM19|MLTNB|FKSG34

0007155 // ce	0016021 // in	0005515 // pr	Disintegrin an	ADA22_HUMAN|ADAM22|MDC2
0007155 // ce	0005887 // in	0008237 // m	Disintegrin an	ADA23_HUMAN|ADAM23|MDC3
0006508 // pr	0005739 // m	0008237 // m	Disintegrin an	ADA28_HUMAN|ADAM28|ADAM23|MDCL
0006508 // pr	0016021 // in	0004222 // m	Disintegrin an	ADA32_HUMAN|ADAM32|UNQ5982/PRO21340
0008150 // bi	0005575 // ce	0003674 // m	Putative disin	ADAM5_HUMAN|ADAM5|ADAM5P|TMDC2
0033089 // po	0002102 // po	0050839 // ce	Disintegrin an	ADAM8_HUMAN|ADAM8|MS2
0032331 // ne	0031012 // ex	0004222 // m	A disintegrin a	ATS12_HUMAN|ADAMTS12|UNQ1918/PRO4389
0014075 // re	0005788 // er	0005515 // pr	A disintegrin a	ATS13_HUMAN|ADAMTS13|C9orf8|UNQ6102/PRO2008
0006508 // pr	0009986 // ce	0008201 // he	A disintegrin a	ATS15_HUMAN|ADAMTS15
0001658 // br	0005578 // pr	0008270 // zir	A disintegrin a	ATS16_HUMAN|ADAMTS16|KIAA2029
0006508 // pr	0005578 // pr	0008270 // zir	A disintegrin a	ATS18_HUMAN|ADAMTS18|ADAMTS21
0006508 // pr	0005578 // pr	0008270 // zir	A disintegrin a	ATS19_HUMAN|ADAMTS19
0016485 // pr	0005615 // ex	0008270 // zir	A disintegrin a	ATS2_HUMAN|ADAMTS2|PCINP|PCPNI
0009967 // po	0005615 // ex	0008270 // zir	A disintegrin a	ATS20_HUMAN|ADAMTS20
0016485 // pr	0005615 // ex	0004222 // m	A disintegrin a	ATS3_HUMAN|ADAMTS3|KIAA0366
0036066 // pr	0005788 // er	0008237 // m	A disintegrin a	ATS5_HUMAN|ADAMTS5|ADAMTS11|ADMP2
0006508 // pr	0005578 // pr	0008270 // zir	A disintegrin a	ATS6_HUMAN|ADAMTS6
0036066 // pr	0005788 // er	0005515 // pr	A disintegrin a	ATS7_HUMAN|ADAMTS7
0006508 // pr	0031012 // ex	0005178 // in	A disintegrin a	ATS8_HUMAN|ADAMTS8|METH2
0036066 // pr	0005788 // er	0008270 // zir	ADAMTS-like	ATL1_HUMAN|ADAMTSL1|ADAMTSR1|C9orf94|UNQ52
0006508 // pr	0043231 // in	0005515 // pr	ADAMTS-like	ATL3_HUMAN|ADAMTSL3|KIAA1233
0036066 // pr	0005788 // er	0005515 // pr	ADAMTS-like	ATL4_HUMAN|ADAMTSL4|TSRC1|PP1396|UNQ2803/PR
0006508 // pr	0005576 // ex	0008201 // he	ADAMTS-like	ATL5_HUMAN|ADAMTSL5|THSD6
0043547 // po	0005634 // nu	0005515 // pr	Arf-GAP with	ADAP1_HUMAN|ADAP1|CENTA1
0043547 // po	0005886 // pl	0005515 // pr	Arf-GAP with	ADAP2_HUMAN|ADAP2|CENTA2
0060384 // in	0005654 // nu	0005515 // pr	Double-strand	RED1_HUMAN|ADARB1|ADAR2|DRADA2|RED1
0006397 // m	0005634 // nu	0003723 // RN	Double-strand	RED2_HUMAN|ADARB2|ADAR3|RED2
0009451 // RN	0005654 // nu	0046872 // m	Probable inac	ADAT3_HUMAN|ADAT3|TAD3
0006468 // pr	0005576 // ex	0004674 // pr	Uncharacteriz	ADCK1_HUMAN|ADCK1
0006468 // pr	0016021 // in	0004674 // pr	Uncharacteriz	ADCK2_HUMAN|ADCK2|AARF
0006468 // pr	0016021 // in	0004674 // pr	Uncharacteriz	ADCK5_HUMAN|ADCK5
0071277 // ce	0005886 // pl	0005524 // AT	Adenylate cyc	ADCY1_HUMAN|ADCY1
0007204 // po	0005886 // pl	0004016 // ac	Adenylate cyc	ADCY5_HUMAN|ADCY5
0030819 // po	0005886 // pl	0004016 // ac	Adenylate cyc	ADCY7_HUMAN|ADCY7|KIAA0037
0006171 // cA	0005886 // pl	0004016 // ac	Adenylate cyc	ADCY9_HUMAN|ADCY9|KIAA0520
0055085 // tra	0044853 // pl	0003779 // ac	Beta-adducin	ADDB_HUMAN|ADD2|ADDB
0055085 // tra	0005903 // br	0005516 // ca	Gamma-addu	ADDG_HUMAN|ADD3|ADDL
0007186 // G-	0016021 // in	0004930 // G-	Adhesion G pr	AGRA3_HUMAN|ADGRA3|GPR125|UNQ556/PRO1113
0007186 // G-	0016021 // in	0004930 // G-	Adhesion G pr	AGRB2_HUMAN|ADGRB2|BAI2
0007186 // G-	0098794 // po	0005096 // GT	Adhesion G pr	AGRB3_HUMAN|ADGRB3|BAI3|KIAA0550
0007186 // G-	0005622 // in	0004930 // G-	Adhesion G-pr	AGRD1_HUMAN|ADGRD1|GPR133|PGR25

0007186 // G-	0016021 // in	0004930 // G-	Adhesion G-p	AGRD2_HUMAN|ADGRD2|GPR144|PGR24
0002250 // ac	0009897 // ex	0005509 // ca	Adhesion G pr	AGRE1_HUMAN|ADGRE1|EMR1|TM7LN3
0007186 // G-	0005886 // pl	0005509 // ca	Adhesion G pr	AGRE3_HUMAN|ADGRE3|EMR3|UNQ683/PRO1562
0007186 // G-	0016021 // in	0004930 // G-	Adhesion G-p	AGRF1_HUMAN|ADGRF1|GPR110|PGR19
0007186 // G-	0016021 // in	0004930 // G-	Adhesion G-p	AGRF2_HUMAN|ADGRF2|GPR111|PGR20
0007186 // G-	0016021 // in	0004930 // G-	Adhesion G pr	AGRF4_HUMAN|ADGRF4|GPR115|PGR18
0016477 // ce	0045121 // m	0050840 // ex	Adhesion G-p	AGRG1_HUMAN|ADGRG1|GPR56|TM7LN4|TM7XN1|UN
0007186 // G-	0005887 // in	0004930 // G-	Adhesion G-p	AGRG2_HUMAN|ADGRG2|GPR64|HE6|TM7LN2
0030183 // B	0016021 // in	0004930 // G-	Adhesion G pr	AGRG3_HUMAN|ADGRG3|GPR97|PGR26
0007186 // G-	0016021 // in	0004930 // G-	Adhesion G-p	AGRG5_HUMAN|ADGRG5|GPR114|PGR27|UNQ2524/PI
0007186 // G-	0016021 // in	0004930 // G-	Adhesion G-p	AGRG7_HUMAN|ADGRG7|GPR128
0007157 // he	0016021 // in	0005515 // pr	Adhesion G pr	AGRL1_HUMAN|ADGRL1|KIAA0821|LEC2|LPHN1
0007166 // ce	0016021 // in	0030246 // ca	Adhesion G pr	AGRL2_HUMAN|ADGRL2|KIAA0786|LEC1|LPHH1|LPHN2
0007186 // G-	0005911 // ce	0005509 // ca	Adhesion G pr	AGRL3_HUMAN|ADGRL3|KIAA0768|LEC3|LPHN3
0006069 // et	0070062 // ex	0004024 // al	Alcohol dehyc	ADH6_HUMAN|ADH6
0006103 // 2-	0005759 // m	0047988 // hy	Hydroxyacid-c	HOT_HUMAN|ADHFE1|HMFT2263
0042304 // re	0005886 // pl	0019901 // pr	Adiponectin r	PAQR1_HUMAN|ADIPOR1|PAQR1|TESBP1A|CGI-45
0071478 // ce	0070062 // ex	0003674 // m	Adipogenesis	ADIRF_HUMAN|ADIRF|AFRO|APM2|C10orf116
0006701 // pr	0005576 // ex	0005102 // re	ADM	ADML_HUMAN|ADM|AM
0001525 // ar	0005576 // ex	0032403 // pr	ADM2	ADM2_HUMAN|ADM2|AM2
0008150 // bi	0005576 // ex	0003674 // m	Putative adre	ADM5_HUMAN|ADM5|C19orf76
0000098 // su	0005739 // m	0046872 // m	2-aminoethan	AEDO_HUMAN|ADO|C10orf22
0045741 // pc	0005886 // pl	0031683 // G-	Adenosine rec	AA1R_HUMAN|ADORA1
0006464 // ce	0005622 // in	0030955 // pc	[Protein ADP-	ADPRH_HUMAN|ADPRH|ARH1
0051725 // pr	0005622 // in	0003875 // AI	[Protein ADP-	ARHL1_HUMAN|ADPRHL1|ARH2
0071451 // ce	0016604 // nu	0046872 // m	Poly(ADP-ribo	ARHL2_HUMAN|ADPRHL2|ARH3
0008283 // ce	0005886 // pl	0004937 // al	Alpha-1D adre	ADA1D_HUMAN|ADRA1D|ADRA1A
0031649 // he	0005886 // pl	0051379 // ep	Beta-1 adrene	ADRB1_HUMAN|ADRB1|ADRB1R|B1AR
0043410 // pc	0005886 // pl	0005515 // pr	Beta-2 adrene	ADRB2_HUMAN|ADRB2|ADRB2R|B2AR
0006511 // ub	0005829 // cy	0005515 // pr	Proteasomal u	ADRM1_HUMAN|ADRM1|GP110
0051262 // pr	0005739 // m	0004018 // N6	Adenylosuccin	PUR8_HUMAN|ADSL|AMPS
0006167 // AI	0005829 // cy	0004019 // ac	Adenylosuccin	PURA1_HUMAN|ADSSL1|ADSS1
0010212 // re	0005730 // nu	0005515 // pr	Apoptosis-enh	AEN_HUMAN|AEN|ISG20L1|SBBI58
0016055 // W	0005634 // nu	0003714 // tra	Amino-termin	AES_HUMAN|AES|GRG|GRG5

0009966 // re	0005925 // fo	0003779 // ac	Actin filament	AFAP1_HUMAN|AFAP1|AFAP
0006954 // in	0005829 // cy	0035591 // sig	Actin filament	AF1L2_HUMAN|AFAP1L2|KIAA1914|XB130
0008150 // bi	0005575 // ce	0003674 // m	Putative unch	AFAS1_HUMAN|AFDN-AS1|C6orf124
0010629 // ne	0016607 // nu	0002151 // G-	AF4/FMR2 far	AFF2_HUMAN|AFF2|FMR2|OX19
0006366 // tra	0005654 // nu	0005515 // pr	AF4/FMR2 far	AFF4_HUMAN|AFF4|AF5Q31|MCEF|HSPC092
0006508 // pr	0005576 // ex	0008233 // pe	N(4)-(beta-N-a	ASPG_HUMAN|AGA
0043547 // po		0005096 // GT	Arf-GAP with	AGA11_HUMAN|AGAP11|KIAA1975
0043547 // po		0005096 // GT	Arf-GAP with	AGP14_HUMAN|AGAP14|AGAP14P|CTGLF11P
0043524 // ne	0005739 // m	0003924 // GT	Arf-GAP with	AGAP2_HUMAN|AGAP2|CENTG1|KIAA0167
0043547 // po		0005096 // GT	Arf-GAP with	AGAP4_HUMAN|AGAP4|AGAP8|CTGLF1|CTGLF5|MRIP2
0043547 // po		0005096 // GT	Arf-GAP with	AGAP6_HUMAN|AGAP6|CTGLF3
0043547 // po		0005096 // GT	Putative Arf-G	AGAP7_HUMAN|AGAP7P|AGAP7|CTGLF4
0043547 // po		0005096 // GT	Arf-GAP with	AGAP9_HUMAN|AGAP9|CTGLF6
0006508 // pr	0005829 // cy	0015631 // tu	Cytosolic carb	CBPC4_HUMAN|AGBL1|CCP4
0006508 // pr	0005829 // cy	0004181 // m	Cytosolic carb	CBPC2_HUMAN|AGBL2|CCP2
0035609 // C-	0005829 // cy	0015631 // tu	Cytosolic carb	CBPC6_HUMAN|AGBL4|CCP6
0043547 // po	0016020 // m	0005096 // GT	Arf-GAP doma	AGFG2_HUMAN|AGFG2|HRBL|RABR
0045039 // pr	0005758 // m	0004143 // di	Acylglycerol k	AGK_HUMAN|AGK|MULK
0033388 // pu	0070062 // ex	0008783 // ag	Agmatinase, r	SPEB_HUMAN|AGMAT
0006643 // m	0005783 // er	0050479 // gl	Alkylglycerol r	ALKMO_HUMAN|AGMO|TMEM195
0010586 // m	0005829 // cy	0005515 // pr	Protein argon	AGO4_HUMAN|AGO4|EIF2C4|KIAA1567
0016024 // CD	0000139 // Go	0003841 // 1-	1-acyl-sn-glyc	PLCC_HUMAN|AGPAT3|LPAAT3|UNQ759/PRO1490
0016024 // CD	0016021 // in	0003841 // 1-	1-acyl-sn-glyc	PLCD_HUMAN|AGPAT4|UNQ499/PRO1016
0016024 // CD	0016021 // in	0003841 // 1-	1-acyl-sn-glyc	PLCE_HUMAN|AGPAT5
0007213 // G-	0005796 // Go	0043395 // he	Agrin	AGRIN_HUMAN|AGRN|AGRIN
0006508 // pr	0005730 // nu	0015631 // tu	Cytosolic carb	CBPC1_HUMAN|AGTPBP1|CCP1|KIAA1035|NNA1
0034374 // lo	0005886 // pl	0031711 // br	Type-1 angiot	AGTR1_HUMAN|AGTR1|AGTR1A|AGTR1B|AT2R1|AT2R1
0006355 // re	0005829 // cy	0003677 // DN	Protein ELYS	ELYS_HUMAN|AHCTF1|ELYS|TMBS62|MSTP108
		0003677 // DN	AT-hook DNA-	AHDC1_HUMAN|AHDC1
1901385 // re	0044291 // ce	0003723 // RN	Neuroblast di	AHNK_HUMAN|AHNAK|PM227
0045944 // po	0005654 // nu	0001191 // tra	Aryl hydrocarl	AHRR_HUMAN|AHRR|BHLHE77|KIAA1234
0006950 // re	0005783 // er	0005515 // pr	Activator of 9	AHSA1_HUMAN|AHSA1|C14orf3|HSPC322
0032781 // po		0001671 // AT	Activator of 9	AHSA2_HUMAN|AHSA2
	0005737 // cy	0051015 // ac	Allograft infla	AIF1L_HUMAN|AIF1L|C9orf58|IBA2|UNQ672/PRO1306
0055114 // ox	0005739 // m	0051537 // 2 i	Apoptosis-ind	AIFM3_HUMAN|AIFM3|AIFL
	0016021 // in	0005515 // pr	Androgen-ind	AIG1_HUMAN|AIG1|CGI-103
0032088 // ne	0097169 // AI	0042802 // id	Interferon-inc	AIM2_HUMAN|AIM2
0007010 // cy	0005912 // ac	0005515 // pr	LIM domain-c	AJUBA_HUMAN|AJUBA|JUB
0006173 // dA	0005829 // cy	0004550 // nu	Adenylate kin	KAD5_HUMAN|AK5
0046940 // nu	0005654 // nu	0005515 // pr	Adenylate kin	KAD6_HUMAN|AK6|CINAP|AD-004|CGI-137

0009142 // nu	0005829 // cy	0004550 // nu	Adenylate kin	KAD7_HUMAN|AK7
0009142 // nu	0005829 // cy	0005515 // pr	Adenylate kin	KAD8_HUMAN|AK8|C9orf98
0090036 // re	0005938 // ce	0008179 // ac	A-kinase anch	AKA12_HUMAN|AKAP12|AKAP250
0006605 // pr	0005886 // pl	0008179 // ac	A-kinase anch	AKAP5_HUMAN|AKAP5|AKAP79
0060306 // re	0005829 // cy	0005515 // pr	A-kinase anch	AKAP9_HUMAN|AKAP9|AKAP350|AKAP450|KIAA0803
0034446 // su	0005654 // nu	0005515 // pr	A-kinase-inter	AKIP1_HUMAN|AKIP1|BCA3|C11orf17
0006366 // tra	0005829 // cy	0000978 // RN	AT-hook-conta	AKNA_HUMAN|AKNA|KIAA1968
0016488 // fa	0070062 // ex	0045550 // ge	Aldo-keto red	AK1BA_HUMAN|AKR1B10|AKR1B11
0006703 // es	0005759 // m	0004303 // es	Aldo-keto red	AK1BF_HUMAN|AKR1B15
0006805 // xe	0005829 // cy	0047006 // 17	Aldo-keto red	AK1C1_HUMAN|AKR1C1|DDH|DDH1
0008202 // ste	0005737 // cy	0047086 // ke	Aldo-keto red	AK1C2_HUMAN|AKR1C2|DDH2
0055114 // ox	0005829 // cy	0036130 // pr	Aldo-keto red	AK1C3_HUMAN|AKR1C3|DDH1|HSD17B5|KIAA0119|PG
0044597 // da	0070062 // ex	0004033 // ald	Aflatoxin B1 a	ARK72_HUMAN|AKR7A2|AFAR|AFAR1|AKR7
0006805 // xe	0070062 // ex	0004033 // ald	Aflatoxin B1 a	ARK74_HUMAN|AKR7L|AFAR3|AKR7A4
0007249 // I-k	0005829 // cy	0005515 // pr	RAC-alpha ser	AKT1_HUMAN|AKT1|PKB|RAC
0038202 // TO	0005829 // cy	0005515 // pr	Proline-rich A	AKTS1_HUMAN|AKT1S1|PRAS40
0032008 // po	0016020 // m	0005515 // pr	RAC-gamma s	AKT3_HUMAN|AKT3|PKBG
0006898 // re	0005794 // Go	0016209 // an	Serum albumi	ALBU_HUMAN|ALB|GIG20|GIG42|PRO0903|PRO1708|F
0008285 // ne	0005829 // cy	0001758 // re	Retinal dehyd	AL1A2_HUMAN|ALDH1A2|RALDH2
0042574 // re	0070062 // ex	0001758 // re	Aldehyde deh	AL1A3_HUMAN|ALDH1A3|ALDH6
0006068 // et	0005739 // m	0051287 // NA	Aldehyde deh	AL1B1_HUMAN|ALDH1B1|ALDH5|ALDHX
0006730 // or	0005739 // m	0003824 // ca	Cytosolic 10-fo	AL1L1_HUMAN|ALDH1L1|FTHFD
0006730 // or	0005739 // m	0004029 // ald	Mitochondria	AL1L2_HUMAN|ALDH1L2
0005975 // ca	0070062 // ex	0004030 // ald	Aldehyde deh	ALDH2_HUMAN|ALDH2|ALDM
0042493 // re	0005829 // cy	0005515 // pr	Aldehyde deh	AL3A1_HUMAN|ALDH3A1|ALDH3
0001561 // fa	0005789 // er	0046577 // lo	Fatty aldehyd	AL3A2_HUMAN|ALDH3A2|ALDH10|FALDH
0006629 // lip	0005829 // cy	0005515 // pr	Aldehyde deh	AL3B1_HUMAN|ALDH3B1|ALDH7
0006066 // alc	0005811 // lip	0004028 // 3-	Aldehyde deh	AL3B2_HUMAN|ALDH3B2|ALDH8
0046487 // gly	0005759 // m	0042802 // ide	Delta-1-pyrrol	AL4A1_HUMAN|ALDH4A1|ALDH4|P5CDH
0006210 // th	0005739 // m	0003723 // RN	Methylmalona	MMSA_HUMAN|ALDH6A1|MMSDH

0019285 // gl	0005739 // m	0004029 // al	Alpha-aminoa	AL7A1_HUMAN|ALDH7A1|ATQ1
0042904 // 9-	0005829 // cy	0001758 // re	Aldehyde deh	AL8A1_HUMAN|ALDH8A1|ALDH12
0030855 // ep	0034774 // se	0004332 // fru	Fructose-bispl	ALDOC_HUMAN|ALDOC|ALDC
0006488 // do	0016021 // in	0004583 // do	Putative Dol-P	AG10B_HUMAN|ALG10B|KCR1
0097502 // m	0005783 // er	0000030 // m	Putative glyco	ALG1L_HUMAN|ALG1L
0097502 // m	0005783 // er	0000030 // m	Putative glyco	AG1L2_HUMAN|ALG1L2
0007165 // sig	0043234 // pr	0005515 // pr	ALK tyrosine k	ALK_HUMAN|ALK
0006281 // DI	0005739 // m	0003906 // DI	Nucleic acid d	ALKB1_HUMAN|ALKBH1|ABH|ABH1|ALKBH
0006307 // DI	0005654 // nu	0051747 // cy	DNA oxidative	ALKB2_HUMAN|ALKBH2|ABH2
0006307 // DI	0005654 // nu	0043734 // DI	Alpha-ketoglu	ALKB3_HUMAN|ALKBH3|ABH3|DEPC1
1902445 // re	0005759 // m	0051213 // di	Alpha-ketoglu	ALKB7_HUMAN|ALKBH7|ABH7|SPATA11|UNQ6002/PRC
0002098 // tR	0016604 // nu	0008270 // zir	Alkylated DNA	ALKB8_HUMAN|ALKBH8|ABH8
0043410 // po	0005829 // cy	1990136 // lir	Arachidonate	LX12B_HUMAN|ALOX12B
0006954 // in	0005811 // lip	0050473 // ar	Arachidonate	LOX15_HUMAN|ALOX15|LOG15
2001300 // lip	0005635 // nu	0005515 // pr	Arachidonate	LOX5_HUMAN|ALOX5|LOG5
0043651 // lir	0005829 // cy	0051120 // he	Hydroperoxid	LOXE3_HUMAN|ALOXE3
0006468 // pr	0005737 // cy	0005524 // AT	Alpha-protein	ALPK1_HUMAN|ALPK1|KIAA1527|LAK
0006468 // pr	0005737 // cy	0005524 // AT	Alpha-protein	ALPK2_HUMAN|ALPK2|HAK
0016311 // de	0009986 // ce	0005515 // pr	Alkaline phos	PPB1_HUMAN|ALPP|PLAP
0016311 // de	0031225 // ar	0004035 // all	Alkaline phos	PPBN_HUMAN|ALPPL2|ALPPL
0043547 // po	0005829 // cy	0005089 // Rh	ALS2 C-termir	AL2CL_HUMAN|ALS2CL
	0005737 // cy	0005515 // pr	Amyotrophic l	AL2SB_HUMAN|ALS2CR12
0010718 // po	0005634 // nu	0005515 // pr	ALX homeobo	ALX1_HUMAN|ALX1|CART1
0042981 // re	0005634 // nu	0000977 // RI	Homeobox pr	ALX4_HUMAN|ALX4|KIAA1788
0031297 // re	0005829 // cy	0003723 // RI	THO complex	THOC4_HUMAN|ALYREF|ALY|BEF|THOC4
0006595 // po	0005829 // cy	0019810 // pu	S-adenosylme	DCAM_HUMAN|AMD1|AMD
0019557 // hi	0005829 // cy	0050480 // im	Probable imid	HUTI_HUMAN|AMDHD1|HMFT1272
0019262 // N-	0005829 // cy	0005515 // pr	N-acetylgluco	NAGA_HUMAN|AMDHD2|CGI-14
0007611 // lea	0005794 // Go	1990381 // ub	E3 ubiquitin-p	AMFR_HUMAN|AMFR|RNF45
0042493 // re	0005615 // ex	0008083 // gr	Muellerian-inl	MIS_HUMAN|AMH|MIF
0043069 // ne	0016021 // in	0005515 // pr	Amphoterin-in	AMGO2_HUMAN|AMIGO2|ALI1
0007275 // m	0005905 // cla	0005102 // re	Protein amnic	AMNLS_HUMAN|AMN|UNQ513/PRO1028
0003365 // es	0009897 // ex	0005515 // pr	Angiomotin	AMOT_HUMAN|AMOT|KIAA1071
0016055 // W	0005923 // bi	0005515 // pr	Angiomotin-li	AMOL1_HUMAN|AMOTL1
0097009 // er	0005829 // cy	0003876 // AI	AMP deamina	AMPD2_HUMAN|AMPD2
0034101 // er	0034774 // se	0003876 // AI	AMP deamina	AMPD3_HUMAN|AMPD3
0019464 // gl	0005739 // m	0004047 // ar	Aminomethyl	GCST_HUMAN|AMT|GCST
0070169 // po	0005788 // en	0005515 // pr	Amelotin	AMTN_HUMAN|AMTN|UNQ689/PRO1329
0007586 // di	0070062 // ex	0046872 // m	Alpha-amylase	AMY2B_HUMAN|AMY2B

0006508 // pr	0005575 // ce	0008233 // pe	Archaemetzin	AMZ1_HUMAN|AMZ1|KIAA1950
0006508 // pr	0005575 // ce	0008233 // pe	Archaemetzin	AMZ2_HUMAN|AMZ2|BM-014
0032431 // ac	0005615 // ex	0005515 // pr	Angiogenin	ANGI_HUMAN|ANG|RNASE5
0070935 // 3'-	0015030 // Ca	0003730 // m	Protein angel	ANGE2_HUMAN|ANGEL2|KIAA0759L
0030097 // he	0005622 // int	0030971 // re	Angiopoietin-	ANGP1_HUMAN|ANGPT1|KIAA0003
0007275 // m	0005615 // ex	0005102 // re	Angiopoietin-	ANGL2_HUMAN|ANGPTL2|ARP2|UNQ170/PRO196
0001666 // re	0005576 // ex	0005515 // pr	Angiopoietin-	ANGL4_HUMAN|ANGPTL4|ARP4|HFARP|PGAR|PP1158|
0006888 // ER	0005829 // cy	0005515 // pr	Ankyrin-1	ANK1_HUMAN|ANK1|ANK
0086015 // SA	0005829 // cy	0044325 // io	Ankyrin-2	ANK2_HUMAN|ANK2
0043001 // Go	0005829 // cy	0030507 // sp	Ankyrin-3	ANK3_HUMAN|ANK3
0007165 // sig			Ankyrin repea	AKD1B_HUMAN|ANKDD1B
0035435 // ph	0016021 // int	0030504 // in	Progressive ar	ANKH_HUMAN|ANKH|KIAA1581|UNQ241/PRO274
0042787 // pr	0005737 // cy	0005515 // pr	Ankyrin repea	AKIB1_HUMAN|ANKIB1|KIAA1386
1905453 // re	0005634 // nu	0004519 // er	Ankyrin repea	ANKL1_HUMAN|ANKLE1|ANKRD41|LEM3
0008589 // re	0005929 // cil	0019899 // er	Ankyrin repea	ANKY2_HUMAN|ANKMY2
0055008 // ca	0031674 // I b	0003677 // DI	Ankyrin repea	ANKR1_HUMAN|ANKRD1|C193|CARP|HA1A2
	0005886 // pl		Ankyrin repea	AN13D_HUMAN|ANKRD13D
0006936 // m	0016605 // PM	0003712 // tra	Ankyrin repea	ANKR2_HUMAN|ANKRD2|ARPP
0048208 // CO	0005829 // cy	0005515 // pr	Serine/threon	ANR28_HUMAN|ANKRD28|KIAA0379
0065009 // re		0008200 // io	Ankyrin repea	AN36C_HUMAN|ANKRD36C
	0005739 // m		Ankyrin repea	ANR37_HUMAN|ANKRD37|LPR2BP
0008150 // bi	0005575 // ce	0003674 // m	Ankyrin repea	ANR39_HUMAN|ANKRD39|HSPC200
0015031 // pr	0005768 // er	0005515 // pr	Ankyrin repea	ANR50_HUMAN|ANKRD50|KIAA1223
0045859 // re	0030496 // m	0019887 // pr	Ankyrin repea	ANR54_HUMAN|ANKRD54|LIAR
0008584 // m			Ankyrin repea	ANKR7_HUMAN|ANKRD7
0048013 // ep	0043005 // ne	0005515 // pr	Ankyrin repea	ANS1A_HUMAN|ANKS1A|ANKS1|KIAA0229|ODIN
1900383 // re	0014069 // po	0046875 // ep	Ankyrin repea	ANS1B_HUMAN|ANKS1B
		0005515 // pr	Ankyrin repea	ANKS3_HUMAN|ANKS3|KIAA1977
	0005737 // cy	0005515 // pr	Ankyrin repea	ANKS6_HUMAN|ANKS6|ANKRD14|PKDR1|SAMD6
0000281 // m	0099738 // ce	0045296 // ca	Anillin	ANLN_HUMAN|ANLN
0007200 // ph	0016324 // ap	0005229 // int	Anoctamin-1	ANO1_HUMAN|ANO1|DOG1|ORAOV2|TAOS2|TMEM16

0006821 // ch	0005886 // pl	0005229 // in	Anoctamin-10	ANO10_HUMAN|ANO10|TMEM16K
1902476 // ch	0097730 // nc	0005229 // in	Anoctamin-2	ANO2_HUMAN|ANO2|C12orf3|TMEM16B
0050982 // de	0016021 // in	0005229 // in	Anoctamin-3	ANO3_HUMAN|ANO3|C11orf25|TMEM16C|GENX-3947
0043065 // po	0005886 // pl	0042803 // pr	Anoctamin-6	ANO6_HUMAN|ANO6|TMEM16F
0006821 // ch	0005886 // pl	0017128 // ph	Anoctamin-7	ANO7_HUMAN|ANO7|NGEP|PCANAP5|TMEM16G
0006821 // ch	0005886 // pl	0005229 // in	Anoctamin-8	ANO8_HUMAN|ANO8|KIAA1623|TMEM16H
1902939 // ne	0005886 // pl	0005515 // pr	Anoctamin-9	ANO9_HUMAN|ANO9|PIG5|TMEM16J|TP53I5
0007155 // ce	0005578 // pr	0004867 // se	Anosmin-1	KALM_HUMAN|ANOS1|ADMLX|KAL|KAL1|KALIG1
0043171 // pe	0009897 // ex	0001618 // vi	Aminopeptida	AMPN_HUMAN|ANPEP|APN|CD13|PEPN
1901998 // to	0005886 // pl	0005515 // pr	Anthrax toxin	ANTR2_HUMAN|ANTXR2|CMG2
0070301 // ce	0005576 // ex	0005515 // pr	Annexin A1	ANXA1_HUMAN|ANXA1|ANX1|LPC1
0030154 // ce	0005886 // pl	0005544 // ca	Annexin A13	ANX13_HUMAN|ANXA13|ANX13
0006357 // re	0005737 // cy	0005509 // ca	Annexin A4	ANXA4_HUMAN|ANXA4|ANX4
0002576 // pl	0005737 // cy	0005543 // ph	Annexin A5	ANXA5_HUMAN|ANXA5|ANX5|ENX2|PP4
0006816 // ca	0070062 // ex	0015485 // ch	Annexin A6	ANXA6_HUMAN|ANXA6|ANX6
0006914 // au	0070062 // ex	0005515 // pr	Annexin A7	ANXA7_HUMAN|ANXA7|ANX7|SNX|OK/SW-cl.95
0007032 // en	0005829 // cy	0043325 // ph	Annexin A8	ANXA8_HUMAN|ANXA8|ANX8
	0070062 // ex	0005509 // ca	Annexin A8-lik	AXA81_HUMAN|ANXA8L1|ANXA8L2
0098609 // ce	0070062 // ex	0005543 // ph	Annexin A9	ANXA9_HUMAN|ANXA9|ANX31
0009308 // ar	0005737 // cy	0009055 // el	Retina-specifi	AOC2_HUMAN|AOC2
0046677 // re	0016021 // in	0048038 // qu	Membrane pr	AOC3_HUMAN|AOC3|VAP1
0042816 // vi	0005829 // cy	0004031 // al	Aldehyde oxic	AOXA_HUMAN|AOX1|AO

0006886 // in	0005829 // cy	0005515 // pr	AP-1 complex	AP1M1_HUMAN|AP1M1|CLTNM
0006605 // pr	0005829 // cy	0005515 // pr	AP-1 complex	AP1M2_HUMAN|AP1M2
0016192 // ve	0005829 // cy	0008565 // pr	AP-1 complex	AP1S2_HUMAN|AP1S2|DC22
0048013 // ep	0005886 // pl	0035615 // cla	AP-2 complex	AP2A2_HUMAN|AP2A2|ADTAB|CLAPA2|HIP9|HYPJ|KIA
0048013 // ep	0005886 // pl	0035615 // cla	AP-2 complex	AP2S1_HUMAN|AP2S1|AP17|CLAPS2
0006886 // in	0005794 // Go	0008565 // pr	AP-3 complex	AP3S2_HUMAN|AP3S2
0006886 // in	0005802 // tra	0005215 // tra	AP-4 complex	AP4B1_HUMAN|AP4B1
0015031 // pr	0005905 // cla	0005215 // tra	AP-4 complex	AP4S1_HUMAN|AP4S1
0000724 // do	0005765 // lys	0005515 // pr	AP-5 complex	AP5S1_HUMAN|AP5S1|C20orf29
0035264 // m	0005794 // Go	0005515 // pr	Amyloid-beta	APBA1_HUMAN|APBA1|MINT1|X11
0007399 // ne	0008021 // sy	0005515 // pr	Amyloid-beta	APBA2_HUMAN|APBA2|MINT2|X11L
0007165 // sig	0042734 // pr	0005515 // pr	Amyloid-beta	APBB1_HUMAN|APBB1|FE65|RIR
0006355 // re	0005829 // cy	0001540 // be	Amyloid-beta	APBB3_HUMAN|APBB3|FE65L2
0097698 // te	0005654 // nu	0003723 // RN	DNA-(apurinic	APEX1_HUMAN|APEX1|APE|APE1|APEX|APX|HAP1|REI
0043065 // po	0005886 // pl	0005515 // pr	Gamma-secre	APH1A_HUMAN|APH1A|PSF|CGI-78|UNQ579/PRO1141
0090305 // nu	0005634 // nu	0005515 // pr	Aprataxin and	APLF_HUMAN|APLF|C2orf13|PALF|XIP1
0006955 // im	0005576 // ex	0001664 // G-	Apelin	APEL_HUMAN|APLN|APEL
0006897 // en	0005604 // ba	0031695 // al	Amyloid-like p	APLP1_HUMAN|APLP1
0034378 // ch	0005576 // ex	0015485 // ch	Apolipoprotei	APOA2_HUMAN|APOA2
0051607 // de	0005737 // cy	0004126 // cy	DNA dC->dU-e	ABC3A_HUMAN|APOBEC3A
0051607 // de	0005634 // nu	0003723 // RN	DNA dC->dU-e	ABC3B_HUMAN|APOBEC3B
0010529 // ne	0005737 // cy	0016814 // hy	DNA dC->dU-e	ABC3D_HUMAN|APOBEC3D
0045087 // in	0000932 // cy	0003723 // RN	DNA dC->dU-e	ABC3F_HUMAN|APOBEC3F
0045087 // in	0005829 // cy	0042803 // pr	DNA dC->dU-e	ABC3G_HUMAN|APOBEC3G|MDS019
0010529 // ne	0005737 // cy	0005515 // pr	DNA dC->dU-e	ABC3H_HUMAN|APOBEC3H
0006898 // re	0034361 // ve	0030229 // ve	Apolipoprotei	APOBR_HUMAN|APOBR|APOB48R

0034379 // ve	0042627 // ch	0031210 // ph	Apolipoprotei	APOC1_HUMAN|APOC1
0010875 // po	0005794 // Go	0001540 // be	Apolipoprotei	APOE_HUMAN|APOE
0006629 // lip	0005576 // ex	0005319 // lip	Apolipoprotei	APOF_HUMAN|APOF
0006898 // re	0005788 // en	0005515 // pr	Apolipoprotei	APOL1_HUMAN|APOL1|APOL
0007165 // sig	0005576 // ex	0004871 // sig	Apolipoprotei	APOL3_HUMAN|APOL3
0042157 // lip	0005576 // ex	0008289 // lip	Apolipoprotei	APOL6_HUMAN|APOL6|UNQ3095/PRO21341
0034393 // po	0005739 // m		Apoptogenic p	APOP1_HUMAN|APOPT1|APOP1|C14orf153
0006266 // DI	0005730 // nu	0033699 // DI	Aprataxin	APTX_HUMAN|APTX|AXA1
0015793 // gly	0005886 // pl	0005515 // pr	Aquaporin-1	AQP1_HUMAN|AQP1|CHIP28
0015793 // gly	0005783 // en	0005215 // tra	Aquaporin-11	AQP11_HUMAN|AQP11|AQPX1|PSEC0027
0006810 // tra	0016021 // in	0015250 // wa	Aquaporin-3	AQP3_HUMAN|AQP3
0007588 // ex	0016324 // ap	0015112 // ni	Aquaporin-6	AQP6_HUMAN|AQP6|AQP2L
0006091 // ge	0005886 // pl	0015250 // wa	Aquaporin-7	AQP7_HUMAN|AQP7|AQP7L|AQP9
0006810 // tra	0016021 // in	0005215 // tra	Putative aqua	AQP73_HUMAN|AQP7P3
0032434 // re	0005829 // cy	0005515 // pr	Serine/threon	ARAF_HUMAN|ARAF|ARAF1|PKS|PKS2
0051270 // re	0005829 // cy	0005096 // GT	Arf-GAP with	ARAP1_HUMAN|ARAP1|CENTD2|KIAA0782
0043547 // po	0005829 // cy	0005547 // ph	Arf-GAP with	ARAP2_HUMAN|ARAP2|CENTD1|KIAA0580
0008360 // re	0005737 // cy	0043325 // ph	Arf-GAP with	ARAP3_HUMAN|ARAP3|CENTD3
0009952 // an	0014069 // po		Activity-regula	ARC_HUMAN|ARC|KIAA0278
0051384 // re	0005789 // en	0005154 // ep	Amphiregulin	AREG_HUMAN|AREG|AREGB|SDGF
2000377 // re	0070062 // ex	0005086 // AF	ADP-ribosylat	ARF4_HUMAN|ARF4|ARF2
0097284 // he	0005768 // en	0005515 // pr	ADP-ribosylat	ARF6_HUMAN|ARF6
0006888 // EF	0014069 // po	0005515 // pr	ADP-ribosylat	ARFG1_HUMAN|ARFGAP1|ARF1GAP
0006886 // in	0005794 // Go	0008565 // pr	ADP-ribosylat	ARFG3_HUMAN|ARFGAP3|ARFGAP1
0032012 // re	0016021 // in	0005086 // AF	Brefeldin A-in	BIG3_HUMAN|ARFGEF3|BIG3|C6orf92|KIAA1244
0007264 // sm	0005737 // cy	0005515 // pr	Arfaptin-2	ARFP2_HUMAN|ARFIP2|POR1
0043001 // Go	0005794 // Go	0005525 // GT	ADP-ribosylat	ARFRP_HUMAN|ARFRP1|ARP1
0006809 // ni	0005739 // m	0004053 // ar	Arginase-2, m	ARGI2_HUMAN|ARG2
0021987 // ce	0005829 // cy	0005096 // GT	Rho GTPase-a	RHGBB_HUMAN|ARHGAP11B|FAM7B1
0043547 // po	0005886 // pl	0005096 // GT	Rho GTPase-a	RHG17_HUMAN|ARHGAP17|RICH1|MSTP066|MSTP110
0043547 // po	0005829 // cy	0005096 // GT	Rho GTPase-a	RHG18_HUMAN|ARHGAP18
0051684 // m	0005794 // Go	0005515 // pr	Rho GTPase-a	RHG21_HUMAN|ARHGAP21|ARHGAP10|KIAA1424
0043547 // po	0005925 // fo	0005515 // pr	Rho GTPase-a	RHG22_HUMAN|ARHGAP22|RHOGAP2
0051056 // re	0005829 // cy	0005096 // GT	Rho GTPase-a	RHG23_HUMAN|ARHGAP23|KIAA1501
0043547 // po	0042995 // ce	0005515 // pr	Rho GTPase-a	RHG24_HUMAN|ARHGAP24|FILGAP
0006911 // ph	0005829 // cy	0005096 // GT	Rho GTPase-a	RHG25_HUMAN|ARHGAP25|KIAA0053
0006898 // re	0016020 // m	0005096 // GT	Rho GTPase-a	RHG27_HUMAN|ARHGAP27|CAMGAP1|SH3D20|PP905
0043547 // po	0005737 // cy	0005096 // GT	Rho GTPase-a	RHG29_HUMAN|ARHGAP29|PARG1
0043547 // po	0043231 // in	0005096 // GT	Rho GTPase-a	RHG30_HUMAN|ARHGAP30
0043547 // po	0030027 // la	0005096 // GT	Rho GTPase-a	RHG31_HUMAN|ARHGAP31|CDGAP|KIAA1204
0043547 // po	0014069 // po	0005515 // pr	Rho GTPase-a	RHG32_HUMAN|ARHGAP32|GRIT|KIAA0712|RICS
0009967 // po	0005874 // m	0005070 // SH	Rho GTPase-a	RHG04_HUMAN|ARHGAP4|KIAA0131|RGC1|RHOGAP4
0043547 // po	0005829 // cy	0005096 // GT	Rho GTPase-a	RHG40_HUMAN|ARHGAP40|C20orf95

0061001 // re	0030054 // ce	0005096 // G	Rho GTPase-a	RHG44_HUMAN|ARHGAP44|KIAA0672|RICH2
0043547 // pc	0005576 // ex	0005096 // G	Rho GTPase-a	HMHA1_HUMAN|ARHGAP45|HMHA1|KIAA0223
0007155 // ce	0016020 // m	0003924 // G	Rho GTPase-a	RHG05_HUMAN|ARHGAP5|RHOGAP5
0009967 // pc	0005884 // ac	0005070 // SH	Rho GTPase-a	RHG06_HUMAN|ARHGAP6|RHOGAP6
0043547 // pc	0005737 // cy	0005515 // pr	Rho GTPase-a	RHG08_HUMAN|ARHGAP8
0043547 // pc	0034774 // se	0005096 // G	Rho GTPase-a	RHG09_HUMAN|ARHGAP9
0071526 // se	0005829 // cy	0005515 // pr	Rho GDP-diss	GDIR1_HUMAN|ARHGDIA|GDIA1
0043547 // pc	0005737 // cy	0005089 // Rh	Rho guanine r	ARGAL_HUMAN|ARHGEF10L|GRINCHGEF|KIAA1626
0035023 // re	0005829 // cy	0005089 // Rh	Rho guanine r	ARHGG_HUMAN|ARHGEF16|EPHEXIN4|NBR
0043547 // pc	0005829 // cy	0005089 // Rh	Rho guanine r	ARHGI_HUMAN|ARHGEF18|KIAA0521
0043547 // pc	0005829 // cy	0005515 // pr	Rho guanine r	ARHGJ_HUMAN|ARHGEF19
0043547 // pc	0030016 // m	0005089 // Rh	Rho guanine r	ARHGP_HUMAN|ARHGEF25|GEFT
0097178 // ru	0001726 // ru	0005089 // Rh	Rho guanine r	ARHGQ_HUMAN|ARHGEF26|SGEF|HMFN1864
0048013 // ep	0005886 // pl	0005089 // Rh	Rho guanine r	ARG28_HUMAN|ARHGEF28|KIAA1998|RGNEF
0043547 // pc	0005829 // cy	0005515 // pr	Rho guanine r	ARHG3_HUMAN|ARHGEF3
0035023 // re	0005737 // cy	0005089 // Rh	Rho guanine r	ARH37_HUMAN|ARHGEF37
0043547 // pc	0005829 // cy	0005085 // gu	Rho guanine r	ARHG4_HUMAN|ARHGEF4|KIAA1112
0043547 // pc	0005737 // cy	0005089 // Rh	Rho guanine r	ARH40_HUMAN|ARHGEF40|SOLO
0071803 // pc	0005634 // nu	0005515 // pr	Rho guanine r	ARHG5_HUMAN|ARHGEF5|TIM
0043547 // pc	0005829 // cy	0005089 // Rh	Rho guanine r	ARHG9_HUMAN|ARHGEF9|ARHDH9|KIAA0424
0006977 // DI	0005634 // nu	0005515 // pr	AT-rich intera	ARI3A_HUMAN|ARID3A|DRIL1|DRIL3|DRX|E2FBP1
0006357 // re	0005634 // nu	0005515 // pr	AT-rich intera	ARI4B_HUMAN|ARID4B|BRCAA1|RBBP1L1|RBP1L1|SAP
0045087 // in	0005730 // nu	0003677 // DI	AT-rich intera	ARI5A_HUMAN|ARID5A|MRF1
0016577 // hi	0005634 // nu	0044212 // tra	AT-rich intera	ARI5B_HUMAN|ARID5B|DESRT|MRF2
0007264 // sn	0005622 // in	0005525 // G	ADP-ribosylat	ARL10_HUMAN|ARL10|ARL10A
0007264 // sn	0005622 // in	0005515 // pr	ADP-ribosylat	ARL11_HUMAN|ARL11|ARLTS1
0007264 // sn	0031410 // cy	0005525 // G	ADP-ribosylat	ARL14_HUMAN|ARL14|ARF7
	0005730 // nu	0005515 // pr	ARL14 effecto	AL14E_HUMAN|ARL14EP|ARF7EP|C11orf46
0008150 // bi	0070062 // ex	0005525 // G	ADP-ribosylat	ARL15_HUMAN|ARL15|ARFRP2
0007264 // sn	0005622 // in	0005515 // pr	ADP-ribosylat	ARL16_HUMAN|ARL16
0016192 // ve	0005794 // Go	0005525 // G	ADP-ribosylat	ARL17_HUMAN|ARL17A|ARL17B|ARF1P2|ARL17A|ARL1
0007098 // ce	0005758 // m	0005515 // pr	ADP-ribosylat	ARL2_HUMAN|ARL2
0000281 // m	0005929 // cil	0005515 // pr	ADP-ribosylat	ARL3_HUMAN|ARL3|ARFL3
0050873 // br	0005634 // nu	0005515 // pr	ADP-ribosylat	ARL4A_HUMAN|ARL4A|ARL4
0032456 // er	0030175 // fil	0005525 // G	ADP-ribosylat	ARL4C_HUMAN|ARL4C|ARL7
0009306 // pr	0005730 // nu	0005515 // pr	ADP-ribosylat	ARL4D_HUMAN|ARL4D|ARF4L
0043066 // ne	0005829 // cy	0005515 // pr	ADP-ribosylat	AR6P1_HUMAN|ARL6IP1|ARL6IP|ARMER|KIAA0069
0008380 // RI	0005730 // nu	0003723 // RI	ADP-ribosylat	AR6P4_HUMAN|ARL6IP4
	0005925 // fo		Armadillo rep	ARMC5_HUMAN|ARMC5
0002244 // he	0005829 // cy		Armadillo rep	ARMC6_HUMAN|ARMC6
	0005737 // cy	0005515 // pr	Armadillo rep	ARMC7_HUMAN|ARMC7
	0070062 // ex		LisH domain-c	ARMC9_HUMAN|ARMC9|KIAA1868
	0016021 // in	0005515 // pr	Armadillo rep	ARMX1_HUMAN|ARMCX1|ALEX1|AD032

	0016021 // in		Armadillo rep	ARMX2_HUMAN|ARMCX2|ALEX2|KIAA0512
	0016021 // in		Armadillo rep	ARMX4_HUMAN|ARMCX4
		0005515 // pr	Armadillo rep	ARMX5_HUMAN|ARMCX5
	0016021 // in		Protein ARMC	ARMX6_HUMAN|ARMCX6
0001938 // pc	0005654 // nu	0005515 // pr	Aryl hydrocarl	ARNT_HUMAN|ARNT|BHLHE2
0043066 // ne	0005667 // tra	0017162 // ar	Aryl hydrocarl	ARNT2_HUMAN|ARNT2|BHLHE1|KIAA0307
0032922 // cir	0016605 // PN	0017162 // ar	Aryl hydrocarl	BMAL1_HUMAN|ARNTL|BHLHE5|BMAL1|MOP3|PASD3
0048013 // ep	0005829 // cy	0005515 // pr	Actin-related	ARC1B_HUMAN|ARPC1B|ARC41
0048013 // ep	0005829 // cy	0005515 // pr	Actin-related	ARPC3_HUMAN|ARPC3|ARC21
0034314 // Ar	0005885 // Ar	0005524 // AT	Protein ARPC4	A0A0A6YYG9_HUMAN|ARPC4-TTLL3
0030336 // ne	0030027 // la	0005515 // pr	Arpin	ARPIN_HUMAN|ARPIN|C15orf38
0043161 // pr	0005829 // cy	0005096 // GT	Beta-arrestin-	ARRB1_HUMAN|ARRB1|ARR1
0016567 // pr	0005829 // cy	0001664 // G-	Beta-arrestin-	ARRB2_HUMAN|ARRB2|ARB2|ARR2
	0070062 // ex	0005515 // pr	Arrestin doma	ARRD1_HUMAN|ARRDC1
0051443 // pc	0005769 // ea	0005515 // pr	Arrestin doma	ARRD4_HUMAN|ARRDC4
0043687 // pc	0005788 // er	0004065 // ar	Arylsulfatase	ARSD_HUMAN|ARSD
0001501 // sk	0005788 // er	0046872 // m	Arylsulfatase	ARSE_HUMAN|ARSE
0043687 // pc	0005788 // er	0004065 // ar	Arylsulfatase	ARSG_HUMAN|ARSG|KIAA1001|UNQ839/PRO1777
0043687 // pc	0005788 // er	0004065 // ar	Arylsulfatase	ARSI_HUMAN|ARSI
0043687 // pc	0005788 // er	0004065 // ar	Arylsulfatase	ARSJ_HUMAN|ARSJ|UNQ372/PRO708
0043547 // pc	0005576 // ex	0008083 // gr	Artemin	ARTN_HUMAN|ARTN|EVN
0090181 // re	0005789 // er	0015248 // st	Protein ARV1	ARV1_HUMAN|ARV1|HT035
0016339 // ca	0005886 // pl	0005515 // pr	Armadillo rep	ARVC_HUMAN|ARVCF
0018872 // ar	0005829 // cy	0030791 // ar	Arsenite meth	AS3MT_HUMAN|AS3MT|CYT19
0006687 // gl	0070062 // ex	0003824 // ca	Acid ceramida	ASAH1_HUMAN|ASAH1|ASAH|HSD-33|HSD33
0061000 // ne	0005829 // cy	0005515 // pr	Arf-GAP with	ASAP1_HUMAN|ASAP1|DDEF1|KIAA1249
0030539 // m	0005829 // cy	0003674 // m	Ankyrin repea	ASB1_HUMAN|ASB1|KIAA1146
0035556 // in	0005829 // cy	0005515 // pr	Ankyrin repea	ASB13_HUMAN|ASB13
0035556 // in	0000151 // ub	0031625 // ub	Ankyrin repea	ASB15_HUMAN|ASB15
0035556 // in	0000151 // ub	0031625 // ub	Ankyrin repea	ASB16_HUMAN|ASB16
0045445 // m	0005829 // cy	0005515 // pr	Ankyrin repea	ASB2_HUMAN|ASB2
0051865 // pr	0031462 // Cu	0031625 // ub	Ankyrin repea	ASB4_HUMAN|ASB4
0035556 // in	0005829 // cy	0005515 // pr	Ankyrin repea	ASB9_HUMAN|ASB9
0003358 // nc	0043025 // ne	0001078 // tra	Achaete-scute	ASCL1_HUMAN|ASCL1|ASH1|BHLHA46|HASH1
0006357 // re	0090575 // RN	0000977 // RN	Achaete-scute	ASCL5_HUMAN|ASCL5|BHLHA47
0031668 // ce	0005887 // in	0005515 // pr	Asialoglycopro	ASGR1_HUMAN|ASGR1|CLEC4H1
0097676 // hi	0005794 // Go	0003677 // DN	Histone-lysine	ASH1L_HUMAN|ASH1L|KIAA1420|KMT2H
0043627 // re	0044666 // M	0044212 // tra	Set1/Ash2 his	ASH2L_HUMAN|ASH2L|ASH2L1
0007600 // se	0005886 // pl	0005261 // ca	Acid-sensing i	ASIC3_HUMAN|ASIC3|ACCN3|SLNAC1|TNAC1
0015992 // pr	0016021 // in	0015252 // hy	Acid-sensing i	ASIC5_HUMAN|ASIC5|ACCN5|HINAC
0045931 // pc	0005829 // cy	0048037 // co	Asparagine sy	ASNS_HUMAN|ASNS|TS11
0006541 // gl	0005575 // ce	0004066 // as	Asparagine sy	ASND1_HUMAN|ASNSD1|NS3TP1|Nbla00058

0006530 // as	0005829 // cy	0003847 // 1-	60 kDa lysoph	LPP60_HUMAN	ASPG|C14orf76
0018193 // pe	0016021 // int	0051213 // di	Aspartate bet	ASPH1_HUMAN	ASPHD1
0018193 // pe	0016021 // int	0051213 // di	Aspartate bet	ASPH2_HUMAN	ASPHD2
0090263 // po	0097431 // m	0005515 // pr	Abnormal spir	ASPM_HUMAN	ASPM|MCPH5
0006886 // in	0019898 // ex	0005515 // pr	Tether contai	ASPC1_HUMAN	ASPSCR1|ASPL|RCC17|TUG|UBXD9|UB
0006559 // L-	0005829 // cy	0004067 // as	Isoaspartyl pe	ASGL1_HUMAN	ASRGL1|ALP|CRASH
0071499 // ce	0005737 // cy	0003723 // RN	Argininosucci	ASSY_HUMAN	ASS1|ASS
0006351 // tra	0005654 // nu	0003677 // DN	Putative Polyc	ASXL2_HUMAN	ASXL2|ASXH2|KIAA1685
0045944 // po	0005654 // nu	0005524 // AT	ATPase family	ATAD2_HUMAN	ATAD2|L16|PRO2000
0016049 // ce	0005739 // m	0005524 // AT	ATPase family	ATD3A_HUMAN	ATAD3A
0043312 // ne	0005886 // pl	0005524 // AT	ATPase family	ATD3B_HUMAN	ATAD3B|KIAA1273|TOB3
	0005739 // m	0005524 // AT	ATPase family	ATD3C_HUMAN	ATAD3C
0045598 // re	0005794 // Go	0019799 // tu	Alpha-tubulin	ATAT_HUMAN	ATAT1|C6orf134|MEC17|Nbla00487
0061394 // re	0005634 // nu	0042802 // id	Cyclic AMP-de	ATF3_HUMAN	ATF3
0010575 // po	0034399 // nu	0005515 // pr	Cyclic AMP-de	ATF4_HUMAN	ATF4|CREB2|TXREB
0009791 // po	0005829 // cy	0000977 // RN	Cyclic AMP-de	ATF5_HUMAN	ATF5|ATFX
0006366 // tra	0030176 // int	0001205 // tra	Cyclic AMP-de	ATF6B_HUMAN	ATF6B|CREBL1|G13
0016032 // vi	0016604 // nu	0003714 // tra	Activating tra	MCAF1_HUMAN	ATF7IP|MCAF|MCAF1
0006351 // tra	0005634 // nu		Activating tra	MCAF2_HUMAN	ATF7IP2|MCAF2
0001934 // po	0005776 // au	0005515 // pr	Beclin 1-assoc	BAKOR_HUMAN	ATG14|ATG14L|KIAA0831
0000045 // au	0000421 // au		Autophagy-re	A16L2_HUMAN	ATG16L2|WDR80
0000045 // au	0019898 // ex		Autophagy-re	ATG2B_HUMAN	ATG2B|C14orf103
0016236 // m	0034774 // se	0005515 // pr	Ubiquitin-like	ATG7_HUMAN	ATG7|APG7L
0000422 // m	0016021 // int		Autophagy-re	ATG9B_HUMAN	ATG9B|APG9L2|NOS3AS
0009168 // pu	0070062 // ex	0042803 // pr	Bifunctional p	PUR9_HUMAN	ATIC|PURH|OK/SW-cl.86
0051260 // pr	0000137 // Go	0042802 // id	Atlastin-1	ATLA1_HUMAN	ATL1|GBP3|SPG3A
0051260 // pr	0016021 // int	0005525 // GT	Atlastin-2	ATLA2_HUMAN	ATL2|ARL6IP2
0021554 // op	0005634 // nu	0003677 // DN	Protein atona	ATOH7_HUMAN	ATOH7|ATH5|BHLHA13
0045892 // ne	0005634 // nu	0070888 // E-	Protein atona	ATOH8_HUMAN	ATOH8|ATH6|BHLHA21
0006878 // ce	0005829 // cy	0005507 // co	Copper transp	ATOX1_HUMAN	ATOX1|HAH1
0006812 // ca	0005886 // pl	0005515 // pr	Probable phos	AT10D_HUMAN	ATP10D|ATPVD|KIAA1487
0006811 // io	0005769 // ea	0004012 // ph	Probable phos	AT11B_HUMAN	ATP11B|ATPIF|ATPIR|KIAA0956
0034220 // io	0016021 // int	0000287 // m	Phospholipid-	AT11C_HUMAN	ATP11C|ATPIG|ATPIQ
0055069 // zi	0030133 // tra	0005515 // pr	Cation-transp	AT132_HUMAN	ATP13A2|PARK9
0099132 /// 0	0016020 // m	0005524 // AT	Probable catio	AT133_HUMAN	ATP13A3|AFURS1
0099132 /// 0	0005886 // pl	0005524 // AT	Probable catio	AT134_HUMAN	ATP13A4|UNQ3052/PRO9871
0086064 // ce	1903561 // ex	0005391 // so	Sodium/potas	AT1A3_HUMAN	ATP1A3
1903281 // po	0005886 // pl	0008022 // pr	Sodium/potas	AT1B1_HUMAN	ATP1B1|ATP1B
0010248 // es	0005886 // pl	0005391 // so	Sodium/potas	AT1B3_HUMAN	ATP1B3
0032469 // en	0031095 // pl	0005388 // ca	Sarcoplasmic/	AT2A2_HUMAN	ATP2A2|ATP2B
0099132 /// 0	0031095 // pl	0005388 // ca	Sarcoplasmic/	AT2A3_HUMAN	ATP2A3
0099132 /// 0	0009898 // cy	0030165 // PD	Plasma memb	AT2B1_HUMAN	ATP2B1|PMCA1

0051001 // ne	0005886 // pl	0030165 // PD	Plasma memb	AT2B4_HUMAN|ATP2B4|ATP2B2|MXRA1
0099132 /// 0	0000139 // Go	0005388 // ca	Calcium-trans	AT2C1_HUMAN|ATP2C1|KIAA1347|PMR1L|HUSSY-28
0099132 /// 0	0000139 // Go	0005515 // pr	Calcium-trans	AT2C2_HUMAN|ATP2C2|KIAA0703|SPCA2
0006813 // po	0005615 // ex	0000287 // m	Potassium-tra	ATP4A_HUMAN|ATP4A
0099132 /// 0	0000275 // m	0016887 // AT	ATP synthase	ATP5E_HUMAN|ATP5E
0006754 // AT	0005743 // m	0016887 // AT	ATP synthase	ATPK_HUMAN|ATP5J2|ATP5JL
0060323 // he	0005886 // pl	0005515 // pr	Renin recepto	A0A1C7CYW4_HUMAN|ATP6AP2
0015991 // AT	0010008 // en	0008553 // hy	V-type proton	VA0D2_HUMAN|ATP6V0D2
0015992 // pr	0010008 // en	0005215 // tra	V-type proton	VA0E1_HUMAN|ATP6V0E1|ATP6H|ATP6V0E
0015991 // AT	0010008 // en	0046961 // pr	V-type proton	VA0E2_HUMAN|ATP6V0E2|ATP6V0E2L|C7orf32
0006885 // re	0005737 // cy	0032403 // pr	V-type proton	VATB1_HUMAN|ATP6V1B1|ATP6B1|VATB|VPP3
0090383 // ph	0005829 // cy	0008553 // hy	V-type proton	VATC2_HUMAN|ATP6V1C2
0015992 // pr	0005829 // cy	0005515 // pr	V-type proton	VATD_HUMAN|ATP6V1D|ATP6M|VATD
0015992 // pr	0005829 // cy	0016820 // hy	V-type proton	VATG2_HUMAN|ATP6V1G2|ATP6G|ATP6G2|NG38
0015992 // pr	0016471 // va	0016820 // hy	V-type proton	F2Z307_HUMAN|ATP6V1G2-DDX39B
0099132 /// 0	0005794 // Go	0005507 // co	Copper-transp	ATP7B_HUMAN|ATP7B|PWD|WC1|WND
0099132 /// 0	0000139 // Go	0019829 // ca	Phospholipid-	AT8A1_HUMAN|ATP8A1|ATPIA
0007409 // ax	0016021 // in	0000287 // m	Phospholipid-	AT8A2_HUMAN|ATP8A2|ATPIB
0015917 // an	0005886 // pl	0004012 // ph	Phospholipid-	AT8B1_HUMAN|ATP8B1|ATPIC|FIC1|PFIC
0007030 // Go	0016021 // in	0000287 // m	Phospholipid-	AT8B2_HUMAN|ATP8B2|ATPID|KIAA1137
0007030 // Go	0005789 // en	0000287 // m	Phospholipid-	AT8B3_HUMAN|ATP8B3|ATP1K|FOS37502_2
0045332 // ph	0016021 // in	0000287 // m	Probable phos	ATP9A_HUMAN|ATP9A|ATPIIA|KIAA0611
1900034 // re	0005794 // Go	0032407 // M	Serine/threon	ATR_HUMAN|ATR|FRP1
0030501 // po	0005765 // lys	0005515 // pr	All-trans retin	ARAID_HUMAN|ATRAID|APR3|C2orf28|HSPC013|UNQ2
1904908 // ne	0005720 // nu	0005515 // pr	Transcriptiona	ATRX_HUMAN|ATRX|RAD54L|XH2
0007268 // ch	0005829 // cy	0036459 // th	Ataxin-3	ATX3_HUMAN|ATXN3|ATX3|MJD|MJD1|SCA3
		0005515 // pr	Ataxin-7-like p	AT7L1_HUMAN|ATXN7L1|ATXN7L4|KIAA1218
0006635 // fa	0005759 // m	0003730 // m	Methylglutaco	AUHM_HUMAN|AUH
0006397 // m	0005743 // m	0005515 // pr	Aurora kinase	AKIP_HUMAN|AURKAIP1|AIP|AKIP|MRPS38
0006915 // ap	0005622 // in	0005515 // pr	Cell death reg	AVEN_HUMAN|AVEN
0007010 // cy	0005737 // cy	0003779 // ac	Advillin	AVIL_HUMAN|AVIL
0007049 // ce		0005515 // pr	Arginine vaso	AVPI1_HUMAN|AVPI1|NPD013|PP5395
0042476 // od	0005634 // nu	0005515 // pr	Axin-2	AXIN2_HUMAN|AXIN2
1902269 // po	1990005 // gr	0008792 // ar	Antizyme inhi	AZIN2_HUMAN|AZIN2|ADC|KIAA1945|ODCP
0001774 // m	0005615 // ex	0008201 // he	Azurocidin	CAP7_HUMAN|AZU1
0006493 // pr	0016021 // in	0008376 // ac	UDP-GalNAc:b	B3GL2_HUMAN|B3GALNT2
0006486 // pr	0016021 // in	0047915 // ga	Beta-1,3-galac	B3GT4_HUMAN|B3GALT4|GALT4
0006486 // pr	0016021 // in	0008499 // UI	Beta-1,3-galac	B3GT5_HUMAN|B3GALT5

0030206 // ch	0016021 // in	0047220 // ga	Beta-1,3-galac	B3GT6_HUMAN|B3GALT6
0018146 // ke	0000139 // Go	0008532 // N-	N-acetyllactos	B3GN3_HUMAN|B3GNT3|B3GALT8|TMEM3|UNQ637/P
0007420 // br	0016021 // in	0047256 // lac	Lactosylceram	B3GN5_HUMAN|B3GNT5
0018146 // ke	0016021 // in	0008532 // N-	UDP-GlcNAc:b	B3GN7_HUMAN|B3GNT7
0016266 // O-	0016021 // in	0016262 // pr	UDP-GlcNAc:b	B3GN8_HUMAN|B3GNT8|B3GALT7|BGALT15
0006486 // pr	0005794 // Go	0008378 // ga	UDP-GlcNAc:b	B3GN9_HUMAN|B3GNT9|UNQ1922/PRO4397
		0016757 // tra	UDP-GlcNAc:b	B3GNL_HUMAN|B3GNTL1|B3GNT8
0001574 // ga	0000139 // Go	0003947 // (N	Beta-1,4 N-ac	B4GN1_HUMAN|B4GALNT1|GALGT|SIAT2
	0016021 // in	0033842 // N-	N-acetyl-beta-	B4GN4_HUMAN|B4GALNT4
0060046 // re	0005794 // Go	0003831 // be	Beta-1,4-galac	B4GT1_HUMAN|B4GALT1|GGTB2
0005975 // ca	0005794 // Go	0003831 // be	Beta-1,4-galac	B4GT3_HUMAN|B4GALT3
0005975 // ca	0000139 // Go	0003831 // be	Beta-1,4-galac	B4GT6_HUMAN|B4GALT6
0005975 // ca	0016021 // in	0008378 // ga	Beta-1,4-galac	B4GT7_HUMAN|B4GALT7|XGALT1|UNQ748/PRO1478
0007411 // ax	0070062 // ex	0008532 // N-	Beta-1,4-glucu	B4GA1_HUMAN|B4GAT1|B3GNT1|B3GNT6
0031100 // ar	0005829 // cy	0016746 // tra	Bile acid-CoA:	BAAT_HUMAN|BAAT
0051301 // ce	0005737 // cy	0005515 // pr	BRISC and BRC	BABA1_HUMAN|BABAM1|C19orf62|MERIT40|NBA1|HS
0030163 // pr	0010008 // en	0008798 // be	Beta-secretase	BACE1_HUMAN|BACE1|BACE|KIAA1149
0050435 // be	0009986 // ce	0004190 // as	Beta-secretase	BACE2_HUMAN|BACE2|AEPLC|ALP56|ASP21|CDA13|UI
0006366 // tra	0031463 // Cu	0001078 // tra	Transcription	BACH1_HUMAN|BACH1
1900034 // re	0005829 // cy	0005515 // pr	BAG family m	BAG1_HUMAN|BAG1|HAP
0007420 // br	0005829 // cy	0005515 // pr	BAG family m	BAG3_HUMAN|BAG3|BIS
1900034 // re	0005829 // cy	0005515 // pr	BAG family m	BAG5_HUMAN|BAG5|KIAA0873
	0005576 // ex		B melanoma a	BAGE2_HUMAN|BAGE2
0006342 // ch	0000785 // ch	0003682 // ch	BAH and coile	BAHC1_HUMAN|BAHCC1|BAHD2|KIAA1447
0032956 // re	0030175 // fil	0005515 // pr	Brain-specific	BAIP2_HUMAN|BAIAP2
0046626 // re	0070062 // ex	0098641 // ca	Brain-specific	BI2L1_HUMAN|BAIAP2L1|IRTKS
2000251 // po	0005829 // cy	0005543 // ph	Brain-specific	BI2L2_HUMAN|BAIAP2L2|UNQ9336/PRO34007
0007269 // ne	0098793 // pr	0008022 // pr	BAI1-associate	BAIP3_HUMAN|BAIAP3|KIAA0734
0008284 // po	0016021 // in	0005114 // ty	BMP and activ	BAMBI_HUMAN|BAMBI|NMA
0045947 // ne		0005102 // re	B-cell scaffold	BANK1_HUMAN|BANK1
0006357 // re	0005634 // nu	0003700 // tra	Homeobox pr	BARX1_HUMAN|BARX1
0000122 // ne	0005829 // cy	0000977 // RN	Homeobox pr	BARX2_HUMAN|BARX2
0060421 // po	0005737 // cy	0003714 // tra	Brain acid solu	BASP1_HUMAN|BASP1|NAP22
0006974 // ce	0005654 // nu	0005515 // pr	Basic leucine z	BATF_HUMAN|BATF
0006351 // tra	0005634 // nu	0003677 // DN	Basic leucine z	BATF2_HUMAN|BATF2
0006977 // DN	0005741 // m	0005515 // pr	Apoptosis reg	BAX_HUMAN|BAX|BCL2L4
0006306 // DN	0005677 // ch	0003723 // RN	Bromodomain	BAZ2A_HUMAN|BAZ2A|KIAA0314|TIP5
0006355 // re	0005634 // nu	0005515 // pr	Bromodomain	BAZ2B_HUMAN|BAZ2B|KIAA1476
0044458 // m	0005737 // cy		Basal body-or	BBOF1_HUMAN|BBOF1|C14orf45|CCDC176
0045329 // ca	0070062 // ex	0008336 // ga	Gamma-butyr	BODG_HUMAN|BBOX1|BBH|BBOX
0043001 // Go	0005829 // cy	0005113 // pa	Bardet-Biedl s	BBS1_HUMAN|BBS1|BBS2L2
0044458 // m	0036064 // cil	0005515 // pr	Bardet-Biedl s	BBS5_HUMAN|BBS5
0007155 // ce	0005887 // in	0004888 // tra	Basal cell adh	BCAM_HUMAN|BCAM|LU|MSK19
0007155 // ce	0005796 // Go	0005540 // hy	Brevican core	PGCB_HUMAN|BCAN|BEHAB|CSPG7|UNQ2525/PRO601
0006886 // in	0016021 // in		B-cell recepto	BAP29_HUMAN|BCAP29|BAP29
0009967 // po	0005622 // in	0005515 // pr	Breast cancer	BCAR3_HUMAN|BCAR3|NSP2|SH2D3B|UNQ271/PRO30

	0070062 // ex		Breast carcinc	BCAS1_HUMAN|BCAS1|AIBC1|NABC1
0000375 // RI	0005654 // nu	0005515 // pr	Pre-mRNA-spl	SPF27_HUMAN|BCAS2|DAM1
0046907 // in	0031083 // BL		Breast carcinc	BCAS4_HUMAN|BCAS4
0009099 // va	0005829 // cy	0052654 // L-I	Branched-cha	BCAT1_HUMAN|BCAT1|BCT1|ECA39
0009083 // br	0005739 // m	0005515 // pr	2-oxoisovalera	ODBA_HUMAN|BCKDHA
0009083 // br	0005759 // m	0005515 // pr	2-oxoisovalera	ODBB_HUMAN|BCKDHB
0009083 // br	0005739 // m	0004674 // pr	[3-methyl-2-o	BCKD_HUMAN|BCKDK
0002223 // sti	0005886 // pl	0005515 // pr	B-cell lympho	BCL10_HUMAN|BCL10|CIPER|CLAP
0021773 // sti	0043005 // ne	0005515 // pr	B-cell lympho	BC11B_HUMAN|BCL11B|CTIP2|RIT1
0022612 // gl	0005634 // nu	0005515 // pr	Apoptosis reg	BCL2_HUMAN|BCL2
0008053 // m	0005741 // m	0005515 // pr	Bcl-2-related	B2LA1_HUMAN|BCL2A1|BCL2L5|BFL1|GRS|HBPA1
0008283 // ce	0005741 // m	0005515 // pr	Bcl-2-like prot	B2CL1_HUMAN|BCL2L1|BCL2L|BCLX
0008630 // in	0005741 // m	0005515 // pr	Bcl-2-like prot	B2L10_HUMAN|BCL2L10|BCLB
0042981 // re	0005829 // cy	0005515 // pr	Apoptosis faci	B2L14_HUMAN|BCL2L14|BCLG
0042981 // re	0005634 // nu	0005515 // pr	Bcl-2-like prot	B2L15_HUMAN|BCL2L15|C1orf178
0045892 // ne	0005634 // nu	0030674 // pr	B-cell lympho	BCL3_HUMAN|BCL3|BCL4|D19S37
0042127 // re	0005654 // nu	0001227 // tr	B-cell lympho	BCL6_HUMAN|BCL6|BCL5|LAZ3|ZBTB27|ZNF51
0035019 // so	0005654 // nu	0005515 // pr	B-cell CLL/lym	BCL9_HUMAN|BCL9
0030512 // ne	0005654 // nu	0003713 // tr	B-cell CLL/lym	BCL9L_HUMAN|BCL9L|DLNB11
1990830 // ce	0005654 // nu	0003723 // RI	Bcl-2-associat	BCLF1_HUMAN|BCLAF1|BTF|KIAA0164
0042472 // in	0005829 // cy	0005515 // pr	Breakpoint clu	BCR_HUMAN|BCR|BCR1|D22S11
0032981 // m	0005750 // m	0005524 // AT	Mitochondria	BCS1_HUMAN|BCS1L|BCS1
0002687 // po	0005886 // pl	0004947 // br	B1 bradykinin	BKRB1_HUMAN|BDKRB1|BRADYB1
0008015 // bl	0005886 // pl	0004435 // ph	B2 bradykinin	BKRB2_HUMAN|BDKRB2|BKR2
2000008 // re	0005739 // m	0005169 // ne	Brain-derived	BDNF_HUMAN|BDNF
0006359 // re	0005654 // nu	0001026 // TF	Transcription	BDP1_HUMAN|BDP1|KIAA1241|KIAA1689|TFNR
	0016021 // in		Protein BEAN1	BEAN1_HUMAN|BEAN1
	0016020 // m	0005515 // pr	Brain-enriche	BEGIN_HUMAN|BEGAIN|KIAA1446
0036124 // hi	0005720 // nu	0005515 // pr	BEN domain-c	BEND3_HUMAN|BEND3|KIAA1553
0000122 // ne	0005634 // nu	0003682 // ch	BEN domain-c	BEND6_HUMAN|BEND6|C6orf65
	0070062 // ex	0005515 // pr	BEN domain-c	BEND7_HUMAN|BEND7|C10orf30
0007601 // vis	0016323 // ba	0015106 // bi	Bestrophin-1	BEST1_HUMAN|BEST1|VMD2
1902476 // ch	0005886 // pl	0005254 // ch	Bestrophin-4	BEST4_HUMAN|BEST4|VMD2L2
0042147 // re	0000139 // Go	0005484 // SN	BET1-like prot	BET1L_HUMAN|BET1L|GS15
0051726 // re	0005634 // nu	0005515 // pr	Protein BEX2	BEX2_HUMAN|BEX2
0007275 // m	0005829 // cy	0005515 // pr	Protein BEX3	BEX3_HUMAN|BEX3|DXS6984E|NADE|NGFRAP1
0007010 // cy	0005938 // ce	0005515 // pr	Filensin	BFSP1_HUMAN|BFSP1
0071305 // ce	0005791 // ro	0005509 // ca	Osteocalcin	OSTCN_HUMAN|BGLAP
0019722 // ca	0005634 // nu	0001228 // tr	Class A basic h	BHA15_HUMAN|BHLHA15|BHLHB8|MIST1
0008283 // ce	0005634 // nu	0042826 // hi	Class E basic h	BHE41_HUMAN|BHLHE41|BHLHB3|DEC2|SHARP1
0000096 // su	0070062 // ex	0008270 // zi	Betaine--hom	BHMT1_HUMAN|BHMT
0007368 // de	0005737 // cy	0003723 // RI	Protein bicau	BICC1_HUMAN|BICC1

0016032 // vi	0005829 // cy	0034452 // dy	Protein bicau	BICD1_HUMAN|BICD1
0000042 // pr	0005794 // G	0005515 // pr	Protein bicau	BICD2_HUMAN|BICD2|KIAA0699
0055107 // G	0005737 // cy	0034452 // dy	BICD family-li	BICL1_HUMAN|BICDL1|BICDR1|CCDC64
		0017137 // Ra	BICD family-li	BICL2_HUMAN|BICDL2|BICDR2|CCDC64B
0007283 // sp	0016021 // in	0005515 // pr	Bcl-2-interacti	BIK_HUMAN|BIK|NBK
0008283 // ce	0031674 // I b	0005515 // pr	Myc box-depe	BIN1_HUMAN|BIN1|AMPHL
0033209 // tu	0005829 // cy	0005515 // pr	Baculoviral IA	BIRC3_HUMAN|BIRC3|API2|MIHC|RNF49
0043066 // ne	0005634 // nu	0005515 // pr	Baculoviral IA	BIRC7_HUMAN|BIRC7|KIAP|LIVIN|MLIAP|RNF50|UNQ5
0007049 // ce	0016021 // in	0005515 // pr	Bladder cance	BLCAP_HUMAN|BLCAP|BC10
0038083 // pe	0031234 // ex	0005515 // pr	Tyrosine-prot	BLK_HUMAN|BLK
0009967 // po	0005737 // cy	0005515 // pr	B-cell linker p	BLNK_HUMAN|BLNK|BASH|SLP65
0060155 // pl	0005765 // lys	0005515 // pr	Biogenesis of	BL1S2_HUMAN|BLOC1S2|BLOS2|CEAP
0050942 // po	0031083 // BL	0005515 // pr	Biogenesis of	BL1S5_HUMAN|BLOC1S5|MUTED
0042167 // he	0005829 // cy	0004074 // bi	Flavin reducta	BLVRB_HUMAN|BLVRB|FLR
0006357 // re	0005794 // G	0005515 // pr	Golgin-45	GO45_HUMAN|BLZF1|JEM1
0034380 // hi	0005615 // ex	0004222 // m	Bone morpho	BMP1_HUMAN|BMP1|PCOLC
0021978 // te	0005576 // ex	0008083 // gr	Bone morpho	BMP2_HUMAN|BMP2|BMP2A
0007500 // m	0005576 // ex	0008083 // gr	Bone morpho	BMP4_HUMAN|BMP4|BMP2B|DVR4
0001654 // ey	0005737 // cy	0070700 // BI	Bone morpho	BMP6_HUMAN|BMP6|VGR
0030509 // BI	0005615 // ex	0008083 // gr	Bone morpho	BMP8A_HUMAN|BMP8A
0030509 // BI	0005615 // ex	0008083 // gr	Bone morpho	BMP8B_HUMAN|BMP8B|BMP8
0045446 // er	0005615 // ex		BMP-binding	BMPER_HUMAN|BMPER|KIAA1965
0006364 // rR	0005654 // nu	0003723 // RI	Ribosome bio	BMS1_HUMAN|BMS1|BMS1L|KIAA0187
0038083 // pe	0005829 // cy	0005515 // pr	Cytoplasmic t	BMX_HUMAN|BMX
0007283 // sp	0005634 // nu	0003700 // tra	Zinc finger pr	BNC1_HUMAN|BNC1|BNC
0003416 // er	0005829 // cy	0003677 // DI	Zinc finger pr	BNC2_HUMAN|BNC2
0016320 // er	0005789 // er	0005484 // SN	Vesicle transp	SEC20_HUMAN|BNIP1|NIP1|SEC20L|TRG8
0010917 // ne	0030425 // de	0005515 // pr	BCL2/adenovi	BNIP3_HUMAN|BNIP3|NIP3
0006915 // ap	0005634 // nu	0005515 // pr	Bcl-2/adenovi	BNIPL_HUMAN|BNIPL
0051301 // ce	0005737 // cy		Biorientation	BOD1_HUMAN|BOD1|FAM44B
0008283 // ce	0005741 // m	0005102 // re	Bcl-2-related	BOK_HUMAN|BOK|BCL2L9
	0005739 // m	0005515 // pr	BolA-like prot	BOLA1_HUMAN|BOLA1|CGI-143
0008150 // bi	0005739 // m	0003674 // m	BolA-like prot	BOLA3_HUMAN|BOLA3

0007283 // sp	0005737 // cy	0005515 // pr	Protein boule-	BOLL_HUMAN	BOLL|BOULE
	0005765 // lys	0005515 // pr	BLOC-1-relate	BORC7_HUMAN	BORCS7|C10orf32
0006351 // tra	0005634 // nu	0000977 // RN	BORCS8-MEF2	H3BNR1_HUMAN	BORCS8-MEF2B
0006520 // ce	0070062 // ex	0016787 // hy	Valacyclovir h	BPHL_HUMAN	BPHL|MCNAA
0046854 // ph	0070062 // ex	0046872 // m	3'(2'),5'-bisph	BPNT1_HUMAN	BPNT1
0032465 // re	0030141 // se	0005515 // pr	Breast cancer	BRCA2_HUMAN	BRCA2|FACD|FANCD1
0070536 // pr	0000922 // sp	0004843 // th	Lys-63-specifi	BRCC3_HUMAN	BRCC3|BRCC36|C6.1A|CXorf53
0006351 // tra	0005575 // ce	0070577 // lys	Bromodomain	BRD9_HUMAN	BRD9|UNQ3040/PRO9856
0006383 // tra	0005654 // nu	0001026 // TF	Transcription	TF3B_HUMAN	BRF1|BRF|GTF3B|TAF3B2|TAF3C
0043312 // ne	0016021 // in	0042802 // id	Brain protein	BRI3_HUMAN	BRI3
0007050 // ce	0030425 // de		BMP/retinoic	BRNP3_HUMAN	BRINP3|DBCCR1L|DBCCR1L1|FAM5C
0032508 // DI	0005634 // nu	0005515 // pr	Fanconi anem	FANCJ_HUMAN	BRIP1|BACH1|FANCJ
0000027 // rib	0005730 // nu	0003723 // RN	Ribosome bio	BRX1_HUMAN	BRIX1|BRIX|BXDC2
0000122 // ne	0070822 // Si	0005515 // pr	Breast cancer	BRM1L_HUMAN	BRMS1L
0043972 // hi	0005654 // nu	0005515 // pr	Peregrin	BRPF1_HUMAN	BRPF1|BR140
0010212 // re	0005813 // ce	0004674 // pr	Serine/threon	BRSK1_HUMAN	BRSK1|KIAA1811|SAD1|SADB
1904152 // re	0005813 // ce	0050321 // ta	Serine/threon	BRSK2_HUMAN	BRSK2|C11orf7|PEN11B|SADA|STK29|I
0034389 // lip	0030176 // in	0005515 // pr	Seipin	BSCL2_HUMAN	BSCL2
0007416 // sy	0005634 // nu	0046872 // m	Protein basso	BSN_HUMAN	BSN|KIAA0434|ZNF231
0006816 // ca	0016020 // m	0046872 // m	B box and SPR	BSPRY_HUMAN	BSPRY
0050848 // re	0005886 // pl	0003953 // NA	ADP-ribosyl cy	BST1_HUMAN	BST1
0030336 // ne	0005794 // Go	0003723 // RN	Bone marrow	BST2_HUMAN	BST2
1901215 // ne	0001650 // fib		BTB/POZ dom	BTBDA_HUMAN	BTBD10|GMRP1
	0005634 // nu		BTB/POZ dom	BTBDG_HUMAN	BTBD16|C10orf87
0022008 // ne	0005829 // cy	0005515 // pr	BTB/POZ dom	BTBD2_HUMAN	BTBD2
0048813 // de	0005829 // cy	0031625 // ub	BTB/POZ dom	BTBD3_HUMAN	BTBD3|KIAA0952
0022008 // ne	0005829 // cy	0031625 // ub	BTB/POZ dom	BTBD6_HUMAN	BTBD6|BDPL
0038111 // in	0005576 // ex	0005154 // ep	Probetacelluli	BTC_HUMAN	BTC
0006768 // bi	0005759 // m	0047708 // bi	Biotinidase	BTD_HUMAN	BTD
0001701 // in	0005829 // cy	0003723 // RN	Transcription	BTF3_HUMAN	BTF3|NACB|OK/SW-cl.8
		0005515 // pr	Transcription	BT3L4_HUMAN	BTF3L4
0006977 // DI	0070062 // ex	0005515 // pr	Protein BTG2	BTG2_HUMAN	BTG2|PC3
0002250 // ac	0005886 // pl	0005515 // pr	Butyrophilin s	BT3A1_HUMAN	BTN3A1|BTF5
0002456 // T c	0016021 // in		Butyrophilin s	BT3A2_HUMAN	BTN3A2|BT3.2|BTF3|BTF4
0002456 // T c	0016021 // in		Butyrophilin s	BT3A3_HUMAN	BTN3A3|BTF3
0002250 // ac	0016021 // in	0005515 // pr	Butyrophilin-l	BTNL8_HUMAN	BTNL8|UNQ702/PRO1347
	0016021 // in		Butyrophilin-l	BTNL9_HUMAN	BTNL9|UNQ1900/PRO4346
0051437 // po	0005829 // cy	0005515 // pr	F-box/WD rep	FBW1A_HUMAN	BTRC|BTRCP|FBW1A|FBXW1A
0007517 // m	0042383 // sa	0030552 // cA	Blood vessel e	POPD1_HUMAN	BVES|POP1|POPDC1

0006351 // tra	0005737 // cy	0003723 // RN	Basic leucine z	BZW1_HUMAN|BZW1|BZAP45|KIAA0005
	0016021 // int		Uncharacteriz	CJ105_HUMAN|C10orf105
	0016021 // int		Uncharacteriz	CJ111_HUMAN|C10orf111
	0005576 // ex		Uncharacteriz	CJ025_HUMAN|C10orf25
	0016021 // int	0005515 // pr	Uncharacteriz	CJ035_HUMAN|C10orf35
	0005739 // m		Uncharacteriz	CJ067_HUMAN|C10orf67|LINC01552
		0005515 // pr	Uncharacteriz	CJ095_HUMAN|C10orf95
	0005576 // ex		Putative unch	CK045_HUMAN|C11orf45
	0070062 // ex		Uncharacteriz	CK052_HUMAN|C11orf52
		0005515 // pr	Uncharacteriz	CK057_HUMAN|C11orf57
0035082 // ax			Uncharacteriz	CK063_HUMAN|C11orf63
		0005515 // pr	Uncharacteriz	CK065_HUMAN|C11orf65
		0003723 // RN	UPF0696 prot	CK068_HUMAN|C11orf68|BLES03
	0005576 // ex		Uncharacteriz	CK094_HUMAN|C11orf94
0043473 // pi	0070062 // ex	0003674 // m	UPF0160 prot	MYG1_HUMAN|C12orf10
0002244 // he			Uncharacteriz	CL029_HUMAN|C12orf29
0043304 // re	0005737 // cy		Protein C12or	CL004_HUMAN|C12orf4
		0005515 // pr	Uncharacteriz	CL043_HUMAN|C12orf43
	0005576 // ex	0005515 // pr	UPF0454 prot	CL049_HUMAN|C12orf49
0061462 // pr	0005765 // lys		KICSTOR comp	CL066_HUMAN|C12orf66
0008150 // bi	0005789 // er	0003674 // m	Probable ergo	ERG28_HUMAN|C14orf1|AD-011|HSPC288|x0006
		0005515 // pr	Uncharacteriz	CN105_HUMAN|C14orf105
	0005739 // m		Uncharacteriz	CN119_HUMAN|C14orf119|My028
	0016021 // int		Uncharacteriz	CN132_HUMAN|C14orf132|C14orf88
0006536 // glu	0005759 // m	0047820 // D-	D-glutamate c	GLUCM_HUMAN|C14orf159|UNQ2439/PRO5000
0050658 // RN	0005737 // cy	0003723 // RN	UPF0568 prot	CN166_HUMAN|C14orf166|CGI-99

	0005576 // ex	0003723 // RN	Uncharacteriz	CN093_HUMAN|C14orf93
0007026 // ne	0015630 // m	0005515 // pr	Uncharacteriz	CP045_HUMAN|C16orf45
	0016021 // in		Transmembra	CP054_HUMAN|C16orf54|UNQ9389/PRO34280
		0005515 // pr	Uncharacteriz	CP059_HUMAN|C16orf59
0006893 // G	0005886 // pl	0005515 // pr	UPF0505 prot	CP062_HUMAN|C16orf62|101F10.2
		0005515 // pr	UPF0547 prot	CP087_HUMAN|C16orf87
0008150 // bi	0016021 // in	0003674 // m	Uncharacteriz	CQ080_HUMAN|C17orf80|HLC8|MIG3
	0005576 // ex		Uncharacteriz	CQ099_HUMAN|C17orf99|UNQ464/PRO809
0007165 // sig		0004871 // sig	UPF0729 prot	CR032_HUMAN|C18orf32
	0005765 // lys		Uncharacteriz	MIC1_HUMAN|C18orf8|MIC1
0006979 // re	0031966 // m		Protein C19or	CS012_HUMAN|C19orf12
	0016021 // in		Uncharacteriz	CS024_HUMAN|C19orf24
		0005515 // pr	UPF0449 prot	CS025_HUMAN|C19orf25
		0005515 // pr	Uncharacteriz	CS044_HUMAN|C19orf44
	0005654 // nu	0005515 // pr	Uncharacteriz	CS047_HUMAN|C19orf47
	0005730 // nu		Leydig cell tur	L10K_HUMAN|C19orf53|HSPC023
0007275 // m	0005575 // ce	0005515 // pr	Uncharacteriz	CS057_HUMAN|C19orf57
0001822 // ki	0000139 // G	0046872 // m	Glycoprotein-	C1GLT_HUMAN|C1GALT1
	0016021 // in		C1GALT1-spec	C1C1L_HUMAN|C1GALT1C1L
		0005515 // pr	Uncharacteriz	CA105_HUMAN|C1orf105
	0005737 // cy	0005515 // pr	Uncharacteriz	CA109_HUMAN|C1orf109
	0016021 // in		Uncharacteriz	CA115_HUMAN|C1orf115
0008150 // bi	0070062 // ex	0003674 // m	UPF0587 prot	CA123_HUMAN|C1orf123
	0005694 // ch	0003723 // RN	Uncharacteriz	CA131_HUMAN|C1orf131|cPERP-A

	0016021 // in		Uncharacteriz	CA186_HUMAN|C1orf186
		0005515 // pr	Uncharacteriz	CA021_HUMAN|C1orf21|PIG13
	0016021 // in		Type III endos	CA210_HUMAN|C1orf210
		0005515 // pr	UPF0500 prot	CA216_HUMAN|C1orf216
	0005576 // ex		Uncharacteriz	CA054_HUMAN|C1orf54
0045944 // pc	0005634 // nu	0003674 // m	Protein CROC-	CROC4_HUMAN|C1orf61|CROC4
0006508 // pr	0005576 // ex	0004252 // se	Complement	C1QB_HUMAN|C1QB
0007597 // bl	0005886 // pl	0005515 // pr	Complement	C1QBP_HUMAN|C1QBP|GC1QBP|HABP1|SF2P32
0050807 // re	0005576 // ex		Complement	C1QL3_HUMAN|C1QL3|CTRP13
0045599 // ne	0005581 // co	0042802 // id	Complement	C1QL4_HUMAN|C1QL4|CTRP11
0010544 // ne	0005887 // in	0005515 // pr	Complement	C1QT1_HUMAN|C1QTNF1|CTRP1|UNQ310/PRO353
0050715 // pc	0005581 // co	0003674 // m	Complement	C1QT3_HUMAN|C1QTNF3|CTRP3|UNQ753/PRO1484
	0005581 // co		C1QTNF3-AM	E9PGA6_HUMAN|C1QTNF3-AMACR
	0005615 // ex	0005125 // cy	Complement	C1QT4_HUMAN|C1QTNF4|CTRP4
0070208 // pr	0005581 // co	0042802 // id	Complement	C1QT6_HUMAN|C1QTNF6|CTRP6|UNQ581/PRO1151
0031638 // zy	0005576 // ex	0005515 // pr	Complement	C1R_HUMAN|C1R
0006508 // pr	0070062 // ex	0004252 // se	Complement	C1RL_HUMAN|C1RL|C1RL1|C1RLP|CLSPA
0006508 // pr	0005576 // ex	0005515 // pr	Complement	C1S_HUMAN|C1S
2000427 // pc	0005576 // ex	0004252 // se	Complement	CO2_HUMAN|C2
	0005737 // cy		Uncharacteriz	CT203_HUMAN|C20orf203
0008150 // bi	0005575 // ce	0005515 // pr	UPF0687 prot	CT027_HUMAN|C20orf27
0055114 // ox		0016491 // ox	Uncharacteriz	CT096_HUMAN|C20orf96
0048858 // ce	0005829 // cy		UPF0769 prot	CU059_HUMAN|C21orf59|C21orf48
0008150 // bi	0005575 // ce	0005515 // pr	UPF0193 prot	EVG1_HUMAN|C22orf23
	0016021 // in		Uncharacteriz	CV024_HUMAN|C22orf24
	0016021 // in		C2 domain-co	C2CD2_HUMAN|C2CD2|C21orf25|C21orf258
0035774 // pc	0032541 // co	0005548 // ph	Phospholipid	C2C2L_HUMAN|C2CD2L|KIAA0285|TMEM24|DLNB23
0006906 // ve	0005886 // pl	0005509 // ca	C2 calcium-de	C2D4D_HUMAN|C2CD4D|FAM148D
0031340 // pc	0005829 // cy	0005544 // ca	C2 domain-co	C2CD5_HUMAN|C2CD5|CDP138|KIAA0528
	0070062 // ex		Uncharacteriz	CB016_HUMAN|C2orf16

	0016021 // in		Uncharacteriz	CB074_HUMAN|C2orf74
	0016021 // in		Uncharacteriz	CB082_HUMAN|C2orf82|UNQ830/PRO1757
0006631 // fa	0034774 // se	0004252 // se	Complement	CO3_HUMAN|C3|CPAMD1
	0016021 // in		Uncharacteriz	CC018_HUMAN|C3orf18
0014066 // re	0000139 // G		Deleted in aut	DIA1_HUMAN|C3orf58|DIA1
	0016021 // in		Uncharacteriz	CC080_HUMAN|C3orf80
0006508 // pr	0005886 // pl	0004252 // se	Complement	CO4A_HUMAN|C4A|CO4|CPAMD2
0006508 // pr	0005615 // ex	0004252 // se	Complement	CO4B_HUMAN|C4B|C4B_2|CO4|CPAMD3
	0016021 // in		Uncharacteriz	CD003_HUMAN|C4orf3
	0016021 // in		Uncharacteriz	CD032_HUMAN|C4orf32
		0005515 // pr	UPF0462 prot	CD033_HUMAN|C4orf33
	0005737 // cy		UPF0602 prot	CD047_HUMAN|C4orf47|Chr4_1746
	0005576 // ex		Neuropeptide	CD048_HUMAN|C4orf48|Chr4_55
0006935 // ch	0005576 // ex	0005515 // pr	Complement	CO5_HUMAN|C5|CPAMD4
0006968 // ce	0005886 // pl	0001856 // co	C5a anaphylat	C5AR1_HUMAN|C5AR1|C5AR|C5R1
		0005515 // pr	UPF0489 prot	CE022_HUMAN|C5orf22
		0005515 // pr	UPF0461 prot	CE024_HUMAN|C5orf24
1900016 // ne	0005737 // cy	0005515 // pr	UNC119-bindi	CE030_HUMAN|C5orf30
	0005576 // ex		Protein CEI	CEI_HUMAN|C5orf38|CEI
0008150 // bi	0070062 // ex	0003674 // m	Uncharacteriz	CE046_HUMAN|C5orf46|UNQ472/PRO839
0055114 // ox			Glutaredoxin-	YD286_HUMAN|C5orf63
0030198 // ex	0005614 // in	0070052 // co	Uncharacteriz	CF015_HUMAN|C6orf15|STG|UNQ1840/PRO3566
0008150 // bi	0005575 // ce	0003674 // m	Uncharacteriz	CF163_HUMAN|C6orf163
		0005515 // pr	Uncharacteriz	CF226_HUMAN|C6orf226

0008150 // bi	0005622 // in	0003674 // m	Uncharacteriz	CF062_HUMAN|C6orf62|XTP12|Nbla00237
1901727 // po	0005730 // nu	0005515 // pr	Bombesin rec	CF089_HUMAN|C6orf89|BRAP|UNQ177/PRO203
0006957 // co	0005576 // ex		Complement	CO7_HUMAN|C7
	0005737 // cy	0005515 // pr	Uncharacteriz	CG031_HUMAN|C7orf31
	0005634 // nu		Uncharacteriz	CG061_HUMAN|C7orf61
	0097546 // cil	0005515 // pr	Protein C8orf3	CH037_HUMAN|C8orf37|smalltalk
	0005654 // nu		Putative unch	CH044_HUMAN|C8orf44
		0005515 // pr	Uncharacteriz	CH048_HUMAN|C8orf48
		0005515 // pr	Uncharacteriz	CH074_HUMAN|C8orf74
0045947 // ne	0005737 // cy	0008190 // eu	Uncharacteriz	CH088_HUMAN|C8orf88
0051260 // pr	0005576 // ex		Complement	CO9_HUMAN|C9
		0005515 // pr	UPF0691 prot	CI116_HUMAN|C9orf116
0006303 // do	0035861 // sit	0005515 // pr	Protein PAXX	PAXX_HUMAN|C9orf142|PAXX|XLS
0008150 // bi	0005575 // ce	0005515 // pr	UPF0184 prot	CI016_HUMAN|C9orf16|EST00098
		0005515 // pr	Uncharacteriz	CI043_HUMAN|C9orf43
	0005576 // ex		Uncharacteriz	CI047_HUMAN|C9orf47|C9orf108
0043547 // po	0005764 // lys	0005515 // pr	Guanine nucle	CI072_HUMAN|C9orf72
0015701 // bi	0005829 // cy	0004064 // ar	Carbonic anhy	CAH1_HUMAN|CA1
	0005576 // ex	0004089 // ca	Carbonic anhy	CAH11_HUMAN|CA11|CARP2|UNQ211/PRO237
0015701 // bi	0016021 // in	0008270 // zir	Carbonic anhy	CAH12_HUMAN|CA12
0015701 // bi	0005829 // cy	0004089 // ca	Carbonic anhy	CAH13_HUMAN|CA13
0015701 // bi	0016021 // in	0004089 // ca	Carbonic anhy	CAH14_HUMAN|CA14|UNQ690/PRO1335
0015701 // bi	0005829 // cy	0004089 // ca	Carbonic anhy	CAH2_HUMAN|CA2
0006979 // re	0005829 // cy	0008270 // zir	Carbonic anhy	CAH3_HUMAN|CA3
0015701 // bi	0005739 // m	0004089 // ca	Carbonic anhy	CAH5B_HUMAN|CA5B
0015701 // bi	0005730 // nu	0008270 // zir	Carbonic anhy	CAH9_HUMAN|CA9|G250|MN
0051726 // re	0005634 // nu	0005515 // pr	CDK5 and ABL	CABL1_HUMAN|CABLES1|CABLES
0043086 // ne	0005938 // ce	0008139 // nu	Calcium-bindi	CABP1_HUMAN|CABP1
0007602 // ph	0045202 // sy	0005509 // ca	Calcium-bindi	CABP4_HUMAN|CABP4

	0016021 // int	0005509 // ca	Calcium-bindi	CABP7_HUMAN|CABP7|CALN2
0048240 // sp	0097229 // sp	0005509 // ca	Calcium-bindi	CABYR_HUMAN|CABYR|CBP86|FSP2
1901379 // re	0005891 // vo	0008331 // hi	Voltage-deper	CAC1D_HUMAN|CACNA1D|CACH3|CACN4|CACNL1A2|C
0050856 // re	0005891 // vo	0005245 // vo	Voltage-deper	CAC1F_HUMAN|CACNA1F|CACNAF1
0045956 // po	0005886 // pl	0005245 // vo	Voltage-deper	CAC1I_HUMAN|CACNA1I|KIAA1120
0086091 // re	0005891 // vo	0086057 // vo	Voltage-deper	CA2D1_HUMAN|CACNA2D1|CACNL2A|CCHL2A|MHS3
0050796 // re	0005891 // vo	0005245 // vo	Voltage-deper	CA2D2_HUMAN|CACNA2D2|KIAA0558
0070588 // ca	0005891 // vo	0005245 // vo	Voltage-deper	CA2D4_HUMAN|CACNA2D4
0006810 // tra	0005829 // cy	0005515 // pr	Voltage-deper	CACB4_HUMAN|CACNB4|CACNLB4
0006810 // tra	0005886 // pl	0016247 // ch	Voltage-deper	CCG4_HUMAN|CACNG4
0006816 // ca	0005886 // pl	0016247 // ch	Voltage-deper	CCG7_HUMAN|CACNG7
2000311 // re	0045211 // po	0016247 // ch	Voltage-deper	CCG8_HUMAN|CACNG8|CACNG6
0032688 // ne	0070062 // ex	0003723 // RN	Cactin	CATIN_HUMAN|CACTIN|C19orf29
0001913 // T	0005911 // ce	0050839 // ce	Cell adhesion	CADM1_HUMAN|CADM1|IGSF4|IGSF4A|NECL2|SYNCAM
0007156 // ho	0005913 // ce	0050839 // ce	Cell adhesion	CADM2_HUMAN|CADM2|IGSF4D|NECL3
0007156 // ho	0005913 // ce	0050839 // ce	Cell adhesion	CADM4_HUMAN|CADM4|IGSF4C|NECL4|TSLL2
0009267 // ce	0045211 // po	0008289 // lip	Calcium-depe	CAPS2_HUMAN|CADPS2|CAPS2|KIAA1591
0010842 // re	0005829 // cy	0005515 // pr	Calbindin	CALB1_HUMAN|CALB1|CAB27
0007566 // en	0005634 // nu	0005515 // pr	Calcitonin	CALC_HUMAN|CALCA|CALC1
0007165 // sig	0005576 // ex	0005184 // ne	Calcitonin ger	CALCB_HUMAN|CALCB|CALC2
0006171 // cA	0005886 // pl	0005515 // pr	Calcitonin ger	CALRL_HUMAN|CALCRL|CGRPR
0006928 // m	0005829 // cy	0045296 // ca	Caldesmon	CALD1_HUMAN|CALD1|CAD|CDM
0034220 // io	0005887 // int	0005261 // ca	Calcium home	CAHM2_HUMAN|CALHM2|FAM26B
0034220 // io	0005887 // int	0005261 // ca	Calcium home	CAHM3_HUMAN|CALHM3|FAM26A
0071902 // po	0014069 // po	0005509 // ca	Calmodulin-1	CALM1_HUMAN|CALM1|CALM|CAM|CAM1
	0070062 // ex	0005515 // pr	Calmodulin-lik	CALL3_HUMAN|CALML3
0008544 // ep	0005576 // ex	0005509 // ca	Calmodulin-lik	CALL5_HUMAN|CALML5|CLSP
0043393 // re	0005622 // int	0005516 // ca	Calcium/calm	KCC1A_HUMAN|CAMK1
0010976 // po	0005634 // nu	0005516 // ca	Calcium/calm	KCC1D_HUMAN|CAMK1D|CAMKID
0038166 // an	0014069 // po	0005515 // pr	Calcium/calm	KCC2A_HUMAN|CAMK2A|CAMKA|KIAA0968
1901844 // re	0005829 // cy	0005515 // pr	Calcium/calm	KCC2D_HUMAN|CAMK2D|CAMKD
0006469 // ne	0014069 // po	0019901 // pr	Calcium/calm	CK2N1_HUMAN|CAMK2N1
0006469 // ne	0005829 // cy	0019901 // pr	Calcium/calm	CK2N2_HUMAN|CAMK2N2
0006468 // pr	0005737 // cy	0009931 // ca	Calcium/calm	KCC4_HUMAN|CAMK4|CAMK|CAMK-GR|CAMKIV
0018107 // pe	0005634 // nu	0004683 // ca	Calcium/calm	KKCC1_HUMAN|CAMKK1|CAMKKA
0016032 // vir	0016021 // int	0005515 // pr	Calcium signa	CAMLG_HUMAN|CAMLG|CAML
0007010 // cy	0005737 // cy	0030507 // sp	Calmodulin-re	CAMP1_HUMAN|CAMSAP1
0031175 // ne	0005813 // ce	0030507 // sp	Calmodulin-re	CAMP3_HUMAN|CAMSAP3|KIAA1543
0045944 // po	0005730 // nu	0043565 // se	Calmodulin-bi	CMTA1_HUMAN|CAMTA1|KIAA0833|MSTP023
0002474 // an	0043209 // m	0003723 // RN	Calnexin	CALX_HUMAN|CANX
0007163 // es	0005886 // pl	0005515 // pr	Adenylyl cycla	CAP2_HUMAN|CAP2
0071803 // po	0005654 // nu	0019904 // pr	Macrophage-c	CAPG_HUMAN|CAPG|AFCP|MCP
0097264 // se	0005829 // cy	0005515 // pr	Calpain-1 cata	CAN1_HUMAN|CAPN1|CANPL1|PIG30

0006508 // pr	0001669 // ac	0008233 // pe	Calpain-11	CAN11_HUMAN|CAPN11
0006508 // pr	0005737 // cy	0004198 // ca	Calpain-12	CAN12_HUMAN|CAPN12
0006508 // pr	0005737 // cy	0005509 // ca	Calpain-13	CAN13_HUMAN|CAPN13
0006508 // pr	0005737 // cy	0004198 // ca	Calpain-14	CAN14_HUMAN|CAPN14
0006508 // pr	0005737 // cy	0008233 // pe	Calpain-15	CAN15_HUMAN|CAPN15|SOLH
0045214 // sa	0030016 // m	0032947 // pr	Calpain-3	CAN3_HUMAN|CAPN3|CANP3|CANPL3|NCL1
0007165 // sig	0070062 // ex	0004198 // ca	Calpain-5	CAN5_HUMAN|CAPN5|NCL3
0007586 // di	0005794 // Go	0005509 // ca	Calpain-8	CAN8_HUMAN|CAPN8|NCL2
0007586 // di	0005737 // cy	0005509 // ca	Calpain-9	CAN9_HUMAN|CAPN9|NCL4
0006508 // pr	0005829 // cy	0005515 // pr	Calpain small	CPNS1_HUMAN|CAPNS1|CAPN4|CAPNS
0045944 // po	0005737 // cy	0005102 // re	Caprin-2	CAPR2_HUMAN|CAPRIN2|C1QDC1|EEG1|KIAA1873|RN
0035556 // int	0070062 // ex	0005509 // ca	Calcyphosin	CAYP1_HUMAN|CAPS
0035725 // so		0005509 // ca	Calcyphosin-2	CAYP2_HUMAN|CAPS2|UG0636c06
0007250 // ac	0032449 // CE	0030159 // re	Caspase recru	CAR10_HUMAN|CARD10|CARMA3
0002377 // im	0005886 // pl	0005515 // pr	Caspase recru	CAR11_HUMAN|CARD11|CARMA1
0051092 // po	0043234 // pr	0005515 // pr	Caspase recru	CAR16_HUMAN|CARD16|COP|COP1
0010951 // ne	0043234 // pr	0005515 // pr	Caspase recru	CAR17_HUMAN|CARD17|INCA
0006954 // inf	0043234 // pr	0050700 // CA	Caspase recru	CAR18_HUMAN|CARD18|ICEBERG|UNQ5804/PRO19611
0006915 // ap			Caspase recru	CARD6_HUMAN|CARD6
0031665 // ne	0005737 // cy	0032089 // NA	Caspase recru	CARD8_HUMAN|CARD8|KIAA0955|NDPP1
0032494 // re	0005886 // pl	0005515 // pr	Caspase recru	CARD9_HUMAN|CARD9
0061400 // po	0005634 // nu	0001077 // tra	Calcium-respo	CARTF_HUMAN|CARF|ALS2CR8
0043488 // re	0005829 // cy	0005515 // pr	Calcium-regul	CHSP1_HUMAN|CARHSP1
0051092 // po	0005829 // cy	0005515 // pr	Histone-argini	CARM1_HUMAN|CARM1|PRMT4
	0005886 // pl		Capping prote	CARL3_HUMAN|CARMIL3|C14orf121|LRRC16B
0035499 // ca	0005829 // cy	0102102 /// 0	Carnosine syn	CRNS1_HUMAN|CARNS1|ATPGD1|KIAA1394
0006423 // cy	0005829 // cy	0000049 // tR	Cysteine--tRN	SYCC_HUMAN|CARS
0043409 // ne	0005575 // ce	0003674 // m	Protein CASC2	CSC2A_HUMAN|CASC2|C10orf5
0046710 // GI	0042734 // pr	0004674 // pr	Peripheral pla	CSKP_HUMAN|CASK|LIN2
0007165 // sig	0016020 // m	0005515 // pr	Caskin-1	CSKI1_HUMAN|CASKIN1|KIAA1306
0008150 // bi	0016020 // m	0003674 // m	Caskin-2	CSKI2_HUMAN|CASKIN2|KIAA1139
0097194 // ex	0005576 // ex	0005515 // pr	Caspase-1	CASP1_HUMAN|CASP1|IL1BC|IL1BCE
0006508 // pr	0031265 // CD	0005515 // pr	Caspase-10	CASPA_HUMAN|CASP10|MCH4
0097194 // ex	0072557 // IP	0004197 // cy	Caspase-5	CASP5_HUMAN|CASP5|ICH3
0009408 // re	0005739 // m	0005509 // ca	Calsequestrin	CASQ1_HUMAN|CASQ1|CASQ
0006351 // tra	0005829 // cy	0003677 // DN	Zinc finger pro	CASZ1_HUMAN|CASZ1|CST|SRG|ZNF693
0007342 // fu	0005886 // pl	0005515 // pr	Cation channe	CTSR1_HUMAN|CATSPER1
0007283 // sp	0005886 // pl	0005227 // ca	Cation channe	CTSR2_HUMAN|CATSPER2

0007275 // m	0005929 // cil		Cation channe	CTSRB_HUMAN	CATSPERB|C14orf161
0045907 // pc	0005901 // ca	0005515 // pr	Caveolin-1	CAV1_HUMAN	CAV1|CAV
0044791 // pc	0005901 // ca	0005515 // pr	Caveolin-2	CAV2_HUMAN	CAV2
1901386 // ne	0016021 // in	0044325 // io	Voltage-deper	CBARP_HUMAN	CBARP|C19orf26
0045892 // ne	0005730 // nu	0005515 // pr	Protein CBFA2	MTG16_HUMAN	CBFA2T3|MTG16|MTGR2|ZMYND4
0042059 // ne	0005634 // nu	0005515 // pr	E3 ubiquitin-p	CBLC_HUMAN	CBLC|CBL3|RNF57
	0005615 // ex		Cerebellin-3	CBLN3_HUMAN	CBLN3|UNQ755/PRO1486
0042376 // ph	0005829 // cy	0004090 // ca	Carbonyl redu	CBR3_HUMAN	CBR3
0055114 // ox	1990204 // ox	0003955 // NA	Carbonyl redu	CBR4_HUMAN	CBR4|SDR45C1
0006535 // cy	0005634 // nu	0046872 // m	Cystathionine	CBSL_HUMAN	CBSL
		0005524 // AT	COBW domair	CBWD1_HUMAN	CBWD1
		0005524 // AT	COBW domair	CBWD5_HUMAN	CBWD5
0006351 // tra	0005654 // nu	0005515 // pr	Chromobox pr	CBX2_HUMAN	CBX2
0043066 // ne	0005654 // nu	0051219 // ph	E3 SUMO-pro	CBX4_HUMAN	CBX4
0045892 // ne	0010369 // ch	0005515 // pr	Chromobox pr	CBX5_HUMAN	CBX5|HP1A
0006351 // tra	0000792 // he	0005515 // pr	Chromobox pr	CBX6_HUMAN	CBX6
0006351 // tra	0005634 // nu	0005515 // pr	Chromobox pr	CBX7_HUMAN	CBX7
0045739 // pc	0005654 // nu	0005515 // pr	Chromobox pr	CBX8_HUMAN	CBX8|PC3|RC1
0006351 // tra	0005634 // nu	0000981 // RN	Coiled-coil an	C2D1B_HUMAN	CC2D1B|KIAA1836
0007368 // de	0005829 // cy		Coiled-coil an	C2D2A_HUMAN	CC2D2A|KIAA1345
1901492 // pc	0005615 // ex	0005518 // co	Collagen and	CCBE1_HUMAN	CCBE1|KIAA1983|UNQ1921/PRO4395
	0016459 // m	0003774 // m	Coiled-coil do	C102A_HUMAN	CCDC102A
		0005515 // pr	Coiled-coil do	C102B_HUMAN	CCDC102B|C18orf14
0001947 // he	0005737 // cy	0042803 // pr	Coiled-coil do	CC103_HUMAN	CCDC103
	0005829 // cy	0005515 // pr	Coiled-coil do	CC106_HUMAN	CCDC106
	0016021 // in		Coiled-coil do	CC107_HUMAN	CCDC107|PSEC0222
		0005515 // pr	Coiled-coil do	CC112_HUMAN	CCDC112|MBC1
0060271 // cil	0043234 // pr	0005515 // pr	Coiled-coil do	CC113_HUMAN	CCDC113|HSPC065
0003341 // cil	0005930 // ax	0005515 // pr	Coiled-coil do	CC114_HUMAN	CCDC114
	0005737 // cy	0005515 // pr	Coiled-coil do	CC116_HUMAN	CCDC116
		0005515 // pr	Coiled-coil do	CC117_HUMAN	CCDC117
0007275 // m	0005768 // er	0005515 // pr	Coiled-coil do	CC120_HUMAN	CCDC120|JM11
0051301 // ce	0005737 // cy	0003723 // RN	Coiled-coil do	CC124_HUMAN	CCDC124
0006974 // ce	0005829 // cy	0005515 // pr	Coiled-coil do	CCD13_HUMAN	CCDC13
	0005576 // ex		Coiled-coil do	CC134_HUMAN	CCDC134
0007283 // sp	0016021 // in		Coiled-coil do	CC136_HUMAN	CCDC136|KIAA1793|NAG6
	0005694 // ch	0003723 // RN	Coiled-coil do	CC137_HUMAN	CCDC137|cPERP-B
	0005737 // cy	0005515 // pr	Coiled-coil do	CC141_HUMAN	CCDC141|CAMDI

	0005814 // ce		Coiled-coil do	CC146_HUMAN|CCDC146|KIAA1505	
		0005515 // pr	Coiled-coil do	CC148_HUMAN|CCDC148	
	0005813 // ce		Coiled-coil do	CCD15_HUMAN|CCDC15	
1902017 // re	0005929 // cil	0005515 // pr	Coiled-coil do	CC151_HUMAN|CCDC151	
		0005515 // pr	Coiled-coil do	CC153_HUMAN|CCDC153	
	0005769 // ea		Coiled-coil do	CC154_HUMAN|CCDC154|C16orf29	
		0005515 // pr	Coiled-coil do	CCD17_HUMAN|CCDC17	
		0005515 // pr	Coiled-coil do	CC170_HUMAN|CCDC170|C6orf97	
	0005737 // cy	0008017 // m	Coiled-coil do	CC181_HUMAN|CCDC181|C1orf114	
0003341 // cil	0005930 // ax		Coiled-coil do	CC183_HUMAN|CCDC183|KIAA1984	
0034453 // m	0005813 // ce	0008017 // m	Coiled-coil do	CC187_HUMAN|CCDC187	
		0005515 // pr	Coiled-coil do	CCD24_HUMAN|CCDC24	
		0005515 // pr	Coiled-coil do	CC28A_HUMAN|CCDC28A|C6orf80	
0010804 // ne	0005576 // ex		Coiled-coil do	CCDC3_HUMAN|CCDC3	
		0005515 // pr	Coiled-coil do	CCD33_HUMAN|CCDC33	
0006310 // DI	0000794 // co	0005515 // pr	Interactor of I	IHO1_HUMAN|CCDC36|IHO1	
	0005737 // cy		Coiled-coil do	CCD38_HUMAN|CCDC38	
0030317 // fla	0005929 // cil	0003674 // m	Coiled-coil do	CCD40_HUMAN|CCDC40|KIAA1640	
		0005515 // pr	Coiled-coil do	CCD57_HUMAN|CCDC57	
	0005739 // m		Coiled-coil do	CCD58_HUMAN|CCDC58	
0006351 // tra	0005654 // nu	0003723 // RN	Thyroid transc	TAP26_HUMAN|CCDC59|BR22|TAP26|HSPC128	
0007010 // cy	0005829 // cy	0005515 // pr	Coiled-coil do	CCDC6_HUMAN|CCDC6|D10S170|TST1	
		0005515 // pr	Coiled-coil do	CCD60_HUMAN|CCDC60	
0071392 // ce	0005634 // nu	0030331 // es	Coiled-coil do	CCD62_HUMAN|CCDC62	
		0005515 // pr	Coiled-coil do	CCD68_HUMAN|CCDC68	
0051255 // sp	0005737 // cy		Coiled-coil do	CCD69_HUMAN|CCDC69	
	0005576 // ex	0005515 // pr	Coiled-coil do	CCD70_HUMAN|CCDC70	
	0016020 // m		Coiled-coil do	CCD77_HUMAN|CCDC77	
0098535 // de	0098536 // de		Coiled-coil do	CCD78_HUMAN|CCDC78|C16orf25|JFP10	

		0005515 // pr	Coiled-coil do	CCD87_HUMAN|CCDC87
0031122 // cy	0005783 // er	0008017 // m	Coiled-coil do	CC88B_HUMAN|CCDC88B|BRLZ
0031648 // pr	0005737 // cy	0008017 // m	Protein Daple	DAPLE_HUMAN|CCDC88C|DAPLE|KIAA1509
	0005634 // nu		Coiled-coil do	CCD89_HUMAN|CCDC89|BOIP
		0005515 // pr	Coiled-coil do	CCD94_HUMAN|CCDC94
	0005737 // cy		Coiled-coil do	CCD96_HUMAN|CCDC96
	0005576 // ex		Coiled-coil do	CCER2_HUMAN|CCER2
0070374 // pc	0005615 // ex	0005102 // re	C-C motif chei	CCL17_HUMAN|CCL17|SCYA17|TARC
0007204 // pc	0005576 // ex	0005515 // pr	C-C motif chei	CCL28_HUMAN|CCL28|SCYA28
0070374 // pc	0005576 // ex	0005515 // pr	C-C motif chei	CCL5_HUMAN|CCL5|D17S136E|SCYA5
0000083 // re	0005654 // nu	0005515 // pr	Cyclin-A1	CCNA1_HUMAN|CCNA1
0007286 // sp	0000795 // sy	0005515 // pr	E3 ubiquitin-p	CIP1_HUMAN|CCNB1IP1|C14orf18|HEI10
0010389 // re	0016607 // nu	0019901 // pr	G2/mitotic-sp	CCNB3_HUMAN|CCNB3|CYCB3
0033598 // m	0005654 // nu	0019901 // pr	G1/S-specific	CCND1_HUMAN|CCND1|BCL1|PRAD1
0045859 // re	0005634 // nu	0005515 // pr	G1/S-specific	CCNE1_HUMAN|CCNE1|CCNE
0000079 // re	0005654 // nu	0005515 // pr	G1/S-specific	CCNE2_HUMAN|CCNE2
0051301 // ce	0005654 // nu	0005515 // pr	Cyclin-G1	CCNG1_HUMAN|CCNG1|CCNG|CYCG1
0051301 // ce	0005737 // cy		Cyclin-G2	CCNG2_HUMAN|CCNG2
	0005634 // nu		Cyclin-J	CCNJ_HUMAN|CCNJ
	0005634 // nu		Cyclin-J-like pi	CCNJL_HUMAN|CCNJL
0045737 // pc	0005654 // nu	0005515 // pr	Cyclin-K	CCNK_HUMAN|CCNK|CPR4
0006396 // RI	0005634 // nu	0005515 // pr	Cyclin-L1	CCNL1_HUMAN|CCNL1|BM-001|UNQ530/PRO1073
0042493 // re	0005737 // cy	0004844 // ur	Cyclin-O	CCNO_HUMAN|CCNO
0016055 // W	0000308 // cy	0005515 // pr	Cyclin-Y	CCNY_HUMAN|CCNY|C10orf9|CBCP1|CFP1
0000079 // re		0019901 // pr	Cyclin-Y-like p	CCYL2_HUMAN|CCNYL2|C10orf21
0006887 // ex	0005886 // pl	0019957 // C-	C-C chemokin	CCR1_HUMAN|CCR1|CMKBR1|CMKR1|SCYAR1
0070098 // ch	0009986 // ce	0005515 // pr	C-C chemokin	CCR10_HUMAN|CCR10|GPR2
0006954 // in	0005886 // pl	0016493 // C-	C-C chemokin	CCR3_HUMAN|CCR3|CMKBR3
0070098 // ch	0005886 // pl	0016493 // C-	C-C chemokin	CCR4_HUMAN|CCR4|CMKBR4
0032735 // pc	0009897 // ex	0035758 // ch	C-C chemokin	CCR7_HUMAN|CCR7|CMKBR7|EBI1|EVI1
0007186 // G-	0005887 // in	0042379 // ch	C-C chemokin	CCRL2_HUMAN|CCRL2|CCR11|CCR6|CKRX|CRAM|HCR
0034599 // ce	0005615 // ex	0004784 // su	Copper chape	CCS_HUMAN|CCS
1901998 // to	0005829 // cy	0005515 // pr	T-complex prc	TCPB_HUMAN|CCT2|99D8.1|CCTB
0051131 // ch	0005832 // ch	0008565 // pr	T-complex prc	TCPW_HUMAN|CCT6B
1904871 // pc	0005829 // cy	0044183 // pr	T-complex prc	TCPQ_HUMAN|CCT8|C21orf112|CCTQ|KIAA0002
0043547 // pc	0005765 // lys	0005515 // pr	Vacuolar fusic	CCZ1_HUMAN|CCZ1|C7orf28A|CGI-43
0007166 // ce	0070062 // ex	0016812 // hy	Immunoglobu	IGSF2_HUMAN|CD101|EWI101|IGSF2|V7
0002576 // pl	0031225 // ar	0004867 // se	CD109 antiger	CD109_HUMAN|CD109|CPAMD7
0032760 // pc	0005576 // ex	0005515 // pr	Monocyte diff	CD14_HUMAN|CD14
0007155 // ce	0005829 // cy	0005515 // pr	CD151 antiger	CD151_HUMAN|CD151|TSPAN24
0008283 // ce	0046658 // ar	0005515 // pr	CD160 antiger	BY55_HUMAN|CD160|BY55
0006898 // re	0016021 // in	0005044 // sc	Scavenger rec	C163B_HUMAN|CD163L1|CD163B|M160|UNQ6434/PR(
	0016021 // in		CD164 sialom	C16L2_HUMAN|CD164L2|UNQ6122/PRO20044
0050776 // re	0009897 // ex	0004872 // re	Cell surface gl	MO2R1_HUMAN|CD200R1|CD200R|CRTR2|MOX2R|OX

	0009897 // ex	0004872 // re	Cell surface gl	MO2R2_HUMAN|CD200R1L|CD200R2
0072139 // gl	0009986 // ce	0005515 // pr	Signal transdu	CD24_HUMAN|CD24|CD24A
0050776 // re	0005886 // pl	0005515 // pr	T-cell surface	CD3Z_HUMAN|CD247|CD3Z|T3Z|TCRZ
1901998 // to	0005886 // pl	0005515 // pr	Programmed	PD1L1_HUMAN|CD274|B7H1|PDCD1L1|PDCD1LG1|PDL
1900182 // po	0030139 // en	0005200 // st	CD2-associate	CD2AP_HUMAN|CD2AP
0000244 // sp	0005634 // nu	0005515 // pr	CD2 antigen c	CD2B2_HUMAN|CD2BP2|KIAA1178
0006909 // ph	0005902 // m	0030246 // ca	CD302 antiger	CD302_HUMAN|CD302|CLEC13A|DCL1|KIAA0022
0009235 // co	0010008 // en	0008083 // gr	CD320 antiger	CD320_HUMAN|CD320|8D6A|UNQ198/PRO224
0007155 // ce	0005887 // in	0005515 // pr	Myeloid cell s	CD33_HUMAN|CD33|SIGLEC3
0034197 // tri	0005886 // pl	0005041 // lo	Platelet glyco	CD36_HUMAN|CD36|GP3B|GP4
0007166 // ce	0070062 // ex	0005515 // pr	Leukocyte ant	CD37_HUMAN|CD37|TSPAN26
0033194 // re	0009986 // ce	0003953 // NA	ADP-ribosyl cy	CD38_HUMAN|CD38
0006361 // tra	0005654 // nu	0003723 // RN	DNA-directed	RPA34_HUMAN|CD3EAP|ASE1|CAST|PAF49
0033209 // tu	0043196 // va	0005515 // pr	Tumor necros	TNR5_HUMAN|CD40|TNFRSF5
0043382 // po	0005886 // pl	0001618 // vi	Membrane co	MCP_HUMAN|CD46|MCP|MIC10
0007155 // ce	0005887 // in	0070053 // th	Leukocyte sur	CD47_HUMAN|CD47|MER6
0031295 // T	0005887 // in	0005044 // sc	T-cell surface	CD5_HUMAN|CD5|LEU1
1901741 // po	0005911 // ce	0005515 // pr	Leukocyte sur	CD53_HUMAN|CD53|MOX44|TSPAN25
0045916 // ne	0005886 // pl	0005515 // pr	Complement	DAF_HUMAN|CD55|CR|DAF
0006888 // ER	0033116 // en	0005515 // pr	CD59 glycopro	CD59_HUMAN|CD59|MIC11|MIN1|MIN2|MIN3|MSK21
0002250 // ac	0042101 // T	0005044 // sc	T-cell differen	CD6_HUMAN|CD6
0071310 // ce	0016021 // in		Macrosialin	CD68_HUMAN|CD68
0007267 // ce	0005886 // pl	0005515 // pr	CD70 antigen	CD70_HUMAN|CD70|CD27L|CD27LG|TNFSF7
0007155 // ce	0005886 // pl	0004888 // tra	B-cell differen	CD72_HUMAN|CD72
0045657 // po	0032588 // tra	0042289 // M	HLA class II his	HG2A_HUMAN|CD74|DHLAG
0007166 // ce	0070062 // ex	0005515 // pr	CD82 antigen	CD82_HUMAN|CD82|KAI1|SAR2|ST6|TSPAN27
0001817 // re	0009897 // ex	0005515 // pr	CD83 antigen	CD83_HUMAN|CD83
0038111 // in	0070062 // ex	0005515 // pr	T-lymphocyte	CD86_HUMAN|CD86|CD28LG2
0002456 // T	0005886 // pl	0005515 // pr	T-cell surface	CD8A_HUMAN|CD8A|MAL
0006955 // im	0005887 // in	0042288 // M	T-cell surface	CD8B_HUMAN|CD8B|CD8B1
0014003 // ol	1903561 // ex	0005178 // in	CD9 antigen	CD9_HUMAN|CD9|MIC3|TSPAN29|GIG2
0042116 // m	0005886 // pl	0001849 // co	Complement	C1QR1_HUMAN|CD93|C1QR1|MXRA4
0007155 // ce	0005737 // cy		T-cell surface	TACT_HUMAN|CD96
0007155 // ce	0005887 // in	0005515 // pr	CD99 antigen	CD99_HUMAN|CD99|MIC2|MIC2X|MIC2Y
0007155 // ce	0016021 // in	0005515 // pr	CD99 antigen-	C99L2_HUMAN|CD99L2|MIC2L1|UNQ1964/PRO4486
0030308 // ne	0034774 // se	0005515 // pr	Cytidine deam	CDD_HUMAN|CDA|CDD
0051436 // ne	0005737 // cy	0005515 // pr	Cell division c	CDC16_HUMAN|CDC16|ANAPC6
1904668 // po		0005515 // pr	Cell division c	CD20B_HUMAN|CDC20B|G6VTS76519
0016567 // pr	0005654 // nu	0004842 // ub	Ubiquitin-con	UB2R1_HUMAN|CDC34|UBCH3|UBE2R1
0050821 // pr	0005829 // cy	0005515 // pr	Hsp90 co-cha	CDC37_HUMAN|CDC37|CDC37A
0007163 // es	0030027 // la	0004672 // pr	Serine/threon	MRCKB_HUMAN|CDC42BPB|KIAA1124
0006468 // pr	0031252 // ce	0005524 // AT	Serine/threon	MRCKG_HUMAN|CDC42BPG|DMPK2
0030036 // ac	0012505 // en	0005515 // pr	Cdc42 effecto	BORG1_HUMAN|CDC42EP2|BORG1|CEP2

0007165 // sig	0012505 // en	0005519 // cy	Cdc42 effecto	BORG2_HUMAN|CDC42EP3|BORG2|CEP3
0043547 // pc	0012505 // en	0005096 // GT	Cdc42 effecto	BORG3_HUMAN|CDC42EP5|BORG3|CEP5
0008360 // re	0005737 // cy	0005198 // str	CDC42 small e	C42S1_HUMAN|CDC42SE1|SPEC1
0006260 // DI	0031298 // re	0005515 // pr	Cell division c	CDC45_HUMAN|CDC45|CDC45L|CDC45L2|UNQ374/PRC
0000076 // DI	0005654 // nu	0000166 // nu	Cell division c	CDC6_HUMAN|CDC6|CDC18L
0035307 // pc	0005694 // ch		Cell division c	CDCA2_HUMAN|CDCA2
	0005829 // cy	0005515 // pr	Cell division c	CDCA4_HUMAN|CDCA4|HEPP
0031175 // ne	0015629 // ac	0045295 // ga	Cadherin-1	CADH1_HUMAN|CDH1|CDHE|UVO
0034332 // ac	0016021 // in	0005509 // ca	Cadherin-10	CAD10_HUMAN|CDH10
0034332 // ac	0016021 // in	0005509 // ca	Cadherin-12	CAD12_HUMAN|CDH12
0034332 // ac	0005886 // pl	0005509 // ca	Cadherin-15	CAD15_HUMAN|CDH15|CDH14|CDH3
0033626 // pc	0016021 // in	0005215 // tra	Cadherin-17	CAD17_HUMAN|CDH17
0034332 // ac	0016021 // in	0005509 // ca	Cadherin-18	CAD18_HUMAN|CDH18|CDH14
0007156 // hc	0016021 // in	0005509 // ca	Cadherin-19	CAD19_HUMAN|CDH19|CDH7L2|UNQ478/PRO941
0016339 // ca	0016324 // ap	0045294 // alp	Cadherin-2	CADH2_HUMAN|CDH2|CDHN|NCAD
0007626 // lo	0016020 // m	0005515 // pr	Cadherin-23	CAD23_HUMAN|CDH23|KIAA1774|KIAA1812|UNQ1894
0034332 // ac	0016021 // in	0005509 // ca	Cadherin-24	CAD24_HUMAN|CDH24|CDH11L|UNQ2834/PRO34009
0007156 // hc	0016021 // in	0005509 // ca	Cadherin-like	CAD26_HUMAN|CDH26
0031424 // ke	0005737 // cy	0005509 // ca	Cadherin-3	CADH3_HUMAN|CDH3|CDHP
0034332 // ac	0005886 // pl	0005509 // ca	Cadherin-4	CADH4_HUMAN|CDH4
0034332 // ac	0005886 // pl	0044325 // io	Cadherin-5	CADH5_HUMAN|CDH5
0034332 // ac	0016021 // in	0005509 // ca	Cadherin-6	CADH6_HUMAN|CDH6
0034332 // ac	0016021 // in	0005509 // ca	Cadherin-7	CADH7_HUMAN|CDH7|CDH7L1
0009409 // re	0016021 // in	0005509 // ca	Cadherin-8	CADH8_HUMAN|CDH8
0046718 // vi	0016021 // in	0001618 // vi	Cadherin-rela	CDHR3_HUMAN|CDHR3|CDH28
0090675 // in	0016324 // ap	0008013 // be	Cadherin-rela	CDHR5_HUMAN|CDHR5|MUCDHL|MUPCDH|UNQ2781/
0042771 // in	0098574 // cy	0005515 // pr	Cell death-ind	CDIP1_HUMAN|CDIP1|C16orf5|CDIP|LITAFL
0043410 // pc	0016592 // m	0004693 // cy	Cyclin-depend	CDK10_HUMAN|CDK10
0006468 // pr	0005634 // nu	0005524 // AT	Cyclin-depend	CD11A_HUMAN|CDK11A|CDC2L2|CDC2L3|PITSLREB
0006468 // pr	0005634 // nu	0005515 // pr	Cyclin-depend	CD11B_HUMAN|CDK11B|CDC2L1|CDK11|PITSLREA|PK5
0007088 // re	0002945 // cy	0003723 // RN	Cyclin-depend	CDK13_HUMAN|CDK13|CDC2L|CDC2L5|CHED|KIAA179
0016055 // W	0000308 // cy	0005515 // pr	Cyclin-depend	CDK14_HUMAN|CDK14|KIAA0834|PFTK1
0006468 // pr	0005829 // cy	0004674 // pr	Cyclin-depend	CDK15_HUMAN|CDK15|ALS2CR7|PFTK2
0006887 // ex	0031234 // ex	0004674 // pr	Cyclin-depend	CDK16_HUMAN|CDK16|PCTAIRE1|PCTK1
0006468 // pr	0005737 // cy	0005515 // pr	Cyclin-depend	CDK17_HUMAN|CDK17|PCTAIRE2|PCTK2
0006468 // pr	0005737 // cy	0005515 // pr	Cyclin-depend	CDK18_HUMAN|CDK18|PCTAIRE3|PCTK3
0006468 // pr	0005737 // cy	0019912 // cy	Cyclin-depend	CDK20_HUMAN|CDK20|CCRK|CDCH
0060079 // ex	0030027 // la	0005515 // pr	Cyclin-depend	CDK5_HUMAN|CDK5|CDKN5
0071902 // pc	0005886 // pl	0005515 // pr	Cyclin-depend	CD5R1_HUMAN|CDK5R1|CDK5R|NCK5A
0035600 // tR	0005739 // m	0019901 // pr	CDK5 regulato	CK5P1_HUMAN|CDK5RAP1|C20orf34|CGI-05|HSPC167
0006362 // tra	0005654 // nu	0005515 // pr	Cyclin-depend	CDK7_HUMAN|CDK7|CAK|CAK1|CDKN7|MO15|STK1
0006468 // pr	0043231 // in	0004693 // cy	Cyclin-depend	CDKL1_HUMAN|CDKL1
0006468 // pr	0005737 // cy	0004693 // cy	Cyclin-depend	CDKL2_HUMAN|CDKL2
0006468 // pr	0005737 // cy	0005515 // pr	Cyclin-depend	CDKL3_HUMAN|CDKL3|NKIAMRE
0043068 // pc	0005829 // cy	0005515 // pr	Cyclin-depend	CDN1A_HUMAN|CDKN1A|CAP20|CDKN1|CIP1|MDA6|P
0060770 // ne	0031464 // Cu	0005515 // pr	Cyclin-depend	CDN1B_HUMAN|CDKN1B|KIP1
0071901 // ne	0005737 // cy	0005515 // pr	Cyclin-depend	CDN1C_HUMAN|CDKN1C|KIP2

0000082 // G1	0005634 // nu	0005515 // pr	Cyclin-depend	CDN2A_HUMAN|CDKN2A|CDKN2|MTS1
0007165 // sig	0005654 // nu		CDKN2AIP N-t	C2AIL_HUMAN|CDKN2AIPNL
0071901 // ne	0005829 // cy	0005515 // pr	Cyclin-depend	CDN2D_HUMAN|CDKN2D
0007050 // ce	0005634 // nu	0005515 // pr	Cyclin-depend	CDKN3_HUMAN|CDKN3|CDI1|CIP2|KAP
0031175 // ne	0005615 // ex	0008083 // gr	Cerebral dopa	CDNF_HUMAN|CDNF|ARMETL1
0010172 // er	0005886 // pl	0005515 // pr	Cell adhesion	CDON_HUMAN|CDON|CDO
		0005515 // pr	Cysteine-rich	CDPF1_HUMAN|CDPF1|C22orf40
		0005515 // pr	Cerebellar deg	CDR1_HUMAN|CDR1
0008150 // bi	0005575 // ce	0003674 // m	CMT1A duplic	CDRT1_HUMAN|CDRT1|C17orf1|C17orf1A|HREP
0007165 // sig	0005789 // er	0004142 // di	Phosphatidate	CDS1_HUMAN|CDS1|CDS
0031334 // po	0005654 // nu	0005515 // pr	DNA replicatio	CDT1_HUMAN|CDT1
0008283 // ce	0005737 // cy	0003674 // m	Protein CDV3	CDV3_HUMAN|CDV3|H41
0006351 // tra	0016604 // nu	0005515 // pr	Chromodoma	CDYL1_HUMAN|CDYL|CDYL1
0008152 // m	0005634 // nu	0035064 // m	Chromodoma	CDYL2_HUMAN|CDYL2
0001558 // re	0005911 // ce	0019900 // kin	Carcinoembry	CEAM1_HUMAN|CEACAM1|BGP|BGP1
	0016021 // int	0005515 // pr	Carcinoembry	CEA19_HUMAN|CEACAM19|CEAL1|UNQ2973/PRO7436
0008284 // po	0031225 // an	0005515 // pr	Carcinoembry	CEAM6_HUMAN|CEACAM6|NCA
0006351 // tra	0005730 // nu	0019900 // kin	CCAAT/enhan	CEBPA_HUMAN|CEBPA|CEBP
0045944 // po	0005654 // nu	0042803 // pr	CCAAT/enhan	CEBPD_HUMAN|CEBPD
0044258 // int	0005737 // cy	0047372 // ac	Bile salt-activa	CEL_HUMAN|CEL|BAL
0006508 // pr	0005576 // ex	0004252 // se	Chymotrypsin	CEL2B_HUMAN|CELA2B|ELA2B
0030575 // nu	0005737 // cy	0003723 // RN	CUGBP Elav-li	CELF3_HUMAN|CELF3|BRUNOL1|CAGH4|ERDA4|TNRC4
0006397 // m	0005737 // cy	0036002 // pr	CUGBP Elav-li	CELF5_HUMAN|CELF5|BRUNOL5
0001736 // es	0005887 // int	0004930 // G-	Cadherin EGF	CELR1_HUMAN|CELSR1|CDHF9|FMI2
0007156 // ho	0005737 // cy	0004930 // G-	Cadherin EGF	CELR2_HUMAN|CELSR2|CDHF10|EGFL2|KIAA0279|MEG
0030213 // hy	0005737 // cy	0005515 // pr	Cell migration	CEMIP_HUMAN|CEMIP|KIAA1199
	0031618 // nu	0019237 // ce	Major centror	CENPB_HUMAN|CENPB
0034080 // CE	0005829 // cy	0019237 // ce	Centromere p	CENPC_HUMAN|CENPC|CENPC1|ICEN7
0015031 // pr	0005813 // ce	0008022 // pr	Centromere p	CENPF_HUMAN|CENPF
0034080 // CE	0005829 // cy	0005515 // pr	Centromere p	CENPI_HUMAN|CENPI|FSHPRH1|ICEN19|LRPR1
0034080 // CE	0005829 // cy	0005515 // pr	Centromere p	CENPK_HUMAN|CENPK|ICEN37|FKSG14
0034080 // CE	0005829 // cy		Centromere p	CENPM_HUMAN|CENPM|C22orf18|ICEN39|PANE1
0034080 // CE	0005829 // cy	0005515 // pr	Centromere p	CENPO_HUMAN|CENPO|ICEN36|MCM21R
0031297 // re	0005654 // nu	0005515 // pr	Centromere p	CENPS_HUMAN|CENPS|APITD1|FAAP16|MHF1
0016032 // vi	0005829 // cy	0005515 // pr	Centromere p	CENPU_HUMAN|CENPU|ICEN24|KLIP1|MLF1IP|PBIP1
0001667 // ar	0005634 // nu	0016846 // ca	Centromere p	CENPV_HUMAN|CENPV|PRR6
0007059 // ch	0005730 // nu	0005515 // pr	Centromere p	CENPW_HUMAN|CENPW|C6orf173|CUG2

0031297 // re	0005654 // nu	0005515 // pr	Centromere p	CENPX_HUMAN	CENPX|FAAP10|MHF2|STRA13
0097120 // re	0005737 // cy		Centrosomal p	CE112_HUMAN	CEP112|CCDC46
0007052 // m	0005737 // cy	0005515 // pr	Centrosomal p	CE126_HUMAN	CEP126|KIAA1377
	0000922 // sp		Centrosomal p	CE128_HUMAN	CEP128|C14orf145|C14orf61
0000086 // G2	0005829 // cy	0005515 // pr	Centrosomal p	CP131_HUMAN	CEP131|AZI1|KIAA1118
0000086 // G2	0005829 // cy	0019901 // pr	Centrosomal p	CE152_HUMAN	CEP152|KIAA0912
0060271 // cil	0005829 // cy	0005515 // pr	Centrosomal p	CE162_HUMAN	CEP162|C6orf84|KIAA1009|QN1
	0005814 // ce	0005515 // pr	Centrosomal p	CE170_HUMAN	CEP170|FAM68A|KAB|KIAA0470
	0005737 // cy		Centrosomal p	C170B_HUMAN	CEP170B|FAM68C|KIAA0284
		0005515 // pr	Cep170-like p	C170L_HUMAN	CEP170P1|CEP170L|KIAA0470L
0034453 // m	0005634 // nu	0008017 // m	Centrosome-a	CE350_HUMAN	CEP350|CAP350|KIAA0480|GM133
0000086 // G2	0005829 // cy	0005515 // pr	Centrosomal p	CEP41_HUMAN	CEP41|TSGA14
	0005737 // cy	0005515 // pr	Centrosomal p	CEP44_HUMAN	CEP44|KIAA1712
0000086 // G2	0005829 // cy	0005515 // pr	Centrosomal p	CEP70_HUMAN	CEP70|BITE
0000086 // G2	0005829 // cy	0005515 // pr	Centrosomal p	CEP72_HUMAN	CEP72|KIAA1519
	0005737 // cy		Centrosomal p	CE85L_HUMAN	CEP85L|C6orf204
0007159 // le	0005788 // er		Probable inac	GT253_HUMAN	CERCAM|CEECAM1|GLT25D3|KIAA150
0046834 // lip	0005739 // m	0005515 // pr	Ceramide kina	CERK1_HUMAN	CERK|KIAA1646
0016310 // ph	0001750 // ph	0046625 // sp	Ceramide kina	CERKL_HUMAN	CERKL
0035690 // ce	0043231 // in	0050291 // sp	Ceramide synt	CERS1_HUMAN	CERS1|LAG1|LASS1|UOG1
1905045 // ne	0016021 // in	0005515 // pr	Ceramide synt	CERS2_HUMAN	CERS2|LASS2|TMSG1
0030216 // ke	0016021 // in	0050291 // sp	Ceramide synt	CERS3_HUMAN	CERS3|LASS3
0030148 // sp	0016021 // in	0050291 // sp	Ceramide synt	CERS4_HUMAN	CERS4|LASS4
0030148 // sp	0016021 // in	0050291 // sp	Ceramide synt	CERS6_HUMAN	CERS6|LASS6
0006805 // xe	0005783 // er	0052689 // ca	Cocaine ester	EST2_HUMAN	CES2|ICE
0006805 // xe	0005788 // er	0052689 // ca	Carboxylester	EST3_HUMAN	CES3|UNQ869/PRO1887
	0005576 // ex	0052689 // ca	Carboxylester	EST4A_HUMAN	CES4A|CES8|UNQ440/PRO873
0007288 // sp	0036064 // cil	0008017 // m	Cilia- and flag	CF157_HUMAN	CFAP157|C9orf117
	0005634 // nu		Cilia- and flag	CFA36_HUMAN	CFAP36|CCDC104|UNQ163/PRO189
	0042995 // ce		Cilia- and flag	CFA43_HUMAN	CFAP43|C10orf79|WDR96
0035082 // ax	0005737 // cy		Cilia- and flag	CFA46_HUMAN	CFAP46|C10orf123|C10orf124|C10orf9
	0005737 // cy	0005515 // pr	Cilia- and flag	CFA52_HUMAN	CFAP52|WDR16|WDRPUH
0003341 // cil	0005575 // ce	0005515 // pr	Cilia- and flag	CFA53_HUMAN	CFAP53|CCDC11
0060271 // cil	0005737 // cy		Cilia- and flag	CFA54_HUMAN	CFAP54|C12orf55|C12orf63
	0005615 // ex	0005515 // pr	Cilia- and flag	CFA58_HUMAN	CFAP58|C10orf80|CCDC147
0008150 // bi	0070062 // ex	0003674 // m	Cilia- and flag	CFA70_HUMAN	CFAP70|TTC18
0035082 // ax	0005737 // cy		Cilia- and flag	CFA74_HUMAN	CFAP74|C1orf222|KIAA1751
	0042995 // ce		Cilia- and flag	CFA99_HUMAN	CFAP99
0006508 // pr	0005886 // pl	0004252 // se	Complement	CFAB_HUMAN	CFB|BF|BFD

0002576 // pl	0005576 // ex	0004252 // se	Complement f	CFAD_HUMAN|CFD|DF|PFD
0016032 // vi	0005576 // ex	0008201 // he	Complement f	CFAH_HUMAN|CFH|HF|HF1|HF2
0006956 // co	0005576 // ex	0042803 // pr	Complement f	FHR1_HUMAN|CFHR1|CFHL|CFHL1|CFHL1P|CFHR1P|FH
	0070062 // ex		Complement f	FHR3_HUMAN|CFHR3|CFHL3|FHR3
0030449 // re	0005576 // ex	0005319 // lip	Complement f	FHR4_HUMAN|CFHR4|CFHL4|FHR4
0006957 // co	0005576 // ex	0005515 // pr	Complement f	FHR5_HUMAN|CFHR5|CFHL5|FHR5
0030043 // ac	0031674 // I b	0005515 // pr	Cofilin-2	COF2_HUMAN|CFL2
0015701 // bi	0005886 // pl	0005515 // pr	Cystic fibrosis	CFTR_HUMAN|CFTR|ABCC7
0006915 // ap	0005576 // ex	0005179 // ho	Choriogonado	CGB7_HUMAN|CGB7
0007179 // tr	0030054 // ce	0003779 // ac	Cingulin	CING_HUMAN|CGN|KIAA1319
0051058 // ne	0016459 // m	0003774 // m	Cingulin-like p	CGNL1_HUMAN|CGNL1|JACOP|KIAA1749
0007050 // ce	0005576 // ex	0005509 // ca	Cell growth re	CGRE1_HUMAN|CGREF1|CGR11
0007050 // ce	0043231 // in	0046872 // m	Cell growth re	CGRF1_HUMAN|CGRRF1|CGR19|RNF197
0035754 // B	0005829 // cy	0001567 // ch	Cholesterol 2	CH25H_HUMAN|CH25H

0006751 // gl	0005737 // cy	0003839 // ga	Putative gluta	CHAC2_HUMAN|CHAC2
0019221 // cy	0005737 // cy	0004860 // pr	Chondroadhe	CHAD_HUMAN|CHAD|SLRR4A
0032331 // ne	0031012 // ex	0005518 // co	Chondroadhe	CHADL_HUMAN|CHADL|SLRR4B
0006461 // pr	0033186 // CA	0042393 // his	Chromatin ass	CAF1B_HUMAN|CHAF1B|CAF1A|CAF1P60|MPHOSPH7|
0051315 // at	0000793 // co	0005515 // pr	Chromosome	CHAP1_HUMAN|CHAMP1|C13orf8|CAMP|CHAMP|KIAA
0070126 // m	0005743 // m	0003723 // RN	Coiled-coil-he	CHCH1_HUMAN|CHCHD1|C10orf34|MRPS37
0045041 // pr	0005758 // m	0015035 // pr	Mitochondria	MIA40_HUMAN|CHCHD4|MIA40
0032508 // DI	0001650 // fib	0005515 // pr	Chromodoma	CHD1_HUMAN|CHD1
0033683 // nu	0005654 // nu	0016887 // AT	Chromodoma	CHD1L_HUMAN|CHD1L|ALC1
0003222 // ve	0005634 // nu	0003682 // ch	Chromodoma	CHD7_HUMAN|CHD7|KIAA1416
0000209 // pr	0005634 // nu	0061630 // ub	E3 ubiquitin-p	CHFR_HUMAN|CHFR|RNF196
0006629 // lip	0005829 // cy	0004104 // ch	Choline kinase	CHKA_HUMAN|CHKA|CHK|CKI
1902188 // po	0000815 // ES	0005515 // pr	Charged multi	CHM2A_HUMAN|CHMP2A|BC2|CHMP2
0007034 // va	0000815 // ES	0005515 // pr	Charged multi	CHM4A_HUMAN|CHMP4A|C14orf123|SHAX2|CDA04|H
0006620 // po	0000815 // ES	0005515 // pr	Charged multi	CHM4B_HUMAN|CHMP4B|C20orf178|SHAX1
0007034 // va	0070062 // ex	0005515 // pr	Charged multi	CHM4C_HUMAN|CHMP4C|SHAX3
0046755 // vi	0070062 // ex	0005515 // pr	Charged multi	CHMP5_HUMAN|CHMP5|C9orf83|SNF7DC2|CGI-34|HS
0007034 // va	0070062 // ex	0005515 // pr	Charged multi	CHMP6_HUMAN|CHMP6|VPS20
0009967 // po	0005737 // cy	0005515 // pr	N-chimaerin	CHIN_HUMAN|CHN1|ARHGAP2|CHN
0009967 // po	0016020 // m	0005070 // SH	Beta-chimaeri	CHIO_HUMAN|CHN2|ARHGAP3|BCH
0007517 // m	0005737 // cy	0030246 // ca	Chondrolectin	CHODL_HUMAN|CHODL|C21orf68|PRED12|UNQ872/PR
0030206 // ch	0016021 // in	0050510 // N-	Chondroitin su	CHPF2_HUMAN|CHPF2|CHSY3|CSGLCAT|KIAA1402|UN
0006663 // pl	0016021 // in	0004142 // di	Cholinephosp	CHPT1_HUMAN|CHPT1|CPT1|MSTP022
0007399 // ne	0005788 // er		Chordin-like p	CRDL1_HUMAN|CHRDL1|NRLN1
0001503 // os	0005737 // cy		Chordin-like p	CRDL2_HUMAN|CHRDL2|BNF1|CHL2|UNQ765/PRO155
0034220 // io	0016021 // in	0005230 // ex	CHRNA7-FAM	CRFM7_HUMAN|CHRFAM7A
0006464 // ce	0016020 // m	0008144 // dr	Muscarinic ac	ACM1_HUMAN|CHRM1
0045987 // po	0005886 // pl	0042166 // ac	Muscarinic ac	ACM3_HUMAN|CHRM3
0007271 // sy	0045211 // po	0022848 // ac	Neuronal acet	ACH10_HUMAN|CHRNA10|NACHRA10
0034220 // io	0005886 // pl	0015276 // lig	Neuronal acet	ACHA2_HUMAN|CHRNA2
0098655 // ca	0005886 // pl	0015276 // lig	Neuronal acet	ACHA6_HUMAN|CHRNA6
0032225 // re	0016021 // in	0005515 // pr	Neuronal acet	ACHA7_HUMAN|CHRNA7|NACHRA7
0095500 // ac	0005886 // pl	0015276 // lig	Neuronal acet	ACHB2_HUMAN|CHRNB2
0001508 // ac	0035579 // sp	0015276 // lig	Neuronal acet	ACHB4_HUMAN|CHRNB4
0006936 // m	0005886 // pl	0022848 // ac	Acetylcholine	ACHE_HUMAN|CHRNE|ACHRE
0007155 // ce	0005794 // Go	0008146 // su	Carbohydrate	CHSTA_HUMAN|CHST10
0009791 // po	0000139 // Go	0047756 // ch	Carbohydrate	CHSTB_HUMAN|CHST11
0019319 // he	0016021 // in	0050659 // N-	Carbohydrate	CHSTF_HUMAN|CHST15|BRAG|GALNAC4S6ST|KIAA059
0007275 // m	0016021 // in	0001517 // N-	Carbohydrate	CHST2_HUMAN|CHST2|GN6ST
0006790 // su	0016021 // in	0001517 // N-	Carbohydrate	CHST3_HUMAN|CHST3
0031667 // re	0016021 // in	0047238 // gl	Chondroitin su	CHSS1_HUMAN|CHSY1|CHSY|CSS1|KIAA0990|UNQ756/
0030206 // ch	0016021 // in	0047238 // gl	Chondroitin su	CHSS3_HUMAN|CHSY3|CHSY2|CSS3
0007275 // m		0008270 // zi	Protein Churc	CHUR_HUMAN|CHURC1|C14orf52|CHCH|My015

0007059 // ch	0097361 // CI	0005515 // pr	Probable cyto	CIAO1_HUMAN|CIAO1|WDR39
0045892 // ne	0016605 // PN	0070888 // E-	Circadian-asso	CIART_HUMAN|CIART|C1orf51
0071318 // ce	0042383 // sa	0005515 // pr	Calcium and in	CIB2_HUMAN|CIB2|KIP2
0050995 // ne	0005739 // m	0042803 // pr	Cell death act	CIDEA_HUMAN|CIDEA
0043569 // ne	0031012 // ex	0004551 // nu	Cartilage inter	CILP1_HUMAN|CILP|UNQ602/PRO1188
	0070062 // ex	0004551 // nu	Cartilage inter	CILP2_HUMAN|CILP2
0051301 // ce	0005634 // nu	0005515 // pr	Cyclin-depend	CINP_HUMAN|CINP
0045892 // ne	0005634 // nu	0005515 // pr	CLOCK-interac	CIPC_HUMAN|CIPC|KIAA1737
0043457 // re	0005739 // m	0051537 // 2 i	CDGSH iron-su	CISD1_HUMAN|CISD1|C10orf70|ZCD1|MDS029
0000278 // m	0005829 // cy	0005515 // pr	Citron Rho-int	CTRO_HUMAN|CIT|CRIK|KIAA0949|STK21
0001658 // br	0005737 // cy	0005515 // pr	Cbp/p300-inte	CITE1_HUMAN|CITED1|MSG1
0045893 // po	0005654 // nu	0003714 // tra	Cbp/p300-inte	CITE2_HUMAN|CITED2|MRG1
0032502 // de	0005654 // nu	0003713 // tra	Cbp/p300-inte	CITE4_HUMAN|CITED4|MRG2
0006600 // cr	0030425 // de	0004111 // cr	Creatine kinas	KCRB_HUMAN|CKB|CKBB
0048246 // m	0005576 // ex	0008009 // ch	Chemokine-lik	CKLF_HUMAN|CKLF|CKLF1|HSPC224|UNQ410/PRO772
0006600 // cr	0070062 // ex	0005524 // AT	Creatine kinas	KCRU_HUMAN|CKMT1A|CKMT1B|CKMT|CKMT
0006508 // pr	0009925 // ba	0005254 // ch	Calcium-activa	CLCA2_HUMAN|CLCA2|CACC3
1902476 // ch	0005615 // ex	0004222 // m	Calcium-activa	CLCA3_HUMAN|CLCA3P|CLCA3
0006508 // pr	0070062 // ex	0004222 // m	Calcium-activa	CLCA4_HUMAN|CLCA4|CaCC2|UNQ562/PRO1124
0043524 // ne	0005576 // ex	0005102 // re	Cardiotrophin	CLCF1_HUMAN|CLCF1|BSF3|CLC|NNT1
1902476 // ch	0005887 // in	0005247 // vo	Chloride chan	CLCN1_HUMAN|CLCN1|CLC1
0006810 // tra	0005886 // pl	0005247 // vo	Chloride chan	CLCN2_HUMAN|CLCN2
1902476 // ch	0008021 // sy	0005515 // pr	H(+)/Cl(-) exch	CLCN3_HUMAN|CLCN3
1902476 // ch	0005794 // Go	0005247 // vo	H(+)/Cl(-) exch	CLCN5_HUMAN|CLCN5|CLCK2
0006810 // tra	0005886 // pl	0005247 // vo	Chloride chan	CLCKA_HUMAN|CLCNKA
0007588 // ex	0005886 // pl	0005247 // vo	Chloride chan	CLCKB_HUMAN|CLCNKB
0016338 // ca	0005737 // cy	0005198 // str	Claudin-10	CLD10_HUMAN|CLDN10
0008366 // ax	0016021 // in	0042802 // id	Claudin-11	CLD11_HUMAN|CLDN11|OSP|OTM
0016338 // ca	0016021 // in	0005198 // str	Claudin-12	CLD12_HUMAN|CLDN12
0016338 // ca	0016021 // in	0005198 // str	Claudin-16	CLD16_HUMAN|CLDN16|PCLN1
0016338 // ca	0016021 // in	0005515 // pr	Claudin-2	CLD2_HUMAN|CLDN2|PSEC0059|SP82|UNQ705/PRO13
0016338 // ca	0016021 // in	0005198 // str	Claudin-22	CLD22_HUMAN|CLDN22
0016338 // ca	0016021 // in	0005198 // str	Claudin-23	CLD23_HUMAN|CLDN23
	0016021 // in	0005198 // str	Putative clauc	CLD24_HUMAN|CLDN24|CLDN21
0045471 // re	0016327 // ap	0004888 // tra	Claudin-3	CLD3_HUMAN|CLDN3|C7orf1|CPETR2
0016338 // ca	0005887 // in	0004888 // tra	Claudin-4	CLD4_HUMAN|CLDN4|CPER|CPETR1|WBSCR8
0043066 // ne	0016021 // in	0050839 // ce	Claudin-7	CLD7_HUMAN|CLDN7|CEPTRL2|CPETRL2
0016338 // ca	0016021 // in	0005198 // str	Claudin-9	CLD9_HUMAN|CLDN9
	0016021 // in		Claudin doma	CLDN2_HUMAN|CLDND2
0001503 // os	0005737 // cy	0008083 // gr	C-type lectin d	CLC11_HUMAN|CLEC11A|CLECSF3|LSLCL|SCGF
0007165 // sig	0005887 // in	0030246 // ca	Putative C-typ	CL20A_HUMAN|CLEC20A|LINC00083|NCRNA00083
0050776 // re	0005886 // pl	0030246 // ca	C-type lectin d	CLC2B_HUMAN|CLEC2B|AICL|CLECSF2|IFNRG1
0002223 // sti	0005887 // in	0030246 // ca	C-type lectin d	CLC4A_HUMAN|CLEC4A|CLECSF6|DCIR|LLIR|HDCGC13P

0006897 // er	0016021 // in	0030246 // ca	C-type lectin d	CLC4F_HUMAN	CLC4F_HUMAN|CLEC4F|CLECSF13
0006457 // pr	0016021 // in	0005515 // pr	Calmegin	CLGN_HUMAN|CLGN
0006749 // gl	0070062 // ex	0045296 // ca	Chloride intra	CLIC1_HUMAN|CLIC1|G6|NCC27
0006749 // gl	0016604 // nu	0005515 // pr	Chloride intra	CLIC3_HUMAN|CLIC3
0001525 // ar	0045177 // ap	0005515 // pr	Chloride intra	CLIC4_HUMAN|CLIC4
0007605 // se	0005938 // ce	0005515 // pr	Chloride intra	CLIC5_HUMAN|CLIC5
0001934 // pc	0005829 // cy	0008017 // m	CAP-Gly doma	CLIP3_HUMAN|CLIP3|CLIPR59
	0043231 // in	0005515 // pr	CAP-Gly doma	CLIP4_HUMAN|CLIP4|RSNL2
0045721 // ne	0016604 // nu	0005515 // pr	Dual specificit	CLK2_HUMAN|CLK2
0046777 // pr	0001669 // ac	0004674 // pr	Dual specificit	CLK3_HUMAN|CLK3
0046777 // pr	0005634 // nu	0004674 // pr	Dual specificit	CLK4_HUMAN|CLK4
0031175 // ne	0005737 // cy	0003779 // ac	Calmin	CLMN_HUMAN|CLMN|KIAA1188
0048565 // di	0005923 // bi		CXADR-like m	CLMP_HUMAN|CLMP|ACAM|ASAM|UNQ318/PRO363
0008203 // ch	0005783 // er		Protein CLN8	CLN8_HUMAN|CLN8|C8orf61
0032922 // ci	0005654 // nu	0005515 // pr	Circadian loco	CLOCK_HUMAN|CLOCK|BHLHE8|KIAA0334
0051603 // pr	0005739 // m	0005515 // pr	ATP-depende	CLPP_HUMAN|CLPP
0006915 // ap	0016021 // in		Cleft lip and p	CLP1L_HUMAN|CLPTM1L|CRR9
0006260 // DI	0005794 // Go	0005515 // pr	Claspin	CLSPN_HUMAN|CLSPN
0051965 // pc	0016021 // in	0019894 // ki	Calsyntenin-1	CSTN1_HUMAN|CLSTN1|CS1|KIAA0911
0051965 // pc	0045211 // pc	0005509 // ca	Calsyntenin-2	CSTN2_HUMAN|CLSTN2|CS2
0072583 // cl	0005829 // cy	0042277 // pe	Clathrin light	CLCA_HUMAN|CLTA
0048268 // cl	0071439 // cl	0005515 // pr	Clathrin heavy	CLH2_HUMAN|CLTCL1|CLH22|CLTCL|CLTD
0006956 // cc	0042583 // ch	0031625 // ub	Clusterin	CLUS_HUMAN|CLU|APOJ|CLI|KUB1|AAG4
0035735 // in	0097542 // cil	0005515 // pr	Clusterin-asso	CLUA1_HUMAN|CLUAP1|KIAA0643
	0005576 // ex		Clusterin-like	CLUL1_HUMAN|CLUL1
	0005739 // m	0004474 // m	Citrate lyase s	CLYBL_HUMAN|CLYBL|CLB
0006054 // N-	0005654 // nu	0008781 // N-	N-acylneuram	NEUA_HUMAN|CMAS
0008283 // ce	0005739 // m		Cx9C motif-co	CMC4_HUMAN|CMC4|C6.1B|MTCP1|MTCP1NB
	0005829 // cy	0005515 // pr	C-Maf-inducir	CMIP_HUMAN|CMIP|KIAA1694|TCMIP
	0005634 // nu	0003723 // RN	Protein CMSS	CMS1_HUMAN|CMSS1|C3orf26
0006935 // ch	0031965 // nu	0005515 // pr	CKLF-like MAF	CKLF3_HUMAN|CMTM3|CKLFSF3
0006935 // ch	0016021 // in	0005125 // cy	CKLF-like MAF	CKLF4_HUMAN|CMTM4|CKLFSF4
0006935 // ch	0035579 // sp	0005125 // cy	CKLF-like MAF	CKLF6_HUMAN|CMTM6|CKLFSF6
0008104 // pr	0045121 // m	0005515 // pr	CKLF-like MAF	CKLF8_HUMAN|CMTM8|CKLFSF8
0006750 // gl	0070062 // ex	0008237 // m	Cytosolic non-	CNDP2_HUMAN|CNDP2|CN2|CPGL|HEL-S-13|PEPA
0031424 // ke	0001533 // co		Cornifelin	CNFN_HUMAN|CNFN
0050908 // de	0005886 // pl	0015276 // lig	Cyclic nucleot	CNGB1_HUMAN|CNGB1|CNCG2|CNCG3L|CNCG4|RCNC
0006810 // tr	0005887 // in	0030553 // cG	Cyclic nucleot	CNGB3_HUMAN|CNGB3
0006888 // EF	0005789 // er	0016247 // ch	Protein cornic	CNIH3_HUMAN|CNIH3
0007266 // Rh	0005938 // ce	0005515 // pr	Connector enl	CNKR1_HUMAN|CNKSR1|CNK1
0009966 // re	0014069 // pc	0042802 // id	Connector enl	CNKR2_HUMAN|CNKSR2|CNK2|KIAA0902|KSR2
0033137 // ne	0005737 // cy	0005515 // pr	Connector enl	CNKR3_HUMAN|CNKSR3|MAGI1

0030855 // ep	0043025 // ne	0098641 // ca	Calponin-3	CNN3_HUMAN|CNN3
0050896 // re	0016021 // int	0005515 // pr	Metal transpo	CNNM4_HUMAN|CNNM4|ACDP4|KIAA1592
0000122 // ne	0000932 // cy	0019904 // pr	CCR4-NOT tra	CNOT1_HUMAN|CNOT1|CDC39|KIAA1007|NOT1|AD-00
0006977 // DI	0005634 // nu	0005515 // pr	CCR4-NOT tra	CNOT6_HUMAN|CNOT6|CCR4|CCR4a|KIAA1194
0006977 // DI	0030015 // CC	0005515 // pr	CCR4-NOT tra	CNOT8_HUMAN|CNOT8|CALIF|POP2
0000079 // re	0016021 // int	0019901 // pr	Protein CNPPI	CNPD1_HUMAN|CNPPD1|C2orf24|CDABP0125|CGI-57
	0043209 // m		CB1 cannabin	CNRP1_HUMAN|CNRIP1|C2orf32
0007155 // ce	0016020 // m	0030246 // ca	Contactin-1	CNTN1_HUMAN|CNTN1
0007155 // ce	0031225 // ar		Contactin-5	CNTN5_HUMAN|CNTN5
0009967 // po	0043209 // m	0004872 // re	Contactin-asso	CNTP1_HUMAN|CNTNAP1|CASPR|NRXN4
0021794 // th	0009986 // ce	0005515 // pr	Contactin-asso	CNTP2_HUMAN|CNTNAP2|CASPR2|KIAA0868
0007155 // ce	0005576 // ex		Contactin-asso	CNTP3_HUMAN|CNTNAP3|CASPR3|KIAA1714
0007155 // ce	0016021 // int		Contactin-asso	CNT3B_HUMAN|CNTNAP3B|CASPR3B
0032228 // re	0016021 // int		Contactin-asso	CNTP4_HUMAN|CNTNAP4|CASPR4|KIAA1763
0000086 // G2	0005829 // cy	0004713 // pr	Centriolin	CNTRL_HUMAN|CNTRL|CEP1|CEP110
	0005739 // m		Cytochrome c	COA7_HUMAN|COA7|C1orf163|RESA1|SELRC1
0016310 // ph	0005741 // m	0005515 // pr	Bifunctional c	COASY_HUMAN|COASY|PSEC0106
0030041 // ac	0070062 // ex	0045296 // ca	Cordon-bleu p	COBL1_HUMAN|COBLL1|KIAA0977
0007605 // se	0031012 // ex	0005518 // co	Cochlin	COCH_HUMAN|COCH|COCH5B2|UNQ257/PRO294
0006888 // ER	0000139 // Go	0005515 // pr	Conserved oli	COG1_HUMAN|COG1|KIAA1381|LDLB
0006888 // ER	0000139 // Go	0005515 // pr	Conserved oli	COG4_HUMAN|COG4
0006891 // int	0000139 // Go	0005515 // pr	Conserved oli	COG5_HUMAN|COG5|GOLTC1|GTC90
0006888 // ER	0000139 // Go	0005515 // pr	Conserved oli	COG6_HUMAN|COG6|KIAA1134
0001503 // os	0005788 // en	0030674 // pr	Collagen alpha	COBA1_HUMAN|COL11A1|COLL6
0030574 // co	1903561 // ex	0030020 // ex	Collagen alpha	COCA1_HUMAN|COL12A1|COL12A1L
0030574 // co	0005576 // ex	0005515 // pr	Collagen alpha	CODA1_HUMAN|COL13A1
0030198 // ex	0005581 // co	0003723 // RN	Collagen alpha	COEA1_HUMAN|COL14A1|UND
0071230 // ce	0005576 // ex	0005515 // pr	Collagen alpha	COGA1_HUMAN|COL16A1|FP1572
0050776 // re	0005886 // pl	0005515 // pr	Collagen alpha	COHA1_HUMAN|COL17A1|BP180|BPAG2
0042493 // re	0005576 // ex	0046872 // m	Collagen alpha	COIA1_HUMAN|COL18A1
0030198 // ex	0005581 // co	0005201 // ex	Collagen alpha	COJA1_HUMAN|COL19A1
0060346 // bo	0005576 // ex	0005515 // pr	Collagen alpha	CO1A1_HUMAN|COL1A1
0007179 // tra	0005576 // ex	0005515 // pr	Collagen alpha	CO1A2_HUMAN|COL1A2
	0005788 // en		Collagen alpha	COKA1_HUMAN|COL20A1|KIAA1510
	0005788 // en		Collagen alpha	COLA1_HUMAN|COL21A1|COL1AL|FP633
0002244 // he	0005788 // en	0005201 // ex	Collagen alpha	COOA1_HUMAN|COL24A1
0007155 // ce	0031012 // ex	0004867 // se	Collagen alpha	COSA1_HUMAN|COL28A1|COL28
0071230 // ce	0005576 // ex	0005201 // ex	Collagen alpha	CO4A1_HUMAN|COL4A1
0030574 // co	0005576 // ex	0005201 // ex	Collagen alpha	CO4A2_HUMAN|COL4A2
0030574 // co	0005576 // ex	0005201 // ex	Collagen alpha	CO4A4_HUMAN|COL4A4
0030574 // co	0005576 // ex	0005201 // ex	Collagen alpha	CO4A5_HUMAN|COL4A5
0030574 // co	0005587 // co	0005201 // ex	Collagen alpha	CO4A6_HUMAN|COL4A6
0016477 // ce	0005788 // en	0005178 // int	Collagen alpha	CO5A1_HUMAN|COL5A1

0030574 // cc	0005588 // cc	0046332 // SN	Collagen alpha	CO5A2_HUMAN|COL5A2
0030574 // cc	0005576 // ex	0048407 // pl	Collagen alpha	CO6A1_HUMAN|COL6A1
0030574 // cc	0005576 // ex	0005515 // pr	Collagen alpha	CO6A2_HUMAN|COL6A2
0030574 // cc	0005576 // ex		Collagen alpha	CO6A6_HUMAN|COL6A6
0008544 // ep	0005576 // ex	0004867 // se	Collagen alpha	CO7A1_HUMAN|COL7A1
0030574 // cc	0005576 // ex	0005515 // pr	Collagen alpha	CO8A1_HUMAN|COL8A1|C3orf7
0030574 // cc	0005576 // ex	0030674 // pr	Collagen alpha	CO8A2_HUMAN|COL8A2
0001501 // sk	0005576 // ex	0030020 // ex	Collagen alpha	CO9A2_HUMAN|COL9A2
0008585 // fe	0005576 // ex	0030020 // ex	Collagen alpha	CO9A3_HUMAN|COL9A3
	0016021 // in		Colorectal car	COLC1_HUMAN|COLCA1|C11orf92
	0005737 // cy		Colorectal car	COLC2_HUMAN|COLCA2|C11orf93|CASC13
0006508 // pr	0005576 // ex	0004252 // se	Collectin-11	COL11_HUMAN|COLEC11|UNQ596/PRO1182
	0005788 // er	0005515 // pr	Procollagen ga	GT252_HUMAN|COLGALT2|C1orf17|GLT25D2|KIAA058
0090150 // es	0031594 // ne	0005515 // pr	Acetylcholines	COLQ_HUMAN|COLQ
0048227 // pl	0005829 // cy	0005515 // pr	COMM domai	COMD1_HUMAN|COMMD1|C2orf5|MURR1
0006351 // tra	0005634 // nu	0005515 // pr	COMM domai	COMDA_HUMAN|COMMD10|HSPC305|PTD002
	0016604 // nu		COMMD3-BM	R4GMX3_HUMAN|COMMD3-BMI1
0006351 // tra	0005737 // cy	0005515 // pr	COMM domai	COMD4_HUMAN|COMMD4
0006351 // tra	0070062 // ex	0051059 // NI	COMM domai	COMD7_HUMAN|COMMD7|C20orf92
0006351 // tra	0005794 // Go	0005515 // pr	COMM domai	COMD9_HUMAN|COMMD9|HSPC166
0006888 // EF	0005793 // er	0005515 // pr	Coatomer sub	COPB_HUMAN|COPB1|COPB|MSTP026
0006888 // EF	0005794 // Go	0005515 // pr	Coatomer sub	COPE_HUMAN|COPE
0043985 // hi	0005654 // nu	0042393 // hi	Coordinator o	COPRS_HUMAN|COPRS|C17orf79|COPR5
0000338 // pr	0005654 // nu	0005515 // pr	COP9 signalos	CSN3_HUMAN|COPS3|CSN3
0000338 // pr	0070062 // ex	0005515 // pr	COP9 signalos	CSN8_HUMAN|COPS8|CSN8
0034644 // ce	0005737 // cy	0005515 // pr	COP9 signalos	CSN9_HUMAN|COPS9|MYEOV2
0006888 // EF	0005789 // er		Coatomer sub	COPZ2_HUMAN|COPZ2
0045333 // ce	0005739 // m	0048039 // ub	Coenzyme Q-l	CQ10A_HUMAN|COQ10A|UNQ6192/PRO20219
0006468 // pr	0005739 // m	0004672 // pr	Atypical kinas	COQ8B_HUMAN|COQ8B|ADCK4
0045087 // in	0070062 // ex	0003723 // RN	Coronin-1A	COR1A_HUMAN|CORO1A|CORO1
0090135 // ac	0030027 // la	0005515 // pr	Coronin-1B	COR1B_HUMAN|CORO1B
0030036 // ac	0015629 // ac	0051015 // ac	Coronin-2A	COR2A_HUMAN|CORO2A|IR10|WDR2
0030036 // ac	0005737 // cy	0003779 // ac	Coronin-2B	COR2B_HUMAN|CORO2B|KIAA0925
0030036 // ac	0015629 // ac	0005515 // pr	Coronin-6	CORO6_HUMAN|CORO6|PP1009|PP1782|PP1881
0008150 // bi	0016021 // in	0003674 // m	Cytochrome c	COX16_HUMAN|COX16|C14orf112|HSPC203|PTD019
0006825 // cc	0005737 // cy	0005515 // pr	Cytochrome c	COX17_HUMAN|COX17
0008535 // re	0031305 // in	0008565 // pr	Mitochondria	COX18_HUMAN|COX18|OXA1L2
0009060 // ae	0005758 // m		Cytochrome c	COX19_HUMAN|COX19
	0030061 // m	0005515 // pr	Cytochrome c	CX6B2_HUMAN|COX6B2
0006123 // m	0005739 // m	0004129 // cy	Cytochrome c	COX6C_HUMAN|COX6C
1902600 // hy	0005746 // m	0004129 // cy	Cytochrome c	CX7A2_HUMAN|COX7A2|COX7AL
0006123 // m	0016021 // in	0005515 // pr	Cytochrome c	COX8A_HUMAN|COX8A|COX8|COX8L
0006825 // cc	0005576 // ex	0005507 // cc	Ceruloplasmir	CERU_HUMAN|CP
0006508 // pr	0005615 // ex	0004181 // m	Carboxypeptic	CBPA2_HUMAN|CPA2
0006508 // pr	0005615 // ex	0004181 // m	Carboxypeptic	CBPA4_HUMAN|CPA4|CPA3|UNQ694/PRO1339

0006508 // pr	0005615 // ex	0004181 // m	Carboxypeptid	CBPA6_HUMAN|CPA6|CPAH
0001654 // ey	0005886 // pl	0004867 // se	C3 and PZP-lik	CPMD8_HUMAN|CPAMD8|KIAA1283
0097421 // liv	0070062 // ex	0004181 // m	Carboxypeptid	CBPB2_HUMAN|CPB2
0006464 // ce	0043025 // ne	0004181 // m	Carboxypeptid	CBPE_HUMAN|CPE
0071243 // ce	0045202 // sy	0003723 // RN	Cytoplasmic p	CPEB2_HUMAN|CPEB2
0002931 // re	0045202 // sy	0003723 // RN	Cytoplasmic p	CPEB4_HUMAN|CPEB4|KIAA1673
	0005783 // er		Cadherin-like	CPED1_HUMAN|CPED1|C7orf58|UNQ9432/PRO34713
0007268 // ch	0005829 // cy	0005326 // ne	Complexin-1	CPLX1_HUMAN|CPLX1
0006518 // pe	0031225 // an	0004180 // ca	Carboxypeptid	CBPM_HUMAN|CPM
0071277 // ce	0070062 // ex	0005515 // pr	Copine-2	CPNE2_HUMAN|CPNE2
1903861 // po	0070062 // ex	0003674 // m	Copine-5	CPNE5_HUMAN|CPNE5|KIAA1599
0046474 // gl	0005737 // cy	0005515 // pr	Copine-7	CPNE7_HUMAN|CPNE7
0008150 // bi	0070062 // ex	0003674 // m	Copine-8	CPNE8_HUMAN|CPNE8
0006508 // pr	0005615 // ex	0004181 // m	Carboxypeptid	CBPO_HUMAN|CPO
0043171 // pe	0005794 // Go	0070573 // m	Carboxypeptid	CBPQ_HUMAN|CPQ|LCH1|PGCP
0050667 // ho	0005759 // m	0004087 // ca	Carbamoyl-ph	CPSM_HUMAN|CPS1
0031124 // m	0005654 // nu	0035925 // m	Cleavage and	CPSF1_HUMAN|CPSF1|CPSF160
0031124 // m	0005654 // nu	0003723 // RN	Cleavage and	CPSF7_HUMAN|CPSF7
0042493 // re	0005739 // m	0004095 // ca	Carnitine O-pa	CPT1A_HUMAN|CPT1A|CPT1
0009437 // ca	0005741 // m	0004095 // ca	Carnitine O-pa	CPT1C_HUMAN|CPT1C|CATL1
0019216 // re	0005743 // m	0004095 // ca	Carnitine O-pa	CPT2_HUMAN|CPT2|CPT1
1902389 // ce	0005829 // cy	0005548 // ph	Ceramide-1-p	CPTP_HUMAN|CPTP|GLTPD1
0006518 // pe	0005615 // ex	0008270 // zi	Inactive carbo	CPXM2_HUMAN|CPXM2|CPX2|UNQ676/PRO1310
0045959 // ne	0005886 // pl	0001851 // co	Complement	CR1_HUMAN|CR1|C3BR
	0016020 // m		Complement	CR1L_HUMAN|CR1L
0030449 // re	0043235 // re	0004875 // co	Complement	CR2_HUMAN|CR2|C3DR
0006355 // re	0005654 // nu	0005501 // re	Cellular retino	RABP2_HUMAN|CRABP2
0051928 // po	0005737 // cy	0005515 // pr	EF-hand calciu	EFC4B_HUMAN|CRACR2A|EFCAB4B
2001256 // re	0005737 // cy	0005509 // ca	EF-hand calciu	EFC4A_HUMAN|CRACR2B|EFCAB4A
0008625 // ex	0005829 // cy	0005515 // pr	Death domain	CRADD_HUMAN|CRADD|RAIDD
0033540 // fa	0005739 // m	0004092 // ca	Carnitine O-ac	CACP_HUMAN|CRAT|CAT1
0006611 // pr	0016021 // in	0005509 // ca	Protein crumb	CRUM1_HUMAN|CRB1
0001707 // m	0016021 // in	0019828 // as	Protein crumb	CRUM2_HUMAN|CRB2
0072659 // pr	0016021 // in	0017124 // SH	Protein crumb	CRUM3_HUMAN|CRB3|UNQ588/PRO1158
0016032 // vi	0005634 // nu	0000977 // RN	Cyclic AMP-re	CREB3_HUMAN|CREB3|LZIP
0051216 // ca	0016021 // in	0000978 // RN	Cyclic AMP-re	CR3L2_HUMAN|CREB3L2|BBF2H7
0045944 // po	0005739 // m	0035497 // cA	Cyclic AMP-re	CR3L4_HUMAN|CREB3L4|AIBZIP|CREB4|JAL
0045893 // po	0005634 // nu	0005515 // pr	Cyclic AMP-re	CREB5_HUMAN|CREB5|CREBPA
0002223 // sti	0005654 // nu	0005515 // pr	CREB-binding	CBP_HUMAN|CREBBP|CBP
0007165 // sig	0005634 // nu	0003677 // DN	cAMP-respons	CRBL2_HUMAN|CREBL2
0042711 // m	0016604 // nu	0000977 // RN	CREB3 regulat	CRERF_HUMAN|CREBRF|C5orf41
0006351 // tra	0005739 // m	0005515 // pr	CREB/ATF bZI	ZHANG_HUMAN|CREBZF|ZF
0006357 // re	0005667 // tra	0003714 // tra	Protein CREG1	CREG1_HUMAN|CREG1|CREG|UNQ727/PRO1409
	0005576 // ex	0048037 // co	Protein CREG2	CREG2_HUMAN|CREG2
0006355 // re	0005634 // nu	0003677 // DN	cAMP-respons	CREM_HUMAN|CREM

0007166 // ce	0005886 // pl	0015056 // co	Corticotropin-	CRFR2_HUMAN|CRHR2|CRF2R|CRH2R	
0007399 // ne	0016021 // in	0004867 // se	Cysteine-rich	CRIM1_HUMAN|CRIM1|S52|UNQ1886/PRO4330	
0030097 // he	0005938 // ce	0008270 // zin	Cysteine-rich	CRIP2_HUMAN|CRIP2|CRP2	
	0005737 // cy	0046872 // m	Cysteine-rich	CRIP3_HUMAN|CRIP3|CRP3	
0043627 // re	0005783 // er	0005515 // pr	Cysteine-rich	CRPAK_HUMAN|CRIPAK	
	0005615 // ex		Cysteine-rich s	CRIS2_HUMAN|CRISP2|GAPDL5|TPX1	
0045087 // in	0042581 // sp	0003674 // m	Cysteine-rich s	CRIS3_HUMAN|CRISP3	
0060325 // fa	0070062 // ex	0003674 // m	Cysteine-rich s	CRLD1_HUMAN|CRISPLD1|CRISP10|LCRISP1|UNQ342/P	
0030198 // ex	0030133 // tra	0008201 // he	Cysteine-rich s	CRLD2_HUMAN|CRISPLD2|CRISP11|LCRISP2|UNQ2914/	
0008284 // po	0005576 // ex	0004872 // re	Cytokine rece	CRLF1_HUMAN|CRLF1|UNQ288/PRO327	
0036148 // ph	0016021 // in	0043337 // CD	Cardiolipin syn	CRLS1_HUMAN|CRLS1|C20orf155|CLS1	
1904530 // ne	0005829 // cy	0005515 // pr	Dihydropyrim	DPYL1_HUMAN|CRMP1|DPYSL1|ULIP3	
0010457 // ce	0035253 // cil	0005515 // pr	Rootletin	CROCC_HUMAN|CROCC|KIAA0445	
0007098 // ce	0035253 // cil		Putative ciliar	CROL2_HUMAN|CROCCP3|CROCCL2|KIAA1922	
1900006 // po	0016604 // nu	0005515 // pr	CREB-regulate	CRTC1_HUMAN|CRTC1|KIAA0616|MECT1|TORC1|WAM	
0050896 // re	0005634 // nu	0005515 // pr	Cone-rod hom	CRX_HUMAN|CRX|CORD2	
0018298 // pr	0005634 // nu	0001046 // co	Cryptochrome	CRY1_HUMAN|CRY1|PHLL1	
0032355 // re	0005829 // cy	0005515 // pr	Alpha-crystall	CRYAB_HUMAN|CRYAB|CRYA2|HSPB5	
0043010 // ca		0042802 // id	Beta-crystallin	CRBB2_HUMAN|CRYBB2|CRYB2|CRYB2A	
0007601 // vis		0005515 // pr	Beta-crystallin	CRBB3_HUMAN|CRYBB3|CRYB3	
0002088 // le		0005212 // st	Beta-crystallin	CRBS_HUMAN|CRYGS|CRYG8	
0006810 // tra	0016021 // in	0005215 // tra	Putative unch	CRAS1_HUMAN|CRYM-AS1|NCRNA00169	
0042412 // ta		0004782 // su	Cysteine sulfin	CSAD_HUMAN|CSAD|CSD	
0042493 // re			Chondrosarco	CSAG2_HUMAN|CSAG2|CSAG3|CSAG3A|TRAG3	
0006355 // re	0005794 // Go	0003723 // RN	Cold shock do	CSDE1_HUMAN|CSDE1|D1S155E|KIAA0885|NRU|UNR	
0006611 // pr	0005635 // nu	0005515 // pr	Exportin-2	XPO2_HUMAN|CSE1L|CAS|XPO2	
1902228 // po	0016021 // in	0005125 // cy	Macrophage c	CSF1_HUMAN|CSF1	
2000147 // po	0043231 // in	0042803 // pr	Macrophage c	CSF1R_HUMAN|CSF1R|FMS	
0045740 // po	0005576 // ex	0008083 // gr	Granulocyte-m	CSF2_HUMAN|CSF2|GMCSF	
0007275 // m	0005576 // ex	0005125 // cy	Granulocyte c	CSF3_HUMAN|CSF3|C17orf33|GCSF	
0019276 // UI	0005622 // in	0008955 // pe	Chondroitin su	CGAT1_HUMAN|CSGALNACT1|CHGN|GALNACT1|UNQ6	
0050650 // ch	0030173 // in	0047237 // glu	Chondroitin su	CGAT2_HUMAN|CSGALNACT2|CHGN2|GALNACT2|PRO(	
	0016021 // in		CUB and sush	CSMD1_HUMAN|CSMD1|KIAA1890|UNQ5952/PRO1986	
0050773 // re	0016021 // in		CUB and sush	CSMD3_HUMAN|CSMD3|KIAA1894	
0006468 // pr	0005654 // nu	0004674 // pr	Casein kinase	CSK23_HUMAN|CSNK2A3|CSNK2A1P	
0016055 // W	0016363 // nu	0005515 // pr	Casein kinase	CSK2B_HUMAN|CSNK2B|CK2N|G5A	
0008283 // ce	0005796 // Go	0019901 // pr	Chondroitin su	CSPG4_HUMAN|CSPG4|MCSP	
0050804 // m	0005796 // Go	0008083 // gr	Chondroitin su	CSPG5_HUMAN|CSPG5|CALEB|NGC	
0009791 // po	0005634 // nu	0005515 // pr	Cysteine/serin	CSRN1_HUMAN|CSRNP1|AXUD1|TAIP3	
0045944 // po	0005634 // nu	0001228 // tra	Cysteine/serin	CSRN3_HUMAN|CSRNP3|FAM130A2|TAIP2	
0070527 // pl	0005634 // nu	0003723 // RN	Cysteine and g	CSRP1_HUMAN|CSRP1|CSRP|CYRP	
0010711 // ne	0005604 // ba	0004869 // cy	Cystatin-C	CYTC_HUMAN|CST3	

0008544 // ep	0001533 // co	0004869 // cy	Cystatin-M	CYTM_HUMAN|CST6
0010951 // ne	0005829 // cy	0002020 // pr	Cystatin-A	CYTA_HUMAN|CSTA|STF1|STFA
0031124 // m	0005654 // nu	0003723 // RN	Cleavage stim	CSTF1_HUMAN|CSTF1
0031124 // m	0005654 // nu	0003723 // RN	Cleavage stim	CSTFT_HUMAN|CSTF2T|KIAA0689
2000117 // ne	0005615 // ex	0004869 // cy	Cystatin-like 1	CSTL1_HUMAN|CSTL1
	0016021 // in		Kita-kyushu lu	KKLC1_HUMAN|CT83|CXorf61|KKLC1
	0016021 // in		cTAGE family	CTGEF_HUMAN|CTAGE15|CTAGE15P
0008150 // bi	0016021 // in		cTAGE family	CTGE4_HUMAN|CTAGE4

0048861 // le	0005576 // ex	0005125 // cy	Cardiotrophin	CTF1_HUMAN|CTF1

0043200 // re	0005794 // Go	0008022 // pr	Connective tis	CTGF_HUMAN|CTGF|CCN2|HCS24|IGFBP8
0090090 // ne	0005615 // ex	0017147 // W	Collagen triple	CTHR1_HUMAN|CTHRC1|UNQ762/PRO1550
0000184 // nu	0048471 // pe	0005515 // pr	CBP80/20-dep	CTIF_HUMAN|CTIF|KIAA0427
0098911 // re	0005737 // cy	0008013 // be	Catenin alpha	CTNA3_HUMAN|CTNNA3
0007266 // Rh	0005886 // pl	0005515 // pr	Alpha-catulin	CTNL1_HUMAN|CTNNAL1
0045944 // po	0005829 // cy	0008134 // tra	Catenin beta-1	CTNB1_HUMAN|CTNNB1|CTNNB|OK/SW-cl.35|PRO228(
0009952 // ar	0005634 // nu	0005515 // pr	Beta-catenin-i	CNBP1_HUMAN|CTNNBIP1|ICAT
0016445 // so	0005654 // nu	0005515 // pr	Beta-catenin-l	CTBL1_HUMAN|CTNNBL1|C20orf33|PP8304
0007155 // ce	0043197 // de	0005515 // pr	Catenin delta-	CTND1_HUMAN|CTNND1|KIAA0384
0006241 // CT	0005829 // cy	0003883 // CT	CTP synthase	PYRG1_HUMAN|CTPS1|CTPS
0001711 // en	0005654 // nu	0005515 // pr	RNA polymera	CTR9_HUMAN|CTR9|KIAA0155|SH2BP1
0006508 // pr	0005615 // ex	0004252 // se	Chymotrypsin	CTRB2_HUMAN|CTRB2
0006508 // pr	0005576 // ex	0004252 // se	Chymotrypsin	CTRC_HUMAN|CTRC|CLCR
0007586 // di	0005615 // ex	0004252 // se	Chymotrypsin	CTRL_HUMAN|CTRL|CTRL1
0006687 // gly	0043202 // lys	0008047 // en	Lysosomal pro	PPGB_HUMAN|CTSA|PPGB
0030574 // co	0005576 // ex	0004197 // cy	Cathepsin D	CATD_HUMAN|CTSD|CPSD
0051603 // pr	0005615 // ex	0004197 // cy	Cathepsin F	CATF_HUMAN|CTSF
0002250 // ad	0034774 // se	0004197 // cy	Pro-cathepsin	CATH_HUMAN|CTSH|CPSB
0030574 // co	0043202 // lys	0001968 // fib	Cathepsin K	CATK_HUMAN|CTSK|CTSO|CTSO2
0002250 // ad	0005576 // ex	0004197 // cy	Cathepsin L1	CATL1_HUMAN|CTSL|CTSL1
0051603 // pr	0005615 // ex	0004197 // cy	Cathepsin O	CATO_HUMAN|CTSO|CTSO1
0002250 // ad	0005576 // ex	0001968 // fib	Cathepsin S	CATS_HUMAN|CTSS
0046697 // de	0005615 // ex	0004197 // cy	Cathepsin L2	CATL2_HUMAN|CTSV|CATL2|CTSL2|CTSU|UNQ268/PRC
0032091 // ne	0005764 // lys	0005515 // pr	Cathepsin Z	CATZ_HUMAN|CTSZ
0030838 // po	0005794 // Go	0005515 // pr	Src substrate c	SRC8_HUMAN|CTTN|EMS1
0007420 // br	0005938 // ce	0017124 // SH	Cortactin-bind	CTTB2_HUMAN|CTTNBP2|C7orf8|CORTBP2|KIAA1758
0032410 // ne	0005737 // cy	0005515 // pr	CTTNBP2 N-te	CT2NL_HUMAN|CTTNBP2NL|KIAA1433
	0016021 // in		Cortexin-1	CTXN1_HUMAN|CTXN1|CTXN
0042953 // lip	0031526 // br	0005515 // pr	Cubilin	CUBN_HUMAN|CUBN|IFCR
0016032 // vir	0005829 // cy	0005515 // pr	Cullin-2	CUL2_HUMAN|CUL2
0010498 // pr	0005654 // nu	0005515 // pr	Cullin-4B	CUL4B_HUMAN|CUL4B|KIAA0695
0007088 // re	0005737 // cy	0005515 // pr	Cullin-7	CUL7_HUMAN|CUL7|KIAA0076
0007088 // re	0005829 // cy	0005515 // pr	Cullin-9	CUL9_HUMAN|CUL9|H7AP1|KIAA0708|PARC
0006825 // co	0005730 // nu	0005515 // pr	Copper home	CUTC_HUMAN|CUTC|CGI-32
0006931 // su	0042589 // zy		CUB and zona	CUZD1_HUMAN|CUZD1|UNQ224/PRO257
0008150 // bi	0005575 // ce	0003674 // m	CWF19-like pr	C19L1_HUMAN|CWF19L1
0010669 // ep	0005886 // pl	0071253 // co	Coxsackievirus	CXAR_HUMAN|CXADR|CAR
0032496 // re	0005576 // ex	0008083 // gr	Growth-regula	GROA_HUMAN|CXCL1|GRO|GRO1|GROA|MGSA|SCYB1
0050965 // de	0005576 // ex	0008083 // gr	Stromal cell-d	SDF1_HUMAN|CXCL12|SDF1|SDF1A|SDF1B
0007186 // G-	0005576 // ex	0008201 // he	C-X-C motif ch	CXL13_HUMAN|CXCL13|BCA1|BLC|SCYB13
0010818 // T c	0005576 // ex	0005041 // lo	C-X-C motif ch	CXL16_HUMAN|CXCL16|SCYB16|SRPSOX|UNQ2759/PR(
0032496 // re	0005576 // ex	0005515 // pr	C-X-C motif ch	CXCL2_HUMAN|CXCL2|GRO2|GROB|MIP2A|SCYB2
0032496 // re	0005576 // ex	0045236 // CX	C-X-C motif ch	CXCL3_HUMAN|CXCL3|GRO3|GROG|SCYB3
0032496 // re	0005576 // ex	0042802 // id	C-X-C motif ch	CXCL5_HUMAN|CXCL5|ENA78|SCYB5
0007186 // G-	0005576 // ex	0045236 // CX	C-X-C motif ch	CXCL6_HUMAN|CXCL6|GCP2|SCYB6
0045766 // po	0005576 // ex	0005515 // pr	Interleukin-8	IL8_HUMAN|CXCL8|IL8

0006935 // ch	0005886 // pl	0005515 // pr	C-X-C chemok	CXCR2_HUMAN|CXCR2|IL8RB
0042113 // B	0005886 // pl	0005515 // pr	C-X-C chemok	CXCR5_HUMAN|CXCR5|BLR1|MDR15
	0005739 // m		Uncharacteriz	CX023_HUMAN|CXorf23
		0005515 // pr	Protein CXorf4	CX04A_HUMAN|CXorf40A|CXorf40|EOLA1
		0003723 // RN	Uncharacteriz	CX057_HUMAN|CXorf57
0045893 // po	0016607 // nu	0000987 // co	CXXC-type zin	CXXC1_HUMAN|CXXC1|CFP1|CGBP|PCCX1|PHF18
0016055 // W	0031410 // cy	0030165 // PD	CXXC-type zin	CXXC4_HUMAN|CXXC4|IDAX
0007165 // sig	0005829 // cy	0004871 // sig	CXXC-type zin	CXXC5_HUMAN|CXXC5|HSPC195|TCCCIA00297
0055085 // tra	0005765 // lys	0005515 // pr	Cytochrome b	CY561_HUMAN|CYB561
0055114 // ox	0016021 // int	0005515 // pr	Cytochrome b	CYAC3_HUMAN|CYB561A3|CYBASC3|LCYTB|PSEC0259
0055114 // ox	0016021 // int	0004129 // cy	Cytochrome b	CYB5_HUMAN|CYB5A|CYB5
		0046872 // m	Cytochrome b	CB5D1_HUMAN|CYB5D1
0045666 // po	0012505 // en	0020037 // he	Neuferricin	NEUFC_HUMAN|CYB5D2
0002576 // pl	0005739 // m	0005515 // pr	NADH-cytochr	NB5R1_HUMAN|CYB5R1|NQO3A2|UNQ3049/PRO9865
0055114 // ox	0005634 // nu	0005515 // pr	NADH-cytochr	NB5R2_HUMAN|CYB5R2
0071333 // ce	0043025 // ne	0046982 // pr	Cytochrome b	CY24A_HUMAN|CYBA
0097202 // ac	0005829 // cy	0005515 // pr	Cytoplasmic F	CYFP2_HUMAN|CYFIP2|KIAA1168|PIR121
0015671 // ox	0005829 // cy	0019825 // ox	Cytoglobin	CYGB_HUMAN|CYGB|STAP
	0048471 // pe	0005515 // pr	Cysteine and I	CYHR1_HUMAN|CYHR1|KIAA0496
0006700 // C2	0005759 // m	0008386 // ch	Cholesterol si	CP11A_HUMAN|CYP11A1|CYP11A
0006702 // ar	0043025 // ne	0004508 // ste	Steroid 17-alp	CP17A_HUMAN|CYP17A1|CYP17|S17AH
0006694 // ste	0016020 // m	0070330 // ar	Aromatase	CP19A_HUMAN|CYP19A1|ARO1|CYAR|CYP19
0007568 // ag	0005789 // en	0004497 // m	Cytochrome P	CP1A1_HUMAN|CYP1A1
0030199 // co	0005739 // m	0004497 // m	Cytochrome P	CP1B1_HUMAN|CYP1B1
0055114 // ox	0016021 // int	0004497 // m	Cytochrome P	CP20A_HUMAN|CYP20A1|UNQ667/PRO1301
0006766 // vit	0005739 // m	0008403 // 25	1,25-dihydrox	CP24A_HUMAN|CYP24A1|CYP24
0048387 // ne	0005789 // en	0016709 // ox	Cytochrome P	CP26A_HUMAN|CYP26A1|CYP26|P450RAI1
0048387 // ne	0005789 // en	0016709 // ox	Cytochrome P	CP26B_HUMAN|CYP26B1|CYP26A2|P450RAI2
0042573 // re	0016020 // m	0005503 // all	Cytochrome P	C27C1_HUMAN|CYP27C1
0006805 // xe	0031090 // or	0004497 // m	Cytochrome P	CP2B6_HUMAN|CYP2B6
0042738 // ex	0005789 // en	0004497 // m	Cytochrome P	CP2C8_HUMAN|CYP2C8
0046483 // he	0031090 // or	0004497 // m	Cytochrome P	CP2D6_HUMAN|CYP2D6|CYP2DL1
0006690 // ico	0005789 // en	0005506 // iro	Cytochrome P	CP2J2_HUMAN|CYP2J2
0019373 // ep	0005789 // en	0008401 // re	Cytochrome P	CP2S1_HUMAN|CYP2S1|UNQ891/PRO1906
0097267 // or	0016021 // int	0004497 // m	Cytochrome P	CP2U1_HUMAN|CYP2U1

0043390 // af	0005789 // er	0008392 // ar	Cytochrome P	CP2W1_HUMAN|CYP2W1
0006699 // bi	0043231 // in	0020037 // he	24-hydroxych	CP39A_HUMAN|CYP39A1
0006631 // fa	0016021 // in	0016709 // ox	Phylloquinone	CP4FB_HUMAN|CYP4F11
0001676 // lo	0016021 // in	0019825 // ox	Cytochrome P	CP4FC_HUMAN|CYP4F12|UNQ568/PRO1129
0008217 // re	0005789 // er	0018685 // al	Phylloquinone	CP4F2_HUMAN|CYP4F2
0036101 // le	0016021 // in	0019825 // ox	Docosahexaer	CP4F3_HUMAN|CYP4F3|LTB4H
0050896 // re	0016021 // in	0016705 // ox	Cytochrome P	CP4V2_HUMAN|CYP4V2
0055114 // ox	0016021 // in	0005506 // irc	Cytochrome P	CP4X1_HUMAN|CYP4X1|UNQ1929/PRO4404
0055114 // ox	0016021 // in	0005506 // irc	Cytochrome P	CP4Z1_HUMAN|CYP4Z1|UNQ3060/PRO9882
0055114 // ox	0016021 // in	0016705 // ox	Putative inact	CP4Z2_HUMAN|CYP4Z2P
0060591 // ch	0031012 // ex	0050840 // ex	Protein CYR61	CYR61_HUMAN|CYR61|CCN1|GIG1|IGFBP10
	0070062 // ex		Cysteine-rich	CRTP1_HUMAN|CYSRT1|C9orf169
0008150 // bi	0016021 // in	0003674 // m	Cysteine-rich	CYTM1_HUMAN|CYSTM1|C5orf32|ORF1-FL49
0032012 // re	0000139 // G	0005515 // pr	Cytohesin-1	CYH1_HUMAN|CYTH1|D17S811E|PSCD1
0008150 // bi	0016021 // in	0003674 // m	Cysteine and	CYYR1_HUMAN|CYYR1|C21orf95
0006103 // 2-	0005739 // m	0004458 // D-	D-2-hydroxygl	D2HDH_HUMAN|D2HGDH|D2HGD
0030036 // ac	0036064 // cil	0005515 // pr	Disheveled-as	DAAM1_HUMAN|DAAM1|KIAA0666
0042493 // re	0005829 // cy	0005515 // pr	Disabled hom	DAB1_HUMAN|DAB1
2000643 // pc	0005925 // fo	0005515 // pr	Disabled hom	DAB2_HUMAN|DAB2|DOC2
0000122 // ne	0043025 // ne	0043184 // va	Disabled hom	DAB2P_HUMAN|DAB2IP|AF9Q34|AIP1|KIAA1743
0048147 // ne	0005737 // cy	0001075 // tr	Dachshund hc	DACH1_HUMAN|DACH1|DACH
0007275 // m	0005667 // tr	0001075 // tr	Dachshund hc	DACH2_HUMAN|DACH2
0048619 // er	0005737 // cy	0001085 // RI	Dapper homo	DACT1_HUMAN|DACT1|DPR1|HNG3
0035414 // ne	0005737 // cy	0005080 // pr	Dapper homo	DACT3_HUMAN|DACT3|RRR1
0042493 // re	0008250 // ol	0004576 // ol	Dolichyl-diphc	DAD1_HUMAN|DAD1
0021682 // ne	0005925 // fo	0002162 // dy	Dystroglycan	DAG1_HUMAN|DAG1
0007405 // ne	0043196 // va	0047372 // ac	Sn1-specific d	DGLA_HUMAN|DAGLA|C11orf11|KIAA0659|NSDDR
0042136 // ne	0016021 // in	0047372 // ac	Sn1-specific d	DGLB_HUMAN|DAGLB
0070178 // D-	0005777 // pe	0003884 // D-	D-amino-acid	OXDA_HUMAN|DAO|DAMOX
0006919 // ac		0070513 // de	Death-associa	DAP1_HUMAN|DAP|DAP1
0046777 // pr	0005634 // nu	0005515 // pr	Death-associa	DAPK3_HUMAN|DAPK3|ZIPK
0007165 // sig	0005737 // cy	0005547 // ph	Dual adapter	DAPP1_HUMAN|DAPP1|BAM32|HSPC066
0070145 // m	0005739 // m	0050560 // as	Aspartate--tRI	SYDM_HUMAN|DARS2
0007507 // he	0005737 // cy		Dynein assem	DAW1_HUMAN|DAW1|WDR69
0006260 // DI	0005654 // nu	0003676 // nu	Protein DBF4	DBF4A_HUMAN|DBF4|ASK|DBF4A|ZDBF1
0050773 // re	0015629 // ac	0005515 // pr	Drebrin	DREB_HUMAN|DBN1|D0S117E
0031175 // ne	0030672 // sy		Dysbindin dor	DBND1_HUMAN|DBNDD1
0031175 // ne	0030672 // sy	0005515 // pr	Dysbindin dor	DBND2_HUMAN|DBNDD2|C20orf35
0045944 // pc	0005634 // nu	0001077 // tr	D site-binding	DBP_HUMAN|DBP

0016567 // pr	0080008 // Cu	0005515 // pr	DDB1- and CU	DCA12_HUMAN|DCAF12|KIAA1892|TCC52|WDR40A
0008380 // RN	0071013 // ca	0003723 // RN	DDB1- and CU	DCAF4_HUMAN|DCAF4|WDR21|WDR21A
0045944 // po	0005654 // nu	0030374 // lig	DDB1- and CU	DCAF6_HUMAN|DCAF6|IQWD1|MSTP055
0016310 // ph	0016020 // m	0004140 // de	Dephospho-Co	DCAKD_HUMAN|DCAKD
	0016021 // in	0070492 // ol	Discoidin, CUB	DCBD1_HUMAN|DCBLD1
0030308 // ne	0005887 // in	0005515 // pr	Discoidin, CUB	DCBD2_HUMAN|DCBLD2|CLCP1|ESDN
0035556 // in	0005622 // in		Doublecortin	DCDC1_HUMAN|DCDC1
1902017 // re	0005929 // cil	0005515 // pr	Doublecortin	DCDC2_HUMAN|DCDC2|KIAA1154|RU2
0022008 // ne	0005622 // in	0005509 // ca	Protocadherin	PCD16_HUMAN|DCHS1|CDH19|CDH25|FIB1|KIAA1773|
1900181 // ne	0005887 // in	0004672 // pr	Serine/threon	DCLK1_HUMAN|DCLK1|DCAMKL1|DCDC3A|KIAA0369
0018107 // pe	0005737 // cy	0005524 // AT	Serine/threon	DCLK2_HUMAN|DCLK2|DCAMKL2|DCDC3B|DCK2
0031860 // te	0000784 // nu	0005515 // pr	5' exonucleas	DCR1B_HUMAN|DCLRE1B|SNM1B
0043928 // ex	0005829 // cy	0003729 // m	mRNA-decapp	DCP1B_HUMAN|DCP1B
0043928 // ex	0005829 // cy	0005515 // pr	m7GpppN-mR	DCP2_HUMAN|DCP2|NUDT20
0060339 // ne	0005886 // pl	0005515 // pr	DC-STAMP do	DCST1_HUMAN|DCST1
	0016021 // in		DC-STAMP do	DCST2_HUMAN|DCST2
0008544 // ep	0005829 // cy	0005507 // co	L-dopachrome	TYRP2_HUMAN|DCT|TYRP2
0006220 // py	0005829 // cy	0005515 // pr	Deoxycytidyla	DCTD_HUMAN|DCTD
0000086 // G2	0005829 // cy	0005515 // pr	Dynactin subu	DCTN3_HUMAN|DCTN3|DCTN22
0006888 // ER	0005634 // nu	0005515 // pr	Dynactin subu	DCTN4_HUMAN|DCTN4
0006888 // ER	0005829 // cy	0005515 // pr	Dynactin subu	DCTN5_HUMAN|DCTN5
0006888 // ER	0005829 // cy	0005515 // pr	Dynactin subu	DCTN6_HUMAN|DCTN6|WS3
0042262 // DN	0005829 // cy	0005515 // pr	dCTP pyropho	DCTP1_HUMAN|DCTPP1|XTP3TPA|CDA03
0051443 // po	0000151 // ub	0032182 // ub	DCN1-like pro	DCNL4_HUMAN|DCUN1D4|KIAA0276
0005997 // xy	0005903 // br	0042802 // id	L-xylulose red	DCXR_HUMAN|DCXR|SDR20C1
0045429 // po	0005739 // m	0016403 // di	N(G),N(G)-dim	DDAH1_HUMAN|DDAH1|DDAH
0006809 // ni	0070062 // ex	0016403 // di	N(G),N(G)-dim	DDAH2_HUMAN|DDAH2|DDAH|G6A|NG30
0010498 // pr	0005654 // nu	0005515 // pr	DNA damage-	DDB1_HUMAN|DDB1|XAP1
0090141 // po	0005829 // cy	0046872 // m	Phospholipase	DDHD1_HUMAN|DDHD1|KIAA1705
0007626 // lo	0005793 // en	0046872 // m	Phospholipase	DDHD2_HUMAN|DDHD2|KIAA0725|SAMWD1
0030182 // ne	0005829 // cy	0071889 // 14	DNA damage-	DDIT4_HUMAN|DDIT4|REDD1|RTP801
0045944 // po	0032591 // de	0005515 // pr	Dendrin	DEND_HUMAN|DDN|KIAA0749
0019478 // D-	0005777 // pe	0005102 // re	D-aspartate o	OXDD_HUMAN|DDO
0038063 // co	0043235 // re	0005515 // pr	Epithelial disc	DDR1_HUMAN|DDR1|CAK|EDDR1|NEP|NTRK4|PTK3A|
0035988 // ch	0016324 // ap	0005515 // pr	Discoidin dom	DDR2_HUMAN|DDR2|NTRKR3|TKT|TYRO10
0008284 // po	0005783 // en	0005515 // pr	DDRGK domai	DDRGK_HUMAN|DDRGK1|C20orf116|UFBP1
0050729 // po	0070062 // ex	0004167 // do	D-dopachrom	DOPD_HUMAN|DDT
	0070062 // ex	0016829 // ly	D-dopachrom	DDTL_HUMAN|DDTL
0006388 // tR	0005634 // nu	0003723 // RN	ATP-depende	DDX1_HUMAN|DDX1
0010501 // RN	0005730 // nu	0003723 // RN	Probable ATP-	DDX10_HUMAN|DDX10
0032091 // ne	0031390 // Ct	0005515 // pr	ATP-depende	DDX11_HUMAN|DDX11|CHL1|CHLR1|KRG2

0032508 // DI	0005634 // nu	0003677 // DI	Putative ATP-(	DDX12_HUMAN|DDX12P|CHLR2|DDX12
0071392 // ce	0005694 // ch	0003723 // RI	ATP-depender	DDX18_HUMAN|DDX18|cPERP-D
0048477 // oo	0005654 // nu	0005515 // pr	Probable ATP-	DDX20_HUMAN|DDX20|DP103|GEMIN3
0006366 // tra	0005730 // nu	0003723 // RI	Nucleolar RNA	DDX21_HUMAN|DDX21
0016070 // RI	0005730 // nu	0003723 // RI	ATP-depender	DDX24_HUMAN|DDX24
0006364 // rR	0005694 // ch	0003723 // RI	Probable ATP-	DDX27_HUMAN|DDX27|RHLP|cPERP-F|HSPC259|PP324
0031124 // m	0005681 // sp	0003723 // RI	ATP-depender	DX39A_HUMAN|DDX39A|DDX39
0000398 // m	0005730 // nu	0003723 // RI	Probable ATP-	DDX41_HUMAN|DDX41|ABS
0000398 // m	0016020 // m	0003723 // RI	Probable ATP-	DDX46_HUMAN|DDX46|KIAA0801
0008625 // ex	0005654 // nu	0003723 // RI	Probable ATP-	DDX47_HUMAN|DDX47
0006364 // rR	0005654 // nu	0003723 // RI	Probable ATP-	DDX49_HUMAN|DDX49
0010501 // RI	0005730 // nu	0003723 // RI	ATP-depender	DDX50_HUMAN|DDX50
0006364 // rR	0005730 // nu	0003723 // RI	ATP-depender	DDX51_HUMAN|DDX51
0010501 // RI	0005730 // nu	0003723 // RI	ATP-depender	DDX55_HUMAN|DDX55|KIAA1595
0039529 // RI	0005829 // cy	0005515 // pr	Probable ATP-	DDX58_HUMAN|DDX58
0010501 // RI	0005730 // nu	0004004 // AT	Probable ATP-	DDX59_HUMAN|DDX59|ZNHIT5
0007283 // sp	0010494 // cy	0005515 // pr	Probable ATP-	DDX6_HUMAN|DDX6|HLR2|RCK
1900245 // po	0005829 // cy	0005515 // pr	Probable ATP-	DDX60_HUMAN|DDX60
		0004386 // he	Probable ATP-	DDX6L_HUMAN|DDX60L
0045892 // ne	0005576 // ex	0005515 // pr	Deformed epi	DEAF1_HUMAN|DEAF1|SPN|ZMYND5
0008285 // ne			Deleted in esc	DEC1_HUMAN|DEC1|CTS9
0051289 // pr	0005739 // m	0008670 // 2,4	2,4-dienoyl-Co	DECR_HUMAN|DECR1|DECR|SDR18C1
0006636 // ur	0005778 // pe	0005102 // re	Peroxisomal 2	DECR2_HUMAN|DECR2|PDCR|SDR17C1
0007283 // sp	0005730 // nu	0005515 // pr	Death effecto	DEDD_HUMAN|DEDD|DEDPRO1|DEFT|KE05
0045087 // in	0005576 // ex		Beta-defensin	DB133_HUMAN|DEFB133
0043547 // po	0005829 // cy	0017112 // Ra	DENN domain	DEN1B_HUMAN|DENND1B|C1orf218|FAM31B
0043547 // po	0005829 // cy	0017112 // Ra	DENN domain	DEN1C_HUMAN|DENND1C|FAM31C
0043547 // po	0015629 // ac	0017112 // Ra	DENN domain	DEN2A_HUMAN|DENND2A|KIAA1277
0043547 // po		0017112 // Ra	DENN domain	DEN2C_HUMAN|DENND2C
0043547 // po	0005829 // cy	0005515 // pr	DENN domain	DEN2D_HUMAN|DENND2D
0043547 // po	0005886 // pl	0017112 // Ra	DENN domain	DEN4C_HUMAN|DENND4C|C9orf55|C9orf55B
0010977 // ne	0005829 // cy	0005262 // ca	DENN domain	DEN5A_HUMAN|DENND5A|KIAA1091|RAB6IP1
0043547 // po	0005829 // cy	0005262 // ca	DENN domain	DEN5B_HUMAN|DENND5B
2000049 // po	0005829 // cy	0017112 // Ra	Protein DENN	DEN6A_HUMAN|DENND6A|FAM116A
0043547 // po	0005829 // cy	0017112 // Ra	Protein DENN	DEN6B_HUMAN|DENND6B|FAM116B
0032790 // rib	0005840 // rib	0005515 // pr	Density-regula	DENR_HUMAN|DENR|DRP1|H14
0043547 // po	0005829 // cy	0005096 // GT	DEP domain-c	DEPD7_HUMAN|DEPDC7
0045184 // es	0016021 // int	0005515 // pr	Derlin-1	DERL1_HUMAN|DERL1|DER1|UNQ243/PRO276
1904153 // ne	0030176 // int	0004252 // se	Derlin-3	DERL3_HUMAN|DERL3|C22orf14|DER3|LLN2
0016567 // pr	0005634 // nu	0005515 // pr	DET1 homolog	DET1_HUMAN|DET1
0006309 // ap	0005829 // cy	0097718 // dis	DNA fragment	DFFB_HUMAN|DFFB|CAD|DFF2|DFF40
0007605 // se	0005829 // cy		Non-syndrom	DFNA5_HUMAN|DFNA5|ICERE1
0007605 // se	0043025 // ne		Pejvakin	PJVK_HUMAN|DFNB59|PJVK
0046339 // di	0016021 // int	0004144 // di	Diacylglycerol	DGAT1_HUMAN|DGAT1|AGRP1|DGAT

0097006 // re	0016021 // in	0050252 // re	Diacylglycerol	DGAT2_HUMAN|DGAT2|HMFN1045|UNQ738/PRO1433
0000398 // m	0071013 // ca	0003674 // m	Protein DGCR	DGC14_HUMAN|DGCR14|DGCR13|DGSH|DGSI|ES2
0007155 // ce	0005578 // pr	0003674 // m	Protein DGCR	DGCR6_HUMAN|DGCR6
	0005634 // nu	0005515 // pr	Protein DGCR	DGC6L_HUMAN|DGCR6L
0046486 // gl	0005829 // cy	0004143 // di	Diacylglycerol	DGKA_HUMAN|DGKA|DAGK|DAGK1
0046486 // gl	0005737 // cy	0004143 // di	Diacylglycerol	DGKG_HUMAN|DGKG|DAGK3
0016310 // ph	0005737 // cy	0004143 // di	Diacylglycerol	DGKH_HUMAN|DGKH
0042127 // re	0005789 // er	0050661 // NA	7-dehydrocho	DHCR7_HUMAN|DHCR7|D7SR
0005975 // ca		0009055 // el	Trans-1,2-dihy	DHDH_HUMAN|DHDH|2DD
0006730 // or	0005739 // m	0003729 // m	Dihydrofolate	DYR2_HUMAN|DHFR2|DHFRL1|DHFRP4
0055114 // ox		0016491 // ox	Dehydrogenas	DHR12_HUMAN|DHRS12|SDR40C1
0008207 // C2	0005739 // m	0004090 // ca	Dehydrogenas	DHRS2_HUMAN|DHRS2|SDR25C1
0042572 // re	0016021 // in	0004745 // re	Short-chain de	DHRS3_HUMAN|DHRS3|RDH17|SDR16C1|UNQ2424/PR
0055114 // ox	0005778 // pe	0000253 // 3-	Dehydrogenas	DHRS4_HUMAN|DHRS4|SDR25C2|UNQ851/PRO1800
0055114 // ox	0005576 // ex	0016491 // ox	Dehydrogenas	DR4L2_HUMAN|DHRS4L2|SDR25C3
0008150 // bi	0016021 // in	0003674 // m	Dehydrogenas	DRS7B_HUMAN|DHRS7B|SDR32C1|CGI-93|UNQ212/PR
0030855 // ep	0030176 // in	0004745 // re	Dehydrogenas	DHRS9_HUMAN|DHRS9|RDH15|SDR9C4|UNQ835/PRO1
0006091 // ge	0005739 // m	0030976 // th	Probable 2-ox	DHTK1_HUMAN|DHTKD1|KIAA1630
0006396 // RN	0016282 // eu	0003723 // RN	ATP-depender	DHX29_HUMAN|DHX29|DDX29
0000398 // m	0005681 // sp	0005515 // pr	Putative pre-r	DHX32_HUMAN|DHX32|DDX32
0006396 // RN	0005730 // nu	0003723 // RN	Putative ATP-	DHX33_HUMAN|DHX33|DDX33
0006364 // rR	0005654 // nu	0005524 // AT	Probable ATP-	DHX37_HUMAN|DHX37|DDX37|KIAA1517
0039534 // ne	0005737 // cy	0005515 // pr	Probable ATP-	DHX58_HUMAN|DHX58|D11LGP2E|LGP2
0031167 // rR	0005654 // nu	0003723 // RN	Probable dime	DIM1_HUMAN|DIMT1|DIMT1L|HUSSY-05
0006590 // th	0016021 // in	0008430 // se	Type I iodothy	IOD1_HUMAN|DIO1|ITDI1|TXDI1
0001514 // se	0016021 // in	0031625 // ub	Type II iodoth	IOD2_HUMAN|DIO2|ITDI2|TXDI2
0008152 // m	0005634 // nu	0005515 // pr	Disco-interact	DIP2A_HUMAN|DIP2A|C21orf106|DIP2|KIAA0184
0000079 // re	0005622 // in	0003924 // G	GTP-binding p	DIRA3_HUMAN|DIRAS3|ARHI|NOEY2|RHOI
0007224 // sn	0016021 // in	0003674 // m	Protein dispat	DISP2_HUMAN|DISP2|C15orf36|DISPB|KIAA1742|LINC0
0007049 // ce	0005829 // cy	0043015 // ga	Dixin	DIXC1_HUMAN|DIXDC1|CCD1|KIAA1735
0090669 // te	0005654 // nu	0003723 // RN	H/ACA ribonu	DKC1_HUMAN|DKC1|NOLA4
0048642 // ne	0005576 // ex	0005515 // pr	Dickkopf-relat	DKK1_HUMAN|DKK1|UNQ492/PRO1008
0016055 // W	0005615 // ex		Dickkopf-relat	DKK3_HUMAN|DKK3|REIC|UNQ258/PRO295
0010510 // re	0005739 // m	0004742 // di	Dihydrolipoyll	ODP2_HUMAN|DLAT|DLTA
0030336 // ne	0005737 // cy	0042169 // SH	Rho GTPase-a	RHG07_HUMAN|DLC1|ARHGAP7|KIAA1723|STARD12
0008285 // ne	0005829 // cy	0003674 // m	Deleted in lun	DLEC1_HUMAN|DLEC1|DLC1
		0005515 // pr	Leukemia-ass	LEU1_HUMAN|DLEU1|LEU1|XTP6
0007093 // m	0005634 // nu	0005515 // pr	Disks large ho	DLG1_HUMAN|DLG1

0006461 // pr	0005886 // pl	0005515 // pr	Disks large-as	DLGP4_HUMAN|DLGAP4|DAP4|KIAA0964|SAPAP4
0008283 // ce	0031616 // sp	0005515 // pr	Disks large-as	DLGP5_HUMAN|DLGAP5|DLG7|KIAA0008
0045598 // re	0016021 // in	0005509 // ca	Protein delta	DLK2_HUMAN|DLK2|EGFL9|UNQ2903/PRO28633
0097150 // ne	0005886 // pl	0005515 // pr	Delta-like pro	DLL1_HUMAN|DLL1|UNQ146/PRO172
0001756 // so	0016021 // in	0005112 // N	Delta-like pro	DLL3_HUMAN|DLL3
0034641 // ce	0045252 // ox	0004149 // di	Dihydrolipoyll	ODO2_HUMAN|DLST|DLTS
0045746 // ne	0005634 // nu	0003682 // ch	Homeobox pr	DLX1_HUMAN|DLX1
0042475 // oc	0005634 // nu	0001077 // tr	Homeobox pr	DLX3_HUMAN|DLX3
0006355 // re	0005634 // nu	0003700 // tr	Homeobox pr	DLX4_HUMAN|DLX4|BP1|DLX7|DLX8|DLX9
0008283 // ce	0000790 // nu	0044212 // tr	Homeobox pr	DLX5_HUMAN|DLX5
0001501 // sk	0005634 // nu	0003700 // tr	Homeobox pr	DLX6_HUMAN|DLX6
0030855 // ep	0005576 // ex	0005044 // sc	Deleted in ma	DMBT1_HUMAN|DMBT1|GP340
0008343 // ac	0005667 // tr	0003700 // tr	Diencephalon	DMBX1_HUMAN|DMBX1|MBX|OTX3|PAXB
0001541 // ov	0000781 // ch	0003690 // do	Meiotic recon	DMC1_HUMAN|DMC1|DMC1H|LIM15
0010880 // re	0016328 // la	0005515 // pr	Dystrophin	DMD_HUMAN|DMD
0019695 // ch	0005739 // m	0003723 // RI	Dimethylglyci	M2GD_HUMAN|DMGDH
1903575 // co	0070062 // ex	0005515 // pr	Dermokine	DMKN_HUMAN|DMKN|UNQ729/PRO1411
0070173 // re	0005788 // er	0005178 // in	Dentin matrix	DMP1_HUMAN|DMP1
0060179 // m	0005634 // nu	0043565 // se	Doublesex- ar	DMRTA_HUMAN|DMRTA1|DMO
0006351 // tr	0005829 // cy	0044212 // tr	Cyclin-D-bindi	DMTF1_HUMAN|DMTF1|DMP1
0043504 // m	0005634 // nu	0017108 // 5'	DNA replicatio	DNA2_HUMAN|DNA2|DNA2L|KIAA0083
0060285 // cil	0005737 // cy	0005515 // pr	Protein kintou	KTU_HUMAN|DNAAF2|C14orf104|KTU
0070286 // ax	0005737 // cy		Dynein assem	DAAF3_HUMAN|DNAAF3|C19orf51
0036158 // ou	0005737 // cy	0045505 // dy	Dynein assem	DAAF5_HUMAN|DNAAF5|HEATR2
0007018 // m	0005737 // cy	0008569 // A	Dynein heavy	DYH10_HUMAN|DNAH10|KIAA2017
0007018 // m	0005737 // cy	0016887 // A	Dynein heavy	DYH12_HUMAN|DNAH12|DHC3|DLP12|DNAH12L|DNAI
0007018 // m	0005737 // cy	0008569 // A	Dynein heavy	DYH14_HUMAN|DNAH14|C1orf67
0007018 // m	0031514 // m	0008569 // A	Dynein heavy	DYH2_HUMAN|DNAH2|DNAHC2|DNHD3|KIAA1503
0036159 // in	0005737 // cy	0008569 // A	Dynein heavy	DYH3_HUMAN|DNAH3|DNAHC3B
0003341 // cil	0005737 // cy	0008569 // A	Dynein heavy	DYH5_HUMAN|DNAH5|DNAHC5|HL1|KIAA1603
0007018 // m	0005737 // cy	0008569 // A	Dynein heavy	DYH6_HUMAN|DNAH6|DNAHC6|DNHL1|HL2|KIAA1697
0036158 // ou	0005737 // cy	0045504 // dy	Dynein interm	DNAI1_HUMAN|DNAI1
0042769 // DI	0070062 // ex	0051087 // ch	DnaJ homolog	DNJA1_HUMAN|DNAJA1|DNAJ2|HDJ2|HSJ2|HSPF4
0090084 // ne	0016020 // m	0051087 // ch	DnaJ homolog	DNJA4_HUMAN|DNAJA4
0006457 // pr	0005737 // cy	0005515 // pr	DnaJ homolog	DJB13_HUMAN|DNAJB13|TSARG3|TSARG6
0051085 // ch	0016021 // in	0030544 // Hs	DnaJ homolog	DJB14_HUMAN|DNAJB14|UNQ9427/PRO34683
0000122 // ne	0005634 // nu	0005515 // pr	DnaJ homolog	DNJB5_HUMAN|DNAJB5|HSC40
1900034 // re	0005634 // nu	0031072 // he	DnaJ homolog	DNJB6_HUMAN|DNAJB6|HSJ2|MRJ|MSJ1
		0051087 // ch	DnaJ homolog	DNJB7_HUMAN|DNAJB7|HSC3
0030433 // EF	0005730 // nu	0051787 // m	DnaJ homolog	DNJB9_HUMAN|DNAJB9|MDG1|UNQ743/PRO1471
0042407 // cr	0005739 // m	0005515 // pr	DnaJ homolog	DJC11_HUMAN|DNAJC11
	0005737 // cy	0005515 // pr	DnaJ homolog	DJC12_HUMAN|DNAJC12|JDP1

2000641 // re	0043231 // in	0005515 // pr	DnaJ homolog	DJC13_HUMAN	DNAJC13|KIAA0678|RME8
0031333 // ne	0016021 // in	0005515 // pr	DnaJ homolog	DJC15_HUMAN	DNAJC15|DNAJD1|GIG22|HSD18
	0016021 // in		DnaJ homolog	DJC18_HUMAN	DNAJC18
0006457 // pr	0005730 // nu	0003723 // RN	DnaJ homolog	DJC21_HUMAN	DNAJC21|DNAJA5
0006457 // pr	0016020 // m	0005515 // pr	DnaJ homolog	DNJC4_HUMAN	DNAJC4|HSPF2|MCG18
0043524 // ne	0035579 // sp	0043008 // AT	DnaJ homolog	DNJC5_HUMAN	DNAJC5|CLN4
0035176 // so	0005615 // ex	0031072 // he	DnaJ homolog	DNJC9_HUMAN	DNAJC9
0036158 // ou	0036157 // ou	0045504 // dy	Dynein light c	DNAL1_HUMAN	DNAL1|C14orf168
2000582 // po	0005886 // pl	0005515 // pr	Dynein light c	DNAL4_HUMAN	DNAL4
0006259 // DI	0005576 // ex	0005509 // ca	Deoxyribonuc	DNSL2_HUMAN	DNASE1L2|DHP1|DNAS1L2
0006309 // ap	0005576 // ex	0005509 // ca	Deoxyribonuc	DNSL3_HUMAN	DNASE1L3|DHP2|DNAS1L3
0006897 // en	0016021 // in	0004888 // tra	Delta and Not	DNER_HUMAN	DNER|BET|UNQ262/PRO299
0036159 // in	0070062 // ex	0005524 // AT	Dynein heavy	DNHD1_HUMAN	DNHD1|C11orf47|CCDC35|DHCD1|DN
0030150 // pr	0005739 // m	0008270 // zin	DNL-type zinc	DNLZ_HUMAN	DNLZ|C9orf151
0048013 // ep	0001917 // ph	0003723 // RN	Dynamin-1	DYN1_HUMAN	DNM1|DNM
0061025 // m	0031966 // m	0003924 // GT	Putative GED	DMP46_HUMAN	DNM1P46|C15orf51
0000086 // G2	0030496 // m	0005515 // pr	Dynamin-2	DYN2_HUMAN	DNM2|DYN2
0051491 // po	0044327 // de	0003924 // GT	Dynamin-3	DYN3_HUMAN	DNM3|KIAA0820
0035023 // re	0030054 // ce	0005515 // pr	Dynamin-bind	DNMBP_HUMAN	DNMBP|KIAA1010
0006351 // tra	0000118 // his	0042803 // pr	Deoxynucleot	TDIF1_HUMAN	DNTTIP1|C20orf167|TDIF1
0006351 // tra	0005730 // nu	0003723 // RN	Deoxynucleot	TDIF2_HUMAN	DNTTIP2|ERBP|TDIF2
0007268 // ch	0030672 // sy	0030276 // cla	Double C2-like	DOC2A_HUMAN	DOC2A
0031340 // po	0098793 // pr	0030276 // cla	Double C2-like	DOC2B_HUMAN	DOC2B|DOC2BL
0001782 // B	0005737 // cy	0005085 // gu	Dedicator of c	DOC10_HUMAN	DOCK10|KIAA0694|ZIZ3
0043547 // po	0005829 // cy	0017048 // Rh	Dedicator of c	DOC11_HUMAN	DOCK11|ZIZ2
0046633 // al	0070062 // ex	0005515 // pr	Dedicator of c	DOCK2_HUMAN	DOCK2|KIAA0209
0043547 // po	0005829 // cy	0005515 // pr	Dedicator of c	DOCK3_HUMAN	DOCK3|KIAA0299|MOCA
0043547 // po	0005794 // Go	0005515 // pr	Dedicator of c	DOCK4_HUMAN	DOCK4|KIAA0716
0007596 // bl	0005829 // cy	0005085 // gu	Dedicator of c	DOCK6_HUMAN	DOCK6|KIAA1395
0001771 // im	0005737 // cy	0005085 // gu	Dedicator of c	DOCK8_HUMAN	DOCK8
0008612 // pe	0005829 // cy	0005515 // pr	Deoxyhypusin	DOHH_HUMAN	DOHH|HLRC1
0007166 // ce	0005634 // nu	0005515 // pr	Docking prote	DOK1_HUMAN	DOK1
0043312 // ne	0005886 // pl	0005515 // pr	Docking prote	DOK3_HUMAN	DOK3
0007411 // ax	0005829 // cy	0005515 // pr	Docking prote	DOK4_HUMAN	DOK4
0061098 // po	0030054 // ce	0008289 // lip	Protein Dok-7	DOK7_HUMAN	DOK7|C4orf25
0016310 // ph	0005789 // en	0004168 // do	Dolichol kinas	DOLK_HUMAN	DOLK|KIAA1094|TMEM15|UNQ2422/PR
0007275 // m	0005829 // cy	0003674 // m	Protein dopey	DOP2_HUMAN	DOPEY2|C21orf5|KIAA0933
0060972 // le	0005634 // nu	0005515 // pr	Protein DPCD	DPCD_HUMAN	DPCD

0007399 // ne	0071565 // nE	0046872 // m	Zinc finger pro	DPF1_HUMAN|DPF1|BAF45B|NEUD4
0007399 // ne	0071565 // nE	0008270 // zir	Zinc finger pro	DPF3_HUMAN|DPF3|BAF45C|CERD4
0017183 // pe	0005829 // cy	0005515 // pr	2-(3-amino-3-	DPH2_HUMAN|DPH2|DPH2L2
0017183 // pe	0005737 // cy	0005515 // pr	DPH3 homolo	DPH3_HUMAN|DPH3|DESR1|ZCSL2
0018279 // pr	0033185 // do	0005515 // pr	Dolichol-phos	DPM1_HUMAN|DPM1
0031647 // re	0030176 // int	0005515 // pr	Dolichol-phos	DPM3_HUMAN|DPM3
0006508 // pr	0005576 // ex	0008239 // di	Dipeptidyl pep	DPP2_HUMAN|DPP7|DPP2|QPP
0006508 // pr	0005829 // cy	0042802 // id	Dipeptidyl pep	DPP9_HUMAN|DPP9|DPRP2
0019827 // st	0005634 // nu	0003682 // ch	Development	DPPA2_HUMAN|DPPA2|PESCRG1
0007275 // m	0005637 // nu	0000030 // m	Probable C-ma	D19L2_HUMAN|DPY19L2|UNQ3127/PRO10284
0007286 // sp	0005637 // nu	0000030 // m	Putative C-ma	D19P1_HUMAN|DPY19L2P1
0018406 // pr	0005637 // nu	0000030 // m	Probable C-ma	D19L3_HUMAN|DPY19L3
0018406 // pr	0005637 // nu	0000030 // m	Probable C-ma	D19L4_HUMAN|DPY19L4
0006212 // ur	0005829 // cy	0017113 // di	Dihydropyrim	DPYD_HUMAN|DPYD
0007420 // br	0005829 // cy	0005515 // pr	Dihydropyrim	DPYL2_HUMAN|DPYSL2|CRMP2|ULIP2
0051017 // ac	0031941 // fil	0035374 // ch	Dihydropyrim	DPYL3_HUMAN|DPYSL3|CRMP4|DRP3|ULIP|ULIP1
0007399 // ne	0005829 // cy	0005515 // pr	Dihydropyrim	DPYL4_HUMAN|DPYSL4|CRMP3|ULIP4
0000398 // m	0005681 // sp	0005524 // AT	ATP-depender	DQX1_HUMAN|DQX1
0010506 // re	0005765 // lys	0005515 // pr	DNA damage-	DRAM1_HUMAN|DRAM1|DRAM
	0005737 // cy		Dynein regula	DRC3_HUMAN|DRC3|LRRC48
	0005856 // cy	0005515 // pr	Dynein regula	DRC7_HUMAN|DRC7|C16orf50|CCDC135
0035176 // so	0005887 // int	0001591 // do	D(4) dopamin	DRD4_HUMAN|DRD4
0007275 // m	0016604 // nu	0005515 // pr	Development	DRG1_HUMAN|DRG1|NEDD3
		0005515 // pr	Aspartate-rich	DRIC1_HUMAN|DRICH1|C22orf43
0030422 // pr	0005654 // nu	0003723 // RN	Ribonuclease	RNC_HUMAN|DROSHA|RN3|RNASE3L|RNASEN
0007417 // ce	0014069 // po	0008270 // zir	Dystrophin-re	DRP2_HUMAN|DRP2
0007156 // ho	0005886 // pl	0005509 // ca	Desmocollin-1	DSC1_HUMAN|DSC1|CDHF1
0086042 // ca	0005886 // pl	0086083 // ce	Desmocollin-2	DSC2_HUMAN|DSC2|CDHF2|DSC3
0007156 // ho	0005886 // pl	0005509 // ca	Desmocollin-3	DSC3_HUMAN|DSC3|CDHF3|DSC4
0007399 // ne	0005886 // pl	0005515 // pr	Down syndror	DSCAM_HUMAN|DSCAM
0008150 // bi	0005575 // ce	0003674 // m	Down syndror	DSC10_HUMAN|DSCR10
0008150 // bi	0005575 // ce	0003674 // m	Down syndror	DSCR4_HUMAN|DSCR4|DCRB|DSCRB
0008150 // bi	0005575 // ce	0005515 // pr	Down syndror	DSCR8_HUMAN|DSCR8|C21orf65|MTAG2
0030206 // ch	0005783 // en	0047757 // ch	Dermatan-sul	DSE_HUMAN|DSE|SART2
0030208 // de	0016021 // int	0047757 // ch	Dermatan-sul	DSEL_HUMAN|DSEL|C18orf4|NCAG1
0098609 // ce	0005829 // cy	0015643 // to	Desmoglein-1	DSG1_HUMAN|DSG1|CDHF4
0007156 // ho	0005886 // pl	0005509 // ca	Desmoglein-2	DSG2_HUMAN|DSG2|CDHF5
0070268 // co	0005886 // pl	0005509 // ca	Desmoglein-3	DSG3_HUMAN|DSG3|CDHF6

0002934 // de	0005886 // pl	0003723 // RN	Desmoplakin	DESP_HUMAN|DSP
0045104 // in	0005938 // ce	0005515 // pr	Dystonin	DYST_HUMAN|DST|BP230|BP240|BPAG1|DMH|DT|KIA
0006450 // re	0005730 // nu	0002161 // ar	D-aminoacyl-t	DTD1_HUMAN|DTD1|C20orf88|DUEB|HARS2
0006399 // tR	0005737 // cy	0002161 // ar	Putative D-tyr	DTD2_HUMAN|DTD2|C14orf126
0007165 // sig			Death domain	DTHD1_HUMAN|DTHD1
0007268 // ch	0030054 // ce	0008270 // zir	Dystrobrevin a	DTNA_HUMAN|DTNA|DRP3
	0045202 // sy	0005515 // pr	Dystrobrevin b	DTNB_HUMAN|DTNB
0043506 // re	0030426 // gr	0005515 // pr	Dysbindin	DTBP1_HUMAN|DTNBP1|BLOC1S8|My031
0007219 // No	0031965 // nu	0005515 // pr	Probable E3 u	DTX2_HUMAN|DTX2|KIAA1528|RNF58
0016567 // pr	0005737 // cy	0005515 // pr	Probable E3 u	DTX3_HUMAN|DTX3|RNF154
0006974 // ce	0005829 // cy	0003723 // RN	E3 ubiquitin-p	DTX3L_HUMAN|DTX3L|BBAP
0007219 // No	0005829 // cy	0004842 // ub	E3 ubiquitin-p	DTX4_HUMAN|DTX4|KIAA0937|RNF155
0051591 // re	0005886 // pl	0016174 // NA	Dual oxidase 1	DUOX1_HUMAN|DUOX1|DUOX|LNOX1|THOX1
0009615 // re	0005886 // pl	0020037 // he	Dual oxidase 2	DUOX2_HUMAN|DUOX2|LNOX2|THOX2
2000379 // po	0005789 // er		Dual oxidase m	DOXA1_HUMAN|DUOXA1|NIP|NUMBIP
2000609 // re	0016021 // in		Dual oxidase m	DOXA2_HUMAN|DUOXA2
0002943 // tR		0003723 // RN	tRNA-dihydro	DUS3L_HUMAN|DUS3L
0002943 // tR		0050660 // fla	tRNA-dihydro	DUS4L_HUMAN|DUS4L
0035556 // in	0005634 // nu	0005515 // pr	Dual specificit	DUS1_HUMAN|DUSP1|CL100|MKP1|PTPN10|VH1
0002819 // re	0005794 // Go	0016791 // ph	Dual specificit	DUS10_HUMAN|DUSP10|MKP5
0006470 // pr		0005515 // pr	Dual specificit	DS13B_HUMAN|DUSP13|DUSP13B|TMDP
0016311 // de	0005634 // nu	0004725 // pr	Dual specificit	DUS18_HUMAN|DUSP18|LMWDSP20
0006469 // ne		0008579 // JU	Dual specificit	DUS19_HUMAN|DUSP19|DUSP17|LMWDSP3|SKRP1
0016311 // de	0005634 // nu	0005515 // pr	Dual specificit	DUS23_HUMAN|DUSP23|LDP3|VHZ
0046329 // ne	0005829 // cy	0019901 // pr	Dual specificit	DUS3_HUMAN|DUSP3|VHR
0016311 // de	0005634 // nu	0004725 // pr	Dual specificit	DUS4_HUMAN|DUSP4|MKP2|VH2
0051409 // re	0005829 // cy	0017017 // M	Dual specificit	DUS6_HUMAN|DUSP6|MKP3|PYST1
0035335 // pe	0005634 // nu	0004725 // pr	Dual specificit	DUS8_HUMAN|DUSP8|C11orf81|VH5
0006470 // pr	0005829 // cy	0005515 // pr	Dual specificit	DUS9_HUMAN|DUSP9|MKP4
0050808 // sy	0030424 // ax	0005109 // fri	Segment pola	DVL1_HUMAN|DVL1
0008152 // m	0005829 // cy	0000287 // m	Decapping an	DXO_HUMAN|DXO|DOM3L|DOM3Z|NG6
0007030 // Go	0016020 // m	0019899 // er	Dymeclin	DYM_HUMAN|DYM
0051293 // es	0005829 // cy	0003723 // RN	Cytoplasmic d	DYHC1_HUMAN|DYNC1H1|DHC1|DNCH1|DNCL|DNECL
0006888 // ER	0005829 // cy	0045504 // dy	Cytoplasmic d	DC1I1_HUMAN|DYNC1I1|DNCI1|DNCIC1
0090267 // po	0005829 // cy	0003723 // RN	Cytoplasmic d	DC1L1_HUMAN|DYNC1LI1|DNCLI1
0007346 // re	0000776 // ki	0005515 // pr	Dynein light c	DYLT3_HUMAN|DYNLT3|TCTE1L|TCTE1XL
0018108 // pe	0043231 // in	0004674 // pr	Dual specificit	DYRK4_HUMAN|DYRK4
0001778 // pl	0042383 // sa	0005515 // pr	Dysferlin	DYSF_HUMAN|DYSF|FER1L1

		0005515 // pr	Double zinc ri	DZAN1_HUMAN|DZANK1|C20orf12|C20orf84
0007283 // sp	0005634 // nu	0005515 // pr	Zinc finger pro	DZIP1_HUMAN|DZIP1|DZIP|DZIP2|KIAA0996
0000209 // pr	0044322 // er	0003723 // RN	E3 ubiquitin-p	DZIP3_HUMAN|DZIP3|KIAA0675
1990090 // ce	0005654 // nu	0005515 // pr	Transcription	E2F1_HUMAN|E2F1|RBBP3
0045944 // po	0005654 // nu	0046983 // pr	Transcription	E2F2_HUMAN|E2F2
0006366 // tra	0005654 // nu	0005515 // pr	ELL-associated	EAF2_HUMAN|EAF2|TRAITS|BM-040
0008284 // po	0005634 // nu		E2F-associated	EAPP_HUMAN|EAPP|C14orf11|BM-036
0006424 // glu	0005739 // m	0004818 // glu	Probable gluta	SYEM_HUMAN|EARS2|KIAA1970
0006915 // ap	0016021 // in	0016505 // pe	Receptor-bind	RCAS1_HUMAN|EBAG9|RCAS1
0007275 // m	0005634 // nu	0003677 // DI	Transcription	COE4_HUMAN|EBF4|COE4|KIAA1442
0006364 // rR	0034399 // nu	0003723 // RN	Probable rRNA	EBP2_HUMAN|EBNA1BP2|EBP2
0006855 // dr	0005887 // in	0004888 // tra	3-beta-hydrox	EBP_HUMAN|EBP
0006366 // tra	0005634 // nu	0005515 // pr	Protein ecdyso	ECD_HUMAN|ECD
0006635 // fa	0005829 // cy	0016831 // ca	Ethylmalonyl-	ECHD1_HUMAN|ECHDC1
0006635 // fa	0005739 // m	0004300 // en	Enoyl-CoA hyd	ECHD2_HUMAN|ECHDC2
0008152 // m	0005739 // m	0003824 // ca	Enoyl-CoA hyd	ECHD3_HUMAN|ECHDC3|PP1494|PP8332
0002576 // pl	0005576 // ex	0004871 // sig	Extracellular r	ECM1_HUMAN|ECM1
0043547 // po		0005089 // Rh	Epithelial cell-	ECT2L_HUMAN|ECT2L|C6orf91|LFDH
0033209 // tu	0005576 // ex	0005515 // pr	Ectodysplasin-	EDA_HUMAN|EDA|ED1|EDA2
0033209 // tu	0016021 // in	0004872 // re	Tumor necros	EDAR_HUMAN|EDAR|DL
0007275 // m	0005829 // cy	0005515 // pr	Ectodysplasin-	EDAD_HUMAN|EDARADD
0006491 // N-	0005783 // en	0005509 // ca	ER degradatio	EDEM3_HUMAN|EDEM3|C1orf22
0045893 // po	0005634 // nu	0003723 // RN	Endothelial di	EDF1_HUMAN|EDF1
0007155 // ce	1903561 // ex	0005509 // ca	EGF-like repea	EDIL3_HUMAN|EDIL3|DEL1
0006885 // re	0005615 // ex	0031708 // en	Endothelin-1	EDN1_HUMAN|EDN1
0014826 // ve	0005576 // ex	0031708 // en	Endothelin-2	EDN2_HUMAN|EDN2
0007202 // ac	0005886 // pl	0004962 // en	Endothelin-1 r	EDNRA_HUMAN|EDNRA|ETA|ETRA
0019934 // cG	0005886 // pl	0004962 // en	Endothelin rec	EDNRB_HUMAN|EDNRB|ETRB
0006351 // tra	0034774 // se	0005525 // GT	Elongation fac	EF1A1_HUMAN|EEF1A1|EEF1A|EF1A|LENG7
0006414 // tra	0005737 // cy	0005525 // GT	Elongation fac	EF1A2_HUMAN|EEF1A2|EEF1AL|STN
0006414 // tra	0005853 // eu	0005515 // pr	Elongation fac	EF1B_HUMAN|EEF1B2|EEF1B|EF1B
0006414 // tra		0005515 // pr	Putative elong	EF1DL_HUMAN|EEF1DP3
0043065 // po	0005829 // cy	0005515 // pr	Eukaryotic tra	MCA3_HUMAN|EEF1E1|AIMP3|P18
0006749 // glu	0070062 // ex	0005515 // pr	Elongation fac	EF1G_HUMAN|EEF1G|EF1G|PRO1608
0042493 // re	0034774 // se	0003723 // RN	Elongation fac	EF2_HUMAN|EEF2|EF2
0018023 // pe	0005829 // cy	0016279 // pr	Protein-lysine	EF2KT_HUMAN|EEF2KMT|FAM86A|SB153
0006281 // DI		0003677 // DI	Endonuclease	EEPD1_HUMAN|EEPD1|KIAA1706
		0005509 // ca	EF-hand calciu	EFCB1_HUMAN|EFCAB1
		0005509 // ca	EF-hand calciu	EFC11_HUMAN|EFCAB11|C14orf143
		0005509 // ca	EF-hand calciu	EFCB2_HUMAN|EFCAB2
		0005509 // ca	EF-hand calciu	EFCB5_HUMAN|EFCAB5
		0005509 // ca	EF-hand calciu	EFCB8_HUMAN|EFCAB8
		0005509 // ca	EF-hand calciu	EFCB9_HUMAN|EFCAB9

	1903561 // ex	0005515 // pr	EGF-containin	FBLN4_HUMAN|EFEMP2|FBLN4|UNQ200/PRO226
1990830 // ce		0005515 // pr	EF-hand doma	EFHC2_HUMAN|EFHC2
0031175 // ne	0070062 // ex	0005509 // ca	EF-hand doma	EFHD1_HUMAN|EFHD1|SWS2|PP3051
	0045121 // m	0045296 // ca	EF-hand doma	EFHD2_HUMAN|EFHD2|SWS1
1902961 // po	0005886 // pl	0005515 // pr	Ephrin-A1	EFNA1_HUMAN|EFNA1|EPLG1|LERK1|TNFAIP4
0048672 // po	0005886 // pl	0030297 // tr	Ephrin-A5	EFNA5_HUMAN|EFNA5|EPLG7|LERK7
0048013 // ep	0005886 // pl	0005515 // pr	Ephrin-B1	EFNB1_HUMAN|EFNB1|EFL3|EPLG2|LERK2
0007267 // ce	0005886 // pl	0001618 // vi	Ephrin-B3	EFNB3_HUMAN|EFNB3|EPLG8|LERK8
0046854 // ph	0070062 // ex		Protein EFR3 I	EFR3A_HUMAN|EFR3A|KIAA0143
0007155 // ce	0005737 // cy	0005515 // pr	Embryonal Fy	EFS_HUMAN|EFS|CASS3
0018108 // pe	0005576 // ex	0005515 // pr	Pro-epiderma	EGF_HUMAN|EGF
0010811 // po	0005615 // ex	0005509 // ca	Epidermal gro	EGFL6_HUMAN|EGFL6|MAEG|PP648|UNQ281/PRO320
0045746 // ne	0005576 // ex	0005509 // ca	Epidermal gro	EGFL7_HUMAN|EGFL7|MEGF7|UNQ187/PRO1449
0001701 // in	0005576 // ex	0005509 // ca	Epidermal gro	EGFL8_HUMAN|EGFL8|C6orf8|NG3|UNQ8752/PRO2992
0030198 // ex	0005614 // in	0005509 // ca	Pikachurin	EGFLA_HUMAN|EGFLAM|AGRINL|AGRNL
0061029 // ey	0005886 // pl	0005515 // pr	Epidermal gro	EGFR_HUMAN|EGFR|ERBB|ERBB1|HER1
0006915 // ap	0005829 // cy	0005515 // pr	Egl nine homo	EGLN3_HUMAN|EGLN3
0045893 // po	0005634 // nu	0043565 // se	Early growth r	EGR1_HUMAN|EGR1|KROX24|ZNF225
0001938 // po	0005634 // nu	0003700 // tr	Early growth r	EGR3_HUMAN|EGR3|PILOT
0008150 // bi	0005768 // en	0003674 // m	EH domain-bi	EH1L1_HUMAN|EHBP1L1
0032456 // en	0070062 // ex	0003676 // nu	EH domain-co	EHD4_HUMAN|EHD4|HCA10|HCA11|PAST4|FKSG7
0006366 // tr	0005794 // Go	0003677 // DI	ETS homolog	EHF_HUMAN|EHF|ESE3|ESE3B|ESEJ
0006635 // fa	0005782 // pe	0005102 // re	Peroxisomal b	ECHP_HUMAN|EHHADH|ECHD
0006306 // DI	0005654 // nu	0005515 // pr	Histone-lysine	EHMT2_HUMAN|EHMT2|BAT8|C6orf30|G9A|KMT1C|N
0045662 // ne	0005634 // nu		EP300-interac	EID2B_HUMAN|EID2B|EID3
0006310 // DI	0005730 // nu	0005515 // pr	EP300-interac	EID3_HUMAN|EID3
0006446 // re	0005737 // cy	0003723 // RI	Eukaryotic tra	EIF1_HUMAN|EIF1|SUI1
0006413 // tr	0005829 // cy	0003723 // RI	Eukaryotic tra	IF1AX_HUMAN|EIF1AX|EIF1A|EIF4C
0006446 // re	0005575 // ce	0003723 // RI	Eukaryotic tra	EIF1B_HUMAN|EIF1B
0007417 // ce	0005851 // eu	0003743 // tr	Translation in	EI2BB_HUMAN|EIF2B2|EIF2BB
0043434 // re	0005851 // eu	0005515 // pr	Translation in	EI2BG_HUMAN|EIF2B3
0043434 // re	0005851 // eu	0005515 // pr	Translation in	EI2BD_HUMAN|EIF2B4|EIF2BD
0032790 // rib	0005737 // cy	0003743 // tr	Eukaryotic tra	EIF2D_HUMAN|EIF2D|HCA56|LGTN
0034644 // ce	0010494 // cy	0003723 // RI	Eukaryotic tra	IF2A_HUMAN|EIF2S1|EIF2A
0002176 // m	0005829 // cy	0003723 // RI	Eukaryotic tra	IF2B_HUMAN|EIF2S2|EIF2B
1902416 // po	0005852 // eu	0003723 // RI	Eukaryotic tra	EIF3C_HUMAN|EIF3C|EIF3S8
0006413 // tr	0005852 // eu	0031369 // tr	Eukaryotic tra	EIFCL_HUMAN|EIF3CL
0075522 // IR	0005829 // cy	0005515 // pr	Eukaryotic tra	EIF3F_HUMAN|EIF3F|EIF3S5
0006413 // tr	0005829 // cy	0003723 // RI	Eukaryotic tra	EIF3G_HUMAN|EIF3G|EIF3S4
0006413 // tr	0070062 // ex	0003743 // tr	Eukaryotic tra	EIF3I_HUMAN|EIF3I|EIF3S2|TRIP1
0006413 // tr	0005829 // cy	0003723 // RI	Eukaryotic tra	EIF3L_HUMAN|EIF3L|EIF3EIP|EIF3S6IP|HSPC021|HSPC0
0002183 // cy	0005852 // eu	0005515 // pr	Eukaryotic tra	EIF3M_HUMAN|EIF3M|HFLB5|PCID1|GA17|PNAS-125

0006413 // tra	0005829 // cy	0003723 // RN	Eukaryotic init	IF4A1_HUMAN|EIF4A1|DDX2A|EIF4A
0000184 // nu	0005829 // cy	0003723 // RN	Eukaryotic init	IF4A3_HUMAN|EIF4A3|DDX48|KIAA0111
0006417 // re	0005829 // cy	0003743 // tra	Eukaryotic tra	IF4E3_HUMAN|EIF4E3
0045947 // ne	0016281 // eu	0005515 // pr	Eukaryotic tra	4EBP3_HUMAN|EIF4EBP3
0006446 // re	0005829 // cy	0003723 // RN	Eukaryotic tra	IF4G2_HUMAN|EIF4G2|DAP5|OK/SW-cl.75
0001731 // fo	0005737 // cy	0003723 // RN	Eukaryotic tra	IF5_HUMAN|EIF5
0042256 // m	0070062 // ex	0005515 // pr	Eukaryotic tra	IF6_HUMAN|EIF6|EIF3A|ITGB4BP|OK/SW-cl.27
0006355 // re	0005654 // nu	0003723 // RN	ELAV-like prot	ELAV2_HUMAN|ELAVL2|HUB
0007399 // ne		0017091 // AU	ELAV-like prot	ELAV3_HUMAN|ELAVL3|HUC|PLE21
0001959 // re	0005654 // nu	0005515 // pr	ETS-related tr	ELF1_HUMAN|ELF1
0030198 // ex	0005794 // Go	0005515 // pr	ETS-related tr	ELF3_HUMAN|ELF3|ERT|ESX|JEN
0045944 // po	0016604 // nu	0005515 // pr	ETS-related tr	ELF4_HUMAN|ELF4|ELFR|MEF
0006366 // tra	0005737 // cy	0000977 // RN	ETS-related tr	ELF5_HUMAN|ELF5|ESE2
0050808 // sy	0060076 // ex	0019902 // ph	Protein ELFN1	ELFN1_HUMAN|ELFN1|PPP1R28
0030154 // ce	0005739 // m	0032422 // pu	ETS domain-co	ELK3_HUMAN|ELK3|NET|SAP2
0070932 // hi	0005829 // cy	0000978 // RN	ETS domain-co	ELK4_HUMAN|ELK4|SAP1
0042795 // sn	0005654 // nu	0005515 // pr	RNA polymera	ELL2_HUMAN|ELL2
0006354 // DN	0005634 // nu	0035326 // en	RNA polymera	ELL3_HUMAN|ELL3
0030036 // ac	0005737 // cy	0005515 // pr	Engulfment an	ELMO1_HUMAN|ELMO1|KIAA0281
0007010 // cy	0005829 // cy	0005515 // pr	Engulfment an	ELMO2_HUMAN|ELMO2|CED12A|KIAA1834
0006915 // ap	0005737 // cy	0005515 // pr	Engulfment an	ELMO3_HUMAN|ELMO3
0031647 // re	0005829 // cy	0005096 // GT	ELMO domain	ELMD1_HUMAN|ELMOD1
0008015 // bl	0005576 // ex	0005515 // pr	Elastin	ELN_HUMAN|ELN
0006357 // re	0005654 // nu	0005515 // pr	Elongin-A	ELOA1_HUMAN|ELOA|TCEB3|MSTP059
0006366 // tra	0005654 // nu	0005515 // pr	Elongin-A2	ELOA2_HUMAN|ELOA2|TCEB3B|TCEB3L
0048096 // ch	0008023 // tra	0005515 // pr	Transcription	ELOF1_HUMAN|ELOF1
0042761 // ve	0030176 // int	0009922 // fa	Elongation of	ELOV3_HUMAN|ELOVL3|CIG30
0042761 // ve	0030176 // int	0008020 // G-	Elongation of	ELOV4_HUMAN|ELOVL4
0042761 // ve	0030176 // int	0102756 /// 0	Elongation of	ELOV5_HUMAN|ELOVL5|ELOVL2|PRO0530
0042759 // lo	0030176 // int	0009922 // fa	Elongation of	ELOV6_HUMAN|ELOVL6|FACE|LCE
0042761 // ve	0030176 // int	0009922 // fa	Elongation of	ELOV7_HUMAN|ELOVL7
0006351 // tra	0033588 // El	0003674 // m	Elongator com	ELP6_HUMAN|ELP6|ATP1|C3orf75|TMEM103
	0005576 // ex		ER membrane	EMC10_HUMAN|EMC10|C19orf63|HSM1|INM02|UNQ7
0034975 // pr	0005737 // cy	0005515 // pr	ER membrane	EMC2_HUMAN|EMC2|KIAA0103|TTC35
	0005737 // cy	0005515 // pr	ER membrane	EMC9_HUMAN|EMC9|C14orf122|FAM158A|CGI-112
0007077 // m	0005635 // nu	0005515 // pr	Emerin	EMD_HUMAN|EMD|EDMD|STA
	0005794 // Go		EMI domain-c	EMID1_HUMAN|EMID1|EMU1
0007155 // ce	0031012 // ex	0030023 // ex	EMILIN-2	EMIL2_HUMAN|EMILIN2

0000226 // m	0005829 // cy	0005515 // pr	Echinoderm m	EMAL1_HUMAN|EML1|EMAP1|EMAPL|EMAPL1
0031115 // ne	0005737 // cy	0008017 // m	Echinoderm m	EMAL2_HUMAN|EML2|EMAP2|EMAPL2
	0005737 // cy	0005515 // pr	Echinoderm m	EMAL3_HUMAN|EML3
0008150 // bi	0005737 // cy	0003824 // ca	Echinoderm m	EMAL5_HUMAN|EML5
0045765 // re	0005737 // cy	0019900 // ki	Epithelial mer	EMP2_HUMAN|EMP2|XMP
0008219 // ce	0016021 // in	0005515 // pr	Epithelial mer	EMP3_HUMAN|EMP3|YMP
1990138 // ne	0005730 // nu	0043565 // se	Homeobox pr	EMX1_HUMAN|EMX1
0009953 // dc	0005634 // nu	0001078 // tr	Homeobox pr	HME1_HUMAN|EN1
0007411 // ax	0030175 // fil	0005515 // pr	Protein enabl	ENAH_HUMAN|ENAH|MENA
0002576 // pl	0070062 // ex	0003676 // nu	Endonuclease	ENDD1_HUMAN|ENDOD1|KIAA0830
0001701 // in	0005829 // cy	0003676 // nu	Endonuclease	NUCG_HUMAN|ENDOG
0006508 // pr	0005615 // ex	0008236 // se	Poly(U)-specif	ENDOU_HUMAN|ENDOU
0001934 // pc	0005615 // ex	0005539 // gl	Endoglin	EGLN_HUMAN|ENG|END
0045747 // pc	0005886 // pl		Adropin	ENHO_HUMAN|ENHO|C9orf165|UNQ470/PRO830
	0015630 // m	0005515 // pr	Enkurin doma	ENKD1_HUMAN|ENKD1|C16orf48|UNQ6410/PRO21183
	0001669 // ac	0005516 // ca	Enkurin	ENKUR_HUMAN|ENKUR|C10orf63
0000122 // ne	0009986 // ce	0001227 // tr	Alpha-enolase	ENOA_HUMAN|ENO1|ENO1L1|MBPB1|MPB1
0006094 // gl	0005829 // cy	0004634 // ph	Gamma-enola	ENOG_HUMAN|ENO2
0006094 // gl	0005829 // cy	0004634 // ph	Beta-enolase	ENOB_HUMAN|ENO3
0007624 // ul	0005615 // ex	0003676 // nu	Ecto-NOX disu	ENOX2_HUMAN|ENOX2|COVA1
0043171 // pe	0005903 // br	0070006 // m	Glutamyl amir	AMPE_HUMAN|ENPEP
0046130 // pu	0005886 // pl	0047710 // bi	Bis(5'-adenos	ENPP4_HUMAN|ENPP4|KIAA0879|NPP4
0008152 // m	0016021 // in	0016787 // hy	Ectonucleotid	ENPP5_HUMAN|ENPP5|UNQ550/PRO1107
0006629 // lip	0031225 // ar	0047390 // gl	Ectonucleotid	ENPP6_HUMAN|ENPP6|UNQ1889/PRO4334
0000278 // m	0005737 // cy	0008200 // io	Alpha-endosu	ENSA_HUMAN|ENSA
0007155 // ce	0005887 // in	0005515 // pr	Ectonucleosid	ENTP1_HUMAN|ENTPD1|CD39
0034656 // nu	0005605 // ba	0005524 // AT	Ectonucleosid	ENTP2_HUMAN|ENTPD2|CD39L1
0009134 // nu	0005886 // pl	0017110 // nu	Ectonucleosid	ENTP3_HUMAN|ENTPD3|CD39L3
0034656 // nu	0000139 // Gc	0017110 // nu	Ectonucleosid	ENTP4_HUMAN|ENTPD4|KIAA0392|LALP70|LYSAL1
0034656 // nu	0005794 // Gc	0008894 // gu	Ectonucleosid	ENTP6_HUMAN|ENTPD6|CD39L2|IL6ST2
0034656 // nu	0016021 // in	0017111 // nu	Ectonucleosid	ENTP7_HUMAN|ENTPD7|LALP1
0034656 // nu	0016021 // in	0017110 // nu	Ectonucleosid	ENTP8_HUMAN|ENTPD8|UNQ2492/PRO5779
0002302 // CI	0005634 // nu	0003682 // ch	Eomesodermi	EOMES_HUMAN|EOMES|TBR2
0030324 // lu	0005829 // cy	0005515 // pr	Endothelial PA	EPAS1_HUMAN|EPAS1|BHLHE73|HIF2A|MOP2|PASD2
0031032 // ac	0005886 // pl	0005515 // pr	Band 4.1-like	E41L1_HUMAN|EPB41L1|KIAA0338
0031032 // ac	0070062 // ex	0005515 // pr	Band 4.1-like	E41L2_HUMAN|EPB41L2
0030865 // cc	0033270 // pa	0005515 // pr	Band 4.1-like	E41L3_HUMAN|EPB41L3|DAL1|KIAA0987
0031032 // ac	0005737 // cy	0003674 // m	Band 4.1-like	E41LA_HUMAN|EPB41L4A|EPB41L4
0042060 // w	0005737 // cy	0008092 // cy	Band 4.1-like	E41LB_HUMAN|EPB41L4B|EHM2|LULU2
0006281 // DI	0032777 // Pi	0004402 // hi	Enhancer of p	EPC2_HUMAN|EPC2

0045944 // po	0005887 // int	0032403 // pr	Epithelial cell	EPCAM_HUMAN|EPCAM|GA733-2|M1S2|M4S1|MIC18
0007160 // ce	0070062 // ex	0005509 // ca	Mammalian e	EPDR1_HUMAN|EPDR1|MERP1|UCC1
0008284 // po	0005887 // int	0008083 // gr	Epigen	EPGN_HUMAN|EPGN|UNQ3072/PRO9904
0034446 // su	0005886 // pl	0019901 // pr	Ephrin type-A	EPHA1_HUMAN|EPHA1|EPH|EPHT|EPHT1
0016477 // ce	0005886 // pl	0005515 // pr	Ephrin type-A	EPHA3_HUMAN|EPHA3|ETK|ETK1|HEK|TYRO4
2001108 // po	0030054 // ce	0005515 // pr	Ephrin type-A	EPHA4_HUMAN|EPHA4|HEK8|SEK|TYRO1
0048013 // ep	0005886 // pl	0005524 // AT	Ephrin type-A	EPHA6_HUMAN|EPHA6|EHK2|HEK12
0001934 // po	0005886 // pl	0005004 // GI	Ephrin type-A	EPHA7_HUMAN|EPHA7|EHK3|HEK11
0033628 // re	0005886 // pl	0005004 // GI	Ephrin type-A	EPHA8_HUMAN|EPHA8|EEK|HEK3|KIAA1459
1902723 // ne	0005886 // pl	0005515 // pr	Ephrin type-B	EPHB1_HUMAN|EPHB1|ELK|EPHT2|HEK6|NET
0007413 // ax	0005886 // pl	0005003 // ep	Ephrin type-B	EPHB3_HUMAN|EPHB3|ETK2|HEK2|TYRO6
0048013 // ep	0005886 // pl	0005003 // ep	Ephrin type-B	EPHB4_HUMAN|EPHB4|HTK|MYK1|TYRO11
0006468 // pr	0005886 // pl	0004872 // re	Ephrin type-B	EPHB6_HUMAN|EPHB6
0097176 // ep	0016021 // int	0033961 // cis	Epoxide hydro	HYEP_HUMAN|EPHX1|EPHX|EPOX
0097176 // ep	0005777 // pe	0005102 // re	Bifunctional e	HYES_HUMAN|EPHX2
	0016021 // int	0016787 // hy	Epoxide hydro	EPHX4_HUMAN|EPHX4|ABHD7|EH4|EPHXRP
	0019897 // ex	0005515 // pr	Epsin-3	EPN3_HUMAN|EPN3
0006357 // re	0070449 // el	0003682 // ch	Elongin BC an	EPOP_HUMAN|EPOP|C17orf96|PRR28
0045109 // int	0005737 // cy	0003723 // RN	Epiplakin	EPIPL_HUMAN|EPPK1|EPIPL
0006424 // glu	0005829 // cy	0004827 // pr	Bifunctional g	SYEP_HUMAN|EPRS|GLNS|PARS|QARS|QPRS|PIG32
0009967 // po	0005903 // br	0005515 // pr	Epidermal gro	EPS8_HUMAN|EPS8
0007266 // Rh	0043234 // pr	0005515 // pr	Epidermal gro	ES8L1_HUMAN|EPS8L1|DRC3|EPS8R1|PP10566
0007266 // Rh	0043234 // pr	0005515 // pr	Epidermal gro	ES8L2_HUMAN|EPS8L2|EPS8R2|PP13181
0072677 // eo	0005615 // ex	0020037 // he	Eosinophil pe	PERE_HUMAN|EPX|EPER|EPO|EPP
0043171 // pe	0005788 // en	0070006 // m	Endoplasmic r	ERAP1_HUMAN|ERAP1|APPILS|ARTS1|KIAA0525|UNQ5
0043171 // pe	0016021 // int	0070006 // m	Endoplasmic r	ERAP2_HUMAN|ERAP2|LRAP
0071363 // ce	0005886 // pl	0005515 // pr	Receptor tyro	ERBB2_HUMAN|ERBB2|HER2|MLN19|NEU|NGL
0061098 // po	0005886 // pl	0005515 // pr	Receptor tyro	ERBB3_HUMAN|ERBB3|HER3
0038111 // int	0005654 // nu	0005154 // ep	Receptor tyro	ERBB4_HUMAN|ERBB4|HER4
0006605 // pr	0005634 // nu	0005515 // pr	Erbin	ERBIN_HUMAN|ERBIN|ERBB2IP|KIAA1225|LAP2
0007275 // m	0005737 // cy	0005515 // pr	ELKS/Rab6-int	RB6I2_HUMAN|ERC1|ELKS|KIAA1081|RAB6IP2
0030282 // bo	0005654 // nu	0005515 // pr	TFIIH basal tra	ERCC2_HUMAN|ERCC2|XPD|XPDC
1904357 // ne	0070522 // ER	0010521 // te	DNA repair en	XPF_HUMAN|ERCC4|ERCC11|XPF
0010225 // re	0005654 // nu	0005515 // pr	DNA repair pr	ERCC5_HUMAN|ERCC5|ERCM2|XPG|XPGC
0051301 // ce	0005829 // cy	0005515 // pr	DNA excision	ERC6L_HUMAN|ERCC6L|PICH
0019217 // re	0005623 // ce	0005179 // ho	Erythroferron	ERFE_HUMAN|ERFE|C1QTNF15|CTRP15|FAM132B
0016477 // ce	0030529 // int	0004871 // sig	Transcriptiona	ERG_HUMAN|ERG
0006888 // ER	0016021 // int	0005515 // pr	Endoplasmic r	ERGI1_HUMAN|ERGIC1|ERGIC32|KIAA1181|HT034
0007049 // ce	0030496 // m	0003723 // RN	Enhancer of ru	ERH_HUMAN|ERH
		0005515 // pr	Glutamate-ric	ERIC2_HUMAN|ERICH2

0045717 // ne	0005789 // er	0005515 // pr	Erlin-2	ERLN2_HUMAN|ERLIN2|C8orf2|SPFH2|UNQ2441/PRO5
	0016021 // in		Erythroid mer	ERMAP_HUMAN|ERMAP|RD|SC
0001763 // m	0030175 // fil	0051015 // ac	Ermin	ERMIN_HUMAN|ERMN|KIAA1189
0006508 // pr	0016021 // in	0008237 // m	Endoplasmic r	ERMP1_HUMAN|ERMP1|FXNA|KIAA1815
0006464 // ce	0005783 // er	0005515 // pr	ERO1-like prot	ERO1A_HUMAN|ERO1A|ERO1L|UNQ434/PRO865
0006457 // pr	0005789 // er	0003756 // pr	Endoplasmic r	ERP44_HUMAN|ERP44|KIAA0573|TXNDC4|UNQ532/PR
0070373 // ne	0005737 // cy	0019901 // pr	ERBB recepto	ERRFI_HUMAN|ERRFI1|MIG6
0008150 // bi	0070062 // ex	0003674 // m	Endogenous r	ENR1_HUMAN|ERV3-1|ERV3
0006949 // sy	0005615 // ex	0005515 // pr	Suppressyn	SUPYN_HUMAN|ERVH48-1|C21orf105|HERV-Fb1|NDUF
	0016021 // in		Endogenous r	MER34_HUMAN|ERVMER34-1|LP9056
0006949 // sy	0016021 // in		Syncytin-1	SYCY1_HUMAN|ERVW-1|ERVWE1
0007156 // hc	0005886 // pl		Endothelial ce	ESAM_HUMAN|ESAM|UNQ220/PRO246
1902204 // pc	0005576 // ex	0005178 // in	Endothelial ce	ESM1_HUMAN|ESM1
0051494 // ne	0005902 // m	0051015 // ac	Espin	ESPN_HUMAN|ESPN|DFNB36|LP2654
0007605 // se	0031941 // fil	0051015 // ac	Espin-like prot	ESPNL_HUMAN|ESPNL
0043484 // re	0016604 // nu	0003729 // m	Epithelial splic	ESRP1_HUMAN|ESRP1|RBM35A
0043484 // re	0005634 // nu	0003723 // RN	Epithelial splic	ESRP2_HUMAN|ESRP2|RBM35B|PP7059
0043401 // st	0005654 // nu	0001077 // tra	Steroid hormc	ERR1_HUMAN|ESRRA|ERR1|ESRL1|NR3B1
0043401 // st	0032039 // in	0043565 // se	Steroid hormc	ERR2_HUMAN|ESRRB|ERRB2|ESRL2|NR3B2
0043401 // st	0005654 // nu	0005515 // pr	Estrogen-relat	ERR3_HUMAN|ESRRG|ERR3|ERRG2|KIAA0832|NR3B3
0006687 // gl	0031227 // in	0008289 // lip	Extended syna	ESYT3_HUMAN|ESYT3|FAM62C
0006449 // re	0005829 // cy	0003723 // RN	Eukaryotic pe	ERF1_HUMAN|ETF1|ERF1|RF1|SUP45L1
0006479 // pr	0005759 // m	0009055 // el	Electron trans	ETFB_HUMAN|ETFB|FP585
0006646 // ph	0005829 // cy	0004305 // et	Ethanolamine	EKI1_HUMAN|ETNK1|EKI1
0006646 // ph	0005759 // m	0050459 // et	Ethanolamine	AT2L1_HUMAN|ETNPPL|AGXT2L1
0008285 // ne	0005654 // nu	0001077 // tra	Protein C-ets-	ETS1_HUMAN|ETS1|EWSR2
0001712 // ec	0005654 // nu	0000978 // RN	Protein C-ets-	ETS2_HUMAN|ETS2
0048935 // pe	0005634 // nu	0005515 // pr	ETS translocat	ETV1_HUMAN|ETV1|ER81
0016055 // W	0005634 // nu	0001077 // tra	ETS translocat	ETV2_HUMAN|ETV2|ER71|ETSRP71
0008285 // ne	0005634 // nu	0017151 // DE	ETS translocat	ETV3_HUMAN|ETV3|METS|PE1
0045944 // pc	0005654 // nu	0001077 // tra	ETS translocat	ETV4_HUMAN|ETV4|E1AF|PEA3
0071340 // sk	0005886 // pl	0000977 // RN	ETS translocat	ETV5_HUMAN|ETV5|ERM
0000122 // ne	0005730 // nu	0003700 // tra	Transcription	ETV6_HUMAN|ETV6|TEL|TEL1
0000122 // ne	0005634 // nu	0005515 // pr	Transcription	ETV7_HUMAN|ETV7|TEL2|TELB|TREF
0006915 // ap	0005765 // lys		Protein eva-1	EVA1A_HUMAN|EVA1A|FAM176A|TMEM166|SP24
0008150 // bi	0016021 // in	0008201 // he	Protein eva-1	EVA1C_HUMAN|EVA1C|C21orf63|C21orf64|FAM176C|
0051216 // ca	0005737 // cy		Ellis-van Creve	EVC_HUMAN|EVC
0030854 // pc	0005887 // in		Protein EVI2B	EVI2B_HUMAN|EVI2B|EVDB
0008283 // ce	0005829 // cy	0005515 // pr	Ecotropic vira	EVI5_HUMAN|EVI5|NB4S
0007166 // ce	0005856 // cy	0017124 // SH	Ena/VASP-like	EVL_HUMAN|EVL|RNB6
0018149 // pe	0005829 // cy	0045296 // ca	Envoplakin	EVPL_HUMAN|EVPL
0006298 // m	0005654 // nu	0051908 // dc	Exonuclease 1	EXO1_HUMAN|EXO1|EXOI|HEX1

0006893 // Go	0005829 // cy	0005515 // pr	Exocyst comp	EXOC1_HUMAN|EXOC1|SEC3|SEC3L1|BM-012
0051601 // ex	0005829 // cy	0045296 // ca	Exocyst comp	EXOC3_HUMAN|EXOC3|SEC6|SEC6L1
	0005576 // ex	0005515 // pr	Uncharacteriz	EXAS1_HUMAN|EXOC3-AS1|C5orf55
0006893 // Go	0005829 // cy	0005515 // pr	Exocyst comp	EXOC5_HUMAN|EXOC5|SEC10|SEC10L1
0006904 // ve	0005829 // cy	0005515 // pr	Exocyst comp	EXOC6_HUMAN|EXOC6|SEC15A|SEC15L|SEC15L1
0071035 // nu	0005829 // cy	0005515 // pr	Exosome com	EXOS3_HUMAN|EXOSC3|RRP40|CGI-102
0071044 // hi	0000176 // nu	0005515 // pr	Exosome com	EXOS4_HUMAN|EXOSC4|RRP41|SKI6
0006886 // in	0005768 // er	0017137 // Ra	Exophilin-5	EXPH5_HUMAN|EXPH5|KIAA0624|SLAC2B
0030307 // po	0016021 // in	0016757 // tra	Exostosin-like	EXTL3_HUMAN|EXTL3|EXTL1L|EXTR1|KIAA0519
2001240 // ne	0005634 // nu	0046872 // m	Eyes absent h	EYA4_HUMAN|EYA4
0050908 // de	0070062 // ex	0005509 // ca	Protein eyes s	EYS_HUMAN|EYS|C6orf178|C6orf179|C6orf180|EGFL10
0050892 // in	0070062 // ex	0005515 // pr	Junctional adh	JAM1_HUMAN|F11R|JAM1|JCAM|UNQ264/PRO301
0002353 // pl	0070062 // ex	0004252 // se	Coagulation fa	FA12_HUMAN|F12
0002576 // pl	0005576 // ex	0003810 // pr	Coagulation fa	F13A_HUMAN|F13A1|F13A
0051930 // re	0005886 // pl	0004930 // G-	Proteinase-ac	PAR1_HUMAN|F2R|CF2R|PAR1|TR
0009611 // re	0005886 // pl	0004930 // G-	Proteinase-ac	PAR3_HUMAN|F2RL2|PAR3
0051897 // po	0016021 // in	0005515 // pr	Tissue factor	TF_HUMAN|F3
0002576 // pl	0033116 // er	0005515 // pr	Coagulation fa	FA5_HUMAN|F5
0008150 // bi	0005634 // nu	0003674 // m	Factor VIII intr	F8I2_HUMAN|F8A1|F8A2|F8A3|F8A|F8A|F8A
0055114 // ox	0016021 // in	0080132 // fa	Fatty acid 2-h	FA2H_HUMAN|FA2H|FAAH
0019369 // ar	0016021 // in	0005515 // pr	Fatty-acid am	FAAH1_HUMAN|FAAH|FAAH1
0019369 // ar	0016021 // in	0017064 // fa	Fatty-acid am	FAAH2_HUMAN|FAAH2|AMDD
0036297 // in	0005654 // nu	0005515 // pr	Fanconi anem	FP100_HUMAN|FAAP100|C17orf70
0006974 // ce	0005694 // ch	0070530 // K6	Fanconi anem	FAP20_HUMAN|FAAP20|C1orf86|FP7162
0036297 // in	0005654 // nu	0003677 // DI	Fanconi anem	FAP24_HUMAN|FAAP24|C19orf40
0008285 // ne	0016528 // sa	0008092 // cy	Fatty acid-bin	FABPH_HUMAN|FABP3|FABP11|MDGI
0050872 // wl	0005737 // cy	0005504 // fa	Fatty acid-bin	FABP4_HUMAN|FABP4
0006629 // lip	0005737 // cy	0005215 // tra	Fatty acid-bin	FABP5_HUMAN|FABP5
0006629 // lip	0016020 // m	0005215 // tra	Gastrotropin	FABP6_HUMAN|FABP6|ILBP|ILLBP
0055114 // ox	0005789 // er	0045485 // or	Fatty acid des	FADS1_HUMAN|FADS1|FADSD5
0055114 // ox	0005789 // er	0004768 // st	Fatty acid des	FADS2_HUMAN|FADS2
0055114 // ox	0016021 // in	0003674 // m	Fatty acid des	FADS3_HUMAN|FADS3|CYB5RP
0008152 // m	0005739 // m	0018773 // ac	Acylpyruvase	FAHD1_HUMAN|FAHD1|C16orf36|YISKL
0008152 // m		0016787 // hy	Fumarylaceto	FAH2B_HUMAN|FAHD2B
0006915 // ap	0005737 // cy	0005515 // pr	Fas apoptotic	FAIM1_HUMAN|FAIM|FAIM1
		0005515 // pr	Protein FAM1	F104A_HUMAN|FAM104A
0007032 // en	0005829 // cy	0005515 // pr	Sesquipedalia	SESQ1_HUMAN|FAM109A
0007032 // en	0005829 // cy	0005515 // pr	Sesquipedalia	SESQ2_HUMAN|FAM109B

	0000922 // sp	0005515 // pr	Protein FAM1	F110A_HUMAN|FAM110A|C20orf55|F10	
0060491 // re	0000922 // sp	0005515 // pr	Protein FAM1	F110C_HUMAN|FAM110C	
0045071 // ne	0005634 // nu	0005515 // pr	Protein FAM1	F111A_HUMAN|FAM111A|KIAA1895	
		0005515 // pr	Protein FAM1	F111B_HUMAN|FAM111B|CANP	
	0005794 // Go	0005515 // pr	Protein NOXP	NXP20_HUMAN|FAM114A1|NOXP20	
		0005515 // pr	Protein FAM1	F117B_HUMAN|FAM117B|ALS2CR13	
	0016021 // in	0005515 // pr	Protein FAM1	F118A_HUMAN|FAM118A|C22orf8	
0030576 // Ca	0015030 // Ca	0005515 // pr	Protein FAM1	F118B_HUMAN|FAM118B	
		0005515 // pr	Protein FAM1	F124A_HUMAN|FAM124A	
0001934 // po	0070062 // ex	0005515 // pr	Protein Niban	NIBAN_HUMAN|FAM129A|C1orf24|NIBAN|GIG39	
	0005829 // cy	0005515 // pr	Protein FAM1	F131B_HUMAN|FAM131B|KIAA0773	
		0005515 // pr	Protein FAM1	F131C_HUMAN|FAM131C|C1orf117	
		0005515 // pr	Protein FAM1	F133A_HUMAN|FAM133A	
0044255 // ce		0052689 // ca	Protein FAM1	F135A_HUMAN|FAM135A|KIAA1411	
	0005737 // cy		Protein FAM1	F136A_HUMAN|FAM136A	
0043547 // po	0005829 // cy	0005096 // GT	Protein FAM1	FA13A_HUMAN|FAM13A|FAM13A1|KIAA0914	
0043547 // po	0005829 // cy	0005096 // GT	Protein FAM1	FA13B_HUMAN|FAM13B|C5orf5|FAM13B1	
0006629 // lip		0008081 // ph	Protein FAM1	F151B_HUMAN|FAM151B|UNQ9217/PRO28685	
	0016021 // in	0005515 // pr	Protein FAM1	FA156_HUMAN|FAM156A|FAM156B|TMEM29|TMEM2	
	0016021 // in		Membrane pr	F159B_HUMAN|FAM159B	
0050896 // re	0005737 // cy	0005515 // pr	Protein FAM1	F161A_HUMAN|FAM161A	
	0016021 // in		Protein FAM1	F162B_HUMAN|FAM162B|C6orf189	
	0005783 // er	0005515 // pr	Protein FAM1	F167A_HUMAN|FAM167A|C8orf13	
	0005637 // nu		Soluble lamin-	F169A_HUMAN|FAM169A|KIAA0888	
	0016021 // in		Protein FAM1	F1711_HUMAN|FAM171A1|C10orf38	
	0016021 // in		Protein FAM1	F1712_HUMAN|FAM171A2	
	0016021 // in		Protein FAM1	F173A_HUMAN|FAM173A|C16orf24|RJD7	
	0016021 // in		Protein FAM1	F173B_HUMAN|FAM173B	
	0016021 // in		Membrane pr	F174B_HUMAN|FAM174B	
	0016021 // in		Protein FAM1	F180B_HUMAN|FAM180B	

0008150 // bi	0005615 // ex	0003674 // m	Protein FAM1	F184A_HUMAN|FAM184A|C6orf60
	0043234 // pr		Protein FAM1	F186B_HUMAN|FAM186B|C12orf25
	0016021 // in		Ig-like V-type	F187A_HUMAN|FAM187A
	0016021 // in		Protein FAM1	F198B_HUMAN|FAM198B|C4orf18|ENED|AD021|UNQ2
	0005737 // cy		Protein FAM1	F19A2_HUMAN|FAM19A2|TAFA2
		0005515 // pr	Protein FAM2	F207A_HUMAN|FAM207A|C21orf70|PRED56
0016310 // ph	0005794 // Go	0005515 // pr	Glycosaminog	XYLK_HUMAN|FAM20B|KIAA0475
0040036 // re	0005615 // ex	0005515 // pr	Extracellular s	FA20C_HUMAN|FAM20C|DMP4
0045648 // po	0016021 // in		Protein FAM2	F210B_HUMAN|FAM210B|C20orf108|PSEC0265
0021915 // ne	0070062 // ex	0019901 // pr	PAK4-inhibito	INKA1_HUMAN|FAM212A|C3orf54|INKA1
0098869 // ce	0005737 // cy	0016209 // an	Redox-regulat	F213A_HUMAN|FAM213A|C10orf58|PAMM|PRO2290|
0098869 // ce	0070062 // ex	0016616 // ox	Prostamide/p	PGFS_HUMAN|FAM213B|C1orf93
		0005515 // pr	Protein FAM2	F214A_HUMAN|FAM214A|KIAA1370
	0005634 // nu	0005515 // pr	Protein FAM2	F214B_HUMAN|FAM214B|KIAA1539
	0005829 // cy		Protein FAM2	F217B_HUMAN|FAM217B|C20orf177
		0005515 // pr	Protein FAM2	F218A_HUMAN|FAM218A|C4orf39
0008150 // bi	0016021 // in	0003674 // m	Protein FAM2	F234A_HUMAN|FAM234A|C16orf9|ITFG3
	0005576 // ex		Protein FAM2	FA24B_HUMAN|FAM24B
0034220 // io	0005887 // in	0005261 // ca	Protein FAM2	FA26F_HUMAN|FAM26F|C6orf187
		0005515 // pr	Protein FAM2	F27E3_HUMAN|FAM27E3
0007049 // ce	0005730 // nu	0003723 // RN	Protein FAM3	FA32A_HUMAN|FAM32A|OTAG12|CGI-144
0002576 // pl	0005576 // ex	0005515 // pr	Protein FAM3	FAM3C_HUMAN|FAM3C|ILEI|GS3786

0030193 // re		0003723 // RI	Protein FAM4	FA46A_HUMAN|FAM46A|C6orf37|XTP11
		0005515 // pr	Protein FAM4	FA46B_HUMAN|FAM46B
		0005515 // pr	Protein FAM4	FA46C_HUMAN|FAM46C
0008150 // bi	0005737 // cy	0003674 // m	Protein FAM4	FA47E_HUMAN|FAM47E
	0005622 // in		Protein FAM4	FA49A_HUMAN|FAM49A
0007283 // sp	0005634 // nu	0003723 // RI	Protein FAM5	FA50A_HUMAN|FAM50A|DXS9928E|HXC26|XAP5
	0045171 // in	0005515 // pr	Protein FAM5	FA50B_HUMAN|FAM50B|X5L
	0005634 // nu		Protein FAM5	FA53A_HUMAN|FAM53A
0046513 // ce	0016021 // in	0050291 // sp	Protein FAM5	FA57B_HUMAN|FAM57B|FP1188
0045737 // po	0000307 // cy	0016538 // cy	Cyclin-related	FA58A_HUMAN|FAM58A
	0016021 // in		Protein FAM6	FA69A_HUMAN|FAM69A
	0016021 // in	0005515 // pr	Protein FAM6	FA69B_HUMAN|FAM69B|C9orf136|PP6977
	0005634 // nu		Protein FAM7	F71F2_HUMAN|FAM71F2|FAM137B
	0005829 // cy	0005515 // pr	Protein FAM7	FA72A_HUMAN|FAM72A|UGENE
	0005829 // cy		Protein FAM7	FA72B_HUMAN|FAM72B
	0005829 // cy		Protein FAM7	FA72C_HUMAN|FAM72C
	0005829 // cy		Protein FAM7	FA72D_HUMAN|FAM72D|GCUD2
0007173 // ep	0005737 // cy	0005515 // pr	Protein FAM8	FA83A_HUMAN|FAM83A|TSGP
0007173 // ep	0016020 // m	0019901 // pr	Protein FAM8	FA83B_HUMAN|FAM83B|C6orf143
		0019901 // pr	Protein FAM8	FA83C_HUMAN|FAM83C|C20orf128
		0019901 // pr	Protein FAM8	FA83E_HUMAN|FAM83E
0044380 // pr	0005737 // cy	0019901 // pr	Protein FAM8	FA83H_HUMAN|FAM83H
	0005737 // cy	0005515 // pr	Protein FAM8	FA84B_HUMAN|FAM84B|BCMP101|NSE2
0032259 // m		0008168 // m	Putative prote	F86B1_HUMAN|FAM86B1
0032259 // m	0043234 // pr	0008168 // m	Putative prote	F86B2_HUMAN|FAM86B2
0032259 // m		0008168 // m	Putative prote	F86C2_HUMAN|FAM86C2P

0032418 // lys	0072669 // tR	0003723 // RN	Protein FAM9	FA98A_HUMAN|FAM98A
0051321 // m	0000800 // lat	0005515 // pr	Protein FAM9	FAM9B_HUMAN|FAM9B
0001541 // ov	0005654 // nu	0005515 // pr	Fanconi anem	FANCF_HUMAN|FANCF
0007005 // m	0005654 // nu	0005515 // pr	Fanconi anem	FANCG_HUMAN|FANCG|XRCC9
0006513 // pr	0005654 // nu	0005515 // pr	E3 ubiquitin-p	FANCL_HUMAN|FANCL|PHF9
0043066 // ne	0005634 // nu	0005515 // pr	Fibronectin ty	FANK1_HUMAN|FANK1|HSD13|UNQ6504/PRO21382
0006432 // ph	0005737 // cy	0003723 // RN	Phenylalanine	SYFA_HUMAN|FARSA|FARS|FARSL|FARSLA
0006432 // ph	0005829 // cy	0005524 // AT	Phenylalanine	SYFB_HUMAN|FARSB|FARSLB|FRSB|HSPC173
0043065 // po	0005886 // pl	0005515 // pr	Tumor necros	TNR6_HUMAN|FAS|APT1|FAS1|TNFRSF6
0006633 // fa	0005794 // Go	0003723 // RN	Fatty acid syn	FAS_HUMAN|FASN|FAS
0007163 // es	0005911 // ce	0005509 // ca	Protocadherin	FAT1_HUMAN|FAT1|CDHF7|FAT
0007156 // ho	0016021 // in	0005509 // ca	Protocadherin	FAT2_HUMAN|FAT2|CDHF8|KIAA0811|MEGF1
0035329 // hi	0005886 // pl	0005509 // ca	Protocadherin	FAT4_HUMAN|FAT4|CDHF14|FATJ|Nbla00548
	0016021 // in		Failed axon co	FAXC_HUMAN|FAXC|C6orf168
0008360 // re	0005938 // ce	0031005 // fil	Filamin-bindin	FBLI1_HUMAN|FBLIM1|FBLP1
0001933 // ne	0005576 // ex	0008022 // pr	Fibulin-1	FBLN1_HUMAN|FBLN1|PP213
0010811 // po	1903561 // ex	0005201 // ex	Fibulin-2	FBLN2_HUMAN|FBLN2
0007155 // ce	0070062 // ex	0005509 // ca	Fibulin-7	FBLN7_HUMAN|FBLN7|TM14
0001656 // m	0001527 // m	0005515 // pr	Fibrillin-1	FBN1_HUMAN|FBN1|FBN
0060346 // bo	0005576 // ex	0005515 // pr	Fibrillin-2	FBN2_HUMAN|FBN2
0006094 // glu	0005829 // cy	0005515 // pr	Fructose-1,6-b	F16P2_HUMAN|FBP2
0000209 // pr	0005829 // cy	0004842 // ub	F-box/LRR-rep	FXL13_HUMAN|FBXL13|FBL13
0000209 // pr	0005829 // cy	0004842 // ub	F-box/LRR-rep	FXL14_HUMAN|FBXL14|FBL14
0000086 // G2	0005829 // cy	0004842 // ub	F-box/LRR-rep	FXL15_HUMAN|FBXL15|FBXO37
0000209 // pr	0005829 // cy	0004842 // ub	F-box/LRR-rep	FXL16_HUMAN|FBXL16|C16orf22|FBL16
0000209 // pr	0005829 // cy	0004842 // ub	F-box and leu	FXL22_HUMAN|FBXL22
0016567 // pr	0005813 // ce	0004842 // ub	F-box/LRR-rep	FBXL7_HUMAN|FBXL7|FBL6|FBL7|KIAA0840
0042981 // re	0005829 // cy	0004842 // ub	F-box only pro	FBX10_HUMAN|FBXO10|FBX10|PRMT11
0043161 // pr	0005829 // cy	0004842 // ub	F-box only pro	FBX15_HUMAN|FBXO15|FBX15
0000209 // pr	0005829 // cy	0004842 // ub	F-box only pro	FBX17_HUMAN|FBXO17|FBG4|FBX17|FBX26|FBXO26
0016567 // pr	0005829 // cy	0004842 // ub	F-box only pro	FBX2_HUMAN|FBXO2|FBX2
0016567 // pr	0000151 // ub	0004842 // ub	F-box only pro	FBX24_HUMAN|FBXO24|FBX24
0000209 // pr	0005829 // cy	0004842 // ub	F-box only pro	FBX27_HUMAN|FBXO27|FBG5|FBX27
0000209 // pr	0005829 // cy	0004842 // ub	F-box only pro	FBX30_HUMAN|FBXO30|FBX30
0014894 // re	0005829 // cy	0004842 // ub	F-box only pro	FBX32_HUMAN|FBXO32
0016567 // pr		0005515 // pr	F-box only pro	FBX33_HUMAN|FBXO33|FBX33
0000209 // pr	0005829 // cy	0005515 // pr	F-box only pro	FBX4_HUMAN|FBXO4|FBX4
0000209 // pr	0005829 // cy	0004842 // ub	F-box only pro	FBX41_HUMAN|FBXO41|FBX41|KIAA1940
0051436 // ne	0005634 // nu	0046872 // m	F-box only pro	FBX43_HUMAN|FBXO43|EMI2
0010498 // pr	0005829 // cy	0004842 // ub	F-box only pro	FBX44_HUMAN|FBXO44|FBG3|FBX30|FBX44|FBX6A|FB
0046785 // m	0005654 // nu	0005515 // pr	F-box only pro	FBX5_HUMAN|FBXO5|EMI1|FBX5
0000077 // DI	0005829 // cy	0004842 // ub	F-box only pro	FBX6_HUMAN|FBXO6|FBG2|FBS2|FBX6

0010975 // re	0005829 // cy	0005515 // pr	F-box only pro	FBX7_HUMAN|FBXO7|FBX7
0032012 // re	0000151 // ub	0005086 // AR	F-box only pro	FBX8_HUMAN|FBXO8|FBS|FBX8|DC10|UNQ1877/PRO4
0043161 // pr	0005829 // cy	0005515 // pr	F-box/WD rep	FBW1B_HUMAN|FBXW11|BTRCP2|FBW1B|FBXW1B|KIA
0000209 // pr	0005829 // cy	0004842 // ub	F-box/WD rep	FBW12_HUMAN|FBXW12|FBW12|FBXO12|FBXO35
0000447 // er	0005654 // nu	0003723 // RN	rRNA-process	FCF1_HUMAN|FCF1|C14orf111|CGI-35
0050776 // re	0005886 // pl	0019864 // Ig	High affinity in	FCGR1_HUMAN|FCGR1A|FCG1|FCGR1|IGFR1
0002250 // ac	0005886 // pl	0019864 // Ig	High affinity in	FCGRB_HUMAN|FCGR1B|IGFRB
0038096 // Fc	0005886 // pl	0019864 // Ig	Low affinity in	FCG2A_HUMAN|FCGR2A|CD32|FCG2|FCGR2A1|IGFR2
0006955 // im	0005886 // pl	0019864 // Ig	Low affinity in	FCG2C_HUMAN|FCGR2C|CD32|FCG2|IGFR2
0038094 // Fc	0016021 // in	0042605 // pe	IgG receptor F	FCGRN_HUMAN|FCGRT|FCRN
0061024 // m	0005829 // cy	0005515 // pr	F-BAR domain	FCHO1_HUMAN|FCHO1|KIAA0290
0007274 // ne	0055037 // re		F-BAR and do	FCSD1_HUMAN|FCHSD1|UNQ737/PRO1431
0002376 // im	0016021 // in		Fas apoptotic	FAIM3_HUMAN|FCMR|FAIM3|TOSO
0006508 // pr	0005576 // ex	0005515 // pr	Ficolin-3	FCN3_HUMAN|FCN3|FCNH|HAKA1
0006696 // er	0016021 // in	0005515 // pr	Squalene synt	FDFT_HUMAN|FDFT1
0006700 // C2	0005739 // m	0009055 // el	Adrenodoxin,	ADX_HUMAN|FDX1|ADX
0006700 // C2	0005759 // m	0051537 // 2	Ferredoxin-2,	FDX2_HUMAN|FDX2|FDX1L
0006432 // ph	0009328 // ph	0004826 // ph	Ferredoxin-fo	FDXA1_HUMAN|FDXACB1
0046685 // re	0005739 // m	0005515 // pr	Ferrochelatas	HEMH_HUMAN|FECH
0016567 // pr	0005829 // cy	0004842 // ub	Protein fem-1	FEM1A_HUMAN|FEM1A|EPRAP
0016567 // pr	0005829 // cy	0005515 // pr	Protein fem-1	FEM1C_HUMAN|FEM1C|KIAA1785
0038083 // pe	0005938 // ce	0005515 // pr	Tyrosine-prot	FER_HUMAN|FER|TYK3
	0016021 // in		Fer-1-like prot	FR1L4_HUMAN|FER1L4|C20orf124
	0016021 // in		Fer-1-like prot	FR1L6_HUMAN|FER1L6|C8orfK23
0001954 // po	0005829 // cy		Fermitin famil	FERM1_HUMAN|FERMT1|C20orf42|KIND1|URP1
0072657 // pr	0005938 // ce	0005515 // pr	Fermitin famil	FERM2_HUMAN|FERMT2|KIND2|MIG2|PLEKHC1
0002576 // pl	0005576 // ex	0005178 // in	Fermitin famil	URP2_HUMAN|FERMT3|KIND3|MIG2B|URP2
0038083 // pe	0031234 // ex	0005515 // pr	Tyrosine-prot	FES_HUMAN|FES|FPS
0010976 // po	0030425 // de	0043015 // ga	Fasciculation a	FEZ1_HUMAN|FEZ1
1902902 // ne	0005737 // cy	0005515 // pr	Fasciculation a	FEZ2_HUMAN|FEZ2
0000122 // ne	0005829 // cy	0001078 // tra	Fez family zinc	FEZF1_HUMAN|FEZF1|FEZ|ZNF312B
0035023 // re	0005737 // cy	0005089 // Rh	FYVE, RhoGEF	FGD1_HUMAN|FGD1|FGDY|ZFYVE3
0046847 // fil	0005737 // cy	0005515 // pr	FYVE, RhoGEF	FGD2_HUMAN|FGD2|ZFYVE4
0046847 // fil	0005737 // cy	0005089 // Rh	FYVE, RhoGEF	FGD3_HUMAN|FGD3|ZFYVE5
0046847 // fil	0030175 // fil	0005089 // Rh	FYVE, RhoGEF	FGD4_HUMAN|FGD4|FRABP|ZFYVE6
0046847 // fil	0005737 // cy	0005089 // Rh	FYVE, RhoGEF	FGD5_HUMAN|FGD5|ZFYVE23
0007165 // sig	0005576 // ex	0008083 // gr	Fibroblast gro	FGF11_HUMAN|FGF11|FHF3
0008344 // ad	0005615 // ex	0008083 // gr	Fibroblast gro	FGF12_HUMAN|FGF12|FGF12B|FHF1
0006814 // so	0016328 // la	0005515 // pr	Fibroblast gro	FGF13_HUMAN|FGF13|FHF2
1903421 // re	0005576 // ex	0005515 // pr	Fibroblast gro	FGF14_HUMAN|FGF14|FHF4
0001936 // re	0005576 // ex	0008083 // gr	Fibroblast gro	FGF18_HUMAN|FGF18|UNQ420/PRO856
0038111 // in	0005622 // in	0008083 // gr	Fibroblast gro	FGF19_HUMAN|FGF19|UNQ334/PRO533
0008543 // fib	0005576 // ex	0005104 // fib	Fibroblast gro	FGF2_HUMAN|FGF2|FGFB
0038111 // in	0005576 // ex	0043395 // he	Fibroblast gro	FGF20_HUMAN|FGF20
0038111 // in	0005576 // ex	0008083 // gr	Fibroblast gro	FGF5_HUMAN|FGF5

0008078 // m	0005576 // ex	0008083 // gr	Fibroblast gro	FGF8_HUMAN|FGF8|AIGF
0007165 // si	0005615 // ex	0005515 // pr	Fibroblast gro	FGFP1_HUMAN|FGFBP1|FGFBP|HBP17
0045743 // po	0031012 // ex	0017134 // fib	Fibroblast gro	FGFP3_HUMAN|FGFBP3|C10orf13|PSEC0101
0018108 // pe	0005829 // cy	0004713 // pr	FGFR1 oncoge	FGOP2_HUMAN|FGFR1OP2|HSPC123
0060615 // m	0005886 // pl	0005007 // fib	Fibroblast gro	FGFR2_HUMAN|FGFR2|BEK|KGFR|KSAM
0007267 // ce	0005886 // pl	0005007 // fib	Fibroblast gro	FGFR3_HUMAN|FGFR3|JTK4
0038111 // in	0005886 // pl	0017134 // fib	Fibroblast gro	FGFR4_HUMAN|FGFR4|JTK2|TKF
0038083 // pe	0016235 // ag	0034987 // im	Tyrosine-prot	FGR_HUMAN|FGR|SRC2
0072332 // in	0070062 // ex	0042802 // id	Bis(5'-adenosy	FHIT_HUMAN|FHIT
1901016 // re	0005829 // cy	0005515 // pr	Four and a ha	FHL1_HUMAN|FHL1|SLIM1
0055003 // ca	0030018 // Z	0003779 // ac	FH1/FH2 dom	FHOD3_HUMAN|FHOD3|FHOS2|KIAA1695
	0016021 // in	0005515 // pr	Fibrinogen C	FBCD1_HUMAN|FIBCD1|UNQ701/PRO1346
0010042 // re	0005576 // ex	0003674 // m	Fin bud initiat	FIBIN_HUMAN|FIBIN|PSEC0235
0051301 // ce	0016363 // nu	0016887 // AT	Fidgetin	FIGN_HUMAN|FIGN
	0005737 // cy		Filamin-A-inte	FLIP1_HUMAN|FILIP1|KIAA1275
0034389 // lip	0005789 // er	0003674 // m	Fat storage-in	FITM1_HUMAN|FITM1|FIT1
0030730 // se	0005789 // er	0003674 // m	Fat storage-in	FITM2_HUMAN|FITM2|C20orf142|FIT2
0010842 // re	0005615 // ex		Four-jointed b	FJX1_HUMAN|FJX1
0000413 // pr	0005737 // cy	0005528 // FK	Peptidyl-proly	FKB11_HUMAN|FKBP11|FKBP19|UNQ336/PRO535
0000413 // pr	0005737 // cy	0005528 // FK	Peptidyl-proly	FKB14_HUMAN|FKBP14|FKBP22|UNQ322/PRO381
0000413 // pr	0070062 // ex	0005515 // pr	Peptidyl-proly	FKBP2_HUMAN|FKBP2|FKBP13
0000413 // pr	0005737 // cy	0003723 // RN	Peptidyl-proly	FKBP3_HUMAN|FKBP3|FKBP25
0031115 // ne	0005737 // cy	0003723 // RN	Peptidyl-proly	FKBP4_HUMAN|FKBP4|FKBP52
0006457 // pr	0005737 // cy	0005515 // pr	Peptidyl-proly	FKBP5_HUMAN|FKBP5|AIG6|FKBP51
0034587 // pi	0005737 // cy	0005515 // pr	Inactive pepti	FKBP6_HUMAN|FKBP6|FKBP36
0000413 // pr	0005737 // cy	0005528 // FK	Peptidyl-proly	FKBP7_HUMAN|FKBP7|FKBP23|UNQ670/PRO1304
0000413 // pr	0005783 // er	0005528 // FK	Peptidyl-proly	FKBP9_HUMAN|FKBP9|FKBP60|FKBP63
		0005509 // ca	Putative FK50	FKB9L_HUMAN|FKBP9P1|FKBP9L
0009314 // re	0005829 // cy	0005515 // pr	FK506-binding	FKBPL_HUMAN|FKBPL|DIR1|NG7
0007517 // m	0005783 // er	0016740 // tra	Fukutin	FKTN_HUMAN|FKTN|FCMD
0098773 // sk	0005829 // cy	0005515 // pr	Filaggrin	FILA_HUMAN|FLG
0061436 // es	0005576 // ex	0005198 // st	Filaggrin-2	FILA2_HUMAN|FLG2|IFPS
0045893 // po	0016604 // nu	0000978 // RN	Friend leukem	FLI1_HUMAN|FLI1
1905000 // re	0030018 // Z	0003723 // RN	Filamin-A	FLNA_HUMAN|FLNA|FLN|FLN1

0048747 // m	0030018 // Z	0008092 // cy	Filamin-C	FLNC_HUMAN|FLNC|ABPL|FLN2
0035023 // re	0045121 // m	0002020 // pr	Flotillin-1	FLOT1_HUMAN|FLOT1
1903905 // pc	0030027 // la	0046982 // pr	Flotillin-2	FLOT2_HUMAN|FLOT2|ESA1|M17S1
0007411 // ax	0005737 // cy	0004860 // pr	Leucine-rich r	FLRT2_HUMAN|FLRT2|KIAA0405|UNQ232/PRO265
0043406 // pc	0043235 // re	0005515 // pr	Vascular endc	VGFR1_HUMAN|FLT1|FLT|FRT|VEGFR1
0071864 // pc	0031233 // in	0030971 // re	Fms-related ty	FLT3L_HUMAN|FLT3LG
0001568 // bl	0005886 // pl	0015232 // he	Feline leukem	FLVC1_HUMAN|FLVCR1|FLVCR
	0005634 // nu	0003677 // DI	FLYWCH-type	FWCH1_HUMAN|FLYWCH1|KIAA1552
0008150 // bi	0005575 // ce	0003723 // RI	FLYWCH famil	FWCH2_HUMAN|FLYWCH2
0048705 // sk	0015630 // m	0017124 // SH	Formin-1	FMN1_HUMAN|FMN1|FMN|LD
0006929 // su	0005829 // cy	0005522 // pr	Formin-like pr	FMNL1_HUMAN|FMNL1|C17orf1|C17orf1B|FMNL|FRL1
0030036 // ac	0005794 // Go	0003779 // ac	Formin-like pr	FMNL3_HUMAN|FMNL3|FHOD3|FRL2|KIAA2014|WBP3
0055114 // ox	0016021 // in	0004499 // N,	Dimethylanilir	FMO4_HUMAN|FMO4|FMO2
0008150 // bi	0016021 // in	0004499 // N,	Dimethylanilir	FMO5_HUMAN|FMO5
0007181 // tra	0005576 // ex		Fibromodulin	FMOD_HUMAN|FMOD|FM|SLRR2E
0018149 // pe	0005576 // ex	0005515 // pr	Fibronectin	FINC_HUMAN|FN1|FN
0030389 // fru	0005829 // cy	0030387 // fru	Fructosamine	FN3K_HUMAN|FN3K
0043687 // pc	0005829 // cy	0016301 // kir	Ketosamine-3	KT3K_HUMAN|FN3KRP
0006897 // er	0005938 // ce	0008289 // lip	Formin-bindir	FNBP1_HUMAN|FNBP1|FBP17|KIAA0554
0097320 // m	0005938 // ce	0005515 // pr	Formin-bindir	FBP1L_HUMAN|FNBP1L|C1orf39|TOCA1
	0016021 // in		Fibronectin ty	FND10_HUMAN|FNDC10|C1orf233
	0070062 // ex	0005515 // pr	Fibronectin ty	FND11_HUMAN|FNDC11|C20orf195
0060009 // Se	0016021 // in	0003723 // RI	Fibronectin ty	FND3A_HUMAN|FNDC3A|FNDC3|HUGO|KIAA0970
0071559 // re	0005615 // ex		Fibronectin ty	FNDC4_HUMAN|FNDC4|FRCP1|UNQ6389/PRO21134
	0005576 // ex		Fibronectin ty	FNDC7_HUMAN|FNDC7
	0005634 // nu		Fibronectin ty	FNDC8_HUMAN|FNDC8
0099601 // re	0005737 // cy	0005515 // pr	Protein farnes	FNTA_HUMAN|FNTA
0048146 // pc	0005829 // cy	0004311 // fa	Protein farnes	FNTB_HUMAN|FNTB
0006508 // pr	0070062 // ex	0016805 // di	Putative N-ac	FOH1B_HUMAN|FOLH1B|PSMAL|GIG26
0061713 // ar	0031526 // br	0005542 // fo	Folate recepto	FOLR1_HUMAN|FOLR1|FOLR
0015884 // fo	0005576 // ex	0005542 // fo	Folate recepto	FOLR3_HUMAN|FOLR3
0008219 // ce	0005634 // nu	0005515 // pr	Fos-related ar	FOSL2_HUMAN|FOSL2|FRA2
1902691 // re	0001650 // fib	0000981 // RI	Hepatocyte nu	FOXA1_HUMAN|FOXA1|HNF3A|TCF3A
0009725 // re	0016604 // nu	0005515 // pr	Forkhead box	FOXC2_HUMAN|FOXC2|FKHL14|MFH1
0030154 // ce	0005634 // nu	0008301 // DI	Forkhead box	FOXD1_HUMAN|FOXD1|FKHL8|FREAC4
0030154 // ce	0005634 // nu	0000981 // RI	Forkhead box	FX4L4_HUMAN|FOXD4L4|FOXD4B|FOXD4L2
0030154 // ce	0005634 // nu	0000981 // RI	Forkhead box	FX4L6_HUMAN|FOXD4L6
0060023 // so	0005634 // nu	0043565 // se	Forkhead box	FOXE1_HUMAN|FOXE1|FKHL15|FOXE2|TITF2|TTF2
0030154 // ce	0005667 // tra	0043565 // se	Forkhead box	FOXF2_HUMAN|FOXF2|FKHL6|FREAC2
0007346 // re	0005634 // nu	0000981 // RI	Forkhead box	FOXG1_HUMAN|FOXG1|FKH2|FKHL1|FKHL2|FKHL3|FKH
0030154 // ce	0001650 // fib	0005515 // pr	Forkhead box	FOXJ2_HUMAN|FOXJ2|FHX

0007275 // m	0005634 // nu	0043565 // se	Forkhead box	FOXL1_HUMAN|FOXL1|FKHL11|FREAC7
	0001650 // fib		FOXL2 neighb	FOXNB_HUMAN|FOXL2NB|C3orf72
0000086 // G2	0005654 // nu	0005515 // pr	Forkhead box	FOXM1_HUMAN|FOXM1|FKHL16|HFH11|MPP2|WIN
0097536 // th	0005634 // nu	0000981 // RN	Forkhead box	FOXN1_HUMAN|FOXN1|RONU|WHN
0030154 // ce	0043231 // int	0005515 // pr	Forkhead box	FOXN2_HUMAN|FOXN2|HTLF
0045892 // ne	0005634 // nu	0003700 // tra	Forkhead box	FOXN3_HUMAN|FOXN3|C14orf116|CHES1
0043525 // po	0005739 // m	0005515 // pr	Forkhead box	FOXO3_HUMAN|FOXO3|FKHRL1|FOXO3A
0045944 // po	0005634 // nu	0005515 // pr	Forkhead box	FOXO4_HUMAN|FOXO4|AFX|AFX1|MLLT7
0030154 // ce	0005737 // cy	0000981 // RN	Forkhead box	FOXO6_HUMAN|FOXO6
0035019 // so	0005634 // nu	0000981 // RN	Forkhead box	FOXP1_HUMAN|FOXP1|HSPC215
0009791 // po	0005634 // nu	0005515 // pr	Forkhead box	FOXP2_HUMAN|FOXP2|CAGH44|TNRC10
0030154 // ce	0005634 // nu	0000981 // RN	Forkhead box	FOXQ1_HUMAN|FOXQ1|HFH1
0055114 // ox	0005739 // m	0016491 // ox	FAD-depende	FXRD1_HUMAN|FOXRED1|FP634
0030433 // ER	0005788 // en	0005515 // pr	FAD-depende	FXRD2_HUMAN|FOXRED2|ERFAD
0030154 // ce	0005634 // nu	0000981 // RN	Forkhead box	FOXS1_HUMAN|FOXS1|FKHL18|FREAC10
0006004 // fu	0005737 // cy	0003824 // ca	Fucose-1-pho:	FPGT_HUMAN|FPGT|GFPP
0035556 // int	0005634 // nu	0005524 // AT	FPGT-TNNI3K	V9GXZ4_HUMAN|FPGT-TNNI3K
0007154 // ce	0031012 // ex	0046872 // m	Extracellular r	FRAS1_HUMAN|FRAS1|KIAA1500
0007154 // ce	0005604 // ba	0030246 // ca	FRAS1-relatec	FREM1_HUMAN|FREM1|C9orf143|C9orf145|C9orf154
0007507 // he	0016021 // int	0046872 // m	FRAS1-relatec	FREM2_HUMAN|FREM2
0007154 // ce	0005604 // ba	0046872 // m	FRAS1-relatec	FREM3_HUMAN|FREM3
0000398 // m	0030018 // Z (	0003723 // RN	Protein FRG1	FRG1_HUMAN|FRG1
0038083 // pe	0005829 // cy	0005515 // pr	Tyrosine-prot	FRK_HUMAN|FRK|PTK5|RAK
0090162 // es	0005737 // cy	0030674 // pr	FERM domain	FRM4A_HUMAN|FRMD4A|FRMD4|KIAA1294
0090162 // es	0005737 // cy		FERM domain	FRM4B_HUMAN|FRMD4B|GRSP1|KIAA1013
0031032 // ac	0016021 // int	0005515 // pr	FERM domain	FRMD5_HUMAN|FRMD5
0070830 // bi	0005923 // bi	0005515 // pr	FERM and PDZ	FRPD2_HUMAN|FRMPD2|PDZD5C|PDZK4|PDZK5C
0043312 // ne	0005856 // cy		FERM and PDZ	FRPD3_HUMAN|FRMPD3|KIAA1817
0055114 // ox	0016021 // int	0000293 // fe	Ferric-chelate	FRRS1_HUMAN|FRRS1|SDFR2|SDR2
0000902 // ce	0030427 // sit	0005515 // pr	Protein furry	FRYL_HUMAN|FRYL|AF4P12|KIAA0826
0008283 // ce	0030175 // fil	0003723 // RN	Fascin	FSCN1_HUMAN|FSCN1|FAN1|HSN|SNL
0030036 // ac	0015629 // ac	0003779 // ac	Fascin-2	FSCN2_HUMAN|FSCN2
0051301 // ce	0005634 // nu		Fibronectin ty	FSD1_HUMAN|FSD1|GLFND|MIR1|VLP27
0008150 // bi	0005622 // int	0003674 // m	FSD1-like prot	FSD1L_HUMAN|FSD1L|CCDC10|CSDUFD1|FSD1CL|FSD1
	0005739 // m		Fibrous sheatl	FSIP2_HUMAN|FSIP2
0042594 // re	0070062 // ex	0005515 // pr	Follistatin-rela	FSTL1_HUMAN|FSTL1|FRP
0030514 // ne	0005788 // en	0005515 // pr	Follistatin-rela	FSTL3_HUMAN|FSTL3|FLRG|UNQ674/PRO1308

	0005576 // ex	0005509 // ca	Follistatin-rela	FSTL5_HUMAN|FSTL5|KIAA1263
0035999 // te	0005783 // er	0030409 // glu	Formimidoyltı	FTCD_HUMAN|FTCD
0008152 // m		0016740 // tra	Formiminotra	FONG_HUMAN|FTCDNL1|FONG
0006879 // ce	0005829 // cy	0005515 // pr	Ferritin heavy	FRIH_HUMAN|FTH1|FTH|FTHL6|OK/SW-cl.84|PIG15
0000463 // m	0005730 // nu	0003723 // RN	pre-rRNA proc	SPB1_HUMAN|FTSJ3|SB92
0016485 // pr	0000139 // Go	0005515 // pr	Furin	FURIN_HUMAN|FURIN|FUR|PACE|PCSK3
0042355 // L-	0005794 // Go	0008417 // fu	Galactoside 2-	FUT1_HUMAN|FUT1|H|HSC
0006486 // pr	0016021 // in	0046920 // al	Alpha-(1,3)-fu	FUT11_HUMAN|FUT11
0042355 // L-	0016021 // in	0008417 // fu	Galactoside 2-	FUT2_HUMAN|FUT2|SEC2
0006672 // ce	0016020 // m	0008417 // fu	Galactoside 3	FUT3_HUMAN|FUT3|FT3B|LE
0042355 // L-	0016021 // in	0008424 // gly	Alpha-(1,6)-fu	FUT8_HUMAN|FUT8
0042355 // L-	0016021 // in	0008417 // fu	Alpha-(1,3)-fu	FUT9_HUMAN|FUT9
0010172 // er	0005856 // cy	0005515 // pr	Protein fuzzy	FUZZY_HUMAN|FUZ|FY
0070301 // ce	0005739 // m	0008199 // fe	Frataxin, mito	FRDA_HUMAN|FXN|FRDA|X25
0036376 // so	0070062 // ex	0005215 // tra	Sodium/potas	ATNG_HUMAN|FXYD2|ATP1C|ATP1G1
0006813 // po	0005886 // pl	0005254 // ch	FXYD domain-	FXYD3_HUMAN|FXYD3|MAT8|PLML
0034220 // io	0016021 // in	0005216 // io	FXYD6-FXYD2	A0A087WZ82_HUMAN|FXYD6-FXYD2
0061077 // ch	0005794 // Go	0044183 // pr	FYVE and coile	FYCO1_HUMAN|FYCO1|ZFYVE7
0038111 // in	0005829 // cy	0005515 // pr	Tyrosine-prot	FYN_HUMAN|FYN
0060070 // ca	0005886 // pl	0005109 // fri	Frizzled-1	FZD1_HUMAN|FZD1
0007416 // sy	0031901 // ea	0001540 // be	Frizzled-5	FZD5_HUMAN|FZD5|C2orf31
1904693 // m	0016021 // in	0004930 // G-	Frizzled-6	FZD6_HUMAN|FZD6
0060070 // ca	0005886 // pl	0005109 // fri	Frizzled-7	FZD7_HUMAN|FZD7
0060070 // ca	0005794 // Go	0004930 // G-	Frizzled-8	FZD8_HUMAN|FZD8
2001238 // po	0005739 // m	0003674 // m	G0/G1 switch	G0S2_HUMAN|G0S2
0007253 // cy	0005737 // cy	0003723 // RN	Ras GTPase-ac	G3BP2_HUMAN|G3BP2|KIAA0660
0006749 // glu	0005634 // nu	0004345 // glu	Glucose-6-pho	G6PD_HUMAN|G6PD
0008284 // po	0005829 // cy	0005515 // pr	GRB2-associat	GAB2_HUMAN|GAB2|KIAA0571
0008625 // ex	0005829 // cy	0005515 // pr	GABARAP-a	H6UMI1_HUMAN|GABARAP
0000422 // m	0000421 // au	0005515 // pr	Gamma-amin	GBRL1_HUMAN|GABARAPL1|GEC1
0007193 // ad	0042734 // pr	0005515 // pr	Gamma-amin	GABR1_HUMAN|GABBR1|GPRC3A
0006810 // tra	0005887 // in	0004890 // GA	Gamma-amin	GBRA3_HUMAN|GABRA3
0007165 // sig	0005635 // nu	0004890 // GA	Gamma-amin	GBRB1_HUMAN|GABRB1
1902476 // ch	0045211 // po	0022851 // GA	Gamma-amin	GBRE_HUMAN|GABRE
1902476 // ch	0045211 // po	0004890 // GA	Gamma-amin	GBRG1_HUMAN|GABRG1
0006810 // tra	0032590 // de	0004890 // GA	Gamma-amin	GBRG2_HUMAN|GABRG2
0007214 // ga	0045211 // po	0005230 // ex	Gamma-amin	GBRG3_HUMAN|GABRG3
1902476 // ch	0045211 // po	0004890 // GA	Gamma-amin	GBRP_HUMAN|GABRP
1902476 // ch	0043235 // re	0004890 // GA	Gamma-amin	GBRT_HUMAN|GABRQ
1902476 // ch	0005886 // pl	0004890 // GA	Gamma-amin	GBRR1_HUMAN|GABRR1
0007214 // ga	0005886 // pl	0004890 // GA	Gamma-amin	GBRR2_HUMAN|GABRR2
0035176 // so	0061202 // cla	0005515 // pr	Glutamate de	DCE1_HUMAN|GAD1|GAD|GAD67

0000186 // ac	0005634 // nu	0005515 // pr	Growth arrest	GA45B_HUMAN|GADD45B|MYD118
0016032 // vi	0005761 // m	0005515 // pr	Growth arrest	G45IP_HUMAN|GADD45GIP1|MRPL59|PLINP1|PRG6
0008652 // ce	0005829 // cy	0004782 // su	Acidic amino a	GADL1_HUMAN|GADL1
1905224 // cl	0005794 // G	0005515 // pr	Cyclin-G-asso	GAK_HUMAN|GAK
0042493 // re	0005576 // ex	0031766 // ty	Galanin pepti	GALA_HUMAN|GAL|GAL1|GALN|GLNN
0007267 // ce	0016021 // in	0050694 // ga	Galactose-3-C	G3ST4_HUMAN|GAL3ST4|PP6968
0006683 // ga	0043202 // ly	0004336 // ga	Galactocerebr	GALC_HUMAN|GALC
0046835 // ca	0016020 // m	0004335 // ga	Galactokinase	GALK1_HUMAN|GALK1|GALK
0005975 // ca	0005737 // cy	0005534 // ga	N-acetylgalact	GALK2_HUMAN|GALK2|GK2
0006006 // gl	0070062 // ex	0004034 // al	Aldose 1-epim	GALM_HUMAN|GALM|BLOCK25
0016266 // O-	0000139 // G	0030246 // ca	Polypeptide N	GALT1_HUMAN|GALNT1
0006493 // pr	0016021 // in	0030246 // ca	Polypeptide N	GLT10_HUMAN|GALNT10
0016266 // O-	0016021 // in	0030246 // ca	Polypeptide N	GLT11_HUMAN|GALNT11
0016266 // O-	0016021 // in	0030246 // ca	Polypeptide N	GLT12_HUMAN|GALNT12
0018243 // pr	0016021 // in	0030246 // ca	Polypeptide N	GLT13_HUMAN|GALNT13|KIAA1918
0018243 // pr	0016021 // in	0030246 // ca	Polypeptide N	GLT16_HUMAN|GALNT16|GALNTL1|KIAA1130
0006493 // pr	0016021 // in	0030246 // ca	Polypeptide N	GLT18_HUMAN|GALNT18|GALNTL4
0018243 // pr	0005794 // G	0004653 // po	Polypeptide N	GALT2_HUMAN|GALNT2
0005975 // ca	0005794 // G	0030246 // ca	Polypeptide N	GALT3_HUMAN|GALNT3
0006024 // gl	0016021 // in	0030246 // ca	Polypeptide N	GALT5_HUMAN|GALNT5
0016266 // O-	0005794 // G	0030246 // ca	Polypeptide N	GALT6_HUMAN|GALNT6
0016266 // O-	0016021 // in	0030246 // ca	Probable poly	GALT8_HUMAN|GALNT8
0016266 // O-	0016021 // in	0004653 // po	Polypeptide N	GALT9_HUMAN|GALNT9
0018243 // pr	0016021 // in	0030246 // ca	Polypeptide N	GLTL6_HUMAN|GALNTL6|GALNT17
0046500 // S-	0070062 // ex	0030731 // gu	Guanidinoace	GAMT_HUMAN|GAMT
0061564 // ax	0005737 // cy	0005515 // pr	Neuromodulin	NEUM_HUMAN|GAP43
0000226 // m	0005829 // cy	0005515 // pr	Glyceraldehyd	G3P_HUMAN|GAPDH|GAPD|CDABP0047|OK/SW-cl.12
	0070062 // ex		GRB2-associat	GARE2_HUMAN|GAREM2|FAM59B|GAREML|KIAA2038
0070150 // m	0005739 // m	0004820 // gl	Glycine--tRNA	GARS_HUMAN|GARS
0007050 // ce	0005886 // pl	0005515 // pr	Growth arrest	GAS1_HUMAN|GAS1
0007050 // ce	0005737 // cy	0008093 // cy	GAS2-like pro	GA2L1_HUMAN|GAS2L1|GAR22
	0005737 // cy	0008017 // m	GAS2-like pro	GA2L2_HUMAN|GAS2L2|GAR17
0033138 // po	0005796 // G	0005515 // pr	Growth arrest	GAS6_HUMAN|GAS6|AXLLG
0033690 // po	0005634 // nu	0005515 // pr	Erythroid tran	GATA1_HUMAN|GATA1|ERYF1|GF1
0045746 // ne	0005634 // nu	0000979 // RN	Endothelial tr	GATA2_HUMAN|GATA2
0045944 // po	0005654 // nu	0005134 // in	Trans-acting T	GATA3_HUMAN|GATA3
0007596 // bl	0005634 // nu	0003700 // tr	Transcription	GATA6_HUMAN|GATA6
0006355 // re	0005634 // nu	0003700 // tr	GATA zinc fing	GATD1_HUMAN|GATAD1|ODAG
0045892 // ne	0005634 // nu	0003700 // tr	Transcriptiona	P66A_HUMAN|GATAD2A

0016139 // gl	0005789 // er	0004348 // gl	Non-lysosoma	GBA2_HUMAN	GBA2|KIAA1605|AD035
0006486 // pr	0016021 // in	0047277 // gl	Globoside alp	GBGT1_HUMAN	GBGT1|UNQ2513/PRO6002
1900041 // ne	0005576 // ex	0019899 // er	Guanylate-bin	GBP1_HUMAN	GBP1
0006955 // im	0005829 // cy	0005525 // G	Guanylate-bin	GBP2_HUMAN	GBP2
0051607 // de	0048471 // pe	0005525 // G	Guanylate-bin	GBP3_HUMAN	GBP3
	0005634 // nu	0005525 // G	Guanylate-bin	GBP4_HUMAN	GBP4
0071346 // ce	0070062 // ex	0005525 // G	Guanylate-bin	GBP6_HUMAN	GBP6
	0016020 // m	0005525 // G	Guanylate-bin	GBP7_HUMAN	GBP7|GBP4L
0061025 // m	0005576 // ex	0005509 // ca	Grancalcin	GRAN_HUMAN	GCA|GCL
0006520 // ce	0005739 // m	0030170 // py	2-amino-3-ket	KBL_HUMAN	GCAT|KBL
0009755 // ho	0005886 // pl	0004967 // gl	Glucagon rece	GLR_HUMAN	GCGR
0006750 // gl	0017109 // gl	0016595 // gl	Glutamate--cy	GSH1_HUMAN	GCLC|GLCL|GLCLC
0060706 // ce	0005634 // nu	0042826 // hi	Chorion-speci	GCM1_HUMAN	GCM1|GCMA
0050901 // le	0016021 // in	0005515 // pr	Beta-1,3-gala	GCNT1_HUMAN	GCNT1|NACGT2
0010718 // po	0016021 // in	0008109 // N-	N-acetyllactos	GNT2A_HUMAN	GCNT2|GCNT5|II|NACGT1
0042403 // th	0016021 // in	0008109 // N-	Beta-1,3-gala	GCNT4_HUMAN	GCNT4
0006486 // pr	0016021 // in	0008375 // ac	Beta-1,3-gala	GCNT7_HUMAN	GCNT7|C20orf105
0035556 // in	0016591 // DI		GRINL1A com	H8Y6P7_HUMAN	GCOM1
0050855 // re	0005886 // pl	0019901 // pr	Germinal cent	GCSAM_HUMAN	GCSAM|GAL|GCET2
0007399 // ne	0070062 // ex	0008892 // gu	Guanine dean	GUAD_HUMAN	GDA|KIAA1258
0006749 // gl	0005739 // m	0004364 // gl	Ganglioside-ir	GDAP1_HUMAN	GDAP1
0007399 // ne	0043234 // pr	0005515 // pr	Growth/differ	GDF11_HUMAN	GDF11|BMP11
0007179 // tra	0005615 // ex	0008083 // gr	Growth/differ	GDF15_HUMAN	GDF15|MIC1|PDF|PLAB|PTGFB
0007267 // ce	0005886 // pl	0008083 // gr	Growth/differ	GDF5_HUMAN	GDF5|BMP14|CDMP1
0051592 // re	0005829 // cy	0005092 // GI	Rab GDP disso	GDIA_HUMAN	GDI1|GDIL|OPHN2|RABGDIA|XAP4
0006629 // lip	0016021 // in	0047394 // gl	Glycerophosp	GDPD2_HUMAN	GDPD2|GDE3|OBDPF|UNQ1935/PRO4
0034638 // ph	0016021 // in	0046872 // m	Glycerophosp	GDPD3_HUMAN	GDPD3
0006955 // im	0051233 // sp	0005515 // pr	GTP-binding p	GEM_HUMAN	GEM|KIR
0006397 // m	0005654 // nu	0005515 // pr	Gem-associat	GEMI2_HUMAN	GEMIN2|SIP1
0006417 // re	0005654 // nu	0005515 // pr	Gem-associat	GEMI5_HUMAN	GEMIN5
0000387 // sp	0005654 // nu	0005515 // pr	Gem-associat	GEMI7_HUMAN	GEMIN7
0000387 // sp	0005634 // nu	0005515 // pr	Gem-associat	GEMI8_HUMAN	GEMIN8|FAM51A1
0051220 // cy	0071818 // BA	0051087 // ch	Golgi to ER tra	GET4_HUMAN	GET4|C7orf20|CEE|TRC35|CGI-20
0001889 // liv	0005739 // m	0008083 // gr	FAD-linked su	ALR_HUMAN	GFER|ALR|HERV1|HPO
0030097 // he	0005634 // nu	0005515 // pr	Zinc finger pro	GFI1_HUMAN	GFI1|ZNF163
0006048 // UI	0005829 // cy	0005515 // pr	Glutamine--fr	GFPT2_HUMAN	GFPT2
0043547 // po	0005622 // in	0005102 // re	GDNF family r	GFRA1_HUMAN	GFRA1|GDNFRA|RETL1|TRNR1
2001240 // ne	0016021 // in		GDNF family r	GFRAL_HUMAN	GFRAL|C6orf144|UNQ9356/PRO34128
0050896 // re	0030173 // in		Golgi-associat	GFY_HUMAN	GFY
0006750 // gl	0005829 // cy	0003839 // ga	Gamma-gluta	GGCT_HUMAN	GGCT|C7orf24|CRF21
0006464 // ce	0005789 // er	0008488 // ga	Vitamin K-dep	VKGC_HUMAN	GGCX|GC
0007283 // sp		0005515 // pr	Gametogenet	GGN_HUMAN	GGN
0045337 // fa	0005829 // cy	0004161 // di	Geranylgeran	GGPPS_HUMAN	GGPS1

1901750 // le	0016021 // in	0102953 /// 0	Glutathione h	GGT1_HUMAN|GGT1|GGT
1901750 // le	0016021 // in	0102953 /// 0	Glutathione h	GGT6_HUMAN|GGT6
1901750 // le	0016021 // in	0005515 // pr	Glutathione h	GGT7_HUMAN|GGT7|GGTL3|GGTL5
0008150 // bi	0034774 // se	0003674 // m	GH3 domain-c	GHDC_HUMAN|GHDC|D11LGP1E|LGP1
0048009 // in	0043234 // pr	0005515 // pr	GRB10-intera	GGYF1_HUMAN|GIGYF1|CDS2|PERQ1|PP3360
	0005811 // lip	0005525 // G	GTPase IMAP	GIMA2_HUMAN|GIMAP2|IMAP2
0001833 // in	0005654 // nu	0043138 // 3'	DNA replicati	PSF1_HUMAN|GINS1|KIAA0186|PSF1
0006271 // DI	0005654 // nu	0005515 // pr	DNA replicati	PSF2_HUMAN|GINS2|PSF2|CGI-122|DC5|HSPC037
0006271 // DI	0005654 // nu		DNA replicati	PSF3_HUMAN|GINS3|PSF3
0008150 // bi	0070062 // ex	0042802 // id	PDZ domain-c	GIPC2_HUMAN|GIPC2
0007204 // pc	0005886 // pl	0016519 // ga	Gastric inhibit	GIPR_HUMAN|GIPR
0048013 // ep	0005829 // cy	0005515 // pr	ARF GTPase-a	GIT1_HUMAN|GIT1
2000810 // re	0005886 // pl	0005515 // pr	Gap junction a	CXA1_HUMAN|GJA1|GJAL
0006810 // tra	0016021 // in	0005243 // ga	Gap junction a	CXA3_HUMAN|GJA3
0007154 // ce	0016021 // in		Gap junction a	CXA9_HUMAN|GJA9|GJA10
0006810 // tra	0016021 // in	0005243 // ga	Gap junction b	CXB2_HUMAN|GJB2
0007283 // sp	0016021 // in	0005243 // ga	Gap junction b	CXB3_HUMAN|GJB3|CX31
0007154 // ce	0016021 // in		Gap junction b	CXB4_HUMAN|GJB4
0060708 // sp	0005922 // co		Gap junction b	CXB5_HUMAN|GJB5
0007154 // ce	0016021 // in		Gap junction b	CXB6_HUMAN|GJB6
0007601 // vis	0005886 // pl	0005216 // io	Gap junction g	CXG1_HUMAN|GJC1|GJA7
0001932 // re	0016021 // in	1903763 // ga	Gap junction g	CXG2_HUMAN|GJC2|GJA12
0007605 // se	0016021 // in	0086077 // ga	Gap junction g	CXG3_HUMAN|GJC3|GJE1
0016310 // ph	0005739 // m	0004370 // gly	Putative glyce	GLPK5_HUMAN|GK5
0005975 // ca	0005615 // ex	0004565 // be	Beta-galactosi	GLB1L_HUMAN|GLB1L|UNQ229/PRO262
0036255 // re	0005739 // m	0009055 // el	Glycine dehyd	GCSP_HUMAN|GLDC|GCSP
0034113 // he	0016021 // in	0086080 // pr	Gliomedin	GLDN_HUMAN|GLDN|COLM|UNQ9339/PRO34011
0033089 // pc	0005829 // cy	0000978 // RI	Zinc finger pro	GLI2_HUMAN|GLI2|THP
0048557 // er	0005829 // cy	0005515 // pr	Transcriptiona	GLI3_HUMAN|GLI3
0006355 // re	0005634 // nu	0003677 // DI	Zinc finger pro	GLI4_HUMAN|GLI4|HKR4
0019216 // re	0016021 // in		Glioma patho	GLIP1_HUMAN|GLIPR1|GLIPR|RTVP1
0007338 // sir	0045121 // m		GLIPR1-like pr	GPRL1_HUMAN|GLIPR1L1|UNQ2972/PRO7434
0010634 // pc	0000139 // Go	0042803 // pr	Golgi-associat	GAPR1_HUMAN|GLIPR2|C9orf19|GAPR1
0006366 // tra	0005634 // nu	0001227 // tra	Zinc finger pro	GLIS1_HUMAN|GLIS1
0000122 // ne	0005634 // nu	0003677 // DI	Zinc finger pro	GLIS3_HUMAN|GLIS3|ZNF515
0030522 // in	0005765 // lys	0004879 // RI	Glycosylated l	GLMP_HUMAN|GLMP|C1orf85|PSEC0030|UNQ2553/PR
0071294 // ce	0043025 // ne	0016934 // ex	Glycine recep	GLRA1_HUMAN|GLRA1
0043200 // re	0005887 // in	0022852 // gly	Glycine recep	GLRA3_HUMAN|GLRA3
0007268 // ch	0045211 // pc	0016594 // gly	Glycine recep	GLRB_HUMAN|GLRB
0055114 // ox	0005829 // cy	0097573 // glu	Glutaredoxin-	GLRX1_HUMAN|GLRX|GRX
0044281 // sn	0005739 // m	0051537 // 2 i	Glutaredoxin-	GLRX5_HUMAN|GLRX5|C14orf87
0002087 // re	0005739 // m	0004359 // glu	Glutaminase k	GLSK_HUMAN|GLS|GLS1|KIAA0838
0006520 // ce	0005759 // m	0004359 // glu	Glutaminase l	GLSL_HUMAN|GLS2|GA
0000271 // pc	0016021 // in	0016757 // tra	Glycosyltransf	GL8D2_HUMAN|GLT8D2|GALA4A|UNQ1901/PRO4347

120009	0005737 // cy	120013	Glycolipid trar	GLTD2_HUMAN|GLTPD2
0019676 // ar	0043679 // ax	0004356 // glu	Glutamine syr	GLNA_HUMAN|GLUL|GLNS
0006544 // gly	0005759 // m	0047961 // gly	Glycine N-acy	GLYAT_HUMAN|GLYAT|ACGNAT|CAT|GAT
0006468 // pr	0005622 // int	0008047 // en	Glia maturatic	GMFB_HUMAN|GMFB
0008285 // ne	0005622 // int		Glycosyl-phos	GML_HUMAN|GML|LY6DL
0007049 // ce	0005634 // nu	0003682 // ch	Geminin coile	GEMC1_HUMAN|GMNC|GEMC1
0009058 // bi	0070062 // ex	0005515 // pr	Mannose-1-pl	GMPPA_HUMAN|GMPPA
0009298 // GI	0005737 // cy	0005525 // GT	Mannose-1-pl	GMPPB_HUMAN|GMPPB
0046037 // GI	1902560 // GI	0003920 // GI	GMP reductas	GMPR2_HUMAN|GMPR2
0001501 // sk	0005886 // pl	0005525 // GT	Guanine nucle	GNA11_HUMAN|GNA11|GA11
0030154 // ce	0005886 // pl	0003924 // GT	Guanine nucle	GNA12_HUMAN|GNA12
0007188 // ac	0005886 // pl	0005525 // GT	Guanine nucle	GNA15_HUMAN|GNA15|GNA16
1904778 // pc	0005886 // pl	0001664 // G-	Guanine nucle	GNAI1_HUMAN|GNAI1
0006906 // ve	0005886 // pl	0019003 // GI	Guanine nucle	GNAI3_HUMAN|GNAI3
0007212 // dc	0005886 // pl	0005525 // GT	Guanine nucle	GNAO_HUMAN|GNAO1
0007188 // ac	0005886 // pl	0005525 // GT	Guanine nucle	GNAZ_HUMAN|GNAZ
0007165 // sig	0005829 // cy	0047391 // all	Guanine nucle	GBB1_HUMAN|GNB1
0035176 // so	0009898 // cy	0003674 // m	Guanine nucle	GNB1L_HUMAN|GNB1L|GY2|KIAA1645|WDR14|FKSG1
0007186 // G-	0005829 // cy	0003924 // GT	Guanine nucle	GBB3_HUMAN|GNB3
0007155 // ce	0005737 // cy	0005515 // pr	Bifunctional U	GLCNE_HUMAN|GNE|GLCNE
0007165 // sig	0005886 // pl	0003924 // GT	Guanine nucle	GBG11_HUMAN|GNG11|GNGT11
0021987 // ce	0005886 // pl	0005515 // pr	Guanine nucle	GBG12_HUMAN|GNG12
0071870 // ce	0005886 // pl	0031681 // G-	Guanine nucle	GBG2_HUMAN|GNG2
0007186 // G-	0005886 // pl	0004871 // sig	Guanine nucle	GBG4_HUMAN|GNG4|GNGT4
0072513 // pc	0005886 // pl	0005515 // pr	Guanine nucle	GBG5_HUMAN|GNG5|GNGT5
0007186 // G-	0005886 // pl	0004871 // sig	Guanine nucle	GBG7_HUMAN|GNG7|GNGT7
0016056 // rh	0097381 // ph	0005515 // pr	Guanine nucle	GBG1_HUMAN|GNGT1
0042254 // rib	0005634 // nu	0003723 // RN	Guanine nucle	GNL3_HUMAN|GNL3|E2IG3|NS
0042254 // rib	0005730 // nu	0003723 // RN	Guanine nucle	GNL3L_HUMAN|GNL3L
0031640 // kil	0097013 // ph		Granulysin	GNLY_HUMAN|GNLY|LAG2|NKG5|TLA519
0007584 // re	0005782 // pe	0016290 // pa	Dihydroxyacet	GNPAT_HUMAN|GNPAT|DAPAT|DHAPAT
0006048 // UI	0005829 // cy	0004343 // glu	Glucosamine (	GNA1_HUMAN|GNPNAT1|GNA1
0007568 // ag	0005576 // ex	0005183 // gc	Progonadolibe	GON1_HUMAN|GNRH1|GNRH|GRH|LHRH
	0005794 // Gc	0005515 // pr	Golgin subfam	GOGA1_HUMAN|GOLGA1
1904668 // pc	0030134 // EF	0005515 // pr	Golgin subfam	GOGA2_HUMAN|GOLGA2
0006891 // int	0005794 // Gc	0005215 // tra	Golgin subfam	GOGA3_HUMAN|GOLGA3
0048193 // Gc	0005794 // Gc	0017137 // Ra	Golgin subfam	GOGA5_HUMAN|GOLGA5|RETII|RFG5|PIG31
	0005794 // Gc		Golgin subfam	GG6LA_HUMAN|GOLGA6L10|GOLGA6L18
	0005794 // Gc		Golgin subfam	GG6L9_HUMAN|GOLGA6L9|GOLGA6L20
	0005794 // Gc		Golgin subfam	GOG8A_HUMAN|GOLGA8A|KIAA0855
	0005794 // Gc		Golgin subfam	GOG8B_HUMAN|GOLGA8B

	0005794 // Go	0005515 // pr	Putative golgi	GOG8F_HUMAN|GOLGA8F|GOLGA8G
	0005794 // Go		Golgin subfam	GOG8H_HUMAN|GOLGA8H
	0005794 // Go		Golgin subfam	GOG8J_HUMAN|GOLGA8J
	0005794 // Go		Golgin subfam	GOG8K_HUMAN|GOLGA8K
	0005794 // Go		Golgin subfam	GOG8N_HUMAN|GOLGA8N
	0005794 // Go		Golgin A8 fam	A0A0J9YX86_HUMAN|GOLGA8Q
	0005794 // Go		Golgin subfam	GOG8R_HUMAN|GOLGA8R
	0005794 // Go		Golgin A8 fam	H3BPF8_HUMAN|GOLGA8S
	0005794 // Go		Golgin A8 fam	H3BQL2_HUMAN|GOLGA8T
0006810 // tra	0005794 // Go		Golgi integral	GOLI4_HUMAN|GOLIM4|GIMPC|GOLPH4|GPP130
0019216 // re	0005794 // Go	0005515 // pr	Golgi membra	GOLM1_HUMAN|GOLM1|C9orf155|GOLPH2|PSEC0242
0008283 // ce	0005794 // Go	0005515 // pr	Golgi phospho	GOLP3_HUMAN|GOLPH3|GPP34
0048194 // Go	0000139 // Go	0005515 // pr	Golgi phospho	GLP3L_HUMAN|GOLPH3L|GPP34R
0006888 // ER	0005802 // tra	0003674 // m	Vesicle transp	GOT1A_HUMAN|GOLT1A|GOT1B
0016192 // ve	0016021 // int	0004871 // sig	Vesicle transp	GOT1B_HUMAN|GOLT1B|GCT2|GOT1A|CGI-141|HDCM
0030183 // B	0016604 // nu	0003677 // DN	GON-4-like pr	GON4L_HUMAN|GON4L|GON4|KIAA1606
	0000408 // EK	0005515 // pr	EKC/KEOPS co	GON7_HUMAN|GON7|C14orf142
0000902 // ce	0005737 // cy	0004860 // pr	Platelet glycop	GP1BA_HUMAN|GP1BA
0006621 // pr	0042765 // GP	0005515 // pr	Glycosylphosp	GPAA1_HUMAN|GPAA1|GAA1
0032006 // re	0016021 // int	0004366 // gly	Glycerol-3-pho	GPAT3_HUMAN|GPAT3|AGPAT9|MAG1|HMFN0839|UN
	0000776 // kin	0005515 // pr	G patch doma	GPT11_HUMAN|GPATCH11|CCDC75|CENP-Y
0010923 // ne	0005730 // nu	0003676 // nu	G patch doma	GPTC2_HUMAN|GPATCH2|GPATC2
		0003723 // RN	G patch doma	GPTC4_HUMAN|GPATCH4|GPATC4
2000810 // re	0005886 // pla	0038181 // bi	G-protein cou	GPBAR_HUMAN|GPBAR1|TGR5
0006024 // gly	0005886 // pla	0017134 // fib	Glypican-1	GPC1_HUMAN|GPC1
0006024 // gly	0005796 // Go	0043395 // he	Glypican-2	GPC2_HUMAN|GPC2
0006024 // gly	0005796 // Go	0043395 // he	Glypican-5	GPC5_HUMAN|GPC5
0006024 // gly	0005887 // int	0043395 // he	Glypican-6	GPC6_HUMAN|GPC6|UNQ369/PRO705
0006127 // gly	0005743 // m	0052591 // sn	Glycerol-3-pho	GPDM_HUMAN|GPD2
0010629 // ne	0005783 // en	0030284 // es	G-protein cou	GPER1_HUMAN|GPER1|CEPR|CMKRL2|DRY12|GPER|GP
0018315 // m	0045211 // po	0008940 // nit	Gephyrin	GEPH_HUMAN|GPHN|GPH|KIAA1385
0035701 // he	0005737 // cy	0004621 // gly	Phosphatidyli	PHLD_HUMAN|GPLD1|PIGPLD1
0007416 // sy	1903561 // ex	0005515 // pr	Neuronal mem	GPM6A_HUMAN|GPM6A|M6A
0032956 // re	0005886 // pla	0003674 // m	Neuronal mem	GPM6B_HUMAN|GPM6B|M6B
0044376 // RN	0043234 // pr	0005525 // GT	GPN-loop GTP	GPN3_HUMAN|GPN3|ATPBD1C|AD-009|UNQ1876/PRO
0001649 // os	0016021 // int	0005515 // pr	Transmembra	GPNMB_HUMAN|GPNMB|HGFIN|NMB|UNQ1725/PRO9
0007186 // G-	0043005 // ne	0005515 // pr	G-protein cou	GPR1_HUMAN|GPR1
0072583 // cla	0005794 // Go	0032050 // cla	Protein GPR10	GP107_HUMAN|GPR107|KIAA1624|LUSTR1
	0016021 // int		Protein GPR10	GP108_HUMAN|GPR108|LUSTR2
0000082 // G1	0005886 // pla	0004930 // G-	Probable G-pr	GP132_HUMAN|GPR132|G2A
0007186 // G-	0016021 // int	0004930 // G-	Probable G-pr	GP135_HUMAN|GPR135
	0016021 // int		Integral meml	G137C_HUMAN|GPR137C|TM7SF1L2
0007186 // G-	0005886 // pla	0004930 // G-	Probable G-pr	GP150_HUMAN|GPR150
0007186 // G-	0016021 // int	0004930 // G-	Probable G-pr	GP153_HUMAN|GPR153|PGR1
0055085 // tra	0005737 // cy	0003674 // m	Integral meml	GP155_HUMAN|GPR155|PGR22

0007186 // G-	0016021 // in	0004930 // G-	Probable G-pr	GP158_HUMAN|GPR158|KIAA1136
0007186 // G-	0016021 // in	0004930 // G-	Probable G-pr	GP160_HUMAN|GPR160|GPCR150
0007186 // G-	0016021 // in	0004930 // G-	Probable G-pr	GP162_HUMAN|GPR162|GRCA
0051482 // po	0005886 // pl	0004950 // ch	Uracil nucleot	GPR17_HUMAN|GPR17
0007186 // G-	0016021 // in	0004930 // G-	Probable G-pr	GP173_HUMAN|GPR173|SREB3
0007601 // vi	0016021 // in	0004930 // G-	Probable G-pr	GP179_HUMAN|GPR179|GPR158L|GPR158L1
0007186 // G-	0005887 // in	0004930 // G-	Probable G-pr	GPR19_HUMAN|GPR19
0051482 // po	0005886 // pl	0004930 // G-	G-protein cou	GPR20_HUMAN|GPR20
0007189 // ac	0005622 // in	0004930 // G-	G-protein cou	GPR3_HUMAN|GPR3|ACCA
0007186 // G-	0000151 // ub	0008528 // G-	Prosaposin re	GPR37_HUMAN|GPR37
0048712 // ne	0043235 // re	0008528 // G-	Prosaposin re	ETBR2_HUMAN|GPR37L1|ETBRLP2
0007186 // G-	0005887 // in	0004930 // G-	G-protein cou	GPR39_HUMAN|GPR39
0007212 // do	0005887 // in	0001588 // do	Probable G-pr	GPR52_HUMAN|GPR52
0007186 // G-	0016021 // in	0004930 // G-	Probable G-pr	GPR61_HUMAN|GPR61|BALGR
0007186 // G-	0016021 // in	0004930 // G-	Probable G-pr	GPR63_HUMAN|GPR63|PSP24B
2001206 // po	0005886 // pl	0004930 // G-	Ovarian cance	OGR1_HUMAN|GPR68|OGR1
0007186 // G-	0005887 // in	0004930 // G-	Probable G-pr	GPR75_HUMAN|GPR75
0051384 // re	0005886 // pl	0004930 // G-	Probable G-pr	GPR83_HUMAN|GPR83|GPR72|KIAA1540
0007218 // ne	0005886 // pl	0004966 // ga	G-protein cou	GPR84_HUMAN|GPR84|EX33
0007186 // G-	0016021 // in	0004930 // G-	Probable G-pr	GPR85_HUMAN|GPR85|SREB2
0035589 // G-	0005887 // in	0045028 // G-	G-protein cou	GPR87_HUMAN|GPR87|GPR95|FKSG78
0051452 // in	0016021 // in	0008308 // vo	Golgi pH regu	GPHRB_HUMAN|GPR89B|GPHRB|GPR89C|HSPC201
1990172 // G-	0005829 // cy	0005515 // pr	G-protein cou	GASP1_HUMAN|GPRASP1|GASP|KIAA0443
0007186 // G-	0043235 // re	0004930 // G-	G-protein cou	GPC5C_HUMAN|GPRC5C|RAIG3|PSEC0087
0007186 // G-	0005886 // pl	0004930 // G-	G-protein cou	GPC6A_HUMAN|GPRC6A
0031175 // ne	0030426 // gr	0051219 // ph	G protein-regu	GRIN1_HUMAN|GPRIN1|KIAA1893
		0005515 // pr	G protein-regu	GRIN2_HUMAN|GPRIN2|KIAA0514
0000338 // pr	0005654 // nu	0005515 // pr	COP9 signalos	CSN1_HUMAN|GPS1|COPS1|CSN1
0007254 // JN	0005634 // nu	0005515 // pr	G protein path	GPS2_HUMAN|GPS2
0042853 // L-a	0070062 // ex	0030170 // py	Alanine amino	ALAT1_HUMAN|GPT|AAT1|GPT1
0042851 // L-a	0005759 // m	0030170 // py	Alanine amino	ALAT2_HUMAN|GPT2|AAT2|ALT2
0098869 // ce	0070062 // ex	0004602 // glu	Glutathione p	GPX2_HUMAN|GPX2
0042744 // hy	0070062 // ex	0005515 // pr	Glutathione p	GPX3_HUMAN|GPX3|GPXP
0098869 // ce	0005576 // ex	0004096 // ca	Glutathione p	GPX7_HUMAN|GPX7|GPX6|UNQ469/PRO828
0098869 // ce	0016021 // in	0004601 // pe	Probable gluta	GPX8_HUMAN|GPX8|UNQ847/PRO1785
	0016021 // in	0005515 // pr	GRAM domain	GRM1A_HUMAN|GRAMD1A|KIAA1533
	0016021 // in	0005515 // pr	GRAM domain	GRM1C_HUMAN|GRAMD1C|UNQ2543/PRO6095
0006915 // ap	0016021 // in	0003674 // m	GRAM domain	GRAM4_HUMAN|GRAMD4|DIP|KIAA0767
0008104 // pr	0045211 // po	0030165 // PD	General recep	GRASP_HUMAN|GRASP
0042326 // ne	0005829 // cy	0005515 // pr	Growth factor	GRB10_HUMAN|GRB10|GRBIR|KIAA0207
0009967 // po	0043231 // in	0042802 // id	Growth factor	GRB14_HUMAN|GRB14
0009967 // po	0005829 // cy	0019901 // pr	Growth factor	GRB7_HUMAN|GRB7
	0016021 // in		GREB1-like pr	GRB1L_HUMAN|GREB1L|C18orf6|KIAA1772
0002934 // de	0005654 // nu	0043565 // se	Grainyhead-lil	GRHL1_HUMAN|GRHL1|LBP32|MGR|TFCP2L2
0044030 // re	0016020 // m	0001228 // tra	Grainyhead-lil	GRHL2_HUMAN|GRHL2|BOM|TFCP2L3

0007389 // pa	0005634 // nu	0001228 // tra	Grainyhead-lil	GRHL3_HUMAN|GRHL3|SOM|TFCP2L4
0055114 // ox	0070062 // ex	0008465 // gly	Glyoxylate red	GRHPR_HUMAN|GRHPR|GLXR|MSTP035
0035249 // sy	0005886 // pla	0005231 // ex	Glutamate rec	GRIA2_HUMAN|GRIA2|GLUR2
0006810 // tra	1903561 // ex	0005231 // ex	Glutamate rec	GRIA4_HUMAN|GRIA4|GLUR4
0060292 // lo	0045211 // po		Delphilin	GRD2I_HUMAN|GRID2IP
0051966 // re	0045211 // po	0005234 // ex	Glutamate rec	GRIK1_HUMAN|GRIK1|GLUR5
0051967 // ne	0005886 // pla	0031625 // ub	Glutamate rec	GRIK2_HUMAN|GRIK2|GLUR6
0043547 // po	0005622 // int	0004972 // NI	Glutamate rec	NMDE4_HUMAN|GRIN2D|GluN2D|NMDAR2D
0006816 // ca	0014069 // po	0004972 // NI	Glutamate rec	NMD3A_HUMAN|GRIN3A|KIAA1973
0070588 // ca	0045211 // po	0016594 // gly	Glutamate rec	NMD3B_HUMAN|GRIN3B
0030521 // an	0055037 // re	0005515 // pr	Glutamate rec	GRIP1_HUMAN|GRIP1
1905244 // re	0016020 // m	0005515 // pr	GRIP1-associa	GRAP1_HUMAN|GRIPAP1|KIAA1167
0060048 // ca	0005886 // pla	0004703 // G-	Beta-adrenerg	ARBK1_HUMAN|GRK2|ADRBK1|BARK|BARK1
0006468 // pr	0005886 // pla	0047696 // be	Beta-adrenerg	ARBK2_HUMAN|GRK3|ADRBK2|BARK2
0016055 // W	0005886 // pla	0005515 // pr	G protein-cou	GRK5_HUMAN|GRK5|GPRK5
0006468 // pr	0005886 // pla	0047696 // be	G protein-cou	GRK6_HUMAN|GRK6|GPRK6
0007165 // sig	0097381 // ph	0050254 // rh	Rhodopsin kin	GRK7_HUMAN|GRK7|GPRK7
0051482 // po	0005886 // pla	0004930 // G-	Metabotropic	GRM1_HUMAN|GRM1|GPRC1A|MGLUR1
0007196 // ad	0043197 // de	0005246 // ca	Metabotropic	GRM3_HUMAN|GRM3|GPRC1C|MGLUR3
0007611 // lea	0005886 // pla	0099583 // ne	Metabotropic	GRM5_HUMAN|GRM5|GPRC1E|MGLUR5
0051966 // re	0005886 // pla	0004930 // G-	Metabotropic	GRM8_HUMAN|GRM8|GPRC1H|MGLUR8
0030150 // pr	0005739 // m	0051087 // ch	GrpE protein l	GRPE2_HUMAN|GRPEL2
0007186 // G-	0005886 // pla	0008528 // G-	Gastrin-releas	GRPR_HUMAN|GRPR
0031338 // re	0005622 // int	0005096 // GT	Growth horm	GRTP1_HUMAN|GRTP1|TBC1D6
0006337 // nu	0043234 // pr	0003723 // RN	Glutamate-ric	GRWD1_HUMAN|GRWD1|GRWD|KIAA1942|WDR28
0007605 // se	0032420 // ste	0051082 // ur	Glutaredoxin	GRCR2_HUMAN|GRXCR2
0030162 // re	0005802 // tra	0001540 // be	Gamma-secre	GSAP_HUMAN|GSAP|PION
0008150 // bi	0005886 // pla	0003674 // m	Gasdermin-B	GSDMB_HUMAN|GSDMB|GSDML|PP4052|PRO2521
0008150 // bi	0005739 // m	0003674 // m	Gasdermin-C	GSDMC_HUMAN|GSDMC|MLZE
0050829 // de	0005615 // ex	0005515 // pr	Gasdermin-D	GSDMD_HUMAN|GSDMD|DFNA5L|GSDMDC1|FKSG10
		0005515 // pr	Genetic suppr	GSE1_HUMAN|GSE1|KIAA0182
	0030054 // ce	0005515 // pr	Germ cell-spe	GSG1L_HUMAN|GSG1L|UNQ5831/PRO19651
0007064 // m	0005737 // cy	0004672 // pr	Serine/threon	HASP_HUMAN|GSG2
0003214 // ca	0005829 // cy	0005515 // pr	Glycogen synt	GSK3A_HUMAN|GSK3A
0008631 // int	0005737 // cy	0005515 // pr	GSK3-beta int	GSKIP_HUMAN|GSKIP|C14orf129|HSPC210

0031648 // pr	0034774 // se	0005515 // pr	Gelsolin	GELS_HUMAN|GSN
0006749 // glu	0005759 // m	0004362 // glu	Glutathione re	GSHR_HUMAN|GSR|GLUR|GRD1
0008150 // bi	0005737 // cy	0005515 // pr	Glutathione S-	GSTCD_HUMAN|GSTCD
0042178 // xe	0005737 // cy	0019899 // er	Glutathione S-	GSTM1_HUMAN|GSTM1|GST1
0042178 // xe	0016529 // sa	0004364 // glu	Glutathione S-	GSTM2_HUMAN|GSTM2|GST4
0043627 // re	0070062 // ex	0042802 // id	Glutathione S-	GSTM3_HUMAN|GSTM3|GST5
0060315 // ne	0070062 // ex	0005515 // pr	Glutathione S-	GSTO1_HUMAN|GSTO1|GSTTLP28
0009407 // to	0005737 // cy	0005515 // pr	Glutathione S-	GSTO2_HUMAN|GSTO2
0000302 // re	0005737 // cy	0008144 // dr	Glutathione S-	GSTP1_HUMAN|GSTP1|FAEES3|GST3
0006749 // glu	0070062 // ex	0004364 // glu	Glutathione S-	GST2_HUMAN|GSTT2
0006749 // glu	0070062 // ex	0004364 // glu	Glutathione S-	GSTT2_HUMAN|GSTT2B|GSTT2
0006559 // L-|	0005829 // cy	0016034 // m	Maleylacetoa	MAAI_HUMAN|GSTZ1|MAAI
0006366 // tra	0005654 // nu	0005515 // pr	Transcription	TF2AA_HUMAN|GTF2A1|TF2A1
0006368 // tra	0005654 // nu	0003723 // RN	Transcription	T2EB_HUMAN|GTF2E2|TF2E2
0006362 // tra	0005654 // nu	0005515 // pr	General trans	TF2H2_HUMAN|GTF2H2|BTF2P44
0006289 // nu	0000439 // co	0005515 // pr	General trans	T2H2L_HUMAN|GTF2H2C|GTF2H2C_2|GTF2H2D
0006362 // tra	0005654 // nu	0000182 // rD	General trans	TF2H5_HUMAN|GTF2H5|C6orf175|TTDA
0014886 // tra	0005654 // nu	0005515 // pr	General trans	GTF2I_HUMAN|GTF2I|BAP135|WBSCR6
0006366 // tra	0005654 // nu	0005515 // pr	General trans	GT2D1_HUMAN|GTF2IRD1|CREAM1|GTF3|MUSTRD1|R
0006351 // tra	0005654 // nu	0005515 // pr	General trans	GTD2A_HUMAN|GTF2IRD2|GTF2IRD2A
0006351 // tra	0005654 // nu	0003677 // DN	General trans	GTD2B_HUMAN|GTF2IRD2B
0006383 // tra	0005654 // nu	0003677 // DN	Transcription	TF3A_HUMAN|GTF3A
0042791 // 5S	0005654 // nu	0005515 // pr	General trans	TF3C6_HUMAN|GTF3C6|C6orf51|CDA020|NPD020
0006400 // tR	0005739 // m	0005525 // GT	tRNA modifica	GTPB3_HUMAN|GTPBP3|MTGP1
0043161 // pr	0005829 // cy	0005515 // pr	G2 and S phas	GTSE1_HUMAN|GTSE1
0007263 // ni	0005886 // pl	0005525 // GT	Guanylate cyc	GCYA3_HUMAN|GUCY1A3|GUC1A3|GUCSA3|GUCY1A1
0035556 // in	0008074 // gu	0005525 // GT	Guanylate cyc	GCYB2_HUMAN|GUCY1B2
0007263 // ni	0005886 // pl	0005525 // GT	Guanylate cyc	GCYB1_HUMAN|GUCY1B3|GUC1B3|GUCSB3|GUCY1B1
0007586 // di	0005886 // pl	0005525 // GT	Heat-stable er	GUC2C_HUMAN|GUCY2C|GUC2C|STAR
0035556 // in	0008074 // gu	0005515 // pr	Retinal guany	GUC2D_HUMAN|GUCY2D|CORD6|GUC1A4|GUC2D|RET
0007165 // sig	0005737 // cy	0004871 // sig	PTB domain-c	GULP1_HUMAN|GULP1|CED6|GULP
0007399 // ne	0005764 // lys	0004553 // hy	Putative inact	GUSP1_HUMAN|GUSBP1|SMA3
	0005829 // cy	0005525 // GT	Interferon-inc	GVIN1_HUMAN|GVINP1|GVIN1|VLIG1
0048741 // sk			Protein GVQW	GVQW1_HUMAN|GVQW1|TIGD1L2

0016266 // O-	0016021 // in	0035252 // UI	Glucoside xylo	GXLT2_HUMAN|GXYLT2|GLT8D4
0005978 // gl	0005829 // cy	0005515 // pr	Glycogenin-1	GLYG_HUMAN|GYG1|GYG
	0005886 // pl		Glycophorin-E	GLPE_HUMAN|GYPE|GPE
0005978 // gl	0005829 // cy	0061547 // gl	Glycogen [sta	GYS2_HUMAN|GYS2
0045892 // ne	0005730 // nu	0001206 // tra	GDNF-inducib	GZF1_HUMAN|GZF1|ZBTB23|ZNF336
0006309 // ap	0005794 // Go	0003723 // RN	Histone H1.0	H10_HUMAN|H1F0|H1FV
0006334 // nu	0005730 // nu	0003723 // RN	Histone H1x	H1X_HUMAN|H1FX
0006397 // m	0000788 // nu	0003677 // DN	Histone H2A-B	H2AB1_HUMAN|H2AFB1
0006342 // ch	0000790 // nu	0003677 // DN	Histone H2A.V	H2AV_HUMAN|H2AFV|H2AV
0006974 // ce	0005654 // nu	0005515 // pr	Histone H2AX	H2AX_HUMAN|H2AFX|H2AX
0050830 // de	0000786 // nu	0046982 // pr	Histone H2B t	H2BFS_HUMAN|H2BFS
0009725 // re	0005654 // nu	0005515 // pr	Histone H3.3	H33_HUMAN|H3F3A|H3F3B|H3.3A|H3.3B|H3F3|PP781
0006006 // gl	0005788 // er	0004345 // gl	GDH/6PGL en	G6PE_HUMAN|H6PD|GDH
0019805 // qu	0070062 // ex	0005515 // pr	3-hydroxyanth	3HAO_HUMAN|HAAO
0002576 // pl	0005730 // nu	0005515 // pr	Intracellular h	HABP4_HUMAN|HABP4
0030148 // sp	0005783 // er	0102344 // 3-	Very-long-cha	HACD4_HUMAN|HACD4|PTPLAD2
0001561 // fa	0005777 // pe	0005515 // pr	2-hydroxyacyl	HACL1_HUMAN|HACL1|HPCL|HPCL2|PHYH2|HSPC279
0006635 // fa	0005759 // m	0070403 // NA	Hydroxyacyl-c	HCDH_HUMAN|HADH|HAD|HADHSC|SCHAD
0006750 // gl	0070062 // ex	0004416 // hy	Hydroxyacylgl	GLO2_HUMAN|HAGH|GLO2|HAGH1
0019557 // hi	0005829 // cy	0004397 // hi	Histidine amm	HUTH_HUMAN|HAL|HIS
0017157 // re	0005814 // ce	0005515 // pr	Huntingtin-as	HAP1_HUMAN|HAP1|HAP2|HLP1
0007155 // ce	0031012 // ex	0005540 // hy	Hyaluronan ar	HPLN1_HUMAN|HAPLN1|CRTL1
0007155 // ce	0005615 // ex	0005540 // hy	Hyaluronan ar	HPLN3_HUMAN|HAPLN3|EXLD1|UNQ238/PRO271
0032543 // m	0005737 // cy	0004821 // hi	Histidine--tRN	SYHC_HUMAN|HARS|HRS
0036302 // at	0005887 // in	0050501 // hy	Hyaluronan sy	HYAS2_HUMAN|HAS2
0030213 // hy	0005886 // pl	0050501 // hy	Hyaluronan sy	HYAS3_HUMAN|HAS3
0006475 // in	0000784 // nu	0005515 // pr	Histone acety	HAT1_HUMAN|HAT1|KAT1
0000086 // G2	0005829 // cy	0005515 // pr	HAUS augmin	HAUS1_HUMAN|HAUS1|CCDC5|HEIC
0000086 // G2	0005829 // cy	0003674 // m	HAUS augmin	HAUS2_HUMAN|HAUS2|C15orf25|CEP27
0000086 // G2	0005829 // cy	0031996 // th	HAUS augmin	HAUS7_HUMAN|HAUS7|UCHL5IP|UIP1
0046718 // vi	0016021 // in	0001618 // vi	Hepatitis A vir	HAVR1_HUMAN|HAVCR1|KIM1|TIM1|TIMD1
0014070 // re	0005833 // he	0005515 // pr	Hemoglobin s	HBE_HUMAN|HBE1|HBE
0007173 // ep	0005576 // ex	0005154 // ep	Proheparin-bi	HBEGF_HUMAN|HBEGF|DTR|DTS|HEGFL
0007596 // bl	0072562 // bl	0020037 // he	Hemoglobin s	HBG2_HUMAN|HBG2
0007050 // ce	0005654 // nu	0003677 // DN	HMG box-con	HBP1_HUMAN|HBP1
0043928 // ex	0005829 // cy	0005525 // GT	HBS1-like prot	HBS1L_HUMAN|HBS1L|HBS1|KIAA1038
0007186 // G-	0005886 // pl	0004930 // G-	Hydroxycarbo	HCAR1_HUMAN|HCAR1|GPR104|GPR81|HCA1|FKSG80
0070165 // po	0005886 // pl	0070553 // ni	Hydroxycarbo	HCAR2_HUMAN|HCAR2|GPR109A|HCA2|HM74A|NIACR
0007186 // G-	0005886 // pl	0004930 // G-	Hydroxycarbo	HCAR3_HUMAN|HCAR3|GPR109B|HCA3|HM74B|NIACR
	0005737 // cy		Host cell facto	HPIP_HUMAN|HCFC1R1|HPIP
0006357 // re	0005737 // cy	0003713 // tra	Host cell facto	HCFC2_HUMAN|HCFC2

0009725 // re	0005634 // nu	0017124 // SH	Hematopoieti	HCLS1_HUMAN|HCLS1|HS1
0035725 // so	0005886 // pl	0042802 // id	Potassium/so	HCN2_HUMAN|HCN2|BCNG2
0006952 // de			HLA class I his	HCP5_HUMAN|HCP5
0010840 // re	0005886 // pl	0016499 // or	Orexin recept	OX2R_HUMAN|HCRTR2
0014003 // ol	0005886 // pl	0004407 // hi	Histone deace	HDA11_HUMAN|HDAC11
0043393 // re	0005634 // nu	0005515 // pr	Histone deace	HDAC5_HUMAN|HDAC5|KIAA0600
0016925 // pr	0005634 // nu	0005515 // pr	Histone deace	HDAC7_HUMAN|HDAC7|HDAC7A
0034983 // pe	0005737 // cy	0008134 // tr	Histone deace	HDAC9_HUMAN|HDAC9|HDAC7|HDAC7B|HDRP|KIAA07
0006351 // tr	0031012 // ex	0003723 // RN	Hepatoma-de	HDGF_HUMAN|HDGF|HMG1L2
0046474 // gl	0005739 // m		Haloacid deha	HDHD5_HUMAN|HDHD5|CECR5
0007623 // ci	0005576 // ex	0020037 // he	Heme-binding	HEBP1_HUMAN|HEBP1|HBP
0045930 // ne	0005737 // cy	0003674 // m	Headcase prot	HDC_HUMAN|HECA|HDC
0043161 // pr	0005737 // cy	0004842 // ub	E3 ubiquitin-p	HECD3_HUMAN|HECTD3
0042787 // pr	0005829 // cy	0061630 // ub	E3 ubiquitin-p	HECW1_HUMAN|HECW1|KIAA0322|NEDL1
0042787 // pr	0005737 // cy	0005515 // pr	E3 ubiquitin-p	HECW2_HUMAN|HECW2|KIAA1301|NEDL2
0003281 // ve	0005911 // ce	0005509 // ca	Protein HEG h	HEG1_HUMAN|HEG1|KIAA1237
0007275 // m	0005654 // nu	0005515 // pr	Hemogen	HEMGN_HUMAN|HEMGN|EDAG|NDR|PRO1037|PRO16
0034587 // pi	0043186 // P	0005515 // pr	Small RNA 2'-(	HENMT_HUMAN|HENMT1|C1orf59
0006825 // co	0016021 // in	0004322 // fe	Hephaestin-lil	HPHL1_HUMAN|HEPHL1
0000209 // pr	0005829 // cy	0004842 // ub	Probable E3 u	HERC6_HUMAN|HERC6
0007283 // sp	0016021 // in	0005515 // pr	Homocysteine	HERP2_HUMAN|HERPUD2
0021557 // oc	0005654 // nu	0003700 // tr	Transcription	HES1_HUMAN|HES1|BHLHB39|HL|HRY
0006351 // tr	0005634 // nu	0001078 // tr	Transcription	HES2_HUMAN|HES2|BHLHB40
0006351 // tr	0005667 // tr	0001227 // tr	Transcription	HES6_HUMAN|HES6|BHLHB41
0045666 // po	0005654 // nu	0050683 // AF	Hairy/enhanc	HEYL_HUMAN|HEYL|BHLHB33|HRT3
0048260 // po	0042612 // M	0005102 // re	Hereditary he	HFE_HUMAN|HFE|HLAH
0006559 // L-	0070062 // ex	0004411 // ho	Homogentisat	HGD_HUMAN|HGD|HGO
0001837 // ep	0005576 // ex	0005515 // pr	Hepatocyte gr	HGF_HUMAN|HGF|HPTA
0072657 // pr	0005829 // cy	0005515 // pr	Hepatocyte gr	HGS_HUMAN|HGS|HRS
0007041 // ly	0016021 // in	0015019 // he	Heparan-alph	HGNAT_HUMAN|HGSNAT|TMEM76
0018345 // pr	0016021 // in	0005525 // G	Protein-cystei	HHAT_HUMAN|HHAT|MART2|SKI1
0007224 // sn	0060170 // cil	0003824 // ca	Hedgehog-int	HHIP_HUMAN|HHIP|HIP|UNQ5825/PRO19644
0008150 // bi	0005576 // ex	0005044 // sc	HHIP-like prot	HIPL1_HUMAN|HHIPL1|HHIP2|KIAA1822|UNQ9245/PR(

	0005576 // ex	0003824 // ca	HHIP-like prot	HIPL2_HUMAN|HHIPL2|HHIP3|KIAA1822L|UNQ841/PRC
	0005576 // ex		HERV-H LTR-a	HHLA1_HUMAN|HHLA1
0001819 // pc	0016021 // int	0005515 // pr	HERV-H LTR-a	HHLA2_HUMAN|HHLA2
		0005515 // pr	HERV-H LTR-a	HHLA3_HUMAN|HHLA3
0006355 // re	0005829 // cy	0005515 // pr	Hypermethyla	HIC1_HUMAN|HIC1|ZBTB29
0045892 // ne	0005634 // nu	0008022 // pr	Hypermethyla	HIC2_HUMAN|HIC2|HRG22|KIAA1020|ZBTB30
0006886 // in	0000138 // Go	0005515 // pr	Protein HID1	HID1_HUMAN|HID1|C17orf28|DMC1
0055114 // ox	0005743 // m		HIG1 domain	HIG1A_HUMAN|HIGD1A|HIG1|HSPC010
0055114 // ox	0016021 // int		HIG1 domain	HIG2A_HUMAN|HIGD2A
0072332 // in	0070062 // ex	0005080 // pr	Histidine triad	HINT1_HUMAN|HINT1|HINT|PKCI1|PRKCNH1
0006694 // st	0005739 // m	0016787 // hy	Histidine triad	HINT2_HUMAN|HINT2
0045742 // pc	0016324 // ap	0042803 // pr	Huntingtin-int	HIP1R_HUMAN|HIP1R|HIP12|KIAA0655
0010842 // re	0005829 // cy	0004674 // pr	Homeodomai	HIPK1_HUMAN|HIPK1|KIAA0630|MYAK|NBAK2
0006336 // DI	0016605 // PN	0005515 // pr	Protein HIRA	HIRA_HUMAN|HIRA|DGCR1|HIR|TUPLE1
0006342 // ch	0000790 // nu	0005515 // pr	Histone H2A t	H2A1_HUMAN|HIST1H2AG|HIST1H2AI|HIST1H2AK|HIST
0050830 // de	0070062 // ex	0003677 // DI	Histone H2B t	H2B1C_HUMAN|HIST1H2BC|HIST1H2BE|HIST1H2BF|HIS
0016567 // pr	0070062 // ex	0003674 // m	Histone H2B t	H2B1D_HUMAN|HIST1H2BD|H2BFB|HIRIP2
0031640 // kil	0000786 // nu	0005515 // pr	Histone H2B t	H2B1J_HUMAN|HIST1H2BJ|H2BFR
0050829 // de	0000786 // nu	0003674 // m	Histone H2B t	H2B1K_HUMAN|HIST1H2BK|H2BFT|HIRIP1
0006334 // nu	0070062 // ex	0003677 // DI	Histone H2B t	H2B1N_HUMAN|HIST1H2BN|H2BFD
0006342 // ch	0000790 // nu	0003677 // DI	Histone H2A t	H2A2A_HUMAN|HIST2H2AA3|HIST2H2AA4|H2AFO|HIST
0006334 // nu	0000786 // nu	0046982 // pr	Putative histo	H2B2D_HUMAN|HIST2H2BD
0006334 // nu	0000786 // nu	0003677 // DI	Histone H2B t	H2B2E_HUMAN|HIST2H2BE|H2BFQ
0006334 // nu	0070062 // ex	0003674 // m	Histone H2B t	H2B2F_HUMAN|HIST2H2BF
0070914 // UV	0000788 // nu	0046982 // pr	Histone H2A t	H2A3_HUMAN|HIST3H2A
0006334 // nu	0000786 // nu	0003674 // m	Histone H2B t	H2B3B_HUMAN|HIST3H2BB
0007275 // m	0005739 // m	0005515 // pr	Zinc finger prc	ZEP1_HUMAN|HIVEP1|ZNF40
0007275 // m	0005634 // nu	0003677 // DI	Transcription	ZEP2_HUMAN|HIVEP2
0007275 // m	0005737 // cy	0003700 // tra	Transcription	ZEP3_HUMAN|HIVEP3|KBP1|KIAA1555|KRC|ZAS3
0072655 // es	0005739 // m	0005515 // pr	Hexokinase-1	HXK1_HUMAN|HK1
0072655 // es	0005741 // m	0005515 // pr	Hexokinase-2	HXK2_HUMAN|HK2
0046835 // ca	0005739 // m	0005536 // glu	Putative hexo	HKDC1_HUMAN|HKDC1
0050776 // re	0071556 // int	0005102 // re	HLA class I his	1B73_HUMAN|HLA-B|HLAB
0050776 // re	0009986 // ce	0042605 // pe	HLA class I his	1C17_HUMAN|HLA-C|D6S204|HLA-JY3|HLAC
0006955 // im	0042613 // M	0023026 // M	HLA class II his	DMA_HUMAN|HLA-DMA|DMA|RING6
0019886 // an	0042613 // M	0005515 // pr	HLA class II his	DMB_HUMAN|HLA-DMB|DMB|RING7
0007165 // sig	0016021 // int	0023026 // M	HLA class II his	DOB_HUMAN|HLA-DOB

0019886 // ar	0005886 // pl	0042605 // pe	HLA class II his	DPA1_HUMAN|HLA-DPA1|HLA-DP1A|HLASB
0019886 // ar	0005886 // pl	0042605 // pe	HLA class II his	DPB1_HUMAN|HLA-DPB1|HLA-DP1B
0002455 // hu	0005886 // pl	0005515 // pr	HLA class II his	DQB1_HUMAN|HLA-DQB1|HLA-DQB
0050776 // re	0071556 // in	0046978 // TA	HLA class I his	HLAF_HUMAN|HLA-F|HLA-5.4|HLAF
0002767 // im	0071556 // in	0042803 // pr	HLA class I his	HLAG_HUMAN|HLA-G|HLA-6.0|HLAG
0050776 // re	0071556 // in	0030881 // be	Putative HLA c	HLAH_HUMAN|HLA-H|HLAH
0001889 // liv	0005634 // nu	0005515 // pr	H2.0-like hom	HLX_HUMAN|HLX|HLX1
0051973 // po	0000784 // nu	0005515 // pr	Homeobox-co	HMBX1_HUMAN|HMBOX1
0050896 // re	0070062 // ex	0005509 // ca	Hemicentin-1	HMCN1_HUMAN|HMCN1|FIBL6
0045087 // in	0005694 // ch	0005515 // pr	High mobility	HMGB3_HUMAN|HMGB3|HMG2A|HMG4
0046951 // ke	0016020 // m	0004419 // hy	3-hydroxymet	HMGC2_HUMAN|HMGCLL1
0042493 // re	0005829 // cy	0008144 // dr	Hydroxymethy	HMCS1_HUMAN|HMGCS1|HMGCS
0031640 // kil	0005615 // ex	0003723 // RN	Non-histone c	HMGN2_HUMAN|HMGN2|HMG17
0006749 // glu	0005634 // nu	0003723 // RN	High mobility	HMGN5_HUMAN|HMGN5|NSBP1
0007264 // sm	0005783 // en	0005515 // pr	Heme oxygen	HMOX1_HUMAN|HMOX1|HO|HO1
0002253 // ac	0005737 // cy	0004867 // se	Serpin-like pro	HMSD_HUMAN|HMSD
0001714 // en	0005634 // nu	0001159 // co	Hepatocyte nu	HNF1B_HUMAN|HNF1B|TCF2
0043401 // ste	0005654 // nu	0001077 // tra	Hepatocyte nu	HNF4G_HUMAN|HNF4G|NR2A2
0032259 // m	0043005 // ne	0046539 // his	Histamine N-m	HNMT_HUMAN|HNMT
0016070 // RN	0005654 // nu	0003723 // RN	Heterogeneou	ROA0_HUMAN|HNRNPA0|HNRPA0
0051170 // nu	0005654 // nu	0003723 // RN	Heterogeneou	ROA1_HUMAN|HNRNPA1|HNRPA1
0051028 // m	1990124 // m	0003723 // RN	Heterogeneou	ROA3_HUMAN|HNRNPA3|HNRPA3
0001837 // ep	0030529 // in	0003723 // RN	Heterogeneou	ROAA_HUMAN|HNRNPAB|ABBP1|HNRPAB
0000398 // m	0005654 // nu	0003723 // RN	Heterogeneou	HNRPF_HUMAN|HNRNPF|HNRPF
0000398 // m	0005654 // nu	0003723 // RN	Heterogeneou	HNRH1_HUMAN|HNRNPH1|HNRPH|HNRPH1
0000398 // m	0005654 // nu	0003723 // RN	Heterogeneou	HNRPM_HUMAN|HNRNPM|HNRPM|NAGR1
0008150 // bi	0005634 // nu	0003723 // RN	Heterogeneou	HNRL2_HUMAN|HNRNPUL2|HNRPUL2
0046487 // gly	0070062 // ex	0008700 // 4-	4-hydroxy-2-o	HOGA1_HUMAN|HOGA1|C10orf65|DHDPSL
0048148 // be	0014069 // po	0005515 // pr	Homer protein	HOME2_HUMAN|HOMER2
0007216 // G-	0014069 // po	0005515 // pr	Homer protein	HOME3_HUMAN|HOMER3
0006351 // tra	0005730 // nu	0003677 // DN	Homeobox an	HOMEZ_HUMAN|HOMEZ|KIAA1443
0007275 // m	0070695 // FH	0005515 // pr	Protein Hook	HOOK1_HUMAN|HOOK1
0031122 // cy	0005813 // ce	0005515 // pr	Protein Hook	HOOK2_HUMAN|HOOK2
0045596 // ne	0005634 // nu	0005515 // pr	Homeodomai	HOP_HUMAN|HOPX|HOD|HOP|LAGY|NECC1|OB1

0048477 // oc	0005694 // ch		HORMA doma	HORM1_HUMAN|HORMAD1|NOHMA
0042473 // ou	0005634 // nu	0005515 // pr	Homeobox pr	HXA1_HUMAN|HOXA1|HOX1F
0007283 // sp	0005634 // nu	0005515 // pr	Homeobox pr	HXA10_HUMAN|HOXA10|HOX1H
0001501 // sk	0005667 // tra	0043565 // se	Homeobox pr	HXA11_HUMAN|HOXA11|HOX1I
0007275 // m	0043231 // in	0003700 // tra	Homeobox pr	HXA2_HUMAN|HOXA2|HOX1K
0007275 // m	0016604 // nu	0043565 // se	Homeobox pr	HXA4_HUMAN|HOXA4|HOX1D
0045944 // po	0031965 // nu	0001077 // tra	Homeobox pr	HXA7_HUMAN|HOXA7|HOX1A
0007389 // pa	0005634 // nu	0019904 // pr	Homeobox pr	HXB1_HUMAN|HOXB1|HOX2I
0040008 // re	0005667 // tra	0001227 // tra	Homeobox pr	HXB13_HUMAN|HOXB13
0048704 // er	0005654 // nu	0043565 // se	Homeobox pr	HXB2_HUMAN|HOXB2|HOX2H
0030878 // th	0005634 // nu	0001077 // tra	Homeobox pr	HXB3_HUMAN|HOXB3|HOX2G
0048704 // er	0005634 // nu	0043565 // se	Homeobox pr	HXB4_HUMAN|HOXB4|HOX2F
0006366 // tra	0005829 // cy	0005515 // pr	Homeobox pr	HXB5_HUMAN|HOXB5|HOX2A
0034101 // er	0005634 // nu	0003723 // RN	Homeobox pr	HXB6_HUMAN|HOXB6|HOX2B
0030099 // m	0016604 // nu	0005515 // pr	Homeobox pr	HXB7_HUMAN|HOXB7|HOX2C
0021516 // do	0005654 // nu	0003700 // tra	Homeobox pr	HXB8_HUMAN|HOXB8|HOX2D
0030879 // m	0090575 // RN	0005515 // pr	Homeobox pr	HXB9_HUMAN|HOXB9|HOX2E
0001501 // sk	0016604 // nu	0000977 // RN	Homeobox pr	HXC10_HUMAN|HOXC10|HOX3I
0006366 // tra	0005634 // nu	0005515 // pr	Homeobox pr	HXC4_HUMAN|HOXC4|HOX3E
0048706 // er	0030054 // ce	0043565 // se	Homeobox pr	HXC5_HUMAN|HOXC5|HOX3D
0006357 // re	0005829 // cy	0003700 // tra	Homeobox pr	HXC6_HUMAN|HOXC6|HOX3C
0000122 // ne	0015630 // m	0003700 // tra	Homeobox pr	HXC8_HUMAN|HOXC8|HOX3A
0048704 // er	0016235 // ag	0005515 // pr	Homeobox pr	HXC9_HUMAN|HOXC9|HOX3B
0006351 // tra	0005654 // nu	0043565 // se	Homeobox pr	HXD1_HUMAN|HOXD1|HOX4|HOX4G
0050905 // ne	0036464 // cy	0000977 // RN	Homeobox pr	HXD10_HUMAN|HOXD10|HOX4D|HOX4E
0001501 // sk	0005634 // nu	0000978 // RN	Homeobox pr	HXD13_HUMAN|HOXD13|HOX4I
0007160 // ce	0016235 // ag	0005515 // pr	Homeobox pr	HXD3_HUMAN|HOXD3|HOX1D|HOX4A
0007275 // m	0005634 // nu	0001228 // tra	Homeobox pr	HXD4_HUMAN|HOXD4|HOX4B
0000122 // ne	0005634 // nu	0001228 // tra	Homeobox pr	HXD8_HUMAN|HOXD8|HOX4E
0009954 // pr	0005730 // nu	0001227 // tra	Homeobox pr	HXD9_HUMAN|HOXD9|HOX4C
	0016020 // m	0005515 // pr	Hippocalcin-li	HPCL1_HUMAN|HPCAL1|BDR1
0006572 // ty		0046872 // m	4-hydroxyphe	HPDL_HUMAN|HPDL|GLOXD1
0007565 // fe	0070062 // ex	0004957 // pr	15-hydroxypro	PGDH_HUMAN|HPGD|PGDH1|SDR36C1
0043473 // pi	0031084 // BL	0005515 // pr	Hermansky-Pu	HPS3_HUMAN|HPS3
0006996 // or	0031084 // BL	0005515 // pr	Hermansky-Pu	HPS5_HUMAN|HPS5|AIBP63|KIAA1017
0051897 // po	0005578 // pr	0030305 // he	Heparanase	HPSE_HUMAN|HPSE|HEP|HPA|HPA1|HPR1|HPSE1|HSE
0030198 // ex	0005578 // pr	0030305 // he	Inactive hepar	HPSE2_HUMAN|HPSE2|HPA2

0006898 // re	0005576 // ex	0005515 // pr	Hemopexin	HEMO_HUMAN|HPX
0033169 // hi	0000785 // ch	0003700 // tra	Lysine-specific	HAIR_HUMAN|HR
0070374 // po	0005886 // pl	0005515 // pr	GTPase HRas	RASH_HUMAN|HRAS|HRAS1
0016042 // lip	0016021 // in	0016787 // hy	Phospholipid-	HRSL1_HUMAN|HRASLS
	0016021 // in		Histidine-rich	HRCT1_HUMAN|HRCT1|UNQ338/PRO537
0007186 // G-	0005886 // pl	0004969 // his	Histamine H4	HRH4_HUMAN|HRH4|GPCR105
0043065 // po	0016021 // in	0005515 // pr	Activator of a	HRK_HUMAN|HRK|BID3
0043163 // ce	0005576 // ex	0046914 // tra	Hornerin	HORN_HUMAN|HRNR|S100A18
0006024 // gly	0005796 // Go	0008467 // [h	Heparan sulfa	HS3S1_HUMAN|HS3ST1|3OST|3OST1
0006024 // gly	0000139 // Go	0008467 // [h	Heparan sulfa	HS3S5_HUMAN|HS3ST5|3OST5|HS3OST5
0006024 // gly	0000139 // Go	0017095 // he	Heparan-sulfa	H6ST1_HUMAN|HS6ST1|HS6ST
0006024 // gly	0016021 // in	0017095 // he	Heparan-sulfa	H6ST2_HUMAN|HS6ST2|PSEC0092
0016226 // iro	0005739 // m	0005515 // pr	Iron-sulfur clu	HSC20_HUMAN|HSCB|DNAJC20|HSC20
0030324 // lu	0016021 // in	0070524 // 11	Corticosteroid	DHI1_HUMAN|HSD11B1|HSD11|HSD11L|SDR26C1
0055114 // ox	0005576 // ex	0016491 // ox	Hydroxysteroi	DHI1L_HUMAN|HSD11B1L|HSD3|SCDR10|SDR26C2
0032868 // re	0005789 // er	0005496 // ste	Corticosteroid	DHI2_HUMAN|HSD11B2|HSD11K|SDR9C3
0007005 // m	0005759 // m	0003857 // 3-	3-hydroxyacyl	HCD2_HUMAN|HSD17B10|ERAB|HADH2|MRPP2|SCHAI
0006710 // an	0005576 // ex	0016229 // ste	Estradiol 17-b	DHB11_HUMAN|HSD17B11|DHRS8|PAN1B|SDR16C2|PS
0006706 // ste	0005829 // cy	0005515 // pr	17-beta-hydro	DHB14_HUMAN|HSD17B14|DHRS10|SDR3|SDR47C1|UN
0006702 // an	0005783 // en	0047035 // te	Testosterone	DHB3_HUMAN|HSD17B3|EDH17B3|SDR12C2
0006695 // ch	0016021 // in	0004303 // es	3-keto-steroid	DHB7_HUMAN|HSD17B7|SDR37C1|UNQ2563/PRO6243
0055114 // ox	0005759 // m	0005515 // pr	Estradiol 17-b	DHB8_HUMAN|HSD17B8|FABGL|HKE6|RING2|SDR30C1
0006699 // bi	0005789 // en	0005515 // pr	3 beta-hydrox	3BHS7_HUMAN|HSD3B7
0061408 // po	0005654 // nu	0003677 // DN	Heat shock fac	HSF1_HUMAN|HSF1|HSTF1
0007283 // sp	0005829 // cy	0005515 // pr	Heat shock fac	HSF2B_HUMAN|HSF2BP
0043010 // ca	0016607 // nu	0043565 // se	Heat shock fac	HSF4_HUMAN|HSF4
0006351 // tra	0005634 // nu	0043565 // se	Heat shock tra	HSFX1_HUMAN|HSFX1|HSFX2|LW-1
0009967 // po	0005829 // cy	0005515 // pr	Hematopoieti	HSH2D_HUMAN|HSH2D|ALX
0046677 // re	0005829 // cy	0005515 // pr	Heat shock pr	HS90A_HUMAN|HSP90AA1|HSP90A|HSPC1|HSPCA
0006950 // re	0005737 // cy	0051082 // un	Putative heat	HS905_HUMAN|HSP90AA5P|HSP90AE
		0005515 // pr	Heat shock 70	HS12B_HUMAN|HSPA12B|C20orf60
1900034 // re	0005737 // cy	0003723 // RN	Heat shock 70	HS71A_HUMAN|HSPA1A|HSP72|HSPA1|HSX70
0051131 // ch	0070062 // ex	0005515 // pr	Heat shock 70	HSP74_HUMAN|HSPA4|APG2
0006983 // ER	0005925 // fo	0005515 // pr	78 kDa glucos	GRP78_HUMAN|HSPA5|GRP78
0006986 // re	0005737 // cy	0031072 // he	Heat shock 70	HSP76_HUMAN|HSPA6|HSP70B'
0008150 // bi	0070062 // ex	0005524 // AT	Putative heat	HSP77_HUMAN|HSPA7|HSP70B
1900034 // re	0005829 // cy	0005515 // pr	Heat shock co	HSP7C_HUMAN|HSPA8|HSC70|HSP73|HSPA10
0006457 // pr	0042645 // m	0005515 // pr	Stress-70 prot	GRP75_HUMAN|HSPA9|GRP75|HSPA9B|mt-HSP70
1902176 // ne	0005737 // cy	0005515 // pr	Heat shock pr	HSPB1_HUMAN|HSPB1|HSP27|HSP28
0045766 // po	0005737 // cy	0042803 // pr	Heat shock pr	HSPB6_HUMAN|HSPB6
0008016 // re	0005737 // cy	0005515 // pr	Heat shock pr	HSPB7_HUMAN|HSPB7|CVHSP
1900034 // re	0005737 // cy	0005515 // pr	Heat shock pr	HSPB8_HUMAN|HSPB8|CRYAC|E2IG1|HSP22|PP1629

0032755 // pc	0005743 // m	0044183 // pr	60 kDa heat sl	CH60_HUMAN|HSPD1|HSP60
0006986 // re	0005759 // m	0003723 // RI	10 kDa heat sl	CH10_HUMAN|HSPE1
0016525 // ne	0005886 // pl	0005515 // pr	Basement me	PGBM_HUMAN|HSPG2
0006357 // re	0005634 // nu	0005515 // pr	Oxidoreducta:	HTAI2_HUMAN|HTATIP2|CC3|TIP30
0019079 // vi	0005634 // nu	0003723 // RI	HIV Tat-specif	HTSF1_HUMAN|HTATSF1
0050795 // re	0005887 // in	0030594 // ne	5-hydroxytryp	5HT1B_HUMAN|HTR1B|HTR1DB
0007193 // ac	0005886 // pl	0004993 // G-	5-hydroxytryp	5HT1D_HUMAN|HTR1D|HTR1DA|HTRL
0007631 // fe	0005886 // pl	0051378 // se	5-hydroxytryp	5HT2C_HUMAN|HTR2C|HTR1C
0007186 // G-	0005886 // pl	0001594 // tr	5-hydroxytryp	5HT4R_HUMAN|HTR4
0042310 // va	0005886 // pl	0030594 // ne	5-hydroxytryp	5HT7R_HUMAN|HTR7
0001890 // pl	0005615 // ex	0008236 // se	Serine protea:	HTRA1_HUMAN|HTRA1|HTRA|PRSS11
0043065 // pc	0005739 // m	0008233 // pe	Serine protea:	HTRA2_HUMAN|HTRA2|OMI|PRSS25
0006508 // pr	0005576 // ex	0004175 // er	Serine protea:	HTRA3_HUMAN|HTRA3|PRSP
0006508 // pr	0005576 // ex	0004175 // er	Serine protea:	HTRA4_HUMAN|HTRA4
0048513 // ar	0005783 // er	0005515 // pr	Huntingtin	HD_HUMAN|HTT|HD|IT15
0031573 // in	0005634 // nu	0005515 // pr	Checkpoint pr	HUS1_HUMAN|HUS1
0010043 // re	0035579 // sp	0030171 // vc	Voltage-gated	HVCN1_HUMAN|HVCN1|VSOP|UNQ578/PRO1140
0045785 // pc	0005737 // cy	0004415 // hy	Hyaluronidase	HYAL1_HUMAN|HYAL1|LUCA1
0071493 // ce	0005576 // ex	0005515 // pr	Hyaluronidase	HYAL3_HUMAN|HYAL3|LUCA3
0030207 // ch	0016021 // in	0004415 // hy	Hyaluronidase	HYAL4_HUMAN|HYAL4
1904158 // ax	1990718 // ax		Hydrocephalu	HYDIN_HUMAN|HYDIN|HYDIN1|KIAA1864
0008150 // bi	0005575 // ce	0008903 // hy	Putative hydr	HYI_HUMAN|HYI|HT036|SB156
0016310 // ph	0005759 // m	0047992 // hy	Hydroxylysine	HYKK_HUMAN|HYKK|AGPHD1
0060271 // cil	0005829 // cy	0005515 // pr	Hydrolethalus	HYLS1_HUMAN|HYLS1|HLS
0016042 // lip	0070062 // ex	0016787 // hy	Isoamyl aceta	IAH1_HUMAN|IAH1
0042755 // ea	0005576 // ex	0001540 // be	Islet amyloid	IAPP_HUMAN|IAPP
0006418 // tR	0005739 // m	0004822 // isc	Isoleucine--tR	SYIM_HUMAN|IARS2
0016226 // irc	0005739 // m	0003723 // RI	Putative trans	CAF17_HUMAN|IBA57|C1orf69
0030198 // ex	0031012 // ex	0003674 // m	Bone sialopro	SIAL_HUMAN|IBSP|BNSP
0006836 // ne	0030667 // se	0005515 // pr	Islet cell auto	ICA69_HUMAN|ICA1
0007286 // sp	0001669 // ac	0005515 // pr	Islet cell auto	ICA1L_HUMAN|ICA1L|ALS2CR14|ALS2CR15
0046813 // re	0070062 // ex	0001618 // vi	Intercellular a	ICAM1_HUMAN|ICAM1
0002223 // sti	0001931 // ur	0005178 // in	Intercellular a	ICAM2_HUMAN|ICAM2
0002223 // sti	0070062 // ex	0005515 // pr	Intercellular a	ICAM3_HUMAN|ICAM3
0050776 // re	0005886 // pl	0005515 // pr	Intercellular a	ICAM5_HUMAN|ICAM5|TLCN|TLN
0035721 // in	0097542 // cil	0004674 // pr	Serine/threon	ICK_HUMAN|ICK|KIAA0936
0007165 // sig	0016021 // in	0005102 // re	ICOS ligand	ICOSL_HUMAN|ICOSLG|B7H2|B7RP1|ICOSL|KIAA0653
0050679 // pc	0005794 // Gc	0008134 // tr	DNA-binding	ID1_HUMAN|ID1|BHLHB24|ID
0019216 // re	0043234 // pr	0005515 // pr	DNA-binding	ID2_HUMAN|ID2|BHLHB26
0007417 // ce	0005737 // cy	0005515 // pr	DNA-binding	ID3_HUMAN|ID3|1R21|BHLHB25|HEIR1
0000122 // ne	0005737 // cy	0003714 // tr	DNA-binding	ID4_HUMAN|ID4|BHLHB27
0006102 // isc	0005739 // m	0004450 // isc	Isocitrate deh	IDHP_HUMAN|IDH2
0050992 // di	0005829 // cy	0016787 // hy	Isopentenyl-d	IDI1_HUMAN|IDI1

0016310 // ph		0005524 // AT	Probable gluc	GNTK_HUMAN|IDNK|C9orf103
0042130 // ne	0032421 // st	0004833 // try	Indoleamine 2	I23O1_HUMAN|IDO1|IDO|INDO
0008152 // m	0043202 // lys	0008484 // su	Iduronate 2-su	B3KWA1_HUMAN|IDS|hCG_1993933
0005984 // di	0043202 // lys	0003940 // L-i	Alpha-L-iduro	IDUA_HUMAN|IDUA
0009653 // ar	0005634 // nu	0005515 // pr	Radiation-indu	IEX1_HUMAN|IER3|DIF2|IEX1|PRG1
0006888 // EF	0005794 // Go		Immediate ea	IR3IP_HUMAN|IER3IP1|HSPC039
	0005882 // int	0005198 // str	Intermediate	IFFO1_HUMAN|IFFO1|IFFO
	0005882 // int	0005198 // str	Intermediate	IFFO2_HUMAN|IFFO2
0000122 // ne	0005634 // nu	0001078 // tra	Gamma-interf	IF16_HUMAN|IFI16|IFNGIP1
0000122 // ne	0016021 // int	0001102 // RN	Interferon alp	IFI27_HUMAN|IFI27
	0016021 // int		Interferon alp	I27L2_HUMAN|IFI27L2|FAM14A|TLH29
0042590 // ar	0005764 // lys	0016667 // ox	Gamma-interf	GILT_HUMAN|IFI30|GILT|IP30
0060337 // ty	0005829 // cy	0005515 // pr	Interferon-inc	IN35_HUMAN|IFI35|IFP35
0009615 // re	0005737 // cy		Interferon-inc	IFI44_HUMAN|IFI44|MTAP44
0051607 // de	0005737 // cy	0003674 // m	Interferon-inc	IF44L_HUMAN|IFI44L|C1orf29|GS3686
0043154 // ne	0016021 // int	0005515 // pr	Interferon alp	IFI6_HUMAN|IFI6|G1P3
0009597 // de	0005829 // cy	0005515 // pr	Interferon-inc	IFIH1_HUMAN|IFIH1|MDA5|RH116
0032091 // ne	0005737 // cy	0005515 // pr	Interferon-inc	IFIT1_HUMAN|IFIT1|G10P1|IFI56|IFNAI1|ISG56
0035457 // ce	0005737 // cy	0005515 // pr	Interferon-inc	IFIT3_HUMAN|IFIT3|CIG-49|IFI60|IFIT4|ISG60
0046597 // ne	0005886 // pl	0005057 // sig	Interferon-inc	IFM1_HUMAN|IFITM1|CD225|IFI17
0009607 // re	0016021 // int		Interferon-inc	IFM10_HUMAN|IFITM10
0042100 // B	0005576 // ex	0005125 // cy	Interferon alp	IFNA1_HUMAN|IFNA1|IFNA13
0042100 // B	0005615 // ex	0005125 // cy	Interferon eps	IFNE_HUMAN|IFNE|IFNE1|UNQ360/PRO655
0034342 // re	0005886 // pl	0005515 // pr	Interferon lam	INLR1_HUMAN|IFNLR1|IL28RA|LICR2
0035735 // int	0005768 // en	0005515 // pr	Intraflagellar t	IFT27_HUMAN|IFT27|RABL4|RAYL
0035721 // int	0097542 // cil	0005515 // pr	Intraflagellar t	IFT43_HUMAN|IFT43|C14orf179
0001841 // ne	0005813 // ce	0008022 // pr	Intraflagellar t	IFT52_HUMAN|IFT52|C20orf9|NGD5|CGI-53
0050680 // ne	0097542 // cil	0005515 // pr	Intraflagellar t	IFT74_HUMAN|IFT74|CCDC2|CMG1
0035735 // int	0030992 // int	0005515 // pr	Intraflagellar t	IFT80_HUMAN|IFT80|KIAA1374|WDR56
	0016021 // int		Immunoglobu	IGDC4_HUMAN|IGDCC4|DDM36|KIAA1628|NOPE
0038083 // pe	0005886 // pl	0005515 // pr	Insulin-like gro	IGF1R_HUMAN|IGF1R
0006349 // re	0005886 // pl	0005515 // pr	Insulin-like gro	IGF2_HUMAN|IGF2|PP1446
0097150 // ne	0030175 // fil	0003723 // RN	Insulin-like gro	IF2B1_HUMAN|IGF2BP1|CRDBP|VICKZ1|ZBP1
0007568 // ag	0005794 // Go	0005102 // re	Insulin-like gro	IBP1_HUMAN|IGFBP1|IBP1
0007584 // re	0005576 // ex	0005102 // re	Insulin-like gro	IBP2_HUMAN|IGFBP2|BP2|IBP2
0006915 // ap	0005576 // ex	0001968 // fib	Insulin-like gro	IBP3_HUMAN|IGFBP3|IBP3
0043567 // re	0042568 // ins	0031995 // ins	Insulin-like gro	IBP6_HUMAN|IGFBP6|IBP6
0007155 // ce	0031012 // ex	0005515 // pr	Insulin-like gro	IBP7_HUMAN|IGFBP7|MAC25|PSF
	0005615 // ex	0005515 // pr	Insulin growth	IGFL1_HUMAN|IGFL1|UNQ644/PRO1274
0008150 // bi	0005634 // nu	0005515 // pr	Immunoglobu	IGFN1_HUMAN|IGFN1|EEF1A2BP1|KYIP1
0050853 // B	0072562 // bl	0003823 // an	Immunoglobu	HV145_HUMAN|IGHV1-45
	0005576 // ex		IgA-inducing p	IGIP_HUMAN|IGIP|C5orf53

0050853 // B	0016020 // m	0003823 // ar	Immunoglobu	IGLL1_HUMAN	IGLL1|IGL1
	0005576 // ex		IgLON family r	IGLO5_HUMAN	IGLON5
0006355 // re	0016021 // in	0034711 // in	Immunoglobu	IGSF1_HUMAN	IGSF1|IGDC1|KIAA0364|PGSF2
2001222 // re	0005576 // ex		Immunoglobu	IGS10_HUMAN	IGSF10|CMF608
0007155 // ce	0016021 // in		Immunoglobu	IGS11_HUMAN	IGSF11|BTIGSF|CXADRL1|VSIG3
	0016021 // in		Immunoglobu	IGS23_HUMAN	IGSF23
0032808 // la	0005886 // pl	0003674 // m	Immunoglobu	IGSF3_HUMAN	IGSF3|EWI3|KIAA0466
0006928 // m	0070062 // ex	0005515 // pr	Immunoglobu	IGSF8_HUMAN	IGSF8|CD81P3|EWI2|KCT4
0007156 // ho	0016021 // in	0098632 // pr	Protein turtle	TUTLA_HUMAN	IGSF9|IGSF9A|KIAA1355|NRT1
0046639 // ne	0005578 // pr	0008233 // pe	Indian hedgeh	IHH_HUMAN	IHH
0034501 // pr	0000228 // nu	0005515 // pr	Protein Red	RED_HUMAN	IK|RED|RER
0010165 // re	0016021 // in	0005515 // pr	Inhibitor of nu	IKIP_HUMAN	IKBIP|IKIP
0007252 // I-k	0005634 // nu	0005515 // pr	Inhibitor of nu	IKKE_HUMAN	IKBKE|IKKE|IKKI|KIAA0151
0000122 // ne	0005634 // nu	0044212 // tra	Zinc finger pro	IKZF2_HUMAN	IKZF2|HELIOS|ZNFN1A2
0006954 // in	0005886 // pl	0004872 // re	Interleukin-10	I10R2_HUMAN	IL10RB|CRFB4|D21S58|D21S66
0030183 // B	0005576 // ex	0005515 // pr	Interleukin-11	IL11_HUMAN	IL11
0045582 // po	0043514 // in	0042163 // in	Interleukin-12	IL12A_HUMAN	IL12A|NKSF1
0071305 // ce	0005576 // ex	0005126 // cy	Interleukin-15	IL15_HUMAN	IL15
0019221 // cy	0005615 // ex	0004896 // cy	Interleukin-15	I15RA_HUMAN	IL15RA
0006955 // im	0005829 // cy	0042609 // CD	Pro-interleuki	IL16_HUMAN	IL16
0032725 // po	0005615 // ex	0005125 // cy	Interleukin-17	IL17D_HUMAN	IL17D|UNQ3096/PRO21175
0019221 // cy	0005737 // cy	0004896 // cy	Interleukin-17	I17RB_HUMAN	IL17RB|CRL4|EVI27|IL17BR|UNQ2501/P
1900017 // po	0009986 // ce	0005102 // re	Interleukin-17	I17RC_HUMAN	IL17RC|UNQ6118/PRO20040/PRO38901
0019221 // cy	0005794 // Go	0030368 // in	Interleukin-17	I17RD_HUMAN	IL17RD|IL17RLM|SEF|UNQ6115/PRO20
0019221 // cy	0005576 // ex	0005515 // pr	Interleukin-17	I17RE_HUMAN	IL17RE|UNQ3056/PRO9877
0032496 // re	0005615 // ex	0048019 // re	Interleukin-18	I18BP_HUMAN	IL18BP
0007165 // sig	0005886 // pl	0005515 // pr	Interleukin-18	IL18R_HUMAN	IL18R1|IL1RRP
0010575 // po	0005576 // ex	0005149 // in	Interleukin-1 a	IL1A_HUMAN	IL1A|IL1F1
0007267 // ce	0005576 // ex	0005149 // in	Interleukin-1 b	IL1B_HUMAN	IL1B|IL1F2
0050727 // re	0005886 // pl	0004888 // tra	Interleukin-1 r	IL1R1_HUMAN	IL1R1|IL1R|IL1RA|IL1RT1
0006955 // im	0016021 // in	0004910 // in	Interleukin-1 r	IL1R2_HUMAN	IL1R2|IL1RB
0006955 // im	0005886 // pl	1990782 // pr	Interleukin-1 r	IL1AP_HUMAN	IL1RAP|C3orf13|IL1R3
0097105 // pr	0005886 // pl	0019966 // in	Interleukin-1 r	IRPL1_HUMAN	IL1RAPL1|OPHN4
0070498 // in	0005887 // in	0004908 // in	Interleukin-1 r	ILRL2_HUMAN	IL1RL2|IL1RRP2
0051384 // re	0005615 // ex	0005151 // in	Interleukin-1 r	IL1RA_HUMAN	IL1RN|IL1F3|IL1RA
0019221 // cy	0005886 // pl	0005515 // pr	Interleukin-20	I20RA_HUMAN	IL20RA|UNQ681/PRO1315
0050829 // de	0016021 // in	0005515 // pr	Interleukin-22	I22R1_HUMAN	IL22RA1|IL22R
2000330 // po	0005576 // ex	0045519 // in	Interleukin-23	IL23A_HUMAN	IL23A|SGRF|UNQ2498/PRO5798
0071353 // ce	0005615 // ex	0005515 // pr	Interleukin-24	IL24_HUMAN	IL24|MDA7|ST16
0007166 // ce	0005887 // in	0004888 // tra	Interleukin-27	I27RA_HUMAN	IL27RA|CRL1|TCCR|WSX1|UNQ296/PRO
0043547 // po	0005886 // pl	0004911 // in	Interleukin-2 r	IL2RB_HUMAN	IL2RB|IL15RB
0043547 // po	0005886 // pl	0004911 // in	Cytokine rece	IL2RG_HUMAN	IL2RG
0008284 // po	0005886 // pl	0019901 // pr	Interleukin-31	IL31R_HUMAN	IL31RA|CRL3|GPL|UNQ6368/PRO21073/
0006952 // de	0005615 // ex	0005515 // pr	Interleukin-32	IL32_HUMAN	IL32|NK4|TAIF
0032436 // po	0005829 // cy	0005515 // pr	Interleukin-33	IL33_HUMAN	IL33|C9orf26|IL1F11|NFHEV
0045087 // in	0005576 // ex	0008083 // gr	Interleukin-34	IL34_HUMAN	IL34|C16orf77

0019221 // cy	0005576 // ex	0005149 // in	Interleukin-36	IL36G_HUMAN	IL36G|IL1E|IL1F9|IL1H1|IL1RP2|UNQ24
0032715 // ne	0005615 // ex	0005149 // in	Interleukin-36	I36RA_HUMAN	IL36RN|FIL1D|IL1F5|IL1HY1|IL1L1|IL1RI
1901741 // po	0005886 // pl	0005057 // sig	Interleukin-4 r	IL4RA_HUMAN	IL4R|IL4RA|582J2.1
0045944 // po	0005576 // ex	0005138 // in	Interleukin-6	IL6_HUMAN	IL6|IFNB2
0034097 // re	0005576 // ex	0019981 // in	Interleukin-6 r	IL6RA_HUMAN	IL6R
0033089 // po	0005886 // pl	0004917 // in	Interleukin-7 r	IL7RA_HUMAN	IL7R
0038113 // in	0005886 // pl	0004919 // in	Interleukin-9 r	IL9R_HUMAN	IL9R
1990830 // ce	0016021 // in	0070506 // hi	Immunoglobu	ILDR1_HUMAN	ILDR1
0006351 // tra	0030529 // in	0003723 // RN	Interleukin en	ILF3_HUMAN	ILF3|DRBF|MPHOSPH4|NF90
0018105 // pe	0001725 // st	0005515 // pr	Integrin-linked	ILK_HUMAN	ILK|ILK1|ILK2
0006470 // pr	0005737 // cy	0005515 // pr	Integrin-linked	ILKAP_HUMAN	ILKAP
0022904 // re	0016021 // in	0008236 // se	Mitochondria	IMP2L_HUMAN	IMMP2L
0006364 // rR	0005654 // nu	0005515 // pr	U3 small nucle	IMP4_HUMAN	IMP4|BXDC4
0006796 // ph	0005737 // cy	0052833 // in	Inositol mono	IMPA2_HUMAN	IMPA2|IMP.18P
0006183 // G	0005737 // cy	0005515 // pr	Inosine-5'-mo	IMDH2_HUMAN	IMPDH2|IMPD2
0007601 // vis	0005578 // pr	0005201 // ex	Interphotorec	IMPG1_HUMAN	IMPG1|IPM150|SPACR
1990830 // ce	0005883 // ne	0005200 // st	Alpha-interne	AINX_HUMAN	INA|NEF5
2001235 // po	0016604 // nu	0005515 // pr	Protein INCA1	INCA1_HUMAN	INCA1|HSD45
0007049 // ce	0005634 // nu	0035064 // m	Inhibitor of gr	ING1_HUMAN	ING1
0007165 // sig	0034673 // in	0046982 // pr	Inhibin alpha	INHA_HUMAN	INHA
0045944 // po	0043512 // in	0005125 // cy	Inhibin beta A	INHBA_HUMAN	INHBA
0006952 // de	0005576 // ex	0005179 // ho	Inhibin beta B	INHBB_HUMAN	INHBB
0042981 // re	0070062 // ex	0008083 // gr	Inhibin beta C	INHBC_HUMAN	INHBC
0042981 // re	0005615 // ex	0008083 // gr	Inhibin beta E	INHBE_HUMAN	INHBE
0006310 // DI	0031011 // In	0005515 // pr	INO80 comple	IN80E_HUMAN	INO80E|CCDC95
0043647 // in	0005829 // cy	0052829 // in	Inositol polyp	INPP_HUMAN	INPP1
0007165 // sig	0005737 // cy	0016316 // ph	Type II inosito	INP4B_HUMAN	INPP4B
0007165 // sig	0005829 // cy	0004445 // in	Phosphatidyli	SHIP1_HUMAN	INPP5D|SHIP|SHIP1
0043647 // in	0005829 // cy	0004445 // in	Phosphatidyli	PI5PA_HUMAN	INPP5J|PIB5PA|PIPP
0051926 // ne	0001726 // ru	0005515 // pr	Inositol polyp	INP5K_HUMAN	INPP5K|PPS|SKIP
0007399 // ne	0043234 // pr	0005515 // pr	Protein inscut	INSC_HUMAN	INSC
0045599 // ne	0005789 // en	0005515 // pr	Insulin-induce	INSI1_HUMAN	INSIG1
0007267 // ce	0005576 // ex	0005102 // re	Insulin-like 3	INSL3_HUMAN	INSL3|RLF|RLNL
0051290 // pr	0005887 // in	0005009 // in	Insulin recept	INSR_HUMAN	INSR
0042795 // sn	0005654 // nu		Integrator cor	INT1_HUMAN	INTS1|KIAA1440|UNQ1821/PRO3434
0042795 // sn	0005654 // nu	0005515 // pr	Integrator cor	INT10_HUMAN	INTS10|C8orf35
0007052 // m	0005634 // nu	0005515 // pr	Integrator cor	INT13_HUMAN	INTS13|ASUN|C12orf11|GCT1
0042795 // sn	0005654 // nu	0005515 // pr	Integrator cor	INT2_HUMAN	INTS2|KIAA1287
0071479 // ce	0005654 // nu	0005515 // pr	Integrator cor	INT7_HUMAN	INTS7|C1orf73
0042795 // sn	0005654 // nu	0005515 // pr	Integrator cor	INT8_HUMAN	INTS8|C8orf52
0042795 // sn	0005654 // nu	0005515 // pr	Integrator cor	INT9_HUMAN	INTS9|RC74
0030216 // ke	0036064 // cil	0005515 // pr	Protein inturn	INTU_HUMAN	INTU|KIAA1284|PDZD6|PDZK6

0043647 // in	0005829 // cy	0000827 // in	Inositol hexak	IP6K1_HUMAN|IP6K1|IHPK1|KIAA0263
0043647 // in	0005829 // cy	0052836 // in	Inositol hexak	IP6K3_HUMAN|IP6K3|IHPK3
0015671 // ox	0005829 // cy	0019904 // pr	Interactor pro	ICEF1_HUMAN|IPCEF1|KIAA0403
0006610 // rib	0005635 // nu	0005515 // pr	Importin-11	IPO11_HUMAN|IPO11|RANBP11
0006336 // DI	0005643 // nu	0005515 // pr	Importin-4	IPO4_HUMAN|IPO4|IMP4B|RANBP4
0006607 // NI	0005730 // nu	0005515 // pr	Importin-5	IPO5_HUMAN|IPO5|KPNB3|RANBP5
0016032 // vi	0005829 // cy	0005515 // pr	Importin-7	IPO7_HUMAN|IPO7|RANBP7
0006606 // pr	0005635 // nu	0005515 // pr	Importin-9	IPO9_HUMAN|IPO9|IMP9|KIAA1192|RANBP9|HSPC273
		0005524 // AT	IQ and AAA do	IQCA1_HUMAN|IQCA1|IQCA
0048496 // m	0005829 // cy	0005515 // pr	IQ calmodulin	IQCB1_HUMAN|IQCB1|KIAA0036|NPHP5|OK/SW-cl.85
	0060170 // cil	0005515 // pr	IQ domain-co	IQCE_HUMAN|IQCE|KIAA1023
0072015 // gl	0005884 // ac	0005515 // pr	Ras GTPase-ac	IQGA1_HUMAN|IQGAP1|KIAA0051
0034314 // Ar	0070062 // ex	0048365 // Ra	Ras GTPase-ac	IQGA2_HUMAN|IQGAP2
0032012 // re	0005737 // cy	0005086 // AR	IQ motif and S	IQEC2_HUMAN|IQSEC2|KIAA0522
0032012 // re	0045211 // po	0005086 // AR	IQ motif and S	IQEC3_HUMAN|IQSEC3|KIAA1110
0071456 // ce	0005829 // cy	0005515 // pr	Interleukin-1 r	IRAK1_HUMAN|IRAK1|IRAK
0001960 // ne	0005634 // nu	0042803 // pr	Interleukin-1 r	IRAK3_HUMAN|IRAK3
0060333 // in	0005829 // cy	0005515 // pr	Interferon reg	IRF1_HUMAN|IRF1
0007596 // bl	0005829 // cy	0000977 // RN	Interferon reg	IRF2_HUMAN|IRF2
0006351 // tra	0005654 // nu	0005515 // pr	Interferon reg	I2BP1_HUMAN|IRF2BP1
0045944 // po	0005829 // cy	0000975 // re	Interferon reg	IRF5_HUMAN|IRF5
0060644 // m	0005829 // cy	0005515 // pr	Interferon reg	IRF6_HUMAN|IRF6
0009615 // re	0005829 // cy	0000982 // tra	Interferon reg	IRF7_HUMAN|IRF7
		0005515 // pr	Immunity-rela	IRGQ_HUMAN|IRGQ|IRGQ1|FKSG27
0045725 // po	0005829 // cy	0005515 // pr	Insulin recept	IRS1_HUMAN|IRS1
0045725 // po	0005829 // cy	0005515 // pr	Insulin recept	IRS2_HUMAN|IRS2
0008286 // in	0005829 // cy	0005070 // SH	Insulin recept	IRS4_HUMAN|IRS4
0072272 // pr	0005634 // nu	0001227 // tra	Iroquois-class	IRX2_HUMAN|IRX2|IRXA2
0001656 // m	0005737 // cy	0043565 // se	Iroquois-class	IRX3_HUMAN|IRX3|IRXB1
0008406 // go	0005634 // nu	0043565 // se	Iroquois-class	IRX5_HUMAN|IRX5|IRX2A|IRXB2
0044281 // sm	0005739 // m	0005198 // str	Iron-sulfur clu	ISCA2_HUMAN|ISCA2|HBLD1
0044281 // sm	0005739 // m	0005506 // iro	Iron-sulfur clu	ISCU_HUMAN|ISCU|NIFUN
0032649 // re	0005654 // nu	0031386 // pr	Ubiquitin-like	ISG15_HUMAN|ISG15|G1P2|UCRP
0033147 // ne	0005634 // nu	0030331 // es	Insulin gene e	ISL1_HUMAN|ISL1
0021520 // sp	0005634 // nu	0043565 // se	Insulin gene e	ISL2_HUMAN|ISL2
	0005576 // ex		Isthmin-1	ISM1_HUMAN|ISM1|C20orf82|ISM
0048672 // po	0070062 // ex	0005515 // pr	IST1 homolog	IST1_HUMAN|IST1|KIAA0174
0000398 // m	0005654 // nu	0003723 // RN	Pre-mRNA-spl	ISY1_HUMAN|ISY1|KIAA1160
0043647 // in	0005737 // cy	0004512 // in	Inositol-3-pho	INO1_HUMAN|ISYNA1|INO1
	0016021 // in		T-cell immunc	TIP_HUMAN|ITFG1|LNKN-1|TIP|CDA08
0008285 // ne	0005925 // fo	0005515 // pr	Integrin alpha	ITA1_HUMAN|ITGA1
0007160 // ce	0005886 // pl	0098639 // co	Integrin alpha	ITA10_HUMAN|ITGA10|UNQ468/PRO827
0006929 // su	0005886 // pl	0005518 // co	Integrin alpha	ITA11_HUMAN|ITGA11|MSTP018

0002576 // pl	0005886 // pl	0005515 // pr	Integrin alpha	ITA2B_HUMAN|ITGA2B|GP2B|ITGAB
0031345 // ne	0005886 // pl	0046982 // pr	Integrin alpha	ITA3_HUMAN|ITGA3|MSK18
0043547 // pc	0005886 // pl	0005515 // pr	Integrin alpha	ITA6_HUMAN|ITGA6
0035987 // er	0005886 // pl	0005515 // pr	Integrin alpha	ITA7_HUMAN|ITGA7|UNQ406/PRO768
0007229 // in	0009986 // ce	0005515 // pr	Integrin alpha	ITAM_HUMAN|ITGAM|CD11B|CR3A
0034446 // su	0005886 // pl	0005515 // pr	Integrin alpha	ITAV_HUMAN|ITGAV|MSK8|VNRA|VTNR
0007219 // N	0005925 // fo	0005515 // pr	Integrin beta-	ITBP1_HUMAN|ITGB1BP1|ICAP1
0034446 // su	0005886 // pl	0005515 // pr	Integrin beta-	ITB3_HUMAN|ITGB3|GP3A
0006914 // au	0005886 // pl	0005515 // pr	Integrin beta-	ITB4_HUMAN|ITGB4
0035987 // er	0005886 // pl	0001618 // vi	Integrin beta-	ITB5_HUMAN|ITGB5
0007229 // in	0005886 // pl	0001618 // vi	Integrin beta-	ITB6_HUMAN|ITGB6
0007229 // in	0009986 // ce	0005515 // pr	Integrin beta-	ITB7_HUMAN|ITGB7
0007155 // ce	0005615 // ex		Integrin beta-	ITGBL_HUMAN|ITGBL1|OSCP|TIED
0030212 // hy	0005576 // ex	0004867 // se	Inter-alpha-tr	ITIH2_HUMAN|ITIH2|IGHEP2
0002576 // pl	0005576 // ex	0004867 // se	Inter-alpha-tr	ITIH3_HUMAN|ITIH3
0002576 // pl	0005576 // ex	0004867 // se	Inter-alpha-tr	ITIH4_HUMAN|ITIH4|IHRP|ITIHL1|PK120|PRO1851
0030212 // hy	0005576 // ex	0004867 // se	Inter-alpha-tr	ITIH6_HUMAN|ITIH6|ITIH5L|UNQ6369/PRO21074
0042985 // ne	0005886 // pl	0005515 // pr	Integral meml	ITM2A_HUMAN|ITM2A|UNQ603/PRO1189
0042985 // ne	0005886 // pl	0005515 // pr	Integral meml	ITM2B_HUMAN|ITM2B|BRI|BRI2
0006020 // in	0005829 // cy	0008440 // in	Inositol-trisph	IP3KA_HUMAN|ITPKA
0016310 // ph	0005829 // cy	0008440 // in	Inositol-trisph	IP3KC_HUMAN|ITPKC|IP3KC
0051209 // re	0031088 // pl	0005509 // ca	Inositol 1,4,5-	ITPR1_HUMAN|ITPR1|INSP3R1
0007613 // m	0005783 // er	0005515 // pr	Inositol 1,4,5-	ITPR3_HUMAN|ITPR3
	0016021 // in	0005515 // pr	Inositol 1,4,5-	IPIL1_HUMAN|ITPRIPL1|KIAA1754L
	0016021 // in		Inositol 1,4,5-	IPIL2_HUMAN|ITPRIPL2
0006897 // er	0070062 // ex	0005089 // Rh	Intersectin-2	ITSN2_HUMAN|ITSN2|KIAA1256|SH3D1B|SWAP
0018149 // pe	0005829 // cy	0005198 // st	Involucrin	INVO_HUMAN|IVL
0016032 // vi	0015629 // ac		Influenza virus	NS1BP_HUMAN|IVNS1ABP|ARA3|FLARA3|KIAA0850|NS
0034113 // he	0016021 // in	0086080 // pr	Izumo sperm-	IZUM1_HUMAN|IZUMO1
0043984 // hi	0000123 // hi	0005515 // pr	E3 ubiquitin-p	JADE2_HUMAN|JADE2|KIAA0239|PHF15
0007049 // ce	0005887 // in	0005112 // N	Protein jagged	JAG2_HUMAN|JAG2
0048008 // pl	0005829 // cy	0004672 // pr	Tyrosine-prot	JAK2_HUMAN|JAK2
0038083 // pe	0005829 // cy	0005515 // pr	Tyrosine-prot	JAK3_HUMAN|JAK3
	0005794 // G	0005515 // pr	Janus kinase a	JKIP2_HUMAN|JAKMIP2|JAMIP2|KIAA0555|NECC1
	0005794 // G	0019900 // ki	Janus kinase a	JKIP3_HUMAN|JAKMIP3|C10orf14|C10orf39|JAMIP3|N
0007162 // ne	0005886 // pl	0046982 // pr	Junctional adh	JAM2_HUMAN|JAM2|C21orf43|VEJAM|UNQ219/PRO2
0007286 // sp	0030057 // de	0042803 // pr	Junctional adh	JAM3_HUMAN|JAM3|UNQ859/PRO1868
0031061 // ne	0035098 // ES	0001227 // tr	Protein Jumor	JARD2_HUMAN|JARID2|JMJ
0006629 // lip	0005829 // cy	0003714 // tr	Juxtaposed wi	JAZF1_HUMAN|JAZF1|TIP27|ZNF802
0045599 // ne	0005634 // nu	0001078 // tr	Jun dimerizati	JDP2_HUMAN|JDP2
0007596 // bl	0000785 // ch	0032454 // hi	Probable JmjC	JHD2C_HUMAN|JMJD1C|JHDM2C|KIAA1380|TRIP8
		0005515 // pr	JmjC domain-	JMJD7_HUMAN|JMJD7
0060314 // re	0016021 // in	0008307 // st	Junctophilin-1	JPH1_HUMAN|JPH1|JP1

0051209 // re	0016021 // in	0080025 // ph	Junctophilin-2	JPH2_HUMAN	JPH2|JP2
0007613 // m	0016021 // in	0003674 // m	Junctophilin-3	JPH3_HUMAN	JPH3|JP3|TNRC22
0043922 // ne	0005654 // nu	0070412 // R-	Transcription	JUN_HUMAN|JUN	
0000122 // ne	0000790 // nu	0005515 // pr	Transcription	JUNB_HUMAN|JUNB	
0086091 // re	0005886 // pl	0005515 // pr	Junction plako	PLAK_HUMAN	JUP|CTNNG|DP3
0033147 // ne	0005739 // m	0005515 // pr	KN motif and	KANK2_HUMAN|KANK2|ANKRD25|KIAA1518|MXRA3|SI	
	0000123 // hi		KAT8 regulato	KAL1L_HUMAN|KANSL1L|C2orf67	
0043982 // hi	0000123 // hi	0046972 // hi	KAT8 regulato	KANL3_HUMAN|KANSL3|KIAA1310|NSL3|PRTD|SI1	
0043970 // hi	0031672 // A	0005515 // pr	Histone acety	KAT2B_HUMAN|KAT2B|PCAF	
0045893 // po	0005634 // nu	0005515 // pr	Histone acety	KAT6A_HUMAN|KAT6A|MOZ|MYST3|RUNXBP2|ZNF220	
0051301 // ce	0000922 // sp	0046982 // pr	Katanin p60 A	KTNA1_HUMAN|KATNA1	
0051013 // m	0000922 // sp	0005515 // pr	Katanin p60 A	KATL1_HUMAN|KATNAL1	
0051013 // m	0000922 // sp	0008568 // m	Katanin p60 A	KATL2_HUMAN|KATNAL2	
0007275 // m	0005614 // in	0005520 // in	Kazal-type ser	KAZD1_HUMAN|KAZALD1|FKSG28|FKSG40|UNQ2945/P	
0070268 // co	0030057 // de	0005515 // pr	Kazrin	KAZRN_HUMAN|KAZN|KAZ|KIAA1026|HRIHFB2003	
0016567 // pr	0031463 // Cu	0004842 // ub	Kelch repeat a	KBTB2_HUMAN|KBTBD2|BKLHD1|KIAA1489|CGI-73	
0016567 // pr	0031463 // Cu	0004842 // ub	Kelch repeat a	KBTB3_HUMAN|KBTBD3|BKLHD3	
0043161 // pr	0005737 // cy	0031625 // ub	Kelch repeat a	KBTB4_HUMAN|KBTBD4|BKLHD4	
0016567 // pr	0031463 // Cu	0005515 // pr	Kelch repeat a	KBTB6_HUMAN|KBTBD6	
0014032 // ne	0005829 // cy	0005515 // pr	Kelch repeat a	KBTB8_HUMAN|KBTBD8|KIAA1842|TAKRP	
0071805 // po	0008076 // vo	0005249 // vo	Potassium vol	KCNA7_HUMAN|KCNA7	
0065009 // re	0044224 // ju	0015459 // po	Voltage-gated	KCAB2_HUMAN|KCNAB2|KCNA2B|KCNK2	
0065009 // re	0016021 // in	0015459 // po	Voltage-gated	KCAB3_HUMAN|KCNAB3|KCNA3B	
0009642 // re	0030673 // ax	0044325 // io	Potassium vol	KCNC1_HUMAN|KCNC1	
0071805 // po	0043025 // ne	0005250 // A-	Potassium vol	KCND1_HUMAN|KCND1	
0071805 // po	0005886 // pl	0005515 // pr	Potassium vol	KCND2_HUMAN|KCND2|KIAA1044	
0086009 // m	0042383 // sa	0044325 // io	Potassium vol	KCND3_HUMAN|KCND3	
0086009 // m	0005886 // pl	0015459 // po	Potassium vol	KCNE1_HUMAN|KCNE1	
1902260 // ne	0030425 // de	0005515 // pr	Potassium vol	KCNE3_HUMAN|KCNE3	
0034765 // re	0005886 // pl	0005515 // pr	Potassium vol	KCNG3_HUMAN|KCNG3	
0023014 // sig	0014069 // po	0005515 // pr	Potassium vol	KCNH1_HUMAN|KCNH1|EAG|EAG1	
0006813 // po	0005887 // in	0005249 // vo	Potassium vol	KCNH3_HUMAN|KCNH3|KIAA1282	
0006813 // po	0008076 // vo	0005249 // vo	Potassium vol	KCNH4_HUMAN|KCNH4	

0071805 // pc	0005887 // in	0005249 // vc	Potassium vol	KCNH7_HUMAN|KCNH7|ERG3	
0000160 // ph	0005887 // in	0005249 // vc	Potassium vol	KCNH8_HUMAN|KCNH8	
1901379 // re	0005737 // cy	0005267 // pc	Kv channel-int	KCIP1_HUMAN|KCNIP1|KCHIP1|VABP	
0072659 // pr	0005886 // pl	0005267 // pc	Calsenilin	CSEN_HUMAN|KCNIP3|CSEN|DREAM|KCHIP3	
0007588 // ex	0005886 // pl	0005524 // AT	ATP-sensitive	KCNJ1_HUMAN|KCNJ1|ROMK1	
0055075 // pc	0005886 // pl	0015272 // AT	ATP-sensitive	KCJ10_HUMAN|KCNJ10	
0010107 // pc	0001669 // ac	0005249 // vc	ATP-sensitive	KCJ11_HUMAN|KCNJ11	
0006813 // pc	0005886 // pl	0015467 // G-	ATP-sensitive	KCJ15_HUMAN|KCNJ15|KCNJ14	
0034765 // re	0005886 // pl	0015467 // G-	Inward rectifie	KCJ16_HUMAN|KCNJ16	
0086013 // m	0008076 // vc	0005242 // in	Inward rectifie	KCNJ2_HUMAN|KCNJ2|IRK1	
0098915 // m	0008076 // vc	0005242 // in	G protein-acti	KCNJ3_HUMAN|KCNJ3|GIRK1	
0086091 // re	0009897 // ex	0005242 // in	G protein-acti	KCNJ5_HUMAN|KCNJ5|GIRK4	
0034765 // re	0005886 // pl	0005242 // in	G protein-acti	KCNJ6_HUMAN|KCNJ6|GIRK2|KATP2|KCNJ7	
0009268 // re	0030016 // m	0015272 // AT	ATP-sensitive	KCNJ8_HUMAN|KCNJ8	
0035094 // re	0005887 // in	0005267 // pc	Potassium cha	KCNK1_HUMAN|KCNK1|HOHO1|KCNO1|TWIK1	
0007588 // ex	0005887 // in	0022841 // pc	Potassium cha	KCNK5_HUMAN|KCNK5|TASK2	
0061337 // ca	0005886 // pl	0005267 // pc	Potassium cha	KCNK6_HUMAN|KCNK6|TOSS|TWIK2	
0006813 // pc	0005887 // in	0005267 // pc	Potassium cha	KCNK7_HUMAN|KCNK7	
0006813 // pc	0016324 // ap	0015269 // ca	Calcium-activa	KCMA1_HUMAN|KCNMA1|KCNMA|SLO	
0006813 // pc	0005887 // in	0015459 // pc	Calcium-activa	KCMB2_HUMAN|KCNMB2	
0006813 // pc	0005887 // in	0015459 // pc	Calcium-activa	KCMB3_HUMAN|KCNMB3|KCNMB2|KCNMBL	
0006813 // pc	0005887 // in	0005515 // pr	Calcium-activa	KCMB4_HUMAN|KCNMB4	
0006884 // ce	0005886 // pl	0016286 // sn	Intermediate	KCNN4_HUMAN|KCNN4|IK1|IKCA1|KCA4|SK4	
0007268 // ch	0008076 // vc	0005516 // ca	Potassium vol	KCNQ2_HUMAN|KCNQ2	
0071805 // pc	0005887 // in	0005249 // vc	Potassium vol	KCNQ5_HUMAN|KCNQ5	
0065009 // re	0005794 // Gc	0005251 // de	Potassium vol	KCNS3_HUMAN|KCNS3	
0071805 // pc	0008076 // vc	0015269 // ca	Potassium cha	KCNT1_HUMAN|KCNT1|KIAA1422	
0071805 // pc	0008076 // vc	0015269 // ca	Potassium cha	KCNT2_HUMAN|KCNT2|SLICK	
0035036 // sp	0008076 // vc	0060072 // la	Potassium cha	KCNU1_HUMAN|KCNU1|KCNMA3|KCNMC1|SLO3	
0071805 // pc	0008076 // vc	0015459 // pc	Potassium vol	KCNV1_HUMAN|KCNV1	
0030513 // pc	0005615 // ex		Kielin/chordin	KCP_HUMAN|KCP|CRIM2|KCP1	
0051260 // pr		0005515 // pr	BTB/POZ dom	KCD14_HUMAN|KCTD14	
0007275 // m		0005515 // pr	BTB/POZ dom	KCD15_HUMAN|KCTD15	
0030030 // ce	0005737 // cy	0005515 // pr	BTB/POZ dom	KCD17_HUMAN|KCTD17	
0043161 // pr	0031463 // Cu	0032403 // pr	BTB/POZ dom	KCTD2_HUMAN|KCTD2|KIAA0176	
0051260 // pr	0005886 // pl		BTB/POZ dom	KCTD3_HUMAN|KCTD3	
0033146 // re	0005829 // cy	0005515 // pr	BTB/POZ dom	KCTD6_HUMAN|KCTD6	
0051260 // pr	0005622 // in	0005515 // pr	BTB/POZ dom	KCTD9_HUMAN|KCTD9	
0006664 // gl	0005788 // er	0046527 // gl	KDEL motif-co	KDEL1_HUMAN|KDELC1|EP58|UNQ1910/PRO4357	
0006886 // in	0030133 // tr	0005046 // KD	ER lumen prot	ERD22_HUMAN|KDELR2|ERD2.2	
0006621 // pr	0030133 // tr	0046923 // ER	ER lumen prot	ERD23_HUMAN|KDELR3	
0061436 // es	0005737 // cy		Keratinocyte c	KDF1_HUMAN|KDF1|C1orf172	
0071480 // ce	0005654 // nu	0005515 // pr	Lysine-specific	KDM1A_HUMAN|KDM1A|AOF2|KDM1|KIAA0601|LSD1	
0006303 // dc	0005654 // nu	0003677 // DI	Lysine-specific	KDM2A_HUMAN|KDM2A|CXXC8|FBL7|FBXL11|JHDM1A	

2000736 // re	0005654 // nu	0032454 // his	Lysine-specific	KDM4C_HUMAN|KDM4C|GASC1|JHDM3C|JMJD2C|KIAA
0006357 // re	0005654 // nu	0032452 // his	Lysine-specific	KDM5B_HUMAN|KDM5B|JARID1B|PLU1|RBBP2H1
0034720 // hi	0005654 // nu	0032452 // his	Lysine-specific	KDM5C_HUMAN|KDM5C|DXS1272E|JARID1C|SMCX|XE
0072358 // ca	0044666 // M	0032452 // his	Lysine-specific	KDM6A_HUMAN|KDM6A|UTX
0033169 // hi	0005634 // nu	0035064 // m	Lysine-specific	KDM7A_HUMAN|KDM7A|JHDM1D|KDM7|KIAA1718
0003158 // en	0005783 // er	0005515 // pr	Vascular endo	VGFR2_HUMAN|KDR|FLK1|VEGFR2
	0016021 // in	0003723 // RN	KH homology	KHDC1_HUMAN|KHDC1|C6orf147|C6orf148
0006351 // tra	0005634 // nu	0005515 // pr	KH domain-co	KHDR3_HUMAN|KHDRBS3|SALP|SLM2
0046835 // ca	0005737 // cy	0004454 // ke	Ketohexokina	KHK_HUMAN|KHK
0008150 // bi	0005575 // ce	0003723 // RN	Protein KHNYN	KHNYN_HUMAN|KHNYN|KIAA0323
		0005515 // pr	Uncharacteriz	K0040_HUMAN|KIAA0040
	0016021 // in	0005515 // pr	Dyslexia-asso	K319L_HUMAN|KIAA0319L|AAVR|KIAA1837|PP791
0001682 // tR	0005759 // m	0004526 // rib	Mitochondria	MRRP3_HUMAN|KIAA0391|MRPP3
		0005515 // pr	Uncharacteriz	K0408_HUMAN|KIAA0408
	0005737 // cy		Uncharacteriz	K0513_HUMAN|KIAA0513
0007099 // ce	0005829 // cy	0005515 // pr	Protein moon	MOONR_HUMAN|KIAA0753|MNR|OFIP
0006376 // m	0005681 // sp	0003723 // RN	UPF0469 prot	K0907_HUMAN|KIAA0907|BLOM7
	0016021 // in		UPF0258 prot	K1024_HUMAN|KIAA1024
0030856 // re	0016021 // in	0005515 // pr	Uncharacteriz	K1109_HUMAN|KIAA1109|FSA|KIAA1371
		0005515 // pr	Uncharacteriz	K1143_HUMAN|KIAA1143
0008150 // bi	0005737 // cy	0005515 // pr	Putative mon	P33MX_HUMAN|KIAA1191|P33MONOX
0048706 // en	0005737 // cy	0003674 // m	Sickle tail prot	SKT_HUMAN|KIAA1217|SKT
0000045 // au	0005765 // lys	0003723 // RN	UPF0577 prot	K1324_HUMAN|KIAA1324|EIG121|UNQ2426/PRO4985
	0016021 // in		UPF0577 prot	K132L_HUMAN|KIAA1324L|EIG121L
0002098 // tR	0005737 // cy	0008175 // tR	Probable tRNA	K1456_HUMAN|KIAA1456|C8orf79
0007155 // ce	0005911 // ce		Junctional pro	JCAD_HUMAN|KIAA1462|JCAD
0030154 // ce			Uncharacteriz	K1522_HUMAN|KIAA1522
	0016021 // in		UPF0606 prot	K1549_HUMAN|KIAA1549
	0016021 // in		UPF0606 prot	K154L_HUMAN|KIAA1549L|C11orf41|C11orf69
	0005739 // m	0005515 // pr	Uncharacteriz	K1683_HUMAN|KIAA1683
0007399 // ne	0005634 // nu		Protein KIAA2	K2022_HUMAN|KIAA2022
0021772 // ol	0005871 // ki	0005515 // pr	Kinesin-like pr	KIF14_HUMAN|KIF14|KIAA0042

0035735 // in	0005871 // ki	0005524 // AT	Kinesin-like pr	KIF17_HUMAN|KIF17|KIAA1405|KIF3X
0007399 // ne	0005856 // cy	0019894 // ki	KIF1-binding p	KBP_HUMAN|KIF1BP|KBP|KIAA1279
0007018 // m	0005871 // ki	0005515 // pr	Kinesin-like pr	KI21A_HUMAN|KIF21A|KIAA1708|KIF2
0007018 // m	0005871 // ki	0005524 // AT	Kinesin-like pr	KI21B_HUMAN|KIF21B|KIAA0449
0007019 // m	0005829 // cy	0042802 // id	Kinesin-like pr	KIF24_HUMAN|KIF24|C9orf48
0030010 // es	0005871 // ki	0016887 // AT	Kinesin-like pr	KI26B_HUMAN|KIF26B
0007052 // m	0005871 // ki	0005515 // pr	Kinesin-like pr	KIF2A_HUMAN|KIF2A|KIF2|KNS2
0007018 // m	0005813 // ce	0005515 // pr	Kinesin-like pr	KIF3B_HUMAN|KIF3B|KIAA0359
0007018 // m	0097542 // cil	0005524 // AT	Kinesin-like pr	KIF3C_HUMAN|KIF3C
0051028 // m	0005737 // cy	0008574 // AT	Kinesin heavy	KIF5C_HUMAN|KIF5C|KIAA0531|NKHC2
0045880 // po	0005871 // ki	0005515 // pr	Kinesin-like pr	KIF7_HUMAN|KIF7|UNQ340/PRO539
1903008 // or	0005871 // ki	0005515 // pr	Kinesin-like pr	KIF9_HUMAN|KIF9
0001933 // ne	0031253 // ce	0005515 // pr	Kin of IRRE-lik	KIRR1_HUMAN|KIRREL|KIRREL1|NEPH1
0060112 // ge	0005576 // ex	0031773 // kis	Metastasis-su	KISS1_HUMAN|KISS1|PP5098
0043069 // ne	0005886 // pl	0005515 // pr	Mast/stem ce	KIT_HUMAN|KIT|SCFR
0045740 // po	0005576 // ex	0008083 // gr	Kit ligand	SCF_HUMAN|KITLG|MGF|SCF
0008543 // fib	0005886 // pl	0017134 // fib	Klotho	KLOT_HUMAN|KL
0008088 // ax	0005871 // ki	0005515 // pr	Kinesin light c	KLC3_HUMAN|KLC3|KLC2|KLC2L
0009267 // ce	0005634 // nu	0001046 // co	Krueppel-like	KLF10_HUMAN|KLF10|TIEG|TIEG1
0045647 // ne	0005634 // nu	0001077 // tra	Krueppel-like	KLF13_HUMAN|KLF13|BTEB3|NSLP1
0045944 // po	0005634 // nu	0043565 // se	Krueppel-like	KLF14_HUMAN|KLF14|BTEB5
0010001 // gli	0005634 // nu	0001077 // tra	Krueppel-like	KLF15_HUMAN|KLF15|KKLF
0001701 // in	0000790 // nu	0003700 // tra	Krueppel-like	KLF2_HUMAN|KLF2|LKLF
0000122 // ne	0005654 // nu	0005515 // pr	Krueppel-like	KLF3_HUMAN|KLF3|BKLF
0043154 // ne	0005737 // cy	0044212 // tra	Krueppel-like	KLF4_HUMAN|KLF4|EZF|GKLF
1990830 // ce	0005794 // Go	0008134 // tra	Krueppel-like	KLF5_HUMAN|KLF5|BTEB2|CKLF|IKLF
0045893 // po	0005730 // nu	0005515 // pr	Krueppel-like	KLF6_HUMAN|KLF6|BCD1|COPEB|CPBP|ST12
0006357 // re	0005634 // nu	0003700 // tra	Krueppel-like	KLF7_HUMAN|KLF7|UKLF
0006351 // tra	0016235 // ag	0000978 // RN	Krueppel-like	KLF8_HUMAN|KLF8|BKLF3|ZNF741
0006351 // tra	0005634 // nu	0003677 // DN	Krueppel-like	KLF9_HUMAN|KLF9|BTEB|BTEB1
	0005829 // cy		Kelch domain-	KLDC1_HUMAN|KLHDC1|MSTP025
0016567 // pr	0031463 // Cu	0004842 // ub	Kelch domain-	KLD7B_HUMAN|KLHDC7B
0016567 // pr	0031463 // Cu	0004842 // ub	Kelch domain-	KLD8B_HUMAN|KLHDC8B|FP17659
0008150 // bi	0005575 // ce	0030332 // cy	Kelch domain-	KLDC9_HUMAN|KLHDC9|KARCA1
0006513 // pr	0000139 // Go	0005515 // pr	Kelch-like pro	KLH12_HUMAN|KLHL12|C3IP1
0016567 // pr	0016235 // ag	0004842 // ub	Kelch-like pro	KLH14_HUMAN|KLHL14|KIAA1384
0071630 // nu	0031463 // Cu	0005515 // pr	Kelch-like pro	KLH15_HUMAN|KLHL15|KIAA1677
0030036 // ac	0014069 // po	0051015 // ac	Kelch-like pro	KLH17_HUMAN|KLHL17
0016567 // pr	0031463 // Cu	0005515 // pr	Kelch-like pro	KLH18_HUMAN|KLHL18|KIAA0795|OK/SW-cl.74
0042787 // pr	0031463 // Cu	0005515 // pr	Kelch-like pro	KLHL2_HUMAN|KLHL2
0006513 // pr	0031463 // Cu	0004842 // ub	Kelch-like pro	KLH22_HUMAN|KLHL22
0016567 // pr	0031463 // Cu	0004842 // ub	Kelch-like pro	KLH23_HUMAN|KLHL23
0009566 // fe	0031463 // Cu	0004842 // ub	Kelch-like pro	KLH26_HUMAN|KLHL26
0016567 // pr	0031463 // Cu	0004842 // ub	Kelch-like pro	KLH28_HUMAN|KLHL28|BTBD5
0016567 // pr	0005829 // cy	0005198 // st	Kelch-like pro	KLHL3_HUMAN|KLHL3|KIAA1129

0016567 // pr	0031463 // Cu	0004842 // ub	Kelch-like pro	KLH30_HUMAN|KLHL30
0042787 // pr	0031463 // Cu	0004842 // ub	Kelch-like pro	KLH31_HUMAN|KLHL31|BKLHD6|KBTBD1|KLHL
0009566 // fe	0031463 // Cu	0004842 // ub	Kelch-like pro	KLH32_HUMAN|KLHL32|BKLHD5|KIAA1900
0016567 // pr	0031463 // Cu	0004842 // ub	Kelch-like pro	KLH36_HUMAN|KLHL36|C16orf44
0016567 // pr	0031463 // Cu	0004842 // ub	Kelch-like pro	KLHL4_HUMAN|KLHL4|KIAA1687
0043161 // pr	0031463 // Cu	0005515 // pr	Kelch-like pro	KLH42_HUMAN|KLHL42|KIAA1340|KLHDC5
0006508 // pr	0070062 // ex	0004252 // se	Kallikrein-1	KLK1_HUMAN|KLK1
0006508 // pr	0005576 // ex	0004252 // se	Kallikrein-10	KLK10_HUMAN|KLK10|NES1|PRSSL1
0006508 // pr	0070062 // ex	0004252 // se	Kallikrein-14	KLK14_HUMAN|KLK14|KLKL6
	0016021 // in		KLRC4-KLRK1	H3BQV0_HUMAN|KLRC4-KLRK1
0050776 // re	0005886 // pl	0023024 // M	Natural killer	KLRD1_HUMAN|KLRD1|CD94
0050776 // re	0005886 // pl	0004888 // tr	Killer cell lecti	KLRF1_HUMAN|KLRF1|CLEC5C|ML
0006954 // in	0016021 // in	0005515 // pr	Killer cell lecti	KLRG1_HUMAN|KLRG1|CLEC15A|MAFA|MAFAL
0045954 // po	0005886 // pl	0005515 // pr	NKG2-D type I	NKG2D_HUMAN|KLRK1|D12S2489E|NKG2D
0045652 // re	0044666 // M	0003723 // RN	Histone-lysine	KMT2C_HUMAN|KMT2C|HALR|KIAA1506|MLL3
0007050 // ce	0005654 // nu	0018024 // his	Histone-lysine	KMT2E_HUMAN|KMT2E|MLL5
0006351 // tra	0005654 // nu	0018024 // his	Histone-lysine	KMT5B_HUMAN|KMT5B|SUV420H1|CGI-85
0043547 // po	0043204 // pe	0005088 // Ra	Protein very K	VKIND_HUMAN|KNDC1|C10orf23|KIAA1768|RASGEF2|V
	0005730 // nu	0003723 // RN	Lysine-rich nu	KNOP1_HUMAN|KNOP1|C16orf88|FAM191A|TSG118
0006309 // ap	0005829 // cy	0005515 // pr	Importin subu	IMA5_HUMAN|KPNA1|RCH2
0006461 // pr	0005643 // nu	0005515 // pr	Importin subu	IMA4_HUMAN|KPNA3|QIP2
0006607 // NI	0005643 // nu	0008139 // nu	Importin subu	IMA8_HUMAN|KPNA7
0008150 // bi	0005575 // ce	0003674 // m	Protein KRBA1	KRBA1_HUMAN|KRBA1|KIAA1862
0006355 // re	0005622 // in	0003676 // nu	KRAB domain-	KRBX1_HUMAN|KRBOX1
0006355 // re	0005622 // in	0003676 // nu	KRAB domain-	KRBX4_HUMAN|KRBOX4|ZNF673
0072332 // in	0005622 // in	0002039 // p5	Lysine-rich co	KRCC1_HUMAN|KRCC1|CHBP2|BM-003|BM-044
0016055 // W	0016021 // in	0005515 // pr	Kremen prote	KREM1_HUMAN|KREMEN1|KREMEN|KRM1
0016055 // W	0005886 // pl		Kremen prote	KREM2_HUMAN|KREMEN2|KRM2
0000447 // en	0005730 // nu	0003723 // RN	Protein KRI1 h	KRI1_HUMAN|KRI1
0010596 // ne	0005737 // cy	0005515 // pr	Krev interacti	KRIT1_HUMAN|KRIT1|CCM1
0009314 // re	0005829 // cy	0005515 // pr	Keratin, type I	K1C13_HUMAN|KRT13
0045110 // in	0045095 // ke	0005515 // pr	Keratin, type I	K1C14_HUMAN|KRT14
0007010 // cy	0005829 // cy	0005515 // pr	Keratin, type I	K1C15_HUMAN|KRT15|KRTB
0030216 // ke	0005829 // cy	0005515 // pr	Keratin, type I	K1C16_HUMAN|KRT16|KRT16A
0007165 // sig	0005829 // cy	0005515 // pr	Keratin, type I	K1C17_HUMAN|KRT17
0016032 // vi	0005829 // cy	0097110 // sc	Keratin, type I	K1C18_HUMAN|KRT18|CYK18|PIG46
0043627 // re	0005829 // cy	0005515 // pr	Keratin, type I	K1C19_HUMAN|KRT19
	0005882 // in	0005198 // st	Keratin-like pr	KT222_HUMAN|KRT222|KA21|KRT222P

<table>
<tr><td></td><td></td><td></td><td></td><td></td></tr>
<tr><td>0045104 // in</td><td>0005829 // cy</td><td>0005198 // st</td><td>Keratin, type I</td><td>K2C3_HUMAN|KRT3</td></tr>
<tr><td>0008544 // ep</td><td>0005829 // cy</td><td>0005515 // pr</td><td>Keratin, type I</td><td>K1H1_HUMAN|KRT31|HHA1|HKA1|KRTHA1</td></tr>
<tr><td>0008544 // ep</td><td>0005829 // cy</td><td>0005198 // st</td><td>Keratin, type I</td><td>K1H2_HUMAN|KRT32|HHA2|HKA2|KRTHA2</td></tr>
<tr><td>0050680 // ne</td><td>0005829 // cy</td><td>0005515 // pr</td><td>Keratin, type I</td><td>K2C4_HUMAN|KRT4|CYK4</td></tr>
<tr><td></td><td></td><td></td><td></td><td></td></tr>
<tr><td>0031581 // he</td><td>0005829 // cy</td><td>0005515 // pr</td><td>Keratin, type I</td><td>K2C5_HUMAN|KRT5</td></tr>
<tr><td>0050830 // de</td><td>0005829 // cy</td><td>0005515 // pr</td><td>Keratin, type I</td><td>K2C6A_HUMAN|KRT6A|K6A|KRT6D</td></tr>
<tr><td>0007010 // cy</td><td>0005829 // cy</td><td>0005515 // pr</td><td>Keratin, type I</td><td>K2C6B_HUMAN|KRT6B|K6B|KRTL1</td></tr>
<tr><td>0045104 // in</td><td>0005829 // cy</td><td>0005515 // pr</td><td>Keratin, type I</td><td>K2C6C_HUMAN|KRT6C|KRT6E</td></tr>
<tr><td></td><td></td><td></td><td></td><td></td></tr>
<tr><td>0070268 // co</td><td>0005829 // cy</td><td>0005515 // pr</td><td>Keratin, type I</td><td>K2C71_HUMAN|KRT71|K6IRS1|KB34|KRT6IRS1</td></tr>
<tr><td>0045104 // in</td><td>0005829 // cy</td><td>1990254 // ke</td><td>Keratin, type I</td><td>K2C74_HUMAN|KRT74|K6IRS4|KB37|KRT5C|KRT6IRS4</td></tr>
<tr><td>0070268 // co</td><td>0005829 // cy</td><td>0005198 // st</td><td>Keratin, type I</td><td>K2C75_HUMAN|KRT75|K6HF|KB18</td></tr>
<tr><td>0070268 // co</td><td>0005829 // cy</td><td>0003674 // m</td><td>Keratin, type I</td><td>K2C1B_HUMAN|KRT77|KRT1B</td></tr>
<tr><td>0070268 // co</td><td>0005829 // cy</td><td>0005198 // st</td><td>Keratin, type I</td><td>K2C78_HUMAN|KRT78|K5B|KB40</td></tr>
<tr><td>0045214 // sa</td><td>0042383 // sa</td><td>0097110 // sc</td><td>Keratin, type I</td><td>K2C8_HUMAN|KRT8|CYK8</td></tr>
<tr><td>0070268 // co</td><td>0005829 // cy</td><td>0005515 // pr</td><td>Keratin, type I</td><td>KRT81_HUMAN|KRT81|KRTHB1|MLN137</td></tr>
<tr><td>0070268 // co</td><td>0005829 // cy</td><td>0005515 // pr</td><td>Keratin, type I</td><td>KRT86_HUMAN|KRT86|KRTHB6</td></tr>
<tr><td></td><td>0005615 // ex</td><td>0005198 // st</td><td>Putative kerat</td><td>KR87P_HUMAN|KRT87P|KRT121P|KRTHBP4</td></tr>
<tr><td></td><td></td><td></td><td></td><td></td></tr>
<tr><td></td><td></td><td></td><td></td><td></td></tr>
<tr><td></td><td></td><td></td><td></td><td></td></tr>
<tr><td>0008544 // ep</td><td>0005829 // cy</td><td>0005200 // st</td><td>Keratin, type I</td><td>K1C9_HUMAN|KRT9</td></tr>
<tr><td>0031424 // ke</td><td>0045095 // ke</td><td>0005515 // pr</td><td>Keratin-associ</td><td>KRA23_HUMAN|KRTAP2-3|KAP2.3|KRTAP2.3</td></tr>
<tr><td>0031424 // ke</td><td>0045095 // ke</td><td>0005515 // pr</td><td>Keratin-associ</td><td>KRA31_HUMAN|KRTAP3-1|KAP3.1|KRTAP3.1</td></tr>
<tr><td>0031424 // ke</td><td>0045095 // ke</td><td></td><td>Keratin-associ</td><td>KRA41_HUMAN|KRTAP4-1|KAP4.10|KRTAP4-10|KRTAP4</td></tr>
<tr><td>0042633 // ha</td><td>0045095 // ke</td><td>0005515 // pr</td><td>Keratin-associ</td><td>KRA47_HUMAN|KRTAP4-7|KAP4.7|KRTAP4.7</td></tr>
<tr><td>0042633 // ha</td><td>0045095 // ke</td><td></td><td>HCG2042992</td><td>A0A0G2JLE6_HUMAN|KRTAP4-8|hCG_2042992</td></tr>
<tr><td>0042633 // ha</td><td>0045095 // ke</td><td></td><td>Keratin-associ</td><td>KRA49_HUMAN|KRTAP4-9|KAP4.9|KRTAP4.9</td></tr>
<tr><td>0031424 // ke</td><td>0045095 // ke</td><td></td><td>Keratin-associ</td><td>KRA57_HUMAN|KRTAP5-7|KAP5-7|KAP5.3|KRTAP5.3|K</td></tr>
<tr><td>0008544 // ep</td><td>0045095 // ke</td><td>0005515 // pr</td><td>Keratin-associ</td><td>KRA59_HUMAN|KRTAP5-9|KAP5.9|KRN1|KRTAP5.9|UH</td></tr>
<tr><td></td><td>0016021 // in</td><td></td><td>Keratinocyte-a</td><td>KCP3_HUMAN|KRTCAP3|KCP3|UNQ3066/PRO9898</td></tr>
<tr><td>0008150 // bi</td><td>0005615 // ex</td><td>0003674 // m</td><td>Keratinocyte d</td><td>KTDAP_HUMAN|KRTDAP|KDAP|UNQ467/PRO826</td></tr>
<tr><td>0043410 // po</td><td>0005829 // cy</td><td>0005078 // M</td><td>Kinase suppre</td><td>KSR1_HUMAN|KSR1|KSR</td></tr>
<tr><td>0007018 // m</td><td>0005887 // in</td><td>0003723 // RN</td><td>Kinectin</td><td>KTN1_HUMAN|KTN1|CG1|KIAA0004</td></tr>
<tr><td></td><td></td><td></td><td></td><td></td></tr>
<tr><td>0016192 // ve</td><td>0005765 // ly</td><td>0005515 // pr</td><td>KxDL motif-co</td><td>KXDL1_HUMAN|KXD1|C19orf50</td></tr>
<tr><td>0070189 // ky</td><td>0005739 // m</td><td>0016212 // ky</td><td>Kynurenine--c</td><td>KAT3_HUMAN|KYAT3|CCBL2|KAT3</td></tr>
<tr><td>0006569 // tr</td><td>0005739 // m</td><td>0030429 // ky</td><td>Kynureninase</td><td>KYNU_HUMAN|KYNU</td></tr>
<tr><td>0007411 // ax</td><td>0005886 // pl</td><td>0005515 // pr</td><td>Neural cell ad</td><td>L1CAM_HUMAN|L1CAM|CAML1|MIC5</td></tr>
<tr><td></td><td></td><td></td><td></td><td></td></tr>
<tr><td></td><td></td><td></td><td></td><td></td></tr>
<tr><td>0006351 // tr</td><td>0005654 // nu</td><td>0008270 // zi</td><td>Lethal(3)malig</td><td>LMBL2_HUMAN|L3MBTL2</td></tr>
<tr><td></td><td></td><td></td><td></td><td></td></tr>
<tr><td></td><td></td><td></td><td></td><td></td></tr>
<tr><td></td><td></td><td></td><td></td><td></td></tr>
</table>

	0005777 // pe	0005507 // co	Laccase doma	LACC1_HUMAN|LACC1|C13orf31|FAMIN
	0005604 // ba	0005198 // str	Ladinin-1	LAD1_HUMAN|LAD1|LAD
0019886 // ar	0016021 // int	0003823 // ar	Lymphocyte a	LAG3_HUMAN|LAG3|FDC
0008033 // tR	0016604 // nu	0005515 // pr	EKC/KEOPS cc	LAGE3_HUMAN|LAGE3|DXS9879E|ESO3|ITBA2
0007155 // ce	0016020 // m	0005515 // pr	Laminin subur	LAMA1_HUMAN|LAMA1|LAMA
0007155 // ce	0005576 // ex	0005198 // str	Laminin subur	LAMA2_HUMAN|LAMA2|LAMM
0007155 // ce	0005576 // ex	0005198 // str	Laminin subur	LAMA3_HUMAN|LAMA3|LAMNA
0007155 // ce	0031012 // ex	0005201 // ex	Laminin subur	LAMA4_HUMAN|LAMA4
0042127 // re	0005576 // ex	0005198 // str	Laminin subur	LAMA5_HUMAN|LAMA5|KIAA0533|KIAA1907
0035987 // en	0005576 // ex	0005198 // str	Laminin subur	LAMB1_HUMAN|LAMB1
0007155 // ce	0005576 // ex	0032403 // pr	Laminin subur	LAMB3_HUMAN|LAMB3|LAMNB1
0007155 // ce	0005604 // ba		Laminin subur	LAMB4_HUMAN|LAMB4
0031581 // he	0005606 // lar	0005201 // ex	Laminin subur	LAMC1_HUMAN|LAMC1|LAMB2
0007155 // ce	0005576 // ex	0008201 // he	Laminin subur	LAMC2_HUMAN|LAMC2|LAMB2T|LAMNB2
0043154 // ne	0005765 // lys		Lysosome-ass	LAMP3_HUMAN|LAMP3|DCLAMP|TSC403
0007050 // ce	0005765 // lys	0032947 // pr	Ragulator com	LTOR4_HUMAN|LAMTOR4|C7orf59
0045892 // ne	0005886 // pl	0032266 // ph	LanC-like prot	LANC2_HUMAN|LANCL2|GPR69B|TASP
0007165 // sig	0005886 // pl		LanC-like prot	LANC3_HUMAN|LANCL3
1990830 // ce	0005765 // lys	0005515 // pr	Lysosomal-ass	LAPM5_HUMAN|LAPTM5|KIAA0085
0046716 // m	0000139 // Go	0015020 // glu	LARGE xylosyl	LARG1_HUMAN|LARGE1|KIAA0609|LARGE
0046716 // m	0016021 // int	0015020 // glu	LARGE xylosyl	LARG2_HUMAN|LARGE2|GYLTL1B|PP5656
1902416 // po	0030529 // int	0048027 // m	La-related pro	LARP6_HUMAN|LARP6
1904263 // po	0005829 // cy	0004832 // va	Leucine--tRNA	SYLC_HUMAN|LARS|KIAA1352
0000460 // m	0071339 // M	0003723 // RN	Ribosomal bio	LAS1L_HUMAN|LAS1L|MSTP060
0006954 // inf	0005886 // pl	0005515 // pr	Linker for acti	LAT_HUMAN|LAT
0002250 // ac	0016021 // int	0042169 // SH	Linker for acti	NTAL_HUMAN|LAT2|LAB|NTAL|WBS15|WBSCR15|WBS
0000188 // in	0005794 // Go	0042169 // SH	Lymphocyte t	LAX1_HUMAN|LAX1|LAX
	0016021 // int	0005540 // hy	Layilin	LAYN_HUMAN|LAYN|UNQ208/PRO234
0016126 // ste	0031965 // nu	0005515 // pr	Lamin-B recep	LBR_HUMAN|LBR
0009653 // an	0005667 // tra	0003700 // tra	Transcription	LBX1_HUMAN|LBX1|LBX1H
0006351 // tra	0005634 // nu	0003677 // DN	Transcription	LBX2_HUMAN|LBX2|LP3727
0006879 // ce	0005576 // ex	0002020 // pr	Neutrophil ge	NGAL_HUMAN|LCN2|HNL|NGAL
0006366 // tra	0005634 // nu	0003677 // DN	Ligand-depen	LCORL_HUMAN|LCORL
0010737 // pr	0005737 // cy	0042802 // id	Plastin-2	PLSL_HUMAN|LCP1|PLS2
0010669 // ep	0005667 // tra	0005515 // pr	LIM domain-b	LDB2_HUMAN|LDB2|CLIM1
0045214 // sa	0030018 // Z c	0008092 // cy	LIM domain-b	LDB3_HUMAN|LDB3|KIAA0613|ZASP
0019674 // NA	0070062 // ex	0042802 // id	L-lactate dehy	LDHA_HUMAN|LDHA|PIG19

0019674 // N/	0005737 // cy	0005515 // pr	L-lactate dehy	LDHB_HUMAN|LDHB
0055114 // ox	0005743 // m	0004458 // D-	Probable D-la	LDHD_HUMAN|LDHD
0071398 // ce	0005886 // pl	0001618 // vi	Low-density li	LDLR_HUMAN|LDLR
	0016021 // in	0005515 // pr	Low-density li	LRAD1_HUMAN|LDLRAD1
	0016021 // in		Low-density li	LRAD2_HUMAN|LDLRAD2
0008285 // ne	0005730 // nu	0005515 // pr	Protein LDOC1	LDOC1_HUMAN|LDOC1|BCUR1
0045944 // po	1990907 // be	0044212 // tra	Lymphoid enh	LEF1_HUMAN|LEF1
0000122 // ne	0005615 // ex	0008083 // gr	Left-right dete	LFTY1_HUMAN|LEFTY1|LEFTB|LEFTYB|UNQ278/PRO317
	0016021 // in		LEM domain-c	LEMD1_HUMAN|LEMD1
0060914 // he	0005639 // in	0005515 // pr	LEM domain-c	LEMD2_HUMAN|LEMD2
0001525 // ar	0031965 // nu	0005515 // pr	Inner nuclear	MAN1_HUMAN|LEMD3|MAN1
0008150 // bi	0005575 // ce	0005515 // pr	Leukocyte rec	LENG1_HUMAN|LENG1
		0046872 // m	Leukocyte rec	LENG9_HUMAN|LENG9
0019953 // se	0016021 // in	0004888 // tra	Leptin recepto	LEPR_HUMAN|LEPR|DB|OBR
0046426 // ne	0016021 // in	0005102 // re	Leptin recepto	OBRG_HUMAN|LEPROT|LEPR|OBR
0006875 // ce	0016021 // in	0043022 // rib	LETM1 domai	LETM2_HUMAN|LETM2
0007386 // co	0030173 // in	0033829 // O-	Beta-1,3-N-ac	LFNG_HUMAN|LFNG
0042981 // re	0005737 // cy	0003723 // RN	Galectin-1	LEG1_HUMAN|LGALS1
		0005515 // pr	Galectin-2	LEG2_HUMAN|LGALS2
2001237 // ne	0005886 // pl	0003723 // RN	Galectin-3	LEG3_HUMAN|LGALS3|MAC2
0002576 // pl	0031089 // pl	0005044 // sc	Galectin-3-bin	LG3BP_HUMAN|LGALS3BP|M2BP
0007155 // ce	0005615 // ex	0030246 // ca	Galectin-4	LEG4_HUMAN|LGALS4
0098792 // xe	0070062 // ex	0005178 // in	Galectin-8	LEG8_HUMAN|LGALS8
2001181 // po	0005737 // cy	0005534 // ga	Galectin-9	LEG9_HUMAN|LGALS9
	0005829 // cy	0005515 // pr	Galectin-9C	LEG9C_HUMAN|LGALS9C
0022011 // m	0005615 // ex		Leucine-rich r	LGI4_HUMAN|LGI4|LGIL3|UNQ6515/PRO21485
0043524 // ne	0005764 // lys	0004197 // cy	Legumain	LGMN_HUMAN|LGMN|PRSC1
0036335 // in	0005886 // pl	0005515 // pr	Leucine-rich r	LGR4_HUMAN|LGR4|GPR48
0016055 // W	0005886 // pl	0004888 // tra	Leucine-rich r	LGR6_HUMAN|LGR6|UNQ6427/PRO21331|VTS20631
0006542 // gl	0005886 // pl	0004356 // gl	Lengsin	LGSN_HUMAN|LGSN|GLULD1|LGS
0030728 // ov	0005576 // ex	0005102 // re	Lutropin subu	LSHB_HUMAN|LHB
0002576 // pl	0016021 // in	0003674 // m	Lipoma HMGI	LHPL2_HUMAN|LHFPL2|KIAA0206
	0016021 // in		Lipoma HMGI	LHPL4_HUMAN|LHFPL4
0006811 // io	0016021 // in	0005515 // pr	Tetraspan me	TMHS_HUMAN|LHFPL5|TMHS
0009168 // pu	0005829 // cy	0008969 // ph	Phospholysine	LHPP_HUMAN|LHPP
0060066 // ov	0005634 // nu	0003714 // tra	LIM/homeobo	LHX1_HUMAN|LHX1|LIM-1|LIM1
0009887 // ar	0005634 // nu	0005515 // pr	LIM/homeobo	LHX4_HUMAN|LHX4
0021702 // ce	0005634 // nu	0043565 // se	LIM/homeobo	LHX5_HUMAN|LHX5
0021800 // ce	0005634 // nu	0003700 // tra	LIM/homeobo	LHX6_HUMAN|LHX6|LHX6.1
0006351 // tra	0001674 // fe	0005515 // pr	LIM/homeobo	LHX8_HUMAN|LHX8
0008584 // m	0005634 // nu	0005515 // pr	LIM/homeobo	LHX9_HUMAN|LHX9

0046697 // de	0005576 // ex	0005515 // pr	Leukemia inhi	LIF_HUMAN|LIF|HILDA
0002250 // ac	0016021 // in		Leukocyte imr	LIRA6_HUMAN|LILRA6|ILT8
0006952 // de	0005887 // in	0005515 // pr	Leukocyte imr	LIRB3_HUMAN|LILRB3|ILT5|LIR3
0030835 // ne	0032154 // cle	0005515 // pr	LIM domain a	LIMA1_HUMAN|LIMA1|EPLIN|SREBP3|PP624
0031032 // ac		0003779 // ac	LIM and calpo	LIMC1_HUMAN|LIMCH1|KIAA1102
0090090 // ne	0005829 // cy	0005515 // pr	LIM domain-c	LIMD1_HUMAN|LIMD1
	0005634 // nu	0046872 // m	LIM domain-c	LIMD2_HUMAN|LIMD2|SB143
0000390 // sp	0071008 // U2		Lck-interacting	LIME1_HUMAN|LIME1|LIME|LP8067
0006468 // pr	0005737 // cy	0005515 // pr	LIM domain k	LIMK1_HUMAN|LIMK1|LIMK
0043066 // ne	0005829 // cy	0046872 // m	LIM and senes	LIMS2_HUMAN|LIMS2|PINCH2
	0005737 // cy	0046872 // m	LIM and senes	LIMS3_HUMAN|LIMS3|PINCH3
0006355 // re	0005730 // nu	0003723 // RN	Protein lin-28	LN28B_HUMAN|LIN28B|CSDD2
0006351 // tra	0005654 // nu	0005515 // pr	Protein lin-52	LIN52_HUMAN|LIN52|C14orf46
0007269 // ne	0014069 // po	0005515 // pr	Protein lin-7 h	LIN7A_HUMAN|LIN7A|MALS1|VELI1
0007269 // ne	0014069 // po	0097016 // L2	Protein lin-7 h	LIN7B_HUMAN|LIN7B|MALS2|VELI2|UNQ3116/PRO102
0007049 // ce	0005654 // nu	0005515 // pr	Protein lin-9 h	LIN9_HUMAN|LIN9|BARA|TGS

0008150 // bi	0005575 // ce	0003674 // m	Putative unch	CR002_HUMAN|LINC00470|C18orf2
		0005515 // pr	Putative unch	CQ055_HUMAN|LINC00482|C17orf55
		0005515 // pr	Putative unch	CF218_HUMAN|LINC00518|C6orf218

	0016021 // in		Long intergen	A0A1B0GWH6_HUMAN|LINC00672
	0016021 // in		Putative trans	SIM16_HUMAN|LINC00862|C1orf98|SMIM16

	0016021 // in		Putative trans	YG055_HUMAN|LINC00998
	0016021 // in		Putative unch	CG013_HUMAN|LINC01006|C7orf13|MY040
	0016021 // in		Long intergen	A0A1B0GVN3_HUMAN|LINC01125

0007409 // ax	0005578 // pr		Leucine-rich r	LIGO2_HUMAN|LINGO2|LERN3|LRRN6C|UNQ9234/PRO
0000902 // ce	0005764 // lys	0004771 // st	Lysosomal aci	LICH_HUMAN|LIPA
0042758 // lo	0005811 // lip	0005515 // pr	Hormone-sen	LIPS_HUMAN|LIPE
0007584 // re	0009986 // ce	0004620 // ph	Endothelial lip	LIPE_HUMAN|LIPG|UNQ387/PRO719
0016042 // lip	0005886 // pl	0008201 // he	Lipase membe	LIPH_HUMAN|LIPH|LPDLR|MPAPLA1|PLA1B
0016042 // lip		0016788 // hy	Lipase membe	LIPJ_HUMAN|LIPJ|LIPL1
0070268 // co	0005576 // ex	0004465 // lip	Lipase membe	LIPM_HUMAN|LIPM|LIPL3
0009249 // pr	0005739 // m	0016874 // lig	Putative lipoy	LIPT2_HUMAN|LIPT2
0006357 // re	0098559 // cy	0005515 // pr	Lipopolysacch	LITAF_HUMAN|LITAF|PIG7|SIMPLE
0008150 // bi	0005575 // ce	0003674 // m	LIX1-like prote	LIX1L_HUMAN|LIX1L
0050708 // re	0005886 // pl	0019905 // sy	Lethal(2) giant	L2GL1_HUMAN|LLGL1|DLG4|HUGL|HUGL1
0050708 // re	0005886 // pl	0019905 // sy	Lethal(2) giant	L2GL2_HUMAN|LLGL2
0097484 // de	0005694 // ch	0003723 // RN	Protein LLP ho	LLPH_HUMAN|LLPH|C12orf31|cPERP-G
0006888 // ER	0005789 // er	0005515 // pr	Protein ERGIC	LMAN1_HUMAN|LMAN1|ERGIC53|F5F8D
	0016021 // int	0030246 // ca	Protein ERGIC	LMA1L_HUMAN|LMAN1L|ERGL|UNQ2784/PRO7174
0016032 // vi	0016021 // int	0005158 // ins	Probable lysos	LMBD1_HUMAN|LMBRD1|C6orf209|NESI|BM-021|CD0
	0016021 // int		LMBR1 domai	LMBD2_HUMAN|LMBRD2
0006641 // tri	0016021 // int		Lipase matura	LMF1_HUMAN|LMF1|C16orf26|TMEM112|HMFN1876|
0051604 // pr	0016021 // int	0005515 // pr	Lipase matura	LMF2_HUMAN|LMF2|TMEM112B|TMEM153

0006508 // pr	0016020 // m	0004222 // m	Leishmanolysi	LMLN_HUMAN|LMLN
0007077 // m	0005654 // nu	0005515 // pr	Prelamin-A/C	LMNA_HUMAN|LMNA|LMN1
0008283 // ce	0005737 // cy	0005198 // str	Lamin tail dor	LMTD1_HUMAN|LMNTD1|IFLTD1
0045944 // pc	0005654 // nu	0005515 // pr	Rhombotin-2	RBTN2_HUMAN|LMO2|RBTN2|RBTNL1|RHOM2|TTG2
0006351 // tra	0005737 // cy	0005515 // pr	LIM domain o	LMO3_HUMAN|LMO3|RBTN3|RBTNL2|RHOM3
0006936 // m	0016020 // m	0005523 // tro	Leiomodin-1	LMOD1_HUMAN|LMOD1
0051694 // pc	0005737 // cy	0003785 // ac	Leiomodin-3	LMOD3_HUMAN|LMOD3
0018105 // pe	0048471 // pe	0004674 // pr	Serine/threon	LMTK2_HUMAN|LMTK2|AATYK2|BREK|KIAA1079|KPI2|
0010923 // ne	0016021 // int	0004674 // pr	Serine/threon	LMTK3_HUMAN|LMTK3|KIAA1883|TYKLM3
0042787 // pr	0005737 // cy	0005515 // pr	E3 ubiquitin-p	LNX1_HUMAN|LNX1|LNX|PDZRN2|UNQ574/PRO1136
0051260 // pr		0005515 // pr	Ligand of Num	LNX2_HUMAN|LNX2|PDZRN1
0007005 // m	0005739 // m	0051880 // G-	Lon protease	LONM_HUMAN|LONP1|PRSS15
0032436 // pc	0005829 // cy	0005515 // pr	LON peptidase	LONF1_HUMAN|LONRF1|RNF191
0048545 // re	0005576 // ex	0005515 // pr	Protein-lysine	LYOX_HUMAN|LOX
0055114 // ox	0005576 // ex	0005507 // cc	Lysyl oxidase l	LOXL1_HUMAN|LOXL1|LOXL
0001666 // re	0005604 // ba	0009055 // ele	Lysyl oxidase l	LOXL2_HUMAN|LOXL2
0006898 // re	0043235 // re	0005044 // sc	Lysyl oxidase l	LOXL4_HUMAN|LOXL4|LOXC
0008150 // bi	0005576 // ex	0003674 // m	Putative apoli	LPAL2_HUMAN|LPAL2|APOARGC
0007186 // G-	0009986 // ce	0005515 // pr	Lysophosphat	LPAR2_HUMAN|LPAR2|EDG4|LPA2
0007268 // ch	0005886 // pl	0005543 // ph	Lysophosphat	LPAR3_HUMAN|LPAR3|EDG7|LPA3
0007186 // G-	0005886 // pl	0004930 // G-	Lysophosphat	LPAR5_HUMAN|LPAR5|GPR92|GPR93
0051482 // pc	0005886 // pl	0070915 // lys	Lysophosphat	LPAR6_HUMAN|LPAR6|P2RY5
0036148 // ph	0005789 // er	0047159 // 1-	Lysophosphat	PCAT1_HUMAN|LPCAT1|AYTL2|PFAAP3
0008654 // ph	0005789 // er	0003841 // 1-	Lysophosphol	MBOA5_HUMAN|LPCAT3|MBOAT5|OACT5
0036148 // ph	0005789 // er	0003841 // 1-	Lysophosphol	LPCT4_HUMAN|LPCAT4|AGPAT7|AYTL3|LPEAT2
0036148 // ph	0016021 // int	0003841 // 1-	Acyl-CoA:lyso	LGAT1_HUMAN|LPGAT1|FAM34A|KIAA0205
0006629 // lip	0005634 // nu	0008195 // ph	Phosphatidate	LPIN2_HUMAN|LPIN2|KIAA0249
0098869 // ce	0005737 // cy	0020037 // he	Lactoperoxida	PERL_HUMAN|LPO|SAPX
0008150 // bi	0016021 // int	0003674 // m	Lipopolysacch	LRBA_HUMAN|LRBA|BGL|CDC4L|LBA
0007165 // sig	0005886 // pl		Leucine-rich r	LRCH2_HUMAN|LRCH2|KIAA1495
0007165 // sig	0016605 // PM	0005515 // pr	Leucine-rich r	LRCH4_HUMAN|LRCH4|LRN|LRRN1|LRRN4
0007586 // di	0005576 // ex	0008047 // en	Leucine-rich c	LRCL1_HUMAN|LRCOL1
0007409 // ax	0014069 // pc		Leucine-rich r	LRFN1_HUMAN|LRFN1|KIAA1484|SALM2
0007409 // ax	0005578 // pr	0005515 // pr	Leucine-rich r	LRFN4_HUMAN|LRFN4|SALM3
0007409 // ax	0005578 // pr		Leucine-rich r	LRFN5_HUMAN|LRFN5|C14orf146|SALM5
0001938 // pc	0005576 // ex	0003674 // m	Leucine-rich a	A2GL_HUMAN|LRG1|LRG
0006351 // tra	0000784 // nu	0005515 // pr	Ligand-depen	LRIF1_HUMAN|LRIF1|C1orf103|RIF1
0007605 // se	0016021 // int		Leucine-rich r	LRIG1_HUMAN|LRIG1|LIG1
0032474 // ot	0016021 // int		Leucine-rich r	LRIG3_HUMAN|LRIG3|LIG3|UNQ287/PRO326/PRO335
0050896 // re	0016021 // int		Leucine-rich r	LRIT3_HUMAN|LRIT3
0006903 // ve	0000922 // sp		Lymphoid-rest	LRMP_HUMAN|LRMP|JAW1
0006898 // re	0005769 // ea	0042954 // lip	Prolow-densit	LRP1_HUMAN|LRP1|A2MR|APR
0035902 // re	0016021 // int	0051219 // ph	Low-density li	LRP11_HUMAN|LRP11
0006898 // re	0005905 // cla	0005515 // pr	Low-density li	LRP12_HUMAN|LRP12|ST7

0015031 // pr	0016021 // in	0005509 // ca	Low-density li	LRP1B_HUMAN	LRP1B|LRPDIT
0042953 // lip	0005886 // pl	0035258 // st	Low-density li	LRP2_HUMAN	LRP2
	0005737 // cy	0005515 // pr	LRP2-binding	LR2BP_HUMAN	LRP2BP|KIAA1325
0006898 // re	0005905 // cl		Low-density li	LRP3_HUMAN	LRP3
0051290 // pr	0031594 // ne	0042813 // W	Low-density li	LRP4_HUMAN	LRP4|KIAA0816|LRP10|MEGF7
0016055 // W	0005886 // pl	0005515 // pr	Low-density li	LRP5_HUMAN	LRP5|LR3|LRP7
0060828 // re	0005886 // pl	0019534 // to	Low-density li	LRP6_HUMAN	LRP6
0007165 // sig	0005829 // cy	0005515 // pr	Leucine-rich r	LLR1_HUMAN	LRR1|PPIL5
0007165 // sig	0005737 // cy		Leucine-rich r	LRRC1_HUMAN	LRRC1|LANO
0045671 // ne	0005615 // ex		Leucine-rich r	LRC17_HUMAN	LRRC17|P37NB|UNQ3076/PRO9909
0051965 // po	0016021 // in		Leucine-rich r	LRC24_HUMAN	LRRC24
		0005515 // pr	Leucine-rich r	LRC29_HUMAN	LRRC29|FBL9|FBXL9
0019221 // cy	0005737 // cy	0004860 // pr	Leucine-rich r	LRRC3_HUMAN	LRRC3|C21orf102|LRRC3A|UNQ9233/P
0010628 // po	0005887 // in	0005515 // pr	Leucine-rich r	LRC32_HUMAN	LRRC32|D11S833E|GARP
0008150 // bi	0005730 // nu	0003674 // m	Leucine-rich r	LRC34_HUMAN	LRRC34
	0016021 // in		Leucine-rich r	L37A2_HUMAN	LRRC37A2
	0016021 // in		Leucine-rich r	L37A3_HUMAN	LRRC37A3|KIAA0563
0019221 // cy	0005737 // cy	0004860 // pr	Leucine-rich r	LRRC4_HUMAN	LRRC4|BAG|NAG14|UNQ554/PRO1111
	0005829 // cy	0005515 // pr	Leucine-rich r	LRC45_HUMAN	LRRC45
0006432 // ph	0009328 // ph	0003723 // RN	Leucine-rich r	LRC47_HUMAN	LRRC47|KIAA1185
	0005737 // cy		Leucine-rich r	LRC49_HUMAN	LRRC49
	0042645 // m	0003723 // RN	Leucine-rich r	LRC59_HUMAN	LRRC59|PRO1855
0030317 // fla	0005929 // cil	0005515 // pr	Protein tilB hc	TILB_HUMAN	LRRC6|LRTP|TSLRP
0019221 // cy	0005737 // cy	0004860 // pr	Leucine-rich r	LRC66_HUMAN	LRRC66
0002237 // re	0005578 // pr	0005149 // in	Leucine-rich r	LRR70_HUMAN	LRRC70|UNQ1891/PRO4337
0006884 // ce	0009986 // ce	0005515 // pr	Volume-regul	LRC8A_HUMAN	LRRC8A|KIAA1437|LRRC8|SWELL1|UN(
0007165 // sig	0005789 // er	0005225 // vo	Volume-regul	LRC8B_HUMAN	LRRC8B|KIAA0231|UNQ6413/PRO2120
0007165 // sig	0005887 // in	0005225 // vo	Volume-regul	LRC8C_HUMAN	LRRC8C|AD158|FAD158
0007165 // sig	0005887 // in	0005225 // vo	Volume-regul	LRC8D_HUMAN	LRRC8D|LRRC5|UNQ213/PRO239
0051301 // ce	0005813 // ce		Leucine-rich r	LRCC1_HUMAN	LRRCC1|CLERC|KIAA1764

		0005515 // pr	Leucine-rich r	LRIQ3_HUMAN	LRRIQ3|LRRC44
0007165 // sig	0005886 // pl	0005225 // vo	Leucine-rich r	LRIQ4_HUMAN	LRRIQ4|LRRC64
0034599 // ce	0005794 // Go	0005515 // pr	Leucine-rich r	LRRK2_HUMAN	LRRK2|PARK8
0007409 // ax	0005578 // pr		Leucine-rich r	LRRN1_HUMAN	LRRN1|KIAA1497|Nbla10449|UNQ693/
0007165 // sig	0005578 // pr	0004872 // re	Leucine-rich r	LRRN2_HUMAN	LRRN2|GAC1|LRRN5|UNQ256/PRO293
0008542 // vis	0070062 // ex		Leucine-rich r	LRRN4_HUMAN	LRRN4|C20orf75
0019221 // cy	0005737 // cy	0004860 // pr	Leucine-rich r	LRRT4_HUMAN	LRRTM4|UNQ3075/PRO9907
0019221 // cy	0005737 // cy	0004860 // pr	Leucine-rich r	LRTM2_HUMAN	LRTM2
0032259 // m	0016021 // in	0016206 // ca	Transmembra	TOMT_HUMAN	LRTOMT|COMT2|TOMT|PP7517
0008380 // RN	0005654 // nu	0005515 // pr	U7 snRNA-ass	LSM10_HUMAN	LSM10
0006398 // m	0005654 // nu	0005515 // pr	U7 snRNA-ass	LSM11_HUMAN	LSM11
0007275 // m	0030529 // in	0003723 // RN	Protein LSM14	LS14B_HUMAN	LSM14B|C20orf40|FAM61B|RAP55B
0043928 // ex	0016020 // m	0003723 // RN	U6 snRNA-ass	LSM4_HUMAN	LSM4
	0016021 // in	0005515 // pr	Leucine-rich s	LSME1_HUMAN	LSMEM1|C7orf53
0006968 // ce	0070062 // ex	0004871 // sig	Lymphocyte-s	LSP1_HUMAN	LSP1|WP34
0001889 // liv	0005886 // pl	0005515 // pr	Lipolysis-stim	LSR_HUMAN	LSR|LISCH
0006695 // ch	0005789 // en	0000250 // la	Lanosterol syn	ERG7_HUMAN	LSS|OSC
0019370 // le	0005737 // cy	0003723 // RN	Leukotriene A	LKHA4_HUMAN	LTA4H|LTA4
0006936 // m	0005886 // pl	0001632 // le	Leukotriene B	LT4R1_HUMAN	LTB4R|BLT|BLT1|BLTR|CMKRL1|GPR16
0007194 // ne	0005886 // pl	0004974 // le	Leukotriene B	LT4R2_HUMAN	LTB4R2|BLT2R|BLTR2
0035583 // se	0031012 // ex	0005509 // ca	Latent-transfo	LTBP1_HUMAN	LTBP1
0097435 // fib	0005622 // in	0005509 // ca	Latent-transfo	LTBP2_HUMAN	LTBP2|C14orf141|LTBP3
0036363 // tra	0031012 // ex	0005509 // ca	Latent-transfo	LTBP3_HUMAN	LTBP3
0045595 // re	0005576 // ex	0005515 // pr	Latent-transfo	LTBP4_HUMAN	LTBP4
0098869 // ce	0005635 // nu	0008289 // lip	Leukotriene C	LTC4S_HUMAN	LTC4S
0010976 // po	0005622 // in	0005515 // pr	Leukocyte tyr	LTK_HUMAN	LTK|TYK1
0000056 // rib	0030688 // pr	0005515 // pr	Protein LTV1 h	LTV1_HUMAN	LTV1|C6orf93
0008380 // RN	0005685 // U1	0003723 // RN	Luc7-like prot	LC7L3_HUMAN	LUC7L3|CREAP1|CROP|O48
0018146 // ke	0005796 // Go	0005515 // pr	Lumican	LUM_HUMAN	LUM|LDC|SLRR2D
0031032 // ac	0042641 // ac	0005515 // pr	Leucine rich a	LURA1_HUMAN	LURAP1|C1orf190|LRAP35A|LRP35A
0021503 // ne	0005634 // nu		Leucine zippe	LUZP1_HUMAN	LUZP1
	0005576 // ex		Leucine zippe	LUZP2_HUMAN	LUZP2|UNQ2566/PRO6246
0010951 // ne	0070062 // ex	0005515 // pr	Latexin	LXN_HUMAN	LXN
0007155 // ce	0016020 // m		Lymphocyte a	LY6D_HUMAN	LY6D|E48
0007166 // ce	0005887 // in		Lymphocyte a	LY6E_HUMAN	LY6E|9804|RIGE|SCA2|TSA1
0051260 // pr	0005576 // ex	0042802 // ide	Lymphocyte a	LY65C_HUMAN	LY6G5C|C6orf20|G5C|NG33
0007339 // bi	0045121 // m		Lymphocyte a	LY6K_HUMAN	LY6K|CO16
0006897 // en	0070062 // ex	0030246 // ca	Lymphocyte a	LY75_HUMAN	LY75|CD205|CLEC13B
0009253 // pe	0005576 // ex	0003796 // lys	Lysozyme g-lik	LYG1_HUMAN	LYG1|UNQ1939/PRO4422
0006355 // re	0005634 // nu	0005515 // pr	Protein lyl-1	LYL1_HUMAN	LYL1|BHLHA18

0001934 // pc	0005829 // cy	0004713 // pr	Tyrosine-prot	LYN_HUMAN	LYN|JTK8
0001775 // ce	0031225 // ar	0030550 // ac	Ly6/PLAUR dc	LYPD1_HUMAN	LYPD1|LYPDC1|PSEC0181|UNQ3079/PF
0001775 // ce	0031225 // ar		Ly6/PLAUR dc	LYPD2_HUMAN	LYPD2|LYPDC2|UNQ430/PRO788
0006501 // C-	0046658 // ar	0043236 // lai	Ly6/PLAUR dc	LYPD3_HUMAN	LYPD3|C4.4A|UNQ491/PRO1007
0006501 // C-	0031225 // ar	0043236 // lai	Ly6/PLAUR dc	LYPD5_HUMAN	LYPD5|UNQ1908/PRO4356
0099601 // re	0031225 // ar	0030548 // ac	Ly6/PLAUR dc	LPD6B_HUMAN	LYPD6B
0002084 // pr	0070062 // ex	0004622 // lys	Lysophosphol	LYPL1_HUMAN	LYPLAL1
	0005739 // m	0005515 // pr	LYR motif-con	LYRM2_HUMAN	LYRM2
	0016021 // in		LysM and puta	LYSM3_HUMAN	LYSMD3
0042742 // de	0005737 // cy	0005515 // pr	Lysosomal-tra	LYST_HUMAN	LYST|CHS|CHS1
0010212 // re		0008013 // be	Protein LZIC	LZIC_HUMAN	LZIC
1903565 // ne	0005829 // cy	0005515 // pr	Leucine zippe	LZTL1_HUMAN	LZTFL1
0009653 // ar	0005794 // Gc	0003700 // tra	Leucine-zippe	LZTR1_HUMAN	LZTR1|TCFL2
0061001 // re	0005737 // cy		Leucine zippe	LZTS3_HUMAN	LZTS3|KIAA0552|PROSAPIP1
0015761 // m	0005886 // pl	0004888 // tra	Cation-depen	MPRD_HUMAN	M6PR|MPR46|MPRD
0003341 // cil	0001536 // ra	0005515 // pr	Cilia- and flag	CFA91_HUMAN	MAATS1|AAT1|C3orf15|CFAP91
0043010 // ca	0005634 // nu	0005525 // G	Putative nucle	MB211_HUMAN	MAB21L1|CAGR1|Nbla00126
		0005515 // pr	Protein mab-2	MB213_HUMAN	MAB21L3|C1orf161
0051781 // pc	0005737 // cy	0008083 // gr	Metastasis-as	MACC1_HUMAN	MACC1
0016055 // W	0005737 // cy	0003779 // ac	Microtubule-a	MACF1_HUMAN	MACF1|ABP620|ACF7|KIAA0465|KIAA
0051725 // pr	0005634 // nu	0016798 // hy	O-acetyl-ADP-	MACD1_HUMAN	MACROD1|LRP16
0051725 // pr	0005730 // nu	0016798 // hy	O-acetyl-ADP-	MACD2_HUMAN	MACROD2|C20orf133
0001558 // re	0005680 // ar	0005515 // pr	Mitotic spindl	MD2L2_HUMAN	MAD2L2|MAD2B|REV7
0002687 // pc	0016021 // in	0098640 // in	Mucosal addr	MADCA_HUMAN	MADCAM1
0006355 // re	0005634 // nu	0003700 // tra	Transcription	MAFA_HUMAN	MAFA
	0005829 // cy		Melanoma-as	MAGAA_HUMAN	MAGEA10|MAGE10
0008150 // bi	0005575 // ce	0005515 // pr	Melanoma-as	MAGAC_HUMAN	MAGEA12|MAGE12
0072331 // sig	0005634 // nu	0005515 // pr	Melanoma-as	MAGA2_HUMAN	MAGEA2|MAGEA2B|MAGE2|MAGE2|
0010955 // ne	0005783 // er	0089720 // ca	Melanoma-as	MAGA3_HUMAN	MAGEA3|MAGE3
0008150 // bi	0005575 // ce	0005515 // pr	Melanoma-as	MAGA4_HUMAN	MAGEA4|MAGE4
0051983 // re	0005575 // ce	0003674 // m	Melanoma-as	MAGA5_HUMAN	MAGEA5|MAGE5
0008150 // bi	0005575 // ce	0005515 // pr	Melanoma-as	MAGA6_HUMAN	MAGEA6|MAGE6
		0005515 // pr	Melanoma-as	MAGC1_HUMAN	MAGEC1
0045893 // pc	0005886 // pl	0005515 // pr	Melanoma-as	MAGD1_HUMAN	MAGED1|NRAGE|PP2250|PRO2292
0002576 // pl	0005576 // ex	0005515 // pr	Melanoma-as	MAGD2_HUMAN	MAGED2|BCG1
0008150 // bi	0048471 // pe	0005515 // pr	Melanoma-as	MAGE1_HUMAN	MAGEE1|HCA1|KIAA1587
0006915 // ap	0005737 // cy	0005515 // pr	Melanoma-as	MAGH1_HUMAN	MAGEH1|APR1
0007166 // ce	0005912 // ac	0005515 // pr	Membrane-as	MAGI1_HUMAN	MAGI1|AIP3|BAIAP1|BAP1|TNRC19
0003402 // pl	0005770 // lat	0004871 // sig	Membrane-as	MAGI2_HUMAN	MAGI2|ACVRINP1|AIP1|KIAA0705

0031124 // m	0043025 // ne	0005515 // pr	Protein mago	MGN2_HUMAN|MAGOHB|MAGOH2
0051204 // pr	0005743 // m	0005515 // pr	m-AAA protea	MAIP1_HUMAN|MAIP1|C2orf47
0007275 // m	0030496 // m	0005515 // pr	Serine/threon	MAK_HUMAN|MAK
0008104 // pr	0031410 // cy	0005515 // pr	MAL-like prot	MALL_HUMAN|MALL|BENE
0070858 // ne	0005794 // Go		MAM and LDL	MALR1_HUMAN|MALRD1|C10orf112|DIET1
0051168 // nu	0005829 // cy	0008233 // pe	Mucosa-assoc	MALT1_HUMAN|MALT1|MLT
0006367 // tra	0005654 // nu	0005515 // pr	Mastermind-l	MAML1_HUMAN|MAML1|KIAA0200
0006367 // tra	0005634 // nu	0003713 // tra	Mastermind-l	MAML2_HUMAN|MAML2|KIAA1819
0006367 // tra	0005634 // nu	0003713 // tra	Mastermind-l	MAML3_HUMAN|MAML3|KIAA1816
0006351 // tra	0005634 // nu	0001076 // tra	MEF2-activati	MASTR_HUMAN|MAMSTR|MASTR
0006013 // m	0043202 // lys	0008496 // m	Epididymis-sp	MA2B2_HUMAN|MAN2B2|KIAA0935
0006013 // m	0005773 // va	0004559 // alp	Alpha-mannos	MA2C1_HUMAN|MAN2C1|MANA|MANA1
0006464 // ce	0005886 // pl	0005537 // m	Beta-mannosi	MANBA_HUMAN|MANBA|MANB1
	0016021 // int	0016798 // hy	Glycoprotein	MANEL_HUMAN|MANEAL
	0016021 // int		MANSC doma	MANS1_HUMAN|MANSC1|LOH12CR3|UNQ316/PRO361
0042135 // ne	0005739 // m	0008131 // pr	Amine oxidase	AOFA_HUMAN|MAOA
0045964 // po	0005739 // m	0008131 // pr	Amine oxidase	AOFB_HUMAN|MAOB
0032886 // re	0097431 // m	0008017 // m	Microtubule-a	MAP10_HUMAN|MAP10|KIAA1383|MTR120
0048675 // ax	0014069 // po	0005198 // str	Microtubule-a	MAP1B_HUMAN|MAP1B
0000422 // m	0005829 // cy	0005515 // pr	Microtubule-a	MLP3A_HUMAN|MAP1LC3A
0007420 // br	0005874 // m	0008017 // m	Microtubule-a	MAP1S_HUMAN|MAP1S|BPY2IP1|C19orf5|MAP8|VCY2
0030010 // es	0005875 // m	0002162 // dy	Microtubule-a	MTAP2_HUMAN|MAP2
0030182 // ne	0005829 // cy	0004712 // pr	Dual specificit	MP2K1_HUMAN|MAP2K1|MEK1|PRKMK1
0009611 // re	0005829 // cy	0004713 // pr	Dual specificit	MP2K4_HUMAN|MAP2K4|JNKK1|MEK4|MKK4|PRKMK4
0043065 // po	0005829 // cy	0004674 // pr	Dual specificit	MP2K6_HUMAN|MAP2K6|MEK6|MKK6|PRKMK6|SKK3
0018107 // pe	0030426 // gr	0042803 // pr	Mitogen-activ	M3K12_HUMAN|MAP3K12|ZPK
0000186 // ac	0005622 // int	0004709 // M	Mitogen-activ	M3K15_HUMAN|MAP3K15|ASK3
0007010 // cy	0005737 // cy	0003723 // RN	Mitogen-activ	M3K20_HUMAN|MAP3K20|MLTK|ZAK|HCCS4
0046777 // pr	0005737 // cy	0004706 // JU	Mitogen-activ	M3K21_HUMAN|MAP3K21|KIAA1804|MLK4
1900745 // po	0005829 // cy	0005515 // pr	Mitogen-activ	M3K3_HUMAN|MAP3K3|MAPKKK3|MEKK3
0071356 // ce	0005829 // cy	0005515 // pr	Mitogen-activ	M3K5_HUMAN|MAP3K5|ASK1|MAPKKK5|MEKK5
0000186 // ac	0005829 // cy	0005515 // pr	MAP3K7 C-ter	M3KCL_HUMAN|MAP3K7CL|C21orf7|TAK1L
0023014 // sig	0005829 // cy	0005515 // pr	Mitogen-activ	M3K8_HUMAN|MAP3K8|COT|ESTF
0043066 // ne	0005737 // cy	0004111 // cr	Mitogen-activ	M4K4_HUMAN|MAP4K4|HGK|KIAA0687|NIK
0007257 // ac	0005737 // cy	0005515 // pr	Mitogen-activ	M4K5_HUMAN|MAP4K5
0007163 // es	0005829 // cy	0005515 // pr	Ensconsin	MAP7_HUMAN|MAP7
0000226 // m	0005737 // cy	0015631 // tu	MAP7 domair	MA7D3_HUMAN|MAP7D3|MDP3
0006468 // pr	0005737 // cy	0005515 // pr	Mitogen-activ	MK10_HUMAN|MAPK10|JNK3|JNK3A|PRKM10|SAPK1B
0007265 // Ra	0005829 // cy	0004707 // M	Mitogen-activ	MK11_HUMAN|MAPK11|PRKM11|SAPK2|SAPK2B
0018105 // pe	0005829 // cy	0004674 // pr	Mitogen-activ	MK12_HUMAN|MAPK12|ERK6|SAPK3

0035556 // in	0005829 // cy	0004707 // M	Mitogen-activ	MK13_HUMAN|MAPK13|PRKM13|SAPK4
0051973 // pc	0005576 // ex	0017124 // SH	Mitogen-activ	MK15_HUMAN|MAPK15|ERK7|ERK8
		0005515 // pr	MAPK-interac	MISSL_HUMAN|MAPK1IP1L|C14orf32
0061308 // ca	0005829 // cy	0005515 // pr	Mitogen-activ	MK03_HUMAN|MAPK3|ERK1|PRKM3
0034115 // ne	0005829 // cy	0051019 // m	Mitogen-activ	MK07_HUMAN|MAPK7|BMK1|ERK5|PRKM7
0018107 // pe	0005654 // nu	0004705 // JU	Mitogen-activ	MK08_HUMAN|MAPK8|JNK1|PRKM8|SAPK1|SAPK1C
0043508 // ne	0005737 // cy	0005515 // pr	C-Jun-amino-t	JIP1_HUMAN|MAPK8IP1|IB1|JIP1|PRKM8IP
0046958 // nc	0043234 // pr	0005515 // pr	C-Jun-amino-t	JIP2_HUMAN|MAPK8IP2|IB2|JIP2|PRKM8IPL
0071310 // ce	0005739 // m	0008134 // tra	Mitogen-activ	MK09_HUMAN|MAPK9|JNK2|PRKM9|SAPK1A
1900034 // re	0005829 // cy	0005515 // pr	MAP kinase-a	MAPK2_HUMAN|MAPKAPK2
0044351 // m	0005829 // cy	0005515 // pr	MAP kinase-a	MAPK3_HUMAN|MAPKAPK3
0000086 // G2	0005829 // cy	0003723 // RN	Microtubule-a	MARE1_HUMAN|MAPRE1
0045737 // pc	0030496 // m	0005515 // pr	Microtubule-a	MARE3_HUMAN|MAPRE3
0051259 // pr	0097418 // ne	0005515 // pr	Microtubule-a	TAU_HUMAN|MAPT|MAPTL|MTBT1|TAU
0042126 // ni	0005739 // m	0030170 // py	Mitochondria	MARC1_HUMAN|MARC1|MOSC1
0042126 // ni	0005777 // pe	0030170 // py	Mitochondria	MARC2_HUMAN|MARC2|MOSC2
0006955 // im	0016021 // in	0008270 // zi	E3 ubiquitin-p	MARH1_HUMAN|MARCH1|RNF171
0006897 // er	0005765 // ly	0005515 // pr	E3 ubiquitin-p	MARH2_HUMAN|MARCH2|RNF172|HSPC240
0006897 // er	0016021 // in	0008270 // zi	E3 ubiquitin-p	MARH3_HUMAN|MARCH3|RNF173
0016567 // pr	0016021 // in	0008270 // zi	E3 ubiquitin-p	MARH4_HUMAN|MARCH4|KIAA1399|RNF174
0070936 // pr	0000835 // ER	0008270 // zi	E3 ubiquitin-p	MARH6_HUMAN|MARCH6|KIAA0597|RNF176|TEB4
0000209 // pr	0005765 // ly	0008270 // zi	E3 ubiquitin-p	MARH8_HUMAN|MARCH8|MIR|RNF178
0016567 // pr	0005765 // ly	0008270 // zi	E3 ubiquitin-p	MARH9_HUMAN|MARCH9|RNF179
0008284 // pc	0070062 // ex	0005515 // pr	MARCKS-relat	MRP_HUMAN|MARCKSL1|MLP|MRP
0006468 // pr	0005856 // cy	0070300 // ph	Serine/threon	MARK1_HUMAN|MARK1|KIAA1477|MARK
0016055 // W	0005884 // ac	0004674 // pr	Serine/threon	MARK2_HUMAN|MARK2|EMK1
0000226 // m	0005829 // cy	0005515 // pr	MAP/microtu	MARK3_HUMAN|MARK3|CTAK1|EMK2
0006418 // tR	0005759 // m	0004825 // m	Methionine--t	SYMM_HUMAN|MARS2
0061028 // es	0005923 // bi	0005515 // pr	MARVEL dom	MALD2_HUMAN|MARVELD2|TRIC
0006970 // re	0005923 // bi	0005515 // pr	MARVEL dom	MALD3_HUMAN|MARVELD3|MRVLDC3
0006508 // pr	0005576 // ex	0042803 // pr	Mannan-bindi	MASP1_HUMAN|MASP1|CRARF|CRARF1|PRSS5
0006508 // pr	0005576 // ex	0005509 // ca	Mannan-bindi	MASP2_HUMAN|MASP2
0018105 // pe	0005622 // in	0004674 // pr	Microtubule-a	MAST3_HUMAN|MAST3|KIAA0561
0018105 // pe	0005737 // cy	0004674 // pr	Microtubule-a	MAST4_HUMAN|MAST4|KIAA0303
0006556 // S-	0048269 // m	0019899 // er	Methionine a	MAT2B_HUMAN|MAT2B|TGR|MSTP045|Nbla02999|UN
0030500 // re	0005576 // ex	0005201 // ex	Cartilage mat	MATN1_HUMAN|MATN1|CMP|CRTM
0031104 // de	0005604 // ba	0005515 // pr	Matrilin-2	MATN2_HUMAN|MATN2|UNQ193/PRO219
0001501 // sk	0005578 // pr	0005515 // pr	Matrilin-3	MATN3_HUMAN|MATN3
0006461 // pr	0071339 // M	0000978 // RN	Protein max	MAX_HUMAN|MAX|BHLHD4
0006367 // tra	0005634 // nu	0003723 // RN	Myc-associate	MAZ_HUMAN|MAZ|ZNF801
0031444 // slo	0070062 // ex	0019825 // ox	Myoglobin	MYG_HUMAN|MB
		0032403 // pr	Protein MB21	M21D2_HUMAN|MB21D2|C3orf59
0035563 // pc	0000792 // he	0005515 // pr	Methyl-CpG-b	MBD2_HUMAN|MBD2
0001701 // in	0016581 // Nu	0003677 // DI	Methyl-CpG-b	MBD3_HUMAN|MBD3

0045008 // de	0005654 // nu	0005515 // pr	Methyl-CpG-b	MBD4_HUMAN|MBD4|MED1
0016579 // pr	0010369 // ch	0003677 // DI	Methyl-CpG-b	MBD6_HUMAN|MBD6|KIAA1887
0032324 // m	0005829 // cy	0005515 // pr	MAP3K12-bin	MBIP1_HUMAN|MBIP|BM-015
0045445 // m	0005634 // nu	0003723 // RI	Muscleblind-li	MBNL1_HUMAN|MBNL1|EXP|KIAA0428|MBNL
0008380 // RI	0005737 // cy	0003723 // RI	Muscleblind-li	MBNL2_HUMAN|MBNL2|MBLL|MBLL39|MLP1
0007275 // m	0005634 // nu	0003723 // RI	Muscleblind-li	MBNL3_HUMAN|MBNL3|CHCR|MBLX39|MBXL
0007268 // ch	0043218 // co	0002020 // pr	Myelin basic p	MBP_HUMAN|MBP
0007040 // ly	0000139 // Go	0004252 // se	Membrane-bo	MBTP1_HUMAN|MBTPS1|KIAA0091|S1P|SKI1
0051091 // po	0016021 // in	0004222 // m	Membrane-bo	MBTP2_HUMAN|MBTPS2|S2P
0007155 // ce	0009897 // ex		Cell surface gl	MUC18_HUMAN|MCAM|MUC18
0008152 // m	0005739 // m	0003723 // RI	Malonyl-CoA-	FABD_HUMAN|MCAT|MT
0006552 // le	0005759 // m	0004075 // bi	Methylcroton	MCCA_HUMAN|MCCC1|MCCA
0043547 // po	0005856 // cy	0005089 // Rh	Proto-oncoge	MCF2_HUMAN|MCF2|DBL
0043547 // po	0012505 // en	0035091 // ph	Guanine nucle	MCF2L_HUMAN|MCF2L|KIAA0362|OST
0043547 // po		0005089 // Rh	Probable guar	MF2L2_HUMAN|MCF2L2|DRG|KIAA0861
0006888 // ER	0005789 // en	0005509 // ca	Multiple coag	MCFD2_HUMAN|MCFD2|SDNSF
0007346 // re	0016604 // nu	0042802 // id	Multicilin	MCIN_HUMAN|MCIDAS|IDAS|MCI|MCIN
0006260 // DI	0005654 // nu	0005515 // pr	Protein MCM1	MCM10_HUMAN|MCM10|PRO2249
0006260 // DI	0005654 // nu	0005515 // pr	DNA replicatio	MCM5_HUMAN|MCM5|CDC46
0006260 // DI	0005654 // nu	0005515 // pr	DNA helicase	MCM8_HUMAN|MCM8|C20orf154
0006270 // DI		0005524 // AT	MCM domain	MCMD2_HUMAN|MCMDC2|C8orf45
0002250 // ac	0005765 // ly	0005262 // ca	Mucolipin-2	MCLN2_HUMAN|MCOLN2
0042491 // au	0005765 // ly	0005262 // ca	Mucolipin-3	MCLN3_HUMAN|MCOLN3
0010717 // re	0005634 // nu	0005515 // pr	Mapk-regulato	MCRI1_HUMAN|MCRIP1|FAM195B|GRAN2
	0005634 // nu	0005515 // pr	MAPK regulat	MCRI2_HUMAN|MCRIP2|C16orf14|FAM195A
0030336 // ne	0005789 // en	0005544 // ca	Multiple C2 ar	MCTP1_HUMAN|MCTP1
0007275 // m	0005783 // en	0005544 // ca	Multiple C2 ar	MCTP2_HUMAN|MCTP2
0032790 // rib	0022627 // cy	0005515 // pr	Malignant T-c	MCTS1_HUMAN|MCTS1|MCT1
0036444 // ca	0031305 // in	0005515 // pr	Calcium unipo	MCU_HUMAN|MCU|C10orf42|CCDC109A
0051560 // m	0034704 // ca	0005216 // io	Calcium unipo	MCUB_HUMAN|MCUB|CCDC109B
0000122 // ne	0005634 // nu	0005515 // pr	MyoD family i	MDFI_HUMAN|MDFI
0016032 // vi	0005634 // nu	0005515 // pr	MyoD family i	MDFIC_HUMAN|MDFIC
0021522 // sp	0031225 // an		MAM domain	MDGA2_HUMAN|MDGA2|MAMDC1|UNQ8188/PRO231
0030325 // ad	0005737 // cy	0008083 // gr	Midkine	MK_HUMAN|MDK|MK1|NEGF2
0070301 // ce	0005654 // nu	0005515 // pr	E3 ubiquitin-p	MDM2_HUMAN|MDM2
0071456 // ce	0005654 // nu	0005515 // pr	Protein Mdm4	MDM4_HUMAN|MDM4|MDMX
0035335 // pe	0070062 // ex	0004725 // pr	Magnesium-d	MGDP1_HUMAN|MDP1
0009725 // re	0005739 // m	0004470 // m	NADP-depend	MAOX_HUMAN|ME1
0072592 // ox	0005739 // m	0048037 // co	NADP-depend	MAON_HUMAN|ME3
0043984 // hi	0000777 // co	0005515 // pr	Chromatin mo	EAF6_HUMAN|MEAF6|C1orf149|CENP-28|EAF6
0045892 // ne	0000118 // his	0005515 // pr	MDS1 and EVI	EVI1_HUMAN|MECOM|EVI1
0045944 // po	0016592 // m	0008013 // be	Mediator of R	MD12L_HUMAN|MED12L|KIAA1635|TNRC11L|TRALP|T

0006367 // tra	0005654 // nu	0005515 // pr	Mediator of R	MED14_HUMAN|MED14|ARC150|CRSP2|CXorf4|DRIP1!
0019827 // ste	0016020 // m	0005515 // pr	Mediator of R	MED28_HUMAN|MED28|EG1|FKSG20
0016567 // pr	0005654 // nu	0030374 // lig	Mediator of R	MED30_HUMAN|MED30|THRAP6|TRAP25
0051123 // RN	0005654 // nu	0005515 // pr	Mediator of R	MED6_HUMAN|MED6|ARC33
0006357 // re	0016592 // m	0005515 // pr	Mediator of R	MED8_HUMAN|MED8
0055005 // ve	0005654 // nu	0042826 // his	Myocyte-spec	MEF2A_HUMAN|MEF2A|MEF2
0001782 // B	0005654 // nu	0000981 // RN	Myocyte-spec	MEF2C_HUMAN|MEF2C
0001958 // en	0005634 // nu	0000977 // RN	Myocyte-spec	MEF2D_HUMAN|MEF2D
0043652 // en	0016021 // in	0001849 // co	Multiple epide	MEG10_HUMAN|MEGF10|KIAA1780
0034109 // ho	0016021 // in		Multiple epide	MEG11_HUMAN|MEGF11|KIAA1781|UNQ1949/PRO443
0008150 // bi	0005576 // ex	0005515 // pr	Multiple epide	MEGF6_HUMAN|MEGF6|EGFL3|KIAA0815
0008150 // bi	0016021 // in		Multiple epide	MEGF9_HUMAN|MEGF9|EGFL5|KIAA0818|UNQ671/PR(
0007127 // m	0005623 // ce		Meiosis inhibi	MEI1_HUMAN|MEI1
0048477 // oo	0000800 // lat		Meiosis-specif	MEI4_HUMAN|MEI4
0001525 // an	0005667 // tra	0005515 // pr	Homeobox pr	MEIS1_HUMAN|MEIS1
0001654 // ey	0005634 // nu	0000978 // RN	Homeobox pr	MEIS2_HUMAN|MEIS2|MRG1
2001234 // ne	0005634 // nu	0003682 // ch	Homeobox pr	MEIS3_HUMAN|MEIS3|MRG2
0006355 // re	0005634 // nu	0003677 // DN	Putative home	ME3L1_HUMAN|MEIS3P1
0001558 // re	0005887 // in	0005506 // iro	Melanotransfe	TRFM_HUMAN|MELTF|MAP97|MFI2
0060218 // he	0005634 // nu	0001077 // tra	Homeobox pr	MEOX1_HUMAN|MEOX1|MOX1
0006366 // tra	0005634 // nu	0005515 // pr	Homeobox pr	MEOX2_HUMAN|MEOX2|GAX|MOX2
0006508 // pr	0005887 // in	0005515 // pr	Meprin A subu	MEP1A_HUMAN|MEP1A
0001501 // sk	0005578 // pr	0005201 // ex	Matrix extrace	MEPE_HUMAN|MEPE
0001707 // m	0005634 // nu	0003700 // tra	Mesoderm po	MESP1_HUMAN|MESP1|BHLHC5
0001707 // m	0070062 // ex	0000978 // RN	Mesoderm po	MESP2_HUMAN|MESP2|BHLHC6|SCDO2
0007498 // m	0016021 // in	0016787 // hy	Mesoderm-sp	MEST_HUMAN|MEST|PEG1
0018206 // pe	0005829 // cy	0003723 // RN	Methionine ar	MAP2_HUMAN|METAP2|MNPEP|P67EIF2
0010001 // gli	0005615 // ex		Meteorin	METRN_HUMAN|METRN|C16orf23|RJD6
0032259 // m		0005515 // pr	Methyltransfe	MET13_HUMAN|METTL13|KIAA0859|CGI-01
0070475 // rR		0071424 // rR	Putative meth	ME15P_HUMAN|METTL15P1|METT5D2
0032259 // m		0008168 // m	Putative meth	MT21E_HUMAN|METTL21EP|METTL21CP1
0032259 // m		0008168 // m	Methyltransfe	MET25_HUMAN|METTL25|C12orf26
0000398 // m	0005634 // nu	0016422 // m	N6-adenosine	MTA70_HUMAN|METTL3|MTA70
0032259 // m	0016020 // m	0008168 // m	Methyltransfe	MET7A_HUMAN|METTL7A|PRO0066|UNQ1902/PRO434
0032259 // m		0008168 // m	Methyltransfe	MET7B_HUMAN|METTL7B|UNQ594/PRO1180
		0005515 // pr	Methyltransfe	METL9_HUMAN|METTL9|DREV|CGI-81
0006468 // pr	0005829 // cy	0003723 // RN	RNA-binding p	MEX3B_HUMAN|MEX3B|KIAA2009|RKHD3|RNF195
0097009 // en	0005634 // nu	0003723 // RN	RNA-binding E	MEX3C_HUMAN|MEX3C|RKHD2|RNF194|BM-013
0010609 // m	0005634 // nu	0003723 // RN	RNA-binding p	MEX3D_HUMAN|MEX3D|KIAA2031|RKHD1|RNF193
	0016021 // in	0005515 // pr	Microfibrillar-	MFA3L_HUMAN|MFAP3L|KIAA0626|HSD-39|HSD39
0008053 // m	0005739 // m	0003924 // G	Mitofusin-1	MFN1_HUMAN|MFN1

0045747 // pc	0030173 // in	0033829 // O-	Beta-1,3-N-ac	MFNG_HUMAN|MFNG
0006810 // tra	0016021 // in		Major facilitat	MFS12_HUMAN|MFSD12|C19orf28
0055085 // tra	0016021 // in	0005215 // tra	Hippocampus	MF14A_HUMAN|MFSD14A|HIAT1
0055085 // tra	0016021 // in	0005215 // tra	Hippocampus	MF14B_HUMAN|MFSD14B|HIATL1
0055085 // tra	0016021 // in	0005215 // tra	Hippocampus	MF14C_HUMAN|MFSD14C|HIATL2
0015992 // pr	0005887 // in	0015295 // so	Major facilitat	MFSD3_HUMAN|MFSD3
1904659 // glu	0016021 // in	0005355 // glu	Major facilitat	MFD4A_HUMAN|MFSD4A|MFSD4|UNQ3064/PRO9894
	0016021 // in		Major facilitat	MFSD6_HUMAN|MFSD6|MMR2
0055085 // tra	0016021 // in		Major facilitat	MFSD7_HUMAN|MFSD7|LP2561|UNQ385/PRO717
0055085 // tra	0016021 // in		Major facilitat	MFSD8_HUMAN|MFSD8|CLN7
0006487 // pr	0016021 // in	0003830 // be	Beta-1,4-man	MGAT3_HUMAN|MGAT3|GGNT3
0006487 // pr	0016021 // in	0008454 // alp	Alpha-1,3-man	MGT4A_HUMAN|MGAT4A
0006487 // pr	0016021 // in	0008454 // alp	Alpha-1,3-man	MGT4B_HUMAN|MGAT4B|UNQ906/PRO1927
0006487 // pr	0016021 // in	0005515 // pr	Alpha-1,6-man	MGT5B_HUMAN|MGAT5B|KIAA2008
0006954 // in	0019898 // ex	0047372 // ac	Monoglycerid	MGLL_HUMAN|MGLL
0009636 // re	0005654 // nu	0003677 // DN	Methylated-D	MGMT_HUMAN|MGMT
0008333 // en	0070062 // ex	0004842 // ub	E3 ubiquitin-p	MGRN1_HUMAN|MGRN1|KIAA0544|RNF156
1901687 // glu	0005789 // en	0004602 // glu	Microsomal gl	MGST2_HUMAN|MGST2|GST2
0006888 // EF	0005789 // en	0005515 // pr	Transport and	TGO1_HUMAN|MIA3|KIAA0268|TANGO|UNQ6077/PRO
0007165 // sig	1990026 // hi	0004497 // m	[F-actin]-meth	MICA1_HUMAN|MICAL1|MICAL|NICAL
0019417 // su	0005634 // nu	0016709 // ox	[F-actin]-meth	MICA2_HUMAN|MICAL2|KIAA0750|MICAL2PV1|MICAL2
0055114 // ox	0005938 // ce	0003779 // ac	[F-actin]-meth	MICA3_HUMAN|MICAL3|KIAA0819|KIAA1364
0007283 // sp	0005737 // cy	0051019 // m	MICAL C-term	MICLK_HUMAN|MICALCL
0031175 // ne	0055038 // re	0005515 // pr	MICAL-like pro	MILK1_HUMAN|MICALL1|KIAA1668|MIRAB13
0034446 // su	0032432 // ac	0017137 // Ra	MICAL-like pro	MILK2_HUMAN|MICALL2|JRAB
0050776 // re	0016021 // in	0046703 // na	MHC class I po	MICB_HUMAN|MICB|PERB11.2
0007389 // pa	0005875 // m	0005515 // pr	E3 ubiquitin-p	TRI18_HUMAN|MID1|FXY|RNF59|TRIM18|XPRF
0046890 // re	0005829 // cy	0008022 // pr	Mid1-interact	M1IP1_HUMAN|MID1IP1|MIG12
0045087 // in	0070062 // ex	0008270 // zin	Probable E3 u	TRIM1_HUMAN|MID2|FXY2|RNF60|TRIM1
0008053 // m	0005741 // m	0005515 // pr	Mitochondria	MID51_HUMAN|MIEF1|MID51|SMCR7L
0006351 // tra	0005634 // nu	0042826 // his	Mesoderm ind	MIER2_HUMAN|MIER2|KIAA1193
0006351 // tra	0005634 // nu	0003677 // DN	Mesoderm ind	MIER3_HUMAN|MIER3
0032269 // ne	0005576 // ex	0004167 // do	Macrophage r	MIF_HUMAN|MIF|GLIF|MMIF
0010972 // ne		0005515 // pr	Migration and	MIIP_HUMAN|MIIP|IIP45
0033004 // ne	0042629 // m		Allergin-1	MILR1_HUMAN|MILR1|C17orf60|MCA32
0008150 // bi	0070062 // ex	1990380 // Ly	Ubiquitin carb	MINY1_HUMAN|MINDY1|FAM63A|KIAA1390
0008150 // bi	0005654 // nu	0071795 // K1	Ubiquitin carb	MINY2_HUMAN|MINDY2|FAM63B|KIAA1164
0006508 // pr		0036459 // th	Probable ubiq	MINY4_HUMAN|MINDY4|C7orf67|FAM188B
0043647 // in	0070062 // ex	0016701 // ox	Inositol oxyge	MIOX_HUMAN|MIOX|ALDRL6|KSP32|RSOR
0006627 // pr	0005739 // m	0004222 // m	Mitochondria	MIPEP_HUMAN|MIPEP|MIP

	0005634 // nu	0005515 // pr	Mirror-image	MIPO1_HUMAN|MIPOL1
	0016021 // in		Putative micr	MIRH1_HUMAN|MIR17HG|C13orf25|MIRH1|MIRHG1
0007059 // ch	0000818 // nu	0005515 // pr	Protein MIS12	MIS12_HUMAN|MIS12
0000122 // ne	0043234 // pr	0005515 // pr	Microphthalm	MITF_HUMAN|MITF|BHLHE32
0001947 // he	0036064 // cil	0044183 // pr	McKusick-Kau	MKKS_HUMAN|MKKS|BBS6
0045944 // po	0005654 // nu	0005515 // pr	MKL/myocard	MKL1_HUMAN|MKL1|KIAA1438|MAL
0006468 // pr	0005737 // cy	0004674 // pr	MAP kinase-ir	MKNK1_HUMAN|MKNK1|MNK1
0018105 // pe	0016605 // PM	0005515 // pr	MAP kinase-ir	MKNK2_HUMAN|MKNK2|GPRK7|MNK2
0008150 // bi	0005622 // in	0003723 // RN	Probable E3 u	MKRN2_HUMAN|MKRN2|RNF62|HSPC070
0016567 // pr	0030529 // in	0042802 // id	Probable E3 u	MKRN3_HUMAN|MKRN3|D15S9|RNF63|ZNF127
0002318 // m	0005737 // cy	0005515 // pr	Myeloid leuke	MLF1_HUMAN|MLF1
0006298 // m	0005712 // ch	0005524 // AT	DNA mismatc	MLH3_HUMAN|MLH3
0006366 // tra	0005654 // nu	0005515 // pr	Protein ENL	ENL_HUMAN|MLLT1|ENL|LTG19|YEATS1
0045893 // po	0005622 // in	0003674 // m	Protein AF1q	AF1Q_HUMAN|MLLT11|AF1Q
0045893 // po	0008023 // tra	0005515 // pr	Protein AF-9	AF9_HUMAN|MLLT3|AF9|YEATS3
0006355 // re	0005634 // nu	0005515 // pr	Protein AF-17	AF17_HUMAN|MLLT6|AF17
0006886 // in	0030864 // co	0005515 // pr	Melanophilin	MELPH_HUMAN|MLPH|SLAC2A
0045944 // po	0005741 // m	0000977 // RN	MLX-interacti	MLXIP_HUMAN|MLXIP|BHLHE36|KIAA0867|MIR|MOND
0046889 // po	0005634 // nu	0035538 // ca	Carbohydrate	MLXPL_HUMAN|MLXIPL|BHLHD14|MIO|WBSCR14
0071492 // ce	0030425 // de	0004175 // er	Neprilysin	NEP_HUMAN|MME|EPN

0030574 // cc	0005576 // ex	0004222 // m	Interstitial col	MMP1_HUMAN|MMP1|CLG
0030574 // cc	0005576 // ex	0004252 // se	Stromelysin-2	MMP10_HUMAN|MMP10|STMY2
0030574 // cc	0005576 // ex	0004222 // m	Stromelysin-3	MMP11_HUMAN|MMP11|STMY3
0060339 // ne	0005576 // ex	0004222 // m	Macrophage r	MMP12_HUMAN|MMP12|HME
0001958 // er	0005576 // ex	0004222 // m	Collagenase 3	MMP13_HUMAN|MMP13
0030574 // cc	0005886 // pl	0070006 // m	Matrix metall	MMP15_HUMAN|MMP15
0006508 // pr	0005887 // in	0008270 // zi	Matrix metall	MMP16_HUMAN|MMP16|C8orf57|MMPX2
0006508 // pr	0031225 // ar	0070006 // m	Matrix metall	MMP17_HUMAN|MMP17|MT4MMP
0048013 // ep	0005576 // ex	0004222 // m	72 kDa type IV	MMP2_HUMAN|MMP2|CLG4A
0006508 // pr	0005578 // pr	0004222 // m	Matrix metall	MMP25_HUMAN|MMP25|MMP20|MMPL1|MT6MMP
0006508 // pr	0005578 // pr	0004222 // m	Matrix metall	MMP28_HUMAN|MMP28|MMP25|UNQ1893/PRO4339
0030574 // cc	0005576 // ex	0004222 // m	Matrilysin	MMP7_HUMAN|MMP7|MPSL1|PUMP1
0030948 // ne	0005604 // ba	0005515 // pr	Multimerin-2	MMRN2_HUMAN|MMRN2|EMILIN3
0007275 // m	0005575 // ce	0003674 // m	Transcriptiona	MN1_HUMAN|MN1
0006362 // tr	0005654 // nu	0005515 // pr	CDK-activating	MAT1_HUMAN|MNAT1|CAP35|MAT1|RNF66
0006357 // re	0005730 // nu	0003700 // tr	Motor neuron	MNX1_HUMAN|MNX1|HLXB9
0043065 // pc	0005829 // cy	0005515 // pr	Modulator of	MOAP1_HUMAN|MOAP1|PNMA4
0030036 // ac	0044306 // ne	0005515 // pr	MOB kinase a	MOB2_HUMAN|MOB2|HCCA2
	0005622 // in	0046872 // m	MOB kinase a	MOB3A_HUMAN|MOB3A|MOBKL2A
		0046872 // m	MOB kinase a	MOB3B_HUMAN|MOB3B|C9orf35|MOBKL2B
		0005515 // pr	MOB kinase a	MOB3C_HUMAN|MOB3C|MOBKL2C
0006777 // M	0019008 // m	0061799 // cy	Molybdenum	MOCS1_HUMAN|MOCS1|MIG11
0006777 // M	0005829 // cy	0030366 // m	Molybdopteri	MOC2B_HUMAN|MOCS2|MCBPE|MOCO1
0032447 // pr	0005829 // cy	0016783 // su	Adenylyltrans	MOCS3_HUMAN|MOCS3|UBA4
0006071 // gl	0005789 // er	0003846 // 2-	2-acylglycerol	MOGT3_HUMAN|MOGAT3|DC7|DGAT2L7|UNQ9383/PI
0006468 // pr	0005929 // cil	0004693 // cy	MAPK/MAK/M	MOK_HUMAN|MOK|RAGE|RAGE1
0043547 // pc	0005829 // cy	0005515 // pr	Vacuolar fusic	MON1A_HUMAN|MON1A|SAND1
0006895 // Gc	0005829 // cy	0005515 // pr	Protein MON2	MON2_HUMAN|MON2|KIAA1040|SF21
	0005654 // nu	0008270 // zi	MORC family	MORC4_HUMAN|MORC4|ZCW4|ZCWCC2
0007283 // sp	0001669 // ac	0005515 // pr	MORN repeat	MORN2_HUMAN|MORN2|MOPT
	0005634 // nu	0005515 // pr	MORN repeat	MORN3_HUMAN|MORN3
0045944 // pc	0016021 // in		Motile sperm	MSPD1_HUMAN|MOSPD1
0090026 // pc	0005887 // in		Motile sperm	MSPD2_HUMAN|MOSPD2
0042421 // nc	0016021 // in	0005507 // cc	DBH-like mon	MOXD1_HUMAN|MOXD1|MOX|UNQ2493/PRO5780
0006364 // rR	0005654 // nu	0003723 // RN	U3 small nucl	MPP10_HUMAN|MPHOSPH10|MPP10
0045892 // ne	0000151 // ub	0031625 // ub	M-phase phos	MPP8_HUMAN|MPHOSPH8|MPP8
0007166 // ce	0005794 // Gc	0005515 // pr	Thrombopoie	TPOR_HUMAN|MPL|TPOR
0051301 // ce	0030496 // m	0005515 // pr	M-phase-spec	MPLKI_HUMAN|MPLKIP|C7orf11|TTDN1
0046710 // GI	0005887 // in	0004385 // gu	55 kDa erythr	EM55_HUMAN|MPP1|DXS552E|EMP55
0046710 // GI	0032591 // de	0004385 // gu	MAGUK p55 s	MPP2_HUMAN|MPP2|DLG2
0046710 // GI	0005887 // in	0004385 // gu	MAGUK p55 s	MPP3_HUMAN|MPP3|DLG3
	0005737 // cy		MAGUK p55 s	MPP4_HUMAN|MPP4|ALS2CR5|DLG6
0035750 // pr	0043219 // la	0005515 // pr	MAGUK p55 s	MPP5_HUMAN|MPP5
0071896 // pr	0097025 // M	0035591 // si	MAGUK p55 s	MPP7_HUMAN|MPP7

0006888 // EF	0005793 // er	0030145 // m	Metallophosp	MPPE1_HUMAN|MPPE1|PGAP5|PP579
	0005829 // cy	0005515 // pr	Myosin phosp	MPRIP_HUMAN|MPRIP|KIAA0864|MRIP|RHOIP3
0019346 // tra	0070062 // ex	0004792 // th	3-mercaptopy	THTM_HUMAN|MPST|TST2
0034614 // ce	0005777 // pe	0003674 // m	Protein Mpv1	MPV17_HUMAN|MPV17
0061668 // m	0016021 // in		Mpv17-like pr	M17L2_HUMAN|MPV17L2|FKSG24
0098742 // ce	0005791 // ro	0005198 // st	Myelin protei	MYP0_HUMAN|MPZ
0007169 // tra	0005925 // fo	0005515 // pr	Myelin protei	MPZL1_HUMAN|MPZL1|PZR|UNQ849/PRO1787
0007156 // ho	0016021 // in	0005515 // pr	Myelin protei	MPZL2_HUMAN|MPZL2|EVA|EVA1|UNQ606/PRO1192
0007155 // ce	0016021 // in	0005515 // pr	Myelin protei	MPZL3_HUMAN|MPZL3|UNQ2966/PRO7425
0006955 // im	0016021 // in	0005515 // pr	Major histoco	HMR1_HUMAN|MR1
0030819 // po	0016021 // in	0005515 // pr	Melanocortin-	MRAP_HUMAN|MRAP|C21orf61|FALP
0030819 // po	0016021 // in	0031781 // ty	Melanocortin-	MRAP2_HUMAN|MRAP2|C6orf117
0007265 // Ra	0005622 // in	0003924 // GT	Ras-related pr	RASM_HUMAN|MRAS|RRAS3
0016573 // hi	0035267 // Nu	0005515 // pr	MRG/MORF4l	MRGBP_HUMAN|MRGBP|C20orf20
0007186 // G-	0005887 // in	0004930 // G-	Mas-related G	MRGRD_HUMAN|MRGPRD|MRGD
0007186 // G-	0005887 // in	0004930 // G-	Mas-related G	MRGRF_HUMAN|MRGPRF|GPR140|GPR168|MRGF|PSE
0019509 // L-	0005829 // cy	0046523 // S-	Methylthiorib	MTNA_HUMAN|MRI1|MRDI|UNQ6390/PRO21135
1902081 // ne	0016021 // in	0004857 // er	Myoregulin	MLN_HUMAN|MRLN|MLN
	0016021 // in		Maestro heat-	MROH7_HUMAN|MROH7|C1orf175|HEATR8
0006412 // tra	0005743 // m	0003723 // RN	39S ribosoma	RM01_HUMAN|MRPL1|BM-022
0045893 // po	0005743 // m	0003735 // st	39S ribosoma	RM12_HUMAN|MRPL12|MRPL7|RPML12
0070126 // m	0005762 // m	0003723 // RN	39S ribosoma	RM15_HUMAN|MRPL15|HSPC145
0070126 // m	0005762 // m	0003735 // st	39S ribosoma	RM17_HUMAN|MRPL17|LIP2
0006412 // tra	0005762 // m	0008097 // 5S	39S ribosoma	RM18_HUMAN|MRPL18|HSPC071
0070126 // m	0005762 // m	0003735 // st	39S ribosoma	RM19_HUMAN|MRPL19|KIAA0104|MRPL15
0000027 // rib	0005762 // m	0003723 // RN	39S ribosoma	RM20_HUMAN|MRPL20
0070126 // m	0005762 // m	0003723 // RN	39S ribosoma	RM22_HUMAN|MRPL22|MRPL25|RPML25|HSPC158
0006412 // tra	0005762 // m	0003735 // st	39S ribosoma	RM23_HUMAN|MRPL23|L23MRP|RPL23L
0070126 // m	0005762 // m	0003723 // RN	39S ribosoma	RM27_HUMAN|MRPL27|HSPC250
0006412 // tra	0005762 // m	0003723 // RN	39S ribosoma	RM28_HUMAN|MRPL28|MAAT1
0070126 // m	0005762 // m	0003735 // st	39S ribosoma	RM30_HUMAN|MRPL30|MRPL28|RPML28|HSPC249
0006412 // tra	0005762 // m	0003735 // st	39S ribosoma	RM34_HUMAN|MRPL34
0070126 // m	0005762 // m		39S ribosoma	RM38_HUMAN|MRPL38|HSPC262
0070126 // m	0005762 // m	0003723 // RN	39S ribosoma	RM04_HUMAN|MRPL4|CDABP0091|CGI-28
0070126 // m	0005762 // m	0003723 // RN	39S ribosoma	RM40_HUMAN|MRPL40|NLVCF|URIM
0006412 // tra	0005762 // m	0003723 // RN	39S ribosoma	RM41_HUMAN|MRPL41|BMRP|MRPL27|RPML27|PIG3
0070126 // m	0005762 // m	0003723 // RN	39S ribosoma	RM44_HUMAN|MRPL44
0070126 // m	0005762 // m	0003735 // st	39S ribosoma	RM49_HUMAN|MRPL49|C11orf4|NOF1|OK/SW-cl.67
0032543 // m	0005762 // m	0003735 // st	39S ribosoma	RM51_HUMAN|MRPL51|MRP64|CDA09|HSPC241
0006412 // tra	0005762 // m	0003735 // st	39S ribosoma	RM52_HUMAN|MRPL52
0070126 // m	0005762 // m	0005515 // pr	39S ribosoma	RM53_HUMAN|MRPL53

0070126 // m	0005743 // m	0003723 // RN	39S ribosoma	RM54_HUMAN|MRPL54
0032543 // m	0005743 // m	0003723 // RN	28S ribosoma	RT12_HUMAN|MRPS12|RPMS12|RPSM12
0070126 // m	0005743 // m	0003723 // RN	28S ribosoma	RT14_HUMAN|MRPS14
0070126 // m	0005743 // m	0003723 // RN	28S ribosoma	RT15_HUMAN|MRPS15|RPMS15|DC37
0032543 // m	0005743 // m	0003735 // st	28S ribosoma	RT16_HUMAN|MRPS16|RPMS16|CGI-132
0070126 // m	0005743 // m	0019843 // rR	28S ribosoma	RT17_HUMAN|MRPS17|RPMS17|HSPC011
0006412 // tr	0005743 // m	0003735 // st	28S ribosoma	RT02_HUMAN|MRPS2|CGI-91
0032543 // m	0005743 // m	0003723 // RN	28S ribosoma	RT21_HUMAN|MRPS21|RPMS21|MDS016
0070126 // m	0005743 // m	0003723 // RN	28S ribosoma	RT23_HUMAN|MRPS23|CGI-138|HSPC329
0042769 // DI	0005743 // m	0003723 // RN	28S ribosoma	RT26_HUMAN|MRPS26|C20orf193|RPMS13
0008283 // ce	0005743 // m	0005515 // pr	28S ribosoma	RT27_HUMAN|MRPS27|KIAA0264
0070126 // m	0005743 // m	0003735 // st	28S ribosoma	RT33_HUMAN|MRPS33|CGI-139|PTD003
0070126 // m	0005743 // m	0003735 // st	28S ribosoma	RT34_HUMAN|MRPS34
0042769 // DI	0005763 // m	0003723 // RN	28S ribosoma	RT35_HUMAN|MRPS35|MRPS28|HDCMD11P|MDS023|
0070126 // m	0005743 // m	0003735 // st	28S ribosoma	RT36_HUMAN|MRPS36|DC47
0000956 // nu	0005730 // nu	0003723 // RN	mRNA turnov	MRT4_HUMAN|MRTO4|C1orf33|MRT4
		0042802 // id	Myb/SANT-lik	MSD3_HUMAN|MSANTD3|C9orf30|L8
0014707 // br	0005634 // nu	0003714 // tr	Musculin	MUSC_HUMAN|MSC|ABF1|BHLHA22
0006298 // m	0032302 // M	0032142 // si	DNA mismatc	MSH3_HUMAN|MSH3|DUC1|DUG
0006351 // tr	0035267 // Nu		Putative male	MS3L2_HUMAN|MSL3P1|MSL3L2
0007155 // ce	0009986 // ce	0005515 // pr	Mesothelin	MSLN_HUMAN|MSLN|MPF
0016126 // st	0016021 // in	0000254 // C-	Methylsterol	MSMO1_HUMAN|MSMO1|DESP4|ERG25|SC4MOL
0071394 // ce	0005829 // cy	0005515 // pr	Moesin	MOES_HUMAN|MSN
0042953 // lip	0005829 // cy	0005515 // pr	Macrophage s	MSRE_HUMAN|MSR1|SCARA1
0006555 // m	0016020 // m	0008113 // pe	Mitochondria	MSRA_HUMAN|MSRA
0030041 // ac	0005829 // cy	0003779 // ac	Methionine-R	MSRB1_HUMAN|MSRB1|SEPX1|HSPC270
0006979 // re	0005829 // cy	0003700 // tr	Methionine-R	MSRB2_HUMAN|MSRB2|CBS-1|MSRB|CGI-131
0006979 // re	0005739 // m	0005515 // pr	Methionine-R	MSRB3_HUMAN|MSRB3|UNQ1965/PRO4487
0009615 // re	0005886 // pl	0005515 // pr	Macrophage-s	RON_HUMAN|MST1R|PTK8|RON
0046716 // m	0005737 // cy	0008083 // gr	Growth/differ	GDF8_HUMAN|MSTN|GDF8
0007517 // m	0005634 // nu	0000982 // tr	Homeobox pr	MSX1_HUMAN|MSX1|HOX7
0008285 // ne	0005829 // cy	0001227 // tr	Homeobox pr	MSX2_HUMAN|MSX2|HOX8
0006754 // AT	0005743 // m	0015078 // hy	ATP synthase	ATP6_HUMAN|MT-ATP6|ATP6|ATPASE6|MTATP6
0006754 // AT	0005743 // m	0016887 // AT	ATP synthase	ATP8_HUMAN|MT-ATP8|ATP8|ATPASE8|MTATP8
0006123 // m	0005751 // m	0020037 // he	Cytochrome c	COX1_HUMAN|MT-CO1|COI|COXI|MTCO1
0006123 // m	0016021 // in	0005507 // co	Cytochrome c	COX2_HUMAN|MT-CO2|COII|COXII|MTCO2
0006123 // m	0016021 // in	0004129 // cy	Cytochrome c	COX3_HUMAN|MT-CO3|COIII|COXIII|MTCO3
0006122 // m	0005739 // m	0046872 // m	Cytochrome b	CYB_HUMAN|MT-CYB|COB|CYTB|MTCYB
0045333 // ce	0005743 // m	0008137 // N	NADH-ubiquir	NU1M_HUMAN|MT-ND1|MTND1|NADH1|ND1
0006120 // m	0005743 // m	0008137 // N	NADH-ubiquir	NU2M_HUMAN|MT-ND2|MTND2|NADH2|ND2
0055114 // ox	0005743 // m	0005515 // pr	NADH-ubiquir	NU3M_HUMAN|MT-ND3|MTND3|NADH3|ND3
0006120 // m	0005743 // m	0008137 // N	NADH-ubiquir	NU4M_HUMAN|MT-ND4|MTND4|NADH4|ND4

0006120 // m	0005747 // m	0008137 // N/	NADH-ubiquir	NU4LM_HUMAN|MT-ND4L|MTND4L|NADH4L|ND4L
0006120 // m	0005743 // m	0008137 // N/	NADH-ubiquir	NU5M_HUMAN|MT-ND5|MTND5|NADH5|ND5
0006120 // m	0005739 // m	0008137 // N/	NADH-ubiquir	NU6M_HUMAN|MT-ND6|MTND6|NADH6|ND6
0032147 // ac	0005615 // ex		Mitochondria	MOTSC_HUMAN|MT-RNR1
0006879 // ce	0005576 // ex	0005102 // re	Humanin	HUNIN_HUMAN|MT-RNR2|HN
0071294 // ce	0005737 // cy	0008270 // zir	Metallothione	MT1B_HUMAN|MT1B|MT1Q
0071294 // ce	0005737 // cy	0008270 // zir	Metallothione	MT1E_HUMAN|MT1E
0045926 // ne	0005737 // cy	0008270 // zir	Metallothione	MT1F_HUMAN|MT1F|PRO0376
0071294 // ce	0005737 // cy	0008270 // zir	Metallothione	MT1L_HUMAN|MT1L
0071294 // ce	0005737 // cy	0008270 // zir	Metallothione	MT1X_HUMAN|MT1X
0010038 // re	0005737 // cy	0008144 // dr	Metallothione	MT2_HUMAN|MT2A|CES1|MT2
0043161 // pr	0005634 // nu	0005515 // pr	Metastasis-as	MTA1_HUMAN|MTA1
0000122 // ne	0000118 // hi	0000989 // tra	Metastasis-as	MTA2_HUMAN|MTA2|MTA1L1|PID
0007050 // ce	0000776 // kir		Mdm2-bindin	MTBP_HUMAN|MTBP
0001578 // m	0016328 // lat	0003723 // RN	Microtubule c	MTCL1_HUMAN|MTCL1|CCDC165|KIAA0802|SOGA2
0008283 // ce	0043234 // pr	0043539 // pr	Protein p13 M	MTCP1_HUMAN|MTCP1|C6.1B
0031663 // lip	0005783 // er	0003723 // RN	Protein LYRIC	LYRIC_HUMAN|MTDH|AEG1|LYRIC
0006393 // te	0005759 // m	0005515 // pr	Transcription	MTEF1_HUMAN|MTERF1|MTERF
0000266 // m	0016021 // int		Mitochondria	MTFP1_HUMAN|MTFP1|MTP18|HSPC242|My022
0042254 // rib	0005743 // m	0003924 // GT	Mitochondria	MTG1_HUMAN|MTG1|GTPBP7
0070129 // re	0005743 // m	0003924 // GT	Mitochondria	MTG2_HUMAN|MTG2|GTPBP5|OBGH1
0006555 // m	0005829 // cy	0004477 // m	C-1-tetrahydro	C1TC_HUMAN|MTHFD1|MTHFC|MTHFD
0033274 // re	0005829 // cy	0004489 // m	Methylenetet	MTHR_HUMAN|MTHFR
0035999 // te	0005829 // cy	0030272 // 5-	5-formyltetral	MTHFS_HUMAN|MTHFS
0008298 // int	0005737 // cy	0003723 // RN	Methenyltetra	MTHSD_HUMAN|MTHFSD
	0070062 // ex		Myotubularin	MTMRB_HUMAN|MTMR11
0035335 // pe	0005829 // cy	0052629 // ph	Myotubularin	MTMR4_HUMAN|MTMR4|KIAA0647|ZFYVE11
0006415 // tra	0005739 // m	0003747 // tra	Peptide chain	RF1M_HUMAN|MTRF1
0070126 // m	0005759 // m	0003747 // tra	Peptide chain	RF1ML_HUMAN|MTRF1L|MTRF1A
	0005576 // ex		Humanin-like	HMN12_HUMAN|MTRNR2L12
0050680 // ne	0005737 // cy	0005515 // pr	Metastasis su	MTSS1_HUMAN|MTSS1|KIAA0429|MIM
0097581 // lar	0030027 // lar	0005546 // ph	MTSS1-like pr	MTSSL_HUMAN|MTSS1L
0034378 // ch	0005783 // er	0005319 // lip	Microsomal tr	MTP_HUMAN|MTTP|MTP
0007275 // m			Maturin	MTURN_HUMAN|MTURN|C7orf41
	0005730 // nu		Microtubule-a	MTUS1_HUMAN|MTUS1|ATBP|ATIP|GK1|KIAA1288|MT
0002223 // sti	0000790 // nu	0005515 // pr	Mucin-1	MUC1_HUMAN|MUC1|PUM
0002223 // sti	0005796 // Go	0003674 // m	Mucin-12	MUC12_HUMAN|MUC12|MUC11
0002223 // sti	0005796 // Go	0005515 // pr	Mucin-16	MUC16_HUMAN|MUC16|CA125
0002223 // sti	0005796 // Go		Mucin-19	MUC19_HUMAN|MUC19
0000187 // ac	0005796 // Go		Mucin-20	MUC20_HUMAN|MUC20|KIAA1359|UNQ2782/PRO717(

0002223 // sti	0005796 // Go	0030197 // ex	Mucin-3A	MUC3A_HUMAN|MUC3A|MUC3
0002223 // sti	0005796 // Go	0005176 // Er	Mucin-4	MUC4_HUMAN|MUC4
0002223 // sti	0005796 // Go	0005201 // ex	Mucin-5AC	MUC5A_HUMAN|MUC5AC|MUC5
0002223 // sti	0005796 // Go	0005201 // ex	Mucin-6	MUC6_HUMAN|MUC6
0006325 // ch	0005634 // nu	0031491 // nu	PWWP domai	MUM1_HUMAN|MUM1|EXPAND1
	0070062 // ex		PWWP domai	MUML1_HUMAN|MUM1L1
0019626 // sh	0005739 // m	0072341 // m	Methylmalony	MUTA_HUMAN|MUT
0016197 // er	0005794 // Go	0043130 // ub	Multivesicular	MB12A_HUMAN|MVB12A|CFBP|FAM125A
0016197 // er	0010008 // er	0005515 // pr	Multivesicular	MB12B_HUMAN|MVB12B|C9orf28|FAM125B
0008299 // iso	0005829 // cy	0004163 // di	Diphosphome	MVD1_HUMAN|MVD|MPD
0008299 // iso	0005829 // cy	0005515 // pr	Mevalonate k	KIME_HUMAN|MVK
0009615 // re	0005829 // cy	0005515 // pr	Interferon-inc	MX1_HUMAN|MX1
0046822 // re	0005829 // cy	0003924 // GT	Interferon-inc	MX2_HUMAN|MX2
0007275 // m	0005739 // m	0003700 // tra	Max dimerizat	MAD1_HUMAN|MXD1|MAD
0006351 // tra	0090575 // RN	0005515 // pr	Max dimerizat	MAD3_HUMAN|MXD3|BHLHC13|MAD3
	0031012 // ex		Matrix-remod	MXRA7_HUMAN|MXRA7|TMAP1
0060857 // es	0016021 // int	0003674 // m	Matrix remod	MXRA8_HUMAN|MXRA8
0031579 // m	0030864 // co	0003674 // m	Myeloid-assoc	MYADM_HUMAN|MYADM|UNQ553/PRO1110
	0016021 // int		Myeloid-assoc	MADL2_HUMAN|MYADML2
0045893 // po	0016363 // nu	0005515 // pr	Transcriptiona	MYB_HUMAN|MYB
0043525 // po	0005654 // nu	0005515 // pr	Myb-related p	MYBB_HUMAN|MYBL2|BMYB
0007275 // m	0016020 // m	0005515 // pr	MYCBP-associ	MYBPP_HUMAN|MYCBPAP|AMAP1
0045607 // re	0005634 // nu	0003700 // tra	Protein L-Myc	MYCL_HUMAN|MYCL|BHLHE38|LMYC|MYCL1
0030182 // ne	0005634 // nu	0003723 // RN	Myelin expres	MYEF2_HUMAN|MYEF2|KIAA1341
0030241 // sk	0005859 // m	0003774 // m	Myosin-11	MYH11_HUMAN|MYH11|KIAA0866
0007519 // sk	0097513 // m	0051015 // ac	Myosin-14	MYH14_HUMAN|MYH14|KIAA2034|FP17425
	0016459 // m	0003774 // m	Putative unch	MYH16_HUMAN|MYH16|MYH5
	0016020 // m	0003774 // m	Myosin-7B	MYH7B_HUMAN|MYH7B|KIAA1512
0006936 // m	0016461 // un	0005515 // pr	Myosin light c	MYL6B_HUMAN|MYL6B|MLC1SA
0006937 // re	0005859 // m	0008307 // str	Myosin regula	MYL9_HUMAN|MYL9|MLC2|MRLC1|MYRL2
0042632 // ch	0005829 // cy	0004842 // ub	E3 ubiquitin-p	MYLIP_HUMAN|MYLIP|BZF1|IDOL|BM-023|PP5242
0090303 // po	0005829 // cy	0005524 // AT	Myosin light c	MYLK_HUMAN|MYLK|MLCK|MLCK1|MYLK1
0018105 // pe	0005634 // nu	0004687 // m	Myosin light c	MYLK2_HUMAN|MYLK2
0045214 // sa	0005829 // cy	0004687 // m	Myosin light c	MYLK3_HUMAN|MYLK3|MLCK
0018107 // pe	0070062 // ex	0004687 // m	Myosin light c	MYLK4_HUMAN|MYLK4|SGK085
0006351 // tra	0005634 // nu	0003677 // DN	Myoneurin	MYNN_HUMAN|MYNN|OSZF|ZBTB31|SBBIZ1
0042472 // in	0005737 // cy	0003774 // m	Unconvention	MYO15_HUMAN|MYO15A|MYO15
0008150 // bi	0016459 // m	0003774 // m	Unconvention	MY15B_HUMAN|MYO15B|KIAA1783|MYO15BP
0048194 // Go	0000139 // Go	0005515 // pr	Unconvention	MY18A_HUMAN|MYO18A|CD245|KIAA0216|MYSPDZ
0010923 // ne	0030673 // ax	0005516 // ca	Unconvention	MYO1D_HUMAN|MYO1D|KIAA0727
0008150 // bi	0016461 // un	0003779 // ac	Unconvention	MYO1F_HUMAN|MYO1F
0071976 // ce	0001891 // ph	0005547 // ph	Unconvention	MYO1G_HUMAN|MYO1G|HA2
0006810 // tra	0005882 // int	0017137 // Ra	Unconvention	MYO5A_HUMAN|MYO5A|MYH12
0032439 // en	0016459 // m	0005515 // pr	Unconvention	MYO5B_HUMAN|MYO5B|KIAA1119

	0016459 // m	0003774 // m	Unconvention	MYO5C_HUMAN|MYO5C
0048563 // pc	0005938 // ce	0000146 // m	Unconvention	MYO7A_HUMAN|MYO7A|USH1B
1904970 // br	0016459 // m	0005515 // pr	Unconvention	MYO7B_HUMAN|MYO7B
0045214 // sa	0031430 // M	0097493 // st	Myomesin-1	MYOM1_HUMAN|MYOM1
0006936 // m	0005739 // m	0005515 // pr	Myomesin-2	MYOM2_HUMAN|MYOM2
0045214 // sa	0031430 // M	0042803 // pr	Myomesin-3	MYOM3_HUMAN|MYOM3
0006936 // m	0042383 // sa	0005515 // pr	Myotilin	MYOTI_HUMAN|MYOT|TTID
0030239 // m	0015629 // ac	0003779 // ac	Myozenin-3	MYOZ3_HUMAN|MYOZ3|FRP3
0031643 // pc	0005794 // Gc	0008233 // pe	Myelin regula	MYRF_HUMAN|MYRF|C11orf9|KIAA0954|MRF
0035556 // in	0031234 // ex	0005515 // pr	Myocardial zo	MYZAP_HUMAN|MYZAP|MYOZAP
0045944 // pc	0005634 // nu	0005515 // pr	Myeloid zinc f	MZF1_HUMAN|MZF1|MZF|ZNF42|ZSCAN6
	0005829 // cy	0005515 // pr	Mitotic-spindl	MZT2B_HUMAN|MZT2B|FAM128B|MOZART2B
0032435 // ne	0005730 // nu	0005515 // pr	NEDD4-bindir	N4BP1_HUMAN|N4BP1|KIAA0615
1902035 // pc	0017053 // tra	0005515 // pr	NEDD4-bindir	N42L2_HUMAN|N4BP2L2|CG005|PFAAP5
	0030659 // cy	0005515 // pr	NEDD4-bindir	N4BP3_HUMAN|N4BP3|KIAA0341
0006474 // N-	0005737 // cy	0005515 // pr	N-alpha-acety	NAA10_HUMAN|NAA10|ARD1|ARD1A|TE2
0006474 // N-	0031415 // Na	0005515 // pr	N-alpha-acety	NAA11_HUMAN|NAA11|ARD1B|ARD2
0017196 // N-	0005829 // cy	0005515 // pr	N-alpha-acety	NAA30_HUMAN|NAA30|C14orf35|MAK3|NAT12
0043066 // ne	0031417 // Na	0005515 // pr	N-alpha-acety	LSMD1_HUMAN|NAA38|LSMD1|MAK31|PFAAP2
0043967 // hi	0005634 // nu	0010485 // H4	N-alpha-acety	NAA40_HUMAN|NAA40|NAT11|PATT1
0006629 // lip	0098793 // pr	0016810 // hy	N-acylethanol	NAAA_HUMAN|NAAA|ASAHL|PLT
0006508 // pr	0016021 // in	0008237 // m	N-acetylated-a	NALDL_HUMAN|NAALADL1|NAALADASEL|NAALADL
	0016021 // in	0005515 // pr	Inactive N-ace	NADL2_HUMAN|NAALADL2
0006355 // re	0005634 // nu	0008134 // tra	NGFI-A-bindir	NAB1_HUMAN|NAB1
0008283 // ce	0005634 // nu	0042802 // ide	NGFI-A-bindir	NAB2_HUMAN|NAB2|MADER
0006281 // DI	0000784 // nu	0005515 // pr	SOSS complex	SOSB1_HUMAN|NABP2|OBFC2B|SSB1|LP3587
0015031 // pr	0005634 // nu		NAC-alpha do	NACAD_HUMAN|NACAD|KIAA0363
	0005854 // na		Putative nasce	NACP1_HUMAN|NACAP1|FKSG17
0051260 // pr	0016604 // nu	0042826 // his	Nucleus accur	NACC2_HUMAN|NACC2|BTBD14A|NAC2|RBB
0006741 // NA	0005759 // m	0003951 // NA	NAD kinase 2,	NAKD2_HUMAN|NADK2|C5orf33|MNADK|NADKD1
0019674 // NA	0005829 // cy	0004359 // glu	Glutamine-de	NADE_HUMAN|NADSYN1
0019377 // gly	0070062 // ex	0008456 // alp	Alpha-N-acety	NAGAB_HUMAN|NAGA
0006044 // N-	0070062 // ex	0005515 // pr	N-acetyl-D-glu	NAGK_HUMAN|NAGK
0021680 // ce	0005764 // lys	0004561 // alp	Alpha-N-acety	ANAG_HUMAN|NAGLU|UFHSD1
0007040 // lys	0016021 // in	0003944 // N-	N-acetylgluco	NAGPA_HUMAN|NAGPA
0006816 // ca	0005886 // pl	0005515 // pr	Sodium leak c	NALCN_HUMAN|NALCN|VGCNL1
0007165 // sig	0005829 // cy	0004514 // nic	Nicotinamide	NAMPT_HUMAN|NAMPT|PBEF|PBEF1
0098749 // ce	0048471 // pe	0005515 // pr	Nanos homolo	NANO1_HUMAN|NANOS1|NOS1
0006055 // CM	0070062 // ex	0050462 // N-	Sialic acid syn	SIAS_HUMAN|NANS|SAS
0006334 // nu	0042470 // m	0003723 // RN	Nucleosome a	NP1L1_HUMAN|NAP1L1|NRP
2000035 // re	0005634 // nu	0003682 // ch	Nucleosome a	NP1L2_HUMAN|NAP1L2|BPX
0006334 // nu	0005634 // nu		Nucleosome a	NP1L3_HUMAN|NAP1L3|BNAP
0006334 // nu	0005634 // nu		Nucleosome a	NP1L5_HUMAN|NAP1L5|DRLM

0007568 // ag	0042622 // ph	0008270 // zir	N-acyl-phosph	NAPEP_HUMAN|NAPEPLD|C7orf18
0019358 // ni	0005576 // ex	0005515 // pr	Nicotinate ph	PNCB_HUMAN|NAPRT|FHIP|NAPRT1
0030163 // pr	0005615 // ex	0004190 // as	Napsin-A	NAPSA_HUMAN|NAPSA|NAP1|NAPA
0055114 // ox	0005638 // la	0003954 // N/	Nuclear prelar	NARF_HUMAN|NARF
0006421 // as	0070062 // ex	0003676 // nu	Asparagine--tl	SYNC_HUMAN|NARS
0006805 // xe	0005829 // cy	0004060 // ar	Arylamine N-a	ARY1_HUMAN|NAT1|AAC1
0008652 // ce	0005739 // m	0017188 // as	N-acetylaspar	NAT8L_HUMAN|NAT8L|CML3
	0043234 // pr	0005515 // pr	N-acetyltransf	NAT9_HUMAN|NAT9|EBS
		0005515 // pr	Protein NATD	NATD1_HUMAN|NATD1|C17orf103|GTLF3B
0001764 // ne	0005737 // cy		Neuron naviga	NAV1_HUMAN|NAV1|KIAA1151|KIAA1213|POMFIL3|ST
0032703 // ne	0005640 // nu	0005524 // AT	Neuron naviga	NAV3_HUMAN|NAV3|KIAA0938|POMFIL1|STEERIN3
0008104 // pr	0005829 // cy	0019901 // pr	Neurobeachin	NBEA_HUMAN|NBEA|BCL8B|KIAA1544|LYST2
0030220 // pl	0005886 // pl		Neurobeachin	NBEL2_HUMAN|NBEAL2|KIAA0540|UNQ253/PRO290
0055114 // ox	0005737 // cy	0016491 // ox	Neuroblastom	NBPF1_HUMAN|NBPF1|KIAA1693
	0005737 // cy		Neuroblastom	NBPFF_HUMAN|NBPF15|NBPF16
	0005737 // cy		Neuroblastom	NBPFJ_HUMAN|NBPF19
0055114 // ox	0005737 // cy	0016491 // ox	Neuroblastom	NBPFK_HUMAN|NBPF20
	0005737 // cy		Neuroblastom	NBPF6_HUMAN|NBPF6
0055114 // ox	0005737 // cy	0016491 // ox	Putative neur	NBPF8_HUMAN|NBPF8|NBPF8P
	0005737 // cy		Neuroblastom	NBPF9_HUMAN|NBPF9
0008150 // bi	0005575 // ce	0003674 // m	Next to BRCA1	NBR2_HUMAN|NBR2
0016192 // ve	0005829 // cy	0005515 // pr	Neurocalcin-d	NCALD_HUMAN|NCALD
0007158 // ne	0016021 // in		Neural cell ad	NCAM2_HUMAN|NCAM2|NCAM21
0030261 // ch	0005654 // nu	0005515 // pr	Condensin-2 c	CNDH2_HUMAN|NCAPH2|CAPH2
0008284 // po	0070062 // ex		F-box only pro	FBX50_HUMAN|NCCRP1|FBXO50
0002479 // an	0032010 // ph	0005515 // pr	Neutrophil cyt	NCF1_HUMAN|NCF1|NOXO2|SH3PXD1A
0071800 // po	0005737 // cy	0016176 // su	Putative neutr	NCF1B_HUMAN|NCF1B|SH3PXD1B
0002479 // an	0032010 // ph	0005515 // pr	Neutrophil cyt	NCF2_HUMAN|NCF2|NOXA2|P67PHOX
0007173 // ep	0005829 // cy	0005515 // pr	Cytoplasmic p	NCK2_HUMAN|NCK2|GRB4
0008150 // bi	0005575 // ce		Nck-associate	NCKP5_HUMAN|NCKAP5|ERIH|NAP5
0001525 // an	0005654 // nu	0005515 // pr	Nucleolin	NUCL_HUMAN|NCL
0006351 // tra	0005615 // ex	0003723 // RN	Nuclear recep	NCOA5_HUMAN|NCOA5|KIAA1637
0000122 // ne	0005654 // nu	0005515 // pr	Nuclear recep	NCOR1_HUMAN|NCOR1|KIAA1047
0000122 // ne	0017053 // tra	0005515 // pr	Nuclear recep	NCOR2_HUMAN|NCOR2|CTG26
0050776 // re	0016021 // in	0005515 // pr	Natural cytoto	NR3L1_HUMAN|NCR3LG1|B7H6
0043065 // po	0005886 // pl	0005515 // pr	Nicastrin	NICA_HUMAN|NCSTN|KIAA0253|UNQ1874/PRO4317
0000070 // m	0000776 // ki	0005515 // pr	Kinetochore p	NDC80_HUMAN|NDC80|HEC|HEC1|KNTC2
0010629 // ne	0005794 // Go	0050699 // W	NEDD4 family	NFIP2_HUMAN|NDFIP2|KIAA1165|N4WBP5A
0019800 // pe	0031012 // ex	0008201 // he	Protein NDNF	NDNF_HUMAN|NDNF|C4orf31|UNQ2748/PRO6487
0055114 // ox	0005829 // cy	0005515 // pr	NADPH-deper	NDOR1_HUMAN|NDOR1|NR1
0032287 // pe	0015630 // m	0005515 // pr	Protein NDRG	NDRG1_HUMAN|NDRG1|CAP43|DRG1|RTP

0016055 // W	0030426 // gr	0005515 // pr	Protein NDRG	NDRG2_HUMAN|NDRG2|KIAA1248|SYLD	
0070374 // pc	0031253 // ce	0005515 // pr	Protein NDRG	NDRG4_HUMAN|NDRG4|BDM1|KIAA1180	
0030900 // fo	0016021 // in	0050119 // N-	Bifunctional h	NDST1_HUMAN|NDST1|HSST|HSST1	
0006024 // gl	0016021 // in	0050119 // N-	Bifunctional h	NDST2_HUMAN|NDST2|HSST2	
0015012 // he	0016021 // in	0016787 // hy	Bifunctional h	NDST3_HUMAN|NDST3|HSST3|UNQ2544/PRO4998	
0030210 // he	0016021 // in	0019213 // de	Bifunctional h	NDST4_HUMAN|NDST4|HSST4	
0006120 // m	0005743 // m		NADH dehydr	NDUAB_HUMAN|NDUFA11	
0043280 // pc	0005739 // m	0005524 // AT	NADH dehydr	NDUAD_HUMAN|NDUFA13|GRIM19|CDA016|CGI-39	
1902600 // hy	0005751 // m	0004129 // cy	NADH dehydr	NUA4L_HUMAN|NDUFA4L2	
0006979 // re	0005743 // m	0008137 // NA	NADH dehydr	NDUA6_HUMAN|NDUFA6|LYRM6|NADHB14	
0006814 // so	0005747 // m	0005515 // pr	NADH dehydr	NDUA9_HUMAN|NDUFA9|NDUFS2L	
0022904 // re	0005743 // m	0009055 // el	NADH dehydr	NDUF2_HUMAN|NDUFAF2|NDUFA12L	
0032981 // m	0005743 // m	0016740 // tra	NADH dehydr	NDUF6_HUMAN|NDUFAF6|C8orf38	
0032981 // m	0005739 // m	0005515 // pr	NADH dehydr	NDUF8_HUMAN|NDUFAF8|C17orf89	
0006120 // m	0005743 // m	0008137 // NA	NADH dehydr	NDUB1_HUMAN|NDUFB1	
0006120 // m	0005743 // m	0005515 // pr	NADH dehydr	NDUBA_HUMAN|NDUFB10	
0006120 // m	0005743 // m	0008137 // NA	NADH dehydr	NDUB2_HUMAN|NDUFB2	
0006120 // m	0005743 // m	0008137 // NA	NADH dehydr	NDUB5_HUMAN|NDUFB5	
0006120 // m	0005747 // m	0008137 // NA	NADH dehydr	NDUB6_HUMAN|NDUFB6	
0007605 // se	0005743 // m	0005515 // pr	NADH dehydr	NDUB9_HUMAN|NDUFB9|LYRM3|UQOR22	
0006120 // m	0005739 // m	0008137 // NA	NADH dehydr	NDUCR_HUMAN|NDUFC2-KCTD14	
0006979 // re	0005747 // m	0005515 // pr	NADH dehydr	NDUS2_HUMAN|NDUFS2	
0032981 // m	0005743 // m	0008137 // NA	NADH dehydr	NDUS4_HUMAN|NDUFS4	
0032981 // m	0005743 // m	0008137 // NA	NADH dehydr	NDUS5_HUMAN|NDUFS5	
0006120 // m	0005743 // m	0010181 // FN	NADH dehydr	NDUV1_HUMAN|NDUFV1|UQOR1	
0030049 // m	0005829 // cy	0005515 // pr	Nebulin	NEBU_HUMAN|NEB	
0071691 // ca	0030018 // Z	0051015 // ac	Nebulette	NEBL_HUMAN|NEBL|LNEBL	
0008150 // bi	0005737 // cy	0003674 // m	N-terminal EF	NECA1_HUMAN|NECAB1|EFCBP1	
1900451 // pc	0030424 // ax	0005515 // pr	N-terminal EF	NECA2_HUMAN|NECAB2|EFCBP2	
0006897 // er	0005829 // cy		Adaptin ear-b	NECP1_HUMAN|NECAP1	
0007157 // he	0016021 // in	0015026 // co	Nectin-1	NECT1_HUMAN|NECTIN1|HVEC|PRR1|PVRL1	
0002891 // pc	0005886 // pl	0001618 // vi	Nectin-2	NECT2_HUMAN|NECTIN2|HVEB|PRR2|PVRL2	
0007156 // hc	0005886 // pl	0050839 // ce	Nectin-4	NECT4_HUMAN|NECTIN4|LNIR|PRR4|PVRL4	
0042787 // pr	0005829 // cy	0005515 // pr	E3 ubiquitin-p	NEDD4_HUMAN|NEDD4|KIAA0093|NEDD4-1|PIG53	
1902306 // ne	0005829 // cy	0044325 // io	E3 ubiquitin-p	NED4L_HUMAN|NEDD4L|KIAA0439|NEDL3	
0006879 // ce	0005829 // cy	0005515 // pr	NEDD8	NEDD8_HUMAN|NEDD8	
0016311 // de		0016791 // ph	NEDD8-MDP1	E9PL57_HUMAN|NEDD8-MDP1	
0007155 // ce	0005938 // ce	0005515 // pr	Enhancer of fi	CASL_HUMAN|NEDD9|CASL|CASS2	
0048936 // pe	0005883 // ne	0019901 // pr	Neurofilamen	NFH_HUMAN|NEFH|KIAA0845|NFH	
0045109 // in	0030424 // ax	0030674 // pr	Neurofilamen	NFL_HUMAN|NEFL|NF68|NFL	
0007155 // ce	0031225 // ar	0005515 // pr	Neuronal grov	NEGR1_HUMAN|NEGR1|IGLON4|UNQ2433/PRO4993	

0032074 // ne	0005654 // nu	0140080 /// 0	Endonuclease	NEIL1_HUMAN|NEIL1
0023014 // sig	0000242 // pe	0005515 // pr	Serine/threon	NEK1_HUMAN|NEK1|KIAA1901
0043406 // po	1902911 // pr	0004674 // pr	Serine/threon	NEK10_HUMAN|NEK10
0051299 // ce	0005829 // cy	0004672 // pr	Serine/threon	NEK2_HUMAN|NEK2|NEK2A|NLK1
0090043 // re	0005634 // nu	0004674 // pr	Serine/threon	NEK3_HUMAN|NEK3
0023014 // sig	0005737 // cy	0005515 // pr	Serine/threon	NEK4_HUMAN|NEK4|STK2
0006468 // pr	0097546 // cil	0005515 // pr	Serine/threon	NEK8_HUMAN|NEK8|JCK|NEK12A
0070050 // ne	0005576 // ex	0005509 // ca	Protein kinase	NELL2_HUMAN|NELL2|NRP2
0051168 // nu	0005634 // nu		Nuclear expor	NEMF_HUMAN|NEMF|SDCCAG1
0008150 // bi	0016021 // in	0003674 // m	Nuclear envel	NEMP1_HUMAN|NEMP1|KIAA0286|TMEM194|TMEM1
	0016021 // in		Nuclear envel	NEMP2_HUMAN|NEMP2|TMEM194B
0055072 // iro	0005794 // Go	0005515 // pr	Neogenin	NEO1_HUMAN|NEO1|IGDCC2|NGN
0007420 // br	0045111 // in	0019215 // in	Nestin	NEST_HUMAN|NES|Nbla00170
0001558 // re	0005829 // cy	0005089 // Rh	Neuroepitheli	ARHG8_HUMAN|NET1|ARHGEF8
0007613 // m	0016021 // in	0035255 // io	Neuropilin an	NETO1_HUMAN|NETO1|BTCL1
2000312 // re	0016021 // in	0035255 // io	Neuropilin an	NETO2_HUMAN|NETO2|BTCL2|UNQ1926/PRO4401
0007219 // No	0005886 // pl	0046872 // m	E3 ubiquitin-p	NEUL1_HUMAN|NEURL1|NEURL|NEURL1A|RNF67
0007219 // No	0005769 // ea	0005515 // pr	E3 ubiquitin-p	NEU1B_HUMAN|NEURL1B|NEURL3
	0005814 // ce	0005515 // pr	Neuralized-lik	NEUL4_HUMAN|NEURL4|KIAA1787
0048739 // ca	0030018 // Z	0051015 // ac	Nexilin	NEXN_HUMAN|NEXN
0008542 // vis	0005634 // nu	0005515 // pr	Neurofibromi	NF1_HUMAN|NF1
0045597 // po	0005634 // nu	0005515 // pr	Merlin	MERL_HUMAN|NF2|SCH
0007165 // sig	0016021 // in	0004888 // tra	NFAT activatio	NFAM1_HUMAN|NFAM1|CNAIP
0071205 // pr	0005886 // pl	0005515 // pr	Neurofascin	NFASC_HUMAN|NFASC|KIAA0756
0033173 // ca	0005654 // nu	0003705 // tra	Nuclear facto	NFAC1_HUMAN|NFATC1|NFAT2|NFATC
0000122 // ne	0005634 // nu	0005515 // pr	Nuclear facto	NFAC2_HUMAN|NFATC2|NFAT1|NFATP
0045944 // po	0005634 // nu	0005515 // pr	Nuclear facto	NFAC3_HUMAN|NFATC3|NFAT4
1902894 // ne	0005634 // nu	0001078 // tra	Nuclear facto	NFAC4_HUMAN|NFATC4|NFAT3
0034599 // ce	0005794 // Go	0005515 // pr	Nuclear facto	NF2L2_HUMAN|NFE2L2|NRF2
0006357 // re	0005634 // nu	0005515 // pr	Nuclear facto	NF2L3_HUMAN|NFE2L3|NRF3
0006351 // tra	0005634 // nu		Transcription	NFE4_HUMAN|NFE4
0071679 // co	0044300 // ce	0000978 // RN	Nuclear facto	NFIB_HUMAN|NFIB
0006366 // tra	0001650 // fib	0000978 // RN	Nuclear facto	NFIC_HUMAN|NFIC|NFI
0045944 // po	0005634 // nu	0003700 // tra	Nuclear facto	NFIX_HUMAN|NFIX
0045944 // po	0005829 // cy	0005515 // pr	Nuclear facto	NFKB1_HUMAN|NFKB1
0070417 // ce	0005622 // in	0005515 // pr	NF-kappa-B in	IKBA_HUMAN|NFKBIA|IKBA|MAD3|NFKBI
0006351 // tra	0005829 // cy	0004871 // sig	NF-kappa-B in	IKBB_HUMAN|NFKBIB|IKBB|TRIP9
0042942 // D-	0005829 // cy	0005515 // pr	NF-kappa-B in	IKBE_HUMAN|NFKBIE|IKBE
0006357 // re	0005634 // nu	0005515 // pr	NF-kappa-B in	IKBZ_HUMAN|NFKBIZ|IKBZ|INAP|MAIL
0006954 // in	0005730 // nu	0003723 // RN	Transcriptiona	NFX1_HUMAN|NFX1|NFX2
0006351 // tra	0005634 // nu	0003677 // DN	Nuclear transc	NFYB_HUMAN|NFYB|HAP3
0006417 // re	0030175 // fil	0003723 // RN	Neuroguidin	NGDN_HUMAN|NGDN|C14orf120
0048013 // ep	0016020 // m	0005089 // Rh	Ephexin-1	NGEF_HUMAN|NGEF
1900182 // po	0005886 // pl	0005515 // pr	Tumor necros	TNR16_HUMAN|NGFR|TNFRSF16

0051351 // pc	0005634 // nu	0005515 // pr	Non-homolog	NHEJ1_HUMAN|NHEJ1|XLF
0051091 // pc	0005667 // tra	0000978 // RN	Helix-loop-hel	HEN2_HUMAN|NHLH2|BHLHA34|HEN2|KIAA0490
0005978 // gl	0048471 // pe	0004842 // ub	E3 ubiquitin-p	NHLC1_HUMAN|NHLRC1|EPM2B
0043312 // ne	0005576 // ex		NHL repeat-co	NHLC3_HUMAN|NHLRC3
0007004 // te	0000784 // nu	0003723 // RN	H/ACA ribonu	NHP2_HUMAN|NHP2|NOLA2|HSPC286
0030154 // ce	0005794 // Go		Nance-Horan	NHS_HUMAN|NHS
0010811 // pc	0031012 // ex	0043237 // lai	Nidogen-1	NID1_HUMAN|NID1|NID
0007160 // ce	0031012 // ex	0005509 // ca	Nidogen-2	NID2_HUMAN|NID2
0010923 // ne	0005730 // nu	0003723 // RN	MKI67 FHA dc	MK67I_HUMAN|NIFK|MKI67IP|NOPP34
0007155 // ce	0016021 // int	0005515 // pr	Ninjurin-1	NINJ1_HUMAN|NINJ1
0042246 // tis	0005887 // int	0005515 // pr	Ninjurin-2	NINJ2_HUMAN|NINJ2
1903830 // m	0016021 // int	0015095 // m	NIPA-like prot	NPAL2_HUMAN|NIPAL2|NPAL2
1903830 // m	0016021 // int	0005515 // pr	NIPA-like prot	NPAL3_HUMAN|NIPAL3|NPAL3
1903830 // m	0016021 // int	0015095 // m	Magnesium tr	NIPA4_HUMAN|NIPAL4|ICHN|NIPA4
0061038 // ut	0005634 // nu	0005515 // pr	Nipped-B-like	NIPBL_HUMAN|NIPBL|IDN3
0008150 // bi	0070062 // ex	0000257 // nit	Nitrilase hom	NIT1_HUMAN|NIT1
0006107 // ox	0005739 // m	0050152 // or	Omega-amida	NIT2_HUMAN|NIT2|CUA002
0002028 // re	0016021 // int		Sodium/potas	NKAI2_HUMAN|NKAIN2|FAM77B|TCBA1
0002028 // re	0016021 // int		Sodium/potas	NKAI4_HUMAN|NKAIN4|C20orf58|FAM77A
0016055 // W	0031410 // cy	0005515 // pr	Protein naked	NKD2_HUMAN|NKD2
	0016021 // int		NTPase KAP fa	NKPD1_HUMAN|NKPD1
0006457 // pr	0005739 // m	0016018 // cy	NK-tumor rec	NKTR_HUMAN|NKTR
0007275 // m	0005634 // nu	0043565 // se	NK1 transcrip	NKX12_HUMAN|NKX1-2|C10orf121|NKX1.1
0048646 // ar	0005634 // nu	0001012 // RN	Homeobox pr	NKX21_HUMAN|NKX2-1|NKX2A|TITF1|TTF1
0010765 // pc	0005667 // tra	0010736 // se	Homeobox pr	NKX25_HUMAN|NKX2-5|CSX|NKX2.5|NKX2E
0050680 // ne	0005634 // nu	0003690 // dc	Homeobox pr	NKX28_HUMAN|NKX2-8|NKX-2.8|NKX2G|NKX2H
0072560 // ty	0070062 // ex	0000978 // RN	Homeobox pr	NKX61_HUMAN|NKX6-1|NKX6A
0090263 // pc	0005730 // nu		Notchless pro	NLE1_HUMAN|NLE1|HUSSY-07
0097105 // pr	0030054 // ce	0042043 // ne	Neuroligin-1	NLGN1_HUMAN|NLGN1|KIAA1070
0097105 // pr	0030054 // ce	0042043 // ne	Neuroligin-2	NLGN2_HUMAN|NLGN2|KIAA1366
0097105 // pr	0009986 // ce	0004872 // re	Neuroligin-3	NLGN3_HUMAN|NLGN3|KIAA1480|NL3
0021549 // ce	0014069 // pc	0052689 // ca	Neuroligin-4,	NLGNX_HUMAN|NLGN4X|KIAA1260|NLGN4|UNQ365/P
0006508 // pr	0005576 // ex	0042277 // pe	Neurolysin, m	NEUL_HUMAN|NLN|AGTBP|KIAA1226
0042110 // T	0005829 // cy	0005515 // pr	NLR family CA	NLRC3_HUMAN|NLRC3|NOD3
0043549 // re	0005737 // cy	0005515 // pr	Protein NLRC	NLRC5_HUMAN|NLRC5|NOD27|NOD4
0032495 // re	0005634 // nu	0005515 // pr	NACHT, LRR a	NLRP1_HUMAN|NLRP1|CARD7|DEFCAP|KIAA0926|NAC
0007275 // m		0005524 // AT	NACHT, LRR a	NAL14_HUMAN|NLRP14|NALP14|NOD5
0045087 // in	0005737 // cy	0005515 // pr	NACHT, LRR a	NALP2_HUMAN|NLRP2|NALP2|NBS1|PAN1|PYPAF2
0042060 // w	0005886 // pl	0005000 // va	NACHT, LRR a	NLRP6_HUMAN|NLRP6|NALP6|PYPAF5
0008284 // pc	0005576 // ex	0031710 // ne	Neuromedin-I	NMB_HUMAN|NMB
0006259 // DI	0005737 // cy	0005515 // pr	Nucleoside di	NDKA_HUMAN|NME1|NDPKA|NM23
0009116 // nu	0005739 // m	0005515 // pr	Nucleoside di	NDKM_HUMAN|NME4|NM23D
0009116 // nu	0005622 // int	0005515 // pr	Nucleoside di	NDK5_HUMAN|NME5

0045839 // ne	0005829 // cy	0004550 // nu	Nucleoside di	NDK6_HUMAN|NME6
0006954 // in	0005737 // cy	0005515 // pr	N-myc-interac	NMI_HUMAN|NMI
0019674 // N/	0045202 // sy	0000309 // ni	Nicotinamide/	NMNA2_HUMAN|NMNAT2|C1orf15|KIAA0479
0019674 // N/	0005739 // m	0000309 // ni	Nicotinamide/	NMNA3_HUMAN|NMNAT3|FKSG76
0019674 // N/	0005829 // cy	0005515 // pr	Nicotinamide	NRK1_HUMAN|NMRK1|C9orf95|NRK1
0018008 // N-	0019898 // ex	0004379 // gl	Glycylpeptide	NMT2_HUMAN|NMT2
0007165 // sig	0005576 // ex	0005515 // pr	Neuromedin-U	NMU_HUMAN|NMU
0030182 // ne	0005737 // cy		Neuronatin	NNAT_HUMAN|NNAT
0042493 // re	0005829 // cy	0030760 // py	Nicotinamide	NNMT_HUMAN|NNMT
0055114 // ox	0005743 // m	0051287 // N/	NAD(P) transh	NNTM_HUMAN|NNT
0042273 // rik	0005654 // nu	0003723 // RN	Nucleolar com	NOC2L_HUMAN|NOC2L|NIR
0045444 // fa	0005634 // nu	0003723 // RN	Nucleolar com	NOC3L_HUMAN|NOC3L|AD24|C10orf117|FAD24
0042752 // re	0048471 // pe	0003700 // tra	Nocturnin	NOCT_HUMAN|NOCT|CCR4|CCRN4L|NOC
0046330 // po	0005829 // cy	0005515 // pr	Nucleotide-bi	NOD1_HUMAN|NOD1|CARD4
0045747 // po	0005794 // Go	0005515 // pr	Nucleotide-bi	NOD2_HUMAN|NOD2|CARD15|IBD1
0010470 // re	0005615 // ex	0008083 // gr	Nodal homolo	NODAL_HUMAN|NODAL
0061037 // ne	0005615 // ex	0042803 // pr	Noggin	NOGG_HUMAN|NOG
0006364 // rR	0005654 // nu	0003723 // RN	Nucleolar prot	NOL11_HUMAN|NOL11|L14
0010667 // ne	0016020 // m	0005509 // ca	Nucleolar prot	NOL3_HUMAN|NOL3|ARC|NOP
	0005730 // nu	0003723 // RN	Nucleolar prot	NOL4_HUMAN|NOL4|NOLP|HRIHFB2255
	0005829 // cy	0005515 // pr	Nucleolar prot	NOL4L_HUMAN|NOL4L|C20orf112|C20orf113
0006364 // rR	0005730 // nu	0003723 // RN	Nucleolar prot	NOL6_HUMAN|NOL6
	0005739 // m	0003723 // RN	Nucleolar prot	NOL7_HUMAN|NOL7|C6orf90|NOP27
0006364 // rR	0005654 // nu	0005515 // pr	Polynucleotid	NOL9_HUMAN|NOL9
0014032 // ne	0005730 // nu	0003723 // RN	Nucleolar and	NOLC1_HUMAN|NOLC1|KIAA0035|NS5ATP13
0008150 // bi	0005730 // nu	0003723 // RN	Nucleolar MIF	NOM1_HUMAN|NOM1|C7orf3
0031648 // pr	0016021 // in	0005515 // pr	Nodal modula	NOMO2_HUMAN|NOMO2
0008150 // bi	0016021 // in	0003674 // m	Nodal modula	NOMO3_HUMAN|NOMO3
0006397 // m	0005730 // nu	0003723 // RN	Non-POU dom	NONO_HUMAN|NONO|NRB54
0007004 // te	0005697 // te	0003723 // RN	H/ACA ribonu	NOP10_HUMAN|NOP10|NOLA3
0042273 // rik	0005730 // nu	0003723 // RN	Nucleolar prot	NOP16_HUMAN|NOP16|CGI-117|HSPC111
0070475 // rR	0005654 // nu	0003723 // RN	Probable 28S	NOP2_HUMAN|NOP2|NOL1|NSUN1
0006364 // rR	0005654 // nu	0003723 // RN	Nucleolar prot	NOP56_HUMAN|NOP56|NOL5A
0006364 // rR	0015030 // Ca	0003723 // RN	Nucleolar prot	NOP58_HUMAN|NOP58|NOL5|NOP5|HSPC120
0008150 // bi	0005575 // ce	0003723 // RN	Nucleolar prot	NOP9_HUMAN|NOP9|C14orf21|KIAA2021
0010628 // po	0005634 // nu	0050998 // ni	Carboxyl-term	CAPON_HUMAN|NOS1AP|CAPON|KIAA0464
0003100 // re	0005829 // cy	0004517 // ni	Nitric oxide sy	NOS3_HUMAN|NOS3
0007275 // m	0005737 // cy	0003723 // RN	Nitric oxide sy	NOSIP_HUMAN|NOSIP|CGI-25
0006897 // en	0030666 // en	0005515 // pr	Nostrin	NOSTN_HUMAN|NOSTRIN
0006367 // tra	0005576 // ex	0004872 // re	Neurogenic lo	NOTC2_HUMAN|NOTCH2
0048661 // po	0043235 // re	0005515 // pr	Neurogenic lo	NOTC3_HUMAN|NOTCH3
0007267 // ce	0005921 // ga	0005178 // in	Protein NOV h	NOV_HUMAN|NOV|CCN3|IGFBP9|NOVH
0000398 // m	0005730 // nu	0003729 // m	RNA-binding p	NOVA1_HUMAN|NOVA1

0001659 // te	0005737 // cy	0016175 // su	NADPH oxidas	NOX3_HUMAN|NOX3|MOX2	
0050667 // hc	0016021 // in	0016175 // su	NADPH oxidas	NOX4_HUMAN|NOX4|RENOX	
0010310 // re	0005829 // cy	0005515 // pr	NADPH oxidas	NOXA1_HUMAN|NOXA1|P51NOX	
0006357 // re	0005634 // nu	0003700 // tra	Neuronal PAS	NPAS1_HUMAN|NPAS1|BHLHE11|MOP5|PASD5	
2001020 // re	0005654 // nu	0005515 // pr	Neuronal PAS	NPAS2_HUMAN|NPAS2|BHLHE9|MOP4|PASD4	
0090150 // es	0005576 // ex	0001618 // vi	Niemann-Pick	NPC1_HUMAN|NPC1	
0050776 // re	0016021 // in	0005515 // pr	Neural prolife	NPDC1_HUMAN|NPDC1	
0006508 // pr	0005634 // nu	0030145 // m	Probable amir	PEPL1_HUMAN|NPEPL1|KIAA1974	
0007186 // G-	0005886 // pl	0004930 // G-	Neuropeptide	NPFF2_HUMAN|NPFFR2|GPR74|NPFF2|NPGPR	
0051028 // m	0005643 // nu		Nuclear pore	NPIA1_HUMAN|NPIPA1|NPIP	
	0005654 // nu		Nuclear pore	NPIA2_HUMAN|NPIPA2	
	0005654 // nu		Nuclear pore	NPIA5_HUMAN|NPIPA5	
	0005654 // nu		Nuclear pore	NPIA8_HUMAN|NPIPA8	
	0016021 // in		Nuclear pore	NPB13_HUMAN|NPIPB13	
	0005576 // ex		Nuclear pore	NPB15_HUMAN|NPIPB15|NPIPL2	
	0016021 // in		Nuclear pore	NPIB4_HUMAN|NPIPB4	
	0005654 // nu		Nuclear pore	NPIB6_HUMAN|NPIPB6	
0019262 // N-	0005829 // cy	0008747 // N-	N-acetylneura	NPL_HUMAN|NPL|C1orf13	
0000055 // rik	0005829 // cy	0001105 // RN	Nucleophosm	NPM_HUMAN|NPM1|NPM	
0051260 // pr	0000789 // cy	0005515 // pr	Nucleoplasmi	NPM2_HUMAN|NPM2	
0033631 // ce	0030485 // sn	0005178 // in	Nephronectin	NPNT_HUMAN|NPNT|EGFL6L|POEM|UNQ295/PRO334	
0035810 // pc	0043234 // pr	0042277 // pe	Atrial natriure	ANPRC_HUMAN|NPR3|ANPRC|C5orf23|NPRC	
0010508 // pc	0005765 // lys	0004672 // pr	GATOR compl	NPRL2_HUMAN|NPRL2|TUSC4	
0001934 // pc	0060077 // in	0005105 // ty	Neuroplastin	NPTN_HUMAN|NPTN|SDFR1|SDR1	
	0016021 // in	0046872 // m	Neuronal pen	NPTXR_HUMAN|NPTXR	
0007186 // G-	0005576 // ex	0005515 // pr	Neuropeptide	NPW_HUMAN|NPW|PPL8|PPNPW	
0007218 // ne	0005886 // pl	0001601 // pe	Neuropeptide	NPY1R_HUMAN|NPY1R|NPYR|NPYY1	
0007218 // ne	0005886 // pl	0004930 // G-	Neuropeptide	NPY4R_HUMAN|NPY4R|PPYR1	
0007186 // G-	0016021 // in	0004983 // ne	Putative neur	NPY6R_HUMAN|NPY6R|NPY1RL|Y2B	
0045471 // re	0005737 // cy	0003723 // RN	NAD(P)H dehy	NQO1_HUMAN|NQO1|DIA4|NMOR1	
0055114 // ox	0005829 // cy	1904408 // m	Ribosyldihydr	NQO2_HUMAN|NQO2|NMOR2	
0000122 // ne	0005654 // nu	0005515 // pr	Nuclear recep	NR1D1_HUMAN|NR1D1|EAR1|HREV|THRAL	
0043401 // st	0090575 // RN	0005515 // pr	Oxysterols rec	NR1H3_HUMAN|NR1H3|LXRA	
0043401 // st	0016604 // nu	0003713 // tra	Nuclear recep	NR1I2_HUMAN|NR1I2|PXR	
0007165 // sig	0005737 // cy	0001228 // tra	Nuclear recep	NR1I3_HUMAN|NR1I3|CAR	
0006367 // tra	0070062 // ex	0005515 // pr	Nuclear recep	NR2CA_HUMAN|NR2C2AP|TRA16	
0043401 // st	0005654 // nu	0001077 // tra	Nuclear recep	NR2E1_HUMAN|NR2E1|TLX	
0043401 // st	0005634 // nu	0005515 // pr	Photorecepto	NR2E3_HUMAN|NR2E3|PNR|RNR	
0043401 // st	0005654 // nu	0001078 // tra	COUP transcri	COT1_HUMAN|NR2F1|EAR3|ERBAL3|TFCOUP1	
0043401 // st	0005634 // nu	0005515 // pr	COUP transcri	COT2_HUMAN|NR2F2|ARP1|TFCOUP2	
0043401 // st	0005654 // nu	0005515 // pr	Nuclear recep	NR2F6_HUMAN|NR2F6|EAR2|ERBAL2	

0000122 // ne	0005737 // cy	0004883 // gl	Glucocorticoi	GCR_HUMAN|NR3C1|GRL
0043401 // st	0005634 // nu	0004879 // RN	Nuclear recep	NR4A2_HUMAN|NR4A2|NOT|NURR1|TINUR
0043401 // st	0090575 // RN	0003682 // ch	Nuclear recep	NR5A2_HUMAN|NR5A2|B1F|CPF|FTF
0030518 // in	0005654 // nu	0004879 // RN	Nuclear recep	NR6A1_HUMAN|NR6A1|GCNF
0000122 // ne			Notch-regulat	NRARP_HUMAN|NRARP
0002223 // st	0005886 // pl	0003924 // G	GTPase NRas	RASN_HUMAN|NRAS|HRAS1
0006367 // tr	0035032 // ph	0005515 // pr	Nuclear recep	NRBF2_HUMAN|NRBF2|COPR
0007417 // ce	0043005 // ne	0005515 // pr	Neuronal cell	NRCAM_HUMAN|NRCAM|KIAA0343
0016246 // RN	0071013 // ca	0003674 // m	Protein NRDE	NRDE2_HUMAN|NRDE2|C14orf102
	0005737 // cy	0005515 // pr	Neuronal rege	NREP_HUMAN|NREP|C5orf13|P311
0000902 // ce	0005576 // ex	0030971 // re	Pro-neureguli	NRG1_HUMAN|NRG1|GGF|HGL|HRGA|NDF|SMDF
0038111 // in	0005576 // ex	0008083 // gr	Pro-neureguli	NRG2_HUMAN|NRG2|NTAK
0035556 // in	0005576 // ex	0030297 // tr	Pro-neureguli	NRG3_HUMAN|NRG3
0043547 // po	0005576 // ex	0008083 // gr	Pro-neureguli	NRG4_HUMAN|NRG4
0007399 // ne	0031966 // m	0005547 // ph	Neurogranin	NEUG_HUMAN|NRGN
0006508 // pr		0005515 // pr	Nuclear recep	NRIP3_HUMAN|NRIP3|C11orf14
0042981 // re	0005737 // cy	0005524 // A	Nik-related pr	NRK_HUMAN|NRK
0007468 // re	0005634 // nu	0000978 // RN	Neural retina-	NRL_HUMAN|NRL|D14S46E
1990138 // ne	0046658 // an	0042803 // pr	Neuritin-like p	NRN1L_HUMAN|NRN1L|UNQ2446/PRO5725
0061299 // re	0005925 // fo	0015026 // co	Neuropilin-1	NRP1_HUMAN|NRP1|NRP|VEGF165R
0097491 // sy	0005576 // ex	0019955 // cy	Neuropilin-2	NRP2_HUMAN|NRP2|VEGF165R2
0006801 // su	0016021 // in		Negative regu	NRROS_HUMAN|NRROS|LRRC33|UNQ3030/PRO9833
0008150 // bi	0030133 // tr	0003674 // m	Neurensin-2	NRSN2_HUMAN|NRSN2|C20orf98
0043547 // po	0005576 // ex	0008083 // gr	Neurturin	NRTN_HUMAN|NRTN
0007416 // sy	0005886 // pl	0004872 // re	Neurexin-3	NRX3A_HUMAN|NRXN3|C14orf60|KIAA0743
0000470 // m	0005730 // nu	0003723 // RN	Ribosome bio	NSA2_HUMAN|NSA2|TINP1|HUSSY-29
0016571 // hi	0005654 // nu	0018024 // hi	Histone-lysine	NSD3_HUMAN|NSD3|WHSC1L1|DC28
0048814 // re	0097440 // ap	0048306 // ca	NMDA recept	NSMF_HUMAN|NSMF|NELF
0002127 // w	0005739 // m	0016428 // tR	tRNA (cytosin	NSUN3_HUMAN|NSUN3|MSTP077|UG0651E06
0070475 // rR	0005730 // nu	0008173 // RN	Putative NOL1	NSN5B_HUMAN|NSUN5P1|NSUN5B|WBSCR20B
0032259 // m		0008168 // m	Putative meth	NSUN7_HUMAN|NSUN7
0006195 // pu	0005829 // cy	0000166 // nu	Cytosolic 5'-nu	5NT1B_HUMAN|NT5C1B|AIRP|FKSG85
0046040 // IM	0005829 // cy	0005515 // pr	Cytosolic puri	5NTC_HUMAN|NT5C2|NT5B|NT5CP|PNT5
0009117 // nu	0005737 // cy	0008253 // 5'	Cytosolic 5'-nu	5NT3A_HUMAN|NT5C3A|NT5C3|P5N1|UMPH1|HSPC23
0016311 // de	0043235 // re	0008253 // 5'	5'-nucleotidas	NT5D3_HUMAN|NT5DC3|GNN|TU12B1-TY
0006260 // DN	0005739 // m	0008252 // nu	5'(3')-deoxyrib	NT5M_HUMAN|NT5M|DNT2
0006511 // ub	0005634 // nu	0008418 // pr	Protein N-term	NTAN1_HUMAN|NTAN1
0043524 // ne	0005576 // ex	0008083 // gr	Neurotrophin	NTF3_HUMAN|NTF3
0060384 // in	0005576 // ex	0005166 // ne	Neurotrophin	NTF4_HUMAN|NTF4|NTF5
0045008 // de	0005654 // nu	0000703 // ox	Endonuclease	NTH_HUMAN|NTHL1|NTH1|OCTS3
0007155 // ce	0031225 // an	0005515 // pr	Neurotrimin	NTRI_HUMAN|NTM|IGLON2|NT|UNQ297/PRO337
0018012 // N-	0005737 // cy	0008276 // pr	N-terminal Xa	NTM1A_HUMAN|NTMT1|C9orf32|METTL11A|NRMT|NI
0016322 // ne	0005886 // pl	0043237 // la	Netrin-4	NET4_HUMAN|NTN4

0007409 // ax	0046658 // ar	0005515 // pr	Netrin-G1	NTNG1_HUMAN|NTNG1|KIAA0976|LMNT1|UNQ571/PF
0007409 // ax	0046658 // ar	0003674 // m	Netrin-G2	NTNG2_HUMAN|NTNG2|KIAA1857|LMNT2|UNQ9381/F
0050880 // re	0005576 // ex	0005184 // ne	Neurotensin/ı	NEUT_HUMAN|NTS
0014049 // pc	0005886 // pl	0016492 // G-	Neurotensin r	NTR1_HUMAN|NTSR1|NTRR
0007155 // ce	0005634 // nu	0005515 // pr	NUAK family S	NUAK1_HUMAN|NUAK1|ARK5|KIAA0537|OMPHK1
0006468 // pr	0005634 // nu	0005515 // pr	NUAK family S	NUAK2_HUMAN|NUAK2|OMPHK2|SNARK
0034612 // re	0005634 // nu	0005515 // pr	NEDD8 ultima	NUB1_HUMAN|NUB1|NYREN18
0016226 // irc	0005634 // nu	0005515 // pr	Cytosolic Fe-S	NUBP2_HUMAN|NUBP2
	0005829 // cy	0003677 // DI	Nucleobindin-	NUCB2_HUMAN|NUCB2|NEFA
0006457 // pr	0005829 // cy	0005515 // pr	Nuclear migra	NUDC_HUMAN|NUDC
0032502 // de	0005737 // cy	0005515 // pr	NudC domain	NUDC3_HUMAN|NUDCD3|KIAA1068
		0016787 // hy	Nucleoside di	NUD17_HUMAN|NUDT17
0034656 // nu	0005829 // cy	0044715 // 8-	8-oxo-dGDP p	NUD18_HUMAN|NUDT18|MTH3
0008150 // bi	0005782 // pe	0005102 // re	Nucleoside di	NUD19_HUMAN|NUDT19
0034599 // ce	0005759 // m	0004081 // bi	Bis(5'-nucleos	AP4A_HUMAN|NUDT2|APAH1
0043647 // in	0005829 // cy	0052843 // in	Diphosphoino	NUDT4_HUMAN|NUDT4|DIPP2|KIAA0487|HDCMB47P
0008150 // bi	0005739 // m	0016787 // hy	Nucleoside di	NUDT6_HUMAN|NUDT6|FGF2AS
0046356 // ac	0005782 // pe	0005102 // re	Peroxisomal c	NUDT7_HUMAN|NUDT7
0006396 // RI	0043234 // pr	0005515 // pr	Nuclear fragile	NUFP1_HUMAN|NUFIP1
0045618 // pc	0005813 // ce	0005515 // pr	Nuclear mitot	NUMA1_HUMAN|NUMA1|NMP22|NUMA
0007077 // m	0005635 // nu	0005515 // pr	Nuclear pore c	NU107_HUMAN|NUP107
0007077 // m	0005635 // nu	0005515 // pr	Nuclear pore c	NU133_HUMAN|NUP133
0007077 // m	0031965 // nu	0008139 // nu	Nuclear pore c	NU153_HUMAN|NUP153
0007077 // m	0005635 // nu	0046983 // pr	Nuclear pore	PO210_HUMAN|NUP210|KIAA0906|PSEC0245
0007077 // m	0005635 // nu	0005515 // pr	Nucleoporin N	NUP53_HUMAN|NUP35|MP44|NUP53
0007077 // m	0005635 // nu	0005515 // pr	Nucleoporin N	NUP43_HUMAN|NUP43
0007077 // m	0031965 // nu	0005515 // pr	Nuclear pore c	NUP50_HUMAN|NUP50|NPAP60L|PRO1146
0006606 // pr	0044613 // nu	0005487 // nu	Nucleoporin-6	N62CL_HUMAN|NUP62CL|NUP62L
0007077 // m	0005635 // nu	0005515 // pr	Nuclear pore c	NUP85_HUMAN|NUP85|NUP75|PCNT1
0007077 // m	0005635 // nu	0005515 // pr	Nuclear pore c	NUP98_HUMAN|NUP98|ADAR2
0016055 // W	0005829 // cy	0047134 // pr	Nucleoredoxir	NXN_HUMAN|NXN|NRX
0007608 // se	0005737 // cy	0004791 // th	Nucleoredoxir	NXNL2_HUMAN|NXNL2|C9orf121
	0005576 // ex	0005102 // re	Neurexophilin	NXPH1_HUMAN|NXPH1|NPH1|Nbla00697
0007218 // ne	0005576 // ex	0003674 // m	Neurexophilin	NXPH4_HUMAN|NXPH4|NPH4|UNQ1928/PRO4403
0014065 // pł	0005622 // in		Neuronal tyro	NYAP1_HUMAN|NYAP1|C7orf51
0008150 // bi	0016021 // in	0003676 // nu	Protein NYNR	NYNRI_HUMAN|NYNRIN|CGIN1|KIAA1305
	0070062 // ex		Out at first pr	OAF_HUMAN|OAF|NS5ATP13TP2
0006006 // gl	0005829 // cy	0005515 // pr	2'-5'-oligoade	OAS1_HUMAN|OAS1|OIAS
0006139 // nu	0005829 // cy	0003725 // dc	2'-5'-oligoade	OAS2_HUMAN|OAS2
0045071 // ne	0005829 // cy	0003725 // dc	2'-5'-oligoade	OAS3_HUMAN|OAS3|P/OKcl.4

0007030 // Go	0005737 // cy	0097493 // st	Obscurin-like	OBSL1_HUMAN|OBSL1|KIAA0657	
	0005768 // er		OCIA domain-	OCAD2_HUMAN|OCIAD2	
0007601 // vis			Oculomedin	TISR_HUMAN|OCLM	
0006461 // pr	0005923 // bi	0005515 // pr	Occludin	OCLN_HUMAN|OCLN	
0006595 // po	0005737 // cy	0042803 // pr	Ornithine dec	DCOR_HUMAN|ODC1	
	0005881 // cy		Outer dense f	OD3L2_HUMAN|ODF3L2|C19orf19	
		0005515 // pr	Orofacial cleft	OFCC1_HUMAN|OFCC1|MRDS1	
0000086 // G2	0005829 // cy	0005515 // pr	Oral-facial-dig	OFD1_HUMAN|OFD1|CXorf5	
0006096 // gly	0005739 // m	0034602 // ox	2-oxoglutarate	ODO1_HUMAN|OGDH	
0006096 // gly	0005829 // cy	0030976 // th	2-oxoglutarate	OGDHL_HUMAN|OGDHL|KIAA1290	
0034063 // st	0010494 // cy	0031543 // pe	Prolyl 3-hydro	OGFD1_HUMAN|OGFOD1|KIAA1612|TPA1	
0001558 // re	0005634 // nu	0004985 // op	Opioid growth	OGFR_HUMAN|OGFR	
0009314 // re	0016607 // nu	0140080 /// 0	N-glycosylase,	OGG1_HUMAN|OGG1|MMH|MUTM|OGH1	
0002576 // pl	0005576 // ex	0005515 // pr	Obg-like ATPa	OLA1_HUMAN|OLA1|GTPBP9|PRO2455|PTD004	
0051152 // po	0005737 // cy	0005515 // pr	Noelin-2	NOE2_HUMAN|OLFM2|NOE2	
0042462 // ey	0005615 // ex	0005515 // pr	Noelin-3	NOE3_HUMAN|OLFM3|NOE3|UNQ1924/PRO4399	
0030198 // ex	0005578 // pr	0050840 // ex	Olfactomedin	OLM2A_HUMAN|OLFML2A|UNQ9394/PRO34319	
	0005576 // ex		Olfactomedin	OLM2B_HUMAN|OLFML2B	
0006898 // re	0005886 // pl	0030246 // ca	Oxidized low-	OLR1_HUMAN|OLR1|CLEC8A|LOX1	
0042981 // re	0016021 // in	0004222 // m	Metalloendop	OMA1_HUMAN|OMA1|MPRP1	
0006355 // re	0005634 // nu	0001077 // tra	Hepatocyte nu	HNF6_HUMAN|ONECUT1|HNF6|HNF6A	
0045944 // po	0005634 // nu	0001077 // tra	One cut doma	ONEC2_HUMAN|ONECUT2|HNF6B	
0045944 // po	0005634 // nu	0001077 // tra	One cut doma	ONEC3_HUMAN|ONECUT3	
	0005576 // ex		Putative oocy	OOSP1_HUMAN|OOSP1	
0008053 // m	0005758 // m	0003924 // GT	Dynamin-like	OPA1_HUMAN|OPA1|KIAA0567	
0006750 // glu	0005829 // cy	0017168 // 5-	5-oxoprolinas	OPLA_HUMAN|OPLAH	
0018298 // pr	0097381 // ph	0004872 // re	Short-wave-se	OPSB_HUMAN|OPN1SW|BCP	
0018298 // pr	0005887 // in	0004930 // G-	Opsin-3	OPN3_HUMAN|OPN3|ECPN	
0007218 // ne	0005886 // pl	0004930 // G-	Nociceptin rec	OPRX_HUMAN|OPRL1|OOR|ORL1	
0033554 // ce	0005886 // pl	0031681 // G-	Mu-type opio	OPRM_HUMAN|OPRM1|MOR1	
0090161 // Go	0005794 // Go	0005515 // pr	Optineurin	OPTN_HUMAN|OPTN|FIP2|GLC1E|HIP7|HYPL|NRP	
0050907 // de	0016021 // in	0004984 // ol	Olfactory rece	OR2A7_HUMAN|OR2A7	
0007186 // G-	0016021 // in	0004984 // ol	Olfactory rece	OR2B6_HUMAN|OR2B6|OR2B1|OR2B1P|OR2B5|OR2B6	
0050907 // de	0016021 // in	0004984 // ol	Olfactory rece	OR4C6_HUMAN|OR4C6	
0007186 // G-	0016021 // in	0004984 // ol	Olfactory rece	O51B4_HUMAN|OR51B4	
0007186 // G-	0016021 // in	0004984 // ol	Olfactory rece	OR8A1_HUMAN|OR8A1	
0007186 // G-	0005886 // pl	0004984 // ol	Putative olfac	OR83P_HUMAN|OR8G3P	
0007186 // G-	0016021 // in	0004984 // ol	Olfactory rece	OR8G5_HUMAN|OR8G5|OR8G5P|OR8G6	

0050907 // de	0005886 // pl	0004984 // ol	Olfactory rece	OR8S1_HUMAN|OR8S1
0070588 // ca	0016021 // in	0015279 // st	Protein orai-2	ORAI2_HUMAN|ORAI2|C7orf19|CBCIP2|TMEM142B|PP
0070588 // ca	0016021 // in	0015279 // st	Protein orai-3	ORAI3_HUMAN|ORAI3|TMEM142C
0031573 // in	0030896 // ch	0003674 // m	Oral cancer-o	ORAV1_HUMAN|ORAOV1|TAOS1
0006260 // DI	0005654 // nu	0005515 // pr	Origin recogni	ORC2_HUMAN|ORC2|ORC2L
1900060 // ne	0016021 // in		ORM1-like pro	ORML2_HUMAN|ORMDL2|HSPC160|MSTP095
0030433 // EF	0005789 // er	0005515 // pr	Protein OS-9	OS9_HUMAN|OS9
0044128 // po	0005794 // Go	0005515 // pr	Oxysterol-bin	OSBP1_HUMAN|OSBP|OSBP1
0007286 // sp	0016020 // m	0015485 // ch	Oxysterol-bin	OSBP2_HUMAN|OSBP2|KIAA1664|ORP4|OSBPL4
0036150 // ph	0005856 // cy	0015485 // ch	Oxysterol-bin	OSB10_HUMAN|OSBPL10|ORP10|OSBP9
0045444 // fa	0005794 // Go	0008289 // lip	Oxysterol-bin	OSB11_HUMAN|OSBPL11|ORP11|OSBP12
0006893 // Go	0005829 // cy	0008142 // ox	Oxysterol-bin	OSBL5_HUMAN|OSBPL5|KIAA1534|OBPH1|ORP5
0015918 // st	0005829 // cy	0008289 // lip	Oxysterol-bin	OSBL6_HUMAN|OSBPL6|ORP6
0010506 // re	0005829 // cy	0005515 // pr	Oxysterol-bin	OSBL7_HUMAN|OSBPL7|ORP7
0050776 // re	0016021 // in		Osteoclast-as	OSCAR_HUMAN|OSCAR
1903955 // po	0009925 // ba		Protein OSCP1	OSCP1_HUMAN|OSCP1|C1orf102|NOR1
0002949 // tR	0000408 // EK	0005515 // pr	Probable tRN/	OSGEP_HUMAN|OSGEP|GCPL1
0007275 // m	0005575 // ce	0005515 // pr	Oxidative stre	OSGI1_HUMAN|OSGIN1|OKL38
0034097 // re	0005886 // pl	0004924 // or	Oncostatin-M	OSMR_HUMAN|OSMR|OSMRB
0045944 // po	0019898 // ex	0005515 // pr	Protein odd-sl	OSR1_HUMAN|OSR1|ODD
0045893 // po	0005634 // nu	0043565 // se	Protein odd-sl	OSR2_HUMAN|OSR2
0007165 // sig	0034774 // se	0005515 // pr	Osteoclast-sti	OSTF1_HUMAN|OSTF1
0046373 // L-	0005576 // ex	0046556 // al	Otogelin-like	OTOGL_HUMAN|OTOGL|C12orf64
0002250 // ac	0070062 // ex	0005515 // pr	Ubiquitin thio	OTUB1_HUMAN|OTUB1|OTB1|OTU1|HSPC263
0051898 // ne	0005829 // cy	0004843 // th	OTU domain-c	OTUD3_HUMAN|OTUD3|KIAA0459
0043124 // ne	0005737 // cy	0003677 // DI	OTU domain-c	OTU7A_HUMAN|OTUD7A|C15orf16|CEZANNE2|OTUD7
0070431 // nu	0005737 // cy	0004843 // th	Ubiquitin thio	OTUL_HUMAN|OTULIN|FAM105B
0032526 // re	0005737 // cy	0016787 // hy	Esterase OVC/	OVCA2_HUMAN|OVCA2
0006508 // pr	0005576 // ex	0004252 // se	Ovochymase-	OVCH1_HUMAN|OVCH1
0006032 // ch	0005829 // cy	0008061 // ch	Oviduct-speci	OVGP1_HUMAN|OVGP1|MUC9|OGP
0007498 // m	0005634 // nu	0000978 // RI	Putative trans	OVOL1_HUMAN|OVOL1
0048557 // er	0005634 // nu	0000981 // RI	Transcription	OVOL2_HUMAN|OVOL2|ZNF339
0006351 // tr	0005634 // nu	0000981 // RI	Putative trans	OVOL3_HUMAN|OVOL3
0010951 // ne	0005615 // ex	0004867 // se	Ovostatin hon	OVOS2_HUMAN|OVOS2
0046952 // ke	0005759 // m	0008260 // 3-	Succinyl-CoA:	SCOT2_HUMAN|OXCT2|FKSG25
0007186 // G-	0005886 // pl	0050646 // 5-	Oxoeicosanoi	OXER1_HUMAN|OXER1|GPR170|TG1019
0007186 // G-	0005886 // pl	0004930 // G-	2-oxoglutarat	OXGR1_HUMAN|OXGR1|GPR80|GPR99|P2RY15|P2Y15
0007628 // ad	0005739 // m	0016491 // ox	Oxidation resi	OXR1_HUMAN|OXR1|Nbla00307
0007613 // m	0005886 // pl	0004990 // ox	Oxytocin rece	OXYR_HUMAN|OXTR
0098655 // ca	0005886 // pl	0001614 // pu	P2X purinocep	P2RX5_HUMAN|P2RX5|P2X5

0098655 // ca	0005886 // pl	0004931 // ex	P2X purinocep	P2RX6_HUMAN|P2RX6|P2RXL1|P2X6
0045944 // pc	0005886 // pl	0045031 // AT	P2Y purinocep	P2RY1_HUMAN|P2RY1
0019722 // ca	0005886 // pl	0004872 // re	P2Y purinocep	P2Y11_HUMAN|P2RY11
0007200 // ph	0005886 // pl	0004872 // re	P2Y purinocep	P2RY2_HUMAN|P2RY2|P2RU1
0030321 // tra	0005886 // pl	0004930 // G-	P2Y purinocep	P2RY6_HUMAN|P2RY6|PP2891
0008285 // ne	0005783 // er	0031418 // L-a	Prolyl 3-hydro	P3H2_HUMAN|P3H2|LEPREL1|MLAT4
0032963 // co	1902494 // ca	0031418 // L-a	Prolyl 3-hydro	P3H3_HUMAN|P3H3|LEPREL2
0019511 // pe	0005788 // er	0005515 // pr	Prolyl 4-hydro	P4HA3_HUMAN|P4HA3|UNQ711/PRO1374
0045646 // re	0016021 // int	0016706 // ox	Transmembra	P4HTM_HUMAN|P4HTM|PH4
0045892 // ne	0005576 // ex	0003723 // RN	Proliferation-a	PA2G4_HUMAN|PA2G4|EBP1
0016032 // vir	0000502 // pr	0005515 // pr	Proteasomal A	PAAF1_HUMAN|PAAF1|WDR71
0031047 // ge	0005925 // fo	0003723 // RN	Polyadenylate	PABP1_HUMAN|PABPC1|PAB1|PABP1|PABPC2
0048096 // ch	0070062 // ex	0003723 // RN	Polyadenylate	PAP1L_HUMAN|PABPC1L|C20orf119
		0003723 // RN	Polyadenylate	PAB4L_HUMAN|PABPC4L
	0005759 // m	0003723 // RN	Polyadenylate	PABP5_HUMAN|PABPC5|PABP5
0031124 // m	0005654 // nu	0003723 // RN	Polyadenylate	PABP2_HUMAN|PABPN1|PAB2|PABP2
0050690 // re	0005829 // cy	0005515 // pr	Phosphofurin	PACS1_HUMAN|PACS1|KIAA1175
0045806 // ne	0043679 // ax	0008092 // cy	Protein kinase	PACN1_HUMAN|PACSIN1|KIAA1379
0006325 // ch	0005634 // nu	0005509 // ca	Protein-argini	PADI1_HUMAN|PADI1|PAD1|PDI1
0006325 // ch	0005634 // nu	0004668 // pr	Protein-argini	PADI3_HUMAN|PADI3|PAD3|PDI3
0006325 // ch	0005654 // nu	0004668 // pr	Protein-argini	PADI4_HUMAN|PADI4|PAD4|PADI5|PDI5
0006310 // DN	0044666 // M	0030331 // es	PAXIP1-associ	PAGR1_HUMAN|PAGR1|C16orf53|PA1
0006559 // L-p	0070062 // ex	0004505 // ph	Phenylalanine	PH4H_HUMAN|PAH
1901796 // re	0005730 // nu	0005515 // pr	p21-activated	PK1IP_HUMAN|PAK1IP1|PIP1|WDR84
0071407 // ce	0005829 // cy	0004674 // pr	Serine/threon	PAK2_HUMAN|PAK2
0002223 // sti	0005768 // en	0004674 // pr	Serine/threon	PAK3_HUMAN|PAK3|OPHN3
0023014 // sig	0001650 // fib	0005515 // pr	Serine/threon	PAK6_HUMAN|PAK6|PAK5
0008360 // re	0005886 // pl		Paralemmin-2	PALM2_HUMAN|PALM2
0008360 // re	0016020 // m		PALM2-AKAP2	B1ALY0_HUMAN|PALM2-AKAP2
0001960 // ne	0005886 // pl	0005524 // AT	Paralemmin-3	PALM3_HUMAN|PALM3
0008360 // re	0016020 // m		Palmdelphin	PALMD_HUMAN|PALMD|C1orf11|PALML
0018032 // pr	0009986 // ce	0005507 // co	Peptidyl-glycin	AMD_HUMAN|PAM
	0005615 // ex	0005509 // ca	Inactive serine	PAMR1_HUMAN|PAMR1|RAMP|FP938|UNQ699/PRO13
0090503 // RN	0000932 // cy	0003676 // nu	PAN2-PAN3 d	PAN2_HUMAN|PAN2|KIAA0710|USP52
0016310 // ph	0005737 // cy	0004594 // pa	Pantothenate	PANK3_HUMAN|PANK3
0016310 // ph	0005737 // cy	0004594 // pa	Pantothenate	PANK4_HUMAN|PANK4
0043065 // po	0005730 // nu		Proapoptotic	PANO1_HUMAN|PANO1|PANO
0006812 // ca	0005886 // pl	0055077 // ga	Pannexin-2	PANX2_HUMAN|PANX2
0006812 // ca	0005886 // pl	0055077 // ga	Pannexin-3	PANX3_HUMAN|PANX3
0042493 // re	0005794 // Go	0004652 // po	Non-canonica	PAPD7_HUMAN|PAPD7|POLS|TRF4
0006508 // pr	0005576 // ex	0004867 // se	Papilin	PPN_HUMAN|PAPLN|UNQ2420/PRO4977

0006378 // m	0005654 // nu	0000287 // m	Poly(A) polym	PAPOA_HUMAN|PAPOLA|PAP
0006508 // pr	0005576 // ex	0004222 // m	Pappalysin-1	PAPP1_HUMAN|PAPPA
0001558 // re	0070062 // ex	0004222 // m	Pappalysin-2	PAPP2_HUMAN|PAPPA2|PLAC3
0000103 // su	0005829 // cy	0004020 // ac	Bifunctional 3	PAPS2_HUMAN|PAPSS2|ATPSK2
0007275 // m	0016021 // in	0005515 // pr	Membrane pr	PAQR5_HUMAN|PAQR5|MPRG
0043401 // st	0016021 // in	0003707 // st	Membrane pr	PAQR6_HUMAN|PAQR6
0007275 // m	0016021 // in	0003707 // st	Membrane pr	PAQR7_HUMAN|PAQR7|MRPA
0043401 // st	0016021 // in	0003707 // st	Membrane pr	PAQR9_HUMAN|PAQR9
0008356 // as	0005938 // ce	0005515 // pr	Partitioning d	PARD3_HUMAN|PARD3|PAR3|PAR3A
0051301 // ce	0012505 // en	0005515 // pr	Partitioning d	PAR3L_HUMAN|PARD3B|ALS2CR19|PAR3B|PAR3L
0001933 // ne	0005829 // cy	0005515 // pr	Partitioning d	PAR6A_HUMAN|PARD6A|PAR6A
0030334 // re	0005634 // nu	0005515 // pr	Partitioning d	PAR6B_HUMAN|PARD6B|PAR6B
0051301 // ce	0043234 // pr	0005515 // pr	Partitioning d	PAR6G_HUMAN|PARD6G|PAR6G
0005975 // ca	0005634 // nu	0004649 // pc	Poly(ADP-ribo	PARG_HUMAN|PARG
0033234 // ne	0005739 // m	0005515 // pr	Protein/nucle	PARK7_HUMAN|PARK7
0051973 // pc	0005769 // ea		Prostate andr	PARM1_HUMAN|PARM1|UNQ1879/PRO4322
0090503 // RN	0005634 // nu	0003723 // RN	Poly(A)-specif	PARN_HUMAN|PARN|DAN
2000679 // pc	0005667 // tr	0003723 // RN	Poly [ADP-ribo	PARP1_HUMAN|PARP1|ADPRT|PPOL
1900045 // ne	0005737 // cy	0003950 // NA	Poly [ADP-ribo	PAR10_HUMAN|PARP10
0007283 // sp	0005643 // nu	0003950 // NA	Poly [ADP-ribo	PAR11_HUMAN|PARP11|C12orf6
	0005634 // nu	0003723 // RN	Poly [ADP-ribo	PAR12_HUMAN|PARP12|ZC3HDC1
0010629 // ne	0005829 // cy	0003723 // RN	Poly [ADP-ribo	PAR14_HUMAN|PARP14|BAL2|KIAA1268
0070212 // pr	0005634 // nu	0070403 // NA	Poly [ADP-ribo	PAR15_HUMAN|PARP15|BAL3
0070213 // pr	0016021 // in	0003950 // NA	Mono [ADP-ri	PAR16_HUMAN|PARP16|ARTD15|C15orf30
0051103 // DI	0005737 // cy	0005515 // pr	Poly [ADP-ribo	PARP2_HUMAN|PARP2|ADPRT2|ADPRTL2
0051103 // DI	0005737 // cy	0003910 // DI	Poly [ADP-ribo	PARP3_HUMAN|PARP3|ADPRT3|ADPRTL3
0006974 // ce	0005737 // cy	0003950 // NA	Poly [ADP-ribo	PARP4_HUMAN|PARP4|ADPRTL1|KIAA0177|PARPL
0010629 // ne	0005634 // nu	0003950 // NA	Poly [ADP-ribo	PARP9_HUMAN|PARP9|BAL|BAL1
0003148 // ou	0030018 // Z	0045296 // ca	Alpha-parvin	PARVA_HUMAN|PARVA|MXRA2
0035556 // in	0005923 // bi	0005515 // pr	InaD-like prot	INADL_HUMAN|PATJ|INADL
0010607 // ne	0030529 // in	0003723 // RN	Protein PAT1	PATL2_HUMAN|PATL2
0045944 // pc	0005634 // nu	0001077 // tr	POZ-, AT hook	PATZ1_HUMAN|PATZ1|PATZ|RIAZ|ZBTB19|ZNF278|ZSG
0007283 // sp	0005654 // nu	0001077 // tr	Paired box pro	PAX5_HUMAN|PAX5
0001823 // m	0005654 // nu	0003677 // DI	Paired box pro	PAX8_HUMAN|PAX8
0045944 // pc	0005634 // nu	0001228 // tr	Paired box pro	PAX9_HUMAN|PAX9
0060261 // pc	0005654 // nu	0005515 // pr	PAX-interactin	PAXI1_HUMAN|PAXIP1|PAXIP1L|PTIP|CAGF28
0008150 // bi	0005737 // cy	0003674 // m	Protein PBDC1	PBDC1_HUMAN|PBDC1|CXorf26
0000278 // m	0005634 // nu	0005515 // pr	Lymphokine-a	TOPK_HUMAN|PBK|TOPK
0035019 // so	0005737 // cy	0005515 // pr	Pre-B-cell leuk	PBX1_HUMAN|PBX1|PRL
0006366 // tr	0005634 // nu	0001205 // tr	Pre-B-cell leuk	PBX3_HUMAN|PBX3

0006366 // tra	0001741 // XY	0005515 // pr	Pre-B-cell leuk	PBX4_HUMAN|PBX4
0007275 // m	0005829 // cy	0005515 // pr	Pre-B-cell leuk	PBIP1_HUMAN|PBXIP1|HPIP
0006090 // py	0005759 // m	0004736 // py	Pyruvate carb	PYC_HUMAN|PC
0006559 // L-	0070062 // ex	0005515 // pr	Pterin-4-alpha	PHS_HUMAN|PCBD1|DCOH|PCBD
0006977 // DI	0005829 // cy	0003723 // RI	Poly(rC)-bindi	PCBP4_HUMAN|PCBP4
0019626 // sh	0005759 // m	0004658 // pr	Propionyl-CoA	PCCB_HUMAN|PCCB
0007399 // ne	0005887 // in	0005509 // ca	Protocadherin	PCDH1_HUMAN|PCDH1
0007156 // hc	0005887 // in	0005509 // ca	Protocadherin	PCD10_HUMAN|PCDH10|KIAA1400
0010923 // ne	0005887 // in	0005509 // ca	Protocadherin	PC11X_HUMAN|PCDH11X|KIAA1326|PCDH11|PCDHX
0007156 // hc	0016021 // in	0005509 // ca	Protocadherin	PC11Y_HUMAN|PCDH11Y|PCDH11|PCDH22|PCDHY
0060711 // lal	0005886 // pl	0005509 // ca	Protocadherin	PCD12_HUMAN|PCDH12|UNQ395/PRO731
0048839 // in	0032420 // st	0005509 // ca	Protocadherin	PCD15_HUMAN|PCDH15|USH1F
0030534 // ac	0005887 // in	0005515 // pr	Protocadherin	PCD17_HUMAN|PCDH17|PCDH68|PCH68
0007155 // ce	0005887 // in	0005509 // ca	Protocadherin	PCD18_HUMAN|PCDH18|KIAA1562
0007156 // hc	0016021 // in	0003723 // RI	Protocadherin	PCD20_HUMAN|PCDH20|PCDH13
0002576 // pl	0005886 // pl	0005509 // ca	Protocadherin	PCDH7_HUMAN|PCDH7|BHPCDH
0030900 // fo	0016021 // in	0005509 // ca	Protocadherin	PCDH9_HUMAN|PCDH9
0007155 // ce	0005634 // nu	0005509 // ca	Protocadherin	PCDA2_HUMAN|PCDHA2
0007155 // ce	0005887 // in	0005509 // ca	Protocadherin	PCDA3_HUMAN|PCDHA3
0007155 // ce	0005887 // in	0005509 // ca	Protocadherin	PCDA4_HUMAN|PCDHA4
0007155 // ce	0005887 // in	0005509 // ca	Protocadherin	PCDA5_HUMAN|PCDHA5|CNRS6
0007155 // ce	0005887 // in	0042802 // id	Protocadherin	PCDA7_HUMAN|PCDHA7|CNRS4
0007416 // sy	0005887 // in	0005509 // ca	Protocadherin	PCDBA_HUMAN|PCDHB10|UNQ1906/PRO4352
0007416 // sy	0016021 // in	0005509 // ca	Protocadherin	PCDBB_HUMAN|PCDHB11
0007416 // sy	0005887 // in	0005509 // ca	Protocadherin	PCDBC_HUMAN|PCDHB12
0007416 // sy	0005887 // in	0005509 // ca	Protocadherin	PCDBD_HUMAN|PCDHB13|UNQ332/PRO531
0007416 // sy	0005887 // in	0005515 // pr	Protocadherin	PCDBE_HUMAN|PCDHB14
0007416 // sy	0005887 // in	0005509 // ca	Protocadherin	PCDBF_HUMAN|PCDHB15
0007416 // sy	0005887 // in	0005509 // ca	Protocadherin	PCDBG_HUMAN|PCDHB16|KIAA1621|PCDH3X
0007416 // sy	0016021 // in	0005509 // ca	Protocadherin	PCDB4_HUMAN|PCDHB4
0007416 // sy	0005887 // in	0005509 // ca	Protocadherin	PCDB5_HUMAN|PCDHB5
0007416 // sy	0005887 // in	0005509 // ca	Protocadherin	PCDB6_HUMAN|PCDHB6
0007416 // sy	0005887 // in	0005509 // ca	Protocadherin	PCDB7_HUMAN|PCDHB7
0007416 // sy	0005887 // in	0005509 // ca	Protocadherin	PCDB8_HUMAN|PCDHB8|PCDH3I
0007416 // sy	0005887 // in	0005509 // ca	Protocadherin	PCDB9_HUMAN|PCDHB9|PCDH3H
0007155 // ce	0005887 // in	0005509 // ca	Protocadherin	PCDG1_HUMAN|PCDHGA1
0007155 // ce	0005887 // in	0005509 // ca	Protocadherin	PCDG4_HUMAN|PCDHGA4
0007155 // ce	0005887 // in	0005509 // ca	Protocadherin	PCDG6_HUMAN|PCDHGA6
0007155 // ce	0005887 // in	0003723 // RI	Protocadherin	PCDG9_HUMAN|PCDHGA9
0007155 // ce	0005887 // in	0005509 // ca	Protocadherin	PCDGD_HUMAN|PCDHGB1
0007155 // ce	0005887 // in	0005509 // ca	Protocadherin	PCDGE_HUMAN|PCDHGB2

0007155 // ce	0005887 // in	0005509 // ca	Protocadherin	PCDGF_HUMAN|PCDHGB3
0007155 // ce	0005887 // in	0003674 // m	Protocadherin	PCDGH_HUMAN|PCDHGB5
		0005515 // pr	PC-esterase d	PED1B_HUMAN|PCED1B|FAM113B
0045944 // po	0005813 // ce	0005515 // pr	Polycomb gro	PCGF5_HUMAN|PCGF5|RNF159
0000122 // ne	0031519 // Pc	0003700 // tra	Polycomb gro	PCGF6_HUMAN|PCGF6|MBLR|RNF134
0099526 // pr	0016020 // m	0005509 // ca	Protein piccol	PCLO_HUMAN|PCLO|ACZ|KIAA0559
0006479 // pr	0005737 // cy	0004719 // pr	Protein-L-isoa	PCMD2_HUMAN|PCMTD2|C20orf36
0007049 // ce	0016604 // nu	0005515 // pr	PEST proteoly	PCNP_HUMAN|PCNP
	0016021 // in		Pecanex-like p	PCX3_HUMAN|PCNX3|PCNXL3
	0016021 // in	0005515 // pr	Pecanex-like p	PCX4_HUMAN|PCNX4|C14orf135|FBP2|PCNXL4
0010952 // po	0070062 // ex	0005518 // co	Procollagen C	PCOC2_HUMAN|PCOLCE2|PCPE2|UNQ250/PRO287
0022414 // re	0002080 // ac	0004252 // se	Proprotein co	PCSK4_HUMAN|PCSK4|PC4|UNQ2757/PRO6496
0032455 // ne	0005794 // Go	0004252 // se	Proprotein co	PCSK5_HUMAN|PCSK5|PC5|PC6
0032455 // ne	0009986 // ce	0004175 // en	Proprotein co	PCSK6_HUMAN|PCSK6|PACE4
0006644 // ph	0005791 // ro	0003723 // RN	Proprotein co	PCSK9_HUMAN|PCSK9|NARC1|PSEC0052
0006869 // lip	0005829 // cy	0008525 // ph	Phosphatidylc	PPCT_HUMAN|PCTP|STARD2
0030327 // pr	0005774 // va	0001735 // pr	Prenylcysteine	PCYOX_HUMAN|PCYOX1|KIAA0908|PCL1|UNQ597/PRO
0030327 // pr	0005774 // va	0001735 // pr	Prenylcysteine	PCYXL_HUMAN|PCYOX1L|PSEC0105
0006657 // CD	0005635 // nu	0042803 // pr	Choline-phosp	PCY1A_HUMAN|PCYT1A|CTPCT|PCYT1
0007283 // sp	0005737 // cy	0004105 // ch	Choline-phosp	PCY1B_HUMAN|PCYT1B|CCTB
0008616 // qu	0001917 // ph	0004859 // ph	Phosducin	PHOS_HUMAN|PDC
0033138 // po	0005829 // cy	0005515 // pr	Programmed	PDC10_HUMAN|PDCD10|CCM3|TFAR15
0000466 // m	0005654 // nu	0003723 // RN	Protein RRP5	RRP5_HUMAN|PDCD11|KIAA0185
0006955 // im	0005886 // pl	0003674 // m	Programmed	PD1L2_HUMAN|PDCD1LG2|B7DC|CD273|PDCD1L2|PDL
0007049 // ce	0005737 // cy		Programmed	PDD2L_HUMAN|PDCD2L
0045786 // ne	0005737 // cy	0005515 // pr	Programmed	PDCD4_HUMAN|PDCD4|H731
0043280 // po	0005829 // cy	0010698 // ac	Programmed	PDCD5_HUMAN|PDCD5|TFAR19
0006888 // ER	0005737 // cy	0048306 // ca	Programmed	PDCD6_HUMAN|PDCD6|ALG2
0008616 // qu	0005737 // cy	0008479 // qu	Phosducin-like	PDCL2_HUMAN|PDCL2
0008616 // qu	0005737 // cy	0044183 // pr	Phosducin-like	PDCL3_HUMAN|PDCL3|PhLP2A|VIAF1
0043949 // re	0016020 // m	0004115 // 3',	cAMP and cAM	PDE10_HUMAN|PDE10A
0008152 // m	0005829 // cy	0004115 // 3',	Dual 3',5'-cycl	PDE11_HUMAN|PDE11A
0071346 // ce	0005759 // m	0000175 // 3'-	2',5'-phospho	PDE12_HUMAN|PDE12
0034391 // re	0005829 // cy	0004115 // 3',	Calcium/calm	PDE1A_HUMAN|PDE1A
0071560 // ce	0005886 // pl	0004115 // 3',	cGMP-depend	PDE2A_HUMAN|PDE2A
0071560 // ce	0016021 // in	0004115 // 3',	cGMP-inhibite	PDE3A_HUMAN|PDE3A
0032869 // ce	0032045 // gu	0004115 // 3',	cGMP-inhibite	PDE3B_HUMAN|PDE3B
0006198 // cA	0016020 // m	0004115 // 3',	cAMP-specific	PDE4A_HUMAN|PDE4A|DPDE2
0050852 // T c	0000930 // ga	0004115 // 3',	cAMP-specific	PDE4B_HUMAN|PDE4B|DPDE4
0006198 // cA	0005891 // vo	0004115 // 3',	cAMP-specific	PDE4D_HUMAN|PDE4D|DPDE3
0046069 // cG	0005829 // cy	0047555 // 3',	cGMP-specific	PDE5A_HUMAN|PDE5A|PDE5
0046037 // GI	0005886 // pl	0047555 // 3',	Rod cGMP-spe	PDE6A_HUMAN|PDE6A|PDEA

0007268 // ch	0005829 // cy	0004115 // 3',	cAMP-specific	PDE7B_HUMAN|PDE7B
0046676 // ne	0005829 // cy	0004115 // 3',	High affinity c	PDE8B_HUMAN|PDE8B|PIG22
0019934 // cG	0005829 // cy	0005515 // pr	High affinity c	PDE9A_HUMAN|PDE9A
0008284 // po	0005739 // m	0042586 // pe	Peptide defor	DEFM_HUMAN|PDF|PDF1A
0008284 // po	0005615 // ex	0005515 // pr	Platelet-deriv	PDGFA_HUMAN|PDGFA|PDGF1
0007507 // he	0009986 // ce	0005515 // pr	Platelet-deriv	PDGFB_HUMAN|PDGFB|PDGF2|SIS
0007171 // ac	0009986 // ce	0008083 // gr	Platelet-deriv	PDGFC_HUMAN|PDGFC|SCDGF|UNQ174/PRO200
0048008 // pl	0000139 // Go	0008083 // gr	Platelet-deriv	PDGFD_HUMAN|PDGFD|IEGF|SCDGFB|MSTP036|UNQ1
0071230 // ce	0005902 // m	0005515 // pr	Platelet-deriv	PGFRA_HUMAN|PDGFRA|PDGFR2|RHEPDGFRA
0072075 // m	0005737 // cy	0005515 // pr	Platelet-deriv	PGFRB_HUMAN|PDGFRB|PDGFR|PDGFR1
0007186 // G-	0005576 // ex	0005019 // pl	Platelet-deriv	PGFRL_HUMAN|PDGFRL|PRLTS
0055114 // ox	0005783 // en	0015036 // di	Protein disulfi	PDIA2_HUMAN|PDIA2|PDIP
0008283 // ce	0005739 // m	0004740 // py	[Pyruvate deh	PDK1_HUMAN|PDK1|PDHK1
0010906 // re	0005967 // m	0004740 // py	[Pyruvate deh	PDK2_HUMAN|PDK2|PDHK2
0009267 // ce	0005759 // m	0004740 // py	[Pyruvate deh	PDK4_HUMAN|PDK4|PDHK4
0006979 // re	0005913 // ce	0098641 // ca	PDZ and LIM c	PDLI1_HUMAN|PDLIM1|CLIM1|CLP36
	0070062 // ex	0046872 // m	PDZ and LIM c	PDLI2_HUMAN|PDLIM2|PP6345
0007507 // he	0030018 // Z	0005515 // pr	PDZ and LIM c	PDLI3_HUMAN|PDLIM3|ALP
		0005515 // pr	PDZ and LIM c	PDLI4_HUMAN|PDLIM4|RIL
0061001 // re	0045211 // po	0098641 // ca	PDZ and LIM c	PDLI5_HUMAN|PDLIM5|ENH|L9
0001946 // lyı	0031528 // m	0005515 // pr	Podoplanin	PDPN_HUMAN|PDPN|GP36|PSEC0003|PSEC0025
0006457 // pr	0005737 // cy	0005515 // pr	p53 and DNA	PDRG1_HUMAN|PDRG1|C20orf126|PDRG
0007062 // sis	0000785 // ch	0005515 // pr	Sister chroma	PDS5B_HUMAN|PDS5B|APRIN|AS3|KIAA0979
0042816 // vit	0034774 // se	0005524 // AT	Pyridoxal kina	PDXK_HUMAN|PDXK|C21orf124|C21orf97|PKH|PNK|PF
0031247 // ac	0070938 // co	0005515 // pr	Pyridoxal pho	PLPP_HUMAN|PDXP|CIN|PLP|PLPP
0055085 // tra	0098793 // pr	0005515 // pr	PDZ domain-c	PDZ11_HUMAN|PDZD11|AIPP1|PDZK11|PISP|HSPC227|
	0005938 // ce		PDZ domain-c	PDZD4_HUMAN|PDZD4|KIAA1444|PDZK4|PDZRN4L
0045184 // es	0005929 // cil	0046982 // pr	PDZ domain-c	PDZD7_HUMAN|PDZD7|PDZK7
	0016021 // int		PDZK1-interac	PDZ1I_HUMAN|PDZK1IP1|MAP17
1902042 // ne	0005829 // cy	0005515 // pr	Astrocytic pho	PEA15_HUMAN|PEA15
0045746 // ne	0001891 // ph		Platelet endot	PEAR1_HUMAN|PEAR1|MEGF12
0000165 // M	0070062 // ex	0003723 // RN	Phosphatidyle	PEBP1_HUMAN|PEBP1|PBP|PEBP
	0070062 // ex	0005515 // pr	Phosphatidyle	PEBP4_HUMAN|PEBP4|CORK1|UNQ1933/PRO4408
0051592 // re	0000139 // Go	0003723 // RN	Peflin	PEF1_HUMAN|PEF1|ABP32|UNQ1845/PRO3573
0006915 // ap	0005829 // cy	0003723 // RN	Retrotranspos	PEG10_HUMAN|PEG10|EDR|KIAA1051|MAR2|MART2|I
0000209 // pr	0005829 // cy	0005515 // pr	E3 ubiquitin-p	PELI2_HUMAN|PELI2
0010804 // ne	0005829 // cy	0061630 // ub	E3 ubiquitin-p	PELI3_HUMAN|PELI3
0032790 // rib	0005737 // cy	0043022 // rib	Protein pelota	PELO_HUMAN|PELO|CGI-17
0006508 // pr	0070062 // ex	0005515 // pr	Xaa-Pro dipep	PEPD_HUMAN|PEPD|PRD
0051591 // re	0005737 // cy	0070888 // E-	Period circadi	PER1_HUMAN|PER1|KIAA0482|PER|RIGUI
0045187 // re	0005634 // nu	0031625 // ub	Period circadi	PER3_HUMAN|PER3|GIG13
0097186 // ar	0005886 // pl		p53 apoptosis	PERP_HUMAN|PERP|KCP1|KRTCAP1|PIGPC1|THW
0033365 // pr	0005654 // nu	0003723 // RN	Pescadillo hor	PESC_HUMAN|PES1
0033617 // m	0005739 // m		Protein PET11	PT117_HUMAN|PET117|UNQ607/PRO1194

0060152 // m	0005777 // pe	0005515 // pr	Peroxisome b	PEX1_HUMAN|PEX1
0016558 // pr	0005778 // pe	0005515 // pr	Peroxisome b	PEX10_HUMAN|PEX10|RNF69
0016559 // pe	0005777 // pe	0005515 // pr	Peroxisomal n	PX11C_HUMAN|PEX11G|PEX11C
0016558 // pr	0005783 // er	0005515 // pr	Peroxisomal n	PEX16_HUMAN|PEX16
0007031 // pe	0005777 // pe	0005515 // pr	Peroxisomal b	PEX3_HUMAN|PEX3
0016558 // pr	0005737 // cy	0005052 // pe	Peroxisomal t	PEX5_HUMAN|PEX5|PXR1
0050821 // pr	0005777 // pe	0005515 // pr	Peroxisome as	PEX6_HUMAN|PEX6|PXAAA1
0009168 // pu	0070062 // ex	0004642 // ph	Phosphoribos	PUR4_HUMAN|PFAS|KIAA0361
0007010 // cy	0005737 // cy	0044183 // pr	Prefoldin subu	PFD1_HUMAN|PFDN1|PFD1
0006457 // pr	0005634 // nu	0005515 // pr	Prefoldin subu	PFD4_HUMAN|PFDN4|PFD4
0031100 // ar	0043540 // 6-	0005515 // pr	6-phosphofru	F261_HUMAN|PFKFB1|F6PK|PFRX
0006007 // gl	0005829 // cy	0003873 // 6-	6-phosphofru	F262_HUMAN|PFKFB2
0010634 // pc	0005938 // ce	0003723 // RN	Profilin-1	PROF1_HUMAN|PFN1
0030163 // pr	0070062 // ex	0008233 // pe	Pepsin A-5	PEPA5_HUMAN|PGA5
0006941 // st	0005829 // cy	0004082 // bi	Phosphoglyce	PGAM2_HUMAN|PGAM2|PGAMM
0015798 // m	0016021 // in	0016788 // hy	GPI inositol-de	PGAP1_HUMAN|PGAP1|UNQ3024/PRO9822
0006505 // GI	0016021 // in	0016788 // hy	Post-GPI attac	PGAP3_HUMAN|PGAP3|CAB2|PERLD1|UNQ546/PRO11
0006355 // re	0016020 // m	0005515 // pr	PiggyBac trans	PGBD1_HUMAN|PGBD1|hucep-4
0050930 // in	0005576 // ex	0005515 // pr	Placenta grow	PLGF_HUMAN|PGF|PGFL|PLGF
0005978 // gl	0005829 // cy	0004614 // ph	Phosphogluco	PGM1_HUMAN|PGM1
0005978 // gl	0005829 // cy	0005515 // pr	Phosphogluco	PGM2_HUMAN|PGM2|MSTP006
0006048 // UI	0005829 // cy	0004610 // ph	Phosphoacety	AGM1_HUMAN|PGM3|AGM1
0005978 // gl	0016010 // dy	0005198 // st	Phosphogluco	PGM5_HUMAN|PGM5|PGMRP
0006508 // pr	0005829 // cy	0008234 // cy	Pyroglutamyl-	PGPI_HUMAN|PGPEP1|PGPI
0050847 // pr	0005654 // nu	0004879 // RN	Progesterone	PRGR_HUMAN|PGR|NR3C3
0043149 // st	0005829 // cy	0008157 // pr	Phosphatase a	PHAR1_HUMAN|PHACTR1|KIAA1733|RPEL1
0051168 // nu	0005654 // nu	0005515 // pr	Phosphorylate	PHAX_HUMAN|PHAX|RNUXA
0030308 // ne	0005743 // m	0005515 // pr	Prohibitin	PHB_HUMAN|PHB
0070374 // pc	0005743 // m	0005515 // pr	Prohibitin-2	PHB2_HUMAN|PHB2|BAP|REA
0007275 // m	0005654 // nu	0008270 // zir	Polyhomeotic	PHC1_HUMAN|PHC1|EDR1|PH1
0007275 // m	0005654 // nu	0005515 // pr	Polyhomeotic	PHC2_HUMAN|PHC2|EDR2|PH2
0007275 // m	0005654 // nu	0008270 // zir	Polyhomeotic	PHC3_HUMAN|PHC3|EDR3|PH3
0006351 // tr	0005634 // nu	0005515 // pr	PHD finger pro	PHF1_HUMAN|PHF1|PCL1
0007399 // ne	0071564 // np	0046872 // m	PHD finger pro	PHF10_HUMAN|PHF10|BAF45A
0006351 // tr	0031965 // nu	0046872 // m	PHD finger pro	PHF11_HUMAN|PHF11|BCAP
0000278 // m	0005634 // nu	0035064 // m	PHD finger pro	PHF13_HUMAN|PHF13
0000122 // ne	0005634 // nu	0046872 // m	PHD finger pro	PHF14_HUMAN|PHF14|KIAA0783
0061087 // pc	0035098 // ES	0035064 // m	PHD finger pro	PHF19_HUMAN|PHF19|PCL3
0006355 // re	0005634 // nu	0000977 // RN	PHD finger pro	PF21B_HUMAN|PHF21B|KIAA1661
0007076 // m	0005634 // nu	0005515 // pr	PHD finger pro	PHF23_HUMAN|PHF23
0050966 // de		0005509 // ca	PHD finger pro	PHF24_HUMAN|PHF24|KIAA1045

0005980 // gly	0005829 // cy	0005515 // pr	Phosphorylase	KPB2_HUMAN	PHKA2 | PHKLA | PYK
0000086 // G2	0005829 // cy	0005515 // pr	Pleckstrin hor	PHLA1_HUMAN	PHLDA1 | PHRIP | TDAG51
0006915 // ap	0016020 // m		Pleckstrin hor	PHLA2_HUMAN	PHLDA2 | BWR1C | HLDA2 | IPL | TSSC3
0043065 // po	0070062 // ex	0043325 // ph	Pleckstrin hor	PHLA3_HUMAN	PHLDA3 | TIH1
		0019899 // en	Pleckstrin hor	PHLB3_HUMAN	PHLDB3
0007165 // sig	0005737 // cy	0005515 // pr	PH domain leu	PHLP2_HUMAN	PHLPP2 | KIAA0931 | PHLPPL
0006397 // m	0016020 // m	0019904 // pr	PHD and RING	PHRF1_HUMAN	PHRF1 | KIAA1542
0055114 // ox		0005515 // pr	Phytanoyl-CoA	PHYD1_HUMAN	PHYHD1
0007275 // m	0070062 // ex	0030414 // pe	Peptidase inhi	PI15_HUMAN	PI15 | CRISP8 | P25TI
0010466 // ne	0070062 // ex	0030414 // pe	Peptidase inhi	PI16_HUMAN	PI16 | CRISP9 | PSPBP | PSEC0164 | UNQ289/I
0098773 // sk	0005829 // cy	0004867 // se	Elafin	ELAF_HUMAN	PI3 | WAP3 | WFDC14
0046854 // ph	0005737 // cy	0004430 // 1-	Putative inact	PI4P1_HUMAN	PI4KAP1
0050776 // re	0005912 // ad		PILR alpha-ass	PIANP_HUMAN	PIANP | C12orf53 | PANP | UNQ828/PRO17
0033235 // po	0005654 // nu	0019904 // pr	E3 SUMO-prot	PIAS1_HUMAN	PIAS1 | DDXBP1
0070584 // m	0005737 // cy	0005515 // pr	PTB-containin	PCLI1_HUMAN	PID1 | NYGGF4 | PCLI1 | HMFN2073
0007165 // sig	0005794 // Go	0005515 // pr	p53-induced c	PIDD1_HUMAN	PIDD1 | LRDD | PIDD
0033625 // po	0016021 // in	0005261 // ca	Piezo-type me	PIEZ1_HUMAN	PIEZO1 | FAM38A | KIAA0233
0097502 // m	0005789 // en	0000030 // m	GPI mannosyl	PIGB_HUMAN	PIGB
0006506 // GI	0000506 // gly	0003824 // ca	Phosphatidyli	PIGC_HUMAN	PIGC | GPI2
0006506 // GI	0005783 // en	0051267 // CP	GPI ethanolan	PIGG_HUMAN	PIGG | GPI7 | UNQ1930/PRO4405
0006506 // GI	0000506 // gly	0003824 // ca	Phosphatidyli	PIGH_HUMAN	PIGH
0016254 // pr	0005789 // en	0000225 // N-	N-acetylgluco	PIGL_HUMAN	PIGL
0016254 // pr	0016021 // in	0016758 // tra	GPI mannosyl	PIGM_HUMAN	PIGM
0001580 // de	0043235 // re	0001792 // po	Polymeric imr	PIGR_HUMAN	PIGR
0016255 // at	0042765 // GI	0005515 // pr	GPI transamid	PIGS_HUMAN	PIGS | UNQ1873/PRO4316
0097502 // m	0016021 // in	0000030 // m	GPI mannosyl	PIGV_HUMAN	PIGV
0016254 // pr	0016021 // in		Phosphatidyli	PIGX_HUMAN	PIGX
0006506 // GI	0016021 // in	0000026 // alp	GPI mannosyl	PIGZ_HUMAN	PIGZ | SMP3
0016477 // ce	0005737 // cy	0005515 // pr	Phosphatidyli	P3C2B_HUMAN	PIK3C2B
0016477 // ce	0005737 // cy	0016303 // 1-	Phosphatidyli	P3C2G_HUMAN	PIK3C2G
0001952 // re	0005829 // cy	0005515 // pr	Phosphatidyli	PK3CB_HUMAN	PIK3CB | PIK3C1
0006954 // in	0005737 // cy	0016303 // 1-	Phosphatidyli	PK3CD_HUMAN	PIK3CD
0006954 // in	0042629 // m	0004674 // pr	Phosphatidyli	PK3CG_HUMAN	PIK3CG
0014067 // ne	0016021 // in	0005515 // pr	Phosphoinosit	P3IP1_HUMAN	PIK3IP1 | HGFL
0038111 // in	0005829 // cy	0005515 // pr	Phosphatidyli	P85A_HUMAN	PIK3R1 | GRB1
0006623 // pr	0005829 // cy	0005515 // pr	Phosphoinosit	PI3R4_HUMAN	PIK3R4 | VPS15
0043406 // po	0005829 // cy	0046935 // 1-	Phosphoinosit	PI3R5_HUMAN	PIK3R5
0043406 // po	0005944 // ph	0005515 // pr	Phosphoinosit	PI3R6_HUMAN	PIK3R6 | C17orf38
0007169 // tra	0005887 // in	0005515 // pr	Paired immun	PILRB_HUMAN	PILRB | FDFACT | PP1551
0008283 // ce	0005730 // nu	0004674 // pr	Serine/threon	PIM1_HUMAN	PIM1
0001934 // po	0005654 // nu	0005515 // pr	Peptidyl-proly	PIN1_HUMAN	PIN1
0043086 // ne	0005576 // ex	0004859 // ph	phospholipase	PINLY_HUMAN	PINLYP

0010506 // re	0016020 // m	0005524 // AT	Phosphatidyli	PI42C_HUMAN|PIP4K2C|PIP5K2C
0034333 // ac	0005829 // cy	0052811 // 1-	Phosphatidyli	PI51C_HUMAN|PIP5K1C|KIAA0589
0046854 // ph	0016020 // m	0005524 // AT	Phosphatidyli	PI5L1_HUMAN|PIP5KL1
0055114 // ox	0005777 // pe	0008115 // sa	Peroxisomal s	SOX_HUMAN|PIPOX|LPIPOX|PSO
0046854 // ph	0005737 // cy	0016307 // ph	Putative PIP5I	PIPSL_HUMAN|PIPSL|PSMD4P2
0007586 // di	0005737 // cy	0046872 // m	Pirin	PIR_HUMAN|PIR
0007165 // sig	0005829 // cy	0008526 // ph	Cytoplasmic p	PITC1_HUMAN|PITPNC1
0006629 // lip	0030496 // m	0008526 // ph	Membrane-as	PITM1_HUMAN|PITPNM1|DRES9|NIR2|PITPNM
0015914 // ph	0016021 // in	0008526 // ph	Membrane-as	PITM2_HUMAN|PITPNM2|KIAA1457|NIR3
0060578 // su	0005634 // nu	0005515 // pr	Pituitary hom	PITX2_HUMAN|PITX2|ARP1|RGS|RIEG|RIEG1
0045944 // po	0043025 // ne	0000978 // RN	Pituitary hom	PITX3_HUMAN|PITX3|PTX3
0010529 // ne	0043186 // P g	0004521 // en	Piwi-like prote	PIWL4_HUMAN|PIWIL4|HIWI2|PIWI
0060428 // lu	0016328 // lat	0005515 // pr	Polycystin-1	PKD1_HUMAN|PKD1
0070588 // ca	0016021 // in	0005262 // ca	Polycystic kid	PK1L2_HUMAN|PKD1L2|KIAA1879|PC1L2
0001581 // de	0005886 // pl	0008324 // ca	Polycystic kid	PK1L3_HUMAN|PKD1L3
0035725 // so	0005737 // cy	0005515 // pr	Polycystin-2	PKD2_HUMAN|PKD2|TRPP2
0060271 // cil	0072686 // m	0005515 // pr	Fibrocystin	PKHD1_HUMAN|PKHD1|FCYT|TIGM1
0006955 // im	0005829 // cy	0004872 // re	Fibrocystin-L	PKHL1_HUMAN|PKHD1L1
0042308 // ne	0005737 // cy	0004862 // cA	cAMP-depend	IPKA_HUMAN|PKIA|PRKACN1
0051973 // po	0005737 // cy	0004862 // cA	cAMP-depend	IPKB_HUMAN|PKIB|PRKACN2
0000122 // ne	0005737 // cy	0004862 // cA	cAMP-depend	IPKG_HUMAN|PKIG
0018105 // pe	0005654 // nu	0030374 // lig	Serine/threon	PKN1_HUMAN|PKN1|PAK1|PKN|PRK1|PRKCL1
0018105 // pe	0005634 // nu	0005515 // pr	Serine/threon	PKN3_HUMAN|PKN3|PKNBETA
0007155 // ce	0005886 // pl	0004871 // sig	Plakophilin-1	PKP1_HUMAN|PKP1
0007507 // he	0005634 // nu	0005515 // pr	Plakophilin-2	PKP2_HUMAN|PKP2
0032270 // po	0005576 // ex	0004623 // ph	Group 10 secr	PA2GX_HUMAN|PLA2G10
0036148 // ph	0005783 // er	0004623 // ph	Group XIIA se	PG12A_HUMAN|PLA2G12A|PLA2G12|FKSG38|UNQ2519
0046485 // et	0005783 // er	0005515 // pr	HRAS-like sup	PA216_HUMAN|PLA2G16|HRASLS3|HREV107
0019369 // ar	0005794 // Go	0004622 // lys	Cytosolic phos	PA24A_HUMAN|PLA2G4A|CPLA2|PLA2G4
0007567 // pa	0005576 // ex	0004622 // lys	Cytosolic phos	PA24B_HUMAN|PLA2G4B
0007567 // pa	0005789 // en	0004623 // ph	Cytosolic phos	PA24C_HUMAN|PLA2G4C
0036148 // ph	0005829 // cy	0004622 // lys	Cytosolic phos	PA24D_HUMAN|PLA2G4D
0036150 // ph	0005765 // lys	0004622 // lys	Cytosolic phos	PA24E_HUMAN|PLA2G4E
0036148 // ph	0005829 // cy	0004622 // lys	Cytosolic phos	PA24F_HUMAN|PLA2G4F
0006935 // ch	0016020 // m	0102568 /// 0	85/88 kDa cal	PLPL9_HUMAN|PLA2G6|PLPLA9
0090026 // po	0005737 // cy	0003847 // 1-	Platelet-activa	PAFA_HUMAN|PLA2G7|PAFAH
0090238 // po	0043235 // re	0030246 // ca	Secretory pho	PLA2R_HUMAN|PLA2R1|CLEC13C
0043085 // po	0005737 // cy	0016005 // ph	Phospholipase	PLAP_HUMAN|PLAA|PLAP
0090214 // sp	0005576 // ex	0003674 // m	Placenta-spec	PLAC1_HUMAN|PLAC1
0008284 // po	0005576 // ex	0003682 // ch	Placenta-spec	PLAC8_HUMAN|PLAC8|BM-004

	0005576 // ex	0005515 // pr	Placenta-spec	PLAC9_HUMAN|PLAC9
0006977 // DI	0005794 // Go	0000977 // RN	Zinc finger pro	PLAL1_HUMAN|PLAGL1|LOT1|ZAC
0006915 // ap	0005634 // nu	0001077 // tra	Zinc finger pro	PLAL2_HUMAN|PLAGL2|KIAA0198
0031639 // pl	0009986 // ce	0005515 // pr	Tissue-type pl	TPA_HUMAN|PLAT
0006508 // pr	0005886 // pl	0004252 // se	Urokinase-typ	UROK_HUMAN|PLAU
0007165 // sig	0005886 // pl	0019899 // en	Urokinase pla	UPAR_HUMAN|PLAUR|MO3|UPAR
2000344 // po	0005886 // pl	0102568 /// 0	Phospholipase	PLB1_HUMAN|PLB1|PLB
0036152 // ph	0005829 // cy	0004622 // lys	Phospholipase	PLBL1_HUMAN|PLBD1
0007202 // ac	0005829 // cy	0004435 // ph	1-phosphatidy	PLCB2_HUMAN|PLCB2
0007268 // ch	0005829 // cy	0004435 // ph	1-phosphatidy	PLCB3_HUMAN|PLCB3
0016042 // lip	0005829 // cy	0004435 // ph	1-phosphatidy	PLCB4_HUMAN|PLCB4
0042127 // re	0070062 // ex	0004435 // ph	1-phosphatidy	PLCD1_HUMAN|PLCD1
0060716 // lal	0005829 // cy	0004435 // ph	1-phosphatidy	PLCD3_HUMAN|PLCD3|KIAA1964
0016042 // lip	0031965 // nu	0004871 // sig	1-phosphatidy	PLCD4_HUMAN|PLCD4
0006651 // di	0005829 // cy	0004435 // ph	1-phosphatidy	PLCE1_HUMAN|PLCE1|KIAA1516|PLCE|PPLC
0000186 // ac	0005829 // cy	0005515 // pr	1-phosphatidy	PLCG1_HUMAN|PLCG1|PLC1
0050852 // T	0005829 // cy	0004435 // ph	1-phosphatidy	PLCG2_HUMAN|PLCG2
0048015 // ph	0005829 // cy	0004435 // ph	1-phosphatidy	PLCH1_HUMAN|PLCH1|KIAA1069|PLCL3
0033135 // re	0005737 // cy	0004435 // ph	Inactive phosp	PLCL1_HUMAN|PLCL1
0007214 // ga	0005737 // cy	0004435 // ph	Inactive phosp	PLCL2_HUMAN|PLCL2|KIAA1092|PLCE2
0006629 // lip	0005737 // cy	0008081 // ph	PI-PLC X doma	PLCX1_HUMAN|PLCXD1
0006816 // ca	0005829 // cy	0004435 // ph	1-phosphatidy	PLCZ1_HUMAN|PLCZ1
0006935 // ch	0016324 // ap	0005515 // pr	Phospholipase	PLD1_HUMAN|PLD1
0016042 // lip	0016021 // in	0004630 // ph	Phospholipase	PLD3_HUMAN|PLD3
0034587 // pi	0005741 // m	0042803 // pr	Mitochondria	PLD6_HUMAN|PLD6
0031581 // he	0005829 // cy	0003723 // RN	Plectin	PLEC_HUMAN|PLEC|PLEC1
0030036 // ac	0005737 // cy	0080025 // ph	Pleckstrin-2	PLEK2_HUMAN|PLEK2
0008209 // an	0005886 // pl	0005515 // pr	Pleckstrin hor	PKHA1_HUMAN|PLEKHA1|TAPP1
0006661 // ph	0005886 // pl	0005515 // pr	Pleckstrin hor	PKHA2_HUMAN|PLEKHA2|TAPP2
0006661 // ph	0005794 // Go	0070273 // ph	Pleckstrin hor	PKHA3_HUMAN|PLEKHA3|FAPP1
0006661 // ph	0005886 // pl	0032266 // ph	Pleckstrin hor	PKHA4_HUMAN|PLEKHA4|PEPP1
0006869 // lip	0005794 // Go	0017089 // gly	Pleckstrin hor	PKHA8_HUMAN|PLEKHA8|FAPP2
120009	0005737 // cy	120013	Putative prote	PKHA9_HUMAN|PLEKHA8P1|PLEKHA9|HAW1053
0007275 // m	0016021 // in	0005515 // pr	Pleckstrin hor	PKHB1_HUMAN|PLEKHB1|EVT1|KPL1|PHR1|PHRET1
0072659 // pr	0005765 // lys	0070273 // ph	Pleckstrin hor	PKHF1_HUMAN|PLEKHF1|APPD|LAPF|ZFYVE15
0043547 // po	0005634 // nu	0005089 // Rh	Pleckstrin hor	PKHG1_HUMAN|PLEKHG1|KIAA1209
0043547 // po	0005829 // cy	0005089 // Rh	Pleckstrin hor	PKHG2_HUMAN|PLEKHG2
0035023 // re		0005515 // pr	Pleckstrin hor	PKH4B_HUMAN|PLEKHG4B|KIAA1909
0043547 // po	0005902 // m	0005089 // Rh	Pleckstrin hor	PKHG6_HUMAN|PLEKHG6
0030835 // ne	0005886 // pl	0005515 // pr	Pleckstrin hor	PKHH2_HUMAN|PLEKHH2|KIAA2028
0007165 // sig	0005856 // cy		Pleckstrin hor	PKHH3_HUMAN|PLEKHH3
0007032 // en	0055037 // re		Pleckstrin hor	PKHJ1_HUMAN|PLEKHJ1|GNRPX

0043312 // ne	0005576 // ex		Pleckstrin hor	PKHO2_HUMAN|PLEKHO2|PLEKHQ1|PP9099
	0005576 // ex		Plasminogen-l	PLGA_HUMAN|PLGLA|PLGLA1|PLGP2|PRGA
	0005576 // ex		Plasminogen-l	PLGB_HUMAN|PLGLB1|PLGLB2|PLGL|PLGP1|PRGB
0014070 // re	0005783 // er		Perilipin-2	PLIN2_HUMAN|PLIN2|ADFP
	0005811 // lip		Perilipin-4	PLIN4_HUMAN|PLIN4|KIAA1881
0010890 // pc	0005737 // cy	0035473 // lip	Perilipin-5	PLIN5_HUMAN|PLIN5|LSDP5|OXPAT|PAT-1
0048167 // re	0005622 // in	0005515 // pr	Serine/threon	PLK2_HUMAN|PLK2|SNK
0000086 // G2	0005829 // cy	0042802 // id	Serine/threon	PLK4_HUMAN|PLK4|SAK|STK18
0008104 // pr	0043218 // co	0019911 // st	Plasmolipin	PLLP_HUMAN|PLLP|PMLP|TM4SF11
0019216 // re	0070062 // ex	0042577 // lip	Phospholipid	PLPP1_HUMAN|PLPP1|LPP1|PPAP2A
0006644 // ph	0005886 // pl	0005515 // pr	Phospholipid	PLPP2_HUMAN|PLPP2|LPP2|PPAP2C
0044329 // ca	0005794 // Go	0042577 // lip	Phospholipid	PLPP3_HUMAN|PLPP3|LPP3|PPAP2B
0006644 // ph	0005737 // cy	0008195 // ph	Phospholipid	PLPP5_HUMAN|PLPP5|DPPL1|HTPAP|PPAPDC1B
0006695 // ch	0016021 // in	0016787 // hy	Phospholipid	PLPP6_HUMAN|PLPP6|PPAPDC2
0006644 // ph	0005887 // in	0042577 // lip	Phospholipid	PLPR1_HUMAN|PLPPR1|LPPR1|PRG3
0006644 // ph	0005887 // in	0042577 // lip	Phospholipid	PLPR2_HUMAN|PLPPR2|LPPR2|PRG4
0006644 // ph	0005886 // pl	0042577 // lip	Phospholipid	PLPR4_HUMAN|PLPPR4|KIAA0455|LPPR4|PHP1|PRG1
1903078 // pc	0005737 // cy	0005200 // st	Plastin-1	PLSI_HUMAN|PLS1
0051639 // ac	0005737 // cy	0051015 // ac	Plastin-3	PLST_HUMAN|PLS3
0045071 // ne	0005794 // Go	0005515 // pr	Phospholipid	PLS1_HUMAN|PLSCR1
0017121 // ph	0005886 // pl	0017128 // ph	Phospholipid	PLS2_HUMAN|PLSCR2
0017121 // ph	0016021 // in	0005515 // pr	Phospholipid	PLS4_HUMAN|PLSCR4|GIG43
0034375 // hi	0005576 // ex	0005319 // lip	Phospholipid	PLTP_HUMAN|PLTP
0021510 // sp	0016021 // in	0005515 // pr	Plexin domain	PLDX1_HUMAN|PLXDC1|TEM3|TEM7
	0016021 // in	0005515 // pr	Plexin domain	PXDC2_HUMAN|PLXDC2|TEM7R|UNQ2514/PRO6003
0007275 // m	0005886 // pl	0017154 // se	Plexin-A1	PLXA1_HUMAN|PLXNA1|NOV|PLXN1
0021935 // ce	0005886 // pl	0005515 // pr	Plexin-A2	PLXA2_HUMAN|PLXNA2|KIAA0463|OCT|PLXN2|UNQ20
0016477 // ce	0005886 // pl	0005515 // pr	Plexin-B1	PLXB1_HUMAN|PLXNB1|KIAA0407|PLXN5|SEP
0071526 // se	0070062 // ex	0005515 // pr	Plexin-B2	PLXB2_HUMAN|PLXNB2|KIAA0315
0007162 // ne	0016021 // in	0019904 // pr	Plexin-B3	PLXB3_HUMAN|PLXNB3|KIAA1206|PLXN6
0007155 // ce	0016021 // in	0005515 // pr	Plexin-C1	PLXC1_HUMAN|PLXNC1|VESPR
0097009 // er	0070062 // ex	0016829 // ly	N-fatty-acyl-a	P20D1_HUMAN|PM20D1
0032268 // re	0070062 // ex	0016805 // di	Peptidase M2	P20D2_HUMAN|PM20D2|ACY1L2
0071456 // ce	0005739 // m	0005515 // pr	Phorbol-12-m	APR_HUMAN|PMAIP1|NOXA
0032438 // m	0005576 // ex	0005515 // pr	Melanocyte p	PMEL_HUMAN|PMEL|D12S53E|PMEL17|SILV
0051301 // ce	0005794 // Go	0005515 // pr	Polyamine-mo	PMF1_HUMAN|PMF1
	0005794 // Go		HCG2044777	U3KQ54_HUMAN|PMF1-BGLAP|hCG_2044777
0008150 // bi	0005737 // cy	0003674 // m	Polyamine-mo	PMFBP_HUMAN|PMFBP1
0045047 // pr	0005829 // cy	0004615 // ph	Phosphomanr	PMM2_HUMAN|PMM2
0008219 // ce	0016021 // in	0005515 // pr	Peripheral my	PMP22_HUMAN|PMP22|GAS3
0016446 // so	0032389 // M	0003697 // sir	Putative postr	PM2P2_HUMAN|PMS2P2|PMS2L14|PMS2L2|PMS4

0016310 // ph	0005777 // pe	0005524 // AT	Phosphomeva	PMVK_HUMAN|PMVK|PMKI
0018107 // pe	0005622 // in	0004683 // ca	Calcium/calm	KCC1B_HUMAN|PNCK
	0005829 // cy	0003723 // RN	Arginine/serin	PNISR_HUMAN|PNISR|C6orf111|SFRS18|SRRP130|HSPC
0000289 // nu	0016021 // in	0046872 // m	Poly(A)-specif	PNDC1_HUMAN|PNLDC1
0043065 // pc	0005737 // cy	0005515 // pr	Paraneoplasti	PNMA1_HUMAN|PNMA1|MA1
0000398 // m	0035145 // ex	0003723 // RN	Pinin	PININ_HUMAN|PNN|DRS|MEMA
0006364 // rR	0005730 // nu	0003723 // RN	RNA-binding p	PNO1_HUMAN|PNO1
0042493 // re	0005737 // cy	0008144 // dr	Purine nucleo	PNPH_HUMAN|PNP|NP
0019915 // lip	0005789 // er	0004465 // lip	Patatin-like ph	PLPL2_HUMAN|PNPLA2|ATGL|FP17548
0019433 // tri	0005829 // cy	0050253 // re	Patatin-like ph	PLPL4_HUMAN|PNPLA4|DXS1283E|GS2
0006631 // fa	0016020 // m	0004623 // ph	Calcium-indep	PLPL8_HUMAN|PNPLA8|IPLA22|IPLA2G|BM-043
0019221 // cy	0005737 // cy	0004860 // pr	Podocan-like p	PONL1_HUMAN|PODNL1|SLRR5B
0016477 // ce	0044297 // ce	0005515 // pr	Podocalyxin	PODXL_HUMAN|PODXL|PCLP|PCLP1
0003382 // ep	0005923 // bi	0051015 // ac	Protein POF1E	POF1B_HUMAN|POF1B
0009790 // en	0005783 // er	0046922 // pe	GDP-fucose pr	OFUT1_HUMAN|POFUT1|FUT12|KIAA0180
0036066 // pr	0005794 // Go	0046922 // pe	GDP-fucose pr	OFUT2_HUMAN|POFUT2|C21orf80|FUT13|KIAA0958
0060537 // m	0005788 // en	0035251 // UI	Protein O-gluc	PGLT1_HUMAN|POGLUT1|C3orf9|CLP46|KTELC1|MDSR
0051301 // ce	0005829 // cy	0005515 // pr	Pogo transpos	POGZ_HUMAN|POGZ|KIAA0461|SUHW5|ZNF280E|ZNF6
0006261 // DI	0005654 // nu	0005515 // pr	DNA polymera	DPOD4_HUMAN|POLD4|POLDS
0045931 // pc	0042645 // m	0005515 // pr	Polymerase de	PDIP2_HUMAN|POLDIP2|PDIP38|POLD4|HSPC017
0006270 // DI	0005634 // nu	0005515 // pr	DNA polymera	DPOE3_HUMAN|POLE3|CHRAC17
0006260 // DI	0005654 // nu	0005515 // pr	DNA polymera	POLI_HUMAN|POLI|RAD30B
0006310 // DI	0005654 // nu	0005515 // pr	DNA-directed	DPOLM_HUMAN|POLM|polmu
0036297 // in	0005654 // nu	0003887 // DI	DNA polymera	DPOLN_HUMAN|POLN
0006361 // tra	0005654 // nu	0003677 // DI	DNA-directed	RPA2_HUMAN|POLR1B
0001189 // RN	0005654 // nu	0003677 // DI	DNA-directed	RPA49_HUMAN|POLR1E|PAF53|PRAF1
0006362 // tra	0005654 // nu	0001055 // RN	DNA-directed	RPAB2_HUMAN|POLR2F|POLRF
0016070 // RN	0005654 // nu	0003899 // DI	DNA-directed	RPB9_HUMAN|POLR2I
0006366 // tra	0005665 // DI	0001055 // RN	DNA-directed	RPB1C_HUMAN|POLR2J3
0006362 // tra	0005654 // nu	0005515 // pr	DNA-directed	RPAB5_HUMAN|POLR2L
0045087 // in	0005654 // nu	0003899 // DI	DNA-directed	RPC1_HUMAN|POLR3A
0032481 // pc	0005654 // nu	0001056 // RN	DNA-directed	RPC2_HUMAN|POLR3B
0032481 // pc	0005654 // nu	0003899 // DI	DNA-directed	RPC3_HUMAN|POLR3C
0032481 // pc	0005634 // nu	0003899 // DI	DNA-directed	RPC7_HUMAN|POLR3G
0006384 // tra	0005654 // nu	0003899 // DI	DNA-directed	RPC8_HUMAN|POLR3H|KIAA1665|RPC8
0032481 // pc	0005654 // nu	0001056 // RN	DNA-directed	RPC10_HUMAN|POLR3K|RPC11|My010
0006390 // tra	0042645 // m	0003723 // RN	DNA-directed	RPOM_HUMAN|POLRMT
0006405 // RN	0044615 // nu	0008139 // nu	Putative nucle	P121B_HUMAN|POM121B
0006493 // pr	0016021 // in	0008375 // ac	Protein O-link	PMGT2_HUMAN|POMGNT2|AGO61|C3orf39|EOGTL|GT
1904100 // pc	0016021 // in	0004169 // dc	Protein O-mar	POMT2_HUMAN|POMT2
0016311 // de	0005576 // ex	0004064 // ar	Serum paraox	PON3_HUMAN|PON3
0001682 // tR	0005654 // nu	0005515 // pr	Ribonuclease	RPP29_HUMAN|POP4|RPP29

0001682 // tR	0005655 // nu	0005515 // pr	Ribonuclease	POP5_HUMAN|POP5|AD-008|HSPC004|x0003
0042391 // re	0016021 // in	0030552 // cA	Popeye doma	POPD3_HUMAN|POPDC3|POP3
0060070 // ca	0032281 // AM	1990698 // pa	Protein-serine	PORCN_HUMAN|PORCN|MG61|PORC|PPN
0014850 // re	0031012 // ex	0008201 // he	Periostin	POSTN_HUMAN|POSTN|OSF2
0045944 // pc	0005654 // nu	0005515 // pr	POU domain,	PO2F1_HUMAN|POU2F1|OCT1|OTF1
0006366 // tra	0005634 // nu	0000978 // RN	POU domain,	PO2F3_HUMAN|POU2F3|OTF11|PLA1
0022011 // m	0005667 // tra	0005515 // pr	POU domain,	PO3F2_HUMAN|POU3F2|BRN2|OCT7|OTF7
0021879 // fo	0005634 // nu	0003700 // tra	POU domain,	PO3F4_HUMAN|POU3F4|BRN4|OTF9
0043065 // pc	0043005 // ne	0000981 // RN	POU domain,	PO4F1_HUMAN|POU4F1|BRN3A|RDC1
0010468 // re	0005737 // cy	0003723 // RN	POU domain,	PO5F1_HUMAN|POU5F1|OCT3|OCT4|OTF3
0006351 // tra	0005739 // m	0003700 // tra	Putative POU	P5F1B_HUMAN|POU5F1B|OCT4PG1|OTF3C|OTF3P1|PC
0007507 // he	0016604 // nu	0001227 // tra	POU domain,	PO6F1_HUMAN|POU6F1|BRN5|MPOU|TCFB1
0006355 // re	0005634 // nu	0003677 // DI	POU domain,	PO6F2_HUMAN|POU6F2|RPF1
0071344 // di	0005737 // cy	0004427 // in	Inorganic pyro	IPYR_HUMAN|PPA1|IOPPP|PP
0071344 // di	0070062 // ex	0004427 // in	Inorganic pyro	IPYR2_HUMAN|PPA2|HSPC124
0000027 // rik	0005730 // nu	0003723 // RN	Suppressor of	SSF1_HUMAN|PPAN|BXDC3|SSF1
0010745 // ne	0005634 // nu	0032403 // pr	Peroxisome p	PPARA_HUMAN|PPARA|NR1C1|PPAR
0043401 // ste	0043234 // pr	0008144 // dr	Peroxisome p	PPARG_HUMAN|PPARG|NR1C3
0031017 // ex			Pancreatic pro	PPDPF_HUMAN|PPDPF|C20orf149|EXPDF
0051497 // ne	0005829 // cy	0005515 // pr	Liprin-alpha-1	LIPA1_HUMAN|PPFIA1|LIP1
0007269 // ne	0005829 // cy	0005515 // pr	Liprin-alpha-2	LIPA2_HUMAN|PPFIA2
0007269 // ne	0005829 // cy	0005515 // pr	Liprin-alpha-3	LIPA3_HUMAN|PPFIA3|KIAA0654
0007269 // ne	0005829 // cy	0005515 // pr	Liprin-alpha-4	LIPA4_HUMAN|PPFIA4|KIAA0897
0007155 // ce	0005829 // cy	0005515 // pr	Liprin-beta-1	LIPB1_HUMAN|PPFIBP1|KIAA1230
0006457 // pr	0070062 // ex	0005515 // pr	Peptidyl-proly	PPIC_HUMAN|PPIC|CYPC
0008637 // ap	0005739 // m	0016018 // cy	Peptidyl-proly	PPIF_HUMAN|PPIF|CYP3
1901407 // re	0005829 // cy	0003723 // RN	Peptidyl-proly	PPIL4_HUMAN|PPIL4
0000413 // pr		0003755 // pe	Peptidyl-proly	PPIL6_HUMAN|PPIL6
0007010 // cy	0005829 // cy	0005515 // pr	Periplakin	PEPL_HUMAN|PPL|KIAA0568
0030512 // ne	0005829 // cy	0033192 // ca	Protein phosp	PPM1A_HUMAN|PPM1A|PPPM1A
0000086 // G2	0005634 // nu	0005515 // pr	Protein phosp	PPM1D_HUMAN|PPM1D|WIP1
0016576 // hi	0005829 // cy	0005515 // pr	Protein phosp	PPM1F_HUMAN|PPM1F|KIAA0015|POPX2
0006470 // pr	0005737 // cy	0004721 // ph	Protein phosp	PPM1H_HUMAN|PPM1H|ARHCL1|KIAA1157|URCC2
0006470 // pr		0004722 // pr	Protein phosp	PPM1J_HUMAN|PPM1J|PPP2CZ
0006470 // pr	0005739 // m	0004722 // pr	Protein phosp	PPM1K_HUMAN|PPM1K|PP2CM
0007178 // tra	0005789 // er	0004722 // pr	Protein phosp	PPM1L_HUMAN|PPM1L|PP2CE
0006470 // pr	0005634 // nu	0008420 // CT	Protein phosp	PPM1M_HUMAN|PPM1M|PPM1E
0016311 // de	0005737 // cy	0005515 // pr	Serine/threon	PP1A_HUMAN|PPP1CA|PPP1A
0043086 // ne	0072357 // PT	0003723 // RN	Serine/threon	PP1RA_HUMAN|PPP1R10|CAT53|FB19|PNUTS
0000086 // G2	0005829 // cy	0008047 // en	Protein phosp	MYPT2_HUMAN|PPP1R12B|MYPT2
0045786 // ne	0005739 // m	0005515 // pr	Apoptosis-stir	ASPP1_HUMAN|PPP1R13B|ASPP1|KIAA0771

0003229 // ve	0005654 // nu	0005515 // pr	RelA-associate	IASPP_HUMAN	PPP1R13L|IASPP|NKIP1|PPP1R13BL|RAI
0045087 // in	0005737 // cy	0004864 // pr	Protein phosp	PP14B_HUMAN	PPP1R14B|PLCB3N|PNG
0042325 // re	0016020 // m	0004865 // pr	Protein phosp	PP14C_HUMAN	PPP1R14C|KEPI
1903898 // ne	0005739 // m	0008157 // pr	Protein phosp	PR15A_HUMAN	PPP1R15A|GADD34
	0005737 // cy	0005515 // pr	Phostensin	PPR18_HUMAN	PPP1R18|HKMT1098|KIAA1949
0006469 // ne	0005615 // ex	0004865 // pr	Protein phosp	PPR1A_HUMAN	PPP1R1A|IPP1
0006469 // ne	0005737 // cy	0004865 // pr	Protein phosp	PPR1C_HUMAN	PPP1R1C
		0005515 // pr	Protein phosp	PPR32_HUMAN	PPP1R32|C11orf66
0010923 // ne		0019902 // ph	Protein phosp	PPR35_HUMAN	PPP1R35|C7orf47
0010923 // ne		0019902 // ph	Protein phosp	PPR36_HUMAN	PPP1R36|C14orf50
0005979 // re	0042587 // gl	0004721 // ph	Protein phosp	PPR3B_HUMAN	PPP1R3B|PPP1R4
0005978 // gl	0005829 // cy	0005515 // pr	Protein phosp	PPR3C_HUMAN	PPP1R3C|PPP1R5
2000465 // re	0016021 // in	2001069 // gl	Protein phosp	PPR3F_HUMAN	PPP1R3F
0019722 // ca	0030864 // co	0005515 // pr	Neurabin-1	NEB1_HUMAN	PPP1R9A|KIAA1222
0010033 // re	0005829 // cy	0005515 // pr	Serine/threon	PP2AA_HUMAN	PPP2CA
0008637 // ap	0005829 // cy	0008022 // pr	Serine/threon	PP2AB_HUMAN	PPP2CB
0000086 // G2	0000159 // pr	0005515 // pr	Serine/threon	2ABA_HUMAN	PPP2R2A
0061053 // so	0000159 // pr	0005515 // pr	Serine/threon	P2R3A_HUMAN	PPP2R3A|PPP2R3
0050790 // re	0005654 // nu	0005515 // pr	Serine/threon	P2R3B_HUMAN	PPP2R3B|PPP2R3L
0090219 // ne	0005737 // cy	0005515 // pr	Serine/threon	2A5A_HUMAN	PPP2R5A
0031334 // po	0005829 // cy	0005515 // pr	Serine/threon	2A5B_HUMAN	PPP2R5B
0007165 // sig	0005634 // nu	0005515 // pr	Serine/threon	2A5G_HUMAN	PPP2R5C|KIAA0044
0050790 // re	0000159 // pr	0005515 // pr	Serine/threon	2A5E_HUMAN	PPP2R5E
	0005737 // cy	0005515 // pr	Serine/threon	P4R3A_HUMAN	PPP4R3A|KIAA2010|PP4R3A|SMEK1|M
0080163 // re	0008287 // pr	0005515 // pr	Serine/threon	PP4R4_HUMAN	PPP4R4|KIAA1622|PP4R4
0048208 // CO	0005829 // cy	0005515 // pr	Serine/threon	PP6R1_HUMAN	PPP6R1|KIAA1115|PP6R1|SAPS1
0048208 // CO	0005829 // cy	0005515 // pr	Serine/threon	PP6R3_HUMAN	PPP6R3|C11orf23|KIAA1558|PP6R3|SA
0006351 // tra	0005654 // nu	0003723 // RN	Peroxisome p	PPRC1_HUMAN	PPRC1|KIAA0595
	0016021 // in		PQ-loop repea	PQLC1_HUMAN	PQLC1
0055085 // tra	0043231 // in	0015174 // ba	Lysosomal am	LAAT1_HUMAN	PQLC2
0043066 // ne	0005886 // pl	0005515 // pr	Melanoma an	PRAME_HUMAN	PRAME|MAPE|OIP4
0008150 // bi	0005576 // ex	0003674 // m	Basic salivary	PRP1_HUMAN	PRB1
0008150 // bi	0005576 // ex	0003674 // m	Basic salivary	PRB2_HUMAN	PRB2
0050829 // de	0005576 // ex		Basic salivary	PRB3_HUMAN	PRB3
0050896 // re	0005737 // cy		Progressive ro	PRCD_HUMAN	PRCD
2000377 // re	0005886 // pl	0008239 // di	Lysosomal Pro	PCP_HUMAN	PRCP|PCP
0033082 // re	0005654 // nu	0000978 // RN	PR domain zin	PRDM1_HUMAN	PRDM1|BLIMP1
0022008 // ne	0005634 // nu	0003682 // ch	PR domain zin	PRD13_HUMAN	PRDM13|PFM10
0045892 // ne	0016604 // nu	0000978 // RN	PR domain zin	PRDM5_HUMAN	PRDM5|PFM2
0034968 // his	0005634 // nu	0018024 // his	Putative histo	PRDM6_HUMAN	PRDM6|PFM3
0032259 // m	0005634 // nu	0003677 // DN	PR domain zin	PRDM8_HUMAN	PRDM8|PFM5
0006979 // re	0005737 // cy	0008379 // th	Peroxiredoxin	PRDX2_HUMAN	PRDX2|NKEFB|TDPX1
0006915 // ap	0031410 // cy	0005102 // re	Peroxiredoxin	PRDX5_HUMAN	PRDX5|ACR1|SBBI10

0051881 // re	0005739 // m	0005515 // pr	PRELI domain	PRLD1_HUMAN|PRELID1|PRELI|CGI-106|SBBI12
0015914 // ph	0005758 // m	1990050 // ph	PRELI domain	PLD3B_HUMAN|PRELID3B|C20orf45|SLMO2|CGI-107
0006469 // ne	0030426 // gr	0005515 // pr	Phosphatidyli	PREX1_HUMAN|PREX1|KIAA1415
0006606 // pr	0031965 // nu	0005515 // pr	Prickle-like pr	PRIC1_HUMAN|PRICKLE1|RILP
0008150 // bi	0005575 // ce	0005515 // pr	Prickle-like pr	PRIC3_HUMAN|PRICKLE3|LMO6
0008150 // bi	0005634 // nu	0003674 // m	Prickle-like pr	PRIC4_HUMAN|PRICKLE4|C6orf49|OEBT
0019985 // tr	0005634 // nu	0003896 // DI	DNA-directed	PRIPO_HUMAN|PRIMPOL|CCDC111
0016236 // m	0005829 // cy	0005515 // pr	5'-AMP-activa	AAPK1_HUMAN|PRKAA1|AMPK1
0016236 // m	0005829 // cy	0005515 // pr	5'-AMP-activa	AAPK2_HUMAN|PRKAA2|AMPK|AMPK2
0007050 // ce	0005829 // cy	0005515 // pr	5'-AMP-activa	AAKB1_HUMAN|PRKAB1|AMPK
0007050 // ce	0005829 // cy	0005515 // pr	5'-AMP-activa	AAKB2_HUMAN|PRKAB2
0045860 // pc	0005829 // cy	0008607 // ph	5'-AMP-activa	AAKG2_HUMAN|PRKAG2
0007050 // ce	0005829 // cy	0019901 // pr	5'-AMP-activa	AAKG3_HUMAN|PRKAG3|AMPKG3
0003091 // re	0005737 // cy	0034236 // pr	cAMP-depenc	KAP2_HUMAN|PRKAR2A|PKR2|PRKAR2
0007077 // m	0005737 // cy	0005515 // pr	Protein kinase	KPCA_HUMAN|PRKCA|PKCA|PRKACA
0034351 // ne	0005737 // cy	0004697 // pr	Protein kinase	KPCD_HUMAN|PRKCD
0018105 // pe	0030425 // de	0004697 // pr	Protein kinase	KPCG_HUMAN|PRKCG|PKCG
0018105 // pe	0005737 // cy	0017160 // Ra	Protein kinase	KPCL_HUMAN|PRKCH|PKCL|PRKCL
0006612 // pr	0000139 // Gc	0004697 // pr	Protein kinase	KPCI_HUMAN|PRKCI|DXS1179E
0035924 // ce	0000421 // au	0016301 // ki	Serine/threon	KPCD1_HUMAN|PRKD1|PKD|PKD1|PRKCM
0033138 // pc	0005829 // cy	0004672 // pr	Serine/threon	KPCD2_HUMAN|PRKD2|PKD2|HSPC187
0043087 // re	0005829 // cy	0005246 // ca	cGMP-depenc	KGP1_HUMAN|PRKG1|PRKG1B|PRKGR1A|PRKGR1B
2001226 // ne	0005829 // cy	0030553 // cG	cGMP-depenc	KGP2_HUMAN|PRKG2|PRKGR2
0016571 // hi	0005634 // nu	0042974 // re	Protein argini	ANM2_HUMAN|PRMT2|HMT1|HRMT1L1
0097421 // liv	0005654 // nu	0005515 // pr	Protein argini	ANM5_HUMAN|PRMT5|HRMT1L5|IBP72|JBP1|SKB1
0016571 // hi	0005654 // nu	0044020 // hi	Protein argini	ANM6_HUMAN|PRMT6|HRMT1L6
0018216 // pe	0005829 // cy	0046982 // pr	Protein argini	ANM8_HUMAN|PRMT8|HRMT1L3|HRMT1L4
0050819 // ne	0005576 // ex	0005515 // pr	Vitamin K-dep	PROC_HUMAN|PROC
0006644 // ph		0004623 // ph	Protein PROC	PRCA1_HUMAN|PROCA1
0007596 // bl	0005576 // ex	0004872 // re	Endothelial pr	EPCR_HUMAN|PROCR|EPCR
0006562 // pr	0005739 // m	0004657 // pr	Proline dehyd	PROD_HUMAN|PRODH|PIG6|POX2|PRODH2
0043087 // re	0005887 // in	0015485 // ch	Prominin-2	PROM2_HUMAN|PROM2|PROML2|UNQ2521/PRO6014
0006450 // re		0002161 // ar	Putative proly	PRXD1_HUMAN|PRORSD1P|NCRNA00117|PRDXDD1P
0002576 // pl	0000139 // Gc	0005509 // ca	Vitamin K-dep	PROS_HUMAN|PROS1|PROS
		0005515 // pr	Proline and se	PRSR2_HUMAN|PROSER2|C10orf47
0001889 // liv	0005634 // nu	0016922 // lig	Prospero hom	PROX1_HUMAN|PROX1
0000122 // ne	0005634 // nu	0003677 // DI	Prospero hom	PROX2_HUMAN|PROX2
0006888 // EF	0005788 // er	0005509 // ca	Vitamin K-dep	PROZ_HUMAN|PROZ
0000398 // m	0005685 // U1	0003723 // RI	Pre-mRNA-pr	PR40B_HUMAN|PRPF40B|HYPC
0000398 // m	0005654 // nu	0003723 // RI	Pre-mRNA-pr	PRP6_HUMAN|PRPF6|C20orf14
0007155 // ce	0016021 // in		Peripherin-2	PRPH2_HUMAN|PRPH2|PRPH|RDS|TSPAN22

0043086 // ne		0004857 // er	Phosphoribos	KPRA_HUMAN|PRPSAP1
		0005515 // pr	Proline-rich pı	PRR14_HUMAN|PRR14
0007275 // m			Proline-rich pı	PRR15_HUMAN|PRR15
0045793 // pc		0005515 // pr	Protein Larger	LARGN_HUMAN|PRR16
		0005515 // pr	Proline-rich pı	PRR19_HUMAN|PRR19
		0005515 // pr	Proline-rich pı	PR20C_HUMAN|PRR20C
		0003723 // RN	Proline-rich pı	PRR3_HUMAN|PRR3|CAT56
0001934 // pc	0031932 // TC		Proline-rich pı	PRR5_HUMAN|PRR5|PROTOR1|PP610
0007165 // sig	0005737 // cy		PRR5-ARHGAI	B1AHC4_HUMAN|PRR5-ARHGAP8
0001933 // ne	0031932 // TC	0005515 // pr	Proline-rich pı	PRR5L_HUMAN|PRR5L|PROTOR2
	0016021 // int		Proline-rich pı	PRR7_HUMAN|PRR7
	0005794 // Go	0005515 // pr	Protein PRRC1	PRRC1_HUMAN|PRRC1
0030154 // ce		0003723 // RN	Protein PRRC2	PRC2B_HUMAN|PRRC2B|BAT2L|BAT2L1|KIAA0515
	0005576 // ex	0005509 // ca	Transmembra	TMG1_HUMAN|PRRG1|PRGP1|TMG1
0006508 // pr	0005615 // ex	0005515 // pr	Transmembra	TMG2_HUMAN|PRRG2|PRGP2|TMG2
0008150 // bi	0016021 // int	0005509 // ca	Transmembra	TMG4_HUMAN|PRRG4|PRGP4|TMG4
0050884 // ne	0016021 // int	0017124 // SH	Proline-rich tr	PRRT2_HUMAN|PRRT2
	0016021 // int		Proline-rich tr	PRRT4_HUMAN|PRRT4
0006508 // pr	0005576 // ex	0004252 // se	Trypsin-1	TRY1_HUMAN|PRSS1|TRP1|TRY1|TRYP1
0006887 // ex	0043195 // te	0005044 // sc	Neurotrypsin	NETR_HUMAN|PRSS12
0006508 // pr	0005764 // lys	0008236 // se	Thymus-speci	TSSP_HUMAN|PRSS16|TSSP
0030307 // pc	0005576 // ex	0004252 // se	Trypsin-2	TRY2_HUMAN|PRSS2|TRY2|TRYP2
0006508 // pr	0016020 // m	0004252 // se	Testisin	TEST_HUMAN|PRSS21|ESP1|TEST1|UNQ266/PRO303
0006508 // pr	0019897 // ex	0004252 // se	Brain-specific	BSSP4_HUMAN|PRSS22|BSSP4|PRSS26|SP001LA|UNQ3
0006508 // pr	0005788 // er	0004252 // se	Serine proteas	PRS23_HUMAN|PRSS23|ZSIG13|UNQ270/PRO307
0007586 // di	0005576 // ex	0005515 // pr	Trypsin-3	TRY3_HUMAN|PRSS3|PRSS4|TRY3|TRY4
	0005739 // m		Inactive serine	PRS35_HUMAN|PRSS35|C6orf158|UNQ522/PRO1057
0006508 // pr	0005578 // pr	0004252 // se	Polyserase-2	POLS2_HUMAN|PRSS36
0006508 // pr	0005576 // ex	0004252 // se	Serine proteas	PRS38_HUMAN|PRSS38|MPN2
0006508 // pr	0005886 // pl	0004252 // se	Serine proteas	PRS41_HUMAN|PRSS41|TESSP1
0006508 // pr	0005737 // cy	0004252 // se	Serine proteas	PRS42_HUMAN|PRSS42|TESSP2
0006508 // pr		0004252 // se	Serine proteas	PRS45_HUMAN|PRSS45|TESSP5
0006508 // pr	0005576 // ex	0004252 // se	Serine proteas	PRS51_HUMAN|PRSS51
0006508 // pr	0005737 // cy	0004252 // se	Serine proteas	PRS53_HUMAN|PRSS53
0006508 // pr	0005886 // pl	0004252 // se	Prostasin	PRSS8_HUMAN|PRSS8
0006178 // gu	0005737 // cy	0005515 // pr	Phosphoribos	PRDC1_HUMAN|PRTFDC1|HHGP
0007275 // m	0016021 // int		Protogenin	PRTG_HUMAN|PRTG
0050767 // re	0005829 // cy	0005515 // pr	Exopolyphosp	PRUN1_HUMAN|PRUNE1|PRUNE

0006915 // ap	0005737 // cy	0016462 // py	Protein prune	PRUN2_HUMAN|PRUNE2|BMCC1|BNIPXL|C9orf65|KIAA
0008366 // ax	0005737 // cy	0005515 // pr	Periaxin	PRAX_HUMAN|PRX|KIAA1620
0070373 // ne	0031225 // ar	0033130 // ac	Prostate stem	PSCA_HUMAN|PSCA|UNQ206/PRO232
0032012 // re	0098999 // ex	0005086 // AF	PH and SEC7 c	PSD1_HUMAN|PSD|EFA6|EFA6A|KIAA2011|PSD1|TYL
0032012 // re	0016021 // in	0005086 // AF	PH and SEC7 c	PSD2_HUMAN|PSD2|EFA6C
0032012 // re	0014069 // pc	0005086 // AF	PH and SEC7 c	PSD3_HUMAN|PSD3|EFA6D|EFA6R|HCA67|KIAA0942
0032012 // re	0032154 // cl	0005086 // AF	PH and SEC7 c	PSD4_HUMAN|PSD4|EFA6B|TIC
0007565 // fe	0005576 // ex	0005515 // pr	Pregnancy-sp	PSG5_HUMAN|PSG5
0007565 // fe	0005576 // ex		Pregnancy-sp	PSG9_HUMAN|PSG9|PSG11
0018107 // pe	0005789 // er	0005524 // AT	Serine/threon	KPSH1_HUMAN|PSKH1
0051437 // pc	0005829 // cy	0005515 // pr	Proteasome s	PSA1_HUMAN|PSMA1|HC2|NU|PROS30|PSC2
0051437 // pc	0034774 // se	0005515 // pr	Proteasome s	PSA5_HUMAN|PSMA5
0051437 // pc	0005829 // cy	0003723 // RN	Proteasome s	PSA6_HUMAN|PSMA6|PROS27
0051437 // pc	0005829 // cy	0005515 // pr	Proteasome s	PSA7_HUMAN|PSMA7|HSPC
0051437 // pc	0005829 // cy	0004298 // th	Proteasome s	PSA7L_HUMAN|PSMA8|PSMA7L
0051437 // pc	0005829 // cy	0005515 // pr	Proteasome s	PSB5_HUMAN|PSMB5|LMPX|MB1|X
0051437 // pc	0005829 // cy	0005515 // pr	Proteasome s	PSB8_HUMAN|PSMB8|LMP7|PSMB5i|RING10|Y2
0051437 // pc	0005829 // cy	0005515 // pr	Proteasome s	PSB9_HUMAN|PSMB9|LMP2|PSMB6i|RING12
0051437 // pc	0005829 // cy	0005515 // pr	26S proteasor	PRS4_HUMAN|PSMC1
0051437 // pc	0034774 // se	0005515 // pr	26S proteasor	PRS6A_HUMAN|PSMC3|TBP1
0007131 // re	0005575 // ce	0003677 // DI	Homologous-p	HOP2_HUMAN|PSMC3IP|HOP2|TBPIP
0051437 // pc	0005829 // cy	0005515 // pr	26S proteasor	PRS6B_HUMAN|PSMC4|MIP224|TBP7
0051437 // pc	0005829 // cy	0005515 // pr	26S proteasor	PRS8_HUMAN|PSMC5|SUG1
0051437 // pc	0005829 // cy	0005515 // pr	26S proteasor	PRS10_HUMAN|PSMC6|SUG2
0051437 // pc	0034774 // se	0005515 // pr	26S proteasor	PSD13_HUMAN|PSMD13
0051437 // pc	0034774 // se	0005515 // pr	26S proteasor	PSMD2_HUMAN|PSMD2|TRAP2
0051437 // pc	0034774 // se	0005515 // pr	26S proteasor	PSMD6_HUMAN|PSMD6|KIAA0107|PFAAP4
0051437 // pc	0034774 // se	0005515 // pr	26S proteasor	PSMD7_HUMAN|PSMD7|MOV34L
0051437 // pc	0005829 // cy	0005515 // pr	26S proteasor	PSMD9_HUMAN|PSMD9
0043248 // pr	0000502 // pr	0005515 // pr	Proteasome a	PSMG2_HUMAN|PSMG2|HCCA3|PAC2|TNFSF5IP1
0008150 // bi	0005576 // ex	0003674 // m	Psoriasis susc	PS1C2_HUMAN|PSORS1C2|C6orf17|SPR1
0043547 // pc	0005576 // ex	0008083 // gr	Persephin	PSPN_HUMAN|PSPN
1904306 // pc	0005886 // pl	0005543 // ph	Platelet-activa	PTAFR_HUMAN|PTAFR|PAFR
0018342 // pr	0005737 // cy	0008318 // pr	Protein preny	PTAR1_HUMAN|PTAR1
0009653 // ar	0005634 // nu	0003723 // RN	Polypyrimidin	PTBP3_HUMAN|PTBP3|ROD1
0042780 // tR	0005739 // m	0003723 // RN	Pentatricopep	PTCD1_HUMAN|PTCD1|KIAA0632
0042633 // ha	0016021 // in	0097108 // he	Protein patch	PTC2_HUMAN|PTCH2|UNQ560/PRO1121/PRO57079
0008150 // bi	0016021 // in	0003674 // m	Patched doma	PTHD4_HUMAN|PTCHD4|C6orf138|PTCH53
0006659 // ph	0016021 // in	0016740 // tra	Phosphatidyls	PTSS1_HUMAN|PTDSS1|KIAA0024|PSSA
0006659 // ph	0016021 // in	0016740 // tra	Phosphatidyls	PTSS2_HUMAN|PTDSS2|PSS2

0030855 // ep	0070062 // ex	0003674 // m	Phosphotriest	PTER_HUMAN|PTER
0007186 // G-	0005887 // in	0004957 // pr	Prostaglandin	PE2R1_HUMAN|PTGER1
0032570 // re	0005886 // pl	0004957 // pr	Prostaglandin	PE2R2_HUMAN|PTGER2
0033624 // ne	0005622 // in	0004957 // pr	Prostaglandin	PE2R4_HUMAN|PTGER4|PTGER2
0019371 // cy	0016020 // m	0050220 // pr	Prostaglandin	PTGES_HUMAN|PTGES|MGST1L1|MPGES1|PGES|PIG12
0071799 // ce	0005886 // pl	0004958 // pr	Prostaglandin	PF2R_HUMAN|PTGFR
0034389 // lip	0005789 // er	0005515 // pr	Prostaglandin	FPRP_HUMAN|PTGFRN|CD9P1|EWIF|FPRP|KIAA1436
0098869 // ce	0005789 // er	0020037 // he	Prostaglandin	PGH1_HUMAN|PTGS1|COX1
0046697 // de	0043234 // pr	0019899 // er	Prostaglandin	PGH2_HUMAN|PTGS2|COX2
0007189 // ad	0005886 // pl	0004991 // pa	Parathyroid h	PTH1R_HUMAN|PTH1R|PTHR|PTHR1
0007186 // G-	0005886 // pl	0004991 // pa	Parathyroid h	PTH2R_HUMAN|PTH2R|PTHR2
1900024 // re	0005829 // cy	0005515 // pr	Focal adhesio	FAK1_HUMAN|PTK2|FAK|FAK1
0038083 // pe	0005925 // fo	0005515 // pr	Protein-tyrosi	FAK2_HUMAN|PTK2B|FAK2|PYK2|RAFTK
0038083 // pe	0005654 // nu	0004713 // pr	Protein-tyrosi	PTK6_HUMAN|PTK6|BRK
0001736 // es	0005887 // in	0004672 // pr	Inactive tyrosi	PTK7_HUMAN|PTK7|CCK4
0002376 // im	0005634 // nu		Parathymosin	PTMS_HUMAN|PTMS
0044849 // es	0005604 // ba	0035374 // ch	Pleiotrophin	PTN_HUMAN|PTN|HBNF1|NEGF1
0035335 // pe	0005634 // nu	0004727 // pr	Protein tyrosi	TP4A2_HUMAN|PTP4A2|PRL2|PTPCAAX2|BM-008
0035335 // pe	0005769 // ea	0004727 // pr	Protein tyrosi	TP4A3_HUMAN|PTP4A3|PRL3
0035335 // pe	0005634 // nu	0008138 // pr	Protein tyrosi	PTPC1_HUMAN|PTPDC1|PTP9Q22
0006661 // ph	0005886 // pl	0004725 // pr	Tyrosine-prot	PTN13_HUMAN|PTPN13|PNP1|PTP1E|PTPL1
0006470 // pr	0005737 // cy	0005515 // pr	Tyrosine-prot	PTN21_HUMAN|PTPN21|PTPD1
1900165 // ne	0048471 // pe	0004725 // pr	Tyrosine-prot	PTN22_HUMAN|PTPN22|PTPN8
0006470 // pr	0005856 // cy	0005515 // pr	Tyrosine-prot	PTN3_HUMAN|PTPN3|PTPH1
0033277 // ab	0005829 // cy	0005515 // pr	Tyrosine-prot	PTN6_HUMAN|PTPN6|HCP|PTP1C
0016311 // de	0005887 // in	0005515 // pr	Receptor-type	PTPRB_HUMAN|PTPRB|PTPB
0007185 // tr	0070062 // ex	0005102 // re	Receptor-type	PTPRD_HUMAN|PTPRD
0046627 // ne	0016021 // in	0005515 // pr	Receptor-type	PTPRE_HUMAN|PTPRE
0007155 // ce	0005886 // pl	0005001 // tr	Receptor-type	PTPRF_HUMAN|PTPRF|LAR
0010977 // ne	0070062 // ex	0005515 // pr	Receptor-type	PTPRG_HUMAN|PTPRG|PTPG
0042493 // re	0030027 // la	0004725 // pr	Receptor-type	PTPRM_HUMAN|PTPRM|PTPRL1
0035335 // pe	0005886 // pl	0005001 // tr	Receptor-type	PTPR2_HUMAN|PTPRN2|KIAA0387
0046856 // ph	0016021 // in	0004725 // pr	Phosphatidyli	PTPRQ_HUMAN|PTPRQ
0035335 // pe	0016021 // in	0005515 // pr	Receptor-type	PTPRR_HUMAN|PTPRR|ECPTP|PTPRQ
0098609 // ce	0005886 // pl	0005001 // tr	Receptor-type	PTPRU_HUMAN|PTPRU|FMI|PCP2|PTPRO
0006470 // pr	0005578 // pr	0004725 // pr	Receptor-type	PTPRZ_HUMAN|PTPRZ1|HTPZP2|PTPRZ|PTPRZ2|PTPZ
	0005739 // m	0003723 // RN	Probable pept	PTH_HUMAN|PTRH1|C9orf115
0050999 // re	0005737 // cy	0005515 // pr	6-pyruvoyl tet	PTPS_HUMAN|PTS

0008228 // op	0005615 // ex	0005515 // pr	Pentraxin-rela	PTX3_HUMAN|PTX3|TNFAIP5|TSG14
0009117 // nu	0070062 // ex	0016791 // ph	Pseudouridine	HDHD1_HUMAN|PUDP|DXF68S1E|FAM16AX|GS1|HDHI
0008284 // po	0043025 // ne	0003691 // do	Transcriptiona	PURA_HUMAN|PURA|PUR1
0045637 // re	0005662 // DI	0003723 // RI	Transcriptiona	PURB_HUMAN|PURB
	0005634 // nu	0003723 // RI	Purine-rich ele	PURG_HUMAN|PURG
0031119 // tR	0043231 // in	0003723 // RI	tRNA pseudou	PUSL1_HUMAN|PUSL1
0050860 // ne	0016021 // in	0005515 // pr	Transmembra	PVRIG_HUMAN|PVRIG|C7orf15
0034773 // hi	0005794 // Go	1990889 // H4	Periodic trypt	PWP1_HUMAN|PWP1
	0005634 // nu	0003682 // ch	PWWP domai	PWP2A_HUMAN|PWWP2A|KIAA1935|MST101
	0005654 // nu	0005515 // pr	PWWP domai	PWP2B_HUMAN|PWWP2B|PWWP2
		0035091 // ph	PX domain-co	PXDC1_HUMAN|PXDC1|C6orf145
0098869 // ce	0070062 // ex	0020037 // he	Peroxidasin ho	PXDN_HUMAN|PXDN|KIAA0230|MG50|PRG2|VPO|VPC
0098869 // ce	0005615 // ex	0020037 // he	Peroxidasin-lil	PXDNL_HUMAN|PXDNL|VPO2
0006954 // in	0005886 // pl	0005524 // AT	PX domain-co	PXK_HUMAN|PXK
0008150 // bi	0005777 // pe	0005515 // pr	Peroxisomal n	PXMP4_HUMAN|PXMP4|PMP24
0051496 // po	0005829 // cy	0005178 // in	Paxillin	PAXI_HUMAN|PXN
0016311 // de	0016021 // in	0003993 // ac	2-phosphoxyl	PXYP1_HUMAN|PXYLP1|ACPL2|HEL124|XYLP|UNQ370/
0090200 // po	0005829 // cy	0005515 // pr	Apoptosis-ass	ASC_HUMAN|PYCARD|ASC|CARD5|TMS1
1903206 // ne	0005739 // m	0042802 // id	Pyrroline-5-ca	P5CR1_HUMAN|PYCR1
0034599 // ce	0005739 // m	0004735 // py	Pyrroline-5-ca	P5CR2_HUMAN|PYCR2
0005980 // gl	0005576 // ex	0005515 // pr	Glycogen pho	PYGB_HUMAN|PYGB
1904837 // be	0005654 // nu	0046872 // m	Pygopus hom	PYGO1_HUMAN|PYGO1
1903259 // ex	0005730 // nu	0005515 // pr	Partner of Y14	PYM1_HUMAN|PYM1|PYM|WIBG
0034599 // ce	0030017 // sa	0005515 // pr	Pyridine nucle	PYRD1_HUMAN|PYROXD1
0007218 // ne	0005576 // ex	0005184 // ne	Peptide YY	PYY_HUMAN|PYY
0007218 // ne	0005615 // ex	0005184 // ne	Putative pepti	PYY2_HUMAN|PYY2
0008380 // RI	0005634 // nu	0005515 // pr	Protein quakir	QKI_HUMAN|QKI|HKQ
0017186 // pe	0070062 // ex	0016603 // gl	Glutaminyl-pe	QPCT_HUMAN|QPCT
0019674 // N/	0005737 // cy	0004514 // ni	Nicotinate-nu	NADC_HUMAN|QPRT
0007186 // G-	0005886 // pl	0004983 // ne	Pyroglutamyla	QRFPR_HUMAN|QRFPR|GPR103
0002576 // pl	0005794 // Go	0003756 // pr	Sulfhydryl oxi	QSOX1_HUMAN|QSOX1|QSCN6|UNQ2520/PRO6013
0006400 // tR	0005634 // nu	0046982 // pr	Queuine tRNA	TGT_HUMAN|QTRT1|TGT|TGUT
	0005634 // nu	0003723 // RI	R3H domain-c	R3HD2_HUMAN|R3HDM2|KIAA1002
	0005634 // nu	0003676 // nu	R3H domain-c	R3HD4_HUMAN|R3HDM4|C19orf22
0045055 // re	0005829 // cy	0005515 // pr	Rab11 family-	RFIP1_HUMAN|RAB11FIP1|RCP
1903078 // po	0005768 // er	0019901 // pr	Rab11 family-	RFIP2_HUMAN|RAB11FIP2|KIAA0941
0032456 // er	0055037 // re	0017137 // Ra	Rab11 family-	RFIP3_HUMAN|RAB11FIP3|ARFO1|KIAA0665
0016032 // vi	0055038 // re	0005515 // pr	Rab11 family-	RFIP4_HUMAN|RAB11FIP4|ARFO2|KIAA1821
0071468 // ce	0005794 // Go	0005515 // pr	Rab11 family-	RFIP5_HUMAN|RAB11FIP5|GAF1|KIAA0857|RIP11
0032402 // m	0005769 // ea	0005515 // pr	Ras-related pr	RAB17_HUMAN|RAB17
	0005768 // er	0003924 // G	Ras-related pr	RAB19_HUMAN|RAB19|RAB19B
0090383 // ph	0005794 // Go	0003924 // G	Ras-related pr	RAB20_HUMAN|RAB20
0006914 // au	0005776 // au	0003924 // G	Ras-related pr	RAB24_HUMAN|RAB24
0060627 // re	0031410 // cy	0003924 // G	Ras-related pr	RAB25_HUMAN|RAB25|CATX8

0006904 // ve	0030667 // se	0003924 // G	Ras-related pr	RAB26_HUMAN|RAB26
0002576 // pl	0005795 // G	0019003 // GI	Ras-related pr	RB27B_HUMAN|RAB27B
1901998 // to	0005737 // cy	0003924 // G	Ras-related pr	RAB28_HUMAN|RAB28
1990967 // m	0042470 // m	0003924 // G	Ras-related pr	RAB7L_HUMAN|RAB29|RAB7L1
0007030 // G	0000139 // G	0003924 // G	Ras-related pr	RAB30_HUMAN|RAB30
0035646 // er	0016020 // m	0030742 // G	Ras-related pr	RAB32_HUMAN|RAB32
0061024 // m	0000139 // G	0003924 // G	Ras-related pr	RB33A_HUMAN|RAB33A|RABS10
0015031 // pr	0005794 // G	0003924 // G	Ras-related pr	RAB36_HUMAN|RAB36
0006904 // ve	0005793 // er	0003924 // G	Ras-related pr	RAB37_HUMAN|RAB37
0015031 // pr	0016020 // m	0030742 // G	Ras-related pr	RAB38_HUMAN|RAB38
0016192 // ve	0043005 // ne	0003924 // G	Ras-related pr	RB39B_HUMAN|RAB39B
0051602 // re	0005829 // cy	0003924 // G	Ras-related pr	RAB3A_HUMAN|RAB3A
0017157 // re	0005737 // cy	0005525 // G	Ras-related pr	RAB3B_HUMAN|RAB3B
0018125 // pe	0042588 // zy	0003924 // G	Ras-related pr	RAB3D_HUMAN|RAB3D|GOV|RAB16
0006886 // in	0043234 // pr	0030234 // er	Rab3 GTPase-	RBGPR_HUMAN|RAB3GAP2|KIAA0839
0043547 // po	0005829 // cy	0005515 // pr	Guanine nucle	R3GEF_HUMAN|RAB3IL1
0009306 // pr	0030667 // se	0003924 // G	Ras-related pr	RB40A_HUMAN|RAB40A
0009306 // pr	0005635 // nu	0003924 // G	Ras-related pr	RB40B_HUMAN|RAB40B|SEC4L
	0016020 // m	0003924 // G	Putative Ras-r	RAB42_HUMAN|RAB42
0007030 // G	0005768 // er	0005515 // pr	Ras-related pr	RAB43_HUMAN|RAB43|RAB41
0046323 // gl	0032593 // in	0003924 // G	Ras-related pr	RAB4B_HUMAN|RAB4B|PP1596
0016032 // vi	0005794 // G	0005515 // pr	Ras-related pr	RAB6A_HUMAN|RAB6A|RAB6
0006890 // re	0005793 // er	0003924 // G	Ras-related pr	RAB6B_HUMAN|RAB6B
0061024 // m	0031902 // la	0003924 // G	Ras-related pr	RAB7B_HUMAN|RAB7B
0061024 // m	0055038 // re	0005515 // pr	Ras-related pr	RAB8A_HUMAN|RAB8A|MEL|RAB8
0006904 // ve	0070062 // ex	0005525 // G	Ras-related pr	RAB8B_HUMAN|RAB8B
	0016021 // in	0005515 // pr	Prenylated Ra	PRAF1_HUMAN|RABAC1|PRA1|PRAF1
0031338 // re	0005829 // cy	0015631 // tu	Rab GTPase-a	RBGP1_HUMAN|RABGAP1|HSPC094
0032880 // re	0005794 // G	0005096 // G	Rab GTPase-a	RBG1L_HUMAN|RABGAP1L|HHL|KIAA0471
0018344 // pr	0005968 // Ra	0005515 // pr	Geranylgeran	PGTB2_HUMAN|RABGGTB|GGTB
		0005525 // G	Rab-like prote	RABL3_HUMAN|RABL3
0071526 // se	0005829 // cy	0005515 // pr	Ras-related C	RAC1_HUMAN|RAC1|TC25|MIG5
0021894 // ce	0005886 // pl	0005515 // pr	Ras-related C	RAC3_HUMAN|RAC3
0030822 // po	0005634 // nu	0005080 // pr	Receptor of a	RACK1_HUMAN|RACK1|GNB2L1|HLC7|PIG21
0000076 // DI	0005654 // nu	0005515 // pr	Cell cycle chec	RAD17_HUMAN|RAD17|R24L
0007131 // re	0000775 // ch	0005515 // pr	Double-stranc	RAD21_HUMAN|RAD21|HR21|KIAA0078|NXP1
0071479 // ce	0043234 // pr	0005515 // pr	RAD51-associ	R51A1_HUMAN|RAD51AP1|PIR51
0008150 // bi	0043234 // pr	0005515 // pr	RAD51-associ	R51A2_HUMAN|RAD51AP2
0006281 // DI	0005654 // nu	0003690 // do	DNA repair pr	RA51C_HUMAN|RAD51C|RAD51L2
0006281 // DI	0005654 // nu	0003690 // do	DNA repair pr	RA51D_HUMAN|RAD51D|RAD51L3
0034599 // ce	0005654 // nu	0042802 // id	DNA repair pr	RAD52_HUMAN|RAD52
0006310 // DI	0043234 // pr	0003677 // DI	DNA repair an	RAD54_HUMAN|RAD54L|RAD54A
0031573 // in	0005654 // nu	0005515 // pr	Cell cycle chec	RAD9A_HUMAN|RAD9A
0031573 // in	0005654 // nu	0005515 // pr	Cell cycle chec	RAD9B_HUMAN|RAD9B
0007077 // m	0005635 // nu	0005515 // pr	mRNA export	RAE1L_HUMAN|RAE1|MRNP41

0001913 // T (	0005886 // pl	0046703 // na	NKG2D ligand	N2DL4_HUMAN|RAET1E|LETAL|N2DL4|ULBP4|UNQ186
0042267 // na	0005886 // pl	0046703 // na	Retinoic acid e	RET1G_HUMAN|RAET1G
0042267 // na	0031225 // ar	0046703 // na	Retinoic acid e	RET1L_HUMAN|RAET1L
0051865 // pr	0005654 // nu	0043565 // se	V(D)J recombi	RAG1_HUMAN|RAG1|RNF74
	0005938 // ce	0005515 // pr	Ankycorbin	RAI14_HUMAN|RAI14|KIAA1334|NORPEG
0009790 // er	0005575 // ce	0005515 // pr	Retinoic acid-i	RAI2_HUMAN|RAI2
0090630 // ac	0005829 // cy	0005096 // G	Ral GTPase-ac	RGPA2_HUMAN|RALGAPA2|C20orf74|KIAA1272
0043547 // pc	0005829 // cy	0005515 // pr	Ral guanine nu	GNDS_HUMAN|RALGDS|KIAA1308|RGF
0007264 // sn	0005886 // pl	0005515 // pr	Ras-specific gu	RGPS2_HUMAN|RALGPS2
	0005634 // nu	0003723 // RN	RNA-binding R	RALYL_HUMAN|RALYL|HNRPCL3
0006409 // tR	0005829 // cy	0003723 // RN	GTP-binding n	RAN_HUMAN|RAN|ARA24|OK/SW-cl.81
0006511 // ub	0005737 // cy	0005515 // pr	Ran-specific G	RANG_HUMAN|RANBP1
0006606 // pr	0005737 // cy	0005049 // nu	Ran-binding p	RBP17_HUMAN|RANBP17
1901706 // m	0005737 // cy	0005096 // G	Ran-binding p	RNB3L_HUMAN|RANBP3L
0006607 // NI	0031965 // nu	0008565 // pr	Ran-binding p	RNBP6_HUMAN|RANBP6
0016925 // pr	0005829 // cy	0005515 // pr	Ran GTPase-a	RAGP1_HUMAN|RANGAP1|KIAA1835|SD
0032527 // pr	0014704 // in	0017080 // so	Ran guanine n	MOG1_HUMAN|RANGRF|MOG1|RANGNRF|HSPC165|H
0009743 // re	0005886 // pl	0003924 // G	Ras-related pr	RAP1B_HUMAN|RAP1B|OK/SW-cl.11
0007165 // sig	0005829 // cy	0042803 // pr	Rap1 GTPase-	RPGP1_HUMAN|RAP1GAP|KIAA0474|RAP1GA1
0010977 // ne	0043005 // ne	0005096 // G	Rap1 GTPase-	RPGP2_HUMAN|RAP1GAP2|GARNL4|KIAA1039|RAP1GA
0034613 // ce	0055037 // re	0000287 // m	Ras-related pr	RAP2A_HUMAN|RAP2A
0019933 // cA	0016324 // ap	0030552 // cA	Rap guanine n	RPGF2_HUMAN|RAPGEF2|KIAA0313|NRAPGEP|PDZGEF
0007165 // sig	0005886 // pl	0005515 // pr	Rap guanine n	RPGF3_HUMAN|RAPGEF3|CGEF1|EPAC|EPAC1
0043547 // pc	0016604 // nu	0017034 // Ra	Rap guanine n	RPGF5_HUMAN|RAPGEF5|GFR|KIAA0277|MRGEF
0007264 // sn	0005622 // in	0005085 // gu	Rap guanine n	RPGFL_HUMAN|RAPGEFL1
1900075 // pc	0031594 // ne	0033130 // ac	43 kDa recept	RAPSN_HUMAN|RAPSN|RNF205
0008285 // ne	0005654 // nu	0000977 // RN	Retinoic acid r	RARB_HUMAN|RARB|HAP|NR1B2
0032526 // re	0016021 // in	0003700 // tra	Retinoic acid r	RARG_HUMAN|RARG|NR1B3
0001523 // re	0005576 // ex	0005102 // re	Retinoic acid r	RARR2_HUMAN|RARRES2|TIG2
0008285 // ne	0016021 // in	0004623 // ph	Retinoic acid r	HRSL4_HUMAN|RARRES3|RIG1|TIG3
0006420 // ar	0005829 // cy	0004814 // ar	Arginine--tRN	SYRC_HUMAN|RARS
0046580 // ne	0005829 // cy	0015278 // ca	Ras GTPase-ac	RASA3_HUMAN|RASA3
0046580 // ne	0005829 // cy	0005096 // G	Ras GTPase-ac	RASL2_HUMAN|RASA4|CAPRI|GAPL|KIAA0538
0046580 // ne	0005737 // cy	0005096 // G	Ras GTPase-ac	RAS4B_HUMAN|RASA4B
0046580 // ne	0005829 // cy	0005543 // ph	RasGAP-activa	RASL1_HUMAN|RASAL1|RASAL
0046580 // ne	0005829 // cy	0005515 // pr	Ras GTPase-ac	NGAP_HUMAN|RASAL2|NGAP
0001963 // sy	0005622 // in	0031681 // G-	GTP-binding p	RHES_HUMAN|RASD2|TEM2
	0048471 // pe	0003924 // G	Ras and EF-ha	RASEF_HUMAN|RASEF|RAB45
0043547 // pc	0005829 // cy	0005088 // Ra	Ras-GEF doma	RGF1A_HUMAN|RASGEF1A
0007264 // sn	0030496 // m	0005088 // Ra	Ras-GEF doma	RGF1B_HUMAN|RASGEF1B|GPIG4

0007165 // sig	0005886 // pl	0005089 // Rh	Ras-specific gu	RGRF1_HUMAN|RASGRF1|CDC25|GNRP|GRF1
0035023 // re	0005829 // cy	0005089 // Rh	Ras-specific gu	RGRF2_HUMAN|RASGRF2|GRF2
0043547 // po	0005829 // cy	0019992 // dia	RAS guanyl-re	GRP2_HUMAN|RASGRP2|CDC25L|MCG7
0048754 // br	0048471 // pe		Ras-interactin	RAIN_HUMAN|RASIP1
0007264 // sm	0005730 // nu	0003924 // GT	Ras-like prote	RSLAA_HUMAN|RASL10A|RRP22
0007165 // sig	0005886 // pl	0003924 // GT	Ras-like prote	RSLAB_HUMAN|RASL10B
0007165 // sig	0005730 // nu	0003924 // GT	Ras-like prote	RSLBA_HUMAN|RASL11A
0007165 // sig	0016020 // m	0003924 // GT	Ras-like prote	RSLBB_HUMAN|RASL11B
0070507 // re	0000922 // sp	0005515 // pr	Ras associatio	RASF1_HUMAN|RASSF1|RDA32
0007165 // sig	0000922 // sp		Ras associatio	RASFA_HUMAN|RASSF10
0043065 // po	0005737 // cy	0005515 // pr	Ras associatio	RASF2_HUMAN|RASSF2|CENP-34|KIAA0168
0042981 // re	0005829 // cy	0005515 // pr	Ras associatio	RASF3_HUMAN|RASSF3
0007049 // ce		0005515 // pr	Ras associatio	RASF4_HUMAN|RASSF4|AD037
0008285 // ne	0005634 // nu	0005515 // pr	Ras associatio	RASF5_HUMAN|RASSF5|NORE1|RAPL
0006915 // ap		0005515 // pr	Ras associatio	RASF6_HUMAN|RASSF6
0070507 // re	0005737 // cy	0005515 // pr	Ras associatio	RASF7_HUMAN|RASSF7|C11orf13|HRC1
0007165 // sig		0005515 // pr	Ras associatio	RASF8_HUMAN|RASSF8|C12orf2
0007165 // sig	0005829 // cy	0005215 // tra	Ras associatio	RASF9_HUMAN|RASSF9|PAMCI|PCIP1
0000398 // m	0005634 // nu	0003723 // RN	Ribonucleopro	RAVR1_HUMAN|RAVER1|KIAA1978
	0005615 // ex		RBBP8 N-term	RB8NL_HUMAN|RBBP8NL|C20orf151
0042127 // re	0070062 // ex	0016787 // hy	Putative hydro	RBBP9_HUMAN|RBBP9|BOG|RBBP10
0008380 // RN	0005737 // cy	0003729 // m	RNA binding p	RFOX3_HUMAN|RBFOX3
0006977 // DI	0005654 // nu	0005515 // pr	Retinoblastom	RBL2_HUMAN|RBL2|RB2
0007275 // m	0005634 // nu	0005515 // pr	Splicing regula	RBM11_HUMAN|RBM11
		0003723 // RN	RBM14-RBM4	A0A0A0MSL8_HUMAN|RBM14-RBM4
0008380 // RN	0005634 // nu	0003723 // RN	RNA-binding p	RBM20_HUMAN|RBM20
0000398 // m	0005737 // cy	0003723 // RN	Pre-mRNA-spl	RBM22_HUMAN|RBM22|ZC3H16|199G4
0006397 // m	0005634 // nu	0005515 // pr	Probable RNA	RBM23_HUMAN|RBM23|RNPC4|PP239
0030154 // ce	0005829 // cy	0003730 // m	RNA-binding p	RBM24_HUMAN|RBM24|RNPC6
0008380 // RN	0005681 // sp	0003723 // RN	RNA-binding p	RBM25_HUMAN|RBM25|RNPC7
0006417 // re	0005737 // cy	0003723 // RN	RNA-binding p	RBM3_HUMAN|RBM3|RNPL
0043484 // re	0005829 // cy	0003723 // RN	RNA-binding p	RBM38_HUMAN|RBM38|RNPC1|SEB4
0008380 // RN	0005634 // nu	0003723 // RN	RNA-binding p	RBM4_HUMAN|RBM4|RBM4A
0048025 // ne	0005634 // nu	0003723 // RN	RNA-binding p	RBM42_HUMAN|RBM42
		0003723 // RN	RNA-binding p	RBM43_HUMAN|RBM43|C2orf38
	0045171 // int	0042803 // pr	RNA-binding p	RBM44_HUMAN|RBM44
0016554 // cy	0005634 // nu	0003723 // RN	RNA-binding p	RBM47_HUMAN|RBM47
0008380 // RN	0005730 // nu	0003723 // RN	RNA-binding p	RBM4B_HUMAN|RBM4B|RBM30
0000184 // nu	0005654 // nu	0005515 // pr	RNA-binding p	RBM8A_HUMAN|RBM8A|RBM8|HSPC114|MDS014
0006396 // RN	0005634 // nu	0003723 // RN	RNA-binding m	RBMS1_HUMAN|RBMS1|C2orf12|MSSP|MSSP1|SCR2
0006396 // RN	0005634 // nu	0003723 // RN	RNA-binding m	RBMS2_HUMAN|RBMS2|SCR3
0090090 // ne	0005737 // cy	0008266 // po	RNA-binding m	RBMS3_HUMAN|RBMS3
0006406 // m	0070274 // RE	0003723 // RN	RNA-binding m	RBMX2_HUMAN|RBMX2|CGI-79

0008380 // RI	0030529 // in	0001047 // co	RNA binding r	RMXL1_HUMAN|RBMXL1
0001523 // re	0005829 // cy	0016918 // re	Retinol-bindir	RET1_HUMAN|RBP1|CRBP1
0001523 // re	1903561 // ex	0008236 // se	Retinol-bindir	RET3_HUMAN|RBP3
0007417 // ce	0005634 // nu	0003677 // DI	Calcipressin-1	RCAN1_HUMAN|RCAN1|ADAPT78|CSP1|DSC1|DSCR1
0019722 // ca	0005737 // cy	0008597 // ca	Calcipressin-2	RCAN2_HUMAN|RCAN2|DSCR1L1|ZAKI4
0043547 // pc		0005515 // pr	RCC1 and BTB	RCBT2_HUMAN|RCBTB2|CHC1L|RLG
0043547 // pc	0000794 // co	0005087 // Ra	Regulator of c	RCC1_HUMAN|RCC1|CHC1
	0005829 // cy		RCC1 domain-	RCCD1_HUMAN|RCCD1
0006508 // pr	0005887 // in	0004175 // er	CAAX prenyl p	FACE2_HUMAN|RCE1|FACE2|RCE1A|RCE1B
0000479 // er	0005654 // nu	0004521 // er	RNA 3'-termin	RCL1_HUMAN|RCL1|RNAC|RPC2|RPCL1|RTC2|HSPC338
	0005788 // er	0005515 // pr	Reticulocalbin	RCN3_HUMAN|RCN3|UNQ239/PRO272
0006351 // tra	0005634 // nu	0019899 // er	REST corepres	RCOR2_HUMAN|RCOR2
0006351 // tra	0005634 // nu	0005515 // pr	REST corepres	RCOR3_HUMAN|RCOR3|KIAA1343
0003009 // sk	0005884 // ac	0051015 // ac	CapZ-interacti	CPZIP_HUMAN|RCSD1|CAPZIP
0042572 // re	0005789 // er	0052650 // NA	Retinol dehyd	RDH11_HUMAN|RDH11|ARSDR1|PSDR1|SDR7C1|CGI-8
0050896 // re	0060342 // ph	0005515 // pr	Retinol dehyd	RDH12_HUMAN|RDH12|SDR7C2
0001649 // os	0005789 // er	0016491 // ox	Retinol dehyd	RDH14_HUMAN|RDH14|PAN2|SDR7C4|UNQ529/PRO10
0006629 // lip	0016021 // in	0004745 // re	Retinol dehyd	RDH16_HUMAN|RDH16|RODH4|SDR9C8
0050896 // re	0005789 // er	0004745 // re	11-cis retinol	RDH1_HUMAN|RDH5|HSD17B9|RDH1|SDR9C5
	0016605 // PN	0003677 // DI	RAD52 motif-	RDM1_HUMAN|RDM1|RAD52B
0006310 // DI			Meiotic recon	RE114_HUMAN|REC114|C15orf60
0007566 // er	0031225 // an	0004866 // er	Reversion-ind	RECK_HUMAN|RECK|ST15
0032508 // DI	0005694 // ch	0003677 // DI	ATP-depender	RECQ1_HUMAN|RECQL|RECQ1|RECQL1
0051205 // pr	0071782 // er	0031849 // ol	Receptor expr	REEP1_HUMAN|REEP1|C2orf23|SPG31
0032386 // re	0005783 // er	0031883 // ta	Receptor expr	REEP2_HUMAN|REEP2|C5orf19|SGC32445
0007084 // m	0016021 // in		Receptor expr	REEP3_HUMAN|REEP3|C10orf74
0007084 // m	0016021 // in	0008017 // m	Receptor expr	REEP4_HUMAN|REEP4|C8orf20|PP432
0006366 // tra	0005829 // cy	0005515 // pr	Proto-oncoge	REL_HUMAN|REL
0002223 // sti	0005654 // nu	0005515 // pr	Transcription	RELB_HUMAN|RELB
0006508 // pr	0005576 // ex	0070326 // ve	Reelin	RELN_HUMAN|RELN
0008217 // re	0005737 // cy	0004190 // as	Renin	RENI_HUMAN|REN
0033572 // tra	0010008 // er	0005515 // pr	Rab15 effecto	REP15_HUMAN|REP15
0007173 // ep	0005829 // cy	0005515 // pr	RalBP1-associ	REPS2_HUMAN|REPS2|POB1
0006890 // re	0005793 // er	0033130 // ac	Protein RER1	RER1_HUMAN|RER1
0006607 // NI	0005634 // nu	0001106 // RI	Arginine-gluta	RERE_HUMAN|RERE|ARG|ARP|ATN1L|KIAA0458
0008283 // ce	0005615 // ex	0003674 // m	Resistin-like b	RETNB_HUMAN|RETNLB|CCRG|FIZZ2|HXCP2|RETNL2|U
0032201 // te	0005654 // nu	0003689 // DI	Replication fa	RFC5_HUMAN|RFC5
0072593 // re	0005829 // cy	0008531 // rib	Riboflavin kin	RIFK_HUMAN|RFK
0061181 // re	0005737 // cy	0005515 // pr	Refilin-A	RFLA_HUMAN|RFLNA|FAM101A
0061572 // ac	0005737 // cy	0031005 // fil	Refilin-B	RFLB_HUMAN|RFLNB
0007399 // ne	0030173 // in	0033829 // O-	Beta-1,3-N-ac	RFNG_HUMAN|RFNG
0050853 // B	0005768 // er	0003725 // dc	Raftlin	RFTN1_HUMAN|RFTN1|KIAA0084|MIG2
0033227 // ds	0045121 // m		Raftlin-2	RFTN2_HUMAN|RFTN2|C2orf11
0043161 // pr	0005654 // nu	0005515 // pr	E3 ubiquitin-p	RFWD2_HUMAN|RFWD2|COP1|RNF200
0001675 // ac	0005737 // cy	0005515 // pr	DNA-binding p	RFX2_HUMAN|RFX2

0000122 // ne	0005634 // nu	0003677 // DI	DNA-binding p	RFX5_HUMAN|RFX5
0050796 // re	0005634 // nu	0005515 // pr	DNA-binding p	RFX6_HUMAN|RFX6|RFXDC1
0006357 // re	0005634 // nu	0003677 // DI	DNA-binding p	RFXK_HUMAN|RFXANK|ANKRA1|RFXB
0006956 // co	0005634 // nu	0030295 // pr	Regulator of c	RGCC_HUMAN|RGCC|C13orf15|RGC32
0007264 // sn	0005829 // cy	0008321 // Ra	Ral guanine nu	RGL1_HUMAN|RGL1|KIAA0959|RGL
0043547 // po	0005622 // int	0005515 // pr	Ral guanine nu	RGL2_HUMAN|RGL2|RAB2L
0007165 // sig	0005793 // er	0042802 // ide	RGM domain	RGMB_HUMAN|RGMB
0034260 // ne	0005576 // ex	0004341 // glu	Regucalcin	RGN_HUMAN|RGN|SMP30
0061024 // m	0005829 // cy	0005515 // pr	RAB6A-GEF co	RGP1_HUMAN|RGP1|KIAA0258
0046907 // int	0005622 // int		RANBP2-like a	RGPD1_HUMAN|RGPD1|RANBP2L6|RGP1
0046907 // int	0005622 // int		RANBP2-like a	RGPD2_HUMAN|RGPD2|RANBP2L2|RGP2
0046907 // int	0005622 // int		RanBP2-like a	RGPD3_HUMAN|RGPD3|RGP3
0046907 // int	0005737 // cy		RANBP2-like a	RGPD5_HUMAN|RGPD5|RGPD6|RANBP2L1|RANBP2L2|
0046907 // int	0005643 // nu	0008536 // Ra	RANBP2-like a	RGPD8_HUMAN|RGPD8|RANBP2ALPHA|RANBP2L1|RAN
0007213 // G-	0043025 // ne	0001965 // G-	Regulator of G	RGS10_HUMAN|RGS10
0008277 // re	0005634 // nu	0005057 // sig	Regulator of G	RGS12_HUMAN|RGS12
0006913 // nu	0016605 // PM	0030159 // re	Regulator of G	RGS14_HUMAN|RGS14
0007186 // G-	0031224 // int	0005515 // pr	Regulator of G	RGS16_HUMAN|RGS16|RGSR
0043547 // po	0030054 // ce	0005515 // pr	Regulator of G	RGS17_HUMAN|RGS17|RGSZ2
0008277 // re	0005737 // cy	0005096 // GT	Regulator of G	RGS18_HUMAN|RGS18|RGS13
0043547 // po	0030136 // cla	0001965 // G-	Regulator of G	RGS19_HUMAN|RGS19|GAIP|GNAI3IP
0007283 // sp	0005737 // cy	0005515 // pr	Regulator of G	RGS2_HUMAN|RGS2|G0S8|GIG31
0043547 // po	0005737 // cy	0005515 // pr	Regulator of G	RGS20_HUMAN|RGS20|RGSZ1|ZGAP1
0043547 // po	0005737 // cy	0005096 // GT	Regulator of G	RGS21_HUMAN|RGS21
0043547 // po	0005737 // cy	0005515 // pr	Regulator of G	RGS3_HUMAN|RGS3
0007186 // G-	0043234 // pr	0001965 // G-	Regulator of G	RGS4_HUMAN|RGS4
0007186 // G-	0005834 // he	0004871 // sig	Regulator of G	RGS6_HUMAN|RGS6
0007186 // G-	0005886 // pl	0031681 // G-	Regulator of G	RGS7_HUMAN|RGS7
0043547 // po	0097381 // ph	0004871 // sig	Regulator of G	RGS9_HUMAN|RGS9
0006508 // pr	0048471 // pe	0004252 // se	Rhomboid dom	RHBD2_HUMAN|RHBDD2|RHBDL7
0042058 // re	0005789 // er	0019838 // gr	Inactive rhom	RHDF1_HUMAN|RHBDF1|C16orf8|DIST1|IRHOM1
0050709 // ne	0005789 // er	0004252 // se	Inactive rhom	RHDF2_HUMAN|RHBDF2|IRHOM2|RHBDL5|RHBDL6
0016485 // pr	0016020 // m	0004252 // se	Rhomboid-rel	RHBL1_HUMAN|RHBDL1|RHBDL
0016485 // pr	0016021 // int	0004252 // se	Rhomboid-rel	RHBL2_HUMAN|RHBDL2
0016485 // pr	0016021 // int	0004252 // se	Rhomboid-rel	RHBL3_HUMAN|RHBDL3|RHBDL4|VRHO
0015695 // or	0005886 // pl	0008519 // ar	Ammonium tr	RHBG_HUMAN|RHBG
0015695 // or	0005887 // int	0008519 // ar	Blood group R	RHCE_HUMAN|RHCE|RHC|RHE
0015695 // or	0016324 // ap	0008519 // ar	Ammonium tr	RHCG_HUMAN|RHCG|C15orf6|CDRC2|PDRC2|RHGK
0071479 // ce	0005654 // nu	0005515 // pr	RAD9, HUS1, I	RHNO1_HUMAN|RHNO1|C12orf32|RHINO|HKMT1188
0051056 // re	0005886 // pl	0003924 // GT	Rho-related B	RHBT1_HUMAN|RHOBTB1|KIAA0740
0042147 // re	0005829 // cy	0003924 // GT	Rho-related B	RHBT3_HUMAN|RHOBTB3|KIAA0878
0044319 // wo	0005886 // pl	0003924 // GT	Rho-related G	RHOC_HUMAN|RHOC|ARH9|ARHC
0045785 // po	0005886 // pl	0003924 // GT	Rho-related G	RHOD_HUMAN|RHOD|ARHD

0007015 // ac	0005886 // pl	0005525 // G	Rho-related G	RHOF_HUMAN	RHOF_HUMAN|RHOF|ARHF|RIF
1900027 // re	0005886 // pl	0005515 // pr	Rho-related G	RHOG_HUMAN	RHOG_HUMAN|RHOG|ARHG
0007266 // Rh	0005886 // pl	0005515 // pr	Rho-related G	RHOJ_HUMAN	RHOJ_HUMAN|RHOJ|ARHJ|RASL7B|RHOI|TCL
0007010 // cy	0005886 // pl	0005515 // pr	Rho-related G	RHOU_HUMAN	RHOU_HUMAN|RHOU|ARHU|CDC42L1|G28K|WRCH1|S
0051056 // re	0005886 // pl	0003924 // G	Rho-related G	RHOV_HUMAN	RHOV_HUMAN|RHOV|ARHV|WRCH2
0007165 // sig	0005829 // cy		Rhophilin-2	RHPN2_HUMAN	RHPN2_HUMAN|RHPN2
		0005515 // pr	RIB43A-like w	RIBC1_HUMAN	RIBC1_HUMAN|RIBC1
	0005634 // nu	0005515 // pr	RIB43A-like w	RIBC2_HUMAN	RIBC2_HUMAN|RIBC2|C22orf11
0070586 // ce	0005737 // cy	0005096 // G	Synembryn-A	RIC8A_HUMAN	RIC8A_HUMAN|RIC8A
0031532 // ac	0031932 // TO	0005515 // pr	Rapamycin-ins	RICTR_HUMAN	RICTR_HUMAN|RICTOR|KIAA1999
0032509 // en	0005770 // lat	0005515 // pr	Rab-interactin	RILP_HUMAN|RI	RILP_HUMAN|RILP|PP10141
0060271 // cil	0005737 // cy	0031267 // sm	RILP-like prote	RIPL2_HUMAN	RIPL2_HUMAN|RILPL2|RLP2
		0005515 // pr	RIMS-binding	RIM3A_HUMAN	RIM3A_HUMAN|RIMBP3|KIAA1666|RIMBP3A
0006464 // ce	0005829 // cy	0072590 // N-	N-acetylaspar	RIMKA_HUMAN	RIMKA_HUMAN|RIMKLA|FAM80A
0006464 // ce	0005829 // cy	0072590 // N-	Beta-citrylglut	RIMKB_HUMAN	RIMKB_HUMAN|RIMKLB|FAM80B|KIAA1238
0048791 // ca	0005829 // cy	0003723 // RN	Regulating syn	RIMS1_HUMAN	RIMS1_HUMAN|RIMS1|KIAA0340|RAB3IP2|RIM1|NblaC
0017156 // ca	0005622 // int	0044325 // io	Regulating syn	RIMS2_HUMAN	RIMS2_HUMAN|RIMS2|KIAA0751|RAB3IP3|RIM2
0006897 // en	0005769 // ea	0017112 // Ra	Ras and Rab in	RIN3_HUMAN|	RIN3_HUMAN|RIN3
0043547 // po	0015629 // ac	0005096 // G	Ras and Rab in	RINL_HUMAN|	RINL_HUMAN|RINL
2000208 // po	0005737 // cy	0004674 // pr	Serine/threon	RIOK2_HUMAN	RIOK2_HUMAN|RIOK2
0002009 // m	0016020 // m	0004674 // pr	Receptor-inte	RIPK4_HUMAN	RIPK4_HUMAN|RIPK4|ANKRD3|DIK
0007265 // Ra	0005622 // int	0005515 // pr	GTP-binding p	RIT1_HUMAN|	RIT1_HUMAN|RIT1|RIBB|RIT|ROC1
0045746 // ne	0005634 // nu	0005515 // pr	RBPJ-interacti	RITA1_HUMAN	RITA1_HUMAN|RITA1|C12orf52|RITA|PSEC0043
0043161 // pr	0005829 // cy	0004842 // ub	E3 ubiquitin-p	RNF12_HUMAN	RNF12_HUMAN|RLIM|RNF12
	0000922 // sp		Regulator of m	RMD1_HUMAN	RMD1_HUMAN|RMDN1|FAM82B|CGI-90
0042787 // pr	0034657 // GI	0005515 // pr	Protein RMD5	RMD5A_HUMAN	RMD5A_HUMAN|RMND5A
0090502 // RN	0005576 // ex	0003676 // nu	Ribonuclease	RNAS4_HUMAN	RNAS4_HUMAN|RNASE4|RNS4
0090502 // RN	0005634 // nu	0003676 // nu	Ribonuclease	RNH1_HUMAN|	RNH1_HUMAN|RNASEH1|RNH1
0006401 // RN	0005829 // cy	0004523 // RN	Ribonuclease	RNH2A_HUMAN	RNH2A_HUMAN|RNASEH2A|RNASEHI|RNHIA
0090502 // RN	0016021 // int	0004521 // en	Ribonuclease	RNK_HUMAN|R	RNK_HUMAN|RNASEK
	0016021 // int	0003677 // DN	RNASEK-C17o	H0YIS7_HUMA	H0YIS7_HUMAN|RNASEK-C17orf49
0043488 // re	0005829 // cy	0004521 // en	2-5A-depende	RN5A_HUMAN	RN5A_HUMAN|RNASEL|RNS4
0090502 // RN	0005576 // ex	0033897 // rib	Ribonuclease	RNT2_HUMAN	RNT2_HUMAN|RNASET2|RNASE6PL
0048672 // po	0002080 // ac	0003924 // G	Rho-related G	RND2_HUMAN	RND2_HUMAN|RND2|ARHN|RHO7
0043161 // pr	0005769 // ea	0005515 // pr	RING finger pr	RNF11_HUMAN	RNF11_HUMAN|RNF11|CGI-123
0034247 // sn	0005684 // U2	0005515 // pr	RING finger pr	R113A_HUMAN	R113A_HUMAN|RNF113A|RNF113|ZNF183
0043161 // pr	0005829 // cy	0005515 // pr	E3 ubiquitin-p	RN115_HUMAN	RN115_HUMAN|RNF115|ZNF364
0032436 // po	0000139 // Go	0005515 // pr	E3 ubiquitin-p	RN125_HUMAN	RN125_HUMAN|RNF125
0042036 // ne	0016021 // int	0005515 // pr	E3 ubiquitin-p	RN128_HUMAN	RN128_HUMAN|RNF128
0051865 // pr	0016021 // int	0061630 // ub	E3 ubiquitin-p	RNF13_HUMAN	RNF13_HUMAN|RNF13|RZF

0006915 // ap	0016021 // in	0004842 // ub	E3 ubiquitin-p	GOLI_HUMAN|RNF130
0016567 // pr	0005829 // cy	0004842 // ub	E3 ubiquitin-p	RN135_HUMAN|RNF135|L13
0032436 // pc	0035861 // sit	0005515 // pr	E3 ubiquitin-p	RN138_HUMAN|RNF138|NARF|HSD-4|HSD4
0042787 // pr	0005829 // cy	0003713 // tr	E3 ubiquitin-p	RNF14_HUMAN|RNF14|ARA54|HRIHFB2038
0051865 // pr	0016020 // m	0004842 // ub	RING finger pr	RN141_HUMAN|RNF141|ZNF230
0042787 // pr	0005794 // Go	0061630 // ub	E3 ubiquitin-p	R144A_HUMAN|RNF144A|KIAA0161|RNF144|UBCE7IP4
	0016021 // in	0046872 // m	RING finger pr	RN148_HUMAN|RNF148
	0016021 // in	0046872 // m	RING finger pr	RN150_HUMAN|RNF150|KIAA1214
0007283 // sp	0005634 // nu	0008270 // zi	RING finger pr	RN151_HUMAN|RNF151
0034198 // ce	0005764 // ly	0061630 // ub	E3 ubiquitin-p	RN152_HUMAN|RNF152
		0046872 // m	RING finger pr	RN157_HUMAN|RNF157|KIAA1917
0030513 // pc	0005634 // nu	0008270 // zi	E3 ubiquitin-p	RN165_HUMAN|RNF165
0007275 // m	0005634 // nu	0042803 // pr	RING finger pr	RNF17_HUMAN|RNF17|TDRD4
0016567 // pr	0016021 // in	0046872 // m	E3 ubiquitin-p	RN170_HUMAN|RNF170
0030433 // EF	0016021 // in	0061630 // ub	RING finger pr	RN175_HUMAN|RNF175
0032436 // pc	0016021 // in	0061630 // ub	E3 ubiquitin-p	RN180_HUMAN|RNF180
0051865 // pr	0005741 // m	0044390 // ub	E3 ubiquitin-p	RN185_HUMAN|RNF185
0000226 // m	0005829 // cy	0004842 // ub	E3 ubiquitin-p	RN19A_HUMAN|RNF19A|RNF19
0002250 // ac	0005829 // cy	0004842 // ub	E3 ubiquitin-p	RN19B_HUMAN|RNF19B|IBRDC3|NKLAM
1903954 // pc	0048471 // pe	0030544 // Hs	RING finger pr	RN207_HUMAN|RNF207|C1orf188
0016925 // pr	0000795 // sy	0019789 // SU	RING finger pr	R212B_HUMAN|RNF212B|C14orf164
	0016021 // in	0046872 // m	RING finger pr	RN215_HUMAN|RNF215
	0016021 // in	0046872 // m	RING finger pr	RN223_HUMAN|RNF223
		0046872 // m	RING finger pr	RN224_HUMAN|RNF224
	0016021 // in	0046872 // m	RING finger pr	RN225_HUMAN|RNF225
0016567 // pr	0005634 // nu	0051059 // NI	E3 ubiquitin-p	RNF25_HUMAN|RNF25
0050852 // T	0009898 // cy	0005515 // pr	E3 ubiquitin-p	RNF31_HUMAN|RNF31|ZIBRA
0016567 // pr	0016604 // nu	0031625 // ub	E3 ubiquitin-p	RNF34_HUMAN|RNF34
0008584 // m	0005634 // nu	0004842 // ub	E3 ubiquitin-p	RNF38_HUMAN|RNF38
0008150 // bi	0005737 // cy	0046872 // m	RING finger pr	RNF39_HUMAN|RNF39|HZFW
0010390 // hi	0043679 // ax	0005515 // pr	E3 ubiquitin-p	BRE1B_HUMAN|RNF40|BRE1B|KIAA0661
0016055 // W	0005887 // in	0004842 // ub	E3 ubiquitin-p	RNF43_HUMAN|RNF43
	0016021 // in	0046872 // m	RING finger ar	RNFT2_HUMAN|RNFT2|TMEM118
0006402 // m	0032311 // ar	0005515 // pr	Ribonuclease	RINI_HUMAN|RNH1|PRI|RNH
0071871 // re	0005615 // ex	0051379 // ep	Renalase	RNLS_HUMAN|RNLS|C10orf59
0000398 // m	0005634 // nu	0097157 // pr	RNA-binding p	RBM40_HUMAN|RNPC3|KIAA1839|RBM40|RNP
0043171 // pe	0005886 // pl	0070006 // m	Aminopeptida	AMPB_HUMAN|RNPEP|APB
0007155 // ce	0005886 // pl	0005515 // pr	Roundabout h	ROBO1_HUMAN|ROBO1|DUTT1
0035385 // Ro	0030424 // ax		Roundabout h	ROBO3_HUMAN|ROBO3
0001525 // ar	0070062 // ex	0004872 // re	Roundabout h	ROBO4_HUMAN|ROBO4|UNQ421/PRO3674
0010468 // re	0042622 // ph		Rod outer seg	ROM1_HUMAN|ROM1|TSPAN23
0001932 // re	0005737 // cy	0046982 // pr	Ropporin-1B	ROP1B_HUMAN|ROPN1B

0018108 // pe	0043235 // re	1904929 // co	Inactive tyrosi	ROR1_HUMAN|ROR1|NTRKR1
0043401 // st	0005634 // nu	0001077 // tra	Nuclear recep	RORB_HUMAN|RORB|NR1F2|RZRB
0043401 // st	0016604 // nu	0005515 // pr	Nuclear recep	RORG_HUMAN|RORC|NR1F3|RORG|RZRG

0042461 // pł	0005930 // ax		Retinitis pigm	RP1L1_HUMAN|RP1L1
0006457 // pr	0005814 // ce	0005525 // G	Protein XRP2	XRP2_HUMAN|RP2

0006260 // DI	0005654 // nu	0005515 // pr	Replication pr	RFA2_HUMAN|RPA2|REPA2|RPA32|RPA34
0006366 // tra	0005634 // nu	0005515 // pr	RNA polymera	RPAP1_HUMAN|RPAP1|KIAA1403
0070940 // de	0005654 // nu	0008420 // CT	Putative RNA	RPAP2_HUMAN|RPAP2|C1orf82
0003407 // ne	0031090 // or	0001786 // ph	Retinoid isom	RPE65_HUMAN|RPE65
0000470 // m	0005730 // nu	0003723 // RN	Ribosome pro	RPF1_HUMAN|RPF1|BXDC5
0000027 // rib	0005730 // nu	0003723 // RN	Ribosome pro	RPF2_HUMAN|RPF2|BXDC1
0006886 // in	0005737 // cy	0005509 // ca	Rab effector N	RPH3L_HUMAN|RPH3AL|NOC2
0006014 // D-	0005829 // cy	0005515 // pr	Ribose-5-phos	RPIA_HUMAN|RPIA|RPI
0043066 // ne	0005829 // cy	0003723 // RN	60S ribosoma	RL10_HUMAN|RPL10|DXS648E|QM
1901796 // re	0005829 // cy	0003723 // RN	60S ribosoma	RL11_HUMAN|RPL11
0017148 // ne	0005829 // cy	0003723 // RN	60S ribosoma	RL13A_HUMAN|RPL13A
0006364 // rR	0005829 // cy	0003723 // RN	60S ribosoma	RL14_HUMAN|RPL14
0002181 // cy	0016020 // m	0003723 // RN	60S ribosoma	RL15_HUMAN|RPL15|EC45|TCBAP0781
0006364 // rR	0005829 // cy	0003723 // RN	60S ribosoma	RL17_HUMAN|RPL17
0006412 // tra	0015934 // la	0003735 // st	Protein RPL17	A0A0A6YYL6_HUMAN|RPL17-C18orf32
0006364 // rR	0005829 // cy	0003723 // RN	60S ribosoma	RL18A_HUMAN|RPL18A
0006364 // rR	0005829 // cy	0003723 // RN	60S ribosoma	RL21_HUMAN|RPL21
0006364 // rR	0005829 // cy	0003723 // RN	60S ribosoma	RL22_HUMAN|RPL22
0002181 // cy	0022625 // cy	0003723 // RN	60S ribosoma	RL22L_HUMAN|RPL22L1
0021554 // op	0005829 // cy	0003723 // RN	60S ribosoma	RL24_HUMAN|RPL24
1904803 // re	0005829 // cy	0003723 // RN	60S ribosoma	RL26_HUMAN|RPL26
0042273 // rib	0070062 // ex	0003723 // RN	60S ribosoma	RL26L_HUMAN|RPL26L1|RPL26P1
0006364 // rR	0005829 // cy	0003723 // RN	60S ribosoma	RL27A_HUMAN|RPL27A
0006412 // tra	0005829 // cy	0003723 // RN	60S ribosoma	RL28_HUMAN|RPL28
0006364 // rR	0005829 // cy	0003723 // RN	60S ribosoma	RL29_HUMAN|RPL29

0006364 // rR	0005829 // cy	0003723 // RI	60S ribosoma	RL31_HUMAN	RPL31
0097421 // liv	0005829 // cy	0003723 // RI	60S ribosoma	RL32_HUMAN	RPL32 | PP9932
0000463 // m	0005829 // cy	0003723 // RI	60S ribosoma	RL35_HUMAN	RPL35
0002181 // cy	0005829 // cy	0003723 // RI	60S ribosoma	RL36_HUMAN	RPL36
0006364 // rR	0005829 // cy	0003723 // RI	60S ribosoma	RL36A_HUMAN	RPL36A | RPL44 | GIG15 | MIG6
0006412 // tra	0022625 // cy	0005515 // pr	60S ribosoma	RL36L_HUMAN	RPL36AL
0048318 // ax	0005829 // cy	0003723 // RI	60S ribosoma	RL38_HUMAN	RPL38
0006412 // tra	0005829 // cy	0003735 // st	60S ribosoma	RL39_HUMAN	RPL39
0007283 // sp	0022625 // cy	0003723 // RI	60S ribosoma	RL39L_HUMAN	RPL39L | RPL39L1
1901796 // re	0005829 // cy	0003723 // RI	60S ribosoma	RL5_HUMAN	RPL5 | MSTP030
0006364 // rR	0005829 // cy	0003723 // RI	60S ribosoma	RL6_HUMAN	RPL6 | TXREB1
0006364 // rR	0005829 // cy	0003677 // DI	60S ribosoma	RL7_HUMAN	RPL7
0006364 // rR	0005829 // cy	0005515 // pr	60S acidic ribo	RLA2_HUMAN	RPLP2 | D11S2243E | RPP2
0006487 // pr	0005791 // ro	0043022 // rib	Dolichyl-diphc	RPN2_HUMAN	RPN2
0001682 // tR	0005654 // nu	0003723 // RI	Ribonuclease	RPP14_HUMAN	RPP14
0001682 // tR	0005654 // nu	0003723 // RI	Ribonuclease	RPP25_HUMAN	RPP25
0001682 // tR	0005654 // nu	0003723 // RI	Ribonuclease	RPP30_HUMAN	RPP30 | RNASEP2
0001682 // tR	0005654 // nu	0004526 // rib	Ribonuclease	RPP40_HUMAN	RPP40 | RNASEP1
0042795 // sn	0005654 // nu		Regulation of	RPRD2_HUMAN	RPRD2 | KIAA0460 | HSPC099
0007050 // ce	0016021 // in	0005515 // pr	Protein reprin	RPRM_HUMAN	RPRM
0006364 // rR	0005829 // cy	0003723 // RI	40S ribosoma	RS12_HUMAN	RPS12
0006364 // rR	0005829 // cy	0003735 // st	40S ribosoma	RS13_HUMAN	RPS13
0000028 // rib	0005829 // cy	0048027 // m	40S ribosoma	RS14_HUMAN	RPS14 | PRO2640
0006364 // rR	0005829 // cy	0003723 // RI	40S ribosoma	RS15_HUMAN	RPS15 | RIG
0006364 // rR	0005829 // cy	0003723 // RI	40S ribosoma	RS15A_HUMAN	RPS15A | OK/SW-cl.82
1990830 // ce	0005829 // cy	0003723 // RI	40S ribosoma	RS16_HUMAN	RPS16
0030490 // m	0005829 // cy	0003723 // RI	40S ribosoma	RS19_HUMAN	RPS19
	0005730 // nu	0003723 // RI	Active regulat	AROS_HUMAN	RPS19BP1 | AROS
0006412 // tra	0005829 // cy	0003723 // RI	40S ribosoma	RS2_HUMAN	RPS2 | RPS4
0006364 // rR	0005829 // cy	0003723 // RI	40S ribosoma	RS21_HUMAN	RPS21
0018126 // pr	0005829 // cy	0003723 // RI	40S ribosoma	RS23_HUMAN	RPS23
0000462 // m	0005829 // cy	0003723 // RI	40S ribosoma	RS24_HUMAN	RPS24
0006364 // rR	0005829 // cy	0003723 // RI	40S ribosoma	RS25_HUMAN	RPS25
0006364 // rR	0005829 // cy	0003723 // RI	40S ribosoma	RS26_HUMAN	RPS26
0042771 // in	0005634 // nu	0003723 // RI	40S ribosoma	RS27L_HUMAN	RPS27L
0006364 // rR	0005829 // cy	0003723 // RI	40S ribosoma	RS28_HUMAN	RPS28
0006364 // rR	0005829 // cy	0003735 // st	40S ribosoma	RS29_HUMAN	RPS29
0045727 // pc	0005829 // cy	0003723 // RI	40S ribosoma	RS4X_HUMAN	RPS4X | CCG2 | RPS4 | SCAR

0043066 // ne	0005737 // cy	0000287 // m	Ribosomal pro	KS6A1_HUMAN|RPS6KA1|MAPKAPK1A|RSK1
0007165 // sig	0005654 // nu	0000287 // m	Ribosomal pro	KS6A2_HUMAN|RPS6KA2|MAPKAPK1C|RSK3
0006915 // ap	0005737 // cy	0000287 // m	Ribosomal pro	KS6A3_HUMAN|RPS6KA3|ISPK1|MAPKAPK1B|RSK2
0035066 // po	0005737 // cy	0000287 // m	Ribosomal pro	KS6A4_HUMAN|RPS6KA4|MSK2
0002223 // sti	0005737 // cy	0000287 // m	Ribosomal pro	KS6A5_HUMAN|RPS6KA5|MSK1
0007165 // sig	0005737 // cy	0000287 // m	Ribosomal pro	KS6A6_HUMAN|RPS6KA6|RSK4
0006468 // pr	0005737 // cy	0004711 // rib	Ribosomal pro	KS6B2_HUMAN|RPS6KB2|STK14B
0006468 // pr	0016020 // m	0004674 // pr	Ribosomal pro	KS6C1_HUMAN|RPS6KC1|RPK118
0006468 // pr	0005737 // cy	0004674 // pr	Ribosomal pro	RPKL1_HUMAN|RPS6KL1
0000462 // m	0005829 // cy	0003723 // RN	40S ribosoma	RS8_HUMAN|RPS8|OK/SW-cl.83
0006364 // rR	0005829 // cy	0003723 // RN	40S ribosoma	RS9_HUMAN|RPS9
0006407 // rR	0005737 // cy	0003723 // RN	40S ribosoma	RSSA_HUMAN|RPSA|LAMBR|LAMR1
0070268 // co	0005829 // cy	0046914 // tra	Repetin	RPTN_HUMAN|RPTN
1900087 // po	0005737 // cy	0005515 // pr	Regulatory-as	RPTOR_HUMAN|RPTOR|KIAA1303|RAPTOR
0031119 // tR	0005575 // ce	0003723 // RN	RNA pseudou	RUSD1_HUMAN|RPUSD1|C16orf40|RLUCL
0031119 // tR	0005739 // m	0003723 // RN	RNA pseudou	RUSD4_HUMAN|RPUSD4
1904263 // po	0005829 // cy	0005515 // pr	Ras-related G	RRAGD_HUMAN|RRAGD
0007265 // Ra	0005886 // pl	0003924 // G	Ras-related pr	RRAS2_HUMAN|RRAS2|TC21
0018298 // pr	0001750 // ph	0008020 // G-	Visual pigmen	OPSX_HUMAN|RRH
0006260 // DI	0005829 // cy	0004748 // rib	Ribonucleosid	RIR1_HUMAN|RRM1|RR1
0006260 // DI	0005829 // cy	0004748 // rib	Ribonucleosid	RIR2_HUMAN|RRM2|RR2
0007000 // nu	0005654 // nu	0001164 // RN	RNA polymera	RRN3_HUMAN|RRN3|TIFIA
0001180 // tra	0005634 // nu	0001042 // RN	Putative RRN3	RN3P1_HUMAN|RRN3P1
0031167 // rR	0016021 // in	0000179 // rR	Protein RRNA	RRNAD_HUMAN|RRNAD1|C1orf66|CGI-41
0006364 // rR	0005634 // nu	0003723 // RN	Ribosomal RN	RRP1_HUMAN|RRP1|D21S2056E|NNP1|NOP52|RRP1A
	0016021 // in	0003723 // RN	RRP12-like pro	RRP12_HUMAN|RRP12|KIAA0690
0000462 // m	0005654 // nu	0003723 // RN	Ribosomal RN	RRP36_HUMAN|RRP36|C6orf153|HSPC253
0000028 // rib	0034456 // U		Putative ribos	RRP7B_HUMAN|RRP7BP|RRP7B
0000183 // ch	0005654 // nu	0003723 // RN	Ribosomal RN	RRP8_HUMAN|RRP8|KIAA0409|NML|hucep-1
0006364 // rR	0005654 // nu	0003723 // RN	U3 small nucle	U3IP2_HUMAN|RRP9|RNU3IP2|U355K
0042273 // rib	0030687 // pr	0003723 // RN	Ribosome bio	RRS1_HUMAN|RRS1|KIAA0112|RRR
0006779 // po	0005739 // m	0004109 // co	Radical S-ade	RSAD1_HUMAN|RSAD1
0051607 // de	0005739 // m	0005515 // pr	Radical S-ade	RSAD2_HUMAN|RSAD2|CIG5
0017157 // re	0005737 // cy	0003924 // G	REM2- and Ra	RSG1_HUMAN|RSG1|C1orf89
0006412 // tra	0030686 // 90	0003723 // RN	Ribosomal L1	RL1D1_HUMAN|RSL1D1|CATX11|CSIG|PBK1|L12
0008150 // bi	0005575 // ce	0005515 // pr	Radial spoke h	RSP14_HUMAN|RSPH14|RTDR1
	0005737 // cy	0005515 // pr	Radial spoke h	RSPH3_HUMAN|RSPH3|RSHL2|RSP3
0003341 // cil	0005737 // cy	0005515 // pr	Radial spoke h	RSPH9_HUMAN|RSPH9|C6orf206|MRPS18AL1
0016055 // W	0005576 // ex	0005109 // fri	R-spondin-3	RSPO3_HUMAN|RSPO3|PWTSR|THSD2
0016055 // W	0005576 // ex	0008201 // he	R-spondin-4	RSPO4_HUMAN|RSPO4|C20orf182
0010810 // re	0070062 // ex	0005515 // pr	Ras suppresso	RSU1_HUMAN|RSU1|RSP1
	0019897 // ex	1902444 // rib	Retbindin	RTBDN_HUMAN|RTBDN

0006388 // tR	0005737 // cy	0003723 // RN	tRNA-splicing	RTCB_HUMAN|RTCB|C22orf28|HSPC117
1904430 // ne	0005634 // nu	0004003 // AT	Regulator of t	RTEL1_HUMAN|RTEL1|C20orf41|KIAA1088|NHL
0010569 // re	0005634 // nu	0005524 // AT	Regulator of t	F6WH68_HUMAN|RTEL1-TNFRSF6B|RTEL1
0071171 // sit	0005634 // nu	0003674 // m	Protein RTF2 I	RTF2_HUMAN|RTFDC1|C20orf43|AD-007|CDA05|HSPC:
0032185 // se	0005829 // cy	0005515 // pr	Rhotekin	RTKN_HUMAN|RTKN|RTKN1
0030097 // he			Rhotekin-2	RTKN2_HUMAN|RTKN2|PLEKHK1
	0030176 // in	0005515 // pr	Reticulon-1	RTN1_HUMAN|RTN1|NSP
0065002 // in	0030176 // in	0005515 // pr	Reticulon-2	RTN2_HUMAN|RTN2|NSPL1
0071786 // er	0016021 // in	0005515 // pr	Reticulon-3	RTN3_HUMAN|RTN3|ASYIP|NSPL2
0006612 // pr	0016021 // in	0031849 // ol	Receptor-tran	RTP4_HUMAN|RTP4|IFRG28|Z3CXXC4
0044782 // cil	0005737 // cy		Rotatin	RTTN_HUMAN|RTTN
0006897 // er	0031901 // ea	0005515 // pr	RUN and FYVE	RUFY1_HUMAN|RUFY1|RABIP4|ZFYVE12
	0005634 // nu	0046872 // m	RUN and FYVE	RUFY2_HUMAN|RUFY2|KIAA1537|RABIP4R
0030335 // po	0030027 // la	0005515 // pr	Protein RUFY3	RUFY3_HUMAN|RUFY3|KIAA0871
0002076 // os	0000790 // nu	0005515 // pr	Runt-related t	RUNX2_HUMAN|RUNX2|AML3|CBFA1|OSF2|PEBP2A
		0005515 // pr	Putative unch	RUAS1_HUMAN|RUSC1-AS1|C1orf104
0008150 // bi	0005829 // cy	0017137 // Ra	Iporin	RUSC2_HUMAN|RUSC2|KIAA0375
2000825 // po	0005737 // cy	0005515 // pr	RWD domain-	RWDD1_HUMAN|RWDD1|DFRP2|CGI-24|PTD013
0007188 // ad	0005886 // pl	0004930 // G-	Relaxin recept	RXFP1_HUMAN|RXFP1|LGR7
0043401 // st	0043234 // pr	0003700 // tra	Retinoic acid r	RXRA_HUMAN|RXRA|NR2B1
0022008 // ne	0005886 // pl	0017147 // W	Tyrosine-prot	RYK_HUMAN|RYK|JTK5A
0043588 // sk	0005886 // pl	0005245 // vo	Ryanodine rec	RYR1_HUMAN|RYR1|RYDR
0097050 // ty	0016529 // sa	0005515 // pr	Ryanodine rec	RYR2_HUMAN|RYR2
0051290 // pr	0070062 // ex	0005515 // pr	Protein S100-/	S10AA_HUMAN|S100A10|ANX2LG|CAL1L|CLP11
0008285 // ne	0005634 // nu	0098641 // ca	Protein S100-/	S10AB_HUMAN|S100A11|MLN70|S100C
0034142 // to	0016604 // nu	0042379 // ch	Protein S100-/	S10AE_HUMAN|S100A14|S100A15
0051592 // re	0070062 // ex	0003723 // RN	Protein S100-/	S10AG_HUMAN|S100A16|S100F|AAG13
0043542 // er	0005575 // ce	0005515 // pr	Protein S100-/	S10A2_HUMAN|S100A2|S100L
	0005730 // nu	0005509 // ca	Protein S100-/	S10A3_HUMAN|S100A3|S100E
0001837 // ep	0048471 // pe	0003723 // RN	Protein S100-/	S10A4_HUMAN|S100A4|CAPL|MTS1
	0005634 // nu	0005509 // ca	Protein S100-/	S10A5_HUMAN|S100A5|S100D
0002526 // ac	0005576 // ex	0005509 // ca	Protein S100-/	S10A8_HUMAN|S100A8|CAGA|CFAG|MRP8
0032119 // se	0005576 // ex	0005509 // ca	Protein S100-/	S10A9_HUMAN|S100A9|CAGB|CFAG|MRP14
0043542 // er	0034774 // se	0005515 // pr	Protein S100-I	S100P_HUMAN|S100P|S100E
0043547 // po	0005886 // pl	0004930 // G-	Sphingosine 1	S1PR1_HUMAN|S1PR1|CHEDG1|EDG1
0007193 // ad	0005886 // pl	0005515 // pr	Sphingosine 1	S1PR3_HUMAN|S1PR3|EDG3
0007189 // ad	0005886 // pl	0038036 // sp	Sphingosine 1	S1PR4_HUMAN|S1PR4|EDG6
0003376 // sp	0005886 // pl	0038036 // sp	Sphingosine 1	S1PR5_HUMAN|S1PR5|EDG8
0048246 // m	0005576 // ex	0008201 // he	Serum amyloi	SAA1_HUMAN|SAA1
0050918 // po	0070062 // ex	0042056 // ch	Serum amyloi	SAA2_HUMAN|SAA2
	0005615 // ex		Protein SAAL1	SAAL1_HUMAN|SAAL1
0051301 // ce	0005737 // cy	0005515 // pr	SAC3 domain-	SAC31_HUMAN|SAC3D1|SHD1

0090084 // ne	0030424 // ax	0030544 // Hs	Sacsin	SACS_HUMAN|SACS|KIAA0730
0016925 // pr	0005737 // cy	0005515 // pr	SUMO-activat	SAE1_HUMAN|SAE1|AOS1|SUA1|UBLE1A
0035019 // so	0005634 // nu	0003700 // tra	Sal-like protei	SALL1_HUMAN|SALL1|SAL1|ZNF794
0001654 // ey	0005634 // nu	0003700 // tra	Sal-like protei	SALL2_HUMAN|SALL2|KIAA0360|SAL2|ZNF795
0000122 // ne	0000792 // he	0003700 // tra	Sal-like protei	SALL4_HUMAN|SALL4|ZNF797
0006364 // rR	0030687 // pr		Sterile alpha r	SAM15_HUMAN|SAMD15|C14orf174|FAM15A
0006355 // re	0005829 // cy	0003723 // RN	Protein Smau	SMAG1_HUMAN|SAMD4A|KIAA1053|SAMD4|SMAUG1
2000303 // re	0030173 // int	0033188 // sp	Sphingomyeli	SAMD8_HUMAN|SAMD8
0034058 // en	0005829 // cy	0005515 // pr	Sterile alpha r	SAMD9_HUMAN|SAMD9|C7orf5|DRIF1|KIAA2004|OEF1
0034058 // en	0005769 // ea	0005515 // pr	Sterile alpha r	SAM9L_HUMAN|SAMD9L|C7orf6|DRIF2|KIAA2005|UEF
0090501 // RN	0005654 // nu	0032567 // dG	Deoxynucleos	SAMH1_HUMAN|SAMHD1|MOP5
0033108 // m	0005739 // m	0005515 // pr	Sorting and as	SAM50_HUMAN|SAMM50|SAM50|CGI-51|TRG3
0006351 // tra	0005634 // nu		Histone deace	SAP25_HUMAN|SAP25
0098725 // sy	0005730 // nu	0005515 // pr	Suppressor AP	SAPC2_HUMAN|SAPCD2|C9orf140
0090110 // ca	0030127 // CO	0005525 // GT	GTP-binding p	SAR1A_HUMAN|SAR1A|SAR1|SARA|SARA1
1901214 // re	0005829 // cy	0005515 // pr	Sterile alpha a	SARM1_HUMAN|SARM1|KIAA0524|SAMD2|SARM
0032733 // po	0005634 // nu		SAM and SH3	SASH3_HUMAN|SASH3|CXorf9|SLY
0046208 // sp	0005829 // cy	0005515 // pr	Diamine acety	SAT1_HUMAN|SAT1|SAT
0032919 // sp	0070062 // ex	0042802 // id	Diamine acety	SAT2_HUMAN|SAT2|SSAT2
0000122 // ne	0005720 // nu	0003682 // ch	DNA-binding p	SATB1_HUMAN|SATB1
0030030 // ce	0036126 // sp	0005515 // pr	Stabilizer of a	SAXO1_HUMAN|SAXO1|C9orf138|FAM154A
		0008017 // m	Stabilizer of a	SAXO2_HUMAN|SAXO2|FAM154B
	0016021 // int		SAYSvFN dom	SMDC1_HUMAN|SAYSD1|C6orf64
0043547 // po	0005774 // va	0019902 // ph	Myotubularin	MTMRD_HUMAN|SBF2|CMT4B2|KIAA1766|MTMR13
0018105 // pe	0005737 // cy	0005524 // AT	Serine/threon	SBK1_HUMAN|SBK1
0071222 // ce	0005575 // ce		Protein strawl	SBNO2_HUMAN|SBNO2|KIAA0963
0034976 // re	0016021 // int	0005515 // pr	Secretory carr	SCAM5_HUMAN|SCAMP5
0006355 // re	0005634 // nu	0042802 // id	SCAN domain-	SCND1_HUMAN|SCAND1|SDP1
0009650 // UV	0016021 // int	0005515 // pr	Scavenger rec	SCAR3_HUMAN|SCARA3|CSR
0010899 // re	0070062 // ex	0005545 // 1-	Scavenger rec	SCRB1_HUMAN|SCARB1|CD36L1|CLA1
1905123 // re	0005886 // pl	0001618 // vir	Lysosome me	SCRB2_HUMAN|SCARB2|CD36L2|LIMP2|LIMPII
0007155 // ce	0005886 // pl	0030169 // lo	Scavenger rec	SREC_HUMAN|SCARF1|KIAA0149|SREC
0007157 // he	0016021 // int	0005515 // pr	Scavenger rec	SREC2_HUMAN|SCARF2|SREC2|SREPCR
0002576 // pl	0005739 // m	0016491 // ox	Saccharopine	SCPDL_HUMAN|SCCPDH|CGI-49
0006636 // un	0005789 // en	0004768 // st	Acyl-CoA desa	ACOD_HUMAN|SCD
1903966 // m	0016021 // int	0004768 // st	Stearoyl-CoA	SCD5_HUMAN|SCD5|ACOD4|SCD2|SCD4

0009790 // er	0005737 // cy	0005515 // pr	Sciellin	SCEL_HUMAN|SCEL
0006954 // in	0005788 // er	0005515 // pr	Secretogranin	SCG2_HUMAN|SCG2|CHGC
0043086 // ne	0030141 // se	0005515 // pr	Neuroendocri	7B2_HUMAN|SCG5|SGNE1
0032714 // ne	0005791 // ro	0005515 // pr	Uteroglobin	UTER_HUMAN|SCGB1A1|CC10|CCSP|UGB
	0005576 // ex		Secretoglobin	SC2B2_HUMAN|SCGB2B2|SCGB4A2|SCGBL
0006898 // re	0005576 // ex		Secretoglobin	SG3A2_HUMAN|SCGB3A2|PNSP1|UGRP1|UNQ566/PRO
0051047 // po	0005903 // br	0001786 // ph	Adseverin	ADSV_HUMAN|SCIN|KIAA1905
0001887 // se	0005829 // cy	0005515 // pr	Selenocystein	SCLY_HUMAN|SCLY|SCL
0006351 // tra	0005634 // nu	0003700 // tra	Sex comb on n	SCML2_HUMAN|SCML2
2000649 // re	0005576 // ex	0019871 // so	Sodium chann	SCN1B_HUMAN|SCN1B
0008627 // in	0031226 // in	0005248 // vo	Sodium chann	SCN2A_HUMAN|SCN2A|NAC2|SCN2A1|SCN2A2
0060371 // re	0001518 // vo	0086006 // vo	Sodium chann	SCN2B_HUMAN|SCN2B|UNQ326/PRO386
0006814 // so	0005886 // pl	0005248 // vo	Sodium chann	SCN3A_HUMAN|SCN3A|KIAA1356|NAC3
0060307 // re	0001518 // vo	0017080 // so	Sodium chann	SCN4B_HUMAN|SCN4B
0042552 // m	0005886 // pl	0005515 // pr	Sodium chann	SCN8A_HUMAN|SCN8A|MED
0050891 // m	0005886 // pl	0050699 // W	Amiloride-sen	SCNNA_HUMAN|SCNN1A|SCNN1
0050896 // re	0005886 // pl	0005515 // pr	Amiloride-sen	SCNND_HUMAN|SCNN1D|DNACH
1901799 // ne	0005739 // m	0005507 // co	Protein SCO1	SCO1_HUMAN|SCO1|SCOD1
0045776 // ne	0070062 // ex	0004185 // se	Retinoid-indu	RISC_HUMAN|SCPEP1|RISC|SCP1|MSTP034|UNQ265/PI
0007586 // di	0005886 // pl	0015055 // se	Secretin recep	SCTR_HUMAN|SCTR
0007165 // sig	0009986 // ce	0005509 // ca	Signal peptide	SCUB2_HUMAN|SCUBE2|CEGP1
0051291 // pr	0005886 // pl	0005509 // ca	Signal peptide	SCUB3_HUMAN|SCUBE3|CEGF3
0003179 // he	0005634 // nu	0000980 // RN	Basic helix-loo	SCX_HUMAN|SCX|BHLHA41|BHLHA48|SCXA|SCXB
0042542 // re	0005796 // Go	0005515 // pr	Syndecan-1	SDC1_HUMAN|SDC1|SDC
0048013 // ep	0005796 // Go	0005515 // pr	Syndecan-2	SDC2_HUMAN|SDC2|HSPG1
0045860 // po	0005796 // Go	0005515 // pr	Syndecan-4	SDC4_HUMAN|SDC4
0008283 // ce	0070062 // ex	0005546 // ph	Syntenin-2	SDCB2_HUMAN|SDCBP2|SITAC18
	0005634 // nu		Protein SDE2 I	SDE2_HUMAN|SDE2|C1orf55
0042981 // re	0005788 // er	0051087 // ch	Stromal cell-d	SDF2L_HUMAN|SDF2L1|UNQ1941/PRO4424
0006099 // tri	0005749 // m	0050660 // fla	Succinate deh	SDHA_HUMAN|SDHA|SDH2|SDHF
0090090 // ne	0005739 // m	0005515 // pr	Succinate deh	SDHF2_HUMAN|SDHAF2|C11orf79|PGL2|SDH5
0003407 // ne	0005749 // m	0003674 // m	Succinate deh	SDHF4_HUMAN|SDHAF4|C6orf57
0007156 // ho	0016021 // in	0042802 // id	Protein sideki	SDK1_HUMAN|SDK1
0031065 // po	0000785 // ch	0004745 // re	Epidermal ret	RDHE2_HUMAN|SDR16C5|RDHE2
0055114 // ox	0005634 // nu	0016491 // ox	Epimerase far	D39U1_HUMAN|SDR39U1|C14orf124|HCDI
0006694 // ste	0016021 // in	0003854 // 3-	Short-chain de	D42E1_HUMAN|SDR42E1|HSPC105
0006694 // ste		0003854 // 3-	Putative short	D42E2_HUMAN|SDR42E2

0055114 // ox	0005730 // nu	0004745 // re	Short-chain de	DR9C7_HUMAN|SDR9C7|RDHS|SDRO
0006465 // sig	0016021 // int	0008236 // se	Signal peptida	SC11C_HUMAN|SEC11C|SEC11L3|SPC21|SPCS4C
0048208 // CO	0005635 // nu	0005515 // pr	Protein SEC13	SEC13_HUMAN|SEC13|D3S1231E|SEC13L1|SEC13R
0065009 // re	0005737 // cy	0039552 // RI	SEC14-like pro	S14L1_HUMAN|SEC14L1|SEC14L
0045893 // po	0016021 // int	0005215 // tra	SEC14-like pro	S14L2_HUMAN|SEC14L2|C22orf6|KIAA1186|KIAA1658
0006810 // tra	0016021 // int	0005215 // tra	SEC14-like pro	S14L4_HUMAN|SEC14L4|TAP3
0006810 // tra	0016021 // int	0005215 // tra	SEC14-like pro	S14L5_HUMAN|SEC14L5|KIAA0420
0006810 // tra	0016021 // int	0005215 // tra	Putative SEC1	S14L6_HUMAN|SEC14L6
0007031 // pe	0005829 // cy		Protein transp	SC16B_HUMAN|SEC16B|KIAA1928|LZTR2|RGPR|SEC16S
0006888 // ER	0005789 // en	0005515 // pr	Vesicle-traffic	SC22B_HUMAN|SEC22B|SEC22L1
0006887 // ex	0016021 // int	0000149 // SN	Vesicle-traffic	SC22C_HUMAN|SEC22C|SEC22L3|UNQ459/PRO784
0006888 // ER	0005789 // en	0008270 // zin	Protein transp	SC23A_HUMAN|SEC23A
0006888 // ER	0005789 // en	0008270 // zin	Protein transp	SC24A_HUMAN|SEC24A
0006888 // ER	0005789 // en	0008270 // zin	Protein transp	SC24C_HUMAN|SEC24C|KIAA0079
0006620 // po	0016021 // int	0004872 // re	Translocation	SEC62_HUMAN|SEC62|TLOC1
0006955 // im	0016021 // int	0004871 // sig	Secreted and	SCTM1_HUMAN|SECTM1|K12
0007077 // m	0005635 // nu	0005515 // pr	Nucleoporin S	SEH1_HUMAN|SEH1L|SEC13L|SEH1
	0016021 // int		Protein sel-1 h	SE1L2_HUMAN|SEL1L2|C20orf50
	0016021 // int		Protein sel-1 h	SE1L3_HUMAN|SEL1L3|KIAA0746
0015031 // pr	0016020 // m	0005515 // pr	Selenium-bind	SBP1_HUMAN|SELENBP1|SBP
0006646 // ph	0016021 // int	0004307 // et	Ethanolamine	EPT1_HUMAN|SELENOI|EPT1|KIAA1724|SELI
2000406 // po	0016021 // int	0042802 // ide	Selenoprotein	SELK_HUMAN|SELENOK|SELK|HSPC030|HSPC297
0035934 // co	0005783 // en	0005515 // pr	Selenoprotein	SELM_HUMAN|SELENOM|SELM
0048741 // sk	0005789 // en	0005515 // pr	Selenoprotein	SELN_HUMAN|SELENON|SELN|SEPN1
0040007 // gr	0005576 // ex	0008430 // se	Selenoprotein	SEPP1_HUMAN|SELENOP|SELP|SEPP1
0007155 // ce	0005886 // pl	0005515 // pr	L-selectin	LYAM1_HUMAN|SELL|LNHR|LYAM1
0050901 // le	0005886 // pl	0005102 // re	P-selectin glyc	SELPL_HUMAN|SELPLG
1901166 // ne	0005576 // ex	0038191 // ne	Semaphorin-3	SEM3A_HUMAN|SEMA3A|SEMAD
0050919 // ne	0070062 // ex	0038191 // ne	Semaphorin-3	SEM3B_HUMAN|SEMA3B|SEMA5|SEMAA
0007399 // ne	0005615 // ex	0038191 // ne	Semaphorin-3	SEM3D_HUMAN|SEMA3D|UNQ760/PRO1491
0071526 // se	0005576 // ex	0038191 // ne	Semaphorin-3	SEM3E_HUMAN|SEMA3E|KIAA0331|SEMAH
0097490 // sy	0005615 // ex	0038191 // ne	Semaphorin-3	SEM3F_HUMAN|SEMA3F
0050919 // ne	0005615 // ex	0038191 // ne	Semaphorin-3	SEM3G_HUMAN|SEMA3G
0010594 // re	0016021 // int	0038191 // ne	Semaphorin-4	SEM4A_HUMAN|SEMA4A|SEMAB|SEMB|UNQ783/PRO1
0007399 // ne	0016021 // int	0038191 // ne	Semaphorin-4	SEM4B_HUMAN|SEMA4B|KIAA1745|SEMAC|UNQ749/P
0071526 // se	0045211 // po	0005515 // pr	Semaphorin-4	SEM4C_HUMAN|SEMA4C|KIAA1739|SEMAI|UNQ5855/I
0043931 // os	0005615 // ex	0030215 // se	Semaphorin-4	SEM4D_HUMAN|SEMA4D|C9orf164|CD100|SEMAJ
0007399 // ne	0016021 // int	0005515 // pr	Semaphorin-4	SEM4G_HUMAN|SEMA4G|KIAA1619
0001938 // po	0016020 // m	0038191 // ne	Semaphorin-5	SEM5A_HUMAN|SEMA5A|SEMAF
0007166 // ce	0005887 // int	0005515 // pr	Semaphorin-6	SEM6A_HUMAN|SEMA6A|KIAA1368|SEMAQ
0007399 // ne	0005887 // int	0030215 // se	Semaphorin-6	SEM6B_HUMAN|SEMA6B|SEMAN|SEMAZ|UNQ1907/PR
0007411 // ax	0005887 // int		Semaphorin-6	SEM6C_HUMAN|SEMA6C|KIAA1869|SEMAY
0048675 // ax	0031225 // an	0038191 // ne	Semaphorin-7	SEM7A_HUMAN|SEMA7A|CD108|SEMAL
0016926 // pr	0005634 // nu	0005515 // pr	Sentrin-specif	SENP5_HUMAN|SENP5|FKSG45

0016310 // pł	0005886 // pl	0004756 // se	Selenide, wat	SPS1_HUMAN|SEPHS1|SELD|SPS|SPS1
0016260 // se	0005829 // cy	0004756 // se	Selenide, wat	SPS2_HUMAN|SEPHS2|SPS2
0016259 // se	0005737 // cy	0005515 // pr	O-phosphoser	SPCS_HUMAN|SEPSECS|TRNP48
0030382 // sp	0097227 // sp	0005515 // pr	Septin-4	SEPT4_HUMAN|SEPT4|ARTS|PNUTL2|SEP4|hucep-7
0000910 // cy	0008021 // sy	0005515 // pr	Septin-5	SEPT5_HUMAN|SEPT5|PNUTL1
0000910 // cy	0030496 // m	0005525 // G	Septin-7	SEPT7_HUMAN|SEPT7|CDC10
	0005856 // cy	0005525 // G	Septin-8	SEPT8_HUMAN|SEPT8|KIAA0202
0008654 // pł	0016021 // in		Protein SERAC	SRAC1_HUMAN|SERAC1
0006658 // pł	0016021 // in	0015194 // L-	Serine incorp	SERC2_HUMAN|SERINC2|TDE2L|FKSG84|UNQ263/PRO3
0045087 // in	0005886 // pl	1905361 // L-	Serine incorp	SERC5_HUMAN|SERINC5|C5orf12
0030968 // er	0016021 // in	0005515 // pr	Stress-associa	SERP2_HUMAN|SERP2|C13orf21
0008218 // bi	0070062 // ex	0004867 // se	Protein Z-dep	ZPI_HUMAN|SERPINA10|ZPI|UNQ707/PRO1358
0002576 // pl	0034774 // se	0004867 // se	Alpha-1-anticl	AACT_HUMAN|SERPINA3|AACT|GIG24|GIG25
0007342 // fu	0002080 // ac	0005515 // pr	Plasma serine	IPSP_HUMAN|SERPINA5|PCI|PLANH3|PROCI
0010951 // ne	0031012 // ex	0004867 // se	Leukocyte ela	ILEU_HUMAN|SERPINB1|ELANH2|MNEI|PI2
0010951 // ne	0005737 // cy	0004867 // se	Serpin B11	SPB11_HUMAN|SERPINB11
0030162 // re	0043202 // lys	0002020 // pr	Serpin B13	SPB13_HUMAN|SERPINB13|PI13
0043066 // ne	0005576 // ex	0004867 // se	Plasminogen a	PAI2_HUMAN|SERPINB2|PAI2|PLANH2
0008284 // po	0005576 // ex	0004867 // se	Serpin B3	SPB3_HUMAN|SERPINB3|SCCA|SCCA1
0042270 // pr	0005622 // in	0019899 // er	Serpin B4	SPB4_HUMAN|SERPINB4|PI11|SCCA2
0002009 // m	0070062 // ex	0005515 // pr	Serpin B5	SPB5_HUMAN|SERPINB5|PI5
0010951 // ne	0070062 // ex	0004867 // se	Serpin B8	SPB8_HUMAN|SERPINB8|PI8
0006915 // ap	0005737 // cy	0004867 // se	Serpin B9	SPB9_HUMAN|SERPINB9|PI9
0010469 // re	0005576 // ex	0004867 // se	Plasminogen a	PAI1_HUMAN|SERPINE1|PAI1|PLANH1
0032940 // se	1903561 // ex	0004867 // se	Glia-derived n	GDN_HUMAN|SERPINE2|PI7|PN1
0010951 // ne	0005615 // ex	0004867 // se	Serpin E3	SERP3_HUMAN|SERPINE3
0010976 // po	0005604 // ba	0004867 // se	Pigment epith	PEDF_HUMAN|SERPINF1|PEDF|PIG35
0002034 // re	0005576 // ex	0002020 // pr	Alpha-2-antip	A2AP_HUMAN|SERPINF2|AAP|PLI
0002576 // pl	0005576 // ex	0005515 // pr	Plasma protea	IC1_HUMAN|SERPING1|C1IN|C1NH
0007417 // ce	0043025 // ne	0004867 // se	Neuroserpin	NEUS_HUMAN|SERPINI1|PI12
0006351 // tra	0005634 // nu	0005515 // pr	SERTA domair	SRTD1_HUMAN|SERTAD1|SEI1
	0005634 // nu		SERTA domair	SRTD4_HUMAN|SERTAD4
0042149 // ce	0061700 // GA	0016491 // ox	Sestrin-3	SESN3_HUMAN|SESN3|SEST3
	0016604 // nu	0005515 // pr	SET-binding p	SETBP_HUMAN|SETBP1|KIAA0437
1902036 // re	0035097 // his	0005515 // pr	Histone-lysine	SET1A_HUMAN|SETD1A|KIAA0339|KMT2F|SET1|SET1A
0018023 // pe	0000790 // nu	0042800 // his	Histone-lysine	SETD3_HUMAN|SETD3|C14orf154
0032259 // m	0005654 // nu	0016278 // lys	SET domain-co	SETD9_HUMAN|SETD9|C5orf35
0034968 // hi	0005654 // nu	0005515 // pr	Histone-lysine	SETB1_HUMAN|SETDB1|KIAA0067|KMT1E
0000737 // DI	0035861 // sit	0005515 // pr	Histone-lysine	SETMR_HUMAN|SETMAR
	0016021 // in		Seizure 6-like	SE6L2_HUMAN|SEZ6L2|PSK|UNQ1903/PRO4349
0000389 // m	0005654 // nu	0003723 // RN	Splicing factor	SF3A1_HUMAN|SF3A1|SAP114

0000389 // m	0005654 // nu	0003723 // RN	Splicing factor	SF3A3_HUMAN|SF3A3|SAP61
0000398 // m	0071011 // pr		Splicing factor	SF3B5_HUMAN|SF3B5|SF3B10
0010629 // ne	0016235 // ag	0005515 // pr	Scm-like with	SMBT2_HUMAN|SFMBT2|KIAA1617
0007165 // sig	0005829 // cy	0005515 // pr	14-3-3 protein	1433S_HUMAN|SFN|HME1
0008285 // ne	0016021 // in	0004930 // G-	Secreted frizzl	SFRP4_HUMAN|SFRP4|FRPHE
0016192 // ve	0016021 // in	0003674 // m	Vesicle transp	SFT2B_HUMAN|SFT2D2|UNQ512/PRO1027
0007585 // re	0005789 // er	0005515 // pr	Pulmonary su	SFTPD_HUMAN|SFTPD|COLEC7|PSPD|SFTP4
0034220 // io	0016021 // in	0015075 // io	Sideroflexin-2	SFXN2_HUMAN|SFXN2
0034220 // io	0005739 // m	0015075 // io	Sideroflexin-3	SFXN3_HUMAN|SFXN3
0034220 // io	0016021 // in	0015075 // io	Sideroflexin-4	SFXN4_HUMAN|SFXN4|BCRM1
0055001 // m	0005737 // cy	0005515 // pr	Delta-sarcogly	SGCD_HUMAN|SGCD
0061024 // m	0042383 // sa		Epsilon-sarcog	SGCE_HUMAN|SGCE|ESG|UNQ433/PRO840
0055001 // m	0005737 // cy	0005515 // pr	Gamma-sarco	SGCG_HUMAN|SGCG
0071169 // es	0070461 // SA	0005515 // pr	SAGA-associat	SGF29_HUMAN|SGF29|CCDC101
0006897 // er	0005829 // cy	0008017 // m	SH3-containin	SGIP1_HUMAN|SGIP1
0006468 // pr		0004674 // pr	Uncharacteriz	L7N484_HUMAN|SGK494
0016310 // ph	0030173 // in	0016301 // kir	Phosphatidylc	SMS2_HUMAN|SGMS2|SMS2
0006027 // gl	0070062 // ex	0016250 // N-	N-sulphogluco	SPHM_HUMAN|SGSH|HSS
0031338 // re	0005737 // cy	0017137 // Ra	Small G protei	SGSM2_HUMAN|SGSM2|KIAA0397|RUTBC1
1903071 // pc	0005737 // cy	0005515 // pr	Small glutami	SGTA_HUMAN|SGTA|SGT|SGT1
		0005515 // pr	Small glutami	SGTB_HUMAN|SGTB|SGT2
0007165 // sig	0005829 // cy	0005515 // pr	SH2B adapter	SH2B2_HUMAN|SH2B2|APS
0007596 // bl	0005829 // cy	0004871 // sig	SH2B adapter	SH2B3_HUMAN|SH2B3|LNK
0050776 // re	0005622 // in	0005515 // pr	SH2 domain-c	SH21B_HUMAN|SH2D1B|EAT2
0009967 // pc	0005829 // cy	0005515 // pr	SH2 domain-c	SH22A_HUMAN|SH2D2A|SCAP|TSAD|VRAP
0009967 // pc	0005622 // in	0005085 // gu	SH2 domain-c	SH23A_HUMAN|SH2D3A|NSP1|UNQ175/PRO201
0009967 // pc	0016020 // m	0005515 // pr	SH2 domain-c	SH2D3_HUMAN|SH2D3C|NSP3|UNQ272/PRO309/PRO3
0010923 // ne	0005829 // cy	0005515 // pr	SH2 domain-c	SH24A_HUMAN|SH2D4A|PPP1R38|SH2A
	0045211 // pc		SH2 domain-c	SH2D5_HUMAN|SH2D5
0009967 // pc	0005829 // cy	0005070 // SH	SH3 domain-b	SH3BG_HUMAN|SH3BGR
0045454 // ce	0016604 // nu	0015035 // pr	SH3 domain-b	SH3L3_HUMAN|SH3BGRL3|P1725
0071526 // se	0031252 // ce	0005096 // GT	SH3 domain-b	3BP1_HUMAN|SH3BP1
0007165 // sig	0005739 // m	0004860 // pr	SH3 domain-b	3BP5_HUMAN|SH3BP5|SAB
0061099 // ne	0005737 // cy	0005515 // pr	SH3 domain-b	3BP5L_HUMAN|SH3BP5L|KIAA1720|UNQ2766/PRO713
0007010 // cy	0005886 // pl	0005515 // pr	SH3 domain-c	SH319_HUMAN|SH3D19
0006897 // er	0070062 // ex	0005515 // pr	Endophilin-A3	SH3G3_HUMAN|SH3GL3|CNSA3|SH3D2C
0006897 // er	0005829 // cy	0005515 // pr	SH3 domain-c	SH3K1_HUMAN|SH3KBP1|CIN85
0071800 // pc	0005829 // cy	0005515 // pr	SH3 and PX do	SPD2A_HUMAN|SH3PXD2A|FISH|KIAA0418|SH3MD1|T
1904888 // cr	0005737 // cy	0005515 // pr	SH3 and PX do	SPD2B_HUMAN|SH3PXD2B|FAD49|KIAA1295|TKS4
0043154 // ne	0005829 // cy	0005515 // pr	E3 ubiquitin-p	SH3R1_HUMAN|SH3RF1|KIAA1494|POSH|RNF142|SH3
0010923 // ne	0005654 // nu	0005515 // pr	Putative E3 ub	SH3R2_HUMAN|SH3RF2|PPP1R39|RNF158
		0005515 // pr	SH3 domain-c	SH3R3_HUMAN|SH3RF3|POSH2|SH3MD4

0008150 // bi	0005575 // ce		SH3 domain a	S3TC1_HUMAN|SH3TC1
0033157 // re	0005886 // pl		SH3 domain a	S3TC2_HUMAN|SH3TC2|KIAA1985|PP12494
0006661 // ph	0032587 // ru	0005515 // pr	SH3 domain-c	SH3Y1_HUMAN|SH3YL1
0006461 // pr	0005886 // pl	0035255 // io	SH3 and multi	SHAN1_HUMAN|SHANK1
0060292 // lo	0045211 // po	0005515 // pr	SH3 and multi	SHAN2_HUMAN|SHANK2|CORTBP1|KIAA1022|PROSAP1
0010803 // re	0005829 // cy	0005515 // pr	Sharpin	SHRPN_HUMAN|SHARPIN|SIPL1|PSEC0216
0071363 // ce	0005829 // cy	0005515 // pr	SHC-transforn	SHC1_HUMAN|SHC1|SHC|SHCA
0043547 // po	0005829 // cy	0001784 // ph	SHC-transforn	SHC3_HUMAN|SHC3|NSHC|SHCC
0035556 // in	0045211 // po	0019904 // pr	SHC-transforn	SHC4_HUMAN|SHC4|SHCD|UNQ6438/PRO21364
0009967 // po		0005070 // SH	SH2 domain-c	SHF_HUMAN|SHF
0030178 // ne	0016021 // in		Protein shisa-	SHSA2_HUMAN|SHISA2|C13orf13|TMEM46|UNQ9166/I
0007275 // m	0016021 // in	0005515 // pr	Protein shisa-	SHSA3_HUMAN|SHISA3
0017148 // ne	0005739 // m	0005515 // pr	Serine hydrox	GLYC_HUMAN|SHMT1
0002053 // po	0005634 // nu	0043565 // se	Short stature	SHOX2_HUMAN|SHOX2|OG12X|SHOT
0050727 // re	0005737 // cy	0050277 // se	Sedoheptulok	SHPK_HUMAN|SHPK|CARKL
0000902 // ce	0005737 // cy	0051015 // ac	Protein Shroo	SHRM1_HUMAN|SHROOM1|APXL2|KIAA1960
0000902 // ce	0005913 // ce	0015280 // lig	Protein Shroo	SHRM2_HUMAN|SHROOM2|APXL
0008360 // re	0005737 // cy	0051015 // ac	Protein Shroo	SHRM3_HUMAN|SHROOM3|KIAA1481|SHRML|MSTP01
0007420 // br	0009925 // ba	0051015 // ac	Protein Shroo	SHRM4_HUMAN|SHROOM4|KIAA1202|SHAP
0007265 // Ra	0031252 // ce	0005515 // pr	Shootin-1	SHOT1_HUMAN|SHTN1|KIAA1598
0043433 // ne	0016021 // in	0005515 // pr	Single Ig IL-1-r	SIGIR_HUMAN|SIGIRR|UNQ301/PRO342
0006897 // er	0005576 // ex	0030246 // ca	Sialoadhesin	SN_HUMAN|SIGLEC1|SN
0007155 // ce	0016021 // in	0030246 // ca	Sialic acid-bin	SIG10_HUMAN|SIGLEC10|SLG2|UNQ477/PRO940
0045124 // re	0016021 // in		Sialic acid-bin	SIG15_HUMAN|SIGLEC15|CD33L3
0006357 // re	0005634 // nu	0003700 // tr	Single-mindec	SIM1_HUMAN|SIM1|BHLHE14
0009880 // er	0016604 // nu	0046982 // pr	Single-mindec	SIM2_HUMAN|SIM2|BHLHE15
		0032184 // SU	SUMO-interac	SIMC1_HUMAN|SIMC1|C5orf25
0043087 // re	0005737 // cy	0005096 // GT	Signal-inducec	SI1L1_HUMAN|SIPA1L1|E6TP1|KIAA0440
0007155 // ce	0005886 // pl	0017124 // SH	Tyrosine-prote	SHPS1_HUMAN|SIRPA|BIT|MFR|MYD1|PTPNS1|SHPS1|
0007166 // ce	0070062 // ex	0005515 // pr	Signal-regulat	SIRB1_HUMAN|SIRPB1
	0016021 // in		Signal-regulat	SIRB2_HUMAN|SIRPB2|PTPN1L|PTPNS1L3
0032007 // ne	0005654 // nu	0017136 // NA	NAD-depende	SIR1_HUMAN|SIRT1|SIR2L1
0035729 // ce	0033270 // pa	0005515 // pr	NAD-depende	SIR2_HUMAN|SIRT2|SIR2L|SIR2L2
0007568 // ag	0005739 // m	0017136 // NA	NAD-depende	SIR3_HUMAN|SIRT3|SIR2L3
0046889 // po	0005743 // m	0003950 // NA	NAD-depende	SIR4_HUMAN|SIRT4|SIR2L4
0003247 // po	0005654 // nu	0017136 // NA	NAD-depende	SIR6_HUMAN|SIRT6|SIR2L6
0046718 // vi	0005737 // cy	0005515 // pr	Apoptosis reg	SIVA_HUMAN|SIVA1|SIVA
0061055 // m	0005667 // tra	0003700 // tra	Homeobox pr	SIX1_HUMAN|SIX1
0007501 // m	0005634 // nu	0008134 // tra	Homeobox pr	SIX2_HUMAN|SIX2
0002088 // le	0005634 // nu	0005515 // pr	Homeobox pr	SIX5_HUMAN|SIX5|DMAHP
0031110 // re	0000940 // co	0005515 // pr	Spindle and ki	SKA1_HUMAN|SKA1|C18orf24
0031110 // re	0005737 // cy	0005515 // pr	Spindle and ki	SKA3_HUMAN|SKA3|C13orf3|RAMA1
0032926 // ne	0017053 // tra	0005515 // pr	Ski oncogene	SKI_HUMAN|SKI
0000398 // m	0005654 // nu	0003723 // RN	Superkiller vir	SK2L2_HUMAN|SKIV2L2|DOB1|KIAA0052|Mtr4

0006879 // ce	0005829 // cy	0005515 // pr	S-phase kinas	SKP1_HUMAN|SKP1|EMC19|OCP2|SKP1A|TCEB1L
0048661 // pc	0005829 // cy	0005515 // pr	S-phase kinas	SKP2_HUMAN|SKP2|FBXL1
		0005515 // pr	SLAIN motif-c	SLAI1_HUMAN|SLAIN1|C13orf32
0015721 // bi	0016021 // in	0008508 // bi	P3 protein	P3_HUMAN|SLC10A3|DXS253E|P3
0055085 // tr	0016021 // in	0008508 // bi	Sodium/bile a	NTCP4_HUMAN|SLC10A4
0015721 // bi	0016021 // in	0008508 // bi	Sodium/bile a	NTCP5_HUMAN|SLC10A5
0055085 // tr	0016021 // in	0015293 // sy	Sodium/bile a	NTCP7_HUMAN|SLC10A7|C4orf13|P7|PSEC0051
0071805 // pc	0005886 // pl	0015379 // pc	Solute carrier	S12A7_HUMAN|SLC12A7|KCC4
0071805 // pc	0016021 // in	0015379 // pc	Solute carrier	S12A8_HUMAN|SLC12A8|CCC9
0071805 // pc	0016021 // in	0015379 // pc	Solute carrier	S12A9_HUMAN|SLC12A9|CCC6|CIP1
0015746 // cit	0005887 // in	0015141 // su	Solute carrier	S13A3_HUMAN|SLC13A3|NADC3|SDCT2
0098656 // ar	0005886 // pl	0005215 // tr	Solute carrier	S13A4_HUMAN|SLC13A4|SUT1
0055085 // tr	0016020 // m	0050839 // ce	Urea transpor	UT2_HUMAN|SLC14A2|HUT2|UT2
0042938 // di	0005886 // pl	0005427 // pr	Solute carrier	S15A1_HUMAN|SLC15A1|PEPT1
0006857 // ol	0005886 // pl	0005515 // pr	Solute carrier	S15A2_HUMAN|SLC15A2|PEPT2
0055085 // tr	0016021 // in	0015333 // pe	Solute carrier	S15A3_HUMAN|SLC15A3|OCTP|PHT2|PTR3
1902598 // cr	0005887 // in	0015293 // sy	Monocarboxy	MOT12_HUMAN|SLC16A12|MCT12
0055085 // tr	0005887 // in	0015293 // sy	Monocarboxy	MOT14_HUMAN|SLC16A14|MCT14
0015718 // m	0005886 // pl	0015349 // th	Monocarboxy	MOT8_HUMAN|SLC16A2|MCT8|XPCT
0035879 // pl	0016021 // in	0003723 // RN	Monocarboxy	MOT4_HUMAN|SLC16A3|MCT4
0015718 // m	0005887 // in	0015293 // sy	Monocarboxy	MOT6_HUMAN|SLC16A5|MCT5|MCT6
0015718 // m	0005887 // in	0015293 // sy	Monocarboxy	MOT7_HUMAN|SLC16A6|MCT6|MCT7
1901475 // py	0005886 // pl	0015129 // la	Monocarboxy	MOT2_HUMAN|SLC16A7|MCT2
0015727 // la	0016021 // in	0008028 // m	Monocarboxy	MOT3_HUMAN|SLC16A8|MCT3
0055085 // tr	0005887 // in	0005515 // pr	Monocarboxy	MOT9_HUMAN|SLC16A9|C10orf36|MCT9
0006865 // ar	0016020 // m	0015538 // sia	Sialin	S17A5_HUMAN|SLC17A5
1903955 // pc	0016021 // in	0022857 // tr	Solute carrier	S17A9_HUMAN|SLC17A9|C20orf59
0009636 // re	0005886 // pl	0008504 // m	Synaptic vesic	VMAT2_HUMAN|SLC18A2|SVMT|VMAT2
0055085 // tr	0005887 // in	0022857 // tr	MFS-type trar	S18B1_HUMAN|SLC18B1|C6orf192
0015884 // fo	0005887 // in	0005542 // fo	Folate transpo	S19A1_HUMAN|SLC19A1|FLOT1|RFC1
0071934 // th	0016021 // in	0005515 // pr	Thiamine tran	S19A2_HUMAN|SLC19A2|THT1|TRMA
0140016 /// 0	0005887 // in	0005314 // hi	Excitatory am	EAA1_HUMAN|SLC1A3|EAAT1|GLAST|GLAST1
0015808 // L-	0005886 // pl	0015193 // L-	Neutral aminc	SATT_HUMAN|SLC1A4|ASCT1|SATT
0006868 // gl	0070062 // ex	0001618 // vi	Neutral aminc	AAAT_HUMAN|SLC1A5|ASCT2|M7V1|RDR|RDRC
0015813 // L-	0005887 // in	0005314 // hi	Excitatory am	EAA4_HUMAN|SLC1A6|EAAT4
0035435 // ph	0016020 // m	0001618 // vi	Sodium-deper	S20A2_HUMAN|SLC20A2|GLVR2|PIT2
0072488 // ar	0005886 // pl	0005515 // pr	Solute carrier	S22A1_HUMAN|SLC22A1|OCT1
0015698 // in	0009897 // ex	0005515 // pr	Solute carrier	S22AB_HUMAN|SLC22A11|OAT4
0055085 // tr	0005887 // in	0005452 // in	Solute carrier	S22AE_HUMAN|SLC22A14|OCTL2|ORCTL4
0006879 // ce	0005887 // in	0022857 // tr	Solute carrier	S22AH_HUMAN|SLC22A17|BOCT|BOIT
0008150 // bi	0005575 // ce	0003674 // m	Beckwith-Wie	BWR1B_HUMAN|SLC22A18AS|BWR1B|BWSCR1B|ORCT
0015698 // in	0005887 // in	0005452 // in	Solute carrier	S22AK_HUMAN|SLC22A20|OAT6
0015711 // or	0016021 // in	0005515 // pr	Solute carrier	S22AN_HUMAN|SLC22A23|C6orf85
0055085 // tr	0016021 // in	0005215 // tr	Solute carrier	S23A3_HUMAN|SLC23A3|E2BP3|SVCT3
0006813 // pc	0005886 // pl	0005262 // ca	Sodium/potas	NCKX2_HUMAN|SLC24A2|NCKX2
0006813 // pc	0005886 // pl	0005262 // ca	Sodium/potas	NCKX3_HUMAN|SLC24A3|NCKX3

0008272 // su	0016021 // in	0005515 // pr	Mitochondria	DIC_HUMAN|SLC25A10|DIC
0006094 // gl	0005739 // m	0003723 // RN	Mitochondria	M2OM_HUMAN|SLC25A11|SLC20A4
0015992 // pr	0005743 // m	0022857 // tra	Brain mitocho	UCP5_HUMAN|SLC25A14|BMCP1|UCP5|UNQ791/PRO1
0001561 // fa	0005778 // pe	0051724 // NA	Peroxisomal m	PM34_HUMAN|SLC25A17|PMP34
0042723 // th	0005743 // m	0015234 // th	Mitochondria	TPC_HUMAN|SLC25A19|DNC|MUP1
1902603 // ca	0005739 // m	0015227 // ac	Mitochondria	MCAT_HUMAN|SLC25A20|CAC|CACT
0089711 // L-g	0005743 // m	0005314 // hi	Mitochondria	GHC1_HUMAN|SLC25A22|GC1
0015867 // AT	0005739 // m	0005509 // ca	Calcium-bindi	SCMC1_HUMAN|SLC25A24|APC1|MCSC1|SCAMC1
0008284 // po	0005743 // m	0022857 // tra	Mitochondria	UCP4_HUMAN|SLC25A27|UCP4|UNQ772/PRO1566
1902616 // ac	0016021 // in	0015227 // ac	Mitochondria	MCATL_HUMAN|SLC25A29|C14orf69|ORNT3
0006810 // tra	0043209 // m	0032403 // pr	Phosphate ca	MPCP_HUMAN|SLC25A3|PHC|OK/SW-cl.48
0031930 // m	0016021 // in	0015218 // py	Solute carrier	S2533_HUMAN|SLC25A33
0006839 // m	0016021 // in	0022857 // tra	Solute carrier	S2534_HUMAN|SLC25A34
0006839 // m	0016021 // in	0022857 // tra	Solute carrier	S2535_HUMAN|SLC25A35
1990519 // m	0016021 // in	0015218 // py	Solute carrier	S2536_HUMAN|SLC25A36
0006783 // he	0016021 // in	0015187 // gl	Solute carrier	S2538_HUMAN|SLC25A38
0008637 // ap	0005739 // m	0005515 // pr	ADP/ATP tran	ADT1_HUMAN|SLC25A4|ANT1
0006839 // m	0016021 // in	0022857 // tra	Solute carrier	S2540_HUMAN|SLC25A40|MCFP
0015867 // AT	0016021 // in	0015217 // AD	Solute carrier	S2541_HUMAN|SLC25A41
0035349 // co	0016021 // in	0015217 // AD	Mitochondria	S2542_HUMAN|SLC25A42
0006839 // m	0005743 // m	0022857 // tra	Solute carrier	S2544_HUMAN|SLC25A44|KIAA0446
0006839 // m	0016021 // in	0000064 // L-(	Solute carrier	S2545_HUMAN|SLC25A45
0090149 // m	0005739 // m	0005515 // pr	Solute carrier	S2546_HUMAN|SLC25A46|TB1
0007059 // ch	0042645 // m	0003723 // RN	ADP/ATP tran	ADT2_HUMAN|SLC25A5|ANT2
0006839 // m	0016021 // in	0022857 // tra	Solute carrier	S2551_HUMAN|SLC25A51|MCART1
0006626 // pr	0005739 // m	0005515 // pr	ADP/ATP tran	ADT3_HUMAN|SLC25A6|ANT3|CDABP0051
0042391 // re	0005794 // Go	0015116 // su	Sodium-indep	S2611_HUMAN|SLC26A11
0001696 // ga	0005737 // cy	0005254 // ch	Anion exchan	S26A7_HUMAN|SLC26A7|SUT2
0051453 // re	0005886 // pl	0005254 // ch	Solute carrier	S26A9_HUMAN|SLC26A9
0001676 // lo	0016021 // in	0031957 // ve	Long-chain fat	S27A3_HUMAN|SLC27A3|ACSVL3|FATP3|PSEC0067|UN
0006699 // bi	0005789 // er	0004467 // lo	Bile acyl-CoA s	S27A5_HUMAN|SLC27A5|ACSB|ACSVL6|FACVL3|FATP5
0001676 // lo	0042383 // sa	0015245 // fa	Long-chain fat	S27A6_HUMAN|SLC27A6|ACSVL2|FACVL2|FATP1
1904823 // pu	0005886 // pl	0015389 // py	Sodium/nucle	S28A1_HUMAN|SLC28A1|CNT1
0006139 // nu	0005887 // in	0005337 // nu	Equilibrative n	S29A2_HUMAN|SLC29A2|DER12|ENT2|HNP36
1901642 // nu	0005794 // Go	0005515 // pr	Equilibrative n	S29A3_HUMAN|SLC29A3|ENT3|UNQ717/PRO1380
1904659 // gl	0070062 // ex	0005355 // gl	Solute carrier	GTR1_HUMAN|SLC2A1|GLUT1
1904659 // gl	0016021 // in	0055056 // D-	Solute carrier	GTR12_HUMAN|SLC2A12|GLUT12|GLUT8
1904659 // gl	0016021 // in	0005366 // m	Proton myo-in	MYCT_HUMAN|SLC2A13
0005975 // ca	0005886 // pl	0005355 // gl	Solute carrier	GTR3_HUMAN|SLC2A3|GLUT3
0005975 // ca	0012505 // en	0005355 // gl	Solute carrier	GTR4_HUMAN|SLC2A4|GLUT4
0006355 // re	0005634 // nu	0003700 // tra	SLC2A4 regula	S2A4R_HUMAN|SLC2A4RG|HDBP1
1904659 // gl	0005886 // pl	0055056 // D-	Solute carrier	GTR8_HUMAN|SLC2A8|GLUT8|GLUTX1
0046415 // ur	0005886 // pl	0022857 // tra	Solute carrier	GTR9_HUMAN|SLC2A9|GLUT9
0061088 // re	0016021 // in	0005384 // m	Zinc transport	ZNT10_HUMAN|SLC30A10|ZNT10|ZNT8
0061088 // re	0005765 // ly	0015633 // zi	Zinc transport	ZNT3_HUMAN|SLC30A3|ZNT3
0009636 // re	0005765 // ly	0005515 // pr	Zinc transport	ZNT4_HUMAN|SLC30A4|ZNT4

0034341 // re	0030141 // se	0005515 // pr	Zinc transport	ZNT8_HUMAN|SLC30A8|ZNT8
0015876 // ac	0005789 // er	0008521 // ac	Acetyl-coenzy	ACATN_HUMAN|SLC33A1|ACATN|AT1
0042493 // re	0016324 // ap	0030165 // PD	Sodium-deper	NPT2A_HUMAN|SLC34A1|NPT2|SLC17A2
0006817 // ph	0005886 // pl	0042301 // ph	Sodium-deper	NPT2B_HUMAN|SLC34A2
0030643 // ce	0031526 // br	0005436 // so	Sodium-deper	NPT2C_HUMAN|SLC34A3|NPT2C|NPTIIC
1990569 // UI	0016021 // in	0005515 // pr	UDP-N-acetyl	S35A3_HUMAN|SLC35A3
0015790 // UI	0000139 // G	0005515 // pr	UDP-xylose ar	S35B4_HUMAN|SLC35B4|YEA4|PSEC0055
0045746 // ne	0005794 // G	0005457 // GI	GDP-fucose tr	FUCT1_HUMAN|SLC35C1|FUCT1
	0016021 // in		Solute carrier	S352B_HUMAN|SLC35E2B|KIAA0447
	0016021 // in		Solute carrier	S35E3_HUMAN|SLC35E3|BLOV1|UNQ3043/PRO9859
	0016021 // in		Solute carrier	S35E4_HUMAN|SLC35E4
0008150 // bi	0016021 // in	0003674 // m	Solute carrier	S35F2_HUMAN|SLC35F2
0015888 // th	0016021 // in		Putative thian	S35F3_HUMAN|SLC35F3
0015808 // L-	0016021 // in	0015078 // hy	Proton-couple	S36A1_HUMAN|SLC36A1|PAT1
0015808 // L-	0016021 // in	0015193 // L-	Proton-couple	S36A4_HUMAN|SLC36A4|PAT4
0015760 // gl	0005789 // er	0061513 // gl	Glucose-6-ph	G6PT2_HUMAN|SLC37A1|G3PP
0015760 // gl	0005789 // er	0061513 // gl	Glucose-6-ph	G6PT3_HUMAN|SLC37A2
0003333 // ar	0005794 // G	0015171 // ar	Putative sodiu	S38AA_HUMAN|SLC38A10|PP1744
0003333 // ar	0005886 // pl	0015171 // ar	Sodium-coupl	S38A4_HUMAN|SLC38A4|ATA3|NAT3|SNAT4
0003333 // ar	0005886 // pl	0015171 // ar	Sodium-coupl	S38A5_HUMAN|SLC38A5|JM24|SN2|SNAT5|PP7194
0048701 // er	0016021 // in	0005385 // zi	Zinc transport	S39A1_HUMAN|SLC39A1|IRT1|ZIP1|ZIRTL|CGI-08|CGI-7
0071577 // zi	0005634 // nu	0005385 // zi	Zinc transport	S39AB_HUMAN|SLC39A11|C17orf26|ZIP11
0006882 // ce	0005783 // er	0005385 // zi	Zinc transport	S39AD_HUMAN|SLC39A13|ZIP13
0048701 // er	0016021 // in	0005385 // zi	Zinc transport	S39A3_HUMAN|SLC39A3|ZIP3
0007165 // si	0005887 // in	0005385 // zi	Zinc transport	S39A4_HUMAN|SLC39A4|ZIP4
0071578 // zi	0005886 // pl	0005385 // zi	Zinc transport	S39A8_HUMAN|SLC39A8|BIGM103|ZIP8|PP3105
0016049 // ce	0005886 // pl	0003723 // RI	4F2 cell-surfa	4F2_HUMAN|SLC3A2|MDU1
0006879 // ce	0005886 // pl	0097689 // irc	Solute carrier	S40A1_HUMAN|SLC40A1|FPN1|IREG1|SLC11A3|MSTP0
0035725 // so	0005886 // pl	0022892 // su	Solute carrier	S41A1_HUMAN|SLC41A1
1903830 // m	0016021 // in	0005515 // pr	Solute carrier	S41A2_HUMAN|SLC41A2
0015804 // ne	0005886 // pl	0015171 // ar	Large neutral	LAT3_HUMAN|SLC43A1|LAT3|PB39|POV1
1902475 // L-	0005887 // in	0015171 // ar	Large neutral	LAT4_HUMAN|SLC43A2|LAT4|PP7664
0055085 // tr	0016021 // in	0003674 // m	Solute carrier	S43A3_HUMAN|SLC43A3|PSEC0252
0015871 // ch	0005739 // m	0015220 // ch	Choline transp	CTL1_HUMAN|SLC44A1|CD92|CDW92|CTL1
0015871 // ch	0070062 // ex	0004871 // si	Choline transp	CTL2_HUMAN|SLC44A2|CTL2|PSEC0210
0015871 // ch	0016021 // in	0005515 // pr	Choline transp	CTL3_HUMAN|SLC44A3|CTL3|UNQ558/PRO1115
0030974 // th	0005886 // pl	0015220 // ch	Choline transp	CTL4_HUMAN|SLC44A4|C6orf29|CTL4|NG22|TPPT1|UN
0015871 // ch	0016021 // in	0015220 // ch	Choline transp	CTL5_HUMAN|SLC44A5|CTL5
0008643 // ca	0016021 // in	0015293 // sy	Proton-associ	S45A1_HUMAN|SLC45A1|DNB5
0015770 // su	0016021 // in	0008506 // su	Solute carrier	S45A4_HUMAN|SLC45A4|KIAA1126
0045580 // re	0005886 // pl	0015293 // sy	Thymic strom	TSCOT_HUMAN|SLC46A2|TSCOT
0055085 // tr	0016021 // in	0015238 // dr	Multidrug and	S47A1_HUMAN|SLC47A1|MATE1
0055085 // tr	0016021 // in	0015238 // dr	Multidrug and	S47A2_HUMAN|SLC47A2|MATE2
0015886 // he	0010008 // er	0015232 // he	Heme transpo	HRG1_HUMAN|SLC48A1|HRG1
1902600 // hy	0016021 // in	0015301 // ar	Sodium-driver	S4A10_HUMAN|SLC4A10|NCBE
0046713 // bo	0016323 // ba	0005452 // in	Sodium bicarb	S4A11_HUMAN|SLC4A11|BTR1

0015698 // in	0005925 // fo	0008509 // ar	Anion exchan	B3A2_HUMAN	SLC4A2|AE2|EPB3L1|HKB3|MPB3L
0006814 // so	0070062 // ex	0008510 // so	Electrogenic s	S4A4_HUMAN	SLC4A4|NBC|NBC1|NBCE1
0015698 // in	0016021 // in	0005452 // in	Electrogenic s	S4A5_HUMAN	SLC4A5|NBC4
0060117 // au	0032420 // st	0005452 // in	Sodium bicarb	S4A7_HUMAN	SLC4A7|BT|NBC2|NBC2B|NBC3|NBCn1|
0015698 // in	0043005 // ne	0015301 // ar	Electroneutra	S4A8_HUMAN	SLC4A8|KIAA0739|NBC|NBC3|NDCBE1
0015698 // in	0045177 // ap	0005452 // in	Anion exchan	B3A4_HUMAN	SLC4A9|AE4|SBC5
0006810 // tr	0016021 // in	0005215 // tr	Organic solut	OSTA_HUMAN	SLC51A|OSTA
0006771 // rib	0005886 // pl	0001618 // vi	Solute carrier	S52A1_HUMAN	SLC52A1|GPR172B|PAR2|RFT1
0007605 // se	0016324 // ap	0032217 // rib	Solute carrier	S52A3_HUMAN	SLC52A3|C20orf54|RFT2|RFVT3
0006814 // so	0005886 // pl	0005412 // gl	Sodium/gluco	SC5AA_HUMAN	SLC5A10|SGLT5
0006814 // so	0005887 // in	0015166 // po	Sodium/myo-	SC5AB_HUMAN	SLC5A11|KST1|SLGTX|SMIT2
0006814 // so	0005886 // pl	0015129 // la	Sodium-coupl	SC5AC_HUMAN	SLC5A12|SMCT2
1904659 // gl	0005887 // in	0005412 // gl	Sodium/gluco	SC5A2_HUMAN	SLC5A2|SGLT2
0006814 // so	0005887 // in	0005412 // gl	Solute carrier	SC5A4_HUMAN	SLC5A4|SAAT1|SGLT3
0015878 // bi	0016020 // m	0008523 // so	Sodium-deper	SC5A6_HUMAN	SLC5A6|SMVT
0006836 // ne	0005886 // pl	0005215 // tr	Sodium- and c	S6A12_HUMAN	SLC6A12
0006836 // ne	0070062 // ex	0005215 // tr	Sodium- and c	S6A13_HUMAN	SLC6A13|GAT2
0009636 // re	0031526 // br	0005215 // tr	Sodium- and c	S6A14_HUMAN	SLC6A14
0015820 // le	0005886 // pl	0005328 // ne	Sodium-deper	S6A15_HUMAN	SLC6A15|B0AT2|NTT73|SBAT1
0006836 // ne	0005622 // in	0015171 // ar	Orphan sodiu	S6A16_HUMAN	SLC6A16|NTT5
0032328 // al	0030672 // sy	0005328 // ne	Sodium-deper	S6A17_HUMAN	SLC6A17|NTT4
0006520 // ce	0016021 // in	0005369 // ta	Sodium- and c	SC6A6_HUMAN	SLC6A6
0006810 // tr	0005887 // in	0005298 // pr	Sodium-deper	SC6A7_HUMAN	SLC6A7|PROT
0006600 // cr	0016021 // in	0005308 // cr	Sodium- and c	SC6A8_HUMAN	SLC6A8
0006810 // tr	0016020 // m	0005215 // tr	Sodium- and c	SC6A9_HUMAN	SLC6A9
0006810 // tr	0005887 // in	0005515 // pr	High affinity c	CTR1_HUMAN	SLC7A1|ATRC1|ERR|REC1L
0009636 // re	0005886 // pl	0015327 // cy	Cystine/gluta	XCT_HUMAN	SLC7A11
0015804 // ne	0005887 // in	0005515 // pr	Large neutral	LAT1_HUMAN	SLC7A5|CD98LC|LAT1|MPE16
0015695 // or	0005887 // in	0015171 // ar	Large neutral	LAT2_HUMAN	SLC7A8|LAT2
0007154 // ce	0005886 // pl	0005516 // ca	Sodium/calciu	NAC2_HUMAN	SLC8A2|KIAA1087|NCX2
0006851 // m	0032592 // in	0005432 // ca	Mitochondria	NCLX_HUMAN	SLC8B1|NCKX6|NCLX|SLC24A6
0051930 // re	0005886 // pl	0030346 // pr	Sodium/hydro	SL9A1_HUMAN	SLC9A1|APNH1|NHE1
0071805 // po	0016021 // in	0015385 // so	Sodium/hydro	SL9A2_HUMAN	SLC9A2|NHE2
0008285 // ne	0005737 // cy	0005515 // pr	Na(+)/H(+) ex	NHRF1_HUMAN	SLC9A3R1|NHERF|NHERF1
0006461 // pr	0005886 // pl	0005515 // pr	Na(+)/H(+) ex	NHRF2_HUMAN	SLC9A3R2|NHERF2
0006810 // tr	0016021 // in	0015385 // so	Sodium/hydro	SL9A5_HUMAN	SLC9A5|NHE5
0071805 // po	0016021 // in	0005515 // pr	Sodium/hydro	SL9A9_HUMAN	SLC9A9|NHE9|Nbla00118
0030317 // fla	0005886 // pl	0015385 // so	Sodium/hydro	SL9B1_HUMAN	SLC9B1|NHA1|NHEDC1
0007283 // sp	0016021 // in	0005216 // io	Sodium/hydro	SL9C1_HUMAN	SLC9C1|SLC9A10
0015721 // bi	0005887 // in	0008514 // or	Solute carrier	SO1A2_HUMAN	SLCO1A2|OATP|OATP1|OATP1A2|SLC2
0015721 // bi	0005887 // in	0015125 // bi	Solute carrier	SO1B1_HUMAN	SLCO1B1|LST1|OATP1B1|OATP2|OATP
0015721 // bi	0005886 // pl	0015125 // bi	Solute carrier	SO1B3_HUMAN	SLCO1B3|LST2|OATP1B3|OATP8|SLC21
0015721 // bi	0005887 // in	0015125 // bi	Putative solut	SO1B7_HUMAN	SLCO1B7|LST3|LST3TM12
0015721 // bi	0005887 // in	0015347 // so	Solute carrier	SO2B1_HUMAN	SLCO2B1|KIAA0880|OATP2B1|OATPB|
0015732 // pr	0005887 // in	0015347 // so	Solute carrier	SO3A1_HUMAN	SLCO3A1|OATP3A1|OATPD|SLC21A11
0070327 // th	0005887 // in	0015349 // th	Solute carrier	SO4A1_HUMAN	SLCO4A1|OATP1|OATP4A1|OATPE|SLC

0007283 // sp	0005887 // in	0015347 // so	Solute carrier	SO4C1_HUMAN|SLCO4C1|OATP4C1|OATPX|SLC21A20
0043252 // so	0005887 // in	0015347 // so	Solute carrier	SO5A1_HUMAN|SLCO5A1|OATP5A1|SLC21A15
0043252 // so	0005887 // in	0015347 // so	Solute carrier	SO6A1_HUMAN|SLCO6A1|OATP6A1|SLC21A19
0031334 // pc	0005634 // nu	0032403 // pr	SMC5-SMC6 c	SLF2_HUMAN|SLF2|C10orf6|FAM178A
0051607 // de	0005634 // nu	0000049 // tR	Schlafen famil	SLN11_HUMAN|SLFN11
		0005524 // AT	Schlafen famil	SLN12_HUMAN|SLFN12
	0005622 // in	0005524 // AT	Schlafen famil	SLN13_HUMAN|SLFN13
0090502 // RI	0005634 // nu	0043022 // rik	Protein SLFN1	SLN14_HUMAN|SLFN14
0030154 // ce	0005634 // nu	0005524 // AT	Schlafen famil	SLFN5_HUMAN|SLFN5
0030317 // fla	0005739 // m	0003723 // RI	SRA stem-loop	SLIRP_HUMAN|SLIRP|C14orf156|DC23|DC50|PD04872
0048846 // ax	0005615 // ex	0048495 // Rc	Slit homolog 1	SLIT1_HUMAN|SLIT1|KIAA0813|MEGF4|SLIL1
0008045 // m	0005615 // ex	0048495 // Rc	Slit homolog 2	SLIT2_HUMAN|SLIT2|SLIL3
0007409 // ax	0016021 // in		SLIT and NTRK	SLIK3_HUMAN|SLITRK3|KIAA0848
0007409 // ax	0016021 // in		SLIT and NTRK	SLIK4_HUMAN|SLITRK4
0007625 // gr	0016021 // in		SLIT and NTRK	SLIK5_HUMAN|SLITRK5|KIAA0918|LRRC11
0007601 // vi	0009986 // ce		SLIT and NTRK	SLIK6_HUMAN|SLITRK6
0045087 // in	0005794 // Go	0003729 // m	Antileukoprot	SLPI_HUMAN|SLPI|WAP4|WFDC4
0000380 // al	0005737 // cy	0000386 // se	Pre-mRNA-spl	SLU7_HUMAN|SLU7
0060395 // SI	0005622 // in	0005515 // pr	Mothers agair	SMAD1_HUMAN|SMAD1|BSP1|MADH1|MADR1
0010718 // pc	0005829 // cy	0005515 // pr	Mothers agair	SMAD3_HUMAN|SMAD3|MADH3
0007183 // SI	0005634 // nu	0070412 // R-	Mothers agair	SMAD4_HUMAN|SMAD4|DPC4|MADH4
0001657 // ur	0005667 // tra	0070410 // cc	Mothers agair	SMAD6_HUMAN|SMAD6|MADH6
2000320 // ne	0005634 // nu	0005515 // pr	Mothers agair	SMAD7_HUMAN|SMAD7|MADH7|MADH8
0007179 // tra	0005737 // cy	0005515 // pr	Mothers agair	SMAD9_HUMAN|SMAD9|MADH6|MADH9|SMAD8
0007156 // hc	0005913 // ce	0050839 // ce	Small cell adh	SMAGP_HUMAN|SMAGP
0030308 // ne	0071564 // np	0008094 // DI	Probable glob	SMCA2_HUMAN|SMARCA2|BAF190B|BRM|SNF2A|SNF2
2000134 // ne	0071564 // np	0005515 // pr	Transcription	SMCA4_HUMAN|SMARCA4|BAF190A|BRG1|SNF2B|SNF
1900113 // ne	0000790 // nu	0003713 // tra	SWI/SNF-relat	SNF5_HUMAN|SMARCB1|BAF47|INI1|SNF5L1
0006338 // ch	0071564 // np	0008134 // tra	SWI/SNF-relat	SMRD3_HUMAN|SMARCD3|BAF60C
0007062 // sis	0000800 // la	0003677 // DI	Structural ma	SMC1B_HUMAN|SMC1B|SMC1L2
0051383 // ki	0005634 // nu	0005515 // pr	Structural ma	SMC4_HUMAN|SMC4|CAPC|SMC4L1
	0016021 // in		Single-pass m	SMCO2_HUMAN|SMCO2|C12orf70
0043547 // pc	0000785 // ch	0017112 // Ra	Guanine nucle	SMCR8_HUMAN|SMCR8
0090501 // RI	0005829 // cy	0005515 // pr	Protein SMG7	SMG7_HUMAN|SMG7|C1orf16|EST1C|KIAA0250
	0016021 // in		Small integral	SIM10_HUMAN|SMIM10|CXorf69
	0016021 // in		Small integral	SIML1_HUMAN|SMIM10L1
0008150 // bi	0016021 // in	0003674 // m	Small integral	SI11A_HUMAN|SMIM11A|C21orf51|FAM165B|SMIM11
	0016021 // in		Small integral	SIM13_HUMAN|SMIM13|C6orf228

	0016021 // in	0005515 // pr	Small integral	SIM14_HUMAN|SMIM14|C4orf34
	0016021 // in		Small integral	SIM15_HUMAN|SMIM15|C5orf43
	0016021 // in		Small integral	SIM17_HUMAN|SMIM17
	0016021 // in		Small integral	SMI19_HUMAN|SMIM19|C8orf40
	0016021 // in		Small integral	SIM22_HUMAN|SMIM22
	0016021 // in		Small integral	SIM23_HUMAN|SMIM23|C5orf50
	0016021 // in	0005515 // pr	Small integral	SMIM3_HUMAN|SMIM3|C5orf62|NID67
	0016021 // in		Small integral	SMIM5_HUMAN|SMIM5|C17orf109
	0016021 // in	0005515 // pr	Small leucine-	SMLR1_HUMAN|SMLR1
0060070 // ca	0005886 // pl	0004930 // G-	Smoothened	SMO_HUMAN|SMO|SMOH
0001654 // ey	0005604 // ba	0005515 // pr	SPARC-relatec	SMOC1_HUMAN|SMOC1
0006598 // po	0031965 // nu	0052901 // sp	Spermine oxic	SMOX_HUMAN|SMOX|C20orf16|SMO|UNQ3039/PRO9
0007399 // ne	0005768 // en	0016798 // hy	Sphingomyeli	ASM_HUMAN|SMPD1|ASM
0006685 // sp	0000137 // Go	0004767 // sp	Sphingomyeli	NSMA2_HUMAN|SMPD3
0046475 // gl	0005783 // en	0004767 // sp	Sphingomyeli	NSMA3_HUMAN|SMPD4|KIAA1418
0009143 // nu	0070062 // ex	0008081 // ph	Acid sphingon	ASM3A_HUMAN|SMPDL3A|ASML3A
0034122 // ne	0031225 // an	0016798 // hy	Acid sphingon	ASM3B_HUMAN|SMPDL3B|ASML3B
0008150 // bi	0070062 // ex	0005515 // pr	Submaxillary g	SMR3B_HUMAN|SMR3B|PBII|PRL3|PROL3
0006939 // sn	0005737 // cy	0008307 // st	Smoothelin	SMTN_HUMAN|SMTN|SMSMO
0045907 // po	0005737 // cy	0005516 // ca	Smoothelin-lil	SMTL1_HUMAN|SMTNL1
0000381 // re	0016607 // nu	0005515 // pr	WD40 repeat-	SMU1_HUMAN|SMU1
0060071 // W	0005829 // cy	0005515 // pr	E3 ubiquitin-p	SMUF2_HUMAN|SMURF2
0043516 // re	0005634 // nu	0002039 // p5	N-lysine meth	SMYD2_HUMAN|SMYD2|KMT3C
0006334 // nu	0005654 // nu	0001162 // RN	Histone-lysine	SMYD3_HUMAN|SMYD3|ZMYND1|ZNFN3A1
0032259 // m		0008168 // m	SET and MYNI	SMYD4_HUMAN|SMYD4|KIAA1936
0060536 // ca	0005634 // nu	0005515 // pr	Zinc finger pro	SNAI1_HUMAN|SNAI1|SNAH
0007219 // No	0005634 // nu	0003682 // ch	Zinc finger pro	SNAI2_HUMAN|SNAI2|SLUG|SLUGH
0006351 // tra	0005667 // tra	0005507 // co	Zinc finger pro	SNAI3_HUMAN|SNAI3|ZNF293
0060291 // lo	0005886 // pl	0005515 // pr	Synaptosomal	SNP25_HUMAN|SNAP25|SNAP
0016082 // sy	0000139 // Go	0005484 // SN	Synaptosomal	SNP29_HUMAN|SNAP29
0048268 // cla	0005905 // cla	0005545 // 1-	Clathrin coat a	AP180_HUMAN|SNAP91|KIAA0656
0006383 // tra	0005654 // nu	0003700 // tra	snRNA-activat	SNPC2_HUMAN|SNAPC2|SNAP45
0042795 // sn	0005654 // nu	0044212 // tra	snRNA-activat	SNPC4_HUMAN|SNAPC4|SNAP190
0032026 // re	0005829 // cy	0030544 // Hs	Alpha-synucle	SYUA_HUMAN|SNCA|NACP|PARK1
0042417 // do	0005829 // cy	0005515 // pr	Synphilin-1	SNCAP_HUMAN|SNCAIP
0008344 // ac	0043025 // ne	0005515 // pr	Gamma-synuc	SYUG_HUMAN|SNCG|BCSG1|PERSYN|PRSN

0009636 // re	0005741 // m	0046872 // m	Stannin	SNN_HUMAN|SNN
0016081 // sy	0016021 // in	0017075 // sy	Syntaphilin	SNPH_HUMAN|SNPH|KIAA0374
0030099 // m	0005634 // nu	0005524 // AT	SNF-related se	SNRK_HUMAN|SNRK|KIAA0096|SNFRK
0000398 // m	0005634 // nu	0005515 // pr	U11/U12 sma	SNR25_HUMAN|SNRNP25|C16orf33
0000398 // m	0005654 // nu	0005515 // pr	U2 small nucle	RU2B_HUMAN|SNRPB2
0000398 // m	0005654 // nu	0003723 // RN	Small nuclear	SMD1_HUMAN|SNRPD1
0000387 // sp	0005654 // nu	0005515 // pr	Small nuclear	RUXF_HUMAN|SNRPF|PBSCF
0000398 // m	0005654 // nu	0003723 // RN	Small nuclear	RSMN_HUMAN|SNRPN|HCERN3|SMN
0086005 // ve	0016013 // sy	0005515 // pr	Alpha-1-syntro	SNTA1_HUMAN|SNTA1|SNT1
0007154 // ce	0005737 // cy	0008022 // pr	Gamma-1-syn	SNTG1_HUMAN|SNTG1
0007417 // ce	0042383 // sa	0097109 // ne	Gamma-2-syn	SNTG2_HUMAN|SNTG2
0008150 // bi	0005634 // nu	0003674 // m	SNRPN upstre	SNURF_HUMAN|SNURF
0070562 // re	0016363 // nu	0005515 // pr	SNW domain-	SNW1_HUMAN|SNW1|SKIIP|SKIP
0060271 // cil	0005634 // nu	0005545 // 1-	Sorting nexin-	SNX10_HUMAN|SNX10
0006886 // in	0005730 // nu	0035091 // ph	Sorting nexin-	SNX15_HUMAN|SNX15
0006622 // pr	0031313 // ex	0035091 // ph	Sorting nexin-	SNX16_HUMAN|SNX16
0036089 // cle	0031234 // ex	0005515 // pr	Sorting nexin-	SNX18_HUMAN|SNX18|SH3PXD3B|SNAG1
0016050 // ve	0016020 // m	0005515 // pr	Sorting nexin-	SNX2_HUMAN|SNX2|TRG9
0015031 // pr	0030659 // cy	0005546 // ph	Sorting nexin-	SNX21_HUMAN|SNX21|C20orf161|SNXL
0015031 // pr	0030659 // cy	0010314 // ph	Sorting nexin-	SNX24_HUMAN|SNX24|SBBI31|UNQ654/PRO1284
0032801 // re	0043231 // in	0034713 // ty	Sorting nexin-	SNX25_HUMAN|SNX25|MSTP043
0016050 // ve	0005768 // en	0005515 // pr	Sorting nexin-	SNX30_HUMAN|SNX30
0006886 // in	0043234 // pr	0035091 // ph	Sorting nexin-	SNX31_HUMAN|SNX31
0097320 // m	0031410 // cy	0042802 // ide	Sorting nexin-	SNX33_HUMAN|SNX33|SH3PX3|SH3PXD3C|SNX30
0016050 // ve	0016020 // m	1990459 // tra	Sorting nexin-	SNX4_HUMAN|SNX4
0016050 // ve	0005737 // cy	0005515 // pr	Sorting nexin-	SNX6_HUMAN|SNX6
0006886 // in	0031410 // cy	0071933 // Ar	Sorting nexin-	SNX9_HUMAN|SNX9|SH3PX1|SH3PXD3A
0034435 // ch	0016021 // in	0015485 // ch	Sterol O-acylt	SOAT2_HUMAN|SOAT2|ACACT2|ACAT2
0007605 // se	0005634 // nu	0032184 // SU	Sine oculis-bir	SOBP_HUMAN|SOBP|JXC1
0009967 // po	0005737 // cy	0004860 // pr	Suppressor of	SOCS2_HUMAN|SOCS2|CIS2|SSI2|STATI2
0042532 // ne	0005829 // cy	0004860 // pr	Suppressor of	SOCS3_HUMAN|SOCS3|CIS3|SSI3
0097699 // va	0005737 // cy	0004860 // pr	Suppressor of	SOCS5_HUMAN|SOCS5|CIS6|CISH5|CISH6|KIAA0671
0007259 // JA	0005737 // cy	0004860 // pr	Suppressor of	SOCS6_HUMAN|SOCS6|CIS4|SOCS4
0000303 // re	0005759 // m	0004784 // su	Superoxide di	SODM_HUMAN|SOD2
0019430 // re	0005576 // ex	0004784 // su	Extracellular s	SODE_HUMAN|SOD3
0007283 // sp	0005634 // nu	0001046 // co	Spermatogene	SOLH2_HUMAN|SOHLH2|TEB1
0000226 // m	0016607 // nu	0003723 // RN	Protein SON	SON_HUMAN|SON|C21orf50|DBP5|KIAA1019|NREBP|H
0009967 // po	0001725 // st	0005515 // pr	Sorbin and SH	SRBS1_HUMAN|SORBS1|KIAA0894|KIAA1296|SH3D5

0007155 // ce	0016324 // ap	0003723 // RN	Sorbin and SH	SRBS2_HUMAN|SORBS2|ARGBP2|KIAA0777
0007218 // ne	0016021 // int	0008188 // ne	VPS10 domain	SORC1_HUMAN|SORCS1|SORCS
0007218 // ne	0016021 // int	0008188 // ne	VPS10 domain	SORC2_HUMAN|SORCS2|KIAA1329
0045053 // pr	0055037 // re	0001540 // be	Sortilin-relate	SORL_HUMAN|SORL1|C11orf32
0048011 // ne	0005794 // Go	0030379 // ne	Sortilin	SORT_HUMAN|SORT1
0007389 // pa	0005615 // ex	0036122 // BN	Sclerostin dor	SOSD1_HUMAN|SOSTDC1|USAG1|CDA019
0021510 // sp	0032993 // pr	0001105 // RN	Transcription	SOX12_HUMAN|SOX12|SOX22
0045586 // re	0005654 // nu	0043565 // se	Transcription	SOX13_HUMAN|SOX13
0000122 // ne	0005634 // nu	0001077 // tra	Transcription	SOX18_HUMAN|SOX18
0010468 // re	0005654 // nu	0003677 // DN	Transcription	SOX2_HUMAN|SOX2
0045893 // po	0005739 // m	0001046 // co	Transcription	SOX4_HUMAN|SOX4
0006366 // tra	0005634 // nu	0005515 // pr	Transcription	SOX5_HUMAN|SOX5
0001706 // en	0005634 // nu	0003700 // tra	Transcription	SOX7_HUMAN|SOX7
0002053 // po	0005634 // nu	0001158 // en	Transcription	SOX9_HUMAN|SOX9
1904828 // po	0005654 // nu	0000978 // RN	Transcription	SP1_HUMAN|SP1|TSFP1
	0016604 // nu	0003677 // DN	Nuclear body	SP14L_HUMAN|SP140L
0006357 // re	0005829 // cy	0005515 // pr	Transcription	SP4_HUMAN|SP4
0071407 // ce	0005634 // nu	0000981 // RN	Transcription	SP5_HUMAN|SP5
0042481 // re	0005829 // cy	0000981 // RN	Transcription	SP6_HUMAN|SP6|KLF14
0009953 // do	0005634 // nu	0000981 // RN	Transcription	SP8_HUMAN|SP8
0030326 // en	0005634 // nu	0000981 // RN	Transcription	SP9_HUMAN|SP9
0007342 // fu	0016021 // int	0005515 // pr	Sperm acroso	SACA6_HUMAN|SPACA6|SPACA6P|UNQ2487/PRO5774
	0001669 // ac	0005515 // pr	Sperm acroso	SACA9_HUMAN|SPACA9|C9orf9
0070286 // ax	0005737 // cy	0005525 // GT	Sperm-associa	SPAG1_HUMAN|SPAG1
0003351 // ep	0031514 // m		Sperm-associa	SPG17_HUMAN|SPAG17
0090286 // cy	0005737 // cy	0005198 // str	Sperm-associa	SPAG4_HUMAN|SPAG4|SUN4
0030030 // ce	0005737 // cy		Sperm-associa	SPAG6_HUMAN|SPAG6|PF16
0043065 // po	0005739 // m	0008017 // m	Putative unch	SPOT1_HUMAN|SPANXA2-OT1|CXorf18
0022604 // re	0009986 // ce	0050840 // ex	SPARC	SPRC_HUMAN|SPARC|ON
0007165 // sig	0005788 // en	0050840 // ex	SPARC-like pro	SPRL1_HUMAN|SPARCL1
	0005737 // cy	0005516 // ca	Spermatogene	SPT17_HUMAN|SPATA17
0035694 // m	0043231 // int	0005515 // pr	Mitochondria-	MIEAP_HUMAN|SPATA18|MIEAP
0007283 // sp	0048471 // pe	0003779 // ac	Spermatogene	SPT32_HUMAN|SPATA32|C17orf46|TEX34
	0005737 // cy	0005515 // pr	Spermatogene	SPAT4_HUMAN|SPATA4|TSARG2
0007283 // sp	0031965 // nu	0005515 // pr	Spermatogene	SPT46_HUMAN|SPATA46|C1orf111|HSD20
0050896 // re	0015630 // m	0005515 // pr	Spermatogene	SPAT7_HUMAN|SPATA7|HSD3

	0005737 // cy		Speriolin	SPERI_HUMAN|SPATC1|SPATA15|SPRN
0008150 // bi	0005813 // ce	0005515 // pr	Speriolin-like	SPC1L_HUMAN|SPATC1L|C21orf56
0007052 // m	0031262 // Nc	0005515 // pr	Kinetochore p	SPC25_HUMAN|SPC25|SPBC25|AD024
0045047 // pr	0016021 // in	0008233 // pe	Signal peptida	SPCS2_HUMAN|SPCS2|KIAA0102|SPC25
0006465 // sig	0016021 // in	0008233 // pe	Signal peptida	SPCS3_HUMAN|SPCS3|SPC22|UNQ1841/PRO3567
0034501 // pr	0005829 // cy	0005515 // pr	Protein Spindl	SPDLY_HUMAN|SPDL1|CCDC99
0007275 // m	0005634 // nu	0019901 // pr	Speedy protei	SPDYA_HUMAN|SPDYA|SPDY1|SPY1
0007049 // ce	0005737 // cy	0019901 // pr	Speedy protei	SPDYC_HUMAN|SPDYC
0045859 // re		0019901 // pr	Speedy protei	SPDE1_HUMAN|SPDYE1|WBSCR19
0045859 // re		0019901 // pr	Putative spee	SPD16_HUMAN|SPDYE16
0045859 // re		0019901 // pr	Speedy protei	SPDE2_HUMAN|SPDYE2
0045859 // re		0019901 // pr	Speedy protei	SPE2B_HUMAN|SPDYE2B
0045859 // re		0019901 // pr	Speedy protei	SPDE3_HUMAN|SPDYE3
0045859 // re		0019901 // pr	Putative spee	SPDE5_HUMAN|SPDYE5
0045859 // re		0019901 // pr	Putative spee	SPDE6_HUMAN|SPDYE6
	0016020 // m		Cytospin-B	CYTSB_HUMAN|SPECC1|CYTSB|NSP5
0051301 // ce	0005829 // cy		Cytospin-A	CYTSA_HUMAN|SPECC1L|CYTSA|KIAA0376
0007026 // ne	0005930 // ax	0008017 // m	Sperm flagella	SPEF1_HUMAN|SPEF1|C20orf28
0008285 // ne	0005634 // nu	0004674 // pr	Striated musc	SPEG_HUMAN|SPEG|APEG1|KIAA1297
	0031410 // cy	0005515 // pr	Spermatid-ass	SPERT_HUMAN|SPERT|CBY2
0007340 // ac	0001669 // ac		Sperm equato	SPESP_HUMAN|SPESP1|UNQ732/PRO1418
0007268 // ch	0005737 // cy	0005515 // pr	Spatacsin	SPTCS_HUMAN|SPG11|KIAA1840
0045931 // pc	0005829 // cy	0005515 // pr	Sphingosine k	SPHK1_HUMAN|SPHK1|SPHK|SPK
0007565 // fe	0005829 // cy	0017050 // D-	Sphingosine k	SPHK2_HUMAN|SPHK2
0051726 // re	0005829 // cy	0003674 // m	Spindlin-2A	SPI2A_HUMAN|SPIN2A|DXF34|SPIN2
0035315 // ha	0048471 // pe	0004867 // se	Serine proteas	ISK5_HUMAN|SPINK5
2000178 // ne	0005886 // pl	0004867 // se	Kunitz-type pr	SPIT1_HUMAN|SPINT1|HAI1|UNQ223/PRO256
2000146 // ne	0016021 // in	0004866 // er	Kunitz-type pr	SPIT2_HUMAN|SPINT2|HAI2|KOP
0040038 // pc	0005829 // cy	0003779 // ac	Protein spire I	SPIR1_HUMAN|SPIRE1|KIAA1135|SPIR1
0070649 // fo	0032154 // cl	0003779 // ac	Protein spire I	SPIR2_HUMAN|SPIRE2|KIAA1832|SPIR2
0003376 // sp	0005765 // ly	0046624 // sp	Protein spinst	SPNS2_HUMAN|SPNS2
0003376 // sp	0005765 // ly	0046624 // sp	Protein spinst	SPNS3_HUMAN|SPNS3
0022008 // ne	0014069 // pc	0008191 // m	Testican-1	TICN1_HUMAN|SPOCK1|SPOCK|TIC1|TICN1
0007165 // sig	0005578 // pr	0005539 // gl	Testican-3	TICN3_HUMAN|SPOCK3|TICN3|UNQ409/PRO771
0007155 // ce	0070062 // ex	0003823 // ar	Spondin-2	SPON2_HUMAN|SPON2|DIL1|UNQ435/PRO866
0007566 // er	0005794 // Gc	0050840 // ex	Osteopontin	OSTP_HUMAN|SPP1|BNSP|OPN|PSEC0156
0006509 // m	0005770 // la	0042500 // as	Signal peptide	SPP2A_HUMAN|SPPL2A|IMP3|PSL2|PSEC0147
0006809 // ni	0005829 // cy	0004757 // se	Sepiapterin re	SPRE_HUMAN|SPR
0010801 // ne	0005886 // pl	0005173 // st	Sprouty-relate	SPRE1_HUMAN|SPRED1
0009966 // re	0016020 // m		Sprouty-relate	SPRE3_HUMAN|SPRED3|EVE-3
0098773 // sk	0005829 // cy	0005515 // pr	Cornifin-A	SPR1A_HUMAN|SPRR1A
0018149 // pe	0005829 // cy	0005198 // st	Cornifin-B	SPR1B_HUMAN|SPRR1B
0030216 // ke	0005829 // cy	0005515 // pr	Small proline-	SPR2A_HUMAN|SPRR2A

0042060 // w	0005829 // cy	0005198 // st	Small proline-	SPRR3_HUMAN|SPRR3|SPRC
0040037 // ne	0005794 // Go	0005515 // pr	Protein sprout	SPY1_HUMAN|SPRY1
0045595 // re	0005886 // pl	0005515 // pr	Protein sprout	SPY2_HUMAN|SPRY2
0061564 // ax	0016020 // m	0003674 // m	Protein sprout	SPY3_HUMAN|SPRY3
0007275 // m	0005925 // fo	0005515 // pr	Protein sprout	SPY4_HUMAN|SPRY4
0008150 // bi	0005634 // nu	0003674 // m	SPRY domain-	SPRY4_HUMAN|SPRYD4
0035556 // in	0005829 // cy	0005515 // pr	SPRY domain-	SPSB2_HUMAN|SPSB2|GRCC9|SSB2
0043547 // po	0005829 // cy	0030506 // ar	Spectrin beta	SPTB1_HUMAN|SPTB|SPTB1
0043547 // po	0005829 // cy	0005200 // st	Spectrin beta	SPTN2_HUMAN|SPTBN2|KIAA0302|SCA5
0043547 // po	0005829 // cy	0034452 // dy	Spectrin beta	SPTN5_HUMAN|SPTBN5|BSPECV|HUBSPECV|HUSPECV
1904504 // po	0016021 // in	0030170 // py	Serine palmito	SPTC1_HUMAN|SPTLC1|LCB1
0046520 // sp	0016021 // in	0030170 // py	Serine palmito	SPTC3_HUMAN|SPTLC3|C20orf38|SPTLC2L
0046513 // ce	0016021 // in	0004758 // se	Serine palmito	SPTSA_HUMAN|SPTSSA|C14orf147|SSSPTA
0007029 // en	0016021 // in	0004758 // se	Serine palmito	SPTSB_HUMAN|SPTSSB|ADMP|C3orf57|SSSPTB
	0016021 // in		SPTY2D1 antis	A0A0U1RR47_HUMAN|SPTY2D1-AS1
0044539 // lo	0005615 // ex	0005184 // ne	Spexin	SPXN_HUMAN|SPX|C12orf39
0016126 // st	0016021 // in	0004506 // sq	Squalene mor	ERG1_HUMAN|SQLE|ERG1
0008283 // ce	0005737 // cy	0005515 // pr	Steroid recept	SRA1_HUMAN|SRA1|PP7684
0006702 // ar	0043209 // m	0009055 // el	3-oxo-5-alpha	S5A1_HUMAN|SRD5A1
0006702 // ar	0005789 // er	0016628 // ox	Polyprenol re	PORED_HUMAN|SRD5A3|SRD5A2L
0030522 // in	0005737 // cy	0003677 // DI	Sterol regulat	SRBP1_HUMAN|SREBF1|BHLHD1|SREBP1
0072368 // re	0005789 // er	0008022 // pr	Sterol regulat	SRBP2_HUMAN|SREBF2|BHLHD2|SREBP2
0006351 // tr	0005634 // nu	0003723 // RI	Serum respon	SRFB1_HUMAN|SRFBP1
0060548 // ne	0005829 // cy	0005096 // G	SLIT-ROBO Rh	SRGP2_HUMAN|SRGAP2|ARHGAP34|FNBP2|KIAA0456|
0007399 // ne	0005737 // cy	0005096 // G	SLIT-ROBO Rh	SRG2B_HUMAN|SRGAP2B|SRGAP2P2
0043547 // po	0005737 // cy	0005096 // G	SLIT-ROBO Rh	SRG2C_HUMAN|SRGAP2C|SRGAP2P1
0043547 // po	0005829 // cy	0005515 // pr	SLIT-ROBO Rh	SRGP3_HUMAN|SRGAP3|ARHGAP14|KIAA0411|KIAA11
0002576 // pl	0005576 // ex	0005515 // pr	Serglycin	SRGN_HUMAN|SRGN|PRG|PRG1
0006595 // po	0005829 // cy	0004766 // sp	Spermidine sy	SPEE_HUMAN|SRM|SPS1|SRML1
0009968 // ne	0005829 // cy	0004715 // no	Tyrosine-prot	SRMS_HUMAN|SRMS|C20orf148
0042493 // re	0005829 // cy	0003723 // RI	Signal recogni	SRP19_HUMAN|SRP19
0043525 // po	0005730 // nu	0003723 // RI	SRSF protein k	SRPK2_HUMAN|SRPK2
0006468 // pr	0005634 // nu	0005515 // pr	SRSF protein k	SRPK3_HUMAN|SRPK3|MSSK1|STK23
0007155 // ce	0016020 // m		Sushi repeat-c	SRPX_HUMAN|SRPX|ETX1
0070179 // D-	0045177 // ap	0030170 // py	Serine racema	SRR_HUMAN|SRR
0048511 // rh			SRR1-like prot	SRR1L_HUMAN|SRRD|SRR1L
0031124 // m	0005654 // nu	0003723 // RI	Serine/argini	SRSF6_HUMAN|SRSF6|SFRS6|SRP55
0008380 // RI	0005829 // cy	0003723 // RI	Serine/argini	SRSF8_HUMAN|SRSF8|SFRS2B|SRP46
0035556 // in	0071564 // np	0005515 // pr	Protein SSXT	SSXT_HUMAN|SS18|SSXT|SYT
0051096 // po	0042645 // m	0003723 // RI	Single-strande	SSBP_HUMAN|SSBP1|SSBP
0006366 // tr	0005634 // nu	0001077 // tr	Single-strande	SSBP2_HUMAN|SSBP2|SSDP2
0006366 // tr	0005634 // nu	0001077 // tr	Single-strande	SSBP4_HUMAN|SSBP4

<table>
<tr><td>0006898 // re</td><td>0070062 // ex</td><td>0005044 // sc</td><td>Scavenger rec</td><td>SRB4D_HUMAN|SSC4D|SRCRB4D</td></tr>
<tr><td>0042494 // de</td><td>0005622 // in</td><td>0005515 // pr</td><td>Soluble scave</td><td>SRCRL_HUMAN|SSC5D</td></tr>
<tr><td></td><td>0005829 // cy</td><td>0051015 // ac</td><td>Sperm-specifi</td><td>SSFA2_HUMAN|SSFA2|CS1|KIAA1927|KRAP</td></tr>
<tr><td>1901216 // po</td><td>0030426 // gr</td><td>0005515 // pr</td><td>Protein phosp</td><td>SSH1_HUMAN|SSH1|KIAA1298|SSH1L</td></tr>
<tr><td>0010591 // re</td><td>0005737 // cy</td><td>0004725 // pr</td><td>Protein phosp</td><td>SSH3_HUMAN|SSH3|SSH3L</td></tr>
<tr><td>0000086 // G2</td><td>0005829 // cy</td><td>0005515 // pr</td><td>Sjoegren synd</td><td>SSNA1_HUMAN|SSNA1|NA14</td></tr>
<tr><td>0007155 // ce</td><td>0005887 // in</td><td></td><td>Sarcospan</td><td>SSPN_HUMAN|SSPN|KRAG</td></tr>
<tr><td>0006614 // SR</td><td>0016021 // in</td><td></td><td>Translocon-as</td><td>SSRG_HUMAN|SSR3|TRAPG</td></tr>
<tr><td></td><td>0005784 // Se</td><td></td><td>Translocon-as</td><td>SSRD_HUMAN|SSR4|TRAPD</td></tr>
<tr><td></td><td></td><td></td><td></td><td></td></tr>
<tr><td></td><td></td><td></td><td></td><td></td></tr>
<tr><td>0007155 // ce</td><td>0043234 // pr</td><td>0005515 // pr</td><td>Afadin- and al</td><td>ADIP_HUMAN|SSX2IP|KIAA0923</td></tr>
<tr><td>0006508 // pr</td><td>0016323 // ba</td><td>0004252 // se</td><td>Suppressor of</td><td>ST14_HUMAN|ST14|PRSS14|SNC19|TADG15</td></tr>
<tr><td></td><td></td><td></td><td></td><td></td></tr>
<tr><td>0018146 // ke</td><td>0000139 // Go</td><td>0003836 // be</td><td>CMP-N-acetyl</td><td>SIA4B_HUMAN|ST3GAL2|SIAT4B</td></tr>
<tr><td>0097503 // sia</td><td>0000139 // Go</td><td>0003836 // be</td><td>CMP-N-acetyl</td><td>SIAT6_HUMAN|ST3GAL3|SIAT6</td></tr>
<tr><td>0006688 // gly</td><td>0005887 // in</td><td>0003836 // be</td><td>Lactosylceram</td><td>SIAT9_HUMAN|ST3GAL5|SIAT9|UNQ2510/PRO5998</td></tr>
<tr><td>0097503 // sia</td><td>0000139 // Go</td><td>0003836 // be</td><td>Type 2 lactosa</td><td>SIA10_HUMAN|ST3GAL6|SIAT10</td></tr>
<tr><td></td><td></td><td></td><td></td><td></td></tr>
<tr><td>0043547 // po</td><td></td><td>0017112 // Ra</td><td>Suppression o</td><td>ST5_HUMAN|ST5|DENND2B|HTS1</td></tr>
<tr><td>0097503 // sia</td><td>0016021 // in</td><td>0001665 // al</td><td>Alpha-N-acety</td><td>SIA7A_HUMAN|ST6GALNAC1|SIAT7A|UNQ543/PRO848</td></tr>
<tr><td>0009311 // ol</td><td>0016021 // in</td><td>0008373 // sia</td><td>Alpha-N-acety</td><td>SIA7B_HUMAN|ST6GALNAC2|SIAT7B|SIATL1|STHM</td></tr>
<tr><td>0009100 // gly</td><td>0016021 // in</td><td>0008373 // sia</td><td>Alpha-N-acety</td><td>SIA7C_HUMAN|ST6GALNAC3|SIAT7C|UNQ2787/PRO717</td></tr>
<tr><td>0097503 // sia</td><td>0016021 // in</td><td>0008373 // sia</td><td>Alpha-N-acety</td><td>SIA7E_HUMAN|ST6GALNAC5|SIAT7E</td></tr>
<tr><td>0009100 // gly</td><td>0016021 // in</td><td>0001665 // al</td><td>Alpha-N-acety</td><td>SIA7F_HUMAN|ST6GALNAC6|SIAT7F|UNQ708/PRO1359</td></tr>
<tr><td></td><td></td><td></td><td></td><td></td></tr>
<tr><td>0030308 // ne</td><td>0016021 // in</td><td></td><td>Suppressor of</td><td>ST7L_HUMAN|ST7L|ST7R</td></tr>
<tr><td>0006464 // ce</td><td>0016021 // in</td><td>0003828 // al</td><td>CMP-N-acetyl</td><td>SIA8D_HUMAN|ST8SIA4|PST|PST1|SIAT8D</td></tr>
<tr><td>0097503 // sia</td><td>0016021 // in</td><td>0008373 // sia</td><td>Alpha-2,8-sial</td><td>SIA8E_HUMAN|ST8SIA5|SIAT8E</td></tr>
<tr><td>0006898 // re</td><td>0005886 // pl</td><td>0005041 // lo</td><td>Stabilin-2</td><td>STAB2_HUMAN|STAB2|FEEL2|FELL|FEX2|HARE</td></tr>
<tr><td>0006936 // m</td><td>0030315 // T-</td><td>0005515 // pr</td><td>SH3 and cyste</td><td>STAC_HUMAN|STAC</td></tr>
<tr><td>0007130 // sy</td><td>0030893 // m</td><td></td><td>Cohesin subu</td><td>STAG3_HUMAN|STAG3</td></tr>
<tr><td></td><td>0005634 // nu</td><td></td><td>Putative STAG</td><td>ST3L1_HUMAN|STAG3L1</td></tr>
<tr><td></td><td>0005634 // nu</td><td></td><td>Putative STAG</td><td>ST3L2_HUMAN|STAG3L2</td></tr>
<tr><td></td><td></td><td></td><td></td><td></td></tr>
<tr><td></td><td></td><td></td><td></td><td></td></tr>
<tr><td>0016579 // pr</td><td>0016020 // m</td><td>0061578 // Ly</td><td>AMSH-like pro</td><td>STALP_HUMAN|STAMBPL1|AMSHLP|KIAA1373</td></tr>
<tr><td>0042531 // po</td><td>0005886 // pl</td><td>0005515 // pr</td><td>Signal-transdu</td><td>STAP2_HUMAN|STAP2|BKS</td></tr>
<tr><td>0032782 // bi</td><td>0005829 // cy</td><td>0008289 // lip</td><td>START domain</td><td>STA10_HUMAN|STARD10|SDCCAG28|CGI-52</td></tr>
<tr><td>0097498 // en</td><td>0005829 // cy</td><td>0005515 // pr</td><td>StAR-related l</td><td>STA13_HUMAN|STARD13|DLC2|GT650</td></tr>
<tr><td>0010879 // ch</td><td>0005739 // m</td><td>0017127 // ch</td><td>StAR-related l</td><td>STAR4_HUMAN|STARD4</td></tr>
<tr><td></td><td></td><td></td><td></td><td></td></tr>
<tr><td>0015721 // bi</td><td>0005829 // cy</td><td>0032052 // bi</td><td>StAR-related l</td><td>STAR5_HUMAN|STARD5</td></tr>
<tr><td>0043547 // po</td><td>0005925 // fo</td><td>0005096 // GT</td><td>StAR-related l</td><td>STAR8_HUMAN|STARD8|DLC3|KIAA0189</td></tr>
<tr><td>0051225 // sp</td><td>0005634 // nu</td><td>0015485 // ch</td><td>StAR-related l</td><td>STAR9_HUMAN|STARD9|KIAA1300</td></tr>
<tr><td>0002230 // po</td><td>0005634 // nu</td><td>0005515 // pr</td><td>Signal transdu</td><td>STAT1_HUMAN|STAT1</td></tr>
<tr><td>0051607 // de</td><td>0005829 // cy</td><td>0005515 // pr</td><td>Signal transdu</td><td>STAT2_HUMAN|STAT2</td></tr>
</table>

0045944 // pc	0005654 // nu	0003700 // tr	Signal transdu	STAT4_HUMAN|STAT4
0019530 // ta	0005829 // cy	0005515 // pr	Signal transdu	STA5A_HUMAN|STAT5A|STAT5
0035771 // in	0005829 // cy	0005515 // pr	Signal transdu	STAT6_HUMAN|STAT6
0034599 // ce	0005737 // cy	0005515 // pr	Double-strand	STAU1_HUMAN|STAU1|STAU
0005980 // gl	0005886 // pl	0005515 // pr	Starch-binding	STBD1_HUMAN|STBD1|GENX-3414
0030336 // ne	0005634 // nu	0005179 // hc	Stanniocalcin-	STC1_HUMAN|STC1|STC
0006874 // ce	0005783 // er	0020037 // he	Stanniocalcin-	STC2_HUMAN|STC2
0055114 // ox	0005911 // ce	0005215 // tr	Metalloreduct	STEA1_HUMAN|STEAP1|PRSS24|STEAP
0006893 // Gc	0005769 // ea	0052851 // fe	Metalloreduct	STEA2_HUMAN|STEAP2|PCANAP1|STAMP1|UNQ6507/I
0009306 // pr	0010008 // er	0052851 // fe	Metalloreduct	STEA3_HUMAN|STEAP3|TSAP6
0055114 // ox	0005794 // Gc	0052851 // fe	Metalloreduct	STEA4_HUMAN|STEAP4|STAMP2|TNFAIP9
0051924 // re	0030176 // in	0002020 // pr	Stromal intera	STIM1_HUMAN|STIM1|GOK
0032237 // ac	0016021 // in	0005509 // ca	Stromal intera	STIM2_HUMAN|STIM2|KIAA1482
0006950 // re	0005829 // cy	0003723 // RN	Stress-induced	STIP1_HUMAN|STIP1
0023014 // sig	0070062 // ex	0042802 // id	Serine/threon	STK10_HUMAN|STK10|LOK
0035556 // in	0005634 // nu	0005524 // AT	Serine/threon	ST17A_HUMAN|STK17A|DRAK1
0006468 // pr	0005737 // cy	0005515 // pr	Serine/threon	STK3_HUMAN|STK3|KRS1|MST2
0006468 // pr	0005634 // nu	0004674 // pr	Serine/threon	STK31_HUMAN|STK31|SGK396
0018105 // pe	0005622 // in	0004674 // pr	Serine/threon	ST32C_HUMAN|STK32C|YANK3
0018107 // pe	0005622 // in	0005524 // AT	Serine/threon	STK33_HUMAN|STK33
0051128 // re	0016020 // m	0005515 // pr	Serine/threon	ST38L_HUMAN|STK38L|KIAA0965|NDR2
0060215 // pr	0005737 // cy	0004672 // pr	Serine/threon	STK4_HUMAN|STK4|KRS2|MST1
0007019 // m	0070062 // ex	0004871 // sig	Stathmin	STMN1_HUMAN|STMN1|C1orf215|LAP18|OP18
0031110 // re	0005737 // cy	0019904 // pr	Stathmin-3	STMN3_HUMAN|STMN3|SCLIP
0051260 // pr	0005783 // er	0042803 // pr	Erythrocyte ba	STOM_HUMAN|STOM|BND7|EPB72
0006851 // m	0045121 // m	0005515 // pr	Stomatin-like	STML2_HUMAN|STOML2|SLP2|HSPC108
0002244 // he	0016020 // m	0005515 // pr	Stonin-2	STON2_HUMAN|STON2|STN2|STNB
0009790 // er	0005575 // ce	0003674 // m	Storkhead-bo:	STOX2_HUMAN|STOX2|KIAA1392
0007612 // le	0005886 // pl	0004872 // re	Stimulated by	STRA6_HUMAN|STRA6|PP14296|UNQ3126/PRO10282/I
0048133 // m	0005737 // cy	0046983 // pr	Stimulated by	STRA8_HUMAN|STRA8
0007050 // ce	0005634 // nu	0005515 // pr	STE20-related	STRAB_HUMAN|STRADB|ALS2CR2|ILPIP|PRO1038
0000122 // ne	0005829 // cy	0003723 // RN	Serine-threon	STRAP_HUMAN|STRAP|MAWD|UNRIP
0050910 // de	0060091 // ki		Stereocilin	STRC_HUMAN|STRC
0007605 // se	0060091 // ki		Putative stere	STRCL_HUMAN|STRCP1|STRCP
0007010 // cy	0005737 // cy	0003674 // m	Striatin-intera	STRP2_HUMAN|STRIP2|FAM40B|KIAA1170
0032355 // re	0005886 // pl	0005515 // pr	Striatin-3	STRN3_HUMAN|STRN3|GS2NA|SG2NA
0043686 // cc	0008250 // ol	0004579 // dc	Dolichyl-diphc	STT3B_HUMAN|STT3B|SIMP
0031398 // pc	0005783 // er	0005515 // pr	E3 ubiquitin-p	CHIP_HUMAN|STUB1|CHIP|PP1131
0006906 // ve	0032588 // tr	0005515 // pr	Syntaxin-10	STX10_HUMAN|STX10|SYN10
0061025 // m	0005794 // Gc	0005515 // pr	Syntaxin-11	STX11_HUMAN|STX11
0042147 // re	0000139 // Gc	0005515 // pr	Syntaxin-16	STX16_HUMAN|STX16
0006906 // ve	0012505 // er	0000149 // SN	STX16-NPEPL1	H3BU86_HUMAN|STX16-NPEPL1

0006886 // in	0031201 // SN	0005515 // pr	Syntaxin-19	STX19_HUMAN|STX19
0072657 // pr	0005886 // pl	0048306 // ca	Syntaxin-1A	STX1A_HUMAN|STX1A|STX1
0016081 // sy	0005886 // pl	0000149 // SN	Syntaxin-1B	STX1B_HUMAN|STX1B|STX1B1|STX1B2
0009887 // ar	0016323 // ba	0046983 // pr	Syntaxin-2	STX2_HUMAN|STX2|EPIM|STX2A|STX2B|STX2C
0042147 // re	0000139 // Go	0005515 // pr	Syntaxin-6	STX6_HUMAN|STX6
0031630 // re	0005829 // cy	0000149 // SN	Syntaxin-bind	STXB1_HUMAN|STXBP1|UNC18A
0050708 // re	0005886 // pl	0019905 // sy	Syntaxin-bind	STB5L_HUMAN|STXBP5L|KIAA1006|LLGL4
0006893 // Go	0005913 // ce	0098641 // ca	Syntaxin-bind	STXB6_HUMAN|STXBP6|HSPC156
0038083 // pe	0016021 // in	0004715 // no	Tyrosine-prot	STYK1_HUMAN|STYK1|NOK
0070372 // re	0005634 // nu	0008138 // pr	Serine/threon	STYX_HUMAN|STYX
0007186 // G-	0005886 // pl	0004930 // G-	Succinate rece	SUCR1_HUMAN|SUCNR1|GPR91
0008380 // RN	0016604 // nu	0003723 // RN	SURP and G-p	SUGP2_HUMAN|SUGP2|KIAA0365|SFRS14
0030201 // he	0009986 // ce	0005509 // ca	Extracellular s	SULF1_HUMAN|SULF1|KIAA1077
0030201 // he	0009986 // ce	0005509 // ca	Extracellular s	SULF2_HUMAN|SULF2|KIAA1247|UNQ559/PRO1120
0051923 // su	0005829 // cy	0004062 // ar	Sulfotransfera	ST1A1_HUMAN|SULT1A1|STP|STP1|OK/SW-cl.88
0051923 // su	0005829 // cy	0004062 // ar	Sulfotransfera	ST1A2_HUMAN|SULT1A2|STP2
0050427 // 3'	0005829 // cy	0004062 // ar	Sulfotransfera	ST1A4_HUMAN|SULT1A4
0051923 // su	0005829 // cy	0005515 // pr	Sulfotransfera	ST1B1_HUMAN|SULT1B1|ST1B2|SULT1B2
0050427 // 3'	0005829 // cy	0050294 // st	Sulfotransfera	ST2B1_HUMAN|SULT2B1|HSST2
0043687 // po	0005788 // en	0016491 // ox	Sulfatase-mod	SUMF1_HUMAN|SUMF1|FGE|PSEC0152|UNQ3037/PRO
0043687 // po	0005788 // en	0046872 // m	Sulfatase-mod	SUMF2_HUMAN|SUMF2|PSEC0171|UNQ1968/PRO4500
0007283 // sp	0002080 // ac	0005515 // pr	SUN domain-c	SUN1_HUMAN|SUN1|KIAA0810|UNC84A
0090286 // cy	0016021 // in	0043495 // pr	SUN domain-c	SUN3_HUMAN|SUN3|SUNC1
0000122 // ne	0005654 // nu	0005515 // pr	Transcription	SPT4H_HUMAN|SUPT4H1|SPT4H|SUPT4H
0000958 // m	0005739 // m	0003723 // RN	ATP-depende	SUV3_HUMAN|SUPV3L1|SUV3
0008150 // bi	0005730 // nu	0003674 // m	Surfeit locus p	SURF2_HUMAN|SURF2
0042273 // rib	0022625 // cy	0003723 // RN	Surfeit locus p	SURF6_HUMAN|SURF6|SURF-6
	0016021 // in	0005509 // ca	Sushi domain-	SUSD1_HUMAN|SUSD1|UNQ2438/PRO4999
0007155 // ce	0016021 // in	0005540 // hy	Sushi domain-	SUSD5_HUMAN|SUSD5|KIAA0527
0000122 // ne	0005652 // nu	0005515 // pr	Histone-lysine	SUV91_HUMAN|SUV39H1|KMT1A|SUV39H
0006836 // ne	0043025 // ne	0019901 // pr	Synaptic vesic	SV2A_HUMAN|SV2A|KIAA0736|PSEC0174
0007155 // ce	0016020 // m	0005509 // ca	Sushi, von Wil	SVEP1_HUMAN|SVEP1|C9orf13|CCP22|SELOB
0007519 // sk	0005634 // nu	0051015 // ac	Supervillin	SVIL_HUMAN|SVIL
0010508 // po	0070062 // ex	0043621 // pr	Small VCP/p97	SVIP_HUMAN|SVIP
0055085 // tra	0030672 // sy	0022891 // su	Synaptic vesic	SVOP_HUMAN|SVOP
0032880 // re	0005737 // cy	0005515 // pr	Switch-associa	SWP70_HUMAN|SWAP70|KIAA0640|HSPC321
0061178 // re	0016021 // in	0017075 // sy	Syntabulin	SYBU_HUMAN|SYBU|GOLSYN|KIAA1472
0007130 // sy	0000795 // sy		Synaptonema	SYC1L_HUMAN|SYCE1L
0051301 // ce	0000801 // ce		Synaptonema	SYCE2_HUMAN|SYCE2|CESC1
0043065 // po	0005694 // ch	0005515 // pr	Synaptonema	SYCE3_HUMAN|SYCE3|C22orf41|THEG2
0007129 // sy	0001673 // m	0003677 // DN	Synaptonema	SYCP1_HUMAN|SYCP1|SCP1

0060548 // ne	0000780 // co		Synaptonema	SYC2L_HUMAN|SYCP2L|C6orf177|NO145
1901165 // po	0005737 // cy	0005096 // GT	Rho GTPase-a	SYDE1_HUMAN|SYDE1
0045087 // in	0005829 // cy	0004713 // pr	Tyrosine-prot	KSYK_HUMAN|SYK
0046928 // re	0030425 // de	0019901 // pr	Synapsin-1	SYN1_HUMAN|SYN1
0007269 // ne	0030672 // sy	0003824 // ca	Synapsin-2	SYN2_HUMAN|SYN2
0045103 // in	0005829 // cy	0005198 // st	Syncoilin	SYNCI_HUMAN|SYNC|SYNC1
0009607 // re	0045211 // po	0005515 // pr	Synapse differ	SYNG1_HUMAN|SYNDIG1|C20orf39|TMEM90B
0006997 // nu	0005635 // nu	0003723 // RN	Nesprin-1	SYNE1_HUMAN|SYNE1|C6orf98|KIAA0796|KIAA1262|KI
0007010 // cy	0016021 // in	0051015 // ac	Nesprin-3	SYNE3_HUMAN|SYNE3|C14orf139|C14orf49|LINC00341
0045198 // es	0031309 // in	0005515 // pr	Nesprin-4	SYNE4_HUMAN|SYNE4|C19orf46
0046580 // ne	0005829 // cy	0005096 // GT	Ras/Rap GTPa	SYGP1_HUMAN|SYNGAP1|KIAA1938
0045055 // re	0016021 // in	0005515 // pr	Synaptogyrin-	SNG1_HUMAN|SYNGR1
0048499 // sy	0030672 // sy	0005515 // pr	Synaptogyrin-	SNG2_HUMAN|SYNGR2|UNQ352/PRO615
0045055 // re	0008021 // sy	0047485 // pr	Synaptogyrin-	SNG3_HUMAN|SYNGR3
0006661 // ph	0005829 // cy	0005515 // pr	Synaptojanin-	SYNJ2_HUMAN|SYNJ2|KIAA0348
	0031966 // m		SYNJ2BP-COX	A0A087WUM0_HUMAN|SYNJ2BP-COX16
0031443 // fa	0043034 // co	0005515 // pr	Synemin	SYNEM_HUMAN|SYNM|DMN|KIAA0353|SYN
0008542 // vis	0014069 // po	0005515 // pr	Synaptopodin	SYNPO_HUMAN|SYNPO|KIAA1029
0032233 // po	0005634 // nu	0005515 // pr	Synaptopodin	SYNP2_HUMAN|SYNPO2
0032233 // po	0005634 // nu	0003779 // ac	Synaptopodin	SYP2L_HUMAN|SYNPO2L
0048172 // re	0008021 // sy	0005215 // tra	Synaptophysin	SYPH_HUMAN|SYP
0007268 // ch	0042470 // m	0005215 // tra	Synaptophysin	SYPL1_HUMAN|SYPL1|SYPL
0006874 // ce	0030285 // in	0005215 // tra	Synaptophysin	SYPL2_HUMAN|SYPL2
0000042 // pr	0005829 // cy		Protein SYS1 h	SYS1_HUMAN|SYS1|C20orf169
0000042 // pr	0005829 // cy		SYS1-DBNDD2	H3BUS1_HUMAN|SYS1-DBNDD2
0007269 // ne	0005886 // pl	0017075 // sy	Synaptotagmi	SYT1_HUMAN|SYT1|SVP65|SYT
1900243 // ne	0043197 // de	0048487 // be	Synaptotagmi	SYT11_HUMAN|SYT11|KIAA0080
0048791 // ca	0016021 // in	0030276 // cla	Synaptotagmi	SYT12_HUMAN|SYT12
0017158 // re	0016021 // in	0030276 // cla	Synaptotagmi	SYT14_HUMAN|SYT14
0048791 // ca	0016021 // in	0030276 // cla	Synaptotagmi	SYT15_HUMAN|SYT15
0048791 // ca	0098793 // pr	0005515 // pr	Synaptotagmi	SYT17_HUMAN|SYT17
0048791 // ca	0005886 // pl	0030276 // cla	Synaptotagmi	SYT2_HUMAN|SYT2
0048791 // ca	1990769 // pr	0005546 // ph	Synaptotagmi	SYT5_HUMAN|SYT5
0070092 // re	0043025 // ne	0030276 // cla	Synaptotagmi	SYT7_HUMAN|SYT7|PCANAP7
0048791 // ca	0016021 // in	0030276 // cla	Synaptotagmi	SYT8_HUMAN|SYT8
0006886 // in	0070062 // ex	0030276 // cla	Synaptotagmi	SYTL1_HUMAN|SYTL1|SLP1|SB146
0006886 // in	0098793 // pr	0030276 // cla	Synaptotagmi	SYTL3_HUMAN|SYTL3|SLP3
0006886 // in	0019898 // ex	0030276 // cla	Synaptotagmi	SYTL4_HUMAN|SYTL4
0006886 // in	0098793 // pr	0030276 // cla	Synaptotagmi	SYTL5_HUMAN|SYTL5|SLP5
0030433 // ER	0005789 // en	0005515 // pr	E3 ubiquitin-p	SYVN1_HUMAN|SYVN1|HRD1|KIAA1810
0002223 // sti	0005829 // cy	0005515 // pr	TGF-beta-activ	TAB3_HUMAN|TAB3|MAP3K7IP3
0007204 // po	0005615 // ex	0005102 // re	Tachykinin-4	TKN4_HUMAN|TAC4
0021987 // ce	0005737 // cy	0035257 // nu	Transforming	TACC2_HUMAN|TACC2
0006417 // re	0005739 // m	0005515 // pr	Translational a	TACO1_HUMAN|TACO1|CCDC44|PRO0477
0006954 // in	0005886 // pl	0005515 // pr	Substance-P r	NK1R_HUMAN|TACR1|NK1R|TAC1R
0042053 // re	0005886 // pl	0004995 // ta	Neuromedin-I	NK3R_HUMAN|TACR3|NK3R|TAC3R

0050678 // re	0016328 // lat	0005515 // pr	Tumor-associa	TACD2_HUMAN|TACSTD2|GA733-1|M1S1|TROP2
0051101 // re	0005654 // nu	0003677 // DI	Transcription	TAF10_HUMAN|TAF10|TAF2A|TAF2H|TAFII30
0006366 // tra	0005654 // nu	0005515 // pr	Transcription	TAF13_HUMAN|TAF13|TAF2K|TAFII18
0006361 // tra	0005654 // nu	0005515 // pr	TATA box-bind	TAF1A_HUMAN|TAF1A
0006361 // tra	0005654 // nu	0001187 // tra	TATA box-bind	TAF1B_HUMAN|TAF1B
0006361 // tra	0005654 // nu	0005515 // pr	TATA box-bind	TAF1C_HUMAN|TAF1C
0016032 // vi	0005654 // nu	0005515 // pr	Transcription	TAF4_HUMAN|TAF4|TAF2C|TAF2C1|TAF4A|TAFII130|T/
0007283 // sp	0005654 // nu	0051059 // NI	Transcription	TAF4B_HUMAN|TAF4B|TAF2C2|TAFII105
0000122 // ne	0005654 // nu	0003713 // tra	Transcription	TAF7_HUMAN|TAF7|TAF2F|TAFII55
0007283 // sp	0005669 // tra	0044212 // tra	Transcription	TAF7L_HUMAN|TAF7L|TAF2Q
0006974 // ce	0005654 // nu	0070742 // C2	Transcription	TAF9_HUMAN|TAF9|TAF2G|TAFII31
0043066 // ne	0005654 // nu	0005515 // pr	Transcription	TAF9B_HUMAN|TAF9B|TAF9L
0043547 // po	0005829 // cy	0005096 // G	T-cell activatio	TAGAP_HUMAN|TAGAP|TAGAP1|FKSG15
0030855 // ep	0005737 // cy	0051015 // ac	Transgelin	TAGL_HUMAN|TAGLN|SM22|WS3-10
0007417 // ce	0005634 // nu	0051015 // ac	Transgelin-3	TAGL3_HUMAN|TAGLN3|NP25
0005975 // ca	0005737 // cy	0005515 // pr	Transaldolase	TALDO_HUMAN|TALDO1|TAL|TALDO|TALDOR
0008542 // vis	0014069 // po		Protein TANC1	TANC1_HUMAN|TANC1|KIAA1728
0001701 // in			Protein TANC2	TANC2_HUMAN|TANC2|KIAA1148|KIAA1636
0000165 // M	0005886 // pl	0016740 // tra	Serine/threon	TAOK3_HUMAN|TAOK3|DPK|JIK|KDS|MAP3K18
0015833 // pe	0005739 // m	0015197 // pe	Antigen pepti	TAP1_HUMAN|TAP1|ABCB2|PSF1|RING4|Y3
0055085 // tra	0005789 // er	0005515 // pr	Antigen pepti	TAP2_HUMAN|TAP2|ABCB3|PSF2|RING11|Y1
0050823 // pe	0071556 // int	0005515 // pr	Tapasin	TPSN_HUMAN|TAPBP|NGS17|TAPA
0002376 // im	0016021 // int	0005515 // pr	Tapasin-relate	TPSNR_HUMAN|TAPBPL
0007186 // G-	0016021 // int	0016520 // gr	Transmembra	TAPT1_HUMAN|TAPT1|CMVFR
0006357 // re	0005634 // nu	0016423 // tR	Probable met	TARB1_HUMAN|TARBP1|TRM3|TRP185
2000515 // ne	0005886 // pl	0034987 // im	T-cell-interact	TARM1_HUMAN|TARM1
0070159 // m	0005739 // m	0042803 // pr	Threonine--tR	SYTM_HUMAN|TARS2|TARSL1
0006435 // th	0005737 // cy	0005515 // pr	Probable thre	SYTC2_HUMAN|TARSL2
0050916 // se	0016021 // int	0004930 // G-	Taste recepto	TS1R3_HUMAN|TAS1R3|T1R3|TR3
0007186 // G-	0005886 // pl	0004930 // G-	Taste recepto	T2R20_HUMAN|TAS2R20|TAS2R49
0006508 // pr		0004298 // th	Threonine asp	TASP1_HUMAN|TASP1|C20orf13
0031338 // re	0005622 // int	0005096 // G	TBC1 domain	TBCD1_HUMAN|TBC1D1|KIAA1108
2000785 // re	0055037 // re	0005096 // G	TBC1 domain	TBC12_HUMAN|TBC1D12|KIAA0608
0031338 // re	0005829 // cy	0005515 // pr	TBC1 domain	TBC16_HUMAN|TBC1D16
0031338 // re	0030054 // ce	0005096 // G	TBC1 domain	TBD2A_HUMAN|TBC1D2|PARIS1|PP8997|TBC1D2A
0031338 // re	0005622 // int	0005515 // pr	TBC1 domain	TB22B_HUMAN|TBC1D22B|C6orf197
0031338 // re	0005622 // int	0005096 // G	TBC1 domain	TBD2B_HUMAN|TBC1D2B|KIAA1055
0031338 // re	0005622 // int	0005096 // G	TBC1 domain	TBC3A_HUMAN|TBC1D3|PRC17|TBC1D3A
0031338 // re	0005829 // cy	0005096 // G	TBC1 domain	TBC30_HUMAN|TBC1D30|KIAA0984
	0005813 // ce		TBC1 domain	TBC31_HUMAN|TBC1D31|WDR67
0042733 // en	0005737 // cy		Protein broad	BROMI_HUMAN|TBC1D32|BROMI|C6orf170|C6orf171
0031338 // re	0005622 // int	0005096 // G	TBC1 domain	TBC3B_HUMAN|TBC1D3B|TBC1D3I
0031338 // re	0005622 // int	0005096 // G	TBC1 domain	TBC3C_HUMAN|TBC1D3C|TBC1D3D

0031338 // re	0005622 // in	0005096 // G	TBC1 domain	TBC3D_HUMAN|TBC1D3D
0031338 // re	0005622 // in	0005096 // G	TBC1 domain	TBC3F_HUMAN|TBC1D3F|TBC1D3E
0031338 // re	0005622 // in	0005096 // G	TBC1 domain	TBC3H_HUMAN|TBC1D3H
0031338 // re	0005622 // in	0005096 // G	TBC1 domain	TBC3L_HUMAN|TBC1D3L
0031339 // ne	0005622 // in	0005096 // G	TBC1 domain	TBCD4_HUMAN|TBC1D4|AS160|KIAA0603
0042594 // re	0005794 // Go	0005515 // pr	TBC1 domain	TBCD5_HUMAN|TBC1D5|KIAA0210
0031338 // re	0005622 // in	0005096 // G	TBC1 domain	TBC8B_HUMAN|TBC1D8B
0007399 // ne	0005829 // cy	0005515 // pr	Tubulin-foldin	TBCB_HUMAN|TBCB|CG22|CKAP1
0006457 // pr	0005737 // cy	0005515 // pr	Tubulin-specif	TBCE_HUMAN|TBCE
0030036 // ac	0072686 // m	0005096 // G	TBC domain-c	TBCK_HUMAN|TBCK|TBCKL|HSPC302
0016032 // vi		0005515 // pr	TANK-binding	TBKB1_HUMAN|TBKBP1|KIAA0775|SINTBAD
0016575 // hi	0005876 // sp	0005515 // pr	F-box-like/WD	TBL1X_HUMAN|TBL1X|TBL1
0016575 // hi	0005876 // sp	0003714 // tr	F-box-like/WD	TBL1R_HUMAN|TBL1XR1|IRA1|TBLR1
0006364 // rR	0005654 // nu	0003723 // RN	Transducin be	TBL3_HUMAN|TBL3|SAZD
0003007 // he	0005634 // nu	0003700 // tr	T-box transcri	TBX1_HUMAN|TBX1
0048701 // er	0070722 // Tl	0046982 // pr	T-box transcri	TBX15_HUMAN|TBX15|TBX14
0045165 // ce	0005634 // nu	0000978 // RN	T-box transcri	TBX19_HUMAN|TBX19|TPIT
0045892 // ne	0005634 // nu	0003677 // DI	T-box transcri	TBX2_HUMAN|TBX2
0003193 // pu	0005634 // nu	0000978 // RN	T-box transcri	TBX20_HUMAN|TBX20
0008284 // po	0005634 // nu	0005515 // pr	T-box transcri	TBX3_HUMAN|TBX3
0032525 // so	0005634 // nu	0001102 // RN	T-box transcri	TBX6_HUMAN|TBX6
0045907 // po	0005886 // pl	0004961 // th	Thromboxane	TA2R_HUMAN|TBXA2R
0019371 // cy	0005789 // er	0004497 // m	Thromboxane	THAS_HUMAN|TBXAS1|CYP5|CYP5A1
0017158 // re	0005634 // nu	0005544 // ca	Tandem C2 do	TAC2N_HUMAN|TC2N|C14orf47|MTAC2D1
0090314 // po	0005886 // pl	0005515 // pr	TRPM8 chann	TCAF1_HUMAN|TCAF1|FAM115A|KIAA0738
0030335 // po	0005886 // pl	0044325 // io	TRPM8 chann	TCAF2_HUMAN|TCAF2|FAM115C|FAM139A
0032784 // re	0008023 // tr	0005515 // pr	Transcription	TCEA2_HUMAN|TCEA2
0006351 // tr	0005634 // nu	0003677 // DI	Transcription	TCEA3_HUMAN|TCEA3|TFIISH
0006357 // re	0005634 // nu	0050699 // W	Transcription	TCAL3_HUMAN|TCEAL3|MSTP072
0006357 // re	0005634 // nu	0050699 // W	Transcription	TCAL4_HUMAN|TCEAL4|NPD017
0006357 // re	0005634 // nu	0050699 // W	Transcription	TCAL8_HUMAN|TCEAL8
0006357 // re	0005634 // nu	0050699 // W	Transcription	TCAL9_HUMAN|TCEAL9|WBP5
0006351 // tr	0005634 // nu	0046872 // m	Transcription	TCF19_HUMAN|TCF19|SC1
0006351 // tr	0005634 // nu	0003677 // DI	Transcription	TCF24_HUMAN|TCF24
0030183 // B	0005654 // nu	0035326 // er	Transcription	TFE2_HUMAN|TCF3|BHLHB21|E2A|ITF1
0006352 // DI	0005634 // nu	0005515 // pr	Transcription	ITF2_HUMAN|TCF4|BHLHB19|ITF2|SEF2
0033153 // T	0016604 // nu	0044212 // tr	Transcription	TCF7_HUMAN|TCF7|TCF1
0006325 // ch	0005654 // nu	0003677 // DI	Transcription	TF7L1_HUMAN|TCF7L1|TCF3
0042127 // re	0001673 // m	0003677 // DI	Transcription	TCFL5_HUMAN|TCFL5|CHA|E2BP1
0070268 // co	0005829 // cy	0005509 // ca	Trichohyalin	TRHY_HUMAN|TCHH|THH|THL|TRHY
0015992 // pr	0005886 // pl	0046961 // pr	V-type proton	VPP3_HUMAN|TCIRG1|ATP6N1C|ATP6V0A3
0015889 // co	0005576 // ex	0031419 // co	Transcobalam	TCO2_HUMAN|TCN2|TC2
0007339 // bi	0005829 // cy	0044183 // pr	T-complex pro	TCPA_HUMAN|TCP1|CCT1|CCTA

0072675 // os	0005622 // in	0003674 // m	T-cell leukemi	TCTA_HUMAN|TCTA	
1902017 // re	0005868 // cy	0005515 // pr	Tctex1 domai	TC1D2_HUMAN|TCTEX1D2	
	0005815 // m	0005515 // pr	Tctex1 domai	TC1D4_HUMAN|TCTEX1D4	
0021537 // te	0005829 // cy		Tectonic-1	TECT1_HUMAN|TCTN1|TECT1|UNQ9369/PRO34160	
0060271 // cil	0016021 // in		Tectonic-3	TECT3_HUMAN|TCTN3|C10orf61|TECT3|PSEC0041|UN(	
0051289 // pr	0005829 // cy	0005515 // pr	Tryptophan 2,	T23O_HUMAN|TDO2|TDO	
0006302 // dc	0043231 // in	0017005 // 3'	Tyrosyl-DNA p	TYDP1_HUMAN|TDP1	
0006302 // dc	0016235 // ag	0003714 // tr	Tyrosyl-DNA p	TYDP2_HUMAN|TDP2|EAP2|TTRAP|AD-022	
0007275 // m	0033391 // ch		Tudor domain	TDRD6_HUMAN|TDRD6	
0070306 // le	0035770 // rib	0005515 // pr	Tudor domain	TDRD7_HUMAN|TDRD7|PCTAIRE2BP	
0007283 // sp	0005737 // cy	0003674 // m	Testis develop	TDRP_HUMAN|TDRP|C8orf42	
1902895 // pc	0005654 // nu	0005515 // pr	Transcriptiona	TEAD1_HUMAN|TEAD1|TCF13|TEF1	
0006357 // re	0005654 // nu	0005515 // pr	Transcriptiona	TEAD3_HUMAN|TEAD3|TEAD5|TEF5	
0035329 // hi	0005654 // nu	0005515 // pr	Transcriptiona	TEAD4_HUMAN|TEAD4|RTEF1|TCF13L1|TEF3	
0042246 // tis	0005856 // cy	0004715 // nc	Tyrosine-prot	TEC_HUMAN|TEC|PSCTK4	
0042761 // ve	0005634 // nu	0005515 // pr	Very-long-cha	TECR_HUMAN|TECR|GPSN2|SC2	
0042761 // ve	0016021 // in	0016491 // ox	Trans-2,3-eno	TECRL_HUMAN|TECRL|SRD5A2L2	
0007160 // ce	0031225 // an	0005201 // ex	Alpha-tectorir	TECTA_HUMAN|TECTA	
0006357 // re	0005634 // nu	0005515 // pr	Thyrotroph er	TEF_HUMAN|TEF|KIAA1655	
0014068 // pc	0005886 // pl	0004713 // pr	Angiopoietin-	TIE2_HUMAN|TEK|TIE2|VMCM|VMCM1	
0060271 // cil	0005737 // cy		Tektin-2	TEKT2_HUMAN|TEKT2	
0071902 // pc	0016020 // m	0005515 // pr	Telomere leng	TELO2_HUMAN|TELO2|KIAA0683	
0032211 // ne	0005634 // nu	0003697 // sir	CST complex s	TEN1L_HUMAN|TEN1|C17orf106	
0008285 // ne	0005576 // ex	0046982 // pr	Teneurin-1	TEN1_HUMAN|TENM1|ODZ1|TNM1	
0007165 // sig	0043005 // ne	0046982 // pr	Teneurin-3	TEN3_HUMAN|TENM3|KIAA1455|ODZ3|TNM3	
0008285 // ne	0005925 // fo	0003723 // RI	Testin	TES_HUMAN|TES	
0032417 // pc	0005634 // nu	0005509 // ca	Calcineurin B	CHP3_HUMAN|TESC|CHP3	
0035556 // in	0005737 // cy	0005515 // pr	Dual specificit	TESK1_HUMAN|TESK1	
0030036 // ac	0016604 // nu	0004672 // pr	Dual specificit	TESK2_HUMAN|TESK2	
0007275 // m	0005634 // nu	0046872 // m	Tesmin	MTL5_HUMAN|TESMIN|MTL5	
0001826 // in	0005634 // nu	0070579 // m	Methylcytosir	TET1_HUMAN|TET1|CXXC6|KIAA1676|LCX	
0000712 // re	0000801 // ce	0005515 // pr	Testis-express	TEX11_HUMAN|TEX11	
0007283 // sp	0005737 // cy	0005515 // pr	Testis-express	TEX19_HUMAN|TEX19	
	0001669 // ac		Testis-express	TEX22_HUMAN|TEX22	
0006888 // EF	0030176 // in	0097020 // CC	Protein TEX26	TX261_HUMAN|TEX261|UNQ1882/PRO4325	
	0016021 // in		Testis-express	TEX29_HUMAN|TEX29|C13orf16	
		0016787 // hy	Testis-express	TEX30_HUMAN|TEX30|C13orf27	
	0015630 // m		Testis-express	TEX35_HUMAN|TEX35|C1orf49	
0006879 // ce	0005576 // ex	1990459 // tr	Serotransferri	TRFE_HUMAN|TF|PRO1400	
0006366 // tr	0042645 // m	0003723 // RI	Transcription	TFAM_HUMAN|TFAM|TCF6|TCF6L2	
0045892 // ne	0005634 // nu	0005515 // pr	Transcription	AP2A_HUMAN|TFAP2A|AP2TF|TFAP2	
0006357 // re	0005654 // nu	0003700 // tr	Transcription	AP2C_HUMAN|TFAP2C	
0045944 // pc	0005634 // nu	0000981 // RI	Transcription	AP2E_HUMAN|TFAP2E	

0043922 // ne	0005634 // nu	0005515 // pr	Transcription	TFAP4_HUMAN|TFAP4|BHLHC41
0006357 // re	0005654 // nu	0003700 // tra	Transcription	TFDP2_HUMAN|TFDP2|DP2
0007040 // lys	0005634 // nu	0003700 // tra	Transcription	TFEB_HUMAN|TFEB|BHLHE35
0042060 // wo	0005576 // ex	0003674 // m	Trefoil factor 3	TFF3_HUMAN|TFF3|ITF|TFI
2001033 // ne	0005654 // nu	0005515 // pr	Tuftelin-intera	TFP11_HUMAN|TFIP11|STIP|HSPC006
0007598 // bl	0005576 // ex	0004867 // se	Tissue factor p	TFPI1_HUMAN|TFPI|LACI|TFPI1
0007596 // bl	0031012 // ex	0004867 // se	Tissue factor p	TFPI2_HUMAN|TFPI2
0006310 // DI	0005634 // nu	0005515 // pr	TCF3 fusion pa	TFPT_HUMAN|TFPT|INO80F
0006826 // iro	1990712 // HI	0005515 // pr	Transferrin re	TFR2_HUMAN|TFR2
1990830 // ce	0005886 // pl	0003723 // RN	Transferrin re	TFR1_HUMAN|TFRC
0045840 // po	0005789 // en	0005515 // pr	Protransformi	TGFA_HUMAN|TGFA
0016055 // W	0005737 // cy	0005515 // pr	Transforming	TGFI1_HUMAN|TGFB1I1|ARA55
0001843 // ne	0005576 // ex	0005160 // tra	Transforming	TGFB2_HUMAN|TGFB2
0030198 // ex	0005886 // pl	0005518 // co	Transforming	BGH3_HUMAN|TGFBI|BIGH3
0001701 // in	0005886 // pl	0005515 // pr	TGF-beta rece	TGFR2_HUMAN|TGFBR2
0030511 // po	0034673 // in	0005515 // pr	Transforming	TGBR3_HUMAN|TGFBR3
	0016021 // int		Transforming	TGR3L_HUMAN|TGFBR3L
0034058 // en	0043231 // int	0046332 // SM	Transforming	TGFA1_HUMAN|TGFBRAP1
0000122 // ne	0005654 // nu	0003677 // DI	Homeobox pr	TGIF2_HUMAN|TGIF2
0006351 // tra	0005634 // nu	0003677 // DI	Homeobox pr	TF2LX_HUMAN|TGIF2LX|TGIFLX
0010838 // po	0005829 // cy	0003810 // pr	Protein-glutar	TGM1_HUMAN|TGM1|KTG
0018149 // pe	0070062 // ex	0003810 // pr	Protein-glutar	TGM4_HUMAN|TGM4
0008544 // ep	0005886 // pl	0003810 // pr	Protein-glutar	TGM5_HUMAN|TGM5|TGMX
		0003677 // DI	THAP domain-	THAP3_HUMAN|THAP3
	0015630 // m	0003677 // DI	THAP domain-	THAP6_HUMAN|THAP6
0045892 // ne	0031965 // nu	0005515 // pr	THAP domain-	THAP7_HUMAN|THAP7
0007565 // fe	0016327 // ap	0005509 // ca	Thrombomod	TRBM_HUMAN|THBD|THRM
0000187 // ac	0005788 // en	0005515 // pr	Thrombospon	TSP1_HUMAN|THBS1|TSP|TSP1
0043931 // os	0048471 // pe	0005509 // ca	Thrombospon	TSP3_HUMAN|THBS3|TSP3
0006631 // fa	0005759 // m	0047617 // ac	Acyl-coenzym	THEM4_HUMAN|THEM4|CTMP
0035965 // ca	0005759 // m	0005515 // pr	Acyl-coenzym	ACO15_HUMAN|THEM5|ACOT15
	0005576 // ex		Protein THEM	THEM6_HUMAN|THEM6|C8orf55|PSEC0098
0002250 // ac	0008180 // CO	0005515 // pr	Protein THEM	THMS1_HUMAN|THEMIS|C6orf190|C6orf207
0006954 // in	0005634 // nu	0005515 // pr	Protein THEM	THMS2_HUMAN|THEMIS2|C1orf38|ICB1
0051289 // pr	0005829 // cy	0000049 // tR	Probable tRN/	THG1_HUMAN|THG1L|ICF45
0008150 // bi	0005575 // ce	0003674 // m	Threonine syr	THNS1_HUMAN|THNSL1
0009088 // th	0005615 // ex	0030170 // py	Threonine syr	THNS2_HUMAN|THNSL2
0031124 // m	0000445 // TH	0005515 // pr	THO complex	THOC1_HUMAN|THOC1|HPR1
0031124 // m	0000445 // TH	0005515 // pr	THO complex	THOC5_HUMAN|THOC5|C22orf19|KIAA0983
0006508 // pr	0005829 // cy	0042277 // pe	Thimet oligop	THOP1_HUMAN|THOP1
0043401 // st	0016604 // nu	0003677 // DI	Thyroid horm	THB_HUMAN|THRB|ERBA2|NR1A2|THR1
	0016021 // int		Thrombospon	THSD1_HUMAN|THSD1|TMTSP|UNQ3010/PRO9769

0001525 // ar	0016021 // in		Thrombospon	THS7A_HUMAN|THSD7A|KIAA0960
	0016021 // in		Thrombospon	THS7B_HUMAN|THSD7B|KIAA1679
0042357 // th	0005829 // cy	0050333 // th	Thiamine-trip	THTPA_HUMAN|THTPA
0000154 // rR	0005654 // nu	0003723 // RN	THUMP doma	THUM1_HUMAN|THUMPD1
0007249 // I-k	0005634 // nu	0003674 // m	TGFB1-induce	TIAF1_HUMAN|TIAF1
0008543 // fib	0010494 // cy	0003723 // RN	Nucleolysin TI	TIAR_HUMAN|TIAL1
0043507 // po	0005874 // m	0030971 // re	T-lymphoma i	TIAM1_HUMAN|TIAM1
0002281 // m	0005829 // cy	0005515 // pr	TIR domain-co	TCAM1_HUMAN|TICAM1|PRVTIRB|TRIF
0006954 // in	0005783 // er	0004871 // sig	TIR domain-co	TCAM2_HUMAN|TICAM2|TIRAP3|TIRP|TRAM
1903301 // po	0005829 // cy	0004083 // bi	Fructose-2,6-b	TIGAR_HUMAN|TIGAR|C12orf5
	0005634 // nu	0003677 // DN	Tigger transpo	TIGD3_HUMAN|TIGD3
0032733 // po	0009986 // ce	0050839 // ce	T-cell immuno	TIGIT_HUMAN|TIGIT|VSIG9|VSTM3
0045039 // pr	0005739 // m	0008270 // zir	Mitochondria	TIM10_HUMAN|TIMM10|TIM10
0006626 // pr	0042719 // m	0005515 // pr	Mitochondria	T10B_HUMAN|TIMM10B|FXC1|TIM9B|TIMM9B
0045039 // pr	0005743 // m	0030943 // m	Mitochondria	TIM22_HUMAN|TIMM22|TEX4|TIM22
0030150 // pr	0005743 // m	0005515 // pr	Mitochondria	TIM23_HUMAN|TIMM23|TIM23
0030150 // pr	0005744 // m	0015450 // P-	Putative mito	TI23B_HUMAN|TIMM23B
0045039 // pr	0016021 // in	0005515 // pr	Mitochondria	TIM29_HUMAN|TIMM29|c19orf52
0006626 // pr	0005743 // m	0005515 // pr	Mitochondria	TIM44_HUMAN|TIMM44|MIMT44|TIM44
0006470 // pr	0005739 // m	0005134 // in	Mitochondria	TIM50_HUMAN|TIMM50|TIM50|PRO1512
0072321 // ch	0005739 // m	0005515 // pr	Mitochondria	TIM8A_HUMAN|TIMM8A|DDP|DDP1|TIM8A
0006626 // pr	0005739 // m	0008270 // zir	Mitochondria	TIM9_HUMAN|TIMM9|TIM9|TIM9A|TIMM9A
0002576 // pl	0005576 // ex	0008083 // gr	Metalloprotei	TIMP1_HUMAN|TIMP1|CLGI|TIMP
0034097 // re	0005576 // ex	0005515 // pr	Metalloprotei	TIMP2_HUMAN|TIMP2
0002576 // pl	0005615 // ex	0005515 // pr	Metalloprotei	TIMP3_HUMAN|TIMP3
0010951 // ne	0005615 // ex	0046872 // m	Metalloprotei	TIMP4_HUMAN|TIMP4
0007155 // ce	0005615 // ex	0000166 // nu	Tubulointersti	TINAG_HUMAN|TINAG
0007155 // ce	0005615 // ex	0043236 // lar	Tubulointersti	TINAL_HUMAN|TINAGL1|GIS5|LCN7|OLRG2|TINAGL|PP
0070198 // pr	0000781 // ch	0005515 // pr	TERF1-interac	TINF2_HUMAN|TINF2|TIN2
0044236 // m	0005634 // nu	0003950 // NA	TCDD-inducib	PARPT_HUMAN|TIPARP|PARP7
0033262 // re	0000790 // nu	0003677 // DN	TIMELESS-inte	TIPIN_HUMAN|TIPIN
0007043 // ce	0005886 // pl	0005515 // pr	Tight junction	ZO1_HUMAN|TJP1|ZO1
	0005886 // pl	0005515 // pr	Tight junction	ZO3_HUMAN|TJP3|ZO3
0061624 // fru	0005829 // cy	0005515 // pr	Triokinase/FM	TKFC_HUMAN|TKFC|DAK
0005999 // xy	0005777 // pe	0004802 // tra	Transketolase	TKT_HUMAN|TKT
	0016021 // in		Calfacilitin	TLCD1_HUMAN|TLCD1
	0016021 // in		TLC domain-co	TLCD2_HUMAN|TLCD2
1903204 // ne	0005765 // lys	0005515 // pr	TLD domain-c	TLDC1_HUMAN|TLDC1|KIAA1609
0007165 // sig	0005829 // cy	0005515 // pr	Transducin-lik	TLE1_HUMAN|TLE1
0007165 // sig	0016604 // nu	0003714 // tra	Transducin-lik	TLE2_HUMAN|TLE2
0007165 // sig	0005634 // nu	0005515 // pr	Transducin-lik	TLE3_HUMAN|TLE3|KIAA1547
0016055 // W	0005634 // nu	0003682 // ch	Transducin-lik	TLE4_HUMAN|TLE4|GRG4|KIAA1261
0045666 // po	0005938 // ce	0005515 // pr	Transducin-lik	TLE6_HUMAN|TLE6
0006508 // pr	0005576 // ex	0004222 // m	Tolloid-like pr	TLL1_HUMAN|TLL1|TLL
0002576 // pl	0005829 // cy	0017166 // vir	Talin-1	TLN1_HUMAN|TLN1|KIAA1027|TLN
0007165 // sig	0005886 // pl	0046982 // pr	Toll-like recep	TLR1_HUMAN|TLR1|KIAA0012

0050729 // pc	0005886 // pl	0004888 // tra	Toll-like recep	TLR10_HUMAN|TLR10|UNQ315/PRO358
0006691 // le	0005886 // pl	0005515 // pr	Toll-like recep	TLR2_HUMAN|TLR2|TIL4
0007165 // sig	0010008 // en	0005515 // pr	Toll-like recep	TLR3_HUMAN|TLR3
0032735 // pc	0005886 // pl	0005102 // re	Toll-like recep	TLR4_HUMAN|TLR4
0008584 // m	0005886 // pl	0004888 // tra	Toll-like recep	TLR5_HUMAN|TLR5|TIL3
0002224 // to	0005886 // pl	0042802 // id	Toll-like recep	TLR6_HUMAN|TLR6
0007186 // G-	0005887 // in	0001540 // be	TM2 domain-c	TM2D1_HUMAN|TM2D1|BBP
0008150 // bi	0005887 // in	0005515 // pr	Transmembra	T4S1_HUMAN|TM4SF1|M3S1|TAAL6
	0016021 // in		Transmembra	T4S19_HUMAN|TM4SF19|OCTM4
1902042 // ne	0016021 // in	0005123 // de	Protein lifegua	LFG3_HUMAN|TMBIM1|LFG3|RECS1|PP1201|PSEC0158
0050848 // re	0016021 // in	0005515 // pr	Protein lifegua	LFG4_HUMAN|TMBIM4|GAAP|LFG4|CGI-119
0070059 // in	0005737 // cy	0031625 // ub	Bax inhibitor 1	BI1_HUMAN|TMBIM6|BI1|TEGT
0050910 // de	0009897 // ex	0005245 // vo	Transmembra	TMC1_HUMAN|TMC1
1903169 // re	0016021 // in	0005245 // vo	Transmembra	TMC2_HUMAN|TMC2|C20orf145|UNQ907/PRO1928
0006811 // io	0016021 // in		Transmembra	TMC3_HUMAN|TMC3
0006811 // io	0016021 // in		Transmembra	TMC4_HUMAN|TMC4
0006811 // io	0016021 // in		Transmembra	TMC5_HUMAN|TMC5|UNQ8238/PRO33604
0006811 // io	0016021 // in		Transmembra	TMC7_HUMAN|TMC7
0032091 // ne	0005783 // en	0005515 // pr	Transmembra	TMC8_HUMAN|TMC8|EVER2|EVIN2
0007029 // en	0016021 // in	0005515 // pr	Transmembra	TMCC1_HUMAN|TMCC1|KIAA0779
0042982 // ar	0016021 // in	0005515 // pr	Transmembra	TMCC2_HUMAN|TMCC2|KIAA0481|hucep-11
	0016021 // in	0005515 // pr	Transmembra	TMCC3_HUMAN|TMCC3|KIAA1145
0032469 // en	0030176 // in	0005262 // ca	Calcium load-a	TMCO1_HUMAN|TMCO1|TMCC4|PNAS-10|PNAS-136|U
	0016021 // in		Transmembra	TMCO4_HUMAN|TMCO4
0007165 // sig	0016021 // in	0004871 // sig	Transmembra	TMED4_HUMAN|TMED4|ERS25
0006810 // tra	0016021 // in		Protein TMED	A0A0A6YYA0_HUMAN|TMED7-TICAM2
0006810 // tra	0016021 // in		Protein TMED	TMED8_HUMAN|TMED8|FAM15B|L10
0007275 // m	0016021 // in		Tomoregulin-1	TEFF1_HUMAN|TMEFF1|C9orf2
0044319 // wo	0005576 // ex	0003674 // m	Tomoregulin-2	TEFF2_HUMAN|TMEFF2|HPP1|TENB2|TPEF|UNQ178/PI
0008150 // bi	0016021 // in	0003674 // m	Transmembra	TM104_HUMAN|TMEM104
	0016021 // in		Transmembra	TM105_HUMAN|TMEM105
0008150 // bi	0016020 // m	0003674 // m	Transmembra	T106A_HUMAN|TMEM106A
0008150 // bi	0016020 // m	0003674 // m	Transmembra	T106C_HUMAN|TMEM106C|EMOC
1990416 // ce	0010008 // en	0003674 // m	Transmembra	TM108_HUMAN|TMEM108|KIAA1690|RTLN|UNQ1875/
0070059 // in	0005886 // pl	0003674 // m	Transmembra	TM117_HUMAN|TMEM117
0008150 // bi	0016021 // in	0003674 // m	Transmembra	TM121_HUMAN|TMEM121|HHOLE
0008150 // bi	0016021 // in	0003674 // m	Transmembra	TM125_HUMAN|TMEM125
0008150 // bi	0005743 // m	0003674 // m	Complex I ass	T126B_HUMAN|TMEM126B|HT007
0000209 // pr	0016021 // in	1904264 // ub	E3 ubiquitin-p	TM129_HUMAN|TMEM129
	0005794 // Go		Transmembra	TM130_HUMAN|TMEM130|UNQ719/PRO1383

0044267 // ce	0005783 // er	0003674 // m	Transmembra	T132A_HUMAN|TMEM132A|HSPA5BP1|KIAA1583
0008150 // bi	0016021 // in	0003674 // m	Transmembra	T132B_HUMAN|TMEM132B|KIAA1786|KIAA1906
	0016021 // in	0005515 // pr	Transmembra	TM134_HUMAN|TMEM134
0009409 // re	0016021 // in		Transmembra	TM135_HUMAN|TMEM135
	0016021 // in		Transmembra	TM136_HUMAN|TMEM136
0060271 // cil	0016021 // in		Transmembra	TM138_HUMAN|TMEM138|HSPC196|HSPC198
0008150 // bi	0016021 // in	0003674 // m	Transmembra	TM143_HUMAN|TMEM143|UNQ5922/PRO19813
0034219 // ca	0016021 // in	0015144 // ca	Transmembra	TM144_HUMAN|TMEM144
0019236 // re	0016021 // in		Transmembra	TM145_HUMAN|TMEM145
	0016021 // in		Transmembra	TM14B_HUMAN|TMEM14B
0072659 // pr	0005764 // ly	0005515 // pr	Transmembra	T150A_HUMAN|TMEM150A|TMEM150
0034220 // io	0005765 // ly	0008381 // m	Transmembra	T150C_HUMAN|TMEM150C
	0016021 // in		Transmembra	T151A_HUMAN|TMEM151A|TMEM151
	0016021 // in		Transmembra	TM154_HUMAN|TMEM154
	0016021 // in		Transmembra	TM156_HUMAN|TMEM156
	0016021 // in	0042277 // pe	Transmembra	TM158_HUMAN|TMEM158|HBBP|RIS1
	0016021 // in	0005515 // pr	Promethin	TM159_HUMAN|TMEM159
0032526 // re	0016021 // in		Transmembra	T161A_HUMAN|TMEM161A|UNQ582/PRO1152
	0030285 // in	0008270 // zi	Transmembra	TM163_HUMAN|TMEM163
0046907 // in	0016021 // in		Protein kish-B	KISHB_HUMAN|TMEM167B|C1orf119|AD-020
	0030133 // tr		Transmembra	TM168_HUMAN|TMEM168
	0016021 // in		Transmembra	TM169_HUMAN|TMEM169
0007224 // sm	0016021 // in		Transmembra	TMM17_HUMAN|TMEM17
	0016021 // in		Transmembra	T170B_HUMAN|TMEM170B
0032608 // in	0005789 // er	0061507 // cy	Stimulator of	STING_HUMAN|TMEM173|ERIS|MITA|STING
0035751 // re	0016021 // in	0005267 // po	Endosomal/ly	TM175_HUMAN|TMEM175
0051480 // re	0016021 // in		Transmembra	T178A_HUMAN|TMEM178A|TMEM178|PSEC0131|UNQ
	0016021 // in		Transmembra	T178B_HUMAN|TMEM178B
0006810 // tr	0030659 // cy	0008201 // he	Transmembra	T184A_HUMAN|TMEM184A
0008150 // bi	0030133 // tr	0003674 // m	Transmembra	TM187_HUMAN|TMEM187|CXorf12|DXS9878E|ITBA1
0016567 // pr	0016021 // in	0061630 // ub	Transmembra	TM189_HUMAN|TMEM189|KUA
	0016021 // in	0005515 // pr	Transmembra	TMM19_HUMAN|TMEM19
	0016021 // in		Transmembra	T191A_HUMAN|TMEM191A
	0016021 // in		Transmembra	T191B_HUMAN|TMEM191B
	0016021 // in		Transmembra	T191C_HUMAN|TMEM191C
	0005764 // ly	0042803 // pr	Transmembra	TM192_HUMAN|TMEM192
1903670 // re	0016021 // in	0004415 // hy	Cell surface hy	TMEM2_HUMAN|TMEM2|KIAA1412
0007097 // nu	0000922 // sp	0005521 // la	Transmembra	TM201_HUMAN|TMEM201|NET5|SAMP1
	0016021 // in		Transmembra	TM205_HUMAN|TMEM205|UNQ501/PRO1018
0060271 // cil	0005829 // cy		Transmembra	TM216_HUMAN|TMEM216|HSPC244
	0016021 // in		Transmembra	TM217_HUMAN|TMEM217|C6orf128
0007399 // ne	0016021 // in		Transmembra	TM223_HUMAN|TMEM223
	0016021 // in		Transmembra	T229B_HUMAN|TMEM229B|C14orf83
	0016021 // in		Transmembra	TM236_HUMAN|TMEM236|FAM23A|FAM23B
	0016021 // in		Transmembra	TM238_HUMAN|TMEM238
	0016021 // in		Transmembra	TM240_HUMAN|TMEM240|C1orf70

	0016021 // in		Transmembra	TM243_HUMAN|TMEM243|C7orf23
	0016021 // in		Transmembra	TM246_HUMAN|TMEM246|C9orf125
	0016021 // in		Transmembra	TM249_HUMAN|TMEM249|C8orfK29
	0016021 // in	0005515 // pr	Transmembra	TMM25_HUMAN|TMEM25|UNQ2531/PRO6030
	0016021 // in		Transmembra	TM251_HUMAN|TMEM251|C14orf109
	0016021 // in		Transmembra	TM254_HUMAN|TMEM254|C10orf57
	0016021 // in		Transmembra	T255A_HUMAN|TMEM255A|FAM70A
	0016021 // in		Transmembra	T255B_HUMAN|TMEM255B|FAM70B
0006487 // pr	0016021 // in		Transmembra	TM258_HUMAN|TMEM258|C11orf10|HSPC005
	0016021 // in		Transmembra	TM263_HUMAN|TMEM263|C12orf23
0009607 // re	0016021 // in		Transmembra	TM265_HUMAN|TMEM265
	0016021 // in		Transmembra	TM266_HUMAN|TMEM266|C15orf27
	0016021 // in		Transmembra	TM267_HUMAN|TMEM267|C5orf28
0008150 // bi	0016021 // in	0003674 // m	Transmembra	TM268_HUMAN|TMEM268|C9orf91
	0016021 // in		Transmembra	TM269_HUMAN|TMEM269
0051957 // pc	0016021 // in	0008241 // pe	Collectrin	TMM27_HUMAN|TMEM27|UNQ679/PRO1312
0070863 // pc	0016021 // in	0015247 // ar	Cell cycle cont	CC50B_HUMAN|TMEM30B|CDC50B
0051292 // nu	0042470 // m	0005515 // pr	Transmembra	TMM33_HUMAN|TMEM33|DB83
	0016021 // in		Transmembra	TM35B_HUMAN|TMEM35B|ZMYM6NB
0034765 // re	0016021 // in	0005244 // vc	Voltage-deper	CCGL_HUMAN|TMEM37|PR
0071805 // pc	0031965 // nu	0005267 // pc	Trimeric intra	TM38A_HUMAN|TMEM38A
	0016021 // in		Transmembra	TMM40_HUMAN|TMEM40
0071763 // nu	0005788 // er	0005515 // pr	Transmembra	TMM43_HUMAN|TMEM43|UNQ2564/PRO6244
	0005765 // lys	0015189 // L-l	Transmembra	TMM44_HUMAN|TMEM44
	0016021 // in		Transmembra	TM45A_HUMAN|TMEM45A|DERP7|DNAPTP4
	0016021 // in		Transmembra	TM45B_HUMAN|TMEM45B
0008150 // bi	0016021 // in	0003674 // m	Transmembra	TMM47_HUMAN|TMEM47|BCMP1|TM4SF10
0008150 // bi	0016021 // in	0005515 // pr	Transmembra	TMM51_HUMAN|TMEM51|C1orf72
	0016021 // in		Transmembra	TMM52_HUMAN|TMEM52|UNQ3048/PRO9864
0008150 // bi	0016021 // in	0003674 // m	Transmembra	TM52B_HUMAN|TMEM52B|C12orf59|UNQ5927/PRO19
0008150 // bi	0016021 // in	0003674 // m	Transmembra	TMM54_HUMAN|TMEM54|BCLP|CAC1
0046856 // ph	0016021 // in	0034597 // ph	Type 2 phospl	TM55A_HUMAN|TMEM55A
0008150 // bi	0016021 // in	0003674 // m	Transmembra	TMM56_HUMAN|TMEM56
	0016021 // in		TMEM56-RWI	S4R434_HUMAN|TMEM56-RWDD3
0008150 // bi	0016021 // in	0003674 // m	Macoilin	MACOI_HUMAN|TMEM57
0090285 // ne	0000137 // Gc	0004175 // er	Transmembra	TMM59_HUMAN|TMEM59|C1orf8|HSPC001|UNQ169/F
	0016021 // in		Transmembra	TM59L_HUMAN|TMEM59L|BSMAP|C19orf4
	0016021 // in		Transmembra	TMM60_HUMAN|TMEM60|C7orf35|DC32
	0016021 // in		Transmembra	TMM61_HUMAN|TMEM61
0006811 // io	0016021 // in	0003676 // nu	CSC1-like prot	CSCL1_HUMAN|TMEM63A|KIAA0489|KIAA0792
0006811 // io	0016021 // in		CSC1-like prot	CSCL2_HUMAN|TMEM63B|C6orf110
0098655 // ca	0016021 // in	0005227 // ca	Calcium perm	CSC1_HUMAN|TMEM63C|C14orf171|CSC1
0010826 // ne	0036038 // M	0031005 // fil	Meckelin	MKS3_HUMAN|TMEM67|MKS3

0008152 // m	0016021 // in	0016746 // tra	Transmembra	TMM68_HUMAN|TMEM68
	0016021 // in		Transmembra	TMM69_HUMAN|TMEM69|C1orf154|HSPC229
0033615 // m	0005739 // m	0003674 // m	Transmembra	TMM70_HUMAN|TMEM70
	0016021 // in		Transmembra	TM74B_HUMAN|TMEM74B|C20orf46
0045684 // po	0016021 // in	0005515 // pr	Transmembra	TMM79_HUMAN|TMEM79|MATT
	0016021 // in		Transmembra	TMM80_HUMAN|TMEM80
	0016021 // in		Transmembra	TMM81_HUMAN|TMEM81|UNQ2788/PRO7178
	0016021 // in		Lysoplasmalo	TM86A_HUMAN|TMEM86A
0046485 // et	0016021 // in	0047408 // all	Lysoplasmalo	TM86B_HUMAN|TMEM86B
0042147 // re	0005829 // cy		Transmembra	TM87A_HUMAN|TMEM87A|PSEC0094
	0016021 // in		Transmembra	TM88B_HUMAN|TMEM88B
	0016021 // in		Transmembra	TMM89_HUMAN|TMEM89
	0016021 // in		Transmembra	TMM92_HUMAN|TMEM92|UNQ5801/PRO19608
0042632 // ch	0005791 // ro	0005515 // pr	Sigma intracel	SGMR2_HUMAN|TMEM97|MAC30|S2R
	0016021 // in		Transmembra	TMM98_HUMAN|TMEM98|UNQ536/PRO1079
0042472 // in	0016021 // in		Transmembra	TMIE_HUMAN|TMIE
0030049 // m	0005829 // cy	0003779 // ac	Tropomodulin	TMOD1_HUMAN|TMOD1|D9S57E|TMOD
0045745 // po	0005865 // st	0003779 // ac	Tropomodulin	TMOD2_HUMAN|TMOD2|NTMOD
0006355 // re	0005634 // nu	0003677 // DI	Lamina-associ	LAP2A_HUMAN|TMPO|LAP2
	0016021 // in	0016787 // hy	Transmembra	TMPPE_HUMAN|TMPPE
0006508 // pr	0005887 // in	0004252 // se	Transmembra	TM11A_HUMAN|TMPRSS11A|ECRG1|HATL1|HESP
0006508 // pr	0005887 // in	0004252 // se	Transmembra	TM11F_HUMAN|TMPRSS11F|HATL4
0006508 // pr	0016021 // in	0005044 // sc	Transmembra	TMPSD_HUMAN|TMPRSS13|MSP|TMPRSS11
0006508 // pr	0016021 // in	0005044 // sc	Enteropeptida	ENTK_HUMAN|TMPRSS15|ENTK|PRSS7
0007605 // se	0005789 // en	0004252 // se	Transmembra	TMPS3_HUMAN|TMPRSS3|ECHOS1|TADG12|UNQ323/P
0006898 // re	0016021 // in	0005044 // sc	Transmembra	TMPS4_HUMAN|TMPRSS4|TMPRSS3|UNQ776/PRO157
	0016021 // in		TMPRSS4 anti	A0A1B0GVV1_HUMAN|TMPRSS4-AS1
0006898 // re	0016021 // in	0005044 // sc	Transmembra	TMPS5_HUMAN|TMPRSS5
0055072 // iro	0005886 // pl	0004222 // m	Transmembra	TMPS6_HUMAN|TMPRSS6|UNQ354/PRO618
0006508 // pr	0005887 // in	0004252 // se	Transmembra	TMPS9_HUMAN|TMPRSS9
0007015 // ac	0005737 // cy	0005515 // pr	Thymosin bet	TYB10_HUMAN|TMSB10|PTMB10|THYB10
0030335 // po	0005737 // cy	0003785 // ac	Thymosin bet	A0A087X1C1_HUMAN|TMSB15B
0006396 // RN	0016021 // in		Transmembra	TMTC1_HUMAN|TMTC1|ARG99
0055074 // ca	0016021 // in	0005515 // pr	Transmembra	TMTC2_HUMAN|TMTC2
0030433 // ER	0005730 // nu	0005515 // pr	Transmembra	TMUB1_HUMAN|TMUB1|C7orf21|DULP|HOPS|SB144|
0006457 // pr	0016021 // in	0005515 // pr	Protein disulfi	TMX3_HUMAN|TMX3|KIAA1830|TXNDC10
0042060 // wo	0005614 // in	0045545 // sy	Tenascin	TENA_HUMAN|TNC|HXB
0008625 // ex	0005886 // pl	0005515 // pr	Tumor necros	TNFA_HUMAN|TNF|TNFA|TNFSF2
0051601 // ex	0005615 // ex	0000149 // SN	Tumor necros	TNAP2_HUMAN|TNFAIP2
0034144 // ne	0005737 // cy	0061578 // Ly	Tumor necros	TNAP3_HUMAN|TNFAIP3|OTUD7C
0043066 // ne	0005737 // cy	0005515 // pr	Tumor necros	TFIP8_HUMAN|TNFAIP8
0032007 // ne	0005737 // cy	0005515 // pr	Tumor necros	TP8L1_HUMAN|TNFAIP8L1
0070374 // po	0005886 // pl	0008526 // ph	Tumor necros	TP8L3_HUMAN|TNFAIP8L3|TIPE3
0007275 // m	0005622 // in	0005515 // pr	Tumor necros	TR10B_HUMAN|TNFRSF10B|DR5|KILLER|TRAILR2|TRICK

0006954 // in	0005887 // in	0004888 // tr	Tumor necros	TR10C_HUMAN|TNFRSF10C|DCR1|LIT|TRAILR3|TRID|U
0033209 // tu	0005886 // pl	0004872 // re	Tumor necros	TNR11_HUMAN|TNFRSF11A|RANK
0006954 // in	0005578 // pr	0005125 // cy	Tumor necros	TR11B_HUMAN|TNFRSF11B|OCIF|OPG
0033209 // tu	0009986 // ce	0005515 // pr	Tumor necros	TNR12_HUMAN|TNFRSF12A|FN14
0033209 // tu	0016021 // in		Tumor necros	TR13C_HUMAN|TNFRSF13C|BAFFR|BR3
0006954 // in	0005886 // pl	0001618 // vi	Tumor necros	TNR14_HUMAN|TNFRSF14|HVEA|HVEM|UNQ329/PRO5
0006954 // in	0005886 // pl	0005031 // tu	Tumor necros	TNR18_HUMAN|TNFRSF18|AITR|GITR|UNQ319/PRO36
0033209 // tu	0005622 // in	0004872 // re	Tumor necros	TNR19_HUMAN|TNFRSF19|TAJ|TROY|UNQ1888/PRO43
0051402 // ne	0031226 // in	0005031 // tu	Tumor necros	TNR21_HUMAN|TNFRSF21|DR6|UNQ437/PRO868
0006954 // in	0005576 // ex	0005031 // tu	Tumor necros	TNR25_HUMAN|TNFRSF25|APO3|DDR3|DR3|TNFRSF12
0007165 // si	0005576 // ex	0005515 // pr	Tumor necros	TNF10_HUMAN|TNFSF10|APO2L|TRAIL
0033209 // tu	0005576 // ex	0005164 // tu	Tumor necros	TNF12_HUMAN|TNFSF12|APO3L|DR3LG|UNQ181/PRO
0006955 // im	0005615 // ex	0005164 // tu	Protein TNFSF	A0A0A6YY99_HUMAN|TNFSF12-TNFSF13
0007165 // si	0005654 // nu	0005164 // tu	Tumor necros	TNF13_HUMAN|TNFSF13|APRIL|TALL2|ZTNF2|UNQ383
0033209 // tu	0005887 // in	0005102 // re	Tumor necros	TNF15_HUMAN|TNFSF15|TL1|VEGI
0042129 // re	0005886 // pl	0032813 // tu	Tumor necros	TNF18_HUMAN|TNFSF18|AITRL|GITRL|TL6|UNQ149/PR
0032700 // ne	0005887 // in	0032813 // tu	Tumor necros	TNFL4_HUMAN|TNFSF4|TXGP1
0006915 // ap	0005887 // in	0005164 // tu	Tumor necros	TNFL8_HUMAN|TNFSF8|CD30L|CD30LG
0070373 // ne	0005622 // in	0005515 // pr	TNFAIP3-inter	TNIP1_HUMAN|TNIP1|KIAA0113|NAF1
0038083 // pe	0005737 // cy	0005515 // pr	Non-receptor	TNK1_HUMAN|TNK1
0038083 // pe	0005768 // en	0004674 // pr	Activated CDC	ACK1_HUMAN|TNK2|ACK1
1904743 // ne	0097431 // m	0005515 // pr	Tankyrase-1	TNKS1_HUMAN|TNKS|PARP5A|PARPL|TIN1|TINF1|TNK
0010800 // po	0005720 // nu	0005515 // pr	182 kDa tanky	TB182_HUMAN|TNKS1BP1|KIAA1741|TAB182
0030049 // m	0005829 // cy	0031013 // tr	Troponin C, sl	TNNC1_HUMAN|TNNC1|TNNC
0030049 // m	0005829 // cy	0005515 // pr	Troponin I, slo	TNNI1_HUMAN|TNNI1
0030049 // m	0005634 // nu	0003779 // ac	Troponin I, fas	TNNI2_HUMAN|TNNI2
0002027 // re	0005737 // cy	0004674 // pr	Serine/threon	TNI3K_HUMAN|TNNI3K|CARK
0014883 // tr	0005829 // cy	0005515 // pr	Troponin T, sl	TNNT1_HUMAN|TNNT1|TNT
0051592 // re	0005829 // cy	0030172 // tr	Troponin T, ca	TNNT2_HUMAN|TNNT2
0030049 // m	0005829 // cy	0031013 // tr	Troponin T, fa	TNNT3_HUMAN|TNNT3
0000060 // pr	0005737 // cy	0003723 // RN	Transportin-1	TNPO1_HUMAN|TNPO1|KPNB2|MIP1|TRN
0006342 // ch	0005739 // m	0003682 // ch	Trinucleotide	TNC18_HUMAN|TNRC18|CAGL79|KIAA1856
0035194 // po	0005829 // cy	0003723 // RN	Trinucleotide	TNR6A_HUMAN|TNRC6A|CAGH26|KIAA1460|TNRC6
0060213 // po	0005829 // cy	0005515 // pr	Trinucleotide	TNR6C_HUMAN|TNRC6C|KIAA1582
0010761 // fib	0005737 // cy	0003723 // RN	Tensin-1	TENS1_HUMAN|TNS1|TNS
0008285 // ne	0005925 // fo	0005515 // pr	Tensin-2	TNS2_HUMAN|TNS2|KIAA1075|TENC1
0016477 // ce	0005829 // cy	0005515 // pr	Tensin-3	TENS3_HUMAN|TNS3|TEM6|TENS1|TPP
0006915 // ap	0005856 // cy	0005515 // pr	Tensin-4	TENS4_HUMAN|TNS4|CTEN|PP14434
0030036 // ac	0005622 // in	0005178 // in	Tenascin-X	TENX_HUMAN|TNXB|HXBL|TNX|TNXB1|TNXB2|XB
0030514 // ne	0005634 // nu	0005515 // pr	Protein Tob1	TOB1_HUMAN|TOB1|TOB|TROB1
0034472 // sn	0005730 // nu	0005515 // pr	Target of EGR	TOE1_HUMAN|TOE1
1905515 // no	0005737 // cy		TOG array reg	TGRM1_HUMAN|TOGARAM1|FAM179B|KIAA0423
0007267 // ce	0005737 // cy	0005515 // pr	Toll-interactin	TOLIP_HUMAN|TOLLIP
0006886 // in	0070062 // ex	0005515 // pr	TOM1-like pro	TM1L2_HUMAN|TOM1L2

0016236 // m	0005741 // m	0005515 // pr	Mitochondria	TOM20_HUMAN|TOMM20|KIAA0016
0016236 // m	0005743 // m	0005515 // pr	Mitochondria	TOM22_HUMAN|TOMM22|TOM22
0006626 // pr	0005741 // m	0005515 // pr	Mitochondria	TOM34_HUMAN|TOMM34|URCC3
0016236 // m	0031307 // in	0005515 // pr	Mitochondria	TOM40_HUMAN|TOMM40|C19orf1|PEREC1|TOM40
0016236 // m	0005739 // m	0008565 // pr	Mitochondria	TOM5_HUMAN|TOMM5|C9orf105|TOM5
0016236 // m	0005739 // m		Mitochondria	TOM6_HUMAN|TOMM6|OBTP|TOM6
0006265 // DI	0005654 // nu	0003723 // RI	DNA topoison	TOP1_HUMAN|TOP1
0006265 // DI	0005654 // nu	0003677 // DI	DNA topoison	TOP3A_HUMAN|TOP3A|TOP3
0051457 // m	0016605 // PI	0003823 // ar	E3 ubiquitin-p	TOPRS_HUMAN|TOPORS|LUN|TP53BPL
0071763 // nu	0016021 // in	0005521 // la	Torsin-1A-inte	TOIP1_HUMAN|TOR1AIP1|LAP1
0032781 // po	0005783 // er	0051117 // AT	Torsin-1A-inte	TOIP2_HUMAN|TOR1AIP2|IFRG15|LULL1
0051085 // ch	0005788 // er	0005524 // AT	Torsin-2A	TOR2A_HUMAN|TOR2A|TORP1|UNQ6408/PRO21181
0002576 // pl	0005576 // ex	0005524 // AT	Torsin-4A	TOR4A_HUMAN|TOR4A|C9orf167
	0005634 // nu	0003677 // DI	Thymocyte se	TOX_HUMAN|TOX|KIAA0808
0006366 // tr	0005654 // nu	0000978 // RI	TOX high mob	TOX2_HUMAN|TOX2|C20orf100|GCX1
	0000784 // nu	0005515 // pr	TOX high mob	TOX4_HUMAN|TOX4|C14orf92|KIAA0737
0006977 // DI	0005654 // nu	0005515 // pr	Cellular tumo	P53_HUMAN|TP53|P53
0006915 // ap	0005759 // m	0003674 // m	p53-regulatec	TPIP1_HUMAN|TP53AIP1
0014070 // re	0016021 // in	0003674 // m	Tumor protei	P5I13_HUMAN|TP53I13|DSCP1
0006739 // N/	0005829 // cy	0048038 // qu	Quinone oxid	QORX_HUMAN|TP53I3|PIG3
0098869 // ce	0016605 // PI	0005515 // pr	Tumor protei	T53I1_HUMAN|TP53INP1|P53DINP1|SIP
0008033 // tR	0016020 // m	0005515 // pr	TP53-regulati	PRPK_HUMAN|TP53RK|C20orf64|PRPK
0006974 // ce		0003674 // m	TP53-target g	T53G1_HUMAN|TP53TG1|P53TG1|TP53AP1
0002064 // ep	0005654 // nu	0005515 // pr	Tumor protei	P63_HUMAN|TP63|KET|P63|P73H|P73L|TP73L
0045893 // po	0005654 // nu	0019901 // pr	Tumor protei	P73_HUMAN|TP73|P73
0006874 // ce	0010008 // er	0042802 // id	Two pore calc	TPC2_HUMAN|TPCN2|TPC2
2001235 // po	0048471 // pe	0042802 // id	Tumor protei	TPD53_HUMAN|TPD52L1
		0003723 // RI	Tumor protei	TPD54_HUMAN|TPD52L2
0007288 // sp	0005737 // cy	0008017 // m	Tubulin polyg	TPGS1_HUMAN|TPGS1|C19orf20
0006094 // gl	0005829 // cy	0004807 // tri	Triosephosph	TPIS_HUMAN|TPI1|TPI
0009229 // th	0005829 // cy	0030975 // th	Thiamin pyro	TPK1_HUMAN|TPK1
0051496 // po	0005862 // m	0005200 // st	Tropomyosin	TPM1_HUMAN|TPM1|C15orf13|TMSA
0030049 // m	0005829 // cy	0042802 // id	Tropomyosin	TPM2_HUMAN|TPM2|TMSB
0030049 // m	0070062 // ex	0005515 // pr	Tropomyosin	TPM3_HUMAN|TPM3
0098869 // ce	0005886 // pl	0020037 // he	Thyroid perox	PERT_HUMAN|TPO
0032273 // po	0097427 // m	0015631 // tu	Tubulin polym	TPPP_HUMAN|TPPP|TPPP1
	0005737 // cy		Tumor protei	TPRG1_HUMAN|TPRG1|FAM79B
0008033 // tR	0005634 // nu	0005515 // pr	EKC/KEOPS co	TPRKB_HUMAN|TPRKB|CGI-121|My019
0007605 // se	0032420 // st	0005515 // pr	Taperin	TPRN_HUMAN|TPRN|C9orf75
0006874 // ce	0005737 // cy	0003723 // RI	Translationall	TCTP_HUMAN|TPT1

0035335 // pe	0016021 // in	0008138 // pr	Putative tyros	TPTE_HUMAN|TPTE
0035335 // pe	0016021 // in	0004725 // pr	Phosphatidyli	TPTE2_HUMAN|TPTE2|TPIP
0016055 // W	0031301 // in	0004175 // en	Metalloprotea	TIKI1_HUMAN|TRABD2A|C2orf89|TIKI1
0051092 // po	0005886 // pl	0005515 // pr	Tumor necros	TRADD_HUMAN|TRADD
2001236 // re	0005737 // cy	0005515 // pr	TNF receptor-	TRAF1_HUMAN|TRAF1|EBI6
0033209 // tu	0005829 // cy	0005515 // pr	TNF receptor-	TRAF3_HUMAN|TRAF3|CAP1|CRAF1
0031333 // ne	0030992 // in	0008017 // m	TRAF3-interac	MIPT3_HUMAN|TRAF3IP1|IFT54|MIPT3
0048305 // im	0005622 // in	0005102 // re	Adapter prote	CIKS_HUMAN|TRAF3IP2|C6orf2|C6orf4|C6orf5|C6orf6
	0016021 // in	0005515 // pr	TRAF3-interac	T3JAM_HUMAN|TRAF3IP3|T3JAM
0051092 // po	0009898 // cy	0005515 // pr	TNF receptor-	TRAF5_HUMAN|TRAF5|RNF84
0045824 // ne		0005515 // pr	TRAF-type zin	TRAD1_HUMAN|TRAFD1|FLN29
0048813 // de	0005634 // nu	0005102 // re	Trafficking kin	TRAK2_HUMAN|TRAK2|ALS2CR3|KIAA0549
0015031 // pr	0016021 // in		Translocating	TR1L1_HUMAN|TRAM1L1
0006888 // EF	0005829 // cy	0005515 // pr	Trafficking pro	TPC11_HUMAN|TRAPPC11|C4orf41
0048208 // CO	0005793 // en	0005515 // pr	Trafficking pro	TPC2A_HUMAN|TRAPPC2|SEDL
0048208 // CO	0005829 // cy	0005515 // pr	Trafficking pro	H3BP13_HUMAN|TRAPPC2L
0048208 // CO	0005829 // cy	0017112 // Ra	Trafficking pro	TPPC4_HUMAN|TRAPPC4|SBDN|CGI-104|HSPC172|PTD
0048208 // CO	0005829 // cy	0005515 // pr	Trafficking pro	TPPC5_HUMAN|TRAPPC5
0048208 // CO	0005829 // cy	0005515 // pr	Trafficking pro	TPC6A_HUMAN|TRAPPC6A|HSPC289
0050853 // B	0016021 // in	0003823 // an	T-cell recepto	TRBC2_HUMAN|TRBC2|TCRBC2
0006400 // tR	0005654 // nu	0008175 // tR	tRNA (cytosin	TRDMT_HUMAN|TRDMT1|DNMT2
0060047 // he	0030314 // ju	0030674 // pr	Triadin	TRDN_HUMAN|TRDN
0005993 // tre	0070062 // ex	0004555 // alp	Trehalase	TREA_HUMAN|TREH|TREA
0006357 // re	0000118 // his	0008301 // DN	Transcriptiona	TREF1_HUMAN|TRERF1|BCAR2|RAPA|TREP132
0090305 // nu	0005634 // nu	0003676 // nu	Three prime r	TREX2_HUMAN|TREX2
0043171 // pe	0005737 // cy	0004177 // an	Thyrotropin-re	TRHDE_HUMAN|TRHDE|UNQ2507/PRO5995
0043154 // ne	0005739 // m	0002039 // p5	TP53-regulate	TRIA1_HUMAN|TRIAP1|15E1.1|HSPC132
0014912 // ne	0005634 // nu	0031434 // m	Tribbles homo	TRIB1_HUMAN|TRIB1|C8FW|GIG2|TRB1
0032436 // po	0005634 // nu	0008134 // tra	Tribbles homo	TRIB2_HUMAN|TRIB2|TRB2
0051443 // po	0005634 // nu	0005515 // pr	Tribbles homo	TRIB3_HUMAN|TRIB3|C20orf97|NIPK|SKIP3|TRB3
0050768 // ne	0005737 // cy	0008134 // tra	E3 ubiquitin-p	TRI11_HUMAN|TRIM11|RNF92
1902187 // ne	0005622 // in	0005515 // pr	Tripartite mot	TRI15_HUMAN|TRIM15|RNF93|ZNF178|ZNFB7
0050718 // po	0005829 // cy	0019966 // in	Tripartite mot	TRI16_HUMAN|TRIM16|EBBP
	0005829 // cy		Tripartite mot	TR16L_HUMAN|TRIM16L|TRIM70
0032880 // re	0005622 // in	0005515 // pr	E3 ubiquitin-p	TRI17_HUMAN|TRIM17|RBCC|RNF16|TERF
0006355 // re	0005634 // nu	0003714 // tra	E3 ubiquitin-p	TRI22_HUMAN|TRIM22|RNF94|STAF50
0016032 // vi	0070062 // ex	0005515 // pr	E3 ubiquitin-p	TRI23_HUMAN|TRIM23|ARD1|ARFD1|RNF46
0055074 // ca	0005829 // cy	0035064 // m	Transcription	TIF1A_HUMAN|TRIM24|RNF82|TIF1|TIF1A
0045087 // in	0005829 // cy	0005515 // pr	Zinc finger pro	TRI27_HUMAN|TRIM27|RFP|RNF76
0006366 // tra	0005913 // ce	0005515 // pr	Tripartite mot	TRI29_HUMAN|TRIM29|ATDC

0007399 // ne	0005737 // cy	0005515 // pr	Tripartite mot	TRIM3_HUMAN|TRIM3|BERP|RNF22|RNF97
0046597 // ne	0005739 // m	0008270 // zir	E3 ubiquitin-p	TRI31_HUMAN|TRIM31|C6orf13|RNF
0051607 // de	0005737 // cy	0008270 // zir	Tripartite mot	TRI34_HUMAN|TRIM34|IFP1|RNF21
0051726 // re	0005829 // cy	0004842 // ub	E3 ubiquitin-p	TRI36_HUMAN|TRIM36|RBCC728|RNF98
0046598 // pc	0005829 // cy	0005515 // pr	E3 ubiquitin-p	TRI38_HUMAN|TRIM38|RNF15|RORET
2000059 // ne	0005829 // cy	0005515 // pr	E3 ubiquitin-p	TRI39_HUMAN|TRIM39|RNF23|TFP
0010468 // re	0005622 // in	0005515 // pr	Tripartite mot	TRI44_HUMAN|TRIM44|DIPB
0007409 // ax	1904115 // ax	0008270 // zir	Tripartite mot	TRI46_HUMAN|TRIM46|TRIFIC
	0005829 // cy	0008270 // zir	Tripartite mot	TRI47_HUMAN|TRIM47|GOA|RNF100
0007165 // sig	0005874 // m	0005515 // pr	Tripartite mot	TRI55_HUMAN|TRIM55|MURF2|RNF29
0032608 // in	0005829 // cy	0003723 // RN	E3 ubiquitin-p	TRI56_HUMAN|TRIM56|RNF109
0045087 // in	0016021 // in	0005515 // pr	Tripartite mot	TRI59_HUMAN|TRIM59|RNF104|TRIM57|TSBF1
2000679 // pc	0005737 // cy	0008270 // zir	Tripartite mot	TRIM6_HUMAN|TRIM6|RNF89
	0005622 // in	0008270 // zir	TRIM6-TRIM3	B2RNG4_HUMAN|TRIM6-TRIM34
	0005622 // in	0008270 // zir	Putative tripa	TRI61_HUMAN|TRIM61|RNF35
0051865 // pr	0005794 // Gc	0050681 // ar	E3 ubiquitin-p	TRI68_HUMAN|TRIM68|GC109|RNF137|SS56
	0005829 // cy	0008270 // zir	Tripartite mot	TRI73_HUMAN|TRIM73|TRIM50B
	0005622 // in	0008270 // zir	Tripartite mot	TRI77_HUMAN|TRIM77|TRIM77P
0045087 // in	0016605 // PN	0042802 // id	Probable E3 u	TRIM8_HUMAN|TRIM8|GERP|RNF27
0035544 // ne	0005737 // cy	0000149 // SN	E3 ubiquitin-p	TRIM9_HUMAN|TRIM9|KIAA0282|RNF91
0032526 // re		0005515 // pr	Probable E3 u	TRIMM_HUMAN|TRIML2|SPRYD6
0007185 // tra	0005829 // cy	0004674 // pr	Triple functior	TRIO_HUMAN|TRIO
0051016 // ba	0005737 // cy	0031625 // ub	TRIO and F-ac	TARA_HUMAN|TRIOBP|KIAA1662|TARA|HRIHFB2122
1901315 // ne	0005829 // cy	0004842 // ub	E3 ubiquitin-p	TRIPC_HUMAN|TRIP12|KIAA0045|ULF
0007130 // sy	0005634 // nu	0005515 // pr	Pachytene che	PCH2_HUMAN|TRIP13|PCH2
0008588 // re	0045323 // in	0005515 // pr	Thyroid recep	TRIP6_HUMAN|TRIP6|OIP1
	0016021 // in		Triple QxxK/R	TRIQK_HUMAN|TRIQK|C8orf83
0006400 // tR	0005634 // nu	0003723 // RN	tRNA (guanine	TRM1_HUMAN|TRMT1
0000964 // m	0005759 // m	0003723 // RN	Mitochondria	MRRP1_HUMAN|TRMT10C|MRPP1|RG9MTD1
0018364 // pe	0005829 // cy	0005515 // pr	Multifunction	TR112_HUMAN|TRMT112|AD-001|HSPC152|HSPC170
0008033 // tR		0005515 // pr	tRNA wybutos	TYW2_HUMAN|TRMT12|TRM12|TYW2
0001510 // RN		0003723 // RN	tRNA (uracil-5	TRM2A_HUMAN|TRMT2A|HTF9C
0070901 // m	0005759 // m	0052906 // tR	tRNA (guanine	TRM5_HUMAN|TRMT5|KIAA1393|TRM5
0006400 // tR	0031515 // tR	0016429 // tR	tRNA (adenine	TRM61_HUMAN|TRMT61A|C14orf172|TRM61
0042780 // tR	0005739 // m	0034062 // RN	CCA tRNA nuc	TRNT1_HUMAN|TRNT1|CGI-47
0007156 // hc	0005886 // pl	0005515 // pr	Trophinin	TROP_HUMAN|TRO|KIAA1114|MAGED3
0070588 // ca	0005887 // in	0005262 // ca	Transient rece	TRPA1_HUMAN|TRPA1|ANKTM1
0051592 // re	0005886 // pl	0005515 // pr	Short transien	TRPC1_HUMAN|TRPC1|TRP1
0007338 // sir	0005886 // pl	0005515 // pr	Short transien	TRPC3_HUMAN|TRPC3|TRP3
0048709 // ol	0009986 // ce	0015279 // st	Short transien	TRPC4_HUMAN|TRPC4
0051928 // pc	0005886 // pl	0005515 // pr	Short transien	TRPC6_HUMAN|TRPC6|TRP6
0007338 // sir	0005801 // cis	0005515 // pr	Short transien	TRPC7_HUMAN|TRPC7|TRP7

0035584 // ca	0005886 // pl	0015278 // ca	Transient rece	TRPM2_HUMAN|TRPM2|EREG1|KNP3|LTRPC2|TRPC7
0051262 // pr	0016021 // in	0005227 // ca	Transient rece	TRPM3_HUMAN|TRPM3|KIAA1616|LTRPC3
0042310 // va	0005886 // pl	0005262 // ca	Transient rece	TRPM4_HUMAN|TRPM4|LTRPC4
0009636 // re	0016324 // ap	0004674 // pr	Transient rece	TRPM6_HUMAN|TRPM6|CHAK2
0000122 // ne	0005634 // nu	0000977 // RN	Zinc finger tra	TRPS1_HUMAN|TRPS1
0006388 // tR		0000215 // tR	tRNA 2'-phosp	TRPT1_HUMAN|TRPT1
0007600 // se	0042470 // m	0005261 // ca	Transient rece	TRPV2_HUMAN|TRPV2|VRL
0007231 // os	0005886 // pl	0015275 // st	Transient rece	TRPV4_HUMAN|TRPV4|VRL2|VROAC
1901214 // re	0005829 // cy	0032794 // G	Hamartin	TSC1_HUMAN|TSC1|KIAA0243|TSC
0006355 // re	0005634 // nu	0043426 // M	TSC22 domair	T22D3_HUMAN|TSC22D3|DSIPI|GILZ
0006351 // tr	0005634 // nu	0005515 // pr	TSC22 domair	T22D4_HUMAN|TSC22D4|THG1|TILZ2
0090502 // RN	0005730 // nu	0000213 // tR	tRNA-splicing	SEN15_HUMAN|TSEN15|C1orf19|SEN15
0006388 // tR	0000214 // tR	0005515 // pr	tRNA-splicing	SEN2_HUMAN|TSEN2|SEN2
0006357 // re	0005634 // nu	0003677 // DN	Teashirt homo	TSH2_HUMAN|TSHZ2|C20orf17|TSH2|ZNF218
0007188 // ad	0005886 // pl	0004930 // G-	Tsukushin	TSK_HUMAN|TSKU|E2IG4|LRRC54|TSK|UNQ850/PRO17
	0005615 // ex	0005125 // cy	Thymic strom	TSLP_HUMAN|TSLP
0006310 // DN	0005829 // cy	0042802 // id	Translin	TSN_HUMAN|TSN
0007283 // sp	0005829 // cy	0003723 // RN	Translin-assoc	TSNAX_HUMAN|TSNAX|TRAX
0007283 // sp	0048471 // pe	0003674 // m	Translin-assoc	TXIP1_HUMAN|TSNAXIP1|TXI1
0008283 // ce	0043231 // in	0005515 // pr	Tetraspanin-1	TSN1_HUMAN|TSPAN1
0007166 // ce	0005887 // in		Tetraspanin-1	TSN11_HUMAN|TSPAN11
0007166 // ce	0005887 // in	0042813 // W	Tetraspanin-1	TSN12_HUMAN|TSPAN12|NET2|TM4SF12|UNQ774/PRC
0007166 // ce	0005887 // in	0005246 // ca	Tetraspanin-1	TSN13_HUMAN|TSPAN13|NET6|TM4SF13|UNQ260/PRC
0008593 // re	0070062 // ex	0019899 // er	Tetraspanin-1	TSN15_HUMAN|TSPAN15|NET7|TM4SF15|UNQ677/PRC
0007166 // ce	0016021 // in		Tetraspanin-1	TSN16_HUMAN|TSPAN16|TM4SF16
0072594 // es	0000151 // ub	0019899 // er	Tetraspanin-1	TSN17_HUMAN|TSPAN17|FBXO23|TM4SF17
0007166 // ce	0005887 // in		Putative tetra	TSN19_HUMAN|TSPAN19
0042552 // m	0016021 // in	0005515 // pr	Tetraspanin-2	TSN2_HUMAN|TSPAN2
0008284 // pc	0016020 // m		Tetraspanin-3	TSN31_HUMAN|TSPAN31|SAS
0051604 // pr	0009986 // ce	0019899 // er	Tetraspanin-3	TSN33_HUMAN|TSPAN33|PEN
0006461 // pr	0031982 // ve	0005515 // pr	Tetraspanin-4	TSN4_HUMAN|TSPAN4|NAG2|TM4SF7
0072659 // pr	0005886 // pl	0019899 // er	Tetraspanin-5	TSN5_HUMAN|TSPAN5|TM4SF9
0039532 // ne	0070062 // ex	0004871 // sig	Tetraspanin-6	TSN6_HUMAN|TSPAN6|TM4SF6|UNQ767/PRO1560
0016032 // vi	0005887 // in		Tetraspanin-7	TSN7_HUMAN|TSPAN7|A15|DXS1692E|MXS1|TM4SF2
0030195 // ne	0070062 // ex	0005178 // in	Tetraspanin-8	TSN8_HUMAN|TSPAN8|TM4SF3
0007166 // ce	0097197 // te	0003674 // m	Tetraspanin-9	TSN9_HUMAN|TSPAN9|NET5
0007605 // se	0032420 // st	0003674 // m	Thrombospon	TSEAR_HUMAN|TSPEAR|C21orf29
0007165 // sig	0016021 // in	0017127 // ch	Translocator p	TSPO_HUMAN|TSPO|BZRP|MBR
0006334 // nu	0005634 // nu		Putative testis	TSY26_HUMAN|TSPY26P|TSPYL3
0008156 // ne	0005634 // nu	0000182 // rD	Testis-specific	TSYL2_HUMAN|TSPYL2|CDA1|DENTT|TSPX|HRIHFB2216
0071480 // ce	0005634 // nu	0005515 // pr	Testis-specific	TSYL5_HUMAN|TSPYL5|KIAA1750
0006364 // rR	0030688 // pr	0003723 // RN	Pre-rRNA-proc	TSR1_HUMAN|TSR1|KIAA1401
0000154 // rR	0005829 // cy	0016740 // tra	Ribosome bio	TSR3_HUMAN|TSR3|C16orf42|UND313L

		0005515 // pr	Protein TSSC4	TSSC4_HUMAN|TSSC4
0007275 // m	0001669 // ac	0004674 // pr	Testis-specific	TSSK2_HUMAN|TSSK2|DGSG|SPOGA2|STK22B
0007275 // m	0005634 // nu	0005515 // pr	Testis-specific	TSSK3_HUMAN|TSSK3|SPOGA3|STK22C
0007275 // m	0005737 // cy	0000287 // m	Testis-specific	TSSK6_HUMAN|TSSK6|SSTK|FKSG82
0030855 // ep	0005739 // m	0008097 // 5S	Thiosulfate su	THTR_HUMAN|TST
	0036464 // cy		Thiosulfate su	TSTD1_HUMAN|TSTD1|KAT
0008360 // re	0005737 // cy	0005524 // AT	Tau-tubulin ki	TTBK1_HUMAN|TTBK1|BDTK|KIAA1855
0006457 // pr	0005829 // cy	0005515 // pr	Tetratricopep	TTC1_HUMAN|TTC1|TPR1
0061512 // pr	0005929 // cil		Tetratricopep	TT21A_HUMAN|TTC21A|STI2
0034976 // re	0005737 // cy	0005515 // pr	Tetratricopep	TT23L_HUMAN|TTC23L
	0005856 // cy	0005515 // pr	Tetratricopep	TTC25_HUMAN|TTC25
0007286 // sp	0005929 // cil	0005515 // pr	Intraflagellar t	IFT56_HUMAN|TTC26|IFT56
0007346 // re	0000922 // sp	0019900 // ki	Tetratricopep	TTC28_HUMAN|TTC28|KIAA1043|TPRBK
0060271 // cil	0005929 // cil	0005515 // pr	Tetratricopep	TT30B_HUMAN|TTC30B
		0005515 // pr	Tetratricopep	TTC32_HUMAN|TTC32
	0070062 // ex		Tetratricopep	TTC38_HUMAN|TTC38
0008150 // bi	0005575 // ce	0003674 // m	Tetratricopep	TT39A_HUMAN|TTC39A|C1orf34|KIAA0452
	0005737 // cy		Putative tetra	TTC41_HUMAN|TTC41P|GNN
0046854 // ph	0005829 // cy	0005515 // pr	Tetratricopep	TTC7B_HUMAN|TTC7B|TTC7L1
0048560 // es	0005829 // cy	0005515 // pr	Tetratricopep	TTC8_HUMAN|TTC8|BBS8
	0005829 // cy		TELO2-interac	TTI2_HUMAN|TTI2|C8orf41
0030516 // re	0005623 // ce	0005524 // AT	Tubulin--tyros	TTL_HUMAN|TTL
0018094 // pr	0005829 // cy	0005515 // pr	Inactive polyg	TTL10_HUMAN|TTLL10
0006464 // ce		0003824 // ca	Tubulin--tyros	TTL12_HUMAN|TTLL12|KIAA0153
0060271 // cil	0005930 // ax	0005524 // AT	Tubulin mono	TTLL3_HUMAN|TTLL3|PRO0207
0060041 // re	0005829 // cy	0070740 // tu	Tubulin polygl	TTLL5_HUMAN|TTLL5|KIAA0998|STAMP
0007399 // ne	0005829 // cy	0048487 // be	Tubulin polygl	TTLL7_HUMAN|TTLL7
0018094 // pr	0005829 // cy	0070736 // pr	Protein mono	TTLL8_HUMAN|TTLL8
0010628 // po	0030018 // Z	0005515 // pr	Titin	TITIN_HUMAN|TTN
0006810 // tra	0005622 // in	0005215 // tra	Alpha-tocoph	TTPAL_HUMAN|TTPAL|C20orf121
0031589 // ce	0016021 // in	0005509 // ca	Protein tweet	TTYH1_HUMAN|TTYH1
1902476 // ch	0005886 // pl	0005515 // pr	Protein tweet	TTYH2_HUMAN|TTYH2|C17orf29
1902476 // ch	0070062 // ex	0005254 // ch	Protein tweet	TTYH3_HUMAN|TTYH3|KIAA1691
0000086 // G2	0005829 // cy	0019904 // pr	Tubulin alpha-	TBA1A_HUMAN|TUBA1A|TUBA3
0030705 // cy	0043209 // m	0003924 // GT	Tubulin alpha-	TBA1B_HUMAN|TUBA1B

0000086 // G2	0005829 // cy	0003924 // GT	Tubulin alpha-	TBA4A_HUMAN|TUBA4A|TUBA1
0007010 // cy	0005737 // cy	0003924 // GT	Tubulin alpha-	TBA8_HUMAN|TUBA8|TUBAL2
0007010 // cy	0005737 // cy	0003924 // GT	Tubulin alpha	TBAL3_HUMAN|TUBAL3
0000086 // G2	0005576 // ex	0019904 // pr	Tubulin beta c	TBB5_HUMAN|TUBB|TUBB5|OK/SW-cl.56
0051225 // sp	0070062 // ex	0003924 // GT	Tubulin beta-1	TBB1_HUMAN|TUBB1
0007017 // m	1903561 // ex	0003924 // GT	Tubulin beta-2	TBB2A_HUMAN|TUBB2A|TUBB2
0007017 // m	0005737 // cy	0003924 // GT	Tubulin beta-3	TBB3_HUMAN|TUBB3|TUBB4
0000086 // G2	0005829 // cy	0003924 // GT	Tubulin beta-4	TBB4A_HUMAN|TUBB4A|TUBB4|TUBB5
0007017 // m	0005737 // cy	0003924 // GT	Tubulin beta-6	TBB6_HUMAN|TUBB6
0007098 // ce	0000242 // pe	0003924 // GT	Tubulin epsilo	TBE_HUMAN|TUBE1|TUBE
0000086 // G2	0000930 // ga	0003924 // GT	Tubulin gamm	TBG1_HUMAN|TUBG1|TUBG
1903546 // pr	0045202 // sy	0005546 // ph	Tubby-related	TULP1_HUMAN|TULP1|TUBL1
1903546 // pr	0005929 // cil	0005515 // pr	Tubby-related	TULP3_HUMAN|TULP3|TUBL3
0061512 // pr	0005829 // cy	0035091 // ph	Tubby-related	TULP4_HUMAN|TULP4|KIAA1397|TUBL4|TUSP
0006954 // in	0005739 // m	0005515 // pr	Tumor suppre	TUSC2_HUMAN|TUSC2|C3orf11|FUS1|LGCC|PDAP2
0071044 // hi	0005847 // m	0004652 // po	Speckle target	STPAP_HUMAN|TUT1|RBM21
0009306 // pr	0030173 // in		Golgi apparatu	TV23C_HUMAN|TVP23C|FAM18B2|Nbla10383
0010976 // po	0005737 // cy	0003723 // RN	Twinfilin-2	TWF2_HUMAN|TWF2|PTK9L|MSTP011
0001649 // os	0005634 // nu	0005515 // pr	Twist-related	TWST1_HUMAN|TWIST1|BHLHA38|TWIST
0006264 // m	0042645 // m	0003697 // sin	Twinkle prote	PEO1_HUMAN|TWNK|C10orf2|PEO1
0030900 // fo	0070062 // ex	0050431 // tra	Twisted gastru	TWSG1_HUMAN|TWSG1|TSG|PSEC0250
0038083 // pe	0005737 // cy	0000978 // RN	Tyrosine-prote	TXK_HUMAN|TXK|PTK4|RLK
	0005737 // cy	0005515 // pr	Beta-taxilin	TXLNB_HUMAN|TXLNB|C6orf198|MDP77
0055114 // ox	0005739 // m	0005515 // pr	Thioredoxin, r	THIOM_HUMAN|TXN2|TRX2
0045454 // ce	0005623 // ce		Thioredoxin d	TXD15_HUMAN|TXNDC15|C5orf14|UNQ335/PRO534
0008150 // bi	0005788 // en	0005515 // pr	Thioredoxin d	TXD16_HUMAN|TXNDC16|ERP90|KIAA1344
0098869 // ce	0070062 // ex	0005515 // pr	Thioredoxin d	TXD17_HUMAN|TXNDC17|TXNL5
0006457 // pr	0005576 // ex	0003756 // pr	Thioredoxin d	TXND5_HUMAN|TXNDC5|TLP46|UNQ364/PRO700
0008616 // qu	0030496 // m	0005515 // pr	Thioredoxin d	TXND9_HUMAN|TXNDC9|APACD
0055114 // ox	0070062 // ex	0016671 // ox	Thioredoxin-li	TXNL1_HUMAN|TXNL1|TRP32|TXL|TXNL
0000245 // sp	0005654 // nu	0005515 // pr	Thioredoxin-li	TXN4A_HUMAN|TXNL4A|DIM1|TXNL4
0055114 // ox	0005829 // cy	0004791 // th	Thioredoxin re	TRXR1_HUMAN|TXNRD1|GRIM12|KDRF
0038083 // pe	0005737 // cy	0004715 // no	Non-receptor	TYK2_HUMAN|TYK2
0006206 // py	0005829 // cy	0008083 // gr	Thymidine ph	TYPH_HUMAN|TYMP|ECGF1
0032570 // re	0005743 // m	0000166 // nu	Thymidylate s	TYSY_HUMAN|TYMS|TS|OK/SW-cl.29
		0005515 // pr	TYMS opposit	TYMOS_HUMAN|TYMSOS|C18orf56
0018108 // pe	0005887 // in	0001618 // vir	Tyrosine-prote	TYRO3_HUMAN|TYRO3|BYK|DTK|RSE|SKY|TIF
0032438 // m	0016021 // in	0016716 // ox	5,6-dihydroxy	TYRP1_HUMAN|TYRP1|CAS2|TYRP|TYRRP
0006508 // pr	0005777 // pe	0002020 // pr	Peroxisomal le	TYSD1_HUMAN|TYSND1
0008033 // tR		0010181 // FM	S-adenosyl-L-m	TYW1B_HUMAN|TYW1B|RSAFD2
0031591 // wy	0005737 // cy	0000049 // tR	tRNA wybutos	TYW5_HUMAN|TYW5|C2orf60
0031124 // m	0005654 // nu	0003723 // RN	Splicing factor	U2AF1_HUMAN|U2AF1|U2AF35|U2AFBP|FP793

0000398 // m	0005654 // nu	0003723 // RN	U2 snRNP-ass	SR140_HUMAN|U2SURP|KIAA0332|SR140	
0006048 // UI	0005829 // cy	0003977 // UI	UDP-N-acetyll	UAP1_HUMAN|UAP1|SPAG2	
0006048 // UI	0005829 // cy	0003977 // UI	UDP-N-acetyll	UAP1L_HUMAN|UAP1L1	
1990592 // pr	0005737 // cy	0005515 // pr	Ubiquitin-like	UBA5_HUMAN|UBA5|UBE1DC1	
0038128 // EF	0005829 // cy	0005515 // pr	Ubiquitin-60S	RL40_HUMAN|UBA52|UBCEP2	
0006464 // ce	0005829 // cy	0019782 // IS	Ubiquitin-like	UBA7_HUMAN|UBA7|UBE1L|UBE2	
0006508 // pr	0016021 // in	0004252 // se	Ubiquitin-asso	UBAC2_HUMAN|UBAC2|PHGDHL1|PSEC0110	
0016197 // en	0010008 // en	0005515 // pr	Ubiquitin-asso	UBAP1_HUMAN|UBAP1|NAG20	
0043162 // ub	0000813 // ES	0043130 // ub	Ubiquitin-asso	UBA1L_HUMAN|UBAP1L	
0010628 // po	0005634 // nu	0003723 // RN	Ubiquitin-asso	UBAP2_HUMAN|UBAP2|KIAA1491	
0009968 // ne	0005634 // nu	0005515 // pr	Ubiquitin-asso	UBS3B_HUMAN|UBASH3B|KIAA1959|STS1	
0048812 // ne	0005829 // cy	0005515 // pr	Polyubiquitin-	UBB_HUMAN|UBB	
0070979 // pr	0005829 // cy	0004842 // ub	Ubiquitin-con	UBE2C_HUMAN|UBE2C|UBCH10	
0002223 // sti	0005829 // cy	0005515 // pr	Ubiquitin-con	UB2D1_HUMAN|UBE2D1|SFT|UBC5A|UBCH5|UBCH5A	
0032020 // IS		0005515 // pr	Ubiquitin-con	UB2E2_HUMAN|UBE2E2|UBCH8	
0016567 // pr	0005829 // cy	0061631 // ub	Ubiquitin-con	UBE2H_HUMAN|UBE2H	
0016925 // pr	0000795 // sy	0005515 // pr	SUMO-conjug	UBC9_HUMAN|UBE2I|UBC9|UBCE9	
0042534 // re	0016021 // in	0031625 // ub	Ubiquitin-con	UB2J1_HUMAN|UBE2J1|NCUBE1|CGI-76|HSPC153|HSP	
0000209 // pr	0016021 // in	0005524 // AT	Ubiquitin-con	UB2J2_HUMAN|UBE2J2|NCUBE2	
0019941 // m	0005829 // cy	0005515 // pr	Ubiquitin/ISG	UB2L6_HUMAN|UBE2L6|UBCH8	
0002223 // sti	0005737 // cy	0003723 // RN	Ubiquitin-con	UBE2N_HUMAN|UBE2N|BLU	
0016567 // pr	0005829 // cy	0004842 // ub	Ubiquitin-con	UB2R2_HUMAN|UBE2R2|CDC34B|UBC3B	
0035519 // pr	0005654 // nu	0031625 // ub	Ubiquitin-con	UBE2T_HUMAN|UBE2T|HSPC150|PIG50	
0006282 // re	0005829 // cy	0005515 // pr	Ubiquitin-con	UB2V1_HUMAN|UBE2V1|CROC1|UBE2V|UEV1|P/OKcl.1	
0016567 // pr	0005737 // cy	0061630 // ub	Ubiquitin-prot	UBE3B_HUMAN|UBE3B	
0000209 // pr	0005737 // cy	0030332 // cy	E3 ubiquitin-p	UBE3D_HUMAN|UBE3D|C6orf157|H10BH|UBE2CBP	
0006744 // ub	0005783 // en	0016209 // an	UbiA prenyltra	UBIA1_HUMAN|UBIAD1|TERE1	
0071816 // ta	0071818 // BA	0051087 // ch	Ubiquitin-like	UBL4A_HUMAN|UBL4A|DXS254E|GDX|UBL4	
1903955 // po	0005737 // cy	0005515 // pr	Ubiquitin-like	UBL4B_HUMAN|UBL4B	
		0005515 // pr	Ubiquitin-like	UBL7_HUMAN|UBL7|BMSCUBP|SB132	
0006470 // pr	0005730 // nu	0004722 // pr	Ubiquitin-like	UBCP1_HUMAN|UBLCP1	
0033522 // hi	0000151 // ub	0004842 // ub	E3 ubiquitin-p	UBR2_HUMAN|UBR2|C6orf133|KIAA0349	
0035413 // po	0048471 // pe	0005515 // pr	E3 ubiquitin-p	UBR5_HUMAN|UBR5|EDD|EDD1|HYD|KIAA0896	

0008150 // bi		0008270 // zi	Putative E3 ub	UBR7_HUMAN|UBR7|C14orf130
	0005737 // cy	0043130 // ub	Ubiquitin dom	UBTD2_HUMAN|UBTD2|DCUBP|SB72
0019896 // ax	0005737 // cy	0005515 // pr	Ubiquitin carb	UCHL1_HUMAN|UCHL1
0016567 // pr	0005829 // cy	0004843 // th	Ubiquitin carb	UCHL3_HUMAN|UCHL3
0044211 // CT	0005829 // cy	0004849 // ur	Uridine-cytidi	UCKL1_HUMAN|UCKL1|URKL1|F538
0007565 // fe	0043196 // va	0005184 // ne	Urocortin	UCN1_HUMAN|UCN
0006950 // re	0005622 // in	0051431 // co	Urocortin-2	UCN2_HUMAN|UCN2|SRP|URP
0032870 // ce	0005743 // m	0036041 // lo	Mitochondria	UCP1_HUMAN|UCP1|SLC25A7|UCP
1990542 // m	0005743 // m	0005515 // pr	Mitochondria	UCP2_HUMAN|UCP2|SLC25A8
0006508 // pr	0070062 // ex	0071567 // UI	Inactive Ufm1	UFSP1_HUMAN|UFSP1
0033146 // re	0005634 // nu	0005515 // pr	Ufm1-specific	UFSP2_HUMAN|UFSP2|C4orf20
0006688 // gl	0016021 // in	0102769 /// 0	Ceramide gluc	CEGT_HUMAN|UGCG
0006011 // UI	0005634 // nu	0003979 // UI	UDP-glucose 6	UGDH_HUMAN|UGDH
0052695 // ce	0005789 // er	0015020 // gl	UDP-glucuron	UD11_HUMAN|UGT1A1|GNT1|UGT1
0052695 // ce	0016021 // in	0015020 // gl	UDP-glucuron	UD110_HUMAN|UGT1A10|GNT1|UGT1
0052695 // ce	0016021 // in	0015020 // gl	UDP-glucuron	UD14_HUMAN|UGT1A4|GNT1|UGT1
0052695 // ce	0005789 // er	0015020 // gl	UDP-glucuron	UD16_HUMAN|UGT1A6|GNT1|UGT1
0051552 // fla	0016021 // in	0015020 // gl	UDP-glucuron	UD17_HUMAN|UGT1A7|GNT1|UGT1
0052695 // ce	0043231 // in	0015020 // gl	UDP-glucuron	UD2B7_HUMAN|UGT2B7|UGTB2B9
0071412 // ce	0043231 // in	0008194 // UI	UDP-glucuron	UD3A2_HUMAN|UGT3A2|PSEC0073|UNQ842/PRO1780
0007417 // ce	0043231 // in	0047263 // N-	2-hydroxyacyl	CGT_HUMAN|UGT8|CGT|UGT4
0010216 // m	0000792 // he	0000987 // co	E3 ubiquitin-p	UHRF1_HUMAN|UHRF1|ICBP90|NP95|RNF106
0070537 // hi	0005634 // nu	0042393 // hi	BRCA1-A com	UIMC1_HUMAN|UIMC1|RAP80|RXRIP110
0042267 // na	0005783 // er	0046703 // na	NKG2D ligand	N2DL1_HUMAN|ULBP1|N2DL1|RAET1I
0042267 // na	0005615 // ex	0046703 // na	NKG2D ligand	N2DL2_HUMAN|ULBP2|N2DL2|RAET1H|UNQ463/PRO7
0042267 // na	0046658 // an	0046703 // na	NKG2D ligand	N2DL3_HUMAN|ULBP3|N2DL3|RAET1N
0042594 // re	0030659 // cy	0005515 // pr	Serine/threon	ULK2_HUMAN|ULK2|KIAA0623
0043408 // re		0004674 // pr	Serine/threon	ULK4_HUMAN|ULK4
0010468 // re	0016021 // in	0030414 // pe	Uromodulin-li	UROL1_HUMAN|UMODL1
0035690 // ce	0005737 // cy	0004590 // or	Uridine 5'-mo	UMPS_HUMAN|UMPS|OK/SW-cl.21
0097151 // po	0005829 // cy	0030742 // G	Protein unc-1	UN13B_HUMAN|UNC13B|UNC13
0006909 // ph	0016020 // m	0017137 // Ra	Protein unc-1	UN13D_HUMAN|UNC13D
0007165 // sig	0016021 // in	0005515 // pr	Netrin recept	UNC5B_HUMAN|UNC5B|P53RDL1|UNC5H2|UNQ1883/
0038007 // ne	0016021 // in	0005042 // ne	Netrin recept	UNC5C_HUMAN|UNC5C|UNC5H3
0043123 // po	0016021 // in	0008233 // pe	UNC5C-like pr	UN5CL_HUMAN|UNC5CL|ZUD
0048149 // be	0016021 // in		Protein unc-7	UNC79_HUMAN|UNC79|KIAA1409
0034220 // io	0016021 // in		Protein unc-8	UNC80_HUMAN|UNC80|C2orf21|KIAA1843
0006886 // in	0000139 // Go	0035325 // To	Protein unc-9	UN93B_HUMAN|UNC93B1|UNC93|UNC93B

0000209 // pr	0005829 // cy	0004842 // ub	Putative E3 ub	UNKL_HUMAN|UNKL|C16orf28|ZC3H5L|ZC3HDC5L
0019483 // be	0070062 // ex	0003837 // be	Beta-ureidopr	BUP1_HUMAN|UPB1|BUP1
0030855 // ep	0016324 // ap	0005198 // str	Uroplakin-1b	UPK1B_HUMAN|UPK1B|TSPAN20
0007275 // m	0070062 // ex	0005515 // pr	Uroplakin-2	UPK2_HUMAN|UPK2
0010629 // ne	0016021 // in		Uroplakin-3b	UPK3B_HUMAN|UPK3B
0042149 // ce	0005829 // cy	0004850 // ur	Uridine phosp	UPP1_HUMAN|UPP1|UP
0007565 // fe	0005829 // cy	0005525 // GT	Uracil phosph	UPP_HUMAN|UPRT
0002082 // re	0005758 // m	0005515 // pr	Ubiquinol-cyto	UQCC2_HUMAN|UQCC2|C6orf125|MNF1
1902600 // hy	0005750 // m	0008121 // ub	Cytochrome b	QCR6L_HUMAN|UQCRHL
0030901 // m	0005743 // m	0008121 // ub	Cytochrome b	QCR8_HUMAN|UQCRQ
0006782 // pr	0005829 // cy	0004853 // ur	Uroporphyrin	DCUP_HUMAN|UROD
0006888 // ER	0005789 // en	0005515 // pr	Vesicle transp	USE1_HUMAN|USE1|USE1L|MDS032
0006886 // in	0005794 // Go	0003723 // RN	General vesicu	USO1_HUMAN|USO1|VDP
0009411 // re	0005654 // nu	0036459 // th	Ubiquitin carb	UBP1_HUMAN|USP1
0050920 // re	0005829 // cy	0005515 // pr	Ubiquitin carb	UBP14_HUMAN|USP14|TGT
0035522 // m	0005634 // nu	0004197 // cy	Ubiquitin carb	UBP16_HUMAN|USP16|MSTP039
0016578 // hi	0005829 // cy	0036459 // th	Ubiquitin carb	UBP21_HUMAN|USP21|USP23|PP1490
0006511 // ub	0005634 // nu	0036459 // th	Ubiquitin carb	UBP26_HUMAN|USP26
0070536 // pr	0005829 // cy	1990380 // Ly	Ubiquitin carb	UBP27_HUMAN|USP27X|USP22L|USP27
0016579 // pr	0005654 // nu	0004843 // th	Ubiquitin carb	UBP37_HUMAN|USP37|KIAA1594
0008380 // RN	0005654 // nu	0005515 // pr	U4/U6.U5 tri-s	SNUT2_HUMAN|USP39|CGI-21|HSPC332|PRO2855
0016579 // pr		0036459 // th	Ubiquitin carb	UBP40_HUMAN|USP40
0007283 // sp	0005654 // nu	0005515 // pr	Ubiquitin carb	UBP42_HUMAN|USP42
0019985 // tra	0005654 // nu	0019785 // ISG	Ubiquitin carb	UBP43_HUMAN|USP43
1904667 // ne	0005654 // nu	0036459 // th	Ubiquitin carb	UBP44_HUMAN|USP44
0006974 // ce	0005737 // cy	0005515 // pr	Ubiquitin carb	UBP47_HUMAN|USP47
0010569 // re		0003682 // ch	Ubiquitin carb	UBP51_HUMAN|USP51
0060627 // re	0055037 // re	0005096 // GT	Ubiquitin carb	UBP6_HUMAN|USP6|HRP1|TRE2
0016567 // pr	0005829 // cy	0005515 // pr	Probable ubiq	USP9X_HUMAN|USP9X|DFFRX|FAM|USP9
0030010 // es	0016021 // in	0008146 // su	Uronyl 2-sulfo	UST_HUMAN|UST|DS2ST
0006364 // rR	0005654 // nu	0003723 // RN	Probable U3 s	UTP11_HUMAN|UTP11|UTP11L|CGI-94|HDCMB12P
0006364 // rR	0005654 // nu	0003723 // RN	U3 small nucle	UT14A_HUMAN|UTP14A|SDCCAG16
2000234 // po	0005654 // nu	0003723 // RN	U3 small nucle	UTP15_HUMAN|UTP15
0000447 // en	0005654 // nu	0003723 // RN	Small subunit	UTP20_HUMAN|UTP20|DRIM
0009411 // re	0005654 // nu	0000993 // RN	UV-stimulated	UVSSA_HUMAN|UVSSA|KIAA1530
0043001 // Go	0009986 // ce	0005515 // pr	Vesicle-associ	VAMP5_HUMAN|VAMP5|HSPC191
0006892 // po	0005886 // pl	0000149 // SN	Vesicle-associ	VAMP8_HUMAN|VAMP8
0090103 // co	0005886 // pl	0005515 // pr	Vang-like prot	VANG2_HUMAN|VANGL2|KIAA1215|STB1
0006874 // ce	0005789 // en	0005515 // pr	Vesicle-associ	VAPB_HUMAN|VAPB|UNQ484/PRO983

0006438 // va	0005739 // m	0004832 // va	Valine--tRNA l	SYVM_HUMAN|VARS2|KIAA1885|VARS2L|VARSL
0007050 // ce	0005615 // ex	0005515 // pr	Vasohibin-1	VASH1_HUMAN|VASH1|KIAA1036|VASH
0007264 // sn	0005829 // cy	0005085 // gu	Proto-oncoge	VAV_HUMAN|VAV1|VAV
0030193 // re	0005829 // cy	0005515 // pr	Guanine nucle	VAV2_HUMAN|VAV2
0006974 // ce	0005829 // cy	0005515 // pr	Guanine nucle	VAV3_HUMAN|VAV3
0016055 // W	0005634 // nu	0001162 // RN	Ventral anteri	VAX2_HUMAN|VAX2
0007159 // le	0002102 // pc	0005178 // in	Vascular cell a	VCAM1_HUMAN|VCAM1
0007155 // ce	0005796 // Gc	0005539 // gl	Versican core	CSPG2_HUMAN|VCAN|CSPG2
0006302 // dc	0034774 // se	0016887 // AT	Transitional e	TERA_HUMAN|VCP
0007420 // br	0005634 // nu	0003682 // ch	Variable charg	VCX1_HUMAN|VCX|VCX1|VCX10R|VCXB1
0016236 // m	0042645 // m	0044325 // io	Voltage-deper	VDAC1_HUMAN|VDAC1|VDAC
0007595 // la	0043235 // re	0003700 // tra	Vitamin D3 re	VDR_HUMAN|VDR|NR1I1
0002687 // pc	0005576 // ex	0005161 // pl	Vascular endc	VEGFA_HUMAN|VEGFA|VEGF
0008284 // pc	0005576 // ex	0043185 // va	Vascular endc	VEGFC_HUMAN|VEGFC
0002576 // pl	0005576 // ex	0042803 // pr	Vascular endc	VEGFD_HUMAN|VEGFD|FIGF
	0005886 // pl		Ventricular zo	MELT_HUMAN|VEPH1|KIAA1692|VEPH
0006968 // ce	0005654 // nu	0000977 // RN	Vascular endc	VEZF1_HUMAN|VEZF1|DB1|ZNF161
0042593 // gl	0005788 // er	0008083 // gr	Neurosecreto	VGF_HUMAN|VGF
0006351 // tra	0005634 // nu	0008022 // pr	Transcription	VGLL1_HUMAN|VGLL1|TDU
0007010 // cy	0015629 // ac	0005200 // st	Villin-like prot	VILL_HUMAN|VILL
0060020 // Be	0043005 // ne	0042802 // id	Vimentin	VIME_HUMAN|VIM
0007283 // sp	0005794 // Gc	0005515 // pr	Spermatogene	SPE39_HUMAN|VIPAS39|C14orf133|SPE39|VIPAR
0007586 // di	0005886 // pl	0004999 // va	Vasoactive int	VIPR1_HUMAN|VIPR1
0017187 // pe	0016021 // in	0047057 // vi	Vitamin K epo	VKOR1_HUMAN|VKORC1|VKOR|MSTP134|MSTP576|UI
0034447 // ve	0005886 // pl	0030229 // ve	Very low-dens	VLDLR_HUMAN|VLDLR
0008150 // bi	0070062 // ex	0003674 // m	Vitelline mem	VMO1_HUMAN|VMO1|UNQ6350/PRO21055
0033089 // pc	0031225 // ar	0017159 // pa	Pantetheinase	VNN1_HUMAN|VNN1
0006955 // im	0005783 // er		Pre-B lympho	VPRE3_HUMAN|VPREB3|UNQ355/PRO619
	0070062 // ex		Vacuolar prot	VP13D_HUMAN|VPS13D|KIAA0453
0035542 // re	0005769 // ea	0005515 // pr	Vacuolar prot	VPS18_HUMAN|VPS18|KIAA1475
0016241 // re	0005829 // cy	0005515 // pr	Vacuolar prot	VP26A_HUMAN|VPS26A|VPS26
0030220 // pl	0030123 // AF	0005515 // pr	Vacuolar prot	VP33A_HUMAN|VPS33A
0017185 // pe	0031091 // pl	0005515 // pr	Vacuolar prot	VP33B_HUMAN|VPS33B
0016197 // er	0010008 // er	0005515 // pr	Vacuolar prot	VP37C_HUMAN|VPS37C|PML39
0016197 // er	0010008 // er		Vacuolar prot	VP37D_HUMAN|VPS37D|WBSCR24
1902774 // la	0005765 // lys		Vam6/Vps39-	VPS39_HUMAN|VPS39|KIAA0770|TLP|VAM6
0034058 // er	0005769 // ea	0005515 // pr	Vacuolar prot	VPS41_HUMAN|VPS41
0007596 // bl	0000139 // Gc	0005515 // pr	Vacuolar prot	VPS45_HUMAN|VPS45|VPS45A|VPS45B
0032456 // er	0055037 // re	0000149 // SN	Syndetin	VPS50_HUMAN|VPS50|CCDC132|KIAA1861
0003382 // ep	0016021 // in		V-set and imm	VSIG1_HUMAN|VSIG1|GPA34

	0016021 // in		V-set and imm	VSI10_HUMAN|VSIG10
	0016021 // in		V-set and imm	VS10L_HUMAN|VSIG10L
	0005887 // in		V-set and imm	VSIG2_HUMAN|VSIG2|CTH|CTXL|UNQ2770/PRO7154
	0016021 // in		V-type immur	VISTA_HUMAN|VSIR|C10orf54|SISP1|VISTA|PP2135|UN
0046676 // ne	0005829 // cy	0005515 // pr	Visinin-like pro	VISL1_HUMAN|VSNL1|VISL1
0002376 // im	0016021 // in	0005125 // cy	V-set and trar	VSTM1_HUMAN|VSTM1|UNQ3033/PRO9835
	0016021 // in		V-set and trar	VSTM5_HUMAN|VSTM5|C11orf90
0016197 // er	0070062 // ex	0005515 // pr	Vacuolar prot	VTA1_HUMAN|VTA1|C6orf55|HSPC228|My012
0006891 // in	0031982 // ve	0000149 // SN	Vesicle transp	VTI1B_HUMAN|VTI1B|VTI1|VTI1L|VTI1L1|VTI2
0030198 // ex	0070062 // ex	0042802 // id	von Willebran	VWA1_HUMAN|VWA1
0051260 // pr	0005615 // ex	0042802 // id	von Willebran	VWA2_HUMAN|VWA2|AMACO
	0005576 // ex		von Willebran	VWA3A_HUMAN|VWA3A
	0005737 // cy		von Willebran	VWA3B_HUMAN|VWA3B
0008150 // bi	0005576 // ex	0003674 // m	von Willebran	VWA7_HUMAN|VWA7|C6orf27|G7C|NG37
	0005576 // ex	0016887 // AT	von Willebran	VWA8_HUMAN|VWA8|KIAA0564
0030514 // ne	0030054 // ce	0005515 // pr	von Willebran	VWC2L_HUMAN|VWC2L
0009966 // re	0005576 // ex	0005509 // ca	von Willebran	VWCE_HUMAN|VWCE|URG11
	0005576 // ex		von Willebran	VWDE_HUMAN|VWDE
0002576 // pl	0005576 // ex	0002020 // pr	von Willebran	VWF_HUMAN|VWF|F8VWF
0045765 // re	0005737 // cy	0019901 // pr	Tryptophan--t	SYWC_HUMAN|WARS|IFI53|WRS
0001570 // va	0005739 // m	0004830 // tr	Tryptophan--t	SYWM_HUMAN|WARS2
0008154 // ac	0005829 // cy	0043274 // ph	Wiskott-Aldric	WASP_HUMAN|WAS|IMD2
0007010 // cy	0005737 // cy	0003779 // ac	Wiskott-Aldric	WASF3_HUMAN|WASF3|KIAA0900|SCAR3|WAVE3
0016197 // er	0005829 // cy	0003779 // ac	WAS protein f	WASH2_HUMAN|WASH2P|FAM39B
0016197 // er	0005829 // cy	0003779 // ac	Putative WAS	WASH3_HUMAN|WASH3P|FAM39DP
0015031 // pr	0071203 // W	0008289 // lip	WASH comple	WAC2A_HUMAN|WASHC2A|FAM21A|FAM21B
0042147 // re	0005769 // ea	0005546 // ph	WASH comple	WAC2C_HUMAN|WASHC2C|FAM21C|KIAA0592
0045292 // m	0005634 // nu	0019888 // pr	WW domain-b	WBP11_HUMAN|WBP11|NPWBP|SIPP1|SNP70
0071391 // ce	0000790 // nu	0001105 // RN	WW domain-b	WBP2_HUMAN|WBP2
0051259 // pr		0019900 // kir	WD repeat an	WDCP_HUMAN|WDCP|C2orf44|PP384
0034141 // po	0005829 // cy	0008270 // zir	WD repeat an	WDFY1_HUMAN|WDFY1|KIAA1435|WDF1|ZFYVE17
	0016021 // in	0005515 // pr	WD repeat-co	WDR11_HUMAN|WDR11|BRWD2|KIAA1351|WDR15
0006364 // rR	0070545 // Pe	0005515 // pr	Ribosome bio	WDR12_HUMAN|WDR12

	0005737 // cy		WD repeat-co	WDR17_HUMAN	WDR17
0016579 // pr	0005654 // nu	0005515 // pr	WD repeat-co	WDR20_HUMAN	WDR20
0032008 // po	0005765 // lys	0005515 // pr	GATOR compl	WDR24_HUMAN	WDR24|C16orf21|JFP7
		0005515 // pr	WD repeat-co	WDR25_HUMAN	WDR25|C14orf67
0006364 // rR	0005654 // nu	0003723 // RN	WD repeat-co	WDR3_HUMAN	WDR3
0035735 // in	0005868 // cy	0045504 // dy	WD repeat-co	WDR34_HUMAN	WDR34
0032496 // re	0005929 // cil	0035091 // ph	WD repeat-co	WDR35_HUMAN	WDR35|IFT121|KIAA1336
0006364 // rR	0005654 // nu	0003723 // RN	WD repeat-co	WDR36_HUMAN	WDR36
0000460 // m	0070545 // Pe		WD repeat-co	WDR37_HUMAN	WDR37|KIAA0982
0106004 /// 0	0005829 // cy	0005515 // pr	tRNA (guanine	WDR4_HUMAN	WDR4
0043547 // po	0005737 // cy	0005515 // pr	WD repeat-co	WDR41_HUMAN	WDR41|MSTP048
0006364 // rR	0005654 // nu	0003723 // RN	WD repeat-co	WDR46_HUMAN	WDR46|BING4|C6orf11|FP221
0007275 // m	0005737 // cy	0005515 // pr	WD repeat-co	WDR47_HUMAN	WDR47|KIAA0893
0006364 // rR	0005737 // cy	0003674 // m	WD repeat-co	WDR55_HUMAN	WDR55
0034198 // ce	0005765 // lys	0005515 // pr	GATOR compl	WDR59_HUMAN	WDR59|KIAA1923|FP977
0060271 // cil	0005868 // cy	0045504 // dy	WD repeat-co	WDR60_HUMAN	WDR60
0007018 // m	0030286 // dy	0045504 // dy	WD repeat-co	WDR63_HUMAN	WDR63
0003341 // cil	0005737 // cy		Cilia- and flag	CF251_HUMAN	WDR66|CFAP251
0002244 // he	0008021 // sy		WD repeat-co	WDR7_HUMAN	WDR7|KIAA0541|TRAG
0070166 // er	0005737 // cy		WD repeat-co	WDR72_HUMAN	WDR72
0006974 // ce	0005634 // nu	0003677 // DN	WD repeat-co	WDR76_HUMAN	WDR76
0035246 // pe	0005654 // nu	0005515 // pr	Methylosome	MEP50_HUMAN	WDR77|MEP50|WD45|HKMT1069|Nb
0003341 // cil	0005858 // ax	0045504 // dy	WD repeat-co	WDR78_HUMAN	WDR78
0006511 // ub	0005789 // er	0035014 // ph	WD repeat-co	WDR81_HUMAN	WDR81
0000398 // m	0010008 // en	0005515 // pr	WD repeat do	WDR83_HUMAN	WDR83|MORG1
0022900 // ele		0016651 // ox	WD repeat-co	WDR93_HUMAN	WDR93
0043086 // ne	0005654 // nu	0005515 // pr	WD and tetra	WDTC1_HUMAN	WDTC1|KIAA1037
0000086 // G2	0005654 // nu	0000287 // m	Wee1-like pro	WEE1_HUMAN	WEE1
0010951 // ne	0070062 // ex	0004867 // se	WAP four-disu	WFDC2_HUMAN	WFDC2|HE4|WAP5
0010951 // ne	0005615 // ex	0008191 // m	WAP, Kazal, in	WFKN2_HUMAN	WFIKKN2|GASP1|WFIKKNRP|UNQ923
0006888 // ER	0033116 // en	0071933 // Ar	Putative WAS	WHAL1_HUMAN	WHAMMP3|WHAMML1|WHDC1L1
0008154 // ac	0005829 // cy	0005515 // pr	WAS/WASL-in	WIPF1_HUMAN	WIPF1|WASPIP|WIP
0007283 // sp	0005829 // cy	0017124 // SH	WAS/WASL-in	WIPF3_HUMAN	WIPF3|CR16
0016236 // m	0005829 // cy	0032266 // ph	WD repeat do	WIPI1_HUMAN	WIPI1|WIPI49
0007165 // sig	0005578 // pr	0008083 // gr	WNT1-inducib	WISP3_HUMAN	WISP3|CCN6|UNQ462/PRO790/PRO956
0003084 // po	0016020 // m	0019869 // ch	Serine/threon	WNK1_HUMAN	WNK1|HSN2|KDP|KIAA0344|PRKWNK1
2000021 // re	0005737 // cy	0004674 // pr	Serine/threon	WNK2_HUMAN	WNK2|KIAA1760|PRKWNK2|SDCCAG43
1903078 // po	0005737 // cy	0004672 // pr	Serine/threon	WNK3_HUMAN	WNK3|KIAA1566|PRKWNK3
0006811 // io	0016020 // m	0019869 // ch	Serine/threon	WNK4_HUMAN	WNK4|PRKWNK4
0014033 // ne	0005615 // ex	0048018 // re	Protein Wnt-1	WN10A_HUMAN	WNT10A
0060070 // ca	0005615 // ex	0048018 // re	Protein Wnt-1	WN10B_HUMAN	WNT10B|WNT12

0071300 // ce	0005615 // ex	0030295 // pr	Protein Wnt-1	WNT11_HUMAN|WNT11
0016055 // W	0005615 // ex	0005109 // fri	Protein Wnt-1	WNT16_HUMAN|WNT16
0009267 // ce	0005615 // ex	0005109 // fri	Protein Wnt-2	WNT2B_HUMAN|WNT2B|WNT13
0060070 // ca	0005615 // ex	0005109 // fri	Proto-oncoge	WNT3_HUMAN|WNT3|INT4
0072659 // pr	0005737 // cy	0048018 // re	Protein Wnt-4	WNT4_HUMAN|WNT4|UNQ426/PRO864
0090090 // ne	0005615 // ex	0005102 // re	Protein Wnt-5	WNT5B_HUMAN|WNT5B
0010628 // po	0005615 // ex	0005109 // fri	Protein Wnt-6	WNT6_HUMAN|WNT6
0099068 // po	0098793 // pr	0048018 // re	Protein Wnt-7	WNT7A_HUMAN|WNT7A
0045597 // po	0005615 // ex	0048018 // re	Protein Wnt-9	WNT9A_HUMAN|WNT9A|WNT14
1904867 // pr	0015030 // Ca	0005515 // pr	Telomerase Ca	WAP53_HUMAN|WRAP53|TCAB1|WDR79
0030030 // ce	0005813 // ce	0005515 // pr	WD repeat-co	WRP73_HUMAN|WRAP73|WDR8
0001302 // re	0005654 // nu	0004527 // ex	Werner syndr	WRN_HUMAN|WRN|RECQ3|RECQL2
0000209 // pr	0005829 // cy	0004842 // ub	WD repeat an	WSB1_HUMAN|WSB1|SWIP1
	0016021 // in	0008146 // su	WSC domain-	WSCD1_HUMAN|WSCD1|KIAA0523
	0016021 // in		WSC domain-	WSCD2_HUMAN|WSCD2|KIAA0789
0060421 // po	0005634 // nu	0043565 // se	Wilms tumor	WT1_HUMAN|WT1
0008150 // bi	0005575 // ce	0005515 // pr	Putative Wilm	WIT1_HUMAN|WT1-AS|WIT1
0001666 // re	0005634 // nu	0005515 // pr	Wilms tumor	WTIP_HUMAN|WTIP
0046621 // ne	0005829 // cy	0032947 // pr	Protein WWC	WWC2_HUMAN|WWC2|BOMB
0046621 // ne	0005829 // cy	0032947 // pr	Protein WWC	WWC3_HUMAN|WWC3|KIAA1280
0016567 // pr	0005829 // cy	0004842 // ub	NEDD4-like E3	WWP1_HUMAN|WWP1
0008284 // po	0005829 // cy	0005515 // pr	WW domain-	WWTR1_HUMAN|WWTR1|TAZ
0000398 // m	0005654 // nu	0005515 // pr	Pre-mRNA-spl	SYF1_HUMAN|XAB2|HCNP|KIAA1177|SYF1|PP3898
0031333 // ne	0005739 // m	0008270 // zir	XIAP-associate	XAF1_HUMAN|XAF1|BIRC4BP|XIAPAF1
0006955 // im	0005737 // cy	0003700 // tra	X-box-binding	XBP1_HUMAN|XBP1|TREB5|XBP2
0008150 // bi	0005887 // in	0003674 // m	Glycoprotein X	XG_HUMAN|XG|PBDX
0008361 // re	0016021 // in	0005515 // pr	Membrane tra	XK_HUMAN|XK|XKR1|XRG1
	0016021 // in		XK-related pro	XKR6_HUMAN|XKR6|C8orf21|C8orf5|C8orf7|XRG6
0043652 // en	0016021 // in		XK-related pro	XKR8_HUMAN|XKR8|XRG8
0006611 // pr	0005730 // nu	0008565 // pr	Exportin-6	XPO6_HUMAN|XPO6|KIAA0370|RANBP20
0030643 // ce	0031226 // in	0004888 // tra	Xenotropic an	XPR1_HUMAN|XPR1|SYG1|XR
0000722 // te	0033063 // Ra	0003690 // do	DNA repair pr	XRCC3_HUMAN|XRCC3
0032204 // re	0005654 // nu	0003723 // RN	X-ray repair cr	XRCC5_HUMAN|XRCC5|G22P2
0000956 // nu	0005829 // cy	0003723 // RN	5'-3' exoribon	XRN1_HUMAN|XRN1|SEP1
0010165 // re	0016604 // nu	0003674 // m	X-ray radiatio	XRRA1_HUMAN|XRRA1

0016266 // O-	0030176 // in	0000287 // m	Xyloside xylos	XXLT1_HUMAN	XXYLT1	C3orf21	PSEC0251
0006091 // ge	0070062 // ex	0004856 // xy	Xylulose kinas	XYLB_HUMAN	XYLB
0030206 // ch	0016021 // in	0008375 // ac	Xylosyltransfe	XYLT2_HUMAN	XYLT2	XT2	UNQ3058/PRO9878
0070184 // m	0005739 // m	0004831 // ty	Tyrosine--tRN	SYYM_HUMAN	YARS2	CGI-04
0009386 // tr	0005634 // nu	0003677 // DI	Y-box-binding	YBOX2_HUMAN	YBX2	CSDA3	MSY2
0001701 // in	0005634 // nu	0000977 // RI	Y-box-binding	YBOX3_HUMAN	YBX3	CSDA	DBPA
0005975 // ca		0016787 // hy	Carbohydrate	YDJC_HUMAN	YDJC
0030154 // ce	0005794 // G	0005515 // pr	Protein YIPF3	YIPF3_HUMAN	YIPF3	C6orf109	KLIP1
	0016021 // in		Protein YIPF7	YIPF7_HUMAN	YIPF7	FINGER9	YIP1B
0030968 // er	0005829 // cy	1990380 // Ly	Ubiquitin thio	OTU1_HUMAN	YOD1	DUBA8	HIN7	OTUD2	PRO0907
	0005634 // nu	0046872 // m	Protein yippe	YPEL1_HUMAN	YPEL1	FKSG3
	0005730 // nu	0046872 // m	Protein yippe	YPEL2_HUMAN	YPEL2	FKSG4
2000774 // pc	0005730 // nu	0046872 // m	Protein yippe	YPEL3_HUMAN	YPEL3	FKSG5
0043312 // ne	0005576 // ex	0005515 // pr	Protein yippe	YPEL5_HUMAN	YPEL5	CGI-127
0002949 // tR	0016020 // m	0003725 // dc	YrdC domain-	YRDC_HUMAN	YRDC	DRIP3	IRIP
0045948 // pc	0005737 // cy	0003723 // RI	YTH domain-c	YTHD1_HUMAN	YTHDF1	C20orf21
0061024 // m	0005829 // cy	0005515 // pr	14-3-3 proteir	1433B_HUMAN	YWHAB
0051683 // es	0005737 // cy	0005515 // pr	14-3-3 proteir	1433Z_HUMAN	YWHAZ
0098655 // ca	0016021 // in	0008270 // zi	Zinc-activated	ZACN_HUMAN	ZACN	L2	LGICZ	LGICZ1	ZAC
	0005622 // in	0008270 // zi	Zinc finger B-b	ZBBX_HUMAN	ZBBX
0016925 // pr	0031965 // nu	0001077 // tr	Zinc finger BE	ZBED1_HUMAN	ZBED1	ALTE	DREF	KIAA0785	TRAMP
		0003677 // DI	Zinc finger BE	ZBED2_HUMAN	ZBED2
0006357 // re	0005654 // nu	0003677 // DI	Zinc finger BE	ZBED5_HUMAN	ZBED5	Buster1
0006351 // tr	0005634 // nu	0003700 // tr	Zinc finger BE	ZBED6_HUMAN	ZBED6
0006351 // tr	0005654 // nu	0005515 // pr	Zinc finger an	ZBT10_HUMAN	ZBTB10	RINZF	RINZFC
0006351 // tr	0005634 // nu	0003677 // DI	Zinc finger an	ZBT12_HUMAN	ZBTB12	C6orf46	G10	NG35
0000122 // ne	0000228 // nu	0003700 // tr	Zinc finger an	ZBT18_HUMAN	ZBTB18	RP58	TAZ1	ZNF238
0000122 // ne	0005634 // nu	0005515 // pr	Zinc finger an	ZBTB2_HUMAN	ZBTB2	KIAA1483	ZNF437
0000122 // ne	0005634 // nu	0001078 // tr	Zinc finger an	ZBT20_HUMAN	ZBTB20	DPZF	ZNF288
0006351 // tr	0005829 // cy	0005515 // pr	Zinc finger an	ZBT21_HUMAN	ZBTB21	KIAA1227	ZNF295
0002244 // he	0005634 // nu	0005515 // pr	Zinc finger an	ZBT24_HUMAN	ZBTB24	KIAA0441	ZNF450

0006351 // tra	0005634 // nu	0005515 // pr	Zinc finger and	ZBT26_HUMAN	ZBT26_HUMAN|ZBTB26|KIAA1572|ZNF481
0042769 // DN	0005654 // nu	0008270 // zin	Zinc finger and	ZBT32_HUMAN|ZBTB32|FAZF|TZFP|ZNF538
0006351 // tra	0005634 // nu	0003677 // DN	Zinc finger and	ZBT37_HUMAN|ZBTB37
0006351 // tra	0005634 // nu	0003677 // DN	Zinc finger and	ZBT41_HUMAN|ZBTB41|FRBZ1
0006351 // tra	0005634 // nu	0005515 // pr	Zinc finger and	ZBT43_HUMAN|ZBTB43|KIAA0414|ZBTB22B|ZNF297B
0030853 // ne	0005634 // nu	0046872 // m	Zinc finger and	ZBT46_HUMAN|ZBTB46|BTBD4|ZNF340
0045893 // po	0005829 // cy	0005515 // pr	Telomere zinc	TZAP_HUMAN|ZBTB48|HKR3|TZAP|ZNF855
0007275 // m	0005634 // nu	0001077 // tra	Zinc finger and	ZBT7B_HUMAN|ZBTB7B|ZBTB15|ZFP67|ZNF857B
0006351 // tra	0005634 // nu	0003677 // DN	Zinc finger and	ZBT8B_HUMAN|ZBTB8B
		0005515 // pr	Zinc finger C2	ZC21C_HUMAN|ZC2HC1C|C14orf140|FAM164C
0031124 // m	0005654 // nu	0003723 // RN	Zinc finger CC	ZC11A_HUMAN|ZC3H11A|KIAA0663|ZC3HDC11A
0010628 // po	0005634 // nu	0005515 // pr	Endoribonucle	ZC12A_HUMAN|ZC3H12A|MCPIP|MCPIP1
0090305 // nu		0004519 // en	Probable ribo	ZC12B_HUMAN|ZC3H12B|CXorf32|MCPIP2
0043488 // re	0005730 // nu	0003723 // RN	Zinc finger CC	ZC3HE_HUMAN|ZC3H14
0019221 // cy	0005730 // nu	0003723 // RN	Zinc finger CC	ZC3HF_HUMAN|ZC3H15|DFRP1|LEREPO4|HSPC303|HT(
0008150 // bi	0005575 // ce	0003674 // m	Zinc finger CC	ZC3H6_HUMAN|ZC3H6|KIAA2035|ZC3HDC6
0016032 // vi	0005634 // nu	0003723 // RN	Zinc finger CC	Z3H7B_HUMAN|ZC3H7B|KIAA1031
	0005829 // cy	0046872 // m	Zinc finger CC	ZCCHL_HUMAN|ZC3HAV1L|C7orf39
0007528 // ne	0005737 // cy	0005515 // pr	Zinc finger C4	ZC4H2_HUMAN|ZC4H2|HCA127|KIAA1166
0030509 // BM	0016607 // nu	0008270 // zin	Zinc finger CC	ZCC18_HUMAN|ZCCHC18
	0005737 // cy	0008270 // zin	Zinc finger CC	ZCHC2_HUMAN|ZCCHC2|C18orf49|KIAA1744
		0003723 // RN	Zinc finger CC	ZCH24_HUMAN|ZCCHC24|C10orf56
	0005730 // nu	0003723 // RN	Zinc finger CC	ZCHC7_HUMAN|ZCCHC7|HSPC086
		0008270 // zin	Zinc finger CW	ZCPW1_HUMAN|ZCWPW1
		0008270 // zin	Zinc finger CW	A0A1B0GU75_HUMAN|ZCWPW2
		0003676 // nu	DBF4-type zin	ZDBF2_HUMAN|ZDBF2|KIAA1571
0018345 // pr	0005783 // en	0016409 // pa	Probable palm	ZDHC1_HUMAN|ZDHHC1|C16orf1|ZNF377
	0005783 // en	0019706 // pr	Probable palm	ZDH11_HUMAN|ZDHHC11|ZNF399
	0016021 // int	0019706 // pr	Probable palm	ZH11B_HUMAN|ZDHHC11B
0018345 // pr	0005783 // en	0016409 // pa	Probable palm	ZDH12_HUMAN|ZDHHC12|ZNF400|PSEC0008
1903830 // m	0000139 // Go	0004871 // sig	Palmitoyltrans	ZDH13_HUMAN|ZDHHC13|HIP14L|HIP3RP
0018345 // pr	0005783 // en	0016409 // pa	Probable palm	ZDH14_HUMAN|ZDHHC14
	0005783 // en	0019706 // pr	Probable palm	ZDH19_HUMAN|ZDHHC19
0018345 // pr	0005783 // en	0016409 // pa	Palmitoyltrans	ZDHC2_HUMAN|ZDHHC2|REAM|REC|ZNF372
0018345 // pr	0016021 // int	0016409 // pa	Probable palm	ZDH20_HUMAN|ZDHHC20
	0005783 // en	0005515 // pr	Palmitoyltrans	ZDH22_HUMAN|ZDHHC22|C14orf59
0072659 // pr	0016021 // int	0019706 // pr	Palmitoyltrans	ZDH23_HUMAN|ZDHHC23
	0016021 // int	0005515 // pr	Probable palm	ZDH24_HUMAN|ZDHHC24|UNQ2528/PRO6027
	0016021 // int	0019706 // pr	Probable palm	ZDHC4_HUMAN|ZDHHC4|ZNF374|DC1|UNQ5787/PRO1
0018345 // pr	0016021 // int	0016409 // pa	Palmitoyltrans	ZDHC5_HUMAN|ZDHHC5|KIAA1748|ZNF375
0018345 // pr	0005794 // Go	0016409 // pa	Palmitoyltrans	ZDHC7_HUMAN|ZDHHC7
0018345 // pr	0005739 // m	0016409 // pa	Probable palm	ZDHC8_HUMAN|ZDHHC8|KIAA1292|ZDHHCL1|ZNF378
0018230 // pe	0005783 // en	0005515 // pr	Palmitoyltrans	ZDHC9_HUMAN|ZDHHC9|CXorf11|ZDHHC10|ZNF379|ZI
0051150 // re	0005634 // nu	0003714 // tra	Zinc finger E-b	ZEB1_HUMAN|ZEB1|AREB6|TCF8
0006366 // tra	0005634 // nu	0043565 // se	Zinc finger E-b	ZEB2_HUMAN|ZEB2|KIAA0569|SIP1|ZFHX1B|ZFX1B|HR

<table>
<tr><td></td><td></td><td></td><td></td><td></td></tr>
<tr><td>0016567 // pr</td><td>0031462 // Cu</td><td>0004842 // ub</td><td>Protein zer-1</td><td>ZER1_HUMAN|ZER1|C9orf60|ZYG|ZYG11BL</td></tr>
<tr><td></td><td></td><td>0008270 // zir</td><td>AN1-type zinc</td><td>ZFAN1_HUMAN|ZFAND1</td></tr>
<tr><td></td><td></td><td>0008270 // zir</td><td>AN1-type zinc</td><td>ZFAN4_HUMAN|ZFAND4|ANUBL1</td></tr>
<tr><td></td><td></td><td></td><td></td><td></td></tr>
<tr><td>0002244 // he</td><td>0005829 // cy</td><td>0003677 // DI</td><td>Zinc finger pro</td><td>ZFAT_HUMAN|ZFAT|KIAA1485|ZFAT1|ZNF406</td></tr>
<tr><td>0030534 // ac</td><td>0005634 // nu</td><td>0008270 // zir</td><td>Zinc finger ho</td><td>ZFHX2_HUMAN|ZFHX2|KIAA1056|KIAA1762|ZNF409</td></tr>
<tr><td>0006351 // tra</td><td>0005634 // nu</td><td>0008270 // zir</td><td>Zinc finger ho</td><td>ZFHX4_HUMAN|ZFHX4</td></tr>
<tr><td></td><td></td><td></td><td></td><td></td></tr>
<tr><td>0006351 // tra</td><td>0005634 // nu</td><td>0003677 // DI</td><td>Zinc finger pro</td><td>ZFP1_HUMAN|ZFP1|ZNF475</td></tr>
<tr><td>0006351 // tra</td><td>0005634 // nu</td><td>0005515 // pr</td><td>Zinc finger pro</td><td>ZFP2_HUMAN|ZFP2|ZNF751</td></tr>
<tr><td>0006351 // tra</td><td>0005634 // nu</td><td>0003677 // DI</td><td>Zinc finger pro</td><td>ZFP30_HUMAN|ZFP30|KIAA0961|ZNF745</td></tr>
<tr><td>0043488 // re</td><td>0005737 // cy</td><td>0005515 // pr</td><td>mRNA decay a</td><td>TTP_HUMAN|ZFP36|G0S24|NUP475|RNF162A|TIS11A|</td></tr>
<tr><td>0061158 // 3'</td><td>0005829 // cy</td><td>0003723 // RN</td><td>mRNA decay a</td><td>TISB_HUMAN|ZFP36L1|BERG36|BRF1|ERF1|RNF162B|T</td></tr>
<tr><td>0006351 // tra</td><td>0005634 // nu</td><td>0003677 // DI</td><td>Zinc finger pro</td><td>ZF69B_HUMAN|ZFP69B|ZNF643</td></tr>
<tr><td>0006351 // tra</td><td>0005634 // nu</td><td>0003677 // DI</td><td>Zinc finger pro</td><td>ZFP82_HUMAN|ZFP82|KIAA1948|ZNF545</td></tr>
<tr><td>0045599 // ne</td><td>0005654 // nu</td><td>0003714 // tra</td><td>Zinc finger pro</td><td>FOG2_HUMAN|ZFPM2|FOG2|ZNF89B</td></tr>
<tr><td></td><td></td><td></td><td></td><td></td></tr>
<tr><td>0010923 // ne</td><td>0000407 // pr</td><td>0008270 // zir</td><td>Zinc finger FY</td><td>ZFYV1_HUMAN|ZFYVE1|DFCP1|KIAA1589|TAFF1|ZNFN</td></tr>
<tr><td>0000910 // cy</td><td>0030496 // m</td><td>0005515 // pr</td><td>Zinc finger FY</td><td>ZFY26_HUMAN|ZFYVE26|KIAA0321</td></tr>
<tr><td>0042059 // ne</td><td>0005829 // cy</td><td>0005515 // pr</td><td>Lateral signali</td><td>LST2_HUMAN|ZFYVE28|KIAA1643|LST2</td></tr>
<tr><td>0006351 // tra</td><td>0005634 // nu</td><td>0005515 // pr</td><td>Zinc finger CC</td><td>ZGPAT_HUMAN|ZGPAT|GPATC6|GPATCH6|KIAA1847|Z</td></tr>
<tr><td>0045892 // ne</td><td>0005634 // nu</td><td>0005515 // pr</td><td>Zinc fingers ar</td><td>ZHX1_HUMAN|ZHX1</td></tr>
<tr><td>0045892 // ne</td><td>0005634 // nu</td><td>0005515 // pr</td><td>Zinc fingers ar</td><td>ZHX2_HUMAN|ZHX2|AFR1|KIAA0854|RAF</td></tr>
<tr><td>0045892 // ne</td><td>0005634 // nu</td><td>0005515 // pr</td><td>Zinc fingers ar</td><td>ZHX3_HUMAN|ZHX3|KIAA0395|TIX1</td></tr>
<tr><td>0042472 // in</td><td>0005634 // nu</td><td>0003700 // tra</td><td>Zinc finger pro</td><td>ZIC1_HUMAN|ZIC1|ZIC|ZNF201</td></tr>
<tr><td>0007420 // br</td><td>0005634 // nu</td><td>0031490 // ch</td><td>Zinc finger pro</td><td>ZIC2_HUMAN|ZIC2</td></tr>
<tr><td></td><td>0005634 // nu</td><td>0003677 // DI</td><td>Zinc finger pro</td><td>ZIC4_HUMAN|ZIC4</td></tr>
<tr><td>0006351 // tra</td><td>0005634 // nu</td><td>0003677 // DI</td><td>Zinc finger pro</td><td>ZIK1_HUMAN|ZIK1|ZNF762</td></tr>
<tr><td>0006351 // tra</td><td>0005634 // nu</td><td>0003677 // DI</td><td>Zinc finger pro</td><td>ZKSC1_HUMAN|ZKSCAN1|KOX18|ZNF139|ZNF36</td></tr>
<tr><td>0006351 // tra</td><td>0005634 // nu</td><td>0000981 // RN</td><td>Zinc finger pro</td><td>ZKSC2_HUMAN|ZKSCAN2|ZNF694</td></tr>
<tr><td>0006351 // tra</td><td>0005634 // nu</td><td>0005515 // pr</td><td>Zinc finger pro</td><td>ZKSC7_HUMAN|ZKSCAN7|ZNF167|ZNF448|ZNF64</td></tr>
<tr><td>0072332 // in</td><td>0005634 // nu</td><td>0008270 // zir</td><td>Zinc finger ma</td><td>ZMAT1_HUMAN|ZMAT1|KIAA1789</td></tr>
<tr><td>0043065 // po</td><td>0005730 // nu</td><td>0003723 // RN</td><td>Zinc finger ma</td><td>ZMAT3_HUMAN|ZMAT3|PAG608|WIG1</td></tr>
<tr><td>0000398 // m</td><td>0005654 // nu</td><td>0008270 // zir</td><td>Zinc finger ma</td><td>ZMAT5_HUMAN|ZMAT5</td></tr>
<tr><td>0048146 // po</td><td>0043231 // in</td><td>0008270 // zir</td><td>Zinc finger MI</td><td>ZMIZ1_HUMAN|ZMIZ1|KIAA1224|RAI17|ZIMP10</td></tr>
<tr><td></td><td></td><td></td><td></td><td></td></tr>
<tr><td>0022604 // re</td><td>0005829 // cy</td><td>0008270 // zir</td><td>Zinc finger MY</td><td>ZMYM2_HUMAN|ZMYM2|FIM|RAMP|ZNF198</td></tr>
<tr><td>0036159 // in</td><td>0005737 // cy</td><td>0005515 // pr</td><td>Zinc finger MY</td><td>ZMY10_HUMAN|ZMYND10|BLU|LUCA12.4</td></tr>
<tr><td></td><td>0005622 // in</td><td>0046872 // m</td><td>Zinc finger MY</td><td>ZMY12_HUMAN|ZMYND12</td></tr>
<tr><td>1903758 // ne</td><td>0005737 // cy</td><td>0035064 // m</td><td>Protein kinase</td><td>PKCB1_HUMAN|ZMYND8|KIAA1125|PRKCBP1|RACK7</td></tr>
<tr><td>0006351 // tra</td><td>0005634 // nu</td><td>0005515 // pr</td><td>Zinc finger pro</td><td>ZNF10_HUMAN|ZNF10|KOX1</td></tr>
<tr><td>0006351 // tra</td><td>0005634 // nu</td><td>0003677 // DI</td><td>Zinc finger pro</td><td>ZN100_HUMAN|ZNF100</td></tr>
<tr><td>0006351 // tra</td><td>0005634 // nu</td><td>0003677 // DI</td><td>Zinc finger pro</td><td>ZN101_HUMAN|ZNF101</td></tr>
<tr><td>0006351 // tra</td><td>0005634 // nu</td><td>0003677 // DI</td><td>Zinc finger pro</td><td>ZN107_HUMAN|ZNF107|ZFD25|ZNF588</td></tr>
<tr><td>0006351 // tra</td><td>0070062 // ex</td><td>0003677 // DI</td><td>Zinc finger pro</td><td>ZN114_HUMAN|ZNF114</td></tr>
<tr><td>0045892 // ne</td><td>0005634 // nu</td><td>0003677 // DI</td><td>Zinc finger pro</td><td>ZNF12_HUMAN|ZNF12|GIOT3|KOX3|ZNF325</td></tr>
</table>

0006351 // tra	0005634 // nu	0003677 // DI	Zinc finger pro	ZN121_HUMAN|ZNF121|ZNF20
0006351 // tra	0005634 // nu	0003677 // DI	Zinc finger pro	ZN134_HUMAN|ZNF134
0000122 // ne	0005634 // nu	0005515 // pr	Zinc finger pro	ZN136_HUMAN|ZNF136
0006351 // tra	0005634 // nu	0003677 // DI	Putative zinc f	ZN137_HUMAN|ZNF137P|ZNF137
0006351 // tra	0005634 // nu	0003677 // DI	Zinc finger pro	ZNF14_HUMAN|ZNF14|GIOT4|KOX6
0006351 // tra	0005634 // nu	0003700 // tra	Zinc finger pro	ZN140_HUMAN|ZNF140
0006351 // tra	0005634 // nu	0003677 // DI	Zinc finger pro	ZN142_HUMAN|ZNF142|KIAA0236
0006355 // re	0005730 // nu	0008201 // he	Zinc finger pro	OZF_HUMAN|ZNF146|OZF
0006351 // tra	0005634 // nu	0005515 // pr	Zinc finger pro	ZN165_HUMAN|ZNF165|ZPF165|ZSCAN7
0000122 // ne	0005829 // cy	0000981 // RI	Zinc finger pro	ZN174_HUMAN|ZNF174|ZSCAN8
0000122 // ne	0005829 // cy	0005515 // pr	Zinc finger pro	ZN175_HUMAN|ZNF175
0006351 // tra	0005634 // nu	0003677 // DI	Zinc finger pro	ZN177_HUMAN|ZNF177
0006351 // tra	0005634 // nu	0005515 // pr	Zinc finger pro	ZN180_HUMAN|ZNF180
0006351 // tra	0005634 // nu	0003677 // DI	Zinc finger pro	ZN181_HUMAN|ZNF181
0006351 // tra	0005634 // nu	0003677 // DI	Zinc finger pro	ZN184_HUMAN|ZNF184
	0015629 // ac	0051015 // ac	Zinc finger pro	ZN185_HUMAN|ZNF185
0006351 // tra	0005634 // nu	0003677 // DI	Zinc finger pro	ZN195_HUMAN|ZNF195|ZNFP104
0006351 // tra	0005634 // nu	0005515 // pr	Zinc finger pro	ZNF20_HUMAN|ZNF20|KOX13
0006366 // tra	0005739 // m	0000981 // RI	Zinc finger pro	ZN202_HUMAN|ZNF202|ZKSCAN10
1901030 // po	0005634 // nu	0001227 // tra	Zinc finger pro	ZN205_HUMAN|ZNF205|ZNF210
0006351 // tra	0005634 // nu	0008270 // zir	Zinc finger pro	ZN208_HUMAN|ZNF208|ZNF91L
0006355 // re	0005634 // nu	0005515 // pr	Zinc finger pro	ZN212_HUMAN|ZNF212|ZNFC150
0006351 // tra	0005634 // nu	0008270 // zir	Zinc finger pro	ZNF22_HUMAN|ZNF22|KOX15|KROX26
0006355 // re	0005634 // nu	0003700 // tra	Zinc finger pro	ZN221_HUMAN|ZNF221
0006351 // tra	0005634 // nu	0005515 // pr	Zinc finger pro	ZN223_HUMAN|ZNF223
0006351 // tra	0005634 // nu	0003677 // DI	Zinc finger pro	ZN229_HUMAN|ZNF229
0006351 // tra	0005829 // cy	0005515 // pr	Zinc finger pro	ZN232_HUMAN|ZNF232|ZSCAN11
0000122 // ne	0005634 // nu	0000978 // RI	Zinc finger pro	ZN239_HUMAN|ZNF239|HOK2|MOK2
0006351 // tra	0005634 // nu	0003677 // DI	Zinc finger pro	ZN248_HUMAN|ZNF248
0006351 // tra	0005634 // nu	0003677 // DI	Zinc finger pro	ZNF25_HUMAN|ZNF25|KOX19
0006355 // re	0005634 // nu	0003677 // DI	Zinc finger pro	ZN253_HUMAN|ZNF253|BMZF1|ZNF411
0000122 // ne	0005634 // nu	0003677 // DI	Zinc finger pro	ZN254_HUMAN|ZNF254|BMZF5|ZNF539|ZNF91L
0007275 // m	0005634 // nu	0003700 // tra	Zinc finger pro	ZN256_HUMAN|ZNF256|BMZF3
0006355 // re	0005634 // nu	0003677 // DI	Zinc finger pro	ZN257_HUMAN|ZNF257|BMZF4
0007275 // m	0005829 // cy	0003677 // DI	Zinc finger pro	ZN260_HUMAN|ZNF260|ZFP260
0007275 // m	0005634 // nu	0003677 // DI	Zinc finger pro	ZN267_HUMAN|ZNF267
0043066 // ne	0005829 // cy	0003677 // DI	Zinc finger pro	ZN268_HUMAN|ZNF268
0006351 // tra	0005634 // nu	0003677 // DI	Zinc finger pro	ZN273_HUMAN|ZNF273
0006351 // tra	0005634 // nu	0003677 // DI	Zinc finger pro	ZN275_HUMAN|ZNF275
0006351 // tra	0005634 // nu	0003677 // DI	Zinc finger pro	ZNF28_HUMAN|ZNF28|KOX24
0006351 // tra	0005634 // nu	0003677 // DI	Zinc finger pro	Z280B_HUMAN|ZNF280B|SUHW2|ZNF279|ZNF632
0006355 // re	0005634 // nu	0003677 // DI	Zinc finger pro	ZN282_HUMAN|ZNF282|HUB1
0006355 // re	0005634 // nu	0003700 // tra	Zinc finger pro	ZN284_HUMAN|ZNF284|ZNF284L

0006351 // tra	0005634 // nu	0003677 // DI	Zinc finger pro	ZN285_HUMAN|ZNF285|ZNF285A
0006351 // tra	0005634 // nu	0003677 // DI	Putative zinc f	Z286B_HUMAN|ZNF286B|ZNF286C|ZNF286L
0045893 // po	0005634 // nu	0003677 // DI	Zinc finger pro	ZN287_HUMAN|ZNF287|ZKSCAN13
0006351 // tra	0005634 // nu	0003677 // DI	Zinc finger pro	ZNF30_HUMAN|ZNF30|KOX28
0006351 // tra	0005634 // nu	0003677 // DI	Zinc finger pro	ZN316_HUMAN|ZNF316
0006351 // tra	0005634 // nu	0003677 // DI	Zinc finger pro	ZN317_HUMAN|ZNF317|KIAA1588
0006351 // tra	0005634 // nu	0005515 // pr	Zinc finger pro	ZNF32_HUMAN|ZNF32|KOX30
0006351 // tra	0005634 // nu	0003677 // DI	Zinc finger pro	ZN320_HUMAN|ZNF320
0032784 // re	0005681 // sp	0003723 // RN	DBIRD comple	ZN326_HUMAN|ZNF326|ZIRD
0006351 // tra	0005634 // nu	0003677 // DI	Zinc finger pro	ZN331_HUMAN|ZNF331|RITA|ZNF361|ZNF463
0051569 // re	0035097 // hi	0005515 // pr	Zinc finger pro	ZN335_HUMAN|ZNF335
0006355 // re	0005634 // nu	0003677 // DI	Zinc finger pro	ZN33A_HUMAN|ZNF33A|KIAA0065|KOX31|ZNF11|ZNF1
0006351 // tra	0005829 // cy	0003677 // DI	Zinc finger pro	ZNF34_HUMAN|ZNF34|KOX32
0006351 // tra	0005634 // nu	0003677 // DI	Zinc finger pro	ZN343_HUMAN|ZNF343
0072332 // in	0005730 // nu	0003723 // RN	Zinc finger pro	ZN346_HUMAN|ZNF346|JAZ
0006351 // tra	0005634 // nu	0003677 // DI	Zinc finger pro	ZN347_HUMAN|ZNF347|ZNF1111
0071300 // ce	0005623 // ce	0003700 // tra	Zinc finger pro	ZNF35_HUMAN|ZNF35
0006355 // re	0005634 // nu	0005515 // pr	Zinc finger pro	ZN350_HUMAN|ZNF350|ZBRK1
0001822 // kid	0005634 // nu	0003677 // DI	Zinc finger pro	Z354A_HUMAN|ZNF354A|EZNF|HKL1|TCF17
0006351 // tra	0005634 // nu	0003677 // DI	Zinc finger pro	Z354B_HUMAN|ZNF354B
0019827 // ste	0005634 // nu	0003677 // DI	Zinc finger pro	ZN358_HUMAN|ZNF358
0000723 // te	0005737 // cy	0005515 // pr	Protein ZNF36	ZN365_HUMAN|ZNF365|KIAA0844
0006357 // re	0005634 // nu	0003677 // DI	Zinc finger pro	ZN367_HUMAN|ZNF367|ZFF29
0006355 // re	0005634 // nu	0003677 // DI	Zinc finger pro	ZN37A_HUMAN|ZNF37A|KOX21|ZNF37
0000122 // ne	0005634 // nu	0044212 // tra	Zinc finger pro	ZN382_HUMAN|ZNF382
0006351 // tra	0031965 // nu	0003677 // DI	Zinc finger pro	ZN383_HUMAN|ZNF383|HSD17
0006977 // DI	0030425 // de	0003723 // RN	Zinc finger pro	Z385A_HUMAN|ZNF385A|HZF|RZF|ZNF385
0072332 // in	0001650 // fib	0002039 // p5	Zinc finger pro	Z385B_HUMAN|ZNF385B|ZNF533
0072332 // in	0005634 // nu	0005515 // pr	Zinc finger pro	Z385C_HUMAN|ZNF385C
0072332 // in	0005634 // nu	0008270 // zir	Zinc finger pro	Z385D_HUMAN|ZNF385D|ZNF659
0006355 // re	0005829 // cy	0000978 // RN	Zinc finger pro	ZN395_HUMAN|ZNF395|HDBP2|PBF
0006351 // tra	0005634 // nu	0003677 // DI	Zinc finger pro	ZN396_HUMAN|ZNF396|ZSCAN14
0006351 // tra	0005634 // nu	0005515 // pr	Zinc finger pro	ZN408_HUMAN|ZNF408|PFM14|PRDM17
0006351 // tra	0005634 // nu	0003677 // DI	Zinc finger pro	ZNF41_HUMAN|ZNF41
0006351 // tra	0005634 // nu	0005515 // pr	Zinc finger pro	ZN410_HUMAN|ZNF410|APA1
0006351 // tra	0015630 // m	0005515 // pr	Zinc finger pro	ZN415_HUMAN|ZNF415
0045892 // ne	0005634 // nu	0003677 // DI	Zinc finger pro	ZN418_HUMAN|ZNF418|KIAA1956
0006351 // tra	0070062 // ex	0003677 // DI	Zinc finger pro	ZN420_HUMAN|ZNF420
0006351 // tra	0005634 // nu	0005515 // pr	Zinc finger pro	ZN426_HUMAN|ZNF426

		0046872 // m	Zinc finger pro	ZN428_HUMAN|ZNF428|C19orf37
0006351 // tra	0005634 // nu	0003677 // DI	Zinc finger pro	ZN429_HUMAN|ZNF429
0006351 // tra	0005634 // nu	0003677 // DI	Zinc finger pro	ZNF43_HUMAN|ZNF43|KOX27|ZNF39|ZNF39L1
0006351 // tra	0005634 // nu	0003677 // DI	Zinc finger pro	ZN430_HUMAN|ZNF430
0006351 // tra	0005634 // nu	0046872 // m	Zinc finger pro	ZN431_HUMAN|ZNF431|KIAA1969
0006351 // tra	0005654 // nu	0003677 // DI	Zinc finger pro	ZN432_HUMAN|ZNF432|KIAA0798
0006351 // tra	0005634 // nu	0003677 // DI	Zinc finger pro	ZN433_HUMAN|ZNF433
0006351 // tra	0005829 // cy	0003677 // DI	Zinc finger pro	ZN438_HUMAN|ZNF438
0006351 // tra	0005634 // nu	0005515 // pr	Zinc finger pro	ZN439_HUMAN|ZNF439
0006351 // tra	0005634 // nu	0003677 // DI	Zinc finger pro	ZNF44_HUMAN|ZNF44|GIOT2|KOX7|ZNF55|ZNF58
0006351 // tra	0005634 // nu	0003677 // DI	Zinc finger pro	ZN441_HUMAN|ZNF441
0006351 // tra	0005634 // nu	0003677 // DI	Zinc finger pro	ZN442_HUMAN|ZNF442
0006915 // ap	0005634 // nu	0003677 // DI	Zinc finger pro	ZN443_HUMAN|ZNF443
0006351 // tra	0005634 // nu	0005515 // pr	Zinc finger pro	ZN462_HUMAN|ZNF462|KIAA1803
0006355 // re	0005634 // nu	0003677 // DI	Zinc finger pro	ZN467_HUMAN|ZNF467
0006351 // tra	0005634 // nu	0003677 // DI	Zinc finger pro	ZN468_HUMAN|ZNF468
0006351 // tra	0005634 // nu	0003677 // DI	Zinc finger pro	ZNF48_HUMAN|ZNF48|ZNF553
0006351 // tra	0005829 // cy	0003676 // nu	Zinc finger pro	ZN480_HUMAN|ZNF480
0006351 // tra	0005634 // nu	0003677 // DI	Zinc finger pro	ZN485_HUMAN|ZNF485
0006351 // tra	0070062 // ex	0003677 // DI	Zinc finger pro	ZN486_HUMAN|ZNF486|KRBO2
0006351 // tra	0005634 // nu	0003677 // DI	Putative zinc f	ZN487_HUMAN|ZNF487|KRBO1|ZNF487P
0006351 // tra	0005634 // nu	0005515 // pr	Zinc finger pro	ZN488_HUMAN|ZNF488
0006351 // tra	0005634 // nu	0005515 // pr	Zinc finger pro	ZN490_HUMAN|ZNF490|KIAA1198
0006351 // tra	0005634 // nu	0003677 // DI	Zinc finger pro	ZN491_HUMAN|ZNF491
0006351 // tra	0005634 // nu	0003677 // DI	Zinc finger pro	ZN492_HUMAN|ZNF492|KIAA1473|ZNF115
0006351 // tra	0005634 // nu	0003677 // DI	Zinc finger pro	ZN493_HUMAN|ZNF493
0045893 // po	0005634 // nu	0005515 // pr	Zinc finger pro	ZN496_HUMAN|ZNF496|ZKSCAN17
0006351 // tra	0005634 // nu	0003677 // DI	Zinc finger pro	ZN497_HUMAN|ZNF497
0006351 // tra	0005634 // nu	0003677 // DI	Zinc finger pro	ZN501_HUMAN|ZNF501|ZNF52
0010629 // ne	0005634 // nu	0046872 // m	Zinc finger pro	ZN503_HUMAN|ZNF503|NOLZ1
0006355 // re	0005634 // nu	0003677 // DI	Zinc finger pro	ZN510_HUMAN|ZNF510|KIAA0972
0006351 // tra	0005634 // nu	0003677 // DI	Zinc finger pro	ZN512_HUMAN|ZNF512|KIAA1805
0006351 // tra	0005634 // nu	0005515 // pr	Zinc finger pro	Z512B_HUMAN|ZNF512B|KIAA1196
0007275 // m	0005634 // nu	0003700 // tra	Zinc finger pro	ZN516_HUMAN|ZNF516|KIAA0222
0006351 // tra	0005634 // nu	0000981 // RN	Zinc finger pro	Z518A_HUMAN|ZNF518A|KIAA0335|ZNF518
0006351 // tra	0005634 // nu	0000981 // RN	Zinc finger pro	Z518B_HUMAN|ZNF518B|KIAA1729
0006351 // tra	0005634 // nu	0003677 // DI	Zinc finger pro	ZN527_HUMAN|ZNF527|KIAA1829
0006351 // tra	0005634 // nu	0003677 // DI	Zinc finger pro	ZN528_HUMAN|ZNF528|KIAA1827
0006351 // tra	0005634 // nu	0003677 // DI	Zinc finger pro	ZN529_HUMAN|ZNF529|KIAA1615
0006351 // tra	0005634 // nu	0003677 // DI	Zinc finger pro	ZN530_HUMAN|ZNF530|KIAA1508
0006351 // tra	0005634 // nu	0003677 // DI	Zinc finger pro	ZN532_HUMAN|ZNF532|KIAA1629
0017148 // ne	0005737 // cy	0000900 // tra	Zinc finger pro	ZN540_HUMAN|ZNF540|Nbla10512
0006351 // tra	0005634 // nu	0003677 // DI	Zinc finger pro	ZN546_HUMAN|ZNF546|ZNF49
0006351 // tra	0005634 // nu	0003677 // DI	Zinc finger pro	ZN548_HUMAN|ZNF548

0006351 // tra	0005634 // nu	0003677 // DI	Zinc finger pro	ZN549_HUMAN|ZNF549
0006351 // tra	0005634 // nu	0003677 // DI	Zinc finger pro	ZN551_HUMAN|ZNF551|KOX23
0006351 // tra	0005634 // nu	0003677 // DI	Zinc finger pro	ZN555_HUMAN|ZNF555
0006351 // tra	0005634 // nu	0003677 // DI	Zinc finger pro	ZN557_HUMAN|ZNF557
0006351 // tra	0005634 // nu	0003677 // DI	Zinc finger pro	ZN559_HUMAN|ZNF559
0006355 // re	0005622 // int	0003676 // nu	Protein ZNF55	A0A0A6YYI6_HUMAN|ZNF559-ZNF177
0006351 // tra	0005634 // nu	0003677 // DI	Zinc finger pro	ZN561_HUMAN|ZNF561
0006351 // tra	0005634 // nu	0003677 // DI	Zinc finger pro	ZN562_HUMAN|ZNF562
0006351 // tra	0005634 // nu	0003677 // DI	Zinc finger pro	ZN563_HUMAN|ZNF563
0006351 // tra	0005634 // nu	0003677 // DI	Zinc finger pro	ZN565_HUMAN|ZNF565
0006351 // tra	0005634 // nu	0003677 // DI	Zinc finger pro	ZN566_HUMAN|ZNF566
0001701 // in	0005634 // nu	0046872 // m	Zinc finger pro	ZN568_HUMAN|ZNF568
0006351 // tra	0005634 // nu	0003677 // DI	Zinc finger pro	ZN569_HUMAN|ZNF569
0006351 // tra	0005634 // nu	0003677 // DI	Zinc finger pro	ZNF57_HUMAN|ZNF57|ZNF424
0006351 // tra	0005634 // nu	0003677 // DI	Zinc finger pro	ZN570_HUMAN|ZNF570
0006351 // tra	0005634 // nu	0003677 // DI	Zinc finger pro	ZN571_HUMAN|ZNF571|HSPC059
0006351 // tra	0005634 // nu	0005515 // pr	Zinc finger pro	ZN572_HUMAN|ZNF572
0006351 // tra	0005634 // nu	0003677 // DI	Zinc finger pro	ZN573_HUMAN|ZNF573
0006351 // tra	0005634 // nu	0003677 // DI	Zinc finger pro	ZN577_HUMAN|ZNF577
0006351 // tra	0005634 // nu	0003677 // DI	Zinc finger pro	ZN578_HUMAN|ZNF578
0006351 // tra	0005634 // nu	0003723 // RI	Zinc finger pro	ZN579_HUMAN|ZNF579
0001938 // po	0005634 // nu	0005515 // pr	Zinc finger pro	ZN580_HUMAN|ZNF580
0006351 // tra	0005634 // nu	0005515 // pr	Zinc finger pro	ZN581_HUMAN|ZNF581|HSPC189
0006351 // tra	0005634 // nu	0003676 // nu	Zinc finger pro	Z585A_HUMAN|ZNF585A
0006351 // tra	0005634 // nu	0003676 // nu	Zinc finger pro	Z585B_HUMAN|ZNF585B
0000122 // ne	0005634 // nu	0000977 // RI	Zinc finger pro	ZN589_HUMAN|ZNF589|SZF1
1903026 // ne	0005730 // nu	0043023 // rib	Zinc finger pro	ZN593_HUMAN|ZNF593|ZT86
0006351 // tra	0005634 // nu	0003677 // DI	Zinc finger pro	ZN594_HUMAN|ZNF594|KIAA1871
0006351 // tra	0005634 // nu	0003677 // DI	Zinc finger pro	ZN595_HUMAN|ZNF595
0006351 // tra	0005634 // nu	0003677 // DI	Zinc finger pro	ZN597_HUMAN|ZNF597
0016567 // pr		0003723 // RI	E3 ubiquitin-p	ZN598_HUMAN|ZNF598
0006351 // tra	0005634 // nu	0005515 // pr	Zinc finger pro	ZN599_HUMAN|ZNF599
0006351 // tra	0005634 // nu	0003677 // DI	Zinc finger pro	ZN600_HUMAN|ZNF600
0006351 // tra	0005634 // nu	0000976 // tra	Zinc finger pro	ZN605_HUMAN|ZNF605
0006351 // tra	0005634 // nu	0003677 // DI	Zinc finger pro	ZN606_HUMAN|ZNF606|KIAA1852|ZNF328
0006351 // tra	0005634 // nu	0005515 // pr	Zinc finger pro	ZN607_HUMAN|ZNF607
0006351 // tra		0046872 // m	Zinc finger pro	ZN608_HUMAN|ZNF608|KIAA1281
0006351 // tra	0005634 // nu	0003677 // DI	Zinc finger pro	ZN610_HUMAN|ZNF610
0006351 // tra	0005634 // nu	0003677 // DI	Zinc finger pro	ZN611_HUMAN|ZNF611
0000122 // ne	0005634 // nu	0044212 // tra	Zinc finger pro	ZN613_HUMAN|ZNF613
0006351 // tra	0005634 // nu	0003677 // DI	Zinc finger pro	ZN614_HUMAN|ZNF614
0006351 // tra	0005634 // nu	0003677 // DI	Zinc finger pro	ZN615_HUMAN|ZNF615
0006351 // tra	0005634 // nu	0003677 // DI	Zinc finger pro	ZN620_HUMAN|ZNF620
0006351 // tra	0005634 // nu	0005515 // pr	Zinc finger pro	ZN625_HUMAN|ZNF625

0006351 // tra	0005634 // nu	0003677 // DI	Zinc finger pro	ZN626_HUMAN|ZNF626
0006351 // tra	0005634 // nu	0003677 // DI	Zinc finger pro	ZN627_HUMAN|ZNF627
0006351 // tra	0005634 // nu	0003700 // tra	Zinc finger pro	ZN628_HUMAN|ZNF628
0006351 // tra	0005634 // nu	0003677 // DI	Zinc finger pro	ZN630_HUMAN|ZNF630
0006351 // tra	0005829 // cy	0000981 // RI	Zinc finger pro	ZN641_HUMAN|ZNF641
0006351 // tra	0005634 // nu	0003677 // DI	Zinc finger pro	ZN644_HUMAN|ZNF644|KIAA1221|ZEP2
0000122 // ne	0005615 // ex	0044212 // tra	Zinc finger pro	ZN649_HUMAN|ZNF649
0071294 // ce	0005634 // nu	0000976 // tra	Zinc finger pro	ZN658_HUMAN|ZNF658
0006351 // tra	0005634 // nu	0003677 // DI	Zinc finger pro	Z658B_HUMAN|ZNF658B
0006351 // tra	0005634 // nu	0003677 // DI	Putative zinc f	ZNF66_HUMAN|ZNF66|ZNF66P
0006351 // tra	0005634 // nu	0003677 // DI	Zinc finger pro	ZN660_HUMAN|ZNF660
0006351 // tra	0005634 // nu	0003677 // DI	Zinc finger pro	ZN662_HUMAN|ZNF662
0006351 // tra	0005634 // nu	0003677 // DI	Zinc finger pro	ZN665_HUMAN|ZNF665|ZFP160L
0000122 // ne	0005634 // nu	0000978 // RI	Zinc finger pro	ZN668_HUMAN|ZNF668
0006355 // re	0005622 // in	0003676 // nu	ZNF670-ZNF69	F2Z2N8_HUMAN|ZNF670-ZNF695
0006351 // tra	0005634 // nu	0003677 // DI	Zinc finger pro	ZN671_HUMAN|ZNF671
0006351 // tra	0005634 // nu	0003677 // DI	Zinc finger pro	ZN674_HUMAN|ZNF674
0019221 // cy	0005634 // nu	0003677 // DI	Zinc finger pro	ZN675_HUMAN|ZNF675|TIZ
0006351 // tra	0005634 // nu	0003677 // DI	Zinc finger pro	ZN676_HUMAN|ZNF676
0006351 // tra	0005634 // nu	0003677 // DI	Zinc finger pro	ZN677_HUMAN|ZNF677
0006351 // tra	0005634 // nu	0003677 // DI	Zinc finger pro	ZN679_HUMAN|ZNF679
0006351 // tra	0005634 // nu	0003677 // DI	Zinc finger pro	ZN681_HUMAN|ZNF681
0006351 // tra	0005634 // nu	0003677 // DI	Zinc finger pro	ZN682_HUMAN|ZNF682
0006351 // tra	0005634 // nu	0003677 // DI	Zinc finger pro	ZN684_HUMAN|ZNF684
0006351 // tra	0005829 // cy	0003677 // DI	Zinc finger pro	ZN687_HUMAN|ZNF687|KIAA1441
0006351 // tra	0005634 // nu	0003677 // DI	Zinc finger pro	ZN688_HUMAN|ZNF688
0006351 // tra	0005634 // nu	0003677 // DI	Zinc finger pro	ZN689_HUMAN|ZNF689
0006351 // tra	0005634 // nu	0003677 // DI	Zinc finger pro	ZNF69_HUMAN|ZNF69
0000122 // ne	0005730 // nu	0046872 // m	Zinc finger pro	ZN692_HUMAN|ZNF692
0006351 // tra	0005634 // nu	0003677 // DI	Zinc finger pro	ZN697_HUMAN|ZNF697
0006351 // tra	0005634 // nu	0005515 // pr	Zinc finger pro	ZN699_HUMAN|ZNF699
0006351 // tra	0005634 // nu	0003677 // DI	Zinc finger pro	ZNF70_HUMAN|ZNF70
0006355 // re	0005634 // nu	0003677 // DI	Zinc finger pro	M0R085_HUMAN|ZNF701
0006351 // tra	0005634 // nu	0003677 // DI	Putative zinc f	ZN702_HUMAN|ZNF702P|ZNF702
0060828 // re	0005737 // cy	0070491 // re	Zinc finger pro	ZN703_HUMAN|ZNF703|ZEPPO1|ZPO1
0006351 // tra	0005634 // nu	0003677 // DI	Putative zinc f	Z705E_HUMAN|ZNF705E
0006417 // re	0005737 // cy	0046872 // m	Zinc finger pro	ZN706_HUMAN|ZNF706|HSPC038|PNAS-113
0006351 // tra	0005634 // nu	0003677 // DI	Zinc finger pro	ZN708_HUMAN|ZNF708|KOX8|ZNF15|ZNF15L1
0006351 // tra	0005634 // nu	0000976 // tra	Zinc finger pro	ZN709_HUMAN|ZNF709
0006351 // tra	0005634 // nu	0003677 // DI	Endothelial zi	ZNF71_HUMAN|ZNF71|EZFIT
0045893 // po	0070062 // ex	0005515 // pr	Zinc finger pro	ZN711_HUMAN|ZNF711|CMPX1|ZNF6
0006351 // tra	0005634 // nu	0003677 // DI	Zinc finger pro	ZN713_HUMAN|ZNF713
0006351 // tra	0005634 // nu	0003677 // DI	Zinc finger pro	ZN714_HUMAN|ZNF714
0006351 // tra	0005634 // nu	0003677 // DI	Zinc finger pro	ZN716_HUMAN|ZNF716
0006351 // tra	0005634 // nu	0003677 // DI	Zinc finger pro	ZN718_HUMAN|ZNF718
0006351 // tra	0005634 // nu	0003677 // DI	Zinc finger pro	ZN724_HUMAN|ZNF724|ZNF724P

0006351 // tra	0005634 // nu	0003677 // DN	Zinc finger pro	ZN726_HUMAN	ZNF726
0006351 // tra	0005634 // nu	0003677 // DN	Putative zinc f	ZN727_HUMAN	ZNF727|ZNF727P
0006351 // tra	0005634 // nu	0003677 // DN	Putative zinc f	ZN730_HUMAN	ZNF730
0006351 // tra	0005634 // nu	0003677 // DN	Zinc finger pro	ZN737_HUMAN	ZNF737|ZNF102
0006351 // tra	0005634 // nu	0003677 // DN	Protein ZNF73	ZN738_HUMAN	ZNF738
0006351 // tra	0015629 // ac	0003677 // DN	Zinc finger pro	ZNF74_HUMAN	ZNF74|ZNF520
0045944 // po	0005634 // nu	0001078 // tra	Zinc finger pro	ZN746_HUMAN	ZNF746|PARIS
0006355 // re	0005622 // int	0003676 // nu	KRAB domain-	ZN747_HUMAN	ZNF747
0006351 // tra	0005634 // nu	0003677 // DN	Zinc finger pro	ZN749_HUMAN	ZNF749
0045944 // po	0005634 // nu	0001046 // co	Zinc finger pro	ZN750_HUMAN	ZNF750
0006357 // re	0005634 // nu	0043565 // se	Zinc finger pro	ZNF76_HUMAN	ZNF76|D6S229E|ZNF523
0006351 // tra	0005634 // nu	0003677 // DN	Zinc finger pro	ZN761_HUMAN	ZNF761|KIAA2033
0006351 // tra	0005634 // nu	0003677 // DN	Zinc finger pro	ZN764_HUMAN	ZNF764
0006351 // tra	0005634 // nu	0003677 // DN	Zinc finger pro	ZNF77_HUMAN	ZNF77
0006351 // tra	0005730 // nu	0003677 // DN	Zinc finger pro	ZN771_HUMAN	ZNF771
0006351 // tra	0005634 // nu	0003677 // DN	Zinc finger pro	ZN772_HUMAN	ZNF772
0006351 // tra	0005634 // nu	0003677 // DN	Zinc finger pro	ZN773_HUMAN	ZNF773|ZNF419B
0006351 // tra	0005634 // nu	0005515 // pr	Zinc finger pro	ZN774_HUMAN	ZNF774
0006351 // tra	0005634 // nu	0003677 // DN	Zinc finger pro	ZN775_HUMAN	ZNF775
0006351 // tra	0005634 // nu	0003677 // DN	Zinc finger pro	ZN777_HUMAN	ZNF777|KIAA1285
0006351 // tra	0005634 // nu	0003677 // DN	Zinc finger pro	ZN778_HUMAN	ZNF778
0006351 // tra	0005634 // nu	0005515 // pr	Protein ZNF78	ZN783_HUMAN	ZNF783
0006351 // tra	0005634 // nu	0005515 // pr	Zinc finger pro	ZN785_HUMAN	ZNF785
0006351 // tra	0005634 // nu	0003677 // DN	Zinc finger pro	ZN786_HUMAN	ZNF786
0006351 // tra	0005634 // nu	0003677 // DN	Zinc finger pro	ZN788_HUMAN	ZNF788
0006351 // tra	0005654 // nu	0003677 // DN	Zinc finger pro	ZN789_HUMAN	ZNF789
0006351 // tra	0005634 // nu	0003677 // DN	Zinc finger pro	ZN790_HUMAN	ZNF790
0006351 // tra	0005634 // nu	0005515 // pr	Zinc finger pro	ZN792_HUMAN	ZNF792
0006351 // tra	0005634 // nu	0003677 // DN	Zinc finger pro	ZN793_HUMAN	ZNF793
0006351 // tra	0005634 // nu	0003677 // DN	Zinc finger pro	ZN799_HUMAN	ZNF799|ZNF842
0010975 // re	0005634 // nu	0003674 // m	Zinc finger pro	Z804A_HUMAN	ZNF804A|C2orf10
		0046872 // m	Zinc finger pro	Z804B_HUMAN	ZNF804B
0006351 // tra	0005634 // nu	0003677 // DN	Zinc finger pro	ZN808_HUMAN	ZNF808
0006351 // tra	0005634 // nu	0003677 // DN	Zinc finger pro	ZN813_HUMAN	ZNF813
0006351 // tra	0005634 // nu	0003677 // DN	Zinc finger pro	ZN816_HUMAN	ZNF816|ZNF816A
0006351 // tra	0005634 // nu	0003677 // DN	Zinc finger pro	ZN823_HUMAN	ZNF823|ZFP36
0006351 // tra	0005634 // nu	0003677 // DN	Putative zinc f	ZN826_HUMAN	ZNF826P|ZNF826
0006351 // tra	0005634 // nu	0003677 // DN	Zinc finger pro	ZN829_HUMAN	ZNF829
0006351 // tra	0005634 // nu	0003677 // DN	Zinc finger pro	ZNF83_HUMAN	ZNF83|ZNF816B
		0046872 // m	Putative zinc f	ZN833_HUMAN	ZNF833P|ZNF833
0006351 // tra	0005634 // nu	0003677 // DN	Zinc finger pro	ZN837_HUMAN	ZNF837
		0046872 // m	Zinc finger pro	ZN839_HUMAN	ZNF839|C14orf131
0006351 // tra	0005634 // nu	0005515 // pr	Zinc finger pro	ZN844_HUMAN	ZNF844
0006351 // tra	0005634 // nu	0003677 // DN	Zinc finger pro	ZNF85_HUMAN	ZNF85

0006351 // tra	0005634 // nu	0003677 // DI	Zinc finger pro	ZN850_HUMAN|ZNF850
		0046872 // m	Zinc finger pro	ZN853_HUMAN|ZNF853
0006351 // tra	0005634 // nu	0003674 // m	Zinc finger pro	ZN862_HUMAN|ZNF862|KIAA0543
0006351 // tra	0005634 // nu	0003676 // nu	Zinc finger pro	ZN878_HUMAN|ZNF878
0006351 // tra	0005634 // nu	0003677 // DI	Zinc finger pro	ZN883_HUMAN|ZNF883
0006351 // tra	0005634 // nu	0003677 // DI	Zinc finger pro	ZN888_HUMAN|ZNF888
0006351 // tra	0005634 // nu	0003677 // DI	Zinc finger pro	ZN891_HUMAN|ZNF891
0006351 // tra	0005634 // nu	0003677 // DI	Zinc finger pro	ZNF90_HUMAN|ZNF90
0070895 // ne	0005634 // nu	0003677 // DI	Zinc finger pro	ZNF91_HUMAN|ZNF91
0006351 // tra	0005634 // nu	0003677 // DI	Zinc finger pro	ZNF92_HUMAN|ZNF92
0070895 // ne	0005634 // nu	0000976 // tra	Zinc finger pro	ZNF93_HUMAN|ZNF93|ZNF505
0006351 // tra	0005634 // nu	0003677 // DI	Zinc finger pro	ZNF98_HUMAN|ZNF98|ZNF739
0006351 // tra	0005634 // nu	0003677 // DI	Zinc finger pro	ZNF99_HUMAN|ZNF99|C19orf9
0043161 // pr	0005765 // lys	0004842 // ub	E3 ubiquitin-p	ZNRF2_HUMAN|ZNRF2|RNF202
0030576 // Ca	0005654 // nu	0031369 // tra	Zinc finger pro	ZPR1_HUMAN|ZPR1|ZNF259
0006351 // tra	0005634 // nu	0005515 // pr	Zinc finger and	ZSC12_HUMAN|ZSCAN12|KIAA0426|ZNF305|ZNF96
0006351 // tra	0005634 // nu	0005515 // pr	Zinc finger and	ZSC16_HUMAN|ZSCAN16|ZNF392|ZNF435
0007283 // sp	0005634 // nu	0003677 // DI	Zinc finger and	ZSCA2_HUMAN|ZSCAN2|ZFP29|ZNF854
0006351 // tra	0070062 // ex	0003677 // DI	Zinc finger and	ZSC23_HUMAN|ZSCAN23|ZNF390|ZNF453
0006355 // re	0005829 // cy	0005515 // pr	Zinc finger and	ZSC26_HUMAN|ZSCAN26|ZNF187
0006351 // tra	0005634 // nu	0003677 // DI	Zinc finger and	ZSC31_HUMAN|ZSCAN31|ZNF310P|ZNF323
		0008270 // zin	Zinc finger SW	ZSWM3_HUMAN|ZSWIM3|C20orf164
	0005615 // ex	0008270 // zin	Zinc finger SW	ZSWM5_HUMAN|ZSWIM5|KIAA1511
		0008270 // zin	Zinc finger SW	ZSWM6_HUMAN|ZSWIM6|KIAA1577
0000070 // m	0030425 // de	0005515 // pr	ZW10 interact	ZWINT_HUMAN|ZWINT
0006351 // tra	0005634 // nu	0070742 // C2	Zinc finger X-l	ZXDA_HUMAN|ZXDA
0006351 // tra	0005634 // nu	0003674 // m	Zinc finger X-l	ZXDB_HUMAN|ZXDB
0016567 // pr	0031462 // Cu	0004842 // ub	Protein zyg-11	ZY11A_HUMAN|ZYG11A|ZYG11
0016567 // pr	0031462 // Cu	0004842 // ub	Protein zyg-11	ZY11B_HUMAN|ZYG11B|KIAA1730
0007165 // sig	0005925 // fo	0003723 // RN	Zyxin	ZYX_HUMAN|ZYX

:5

8/PRO1071

ːO34012

NQ540/PRO1083

RO6017

2

2

1B

ɪFS

ᑭRO2044|PRO2619|PRO2675|UNQ696/PRO1341

)34564

|PSEC0166|UNQ171/PRO197

ꞌA

A0899

F1

180

.7P1|PRO2667

XN9

:14/PRO240

RO1266

SPC142

NQ418/PRO852

.8

8

800/PRO19607/PRO21344

HUSSY-12

CHL1A2

M|TSLC1

G140

Ↄ23202

2R|UNQ2522/PRO6015

.1

.

)710

/PRO4340

'PRO7168

8
1

'IC1|SDI1|WAF1

4

GF3

2

2|C10orf93|TTC40

HR1|HFL1|HFL2

MPP7
\1802|ZNF828

SPC134

PC177|PNAS-114|PNAS-2

RO1890
Q299/PRO339

7

3

/PRO1487

56

)5

4

RO541
PRO1156/PRO9783

ITP1

56/PRO1287
)082
;3

6

)305

)6714

|PCDH16

RTK6|TRKE

41

O4983

O238
.773

)0594

H7L|DNAHC3|DNHD2|HDHC3|HL19

IHD1L|UNQ5781/PRO12970

O4980

.A0728

|DYHC|KIAA0325

20

G36

764/PRO1556

|TACSTD1|TROP1

84/PRO1154

003/PRO9924

O1075

V3-AS1

PRED34|UNQ2504/PRO5993

)|EGFL11|SPAM|UNQ9424/PRO34591

9B|PP12994|PRO0659

:512/PRO6001

PSEC0139|UNQ611/PRO1198

XO6A

320
\A0696

HL4|FOXG1A|FOXG1B|FOXG1C

NL

|HRIHFB2063

[illegible]O6182

|UNQ686/PRO1326

A39P|UNQ432/PRO793

NQ2753/PRO6492

PR30

4319
9925

BAP2|WBSCR11|WBSCR12

'GC|RETGC1

R1
R2

744|MITR

620

034761

)1779

T1H2AL|HIST1H2AM|H2AFC|H2AFD|H2AFI|H2AFN|H2AFP
ST1H2BG|HIST1H2BI|H2BFA|H2BFG|H2BFH|H2BFK|H2BFL

T2H2AA

D|SDR5C1|XH98G2
SEC0029|UNQ207/PRO233
NQ502/PRO474

.

'RO19612

026

336

/PRO21384

56/PRO5737
ᑭ3|UNQ1896/PRO4342

;1|NS1BP|HSPC068

ECC2
45

IP

)

RO21184

\|KIAA1004

A0780

169

√KIND|hucep-9

ı.1|KRTAP4.10

RTAP5.7
SK1

;CR5|HSPC046

00

31993

01|MSTP044

JFP11

LMR2

RO31982

Q221/PRO247
7

'PRO1338

|P2RY7

RO9917

1251

MAGEA2|MAGEA2A

IP1

|SEK1|SERK1|SKK1

IQ2435/PRO4995

50|EXLM1|RGR1|TRAP170

32

O1305

48

[illegible]20088

[illegible]2PV2

OA

ꓤO34208

C0142

PSEC0213

TSG1

)

‾EERIN1

94A

RO701

|NALP1

3

RMT1|AD-003

RO1133
PRO34206

[illegible]P

1729

1183

.2

.899/PRO4345

RED79

|UNQ6486/PRO21335

MEF3L1|RGAG3

00

PRO328

'55

϶/PRO6012

9/PRO235

C261|HSPC306

RP|MDS010|UNQ490/PRO1006
535|Nbla00003

TDC2

OU5F1P1|POU5FLC20|POU5FLC8

|

ISTP033

.PL|SAPS3

02/PRO343

\0367

D1|HDHD1A

D1

PRO706

7/PRO4303

SPC236|MDS5

A2

1

3

2

)72

JNQ408/PRO770

RGP5|RGP6|RGP7|RGPD7
NBP2L3

;B128

)0761

L

)1128

RO302

;

1317
PRO1480
PRO34487

RO4353

300

MD2

4088

3

KS5

MD2

L

PRO28631

.3

SIRP

682

Q367/PRO703

71

79

JQ441/PRO874

SBC2|SLC4A6

21A3
C|SLC21A6
1A8

SLC21A9

C21A12

2L2
F2L4

SRGAP2A

56|MEGAP|SRGAP2

77

PRO23203

PRO19578

)9852
)

IAA1756|MYNE1

.

AFII135

Q1881/PRO4324

ʾ6614|PSEC0088|UNQ204/PRO230

3

JNQ151/PRO177

RO204

'PRO4318

[illegible]5926/PRO19820

)821

'RO195

ʼRO382
)

JNQ763/PRO1555

<2|ZTNFR9|UNQ160/PRO186

NQ321/PRO366

509
4
33

|WSL|WSL1|UNQ455/PRO779

207

/PRO715

RO175

S1

009

'88

)1568
)296
)1311

5

C205

19

91

ᑭRO4326

\Q308/PRO351

NQ730/PRO1412

la10071

5/PRO31996

6

3|P/OKcl.13

010|MSTP012|PP730

9576

NF380|CGI-89|UNQ261/PRO298

IHFB2411

TTP
IS11B

2A1|PP10436

C3H9|ZC3HDC9|ZIP

l1A|ZNF33
